# Discovery of High Abundances of Aster-Like Nanoparticles in Pelagic Environments: Characterization and Dynamics

**DOI:** 10.3389/fmicb.2019.02376

**Published:** 2019-10-15

**Authors:** Jonathan Colombet, Hermine Billard, Bernard Viguès, Stéphanie Balor, Christelle Boulé, Lucie Geay, Karim Benzerara, Nicolas Menguy, Guy Ilango, Maxime Fuster, François Enault, Corinne Bardot, Véronique Gautier, Angia Sriram Pradeep Ram, Télesphore Sime-Ngando

**Affiliations:** ^1^Laboratoire Microorganismes: Génome et Environnement, Université Clermont Auvergne, UMR CNRS 6023, Aubière, France; ^2^Plateforme de Microscopie Électronique Intégrative (METI), Centre de Biologie Intégrative (CBI), Université Paul Sabatier Toulouse III, CNRS, Toulouse, France; ^3^Centre Technologique des Microstructures (CTμ), Université Claude Bernard Lyon 1, Villeurbanne, France; ^4^Institut de Minéralogie, de Physique des Matériaux, et de Cosmochimie, Sorbonne Universités, UMR CNRS 7590, Université Pierre et Marie Curie Paris 06, Muséum National d'Histoire Naturelle, Institut de Recherche pour le Développement-Unité Mixte de Recherche 206, Paris, France; ^5^Plateforme GENTYANE, UMR INRA 1095 GDEC, Université Clermont Auvergne, Site de Crouel, Clermont Ferrand, France

**Keywords:** pleomorphic nanoparticles, femtoplankton, femtoplanktonic diversity, aquatic ecosystems, aquatic ecology

## Abstract

This study reports the discovery of Aster-Like Nanoparticles (ALNs) in pelagic environments. ALNs are pleomorphic, with three dominant morphotypes which do not fit into any previously defined environmental entities [i.e., ultramicro-prokaryotes, controversed nanobes, and non-living particles (biomimetic mineralo-organic particles, natural nanoparticles or viruses)] of similar size. Elemental composition and selected-area electron diffraction patterns suggested that the organic nature of ALNs may prevail over the possibility of crystal structures. Likewise, recorded changes in ALN numbers in the absence of cells are at odds with an affiliation to until now described viral particles. ALN abundances showed marked seasonal dynamics in the lakewater, with maximal values (up to 9.0 ± 0.5 × 10^7^ particles·mL^−1^) reaching eight times those obtained for prokaryotes, and representing up to about 40% of the abundances of virus-like particles. We conclude that (i) aquatic ecosystems are reservoirs of novel, abundant, and dynamic aster-like nanoparticles, (ii) not all virus-like particles observed in aquatic systems are necessarily viruses, and (iii) there may be several types of other ultra-small particles in natural waters that are currently unknown but potentially ecologically important.

## Introduction

Recent advances in environmental and nanoparticle sciences have helped to reveal an unexpected diversity of living and non-living femto-entities (0.02–0.2 μm as defined for femtoplankton by Sieburth et al., 1978) in the environment. Previously considered to be mainly composed of viruses (Sieburth et al., 1978), the successive discovery in significant abundance, and in various environments, of mysterious nanobes (Folk, 1993; McKay et al., 1996; Sillitoe et al., 1996; Uwins et al., 1998), extracellular vesicles (EVs) (Soler et al., 2015; Biller et al., 2017), ultramicro-prokaryotes (Duda et al., 2012; Brown et al., 2015; Hug et al., 2016; Ortiz-Alvarez and Casamayor, 2016; Wurch et al., 2016; Castelle et al., 2018; Ghuneim et al., 2018), and biomimetic mineralo-organic particles (BMOPs) (Wu et al., 2016), has significantly increased the complexity within the environmental fraction of femto-entities.

Contrary to viruses or EVs, controversed nanobes, some of which could be affiliable to BMOPs, and ultramicro-prokaryotes, including recently discovered CPR (*Candidate Phyla Radiation*) and DPANN (*Diapherotrites, Parvarchaeota, Aenigmarchaeota, Nanoarchaeota, Nanohaloarchaea*), have the ability to develop outside a host (Benzerara et al., 2003, 2006; Martel and Young, 2008; Raoult et al., 2008; Wu et al., 2016). Nanobes exhibit diverse morphotypes: coccoid, amiboid, ovoid or filamentous shapes (Folk, 1993; McKay et al., 1996; Sillitoe et al., 1996; Uwins et al., 1998). Among them, only ultramicro-prokaryotes are clearly affiliated to living organisms according to the volumetric criteria advanced by the National Research Council (1999), i.e., the theoretical minimal cell volume (TMCV) sufficient to house nucleic acids and the associated biosynthetic machinery is at 0.008 μm3. Though these new entities were described in natural environments, relatively little is known about their ecological significance. Available data however suggests a significant impact on the biogeochemical cycles. EVs are potentially involved in cell communication, competition and survival of bacteria (Liu et al., 2019). Interactions between ultramicro-prokaryotes and other micro-organisms communities may shape natural microbiome function (Castelle et al., 2018). Likewise, BMOPs incorporate trace elements and proteins suggesting that these entities may play a role in the circulation and availability of minerals and organic molecules in the environment (Wu et al., 2016). Characterizing the femtoplankton biomass and the diversity of its representatives seems crucial to our understanding of the functioning of aquatic ecosystems.

In this study, we report the discovery of abundant and seasonally-fluctuating populations of “Aster-Like Nanoparticles” (ALNs) in a freshwater lake of Massif Central (France), with volumes lower than TMCV. ALNs display typical and unique morphological features. Physical-chemical aspects, pleomorphism, flow cytometry and growth analyses of ALNs are presented and compared to distinctive features of living or not-living particles of similar size. Preliminary attempts to evidence DNA-based heredity support are reported.

## Materials and Methods

### Study Sites and Sample Collection

Samples were collected at the surface of an artificial and highly eutrophic freshwater lake (surface area 1.2 ha, maximum depth 2.5 m) near Neuville in the French Massif Central (45°44'24”N; 3°27'39”E; 465 m altitude). Part of the samples were immediately fixed with 1% (v/v) formaldehyde and stored at 4°C until analysis (see below). Unfixed samples were transported at 4°C to the laboratory and treated within two h (see below). *In situ* dynamics of ALNs were monitored in 11 fixed samples collected between November 2016 and January 2018. Table 1 lists the physical-chemical characteristics of the water analyzed once, in February 2017.

**Table 1:** Physical–chemical characteristics of the lakewater on February 2017.

**Parameters**	**Values**
Water temperature, °C	4
pH	7.4
Total carbon, mg·L^−1^	16
Total phosphorous, mg·L^−1^	0.28
Un-ionized ammonia, mg·L^−1^	<0.05
Alkali concentration, °F	0
Complete alkali concentration, °F	27.65
Kjeldahl nitrogen, mg·L^−1^	3.3
Overall nitrogen, mg·L^−1^	4
Ammonium, mg·L^−1^	0.12
Carbonate, mg·L^−1^	Below limit of detection
Chloride, mg·L^−1^	12.5
Nitrate, mg·L^−1^	3.1
Orthophosphate, mg·L^−1^	0.05
Nitrite, mg·L^−1^	0.05
Total potassium, mg·L^−1^	9.6
Total sodium, mg·L^−1^	3.1
Total calcium, mg·L^−1^	9.1
Total magnesium, mg·L^−1^	1.7

Detection of ALNs was also conducted on surface microlayer samples of 16 selected geographical stations (namely HL1 to HL16) from the Ha Long Bay (Vietnam). Details on these samples and their environment were provided in a previous work (Pradeep Ram et al., 2018). ALNs were quantified on electronic microscopy grids prepared as mentioned below.

### ALN, Prokaryote, and Virus-Like Particle (VLP) Counts and Imaging

ALNs in fixed samples were collected by centrifugation at 15,000 *g* for 20 min at 14°C directly onto 400-mesh electron microscopy copper grids covered with carbon-coated Formvar film (Pelanne Instruments, Toulouse, France). Particles were over-contrasted using uranyl salts as described elsewhere (Borrel et al., 2012). ALNs were counted by transmission electron microscopy (TEM) using a Jeol 1200EX microscope (JEOL, Akishima, Tokyo, Japan) at 80 kV and x50,000 magnification. Grids were scanned before counting to check that ALNs were randomly distributed. A defined area of the grid was then randomly selected for counting ALNs. Counts of ALNs were converted into ALNs per milliliter using a conversion factor deduced from control grids prepared with pre-determined concentrations of viruses. Direct magnifications ranging from x50,000 to x150,000 were required for morphological characterization of the particles. Volume of the ALN particles was computed by considering the radial arms as cylinders (extrapolation validated by cryo-TEM and SEM imaging; see below) and the central core as a sphere. Ultra-thin (20-nm thickness) sections were obtained and imaged as previously described (Kéraval et al., 2016). Counts of prokaryotes and VLPs from fixed samples were performed by flow cytometry as described elsewhere (Brussaard, 2004) using a BD FACS Calibur cytometer (BD Sciences, San Jose, CA) equipped with an air-cooled laser, delivering 15 mW at 488 nm with the standard filter set-up.

### Experimental Design

#### Enrichment and Culture of ALNs

**Figure 1:**
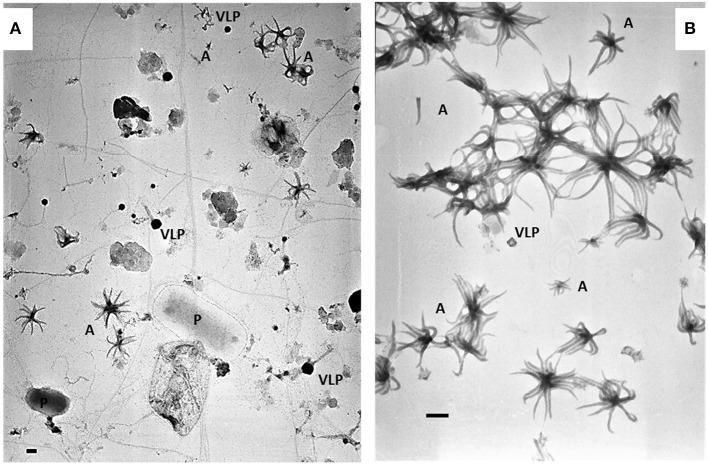
Electromicrographs showing heterogeneity of pelagic communities **(A)** in lakewater collected on March 15th 2017 and ALN-enriched culture **(B)** obtained from this sampling. P, prokaryote; VLP, virus-like particle; A, ALNs. Scale bars = 100 nm.

The sample with the highest density of ALNs collected on March 15th 2017 was used for enrichment and culture of ALNs. Within two h after sampling, 20 L of raw lake water was filtered through a 25-μm-pore-size nylon mesh and filtrates were immediately concentrated by tangential-flow ultrafiltration using a Kross-Flow system (Spectrum, Breda, The Netherlands) equipped with a 0.2-μm cut-off cartridge. Aliquots of this concentrated 0.2 μm−25 μm fraction were sequentially centrifuged at 8,000 *g*, 10,000 *g* (pellets discarded) then 12,000 *g* for 20 min each time at 14°C. ALNs contained in the supernatant of this last run were cultivated at 4°C in the dark with a regular supply of culture medium. To obtain this culture medium, ultra-filtrate <0.2 μm of the initial lake sample was filtered through a 30 KDa cut-off cartridge and autoclaved. Figure 1 shows ALN cultures obtained through this procedure compared to the raw samples. The pellet obtained at 12,000 *g* was suspended in distilled/deionized sterile water (DDW), centrifuged at 10,000 *g*, and the supernatant was directly frozen to−20°C for microscopic and flow cytometry analyses of Enriched-ALNs (E-ALNs).

Detailed procedure of experimental design and analyses is provided in supplementary materials (Figure S1).

#### Growth Monitoring

As state above, ALN cultures were enriched by sequential centrifugations at 8,000 *g*, 10,000 *g* (pellets discarded) then 12,000 *g* for 20 min each time at 14°C. For growth monitoring, this was followed by successive filtrations of the highest-speed supernatant through 0.45-μm and 0.2-μm filters (Sartorius, Göttingen, Germany) to obtain ALN-enriched but prokaryote-free medium. This filtrate (<0.2-μm) was diluted 10-folds in the culture medium (see above) and incubated in triplicate over a 36-day period at 4°C in the dark, then 12 uneven subsamples were taken and formaldehyde-fixed before counts. Absence of prokaryotes at the start and end of the growth monitoring period was checked by flow cytometry, transmission electron microscopy and plate count agar spreading incubated at 4°C and 20°C during 4 weeks.

#### Susceptibility to Chemical or Physical Agents

To address the question of the living nature of the ALNs we examined their susceptibility to various chemical (lysozyme, antibiotics) or physical (heat) agents. Lysozyme is an antimicrobial enzyme that destructs Gram + bacteria cell wall by peptidoglycan hydrolysis (Manchenko, 1994) and which can also act against viruses (Cisani et al., 1984; Lee-Huang et al., 2005). Antibiotics treatments used in this study are all known to block replication processes of bacteria DNA or protein synthesis (Engle et al., 1982; Dar et al., 2007). Novobiocin is principally active against Gram+ bacteria, gentamycin against Gram- bacteria and norfloxacin has a broad-spectrum bactericidal action. Heat shock above 85°C was used owing to the irreversible physiological damage caused by this treatment to biological entities (Mackey et al., 1991).

Prokaryote-free ALN fractions prepared as described under the ‘*growth monitoring’* section were separately treated with 2 mg/mL lysosyme (1 h at room temperature), submitted to heat-shock (1 h at 90°C) or supplemented with antibiotics (50 μg/mL norfloxacin in sterile DDW; 10 μg/mL gentamycin in sterile DDW or 250 μg/mL novobiocin in sterile DDW) (all chemicals from Sigma-Aldrich, Saint-Quentin-Fallavier, France). Treated samples were incubated for 20 days in the dark at 4°C. To test the efficiencies of these biocide treatments, we used two treated “control fractions”: ALN-free bacteria cultures isolated from lake Neuville and grown on the same culture medium as ALNs, and 0.2 μm filtered ALN-free but ultrafiltration-enriched VLP water lake. This second control fraction was obtained from Lake Pavin where ALNs are undetectable over the year. Biocide effects of treatments were determined by direct comparison of treated vs. untreated samples at day 20. ALN, prokaryote and femtoplanktonic communities were performed on formaldehyde-fixed samples at the end of the incubations as previously described. All tests were carried out in triplicates.

### Cryo-Transmission Electron Microscopy (Cryo-TEM) Specimen Preparation and Imaging

For cryo-TEM, 3 μL of unfixed suspensions containing ALNs were deposited onto glow-discharged Lacey Carbon 200-mesh grids and loaded into the thermostatic chamber of a Leica EM-GP automatic plunge freezer, set at 20°C, and 95% humidity. Excess solution was blotted for 1” with a Whatman filter paper No. 1, and the grid was immediately flash-frozen in liquid ethane cooled at−185°C. Specimens were then transferred onto a Gatan 626 cryo-holder, and cryo-TEM was carried out on a Jeol 2100 microscope, equipped with a LaB_6_ cathode and operating at 200 kV, under low-dose conditions. Images were acquired using SerialEM software (Mastronarde, 2005), with defocus ranging of 1,000 nm, on a Gatan US4000 CCD camera. This device was placed at the end of a GIF Quantum energy filter (Gatan Inc., Pleasanton, CA), operated in zero-energy-loss mode, with a slit width of 25 eV. Images were recorded at a magnification corresponding to the calibrated pixel size of 1.80Å or 0.89Å.

### Scanning Electron Microscopy (SEM) Specimen Preparation and Imaging

A fixed suspension (1% (v/v) formaldehyde) containing ALNs was deposited by filtration on 0.2-μm-pore-size filters (Whatman, Maidstone, UK), post-fixed with 1% osmium tetroxide, rinsed, and dehydrated through increasing concentrations of ethanol and then of hexamethyldisilasane. Following Cu sputter coating, dry filters were observed and imaged using a Zeiss Merlin Compact SEM operating at 2, 3 or 5 kV (Zeiss, Oberkochen, Germany).

### Energy-Filtered Transmission Electron Microscopy (EFTEM) and Electron Energy Loss Spectroscopy (EELS) Analyses

Observations of unfixed samples were carried out using a Jeol 2010F transmission electron microscope operating at 200 kV, equipped with a field emission gun, an high-resolution ultra-high-radiation (UHR) pole piece, a STEM device which allows Z-contrast imaging in the high-angular annular dark-field (HAADF) mode, and a GIF 200 Gatan energy filter. EFTEM elemental mapping of C, N, O and Ca was performed on entire ALN using the 3-window technique (Hofer et al., 1997). The 3-window technique requires three energy-filtered images: two positioned before the ionization edge (pre-edge images), which serve to calculate the background, and one positioned just after the edge (post-edge image). Calculated background image was subtracted from the post-edge image to give an elemental map, in which changes in background shape were taken into account. Maps were calculated for C, N and O K-edges and Ca L_2,3_ edges using a 20-eV-wide (for C) or 30-eV-wide (for N, O, Ca) energy window for pre-edge and post-edge. Zero-loss images were obtained by selecting elastically scattered electrons only. EELS spectra were acquired using a dispersion of 0.2 eV/channel to record spectra in the range 270–600 eV. Energy resolution was 1.3 eV as measured by the full width at half maximum of the zero-loss peak. Dwell time was optimized to acquire sufficient signal intensity and limit beam damage. Spectra were corrected from plural scattering using the Egerton procedure available with the EL/P program (Gatan).

### Nucleic Acid Staining, Membrane Markers, and Flow Cytometry (FC) Analyses and Sorting of ALNs

Unfixed suspensions containing ALNs were thawed at 4°C and diluted in 0.02-μm-filtered Tris EDTA buffer prior to FC analyses. Analyses were performed using four nucleic acid dyes [SYBR Green I (Invitrogen S7563, Paisley, UK), SYBR Gold (Invitrogen S11494), propidium iodide (PI) (Sigma-Aldrich P4864) and DAPI (Sigma-Aldrich 32670)] and two lipophilic membrane markers [FM4-64 (Molecular Probes T13320, Eugene, OR) and PKH26 (Sigma-Aldrich P9691)]. ALNs were i) stained at 80°C for 10 min with SYBR Green I or SYBR Gold as described in Brussaard (2004); ii) pre-heated at 80°C for 10 min then stained with 10 μg·mL^−1^ PI or 1 μg·mL^−1^ DAPI for 10 min in the dark. Nucleic acids were also stained without heating. Staining with FM4-64 (5 μg·mL^−1^) and PKH26 (1/500 diluted from commercial solution) was carried out in the dark for 10 min at room temperature. All experimental conditions were reproduced in triplicates. Triplicates of 0.2 μm ALN-free filtrated water lake (i.e., enriched VLPs water from lake Pavin) and cultivated bacteria from lake Neuville were used for biological controls. Cytometric analysis was performed on a BD FACSAria Fusion SORP flow cytometer (BD Biosciences) equipped with a 70-μm nozzle. Laser and filter configuration was as follows: DAPI was excited by a 355-nm UV laser, fluorescence was collected with a 410 long pass (LP) and a 450/50 band pass (BP). SYBR Green I and SYBR Gold were excited at 488 nm and fluorescence was collected with a 502 LP and a 530/30 BP. PI and FM4-64 were excited at 561 nm and fluorescence was collected with a 600 LP and a 610/20 BP for PI, and with a 685 LP and a 710/50 BP for FM4-64. PKH26 was excited at 561 nm and fluorescence was collected with a 582/15 BP. Targeted particles were visualized on a “marker fluorescence vs. side scatter” dotplot. Data were acquired and processed using FACSDivA 8 software (BD Biosciences). Characterization of ALNs and VLPs from samples processed for cytometric analyses was carried out by TEM as previously described. Plots were compared with those of a similarly-processed VLPs community obtained from Lake Pavin (see site description in Borrel et al., 2012) on October 24th 2017. FC sorting was performed on samples stained with SYBR Green I in un-heated conditions for optimal preservation of ALNs morphology and reliable morphotype diagnosis. Commonly described “viral fractions” (Brussaard, 2004) were gated on SYBR Green I fluorescence and sorted out using the continuous “Purity” mode. 0.5-μm fluorescent beads (Polysciences, Warrington, PA) served as control sorted fraction. Particles from sorted gates were re-analyzed by FC and identified and counted by TEM.

### Genomic Analyses

#### Nucleic Acids Extraction and Amplification

Genomic DNA was extracted from unfixed suspensions containing ALNs obtained as described in the section “*Growth monitoring*”. The sample was harvested by centrifugation at 12,000 g for 20 min at 14°C. The pellet was resuspended in 500 μl of sterile DDW and mixed with 600 ml of saturated phenol (pH 8.0). Then, two cycles of freezing in a liquid nitrogen bath (15 min) and thawing in a 100°C water bath (5 min) were conducted. The sample was mixed with 750 μL of chloroform and centrifuged at 14,000 g for 20 min at 4°C. Thereafter, the aqueous layer was transferred to another fresh 1.5 ml microtube and mixed with same volume of cold absolute ethanol and 3 M sodium acetate. The nucleic acid pellet obtained by centrifugation at 14,000 g for 20 min at 4°C was washed twice with ice-cold 70% ethanol and pelleted again. The pellet was resuspended in 50 μL of deionized water. Total extracted DNA was randomly amplified by Whole Genome Amplification (WGA) with GenomiPhi V2 kit (GE Healthcare, Chicago, Illinois, USA).

#### Library Preparation and Sequencing

Single-molecule Real-time long reads sequencing was performed with a PacBio Sequel Sequencer (Pacific Biosciences, Menlo Park, CA, USA). The SMRTBell library was prepared using a DNA Template Prep Kit 1.0, following the “procedure and checklist for greater than 10 kb template using AMPure PB beads” protocol. Genomic DNA(1,7 ug) was slightly sheared using a Covaris g-Tube (Covaris, UK) generating DNA fragments of approximately 20 kb. A Fragment Analyzer (Agilent Technologies, Santa Clara, CA, USA) assay was used to assess the fragment size distribution. Sheared genomic DNA was carried into the first enzymatic reaction to remove single-stranded overhangs followed by treatment with repair enzymes to repair any damages that may be present on the DNA backbone. A blunt-end ligation reaction followed by exonuclease treatment was conducted to generate the SMRT Bell template. Two AMPure PB beads 0.45X purifications, and one at 0.4X were used to obtain the final library. The SMRTBell library was quality inspected and quantified on a Fragment Analyzer (Agilent Technologies) and a Qubit fluorimeter with Qubit dsDNA HS reagent Assay kit (Life Technologies). A ready-to-sequence SMRTBell Polymerase Complex was created using a Binding Kit 2.1 (PacBio) and the primer V4, the diffusion loading protocol was used, according to the manufacturer's instructions. The PacBio Sequel instrument was programmed to load and sequenced the sample on PacBio SMRT cells v2.0 (Pacific Biosciences), acquiring one movie of 600 min per SMRTcell and generate 8 Gb of bases and an insert N50 at 7.75Kb.

#### Sequence Assembly and Annotation

The 1,930,845 raw PacBio reads (4.1 Kb in average) were assembled using the SMRT Analysis software and the Hierarchical Genome Assembly Process (HGAP) workflow (Chin et al., 2013). This procedure includes pre-assembly error correction, assembly and polishing. The circular nature of HGAP derived contigs was assessed via the dot-plotting tool Gepard (Krumsiek et al., 2007) and circular genome sequences were derived through an alignment approach and manual curation. The 5,162 corrected long reads (12.6 Kb in average) produced after the pre-assembly error correction process were utilized to determine the coverage of each contig using BLASTn (threshold of 90% on the identity percent) (Altschul et al., 1997). These corrected reads and contigs were compared using BLASTn to the SILVA 16S rRNA gene reference database (version 132) (Quast et al., 2013). The 233 contigs were also compared to the UniProt (February 2019) (UniProt Consortium, 2019) protein database using Diamond (sensitive mode) (Buchfink et al., 2015). Gene-calling was performed on contigs through the Prodigal software (Hyatt et al., 2010) and proteins were also compared to Uniprot using Diamond. Genomic data are presented in Supplementary Data Sheet 1.

## Results

### Morphological Analyses

**Figure 2:**
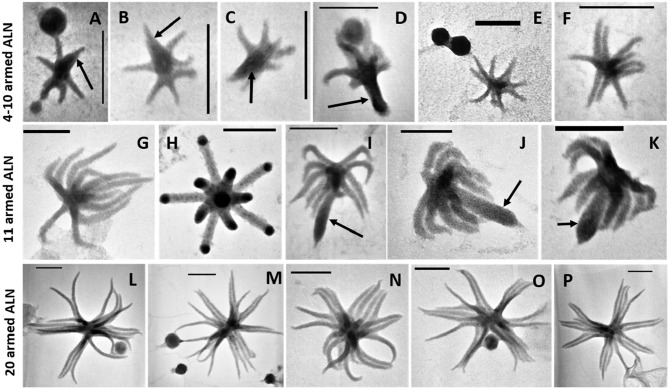
Transmission electron microscopy (TEM) micrographs of different morphotypes of aster-like nanoparticles (ALNs). **(A–F)** 4–10-armed forms with some **(A–D)** presenting a few arms articulated around a delta-shaped excrescence (arrows). **(G–K)** 11-armed forms and their budding 11-armed variants **(I–K)** with elongated and swollen bud-like excrescences (arrows). **(L–P)** 20-armed forms. Scale bars = 100 nm.

The ALN shape corresponds to arm-like segments which extend radially from a unique core structure. Three dominant morphotypes emerged on the basis of size and number of arms. The first morphotype displayed 4 to 10 arms connected to a delta-shaped tail with a mean length of 110 ± 18 nm and an average volume of 0.000055 μm3 (Figures 2A–F). The second morphotype consisted of forms with 11-arms that were consistently observed within the ALN population (Figures 2G–K). They were clearly distinct from the first morphotype by their length (333 ± 28 nm) and volume (mean value: 0.00057 μm3). Some appeared endowed with a singular bud-like appendix that seemed to arise from the center of symmetry of the particle. This appendix is thicker and slightly longer than the radial arms (Figures 2I–K). Finally, the third ALN morphotype corresponds to a sub-population that was composed of 20 arms (Figures 2L–P). These 20-armed forms constituted the lengthiest (439 ± 39 nm) and the most voluminous (0.0014 μm3) ALNs identified in our samples. Their arms displayed characteristic tapered shapes, were frequently associated by pairs (Figure 2P), and there was no indication for supernumerary outgrowths as seen in other ALN morphotypes.

**Figure 3:**
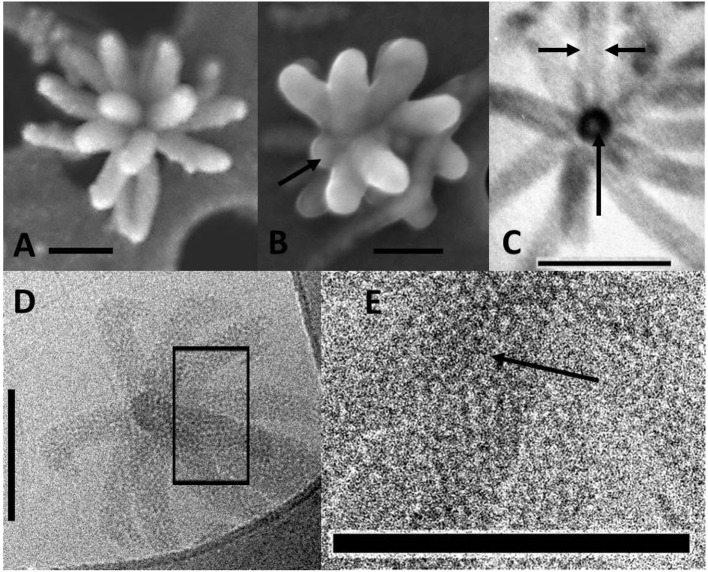
Electromicrographs of aster-like nanoparticles (ALNs). **(A,B)** Scanning electron microscopy (SEM) micrographs showing ALNs with multiple full-grown radial arms **(A)** or a mix of emerging (arrow) and full-grown arms **(B)**. **(C)** TEM micrograph of an ultra-thin section of an ALN. Sagittal sections of arms reveal a tubular appearance with electron light area enclosed by a wall-like structure (arrows). **(D,E)** Cryo-TEM micrographs. **(D)** Radial arms display a similar mottled appearance. **(E)** Magnified view of the box selected from the previous image revealing circular substructures (arrow). Scale bars = 100 nm.

Standard, scanning, and cryo-transmission electron microscopy (cryo-TEM) indicated that the arms of ALN particles project from a central core (Figures 3A–D). This core displayed high and homogeneous electron density, while the arms showed differential contrasts depending on the plane of the section. Arms appeared as hollow structures when viewed in sagittal sections (Figure 3C). Cryo-TEM of whole specimens allowed direct comparison between the central core, the radial arms and the supernumerary appendix of the 11-armed morphotypes (Figures 3D,E). All areas showed a similar dot-pattern, which was more conspicuous in the case of the central-core/appendix complex. Branched chains formed by these elementary components might account for the higher electron contrast and apparent rigidity of the supernumerary appendix compared to the slacker aspect of radial arms.

Descriptively, ALNs are pleomorphic nanoparticles with a reduced biovolume (<0.0014 μm3) exhibiting 4 to 20 radial arms organized around a unique central core.

### Elementary Analysis

**Figure 4:**
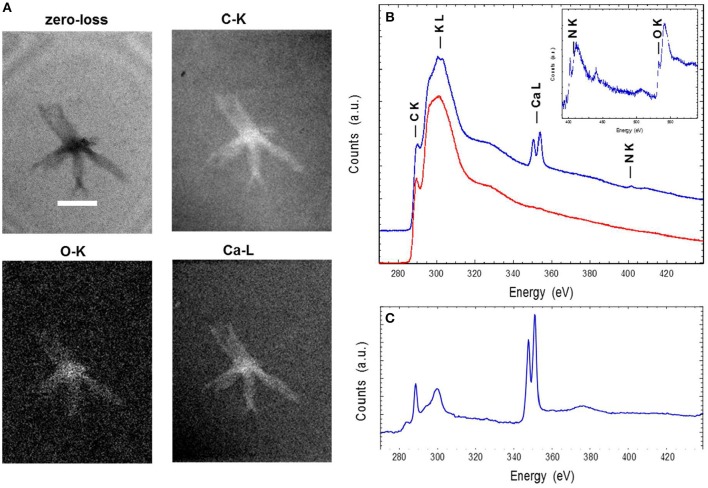
Energy-filtered transmission electron microscopy (EFTEM) **(A)** and electron energy loss spectroscopy (EELS) analyses **(B,C)** of aster-like nanoparticles (ALNs). **(A)** Zero-loss image and EFTEM C, O, and Ca maps of an ALN. Scale bar=200 nm. **(B)** EELS spectra of an ALN particle (blue) and formvar (red). Insert: close-up of the background-normalized spectrum of an ALN particle at the N and O K-edges. **(C)** EELS spectra of a reference calcite crystal at the C K-edge and the Ca L2,3-edges. The relative intensities at the C K-edge and Ca L2,3 edges correlate with C/Ca atomic ratio.

Energy-filtered transmission electron microscopy and electron energy loss spectroscopy (EELS) analyses performed on entire ALNs indicated that these nanoparticles were mostly composed of carbon, oxygen, calcium and nitrogen (Figures 4A,B). Trace amounts of potassium were also identified in association with the particles. EELS spectra at the C K-edge and Ca L_2,3_-edges of ALNs were significantly different from those of Ca-carbonates used as reference (Figure 4C) as they did not show a peak at 290 eV indicative of 1s → π^*^ electronic transitions in carbonates and a much lower Ca/C ratio. Likewise, selected-area electron diffraction of ALNs revealed an amorphous structure (K.B. personal communication).

Elemental composition and selected-area electron diffraction patterns thus suggest that ALNs are presumably formed of organic components, indicating that their organic nature may prevail over the possibility of mineral structures.

### Flow Cytometry Analysis

Flow cytometry (FC) analyses were performed on enriched-ALNs fraction (E-ALNs, *see Materials and methods*) composed of 96% ALNs and 4% of VLPs (Figure 1B) ascertained by TEM observation and counting.

**Figure 5:**
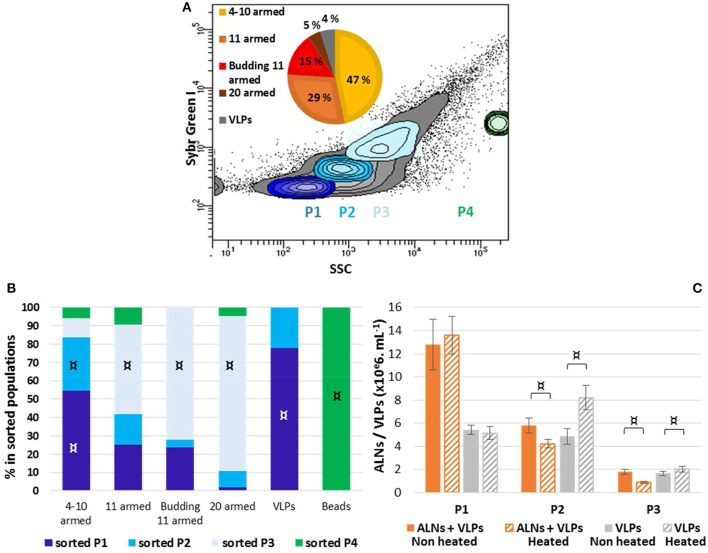
Flow cytometry analysis of aster-like nanoparticle-enriched preparations (E-ALNs). **(A)** Gating of three distinctive populations (P1, P2, P3) of SYBR Green-stained E-ALNs. Heating was omitted during the staining procedure, and beads (0.5 μm) were used as control fraction (P4). The gray area in scatter plot including in P1, P2, and P3 commonly represents VLP fractions (see Brussaard, 2004). A pie chart shows the relative proportions of ALNs morphotypes attested by TEM. **(B)** Distribution of ALNs morphotypes and beads in the four FACS-sorted fractions counted by TEM. **(C)** Cytometry counts of P1, P2, and P3 gated fractions compared to counts obtained when heating was included in the SYBR Green staining procedure, and to counts of particles in ALNs-free viral community stained in heat-driven conditions. Mean values from triplicate and standard errors are plotted. Significant representativeness of morphotypes in sorted populations **(B)** is indicated by symbols: ¤ (Fisher's exact test on a contingency table, *p* < 0.01). Significant differences between “non-heated” and “heated” conditions **(C)** are indicated by an symbol ¤ (Student *T*-test, *p* < 0.05).

No fluorescence signal was obtained using lipophilic markers FM4-64 or PKH26. Different nucleic acid dyes were tested, including DAPI, PI, SYBR Green I, SYBR Gold. While labeling with DAPI (a weakly permeant AT selective dye) and PI (impermeant nucleic acid intercalating dye) were unsuccessful, the SYBR dyes (permeant cyanine dyes), which are more sensitive compounds with high penetrating capacities, allowed to separate distinctive populations from E-ALN samples. As shown in Figure 5A, three populations termed P1, P2, and P3 were reproducibly split on the basis of SYBR Green I signal intensity and side scatter. Because ALNs were shown to be thermo-sensitive, heating was omitted in the protocol used for SYBR labeling. TEM indicated ALNs with familiar shapes in all sorted gates excepted in P4 gate that exclusively contained beads used as a control for sorting quality control (Figure 5B). Absence of ALNs in P4 indicated a non-random but differential sorting of the nanoparticles using the selected sorting gates. This was confirmed by TEM analyses of ALNs from the three sorting gates (Figure 5B). The sub-population from P3 gate provided the strongest SYBR signal, and consisted of large ALN morphotypes, i.e., 20-armed, budding 11-armed, and 11-armed morphotypes. The smaller ALNs (4–10 armed forms) were mostly concentrated in gates P1 and P2 together with virus-like particles and similar-sized particles of undetermined nature (VLPs).

Interference between ALNs and VLPs in FC particle quantification was evaluated using thermo-sensitivity property of ALNs compared to VLPs. This was achieved through comparative analysis of E-ALNs and a VLP community used as an ALN-free control, submitted or not to heating (Figure 5C). Heat induced a significant increase of counted VLPs in P2 (from 4.8 ± 0.7 to 8.2 ± 1.1 × 10^6^ mL^−1^) and P3 (from 1.7 ± 0.2 to 2.0 ± 0.3 × 10^6^ mL^−1^) populations sorted from the ALN-free control. Heating of P2 and P3 sorted from E-ALNs resulted in the opposite effect, i.e., a decrease in number of recorded events (P2: from 5.8 ± 0.6 to 4.2 ± 0.4 × 10^6^ mL^−1^; P3: from 1.8 ± 0.2 to 0.9 ± 0.1 × 10^6^ mL^−1^).

Overall, ALNs are positively labeled with SYBR nucleic acid dyes and interfere with VLPs quantification when using fluorescence based methods.

### Nucleic Acid Detection

Detection of nucleic acid was performed on fraction composed of >99% ALNs and <1% of VLPs ascertained by TEM and obtained as described in section ‘*growth monitoring*’.

**Figure 6:**
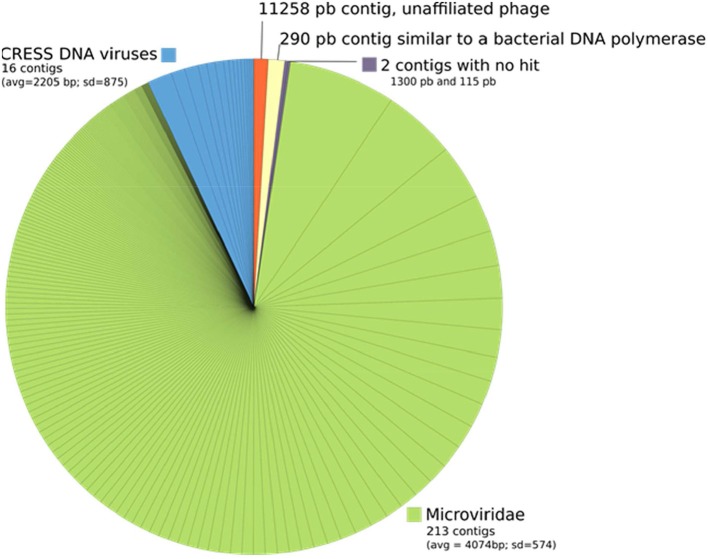
Taxonomic affiliation of the 233 contigs from ALN-enriched DNA templates. Each piece of the pie corresponds to a contig and its size is proportional to the number of reads that were associated to this contig.

No reads or contigs were similar to a prokaryotic 16S rRNA sequence. All the 233 contigs were shorter than 6 Kb except one contig of 11,258 bp. Almost all contigs could be unambigously affiliated to small single-stranded DNA viruses, 213 being affiliated to the Microviridae family and 16 to CRESS DNA viruses (circular Rep-encoding ssDNA viruses) (Figure 6). Two contigs had no similarity to Uniprot proteins and one contig was similar to a bacterial DNA-directed DNA polymerase (49.5 amino acid identity on 94 residues), but these contigs were all very short (1,300, 115, and 290 bp, respectively). Although the largest contig of 11,258 bp had no obvious affiliation, its characteristics are similar to known viruses that infect prokaryotes: (i) short genes (23 protein coding genes, 442 bp long in average), (ii) no strand switching and (iii) only 4 proteins out of 23 being similar to a protein of Uniprot (3 similar to proteins from unaffiliated phages and one to an archaeal protein, all four proteins having an unknown function).

Based on our genomic analyses and on the methodology used presence of nucleic acids in ALNs is not proved.

### Susceptibility to Chemical or Physical Agents

Effects of various life-inhibiting treatments were tested on ALNs, and on ALN-free prokaryotes and femtoplanktonic communities used as control after 20-day incubations.

**Figure 7:**
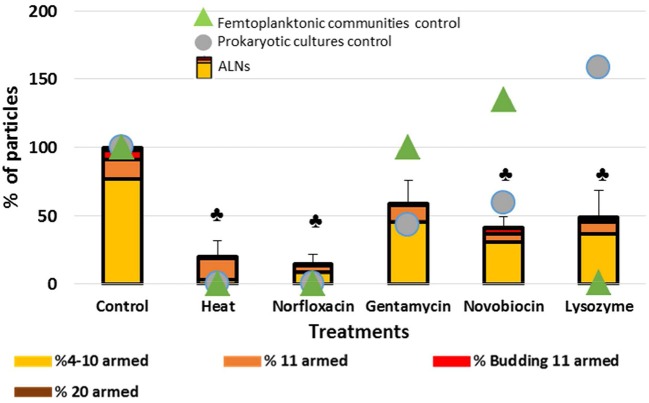
Sensitivity of the particles to heat, antibiotics or lysosyme treatments. Effects of treatments by heat, antibiotics and lysosyme were assessed compared to untreated controls at day 20. Results are expressed as the percentage of ALNs which have resisted to treatments and continued to develop compared to control after a 20-day incubation. ALN-free prokaryotic cultures and femtoplanktonic communities (0.2 μm filtrated water from an eutrophic lake) were used as controls. Mean values from triplicate and standard errors are plotted. Significant differences with control are indicated by symbol 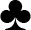 (*T*-test, *p* < 0.05).

Dramatic effects on total ALNs (*T* test, *p* < 0.05) were observed (Figure 7) after heating 1 h at 90°C or lysozyme (2 mg/mL) treatments (80 ± 12% and 51 ± 19% loss after 20 day incubation), and in the presence of the norfloxacin (50 μg/mL) and novobiocin (250 μg/mL) antibiotics (85 ± 7% and 58 ± 8%, respectively). Gentamycin antibiotic treatment had a smaller effect on the nanoparticles (41 ± 17% loss; *p* = 0.05). The losses were more pronounced following heating, lysozyme, norfloxacin, novobiocin and gentamycin treatments for the 4–10–armed form (96 ± 1, 53 ± 15, 89 ± 5, 61 ± 8, 41 ± 14%, respectively) compared to the 11–armed forms (0 ± 10, 37 ± 4, 66 ± 2, 53 ± 1, 12 ± 4%, respectively).

Lysozyme treatment leaded to a rise of prokaryotes (58%) suggesting that this ALN-free fraction was mostly composed of Gram – species. The complete loss of ALN-free femtoplanktonic communities (99%) showed the strong antiviral activity of this enzyme (Cisani et al., 1984; Lee-Huang et al., 2005). As expected prokaryotic “control fraction” displayed drastic loss in response to heat, norfloxacin, gentamicin and novobiocin (100%, 100%, 57%, 41%, respectively) (Figure 7).

Clearly, ALNs are susceptible to the life-inhibiting treatments. It seems also worth noting that responses to the treatments differed depending on the morphotypes. For example, 11-armed morphotypes proved much more resilient than others, while the 4–10-armed appeared more sensitive to the treatments.

### *In vitro* Monitoring

**Figure 8:**
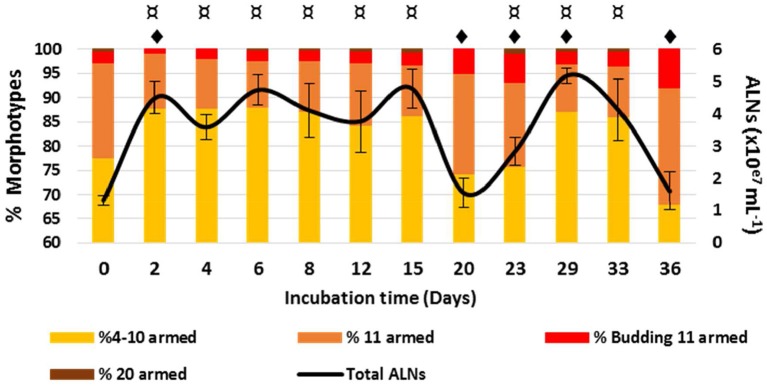
Development monitoring of aster-like nanoparticles (ALNs) in prokaryote-free medium. Temporal variations of ALNs abundances and ratios (in %) of different morphotypes over a 36-day period. Mean values from triplicate and standard errors are plotted. Significant differences between ALNs abundance at t (n) and t (0) and between ALNs abundance at t (n) and t (n-1) are indicated by symbols ¤ and 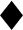, respectively (*T*-test, *p* < 0.05).

ALN population fluctuates over a 36-day period in prokaryote-free medium (PFM) at 4°C (Figure 8). A transient rise of abundance was evident from day 0 to day 1 (multiplication factor MF = 3.6). The population then appeared relatively stable from day 1 to day 15 before a marked decrease up to day 20, preceding a second rise period from day 20 to day 29 (MF = 3.3), then a second decline phase up to day 36. All these fluctuations with time were statistically significant (Figure 8). Quantification of ALN morphotypes in PFM revealed that 4–10-armed and 11-armed morphotypes fluctuate inversely over time (Figure 8, Spearman's r = -0.86, *p* < 0.001). These fluctuations were positively (4–10-armed forms) or negatively (11-armed forms) correlated to total ALN population (Spearman's *r* = 0.66 and *r* = −0.82, respectively, *p* < 0.05). The proportion of the smallest ALN forms (predominant morphotype at day 0) increases concomitantly with total number of ALNs but decreases as the abundance of total ALNs returns to baseline (day 0, day 20, and day 36). Inversely, the proportion of 11-armed forms increases during phases of total ALN decline (days 20, day 36). Throughout the incubation period, we were not able to detect any prokaryotic cells using different approaches: flow cytometry, transmission electron microscopy, and plate count agar spreading.

The above incubation monitoring show that the abundance of ALNs can change significantly over time in the absence of cellular entities, with different patterns registered in contrasted morphotype categories. The mechanisms under these changes remain unclear in the absence of a detectable genomic support.

### Ecosystemic Monitoring

**Figure 9:**
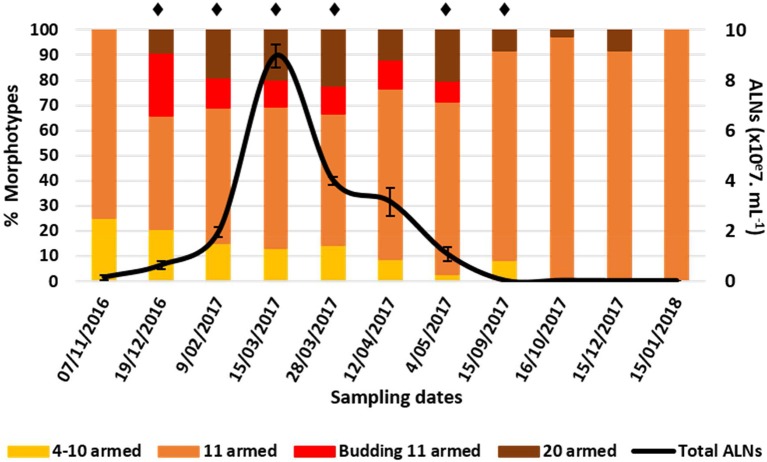
Abundance of aster-like nanoparticles (ALNs) *in situ* (Neuville-France) and ratios (in %) of different morphotypes over a 15-month period. Note the peak of abundance between late December 2017 and mid-March 2017 and the return to low-density populations within a few months. Mean values from triplicate and standard errors are plotted. Significant differences between ALNs abundance and the previous time are indicated by asterisks 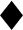 (Student *T*-test, *p* < 0.05).

Analysis of natural samples collected over a 13-month period in an eutrophic lake of the French Massif Central revealed high ALN abundances characterized by marked seasonal fluctuations (Figure 9). The maximal density reached a value of 9.0 ± 0.5 × 10^7^ mL^−1^ (March 15th 2017). ALN abundances were up to 8-fold higher than those obtained for FC-counted prokaryotes and represented up to 39% of the total FC-counted VLPs in corresponding samples. ALN abundances increased with season from autumn to spring (MF = 60). Prokaryote abundances fluctuated slowly from 0.8 ± 0.1 to 2.1 ± 0.4 × 10^7^ mL^−1^, while VLPs ranged from 2.0 ± 0.2 to 48.4 ± 1.5 × 10^7^ particles mL^−1^. For both communities, highest values were recorded in spring and in autumn, respectively (Figure S2). ALN abundance was not correlated to those of prokaryotes or VLPs.

At the morphotype level, we observed a high dominance of 11-armed forms which averaged 79 ± 16% of the total abundance over the 13-month sampling period (Figure 9). The 4–10 and 20-armed forms appeared in much smaller proportions (mean values = 10 ± 11% and 11 ± 11%, respectively). Proportions of these two forms were inversely correlated with those of 11-armed forms over time (Spearman's r = -0.77 and r = -0.73, respectively, *p* < 0.05). Proportions of budding 11-armed forms and 11-armed forms devoid of bud-like appendix were also negatively correlated to each other (Spearman's r = -0.91, *p* < 0.01). Budding forms accounted for the highest proportions at the onset and throughout the increasing phase of total ALNs, but then disappeared with the decline in the total ALN abundance. ALNs were exclusively composed of 11-armed morphotypes a few months after their population was stabilized at its lowest level.

**Figure 10:**
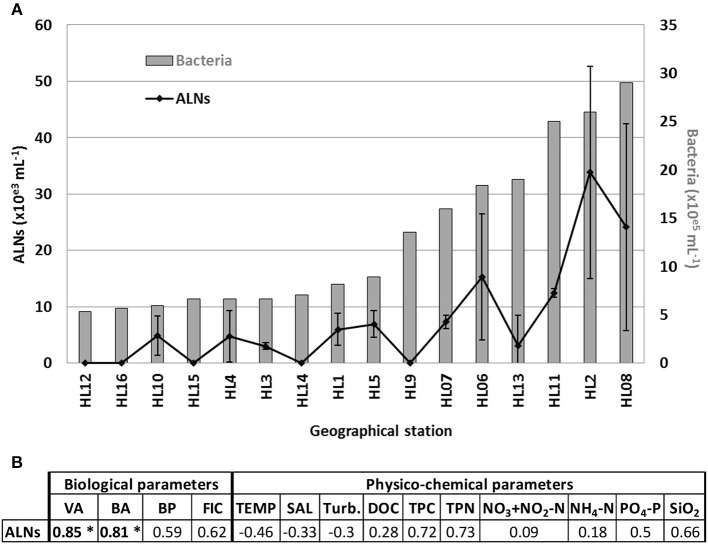
**(A)** Distribution of aster-like nanoparticles (ALNs) and bacteria abundances in 16 selected stations of a tropical coastal ecosystem (Ha Long Bay-Vietnam), and **(B)** analyses of correlations (Spearman's product-moment correlation coefficient) between ALNs and environmental parameters which compile all sampling points. All details on Ha Long Bay (Vietnam) environment are available from Pradeep Ram et al. (2018). Level of significance: ^*^p < 0.001. VA, viral abundance; BA, bacterial abundance; BP, bacterial production; FIC, frequency of infected cells; TEMP, temperature; SAL, salinity; Turb, turbidity; DOC, dissolved organic carbon; TPC, total particulate carbon; TPN, total particulate nitrogen in the bulk sample.

Detection of ALNs conducted on surface microlayer of 16 selected geographical stations (namely HL1 to HL16) from Ha Long Bay (Vietnam) show a high spatial heterogeneity with values ranging from undetectable to 3.4 × 10^4^ mL^−1^ (Figure 10A). The dynamics of ALNs and bacteria were significantly correlated (Spearman's r = 0.81, *p* < 0.01, Figure 10B). No reliable correlation could be established between ALNs and physico-chemical variables (Figure 10B).

ALNs show seasonal and ecosystemic fluctuations probably induced by environmental parameters. Proportions of each recorded morphotype shift according to seasonal dynamics.

## Discussion

### ALNs Are Original Pleomorphic Nanoparticles

Here we report the discovery of ‘Aster-Like Nanoparticles’ (ALNs) in lakewater. These pleomorphic entities, exhibit puzzling aster-like shapes with arm-like processes that project from a central core (Figure 3). All morphotypes exhibit shapes that distinguish ALNs from previously established groups of nanoparticles, including ultramicro-prokaryotes (Duda et al., 2012; Castelle et al., 2018; Ghuneim et al., 2018), controversed nanobes (Folk, 1993; Sillitoe et al., 1996; Uwins et al., 1998; Aho and Kajander, 2003; Yaghobee et al., 2015), biomimetic mineralo-organic particles (BMOPs) (Wu et al., 2016), viruses (King et al., 2018) or extracellular vesicles (EVs) (Soler et al., 2015; Biller et al., 2017). Their mean length ranges from 110 ± 18 nm (4–10-armed morphotype) to 439 ± 39 nm (20-armed morphotype). Volumetric estimates of all ALN types indicated values (averaging 0.000055 μm3, 0.00057 μm3, and 0.0014 μm3 for 4–10, 11, and 20-armed morphotypes respectively) that were significantly lower compared to the smallest known prokaryotes (Ghuneim et al., 2018) and to the Theoretical Minimal Cell Volume (TMCV). Nanobes, BMOPs, viruses (excepted giant viruses) and EVs are the sole examples of entities comparable to ALNs in terms of numerical volume. The composition (mostly carbon, oxygen, calcium and nitrogen with trace amounts of potassium) and the amorphous structure revealed by electronic microscopy (Figure 4) point out that ALN are possible organic particles (Uwins et al., 1998; Benzerara et al., 2003), or at least that their organic content may prevailed over their mineral composition known from mineral forming nanobes (Kajander et al., 2003), BMOPs or “natural nanoparticles” (Wu et al., 2016; Griffin et al., 2018), partly or totally composed of minerals.

ALN volumes were largely under the theoretical minimal cell volume (TMCV) required to house nucleic acids and the associated biosynthetic machinery required for a self-sufficient form of life (National Research Council, 1999). Use of the TMCV established there is 20 years ago to define compatibility with living nature must be however considered with caution. Indeed, recent advances in microbiology and virology have revealed existence of nanosized prokaryotes with biovolumes close to the TMCV. Giant viruses were reported as well. Genomic analysis of nanosized prokaryotes revealed a limited sub-cellular organization coupled with a significant reduction of biosynthetic and energy conservation pathways (Castelle et al., 2018; Ghuneim et al., 2018). Meanwhile, exceptionally large viruses were discovered that contain DNA encoding proteins involved in mRNA translation (Schulz et al., 2017; Abrahão et al., 2018). These discoveries have reopened the debate on the origin and the definition of life. In the absence of scientific consensus on what the TMCV should be exactly, it would be perhaps premature to make the conclusion that ALNs cannot be living particles with the only criteria being their exceptionally small size. Various experimental approaches were developed to address this issue (see below).

The ability of ALNs to develop in the absence of cells (Figure 8) provides additional entry points to discuss the nature of these particles compared to viruses or extracellular vesicles (EVs). It seems worthwhile to underline, at this point, that host-independent morphogenesis is quite unusual in viral world or for EVs, although extracellular morphological plasticity has been reported for ATV viruses *(Acidianius Two-tailed Virus)* that infect archaeons living in particularly harsh aquatic environments (Häring et al., 2005; Prangishvili et al., 2006). ALN morphotype fluctuations that happen in the absence of cells seem at odds with a viral nature of ALNs if viewed as gradual assembly/disassembly processes within a single particle having a pleomorphic lifestyle. However, the alternative, i.e., convergence of otherwise unrelated nanoparticles, toward an “aster-shaped” morphology must also be considered. In this case, morphotype fluctuations could merely reflect survival capabilities of unrelated particles in the absence of cells. Clearly, further studies are required to elucidate morphotype fluctuations related to the exact nature of ALNs.

Sensitivity to a wide range of antibiotics was used as a critical point to establish the non-living nature of biomimetic particles (Raoult et al., 2008). The abundance of ALNs was dramatically affected by biocide agents (norfloxacin, novobiocin, lysozyme or heat shock) (Figure 7). These results could suggest ALNs as self-sufficient forms of life. Differential responses of ALN morphotypes to the multiple damaging treatments should also be considered. 4–10-armed forms appeared more affected than the 11-armed forms, suggesting possibility of more resilient morphotypes within the population of nanoparticles. Comparisons of ALN responses to those of other populations used as controls did not however permit to draw more definite conclusions indicative of the living or non-living nature of these particles.

More basically, the ability of ALN populations to persist in the absence of cells and the sensibility of the particles to biocide agents both raise the question of the existence of endogenous nucleic acids. Hypothesis of an heredity support is also supported by the reoccurrence of different ALN morphotypes whatever was the environmental context or season (see below) and the recurrent radial symmetry of the particles which might reflect a developmental relationship between morphotypes. Flow cytometry (FC) plotting and subsequent TEM analysis of the sorted ALNs provided preliminary insights in this topic. The cytometry step was assessed using permeant cyanine SYBR dyes. These stains preferentially bind to double-stranded DNA, but can also stain single-stranded DNA and RNA with variable efficiency. TEM analyses of sorted fractions showed that SYBR Green I and side scatter signal intensities were morphotype-dependent and allowed to establish a positive correlation between the complexity of morphotypes and the intensity of fluorescence emitted by the particles (Figure 5). Assuming that FC-detected SYBR-staining is indicative for the presence of nucleic acids encased in the nanoparticle (core structure?), highly enriched ALN cultures (0.2 μm filtered) appeared as suitable material from which putative DNA could be directly extracted and characterized at the molecular level. Whole genome sequencing was then developed using the same DNA template. 16S rRNA genes have not been identified as part of the 233 contigs assembled through this approach (Figure 6). Assuming that DNA extraction and amplification were efficient, our data suggests that ALNs lack a detectable genomic features and translation machinery of prokaryotes. The great majority of contigs delivered by whole genome analysis were affiliated to the microviridae, a family of bacteriophages with a single-stranded DNA genome. However, microviridae contigs must be viewed as assemblies of sequence fragments from remnants of viral populations initially comprised in the lake water sample. According to these results, we were not able to demonstrate the presence of nucleic acids in ALNs. Extraction and non-specific amplification efficiencies of nucleic acids are strongly linked to the nature of the particle. Development of a specific protocol to purified ALN enriched-cultures will be a critical point as soon as the exact nature of ALNs will be determined.

Overall, our data on the atypical morphology, the reduced biovolume, the suspected dominant organic nature, the sensibility to biocide treatments, and the ability to develop in the absence of cells indicate than ALNs are new femto-entities which, at the moment, cannot be classified in any known category of femto-entities previously described in environmental samples.

### Ecological Significance of ALNs

Our discovery of ALNs and the existence of other ultra-small non-viral particles raise the ecological question of the accuracy of the “VLP” (i.e., virus-like-particles) fraction in aquatic ecosystems. Commonly used to designate free-occurring viruses, the acronym VLP is also synonymous of “known and yet unknown viral aquatic particles” especially as standardized FC methodologies include heat-driven procedures particularly efficient for detection of viral particles that are, otherwise, refractory or weakly responsive to SYBR-staining (Brussaard, 2004). Interference between ALNs and VLPs in FC particle quantification and successful sorting of largest morphotypes (Figure 5), indicate that ALNs must be viewed as atypical nanoparticles comprised in the VLP fraction. Events recorded from ALNs may lead to overestimate the viral load when analyzing viromes in aquatic ecosystems by counting SYBR-stained particles which is the methodology currently used for optimal detection of viruses by flow cytometry (Weinbauer, 2004). Experimental bias generated by overlapping of fluorescent signals produced by viruses and by other types of nanoparticles encompassed within the viral population was previously assessed in the case of EVs which constitute regular components of VLP fractions in natural environment (Soler et al., 2008, 2015; Forterre et al., 2013). Comparative studies between ecological groups comprising viral communities should therefore be interpreted with caution when pleomorphic nanoparticles such as ALNs occur in samples, notably when seasonal variations favor temporary bloom or predominance of one ALN morphotype over others.

Ecological significance of ALNs was approached by *in situ* seasonal and ecosystemic analyses. Seasonal analyses in a French eutrophic lake revealed a marked seasonal dynamic in ALN abundances from 8.0 ± 3.8 × 10^4^ to 9.0 ± 0.5 × 10^7^ mL^−1^ (Figure 9) and suggest a tight control of the environmental parameters on ALNs. Relative proportions of each morphotype shifted concomitant to fluctuations in total ALN abundance. 11-armed form appeared the alone form in condition of the lowest density of ALNs, suggesting that this peculiar form could be more resistant to adverse environmental factors than others forms. Inverted correlation between 11-armed forms and the others forms, also noted when ALNs were maintained for 36 days in prokaryote-free lake water (in laboratory condition) suggests that these forms may be of importance in maintaining a permanent pool of ALNs in lake water and in promoting propagation of the nanoparticles when growth conditions become more favorable. This assumption is only possible assuming that pleomorphism arises from inter-conversion between morphotypes. The idea that morphotypes described in this study all develop from the same “stem entity” is not demonstrated and remains a fundamental question to be addressed in the future. The importance of ALN degeneration or starvation controlled by environmental factors, which can differently affect the abundances of ALN morphotypes in both controlled and *in situ* conditions, must also be addressed. Such a regulative function by environmental factors has been reported in the case of ultramicro-bacteria (Duda et al., 2012) and in the case of *Phaeodactylum tricornutum*, a 10 μm sized diatom (He et al., 2014).

**Figure 11:**
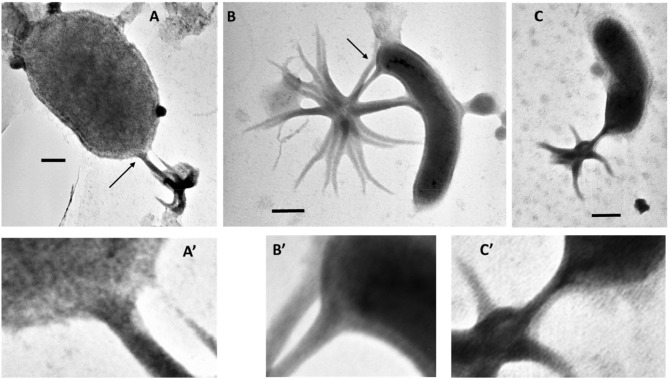
**(A–C)**, Electromicrographs documenting a putative interaction between aster-like nanoparticles (ALNs) and microbial cells. **(A'–C')** are magnified views of sections indicated by arrows in **(A–C)**. Note the close contact between arms of the ALNs and the microbial cells. Scale bars = 100 nm.

Identification of ALNs in a tropical estuarine system and in Saloum river in Senegal (J.C. unpublished data) shows a pan-geographic distribution and adaptability of ALNs. This property prompted us to explore the environmental parameters potentially affecting ALN dynamics at the spatial scale. This was achieved on 16 selected geographical stations from Ha long bay estuary in Vietnam, a highly spatially contrasted environment previously characterized by Pradeep Ram et al. (2018). This spatial survey indicated significant coupling between ALN and prokaryote abundances (Figure 10). No reliable correlation could be established with physico-chemical variables of the bay environment. In contrast, no closed relation between ALNs and prokaryote abundances was recorded at the seasonal scale in the French Lake. However, in this environment, ALNs displayed limited pleomorphism and abundance changes in cell-free medium compared to *in situ* analyses (multiplication factor of 3.6 in cell-free medium compared to 60 in French Lake). These data suggests that microbial communities may help promoting the nanoparticle dynamics. Interestingly, more detailed observations of microbial communities collected from eutrophic lakes revealed arm-mediated contacts between ALNs and bacteria (Figure 11). The role of microbial communities in the control of ALNs and the functional significance of the observed contacts between ALNs and bacteria are still unclear. Further ecological studies of these puzzling nanoparticles should be placed in the context of ecosystemic relationships between ALNs and prokaryotes as well as between ALNs and other biological or physic-chemical components.

Seasonal and spatial dynamics are a characteristic of aquatic microbial communities which regulate energy and matter flows in aquatic systems (Weinbauer, 2004; Diao et al., 2017). To our knowledge, long-term ongoing researches on the ecology and population dynamics of nanobes or non-living particles are currently lacking. This precludes any comparison with our ALN studies. Nevertheless, our observations clearly raise the question of the ecological importance of ALNs in the functioning of aquatic ecosystems. Although reduced on a unit scale, the biomass of total ALNs during bloom periods is likely to mobilize circulating mineral and organic nutrients at the expense (competition?) of other microbial communities of aquatic ecosystems. In addition, direct interplay with bacteria (Figure 11) could significantly influence the energy and material flows mediated by the prokaryotic compartments.

## Conclusion

This study shows, for the first time, that aquatic ecosystems may contain abundant and dynamic nanoparticles of a novel type with ecological potentialities, especially in meso- and eutrophic waters which are predilection sites for ALN detection. Tough the question of the living or non-living nature of ALNs remains unresolved at this time, their original features re-open the debate on the minimal cell volume for a self-sufficient form of life. Experiments are in progress to explore the exact nature of ALNs and identify biotic and abiotic factors involved in regulation of their dynamics in microcosm and environmental conditions. In this context, an upcoming challenge will be to obtain mass cultures of ALN particles grown in VLP-, EV- and prokaryote-free medium. Clearly, we have describe novel types of environmental nanoparticles that, as the most ecological outcome, emphasize that not all virus-like particles observed in aquatic systems are necessarily viruses and that there may be several types of other ultra-small particles in natural waters that are currently unknown but potentially ecologically important.

## Data Availability Statement

The datasets generated for this study are available on request to the corresponding author.

## Author Contributions

JC and HB performed the experiments and the flow cytometric analyses. JC performed the transmission electron microscopy analyses. SB performed the cryo-transmission electron microscopy analysis. ChB and LG performed the scanning electron microscopy analysis. KB and NM performed the EFTEM and EELS analyses. JC, GI, MF, and AP analyzed samples from Ha Long Bay. FE, CoB, and VG realized genomic analyses. JC, HB, TS-N, and BV designed the research and wrote the manuscript. All authors have read, commented, and approved the final version of the manuscript.

### Conflict of Interest

The authors declare that the research was conducted in the absence of any commercial or financial relationships that could be construed as a potential conflict of interest.

## Supplementary Material

Supplementary Data Sheet 1 Contigs from ALN-enriched DNA templates.

## References

[B1] Abrahão J. Silva L. Silva L. S. Khalil J. Y. B. Rodrigues R. Arantes T.. (2018). Tailed giant Tupanvirus possesses the most complete translational apparatus of the known virosphere. Nat. Commun. 9:749. 10.1038/s41467-018-03168-1 29487281 PMC5829246

[B2] Aho K. Kajander E. O. (2003). Pitfalls in detection of novel nanoorganisms. J. Clin. Microbiol. 41, 3460–3461. 10.1128/JCM.41.7.3460-3461.2003 12843126 PMC165317

[B3] Altschul S. F. Madden T. L. Schäffer A. A. Zhang J. Zhang Z. Miller W.. (1997). Gapped BLAST and PSI-BLAST: a new generation of protein database search programs. Nucleic Acids Res. 25, 3389–3402. 10.1093/nar/25.17.3389 9254694 PMC146917

[B4] Benzerara K. Menguy N. Guyot F. Dominici D. Gillet P. (2003). Nanobacteria-like calcite single crystals at the surface of the Tatahouine meteorite. Proc. Natl. Acad. Sci. U.S.A. 100, 7438–7442. 10.1073/pnas.0832464100 12792020 PMC164604

[B5] Benzerara K. Miller V. M. Barell G. Kumar V. Miot J. Brown G. E. Jr.. (2006). Search for microbial signatures within human and microbial calcifications using soft X-ray spectromicroscopy. J. Investig. Med. 54, 367–379. 10.2310/6650.2006.06016 17169258

[B6] Biller S. J. McDaniel L. D. Breitbart M. Rogers E. Paul J. H. Chisholm S. W. (2017). Membrane vesicles in sea water: heterogeneous DNA content and implications for viral abundance estimates. ISME J. 11, 394–404. 10.1038/ismej.2016.134 27824343 PMC5270575

[B7] Borrel G. Joblin K. Guedon A. Colombet J. Tardy V. Lehours A. C.. (2012). *Methanobacterium lacus sp*. nov., isolated from the profundal sediment of a freshwater meromictic lake. Int. J. Syst. Evol. Microbiol. 62, 1625–1629. 10.1099/ijs.0.034538-0 21890730

[B8] Brown C. T. Hug L. A. Thomas B. C. Sharon I. Castelle C. J. Singh A.. (2015). Unusual biology across a group comprising more than 15% of domain Bacteria. Nature 523, 208–211. 10.1038/nature14486 26083755

[B9] Brussaard C. P. (2004). Optimization of procedures for counting viruses by flow cytometry. *Appl. Environ*. Microbiol. 70, 1506–1513. 10.1128/AEM.70.3.1506-1513.2004 PMC368280 15006772

[B10] Buchfink B. Xie C. Huson D. H. (2015). Fast and sensitive protein alignment using DIAMOND. *Nat*. Methods. 12, 59–60. 10.1038/nmeth.3176 25402007

[B11] Castelle C. J. Brown C. T. Anantharaman K. Probst A. J. Huang R. H. Banfield J. F. (2018). Biosynthetic capacity, metabolic variety and unusual biology in the CPR and DPANN radiations. Nat. Rev. 16, 629–645. 10.1038/s41579-018-0076-2 30181663

[B12] Chin C. S. Alexander D. H. Marks P. Klammer A. A. Drake J. Heiner C. (2013). Nonhybrid, finished microbial genome assemblies from long-read SMRT sequencing data. *Nat*. Methods. 10, 563–569. 10.1038/nmeth.2474 23644548

[B13] Cisani G. Varaldo P. E. Ingianni A. Pompei R. Satta G. (1984). Inhibition of herpes simplex virus-induced cytopathic effect by modified hen egg-white lysozymes. Curr. Microbiol. 10, 35–40. 10.1007/BF01576045

[B14] Dar M. A. Sharma A. Mondal N. Dhar S. K. (2007). Molecular cloning of apicoplast-targeted *Plasmodium falciparum* DNA gyrase genes: unique intrinsic ATPase activity and ATP-independent dimerization of Pf GyrB subunit. *Eukaryot*. Cell 6, 398–412. 10.1128/EC.00357-06 PMC1828931 17220464

[B15] Diao M. Sinnige R. Kalbitz K. Huisman J. Muyzer G. (2017). Succession of bacterial communities in a seasonally stratified lake with an anoxic and sulfidic hypolimnion. Front. Microbiol. 8:2511. 10.3389/fmicb.2017.02511 29312212 PMC5735980

[B16] Duda V. I. Suzina N. E. Polivtseva V. N. Boronin A. M. (2012). Ultramicrobacteria: formation of the concept and contribution of ultramicrobacteria to biology. Microbiology 81, 379–390. 10.1134/S0026261712040054 23156684

[B17] Engle E. C. Manes S. H. Drlica K. (1982). Differential effects of antibiotics inhibiting gyrase. *J*. Bacteriol. 149, 92–98. 10.1128/jb.149.1.92-98.1982 PMC216595 6274849

[B18] Folk R. L. (1993). SEM imaging of bacteria and nanobacteria in carbonate sediments and rocks. *J*. Sediment Res. 63, 990–999. 10.1306/D4267C67-2B26-11D7-8648000102C1865D

[B19] Forterre P. Soler N. Krupovic M. Marguet E. Ackermann H. W. (2013). Fake virus particles generated by fluorescence microscopy. Trends Microbiol. 21, 1–5. 10.1016/j.tim.2012.10.005 23140888

[B20] Ghuneim L. J. Jones D. L. Golyshin P. N. Golyshina O. V. (2018). Nano-sized and filterable bacteria and archaea: biodiversity and function. Front. Microbiol. 9:1971. 10.3389/fmicb.2018.01971 30186275 PMC6110929

[B21] Griffin S. Masood M. I. Nasim M. J. Sarfraz M. Ebokaiwe A. P. Schäfer K. H.. (2018). Natural nanoparticles: a particular matter inspired by nature. Antioxidants 7:3. 10.3390/antiox7010003 29286304 PMC5789313

[B22] Häring M. Vestergarrd G. Rachel R. Chen L. Garret R. A. Prangishvili D. (2005). Virology: independent virus development outside a host. Nature 436, 1101–1102. 10.1038/4361101a 16121167

[B23] He L. Han X. Yu Z. (2014). A Rare *Phaeodactylum tricornutum* cruciform morphotype: culture conditions, transformation and unique fatty acid characteristics. PLoS ONE 9:e93922. 10.1371/journal.pone.0093922 24710200 PMC3977982

[B24] Hofer F. Grogger W. Kothleitner G. Warbichler P. (1997). Quantitative analysis of EFTEM elemental distribution images. Ultramicroscopy 67, 83–103. 10.1016/S0304-3991(96)00106-4

[B25] Hug L. A. Baker B. J. Anantharaman K. Brown C. T. Probst A. J. Castelle C. J. (2016). A new view of the tree of life. *Nat*. Microbiol. 1:16048 10.1038/nmicrobiol.2016.48 27572647

[B26] Hyatt D. Chen G. L. Locascio P. F. Land M. L. Larimer F. W. Hauser L. J. (2010). Prodigal: prokaryotic gene recognition and translation initiation site identification. BMC Bioinformatics 11:119. 10.1186/1471-2105-11-119 20211023 PMC2848648

[B27] Kajander E. O. Ciftcioglu N. Aho K. Garcia-Cuerpo E. (2003). Characteristics of Nanobacteria and their possible role in stone formation. Urol. Res. 31, 47–54. 10.1007/s00240-003-0304-7 12669155

[B28] Kéraval B. Lehours A. C. Colombet J. Amblard C. Alvarez G. Fontaine S. (2016). Soil carbon dioxide emissions controlled by an extracellular oxidative metabolism identifiable by its isotope signature. Biogeosciences 13, 6353–6362. 10.5194/bg-13-6353-2016

[B29] King A. M. Q. Lefkowitz E. J. Mushegian A. R. Adams M. J. Dutilh B. E. Gorbalenya A. E. (2018). Changes to taxonomy and the international code of virus classification and nomenclature ratified by the International Committee on Taxonomy of Viruses (2018). *Arch*. Virol. 163, 2601–2631. 10.1007/s00705-018-3847-1 29754305

[B30] Krumsiek J. Arnold R. Rattei T. (2007). Gepard: a rapid and sensitive tool for creating dotplots on genome scale. Bioinformatics 23, 1026–1028. 10.1093/bioinformatics/btm039 17309896

[B31] Lee-Huang S. Maiorov V. Huang P. L. Ng A. Lee H. C. Chang Y. T.. (2005). Structural and functional modeling of human lysozyme reveals a unique nonapeptide, HL9, with anti-HIV activity. Biochemistry 44, 4648–4655. 10.1021/bi0477081 15779891

[B32] Liu Y. Smid E. J. Abee T. Notebaart R. A. (2019). Delivery of genome editing tools by bacterial extracellular vesicles. Microb. Biotechnol. 12, 71–73. 10.1111/1751-7915.13356 30549228 PMC6302703

[B33] Mackey B. M. Miles C. A. Parsons S. E. Seymour D. A. (1991). Thermal denaturation of whole cells and cell components of *Escherichia coli* examined by differential scanning calorimetry. *J. Gen*. Microbiol. 137, 2361–2374. 10.1099/00221287-137-10-2361 1722814

[B34] Manchenko G. P. (1994). Handbook of Detection of Enzymes on Electrophoresis Gels. Boca Raton, FL: CRC Press; Taylor & Francis Group.

[B35] Martel J. Young J. D. (2008). Purported nanobacteria in human blood as calcium carbonate nanoparticles. *Proc. Natl. Acad. Sci*. U.S.A. 105, 5549–5554. 10.1073/pnas.0711744105 PMC2291092 18385376

[B36] Mastronarde D. N. (2005). Automated electron microscope tomography using robust prediction of specimen movements. *J. Struct*. Biol. 152, 36–51. 10.1016/j.jsb.2005.07.007 16182563

[B37] McKay D. S. Gibson E. K. Thomas-Keprta K. L. (1996). Search for past life on Mars: possible relic biogenic activity in martian meteorite ALH84001. Science 273, 924–930. 10.1126/science.273.5277.924 8688069

[B38] National Research Council (1999). Size Limits of Very Small Microorganisms: Proceedings of a Workshop. Washington, DC: The National Academies Press. 25077232

[B39] Ortiz-Alvarez R. Casamayor E. O. (2016). High occurrence of Pacearchaeota and Woesearchaeota (Archaea superphylum DPANN) in the surface waters of oligotrophic high-altitude lakes. *Environ. Microbiol*. Rep. 8, 210–217. 10.1111/1758-2229.12370 26711582

[B40] Pradeep Ram A. S. Mari X. Brune J. Torréton J. P. Chu V. T. Raimbault P. (2018). Bacterial-viral interactions in the sea surface microlayer of a black carbon-dominated tropical coastal ecosystem (Halong Bay, Vietnam). Elem. Sci. Anth. 6, 2–19. 10.1525/elementa.276

[B41] Prangishvili D. Vestergaard G. Häring M. Aramyo R. Basta T. Rachel R. (2006). Structural and genomic properties of the hyperthermophilic archeal virus ATV with an extracellular stage of reproductive cycle. *J. Mol*. Biol. 359, 1203–1216. 10.1016/j.jmb.2006.04.027 16677670

[B42] Quast C. Pruesse E. Yilmaz P. Gerken J. Schweer T. Yarza P.. (2013). The SILVA ribosomal RNA gene database project: improved data processing and web-based tools. Nucleic Acids Res. 41, D590–D596. 10.1093/nar/gks1219 23193283 PMC3531112

[B43] Raoult D. Drancourt M. Azza S. Nappez C. Guieu R. Rolain J. M.. (2008). Nanobacteria are mineralo fetuin complexes. PLoS Pathog. 4:e41. 10.1371/journal.ppat.0040041 18282102 PMC2242841

[B44] Schulz F. Yutin N. Ivanova N. N. Ortega D. R. Lee T. K. Vierheilig J.. (2017). Giant viruses with an expanded complement of translation system components. Science 356, 82–85. 10.1126/science.aal4657 28386012

[B45] Sieburth J. Mc N. Smetacek V. Lenz J. (1978). Pelagic ecosystem structure: heterotrophic compartments of the plankton and their relationship to plankton size fractions. Limnol. Oceanogr. 23, 1256–1263. 10.4319/lo.1978.23.6.1256

[B46] Sillitoe R. H. Folk R. L. Saric N. (1996). Bacteria as mediators of copper sulfide enrichment during weathering. Science 272, 1153–1155. 10.1126/science.272.5265.1153 8662449

[B47] Soler N. Kruprovic M. Marguet E. Forterre P. (2015). Membrane vesicles in natural environments: a major challenge in viral ecology. ISME J. 9, 793–796. 10.1038/ismej.2014.184 25314322 PMC4817693

[B48] Soler N. Marguet E. Verbavatz J. M. Forterre P. (2008). Virus-like vesicles and extracellular DNA produced by hyperthermophilic archaea of the order Thermococcales. Res. Microbiol. 159, 390–399. 10.1016/j.resmic.2008.04.015 18625304

[B49] UniProt Consortium (2019). UniProt: a worldwide hub of protein knowledge. Nucleic Acids Res. 47, D506–D515. 10.1093/nar/gky1049 30395287 PMC6323992

[B50] Uwins P. J. Webb R. I. Taylor A. P. (1998). Novel nano-organisms from Australian sandstones. Am. Mineral. 83, 1541–1550. 10.2138/am-1998-11-1242

[B51] Weinbauer M. G. (2004). Ecology of prokaryotic viruses. FEMS Microbiol. Rev. 28, 127–181. 10.1016/j.femsre.2003.08.001 15109783

[B52] Wu C. Y. Martel J. Wong T. Y. Young D. Liu C. C. Lin C. W.. (2016). Formation and characteristics of biomimetic mineralo-organic particles in natural surface water. Sci. Rep. 6:28817. 10.1038/srep28817 27350595 PMC4923871

[B53] Wurch L. Giannone R. J. Belisle B. S. Swift C. Utturkar S. Hettich R. L.. (2016). Genomics-informed isolation and characterization of a symbiotic Nanoarchaeota system from a terrestrial geothermal environment. Nat. Commun. 7:12115. 10.1038/ncomms12115 27378076 PMC4935971

[B54] Yaghobee S. Mojtaba B. Samiei N. Jahedmanesh N. (2015). What are the nanobacteria? *Biotechnol. Biotechnol*. Equip. 29, 826–833. 10.1080/13102818.2015.1052761

