# Supplementary material for: Discovery of High Abundances of Aster-Like Nanoparticles in Pelagic Environments: Characterization and Dynamics
Source: Front Microbiol. 2019 Oct 15;10:2376. doi: 10.3389/fmicb.2019.02376 (PMC6803438; doi:10.3389/fmicb.2019.02376)
Supplement: Supplementary Data Sheet 1 — Contigs from ALN-enriched DNA templates. [file Data_Sheet_1.PDF]

>000134F|arrow

TCCGCTGGCCTCAATATGGCGATTTCAGCGGAATACGACTCATAGCGCCTTGAATAAGGTAATGAAACGCTATGGAAGAATTTGATAACTTACCG
GCTCAAATTCGTGCTAGGTTCGAAAACGAACCAGCAAACCTGATTGAGTTCTTGCAAGACGAGAAAAATCGACCAGAAGCCGAGAAACTCGGCC
TGGTCGAAAGAGCCATTTCGGAAGAAATGGCGATAAGCACAGTTACTCCACTTGATGTAACTGTGCTAGGTGACACCAACACCAAAAATATCTG
ATAAACGAGGCCAAAAATTATGCTTTATAGAAAACAAACAAACAAGCGCAAAAGCGCTAAATCGTTCCGTAGGAACACTTCAAAAACTAAAGCT
GCAAATATGCAAAAAGCCCCGCAAAGAGGGGGCTGGCGGCTCAATAAAGCGCCAGGCTACCTCACATGGCCTGTTATCACCCACTGACTGCTTA
TTTAAGTAAGCATCAGACAAACTATAAGACCGGCAAATCTTATCGCCGTGTCGCATTCAAAGAATCTGACGAGCATGATCGTCAGATTTCACTGC
CCTGCGGCCAATGCGTTGGCTGCAGGCTAGAAAAATCACGTCAATGGGCCATGCGCTGCATGCATGAAGCCCAATTGCACGAAAAAAACTGCTT
TATAACCCTCACATACAACAATGAAAACCTTCCACAAACTGGATCGCTTGTCAAAAGCGACTTCCAAAAGTTCCTTAAGCGCTTCAGAAAATCCAT
TGCACCTGCAAAATTACGTTACTACATGGCTGGAGAATACGGCACAAGTTTCGGCAGACCTCACTTCCATGCCTGTATCTTCGGATACGATTTTCA
TGATAAGAAACTATTCAAAAGGACTCCCTCTGGTTCTCTCATATATACATCCGACCACCTTGCAACCCTCTGGCCACATGGTTATTCCTCCATTGGA
GACGTTACATTCGAGTCAGCTGCTTACGTTGCTCGATATATTATGCAAAAATACAACGGCCAGATGGAAGAAAACAAACATATAACAAAGGATG
AGCATTACACATACTGTGATAGAAACAGGGGAATTAATAAAGCTATTACCAGAATATAACAATATGAGCCTTAAACCAGGCATTGGTGCTGAGT
GGTACAAAAAATATCGTTCCGACGTATATCCCCATGACTATGTTGTAGTCAACGGAAAAAGGGTAAAACCCCCAAAATACTATGACAAAAAATAT
AAATCAGATTATCCATATGAATACGAAGAATTACTCCACAAACGTGAAACTTCTGCTAAACTCAACCACGAAGACAATACCTATGCCAGACTTGC
CGTAAAGGAAAAAGTCACAAAGGCCAAACTTCAATTATTAAAACGTAACCTCACTTAGGAAATCCTCATGAAATTAGTACTCTGTACCGTTAAAG
ACCGCGCAGCAGATGCGTTCGGTCGTCCAATGTTCGTCCGTTCTATCGGCGAAGCAATCCGGAGCTTTAGCGACGAAGTCCAATCGCCAGAGCG
ATGACAATCAACTTATAACCATTCCCGGACATTTCGACCTATTGAATTAGGCGAATTCGACCGATAAATACGGGTTGTGTCCAATTACATGAACA
ACCCAAACTTGTATCCTTAGGGAAACAAGTCCAAATTACTGATAAAAACTAAGCGTAGAGTAAAAAGGGGGAAACCCCTTTTTCTCACGCAACTA
GGCCTAGGGCTCAAAAAAATGCATCGTAACAAGTCGGTAGACGTCCATCAGTTCACAATGATCCAAAAGCGGATATCCCCCGCTCTACATTTGAC
TGTCAATCAACACATAAAACTACATTCGATGCTGGCTTCCTAGTCCCTGTACTCGTAGACGAAATGTTGCCAGGCGATACATTCCGCTGCAACATG
ACCGCCTTTGCGCGATTGTCTACACCACTCTATCCGATCATGGATAACATGCATCTGGATAGCTTCTTCTTTTTTGTGCCAAATAGACTTATCTGGT
CAAATTGGCAAAAATTTATGGGGCAGCAGGCAAATCCTGCGGACTCGATCTCGTACGTAGTGCCCCAACAAGTAACCCCAGCTGGTGGTTACGC
TATTGGCAGCCTTCAAGATTATATGGGTCTGCCAACCGTAGGCCAAGTAGGTGCTGGTGGCACCGTAAGTCACTGTGCCTTCTGGCCACGTGCTT
ACAACCTT

>000138F|arrow

GAAAGATCAAGATGCAAGTTGTCCATCACAGGCAATAAACGGCTGGCAAAACGTGCAAATGCACGTCATCTTTACACGTGGTATCGCCAGGCAA
TACTTCATCACAATAAATAGGAACTAAAAACCAGCATCAACGTGGTTTTATGAGCATTTGACGTATTAAAACTAGCAGCGGGGAAGCCGCTTTAG
GAACAACTAGCAAATGAATGCGTACTTACTGAACTTATGCGAAAACATAAACATCTCACGTAGTTCCGTACCCATCTTTCGAGTGATACGGTATC
CAAAAAAAACACTTACTCGCCTTCGCGATATCATTACATCTTTTGCACGAGCAATCAACTTAGGGCTTTCCAGTTAGTTCAAAAACACCAGTGGCA
TCGTCAAAAAGACCCCAAATAGAACATATGAAATCATCAGGATGTTTATACAATGATTATCTTCGCTAGCTCGATTGACTTCATCATGAAACTGAC
GTACTGCAACACTTCAGATGCAACCATAAGCTGGACGACCAAAGCATCTGCAGCAGTATCCTTAATAGAAACAATAACCATCTTCATAAAAACTC
CTTAAATAGTACGTTTTAACAATGACAACTTAGCCAACGCAACTTTTTCCTAACAGCAAGTCGCTCAAGCGTGTTGTCCTCATGCCTAGATCGACC
TTCCATCTCTCTGGCAAACTGAATCATATCGAATTCTTCAGGAAACTTCAACTTAAATTTATTATCATAAACCGTGGTGGACGGCACTTTTTGCCAC
GCACCACAACGTGGTCTGACGTATAACGTCTGACATGTACTTATCTAACCACGATTGCCCGATACCGGGCTTCAATGACATCTTATTAAATTCTGG
CTACGCTGAATTATCTCACCAGTCTCTAAATCACAATATTGATAATGGGCACCCGCATCAACCACTTCGTGGTTTTCATTGACAGTAACCCCATTAA
TCTTCTTCATAATATATCTTGCAACATAAGCAGCAGACTCAAAAGTAACATCACCAATTGTAGAATAGCCAAACGGCCACAATTCTTCCAAAATCT
CTGACGTGTAGAGGATAGAGCCAGTCTGCGTTCTTTTAAATATTTTCTTATCCGGAAAATCAAGACCAAACAGACAAGCATGGAAATGAGGACG
ATCAAAAGATTCACCATATTCACCTGCCATATAAAAACGTATCGTTTTCCCAGTAAAACGCTTACGTAACCGCTTCATAAAAAGCTGATAATCATT
GTAATCCAATGACATATCCTTAGGACAATGCTCTGGAGCATATGTCAAAGTAATAAAACAATTACTAGTATGCATTTGTGCCTCATGCATACAAC
GAATCGCCCACTGACGTGAGCGTTCAAGGCGACAACCAACACACTGACACAAGGCAATGATAGGGTACGGACTACATCCGCACCCGGTATTTCC
CGCCAAATTATAGACCTGTCACTGCATTGATAAGCCGTTAAGGGCTTATAACAGGCCATAATTACAAACGATAGCCACCACGCTGGGGAGCGTG
TCTCATATTAATTGACTTCGTCTTACTAGCAGTTCTGCGAAATGACTTTGCAGATTTATATTTGTTTACTGGCTTTCTTCGTAACATGATGAACTCCG
TAGTTAAAATAGTGGTTTGGTGTCACCTAGCACAGTTACATCAAGTAGAGTAACTGTGCTGGCCTCAGGATTTCATCCTTCGGCCTTAGGTGTTTC
TGTAGAAACGAGGGTTCAACCACAGGTTGTCCATCAATAAGACCCAATTGAATCGCTTCATCACGATTCTGGTCGTTCTCAAGGAACTCCAATAA
TTTGACAGGATCATGGTCAAATCGGACTCTTAATTTCGCTGGCAGAGCCATGAAATCGTCCATAGTTGCGTTAATTTGATTCAACGCAGAATGGT
AATCAGTAACACCACTAAAATCGCCGTATTGAGGCGATACAGGGGCCGTTGGAAGTTCCCCTGTAACCCCGAAACGTTCAATGATGACATTAAT
GTCACATTCATCTTTCATATGCTGTTGAGCAAGACTTGGGTCTTGACACAGCAACGCATCGTTTACCGATGCTTCATCTTTATCATAGTTGTACGG
ATTACGTACAAATGGCAAATTCGCTTTACTCATTATTTGACTCCAATTCC

>000156F|arrow

AGCGGACAATCAACTTTATAACCATTCCGACGATTTCGACCTATTTGAATTAGGCGAATTCGACGATAATACGGGTTTGTTCCAATTACATGAACA
ACCCAAACTTGTATCCTTAGGGAAACAAGTCAAAATTACTGATAAAAACTAAGCGTAGAGTAAAAAGGGGGAAACCCCTTTTTCTCACGCAACTA
GGCCTAGGAGCTCAAAAAATGCATCGTAACAAGTCGGTAGACGTCCATCAGTTCACAATGATTCCAAAAGCGGATATCCCCCGCTCTACATTTGA
CTGTCAATCAACACATAAAACTACATTCGATGCTGGCTTCTAGTCCCTGTACTCGTAGACGAAATGTTGCCAGGCGATACATTCCGCTGCAACATG
ACCGCCTTTGCGCGATTGTCTACACCACTCTATCCGATCATGGATAAATGCATTGGATAGCTTCTTCTTTTTGTGCCAAATAGACTTATCTGGTCAA
ATTGGCAAAAATTTATGGGGCAGCAGGCAAATCCTGCGGACTCGATCTCGTACGTAGTGCCCCAACAAGTAACCCCAGCTGGTGGTTACGCTAT
TGGCAGCCTTCAAGATTATATGGGTCTGCCAACTGTAGGCCAAGTAGGTGCTGGTGGCACCGTAAGTCACTGTGCCTTCTGGCCACGTGCTTACA
ACCTTATTTATAACGAATGGTTTCGGGACGAAAACCTTCAAAATTCAGTAGTTGTAGATACTGGCGATGGTCCAGATAACGTAGCCAACTACACA
TTATTACGACGTGGAAAACGTAAAGACTATTTCACGTCAGCATTACCTTGGCCACAAAAGGGCGCAAGCGTTACTTTACCGCTTGGAACATCCGC
CCCAATATTACGCACTAACAATGCGCCTGTTTCCAGACTGTATAACGCTGGAACAAATACATTAAACGCAACCGCCCAGGCTATTAACGTAGGTG
TTACTGGTCAAATTACTGGCGGTGCTGACGGCTTGGCAAAATCATATGATCCTAATGGCGGTTTATATGCAGATTTATACAGCTGCAACCGCTGC
AACAATTAATCCATATGCGTCAAAGCTTCCAGATTCAAAAACTTTTAGAAAGGGACGCCGTGGCGGAACTCGATACACAGAAATTATCCGCAGCC
ATTCGGGGTCGTTAGCCCCGATGCGCGTCTCCAACGGCCTGAATACATTGGAGGCGGTTCAACACACATTAATATCAATCCAATCGCCCAGACGA
ATGGTACCGGAGCTTCCGGGACCACTACTCCTCTCGGTACACTTGGCGCTATGGGTACTGGGCTCGCTCACAATCATGGCTTTACTTATTCAAGC
ACTGAACATGGTGTAATTATCGGTCTCGTTTCAGTACGAGCCGATTTAACATACCAACAAGGTATGCACCGCATGTGGAATCGTTCCACACGTTA
TGATTTTATTTCCCTGCTTTCGCCACTTTGGGCGAACAAGCAGTATTAAATGAAGAAATCTACGTACGAGGCGATGCCAACGATACAGGAGTGTT
TGGATACCAAGAACGTTGGGCAGAATATCGTTATATGCCAAGCCGAATTTCCAGTCTGTTCCGTAGTACGGCAGCTGGAACAATTGACGGCGTG
GCATTTAGCCCCAACGGTTTACAACACTTCCAACTTTGAATAAACACGTTTATTCAAGAAAATCCACCTGTCTCTCGAACCCTTGCGGTCGGAGCA
GCTGCCAACGGCCAGCAAATCATTTTTGATAGCCTTTTTTTGATATAAAAAAGCACGGCCAATGCCAATGTACTCTGTACCTGGCTTAATCGACCA
CTTCTAATGGCACTAGAAGCCGCTGCCTCAGGCGCCGCATCTGGCGCCGCTTTTGGACCTTACGGCTCCTTAATTGGAGCCGGAATAGGTGCGG
CCGCTAGTTATTTTGGTGGTCAAGAACAAAAACGCTGCCCAGCGCACAACAAGCTGCAGCAATGATGCAATTCCAAGATGGTATGCGACGTACT
GCATATCAAGACGCATAGTAGCGGATCTTAAGGCTGCAGGTCTTAACCCTATGCTGGCTTATTCACAAGGCGGAGCCAAAGTCCAGCCTGGTGC
GCAAGCTCCAGTAGGAAATCCACTAGGTGAGGCTGGAAAATTCAGCCCGTGAAGCTGCCATGGCAGTCGCCAATTTTAAACAATTACAACTCAG
AATATCCTGACAC

>000158F|arrow

TGCGAAGCTGACCAAATTTGCCGTATCCCGGCATCTGAGCAATTTCACGAGCTGTATTTGCTCGAGTATATGTTGCCTGATCACGTGATAGATTT
GTATCCGCGTCCGTTTTTTCGGCTTGCGATTGTGTCAGGATATTCTGAGTTTTTAATTGTTTAAAATGGCGGAACTGCCTTGCAGCTTCCACGGGA
TGAATTTCCAGCCTCCACCTAGGGATTTTCTACTGAGCTGCGCAACCCGGCTGGACTTTGGTCCGCCTGTGAAATAAGCCCGCATAGGGTAGACC
TGCAGCCTTAAGATACGCTTTTACTGCGTCTGATATGCAGTAAGTTCGCATAACCATCTTGGAATTGCATTCATGTGCAGCTTTGTTGTGCGCTGG
CAGCGTTTGTTCTTGACCACCAAAATAACTAGCTGTGCCAGCACCTATTCCGGTCCAATTAAGGAGCAGTAAGGTCCAAAAGCGGCGCCAGATG
CGGCGCCTGAGGCAGCGGCTTCTAGTGCCATTAGAAGTGGTCGATTAAGCCAGGTACAGAGTACATTGGCATTGGCCGTGCTTTTTTAATATCA
AAAAAGCTATCAAAAATGATTTGCTGGCCGTTGGCAGCTGCTCCGACCGCAAGGGTTCGAGAGACAGGTGGATTTTCTTGAATAAACGTGTTAT
TCAAAGTTGGAAGTGTTGTAAACCGTTGGGCTAAATGCCAGCCGTCAATTGTTCCAGCTGCCGTACTACGGAACAGACTGGAAATTCGGCTTGG
CATATAACGATATTCTGCCCAACGTTCTTGGTATCCAAACACTCCTGTATCGTTGGCATCGCCTCGTACGTAGATTTCTTCATTTAATACTGCTTGT
TCGCCCAAAGTGGCGAAAGCAGGGAAATAAAATCATAACGTGTGGAACGATTCCACATGCGGTGCATACCTTGTTGGTATGTTAAATCGGCTCG
TACTGAAACGAGACCGATAATTACACCATGTTCAGTGCTTGAATAAGTAAAGCCATGATTGTGAGCGAGCCCAGTACCCATAGCGCCAAGTGTA
CCGAGAGGAGTAGTGGTCCCGGAAGCTCCGGTACCATTCGTCTGGGCGATTGGATTGATATTAATGTGTGTTGAACCGCCTCCAATGTATTCAG
GCCGTTGGAGACGCGCATCGGGGCTAACGACCCCGAAATGGCTGCGGATAATTTCTGTGTATCGAGTTCCGCCACGGGCGTCCCTTTCTAAAAG
TTTTTGAATCTGGAAGCTTTGACGCAATTGATTAATTGTTGCAGCGGTTGCAGCTGATAAATCTGCATATAAACCGCCATTAGGATCATATGATTT
TGCCAAGCCGTCAGCACCGCCAGTAATTTGACCAGTAACACCTACGTTAATAGCCTGGGCGGTTGCGTTTAATGTATTTGTTCCAGCGTTATACA
GTCTGGAAACAGGCGCATTGTTAGTGCGTAATATTGGGGCGGATGTTCCAAGCGGTAAAGTAACGCTTGCGCCCTTTTGTGGCCAAGGTAATGC
TGACGTGAAATAGTCTTTACGTTTTCCACGTCGTAATAATGTGTAGTTGGCTACGTTATCTGGACCATCGCCAGTATCTACAACTACTGAATTTTG
AAGGTTTTCGTCCCGAAACCATTCGTTATAAATAAGGTTGTAAGCACGTGGCCAGAAGGCACAGTGACTTACGGTGCCACCAGCACCTACTTGG
CCTACAGTTGGCAGACCCATATAATCTTGAAGGCTGCCAATAGCGTAACCACCAGCTGGGGTTACTTGTTGGGGCACTACGTACGAAATCGAGT
CCGCAGGATTTGCCTGCTGCCCCATAAATTTTTGCCAATTTGACCAGACAAGTCTATTTGGCACAAAGAAGAAGAAGCTATCCAAATGCATATTG
TCCATGATCGGATAGAGTGGTGTAGACAATCGCGCAAAGGCGGTCATGTTGCAGCGGAATGTATCGCCTGGCAACATTTCGTCTACGAGTACAG
GGACTAGAAAGCCAGCATCGAATGTAGTTTTATGTGTTGATTGACAGTCAAATGTAGAGCGGGGGATATCCGCTTTTGGAATCATTGTGAACTG
ATGGACGTCTACCGACTTGTTACG

>000159F|arrow

GCTCTATATTTGGATTTTCATCCAATTTGTTTAAGTATCAGCAGTAAATTATCTGTTTGATTACTTGTGAGTTCTGTTTGAGCTTCAATTTGTTTAGT
TGGCATAGATGATTTGCTTGCGCAACCGTTTGATAAGCTTGGGTTCCGGACGTAGTTGCATTACCGAGAACATTTGCATATGGGCCATAGCTCAG
CTGGTGTTGTGGCTCGCCTTGTGAATACGCAAGCATGGGATTTAACCCAGCTTTTTTCATATCTTCAACTGCTCGTGATATGATGTTCCAGACATT
TCAGCTTGAAATCTCTGTTGATTACTCGCTGTTCTGCACTCCGCTGCGTTTTGACTTTGTGTTCCAAAGTATTGTCCTGTGCACCTATGCTGCAGGT
GCAAGTGCAGCTAAGGAGAGTCCCCAGTAGCAGGGGCAGTCCTATAGCTATAGCAGGGCCGATTAAATCGGCAATTCCGTCAAATAGTCCATTA
GAAATGGTCTATTAAGCCAGGTACTGAATAACATTGGCATTGGTCTTGCCATTTTGACATCAAAAAATGAGTCAAATAAGAATTGTTGGCCATTT
GCAGCTGCTCCAACGGCTAACACGTGATACAGGAGGCGTATCTTGAATAAACGTATTATTCAAAGTAGGCGCAGCGGTAAATTTTTGAGCCAAA
TGCCAACCATCGATCGTGCCCGCCGATGTTGATTTGAACAAACAGTAATTTTAGATGGTTTGTAACGATATTCCGCCCAGCGTTCGTGATATCCAA
AAAACAGTATTGTCGGCTGCAGTATTCCTGTTGCATAAATTTCTTTTTGCAAAACAGATTGTTCGCCTAGAGTAGCAAATGCTGGGAAATAAAAG
TCATATCGTGTAGATCTAGACCCATACGGTCTAATCCTTGTGATAAGTAAGATCTGCTCTAATGGATACTAATCCAATAATAACGCCATGCTCAGT
AAATGATTGAGTAAATCCATGATTATGAGCGAGAGCCAGTACCCATAGCAGCAAGGTTGCCCAAAGGGGTAGTCGTTCCAGAAGCGTTTGTTCC
CGACGTTTGAGCAATCGGATTAACATTAATTGGTGTTGAACCGCCTCCAAGGTATTCAGGCCTTTGTAAACGGGCGTCTGGGGAAATAACTCCAA
AGTGACTCCGGATAATTTCTGGTATCGTGTTCCGCCACGTGCATCCCTTTCAAGTAATTTTTGAATTTGAAATGACTGTCTTAATTGATTGACAGTT
GCAGCAGTGCTTCTGATAAGTCGGTATATAAATTAGAAACGTTATTTACTACACCAGCAGTATTACACCATAAGCGTTGCATACTAGCTAAAGCA
GTCGTATTTCCAGGATCTGTTTGAATTACCGTAAATTTATCGTTTGATGTTGCGTCTCCTGAAATGGTATCCATTTAATTGGAGCCGTAGTACCTAA
AGGTAAGGTGACACTCGCACTTTTTGTGGCCATGGTAATGCTGACGTAAAGTAATCATGTCTTTTACCACGACGTTTTAGCACATAGTTTGAGGA
AGTATCAGGGCCATCGCCCTTATCAACTACTGCGATTGTTTGTAAATTTTCATCTCGGAACCATTCGTTCCAATAAGATTGTATGCACGTGGCCAA
AAGGCACAGTGCGTAATAGTTCGGCCAGTATCAATTTGGCCTACTGTTGGTAAGCCCATATAGTCTTGAAGGCTGCCTACGGCATAACCATCTGT
TGGCTTGTTTGGTTGGGACAATATAGATATTGAGTCTGTTGGATTTTCTTGTTGACCCATAAATTTTGCCAGTTATTCCATATAAGGCGATTGGGT
ACAAAGAAAAAGAAAGAATCCATAATCATGTTGTCCATGATTGGATATAAAGGCGTGCTAGACGGGCAAATGCCGTCATTTTTAAGTTGAAAGT
GTCCCCAGGGAGCACTTCATCAACATATACAGGAATTAAATAGCCCGCATCGAAAGTCGTTTATGGTTTTTGAGCATCGAATTTACTACGTGGT
ATATAGGCTCTAGGTACCATCGCGAAGCGGTGAGTATTAACTGACTGATTGCGGTGCATGTTTTTCCTTAGTGGTGTTCCGGGGGAAAGATAAA
TCTCTTTCCCCTCGGTTGTTTTATTTAAGTTTAACTTGTTTTCCTAATGATAGGAGT

>000160F|arrow

TATTTAATTCTCTTAAAGCACCAACAGCAGTTTGAAAAACACTATAACCAGCAGTACCATTGCCAGCTGGTATGTCAGAATAAATATTAGCACGTG
TTCCTAACGGTAAAGAAACAGAAGCACCTTCTGTGGCCAAGGCAATGCACCAGTAAAATAATCTTTACGCTTACCACGTCTCAAAGTGTATAGTT
AGCAGGAACATCACCTGAATCACCAAGATTTAACGTTACAGAATTCTGTAAGTTTTCATCTAAACCATTCGTTATAAATCTCATTATAAGCACGTA
ACGGCAAAACATTATGCGTTACTGTATTAGCGCCAGTAATTCTGACCAGCAGTAGGTAAACCAAAATAATCAAAAATTGACCAAACAGCATAACC
ACCAGCAGGACTAGTAATAGTAGGCACTACAAAGAAAATAGAATCACCAGGATTCGTTGTTCACATAAACTTGGCAATGTTCCAAAGTAACAGT
TAGGTACAAAGAAAAGAAAGTATCAAGATGCAAGTTGTCCATCACAGGAAATAACGGTGTGGCCAAACGTGCAAATGCAGTCATCTTTACACGA
TGAGTATCGCCAGGCAATACTTCATCACAATAAATAGGAACTAAAAAACCAGCATCAAACGTGGTTTTATGAGCATATTGAGTATTAAAACTAGA
GCGGGGAATGTCCGCTTTAGGAACCATAGCAAATGAATGCGTACTTACTGACTTATTGCGAAACATAAACATCTCCCGTAGTTCCGTACCACTCT
TTCGAGTGATACGGTATAAAAAAAACCTTACTCGCCTTCGCGAATCATTACATCTTTTGCACGAGCAATCAACTTAGGGCTTTCCAGTAGTTCAAA
AACACCAGTGGCATCGTCAAAAAGACCCAAATAGAACATATGAAAATCATCAGGATGTTTATACAATGATTATCTTCGCTAGCTCGATTGACTTC
ATCCTGAAACTGACGTACTGCAACACCTTCAGATGCAACATAAGCTGGACGACCAAAAGCATCTGCAGCAGTATCCTTAATAGAAACAATAACCA
TCTTCATAAAAACTCCTTAAATAGTACGTTTTAACAATGACAACTTAGCCAACGCAACTTTTTCCTTAACAGCAAGTCGCTCAAGCGTGTTGTCCTC
ATGCCTAGATCGACCTTCCATCTCTCTGGCAAACTGAATCATATCGAATTCTTCAGGAAACTTCAACTTAAATTTATTATCATAAAACCGTGGTGG
ACGGCACTTTTTGCCACGCACCACAACGTGGTCTGACGTATAAACGTCTGACATGTACTTATCTAACCACGATTGCCCGATACCGGGCTTCAATG
ACATCTTATTAAATTCTGGCTTACGCTGAATTATCTCACCAGTCTCTAAATCACAATATTGATAATGGGCACCCGCATCAACCACTTCGTGGTTTTC
ATTGACAGTAACCCCATTAATCTTCTTCATAATATATCTTGCAACATAAGCAGCAGACTCAAAAGTAACATCACCAATTGTAGAATAGCCAAACGG
CCACAATTCTTCCAAAATCTCTGACGTGTAGAGGATAGAGCCAGTCTGCGTTCTTTTAAATATTTTCTTATCCGGAAAATCAAGACCAAACAGACA
AGCATGGAAATGAGGACGATCAAAAGATTCACCATATTCACCTGCCATATAAAAACGTATCGTTTTCCAGTAAAACGCTTACGTAACCGCTTCAT
AAAAAGCTGATAATCATTGTAATCCAATGACATATCCTTAGGACAATGCTCTGGAGCATATGTCAAAGTAATAAAACAATTACTAGTATGCATTT
GTGCCTCATGCATACAACGAATCGCCCACTGACGTGAGCGTTCAAGGCGACAACCAACACACTGACCACAAGGCAATGATAGGGTACGGACTAC
ATCCGCACCCGGTATTTCCCGCCAAATTATAGACCTGTCACTGCATTGATAAGCCGTTAAGGGCTTATAACAGGCCATAATTACAAACGATAGCC
ACCACG

>000171F|arrow

ATACTGTAATTGTTTTATTACTTTGACATATGCTCCAGAGCATTGTCTAAGGATATGTCATTGGATTACAATGATTATCAGCTTTTATGAAGCGGTT
ACGTAAGCGTTTACTGGGAAAACGATACGTTTTTATATGGCAGGTGAATATGGTGAATCTTTGATCGTCCTCATTTCCATGCTTGTCTGTTTGGTC
TGATTTTCCGGATAAGAAAATATTTAAAAGAACGCAGACTGGCTCTATCCTTACACGTCAGAGATTTGGAAGAATTGTGGCCGTTTGGTATTCT

ACAATGGTGATGTTACTTTTGAGTTGATGCGTTATGTTGCAAGATATATTATGAAGAAGATTAATGGGGTTATGTCAATGAAAACCACGAAGTG
GTTGATGCGGGTGCCCATTATCAATATTGTGATTAGAGATGGTGAGTAATTCAGCGTAAGCCAGAATTTAATAGATGTCATTGAAGCCCGGTATC
GGGCAATCGTGGTTAGATAAGTACTGTCAGACGTTTATACGTCAGACCACGTTGTGGTGCGTGGCAAAAAGTGCCGTCCACCACGGTTTATGAT
AATAAATTTAAGTTGAAGTTTCCTGAAGAATTCGATATGATTCAGTTTGCCAGAGAGATGGAAGGTCGATCTAGGCATGAGGACAACACGCTTG
AGCGACTGCTGTTAAGGAAAAAGTTGCGTTGGCTAAGTTGTCATTGTTAAAACGTACTATTAAGGAGTTTTTATGAAGATGGTTATTGTTTCTATT
AAGGATACTGCTGCAGATGCTTTGGTCGTCCAGCTTATGTTGCATCTGAAGGTGTTGCAGTACGTCAGTTTCAGGATGAAGTCAATCGAGCTAGC
GAAGATAATCAGTTGTATAAACATCCTGATGATTTTCATATGTTCTATTTGGGTCTTTTTGACGATGCACTGGTTTTTGAACTACTGGAAAGCCCT
AAGTTGATTGCTCGTGCAAAAGATGTAATGATTCGCGAAGGCGAGTAAGGTTTTTTTATACCGTATCACTCGAAAGAGTGGTACGGAACTACGG
GAGATGTTTATGTTTCGCAATAAGTCAGTAAGTACGCATTCATTTGCTATGGTTCCTAAAGCGGACATTCCCCGCTCTAGTTTTAATACTCAATAT
GCTCATAAAACCACGTTTGATGCTGGTTTTTTAGTTCCTATTTATTGTGATGAAGTATTGCCTGGCGATACTCATCGTGTAAAGATGACTGCATTT
GCACGTTTGGCCACACCGTTATTTCCTGTGATGGACAACTTGCATCTTGATACTTTCTTTTTCTTTGTACCTAATCGTTTACTTTGGAACAATTGGCC
AAAGTTTATGGGTGAACAAACGAATCTGGTGATTCTATTTCTTTTGTAGTGCCTACTATTACTAGTCCTGCTGGTGGTTATGCTGTTTGTTCAATTTT
TTGATTATTTTGGTTTACCTACTGCTGGTCAGATTACTGGCGCTAATACAGTAACGCATAATGTTTTGCCGTTACGTGCTTATAATGAGATTTATAA
CGAATGGTTTAGAGATGAAAACTTACAGAATTCTGTAACGTTAAATTGGTGATTCAGGTGATGTTCCTGCTAACTATACACTTTTGAGACGTGGT
AAGCGTAAAGATTATTTTACTGGTGCATTGCCTTGGCCACAGAAGGGTGCTTCTGTTTTTTACCGTTAGGAAACACGTGCTAATTTATTCTGACAT
ACCAGCTGGCAATGGTATGCTGGTTATAGTGTTTTTCAAACTGCTGTTGGTGCTTTAAGAGAATTAAATCAGCTTCTAATACTTTGTCTAATAGTA
CAAATGCTGGTGTTGCTACTAATCAGTTATACGCTGATTTGTCTACTGCTACTGCTGCGACTATTAACCAACTTCGTCAATCTTTCCAGATTCAGAA
GTTATTGGAGCGCGATGCACGTGGTGGTACTCGTTATACTGAGTTACTACGTGCTCACTTTGGAGTAACTCCACAGGATTATCGTTTACAACGTC
CTGAATATATTGGTGGAGGTTCGACCTTGTTAATGTTAATCCGATTGCTCAGACTTCTGCAACGTCGGTTACTGGTTCTGCTACTCCGCAAGGTAA
CCTTGCTGCAATGGGTACTGCATTGGCTCAGGGACACGGCTTTACGTATGCTGCTCAAGAACATGGATACATTATCGGATTA

>000174F|arrow

TGCTGTGCAAAAGATGTAATGATTCGCGAAGGCGAGTAAGGTTTTTTTATACCGTATCACTCGAAAGAGGGGTACTGAACTACGGGCGATGTT
TATGTTTCGCAATAAGTTCAGTAAGTACAGCATCATTGCTATTGTTCCTAAAAGCGGACATTCCCCGCTTCTAGGTTTTAATAATTCAATATGCTCA
TAAAACCACGGTTGATGCTGTGTTTAGTTCCTATTTATGTGATGAAGTATGCCTTGAGATACTCATGTGTAAAAGATGACTGCATTTGCACGTTGG
GGGCCACACGTTTTTCCTGTTAGGACAACTGCATCTTGATACTTCTTTTTCTTTGTAACAATCGTTATTTGGAACAATTGGCCAAAGTTTATGGGTG
TGAACAAACGCAATCTGGTGATTCTTTTTCTTTTGTAGTGGTACTATTACTAGTCCTGCTGGGTGTTATGCTGTTTGTTCAATTTTTGATTATTTTGG
TTTACCTACTGCTGGTCAGATTACTGGCGCTAATACAGTAACGCATAATGTTTTGCCGTTACGTGCTTATAATGAGATTTATAACGAATGGTTTAG
AGATGAAAACTTACAGAATTCTGTAACGTTAAATCTTGGTGATTCAGGTGATGTTCCTGCTAACTATACACTTTTGAGACGTGGTAAGCGTAAAG
ATTATTTTACTGGTGCATTGCCTTGGCCACAGAAGGGTGCTTCTGTTTCTTTACCGTTAGGAACACGTGCTAATATTTATTCTGACATACCAGCTG
GCAATGGTACTGCTGGTTATAGTGTTTTTCAAACTGCTGTTGGTGCTTTAAGAGAATTAAATTCAGCTTCTAATACTTTGTCTAATAGTACAAATG
CTGGTGTTGCTACTAATCAGTTATACGCTGATTTGTCTACTGCTACTGCTGCGACTATTAACCAACTTCGTCAATCTTTCCAGATTCAGAAGTTATT
GGAGCGCGATGCACGTGGTGGTACTCGTTATACTGAGTTACTACGTGCTCACTTTGGAGTAACTCCACAGGATTATCGTTTACAACGTCCTGAAT
ATATTGGTGGAGGTTCGACCCTTGTTAATGTTAATCCGATTGCTCAGACTTCTGCAACGTCGGTTACTGGTTCTGCTACTCCGCAAGGTAACCTTG
CTGCAATGGGTACTGCATTGGCTCAGGGACACGGCTTTACGTATGCTGCTCAAGAACATGGATACATTATCGGATTAGTTTCTGTACGTGCTGAC
CTCACATATCAACAGGGTCTTCCTAAGATGTGGTCTAGGTCTACACGATATGACTTTTATTTCCCAGTATTTGCCACTTTGGGTGAGCAAGCTATT
TTGAACAAAGAAATTTATGTTCAAGGTACTGCAGCCGACAATGATGTATTTGGTTATCAAGAACGTTGGGCGGAGTATCGTTACAAACCTTCTCA
AATTACTGGTTTCTTTAGGTCTACTTCTGCTGGCACTATTGATGCTTGGCATTATGGACAGCGATTTACTTCTCTTCCTACGTTGAATTCAACGTTT
ATTCAAGAGACCCCTCCAGTTGCTCGTACTACGGCGGTCGGAGCTGCAGCAAATGGTCAGCAATTTTTAATGGATGCTTTCTTTGATTGTCAGAT
GGCCAGACCTATGCCTATGTACAGCGTACCTGGTCTAATTGATCATTTCTAATGTTTTATATAACCTCGACTACTCCGTAAGGAGTAGTGAGGAAA
CAACCGAAGGGCGTTAGTTTATGTTTGGTGGAATACTTGATGCGGTTACTAATGTTGGTTCTAAGCTGTCTTCAGCTTCTAGTTTCTTTACTCCTG
GTGTCGGTATGCTTTGGGCGCTGTTGGTTCTTATTTAGGTTCTACTTCTGCTAATAAAGCTAATCAGGAGATGGCTCAGAGGCAAATGGATTTTC
AAGCCGATATGAGTGGAACAAGTTACCAGCGTGCTGTTAAAGATTTAGAAGCTGCTGGTTTATCTCCTATGTTAGCCTATCAACGTGGTGGTGCT
TCTACCCCATCTGGTTCAACTGCTACTATGGAAAATGTTTTAGGTAATGCAACTAATTCAGCTATTAATACTGCTTCTATGATGCAACAGATTCGTA
ATGCATCAGA

>000181F|arrow

CGTTTCTTAAAGCAATATCAGCCAAAATACGTTTATTTTCGGCTGTAATATTAGGAGCAGTAAGCAACTTATTAACAGTATCAGCCTAGTATTAGC
GGTACCAGCTTCAGTAGCTGTCAGTCTGGGCGATAATCTCGCTTTTTGTTTCTGATGCATTACGAATCTGGTTGCATCATAGAAGCAGTATTAATA
GCTGAATTAGTTTGCATTACCTAAAACATTTTCCATAGTAGCAGTTGAACCAGATGGGGTAGAAGCACCACCACGTTGATAGGCTAATCATAGGA
GATAAACCAGCAGCTTCTAAATCTTTAACAGCACGCTGGTAACTTGTTCCATCATATCGGCTTGAAAATCCATTTGCCTCTGAGCCATCTCCTGATT
AGCTTTATTAGCAGAAGTAGAACCTAAATAAGAACCAACAGCGCCCAAAGCAGTACCGACACCAGGAGTAAAGAAACTAGAAGCTGAAGACAG

CTTAGAACCAACATTAGTAACCGCATCAAGTATTCCACCAAACATAAACTAACGCCCTTCGGTTGTTTCCTCACTACTCCTTACGGAGTAGTCGAG
GTTATATAAAACATTAGAAATGATCAATTAGACCAGGTACGCTGTACATAGGCATAGGTCTGGCCATCTGACAATCAAAGAAAGCATCCATTAAA
AATTGCTGACCATTTGCTGCAGCTCCGACCGCCGTAGTACGAGCAACTGGAGGGGTCTCTTGAATAAACGTTGAATTCAACGTAGGAAGAGAAG
TAAATCGCTGTCCATAATGCCAAGCATCAATAGTGCCAGCAGAAGTAGACCTAAAGAAACCAGTAATTTGAGAAGGTTTGTAACGATACTCCGC
CCAACGTTCTTGATAACCAAATACATCATTGTCGGCTGCAGTACCTTGAACATAAATTTCTTTGTTCAAAATAGCTTGCTCACCCAAAGTGGCAAA
TACTGGGAAATAAAAGTCATATCGTGTAGACCTAGACCACATCTTAGGAAGACCCTGTTGATATGTGAGGTCAGCACGTACAGAAACTAATCCG
ATAATGTATCCATGTTCTTGAGCAGCATACGTAAAGCCGTGTCCCTGAGCCAATGCAGTACCCATTGCAGCAAGGTTACCTTGCGGAGTAGCAGA
ACCAGTAACCGACGTTGCAGAAGTCTGAGCAATCGGATTAACATTAACAAGGGTCGAACCTCCACCAATATATTCAGGACGTTGTAAACGATAA
TCCTGTGGAGTTACTCCAAAGTGAGCACGTAGTAACTCAGTATAACGAGTACCACCACGTGCATCGCGCTCCAATAACTTCTGAATCTGGAAAGA
TTGACGAAGTTGGTTAATAGTCGCAGCAGTAGCAGTAGACAAATCAGCGTATAACTGATTAGTAGCAACACCAGCATTTGTACTATTAGACAAA
GTATTAGAAGCTGAATTTAATTCTCTTAAAGCACCAACAGCAGTTTGAAAAACACTATAACCAGCAGTACCATTGCCAGCTGGTATGTCAGAATA
ATATTAGCACGTGTTCCTAACGGTAAAGAAACAGAAGCACCCTTCTGTGGCCAAGGCAATGCACCAGTAAAATAATCTTTACGCTTACCACGTCT
CAAAAGTGTATAGTTAGCAGGAACATCACCTGAATCACCAAGATTTAACGTTACAGAATTCTGTAAGTTTTCATCTCTAAACCATTCGTTATAAAT
CTCATTATAAGCACGTAACGGCAAAACATTATGCGTTACTGTATTAGCGCCAGTAATCTGACCAGCAGTAGGTAAACCAAAATAATCAAAAATTG
AACAAACAGCATAACCACCAGCAGGACTAGTAATAGTAGGCACTACAAAAGAAATAGAATCACCAGGATTCGTTGTTCACCCATAAACTTTGGC
CAATTGTTCCAAAGTAAACGATTAGGTACAAAGAAAAAGAAAGTATCAAGATGCAAGTTGTCCATCACAGGAAATAACGGTGTGGCCAAACGT
GCAAATGCAGTCATCTTTACACGATGAGTATCGCCAGGCAATACTTCATCACAATAAATAGGAACTAAAAAACCAGCATCAAACGTGGTTTTATG
AGCATATTGAGTATTAAAACTAGAGCGGGGAATGTCCGCTTTAGGAACCATAGCAAATGAATGCGTACTTACTGACTTATTGCGAAACATAAAC
AT

>000191F|arrow

GATACTTCTTTTTCTTTGTACCTAATCGTTTACTTTGGAACAATTGGCCAAAGTTTATGGGTGAACAAAACGAATCCTGGTGATTCATTTCTTTTGT
AGTGCTACTATTACTAGTCCTGCTGGTGGTTATGCTGTTTGTTCAATTTTTGATATTTTGGTTACTACTGATGGTCAGATTACTGGCGCTAATACAG
TAACGCATAATGTTTTGCCGTTACGTGCTTATAATGAGATTTATAACGAATGGTTTAGAGATGAAAACTTACAGAATTCTGTAACGTTAAATCTTG
GTGATTCAGGTGATGTTCCTGCTAACTATACACTTTTGAGACGTGGTAAGCGTAAAGATTTTACTGGTGCATTGCCTTGGCCACAGAAGGGTGCT
TCTGTTTCTTTACCGTTAGGAACACGTGCTAATATTTATTCTGACATACAGCTGGCAATGGTACTGCTGGTTATAGTGTTTTTCAAACTGCTGTTGG
TGCTTTAAGAGAATTAAATTCAGCTTCTAATACTTTGTCTAATAGTACAAATGCTGGTGTTGCTACTAATCAGTTATACGCTGATTTGTCTACTGCT
ACTGCTGCGACTATTAACCAACTTCGTCAATCTTTCCAGATTCAGAAGTTATTGGAGCGCGATGCACGTGGTGGTACTCGTTATACTGAGTTACTA
CGTGCTCACTTTGGAGTAACTCCACAGGATTATCGTTTACAACGTCCTGAATATATTGGTGGAGGTTCGACCCTTGTTAATGTTAATCCGATTGCT
CAGACTTCTGCAACGTCGGTTACTGGTTCTGCTACTCCGCAAGGTAACCTTGCTGCAATGGGTACTGCATTGGCTCAGGGACACGGCTTTACGTA
TGCTGCTCAAGAACATGGATACATTATCGGATTAGTTTCTGTACGTGCTGACCTCACATATCACAGGGGTCTTCCTAAGGTGGTCTAGGTCTACAC
GATATGACTTTTATTTCCCAGTATTTGCCACTTTGGGTGAGCAAGCTATTTTGAACAAAGAAATTTATGTTCAAGGTACTGCAGCCGACAATGATG
TATTTGGTTATCAAGAACGTTGGGGGAGTATCGTTACAAACCTTCTCAAATTACTGGTTCTTTAGGTCTACTTCTGCTGGCACTATTGATGCTTGG
CATTATGGACAGCGATTTACTTCTCTCCTACGTTGAATTCAACGTTTATTCAAGAGACCCCTCCAGTTGCTCGTACTACGGCGGTCGGAGCTGCAG
CAAATGGTCAGCAATTTTAATGGATGCTTCTTTGATTGTCAGATGGCCAGACCTATGCCTATGTACAGCGTACCTGGTCTAATTGATCATTTCTAA
TGTTTTATATAACCTCGACTACTCCGTAAGGAGTAGTGAGGAAACAACCGAAGGGCGTTAGTTTATGTTTGGTGGAATACTTGATGCGGTTACTA
ATGTTGGTTCTAAGCTGTCTTCAGCTTCTAGTTTTTACTCCTGGTGTCGGTACTGCTTTGGGCGCTGTTGGTTCTTATTTAGGTTCTACTTCTGCTAA
TAAAGCTAATCAGGAGATGGCTCAGAGGCAAATGGATTTTCAAGCCGATATGAGTGGAACAAGTTACCAGCGTGCTGTTAAAGATTTAGAAGCT
GCTGGTTTATCCTATGTTAGCCTATCAACGTGGTGGTGCTTCTACCCCATCTGGTTCAACTGCTACTATGGAAAATGTTTTAGGTAATGCAACTAA
TTCAGCTATTAATACTGCTTCTATGATGCAACAGATTCGTAATGCATCAGAAACAGAAAAGCAGATTATCGCCCAGACTGAAGCTACTGAAGCTG
GTACCGCTAATACTAGGGCTGATACTGTTAATAAGTTGCTTACTGTCCTAATATTACAGCCGAAAATAAAACGTATTTTGGCTGATATTGCTTTAA
AGAATACGACTGCGGATTTAACATCGCTCAGTCTAAATACTAAGAGGCTATTGGCTCCATCCCAGCTATTTGGTCTAGGGGTATCGATGCTTCGA
AAGAAATTTTGATAAACTCAAAAATAATCCTAATCAACTAACCCCTTGGGGAATTGGAGTCAAATAATGAGTAAAGCGAATTTGCCATTTGTACG
TAATCCGTAAACTATGATAAAGATGAAGCATCGGTAAACGATGCGTTGCTGTGTCAAGACCCAAGTCTTGCTCAACAGCATA

>000195F|arrow

GTTACTACTTGATGTAACTGTGCTAGGTGCCACCAAACCCACTATTTTAATCCGGAGTTCATCATGTTCGAAGAAAGCAGTAAACCAATATAAATC
TGCAAAGGTCATTTCGCAGGAACTGCTAGTAAGACGAAGTCAATTAATATGAGACCACGCTCCCCAGCGTGGTGGCTATCGTTTGTAAATTATG
GCCTGTTATAAGCCCTTAACGGCTTATACAATGCAGTGACAGGTCTATAATTGGCGGGAAATACCGGGTGCGGATGTAGTCCGTACCCTATCATT
GCCTTGTGGTCAGTGTGTTGGTTGTCGCCTTGAACGCTCACGTCAGTGGGCGATTCGTTGTATGCATGAGGCACAAATGCATACTAGTAATTGTT
TATTACTTTGACATATGCTCAGAGCATTGTCCTAAGGATATGTCATTGGATTACAATGATTATCAGCTTTTTATGAAGCGGTTACGTAAGCGTTTT
ACTGGGAAAACGATACGTTTTATATGGCAGGTGAATATGTGAATCTTTTGATCGTCCTCATTTCCATGCTTGTCTGTTTGGTCTTGATTTTCCGGAT

AAGAAATATTTAAAGAACGCAGACTGGCTCTATCCTCTACACGTCAGCGATTTGAAGAATTGTGGCCGTTTGGCTATTCTACAATTGGTGATGTT
AATTTTGAGTCTGCTGCTTTGTTGCAAGATATATATGAAGAAGATTAATGGGGTTACTGTCAATGAAAACCACGAAGTGGTTGATGCGGGTGCC
CATTCTCAATATTGTGATTTAGAGACTGGTGAGATAATTCAGCGTAAGCCAGAATTTAATAAGATGTCATTGAAGCCCGGTATCGGGCAATCGTG
GTTAGATAAGTACATGTCAGACGTTTATACGTCAGACCACGTTGTGGTGCGTGGCAAAAAGTGCCGTCCACCACGGTTTTATGATAATAAATTTA
AGTTGAAGTTTCCTGAAGAATTCGATATGATTCAGTTTGCCAGAGAGATGGAAGGTCGATCTAGGCATGAGGACAACACGCTTGAGCGACTTGC
TGTTAAGGAAAAAGTTGCGTTGGCTAAGTTGTCATTGTTAAAACGTACTATTTAAGGAGTTTTATGAAGATGGTTATTGTTTCTATTAAGGATACT
GCTGCAGATGCTTTTGGTCGTCCAGCTTATGTTGCATCTGAAGGTGTTGCAGTACGTCAGTTTCAGGATGAAGTCAATCGAGCTAGCGAAGATAA
TCAGTTGTATAAACATCCTGATGATTTTCATATGTTCTATTTGGGTCTTTTTGACGATGCCACTGGTGTTTTTGAACTACTGGAAAGCCCTAAGTTG
ATTGCTCGTGCAAAAGATGTAATGATTCGCGAAGGCGAGTAAGGTTTTTTTATACCGTATCACTCGAAAGAGTGGTACGGAACTACGGGAGAT
GTTTATGTTTCGCAATAAGTCAGTAAGTACGCATTCATTTGCTATGGTTCCTAAAGCGGACATTCCCCGCTCTAGTTTTAATACTCAATATGCTCAT
AAAACCACGTTTGATGCTGGTTTTTTAGTTCCTATTTATTGTGATGAAGTATTGCCTGGCGATACTCATCGTGTAAAGATGACTGCATTTGCACGT
TTGGCCACACCGTTATTTCCTGTGATGGACAACTTGCATCTTGATACTTTCTTTTTCTTTGTACCTAATCGTTTACTTTGGAACAATTGGCCAAAGTT
TATGGGTGAACAAACGAATCCTGGTGATTCTATTCTTTTGTAGTGCCTACTATTACTAGTCCTGCTGGTGGTTATGCTGTTTGTTCAATTTTTGATT
ATTTTGGTTTACCTACTGCTGGTCAGATTACTGGCGCTAATACAGTAACGCATAATGTTTTGCCGTTACGTGCTTATAATGAGATTTATAACGAAT
GGTTTAGAGATGAAAACTTACAGAATTCTGTAACGTTAAATCTTGGTGATTCAGGTGATGTTCCTGCTAACTATACACTTTTGAGACGTGGTAAG
CGTAAAGATTATTTTACTGGTGCATTGCCTTGGCCACAGAAGGGTGCTTCTGTTTCTTTACCGTTAGGAACACGTGCTAATATTTATTCTGACATA
CCAGCTGGCAATGGTACTGCTGGTTATAGTGTTTTTCAAACTGCTGTTGGTGCTTTAAGAGAATTAAATTCAGCTTCTAATAC

>000219F|arrow

GTTGTCAGTAGAAGTACCAGTAACATAAATTTCCTTATTGAGGACGGCTTGTTCGCCTAAGGTTGCAAAAGCAGGAAAGTAGAAGTCGTATCGA
GTAGAACGACTCCACATTTAGAAAGACCTTGTTGATAAGTAAGGTCAGCACGTACTGAGACGAGGCCAATAATTACGCCATGCTCAGTAAACGA
TTGAGTGAAGCCATGATTGTGAGCCAAGGCAGTACCCATAGCAGCAAGGTTGCCCATAGGGGTAGATCCGCCAGTAACGCCTGTAGCTGACGTT
TGAGCGATTGGGTTGATATTAATCGGGGTTGAACCACCGCCGATATATTCAGGAACGTTGTAAGCGAGCATCAGGACTAATAACGCCGAAATGG
CTACGAATAATCTCAGTGTAACGAGTACCGCCACGAGCGTCTCTTTCAAGCAGTTTTGAATTTGAAAAGATTGACGTAATTGATTAATCGTTGCA
GCAGTAGCTTGTGAGAGATCTGCATAAATAGGCATAAGCCCAGTCAGTTTTGTACATAAACAAAAGTACATCCCATTTCTTATCGTTTTGAAGAC
CATAGAAGATAGGAATAAATAACGTATTTGATTTGCCAGCAGTTGCCCAGCAATAGTCATAGTAGTACACATACTTTATAGGAGCTGTATACCTA
AGGTAATGTAACAGAATCGCCTTTTTGTGGCCAGGATGCAGAGTAAAATAATCTTACGTTTTACATGCGTTTTAATATGGTGTAATTAGCGACTG
TATCTGGTACCATCGCCAGTATCTACAGTTACGCTATTTTGTAAGTTTCATCTCGGAACCATTCGGTCAGAATTCAATTGTAAGCACCGTGGCCAA
AAAGCACAGTGGACTTACAGTTTGCTCGGCAGTTACCTGCCCGCACGTGGCAAGCCCAATATAGTCTTGCAGAGAGCCGATGGCATAGCCACCG
GCAGGGACACCTGTTGAGGTACTACGTACGAGATTGAATCCGAAGGATTCGCTTGTGTCCCATAAATTTTTGCCAATTTGACCAAATTAGGCGAT
TAGGAACAAAGAAGGAATGAGTCTAATACCATGTTATCCATAATTGGGAACAATGGTGTAGAAAGACGGGCAAATGCCGTCATATTTAATTTAA
ATGTATCGCCCGGTAGAACTTCGTCTACATATACAGGGACGAGATTACCAGCATCGAAGGTTGTTTATGTGTTGACTGACAGTCAAATGAAGAG
CGGGGAATATCCGCTTTTGGAATCATCGTAAATGATGAACATTTACCGATTTATTGCGATGCATGGATTTTTAGCTCCGTGGGTTGTTGGGGGAG
AAATGTACCATTTCTCTCCCCTTGGTTTTAAGCAGTAGTTTTTACTTGTGCCTAAGGTTAATAGTTTTGGTTGTTCATGTAAAGAGAATAAACCAGT
ATTGTCATCAAATTCGCCTAGCTCATATAGATCGAAGTCATCAGAATGGTTATAGAGTTGATTATCGGAGTCGTTGTTTACTTCGTCGAAATGATC
GAATTGCACCCAGTAGAAGGCACGAACATGGGCGCCATAAGCGTCAGCAGCACGGTCTTTTACGACAAGTTAGTATTTCATGAGGATGTCCTTT
TAAGTGAGGGTACGTTTAGTTTTTGAAGTTTGCTTTGGTGACTTGCTCTTTAACGAAGTCTTTCAAGTATTGTCTTCGAGTTAAGTTTAGCACGTTT
TCACGTTTTATAAGATTCATCATATTCATAGGGATAGTCAGATTTTATATTTTTATCAAGAATTTTGGGGGTCTGACTTTTTTCCACGAACTACACG
TAGTCGTGAGGATATACGTCGAACGAATTTTTTGTACCACTCTGCCCTATTCCCGGCTTTAAGGACATTTATTGTATTCTTTTTGCATCTGACAATT
TCCCAGTATCAAGGATGTGTATTGATAGTGTTTTTCACTTCTTTACCAGTTTGTTTTTCATAATGTATCTAGCCACGTACGCAGCTGATTCAAGTTA
ACGTCTCCAATGGAGGAATAACCAAATGGCCAGACTGTTTCAAGGTCTGCGGATCTATAAATGAGAGAACCAGAGGCAGTCCTTTTCCATAGTTT
CTTATCATGAAAGTCGAATCCGAAGATACAGGCATGGAAGTGAGGTCGGCCGAATTTTC

>000222F|arrow

ATGAGGATTCCTAAGTGGGTTACGTTTAAGTTGTTGAAGTTTTGCTTGTTGGATTTTTCTTTGACGGAGAGTCTTTCAAGGGTATTGTCTTCGCTA
TTTAGTTTACCGTTAATTCCCTTTTGTAAAGTATTTCGTCAAATTCGTAAGGATTATCAACTTTATACTTTTTATCATAACTTAGGTGGTTTACTTTTT
TCCCTTAATAATGACGTAATCATGGGGATACGTCAGATTTGTATGTTTGTACCATTCATAGCCTATTCCAGGCTTAATGACATTTGTTAAATTCGG
GTTTTCTCTTAGTTATTTCACCTGTTTCTGTGTCTATTTCTGTGTGTAATGGCTTTTGCGTTATGTCCTGTTACTTTTTTCATAATATATCTCGCAACG
TATGCTGCTGATTCAAAGTTAACATCTCCAATGGAGGAATAACCAAAATGGCCACAGAGTTTCAAGGTCTTCGGATCTATAAAGCATAGAACCAG
AGGGAGACCTTTTCCATAGTTTTTTATCATGAAAGTCGTATCCGAAGATACAGGCGTGGAAGTGAGGTCTGCCGAAATTTTCGCCATACTCTCCA
GCCATGTAATAGCGGATTCTAGTAGTTTCCGAATTTTTTCGAAGGTCGCTTAATGAACAATTGAAAGTCTTTGTAATGTAAGCTGCCATCGCTTGG
GAGATGTGTATTGTCATATGTGAGTGTTATGAAACAGTTTTTTTCATGTAATTGGGCTTCATGCATACAACGCATGGCCCATTGTCGTGATCTTTC

TAGCCTGCAGCCAATACATTGTCCACAGGGTAAAGATAGTGTTTTGACGGTATTAAACCATCGTCTTTCATTAAAGACAATGGAACCGTCAGCGC
ATTGATATGCGCTTATTGGATGATAGCAAGGCATGTGAGGTGCCTGGGGGTTTTATTAGAACCTCCAGCCTCCGCGCTGGGGGGCTGATCTCAT
ATTTGGGGATTTAGTCTTTCGACTATGGATGCGAAATGTTTATGCTGATTTCTTTTTATAACTCCGGTCTGGAATTACATGGTTTTACTCCTTTTTG
GTGTTTGTGTCACCTAGCACGTTACATCAGTAAGGTAACTGTGACCTTGCGGTCTCTCGACAGCTTTTTCCTGCGGTTAATTCAAACGACTTGCGG
CATTTCCGCAGGTTTAATTTCGACGAGGCCCAATTTTGCGCCCATCTAAAGGTTTTTGGGATTATTGATGGGAAACTCAATAACGTTTGTGTTGGA
TTCCTTATGAAACCTCGCGAAGATCGGCTTGCAGGGACCATAAATTCGTCCTCTGCAGCGATTAATGGTAAGGGCTGAATGGTAGTCACTGACAC
CAGTGAAATAGCCATAGCGGGGCGATACATGGACTTCAGGGTAAAAGTCCCGGTACTTAAATGTCTAAGAATGTTGTTACTTTCACATTCGTCTT
TGTAATGCTGCTGAGCCAGGGAACAGCAATCCTCACAATGCACCCCTGACTCATTTGACGCAGCATCTGTATCATAGTTGTATGGTGTACGTAAA
AACGGTAGTTTTTTCATTTGAATTTTCCAAAAGCGGATGTTGCTGAAGTAATGCCTTGTAAGGCATCTTTTAAATATGGACGAATTTGTCCATATT
GAGTTTTACTCAATTCTTCTTCAGGTTTGGCAATAGTAATATTTTGCCTTGTTTGCGCTTCTTGCGCAGTAGTATATCTTGTTTGGGCTTTAGTTAA
GCCCGCTTGTTCTAATATTTGATTAGTTTGAGCCTGGGTTAAGCCAGGTCTGTACGCTTCTGTTATTGCAGTAGCTCTTTTTAATGCTGCTGCAGCA
TTAGTGTCGTCAATCTGCGAATGTAATAATTCTTGTTGACTATGCAAATTTTGTAATTGTTGGTATTTACTAATACCTTCTTGAGCGCTGGATACAC
CAGCGCCTATTGCATTTTGCATTTGTGCAGATGCACCTGGTTGATTACCAGCTCCTCCTTGTTGATAAGCTAACATAGGATTTAAACCAGCTTTTTT
CATATCTTCTACAGCAGTTTGATATGAGTTTCTCTTTGCTGGTTTGAAAGTTCATTGCTGACGTGCAATGTCAGCATTAGCTTGGTTAGCAGATTG
TGGCCGAAATACGAGCCAATTCCTCCTATAAGTCCTCCACCTAAGATGACATCGTTGTTTTAGCGAC

>000227F|arrow

ATCATGAAAGTCGTATCCGAAGATACAGGCGTGGAAGTGAGGTCTGCCGAAATTTTCGCCATACTCTCCAGCCATGTAATAGCGGATTCTAGAG
TTTCCGAATTTTTTTCGAAGTCGCTTAATGAACAATTGAAAGTCTTTGTAATGTAAGCTGCCATCGCTTGGGAGATGTGTATTGTCATATGTGAGT
GTTATGAAACAGTTTTTCTCATGTAATTGGGCTTCATGCATACAACGCATGGCCCATTGTCGTGATCTTTCTAGCCTGCAGCCAATACATTGTCCG
CAGGGTAAAGATAGTGTTTTGACGGTATTAAACCATCGTCTTTCATTAAAGACAATGGAACCGTCAGCGCATTGATATGCGCTTATTGGATGATA
GCAAGGCATGTGAGGTGCCTGGGGGTTTTATTAGAACCTCCAGCCTCCGCGCTGGGGGGCTGATCTCATATTTGGGGATTTAGTCTTTCGACTAT
GATGCCGAAAGGTTTTAGCTGATTTCTTTTTATTAACTCCGTGTCTGCGCATATACATGGTTTTATCTCCTTTTTTGGGGTTGGTGTCACCTAGCAC
AGTTACATCTAGTAAGGTAACTGTGCTTGCGGTCTACTCGACCGCTTTTTCCTGCGGTAATTCAACGACTTGCGGCATTTCCGCAGGTTTAATTTC
GACGAGGCCCAATTTTTGCGCCTCATCTAAGTTTTGTGGATTATTGAGGAACTCAATAAGTTGTGTTGGATCATTTTGAAACCTCGCCCGAAGATC
GGCTGGCAGGGACATAAATTCGTCCTCTGCAGCGATTACTTGGTTAAGGGCTGAATGGTAGTCACTGATACCAGTGAAATCGCCATAGCGGGGC
GATAATGGACTTTCAGGTAAAAGTCCGGTAACGTTAAATTGTCTAAGAATGTTGTTAATGTCACATTCGTCTTTGTAATGCTGCTGAGCCAGGGA
AGCATCCTCACAATGCAACCCTGACTCATTTGACGCAGCATCTGTATCATAGTTGTATGGTGTACGTAAAAACGGCGGTTTTTTCATTTTTTAATCC
TATTGAAAGCGCCTGTTGCGCTTGTTATGCCATCTAAGGCATCTTTTAAATACGGTCTCATTTGACCGTATGGTGTTTTAGACATAGCCTCTTCTGG
CTGTCTAATTGTATATTCTTGACGAGTTTTTGCTTCTTGAGCGCTCGTCAATTTAGTGCTGGCTACATTTAAGCCAGCTTTAGTTAATATTTCATTAG
TTTGTGCCTGAGTTAAACCAGGCCTGTACGCTTCCGTTATTGCGGTTGCGCGTTTTAGTGCTGCAGCTGCAGTTGTATCGTCAATTTGTGCGCCAA
GCAATTCTTGTTGGCTATTAAGATTTTGTAATTGTTGGTATTTGCTAATACCTTCTTGGGCGCTTGATATACCAGCGCCTATCGCATTTTGCATTTG
TGCGGATGCGCCTGCTTGATTACCAGCGCCTCCTTGTGAATAAGCTAACATAGGATTTAAACCAGCTTTTTTCATATCATCAACTGCTGTTTGATAT
GAAGTTGTTCGTTGCTGGTTCTGAAAATCCATTTGCTGACGTGCAATATCAGCATTAGCTTGGTTTGCAGATTGTTGGCCTAAATAAGAGCCAAC
ACCTCCTATTAATCCTCCACCTAAAGACGACATAACCGTTTTAGGTATGTCGCCTATGGCGGATACAATGCCATCGAACATTCCCATATTAGAAGT
GGTCGATTAAGCCAGGTACGCTGTACATTGGCATTGGTCTTGCTTTCTTTACATCAAAGAAAGAATCAAAGATAAATTGCTGACCGTTAGCAGAG
CTACCGACAGCTACGACACGTGATACTGGTGGAGTATCTTTAATAAATGTGTCGTTAAGTGTTGGGACAGCCGTGAATTTTTGAGCTAAATGCCA
GCCGTCAATTGTTCCGCTAGCAGTTGATCTAAATAAGCTCGAAATTCGTGATGGATAGTATCTATATTCTGCCCAACGTTCTTGGTAACCGAATAC
ACCTGTATCGGTTGTATCTCCTGTTACATAAATTTCTTGGTTTAGTACAGATTGTTCTCCAAGCGTTGCAAACGCTGGAAAATAGAAG

>000231F|arrow

AATGATGAGATAAAGTCTTTGTGTTTCTGCCATGTTTAACTTGTACACTGGGTCAGCTACTGGAAAGCCTTTGGGATTTGAGGATTTGATGGTTTT
TGCCGCCTTTACGAATAGTTACGACCTTGTATTTGCGAGCCATTTTGGACATTTACTTTTCAGTTCAAGTTTACTACGACCTAATAGTCCGTTACGA
AACTAATGGTGTTGACCTGCTAGTAGACCACGAGTAATGATGCGATTGCAATTCCTGCCTTTTTTGATATATACTAATATATGAGAAAATAATTTG
GCATTTCATGATTGATGTTTTAGAGGTACCTAAATAATAGTCGTTCCACCCGACTGCCTAACCCCCTTGAGGATTCTGAAAGCCCTTTGGGCTTCC
ATAATTTACAAAAAGTTGACATACCCTTGCCAAATGGCAGATTGTATATTTTAGTCGGAGCCTTTATTAATGTCTCCGGAGTGTTATTGGTTTTAC
ATAGTGGGAGTTGGCTTTCATAATAGTGCTCTATCTCGGAGACTAAGCCGGTTTAGAGCCTATATAGAGAGTATACGCCTTCATATGCGCGATAG
GCGGTTTTTTTATATTCAAATACATAGGTATTGTGAATAAACAAAGCAATTTGATGAAGGTATGTGATGATTAATCAGATGTTTGGCTAAACATA
ATTCATACAATTTGTCTCTTGAATCCAGTTTTTGGTTGTCTTTGACTGCTTGTACACCGGATATGATTGATTGGATTTCTTCTGCCGAATAATTATGT
AAAGATGATTTACAATCCTTTGCTACATACAATTCGTTGTTTTGGAGCTTTATCACATTTCTTATGATAATATATTGCTCCACCTTTCAAGTCATGTT
TATGCATAAACCGTTGAATAGCTTGTCTTGATGGTTCTGATGTTTGATAGACCAGATGTGCGTGGACATGTTGTACGCCATCTTGTGATACTTCAG
TTCCAGCTAACGCCTTAACTGGATTGAAGAGACGGACAAATTCTTCGATAATTGCCCGTTGATGCACTAACGGTACCGTTATGCGTAAGTGAAAA

TTTAGTTCCATTATATATTATTATATGAGAAATAATTTTATTTTTTTAGTGCGTTTAAAACGCTCTAATATTGTCCGCCCTGACGGACGGACGGACA
CTTCTTCCCTAAGGTGAAGGTCGTGTCCTAATGGTTTTGGTAATGGATATATGCTTAAGCAAATACTGGGTTTACAACAGCGCCAGTGCTTCATTC
ACGAATGTAGTATTGCCGGCATTGACTAACCACCAGCGTAAAAGTTAGCTTTTGGATGTCTTGTGGTTGTTTGAATGTATACTCCATGTAGCGAT
AGCACTTACTTTGGCAGCCGTTGTTGTGGTAACCAAGGCGTATCAGTTGCATTTGCTATTTGACCAGTGATTTGTAATACGAATCCTTTGGTAATA
CCACGTAAAGCTTTCAAGAAAGCATTATTGATTAGTTGACCGTTTATTGTTTGAGTCTTTGGATCATGACAAACCATGATACAGCCTTGTTTGGTG
TTTTAAACAAAGGGTCTTGTCTCTAAACTATACGAAAGTATGTTACGAACATTGGATTATCATATAATGATGAACCCACTTTGACTCGGTTATTTC
ATTATCTGTTTTATTTGTCAAGAAACCGTTTTCTACGACACTTTCCAAACTAGTTAAAGCATCAGGTAAATCATATCGGCTAACGAATTCATACATT
CTTATGGTTACGTTAGCTGATGATTGATTGATTGATTGTAGTCGTAATGCAGTTTTGCATCCGATACCGAGAGCTTAAAATTATCATCGCTTCGATGCCA
ATTTTTGCAGCCATTGCGTAGATAATCGTTGGGGAAGCATTCGAATGATTCGTATTCACATTTACCTTCATCTGCTAAAAATGATTGATGGTTACA
TTGGTATATGTTTTGGTGGATTAATCCTTCTCGAACCTTGTTCATGAATGATGATGATAAAGTCTTGTGGTTTCTGCCATGTTGAACTTGTACACTG
GGTCAGCTACTGGAAAGCCTTT

>000229F|arrow

ACATAATTCTAAGTTTGCTTCCCTTTTCTGTTCTTCGTAAACGTGCTTGGCAGCATGATTATTTTACTGCTGCTTTACCTTTTGCTCAGAAAGGTCCA
GCTGTCGATATTCCTATCGGTGGAGATGTTGGTCTTAAACCTGCTAATGCTGGTCAACGGCAATTATTTGTTGATCCTGCAGCTCATTTACCTTTA
GCTGGTGGCGTTAATAATAATCCTACTACTGGAGCTCTTACTACTTCCACGAATGTTCCTGGTATTATTGACCCTAATGGTACTTTAGAAGTCCAG
GGTACTGCTACAATCAATGAACTCCGCCAAGCTTTTCGTCTTCAAGAATATTACGAAAAGGCCGCCCGTGGGGGTACTCGTTTAATCGAGTGGGT
AAATCTATGTTTGGCGTACAGTCTTCTGATGCCCGTCTCCAGAGACCTGAATATCTTGGTGGATCTGTTCAACCTATGGTGATCAGCGAAGTTCTT
CAAACTTCTGAAACTGCTACCTCTGCTCAGGGTAACATGGCAGGTCATGGCGTTTCTGTTGGTGATGGTCGTAACTTTACTTACCGCTCTGAGGA
GCATGGCGTGATTATTACTATTATGTCTGTTATGCCTAAGACTGCCTATCAGCAAGGTATCCCTCGTATGTTTTCAAAATTTGACAAATTCCAATAT
TTTACCCCGCCATTTGCTCACCTTGGTGAACAAGAGATCCTTAATCGTGAACTTTATTTCGATTATTCTGAAGGCGCTGTTAATGATCAAGTTTTCG
GGTACATTCCTCGATATATGGAATATCGTTATAACGATTGTCGTGTTGCTGGTGACTTTAGGAATACTCTTAGCTATTGGCACATGGGACGTATTT
TCTCTTCTCGCCCTAATCTCAACGCTGCCTTTATTCAGGCTGATCCGACTCATCGAACGTTTGCCGTTATAGATCCCGATGAGGATAAGATTTACG
CTCATTGTTATCATTCCATTAAAGCCGTCCGCAAAATTCCTAAATATGGAACTCCAACTTTCTAAATCATGGCATGCGAATTTCCATTCGGAGTTAA
AAACAAAAAACCCATGTCTCAATCAGAGACGTGGGTTAATGTCCCTTGCGGAAAATGCCCAAACTGCTTACAAAGCCGTTCTAATGCTTGGCTAT
TACGATTACTTACTGAGGAAAAAAAACTGTTATTCCGCGTATTTCGTTACGCTTACGTACGATGAAAATCATATCCCACTCACGCAGGACGGAAA
AAGGACTTTGCTCAAATCAGATTTTCAGAAATTTATTAAACGCTTACGTAAAACAAACACGACCGATCGTAAAATCACTTATTATTGCGCAGGAG
AGTATGGTGAAAAAATTCAAACGACCGCATTACCATGCAATCATATTTAACTCCAATGAATCCGCAATACGATCCTCGTGGGCATTGGCCTCACAA
ACAATTGGAGATGTGGACGTTGCTCCCTCTTCTGCTGCTACTATGGCCTATACCTTAAAGTACATTCACAAAGGTCAAACTATTCCTAAAGGTGAT
TCTCGTCAACCCGAATTCAGCCTCATGTCTAAAGGATTAGGTAAAAATTACCTTACCCCTGAAATGGTGGCCTATCACCAAAGTGATAAAACTCG
CTTTTACGGTACTCTTCCAGGTGGAATAAAAATTCCTTTACCTCGCTATTTCAAAGAGAGGATTTATTCAGAATCTGACCGCCATGCTTATGCTAA
ACTCATTGCTAAAACTTCTGAGTTTGATGAAGCTAAACGGTTTTTAGATTCTGGTCTTTCTCCTGATGAATATCGACGTGAAGTATTTGAACGCAA
AAAAGCTGCAATCATTAACTTTAAAAACAAATCAAAAAGATGAAAACTCAAACTCCTTACATTAATCCAAAACTTCGCCATGTGATGAATTATTCC
CTTGGCGATTCTATCGCTGATATGGAGATCTCTGATCCTATCAGCCTTACTATTCCTGATATGACTATGAGCTTACGCGAATTAGTCGACCGCCAC
ACTCGTGGTCTTCCTACTCCTACTCTTGGAACTCCTACATTTTCGGATGAGGAGTTTCCTGATCTCGAACGTCTCGATCTTGCAGAAATCGAAGAA
CTTCGTTATCAAAATGCTCAACGTATTGAATTTCTTAAAATGCAACATGAAGATCTACTCGCTAAATCTCTCGAATTAAAACAAGCTGCAGCTCCA
GCTGAAAATCCTGACGCTTAGCGTCTAAAAATTGGGGGGTTATAAAGGGGGGCTATGCCCCCCTTTTCTGTTATAAAATAACATAAAAACAAAAA
AAATAATGATCATGATCATTATAAAATACAAAGCTCGCGAGCGACATGATCGAGCCTGCTACCTTGTGAGCAAGCCTGCGCGCGAACTTGGGAC
GCTGCTCGATCCGGAAGCGAGCCGGAGCGTACTGTCGGAAATCCATTTCCGTAAATACAAAAGTCTCCCGAAGGAGACAGGCCGTACTGAGTG
TCGGCCTATGCACTATACTATCTTGGTATATTAGTGCTAATTGACACTTGTGTCAATAAAAAAAATATTTAACTTCGTGAAACGAATTAAATAAAC
CGTCGCGTAGCGACATAAACTTATTGCCTATGCCTATAGATCCCTCAATTGTAGTCGCTGGAGCTAACGCCCTGGGACAAGTTGCGAATGTCGCA
GCTACTTCTTCAATGAATCGGAAAACGCGAGAGTATAATGATCGTACCTGGTTCAGACAACAAGCCGCAAATTTCCATAATTGGGAATTACAAAA
TGCTTATAACGATCCAAAAGCTCAAATGGAACGTCTTAAAGCTGCTGGTTTAAATCCTAACTTAGTTTATGGATCTGGTCAAGCTCAACAAGCAG
CTTCTCCTATTCAGGCTGCATCTACTCCTACATGGTCACCCCGCGCCCCTCAAATCGAAATCGCTCCCGCGATCGCTTCCCATTTCGATACTCGTAT
CAAGCAAGCTCAATACGACAATCTCAAGGCTCAAAATACTGTTCTTACAAACGAAGCCTTGTTAAAGATCGCTCAGATCTCAAATTTAAATCAAG
GAACAACGGGAAAATCCTTGCAAAACTCTTTGGCCTCCGCTACTTACGGTAATACCTTGGAGGCTTCTAATGAATCTTTACGCAGAATGCAAATG
CAAAATCGTATTCTTGGTTATGAAGATTCTAATGCTGAATCGAACGCTACTGCAAAAAACAATCTTTTGCGCGGCCAGGCTGAAAAGATCTGGCA
AGACATTAACGAAAGCAAGTCGCGTATTCAGTCTATGTCACAAGATCAAAAACTTAAACAGTTTGAAATCAACCTTCGTAAAAACGGATTAAATC
CAAATGATCCCATGATCATGCGTATGATCGGTCAAATTATCTCTAATAAGTTTAACCTTAACAACCTAATAAAATGAAAAGATCCTATCGCGGCAA
AACCCGCAGATCCTCAGGCCGTGGCCGTAAACAATTGAAGTATTACACTGTATCAAGGGGAGGTACGCGTTTATGAAAAAATATCTTCAATACCGT
GAAAATGACAAATCTCGATTCTAATGTATTCGATTTGTCGTATGATATGAAACTTTCCTGCAATATGGGCGAACTTATCCCTATCCATTGCCAGGA
AGTTTTGCCTGGTGATGTTATCACCATGTCTACCTCTTCCCTCCTGCGCTTTGCGCCATTAGTTGCACCCGTAATGCATAAAGTTGATGTTTATACT

CATTCATTCTTCGTTCCTAACCGTCTTGTTTGGCGTAATTGGGGGACTTTTCTTACTGGCGGTGATAACGGAACAACTGAGCCAGCTGCCCCGTTC
TTCCAGAACTTTGGTATTACTACGGGAAGCCTTGCCGATTATCTCGGGCTGCCTATCGATCCTGTGGGCATCGACAAAGCTAGTGCAATTCCTTTT
GCTGCTTATCAGTTAATTTGGAACGAATACTACCGCGATCAAAATCTTCAGCCTGGTGTAAATACTACCCTTGACGATGGAAACAATAATTCTAA
G

>000208F|arrow

CTAGAGGATGGAACGACGATGTATGGTGTTCTGGGGAAAAACAGAACTGGGTAAAAGTCGACGTGCATGGGATGAAGCTGGCCTGGATGCTT
ATTGTAGGATCCCAGAACAAAATTTTGGGACGGTTACATGATGAAAAAAATGTTGTCATCGATGAATTTCGAGGAGGTATCGACATTTCCCATTT
ATTGCGATGGCTTGACCGATATCCGGTCAGAGTGGAGAAAGGAAGTTCAAAACCATTAAAAGCCCACTACTATATGGATTACTTCAAACATTTCA
CCTGTAATGTGGTATCCAATGCTTGATGAAGAAACATTAGCTGCTTTAACACGAAGATTAATTATAACAGAGTTTGACTAATAAAAAAATATAGT
ATGTCAGAAGAGCCGTACTATATTATCGTCGCCGTCGTATCACACCTCGTAGGGTTGTTCGTCGCGCACGGGTACCATATCGTCGTAGGGTCCAA
GTGCGTGCACCGATCAGAAGACGACGAACTTTAGGTCGTAGATAGTTTACTTAATAAAAATAATGAAACGAAAATCTTCTGATTTCCACTAATTA
TAAGTAAGATGTATGATACTCGTGGATCTAAAAGATTCAATCTAACCAGCAACTCGTAAAAGAACATGGAAAGAGTTTGGAAAACAAGCTGCTG
GTAGTGCTGCTGGTGCAACTTAGGATTTATTCATGCCAATACTGGTGGTGCCATGGTTGGTGGAAGAAAGCATGGGATTATTTAGCCCAGATTT
GGAAGTTAAAGAAGATGAAATATGATGGCTCCATTGTTAGAAGCTGCAGGAATCTTTCCCAAAAAACAATGGGAGGTGCTGTTACTTATGGAAA
CAGAAAGTTTTCTAAACCCAGCAAGAAATTTATTCCCCTTTGACTAAATATCAAGCTCAAGGAGTTGTATTTAAAAAGGAAGTTTACGGTAAGTTA
CTTCCCAGATTTGTATATAGATGTCATAGTACTTATGATCAAGAGCAGTTGGCTAGAACAATTGCTTTGTGATTCTTAGAAAATTATTTAAGAAAG
CTGGTTATGATCCTACATCACAAGGAAGCTATGCCAATGAAAAGATTTTATGCTAATAGTGCAGAAGTTCGATTTCAGATAACTACTGAAGATAT
CGATACTGGTACACTTATTATGCTTATGATACGAATGCAACTGACAGTTTGGACACAATTGTTGGTGCAAATTTTGTTGGACAAGGGTTTAGTTTT
TTGATTTTATTAATTTAATAATGGCACAAACGATACATCAACTTATACTATTCTTCTCTGTTCGTTTGTTTACAAGATAAATGGTCCTGTTGTGACA
GTTGCAGATTGGCGTTTAAAGACAATTTGAACATGACGAATGAAATTATGAAAATTTATATGTCATAGTCGTTTAGTTGTTCAAAAGCAACTAAA
GGTGCTGCAAGTGGATCTGCGATGATACGCTGGATGCACAACCTTTAACAGGATTCTTTTATCAATTTGCTGGTGGTGCTCCTGCATCTAAACAA
CGTGATAACACTGGTTTGAGTCGTTTAAGATCATTTGGTCTTCAATTGCACAGAGCACAAGATTTAAGTCCACCTGAAGACTTTAAAGAGACCAC
CATTCCTGCTACATTTAACTAATTGTTATAAAGCTTCAAGCATTTGGTTTGGAGCCTGGAAACTATGAAAAGAGCAGATTACACTTCTACTTGGAG
AGTGGTTATTTTAACATATATTGTTTGGGTCATTAACTCAAAACGAGTTGCAACTTTACAATTTAACACTCCTGGTAATCTAAACTTATTGCTTTAG
AAGAAGCATTAAATAGTGGTTCTACAAACCTTATTACTACCTCATATCAATCTGAAAAAAGATGGGTGTTATGCTTATTACTGGTCCTAAACCAAT
AATGATAGCTAAACATGAAGAAAATCAGTATAACTTAGACTTTTAATAATAAACAACAATCAGATTGTATAGAAAAGCGGGTACCAGCTTGCCTG
TGTGCCCCAAGATTCAGCTACCCCCCAGAGTGGCTCCGAAGCTTGCGTAGGTCGCCTCAATCGCGGGGGGGTATCTCAATGTTTCCTACACGTGT
TTACATGTTAGCCATAAAGTGAGAATCGAACCGCTCAGTATTACTTACTAAGAGCGGTTCTCAATTCTCACCCCCCAATATCAAGCCACATGTTTA
CAAAAACTGTGACAAGCGAACTACACGTGAATTTCGGTGCCTAATGAAAGTTTCATATATAAACATATAAATACTAATAAAGATGCATGACACGT
AGACAAGGAATCTTTGGATATTAACAATCCCTCAACATGAATTCACTCCATACACCCCCCGAATGTCCAATACATTGTTGGGCAACTTGAAAAAG
GAAGCAACACTGGATTCTTACATTGGCAAGTTATGGTCGCTTTCAAATCCAAACAGTCTTTATCCGGAGTTAGAAACACCTTTGGGTCCTGTCATG
CTGAATTATCAAGATCATCAGCAGCCACCTCCTATTGCCAAAAGAAGAATCTTCGATTGTGGGAACCCAATTCGAGATGGGAGCAAAACCATTTG
CAAGAAACGAGAAAGTGGAATGGGAGTCTGTTTGGTCCGCCGCCAAGTCCGGAGATCTTGAACGAATCCCGGCGAACGTTCGAGTGGTTAGTT
ACAGGACGATTCGAGCAATTAGCTCAGATTATTCAAACCTAGAGGAATGGAACGACGATGTATGGTGTTCTGGGGAAAAACAGGAACTGGTAA
AAGTCGACGTGCATGGGATGAAGCTGGCCTGGATGCTTATTGTAAGGATCCCAGAACAAAATTTTGGGACGGTTACAATGATGAAGAAAATGTT
GTCATCGATGAATTTCGAGGA

>000189F|arrow

TAAATTGGATGTGTAATTAAACCTGGTTTTAATACATTTACAGCCCCAGTATTCTTAAAAGTTATAAATAATACCACGTTTACACGGGTCAGGCTT
AGGTTAGGGGTTAGGTAAGGGTAAGGAAGTCTTATCGCTTGTCGATCTTGTCCCCGTACGGGACCCTCGTCGAGGGCATACCTGCCCTTGACTT
CTAACTCTCCATGTCCAGTTACTGGACCCATTTTTTTTTTTTAATTTCACCAACTACCCCCTAACATTTTTTAAAAACGACTTTGAAGCCGAGACTCC
GCTAGTATTACTTTACAGAAGCGGAGTCTCAAAGTCTCACCCCCCCTTGAGCCAACCTTATCAGGGATAACCTTACACTCAGGAGAACCTTAAAC
GGTTTAAGGTTAAAAAGGAGTGACGCCCACCAAAAGCCGAATTTGTATAAATAGCCGCTGTTTTGCACATAAATTCAGTAACAAATGTCTGAATC
TCAATCATGTCTGAACAATGTCGCTACTTGGATTCTTACTATCCCCAAGAATGGCAACTTGAAACCCTTTGCTCCTGTCCTGGTCGCCTATGTCAG
GGTCAACAAGAGATTGGCGAAGGTGGAATATGGAACATTGGCAATTACTCGCAGTATTTAAAAGAAAGGCCCGTCGAGGCAGCATTAAGTCGG
CGTTCAATTCCAACCAGGCTCATTGTGAACCGAGCGCTTCAGTTGGCAGCCGGACCAGTATGTGTGGAAAGAAGAAACAGAGTCCCAGGTACAC
AATTGAATTGGGAGGAAAAGCCTCACAAGAGAACAGTGCAGTCGACTGGAAGATTGGTTAAAAGAATAAGGCAATAGCTGTAAATATTGAATA
GCATTGATGTCACATGGTATATTTCAACACTATAGAACCCTTAAGACACAATTTAAAATGGACCATATGGTTCCACCCGATGATCTTAATAAGATA
TGTGGTATATGAGATACAACGGTCCCCCTGGTGTCGGTAAATCCATTATGCTCGCTCTAATTATCCTAATTCTTATTTCAAAATGTGGCAACCAAT
GGTGGTGGTGCTATAAAAATCAAAAGTCGGTTATAATCGACGATCTTGACCTCAGCCACAAGGTCCTTGGTCACCATCTCAAAATTTGGTCTGAT
AATACTCTTTCATAGCCGAAACCAAAGGATATGCAGTGACAATACGCCCAGAACGGATAATTGTCACTTCAAACTATAAAATTGATCAAATATTT

GAAGATCCAGCCTTAATCGATGCTATTGAAAGACGTTTTTATAAGATCTATTTACCACTTCGTATGTTTAATAATAAAAAAAATGGATTCAAGACG
TTATGGAACTAGAGATTATAATAGAATTTATCGCCTTTTTGGCAACATTGGTGCAGTTGGTAGTGCAATTATGGCTAATCGCTATCAAGTCGCAG
AAAATCGATTGAAAAAACAAATGGGAAGTAAAGGATGGTCTATTTCTTATCCAAATAAATATTTAAAAACAATGTATACTCCTCGTAAACGTAAA
ACATCTACTCCTCGCACTCCTAGATCTTTACCTAGATTAAGAAGAAGGAGTGTTGGTGGTGCTAAAGCTAGAGCAAGACTAAGTTCTAACAGACG
TATGGCAGCTAACTCATATAAGATTCGTAAAGTTAGAGGTAAAGTGTCAGCCACAAACAAGTATCTAAGCAAACAATTGGTTAAAATGAAACCA
AAACAGTTTTCTAGTAACACTGGTGGTAATTATGCTGGCGGTGTTGGGAAAACATATAAACTAAAAAAGAGAGACAATGAATTTTCTCAATTGCT
AAAAGGGTCTGTAGTCATAAATGAATATAGTGGCGTTACAGGGACAGCTCAACAAAGTGTTTATCTTGGTCATGCTAATTACGGTTTCGGATTAG
TATTAAAAGTATTTTCACGTGCTCTTACTAAATTGGTATTGAATCGTGTCGGTGTACAATTTGAACAATTCTCGGAGAATGTGATTATCGGAATGC
CTGGCGATATTATTAGTATACGATTTAAAACGTTTTATTTTCCAGCTGCCCCGTCAACTTTTAATCATGTAATTGCTGCTGGTCCATATGATAATAT
TGCTACATCAATAGCAGCTCATTTATTGTTACAGTCGCCACAATATCAAATGCTTGATGTTCAATATATTCCACAAGCAGGGTCAAATTTTAAATAT
AATAAGGTGCACATGAAGAATTCTAAAATATCTATTACAAGCAAGTCTGATTTTGTTTTACAGAATCGTACTACTTCTGCCCCTGATAATTTTGACC
AAGAAAGAATTGATAATCAACCAGTTAGGGTAAAGACATATCTTGGATATGGGAATGGTACTCAAATTGCTAATACACAACAACCTGGTGTTTTG
GAATATATCGCAGGTCAATCAAATGGTGTCATTGATGTCACACCAACTGCTGTTGGTAATGATCAACAAGAACCTCCTAATGCAAGATTTTTTGCC
CAATGTTATGCTCAGGGATCTTTTATTTTAGATCCTGGTCAATCGAAGACATCATCATTATATGATAGTCAAAATTTTTCACAAAGTTCATTTTTTC
AGAAGTTGTTAATGGGAAACATTGGTGATTGGACTAGTACAATGATGAAAATGGGAAAGCATAAGATATTTGGTATTGAAAAGCTTATTGATTG
TGTTGAAGCACAACCAGATGTGTTTATTGGATTTGAACATAATCTTAAAAT

>000115F|arrow

GATACTTCTTTTTCTTTGTACCTAATCGTTTACTTTGGAACAATTGGCCAAAGTTTATGGGTGAACAAAACGAATCCTGGTGATTCATTTCTTTTGT
AGTGCTACTATTACTAGTCCTGCTGGTGGTTATGCTGTTTGTTCAATTTTTGATATTTTGGTTACTACTGATGGTCAGATTACTGGCGCTAATACAG
TAACGCATAATGTTTTGCCGTTACGTGCTTATAATGAGATTTATAACGAATGGTTTAGAGATGAAAACTTACAGAATTCTGTAACGTTAAATCTTG
GTGATTCAGGTGATGTTCCTGCTAACTATACACTTTTGAGACGTGGTAAGCGTAAAGATTTTACTGGTGCATTGCCTTGGCCACAGAAGGGTGCT
TCTGTTTCTTTACCGTTAGGAACACGTGCTAATATTTATTCTGACATACAGCTGGCAATGGTACTGCTGGTTATAGTGTTTTTCAAACTGCTGTTGG
TGCTTTAAGAGAATTAAATTCAGCTTCTAATACTTTGTCTAATAGTACAAATGCTGGTGTTGCTACTAATCAGTTATACGCTGATTTGTCTACTGCT
ACTGCTGCGACTATTAACCAACTTCGTCAATCTTTCCAGATTCAGAAGTTATTGGAGCGCGATGCACGTGGTGGTACTCGTTATACTGAGTTACTA
CGTGCTCACTTTGGAGTAACTCCACAGGATTATCGTTTACAACGTCCTGAATATATTGGTGGAGGTTCGACCCTTGTTAATGTTAATCCGATTGCT
CAGACTTCTGCAACGTCGGTTACTGGTTCTGCTACTCCGCAAGGTAACCTTGCTGCAATGGGTACTGCATTGGCTCAGGGACACGGCTTTACGTA
TGCTGCTCAAGAACATGGATACATTATCGGATTAGTTTCTGTACGTGCTGACCTCACATATCACAGGGGTCTTCCTAAGGTGGTCTAGGTCTACAC
GATATGACTTTTATTTCCCAGTATTTGCCACTTTGGGTGAGCAAGCTATTTTGAACAAAGAAATTTATGTTCAAGGTACTGCAGCCGACAATGATG
TATTTGGTTATCAAGAACGTTGGGGGAGTATCGTTACAAACCTTCTCAAATTACTGGTTCTTTAGGTCTACTTCTGCTGGCACTATTGATGCTTGG
CATTATGGACAGCGATTTACTTCTCTCCTACGTTGAATTCAACGTTTATTCAAGAGACCCCTCCAGTTGCTCGTACTACGGCGGTCGGAGCTGCAG
CAAATGGTCAGCAATTTTAATGGATGCTTCTTTGATTGTCAGATGGCCAGACCTATGCCTATGTACAGCGTACCTGGTCTAATTGATCATTTCTAA
TGTTTTATATAACCTCGACTACTCCGTAAGGAGTAGTGAGGAAACAACCGAAGGGCGTTAGTTTATGTTTGGTGGAATACTTGATGCGGTTACTA
ATGTTGGTTCTAAGCTGTCTTCAGCTTCTAGTTTTTACTCCTGGTGTCGGTACTGCTTTGGGCGCTGTTGGTTCTTATTTAGGTTCTACTTCTGCTAA
TAAAGCTAATCAGGAGATGGCTCAGAGGCAAATGGATTTTCAAGCCGATATGAGTGGAACAAGTTACCAGCGTGCTGTTAAAGATTTAGAAGCT
GCTGGTTTATCCTATGTTAGCCTATCAACGTGGTGGTGCTTCTACCCCATCTGGTTCAACTGCTACTATGGAAAATGTTTTAGGTAATGCAACTAA
TTCAGCTATTAATACTGCTTCTATGATGCAACAGATTCGTAATGCATCAGAAACAGAAAAGCAGATTATCGCCCAGACTGAAGCTACTGAAGCTG
GTACCGCTAATACTAGGGCTGATACTGTTAATAAGTTGCTTACTGTCCTAATATTACAGCCGAAAATAAAACGTATTTTGGCTGATATTGCTTTAA
AGAATACGACTGCGGATTTAACATCGCTCAGTCTAAATACTAAGAGGCTATTGGCTCCATCCCAGCTATTTGGTCTAGGGGTATCGATGCTTCGA
AAGAAATTTTGATAAACTCAAAAATAATCCTAATCAACTAACCCCTTGGGGAATTGGAGTCAAATAATGAGTAAAGCGAATTTGCCATTTGTACG
TAATCCGTAAACTATGATAAAGATGAAGCATCGGTAAACGATGCGTTGCTGTGTCAAGACCCAAGTCTTGCTCAACAGCATATGAAAGATGAAT
GTGACATTAATGTCATCATTGAACGTTTCGGGGTTACAGGGGAACTTCCAACGGCCCCTGTATCGCCTCAATACGGCGATTTTAGTGGTGTTACT
GATTACCATTCTGCGTTGAATCAAATTAACGCAACTATGGACGATTTCATGGCTCTGCCAGCGAAATTAAGAGTCGATTTGACCATGATCCTGTCA
AATTATTGGAGTTCCTTGAGAACGACCAGAATCGTGATGAAGCGATTCAATTGGGTCTTATTGATGGACAACCTGTGGTTGAACCATCGTTTCTA
CAGAAACACTAAGGCGAAGGATGAAATCTGAGGCCAGCACAGTTACTCTACTTGATGTAACTGTGCTAGGTGACACCAAACCACTATTTTAACTA
CGGAGTTCATCATGTTACGAAGAAAGCCAGTAAACAAATAAATCTGCAAAGTCATTTCGCAGAATGCTAGTAAGACGAAGTCAATTAATATGAG
ACACGCTCCCCAGCGTGGTGGCTATCGTTTGTAATTATGGCCTGTTATAAGCCTTAAGGCTTATCAATGCAGTGACAGGTCTATAATTTGGCGGG
AAATACCGGGTGCGGATGTAGTCCGTACCCTATCATTGCCTTGTGGTCAGTGTGTTGGTTGTCGCCTTGAACGCTCACGTCAGTGGGCGATTCGT
TGTATGCATGAGGCACAAATGCATACTAGTAATTGTTTTATTACTTTGACATATGCTCCAGAGCATTGTCCTAAGGATATGTCATTGGATTACAAT
GATTATCAGCTTTTATGAAGCGGTTACGTAAGCGTTTTACTGGGAAAACGATACGTTTTTATATGGCAGGTGAATATGGTGAATCTTTTGATCGT
CCTCATTTCCATGCTTGTCTGTTTGGTCTTGATTTTCCGGATAAGAAAATATTTAAAAGAACGCAGACTGGCTCTATCCTCTACACGTCAGAGATTT
TGGAAGAATTGTGGCCGTTGGCTATTCTACAATTGGTGATGTTACTTTTGAGTCTGCTGCTTATGTTGCAAGATATATATGAAGAAGATTAATGG

GGTTACTGTCAATGAAAACCACGAAGTGGTTGATGCGGGTGCCCATTATCAATATTGTGATTTAGAGACTGGTGAGATAATTCAGCGTAAGCCA
GAATTTAAAAGATGTCATTGAAGCCCGGTATCGGCAATCGTGGTTAGATAAGTACATGTCAGACGTTTATACGTCAGACCACGTTGTGGTGCG
TGGCAAAAAGTGCCGTCCACCACGGTTTTATGATAATAAATTTAAGTTGAAGTTTCCTGAAGAATTCGATATGATTCAGTTGCCAGAGAGATGCG
AAGGTCTCGATCTAGGCATGAGGACAACACGCTGAGCGACTTGCTGCTGTTAAGGAAAAGTTGCGTTGGCGCTAAGTTGTCATTGTTAAACGTA
CTATTTAAGGAGTTTTTATGAAGATGGTTATTGTTTCTATTAAGGATACTGCTGCAGATGCTTTTGGTCGTCCAGCTTATGTTTGCATCTGAAGGT
GTTGCAGTACGTCCGTTCAGGATGAAGTCACATCGAGCTAGCGAAGATAATCAGTTGTATAAACATCCTGATGATTTTCCTATGTTCTATTTGGGT
CTTTTTGACGATGCCACTGGTGTTTTTGAACTACTGGAAAGCCCTAAGTGATTGCTCGTGCAAAAGATGTAATGATTCGCGAAGGCGGAGTAAG
GTTTTTTTTTCTACCGTATCACTCGAAAGAGTGGTACGGAACTACGGGAGATGTTTATGTTTCGCAATAAGTCAGTAAGTACGCATTCATTTGCTA
TGGTTCCTAAAGCGGACATT

>000109F|arrow

TGGACTTTTGCTGATTTCGTCTACTTCTGCAGCAACGATCAATCAGCTTCGTCAGCATTCCAGATTCAGAAGTTGCTTGAGCGCGATGCGCGAGG
TGGTACACGTTATACAGAATTGTTACGTGCGCATTTTGGTGTAACTCCACAGACTATCGTTTACAACGTCCTGAATATATTGGTGGAGGTTCTACT
CTGTGTTAATGTTAATCCGATTGCTCAGACTTCTGCAACGTCAGTTACTGGTTCTGCTACTCCGCAAGTAACCTTGCTGCAATGGGTACTGCATTG
GCTCAGGGACACGGTTTTACGTATGCTGCTCAAGAACATGGATACATTATCGGATTAGTTTCAGTTCGTGCGGATTTGACATATCAACGGTTTAC
CGAGATGTGGTCAAGATCTACACGTTATGATTTTTTATTTCCCAGTTTTTGCGACACTTGGTGAGCAAGCTATTCTTAAAAAGAAATTTATGTAAC
TGGTACATCTACTGATAATGATGTGTTTGGTTATCAAGAGCGTTGGGCTGAGTATCGCTATAAACACACAAATTACTGGTTTAATGAGATCTACA
GCTGCTGGTACTATTGATGCCTGGCATTATGGTCAACGTTTTACTAGTTTGCCTACTCTTAATTCTTCGTTTATTCAAGAGACCCCTCCAGTCGCTC
GTACTACGGCTGTTGGTACCGCAGCAAATGGTCAGCAATTTTTAATGGATGCTTTTCTTTGACTGCAATATGGCTCGACCAATGCCGATGTATAG
CGTACCGGGTTTAATTGATCATTTTTAATGTTTTTATAACCTCGACTACTCCGTAAGGAGTAGTGAGGAAACAACCGAAGGGCGTTAGTTTGTTTG
GTGATATTTTTGGAGCCTTGTTTCTGCTTTTCTAATCATCGTGAAGCAGAGATGAATCGTGATTGGCAAGAGTCGCAGCGAAAGACTGCTTATCA
AGATACTGTTAAGGATTTAACCGCAGCTGGGTTAAGTCCTATGTTGGCTTATTCTAAAGGGCCAACGTCTGGCGGATCAGTGCGACTGCTGCACC
TATGGCCCGCTTAAGTTAGGTGAAACTGAGCAACGTCAGTCCTCTGCTGAACTTGCTCGTGAGCAGCTAATGTGCTAAGTCTACTACTATTGTTA
ATGAGGCAAGTGCCGAGAAATTAAGGCTGAAACCGCTAATATTAATCAAGATACAATTAATAAGGGTCAGCCATTTCAAGGTTTGAACGAGGCT
CAATTAAAGAGCTTTTAGCTCGTGGCCTCAACATGGTGCTTCTGCTGCTCAATTAAATACATTGGTTAAGAGATTGAGCAGAATGTTAATCTTCGC
AAGCCCCGGAGGAATTTAAAGAGAACATCCTACGGCTGCGATGTATATGAATCCTATTAGGGATGCATTGTCTACTATTTTTGGTGGTCTTGGCT
TACTTCGTGGAAGTTCTGCCATGCCTTTTGTACACAACAAGCTCCGAAAGGTAAAAATGAGTAAAGCGAATTTGCCATTTGTACGTAATCCGTAC
AATTATGATAAAGATGAAGCATCGTTAGCCGATGCGTTGCTGTGTCAAGACCCAAGTCTTGCTCAACAGCATATGAAAGATGTGACATAATGTCA
TCATTGAACGTTTTGGGGTTACAGGGGAACTTCCAACGGCCCCTGTATCGCCACAATATGGCGATTTTAGTGGTGTTACTGACTACCACTCTGCG
TTGAATCAAATTAACGCAACTATGGACGATTTCATGGCTTTGCCAGCGAAATTACGGTAAGATTTGACCATGATCCTGTCAAATTGTTGGAGTTTC
TTCAAAACGACCAGAATCGTGATGAAGCGATTCAATTGGGTCTTATTGATGGACAGCCTGTGGCTGAACCCATCGTTTCTACAGAAACACTAAGG
CTGCTGAGTGAAACGAAGCAGCCAGCACAGTTCTCTACTTGATGTAACTGTGCTAGGTGACACCAAACCACTACTTTAACTACGGAGTTTCATCA
TGTTACGAAGAAGCCAGTAAACATATAAGTCTGCGAAGTCATTTCGCAGAACTGCTAGTAAGACGAAGTCTATCAATATGAGAAACGCTCCACA
GCGTGGCGGCTATCGTTTGTAATTATGGCCTGTTATAAGCCCTTAACGGCTTATCAAGCAGTGACAGGTCTATAATTTGGCGGAAATACCGGGTG
CGGATGTAGTTTCGCACCCTATCATTGCCTTGTGGTCAGTGTGTTGGTTGTCGCCTTGAACGCTCACGTCAGTGGGCGATTCGTTGTATGCATGA
GGCACAAATGCATACGAGTAATTGTTTATTACGTTGACATATGCTCCAGAGCATTGTCCTAAGGATATGTCATTGGATTACAATGACTATCAGCTT
TTTATGAAGCGGTTGCGTAACGTTTTACTGGGAAAACGATACGCTTTTATGGCAGGTGAATATGGTGAATCTTTTGATCGTCCTCATTTCCATGCT
TGTTTGTTTGGTCTTGATTTTCCGGATAAGAAAATATTTAAAAGAACGCAGACTGGCTCTATCCTCTACACGTCAGAGATTTTGAAGAATTGTGGC
CGTTGGCTATTCTACAATTGGTGATGTTACTTTTGAGTCTGCTGCTTATGTTGCAAGATATATTATGAAGAAGATTAATGGGGTTACCATCAACGA
GAATCATGAAGTTGTTGATGCGGTGCCCAATCAGTATTGTGATTAGAGACTGGTGAGATTATTCAGCGAAAGCCTGAATTTAATAAGATGTCTTT
GAAGCCCGGAATCGGGCAATCGTGGTTAGATAAGTACATGTCAGACGTTTTACGTCAGACCACGTTGTGGTGCGTGGCAAAAAGTGCCGACCA
CCACGTTTTATGATAATAATTTAAGTTGAAGTTTCCGGATCAGTTTGATATGATCCAGTTTGCCAGAGAGATGGAAGGTCGATCTAGGCATGAGG
ACAACACGCTTGAGCGACTTGCTGTAAAGGAAAAAGTTGCGTTGGCTAAGTTGTCATTGTTAAACGTACTATTTAAGGAGTTTTTATGAAGATGG
TTATTGTTTCTATTAAGGATACTGCTGCAGATGCTTTTGGTCGTCCAGCTTATGTTGCATCTGAAGTGTTGCAGTACGTCAGTTTCAGGATGAGGT
TAATCGAGCAAGCGAAGATAACCAGTTGTATAAGCATCTGATGATTTCATATGTTTTATTTGGGTCTTTTTGACGATGCTACTGGTGCTTTTGAAT
TACTGGAAAGCCCTAAGTTGATTGCTCGTGCAAAGAGTAATGATTCGCGAAGGCGATAAGGTTTTTAATACCGTATCACTCGTAAGAGTGGTAC
GGAACTACGGGAGATGTTTATGTTTCGCAATAAGTCAGTAAGTACGCATTCATTTGCTATGGTTCCTAAAGCGGACATTCCCCGCTCTAGTTTTAA
TACTCAATACGCTCATAAAACTACGTTTGATGCTGGTTTTTTAGTTCCTATTATTGTGATGAAGTATTGCCTGGCGATACTCATCGTGTGAAGATG
ACTGCATTTGCACGTTTGGCCACACCATTATTCCCTGTGATGGACAACTTGCATCTTGATACTTTCTTTTTCTTTGTTCCAAATCGTTTATTGTGGAA
CAATGCAAAAGTTTATGGGTGAACAGACTAACCCAGGTGATTCAATTTCGTTTGTAATACCTACTATTACCAGCCCTGCTGGTGGTTATGCTGTTT
CTATTTTTGATTATTTAGGTCTGCCTACTGCAGGTCAGATATACTGGTAGCAATACAGTAACTCACAACGTTTTACCGTTGAGAGCTTATAACGAG
ATTTATAACGAGTGGTTTAGAGATGAAAACTTACAGAATTCTGTAACGTTGAATCTTGGTGATTCAGGTGATGTCCCAGCGAATTACACGTTATT

GAGACGTGGTAAGCGCAAAGATTATTTTACAGGTGCGCTGCCTTGGCCACAAAAGGGTGCATCTGTTAGTTTGCCATAGGCACTTCTGCTCCTGT
TTTGGCACAGGTCGTGCCCTTGGTTTAACTGATGGTACTAATCAGTTTGGTGCTTCACAGTTTAACGGTGTTGCTACATTACACCGTTCTACTCCT
GCTCTTGGTACTTTAGTTGGTGCAGCCTCTGCTGCTACTTCTTCACCATCTACTGTTTCAGTTGGTGTTGTTACTTCTGGTGTATCTGGACTTTATGC
TGATTTGTCTACTGCTACTGC

>000107F|arrow

ATACTGTAATTGTTTTATTACTTTGACATATGCTCCAGAGCATTGTCTAAGGATATGTCATTGGATTACAATGATTATCAGCTTTTATGAAGCGGTT
ACGTAAGCGTTTACTGGGAAAACGATACGTTTTTATATGGCAGGTGAATATGGTGAATCTTTGATCGTCCTCATTTCCATGCTTGTCTGTTTGGTC
TGATTTTCCGGATAAGAAAAATATTTAAAAGAACGCAGACTGGCTCTATCCTTACACGTCAGAGATTTGGAAGAATTGTGGCCGTTTGGTATTCT
ACAATGGTGATGTTACTTTTGAGTTGATGCGTTATGTTGCAAGATATATTATGAAGAAGATTAATGGGGTTATGTCAATGAAAACCACGAAGTG
GTTGATGCGGGTGCCCATTATCAATATTGTGATTAGAGATGGTGAGTAATTCAGCGTAAGCCAGAATTTAATAGATGTCATTGAAGCCCGGTATC
GGGCAATCGTGGTTAGATAAGTACTGTCAGACGTTTATACGTCAGACCACGTTGTGGTGCGTGGCAAAAAGTGCCGTCCACCACGGTTTATGAT
AATAAATTTAAGTTGAAGTTTCCTGAAGAATTCGATATGATTCAGTTTGCCAGAGAGATGGAAGGTCGATCTAGGCATGAGGACAACACGCTTG
AGCGACTGCTGTTAAGGAAAAAGTTGCGTTGGCTAAGTTGTCATTGTTAAAACGTACTATTAAGGAGTTTTTATGAAGATGGTTATTGTTTCTATT
AAGGATACTGCTGCAGATGCTTTGGTCGTCCAGCTTATGTTGCATCTGAAGGTGTTGCAGTACGTCAGTTTCAGGATGAAGTCAATCGAGCTAGC
GAAGATAATCAGTTGTATAAACATCCTGATGATTTTCATATGTTCTATTTGGGTCTTTTTGACGATGCACTGGTTTTTGAACTACTGGAAAGCCCT
AAGTTGATTGCTCGTGCAAAAGATGTAATGATTCGCGAAGGCGAGTAAGGTTTTTTTTATACCGTATCACTCGAAATGAGTGGTACGGAACTAC
GGGAGATGTTTATGTTCGCAATAAGTCAGTAAGTACGCATTCATTTGCTATGGTTCCTAAAGCGGACATTCCCCGCTCTAGTTTTAATACTCAATA
TGCTCATAAAACCACGTTTGATGCTGGTTTTTTAGTTCCTATTTATTGTGATGAAGTATTGCCTGGCGATATCATCGTGTAAAGATGACTGCATTT
GCACGTTTGGCCACACCGTTATTTTCCTGGTGATGGACAATTGCATCTTGATACTTTCTTTTTCTTTGTACCCTAATCGTTACGTTGGAACAATGTG
GCCAAAGTTTATGGGTGAACAACGAATCTGGTGATTCTATCTTCTTTTGTAGTGCCTACTATACTAGTCTGCTGGTGGTTATGCTGTTTGTTCAATT
TTTGATTATTTGGTTACCTACTGCTGGTCAGATTACTGGCGCTAATACAGTAACGCATAATGTTTTGCCGTTACGTGCTTATATGAGATTTATAAC
GAATGGTTTAGAGATGAAAACTTACAGAATTCTGTAACGTTAAATCTTGGTGATTCAGGTGATGTTCCTGCTAACTATACACTTTTGAGACGTGG
TAAGCGTAAAGATTATTTTACTGGTGCATTGCCTTGGCCACAGAAGGGTGCTTCTGTTTCTTTACGTTAGGAACACGTGCTAATATTATTCTGACT
ACCAGCTGGCAATGGTACTGCTGGTTATAGTGTTTTTCAAACTGCTGTTGGTGCTTTAAGAGAATTAAATTCAGCTCTCATACTTTGTCTAATAGT
ACAAATGCTGGTGTGCTACTAATCAGTTATACGCTGATTTGTCTACTGCTACTGCTGCGACTATTAACCAACTTCGTCAATCTTTCCAGATTCAGAA
GTTATTGGAGCGCGATGCACGTGGTGGTACTCGTTATACTGAGTTACTACGTGCTCACTTTGGAGTAACTCCACAGGATTATCGTTTACAACGTC
CTGAAATATTGGTGGAGGTTCGACCCTTGTTAATGTTAATCCGATTGCTCAGACTTCTGCAACGTCGGTTACTGGTTCTGCTACTCCGCAAGGTAA
CCTGCTGCCAATGGGTTACTGCATTGGCTCAGGGACACGGCTTTACGTATGCTGCTCAAGAACATGGATACATTATCGGATTAGTTTCTGTACGT
GCTGACCTCACATATCAACAGGGTCTCCTAAGATGTGGTCTAGGTCTACACGATATGACTTTTATTTCCCAGTATTTGCCACTTGGGTGAGCAAGC
TATTTTGAACAAGAAATTATGTTCAAGGTACTGCAGCCGACAATGATGTATTGGTTATCAAGAACGTTGGCGGAGTATCGTTACAAACCTTCTCA
AATTACTGGTTTCTTTAGGTCTACTTCTGCTGGCACTATTGATGCTTGGCATTATGGACAGCGATTTACTTCTCTTCCTACGTGAATTCAACGTTTA
TCAAGAGACCCCTCCAGTTGCTCGTACTACGGCGGTCGGAGCTGCAGCAAATGGTCAGCAATTTTAATGGATGCTTTCTTTGATTGTCAGATGGC
CAGACCTATGCCTATGTACAGCGTACTGGTCTAATTGATCATTTCTAATGTTTTATATAACCTCGACTACTCCGTAAGGAGTAGTGAGGAAACAAC
CGAAGGGCGTTAGTTTATGTTTGTGGAATACTTGATGCGGTTACTAATGTTGGTTCTAAGCTGTCTTCAGCTTCTAGTTTCTTTACTCCTGGTGTCG
GTACTGCTTTGGGCGCTGTTGGTTCTTATTAGGTTCTACTTCTGCTAATAAAGCTAATCAGGAGATGGCTCAGAGGCAAATGGATTTTCAAGCCG
ATATGAGTGAACAAGTTACCAGCGTGCTGTTAAAGATTTAGAAGCTGTGGTTTATCTCCTATGTTAGCCTATCAACGTGGTGGTGCTTCTACCCCA
TCTGGTTCAACTGCTTACTATGGAAAATGTTTTAGTGTAATGCAACTAATTCAGCTATTTAATACTGCTTCTATGATGCAACAGATTTCGTAATGCA
TCAGAAAAGAAAAGCAGTTATCAGCCCAGACTGAAGCTACTGAAGGCTGGTACCCGTCCTAATACTAGGGCTGATACCTGTTAATAAGTCCTGC
TTACTGCGTCCTAATATTACAGCCGATAAATAAACGTATTTTTGCTGATATTGCTTTAAAGAATACGGACTGCGGATTTACCATCCGCTCAGTCATT
ATAATTACTAAGAGCTATTGGCTCCCATCCCCAGCTATTTTGGTCTAAGGGGGTTATCGATGCTTTCGACAAGAAATTTTTGATAAAACTCAAAAA
TAATCCTAATCAACTAAACCCTTGGGGAATTGGAGTCAAATAATGAGTAAAGCGCATTTTGCCATTTGTACGTAATCCGTAAACTATGATAAAGA
TGAAGCATCGGTAAACGATGCGTGCTGTGTCAAGGACCCAAGTCTTTGACTCTAACAGATATGAAAGAGGAATGTGACATAATGTCACATTGAA
CGTTCGGGGTTACAGGGGAACTTCCAACGGCCCCTGTATCGCCTCAATACGGCGATTTTAGTGGTGTTACTGATTACCATTCTGAGTTGAATCAA
CTTAAGCGCAACATACTGGACGATTTCATGGCTCTGCCAGCGAATTAAGAGTCCGATTTGACCCTGATCCTGTCAAATTCTTGGAGGTTCCTTGA
GAACGACCAGAATCGTGATGTAAGCGATTCAATGGGATCTTTATGATGGACAACCTGGTGGAAACCCAATCGTTTCTTACAGCAAACCACGTAA
GGGCCGAAGGATGAAATCCTGAGGCCAGCACAGTTACATCTACTTGATGTAACCCTGTGCCTGAGGTGAACACCAAACTCACTATTTTAACTTAC
GGAGTTCATCATGTTACGAAGAAAGCCAGTAAACAAATATAAATCTGCAAAGTCATTTCGCACGAACTGCTAGTAAGACGAAGTCAATTAATAT
GAGACACGCTCACCAGCGTGGTGGCTATCGTTTGTAATTATGGCCTGTTATAAGCCCTTAACGGCTTATCAATGCAGTGACAGGTCTATAATTTG
GCGGGAAATACCGGGTGCGGATGTAGTCCGTACCCTATCATTGCCTTGTGGTCAGTGTGTTGGTTGTCGCCCTTGAACGCTCACGTCAGTGG

>000077F|arrow

CTAGCGACTTTTGCTTATTTGCTGGTCTGCGACGCATCATTTTTTCTGTCCTTGTTTATCGTGTTTTTGTGGTTTGGTGTCACCTAGCACAGTTACAT
CAAGTAGGTAACTGTGCTTCCGAGGTTCCAACAGGGGTTGAAACCTCGGTTTGGGCTGGTTTTACCAGTCCCATTTTTTCAGCTTCGCTGCGATTT
TCAGGGTTTTCTAGAAAATCTATTAGATTCGCGGATCATTAGCGAATCGTTCACGAATATTGGCTGGTAAAGCCATAAATTCGTTGTCTGAAGCC
ATAATCTGGTTCAGAGCAGAGTGGTAGTCATAGACACCACTAAAGTCGCCATATTGAGGCGTTAAAGGAGTTTGAGGAATAAGTCCGGTCATAC
CGAACTTTTCCATAATATTATTGTATCACATTCTTCAGCAAATTGCTGCTGAGTCAGAGTTGCATCCTCACAATGCAGCCCTGACTCATTTGACGCA
GCAATCGTGTCGTAATTGTACGGAGTACGAAGAAATGGGGCTGTAATCTTTGTCATTTTCATTCCATTGGTGGGTGGTTATTTGTACGATTTTGTT
GAATCGGTTGACCTCGATAGTTATCGATGTATCGTTTTGCGCCAGATGCGCTATACGCATACTTTGCGATGTTTTTTACGTCTTGATAAATTGGTTT
GCTAGAACTAGGAGCTGACCCTGATTTAGCAAGTTTTGGTTAACTCGTTTGAATAACGAGATTGTGCCAAAGCTTGAGCAGCTTGAGCACTTGAT
GTTTGCCTATTTCATTTTTCATGAAAGGTATCGGCAAAGATTGTTTTATATTGAGCTCTAATAAGTTTGGATTTTCATCCAATTTATTTAACGTATCA
GCACGTACTTATCTGTTTGATTACTTGTGAGTTCTGTTTGAGCTTCAATTTGTTTAGATTGAGCAATAGCTTGATTTGCTTGCGCAAACCGTTTGAT
AAGCTTGGGTTCCGGACGTAGTTGCATTACCGAGAACATTTTGCATCTGGGCCATAGCTCCAGCTGGTGTTGTGGCTCCGCCTTGTGAATACGCA
AGCATGGGATTTAACCCAGCTTTTTTCATATCTTCAACTGCTCGTTGATATGATTTCCAGACATTTCAGCTTGAAATCTCTGTTGATTACTCGCTTGT
TCTGCACTCGCTGCGTTTTGACTTTGTGTTTCCAAAGAGTTGTCCTGCTGCACCTATTGCTGCAGGTGCAAGTGCAGCTAAGGAGAGTCCCCCAG
TAGCAGGGGCAGCTCCTATAGCTATAGCAGGGCCGATTAAATCGGCAATTCCGTCAAATAGTCCCATTAGAAATGGTCTATTAAGCCAGGTACT
GAATACATTGGCATTGGTCTTGCCATTTTGACATCAAAAAATGAGTCAAATAAGAATTGTTGGCCATTTGCAGCTGCTCCAACGGCTACTACACG
TGATACAGGAGGCGTATACTTGAATAAACGTATTATCAAAGTATGCGCAGCGGTAAAATTTTTGAGCCAAATGCCAACCATCGATCGTGCCCGCC
GATGTTGATTTGAACAAACCAGTAATTTAGATGGTTTGTAACGATATTCGCCCAGCGGTTCTTGATAGCCAAAAACAGTATTGTCGGCTGCAGTA
TCTCCTGTTGCATAAATTCTTTTTGCAAAACAAGATTGTTCGCCTAGAGTAGCAAATGCTGGAAATAAAGTCATATCGTGTAGATCTATACCACAT
ACGGTCTAATCCTTGTTGATAAGTAAGATCTGCTCTAATGGATACTAATCCAATAATAACGCCATGCTCAGTAAATGATTGAGTAAATCCATGATT
ATGAGCGAGAGCAGTAACCCATAGCAGCAAGGTTGCCCAAAGGGGTAGTCGTTCCAGAAGCGTTTGTTCCCGACGTTTGAGCAATCGGATTAAC
ATTAATTGGTGGTTGAAACCGCCTCCAAGGTATTCAGGCCTTTGTAAACGGGCGTCTGGGGAAATAACTCCAAAGTGACCTCCGGATAATTTCTG
TGTATCGTGTTCCGCCACGTGCTCCCTTTCAAGTAATTTTTGAATTTGAAATGACTGTCTTAATTGATTGACAGTTGCAGCAGTTGCTTCTGATAAG
TCGGTATATAAATTAGAAACGTTATTTACTACACCAGCAGTATTAACACCATAAGCGTTGCCATATCTAGCTAAAGCAGTCGTATTTCCAGGATCT
GTTTGAATTACCGTAAATTTATCGTTTGATGTTGCGTCTCCTGAAATGGTATCCCATTTAATTGGAGCCGTAGTACCTAAAAGTAAGGTGACACTC
GCACCTTTTTGTGGCCATGGTAATGCTGACGTAAAAGATCAGGCCTTTTACCACGACGTTTTAGCACATAGTTTGAGGAAGTAGCAGGGCCATCG
CCCTTATCAACTACTGCGCTTGTTTGTAAATTTTCATCTCGAACCATTCGTTCCAGATAAGAATTGTATGCACGTGGCCAAAAGGCACAGTGCGTA
ATAGTTCGGCCAGTATCAATTTGGCCTACTGTTGGTAGACCCAGATAGTCTTGAAGGCTGCCTACGGCATAACCATCTGTTTGGGCTTGTTTGTGT
TGGGACAATATAAGATATTGAGTCTGTTGGATTTTCTTGTTGACCCATAAATTTTTGCCAGTTATTCCATATAAGGCGATTGGGTACAAAGAAAAA
GAAAGAATCCATAATCATGTTATCCATGATTGGATATAAAGGCGTTGCTAGACGGGCAAATGCCGTCATTTTTAAGTTGAAAGTGTCCCCAGGG
AGCACTTCATCAACATATACAGGAATTAGATAGCCCGCATCGAAAGTCGTTTTATGTGTTTTTTTGAGCATCGAATTACTACGTGGTATATCGGCT
CTAGGTACCATCGCGAAGCGGTGAGTATTAACTGACTGATTGCGGTGCATGTTTTTCCTTAGTGTTGTTCCGGGGGAAAGATAAATCTCTTTCCC
CTCGGTTGTTTTATTTAAGTTTAACTTGTTTTCCTAATGATAGGAGTTTTGGTTGTTCATGTAAATCGAATAACCCAGTTGAATCGTCAAATGTTCC
GAATTCATATAGATCGAAATCATCAGGGTGATTAAAGAGTTGATTTTCAGTATCAGAACGATTAATTTCATCTGAAAAAGAGCGTATAGCTACTC
CAGAGGAAGGTACGAACATTGGTCGTGCATATGCTTCAGCAGCACGGTCTTTTACGGAAGCGAGGATAAGTTTCATTATTTTCCTAAGTAAGGTT
ACGTTTTAATAGTTGAAGTTTTGCCATAGTGACTTGTTCTTTTGCAGATAGTCGTTCTGGTGTATTGTCTTCGGAATTAAATTTAGCATTATTTTCCC
GCATGTAAAGTAATTCGTCATACTCATAAGGTTGGTCAATTTTAAACATTTTGTCATAGTATTTTGGTGGTTTGACCTTTTACCTCTAAGTATTACG
TAGTCTTGCGGGTATATATCCGAAGTATATTTTATATAAAAGTCTTTACCGATTCCCGGTTTTAAAGACATTTTATTATATTCCGGCTTTAAGTCTA
AATAGTCGCCGGTTTCAGGGTGTATGCGTTTGTAATGAGATTCCGCATCTTTCCCTGTTTGTTTTTTCATTATGTATCTAGCCACGTAGGCGGCTG
ATTCAAAAGTAACATCTCCAATGGTGGTATAACCAAATGGCCAGAGAGCTTCAAGTTCTGCGGATCTATATAACATAGAACCAGAGGCAGTCCTT
TTCCATAATTTTTTATCAGGAAAGTCGTATCCGAAGATACAGGCATGGAAGTGGGGTCTTGCGAAGAGTTCACCATATTCTCCAGCCATGTAATA
GCGGATTGTAAGTCCTCTTTTTGCGAGAGTTTTTCTAAGTCTTTTAAGGAACAATTGAAAGTCTTTGTGATCCAAAGAGCCATCGCTTGGGAGAT
GTGTATTGTCATATGTGAGTGTTATGAATGAGTTGTTTTTATGCAATTGGGCTTCGTGCATGCACCGAATAGCCCATTGTCTTGATCGTTCTAGAC
GGCATCCAACGCACTGCCCACAGGGCAGGTCTAAAGATCGAACGATGTCATGTTTCCGAACTTCGTCGAAAACAATTGATTTGTCAAAGCATTGA
TATGCTTTGAGAGGGTGATAACAAGGCATGTGAGGTGCCTGAAGACTTTATTAGAGTCTCCAGCCTCCACGCATTGGCGA

>000062F|arrow

GCTCTATATTTGGATTTTCATCCAATTTGTTTAAGTATCAGCAGTAAATTATCTGTTTGATTACTTGTGAGTTCTGTTTGAGCTTCAATTTGTTTAGT
TGGCATAGATGATTTGCTTGCGCAACCGTTTGATAAGCTTGGGTTCCGGACGTAGTTGCATTACCGAGAACATTTGCATATGGGCCATAGCTCAG
CTGGTGTTGTGGCTCGCCTTGTGAATACGCAAGCATGGGATTTAACCCAGCTTTTTTCATATCTTCAACTGCTCGTGATATGATGTTCCAGACATT
TCAGCTTGAAATCTCTGTTGATTACTCGCTGTTCTGCACTCCGCTGCGTTTTGACTTTGTGTTCCAAAGTATTGTCCTGTGCACCTATGCTGCAGGT
GCAAGTGCAGCTAAGGAGAGTCCCCAGTAGCAGGGGCAGTCCTATAGCTATAGCAGGGCCGATTAAATCGGCAATTCCGTCAAATAGTCCATTA
GAAATGGTCTATTAAGCCAGGTACTGAATAACATTGGCATTGGTCTTGCCATTTTGACATCAAAAAATGAGTCAAATAAGAATTGTTGGCCATTT

GCAGCTGCTCCAACGGCTAACACGTGATACAGGAGGCGTATCTTGAATAAACGTATTATTCAAAGTAGGCGCAGCGGTAAATTTTTGAGCCAAA
TGCCAACCATCGATCGTGCCCGCCGATGTTGATTTGAACAAACAGTAATTTTAGATGGTTTGTAACGATATTCCGCCCAGCGTTCGTGATATCCAA
AAAACAGTATTGTCGGCTGCAGTATTCCTGTTGCATAAATTTCTTTTTGCAAAACAGATTGTTCGCCTAGAGTAGCAAATGCTGGGAAATAAAAG
TCATATCGTGTAGATCTAGACCCATACGGTCTAATCCTTGTGATAAGTAAGATCTGCTCTAATGGATACTAATCCAATAATAACGCCATGCTCAGT
AAATGATTGAGTAAATCCATGATTATGAGCGAGAGCCAGTACCCATAGCAGCAAGGTTGCCCAAAGGGGTAGTCGTTCCAGAAGCGTTTGTTCC
CGACGTTTGAGCAATCGGATTAACATTAATTGGTGTTGAACCGCCTCCAAGGTATTCAGGCCTTTGTAAACGGGCGTCTGGGGAAATAACTCCAA
AGTGACTCCGGATAATTTCTGGTATCGTGTTCCGCCACGTGCATCCCTTTCAAGTAATTTTTGAATTTGAAATGACTGTCTTAATTGATTGACAGTT
GCAGCAGTGCTTCTGATAAGTCGGTATATAAATTAGAAACGTTATTTACTACACCAGCAGTATTACACCATAAGCGTTGCATACTAGCTAAAGCA
GTCGTATTTCCAGGATCTGTTTGAATTACCGTAAATTTATCGTTTGATGTTGCGTCTCCTGAAATGGTATCCATTTAATTGGAGCCGTAGTACCTAA
AGGTAAGGTGACACTCGCACTTTTTGTGGCCATGGTAATGCTGACGTAAAGTAATCATGTCTTTTACCACGACGTTTTAGCACATAGTTTGAGGA
AGTATCAGGGCCATCGCCCTTATCAACTACTGCGATTGTTTGTAAATTTTCATCTCGGAACCATTCGTTCCAATAAGATTGTATGCACGTGGCCAA
AAGGCACAGTGCGTAATAGTTCGGCCAGTATCAATTTGGCCTACTGTTGGTAAGCCCATATAGTCTTGAAGGCTGCCTACGGCATAACCATCTGT
TGGCTTGTTTGGTTGGGACAATATAGATATTGAGTCTGTTGGATTTTCTTGTTGACCCATAAATTTTGCCAGTTATTCCATATAAGGCGATTGGGT
ACAAAGAAAAAGAAAGAATCCATAATCATGTTGTCCATGATTGGATATAAAGGCGTGCTAGACGGGCAAATGCCGTCATTTTTAAGTTGAAAGT
GTCCCCAGGGAGCACTTCATCAACATATACAGGAATTAAATAGCCCGCATCGAAAGTCGTTTATGGTTTTTTGAGCATCGAATTTACTACGTGGT
ATATAGGCTCTAGGTACCATCGCGAAGCGGTGAGTATTAACTGACTGATTGCGGTGCATGTTTTTCCTTAGTGGTGTTCCGGGGGAAAGATAAA
TCTCTTTCCCCTCGGTTGTTTTATTTAAGTTTAACTTGTTTTCCTAATGATAGGAGTTTTGGTTGTTCATGTAAATCGAATAACCCAGTTGAATCGTC
AAAGTTCCGAATTCATATAGATCGAAGTCATCAGGGTGATTAAAAAGTTGATTTTCAGTATCAGAACGATTAATTCATCTGAAAAAGAGCGTATA
GCTACTCCAGAGGAAGGTACGAACATGGTCGTGCATATGCTTCAGCAGCACGGTTTTTACGGAAGCGAGGATAAGTTTCATTATTTTCCTAAGGT
TACGTTTTAATAGTTGAAGTTTTGCCAGTGATTGTTCTTTTGCAGATAGTCGTTCTGGTGTATTGTCTTCGGAATTAAGTTTAGCATTATTTTCCCG
CATGTAAAGTAATTCGTCATACTCATAAGGTTGGTCAATTTTAAACATTTTGTCATAGTATTTGGTGGTTGACCTTTTTAACCTCTAAGTATTACGT
AGTCTTGCGGGTATATATCCGAAGTATATTTTTATAAAAGTCTTTACCGATTCCCGGTTTTAAAGACATTATTATATTCCGGCTTTAAGTCTAAAAT
TCGCCGGTTTCAGGGTGTATGCGTTGTAATGAGATTCCGCATCTTTCCCTGTTGTTTTTTCATTATGTATCTAGCCAGTAGGCGGCTGATTCGAAA
GTAACATCTCAATGGTGGTATAACCAAATGGCCAGAGAGCTTCAAGTTGCGGATCTATATAACATAGAACCAGAGGCAGTCCTTTTCCATAATTT
TTATCAGGAAAGTCGTATCCGAAGATACAGGCATGGAAGTGGGTCTTGCGAAGAGTTCACCTATTCTCCAGCCATGTAATAGCGGATTGTAAGT
CCTCTTTTTGCGAGAGTTTTTCTAAGTCTTTTAAGGAACAGTTGAAAGTCTTTGTGATCCAAAGAGCCATCGCTTGGGAGATGTGTATTGTCATAT
GTGAGTGTTATGAATGAGTTGTTTTTATGCAATTGGGCTTCGTGCATGCACCGAATAGCCCATTGTCTTGATCGTTCTAGACGGCATCCAACGCA
CTGCCCACAGGGCAGGTCTAAAGATCGAACGATGTCATGTTTCCGAACTTCGTCGAAAACAATTGATTTGTCAAAGCATTGAAATGCTTTGAGAG
GGTGATAACAAGGCATGTGAGGTGCCTGGGGACTTTATTAGAGTCTCCAGCCTCCACGCATTGGCGAGTTTCGCATATTGCGTGTTTTGTATGTG
AAGCATGTTACGGAAAGTCCTAGCGGACTTTTGCTTATTTGCTGGTCTGCGACGCATCATTTTTCTGTCCTTGTTTATCGTGTTTTTGTGGTTTGGT
GTCACCTAGCACAGTTACATCAAGTAGGTAACTGTGCTTCCGAGGTTCCAACAGGGGTTGAAACCTCGGTTTGGGCTGGTTTTACCAGTCCCATT
TTTTCAGCTTCGCTGCGATTTTCAGGGTTTTCTAGAAAATCTATTAGATTCGCGGGATCATTAGCGAATCGTTCACGAATATTGGTGGTAAAGCCA
TAAATTCGTTGTCTGAAGCCATAATCTGGTTCAGAGCAGAGTGGTAGTCATAGACACCACTAAAGTCGCCATATTGAGGCGTTAAAGGAGTTTG
AGGAATAAGTCCGGTCATACCGAACTTTTCCATAATATTATTAATATCGCATTCTTCAGCAAATTGCTGCTGAGTCAGAGTTGCATCCTCACAATG
CAGCCCTGACTCATTTGACGCAGCAATCGTGTCGTAATTGTACGGAGTACGAAGAAATGGGGCTGTAATCTTTGTCATTTTCATTCCATTGGTGG
TTGGTTATTTGTACGATTTTGTTGAATCGGTTGACCTCGATAGTTATCGATGTATCGTTTTGCGCCAGATGCGCTATACGCATCTTTGGCGATTTTT
TACGTCTTGATAAATTGGTTTGCTAGAACTAGGAGCTGACCCTGATTTAGCAAGTTTTGTTAACTCGTTTGAATAACGAGATTGTGCCAAAGCTTG
AGCAGCTGAGCATTGATGTTTTGCCTATTTCATTTTTCATGAAAGTATCGGCAAGTATT

>000059F|arrow

TTCGCTTCACCCATAAACTTAACCCAATGTTCCAAACTAATCTATTGGAACAAAGAAAAAGAAGTATCTAATGCAAATTGTCCATAACTGGAAACA
AAGGCGTTGCCAAACGGGAAACATTGTAAGCCTTTACATTGTGCATGTCCCCTGGGAGCACTTCATCACAATAAATAGGAACTAGTAACCACCCA
TCAAAAGTAGTTTTATGCGCATATTGCGTATCAAAACTAGAACGCGGAATTTCCGCTTTAGGAACCATAGCAAAACTATGTGAGCTTACTGACTT
ATTACGATGCATAACATCTCCCGAGTTTCCGAACCACTAGCAGCTAGTGATCCGGCTTAAAAAAAACATTACTCGCCATCGCGAATCATAACATCC
TTAGCTCTAGAAATAAGCTTGGGAGAACCAAGCAAATCCATAGTACCAGAATTATCATCAAACGTACCAAAATAATATAAACTGAAAATCATCAG
GGTGTTTATATAACTGATTATCATCGCAGCGCGATTAACTTCGTCCTGAAACTGACGAACAGCAACACCCTCGATGCAACAAAAGCTGGACGACC
ATACGCACCAGCTGCAGTATCTAAATAGAAACAATAACCATCTTCATAAAAACTCCTTTAAATCTACGTTTAAAAGCGATAACTTAGCCAAAGCGA
CTTTTTCCTTTACAGCCAAACGCGCTAAGTGTTATCTTCAAAATGTGAGCGACCTTCTAGCTCACGAGCATATTGTATACCCATCAAATTCTTTGGA
AACAATACTTTAAACTTATTATCATAAAACCGTGGTGGTCGGCACTTTTGCCCGCACCACAACAGAGTCAGTCGTATAAACGTCTGACATGAACTT
ATCAAACCAAGGACCATGCCAGGCTTAGAGACATCTTATTAAATTCAGGCTACGCTGAATAATCTCACCAGTATCTAATCACAATACTGATAATG
GCATCTGCATCAACCACTTCGTGGTTCTCATTAACGGTTTTACCGTTAATCTTCTTCATAATATATCGAGCAACATAAGCAGCAGATTCAAATTGAC
ATCACCAATAGAACTATAGCCATACGGCCAAAGTTCTTCAAGTATCTTTGACGTATATAAGATAGACCCAGTCTGCGTTCTTTGAAAACTTCTTAT

CTTCAAATCAAGCCAAAGATACAAGCATGGAAATGAGGACGATCGAAAGACTCACCATATTCACCTGCCATATAAAAACGAATAGTCTTGCCAG
TATAGCGTTTTCTCAATCGTTTCATAAACAATTGAAAATCTTCATAATGCAAAGACATATCCTTAGGACAATGCTCTGGAGCATATGTCAAAGTAA
TAAAACAATTACTAGTATGCATTTGTGCCTCATGCATACATCTAACGGCCCACTGACGGGACCGTTCAAGGCGACAACCAACACACTGACCACAA
GGCAATGACAAGGTACGGACTACGTCCGCCCCTGGTATCTCCCGCCAAATAATAGATGTCAGCGCATTGATAAGCCGTTAACGGCTTATAACAC
GCCATAAATTACAGTCTAAAACCACCGCGCTGCGGTGAAGTACGCATATTAATGCTCTTGGTCTTGCTTACGCCACGACGAAACTTCTTAGCTGC
GCCATGCTTGCTCATTGGTTTTCTATAAGGCTCTAACATTGCACTCCGTAGTTAATAATGGGTTTTGGTGTCACCTAGCACAAGTTACATCAAGTA
GAGTAACTGTGGCTTGCCATCCGCTTACGCGTCTGGCTTAGGTGTTTCTACTGCAGAAAACGACGGGTTTCAACCACAGGTTTACCGTCAATAAG
ACCATCTGAATCGCTCATCACGATTCTCTTCATTCTGTAAATAATTTAACAAAGCATTAGGATCGTTATCAAACTTAGCCCTAATCTTAGCTGGCAA
AGCCATAAAAGCCTCATCAGAGGCACGAATAGCATTCAATGCGGTGTGATAGTCAGATACACCGCTAAATCGCCTATGATGGCTCTAATGTGCG
CCTGGGGTCAATTGCCCAGTAACGCCAAAAACGCTCAACTATATATTAATATCACATTCGTCTCTCATGTGTTGTTGAGCCAAACTCGGATCTTTA
CATTCAAGACCAGTCTCTTGTGAAACAAGAGCCATATCATAATTGTACGGATTACGTACAAAAGGTAAATTCGTTTTACTCATTTCTACGACCTTC
CAAGGACCAAGGAAAAGACTTATTCTTTTCCAAAAACTTATCAAAAATAGACTTACTACCTTTCTTAATATCGCGATTACCAATATGGATCGCTAG
ACGGTGTTCGTAACTCTAAATTATTCTTAGCATCAATATCAGTCTTAATTGTTGATGCAGAATTAGCATTAACACGAGAACCAGCCTCGGCCTGTT
CCAAATAAAAACGAGCCGGTTCTTGTAAACACGCTGAGAAGCTAAATCAGCTTCTTCAGCAACCTTCCTAGCAGTCTGAACATTTAATTCACGTTG
AGATTTAGCAACTTCCATCTGCTCACGCAATAAATCACTCTCAACATCACGCTTAACAGCTCCACTCAAATCAGACTTAACTGGAGAAGTAGAAGA
TGCAGTAGCACCACTCGGAACAGTAGAACCACCTTTAGAATATGCAAGCATAGGACTCAAACCAGCCTTATTCAAATCAGTAACCATACGCTGAT
ATTGCGTATTGGACATATCTTCTTGAAAGCGTCTATTTGCTTGCGCTTCAGCAGTATTATATTTTTGCTCTTCATTCTGACTCCAAGCAGAAGTAGC
AAAATCAAATAAACCACCAGCAACTGAATCAAACATACCCATACTAACGCGCTCCGCTTGTTTGCTGACTACTGGTTTCCCAGTAGTCCAGCTTAT
ATTACATTAGAAATGGTCAATCAAGCCAGGTACAGAATACATCGGCATTGGACGAGCCATCTTACAATCAAAAAACGCATCCATCAAAAACTGCT
GACCATTAGCAGATGCACCAACAGCAGTAGTACGATCAATAGGTGGCGTTTCTTGAATAAACGTAGAATTCAAAGTCGGCAACGAAGTGAACTT
CTGAGCATAATGCCAAGGGTCAATCGTACCAGCTGAAGTCGACTTAAACAAACCTGTAATCTGAGAAGGTTTGTAACGGTACTCTGCCCAACGTT
CCTGGTATCCAAATACATCATCATCAGTTGAAGTACCAGTAACATAGATTTCCTTATTAAGAACAGCCTGTTCACCTAAATGAGCAAATACAGGG
AAATAGAAATCATAACGTGTCTCACGAGACCACATCTTAGGTAAACCTTGCTGATATGTTAGATCAGCTCTTACGTTTACCAAACCAATTATGTAT
CCATGTTCTTGAGCATGATACGTAAAACCATGTCCACTAGCCAACGCAGTACCCATTGCAGCCAAGTTACCAAGCGGAGTAGCACCGCCAGAAAT
CGAAGTAGCAGACGTTTGAGCAATAGGATTAACGTTGACATAAGTAGAACCACCACCAATATATTCAGGACGTTGTAAACGATAATCTTGTGGA
GTTACACCAAAATGAGCACGTAACAATTCTGTATAGCGTGTACCACCTCGCGCATCGCGCTCTAACAAACGCTGAATCTGGAAAGACTGACGTAA
CTGGTTAATAGTTGCAGCCGTAGCATCACTTAAATCGGCATACAAACCAGTACCAGCAGTACCAGCGTTATTACTACGATACACAGCATGTGTAG
TTGAATTAGCATAAATCTGCTTCAAAGCACCAGCACCGTCAACTAATGACAACGTTGTTGAATCATTAGTAACAGACGTCTTAATAGGAGCAGAC
GTGCCTAAAGGCAACGTAACTGCATCGCCTTTCTGAGGCCAAGGCAAGGCACCAGTAAAATAATCCTTACGCTTACCGCGTCGAACCATAGCATA
ATCACTCGGAGTATCAGGACCGTCACCCTTGTGAACGGTAACAGAATTTTGTAAATTCTCATCCCTAAACCACTCGTTATAAATCAAATTATAAGC
ACGTAACGGTAACGCGTTATGCGTAACCGTATTAGTACCAGTAATCTGACCAGCCGTAGGCAAACCAAAATGATCAAAAATAGAACCTACTGCA
TAACCACCAGCAGTAGAAGTAATCTGTGGAACTACATACGAAATAGAATCACCTGGGTTCGCTTGCT

>000055F|arrow

CATTCAGCATAACGTCCCACAAGATGTAAGGATATTCTTTAGATGACGTGCATATGACACTGCTGGGCAAAACAAGTTCGAATAAGGCAGTGCC
GTCCATGGCCTCTCTTCCTCGGCCGTTTTTGAATAATCAGCAATCTTAGACTGGTGAACCACACTTCCCTTTAGGAGCCGCGCATCATAGGTGCTC
ATTTATACAAAAAAACAGGGTGCGTTCCTTCCTTAAACATACCGAATACACATAATATATTCATATTCTGCTAACCTTACCGAGAAAACTTTCATTA
CCCTGTAACCAGCAGGTCGCAATCTATCCTTAATTTTTCTTAGCCATTTCACAACTCTGGCACAAAATATGACCATGCTCCTTCTGAGTACGAGATT
ATCCCTTTATGCTTAAAAAACAAAGACAGAAAAAAAAAAAACTTTTCTAAAACAATCTAAATCTATATGAGAAATAGACTCGGACGGACACATAA
TCAGCAATTTCAGGAAATTCGCTCGTTTCGACCGCTTCGCTCGACACACAATAAGTAGTGTGTCGATCTTCCATTTTGAGCGATTCGTGGGAACCA
ACTTATATACTCTAAACGGATAATATAATGGACACCAAACGCTACCGCGTTTGTGTCAATCAACTTGCGGGCCTTCGGCCCAAATACAATTACACA
CAATATTTAAATTACATACACATTTATTTAAAATACACATTAGAGTCCCTTTATCGGGAATTAAGAAGCTTCAAAGTGTATAAATACTCAATACGA
ACTAGGAATTTGCCAGAATTCCAAGGAGTAACACCATTTGAATCAAGAATCCACTTGAGTCCATAATGCGGAGTATCAGCAGCATTAATATCAAG
TGACTTAGGCCAACAAGGCTCATAAGCCGTTGTTATAGCAGAGCGATAAATCTGTCTTAAAACAGCAGGTTTAAGCCGTACAGATATAATAGTAT
CAGGCTTCATGACCCTACATTTCGCCTTACCAACTTCACGCATAGCGGCAAGATTAATAGCAGAACTATCATCATAATCTCTATAATACCACAACT
TGGGATACCAAGTAGGATTGTTTATATCCGTAGCCGGAATACCACCAGGTGGATAAATTTGATCAGGATTTGATATGAGCTGAAACTTCACAGTA
ACAGAACAAATCTGATAACGATCATAAAGAGCAGTAAGCTCAGAATAACCAGATACATTTTGCAACGTAAAAGCGATTGCTTGATCCGTTTCAGT
ACCTGTACAATCTACCGTATTAGCAGTACCGTGACGACGATACATATGTACGTTGGACGACAAACCACGACGAAGAGATGGTTTAACGGTACGA
CGACGTTTATAACTAGTACGTTTAGGAGTTTTTGAAAAGACACGTTTTCGTTTATAAGGCATATTATATGAAAATCAATTTTTACTTATATACTAAA
AATTTTTAAAATCTCTTAGGATTCCACCAATCCATATCATAATCATCTTTTTGACCAACTCGGAAATTAAAATCCTCGACATAATGATCATCGACAC
CCTGTTTGACCATTTGATGACAAGGCCTCCATTTGGTATTTTTCATCATTTCTTTAACACATTCACGTAAAGTCCAATGAGACTTCTTAGATCGACG
TGCATATGACACTGCTTGGCAAACCAAGTTCGAATAAGTCAGTTGCCAGTCCCTGTCCTTCTTTTCCTCGGCCGTTTTTGAATAATCAGAA

>000052F|arrow

GCTCTATATTTGGATTTTCATCCAATTTGTTTAAGTATCAGCAGTAAATTATCTGTTTGATTACTTGTGAGTTCTGTTTGAGCTTCAATTTGTTTAGT
TGGCATAGATGATTTGCTTGCGCAACCGTTTGATAAGCTTGGGTTCCGGACGTAGTTGCATTACCGAGAACATTTGCATATGGGCCATAGCTCAG
CTGGTGTTGTGGCTCGCCTTGTGAATACGCAAGCATGGGATTTAACCCAGCTTTTTTCATATCTTCAACTGCTCGTGATATGATGTTCCAGACATT
TCAGCTTGAAATCTCTGTTGATTACTCGCTGTTCTGCACTCCGCTGCGTTTTGACTTTGTGTTCCAAAGTATTGTCCTGTGCACCTATGCTGCAGGT
GCAAGTGCAGCTAAGGAGAGTCCCCAGTAGCAGGGGCAGTCCTATAGCTATAGCAGGGCCGATTAAATCGGCAATTCCGTCAAATAGTCCATTA
GAAATGGTCTATTAAGCCAGGTACTGAATAACATTGGCATTGGTCTTGCCATTTTGACATCAAAAAATGAGTCAAATAAGAATTGTTGGCCATTT
GCAGCTGCTCCAACGGCTAACACGTGATACAGGAGGCGTATCTTGAATAAACGTATTATTCAAAGTAGGCGCAGCGGTAAATTTTTGAGCCAAA
TGCCAACCATCGATCGTGCCCGCCGATGTTGATTTGAACAAACAGTAATTTTAGATGGTTTGTAACGATATTCCGCCCAGCGTTCGTGATATCCAA
AAAACAGTATTGTCGGCTGCAGTATTCCTGTTGCATAAATTTCTTTTTGCAAAACAGATTGTTCGCCTAGAGTAGCAAATGCTGGGAAATAAAAG
TCATATCGTGTAGATCTAGACCCATACGGTCTAATCCTTGTGATAAGTAAGATCTGCTCTAATGGATACTAATCCAATAATAACGCCATGCTCAGT
AAATGATTGAGTAAATCCATGATTATGAGCGAGAGCCAGTACCCATAGCAGCAAGGTTGCCCAAAGGGGTAGTCGTTCCAGAAGCGTTTGTTCC
CGACGTTTGAGCAATCGGATTAACATTAATTGGTGTTGAACCGCCTCCAAGGTATTCAGGCCTTTGTAAACGGGCGTCTGGGGAAATAACTCCAA
AGTGACTCCGGATAATTTCTGGTATCGTGTTCCGCCACGTGCATCCCTTTCAAGTAATTTTTGAATTTGAAATGACTGTCTTAATTGATTGACAGTT
GCAGCAGTGCTTCTGATAAGTCGGTATATAAATTAGAAACGTTATTTACTACACCAGCAGTATTACACCATAAGCGTTGCATACTAGCTAAAGCA
GTCGTATTTCCAGGATCTGTTTGAATTACCGTAAATTTATCGTTTGATGTTGCGTCTCCTGAAATGGTATCCATTTAATTGGAGCCGTAGTACCTAA
AGGTAAGGTGACACTCGCACTTTTTGTGGCCATGGTAATGCTGACGTAAAGTAATCATGTCTTTTACCACGACGTTTTAGCACATAGTTTGAGGA
AGTATCAGGGCCATCGCCCTTATCAACTACTGCGATTGTTTGTAAATTTTCATCTCGGAACCATTCGTTCCAATAAGATTGTATGCACGTGGCCAA
AAGGCACAGTGCGTAATAGTTCGGCCAGTATCAATTTGGCCTACTGTTGGTAAGCCCATATAGTCTTGAAGGCTGCCTACGGCATAACCATCTGT
TGGCTTGTTTGGTTGGGACAATATAGATATTGAGTCTGTTGGATTTTCTTGTTGACCCATAAATTTTGCCAGTTATTCCATATAAGGCGATTGGGT
ACAAAGAAAAAGAAAGAATCCATAATCATGTTGTCCATGATTGGATATAAAGGCGTGCTAGACGGGCAAATGCCGTCATTTTTAAGTTGAAAGT
GTCCCCAGGGAGCACTTCATCAACATATACAGGAATTAAATAGCCCGCATCGAAAGTCGTTTATGGTTTTTTGAGCATCGAATTTACTACGTGGT
ATATAGGCTCTAGGTACCATCGCGAAGCGGTGAGTATTAACTGACTGATTGCGGTGCATGTTTTTCCTTAGTGGTGTTCCGGGGGAAAGATAAA
TCTCTTTCCCCTCGGTTGTTTTATTTAAGTTTAACTTGTTTTCCTAATGATAGGAGTTTTGGTTGTTCATGTAAATCGAATAACCCAGTTGAATCGTC
AAAGTTCCGAATTCATATAGATCGAAGTCATCAGGGTGATTAAAAAGTTGATTTTCAGTATCAGAACGATTAATTCATCTGAAAAAGAGCGTATA
GCTACTCCAGAGGAAGGTACGAACATGGTCGTGCATATGCTTCAGCAGCACGGTTTTTACGGAAGCGAGGATAAGTTTCATTATTTTCCTAAGGT
TACGTTTTAATAGTTGAAGTTTTGCCAGTGATTGTTCTTTTGCAGATAGTCGTTCTGGTGTATTGTCTTCGGAATTAAGTTTAGCATTATTTTCCCG
CATGTAAAGTAATTCGTCATACTCATAAGGTTGGTCAATTTTAAACATTTTGTCATAGTATTTGGTGGTTGACCTTTTTAACCTCTAAGTATTACGT
AGTCTTGCGGGTATATATCCGAAGTATATTTTTATAAAAGTCTTTACCGATTCCCGGTTTTAAAGACATTATTATATTCCGGCTTTAAGTCTAAAAT
TCGCCGGTTTCAGGGTGTATGCGTTGTAATGAGATTCCGCATCTTTCCCTGTTGTTTTTTCATTATGTATCTAGCCAGTAGGCGGCTGATTCGAAA
GTAACATCTCAATGGTGGTATAACCAAATGGCCAGAGAGCTTCAAGTTGCGGATCTATATAACATAGAACCAGAGGCAGTCCTTTTCCATAATTT
TTATCAGGAAAGTCGTATCCGAAGATACAGGCATGGAAGTGGGTCTTGCGAAGAGTTCACCTATTCTCCAGCCATGTAATAGCGGATTGTAAGT
CCTCTTTTTGCGAGAGTTTTTCTAAGTCTTTTAAGGAACAGTTGAAAGTCTTTGTGATCCAAAGAGCCATCGCTTGGGAGATGTGTATTGTCATAT
GTGAGTGTTATGAATGAGTTGTTTTTATGCAATTGGGCTTCGTGCATGCACCGAATAGCCCATTGTCTTGATCGTTCTAGACGGCATCCAACGCA
CTGCCCACAGGGCAGGTCTAAAGATCGAACGATGTCATGTTTCCGAACTTCGTCGAAAACAATTGATTTGTCCCCGCTTGATTGCTTTGCGAGGG
TGTCCCAAGGGTGGTGATCCTGGGGATCCTTTAGTTCGCGTTCCCACCTCCACGCTTGGCGCGTTTCCATATTTGCGTGTTGTTGTGAAGCATGTT
ACGGAAGTCTAGCGGACTTGTGCTTCTATGGTCTGCCGCCGCCATCCTTTTTCTGTCCTTGTTTATCGGTTTTTGTGGTTTTGGTGTCACCTAGCAC
AGTTACATCAAGTAGGTAACTGTGCTTCCGAGGTTCCAACAGGGGTTGAAACCTCGGTTTTGGGCTGGTTTTACCAGTCCATTTTTTCAGCTTTCG
CCTGCGATTTTCAGGTTTTCTAGAAATCTATAGATTCGCGGGATCATTAGCGAATCGTTCACGAATATTGGCTGTAAAGCCATAAATTCGTTGTCT
GAAGCCATAATCTGGTTCAGAGCAGAGTGGTAGTCATAGACACCACTAAAGTCGCCATATGAGGCGATTAAAGGAGTTTGAGGAATAAGTCCG
GTCATACCGAACTTTTCCATACTATTATTAATATCGCATTCTTCAGCAAATTGCTGCTGAGTCAGAGTTGCATCCTCACAATGCAGCCCTGACTCAT
TTGACGCAGCAATCGTGTCGTAATTGTACGGAGTACGAAGAAATGGGGCTGTAATCTTTGTCATTTTCATTCCATTGGTGGTTGGTTATTTGTAC
GATTTTGTTGAATCGGTTGACCTCGATAGTTATCGATGTATCGTTTTTGCGCCAGATGCGCTATACGCATCTTTGGCGATGTTTTTTACGTCTTGAT
AAATTGGTTTGCTAGAACTAGGAGCTGACCCTGATTTAGCAAGTTTTGTTAACTCGTTTGAATAACGAGATTGTGCCAAAGCTTGAGCAGCTTGA
GCACTTGATGTTTTGCCTATTTCATTTTTCATGAAAGT

>000046F|arrow

TTAGGGTAGTGCATTGTCTACTATTTTGGGTGGTCTTGGCTTACTTCGTGGAAGTTCTGCCATGCCTTTTGTAACACAACAAGCTCCGAAAGGTAA
AAAATGAGTAAAGCGAATTGCCATTTGTACGTAATCCGTACAATTATGATAAAGATGAAGCATCGTTAGCCGATGCGTTGCTGTGTCAAGACCCA
AGTCTGCTCAACAGCATATGAAAGATGAATGTGACATTAATGTCATCATTGAACGTTTTGGGGTTACAGGGGAACTTCCAACGGCCCTGTTATCG
CCACAATATGGCGATTTTAGTGGTGTTACTGACTACCACTCTGCGTTGAATCAAATTAACGCAACTATGGACGATTTCATGGCTTTGCCAGCGAA
AATTACGTGTAAGATTTGACCATGATCCTGTCAAATTTGTTGGAGTTTCTTCAAAACGACCAGAATCGTGATGAAGCGATTCAATTGGGTCTTATT

GATGGACAGCCTGTGGCTGAACCCATCGTTTCTACAGAAACACCTAAGGCTGCTGAGTGAAACGAAGCAGCCAGCACAGTTACTCTACTTGATG
TAACTGTGCTAGGTGACACCAAACCACTACTTTAACTACGGAGTTCATCATGTTACGAAGAAAGCCAGTAAACAAATATAAGTCTGCGAAGTCAT
TTCGCAGAACTGCTAGTAAGACGAAGTCTATCAATATGAGAAACGCTCCACAGCGTGGCGGCTATCGTTTGTAATTATGGCCTGTTATAAGCCCT
TAACGGCTTATCAATGCAGTGACAGGTCTATAATTTGGCGGGAAATACCGGGTGCGGATGTAGTTCGCACCCTATCATTGCCTTGTGGTCAGTGT
GTTGGTTGTCGCCTTGAACGCTCACGTCAGTGGGCGATTCGTTGTATGCATGAGGCACAAATGCATACGAGTAATTGTTTATTACGTTGACATA
TGCTCCAGAGCATTGTCCTAAGGATATGTCATTGGATTACAATGACTATCAGCTTTTTATGAAGCGGTTGCGTAAACGTTTTACTGGGAAAACGA
TACGCTTTTATATGGCAGGTGAATATGGTGAATCTTTTGATCGTCCTCATTTCCATGCTTGTTTGTTTGGTCTTGATTTTCCGGATAAGAAAATATT
TAAAAGAACGCAGACTGGCTCTATCCTCTACACGTCAGAGATTTTGGAAGAATTGTGGCCGTTTGGCTATTCTACAATTGGTGATGTTACTTTTGA
GTCTGCTGCTTATGTTGCAAGATATATTATGAAGAAGATTAATGGGGTTACCATCAACGAGAATCATGAAGTTGTTGATGCGGGTGCCCATTATC
AGTATTGTGATTTAGAGACTGGTGAGATTATTCAGCGAAAGCCTGAATTTAATAAGATGTCTTTGAAGCCCGGAATCGGGCAATCGTGGTTAGA
TAAGTACATGTCAGACGTTTATACGTCAGACCACGTTGTGGTGCGTGGCAAAAAGTGCCGACCACCACGTTTTTATGATAATAAATTTAAGTTGA
AGTTTCCGGATCAGTTTGATATGATCCAGTTTGCCAGAGAGATGGAAGGTCGATCTAGGCATGAGGACAACACGCTTGAGCGACTTGCTGTAAA
GGAAAAAGTTGCGTTGGCTAAGTTGTCATTGTTAAAACGTACTATTTAAGGAGTTTTTATGAAGATGGTTATTGTTTCTATTAAGGATACTGCTGC
AGATGCTTTTGGTCGTCCAGCTTATGTTGCATCTGAAGGTGTTGCAGTACGTCAGTTTCAGGATGAGGTTAATCGAGCAAGCGAAGATAACCAG
TTGTATAAGCATCCTGATGATTTTCATATGTTTTATTTGGGTCTTTTTGACGATGCTACTGGTGCTTTTGAATTACTGGAAAGCCCTAAGTTGATTG
CTCGTGCAAAAGATGTAATGATTCGCGAAGGCGAGTAAGGTTTTTTATATACCGTATCACTCGTAAGAGTGGTACGGAACTACGGGAGATGTTT
ATGTTTCGCAATAAGTCAGTAAGTACGCATTCATTTGCTATGGTTCCTAAAGCGGACATTCCCCGCTCTAGTTTTAATACTCAATACGCTCATAAA
ACTACGTTTGATGCTGGTTTTTTAGTTCCTATTTATTGTGATGAAGTATTGCCTGGCGATACTCATCGTGTGAAGATGACTGCATTTGCACGTTTG
GCCACACCATTATTCCCTGTGATGGACAACTTGCATCTTGATACTTTCTTTTTCTTTGTTCCAAATCGTTTATTGTGGAACAATTGGCAAAAGTTTAT
GGGTGAACAGACTAACCCAGGTGATTCAATTTCGTTTGTAATACCTACTATTACCAGCCCTGCTGGTGGTTATGCTGTTGGTTCTATTTTTGATTAT
TTAGGTCTGCCTACTGCAGGTCAGATTACTGGTAGCAATACAGTAACTCACAACGTTTTACCGTTGAGAGCTTATAACGAGATTTATAACGAGTG
GTTTAGAGATGAAAACTTACAGAATTCTGTAACGTTGAATCTTGGTGATTCAGGTGATGTCCCAGCGAATTACACGTTATTGAGACGTGGTAAGC
GCAAAGATTATTTTACAGGTGCGCTGCCTTGGCCACAAAAGGGTGCATCTGTTAGTTTGCCATTAGGCACTTCTGCTCCTGTTTTTGGCACAGGTC
GTGCCCTTGGTTTAACTGATGGTACTAATCAGTTTGGTGCTTCACAGTTTAACGGTGTTGCTACATTACACCGTTCTACTCCTGCTCTTGGTACTTT
AGTTGGTGCAGCCTCTGCTGCTACTTCTTCACCATCTACTGTTTCAGTTGGTGTTGTTACTTCTGGTGTATCTGGACTTTATGCTGATTTGTCTACT
GCTACTGCAGCAACGATCAATCAGCTTCGTCAGTCATTCCAGATTCAGAAGTTGCTTGAGCGCGATGCGCGAGGTGGTACACGTTATACAGAATT
GTTACGTGCGCATTTTGGTGTAACTCCACAGGACTATCGTTTACAACGTCCTGAATATATTGGTGGAGGTTCTACTCTTGTTAATGTTAATCCGAT
TGCTCAGACTTCTGCAACGTCAGTTACTGGTTCTGCTACTCCGCAAGGTAACCTTGCTGCAATGGGTACTGCATTGGCTCAGGGACACGGTTTTA
CGTATGCTGCTCAAGAACATGGATACATTATCGGATTAGTTTCAGTTCGTGCGGATTTGACATATCAACAAGGTTTACCGAGGATGTGGTCAAGA
TCTACACGTTATGATTTTTATTTCCCAGTTTTTGCGACACTTGGTGAGCAAGCTATTCTTAATAAAGAAATTTATGTAACTGGTACATCTACTGATA
ATGATGTGTTTGGTTATCAAGAGCGTTGGGCTGAGTATCGCTATAAACCATCACAAATTACTGGTTTAATGAGATCTACAGCTGCTGGTACTATT
GATGCCTGGCATTATGGTCAACGTTTTACTAGTTTGCCTACTCTTAATTCTTCGTTTATTCAAGAGACCCCTCCAGTCGCTCGTACTACGGCTGTTG
GTACCGCAGCAAATGGTCAGCAATTTTTAATGGATGCTTTCTTTGACTGCAATATGGCTCGACCAATGCCGATGTATAGCGTACCGGGTTTAATT
GATCATTTTTAATGTTTTTATAACCTCGACTACTCCGTAAGGAGTAGTCGAGGTTATAAAAACATTAAAAATGATCAATTAAACCCGGTACGCTAT
ACATCGGCATTGGTCGAGCCATA

>000026F|arrow

TCTGCACTCGCTGCGTTTTGACTTTGTGTTCCAAAGTATTGTCCTGCTGCACCTATTGCTGCAGGTGCAAGTGCAGCTAAGGAGAGTCCCCCAGTA
GCAGGGGCAGCTCCTATAGCTATAGCAGGGCCGATTAAATCGGCAATTCCGTCAAATAGTCCCATTAGAAATGGTCTATTAAGCCAGGTACTGA
ATACATTGGCATTGGTCTTGCCATTTTGACATCAAAAAATGAGTCAAATAAGAATTGTTGGCCATTTGCAGCTGCTCCAACGGCTACTACACGTG
ATACAGGAGGCGTATCTTGAATAAACGTATTATTCAAAGTAGGCGCAGCGGTAAATTTTTGAGCCAAATGCCAACCATCGATCGTGCCCGCCGA
TGTTGATTTGAACAAACCAGTAATTTTAGATGGTTTGTAACGATATTCCGCCCAGCGTTCTTGATATCCAAAAACAGTATTGTCGGCTGCAGTATC
TCCTGTTGCATAAATTTCTTTTTGCAAAACAGATTGTTCGCCTAGAGTAGCAAATGCTGGGAAATAAAAGTCATATCGTGTAGATCTAGACCACAT
ACGGTCTAATCCTTGTTGATAAGTAAGATCTGCTCTAATGGATACTAATCCAATAATAACGCCATGCTCAGTAAATGATTGAGTAAATCCATGATT
ATGAGCGAGAGCAGTACCCATAGCAGCAAGGTTGCCCAAAGGGGTAGTCGTTCCAGAAGCGTTTGTTCCCGACGTTTGAGCAATCGGATTAACA
TTAATTGGTGTTGAACCGCCTCCAAGGTATTCAGGCCTTTGTAAACGGGCGTCTGGGGAAATAACTCCAAAGTGACTCCGGATAATTTCTGTGTA
TCGTGTTCCGCCACGTGCATCCCTTTCAAGTAATTTTGAATTTGAAATGACTGTCTTAATTGATTGACAGTTGCAGCAGTTGCTTCTGATAAGTCG
GTATATAAATTAGAAACGTTATTTACTACACCAGCAGTATTAACACCATAAGCGTTGCCATATCTAGCTAAAGCAGTCGTATTTCCAGGATCTGTT
TGAATTACCGTAAATTTATCGTTTGATGTTGCGTCTCCTGAAATGGTATCCCATTTAATTGGAGCCGTAGTACCTAAAGGTAAGGTGACACTCGCA
CCTTTTGTGGCCATGGTAATGCTGACGTAAAGTAATCATGTCTTTTACCACGACGTTTTAGCACATAGTTTGAGGAAGTATCAGGGCCATCGCCCT
TATCAACTACTGCGCTTGTTTGTAAATTTTCATCTCGGAACCATTCGTTCCAATAAGATTGTATGCACGTGGCCAAAAGGCACAGTGCGTAATAGT
TCGGCCAGTATCAATTTGGCCTACTGTTGGTAAGCCCATATAGTCTTGAAGGCTGCCTACGGCATAACCATCTGTTGGGCTGTTTGAGTTGGGAC

AATATAAGATTGAGTCTGTTGGATTTTCTTGTTGACCCATAAATTTTTGCCAGTTATTCCATATAAGGCGATTGGGTACAAAGAAAAAGAAAGAA
TCCATAATCATGTTGTCCATGATTGGATATAAAGGCGTTGCTAGACGGGCAAATGCCGTCATTTTTAAGTTGAAAGTGTCCCCAGGGAGCACTTC
ATCAACATATACAGGAATTAAATAGCCCGCATCGAAAGTCGTTTTATGTTTTTTGAGCATCGAATTACTACGTGGTATATCGGCTCTAGGTACCAT
CGCGAAGCGGTGAGTATTAACTGACTGATTGCGGTGCATGTTTTCCTTAGTGTTGTTCCGGGGAAAGATAAATCTCTTTTCCCTCGGTTGTTTTAT
TTAAGTTTAACTTGTTTCCTAATGATAGGAGTTTTGGTTGTTCATGTAAATCGAATAACCCAGTTGAATCGTCAAAAGTTCCGAATTCATATAGAT
CGAAGTCATCAGGGTGATTAAAAGTTGATTTTCAGTATCAGAACGATTAATTTCATCTGAAAAAAGAGCGTATAAGGGCGTACTCCAGAGGAAG
GGTACGACATTGGTCGTGCATATGTTCCAGCAGACAGGTCTTTTACGGAAGCAGGGATAAAGTTTCATTTATTTTTCCTAAGTGAGCATTTACGTT
TTAATAGTGAAGTTTTTGGCCATAGTTACTTGTTGCTTTTGCAGATAGTGCCGGTATACGGGTGTATTGTCTGTCAGGACAATTGAAGTTTTAGCG
CTATTAGTCCCGCATGAAAGTAAATCAGTCATACTCATAAGTGGTCAATTTTAAACATTTGTGTCATAGTATTTTGGTGGTTTGACTTTTTACCTCT
AAGTATTACGTAGGTCTTGCGGGTATATATCCGAAGTATATTTTATATAAAAGTCTTTACCGATTCCCGGTTTTAAAGACATTTTATTATATTCCGG
CTTTAAGTCTAAATATTCGCCGGTTTCAGGGTGTATGCGTTGTGTAATGAGATTCCGCATCTTTCCCTGTTGTTGTTTCATTATGTATCTAGCCACG
TAGGCGGCTGATTCGAAATCGTAACATCTCCCAACATGGTGGGTATAACCAAATGCCAGAGAGCTTCAAGTTCTGCGGATCTATAATACCACTAG
AACCCGAGTGCAGTCCTTTCCATAATTTTTTTATCAGGACAAGTCGTATCCGAAGATACAGGGGCATGGAAGTGGGGTCTTGCGAAGAGTTTCCC
CAATATTCTCCAGCCATGTAAATTATGCGGGATGTAAGTCCTCCTTTTTTGCGAGAGTTTTCTAAGTCTTTAGGAAACAGTTGAAAGTCGTTGTGA
TCCAAAAGCCATCGCTTGGAGATGGGTATTGTCAATATGTGAGTGTTATGAATGAGTTGTTTTTATGCAATTGGGCTTCGTGCATGCACCGAATA
GCCCATTGTCTTGATCGTTCTAGACGGCATCCAACGCACTGCCCACAGGGCAGGTCTAAAGATCGAACGATGTCATGTTTCCGAACTTCGTCGAA
AACAATTGATTTGTCAAAGCATTGAAATGCTTTGAGAGGGTGATAACAAGGCATGTGAGGTGCCTGGGGACTTTATTAGAGTCTCCAGCCTCCA
CGCATTGGCGAGTTTCGCATATTTGCGTGTTTTGTATGTGAAGCATGTTTACGGAAAGTCCTAGCGGACTTTTGCTTATTTGCTGGTCTGCGACGC
ATCATTTTTTCTGTCCTTGTTTATCGTGTTTTTGTGGTTTGGTGTCACCTAGCACAGTTACATCAAGTAGGTAACTGTGCTTCCGAGGTTCCAACAG
GGGTTGAAACCTCGGTTTGGGCTGGTTTTACCAGTCCCATTTTTTCAGCTTCGCTGCGATTTTCAGGGTTTTCTAGAAAATCTATTAGATTCGCGG
GATCATTAGCGAATCGTTCACGAATATTGGCTGGTAAAGCCATAAATTCGTTGTCTGAAGCCATAATCTGGTTCAGAGCAGAGTGGTAGTCATAG
ACACCACTAAAGTCGCCATATTGAGGCGTTAAAGGAGTTTGAGGAATAAGTCCGGTCATACCAAACTTTTCCATAATATTATTAATATCGCATTCT
TCAGCAAATTGCTGCTGAGTCAGAGTTGCATCCTCACAATGCAGCCCTGACTCATTTGACGCAGCAATCGTGTCGTAATTGTACGGAGTACGAAG
AAATGGGGCTGTAATCTTGTCATTTTCATTCCATTGGTGGTTGGTTATTTGTACGATTTTGTTGAATCGGTTGACCTCGATAGTTATCGATGTATCG
TTTTGCGCCAGATGCGCTATACGCATCTTTGGCGATATTTTTTACGTCTTGATAAATTGGTTTGCTAGAACTAGGAGCTGACCCTGATTTAGCAAG
TTTTGTTAACTCGTTTGAATAACGAGATTGTGCCAAAGCTTGAGCAGCTTGAGCACTTGATGTTTTGCCTATTTCATTTTTCATGAAAGTATCGGC
AAGTATTTGTTTATATTGAGCTCTAATATTTGGATTTTCATCCAATTTGTTTAACGTATCAGCACGTACATTATCTGTTTGATTACTTGTGAGTTCTG
TTTGAGCTTCAATTTGTTTAGATTGAGCAATAGCTTGATTTGCTTGAGCAACCGTTTGATAAGCTTGGGTTCCGGACGTAGTTGCATTACCGAGAA
CATTTTGCATCTGGGCCATAGCTCCAGCTGGTGTTGTGGCTCCGCCTTGTGAATACGCAAGCATGGGATTTAACCCAGCTTTTTTCATATCTTCAA
CTGCTCGTTGATATGATGTTCCAGACATTTCAGCTTGAAATCTCTGTTGATTACTCGCTTGT

>000043F|arrow

TATGTCAACGTTAATCCTATTGCTCAAACGTCTGCTACTTCGATTTCTGGCGGTGCTACTCCGCTTGGTAACTTGGCTGCAATGGGTACTGCGTTG
GCTAGTGGACATGGTTTTACGTATCATGCTCAAGAACATGGATACATAATTGGTTTGGTAAACGTAAGAGCTGATCTAACATATCAGCAAGGTTT
ACCTAAGATGTGGTCTCGTGAGACACGTTATGATTTCTATTTCCCTGTATTTGCTCATTTAGGTGAACAGGCTGTTCTTAATAAGGAAATCTATGT
TACTGGTACTTCAACTGATGATGATGTATTTGGATACCAGGAACGTTGGGCAGAGTACCGTTACAAACCTTCTCAGATTACAGGTTTGTTTAAGT
CGACTTCAGCTGGTACGATTGACCCTTGGCATTATGCTCAGAAGTTCACTTCGTTGCCGACTTTGAATTCTACGTTTATTCAAGAAACGCCACCTA
TTGATCGTACTACTGCTGTTGGTGCATCTGCTAATGGTCAGCAGTTTTTGATGGATGCGTTTTTTGATTGTAAGATGGCTCGTCCAATGCCGATGT
ATTCTGTACCTGGCTTGATTGACCATTTCTAATGTAATATAAGCTGGACTACTGGGAAACCAGTAGTCAGCAAACAAGCGGAGCGCGTTAGTATG
GGTATGTTTGATTCAGTTGCTGGTGGTTTATTTGATTTTGCTACTTCTGCTTGGAGTCAGAATGAAGAGCAAAAATATACTGCTGAAGCGCAAGC
AAATAGACGCTTTCAAGAAGATATGTCCAATACGCAATATCAGCGTATGGTTACTGATTTGAATAAGGCTGGTTTGAGTCCTATGCTTGCATATTC
TAAAGGTGGTTCTACTGTTCCGAGTGGTGCTACTGCATCTTCTACTTCTCCAGTTAAGTCTGATTTGAGTGGAGCTGTTAAGCGTGATGTTGAGA
GTGATTTATTGCGTGAGCAGATGGAAGTTGCTAAATCTCAACGTGAATTAAATGTTCAGACTGCTAGGAAGGTTGCTGAAGAAGCTGATTTAGC
TTCTCAGCGTGTTTTACAAGAACCGGCTCGTTTTTATTTGGAACAGGCCGAGGCTGGTTCTCGTGTTAATGCTAATTCTGCATCAACAATTAAGAC
TGATATTGATGCTAAGAATAATTTAGAGTTACGAACACCGTCTAGCGATCCATATTGGTATCGCGATATTAAGAAAGGTAGTAAGTCTATTTTGA
TAAGTTTTTGGAAAAGAATAAGTCTTTTCCTTGGTCCTTGGAAGGTCGTAGAAAATGAGTAAAACGAATTTACCTTTTGTACGTAATCCGTACAAT
ATGATATGGCTCTTGTTTCACAAGAGGACTGGTCTTGAATGTAAAGATCCGAGTTTGGTCTCAACAACACATGAGAGACGAATGTGATATTAATA
TTATAGTTGAGCGTTTTGGCGTTACTGGGCAATTGCCCCAGGCGCCATTAGAGCCATCATATGGCGATTTAGCGGTGTATCTGACTATCACACCG
CATTGAATGCTATTCGTGCCTCTGATGAGGCTTTATGGCTTTGCCAGCTAAGATTAGGGCTAAGTTGATAACGATCCTAATGCTTTGTTAAATTAT
TTACAGAATGAAGAGAATCGTGATGAAGCGATTCAGATTGGTCTTATTGACGGTAAACCTGTGGTTGAACCCGTCGTTTCTGCAGTAGAAAACA
CCTAAGCCAGACGCGTAAGCGGATGGCAGCACAGTTACTCTACTTGATGTAACTGTGCTAGGTGACACCAAAAACCACATTATTAACTACGGAG
TGCAATGTTATGAGCCTTTATAGAAACCAATGAGCAAGCATGGCGCAGCTAAGAAGTTTCGTCGTGGCGTAAGCAAGACCAAGAGCATTAATAT

GCGTACTTCACCGCAGCGCGGTGGTTTTAGACTGTAATTTATGGCGTGTTATAAGCCGTTAACGGCTTATCAATGCGCTGACAAGTCTATTATTTG
GCGGGAGATACCAGGGGCGGACGTAGTCCGTACCTTGTCATTGCCTTGTGGTCAGTGTGTTGGTTGTCGCCTTGAACGGTCCCGTCAGTGGGCC
GTTAGATGTATGCATGAGGCACAAATGCATACTAGTAATTGTTTTATTACTTGACATATGCTCCAGAGCATTGTCCTAAGGATATGTCTTTGCATT
ATGAAGATTTTCAATTGTTTATGAAACGATTGAGAAAACGCTATACTGGCAAGACTATTCGTTTTATATGGCAGGTGAATATGGTGAGTCTTTCG
ATCGTCCTCATTTCCATGCTTGTATCTTTGGGCTTGATTTTGAAGATAAGAAGTTTTTCAAAAGAACGCAGACTGGGTCTATCTTATATACGTCAA
AGATACTTGAAGAACTTTGGCCGTATGGCTATAGTTCTATTGGTGATGTCAATTTTGAATCTGCTGCTTATGTTGCTCGATATATTATGAAGAAGA
TTAACGGTAAAACCGTTAATGAGAACCACGAAGTGGTTGATGCAGATGCGCATTATCAGTATTGTGATTTAGATACTGGTGAGATTATTCAGCGT
AAGCCTGAATTTAATAAGATGTCTCTTAAGCCTGGCATTGGTCAGGCTTGGTTTGATAAGTTCATGTCAGACGTTTATACGACCTGACTCTGTTGT
GGTGCGTGGCAAAAAGTGCCGACCACCACGGTTTTATGATAATAAGTTTAAAGTATTGTTTCCAGAAGAATTTGATGGTATACAATATGCTCGTG
AGCTAGAAGGTCGCTCACATTTTGAAGATAACACTTTAGAGCGTTTGGCTGTAAAGGAAAAAGTCGCTTTGGCTAAGTTATCGCTTTTAAAACGT
AAGATTTAAAGGAGTTTTTATGAAGATGGTTATTGTTTCTATTTAGATACTGCAGCTGGTGCGTATGGTCGTCCAGCTTTTGTTGCATCTGAGGGT
GTTGCTGGTTCGTCAGTTTCAGGACGAAGTTAATCGCGCTAGCGATGATAATCAGTTATATAAACACCCTGATGATTTTCAGTTATATTATTTTGG
TACGTTTGATGATAATTCTGGTACTATGGATTTGCTTGGTTCTCCCAAGCTTATTTCTAGAGCTAAGGATGTTATGATTCGCGATGGCGAGTAATG
TTTTTTTTTAAGCCGGATCACTAGCTTGCTAGTGGTTCGGAATACTTCGGGAGATTGTTATGCATCGTAATAAGTCAGTAAGCTCACATAGTTTTG
CTATGGTTCCTAAAGCGGAAATTCCGCGTTCTAGTTTTGATACGCAATATGCGCATAAAACTACTTTTGATGGTGGTTATCTAGTTCCTATTTATTG
TGATGAAGTGCTCCCAGGGGACATGCACAATGTAAAGGCTACAATGTTTGCCCGTTTGGCAACGCCTTTGTTTCCAGTTATGGACAATTTGCATT
TAGATACTTTCTTTTTCTTTGTTCCAAATAGATTAGTTTGGAACAATTGGGTTAAGTTTATGGGTGAGCAAGCGAACCCAGGTGATTCTATTTCGT
ATGTAGTTCCACAGATTACTTCTACTGCTGGTGGTTATGCAGTAGGTTCTATTTTGATCATTTTGGTTTGCCTACGGCTGGTCAGATTACTGGTACT
AATACGGTTACGCATAACGCGTTACCGTTACGTGCTTATAATTTGATTTATAACGAGTGGTTTAGGGATGAGAATTTACAAAATTCTGTTACCGTT
CACAAGGGTGACGGTCCTGATACTCCGAGTGATTATGCTATGGTTCGACGCGGTAAGCGTAAGGATTATTTTACTGGTGCCTTGCCTTGGCCTCA
GAAAGGCGATGCAGTTACGTTGCCTTTAGGCACGTCTGCTCCTATTAAGACGTCTGTTACTAATGATTCAACAACGTTGTCATTAGTTGACGGTG
CTGGTGCTTTGAAGCAGATTTATGCTAATTCAACTACACATGCTGTGTATCGTAGTAATAACGCTGGTACTGCTGGTACTGGTTTGTATGCCGATT
TAAGTGATGCTACGGCTGCAACTATTAACCAGTTACGTCAGTCTTTCCAGATTCAGCGTTTGTTAGAGCGCGATGCGCGAGGTGGTACACGCTAT
ACAGAATTGTTACGTGCTCATTTTGGTGTAACTCCACAAGATTATCGTTTACAACGTCCTGAATATATTGGTGGTGGTTCTACT

>000092F|arrow

GCATCAACCACTTCGTGGTTTTCATTGACAGTAACCCCATTAATCTTCTTCATAATATATCTTGCAACATAAGCAGCAGACTCAAAAGTAACATCAC
CAATTGTAGAATAGCCAAACGGCCACAATTCTTCCAAAATCTCTGACGTGTAGAGGATAGAGCCAGTCTGCGTTCTTTTAAATATTTTCTTATCCG
GAAAATCAAGACCAAACAGACAAGCATGGAAATGAGGACGATCAAAAGATTCACCATATTCACCTGCCATATAAAAACGTATCGTTTTCCCAGT
AAAACGCTTACGTAACCGCTTCATAAAAAGCTGATAATCATTGTAATCCAATGACATATCCTTAGGACAATGCTCTGGAGCATATGTCAAAGTAA
TAAAACAATTACTAGTATGCATTTGTGCCTCATGCATACAACGAATCGCCCACTGACGTGAGCGTTCAAGGCGACAACCAACACACTGACCACAA
GGCAATGATAGGGTACGGACTACATCCGCACCCGGTATTTCCCGCCAAATTATAGACCTGTCACTGCATTGATAAGCCGTTAAGGGCTTATAACA
GGCCATAATTACAAACGATAGCCACCACGCTGGGGAGCGTGTCTCATATTAATTGACTTCGTCTTACTAGCAGTTCTGCGAAATGACTTTGCAGA
TTTATATTTGTTTACTGGCTTTCTTCGTAACATGATGAACTCCGTAGTTAAAATAGTGGTTTGGTGTCACCTAGCACAGTTACATCAAGTAGAGTA
ACTGTGCTGGCCTCAGGATTTCATCCTTCGGCCTTAGGTGTTTCTGTAGAAACGATGGGTTCAACCACAGGTTGTCCATCAATAAGACCCAATTG
AATCGCTTCATCACGATTCTGGTCGTTCTCAAGGAACTCCAATAATTTGACAGGATCATGGTCAAATCGGACTCTTAATTTCGCTGGCAGAGCCAT
GAAATCGTCCATAGTTGCGTTAATTTGATTCAACGCAGAATGGTAATCAGTAACACCACTAAAATCGCCGTATTGAGGCGATACAGGGGCCGTT
GGAAGTTCCCCTGTAACCCCGAAACGTTCAATGATGACATTAATGTCACATTCATCTTTCATATGCTGTTGAGCAAGACTTGGGTCTTGACACAGC
AACGCATCGTTTACCGATGCTTCATCTTTATCATAGTTGTACGGATTACGTACAAATGGCAAATTCGCTTTACTCATTATTTGACTCCAATTCCCCA
AGGGGTTAGTTGATTAGGATTATTTTTGAGTTTATCAAAAATTTCTTTCGAAGCATCGATACCCCTAGACCAAATAGCTGGGGATGGAGCCAATA
GCCTCTTAGTATTATATGACTGAGCGGATGTTAAATCCGCAGTCGTATTCTTTAAAGCAATATCAGCCAAAATACGTTTATTTTCGGCTGTAATATT
AGGAGCAGTAAGCAACTTATTAACAGTATCAGCCCTAGTATTAGCGGTACCAGCTTCAGTAGCTTCAGTCTGGGCGATAATCTGCTTTTCTGTTTC
TGATGCATTACGAATCTGTTGCATCATAGAAGCAGTATTAATAGCTGAATTAGTTGCATTACCTAAAACATTTTCCATAGTAGCAGTTGAACCAGA
TGGGGTAGAAGCACCACCACGTTGATAGGCTAACATAGGAGATAAACCAGCAGCTTCTAAATCTTTAACAGCACGCTGGTAACTTGTTCCACTCA
TATCGGCTTGAAAATCCATTTGCCTCTGAGCCATCTCCTGATTAGCTTTATTAGCAGAAGTAGAACCTAAATAAGAACCAACAGCGCCCAAAGCA
GTACCGACACCAGGAGTAAAGAAACTAGAAGCTGAAGACAGCTTAGAACCAACATTAGTAACCGCATCAAGTATTCCACCAAACATAAACTAAC
GCCCTTCGGTTGTTTCCTCACTACTCCTTACGGAGTAGTCGAGGTTATATAAAACATTAGAAATGATCAATTAGACCAGGTACGCTGTACATAGG
CATAGGTCTGGCCATCTGACAATCAAAGAAAGCATCCATTAAAAATTGCTGACCATTTGCTGCAGCTCCGACCGCCGTAGTACGAGCAACTGGA
GGGGTCTCTTGAATAAACGTTGAATTCAACGTAGGAAGAGAAGTAAATCGCTGTCCATAATGCCAAGCATCAATAGTGCCAGCAGAAGTAGACC
TAAAGAAACCAGTAATTTGAGAAGGTTTGTAACGATACTCCGCCCAACGTTCTTGATAACCAAATACATCATTGTCGGCTGCAGTACCTTGAACA
TAAATTTCTTTGTTCAAAATAGCTTGCTCACCCAAAGTGGCAAATACTGGGAAATAAAAGTCATATCGTGTAGACCTAGACCACATCTTAGGAAG
ACCCTGTTGATATGTGAGGTCAGCACGTACAGAAACTAATCCGATAATGTATCCATGTTCTTGAGCAGCATACGTAAAGCCGTGTCCCTGAGCCA

ATGCAGTACCCATTGCAGCAAGGTTACCTTGCGGAGTAGCAGAACCAGTAACCGACGTTGCAGAAGTCTGAGCAATCGGATTAACATTAACAAG
GGTCGAACCTCCACCAATATATTCAGGACGTTGTAAACGATAATCCTGTGGAGTTACTCCAAAGTGAGCACGTAGTAACTCAGTATAACGAGTAC
CACCACGTGCATCGCGCTCCAATAACTTCTGAATCTGGAAAGATTGACGAAGTTGGTTAATAGTCGCAGCAGTAGCAGTAGACAAATCAGCGTA
TAACTGATTAGTAGCAACACCAGCATTTGTACTATTAGACAAAGTATTAGAAGCTGAATTTAATTCTCTTAAAGCACCAACAGCAGTTTGAAAAAC
ACTATAACCAGCAGTACCATTGCCAGCTGGTATGTCAGAATAAATATTAGCACGTGTTCCTAACGGTAAAGAAACAGAAGCACCCTTCTGTGGCC
AAGGCAATGCACCAGTAAAATAATCTTTACGCTTACCACGTCTCAAAAGTGTATAGTTAGCAGGAACATCACCTGAATCACCAAGATTTAACGTT
ACAGAATTCTGTAAGTTTTCATCTCTAAACCATTCGTTATAAATCTCATTATAAGCACGTAACGGCAAAACATTATGCGTTACTGTATTAGCGCCA
GTAATCTGACCAGCAGTAGGTAAACCAAAATAATCAAAAATTGAACAAACAGCATAACCACCAGCAGGACTAGTAATAGTAGGCACTACAAAAG
AAATAGAATCACCAGGATTCGTTTGTTCACCCATAAACTTTGGCCAATTGTTCCAAAGTAAACGATTAGGTACAAAGAAAAAGAAAGTATCAAGA
TGCAAGTTGTCCATCACAGGAAATAACGGTGTGGCCAAACGTGCAAATGCAGTCATCTTTACACGATGAGTATCGCCAGGCAATACTTCATCACA
ATAAATAGGAACTAAAAAACCAGCATCAAACGTGGTTTTATGAGCATATTGAGTATTAAAACTAGAGCGGGGAATGTCCGCTTTAGGAACCATA
GCAAATGAATGCGTACTTACTGACTTATTGCGAAACATAAACATCTCCCGTAGTTCCGTACCACTCTTTCGAGTGATACGGTATAAAAAAAACCTT
ACTCGCCTTCGCGAATCATTACATCTTTTGCACGAGCAATCAACTTAGGGCTTTCCAGTAGTTCAAAAACACCAGTGGCATCGTCAAAAAGACCC
AAATAGAACATATGAAAATCATCAGGATGTTTATACAACTGATTATCTTCGCTAGCTCGATTGACTTCATCCTGAAACTGACGTACTGCAACACCT
TCAGATGCAACATAAGCTGGACGACCAAAAGCATCTGCAGCAGTATCCTTAATAGAAACAATAACCATCTTCATAAAAACTCCTTAAATAGTACG
TTTTAACAATGACAACTTAGCCAACGCAACTTTTTCCTTAACAGCAAGTCGCTCAAGCGTGTTGTCCTCATGCCTAGATCGACCTTCCATCTCTCTG
GCAAACTGAATCATATCGAATTCTTCAGGAAACTTCAACTTAAATTTATTATCATAAAACCGTGGTGGACGGCACTTTTTGCCACGCACCACAACG
TGGTCTGACGTATAAACGTCTGACATGTACTTATCTAACCACGATTGCCCGATACCGGGCTTCAATGACATCTTATTAAATTCTGGCTTACGCTGA
ATTATCTCACCAGTCTCTAAATCACAATATTGATAATGGGCACCC

>000206F|arrow

GCACAGTTACATCAAGTAGAGTAACTGTGCTGGCCTCAGGATTTCATCCTTCGGCCTTAGGTGTTTCTGTAGAAACGATGGGTTCAACCACAGGT
TGTCCATCAATAAGACCCAATTGAATCGCTTCATCACGATTCTGGTCGTTCTCAAGGAACTCCAATAATTTGACAGGATCATGGTCAAATCGGACT
CTTAATTTCGCTGGCAGAGCCATGAAATCGTCCATAGTTGCGTTAATTTGATTCAACGCAGAATGGTAATCAGTAACACCACTAAAATCGCCGTA
TTGAGGCGATACAGGGGCCGTTGGAAGTTCCCCTGTAACCCCGAAACGTTCAATGATGACATTAATGTCACATTCATCTTTCATATGCTGTTGAG
CAAGACTTGGGTCTTGACACAGCAACGCATCGTTTACCGATGCTTCATCTTTATCATAGTTGTACGGATTACGTACAAATGGCAAATTCGCTTTAC
TCATTATTTGACTCCAATTCCCCAAGGGGTTAGTTGATTAGGATTATTTTTGAGTTTATCAAAAATTTCTTTCGAAGCATCGATACCCCTAGACCAA
ATAGCTGGGGATGGAGCCAATAGCCTCTTAGTATTATATGACTGAGCGGATGTTAAATCCGCAGTCGTATTCTTTAAAGCAATATCAGCCAAAAT
ACGTTTATTTTCGGCTGTAATATTAGGAGCAGTAAGCAACTTATTAACAGTATCAGCCCTAGTATTAGCGGTACCAGCTTCAGTAGCTTCAGTCTG
GGCGATAATCTGCTTTTCTGTTTCTGATGCATTACGAATCTGTTGCATCATAGAAGCAGTATTAATAGCTGAATTAGTTGCATTACCTAAAACATTT
TCCATAGTAGCAGTTGAACCAGATGGGGTAGAAGCACCACCACGTTGATAGGCTAACATAGGAGATAAACCAGCAGCTTCTAAATCTTTAACAG
CACGCTGGTAACTTGTTCCACTCATATCGGCTTGAAAATCCATTTGCCTCTGAGCCATCTCCTGATTAGCTTTATTAGCAGAAGTAGAACCTAAAT
AAGAACCAACAGCGCCCAAAGCAGTACCGACACCAGGAGTAAAGAAACTAGAAGCTGAAGACAGCTTAGAACCAACATTAGTAACCGCATCAA
GTATTCCACCAAACATAAACTAACGCCCTTCGGTTGTTTCCTCACTACTCCTTACGGAGTAGTCGAGGTTATATAAAACATTAGAAATGATCAATT
AGACCAGGTACGCTGTACATAGGCATAGGTCTGGCCATCTGACAATCAAAGAAAGCATCCATTAAAAATTGCTGACCATTTGCTGCAGCTCCGAC
CGCCGTAGTACGAGCAACTGGAGGGGTCTCTTGAATAAACGTTGAATTCAACGTAGGAAGAGAAGTAAATCGCTGTCCATAATGCCAAGCATCA
ATAGTGCCAGCAGAAGTAGACCTAAAGAAACCAGTAATTTGAGAAGGTTTGTAACGATACTCCGCCCAACGTTCTTGATAACCAAATACATCATT
GTCGGCTGCAGTACCTTGAACATAAATTTCTTTGTTCAAAATAGCTTGCTCACCCAAAGTGGCAAATACTGGGAAATAAAAGTCATATCGTGTAG
ACCTAGACCACATCTTAGGAAGACCCTGTTGATATGTGAGGTCAGCACGTACAGAAACTAATCCGATAATGTATCCATGTTCTTGAGCAGCATAC
GTAAAGCCGTGTCCCTGAGCCAATGCAGTACCCATTGCAGCAAGGTTACCTTGCGGAGTAGCAGAACCAGTAACCGACGTTGCAGAAGTCTGAG
CAATCGGATTAACATTAACAAGGGTCGAACCTCCACCAATATATTCAGGACGTTGTAAACGATAATCCTGTGGAGTTACTCCAAAGTGAGCACGT
AGTAACTCAGTATAACGAGTACCACCACGTGCATCGCGCTCCAATAACTTCTGAATCTGGAAAGATTGACGAAGTTGGTTAATAGTCGCAGCAGT
AGCAGTAGACAAATCAGCGTATAACTGATTAGTAGCAACACCAGCATTTGTACTATTAGACAAAGTATTAGAAGCTGAATTTAATTCTCTTAAAG
CACCAACAGCAGTTTGAAAAACACTATAACCAGCAGTACCATTGCCAGCTGGTATGTCAGAATAAATATTAGCACGTGTTCCTAACGGTAAAGAA
ACAGAAGCACCCTTCTGTGGCCAAGGCAATGCACCAGTAAAATAATCTTTACGCTTACCACGTCTCAAAAGTGTATAGTTAGCAGGAACATCACC
TGAATCACCAAGATTTAACGTTACAGAATTCTGTAAGTTTTCATCTCTAAACCATTCGTTATAAATCTCATTATAAGCACGTAACGGCAAAACATTA
TGCGTTACTGTATTAGCGCCAGTAATCTGACCAGCAGTAGGTAAACCAAAATAATCAAAAATTGAACAAACAGCATAACCACCAGCAGGACTAG
TAATAGTAGGCACTACAAAAGAAATAGAATCACCAGGATTCGTTTGTTCACCCATAAACTTTGGCCAATTGTTCCAAAGTAAACGATTAGGTACA
AAGAAAAAGAAAGTATCAAGATGCAAGTTGTCCATCACAGGAAATAACGGTGTGGCCAAACGTGCAAATGCAGTCATCTTTACACGATGAGTAT
CGCCAGGCAATACTTCATCACAATAAATAGGAACTAAAAAACCAGCATCAAACGTGGTTTTATGAGCATATTGAGTATTAAAACTAGAGCGGGG
AATGTCCGCTTTAGGAACCATAGCAAATGAATGCGTACTTACTGACTTATTGCGAAACATAAACATCTCCCGTAGTTCCGTACCACTCTTTCGAGT
GATACGGTATAAAAAAAACCTTACTCGCCTTCGCGAATCATTACATCTTTTGCACGAGCAATCAACTTAGGGCTTTCCAGTAGTTCAAAAACACCA

GTGGCATCGTCAAAAAGACCCAAATAGAACATATGAAAATCATCAGGATGTTTATACAACTGATTATCTTCGCTAGCTCGATTGACTTCATCCTGA
AACTGACGTACTGCAACACCTTCAGATGCAACATAAGCTGGACGACCAAAAGCATCTGCAGCAGTATCCTTAATAGAAACAATAACCATCTTCAT
AAAAACTCCTTAAATAGTACGTTTTAACAATGACAACTTAGCCAACGCAACTTTTTCCTTAACAGCAAGTCGCTCAAGCGTGTTGTCCTCATGCCT
AGATCGACCTTCCATCTCTCTGGCAAACTGAATCATATCGAATTCTTCAGGAAACTTCAACTTAAATTTATTATCATAAAACCGTGGTGGACGGCA
CTTTTTGCCACGCACCACAACGTGGTCTGACGTATAAACGTCTGACATGTACTTATCTAACCACGATTGCCCGATACCGGGCTTCAATGACATCTT
ATTAAATTCTGGCTTACGCTGAATTATCTCACCAGTCTCTAAATCACAATATTGATAATGGGCACCCGCATCAACCACTTCGTGGTTTTCATTGACA
GTAACCCCATTAATCTTCTTCATAATATATCTTGCAACATAAGCAGCAGACTCAAAAGTAACATCACCAATTGTAGAATAGCCAAACGGCCACAAT
TCTTCCAAAATCTCTGACGTGTAGAGGATAGAGCCAGTCTGCGTTCTTTTAAATATTTTCTTATCCGGAAAATCAAGACCAAACAGACAAGCATG
GAAATGAGGACGATCAAAAGATTCACCATATTCACCTGCCATATAAAAACGTATCGTTTTCCCAGTAAAACGCTTACGTAACCGCTTCATAAAAA
GCTGATAATCATTGTAATCCAATGACATATCCTTAGGACAATGCTCTGGAGCATATGTCAAAGTAATAAAACAATTACTAGTATGCATTTGTGCCT
CATGCATACAACGAATCGCCCACTGACGTGAGCGTTCAAGGCGACAACCAACACACTGACCACAAGGCAATGATAGGGTACGGACTACATCCGC
ACCCGGTATTTCCCGCCAAATTATAGACCTGTCACTGCATTGATAAGCCGTTAAGGGCTTATAACAGGCCATAATTACAAACGATAGCCACCACG
CTGGGGAGCGTGTCTCATATTAATTGACTTCGTCTTACTAGCAGTTCTGCGAAATGACTTTGCAGATTTATATTTGTTTACTGGCTTTCTTCGTAAC
ATGATGAACTCCGTAGTTAAAATAGTGGTTTGGTGTCACCTA

>000042F|arrow

CCGTATCACTCGAAAGAGTGGTACGGAACTACGGGAGATGTTTATGTTTCGCAATAAGTCAGTAAGTACGCATTCATTTGCTATGGTTCCTAAAG
CGGACATTCCCCGCTCTAGTTTTAATACTCAATATGCTCATAAAACCACGTTTGATGCTGGTTTTTAGTTCCTATTTATTGTGATGAAGTATTGCC
TGGCGATACTCATCGTGTAAAGATGACTGCATTTGCACGTTTGGCCACACCGTTATTTCCTGTGATGGACAACTTGCATCTTGATACTTTCTTTTTC
TTTGTACCTAATCGTTTACTTTGGAACAATTGGCCAAAGTTTATGGGTGAACAAACGAATCCTGGTGATTCTATTTCTTTTGTAGTGCCTACTATTA
CTAGTCCTGCTGGTGGTTATGCTGTTTGTTCAATTTTTGATTATTTTGGTTTACCTACTGCTGGTCAGATTACTGGCGCTAATACAGTAACGCATAA
TGTTTTGCCGTTACGTGCTTATAATGAGATTTATAACGAATGGTTTAGAGATGAAAACTTACAGAATTCTGTAACGTTAAATCTTGGTGATTCAGG
TGATGTTCCTGCTAACTATACACTTTTGAGACGTGGTAAGCGTAAAGATTATTTTACTGGTGCATTGCCTTGGCCACAGAAGGGTGCTTCTGTTTC
TTTACCGTTAGGAACACGTGCTAATATTTATTCTGACATACCAGCTGGCAATGGTACTGCTGGTTATAGTGTTTTTCAAACTGCTGTTGGTGCTTT
AAGAGAATTAAATTCAGCTTCTAATACTTTGTCTAATAGTACAAATGCTGGTGTTGCTACTAATCAGTTATACGCTGATTTGTCTACTGCTACTGCT
GCGACTATTAACCAACTTCGTCAATCTTTCCAGATTCAGAAGTTATTGGAGCGCGATGCACGTGGTGGTACTCGTTATACTGAGTTACTACGTGCT
CACTTTGGAGTAACTCCACAGGATTATCGTTTACAACGTCCTGAATATATTGGTGGAGGTTCGACCCTTGTTAATGTTAATCCGATTGCTCAGACT
TCTGCAACGTCGGTTACTGGTTCTGCTACTCCGCAAGGTAACCTTGCTGCAATGGGTACTGCATTGGCTCAGGGACACGGCTTTACGTATGCTGC
TCAAGAACATGGATACATTATCGGATTAGTTTCTGTACGTGCTGACCTCACATATCAACAGGGTCTTCCTAAGATGTGGTCTAGGTCTACACGATA
TGACTTTTATTTCCCAGTATTTGCCACTTTGGGTGAGCAAGCTATTTTGAACAAAGAAATTTATGTTCAAGGTACTGCAGCCGACAATGATGTATT
TGGTTATCAAGAACGTTGGGCGGAGTATCGTTACAAACCTTCTCAAATTACTGGTTTCTTTAGGTCTACTTCTGCTGGCACTATTGATGCTTGGCA
TTATGGACAGCGATTTACTTCTCTTCCTACGTTGAATTCAACGTTTATTCAAGAGACCCCTCCAGTTGCTCGTACTACGGCGGTCGGAGCTGCAGC
AAATGGTCAGCAATTTTTAATGGATGCTTTCTTTGATTGTCAGATGGCCAGACCTATGCCTATGTACAGCGTACCTGGTCTAATTGATCATTTCTA
ATGTTTTATATAACCTCGACTACTCCGTAAGGAGTAGTGAGGAAACAACCGAAGGGCGTTAGTTTATGTTTGGTGGAATACTTGATGCGGTTACT
AATGTTGGTTCTAAGCTGTCTTCAGCTTCTAGTTTCTTTACTCCTGGTGTCGGTACTGCTTTGGGCGCTGTTGGTTCTTATTTAGGTTCTACTTCTGC
TAATAAAGCTAATCAGGAGATGGCTCAGAGGCAATGGATTTTCAAGCCGATATGAGTGGAACAAGTTACCAGCGTGCTGTTAAAGATTTAGAAG
CTGCTGGTTTATCTCCTATGTTAGCCTATCAACGTGGTGGTGCTTCTACCCCATCTGGTTCAACTGCTACTATGGAAAATGTTTTAGGTAATGCAA
CTAATTCAGCTATTAATACTGCTTCTATGATGCAACAGATTCGTAATGCATCAGAAACAGAAAAGCAGATTATCGCCCAGACTGAAGCTACTGAA
GCTGGTACCGCTAATACTAGGGCTGATACTGTTAATAAGTTGCTTACTGCTCCTAATATTACAGCCGAAAATAAACGTATTTTGGCTGATATTGCT
TTAAAGAATACGACTGCGGATTTAACATCCGCTCAGTCATATAATACTAAGAGGCTATTGGCTCCATCCCCAGCTATTTGGTCTAGGGGTATCGA
TGCTTCGAAAGAAATTTTTGATAAACTCAAAAATAATCCTAATCAACTAACCCCTTGGGGAATTGGAGTCAAATAATGAGTAAAGCGAATTTGCC
ATTTGTACGTAATCCGTACAACTATGATAAAGATGAAGCATCGGTAAACGATGCGTTGCTGTGTCAAGACCCAAGTCTTGCTCAACAGCATATGA
AAGATGAATGTGACATTAATGTCATCATTGAACGTTTCGGGGTTACAGGGGAACTTCCAACGGCCCCTGTATCGCCTCAATACGGCGATTTTAGT
GGTGTTACTGATTACCATTCTGCGTTGAATCAAATTAACGCAACTATGGACGATTTCATGGCTCTGCCAGCGAAATTAAGAGTCCGATTTGACCAT
GATCCTGTCAAATTATTGGAGTTCCTTGAGAACGACCAGAATCGTGATGAAGCGATTCAATTGGGTCTTATTGATGGACAACCTGTGGTTGAACC
CATCGTTTCTACAGAAACACCTAAGGCCGAAGGATGAAATCCTGAGGCCAGCACAGTTACTCTACTTGATGTAACTGTGCTAGGTGACACCAAAC
CACTATTTTAACTACGGAGTTCATCATGTTACGAAGAAAGCCAGTAAACAAATATAAATCTGCAAAGTCATTTCGCAGAACTGCTAGTAAGACGA
AGTCAATTAATATGAGACACGCTCCCCAGCGTGGTGGCTATCGTTTGTAATTATGGCCTGTTATAAGCCCTTAACGGCTTATCAATGCAGTGACA
GGTCTATAATTTGGCGGGAAATACCGGGTGCGGATGTAGTCCGTACCCTATCATTGCCTTGTGGTCAGTGTGTTGGTTGTCGCCTTGAACGCTCA
CGTCAGTGGGCGATTCGTTGTATGCATGAGGCACAAATGCATACTAGTAATTGTTTTATTACTTTGACATATGCTCCAGAGCATTGTCCTAAGGAT
ATGTCATTGGATTACAATGATTATCAGCTTTTTATGAAGCGGTTACGTAAGCGTTTTACTGGGAAAACGATACGTTTTTATATGGCAGGTGAATAT
GGTGAATCTTTTGATCGTCCTCATTTCCATGCTTGTCTGTTTGGTCTTGATTTTCCGGATAAGAAAATATTTAAAAGAACGCAGACTGGCTCTATCC

TCTACACGTCAGAGATTTTGGAAGAATTGTGGCCGTTTGGCTATTCTACAATTGGTGATGTTACTTTTGAGTCTGCTGCTTATGTTGCAAGATATA
TTATGAAGAAGATTAATGGGGTTACTGTCAATGAAAACCACGAAGTGGTTGATGCGGGTGCCCATTATCAATATTGTGATTTAGAGACTGGTGA
GATAATTCAGCGTAAGCCAGAATTTAATAAGATGTCATTGAAGCCCGGTATCGGGCAATCGTGGTTAGATAAGTACATGTCAGACGTTTATACGT
CAGACCACGTTGTGGTGCGTGGCAAAAAGTGCCGTCCACCACGGTTTTATGATAATAAATTTAAGTTGAAGTTTCCTGAAGAATTCGATATGATT
CAGTTTGCCAGAGAGATGGAAGGTCGATCTAGGCATGAGGACAACACGCTTGAGCGACTTGCTGTTAAGGAAAAAGTTGCGTTGGCTAAGTTG
TCATTGTTAAAACGTACTATTTAAGGAGTTTTTATGAAGATGGTTATTGTTTCTATTAAGGATACTGCTGCAGATGCTTTTGGTCGTCCAGCTTATG
TTGCATCTGAAGGTGTTGCAGTACGTCAGTTTCAGGATGAAGTCAATCGAGCTAGCGAAGATAATCAGTTGTATAAACATCCTGATGATTTTCAT
ATGTTCTATTTGGGTCTTTTTGACGATGCCACTGGTGTTTTTGAACTACTGGAAAGCCCTAAGTTGATTGCTCGTGCAAAAGATGTAATGATTCGC
GAAGGCGAGTAAGGTTTTTTTATA

>000148F|arrow

GCCGTATTCTCCAGCCATGTAGTAACGTAATTTTGCAGGTGCAATGGATTTTCTGAAGCGCTTAAGGAACTTTTGGAAGTCGCTTTTGACAAGCG
ATCCAGTTTGTGGAAGGTTTTCATTGTTGTATGTGAGGGTTTAAAGCAGTTTTTTTCGTGCAATTGGGCTTCATGCATGCAGCGCATGGCCCATTG
ACGTGATTTTTCTAGCCTGCAGCCAACGCATTGGCCGCAGGGCAGTGAAATCTGACGATCATGCTCGTCAGATTCTTTGAATGCGACACGGCGAT
AAGATTTGCCGGTCTTATAGTTTGTCTGATGCTTACTTAAATAAGCAGTCAGTGGGTGATAACAGGCCATGTGAGGTAGCCTGGCGCTTTATTAG
CCGCCAGCCCCCTCTTTGCGGGCTTTTTGCATATTTGCAGCTTTAGTTTTTGAAGTGTTCCTACGGAACGATTTAGCGCTTTTGCGCTTGTTTGTTT
GTTTTCTATAAAGCATAATTTTTGGCCTCGTTTATCAGATATTTTTGGTGTTGGTGTCACCTAGCACAGTTACATCAAGTGGAGTAACTGTGCTTAT
CGCCATTTCTTCCGAAATGGCTCTTTCGACCAGGCCGAGTTTCTCGGCTTCTGGTCGATTTTTCTCGTCTTGCAAGAACTCAATCAGGTTTGCTGGT
TCGTTTTCGAACCTAGCACGAATTTGAGCCGGTAAGTTATCAAATTCTTCCATAGCGTTCATTACCTTATTCAAGGCGCTATGATAGTCAGTAATTC
CGCTGAAATCGCCATATTGAGGCGGCAGCGGACTTTGAGGTAATAGGCCTGTAACGTTAAAACGTTCCAGGATAGTATTTATATCGCATTCGTCT
TTATAATGCTGCTGAGCAGAGTTGGCTCCTCACAAGCCAACCCTGACTCATTTGACGCAGCATCCGTGTCATAGTTGTATGCGGTTCTAAGTTTGA
TTGTTTTCATATCATTTTCCAAATGGTAGATATTTTTGATAGTTATCTAATTGGCTATGTAAATAGCCTTAACATCTTGATAAACGTGGTTTAGTACT
GGATGGCGCTGAGCCAGTTTGCGCTAATCGCTCTAATTGGTCTGTATATGCACTTTGACTAACGCTTTCGTCGCTGTTGTGCAGCTTGTAATGCAC
TTGATGTCCTTAATTGCTCTATTTGGGCATCGCGAAGCTGACCAAAGTTGGCCGTATCCCGGCATCTGAGCAATTTCACGAGCTGTATTTGCTCGA
GTATATGTTGCCTGATCACGTGATTAGATTTGTATCCGCGTCCGTTTTTTCGGCTTGCGATTTGTGTCAGGATATTCATGAGTTTGTAATTGTTTAA
AATTGGCGACTGCCATGGCGCTTCACGGGCTGAATTCCACGCTCACTATGTGGATTCCACTGGAGCTTGCGCACCAGGCTGGACTTTGGCTCCGC
CTTGTGAATAAGCCAGCATAGGGTTAAGACCTGCAGCCTTAAGATCCGCTACTGCGTCTTGATATGCCAGTACCGTCGCCATACCATCTTGGAAT
TGCATCATTGCTGCAGCTTGTTGTGCGCTGGCAGCGTTTTGTTCTTGACCACCAAAATAACTAGCGGCCGCACCTATTCCGGCTCCAATTAAGGA
GCCGTAAGGTCCAAAAGCGCGCGCAAGATGCGGCGCTGAGGCAGCGGCTTCTAGTGCCATTAGAAGTGGTGCGATTAAGCCAGGTACAGAGT
ACATTGGCATTGGCCGTGCTTTTTTATATCAAAAAGCTATCAAAAATGATTTGCTGGCCGTTGGCAGCTGCTCCGACCGCAAGGGTTCGAGAGAC
AGGGTGGATTTTCTTGAATAAACGTGTTATTCAAAGTTGGAAGTGTTGTAAACCGTTGGGCCTAAATGCCAGCCGTCAATTGGTTCCAGCTGCCG
TACTACGGAACAGACTGGAAAATTCGGCTTGGCAATATAACGATATTCTGCCCAACGTTCTTGGTATCCAAACACTCCTGTATCGTGGCATCGCCT
CGTACGTAGATTTCTTCATTTAATAACTGCTGTTCGCCCAAAGTGGCGAAAGCAGGGAAATAGAAATCATAACGTGTGGAACGATTCCACATGC
GGTTGCATACCTTGTTGGTATGTTAAATCGGCTCGTACTGAAACGAGACCGATAATTACACCATGTTCGTGCTTGAATAAGTAAAGCCATGATTG
TGAGCGAGCCGTAACCCATAGCGCCAAGTGTACCGAGAGGAGTAGTGGTCCCCGGAACTCCGGTACCATTCGTCTGGGCGATTGGATTGATAG
TAATGTTTGTTTGAAACCGCCTCCAATGTATTCAGGCCGTTGGAGACGCGCATCCGGGGCTAACGACCCCGAAATGGCTGCGGATAATTTTTCTG
TGTATCGAGTTCCGCCACGGGCGTCCCTTTCTAAAAGTTTTTGAATCTGGAAGCTTTGACGCAATTGATTAATTGTTGCAGCGGTTGCAGCTGATA
AATCTGCATATAAACCGCCATTAGGATCATATGATTTTGCCAAGCCGTCAGCACCGCCAGTAATTTGACCAGTAACACCTACGTTAATAGCCGGG
GCGGTTGCGTTTAATGTATTTGTTCCAGCGTTATACAGTCTGGAAACAGGCGCATTGTTAGTGCGTAATATTGGGGCGGATGTTCCAAGCGGTA
AAGTAACGCTTGCGCCCTTTTGTGGCCAAGGTAATGCTGACGTGAAATAGTCCTTTACGTTTTCCACGTCGTAATAATGTGTAGTTGGCTACGTTA
TCTGGACCATCGCCAGTATCTACAACTACTGAATTTTGAAGGTTTTCGTCCCGAAACCATTCGTTATAAATAAGGTTGTAAGCACGTGGCCAGAA
GGCACAGTGACTTACGGTGCCACCAGCACCTACTTGGCCTACGGTTGGCAGACCCATATAATCTTGAAGGCTGCCAATAGCGTAACCACCAGCT
GGGGTTACTTGTTGGGGCACTACGTACGAGATCGAGTCCGCAGGATTGCCTGCTGCCCCATAAATTTTTGCCAATTTGACCAGATAAGTCTATTT
GGGCACAAAAAAGAAGAAGCTATCCAGATGCATGTTATCCATGATCGGATAGAGTGGTGTAGACAATCGCGCAAAGGCGGTCATGTGCAGCGG
AATGTATCGCCTGGCAACATTTCGTCTACGAGTACAGGGACTAGGAAGCCAGCATCGAATGTAGTTTTATGTGTTGATTGACAGTCAAATGTAGA
GCGGGGGATATCCGCTTTTGGAATCATTGTGAACTGATGGACGTCTACCGACTTGTTACGATGCATTTTTTTGAGCTCCTAGGCCTAGTTGCGTG
AGAAAAAGGGGTTTCCCCCTTTTACTCTACGCTTAGTTTTTATCAGTAATTTTGACTTGTTTCCCTAAGGATACAAGTTTGGGTTGTTCATGTAATT
GGAACAAACCCGTATTATCGTCGAATTCGCCTAATTCAAATAGGTCGAAATCGTCGGAATGGTTATAAAGTTGATTGTCATCGCTCTGGCGATTG
ACTTCGTCGCTAAAGCTCCGGATTGCTTCGCCGATAGAACGGACGAACATTGGACGACCGAACGCATCTGCTGCGCGGTCTTTAACGGTACAGA
GTACTAATTTCATGAGGATTTCCTAAGTGAGGTTACGTTTTAATAATTGAAGTTTGGCCTTTGTGACTTTTTCCTTTACGGCAAGTCTGGCATAGG
TATTGTCTTCGTGGTTGAGTTTAGCAGAAGTTTCACGTTTGTGGAGTAATTCTTCGTATTCATATGGATAATCTGATTTATATTTTTTGTCATAGTA
TTTTGGGGGTTTTACCCTTTTTTCCGGTTGACTACAACATAGTCATGGGGATATACGTCGGAACGATATTTTTTGTACCACTCAGCACCAATGCCT

GGTTTAAGGCTCATATTGTTATATTCTGGTAATAGCTTTATTAATTCCCCTGTTTCTATATCACAGTATGTGTAATGCTCATCCTTTGTTATATGTTT
GTTTTCTTCATCTGGCCGTTGTATTTTTGCATAATATATCGAGCAACGTAAGCAGCTGACTCGAATGTAACGTCTCCAATGGAGGAATAACCATGT
GGCCAGAGGGTTGCAAGGTGGTCGGATGTATATATGAGAGAACCAGAGGGAGTCCTTTTGAATAGTTTCTTATCATGAAAAATCGTATCCGAAG
ATACAGGCATGGAAGTGAGGTCTGCCGAAACTTGT

>000192F|arrow

GGGCAGGATGGTACCGCTAGTCGGGGCCAGACTGTAGTATTACCCTTAGACTTTTTGGCCTGGCCCAGTGGGCCTGGCCCTAGGCCACTGTGTG
TTCGAGTGGGTGTGTCAATTCGAGTACTTACGGAGCGAGAATGAATGTCATACCCCGAGGAAGAACCCTCAAGCGATCAAGAGCGATGTCTCGC
TCGGAGTCTGCGGCTATTATTCAGCGGGCTGTTAAGCGTGCCAAGAATAAGCCTGCTTACCGCAGCAAGTTTCGTTTGTACAAGAACAATAACTT
GTCTCTGTTTCCTCGGCAGATGCGGCAGACGCTGACTTATTCGGAGGCAACTGTTGTGAATTTGGTGGCAACTGTTGGCACCATTTCGAGCTTCC
GAATCCGGGCGAACAGTTTGTTTGACCCCCGTGTTGCTTTGCTTGGGCATCAGCCCATGGGGTTTGACCAGTTGATGGCGATGTATTCCAAGTTT
TGTGTGGTTGGTTCCAAAATCACTGCTTACGTAGGACCTGCGGGAGCTTCAACATTTCTGTATGGTTCCATGGGCATTAATGTGGTGGACCCGGC
CGCCACTCTTGTTACCAGCATCACCGACGCCATCGAGAGCCAGTTCTCCACTTGGAAGAACTTCAACGTCAGCATCACCAACAAGATGCAGTTGG
GGTTTGACACTTCCCAGTACTTTGGTATCAAGGACATCCAGGACGACGATACCCTTACCGGCACCGCAGTGGCGGACTGCAGCAAGCAGGCGTA
CTTTGACATCTGGGTTGCTAGCGACACCGGAGTAGCTGGCCAGAATGTGACGGTTACATTCAACGTGGAGTATGACACGTTGTTCTTTGAGCCG
CGCAATGTACCACCGTCTTAAATACAACTGTATTGTTTGAGTAAGATGCGTAAGCATCGTCGAGTAATCATTAGGCGGTAGCTACCCTATCTAAG
ATATTTTAATATTTAGTGGCCGTTACCCAAAAAAGTCTAAG

>000001F|arrow

ATAACGCTAAACTTAATTCCGAAGACAATACACCAGAACGACTATCTGCAAAAGAACAAGTAACTATGGCAAAACTTCAACTATTAAAACGTAAC
CTCACTTAGGAAAATAATGAAACTTATCCTCGCTTCCGTAAAAGACCGTGCTGCTGAAGCATATGCACGACCAATGTTCGTACCTTCCTCTGGAGT
AGCTATACGCTCTTTTTCAGATGAAATTAATCGTTCTGATACTGAAAATCAACTTTTAATCACCCTGATGACTTCGATCTATATGAATTCGGAACTT
TGACGATTCAACTGGGTTATTCGATTTACATGAACAACCAAAACTCCTATCATTAGGAAAACAAGTTAAACTTAAATAAAACAACCGAGGGGAAA
AGAGATTTATCTTTCCCCCGGAACAACACTAAGGAAAAACATGCACCGCAATCAGTCAGTTAATACTCACCGCTTCGCGATGGTACCTAGAGCCG
ATATACCACGTAGTAAATTCGATGCTCAAAAAACACATAAAACGACTTTCGATGCGGGCTATTTAATTCCTGTATATGTTGATGAAGTGCTCCCTG
GGGACACTTTCAACTTAAAAATGACGGCATTTGCCCGTCTAGCAACGCCTTTATATCCAATCATGGACAACATGATTATGGATTCTTTCTTTTTCTT
TGTACCCAATCGCCTTATATGGAATAACTGGCAAAAATTTATGGGTCAACAAGAAAATCCAACAGACTCAATATCTTATATTGTCCCAACTCAAAC
AAGCCCAACAGATGTTATGCCGTAGGCAGCCTTCAAGACTATATGGGCTTACCAACAGTAGGCCAAATTGATACTGGCCGAACTATTACGCACT
GTGCCTTTTGGCCACGTGCATACAATCTTATTTGGAACGAATGGTTCCGAGATGAAAATTTACAAACAAGCGCAGTAGTTGATAAGGGCGATGG
CCCTGATACTTCCTCAAACTATGTGCTAAAACGTCGTGGTAAAAGACATGATTACTTTACGTCAGCATTACCATGGCCACAAAAAGGTGCGAGTG
TCACCTTACCTTTAGGTACTACGGCTCCAATTAAATGGGATACCATTTCAGGAGACGCAACATCAAACGATAAATTTACGGTAATTCAAACAGATC
CTGGAAATACGACTGCTTTAGCTAGATATGGCAACGCTTATGGTGTTAATACTGCTGGTGTAGTAAATAACGTTTCTAATTTATATACCGACTTAT
CAGAAGCAACTGCTGCAACTGTCAATCAATTAAGACAGTCATTTCAAATTCAAAATTACTTGAAAGGGATGCACGTGGCGGAACACGATACACA
GAAATTATCCGGAGTCACTTTGGAGTTATTTCCCCAGACGCCCGTTTACAAAGGCCTGAATACCTTGGAGGCGGTTCAACACCAATTAATGTTAA
TCCGATTGCTCAAACGTCGGGAACAAACGCTTCTGGAACGACTACCCCTTTGGGCAACCTTGCTGCTATGGGTACTGCTCTCGCTCATAATCATG
GATTTACTCAATCATTTACTGAGCATGGCGTTATTATTGGATTAGTATCCATTAGAGCAGATCTTACTTATCAACAAGGATTAGACCGTATGTGGT
CTAGATCTACACGATATGACTTTTATTTCCCAGCATTTGCTACTCTAGGCGAACAATCTGTTTTGCAAAAAGAAATTTATGCAACAGGAGATACTG
CAGCCGACAATACTGTTTTTGGATATCAAGAACGCTGGGCGGAATATCGTTACAAACCATCTAAAATTACTGGTTTGTTCAAATCAACATCGGCG
GGCACGATCGATGGTTGGCATTTGGCTCAAAAATTTACCGCTGCGCCTACTTTGAATAATACGTTTATTCAAGATACGCCTCCTGTATCACGTGTA
GTAGCCGTTGGAGCAGCTGCAAATGGCCAACATTCTTATTTGACTCATTTTTTGATGTCAAAATGGCAAGACCAATGCCAATGTATTCAGTACCTG
GCTTAATAGACCATTTCTAATGGGACTATTTGACGGAATTGCCGATTTAATCGGCCCTGCTATAGCTATAGGAGCTGCCCCTGCTACTGGGGGAC
TCTCCTTAGCTGCACTTGCACCTGCAGCAATAGGTGCAGCAGGACAATACTTTGGAACACAAAGTCAAAACGCAGCGAGTGCAGAACAAGCGAG
TAATCAACAGAGATTTCAAGCTGAAATGTCTGGAACATCATATCAACGAGCAGTTGAAGATATGAAAAAAGCTGGGTTAAATCCCATGCTTGCG
TATTCACAAGGCGGAGCCACAACACCAGCTGGAGCTATGGCCCAGATGCAAAATGTTCTCGGTAATGCAACTACGTCCGGAACCCAAGCTTATC
AAACGGTTGCTCAAGCAAATCAAGCTATTGCTCAATCTAAACAAATTGAAGCTCAAACAGAACTCACAAGTAATCAAACAGATAATGTACGTGCT
GATACGTTAAACAAATTGGATGAAAATCCAAATATTAGAGCTCAATATAAACAAATACTTGCCGATACTTTCATGAAAAATGAAATAGGCAAAAC
ATCAAGTGCTCAAGCTGCTCAAGCTTTGGCACAATCTCGTTATTCAAACGAGTTAACAAAACTTGCTAAATCAGGGTCAGCTCCTAGTTCTAGCAA
ACCAATTTATCAAGACGTAAAAAACTATCGCCAAAGATGCGTATAGCGCATCTGGCGCAAAACGATACATCGATAACTATCGAGGTCAACCGATT
CAACAAAATCGTACAAATAACCAACCACCAATGGAATGAAAATGACAAAGATTACAGCCCCATTTCTTCGTACTCCGTACAATTACGACACGATT
GCTGCGTCAAATGAGTCAGGGCTGCATTGTGAGGATGCAACTCTGACTCAGCAGCAATTTGCTGAAGAATGCGATATTAATAATATTATGGAAA
AGTTTGGTATGACCGGACTTATTCCTCAAACTCCTTTAACGCCTCAATATGGCGACTTTAGTGGTGTCTATGACTACCACTCTGCTCTGAACCAGA
TTATGGCTTCAGACAACGAATTTATGGCTTTACCAGCCAATATTCGTGAACGATTCGCTAATGATCCCGCGAATCTAATAGATTTTCTAGAAAACC

CTGAAAATCGCAGCGAAGCTGAAAAAATGGGACTGGTAAAACCAGCCCAAACCGAGGTTTCAACCCCTGTTGGAACCTCGGAAGCACAGTTACC
TACTTGATGTAACTGTGCTAGGTGACACCAAACCACAAAAACACGATAAACAAGGACAGAAAAAATGATGCGTCGCAGACCAGCAAATAAGCA
AAAGTCCGCTAGGACTTTCCGTAAACATGCTTCACATACAAAACACGCAAATATGCGAAACTCGCCAATGCGTGGAGGCTGGAGACTCTAATAA
AGTCCCCAGGCACCTCACATGCCTTGTTATCACCCTCTCAAAGCATTTCAATGCTTTGACAAATCAATTGTTTTCGACGAAGTTCGGAAACATGAC
ATCGTTCGATCTTTAGACCTGCCCTGTGGGCAGTGCGTTGGATGCCGTCTAGAACGATCAAGACAATGGGCTATTCGGTGCATGCACGAAGCCC
AATTGCATAAAAACAACTCATTCATAACACTCACATATGACAATACACATCTCCCAAGCGATGGCTCTTTGGATCACAAAGACTTTCAACTGTTCC
TTAAAAGACTTAGAAAAACTCTCGCAAAAAGAGGACTTACAATCCGCTATTACATGGCTGGAGAATATGGTGAACTCTTCGCAAGACCCCACTTC
CATGCCTGTATCTTCGGATACGACTTTCCTGATAAAAAATTATGGAAAAGGACTGCCTCTGGTTCTATGTTATATAGATCCGCAGAACTTGAAGCT
CTCTGGCCATTTGGTTATACCACCATTGGAGATGTTACTTTCGAATCAGCCGCCTACGTGGCTAGATACATAATGAAAAAACAAACAGGGAAAGA
TGCGGAATCTCATTACAAACGCATACACCCTGAAACCGGCGAATATTTAGACTTAAAGCCGGAATATAATAAAATGTCTTTAAAACCGGGAATCG
GTAAAGACTTTTATATAAAATATACTTCGGATATATACCCGCAAGACTACGTAATACTTAGAGGTAAAAAGGTCAAACCACCAAATACTATGACA
AAATGTTTAAAATTGACCAACCTTATGAGTATGACGAATTACTTTACATGCGGGAAA

>000102F|arrow

TAGGCCAAATTGATACTGGCCGAACTATTACGCACTGTGCCTTTTGGCCACGTGCATACAATCTTATCTGGAACGAATGGTTCCGAGATGAAAAT
TTACAAACAAGCGCAGTAGTTGATAAGGGCGATGGCCCTGATACTTCCTCAAACTATGTGCTAAAACGTCGTGGTAAAAGACATGATTACTTTAC
GTCAGCATTACCATGGCCACAAAAAGGTGCGAGTGTCACCTTACCTTTAGGTACTACGGCTCCAATTAAATGGGATACCATTTCAGGAGACGCAA
CATCAAACGATAAATTTACGGTAATTCAAACAGATCCTGGAAATACGACTGCTTTAGCTAGATATGGCAACGCTTATGGTGTTAATACTGCTGGT
GTAGTAAATAACGTTTCTAATTTATATACCGACTTATCAGAAGCAACTGCTGCAACTGTCAATCAATTAAGACAGTCATTTCAAATTCAAAAATTA
CTTGAAAGGGATGCACGTGGCGGAACACGATACACAGAAATTATCCGGAGTCACTTTGGAGTTATTTCCCCAGACGCCCGTTTACAAAGGCCTG
AATACCTTGGAGGCGGTTCAACACCAATTAATGTTAATCCGATTGCTCAAACGTCGGGAACAAACGCTTCTGGAACGACTACCCCTTTGGGCAAC
CTTGCTGCTATGGGTACTGCTCTCGCTCATAATCATGGATTTACTCAATCATTTACTGAGCATGGCGTTATTATTGGATTAGTATCCATTAGAGCA
GATCTTACTTATCAACAAGGATTAGACCGTATGTGGTCTAGATCTACACGATATGACTTTTATTTCCCAGCATTTGCTACTCTAGGCGAACAATCT
GTTTTGCAAAAAGAAATTTATGCAACAGGAGATACTGCAGCCGACAATAGTTTTTGGATATCAAGAACGCTGGGCGGAATATCGTTACAAACCA
TCTAAAATTACTGGTTTGTTCAAATCAACATCGGCGGGCACGATCGATGGTTGGCATTTGGCTCAAAAATTTACCGCTGCGGCCTACTTTGAATA
ATACGTTATTCAGATACGCCTCCTGTATCACGTGTATAGCCGTTGGAGCAGCTGCAAATGGCCACACAATTCTTATTTGACCATCATTTTTGATGT
CAAAATGGCAAGACCAATGCCAATGTATTCAGTACCTCGGCTTAATAGACCATTTCTAATGGACCTATTTGACGGAATTGCCGATTTAATTCGCCC
TGCTATAGCTATAGAGCTGCCCCTGCTATGGGGGACTCTCCTTAGCGGCACTTGCACCTGCAGCAATAGGTGCAGCAGGACAATACTTTGGAAC
ACAAAGTCAAAAACGCAGCGAGTGCAAACAAGCGAGTAATCAACAGAGATTTCAAGCTGAAATGTCTGGAACATCATATCAACGAGCAGTTGA
AGATATGAAAAAAGCTGGGTTAAATTCCCATGCTTGCGTATTCACAAGGCGGAGCCACAACACCAGCTGGAGCTATGGCCCAGATGCAAAATGT
TCTCGGTAATGCAACTACGTCCGGAACCCAAGCTTATCAAACGGGTTGCTCAAGCAAATCAAGCTATTGCTCAATCTAAACAAATTGAAGCTCAA
ACAGACTCACAAGTAATCAAACAGATATGTACGTGCTGATACGTTAAACAAATTGGATGAAATCCAAATATTAGAGCTCAATATAAACAAATACT
TGCCGATACTTCATGAAAAATGAAATAGGCAAAACATCAAGTGCTCAAGCTTGCTCAAGCTTTGGCACAATCTCGTTATTCAAACGAGTTAACAA
AACTTGCTAAATCAGGGTCAGCTCCTAGTTCTAGCAACCAATTTATCAAGACGTAAAAAAATATCGCCAAAGATGCGTATAGCGCATCTGGCGCA
AAACGATACATCGATAACTATCGAGGTCACCGATTCAACAAAATCGTACAAATAACCAACCACCAATGGAATGAAAATGACAAAGATTACAGCC
CCATTTCTTCGTACTCCGTACAATTACGACACGATTGCTGCGTCAAATGAGTCAGGGCTGCATTGTGAGGATGCAACTCTGACTCAGCAGCAATT
TGCTGAAGAATGCGATATTAATAATATTATGGAAAAGTTTGGTATGACCGGACTTATTCCTCAAACTCCTTTATCGCCTCAATATGGCGACTTTAG
TGGTGTCTATGACTACCACTCTGCTCTGAACCAGATTATGGCTTCAGACAAACGAATTTATGGCTTACCAGCCAATATTCGTGAACGATTCGCTAA
TGATCCCGCGAATCTAATAGATTTTCTAGAAAATCCTGAAAATCGCAGCGAAGCTGAAAAAATGGGACTGGTAAAACCAGCCCAAACCGAGGTT
TCAACCCTGTTGGAACCTCGGAAGCACAGTTACCTACTTGATGTAACTGTGCTAGGTGACACCAAACCACAAAAAACACGATAAACAAGGACAG
AAAAAATGATGCGTCGCAGACCAGCAAATAAGCAAAAGTCCGCTAGGGACTTCCGTAAACATGCTTCACATACAAAACACGCAAATATGCGAAA
CTCGCCAATGCGTGGAGGCTGGAGACTCTAATAAAGTCCCCAGGCACCTCACATGCCTTGTTATCACCCTCGTCAAAGCATTTCAATGCTTTGACA
AATCAATTGTTTTCGACGAAGTTCGGAAACATGACATCGTTTCGATCTTTAGACCTGCCCTGTGGGCAGTGCGTTGGATGCCGTCTAGAACGATC
AAGACAATGGGCTATTCGGTGCATGCACGAAGCCCAATTGCATAAAAACAACTCATTCATAACACTCACATATGACAATAACATCTCCCAAGCGA
TGGCTCTTTGGATCACAAAGACTTTCAACTGTTCCTTAAAAGACTTAGAAAACTCTCGCAAAAGAGGACTTAGCAATCCGCTATTACATGGCTGG
AGAATATGGTGAACTCTTCGCAAGACCCCACTTCCATGCCTGTATCTTCGGATACGACTTTCCTGATAAAAAATTATGGAAAAAGGACTGCCTCT
GGTTCTATGTTATATAGATCCGCAGAACTTGAAGCTCTCTGGCCATTGGTTATACCACCATTGGAGATGGTTACTTTCGAATCAGCCGCCTACGTG
GCTAGATACATAATGAAAAAACAAACAGGGAAAGATGCGGAATCTCATACAAACGCATACACCCTGAAACCGCGAATATTTAGACTTAAAGCCG
GAATATAATAAATGTCTTAAAACCGGAATCGTGTAAAAGACTTTATATAAAATACTTGCGGATATATACCCGCAAGACTACGTAATACTTAGAGG
TAAAAAGGTCAAACCACCAAAATACTATGACAAAATGTTTAAATTGACCAACCTTATGAGTATGACGAATTACTTTACATGCGGGAAAATAACGC
TAACTTAATTCCGAAGACAAATACACCAGAACGACTATCTGCAAAGAACAAGTAACTATGGCAAACACTTCAACTAGTAAAACGTAAATCTCACT
TAGGAAAATAATGAAACTTATCCTCGCTTCCGTAAAAGACCGTGCTGCTGAAGCATATGCACGACCAATGTTCGTACCTTCCTCTGGAGTAGCTA

TACGCTCTTTTTCAGATGAAAATTAATCGTTCGATACTGAAAAATCAACTTTTTAATCACCCTGATGACTTCGATCTATATGAATTCGGAAACTTTT
GACGATTCAACTGGGGTTATTCGATTTACATGAACAACCAAAACTTCTATCATTAGGAAAACAAGTTAAAACTTAAATAAAACAACCGAGGGGA
AAAGAGATTTAGTCTTCCCCCGGAACAACACTAAGGAAAAACTGCACCGCAATCAGTCAGTTAATACTCATCGCTTCGCGATGGTACCAGACCGA
TATACCACGTAGTAAATTCGATGCTCAAAAACACATAAAACGACTTTCGATGCGGGCTATTAATTCCTGTATATGTTGATGAAGTGCTCCCTGGG
GGACACTTTCAACTAAAAATGACGGCATTTGCCCGTCTAGCAACGCCTTTATATCCAATCATGACAACATGATTATGGATTCTTCTTTTTCTTTGT
ACCCAATCGCCTTATATGGAATAACTGGCAAAAATTTATGGGTCAACAAGAAAATCCAACAGACTCAATATCTTATATTGTCCCAACTCAAACAA
GCCCAACAGATGGTTATGCCGTAGGCAGCCTTCAAGACTATATGGGCTTACCAACAG

>000199F|arrow

GTAGGAAGAGAAGTAAATCGCTGTCCATAATGCCAAGCATCAATAGTGCCAGCAGAAGTAGACCTAAAGAAACCAGTAATTTGAGAAGGTTTG
TAACGATACTCCGCCCAACGTTCTTGATAACCAAATACATCATTGTCGGCTGCAGTACCTTGAACATAAATTTCTTTGTTCAAAATAGCTTGCTCAC
CCAAAGTGGCAAATACTGGGAAATAAAAGTCATATCGTGTAGACCTAGACCACATCTTAGGAAGACCCTGTTGATATGTGAGGTCAGCACGTAC
AGAAACTAATCCGATAATGTATCCATGTTCTTGAGCAGCATACGTAAAGCCGTGTCCCTGAGCCAATGCAGTACCCATTGCAGCAAGGTTACCTT
GCGGAGTAGCAGAACCAGTAACCGACGTTGCAGAAGTCTGAGCAATCGGATTAACATTAACAAGGGTCGAACCTCCACCAATATATTCAGGACG
TTGTAAACGATAATCCTGTGGAGTTACTCCAAAGTGAGCACGTAGTAACTCAGTATAACGAGTACCACCACGTGCATCGCGCTCCAATAACTTCT
GAATCTGGAAAGATTGACGAAGTTGGTTAATAGTCGCAGCAGTAGCAGTAGACAAATCAGCGTATAACTGATTAGTAGCAACACCAGCATTTGT
ACTATTAGACAAAGTATTAGAAGCTGAATTTAATTCTCTTAAAGCACCAACAGCAGTTTGAAAAACACTATAACCAGCAGTACCATTGCCAGCTG
GTATGTCAGAATAAATATTAGCACGTGTTCCTAACGGTAAAGAAACAGAAGCACCCTTCTGTGGCCAAGGCAATGCACCAGTAAAATAATCTTTA
CGCTTACCACGTCTCAAAAGTGTATAGTTAGCAGGAACATCACCTGAATCACCAAGATTTAACGTTACAGAATTCTGTAAGTTTTCATCTCTAAAC
CATTCGTTATAAATCTCATTATAAGCACGTAACGGCAAAACATTATGCGTTACTGTATTAGCGCCAGTAATCTGACCAGCAGTAGGTAAACCAAA
ATAATCAAAAATTGAACAAACAGCATAACCACCAGCAGGACTAGTAATAGTAGGCACTACAAAAGAAATAGAATCACCAGGATTCGTTTGTTCA
CCCATAAACTTTGGGCCAATTGTTCCAAAGTAACGATTAGGTACAAAGAAAAAGAAAGTATCAAGATGCAAGTTGTCCATCACAGGAAATAACG
GTGTGGCCAAACGTGCAAATGCAGTCATCTTTACACGATGAGTATCGCCAGGCAATACTTCATCACAATAAATAGGAACTAAAAAACCAGCATCA
AACGTGGTTTATGAGCATATTGAGTATTAAAACTAGAGCGGGGAATGTCCGCTTTAGGAACCATAGCAAATGAATGCGTACTTACTGACTTATTG
CGAAAAAAACATCTCCCGTAGTTCCGTACCACTCTTTCGAGTGATACGGTATAAAAAAAACCTTACTCGCCTTCGCGAATCATTACATCTTTTGCA
CGAGCAATCAACTTAGGGCTTTCCAGTAGTTCAAAAACACCAGTGGCATCGTCAAAAAGACCCAAATAGAACATATGAAAATCATCAGGATGTTT
ATACAACTGATTATCTTCGCTAGCTCGATTGACTTCATCCTGAAACTGACGTACTGCAACACCTTCAGATGCAACATAAGCTGGACGACCAAAAG
CATCTGCAGCAGTATCCTTAATAGAAACAATAACCATCTTCATAAAAACTCCTTAAATAGTACGTTTTAACAATGACAACTTAGCCAACGCAACTT
TTTCCTTAACAGCAAGTCGCTCAAGCGTGTTGTCCTCATGCCTAGATCGACCTTCCATCTCTCTGGCAAACTGAATCATATCGAATTCTTCAGGAA
ACTTCAACTTAAATTTATTATCATAAAACCGTGGTGGACGGCACTTTTTGCCACGCACCACAACGTGGTCTGACGTATAAACGTCTGACATGTACT
TATCTAACCACGATTGCCCGATACCGGGCTTCAATGACATCTTATTAAATTCTGGCTTACGCTGAATTATCTCACCAGTCTCTAAATCACAATATTG
ATAATGGGCACCCGCATCAACCACTTCGTGGTTTCATTGACAGTAACCCATTAATCTTCTTCATAATATATCTTGCAACATAAGCAGCAGACTCAA
AAGTAACATCACCAATTGTAGAATAGCCAAACGGCCACAATTCTTCCAAAATCTCTGACGTGTAGAGGATAGAGCCAGTCTGCGTTCTTTTAAAT
ATTTCTTATCCGGAAAATCAAGACCAAACAGACAAGCATGGAAATGAGGACGATCAAAAGATTCACCATATTCACCTGCCATATAAAAACGTATC
GTTTTCCCAGTAAAACGCTTACGTAACCGCTTCATAAAAAGCTGATAATCATTGTAATCCAATGACATATCCTTAGGACAATGCTCTGGAGCATAT
GTCAAAGTAATAAAACAATTACTAGTATGCATTTGTGCCTCATGCATACAACGAATCGCCCACTGACGTGAGCGTTCAAGCGACAACCAACACAC
TGACCACAAGGCAATGATAGGGTACGGACTACATCCGCACCCGGTATTTCCCGCCAAATTATAGACCTGTCACTGCATTGATAAGCCGTTAAGG
GCTTATAACAGGCCATAATTACAAACGATAGCCACCACGCTGGGGAGCGTGTCTCATATTAATTGACTTCGTCTTACTAGCAGTTCTGCGAAATG
ACTTTGCAGATTTATATTTGTTTACTGGCTTTCTTCGTAACATGATGAACTCCGTAGTTAAAATAGTGGTTTGGTGTCACCTAGCACAGTTACATCA
AGTAGAGTAACTGTGCTGGCCTCAGGATTTCATCCTTCGGCCTTAGGTGTTTCTGTAGAAACGATGGGTTCAACCACAGGTTGTCCATCAATAAG
ACCCAATTGAATCGCTTCATCACGATTCTGGTCGTTCTCAAGGAACTCCAATAATTTGACAGGATCATGGTCAAATCGGACTCTTAATTTCGCTGG
CAGAGCCATGAAATCGTCCATAGTTGCGTTAATTTGATTCAACGCAGAATGGTAATCAGTAACACCACTAAAATCGCCGTATTGAGGCGATACAG
GGGCCGTTGGAAGTTCCCCTGTAACCCCGAAACGTTCAATGATGACATTAATGTCACATTCATCTTTCATATGCTGTTGAGCAAGACTTGGGTCTT
GACACAGCAACGCATCGTTTACCGATGCTTCATCTTTATCATAGTTGTACGGATTACGTACAAATGGCAAATTCGCTTTACTCATTATTTGACTCCA
ATTCCCCAAGGGGTTAGTTGATTAGGATTATTTTTGAGTTATCAAAAATTTCTTTCGAAGCATCGATACCCCTAGACCAAATAGCTGGGGATGGA
GCCAATAGCTCTTAGTAGTATTATAGACTGAGCGGATGTTAAATCCGCAGTCGTCTTTAAAGCAATATCAGCCAAAATACGTTTATTTTCGGCTGT
AATATTAGGAGCAGTAAGCAACTTATTAACAGTATCAGCCCTAGTTAGCGGTACCAGCTTCAGTAGCTTCAGTCTGGGCGATAATCTGCTTTTCT
GTTTCTGATGCATTACGAATCTGTTGCATCATAGAAGCAGTATTAATAGCTGAATTAGTTGCATTACCTAAAACATTTTCCATAGTAGCAGTTGAA
CCAGATGGGGTAGAAGCACCACCACGTTGATAGGCTAACATAGGAGATAAACCAGCAGCTTCTAAATCTTTAACAGCACGCTGGTAACTTGTTC
CACTCATATCGGCTTGAAAATCCATTTGCCTCTGAGCCATCTCCTGATTAGCTTTATTAGCAGAAGTAGAACCTAAATAAGAACCAACAGCGCCCA
AAGCAGTACCGACACCAGGAGTAAAGAAACTAGAAGCTGAAGACAGCTTAGAACCAACATTAGTAACCGCATCAAGTATTCCACCAAACATAAA
CTAACGCCCTTCGGTTGTTTCCTCACTACTCCTTACGGAGTAGTCGAGGTTATATAAAACATTAGAAATGATCAATTAGACCAGGTACGCTGTACA

TAGGCATAGGTCTGGCCATCTGACAATCAAAGAAAGCATCCAAAAATTGCTGACCATTTGCTGCAGCTCCGACCGCCGTAGTACGAGCAACTGG
AGGGGTCTCTTGAATAAACGTTGAATTCAAC

>000022F|arrow

AATCGCCCACTGACGTGAGCGTTCAAGGCGACAACCAACACACTGACCACAAGGCAATGATAGGGTACGGACTACATCCGCACCCGGTATTTCC
CGCCAAATTATAGACCTGTCACTGCATTGATAAGCCGTTAAGGGCTTATAACAGGCCATAATTACAAACGATAGCCACCACGCTGGGGAGCGTG
TCTCATATTAATTGACTTCGTCTTACTAGCAGTTCTGCGAAATGACTTTGCAGATTTATATTTGTTTACTGGCTTTCTTCGTAACATGATGAACTCCG
TAGTTAAAATAGTGGTTTTGGTGTCACCTAGCACAGTTACATCAAGTAGAGTAACTGTGCTGGCCTCAGGATTTCATCCTTCGGCCTTAGGTGTTT
CTGTAGAAACGATGGGTTCAACACAGGTTGTCCATCAATAAGACCCAATTGAATCGCCTTCATCACGATTCTGGTCGTTCTCAAGGAACTCCAAT
AATTTGACAGGATCATGGTCAAATCGGACTCTTAATTTCGCTGGCAGAGCCATGAAATCGTCCATAGTTGCGTTAATTTGATTCAACGCAGAATG
GTAATCAGTAACACCACTAAAATCGCCGTATTGAGGCGATACAGGGGCCGTTGGAAGTTCCCCTGTAACCCCGAAACGTTCAATGATGACATTA
ATGTCACATTCATCTTTCATATGCTGTGAGCAAGACTTGGGTCTTGACACAGCAACGCATCGTTTACCGATGCTTCATCTTATCATAGTTGTACGG
ATTACGTACAAATGGCAAATTCGCTTTACTCATTATTTGACTCCAATTCCCAAGGGGTTAGTTGATTAGGATTATTTTTGAGTTATCAAAAATTTCT
TTCGAAGCATCGATACCCCTAGACCAAATAGCTGGGGATGGAGCCAATAGCCTCTTAGTATTATATGACTGAGCGGATGTTAAATCCGCAGTCGT
ATTCTTTAAAGCAATATCAGCCAAAATACGTTTATTTTCGGCTGTAATATTAGGAGCAGTAAGCAACTTATTAACAGTATCAGCCCTAGTATTAGC
GGTACCAGCTTCAGTAGCTTCAGTCTGGGCGATAATCTGCTTTTCTGTTTCTGATGCATTACGAATCTGTTGCATCATAGAAGCAGTATTAATAGC
TGAATTAGTTGCATTACCTAAAACATTTTCCATAGTAGCAGTTGAACCAGATGGGGTAGAAGCACCACCACGTTGATAGGCTAACATAGGAGAT
AAACCAGCAGCTTCTAAATCTTTAACAGCACGCTGGTAACTTGTTCCACTCATATCGGCTTGAAAATCCATTTGCCTCTGAGCCATCTCCTGATTAG
CTTTATTAGCAGAAGTAGAACCTAAATAAGAACCAACAGCGCCCAAAGCAGTACCGACACCAGGAGTAAAGAAACTAGAAGCTGAAGACAGCT
TAGAACCAACATTAGTAACCGCATCAAGTATTCCACCAAACATAAACTAACGCCCTTCGGTTGTTTCCTCACTACTCCTTACGGAGTAGTCGAGGT
TATATAAAACATTAGAAATGATCAATTAGACCAGGTACGCTGTACATAGGCATAGGTCTGGCCATCTGACAATCAAAGAAAGCATCCATTAAAAA
TTGCTGACCATTTGCTGCAGCTCCGACCGCCGTAGTACGAGCAACTGGAGGGGTCTCTTGAATAAACGTTGAATTCAACGTAGGAAGAGAAGTA
AATCGCTGTCCATAATGCCAAGCATCAATAGTGCCAGCAGAAGTAGACCTAAAGAAACCAGTAATTTGAGAAGGTTTGTAACGATACTCCGCCC
AACGTTCTTGATAACCAAATACATCATTGTCGGCTGCAGTACCTTGAACATAAATTTCTTTGTTCAAAATAGCTTGCTCACCCAAAGTGGCAAATA
CTGGGAAATAAAAGTCATATCGTGTAGACCTAGACCACATCTTAGGAAGACCCTGTTGATATGTGAGGTCAGCACGTACAGAAACTAATCCGAT
AATGTATCCATGTTCTTGAGCAGCATACGTAAAGCCGTGTCCCTGAGCCAATGCAGTACCCATTGCAGCAAGGTTACCTTGCGGAAGTAGCAGA
ACCAGTAACCGACGTTGCAGAAGTCTGAGCAATCGGATTAACATTAACAAGGGTCGAACCTCCACCAATATATTCAGGGACGTTGTAAACGATA
ATCCTGTGGAGTTACTCCAAAGTGAGCACGTAGTAACTCAGTATAACGAGTACCACCACGTGCATCGCGCTCCAATAACTTCTGAATCTGGAAAG
ATTGACGAAGTTGGTTAATAGTCGCAGCAGTAGCAGTAGACAAATCAGCGTATAACTGATTAGTAGCAACACCAGCATTTGTACTATTAGACAA
AGTATTAGAAGCTGAATTTAATTCTCTTAAAGCACCAACAGCAGTTTGAAAAACACTATAACCAGCAGTACCATTGCCAGCTGGTATGTCAGAAT
AAATATTAGCACGTGTTCCTAACGGTAAAGAAACAGAAGCACCCTTCTGTGGCCAAGGCAATGCACCAGTAAATAATCTTTACGCTTACCACGTC
TCAAAAGTGTATAGTTAGCAGGAACATCACCTGAATCACCAAGATTTAACGTTACAGAATTCTGTAAGTTTTCATCTCTAAACCATTCGTTATAAA
TCTCATTATAAGCACGTAACGGCAAAACATTATGCGTTACTGTATTAGCGCCAGTAATCTGACCAGCAGTAGGTAAACCAAAATAATCAAAAATT
GAACAAACAGCATAACCACCAGCAGGACTAGTAATAGTAGGCACTACAAAAGAAATAGAATCACCAGGATTCGTTTGTTCACCCATAAACTTTG
GCCAATTGTTCCAAAGTAAACGATTAGGTACAAAGAAAAGAAAGTATCAAGATGCAAGTTGTCCATCACAGGAAATAACGGTGTGGCCAAACGT
GCAAATGCAGTCATCTTTACACGATGAGTATCGCCAGGCAATACTTCATCACAATAAATAGGAACTAAAAACCAGCATCAAACGTGGTTTTATGA
GCATATTGAGTATTAAAACTAGAGCGGGGAATGTCCGCTTTAGGAACCATAGCAAATGAATGCGTACTTACTGACTTATTGCGAAACATAAACAT
CTCCCGTAGTTCCGTACCACTCTTTCGAGTGATACGGTATAAAAAAACCTTACTCGCCTTCGCGAATCATTACATCTTTTGCACGAGCAATCAACTT
AGGGCTTTCCAGTAGTTCAAAAACACCAGTGGCATCGTCAAAAAGACCCAAATAGAACATATGAAAATCATCAGGATGTTTATACAACTGATTAT
CTTCGCTAGCTCGATTGACTTCATCCTGAAACTGACGTACTGCAAACACCTTCAGATGCAACATAAGCTGGACGACCAAAAGCATCTGCAGCAGT
ATCCTTAATAGAAACAATAACCATCTTCATAAAAACTCCTTAAATAGTACGTTTTAACAATGACAACTTAGCCAACGCAACTTTTTCCTTAACAGCA
AGTCGCTCAAGCGTGTTGTCCTCATGCCTAGATCGACCTTCCATCTCTCTGGCAAACTGAATCATATCGATTCTTCAGGAAACTTCAACTTAAATTT
ATTATCATAAAACCGTGGTGGACGGCACTTTTTGCCACGCACCACAACGTGGTCTGACGTATAAACGTCTGACATGTACTTATCTAACCACGATT
GCCCGATACCGGGCTTCAATGACATCTTATTAAATTCTGGCTTACGCTGAATTATCTCACCAGTCTCTAAATCACAATATTGATAATGGGCACCCG
CATCAACCACTTCGTGGTTTTCATTGACAGTAACCCCATTAATCTTCTTCATAATATATCTTGCAACATAAGCAGCAGACTCAAAAGTAACATCACC
AATTGTAGAATAGCCAAACGGCCACAATTCTTCCAAAATCTCTGACGTGTAGAGGATAGAGCCAGTCTGCGTTCTTTTAAATATTTTCTTATCCGG
AAAATCAAGACCAAACAGACAAGCATGGAAATGAGGACGATCAAAAGATTCACCATATTCACCTGCCATATAAAAACGTATCGTTTTTCCCAGTA
AAACGCTTACGTAACCGCTTCATAAAAGCTGATAATCATTGTAATCCAATGACATATCCTTAGGACAATGCTCTGGAGCATATGTCAAAGTAATA
AAACAATTACTAGTATGCATTTGTGCCTCATGCATACAACG

>000039F|arrow

TATTTTCTTATCCGGAAAATCAAGACCAAACAGACAAGCATGGAAATGAGGACGATCAAAAGATTCACCATATTCACCTGCCATATAAAAACGTA
TCGTTTTCCCCAGTAAAACGCTACGTAACCGCTTCATAAAAAGCTGATATCATTGTAATCCAATGACATATCCTTAGGACAATGCTCTGGAGCATA
TGTCAAAGTAATAAAACAATTACTAGTATGCATTTGTGCCTCATGCATACAACGAATCGCCCACTGACGTGAGCGTTCAAGGCGACAACCAACAC
ACTGACCACAAGGCAATGATAGGGTACGGACTACATCCGCACCCGGTATTTCCCGCCAAATTATAGACCTGTCACTGCATTGATAAGCCGTTAAG
GGCTATAACAGGCCATAATTACAAACGATAGCCACCACGCTGGGGAGCGTGTCTCATATTAATTGACTTCGTCTTACTAGCAGTTCTGCGAAATG
ACTTTGCAGATTTTATTTGTTTACTGGCTTTCTTCGTACCATGATGAACTCCGTAGTTAAAATAGTGTTTGGTGTCACCTAGCACAGTTACATCAAG
TAGAGTAACTGTGCTGGCCTCAGGATTTCATCCTTCGGCCTTAGGTGTTCTGTAGAAACGATGGGTCAACAACAGGTTGTCCATCAATAAGACCC
AATTGATCGCTTCATCACGATTCTGGTCGTTCTCAAGGAACTCCAATAATTTGACAGGATCATGGTCAAATCGGACTCTTAATTTCGCTGGCAGAG
CCATGAAATCGTCATAGTTGCGTTAATTTGATTCAACGCAGAATGGTAATCAGTAACACCACTAAAATCGCCGTATGAGGCGATACAGGGGTCC
GTTTGGAAGTTCCCCTGTAACCCCGAAACGTTCAATGATGACATTAATGTCACATTCATCTTTCATATGCTGTTGAGCAAGACTGGGGTCTTGACA
CAGCAACGCATCGTTTACCGATGCTTCATCTTTATCATAGTTGTACGGATTACGTACAAATGGCAAATTCGCTTTACTTCATTATTTGACTCCAATG
CCCCAAGGGGTTAGTTGATTAGGATTATTTTGAGTTTATCAAAAAAATTTCTTTCGAAGCATCGATACCCCTAGACCAAATAGCTGGGGATGGAG
CCAATAGCCTCTTAGTATTATAGGACTGAGCGGATGTTAAATCCGCAGTCGTATTCTTTAAAAGCAATATCAGCCAAAATACGTTTATTTTCGGCT
GTAATATTAGGAGCAGTAAGCAACTTATTAACAGTATCAGCCCTAGTATTAGCGGTACCAGCTTCAGTAGCTTCAGTCTGGGCGATAATCTGCTT
TTCTGTTTCTGATGCATTACGAATCTGTTGCATCATAGAAGCAGTATTAATAGCTGAATTAGTTGCATTACCTAAAACATTTTCCATAGTAGCAGTT
GAACCAGATGGGGTAGAAGCACCACCACGTTGATAGGCTAACATAGGAGATAAACCAGCAGCTTCTAAATCTTTAACAGCACGCTGGTAACTTG
TTCCACTCATATCGGCTTGAAAATCCATTTGCCTCTGAGCCATCTCCTGATTAGCTTTATTAGCAGAAGTAGAACCTAAATAAGAACCAACAGCGC
CCAAAGCAGTACCGACACCAGGAGTAAAGAAACTAGAAGCTGAAGACAGCTTAGAACCAACATTAGTAACCGCATCAAGTATTCCACCAAACAT
AAACTAACGCCCTTCGGTTGTTTCCTCACTACTCCTTACGGAGTAGTCGAGGTTATATAAAACATTAGAAATGATCAATTAGACCAGGTACGCTGT
ACATAGGCATAGGTCTGGCCATCTGACAATCAAAGAAAGCATCCATTAAAAATTGCTGACCATTTGCTGCAGCTCCGACCGCCGTAGTACGAGCA
ACTGGAGGGGTCTCTTGAATAAACGTTGAATTCAACGTAGGAAGAGAAGTAAATCGCTGTCCATAATGCCAAGCATCAATAGTGCCAGCAGAAG
TAGACCTAAAGAAACCAGTAATTTGAGAAGGTTTGTAACGATACTCCGCCCAACGTTCTTGATAACCAAATACATCATTGTCGGCTGCAGTACCT
TGAACATAAATTTCTTTGTTCAAAATAGCTTGCTCACCCAAAGTGGCAAATACTGGGAAATAAAAGTCATATCGTGTAGACCTAGACCACATCTTA
GGAAGACCCTGTTGATATGTGAGGTCAGCACGTACAGAAACTAATCCGATAATGTATCCATGTTCTTGAGCAGCATACGTAAAGCCGTGTCCCTG
AGCCAATGCAGTACCCATTGCAGCAAGGTTACCTTGCGGAGTAGCAGAACCAGTAACCGACGTTGCAGAAGTCTGAGCAATCGGATTAACATTA
ACAAGGGTCGAACCTCCACCAATATATTCAGGACGTTGTAAACGATAATCCTGTGGAGTTACTCCAAAGTGAGCACGTAGTAACTCAGTATAACG
AGTACCACCACGTGCATCGCGCTCCAATAACTTCTGAATCTGGAAAGATTGACGAAGTTGGTTAATAGTCGCAGCAGTAGCAGTAGACAAATCA
GCGTATAACTGATTAGTAGCAACACCAGCATTTGTACTATTAGACAAAGTATTAGAAGCTGAATTTAATTCTCTTAAAGCACCAACAGCAGTTTGA
AAAACACTATAACCAGCAGTACCATTGCCAGCTGGTATGTCAGAATAAATATTAGCACGTGTTCCTAACGGTAAAGAAACAGAAGCACCCTTCTG
TGGCCAAGGCAATGCACCAGTAAAATAATCTTTACGCTTACCACGTCTCAAAAGTGTATAGTTAGCAGGAACATCACCTGAATCACCAAGATTTA
ACGTTACAGAATTCTGTAAGTTTTCATCTCTAAACCATTCGTTATAAATCTCATTATAAGCACGTAACGGCAAAACATTATGCGTTACTGTATTAGC
GCCAGTAATCTGACCAGCAGTAGGTAAACCAAAATAATCAAAAATTGAACAAACAGCATAACCACCAGCAGGACTAGTAATAGTAGGCACTACA
AAAGAAATAGAATCACCAGGATTCGTTTGTTCACCCATAAACTTTGGCCAATTGTTCCAAAGTAAACGATTAGGTACAAAGAAAAAGAAAGTATC
AAGATGCAAGTTGTCCATCACAGGAAATAACGGTGTGGCCAAACGTGCAAATGCAGTCATCTTTACACGATGAGTATCGCCAGGCAATACTTCA
TCACAATAAATAGGAACTAAAAAACCAGCATCAAACGTGGTTTTATGAGCATATTGAGTATTAAAACTAGAGCGGGGAATGTCCGCTTTAGGAA
CCATAGCAAATGAATGCGTACTTACTGACTTATTGCGAAACATAAACATCTCCCGTAGTTCCGTACCACTCTTTCGAGTGATACGGTATAAAAAAA
ACCTTACTCGCCTTCGCGAATCATTACATCTTTTGCACGAGCAATCAACTTAGGGCTTTCCAGTAGTTCAAAAACACCAGTGGCATCGTCAAAAAG
ACCCAAATAGAACATATGAAAATCATCAGGATGTTTATACAACTGATTATCTTCGCTAGCTCGATTGACTTCATCCTGAAACTGACGTACTGCAAC
ACCTTCAGATGCAACATAAGCTGGACGACCAAAAGCATCTGCAGCAGTATCCTTAATAGAAACAATAACCATCTTCATAAAAACTCCTTAAATAG
TACGTTTTAACAATGACAACTTAGCCAACGCAACTTTTTCCTTAACAGCAAGTCGCTCAAGCGTGTTGTCCTCATGCCTAGATCGACCTTCCATCTC
TCTGGCAAACTGAATCATATCGAATTCTTCAGGAAACTTCAACTTAAATTTATTATCATAAAACCGTGGTGGACGGCACTTTTTGCCACGCACCAC
AACGTGGTCTGACGTATAAACGTCTGACATGTACTTATCTAACCACGATTGCCCGATACCGGGCTTCAATGACATCTTATTAAATTCTGGCTTACG
CTGAATTATCTCACCAGTCTCTAAATCACAATATTGATAATGGGCACCCGCATCAACCACTTCGTGGTTTTCATTGACAGTAACCCCATTAATCTTC
TTCATAATATATCTTGCAACATAAGCAGCAGACTCAAAAGTAACATCACCAATTGTAGAATAGCCAAACGGCCACAATTCTTCCAAAATCTCTGAC
GTGTAGAGGATAGAGCCAGTCTGCGTTCTTTTAAA

>000176F|arrow

CCCGATGCGCGTCTCCAACGGCCTGAATACATTGGAGGCGGTTCAACACACATTAATATCAATCCAATCGCCCAGACGAATGGTACCGGAGCTTC
CGGGACCACTACTCCTCTCGGTACACTTGGCGCTATGGGTACTGGGCTCGCTCACAATCATGGCTTTACTTATTCAAGCACTGAACATGGTGTAAT
TATCGGTCTCGTTTCAGTACGAGCCGATTTAACATACCAACAAGGTATGCACCGCATGTGGAATCGTTCCACACGTTATGATTTCTATTTCCCTGC
TTTCGCCACTTTGGGCGAACAAGCAGTATTAAATGAAGAAATCTACGTACGAGGCGATGCCAACGATACAGGAGTGTTTGGATACCAAGAACGT
TGGGCAGAATATCGTTATATGCCAAGCCGAATTTCCAGTCTGTTCCGTAGTACGGCAGCTGGAACAATTGACGGCTGGCATTTAGCCCAACGGTT

TACAACACTTCCAACTTTGAATAACACGTTTATTCAAGAAAATCCACCTGTCTCTCGAACCCTTGCGGTCGGAGCAGCTGCCAACGGCCAGCAAA
TCATTTTTGATAGCTTTTTGATATTAAAAAAGCACGGCCAATGCCAATGTACTCTGTACCTGGCTTAATCGACCACTTCTAATGGCACTAGAAGC
CGCTGCCTCAGGCGCCGCATCTGGCGCCGCTTTTGGACCTTACGGCTCCTTAATTGGAGCCGGAATAGGTGCGGCCGCTAGTTATTTTGGTGGTC
AAGAACAAACGCTGCCAGCGCACAACAAGCTGCAGCAATGATGCAATTCCAAGATGGTATGCGACGTACTGCATATCAAGACGCAGTAGCGAT
CTTAAGGCTGCAGGTCTTAACCCTATGCTGGCTTATTCACAAGGCGGAGCCAAAGTCCAGCCTGGTGCGCAAGCTCCAGTAGGAAATCACTAG
GTGAGGCTGGAAATTCAGCCCGTGAAGCTGCCATGGCAGTCGCCAATTTTAAACAATTACAAACTCAGAATATCCTGACACATCGCAAGCCGAA
AAAACGGACGCGGATACAAATCTATCACGTGATCAGGCAACATATACTCGAGCAAATACAGCTCGTGAAATTGCTCAGATGCCGGGATACGGCA
AATTTGGTCAGCTTCGCGATGCCCAAATAGAGCAATTAAGGACATCAAGTGCATTACAAGCTGCACAACAGCGACAAGCGTTAAGTCAAAGTGC
ATATACAGACCAATTAGAGCGATTAGCGCAAACTGGATCAGCGCCATCCAGTACTAAACCAATTTATCAAGATGTTAAAGGCTATTTACATAGCC
AATATGATAATATCAAAATATCTACCATTTGGAAAAATGAAATGAAAACAATCAAACTTAGAACCGCATACAACTATGACACTGCTGCGTCAAAT
GAGTCAGGGTTGGCTTGTGAGGAGCCAACTCTGGCTCAGCAGCATTATAAAGACGAATGCGATATAAATACTATCCTGGAACGTTTTAACGTTA
CAGGCCTATTACCTCAAAGTCCGCTGCCGCCTCAATATGGCGATTTCAGCGGAATTACTGACTATCATAGCGCCTTGAATAAGGTAATGAACGCT
ATGGAAGAATTTGATAACTTACCGGCTCAAATTCGTGCTAGGTTCGAAAACGAACCAGCAAACCTGATTGAGTTCTTGCAAGACGAGAAAATCG
ACCAGAAGCCGAGAAACTCGGCCTGGTCGAAAGAGCCATTTCGGAAGAAATGGCGATAAGCACAGTTACTCCACTTGATGTAACTGTGCTAGGT
GACACCAACACCAAAAATCTGATAAACGAGGCCAAAATTATGCTTTATAGAAAACAAACAAACAAGCGCAAAAGCGCTAAATCGTTCCGTAGGA
ACACTTCAAAAACTAAAGCTGCAAATATGCAAAAAGCCCCGCAAAGAGGGGGCTGGCGGCTCTAATAAAGCGCCAGGCTACCTCACATGGCCT
GTTATCACCCACTGACTGCTTTTAAGTAAGCATCAGACAAACTATAAGACCGGCAAATCTTATCGCCGTGTCGCATTCAAAGAATCTGACGAGCA
TGATCGTCAGATTTCACTGCCCTGCGGCCAATGCGTTGGCTGCAGGCTAGAAAAATCACGTCAATGGGCCATGCGCTGCATGCATGAAGCCCAA
TTGCACGAAAAAAACTGCTTTATAACCCTCACATACAACAATGAAAACCTTCCACAAACTGGATCGCTTGTCAAAAGCGACTTCCAAAAGTTCCTT
AAGCGCTTCAGAAAATCCATTGCACCTGCAAAATTACGTTACTACATGGCTGGAGAATACGGCACAAGTTTCGGCAGACCTCACTTCCATGCCTG
TATCTTCGGATACGATTTTCATGATAAGAACTATTCAAAAGGACTCCCTCTGGTTCTCTCATATATACATCCGACCACCTTGCAACCCTCTGGCCAC
ATGGTTATTCCTCCATTGGAGACGTTACATTCGAGTCAGCTGCTTACGTTGCTCGATATAATGCAAAAATACAACGGCCAGATGGAAGAAAACAA
ACATATACAAAGGATGAGCATTACACATACTGTGATATAGAAACAGGGGAATTAATAAAGCTATTACCAGAATATAACAATATGAGCCTTAAAC
CAGGCATTGGTGCTGAGTGGTACAAAAAATCGTTCCGACGTATATCCCCATGACTATGTTGTAGTCAACGGAAAAAGGGTAAAACCCCCAAAAT
ACTATGACAAAAAATAAATCAGATTATCCTATGAATACGAAGAATTACTCCACAAACGTGAAACTTCTGCTAAACTCAACCACGAAGACAATACC
TATGCCAGACTTGCCGTAAAGGAAAAAGTCACAAAGGCCAAACTTCAATTATTAAACGTAACCTCACTTAGGAAATCCTCATGAAATTAGTACTC
TGTACCGTTAAAGACCGCGCAGCAGATGCGTTCGGTCGTCCAATGTTCGTCCGTTCTATCGGCGAAGCAATCCGGAGCTTTAGCGACGAAGTCA
ATCGCCAGAGCGATGACAATCAACTTTATAACCATTCCGACGATTTCGACCTATTTGAATTAGGCGAATTCGACGATAATACGGTTTGTTCCAATT
ACATGAACAACCCAAACTTGTATCCTTAGGGAAACAAGTCAAAATTACTGATAAAAACTAAGCGTAGAGTAAAAAGGGGAAACCCCTTTTTCTCA
CGCAACTAGGCCTAGGAGCTCAAAAAATGCATCGTAACAAGTCGGTAGACGTCCATCAGTTCACAATGATTCCAAAAGCGGATATCCCCCGCTCT
ACATTTGACTGTCAATCAACACATAAAACTACATTCGATGCTGGCTTCCTAGTCCCTGTACTCGTAGACGAAATGTTGCCAGGCGATACATTCCGC
TGCAACATGACCGCCTTTGCGCGATTGTCTACACCACTCTATCCGATCATGGATAACATGCATCTGGATAGCTTCTTCTTTTTGTGCCAAATAGACT
TATCTGGTCAAATTGGCAAAAATTTATGGGCAGCAGGCAAATCCTGCGGACTCGATCTCGTACGTAGTGCCCCAACAAGTAACCCCAGCTGGTG
GTTACGCTATTGGCAGCCTTCAAGATTATATGGGTCTGCCAACCGTAGGCCAAGTAGGTGCTGGTGGCACCGTAAGTCACTGTGCCTTCTGGCCA
CGTGCTTACAACCTTATTTATAACGAATGGTTTCGGGACGAAAACCTTCAAAATTCAGTAGTTGTAGATACTGGCGATGGTCCAGATAACGTAGC
CAACTACACATTATTACGACGTGGAAAACGTAAAGACTATTTCACGTCAGCATTACCTTGGCCACAAAAGGGCGCAAGCGTTACTTTACCGCTTG
GAACATCCGCCCCAATATTACGCACTAACAATGCGCCTGTTTCCAGACTGTATAACGCTGGAACAAATACATTAAACGCAACCGCCCAGGCTATT
AACGTAGGTGTTACTGGTCAAATTACTGGCGGTGCTGACGGCTTGGCAAAATCATATGATCCTAATGGCGGTTTATATGCAGATTTATCAGCTGC
AACCGCTGCAACAATTAATCAATTGCGTCAAAGCTTCCAGATTCAAAAACTTTTAGAAAGGGACGCCCGTGGCGGAACTCGATACACAGAAATT
ATCCGCAGCCATTTCGGGGTCGTTAGC

>000044F|arrow

GGTGACACCAAACCACAAAAACACGATAAACAAGGACAGAAAAAATGATGCGTCGCAGACCAGCAAATAAGCAAAAGTCCGCTAGGACTTTCC
GTAAACATGCTTCACATACAAAACACGCAAATATGCGAAACTCGCCAATGCGTGGAGGCTGGAGACTCTAATAAAGTCTTCAGGCACCTCACAT
GCCTTGTTATCACCCTCTCAAAGCATATCAATGCTTTGACAAATCAATTGTTTTCGACGAAGTTCGGAAACATGACATCGTTCGATCTTTAGACCT
GCCCTGTGGGCAGTGCGTTGGATGCCGTCTAGAACGATCAAGACAATGGGCTATTCGGTGCATGCACGAAGCCCAATTGCATAAAAACAACTCA
TTCATAACACTCACATATGACAATACACATCTCCCAAGCGATGGCTCTTTGGATCACAAAGACTTTCAATTGTTCCTTAAAAGACTTAGAAAAACT
CTCGCAAAAAGAGGACTTACAATCCGCTATTACATGGCTGGAGAATATGGTGAACTCTTCGCAAGACCCCACTTCCATGCCTGTATCTTCGGATA
CGACTTTCCTGATAAAAAATTATGGAAAAGGACTGCCTCTGGTTCTATGTTATATAGATCCGCAGAACTTGAAGCTCTCTGGCCATTTGGTTATAC
CACCATTGGAGATGTTACTTTTGAATCAGCCGCCTACGTGGCTAGATACATAATGAAAAAACAAACAGGGAAAGATGCGGAATCTCATTACAAA
CGCATACACCCTGAAACCGGCGAATATTTAGACTTAAAGCCGGAATATAATAAAATGTCTTTAAAACCGGGAATCGGTAAAGACTTTTATATAAA
ATATACTTCGGATATATACCCGCAAGACTACGTAATACTTAGAGGTAAAAAGGTCAAACCACCAAAATACTATGACAAAATGTTTAAAATTGACC

AACCTTATGAGTATGACGAATTACTTTACATGCGGGAAAATAATGCTAAATTTAATTCCGAAGACAATACACCAGAACGACTATCTGCAAAAGAA
CAAGTCACTATGGCAAAACTTCAACTATTAAAACGTAACCTTACTTAGGAAAATAATGAAACTTATCCTCGCTTCCGTAAAAGACCGTGCTGCTGA
AGCATATGCACGACCAATGTTCGTACCTTCCTCTGGAGTAGCTATACGCTCTTTTTCAGATGAAATTAATCGTTCTGATACTGAAAATCAACTCTTT
AATCACCCTGATGATTTCGATCTATATGAATTCGGAACATTTGACGATTCAACTGGGTTATTCGATTTACATGAACAACCAAAACTCCTATCATTA
GGAAAACAAGTTAAACTTAAATAAAACAACCGAGGGGAAAAGAGATTTATCTTTCCCCCGGAACAACACTAAGGAAAAACATGCACCGCAATCA
GTCAGTTAATACTCACCGCTTCGCGATGGTACCTAGAGCCGATATACCACGTAGTAAATTCGATGCTCAAAAAACACATAAAACGACTTTCGATG
CGGGCTATCTAATTCCTGTATATGTTGATGAAGTGCTCCCTGGGGACACTTTCAACTTAAAAATGACGGCATTTGCCCGTCTAGCAACGCCTTTAT
ATCCAATCATGGATAACATGATTATGGATTCTTTCTTTTTCTTTGTACCCAATCGCCTTATATGGAATAACTGGCAAAAATTTATGGGTCAACAAGA
AAATCCAACAGACTCAATATCTTATATTGTCCCAACACAAACAAGCCCAACAGATGGTTATGCCGTAGGCAGCCTTCAAGACTATATGGGCTTAC
CAACAGTAGGCCAAATTGATACTGGCCGAACTATTACGCACTGTGCCTTTTGGCCACGTGCATACAATCTTATCTGGAACGAATGGTTCCGAGAT
GAAAATTTACAAACAAGCGCAGTAGTTGATAAGGGCGATGGCCCTGATACTTCCTCAAACTATGTGCTAAAACGTCGTGGTAAAAGACATGATT
ACTTTACGTCAGCATTACCATGGCCACAAAAAGGTGCGAGTGTCACCTTACCTTTAGGTACTACGGCTCCAATTAAATGGGATACCATTTCAGGA
GACGCAACATCAAACGATAAATTTACGGTAATTCAAACAGATCCTGGAAATACGACTGCTTTAGCTAGATATGGCAACGCTTATGGTGTTAATAC
TGCTGGTGTAGTAAATAACGTTTCTAATTTATATACCGACTTATCAGAAGCAACTGCTGCAACTGTCAATCAATTAAGACAGTCATTTCAAATTCA
AAAATTACTTGAAAGGGATGCACGTGGCGGAACACGATACACAGAAATTATCCGGAGTCACTTTGGAGTTATTTCCCCAGACGCCCGTTTACAA
AGGCCTGAATACCTTGGAGGCGGTTCAACACCAATTAATGTTAATCCGATTGCTCAAACGTCGGGAACAAACGCTTCTGGAACGACTACCCCTTT
GGGCAACCTTGCTGCTATGGGTACTGCTCTCGCTCATAATCATGGATTTACTCAATCATTTACTGAGCATGGCGTTATTATTGGATTAGTATCCAT
TAGAGCAGATCTTACTTATCAACAAGGATTAGACCGTATGTGGTCTAGATCTACACGATATGACTTTTATTTCCCAGCATTTGCTACTCTAGGCGA
ACAATCTGTTTTGCAAAAAGAAATTTATGCAACAGGAGATACTGCAGCCGACAATACTGTTTTTGGATATCAAGAACGCTGGGCGGAATATCGTT
ACAAACCATCTAAAATTACTGGTTTGTTCAAATCAACATCGGCGGGCACGATCGATGGTTGGCATTTGGCTCAAAAATTTACCGCTGCGCCTACTT
TGAATAATACGTTTATTCAAGATACGCCTCCTGTATCACGTGTAGTAGCCGTTGGAGCAGCTGCAAATGGCCAACAATTCTTATTTGACTCATTTT
TTGATGTCAAAATGGCAAGACCAATGCCAATGTATTCAGTACCTGGCTTAATAGACCATTTCTAATGGGACTATTTGACGGAATTGCCGATTTAAT
CGGCCCTGCTATAGCTATAGGAGCTGCCCCTGCTACTGGGGGACTCTCCTTAGCTGCACTTGCACCTGCAGCAATAGGTGCAGCAGGACAATACT
TTGGAACACAAAGTCAAAACGCAGCGAGTGCAGAACAAGCGAGTAATCAACAGAGATTTCAAGCTGAAATGTCTGGAACATCATATCAACGAG
CAGTTGAAGATATGAAAAAAGCTGGGTTAAATCCCATGCTTGCGTATTCACAAGGCGGAGCCACAACACCAGCTGGAGCTATGGCCCAGATGCA
AAATGTTCTCGGTAATGCAACTACGTCCGGAACCCAAGCTTATCAAACGGTTGCGCAAGCAAATCAAGCTATTGCTCAATCTAAACAAATTGAAG
CTCAAACAGAACTCACAAGTAATCAAACAGATAATGTACGTGCTGATACGTTAAATAAATTGGATGAAAATCCAAATATTAGAGCTCAATATAAA
CAAATACTTGCCGATACTTTCATGAAAAATGAAATAGGCAAAACATCAAGTGCTCAAGCTGCTCAAGCTTTGGCACAATCTCGTTATTCAAACGA
GTTAACAAAACTTGCTAAATCAGGGTCAGCTCCTAGTTCTAGCAAACCAATTTATCAAGACGTAAAAAACATCGCCAAAGATGCGTATAGCGCAT
CTGGCGCAAAACGATACATCGATAACTATCGAGGTCAACCGATTCAACAAAATCGTACAAATAACCAACCACCAATGGAATGAAAATGACAAAG
ATTACAGCCCCATTTCTTCGTACTCCGTACAATTACGACACGATTGCTGCGTCAAATGAGTCAGGGCTGCATTGTGAGGATGCAACTCTGACTCA
GCAGCAATTTGCTGAAGAATGTGATATCAATAATATTATGGAAAAGTTCGGTATGACCGGACTTATTCCTCAAACTCCTTTAACGCCTCAATATGG
CGACTTTAGTGGTGTCTATGACTACCACTCTGCTCTGAACCAGATTATGGCTTCAGACAACGAATTTATGGCTTTACCAGCCAATATTCGTGAACG
ATTCGCTAATGATCCCGCGAATCTAATAGATTTTCTAGAAAACCCTGAAAATCGCAGCGAAGCTGAAAAAATGGGACTGGTAAAACCAGCCCAA
ACCGAGGTTTCAACCCCTGTTGGAACCTCGGAAGCACAGTTACCTACTTGATGTAACTGTGCTA

>000203F|arrow

GGAACTTCCAACGGCCCCTGTATCGCCTCAATACGGCGATTTTAGTGGTGTTACTGATTACCATTCTGCGTTGAATCAAATTAACGCAACTATGGA
CGATTTCATGGCTCTGCCAGCGAAATTAAGAGTCCGATTTGACCATGATCCTGTCAAATTATTGGAGTTCCTTGAGAACGACCAGAATCGTGATG
AAGCGATTCAATTGGGTCTTATTGATGGACAACCTGTGGTTGAACCCATCGTTTCTACAGAAACACCTAAGGCCGAAGGATGAAATCCTGAGGC
CAGCACAGTTACTCTACTTGATGTAACTGTGCTAGGTGACACCAAACCACTATTTTAACTACGGAGTTCATCATGTTACGAAGAAAGCCAGTAAA
CAAATATAAATCTGCAAAGTCATTTCGCAGAACTGCTAGTAAGACGAAGTCATTAATATGAGACACGCTCCCCAGCGTGGTGGCTATCGTTTGTA
ATTATGGCCTGTTATAAGCCCTTAACGGCTTATATCATGCAGTGACAGTCTATAATTTGGCGGGAAATACCGGGTGCGGATGTAGTCCGTACCCT
ATCATTGCCTTGTGGTCAGTGTTTGGTTGTCGCCTTGAACGCTCACGTCAGTGGGCGATTCGTTGTATGCATGAGGCACAAATGCATACTAGTAA
TTGTTTTATTACTTTGACATATGCTCCAGAGCATTGTCCTAAGGATATGTCATTGGATTACAATGATTATCAGCTTTTTATGAAGCGGTTACGTAAG
CGTTTTACTGGAAAACGATACGTTTTTATATGGCAGGTGAATATGGTGAATCTTTTGATCGTCCTCATTTCCATGCTTGTCTGTTTGGTCTTGATTT
TCCGGATAAGAAAATATTTAAAAGAACGCAGACTGGCTCTATCCTCTACACGTCAGAGATTTTGGAAGAATTGTGGCCGTTTGGCTATTCTACAA
TTGGTGATGTTACTTTTGAGTCTGCTGCTTATGTTGCAAGATATATTATGAAGAATTAATGGGGTTACTGTCAAAACCACGAAGTGGTTGATGCG
GGTGCCCATTATCAATATTGTGATTTAGAGACTGGTGAGATAATTCAGCGTAAGCCAGAATTTAATAAGATGTCATTGAAGCCCGGTATCGGGC
AATCGTGGTTAGATAAGTACATGTCAGACGTTTATACGTCAGACCACGTTTGGTGCGTGGCAAAAAGTGCCGTCCACCACGGTTTTATGATAATA
AATTTAAGTTGAAGTTTCCTGAAGAATTCGATATGATTCAGTTTGCCAGAGAGATGGAAGGTCGATCTAGGCATGAGGACAACACGCTTGAGCG
ACTTGCTGTTAAGGAAAAAGTTGCGTTGGCTAAGTTGTCATTGTTAAAACGTACTATTTAAGGAGTTTTTATGAAGATGGTTATTGTTTCTATTAA

GGATACTGCTGCAGATGCTTTTGGTCGTCCAGCTTATGTTGCATCTGAAGGTGTTGCAGTACGTCAGTTTCAGGATGAAGTCAATCGAGCTAGCG
AAGATAATCAGTTGTATAAACATCCTGATGATTTTCATATGTTCTATTTGGGTCTTTTTGACGATGCCACTGGTGTTTTTGAACTACTGGAAAGCCC
TAAGTTGATTGCTCGTGCAAAAGATGTAATGATTCGCGAAGGCGAGTAAGGTTTTTTTTATACCGTATCACTCGAAAGAGTGGTACGGAACTAC
GGAGATGTTTATGTTTCGCAATAAGTCAGTAAGTACGCATTCATTTGCTATGGTTCCTAAAGCGGACATTCCCCGCTCTAGTTTTAATACTCATAT
GCTCATAAAACGTTTGATGCTGGTTTTTTAGTTCCTATTTATTGTGATGAAGTATTGCCTGGCGATACTCATCGTGTAAAGATGACTGCATTTGCA
CGTTTGGCCACACCGTTATTTCCTGTGATGGACAACTTGCATCTTGATACTTTCTTTTTCTTTGTACCTAATCGTTTACTTTGGAACAATTGGCCAAA
GTTTATGGGTGAACAAACGAATCCTGGTGATTCTATTTCTTTTGTAGTGCCTACTATTACTAGTCCTGCTGGTGGTTATGCTGTTTGTTCAATTTTT
GATTATTTTGGTTTACCTACTGCTGGTCAGATTACTGGCGCTAATACAGTAACGCATAATGTTTTGCCGTTACGTGCTTATAATGAGATTTATAAC
GAATGGTTTAGAGATGAAAACTTACAGAATTCTGTAACGTTAAATCTTGGTGATTCAGGTGATGTTCCTGCTAACTATACACTTTTGAGACGTGG
TAAGCGTAAAGATTATTTTACTGGTGCATTGCCTTGGCCACAGAAGGGTGCTTCTGTTTCTTTACCGTTAGGAACACGTGCTAATATTTATTCTGA
CATACCAGCTGGCAATGGTACTGCTGGTTATAGTGTTTTTCAAACTGCTGTTGGTGCTTTAAGAGAATTAAATTCAGCTTCTAATACTTTGTCTAAT
AGTACAAATGCTGGTGTTGCTACTAATCAGTTATACGCTGATTTGTCTACTGCTACTGCTGCGACTATTAACCAACTTCGTCAATCTTTCCAGATTC
AGAAGTTATTGGAGCGCGATGCACGTGGTGGTACTCGTTATACTGAGTTACTACGTGCTCACTTTGGAGTAACTCCACAGGATTATCGTTTACAA
CGTCCTGAATATATTGGTGGAGGTTCGACCCTTGTTAATGTTAATCCGATTGCTCAGACTTCTGCAACGTCGGTTACTGGTTCTGCTACTCCGCAA
GGTAACCTTGCTGCAATGGGTACTGCATTGGCTCAGGGACACGGCTTTACGTATGCTGCTCAAGAACATGGATACATTATCGGATTAGTTTCTGT
ACGTGCTGACCTCACATATCAACAGGGTCTTCCTAAGATGTGGTCTAGGTCTACACGATATGACTTTTATTTCCCAGTATTTGCCACTTTGGGTGA
GCAAGCTATTTTGAACAAAGAAATTTATGTTCAAGGTACTGCAGCCGACAATGATGTATTTGGTTATCAAGAACGTTGGGCGGAGTATCGTTACA
AACCTTCTCAAATTACTGGTTTCTTTAGGTCTACTTCTGCTGGCACTATTGATGCTTGGCATTATGGACAGCGATTTACTTCTCTTCCTACGTTGAAT
TCAACGTTTATTCAAGAGACCCCTCCAGTTGCTCGTACTACGGCGGTCGGAGCTGCAGCAAATGGTCAGCAATTTTTAATGGATGCTTTCTTTGAT
TGTCAGATGGCCAGACCTATGCCTATGTACAGCGTACCTGGTCTAATTGATCATTTCTAATGTTTTATATAACCTCGACTACTCCGTAAGGTAGTG
AGGAAACAACCGAAGGGCGTTAGTTTATGTTTGGTGGAATACTTGATGCGGTTACTAATGTTGGTTCTAAGCTGTCTTCAGCTTCTAGTTTCTTTA
CTCCTGGTGTCGGTACTGCTTTGGGCGCTGTTGGTTCTTATTTAGGTTCTACTTCTGCTAATAAAGCTAATCAGGAGATGGCTCAGAGGCAAATG
GATTTTCAAGCCGATATGAGTGGAACAAGTTACCAGCGTGCTGTTAAAGATTTAGAAGCTGCTGGTTTATCTCCTATGTTAGCCTATCAACGTGG
TGGTGCTTCTACCCCATCTGGTTCAACTGCTACTATGGAAAATGTTTTAGGTAATGCAACTAATTCAGCTATTAATACTGCTTCTATGATGCAACA
GATTCGTAATGCATCAGAAACAGAAAAGCATTATCGCCCAGACTGAAGCTACTGAAGCTGGTACCGCTAATACTAGGGCTGATACTGTTAATAA
GTTGCTTACTGCTCCTAATATTACAGCCGAAAATAAACGTATTTTGGCTGATATTGCTTTAAAGAATACGACTGCGGATTTAACATCCGCTCAGTC
ATATAATACTAAGAGGCTATTGGCTCCATCCCCAGCTATTTGGTCTAGGGTATCGATGCTTCGAAAGAAATTTTTGATAAACTCAAAAATAATCCT
AATCAACTAACCCCTTGGGGAATTGGAGTCAAATAATGAGTAAAGCGAATTTGCCATTTGTACGTAATCCGTACAACTATGATAAAGATGAAGCA
TCGGTAAACGATGCGTTGCTGTGTCAAGACCCAAGTCTTGCTCAACAGCATATGAAAGATGAATGTGACATTAATGTCATCATTGAACGTTTCGG
GGTTACAG

>000020F|arrow

AGGCATGTGAGGTGCCTGGGGACTTTATTAGAGTCTCCAGCCTCCACGCATGGCGAGTTTCGCATATTTGCGTGTTTTGTATGTGAAGCATGTTT
ACGGAAAGTCCTAGCGGACTTTTGCTTATTTGCTGGTCTGCGACGCATCATTTTTTCTGTCCTTGTTTATCGTGTTTTTGTGGTTTGGTGTCACCTA
GCACAGTTACATCAAGTAGGTAACTGTGCTTCCGAGGTTCCAACAGGGGTTGAAACCTCGGTTGGGCTGGTTTTACCAGTCCCATTTTTTCAGCT
TCGCTGCGATTTTCAGGGTTTTCTAGAAAATCTATTAGATTCGCGGGATCATTAGCGAATCGTTCACGAATATTGGCTGGTAAAGCCATAAATTC
GTTGTCTGAAGCCATAATCTGGTTCAGAGCAGAGTGGTAGTCATAGACACCACTAAAGTCGCCATATTGAGGCGTTAAGGAGTTTGAGGAATAA
GTCCGGTCATACCGAACTTTTCCATAATATTATTGATATCACATTCTTCAGCAAATTGCTGCTGAGTCAGAGTTGCATCCTCACAATGCAGCCCTG
ACTCATTTGACGCAGCAATCGTGTCGTAATTGTACGGAGTACGAAGAAATGGGGCTGTAATCTTTGTCATTTTCATTCCATTGGTGGTTGGTTATT
TGTACGATTTTGTTGAATCGGTTGACCTCGATAGTTATCGATGTATCGTTTTGCGCCAGATGCGCTATACGCATCTTTGGCGATGTTTTTACGTCTT
GATAAATTGGTTTGCTAGAACTAGGAGCTGACCCTGATTTAGCAAGTTTTGTTAACTCGTTTGAATAACGAGATTGTGCCAAAGCTTGAGCAGCT
TGAGCACTTGATGTTTTGCCTATTTCATTTTTCATGAAAGTATCGGCAAGTATTTGTTTATATTGAGCTCTAATATTTGGATTTTCATCCAATTTATT
TAACGTATCAGCACGTACATTATCTGTTTGATTACTTGTAGTTCTGTTTGAGCTTCAATTTGTTTAGATTGAGCAATAGCTTGATTTGCTTGCGCAA
CCGTTTGATAAGCTTGGGTTCCGGACGTAGTTGCATTACCGAGAACATTTGCATCTGGGCCATAGCTCCAGCTGGTGTTGTGGCTCCGCCTTGTG
AATACGCAAGCATGGGATTTAACCCAGCTTTTTTCATATCTTCAACTGCTCGTTGATATGATGTTCCAGACATTTCAGCTTGAAATCTCTGTTGATT
ACTCGCTTGTTCTGCACTCGCTGCGTTTTGACTTTGTGTTCCAAAGTATTGTCCTGCTGCACCTATTGCTGCAGGTGCAAGTGCAGCTAAGGAGAG
TCCCCCAGTAGCAGGGGCAGCTCCTATAGCTATAGCAGGGCCGATTAAATCGGCAATTCCGTCAAATAGTCCCATTAGAAATGGTCTATTAAGCC
AGGTACTGAATACATTGGCATTGGTCTTGCCATTTTGACATCAAAAAATGAGTCAAATAAGAATTGTTGGCCATTTGCAGCTGCTCCAACGGCTA
CTACACGTGATACAGGAGGCGTATCTTGAATAAACGTATTATTCAAAGTAGGCGCAGCGGTAAATTTTTGAGCCAAATGCCAACCATCGATCGT
GCCCGCCGATGTTGATTTGAACAAACCAGTAATTTTAGATGGTTTGTAACGATATTCCGCCCAGCGTTCTTGATATCCAAAAACAGTATTGTCGGC
TGCAGTATCTCCTGTTGCATAAATTTCTTTTTGCAAAACAGATTGTTCGCCTAGAGTAGCAAATGCTGGGAAATAAAAGTCATATCGTGTAGATCT
AGACCACATACGGTCTAATCCTTGTTGATAAGTAAGATCTGCTCTAATGGATACTAATCCAATAATAACGCCATGCTCAGTAAATGATTGAGTAA

ATCCATGATTATGAGCGAGAGCAGTACCCATAGCAGCAAGGTTGCCCAAAGGGGTAGTCGTTCCAGAAGCGTTTGTTCCCGACGTTTGAGCAAT
CGGATTAACATTAATTGGTGTTGAACCGCCTCCAAGGTATTCAGGCCTTTGTAAACGGGCGTCTGGGGAAATAACTCCAAAGTGACTCCGGATA
ATTTCTGTGTATCGTGTTCCGCCACGTGCATCCCTTTCAAGTAATTTTTGAATTTGAAATGACTGTCTTAATTGATTGACAGTTGCAGCAGTTGCTT
CTGATAAGTCGGTATATAAATTAGAAACGTTATTTACTACACCAGCAGTATTAACACCATAAGCGTTGCCATATCTAGCTAAAGCAGTCGTATTTC
CAGGATCTGTTTGAATTACCGTAAATTTATCGTTTGATGTTGCGTCTCCTGAAATGGTATCCCATTTAATTGGAGCCGTAGTACCTAAAGGTAAGG
TGACACTCGCACCTTTTTGTGGCCATGGTAATGCTGACGTAAAGTAATCATGTCTTTTACCACGACGTTTTAGCACATAGTTTGAGGAAGTATCAG
GGCCATCGCCCTTATCAACTACTGCGCTTGTTTGTAAATTTTCATCTCGGAACCATTCGTTCCAGATAAGATTGTATGCACGTGGCCAAAAGGCAC
AGTGCGTAATAGTTCGGCCAGTATCAATTTGGCCTACTGTTGGTAAGCCCATATAGTCTTGAAGGCTGCCTACGGCATAACCATCTGTTGGGCTT
GTTTGTGTTGGGACAATATAAGATATTGAGTCTGTTGGATTTTCTTGTTGACCCATAAATTTTTGCCAGTTATTCCATATAAGGCGATTGGGTACA
AAGAAAAAGAAAGAATCCATAATCATGTTATCCATGATTGGATATAAAGGCGTTGCTAGACGGGCAAATGCCGTCATTTTTAAGTTGAAAGTGT
CCCCAGGGAGCACTTCATCAACATATACAGGAATTAATAGCCCGCATCGAAAGTCGTTTTATGTGTTTTTTGAGCATCGAATTTACTACGTGGTAT
ATCGGCTCTAGGTACCATCGCGAAGCGGTGAGTATTAACTGACTGATTGCGGTGCATGTTTTTCCTTAGTGTTGTTCCGGGGGAAAGATAAATCT
CTTTTCCCCTCGGTTGTTTTATTTAAGTTTAACTTGTTTTCCTAATGATAGGAGTTTTGGTTGTTCATGTAAATCGAATAACCCAGTTGAATCGTCAA
ATGTTCCGAATTCATATAGATCGAAGTCATCAGGGTGATTAAAAGTTGATTTTCAGTATCAGAACGATTAATTTCATCTGAAAAAGAGCGTATAG
CTACTCCAGAGGAAGGTACGAACATTGGTCGTGCATATGCTTCAGCAGCACGGTCTTTTACGGAAGCGAGGATAAGTTTCATTATTTTCCTAAGT
GAGGTTACGTTTTAATAGTTGAAGTTTTGCCATAGTGACTTGTTCTTTTGCAGATAGTCGTTCTGGTGTATTGTCTTCGGAATTAAGTTTAGCATTA
TTTTCCCGCATGTAAAGTAATTCGTCATACTCATAAGGTTGGTCAATTTTAAACATTTTGTCATAGTATTTTGGTGGTTTGACCTTTTTACCTCTAAG
TATTACGTAGTCTTGCGGGTATATATCCGAAGTATATTTTATATAAAAGTCTTTACCGATTCCCGGTTTTAAAGACATTTTATTATATTCCGGCTTT
AAGTCTAAATATTCGCCGGTTTCAGGGTGTATGCGTTTGTAATGAGATTCCGCATCTTTCCCTGTTTGTTTTTTCATTATGTATCTAGCCACGTAGG
CGGCTGATTCGAAAGTAACATCTCCAATGGTGGTATAACCAAATGGCCAGAGAGCTTCAAGTTCTGCGGATCTATATAACATAGAACCAGAGGC
AGTCCTTTTCCATAATTTTTTATCAGGAAAGTCGTATCCGAAGATACAGGCATGGAAGTGGGGTCTTGCGAAGAGTTCACCATATTCTCCAGCCA
TGTAATAGCGGATTGTAAGTCCTCTTTTTGCGAGAGTTTTTCTAAGTCTTTTAAGGAACAGTTGAAAGTCTTTGTGATCCAAAGAGCCATCGCTTG
GGAGATGTGTATTGTCATATGTGAGTGTTATGAATGAGTTGTTTTTATGCAATTGGGCTTCGTGCATGCACCGAATAGCCCATTGTCTTGATCGTT
CTAGACGGCATCCAACGCACTGCCCACAGGGCAGGTCTAAAGATCGAACGATGTCATGTTTCCGAACTTCGTCGAAAACAATTGATTTGTCAAA
GCATTGAAATGCTTTGAGAGGGTGATAACA

>000215F|arrow

ACGTCAGCATTACCATGGCCACAAAAAGGTGCGAGTGTCACCTTACCTTTAGGTACTACGGCTCCAATTAAATGGGATACCATTTCAGGAGACGC
AACATCAAACGATAAATTTACGGTAATTCAAACAGATCCTGGAAATACGACTGCTTTAGCTAGATATGGCAACGCTTATGGTGTTAATATGCTGG
TGTAGTAAATAACGTTTCTAATTTATATACCGACTTATCAGAAGCAACTGCTGCAACTGTCAATCAATTAAGACAGTCATTTCAAATTCAAAAATT
ACTTGAAAGGGATGCACGTGGCGGAACACGATACACAGAAATTATCCGGAGTCACTTTGGAGTTATTTCCCCAGACGCCCGTTTACAAAGGCCT
GAATACCTTGGAGGCGGTTCAACACCAATTAATGTTAATCCGATTGCTCAAACGTCGGGAACAAACGCTTCTGGAACGACTACCCTTTGGGCAAC
CTTGCTGCTATGGGTACTGCTCTCGCTCATAATCATGGATTTACTCAATCATTTACTGAGCATGGCGTTATTATTGGATTAGTATCCATTAGAGCA
GATCTTACTTATCAACAAGGATTAGACCGTATGTGGTCTAGATCTACACGATATGACTTTTATTTCCCAGCATTTGCTACTCTAGGCGAACAATCT
GTTTTGCAAAAAGAAATTTATGCAACAGGAGATACTGCAGCCGACAATACTGTTTTTGGATATCAAGAACGCTGGGCGGAATATCGTTACAAAC
CATCTAAAATTACTGGTTTGTTCAAATCAACATCGGCGGGCACGATCGATGGTTGGCATTTGGCTCAAAAATTTACCGCTGCGCCTACTTTGAATA
ATACGTTTATTCAAGATACGCCTCCTGTATCACGTGTAGTAGCCGTTGGAGCAGCTGCAAATGGCCAACAATTCTTATTTGACTCATTTTTTGATG
TCAAAATGGCAAGACCAATGCCAATGTATTCAGTACCTGGCTTAATAGACCATTTCTAATGGGACTATTTGACGGAATTGCCGATTTAATCGGCC
CTGCTATAGCTATAGGAGCTGCCCCTGCTACTGGGGGACTCTCCTTAGCTGCACTTGCACCTGCAGCAATAGGTGCAGCAGGACAATACTTTGGA
ACACAAAGTCAAAACGCAGCGAGTGCAGAACAAGCGAGTAATCAACAGAGATTTCAAGCTGAAATGTCTGGAACATCATATCAACGAGCAGTT
GAAGATATGAAAAAAGCTGGGTTAAATCCCATGCTTGCGTATTCACAAGGCGGAGCCACAACACCAGCTGGAGCTATGGCCCAGATGCAAAAT
GTTCTCGGTAATGCAACTACGTCCGGAACCCAAGCTTATCAAACGGTTGCGCAAGCAAATCAAGCTATTGCTCAATCTAAACAAATTGAAGCTCA
AACAGAACTCACAAGTAATCAAACAGATAATGTACGTGCTGATACGTTAAATAAATTGGATGAAAATCCAAATATTAGAGCTCAATATAAACAAA
TACTTGCCGATACTTTCATGAAAAATGAAATAGGCAAAACATCAAGTGCTCAAGCTGCTCAAGCTTTGGCACAATCTCGTTATTCAAACGAGTTA
ACAAAACTTGCTAAATCAGGGTCAGCTCCTAGTTCTAGCAAACCAATTTATCAAGACGTAAAAAACATCGCCAAAGATGCGTATAGCGCATCTGG
CGCAAAACGATACATCGATAACTATCGAGGTCAACCGATTCAACAAAATCGTACAAATAACCAACCACCAATGGAATGAAAATGACAAAGATTA
CAGCCCCATTTCTTCGTACTCCGTACAATTACGACACGATTGCTGCGTCAAATGAGTCAGGGCTGCATTGTGAGGATGCAACTCTGACTCAGCAG
CAATTTGCTGAAGAATGATATCAATAATATTATGGAAAAGTTCGGTATGACCGGACTTATTCCTCAAACTCCTTTAACGCCTCAATATGGCGACTT
TAGTGGTGTCTATGACTACCACTCTGCTCTGAACCAGATTATGGCTTCAGACAACGAATTTATGGCTTTACCAGCCAATATTCGTGAACGATTCGC
TAATGATCCCGCGAATCTAATAGATTTTCTAGAAAACCCTGAAAATCGCAGCGAAGCTGAAAAAAATGGGACTGGTAAAACCAGCCCAAACCGAG
GTTTCAACCCTGTTGGAACCTCGGAAGCACAGTTACCTACTTGATGTAACTGTGCTAGGTGACACCAAACCACAAAAACACGATAAACAAGGAC
AGAAAAAATGATGCGTCGCAGACCAGCAAATAAGCAAAAGTCCGCTAGGACTTTCCGTAAACATGCTTCACATACAAAACACGCAAATATGCGA

AACTCGCCAATGCGTGGAGGCTGGAGACTCTAATAAAGTCTTCAGGCACCTCACATGCCTTGTTATCACCCTCTCAAAGCATATCAATGCTTTGAC
AAATCAATTGTTTTCGACGAAGTTCGGAAACATGACATCGTTCGATCTTTAGACCTGCCCTGTGGGCAGTGCGTTGGATGCCGTCTAGAACGATC
AAGACAATGGGCTATTCGGTGCATGCACGAAGCCCAATTGCATAAAAAACAACTCATTCATAACACTCACATATGACAATACACATCTCCCAAGC
GATGGCTCTTTGGATCACAAAGACTTTCAATGTTCCTTAAAAGACTTAGAAAAACTCTCGCAAAAGAGGACTTACAATCCGCTATTACATGCTGG
AGAATATGGTGAACTCTTCGCAGACCCCACTTCCATGCCTGTATCTTCCGGATACGACTTTCCTGATAAAAAATTATGGAAAGGACTGCCTCTG
GTTCTATGTTATAGGATCCGCAGAACTTGAAGCTCTCTGGCCATTTGGTTTATACCACCATTGGAGATGTTACTTTTGAATCAGCCGCCTACGTGG
CTAGATACATATGAAAAAACAAACAGGGAAAGATGCGGAATCTCATTACAAACGCATACACCCTGAAACCGGCGAATATTTAGACTTAAAGCCG
GAATATAATAAAATGTCTTTAAAACCGGGACTCGGTAAAGACTTTTATATAAAATATTACTTCGGATATATACCCGCAAGACTACGTAATATTAGA
GGTAAAAAGGTCAAACCACCAAAATATATGACAAATGTTTAAAATTGACCAACCTTATGAGTATGACGAATTACTTTACATGCGGGAAAATTAAT
GCTAAATTTAATTCCGAAGACAATACACCAGAACGACTATCTGCAAAAGACAAGTCAGCTATGGCAAAACTTCAACTATTAAAACGTAACCTTAC
TTAGGAAATAATGAAACTTATCCTCGCTTCCGTAAAGACCGTGCTGCTGAAGCATATGCACGACCAATGTTCGTACCTTCCTCTGGAGTAGCTATA
CGCTCTTTTTCAGATGAAATTAATCGTTCTGATACTGAAAATCAACTCTTTAATCACCCTGATGATTTCGATCTAATAGAATTCGGAACATTTGACG
ATTCAACTGGGTTATTCGATTTACATGAAACAACCAAAACTCCTATCATTAGGAAACAAGTTAAACTTAAATAAAACAACCGAGGGGAAAAGAG
ATTTATCTTTCCCCGGAACAACACTAAGGAAAAACATGCACCGCAATCAGTCAGTTAATACTCACCGCTTCGCGATGGTACCTAGAGCCGATATA
CCACGTAGTAAATACGATGCTCAAAAACACATAAAACGACTTTCGATGCGGGCTATCTAATTCCTGTATATGTTGATGAAGTGCTCCCTGGGGAC
ACTTTCAACTTAAAAATGACGGCATTGCCCGTCTAGCAACGCCCTTTATATCCAATCATGGATAACATGATTATGGATTCTTTCTTTTTCTTTGTACC
CAATCGCCTTATATGGAATAACTGGCAAAAATTATGGGTCAACAAGAAAATCCAACAGACTCAATATCTTATATTGTCCCAACACAAACAAGCCC
AACAGATGGTTATGCCGTAGGCAGCCTTCAAGACTATATGGGCTTACCAACAGTAGGCCAAATTGATACTGGCCGAACTATTACGCCTGTGCCTT
TTGGCCACGTGCATACAATCTTATCTGGAACGAATGGTTCCGAGATGAAATTTACAAACAAGCGCAGTAGTTGATAAGGGCGATGGCCCTGATA
CTTCCTCAAACTATGTGCTAAACGTCGTGGTAAAAGACATGATTACTTT

>000194F|arrow

ACAGCTTAGAACCAACATTAGTAACCGCATCAAGTATTCCACCAAACATAAACTAACGCCCTTCGGTTGTTTCCTCACTACTCCTTACGGAGTAGT
CGAGGTTATATAAAACATTAGAAATGATCAATTAGACCAGGTACGCTGTACATAGGCATAGGTCTGGCCATCTGACAATCAAAGAAAGCATCCA
TTAAAAATTGCTGACCATTTGCTGCAGCTCCGACCGCCGTAGTACGAGCAACTGGAGGGGTCTCTTGAATAAACGTTGAATTCAACGTAGGAAG
AGAAGTAAATCGCTGTCCATAATGCCAAGCATCAATAGTGCCAGCAGAAGTAGACCTAAAGAAACCAGTAATTTGAGAAGGTTTGTAACGATAC
TCCGCCCAACGTTCTTGATAACCAAATACATCATTGTCGGCTGCAGTACCTTGAACATAAATTTCTTTGTTCAAAATAGCTTGCTCACCCAAAGTG
GCAAATACTGGGAAATAAAAGTCATATCGTGTAGACCTAGACCACATCTTAGGAAGACCCTGTTGATATGTGAGGTCAGCACGTACAGAAACTA
ATCCGATAATGTATCCATGTTCTTGAGCAGCATACGTAAAGCCGTGTCCCTGAGCCAATGCAGTACCCATTGCAGCAAGGTTACCTTGCGGAGTA
GCAGAACCAGTAACCGACGTTGCAGAAGTCTGAGCAATCGGATTAACATTAACAAGGGTCGAACCTCCACCAATATATTCAGGACGTTGTAAAC
GATAATCCTGTGGAGTTACTCCAAAGTGAGCACGTAGTAACTCAGTATAACGAGTACCACCACGTGCATCGCGCTCCAATAACTTCTGAATCTGG
AAAGATTGACGAAGTTGGTTAATAGTCGCAGCAGTAGCAGTAGACAAATCAGCGTATACTGATTAGTAGCAACACCAGCATTTGTACTATTAGA
CAAAGTATTAGAAGCTGAATTTAATTCTCTTAAAGCACCAACAGCAGTTTGAAAAACACTATAACCAGCAGTACCATTGCCAGCTGGTATGTCAG
AATAAATATTAGCACGTGTTCCTAACGGTAAAGAAACAGAAGCACCCTTCTGTGGCCAAGGCAATGCACCAGTAAAATAATCTTTACGCTTACCA
CGTCTCAAAAGTGTATAGTTAGCAGGAACATCACCTGAATCACCAAGATTTAACGTTACAGAATTCTGTAAGTTTTCATCTCTAAACCATTCGTTA
TAAATCTCATTATAAGCACGTAACGGCAAAACATTATGCGTTACTGTATTAGCGCCAGTAATCTGACCAGCAGTAGGTAAACCAAAATAATCAAA
AATTGAACAAACAGCATAACCACCAGCAGGACTAGTAATAGTAGGCACTACAAAAGAAATAGAATCACCAGGATTCGTTTGTTCACCCATAAAC
TTTGGCCAATTGTTCCAAAGTAAACGATTAGGTACAAAGAAAAAGAAAGTATCAAGATGCAAGTTGTCCATCACAGGAAATAACGGTGTGGCCA
AACGTGCAAATGCAGTCATCTTTACACGATGAGTATCGCCAGGCAATACTTCATCACAATAAATAGGAACTAAAAAACCAGCATCAAACGTGGTT
TTATGAGCATATTGAGTATTAAAACTAGAGCGGGGAATGTCCGCTTTAGGAACCATAGCAAATGAATGCGTACTTACTGACTTATTGCGAAACAT
AAACATCTCCCGTAGTTCCGTACCACTCTTTCGAGTGATACGGTATAAAAAAAACCTTACTCGCCTTCGCGAATCATTACATCTTTTGCACGAGCA
ATCAACTTAGGGCTTTCCAGTAGTTCAAAAACACCAGTGGCATCGTCAAAAAGACCCAAATAGAACATATGAAAATCATCAGGATGTTTATACAA
CTGATTATCTTCGCTAGCTCGATTGACTTCATCCTGAAACTGACGTACTGCAACACCTTCAGATGCAACATAAGCTGGACGACCAAAAGCATCTGC
AGCAGTATCCTTAATAGAAACAATAACCATCTTCATAAAAACTCCTTAAATAGTACGTTTTAACAATGACAACTTAGCCAACGCAACTTTTTCCTTA
ACAGCAAGTCGCTCAAGCGTGTTGTCCTCATGCCTAGATCGACCTTCCATCTCTCTGGCAAACTGAATCATATCGAATTCTTCAGGAAACTTCAAC
TTAAATTTATTATCATAAAACCGTGGTGGACGGCACTTTTTGCCACGCACCACAACGTGGTCTGACGTATAAACGTCTGACATGTACTTATCTAAC
CACGATTGCCCGATACCGGGCTTCAATGACATCTTATTAAATTCTGGCTTACGCTGAATTATCTCACCAGTCTCTAAATCACAATATTGATAATGG
GCACCCGCATCAACCACTTCGTGGTTTTCATTGACAGTAACCCCATTAATCTTCTTCATAATATATCTTGCAACATAAGCAGCAGACTCAAAAGTA
ACATCACCAATTGTAGAATAGCCAAACGGCCACAATTCTTCCAAAATCTCTGACGTGTAGAGGATAGAGCCAGTCTGCGTTCTTTTAAATATTTTC
TTATCCGGAAAATCAAGACCAAACAGACAAGCATGGAAATGAGGACGATCAAAAGATTCACCATATTCACCTGCCATATAAAAACGTATCGTTTT
CCCAGTAAAACGCTTACGTAACCGCTTCATAAAAAGCTGATAATCATTGTAATCCAATGACATATCCTTAGGACAATGCTCTGGAGCATATGTCAA
AGTAATAAAACAATTACTAGTATGCATTTGTGCCTCATGCATACAACGAATCGCCCACTGACGTGAGCGTTCAAGGCGACAACCAACACACTGAC

CACAAGGCAATGATAGGGTACGGACTACATCCGCACCCGGTATTTCCCGCCAAATTATAGACCTGTCACTGCATTGATAAGCCGTTAAGGGCTTA
TAACAGGCCATAATTACAAACGATAGCCACCACGCTGGGGAGCGTGTCTCATATTAATTGACTTCGTCTTACTAGCAGTTCTGCGAAATGACTTT
GCAGATTTATATTTGTTTACTGGCTTTCTTCGTAACATGATGAACTCCGTAGTTAAAATAGTGGTTTGGTGTCACCTAGCACAGTTACATCAAGTA
GAGTAACTGTGCTGGCCTCAGGATTTCATCCTTCGGCCTTAGGTGTTTCTGTAGAAACGATGGGTTCAACCACAGGTTGTCCATCAATAAGACCC
AATTGAATCGCTTCATCACGATTCTGGTCGTTCTCAAGGAACTCCAATAATTTGACAGGATCATGGTCAAATCGGACTCTTAATTTCGCTGGCAGA
GCCATGAAATCGTCCATAGTTGCGTTAATTTGATTCAACGCAGAATGGTAATCAGTAACACCACTAAAATCGCCGTATTGAGGCGATACAGGGG
CCGTTGGAAGTTCCCCTGTAACCCCGAAACGTTCAATGATGACATTAATGTCACATTCATCTTTCATATGCTGTTGAGCAAGACTTGGGTCTTGAC
ACAGCAACGCATCGTTTACCGATGCTTCATCTTTATCATAGTTGTACGGATTACGTACAAATGGCAAATTCGCTTTACTCATTATTTGACTCCAATT
CCCCAAGGGGTTAGTTGATTAGGATTATTTTTGAGTTTATCAAAAATTTCTTTCGAAGCATCGATACCCCTAGACCAAATAGCTGGGGATGGAGC
CAATAGCCTCTTAGTATTATATGACTGAGCGGATGTTAAATCCGCAGTCGTATTCTTTAAAGCAATATCAGCCAAAATACGTTTATTTTCGGCTGT
AATATTAGGAGCAGTAAGCAACTTATTAACAGTATCAGCCCTAGTATTAGCGGTACCAGCTTCAGTAGCTTCAGTCTGGGCGATAATCTGCTTTTC
TGTTTCTGATGCATTACGAATCTGTTGCATCATAGAAGCAGTATTAATAGCTGAATTAGTTGCATTACCTAAAACATTTTCCATAGTAGCAGTTGA
ACCAGATGGGGTAGAAGCACCACCACGTTGATAGGCTAACATAGGAGATAAACCAGCAGCTTCTAAATCTTTAACAGCACGCTGGTAACTTGTT
CCACTCATATCGGCTTGAAAATCCATTTGCCTCTGAGCCATCTCCTGATTAGCTTTATTAGCAGAAGTAGAACCTAAATAAGAACCAACAGCGCCC
AAAGCAGTACCGACACCAGGAGTAAAGAAACTAGAAGCTGAAG

>000143F|arrow

TCACATATCAACAGGGTCTTCCTAAGATGTGGTCTAGGTCTACACGATATGACTTTTATTTCCCAGTATTTGCCACTTTGGGTGAGCAAGCTATTG
TGAACAAGAAATTTATGTTCAAGGTACTGCAGCCGACAATGAGTGTATTTGGTTATCAAGAACGTTGGGCGGAGTATCGTTACAAACCTTCTCAA
ATTACTGGTTTCTTTAGGTCTACTTCTGCTGGCACTATTGATGCTTGGCATTATGGACAGCGATTTACTTCTCTTCCTACGTTGAATTCAACGTTTAT
TCAAGAGACCCCTCCAGTTGCTCGTACTACGGCGGTCGGAGCTGCAGCAAATGGTCAGCAATTTTTAATGGATGCTTTCTTTGATTGTCAGATGG
CCAGACCTATGCCTATGTACAGCGTACCTGGTCTAATTGATCATTTCTAATGTTTTATATAACCTCGACTACTCCGTAAGGAGTAGTGAGGAAACA
ACCGAAGGGCGTTAGTTTATGTTTGGTGGAATACTTGATGCGGTTACTAATGTTGGTTCTAAGCTGTCTTCAGCTTCTAGTTTCTTTACTCCTGGT
GTCGGTACTGCTTTGGGCGCTGTTGGTTCTTATTTAGGTTCTACTTCTGCTAATAAAGCTAATCAGGAGATGGCTCAGAGGCAAATGGATTTTCA
AGCCGATATGAGTGGAACAAGTTACCAGCGTGCTGTTAAAGATTTAGAAGCTGCTGGTTTATCTCCTATGTTAGCCTATCACGTGGTGGTGCTTC
TACCCCATCTGGTTCAACTGCTACTATGGAAAATGTTTTAGGTAATGCAACTAATTCAGCTATTAATACTGCTTCTATGATGCAACAGATTCGTAAT
GCATCAGAAACAGAAAAGCAGATTATCGCCCAGACTGAAGCTACTGAAGCTGGTACCGCTAATACTAGGGCTGATACTGTTAATAAGTTGCTTA
CTGCTCCTAATATTACAGCCGAAAATAAACGTATTTTGGCTGATATTGCTTTAAAGAATACGACTGCGGATTTAACATCCGCTCAGTCATATAATA
CTAAGAGGCTATTGGCTCCATCCCAGCTATTTGGTCTAGGGGTATCGATGCTTCGAAAGAAATTTTTGATAAACTCAAAAATAATCCTAATCAACT
AACCCCTTGGGGAATTGGAGTCAAATAATGAGTAAAGCGAATTTGCCATTTGTACGTAATCCGTACAACTATGATAAAGATGAAGCATCGGTAA
ACGATGCGTTGCTGTGTCAAGACCCAAGTCTTGCTCAACAGCATATGAAAGATGAATGTGACATTAATGTCATCATTGAACGTTTCGGGGTTACA
GGGGAACTTCCAACGGCCCCTGTATCGCCTCAATACGGCGATTTTAGTGGTGTTACTGTATTACCATTCTGCGTTGAAATCAAATTAACGCAACTA
TGGACGATTTCATGGCTCTGCCAGCGAAATTAAGAGTCCGATTTGACCATGATCCTGTCAAATTATTGGAGTTCCTTGAGAACGACCAGAATCGT
GATGAAGCGATTCATTGGGTCTTATTGATGGACAACCTGTGGTTGAACCCATCGTTTCTACAGAAACACCTAAGGCCGAAGGATGAAATCCTGA
GGCCAGCACAGTTACTCTACTTGATGTAACTGTGCTAGGTGACACCAAACCACTATTTTAACTACGTGAGTTCATCATGTTACGAAGAAAGCCAG
TAACAAATATAAATCTGCAAAGTCATTTCGCAGAACTGCTAGTAAGACGAAGTCAATTAATATGAGACACGCTCCCCAGCGTGGTGGCTATCGTT
TGTAATTATGGCCTGTTATAAGCCCTTAACGGCTTATCAATGCAGTGACAGGTCTATAATTTGGCGGGAAATACCGGGTGCGGATGTAGTCCGTA
CCCTATCATTGCCTTGTGGTCAGTGTGTTGGTTGTCGCCTTGAACGCTCACGTCAGTGGGCGATTCGTTGTATGCATGAGGCACAAATGCATACT
AGTAATTGTTTTAATTACTTTGACATATGCTCCAGAGCATTGTCCTAAGGATATGTCATTGGATTACAATGATTATCAGCTTTTTATGAAGCGGTTA
CGTAAGCGTTTTACTGGGAAAACGATACGTTTTTATATGGCAGGTGAATATGGTGAATCTTTTGATCGTCCTCATTTCCATGCTTGTCTGTTTGGT
CTTGATTTTCCGGATAAGAAAATATTTAAAAGAACGCAGACTGGCTCTATCCTCTACACGTCAGAGATTTTGGAAGAATTGTGGCCGTTTGGCTA
TTCTACAATTGGTGATGTTACTTTTGAGTCCTGCTGCTTATGTTGCAAGATATATTATGAAGAAGATTAATGGGGTTACTGTCAATGAAAACCACG
AAGTGGTTGATGCGGGTGCCCATTATCAATATTGTGATTTAGAGACTGGTGAGATAATTCAGCGTAAGCCAGAATTTAATAAGATGTCATTGAA
GCCCGGTATCGGGCAATCGTGGTTAGATAAGTACATGTCAGACGTTTATACGTCAGACCACGTTGTGGTGCGTGGCAAAAAGTGCCGTCCACCA
CGGTTTTATGATAATAAATTTAAGTTGAAGTTTCCTGAAGAATTCGATATGATTCAGTTTGCCAGAGAGATGGAAGGTCGATCTAGGCATGAGG
ACAACACGCTTGAGCGACTTGCTGTTAAGGAAAAAGTTGCGTTGGCTAAGTTGTCATTGTTAAAACGTACTATTTAAGGAGTTTTTATGAAGATG
GTTATTGTTTCTATTAAGGATACTGCTGCAGATGCTTTTGGTCGTCCAGCTTATGTTGCATCTGAAGGTGTTGCAGTACGTCAGTTTCAGGATGAA
GTCAATCGAGCTAGCGAAGATAATCAGTTGTATAAACATCCTGATGATTTTCATATGTTCTATTTGGGTCTTTTTGACGATGCCACTGGTGTTTTT
GAACTACTGGAAAGCCCTAAGTTGATTGCTCGTGCAAAAGATGTAATGATTCGCGAAGGCGAGTAAGGTTTTTTTATACCGTATCACTCGAAAG
AGTGGTACGGAACTACGGGAGATGTTTATGTTTCGCAATAAGTCAGTAAGTACGCATTCATTTGCTATGGTTCCTAAAGCGGACATTCCCCGCTC
TAGTTTTAATACTCAATATGCTCATAAAACCACGTTTGATGCTGGTTTTTTAGTTCCTATTTATTGTGATGAAGTATTGCCTGGCGATACTCATCGT
GTAAAGATGACTGCATTTGCACGTTTGGCCACACCGTTATTTCCTGTGATGGACAACTTGCATCTTGATACTTTCTTTTTCTTTGTACCTAATCGTTT

ACTTTGGAACAATTGGCCAAAGTTTATGGGTGAACAAACGAATCCTGGTGATTCTATTTCTTTTGTAGTGCCTACTATTACTAGTCCTGCTGGTGG
TTATGCTGTTTGTTCAATTTTTGATTATTTTGGTTTACCTACTGCTGGTCAGATTACTGGCGCTAATACAGTAACGCATAATGTTTTGCCGTTACGT
GCTTATAATGAGATTTATAACGAATGGTTTAGAGATGAAAACTTACAGAATTCTGTAACGTTAAATCTTGGTGATTCAGGTGATGTTCCTGCTAAC
TATACACTTTTGAGACGTGGTAAGCGTAAAGATTATTTTACTGGTGCATTGCCTTGGCCACAGAAGGGTGCTTCTGTTTCTTTACCGTTAGGAACA
CGTGCTAATATTTATTCTGACATACCAGCTGGCAATGGTACTGCTGGTTATAGTGTTTTTCAAACTGCTGTTGGTGCTTTAAGAGAATTAAATTCA
GCTTCTAATACTTTGTCTAATAGTACAAATGCTGGTGTTGCTACTAATCAGTTATACGCTGATTTGTCTACTGCTACTGCTGCGACTATTAACCAAC
TTCGTCAATCTTTCCAGATTCAGAAGTTATTGGAGCGCGATGCACGTGGTGGTACTCGTTATACTGAGTTACTACGTGCTCACTTTGGAGTAACTC
CACAGGATTATCGTTTACAACGTCCTGAATATATTGGTGGAGGTTCGACCCTTGTTAATGTTAATCCGATTGCTCAGACTTCTGCAACGTCGGTTA
CTGGTTCTGCTACTCCGCAAGGTAACCTTGCTGCAATGGGTACTGCATTGGCTCAGGGACACGGCTTTACGTATGCTGCTCAAGAACATGGATAC
ATTATCGGATTAGTTTCTGTACGTGCTGACC

>000126F|arrow

TTTGACATCAAAAAATGAGTCAAATAAGAATTGTTGGCCATTTGCAGCTGCTCCAACGGCTACTACACGTGATACAGGAGGCGTATCTTGAATAA
AGTATTATTCAAAGTAGGCGCAGCGGTAAATTTTTGAGCCAAATGCCAACCATCGATCGTGCCCGCCGATGTTGATTTGAACAAACCAGTAATTT
TAGATGGTTTGTAACGATATTCCGCCCAGCGTTCTTGATATCCAAAAACAGTATTGTCGGCTGCAGTATCTCCTGTTGCATAAATTTCTTTTTGCAA
AACAGATTGTTCGCCTAGAGTAGCAAATGCTGGGAAATAAAAGTCATATCGTGTAGATCTAGACCACATACGGTCTAATCCTTGTTGATAAGTAA
GATCTGCTCTAATGGATACTAATCCAATAATAACGCCATGCTCAGTAAATGATTGAGTAAATCCATGATTATGAGCGAGAGCAGTACCCATAGCA
GCAAGGTTGCCCAAAGGGGTAGTCGTTCCAGAAGCGTTTGTTCCCGACGTTTGAGCAATCGGATTAACATTAATTGGTGTTGAACCGCCTCCAA
GGTATTCAGGCCTTTGTAAACGGGCGTCTGGGGAAATAACTCCAAAGTGACTCCGGATAATTTCTGTGTATCGTGTTCCGCCACGTGCATCCCTT
TCAAGTAATTTTTGAATTTGAAATGACTGTCTTAATTGATTGACAGTTGCAGCAGTTGCTTCTGATAAGTCGGTATATAATTAGAAACGTTATTTA
CTACACCAGCAGTATTACACCATAACGCGTTGCCATATCTAGCTAAAGCAGTCGTATTTCCAGGATCTGTTTGAATTACCGTAAATTTATCGTTTG
ATGTTGCGTCTCCTGAAATGGTATCCCATTTAATTGGAGCCGTAGTACCTAAAGGTAAGGTGACACTCGCACCTTTTTGTGGCCATGGTAATGCT
GACGTAAAGTAATCATGTCTTTTACCACGACGTTTTAGCACATAGTTTGAGGAAGTATCAGGGCCATCGCCCTTATCAACTACTGCGCTTGTTTGT
AAATTTTCATCTCGGAACCATTCGTTCCATAAGATTGTATGCACGTGGCCAAAAGGCACAGTGCGTAATAGTTCGGCCAGTATCAATTTGGCCTA
CTGTTGGTAAGCCCATATAGTCTTGAAGGCTGCCTACGGCATAACCATCTGTTGGGCTTGTTTGTGTTGGGACAATATAAGATATTGAGTCTGTT
GGATTTTCTTGTTGACCCATAAATTTTTGCCAGTTATTCCATATAAGGCGATTGGGTACAAAGAAAAAGAAAGAATCCATAATCATGTTTCCATGA
TTGGATATAAAGGCGTTGCTAGACGGGCAAATGCCGTCATTTTTAAGTTGAAAGTGTCCCCAGGGAGCACTTCATCAACATATACAGGAATTAA
AGCCCGCATCGAAAGTCGTTTTATGTGTTTTTTGAGCATCGAATTTACTACGTGGTATATCGGCTCTAGGTACCATCGCGAAGCGGTGAGTATTA
ACTGACTGATTGCGGTGCATGTTTTTCCTTAGTGTTGTTCCGGGGGAAAGATAAATCTCTTTTCCCCTCGGTTGTTTTATTTAAGTTTAACTTGTTT
TCCTAATGATAGGAGTTTTGGTTGTTCATGTAAATCGAATAACCCAGTTGAATCGTCAAATGTTCCGAATTCATATAGATCGAAGTCATCAGGGT
GATTAAAAGTTGATTTTCAGTATCAGAACGATTAATTTCATCTGAAAAAGAGCGTATAGCTACTCCAGAGGAAGGTACGAACATTGTCGTGCATA
TGCTTCAGCAGCACGGTCTTTTACGGAAGCGAGGATAAGTTTCATTATTTTCCTAAGTAGGTTACGTTTTAATAGTTGAAGTTTTGCCATAGTGAC
TTGTTCTTTTGCAGATAGTCGTTCTGGTGTATTGTCTTCGGAATTAAGTTTAGCATTATTTTCCCGCATGTAAAGTAATTCGTCATACTCATAAGGT
TGGTCAATTTTAAACATTTTGTCATAGTATTTTGGTGGTTTGACCTTTTTACCTCTAAGTATTACGTATCTTGCGGGTATATATCCGAAGTATATTTT
ATATAAAAGTCTTTACCGATTCCCGGTTTTAAAGACATTTTATTATATTCCGGCTTTAAGTCTAAATATTCGCCGGTTTCAGGGTGTATGCGTTTGT
AATGAGATTCCGCATCTTTCCCTGTTTGTTTTTTCATTATGTATCTAGCCACGTAGGCGGCTGATTCGAAAGTAACATCTCCAATGGTGGTATAAC
CAAATGGCCAGAGAGCTTCAAGTTCTGCGGATCTATATAACATAGAACCAGAGGCAGTCCTTTTCCATAATTTTTTATCAGGAAAGTCGTATCCG
AAGATACAGGCATGGAAGTGGGGTCTTGCGAAGAGTTCACCATATTCTCCAGCCATGTAATAGCGGATTGTAAGTCCTCTTTTTGCGAGAGTTTT
TCTAAGTCTTTTAAGGAACAGTTGAAAGTCTTTGTGATCCAAAGAGCCATCGCTTGGGAGATGTGTATTGTCATATGTGAGTGTTATGAATGAGT
TGTTTTTATGCAATTGGGCTTCGTGCATGCACCGAATAGCCCATTGTCTTGATCGTTCTAGACGGCATCCAACGCACTGCCCACAGGGCAGGTCT
AAAGATCGAACGATGTCATGTTTCCGAACTTCGTCGAAAACAATTGATTTGTCAAAGCATTGAATGCTTTGAGAGGGTGATAACAAGGCATGTG
AGGTGCCTGGGACTTTATTAGAGTCTCCAGCCTCCACGCATTGGCGAGTTTCGCATATTTGCGTGTTTTGTATGTGAAGCATGTTTACGGAAAGT
CCTAGCGGACTTTTGCTTATTTGCTGGTCTGCGACGCATCATTTTTTCTGTCCTTGTTTATCGTGTTTTTGTGGTTTGGTGTCACCTAGCACAGTTAC
ATCAAGTAGGTAACTGTGCTTCCGAGGTTCCAACAGGGGTTGAAACCTCGGTTTGGGCTGGTTTTACCAGTCCCATTTTTTCAGCTTCGCTGCGAT
TTTCAGGGTTTTCTAGAAAATCTATTAGATTCGCGGGATCATTAGCGAATCGTTCACGAATATTGGCTGGTAAAGCCATAAATTCGTTGTCTGAA
GCCATAATCTGGTTCAGAGCAGAGTGGTAGTCATAGACACCACTAAAGTCGCCATATTGAGGCGTTAAAGGAGTTTGAGGAATAAGTCCGGTCA
TACCGAACTTTTCCATAATATTATTAATATCGCATTCTTCAGCAAATTGCTGCTGAGTCAGAGTTGCATCCTCACAATGCAGCCCTGACTCATTTGA
CGCAGCAATCGTGTCGTAATTGTACGGAGTACGAAGAAATGGGGCTGTAATCTTTGTCATTTTCATTCCATTGGTGGTTGGTTATTTGTACGATTT
TTTGAATCGGTTGACCTCGATAGTTATCGATGTATCGTTTTGCGCCAGATGCGCTATACGCATCTTTGGCGATGTTTTTTACGTCTTGATAAATTG
GTTTGCTAGAACTAGGAGCTGACCCTGATTTAGCAAGTTTTGTTAACTCGTTTGAATAACGAGATTGTGCCAAAGCTTGAGCAGCTTGAGCACTT
GATGTTTTGCCTATTTCATTTTTCATGAAAGTATCGGCAAGTATTTGTTTATATTGAGCTCTAATATTTGGATTTTCATCCAATTTGTTTAACGTATC
AGCACGTACATTATCTGTTTGATTACTTGTGAGTTCTGTTTGAGCTTCAATTTGTTTAGATTGAGCAATAGCTTGATTTGCTTGCGCAACCGTTTGA

TAAGCTTGGGTTCCGGACGTAGTTGCATTACCGAGAACATTTTGCATCTGGGCCATAGCTCCAGCTGGTGTTGTGGCTCCGCCTTGTGAATACGC
AAGCATGGGATTTAACCCAGCTTTTTCATATCTTCAACTGCTCGTTGATATGATGTTCCAGACATTTCAGCTTGAAATCTCTGTTGATTACTCGCT
TGTTCTGCACTCGCTGCGTTTTGACTTTGTGTTCCAAAGTATTGTCCTGCTGCACCTATTGCTGCAGGTGCAAGTGCAGCTAAGGAGAGTCCCCCA
GTAGCAGGGCAGCTCCTATAGCTATAGCAGGGCCGATTAAATCGGCAATTCCGTCAAATAGTCCCATTAGAAATGGTCTATTAAGCCAGGTACT
GAATACATTGGCATTGGTCTTGCCAT

>000009F|arrow

GTGAATCTTTTGATCGTCATTTCCATGCTTGTCTGTTTGGTCTTGATTTCCGGATAAGAAAATATTAAAAGAACGCAGACTGGCTCTATCCTCTACA
CGTCAGAGATTTTGGAAGAATTGTGGCCGTTTGGCTATTCTACAATTGGTGATGTTACTTTTGAGTCTGCTGCTTATGTTGCAAGATATATTATGA
AGAAGATTAATGGGGTTACTGTCAATGAAAACCACGAAGTGGTTGATGCGGGTGCCCATTATCAATATTGTGATTTAGAGACTGGTGAGATAAT
TCAGCGTAAGCCAGAATTTAATAAGATGTCATTGAAGCCCGGTATCGGGCAATCGTGGTTAGATAAGTACATGTCAGACGTTTATACGTCAGAC
CACGTTGTGGTGCGTGGCAAAAAGTGCCGTCCACCACGGTTTTATGATAATAAATTTAAGTTGAAGTTTCCTGAAGAATTCGATATGATTCAGTT
TGCCAGAGAGATGGAAGGTCGATCTAGGCATGAGGACAACACGCTTGAGCGACTTGCTGTTAAGGAAAAAGTTGCGTTGGCTAAGTTGTCATT
GTTAAAACGTACTATTTAAGGAGTTTTTATGAAGATGGTTATTGTTTCTATTAAGGATACTGCTGCAGATGCTTTTGGTCGTCCAGCTTATGTTGC
ATCTGAAGGTGTTGCAGTACGTCAGTTTCAGGATGAAGTCAATCGAGCTAGCGAAGATAATCAGTTGTATAAACATCCTGATGATTTTCATATGT
TCTATTTGGGTCTTTTTGACGATGCCACTGGTGTTTTGAACTACTGGAAAGCCCTAAGTTGATTGCTCGTGCAAAAGATGTAATGATTCGCGAAG
GCGAGTAAGGTTTTTTTATACCGTATCACTCGAAAGAGTGGTACGGAACTACGGGAGATGTTTATGTTTCGCAATAAGTCAGTACGCATTCATT
TGCTATGGTTCCTAAAGCGGACATTCCCCGCTCTAGTTTTAATACTCAATATGCTCATAAAACCACGTTTGATGCTGGTTTTTTAGTTCCTATTTATT
GTGATGAAGTATTGCCTGGCGATACTCATCGTGTAAAGATGACTGCATTTGCACGTTTGGCCACACCGTTATTTCCTGTGATGGACAACTTGCAT
CTTGATATTCTTTTTCTTTGTACCTAATCGTTTACTTTGGAACAATTGGCCAAAGTTTATGGGTGAACAAACGAATCCTGGTGATTCTATTTTTTGT
AGTGCTACTATTACTAGTCCTGCTGGTGGTTATGCTGTTGTTCAATTTTTGATTATTTTGGTTTACCTACTGCTGGTCAGATTACTGGCGCTAATAC
AGTAACGCATAATGTTTTGCCGTTACGTGCTTATAATGAGATTTATAACGAATGGTTTAGAGATGAAAACTTACAGAATTCTGTAACGTTAAATCT
TGGTGATTCAGGTGATGTTCCTGCTAACTATACACTTTTGAGACGTGGTAAGCGTAAAGATTATTTTACTGGTGCATTGCCTTGGCCACAGAAGG
GTGCTTCTGTTTCTTTACCGTTAGGAACACGTGCTAATATTTATTCTGACATACCAGCTGGCAATGGTACTGCTGGTTATAGTGTTTTTCAAACTGC
TGTTGGTGCTTTAAGAGAATTAAATTCAGCTTCTAATACTTTGTCTAATAGTACAAATGCTGGTGTTGCTACTAATCAGTTATACGCTGATTTGTCT
ACTGCTACTGCTGCGACTATTAACCAACTTCGTCAATCTTTCCAGATTCAGAAGTTATTGGAGCGCGATGCACGTGGTGGTACTCGTTATACTGAG
TTACTACGTGCTCACTTTGGAGTAACTCCACAGGATTATCGTTTACAACGTCCTGAATATATTGGTGGAGGTTCGACCCTTGTTAATGTTAATCCG
ATTGCTCAGACTTCTGCAACGTCGGTTACTGGTTCTGCTACTCCGCAAGGTAACCTTGCTGCAATGGGTACTGCATTGGCTCAGGGACACGGCTT
TACGTATGCTGCTCAAGAACATGGATACATTATCGGATTAGTTTCTGTACGTGCTGACCTCACATATCAACAGGGTCTTCCTAAGATGTGGTCTAG
GTCTACACGATATGACTTTTATTTCCCAGTATTTGCCACTTTGGGTGAGCAAGCTATTTTGAACAAAGAAATTTATGTTCAAGGTACTGCAGCCGA
CAATGATGTATTTGGTTATCAAGAACGTTGGGCGGAGTATCGTTACAAACCTTCTCAAATTACTGGTTTCTTTAGGTCTACTTCTGCTGGCACTAT
TGATGCTTGGCATTATGGACAGCGATTTACTTCTCTTCCTACGTTGAATTCAACGTTTATTCAAGAGACCCTCCAGTTGCTCGTACTACGGCGGTC
GGAGCTGCAGCAAATGGTCAGCAATTTTTAATGGATGCTTTCTTTGATTGTCAGATGGCCAGACCTATGCCTATGTACAGCGTACCTGGTCTAAT
TGATCATTTCTAATGTTTTTATAACCTCGACTACTCCGTAAGGAGTAGTGAGGAAACAACCGAAGGGCGTTAGTTTATGTTTGGTGGAATACTTG
ATGCGGTTACTGTTGGTTCTAAGCTGTCTTCAGCTTCTAGTTTTTACTCCTGGTGTCGGTACTGCTTTGGGCGGTTGGTTCTTATTTAGGTTCTACT
TCTGCTAATAAAGCTAATCAGGAGATGGCTCAGAGGCAAATGGATTTTCAAGCCGATATGAGTGGAACAAGTTACCAGCGTGCTGTTAAAGATT
TAGAAGCTGCTGGTTTATCTCCTATGTTAGCCTATCAACGTGGTGGTGCTTCTACCCCATCTGGTTCAACTGCTACTATGGAAAATGTTTTAGGTA
ATGCAACTAATTCAGCTATTAATACTGCTTCTATGATGCAACAGATTCGTAATGCATCAGAAACAGAAAAGCAGATTATCGCCCAGACTGAAGCT
ACTGAAGCTGGTACCGCTAATACTAGGGCTGATACTGTTAATAAGTTGCTTACTGCTCCTAATATTACAGCCGAAAATAAACGTATTTTGGCTGAT
ATTGCTTTAAAGAATACGACTGCGGATTTAACATCCGCTCAGTCATATAATACTAAGAGGCTATTGGCTCCATCCCAGCTATTTGGTCTAGGGGTA
TCGATGCTTCGAAAGAAATTTTGATAAACTCAAAAATAATCCTAATCAACTAACCCTTGGGGAATTGGAGTCAAATAATGAGTAAAGCGAATTTG
CCATTTGTACGTAATCCGTACAACTATGATAAAGATGAAGCATCGGTAAACGATGCGTTGCTGTGTCAAGACCCAAGTCTGCTCAACAGCATATG
AAAGATGAATGTGACATTAATGTCATCATTGAACGTTTCGGGGTTACAGGGGAACTTCCAACGGCCCCTGTATCGCCTCAATACGGCGATTTTAG
TGGTGTTACTGATTACCATTCTGCGTTGAATCAAATTAACGCAACTATGGACGATTTCATGGCTCTGCCAGCGAAATTAAGAGTCCGATTTGACCA
TGATCCTGTCAAATTATTGGAGTTCCTTGAGAACGACCAGAATCGTGATGAAGCGATTCAATTGGGTCTTATGATGGACAACCTGTGGTTGAACC
CATCGTTTCTACAGAAACACCTAAGGCCGAAGGATGAAATCCTGAGGCCAGCACAGTTACTCTACTTGATGTAACTGTGCTAGGTGACACCAAAC
CACTATTTTAACTACGGAGTTCATCATGTTACGAAGAAAGCCAGTAAACAAATATAAATCTGCAAAGTCATTTCGCAGAACTGCTAGTAAGACGA
AGTCAATTAATATGAGACACGCTCCCAGCGTGGTGGCTATCGTTTGTAATTATGGCCTGTTATAAGCCCTTAACGGCTTATCAATGCAGTGACAG
GTCTATAATTTGGCGGGAAATACCGGGTGCGGATGTAGTCCGTACCCTATCATTGCCTTGTGGTCAGTGTGTTGGTTGTCGCCTTGAACGCTCAC
GTCAGTGGGCGATTCGTTGTATGCATGAGGCACAAATGCATACTAGTAATTTTATTACTTTGACATATGCTCCAGAGCATTGTCTAAGGATATGTC
ATTGGATTACAATGATTATCAGCTTTTTATGAAGCGGTTACGTAAGCGTTTTACTGGGAAAACGATACGTTTTTATATGGCAGGTGAATATG

>000228F|arrow

AATCTGGAAAGATTGACGAAGTTGGTTAATAGTCGCAGCAGTAGCAGTAGACAAATCAGCGTATAACTGATTAGTAGCAACACCAGCATTTGTA
CTATTAGACAAAGTATTAGAAGCTGAATTTAATTCTCTTAAAGCACCAACAGCAGTTTGAAAAACACTATAACCAGCAGTACCATTGCCAGCTGG
TATGTCAGAATAAATATTAGCACGTGTTCCTAACGGTAAAGAAACAGAAGCACCCTTCTGTGGCCAAGGCAATGCACCAGTAAAATAATCTTTAC
GCTTACCACGTCTCAAAAGTGTATAGTTAGCAGGAACATCACCTGAATCACCAAGATTAACGTTACAGAATTCTGTAAGTTTTCATCTCTAAACCA
TTCGTTATAAATCTCATTATAAGCACGTAACGGCAAAACATTATGCGTTACTGTATTAGCGCCAGTAATCTGACCAGCAGTAGGTAAACCAAAAT
AATCAAAAATTGAACAAACAGCATAACCACCAGCAGGACTAGTAATAGTAGGCACTACAAAAGAAATAGAATCACCAGGATTCGTTTGTTCACC
CATAAACTTTGGCCAATTGTTCCAAAGTAAACGATTAGGTACAAAGAAAAAGAAAGTATCAAGATGCAAGTTGTCCATCACAGGAAATAACGGT
GTGGCCAAACGTGCAAATGCAGTCATCTTTACACGATGAGTATCGCCAGGCAATACTTCATCACAATAAATAGGAACTAAAAAAACCAGCATCA
AACGTGGTTTTATGAGCATATTGAGTATTAAAACTAGAGCGGGGAATGTCCGCTTTAGGAACCATAGCAAATGAATGCGTACTTACTATTGCGA
AACATAAACATCTCCGTAGTTCCGTACCACTCTTTCGAGTGACGGTATAAAAAAAACCTTACTCGCCTTCGCGAATCATTACATCTTTTGCACGAG
CAATCAACTTAGGGCTTTCCAGTAGTTCAAAAACACCAGTGGCATCGTCAAAAGACCAAATAGAACATATGAAATCATCAGGATGTTTATACAAC
TGATTATCTTCGCTAGCTCGATTGACTTCATCCTGAAACTGACGTACTGCAACACTTCAGATGCAAACATAAGCTGGACGACCAAAAGCATCTGC
AGCAGTATCCTTAATAGAAACAATAACCATCTTCAAAAAACTCCTTAAATAGTACGTTTTAACAATGACAACTTAGCCAACGCAACTTTTTCCTTAA
CAGCAAGTCGCTCAAGCGTGTTGTCCTCATGCTAGATCGACCTTCCATCTCTCTGGCAAACTGAATCATATCGAATTCTTCAGGAAATCAACTTAA
ATTTATTATCATAAAACCGTGGTGGACGGCACTTTTTGCCACGCACCACAACGTGGTCTGACGTATAAACGTCTGACATGTACTTATCTAACCACG
ATTGCCCGATACCGGGCTTCAATGACATCTTATAAATTCTGGCTTACGCTGAATTATCTCACCAGTCTCTAAATCACAATATTGATAATGGGCACC
CGCATCAACCACTTCGTGGTTTTCATTGACAGTAACCCCATTAATCTTCTTCATAATATATCTTGCAACATAAGCAGCAGACTCAAAAGTAACATCA
CCAATTGTAGAATAGCCAAACGGCCACAATTCTTCCAAAATCTCTGACGTGTAGAGGATAGAGCCAGTCTGCGTTCTTTTAAATATTTTCTTATCC
GGAAAATCAAGACCAAACAGACAAGCATGGAAATGAGGACGATCAAAAGATTCACCATATTCACCTGCCATATAAAAACGTATCGTTTTCCCAG
TAAAACGCTTACGTAACCGCTTCATAAAAAGCTGATAATCATTGTAATCCAATGACATATCCTTAGGACAATGCTCTGGAGCATATGTCAAAGTA
AAAAACAATTACTAGTATGCATTTGTGCCTCATGCATACAACGAATCGCCCACTGACGTGAGCGTTCAAGGCGACAACCAACACACTGACCACAA
GGCAATGATAGGGTACGGACTACATCCGCACCCGGTATTTCCCGCCAAATTATAGACCTGTCACTGCATTGATAAGCCGTTAAGGGCTTATAACA
GGCCATAATTACAAACGATAGCACCACGCTGGGGAGCGTGTCTCATATTAATTGACTTCGTCTTACTAGCAGTTCTGCGAAATGACTTTGCAGAT
TTATATTTGTTTACTGGCTTTCTTCGTAACATGATGAACTCCGTAGTTAAAATAGTGGTTTGGTGTCACCTAGCACAGTTACATCAAGTAGAGTAA
CTGTGCTGGCCTCAGGATTTCATCCTTCGGCCTTAGGTGTTTCTGTAGAAACGATGGGTTCAACCACAGGTTGTCCATCAATAAGACCCAATTGA
ATCGCTTCATCACGATTCTGGTCGTTCTCAAGGAACTCCAATAATTTGACAGGATCATGGTCAAATCGGACTCTTAATTTCGCTGGCAGAGCCATG
AAATCGTCCATAGTTGCGTTAATTTGATTCAACGCAGAATGGTAATCAGTAACACCACTAAAATCGCCGTATTGAGGCGATACAGGGGCCGTTG
GAAGTTCCCTGTAACCCGAAACGTTCAATGATGACATTAATGTCACATTCATCTTTCATATGCTGTTGAGCAAGACTTGGGTCTTGACACAGCAAC
GCATCGTTACCGATGCTTCATCTTTATCATAGTTGTACGGATTACGTACAAATGGCAAATTCGCTTTACTCATTATTGACTCCAATTCCCCAAGGGG
TTAGTTGATTAGGATTATTTTGAGTTTATCAAAATTTCTTTCGAAGCATCGATACCCTAGACCAAATAGCTGGGGATGGAGCCAATAGCCTCTTAG
TATTATATGACTGAGCGGATGTTAAATCCGCAGTCGTATTCTTTAAAGCAATATCAGCCAAAATACGTTATTTTCGGCTGTATTAGGAGCAGTAA
GCAACTTATTAACAGTATCAGCCTAGTATTAGCGGTACCAGCTTCAGTAGCTTCAGTCTGGGCGATAATCTGCTTTTCTGTTTCTGATGCATTACG
AATCTGTGCATCATAGAAGCAGTATTAATAGCTGAATTAGTTGCATTACCTAAAACATTTTCCATAGTAGCAGTTGAACCAGATGGGGTAGAAGC
ACCACCACGTTGATAGGCTAACATAGGAGATAAACCAGCAGCTTCTAAATCTTTAACAGCACGCTGGTAACTTGTTCCACTCATATCGGCTTGAA
AATCCATTTGCCTCTGAGCCATCTCCTGATTAGCTTTATTAGCAGAAGTAGAACCTAAATAAGAACCAACAGCGCCCAAAGCAGTACCGACACCA
GGAGTAAAGAAACTAGAAGCTGAAGACAGCTTAGAACAACATTAGTAACCGCATCAAGTATTCCACCAAACATAAACTAACGCCCTTCGGTTGT
TTCCTCTATCCTTACGGAGTAGTCGAGGTTATATAAAACATTAGAAATGATCAATTAGACCAGGTACGCTGTACATAGGCATAGGTCTGGCCATC
TGACAATCAAAGAAAGCATCCATTAAAAATTGCTGACCATTTGCTGCAGCTCCGACCGCCGTAGTACGAGCAATGGAGGGGTCTCTTGAATAAA
CGTTGAATTCAACGTAGGAAGAGAAGTAAATCGCTGTCCATAATGCCAAGCATCAATAGTGCCAGCAGAAGTAGACCTAAAGAAACCAGTAATT
TGAGAAGGTTTGTAACGATACTCCGCCCAACGTTCTTGATAACCAAATACATCATTGTCGGCTGCAGTACCTTGAACATAAATTTCTTTGTTCAAA
ATAGCTTGCTCACCCAAAGTGGCAAATACTGGGAAATAAAAGTCATATCGTGTAGACCTAGACCACATCTTAGGAAGACCTGTTGATATGTGAG
GTCAGCACGTACAGAAACTAATCCGATAATGTATCCATGTTCTTGAGCAGCATACGTAAAGCCGTGTCCCTGAGCCAATGCAGTACCCATTGCAG
CAAGGTTACCTTGCGGAGTAGCAGAACCAGTAACCGACGTTGCAGAAGTCTGAGCAATCGGATTAACATTAACAAGGGTCGAACCTCCACCAAT
ATATTCAGGACGTTGTAAACGATAATCCTGTGGAGTTACTCCAAAGTGAGCACGTAGTAACTCAGTATAACGAGTACCACCACGTGCATCGCGCT
CCAATAACTCTG

>000151F|arrow

AGCGTAGAGTAAAAAGGGGAAACCCCTTTTTCTCACGCAACTAGGCCTAGGAGCTCAAAAAAATGCATCGTAACAAGTCGGTAGACGTCCATCA
GTTCACAATGATTCCAAAAGCGGATATCCCCCGCTCTACATTTGACTGTCAATCAACACATAAAACTACATTCGATGCTGGCTTCCTAGTCCCTGT
ACTCGTAGACGAAATGTTGCCAGGCGATACATTCCGCTGCAACATGACCGCCTTTGCGCGATTGTCTACACCACTCTATCCGATCATGGATAATAT
GCATCTGGATAGCTTCTTCTTCTTTGTGCCAAATAGACTTATCTGGTCAAATTGGCAAAAATTTATGGGGCAGCAGGCAAATCCTGCGGACTCGA
TTTCGTACGTAGTGCCCCAACAAGTAACCCCAGCTGGTGGTTACGCTATGGCAGCCTTCAAGATTATATGGGTCTGCCAACCTGTAGGCCAAGTA

GGTGCTGGTGGCACCGTAAGTCACTGTGCCTTCTGGCCACGTGCTTACAACCTTATTTATAACGAATGGTTTCGGGACGAAAACCTTCAAAATTC
AGTAGTTGTAGATACTGGCGATGGTCCAGATAACGTAGCCAACTACACATTATTACGACGTGGAAAACGTAAAGACTATTTCACGTCAGCATTAC
CTTGGCCACAAAAGGGCGCAAGCGTTACTTTACCGCTTGGAACATCCGCCCCAATATTACGCACTAACAATGCGCCTGTTTCCAGACTGTATAAC
GCTGGAACAAATACATTAAACGCAACCGCCCAGGCTATTAACGTAGGTGTTACTGGTCAAATTACTGGCGGTGCTGACGGCTTGGCAAAATCAT
ATGATCCTAATGGCGGTTTATATGCAGATTTATCAGCTGCAACCGCTGCAACAATTAATCAATTGCGTCAAAGCTTCCAGATTCAAAAACTTTTAG
AAAGGGACGCCCGTGGCGGAACTCGATACACAGAAATTATCCGCAGCCATTTCGGGGTCGTTAGCCCCGATGCGCGTCTCCAACGGCCTGAATA
CATTGGAGGCGGTTCAACACACATTAATATCAATCCAATCGCCCAGACGAATGGTACCGGAGCTTCCGGGACCACTACTCCTCTCGGTACACTTG
GCGCTATGGGTACTGGGCTCGCTCACAATCATGGCTTTACTTATTCAAGCACTGAACATGGTGTAATTATCGGTCTCGTTTCAGTACGAGCCGATT
TAACATACCAACAAGGTATGCACCGCATGTGGAATCGTTCCACACGTTATGATTTCTATTTCCCTGCTTTCGCCACTTTGGGCGAACAAGCAGTAT
TAAATGAAGAAATCTACGTACGAGGCGATGCCAACGATACAGGAGTGTTTGGATACCAAGAACGTTGGGCAGAATATCGTTATATGCCAAGCC
GAATTTCCAGTCTGTTCCGTAGTACGGCAGCTGGAACAATTGACGGCTGGCATTTAGCCCAACGGTTTACAACACTTCCAACTTTGAATAACACG
TTTATTCAAGAAAATCCACCTGTCTCTCGAACCCTTGCGGTCGGAGCAGCTGCCAACGGCCAGCAAATCATTTTTGATAGCTTTTTGATATTAAA
AAAGCACGGCCAATGCCAATGTACTCTGTACCTGGCTTAATCGACCACTTCTAATGGCACTAGAAGCCGCTGCCTCAGGCGCCGCATCTGGCGCC
GCTTTTGGACCTTACGGCTCCTTAATTGGAGCCGGAATAGGTGCGGCCGCTAGTTATTTTGGTGGTCAAGAACAAAACGCTGCCAGCGCACAAC
AAGCTGCAGCAATGATGCAATTCCAAGATGGTATGCGACGTACTGCATATCAAGACGCAGTAGCGGATCTTAAGGCTGCAGGTCTTAACCCTAT
GCTGGCTTATTCACAAGGCGGAGCCAAAGTCCAGCCTGGTGCGCAAGCTCCAGTAGGAAATCCACTAGGTGAGGCTGGAAATTCAGCCCGTGA
AGCTGCCATGGCAGTCGCCAATTTTAAACAATTACAAACTCAGAATATCCTGACACAATCGCAAGCCGAAAAAACGGACGCGGATACAAATCTA
TCACGTGATCAGGCAACATATACTCGAGCAAATACAGCTCGTGAAATTGCTCAGATGCCGGGATACGGCAAATTTGGTCAGCTTCGCGATGCCC
AAATAGAGCAATTAAGGACATCAAGTGCATTACAAGCTGCACAACAGCGACAAGCGTTAAGTCAAAGTGCATATACAGACCAATTAGAGCGATT
AGCGCAAACTGGATCAGCGCCATCCAGTACTAAACCAATTTATCAAGATGTTAAAGGCTATTTACATAGCCAATATGATAAATATCAAAAATATC
TACCATTTGGAAAAATGAAATGAAAACAATCAAACTTAGAACCGCATACAACTATGACACGGATGCTGCGTCAAATGAGTCAGGGTTGGCTTGT
GAGGAGCCAACTCTGGCTCAGCAGCATTATAAAGACGAATGCGATATAAATACTATCCTGGAACGTTTTAACGTTACAGGCCTATTACCTCAAAG
TCCGCTGCCGCCTCAATATGGCGATTTCAGCGGAATTACTGACTATCATAGCGCCTTGAATAAGGTAATGAACGCTATGGAAGAATTTGATAACT
TACCGGCTCAAATTCGTGCTAGGTTCGAAAACGAACCAGCAAACCTGATTGAGTTCTTGCAAGACGAGAAAAATCGACCAGAAGCCGAGAAACT
CGGCCTGGTCGAAAGAGCCATTTCGGAAGAAATGGCGATAAGCACAGTTACTCCACTTGATGTAACTGTGCTAGGTGACACCAACACCAAAAAT
ATCTGATAAACGAGGCCAAAAATTATGCTTTATAGAAAACAAACAAACAAGCGCAAAAGCGCTAAATCGTTCCGTAGGAACACTTCAAAAACTA
AAGCTGCAAATATGCAAAAAGCCCCGCAAAGAGGGGGCTGGCGGCTCTAATAAAGCGCCAGGCTACCTCACATGGCCTGTTATCACCCACTGAC
TGCTTATTTAAGTAAGCATCAGACAAACTATAAGACCGGCAAATCTTATCGCCGTGTCGCATTCAAAGAATCTGACGAGCATGATCGTCAGATTT
CACTGCCCTGCGGCCAATGCGTTGGCTGCAGGCTAGAAAAATCACGTCAATGGGCCATGCGCTGCATGCATGAAGCCCAATTGCACGAAAAAAA
CTGCTTTATAACCCTCACATACAACAATGAAAACCTTCCACAAACTGGATCGCTTGTCAAAGCGACTTCCAAAAGTTCCTTAAGCGCTTCAGAAAA
TCCATTGCACCTGCAAAATTACGTTACTACATGGCTGGAGAATACGGCACAAGTTTCGGCAGACCTCACTTCCATGCCTGTATCTTCGGATACGAT
TTTCATGATAAGAAACTATTCAAAAGGACTCCCTCTGGTTCTCTCATATATACATCCGACCACCTTGCAACCCTCTGGCCACATGGTTATTCCTCCA
TTGGAGACGTTACATTCGAGTCAGCTGCTTACGTTGCTCGATATATTATGCAAAAATACAACGGCCAGATGGAAGAAAACAAACATATAACAAA
GGATGAGCATTACACATACTGTGATATAGAAACAGGGGAATTAATAAAGCTATTACCAGAATATAACAATATGAGCCTTAAACCAGGCATTGGT
GCTGAGTGGTACAAAAAATATCGTTCCGACGTATATCCCCATGACTACTGTTGTAGTCAACGGAAAAAGGGTAAAACCCCCAAAATACTATGACA
AAAAATATAAATCAGATTATCCATATGAATACGAAGAATTACTCCACAAACGTGAAACTTCTGCTAAACTCAACCACGAAGACAATACCTATGCC
AGACTTGCCGTAAAGGAAAAAGTCACAAAGGCCAAACTTCAATTATTAAAACGTAACCTCACTTAGGAAATCCTCATGAAATTAGTACTCTGTAC
CGTTAAAGACCGCGCAGCAGATGCGTTCGGTCGTCCAATGTTCGTCCGTTCTATCGGCGAAGCAATCCGGAGCTTTAGCGACGAAGTCAATCGC
CAGAGCGATGACAATCAACTTTATAACCATTCCGACGATTTCGACCTATTTGAATTAGGCGAATTCGACGATAATACGGGTTTGTTCCAATTACAT
GAACAACCCAAACTTGTATCCTTAGGGAAACAAGTCAAAATTACTGATAAAAACTA

>000041F|arrow

GCATGAGGACAACACGCTTGAGCGACTTGCTGTTAAGGAAAAAGTTGCGTTGGCTAAGTTGTCATTGTTAAAACGTACTATTTAAGGAGTTTTTA
TGAAGATGGTTATTGTTTCTATTAAGGATACTGCTGCAGATGCTTTTGGTCGTCCAGCTTATGTTGCATCTGAAGGTGTTGCAGTACGTCAGTTTC
AGGATGAAGTCAATCGAGCTAGCGAAGATAATCAGTTGTATAAACATCCTGATGATTTTCATATGTTCTATTTGGGTCTTTTTGACGATGCCACTG
GTGTTTTTGAACTACTGGAAAGCCCTAAGTTGATTGCTCGTGCAAAGATGTAATGATTCGCGAAGGCGAGTAAGGTTTTTTTATACCGTATCACT
CGAAAGAGTGGTACGGAACTACGGGAGATGTTTATGTTTCGCAATAAGTCAGTAAGTACGCATTCATTTGCTATGGTTCCTAAAGCGGACATTCC
CGCTCTAGTTTTAATACTCAATATGCTCATAAAACCACGTTTGATGCTGGTTTTTTAGTTCCTATTTATTGTGATGAAGTATTGCCTGGCGATACTC
ATCGTGTAAAGATGACTGCATTTGCACGTTTGGCCACACCGTTATTTCCTGTGATGGACAACTTGCATCTTGATACTTTCTTTTCTTTGTACCTAAT
CGTTTACTTTGGAACAATTGGCCAAAGTTTATGGGTGAACAAACGAATCCTGGTGATTCTATTTCTTTTGTAGTGCCTACTATTACTAGTCCTGCT
GGTGGTTATGCTGTTTGTTCAATTTTTGATTATTTTGGTTTACCTACTGCTGGTCAGATTACTGGCGCTAATACAGTAACGCATAATGTTTTGCCGT
TACGTGCTTATAATGAGATTTATAACGAATGGTTTAGAGATGAAAACTTACAGAATTCTGTAACGTTAAATCTTGGTGATTCAGGTGATGTTCCTG

CTAACTATACACTTTTGAGACGTGGTAAGCGTAAAGATTATTTTACTGGTGCATTGCCTTGGCCACAGAAGGGTGCTTCTGTTTCTTTACCGTTAG
GAACACGTGCTAATATTTATTCTGACATACCAGCTGGCAATGGTACTGCTGGTTATAGTGTTTTTCAAACTGCTGTTGGTGCTTTAAGAGAATTAA
ATTCAGCTTCTAATACTTTGTCTAATAGTACAAATGCTGGTGTTGCTACTAATCAGTTATACGCTGATTTGTCTACTGCTACTGCTGCGACTATTAA
CCAACTTCGTCAATCTTTCCAGATTCAGAAGTTATTGGAGCGCGATGCACGTGGTGGTACTCGTTATACTGAGTTACTACGTGCTCACTTTGGAGT
AACTCCACAGGATTATCGTTTACAACGTCCTGAATATATTGGTGGAGGTTCGACCCTTGTTAATGTTAATCCGATTGCTCAGACTTCTGCAACGTC
GGTTACTGGTTCTGCTACTCCGCAAGGTAACCTTGCTGCAATGGGTACTGCATTGGCTCAGGGACACGGCTTTACGTATGCTGCTCAAGAACATG
GATACATTATCGGATTAGTTTCTGTACGTGCTGACCTCACATATCAACAGGGTCTTCCTAAGATGTGGTCTAGGTCTACACGATATGACTTTTATT
TCCCAGTATTTGCCACTTTGGGTGAGCAAGCTATTTTGAACAAAGAAATTTATGTTCAAGGTACTGCAGCCGACAATGATGTATTTGGTTATCAA
GAACGTTGGGCGGAGTATCGTTACAAACCTTCTCAAATTACTGGTTTCTTTAGGTCTACTTCTGCTGGCACTATTGATGCTTGGCATTATGGACAG
CGATTTACTTCTCTTCCTACGTTGAATTCAACGTTATTCAAGAGACCCCTCCAGTTGCTCGTACTACGGCGGTCGGAGCTGCAGCAAATGGTCAGC
AATTTTTAATGGATGCTTTCTTTGATTGTCAGATGGCCAGACCTATGCCTATGTACAGCGTACCTGGTCTAATTGATCATTTCTAATGTTTTATATA
ACCTCGACTACTCCGTAAGGAGTAGTGAGGAAACAACCGAAGGGCGTTAGTTTATGTTTGGTGGAATACTTGATGCGGTTACTAATGTTGGTTCT
AAGCTGTCTTCAGCTTCTAGTTTCTTACTCCTGGTGTCGGTACTGCTTTGGGCGCTGTTGGTTCTTATTTAGGTTCTACTTCTGCTAATAAAGCTAA
TCAGGAGATGGCTCAGAGGCAAATGGATTTTCAAGCCGATATGAGTGGAACAAGTTACCAGCGTGCTGTTAAAGATTTAGAAGCTGCTGGTTTA
TCTCCTATGTTAGCCTATCAACGTGGTGGTGCTTCTACCCCATCTGGTTCAACTGCTACTATGGAAAATGTTTTAGGTAATGCACTAATTCAGCTAT
TAATACTGCTTCTATGATGCAACAGATTCGTAATGCATCAGAAACAGAAAAGCAGATTATCGCCCAGACTGAAGCTACTGAAGCTGGTACCGCTA
ATACTAGGGCTGATACTGTTAATAAGTTGCTTACTGCTCCTAATATTACAGCCGAAAATAAACGTATTTTGGCTGATATTGCTTTAAAGAATACGA
CTGCGGATTTAACATCCGCTCAGTCATATAATACTAAGAGGCTATTGGCTCCATCCCCAGCTATTTGGTCTAGGGGTATCGATGCTTCGAAAGAA
ATTTTTGATAAACTCAAAATAATCCTAATCAACTAACCCCTTGGGGAATTGGAGTCAAATAATGAGTAAAGCGAATTTGCCATTTGTACGTAATCC
GTACAACTATGATAAAGATGAAGCATCGGTAAACGATGCGTGCTGTGTCAAGACCCAAGTCTTGCTCAACAGCATATGAAAGATGAATGTGACA
TTAATGTCATCATTGAACGTTTCGGGGTTACAGGGGAACTTCCAACGGCCCCTGTATCGCCTCAATACGGCGATTTTAGTGGTGTTACTGATTACC
ATTCTGCGTTGAATCAAATAACGCAACTATGGACGATTTCATGGCTCTGCCAGCGAAATTAAGAGTCCGATTTGACCATGATCCTGTCAAATTATT
GGAGTTCCTTGAGAACGACCAGAATCGTGATGAAGCGATTCAATTGGTCTTATTGATGGACAACCCTGTGGTTGAACCCATCGTTTCTACAGAAA
CACCTAAGGCCGAAGGATGAAATCCTGAGGCCAGCACAGTTACTTCTACTTGATGTAACTGTGCTAGGTGACACCAAACCACTATTTTAACTACG
GAGTTCATCATGTTACGAAGAAAGCCAGTAAACAAATATAAATCTGCAAGTCATTTCGCAGACTGCTAGTAAGACGAAGTCAATTAATATGAGA
CACGCTCCCCAGCGGGTGGCTATCGTTTGTAATATGGCCTGTATAAGCCCTTAACGGCTTATCAAGTGCAGTGACAGGTCTATAATTTGGCGGAA
ATACGGGTGCGGCTGTAGTCGTACCCTATCATTGCCTTGTGGTCAGTGTGTTGGTTGTCGCCTTGAACGCTCCACGTCAGTGGGCGATTCGTTGT
ATGCAGAGGCACAAATGCATACTAGTAATTGTTTTATTACTTTGACATATGCTCCGAGCATTGTCCTAAGGATATGTCATTGGATTACAATGATTA
TCAGCTTTTTATGGAAGCGGTTACGTAAGGTTTTAATGGGAAAACGATACGTTTTAATGGCAGGTGAATATGGTGAATCTTTTGATCGTCCTCAT
TTCCATGCTGTCTGTTTGGTCTTGATTTTCCGGATAAGAAAATATTTAAAAGAACGCAGACTGGCTCTATCCTCTACACGTCAGAGATTTTGGAAG
AATTGTGGCCGTTTGGCTATTCTACAATTGGTGATGTTACTTTTGAGTCTGCTGCTTATGTTGCAAAGATATATTATGAAGAAGATAATGGGTTAC
TGTCAATGAAACCACGGAAGTGGTTGATGCGGGTGCCCATTATCAATATTGTGATTTAGAGACTGGTGAGATAATTCAGCGTAAGCCAGAATTT
AATAAGATGTCATTGAAGCCCGGTATCGGGCAATCGTGGTTAGATAAGTACATGTCAGACGTTTATACGTCAGACCACGTTGTGGTGCGTGGCA
AAAAGTGCCGTCCACCACGGTTTTATGATAATAAATTTAAGTTGAAGTTTCCTGAAGAATTCGATATGATTCAGTTTGCAGAGAGATGGAAGGTC
GATTAG

>000014F|arrow

GTGGTTGATGCGGGTGCCCATTATCAATATTGTGATTTAGAGACTGGTGAGATAATTCAGCGTAAGCCAGAATTTAATAAGATGTCATTGAAGCC
CGGTATCGGGCAATCGTGGTTAGATAAGTACATGTCAGACGTTTATACGTCAGACCACGTTGTGGTGCGTGGCAAAAAGTGCCGTCCACCACGG
TTTTATGATAATAAATTTAAGTTGAAGTTTCCTGAAGAATTCGATATGATTCAGTTTGCCAGAGAGATGGAAGGTCGATCTAGGCATGAGGACAA
CACGCTTGAGCGACTTGCTGTTAAGGAAAAAGTTGCGTTGGCTAAGTTGTCATTGTTAAAACGTACTATTTAAGGAGTTTTTATGAAGATGGTTA
TTGTTTCTATTAAGGATACTGCTGCAGATGCTTTTGGTCGTCCAGCTTATGTTGCATCTGAAGGTGTTGCAGTACGTCAGTTTCAGGATGAAGTCA
ATCGAGCTAGCGAAGATAATCAGTTGTATAAACATCCTGATGATTTTCATATGTTCTATTTGGGTCTTTTTGACGATGCCACTGGTGTTTTTGAACT
ACTGGAAAGCCCTAAGTTGATTGCTCGTGCAAAAGATGTAATGATTCGCGAAGGCGAGTAAGGTTTTTTTTATACCGTATCACTCGAAAGAGTG
GTACGGAACTACGGGAGATGTTTATGTTTCGCAATAAGTCAGTAAGTACGCATTCATTTGCTATGGTTCCTAAAGCGGACATTCCCCGCTCTAGTT
TTAATACTCAATATGCTCATAAAACCACGTTTGATGCTGGTTTTTTAGTTCCTATTTATTGTGATGAAGTATTGCCTGGCGATACTCATCGTGTAAA
GATGACTGCATTTGCACGTTTGGCCACACCGTTATTTCCTGTGATGGACAACTTGCATCTTGATACTTTCTTTTTCTTTGTACCTAATCGTTTACTTT
GGAACAATTGGCCAAAGTTTATGGGTGAACAAACGAATCCTGGTGATTCTATTTCTTTTGTAGTGCCTACTATTACTAGTCCTGCTGGTGGTTATG
CTGTTTGTTCAATTTTTGATTATTTTGGTTTACCTACTGCTGGTCAGATTACTGGCGCTAATACAGTAACGCATAATGTTTTGCCGTTACGTGCTTA
TAATGAGATTTATAACGAATGGTTTAGAGATGAAAACTTACAGAATTCTGTAACGTTAAATCTTGGTGATTCAGGTGATGTTCCTGCTAACTATAC
ACTTTTGAGACGTGGTAAGCGTAAAGATTATTTTACTGGTGCATTGCCTTGGCCACAGAAGGGTGCTTCTGTTTCTTTACCGTTAGGAACACGTG
CTAATATTTATTCTGACATACCAGCTGGCAATGGTACTGCTGGTTATAGTGTTTTTCAAACTGCTGTTGGTGCTTTAAGAGAATTAAATTCAGCTTC

TAATACTTTGTCTAATAGTACAAATGCTGGTGTTGCTACTAATCAGTTATACGCTGATTTGTCTACTGCTACTGCTGCGACTATTAACCAACTTCGT
CAATCTTTCCAGATTCAGAAGTTATTGGAGCGCGATGCACGTGGTGGTACTCGTTATACTGAGTTACTACGTGCTCACTTTGGAGTAACTCCACA
GGATTATCGTTTACAACGTCCTGAATATATTGGTGGAGGTTCGACCCTTGTTAATGTTAATCCGATTGCTCAGACTTCTGCAACGTCGGTTACTGG
TTCTGCTACTCCGCAAGGTAACCTTGCTGCAATGGGTACTGCATTGGCTCAGGGACACGGCTTTACGTATGCTGCTCAAGAACATGGATACATTA
TCGGATTAGTTTCTGTACGTGCTGACCTCACATATCAACAGGGTCTTCCTAAGATGTGGTCTAGGTCTACACGATATGACTTTTATTTCCCAGTATT
TGCCACTTTGGGTGAGCAAGCTATTTTGAACAAAGAAATTTATGTTCAAGGTACTGCAGCCGACAATGATGTATTTGGTTATCAAGAACGTTGGG
CGGAGTATCGTTACAAACCTTCTCAAATTACTGGTTTCTTTAGGTCTACTTCTGCTGGCACTATTGATGCTTGGCATTATGGACAGCGATTTACTTC
TCTTCCTACGTTGAATTCAACGTTTATTCAAGAGACCCCTCCAGTTGCTCGTACTACGGCGGTCGGAGCTGCAGCAAATGGTCAGCAATTTTTAAT
GGATGCTTTCTTTGATTGTCAGATGGCCAGACCTATGCCTATGTACAGCGTACCTGGTCTAATTGATCATTTCTAATGTTTTATATAACCTCGACTA
CTCCGTAAGGAGTAGTGAGGAAACAACCGAAGGGCGTTAGTTTATGTTTGGTGGAATACTTGATGCGGTTACTAATGTTGGTTCTAAGCTGTCTT
CAGCTTCTAGTTTCTTTACTCCTGGTGTCGGTACTGCTTTGGGCGCTGTTGGTTCTTATTTAGGTTCTACTTCTGCTAATAAAGCTAATCAGGAGAT
GGCTCAGAGGCAAATGGATTTTCAAGCCGATATGAGTGGAACAAGTTACCAGCGTGCTGTTAAAGATTTAGAAGCTGCTGGTTTATCTCCTATGT
TAGCCTATCAACGTGGTGGTGCTTCTACCCCATCTGGTTCAACTGCTACTATGGAAAATGTTTTAGGTAATGCAACTAATTCAGCTATTAATACTG
CTTCTATGATGCAACAGATTCGTAATGCATCAGAAACAGAAAAGCAGATTATCGCCCAGACTGAAGCTACTGAAGCTGGTACCGCTAATACTAG
GGCTGATACTGTTAATAAGTTGCTTACTGCTCCTAATATTACAGCCGAAAATAAACGTATTTTGGCTGATATTGCTTTAAAGAATACGACTGCGGA
TTTAACATCCGCTCAGTCATATAATACTAAGAGGCTATTGGCTCCATCCCCAGCTATTTGGTCTAGGGGTATCGATGCTTCGAAAGAAATTTTTGA
TAAACTCAAAAATAATCCTAATCAACTAACCCCTTGGGGAATTGGAGTCAAATAATGAGTAAAGCGAATTTGCCATTTGTACGTAATCCGTACAA
CTATGATAAAGATGAAGCATCGGTAAACGATGCGTTGCTGTGTCAAGACCCAAGTCTTGCTCAACAGCATATGAAAGATGAATGTGACATTAAT
GTCATCATTGAACGTTTCGGGGTTACAGGGGAACTTCCAACGGCCCCTGTATCGCCTCAATACGGCGATTTTAGTGGTGTTACTGATTACCATTCT
GCGTTGAATCAAATTAACGCAACTATGGACGATTTCATGGCTCTGCCAGCGAAATTAAGAGTCCGATTTGACCATGATCCTGTCAAATTATTGGA
GTTCCTTGAGAACGACCAGAATCGTGATGAAGCGATTCAATTGGGTCTTATTGATGGACAACCTGTGGTTGAACCCATCGTTTCTACAGAAACAC
CTAAGGCCGAAGGATGAAATCCTGAGGCCAGCACAGTTACTCTACTTGATGTAACTGTGCTAGGTGACACCAAACCACTATTTTAACTACGGAGT
TCATCATGTTACGAAGAAAGCCAGTAAACAAATATAAATCTGCAAAGTCATTTCGCAGAACTGCTAGTAAGACGAAGTCAATTAATATGAGACAC
GCTCCCCAGCGTGGTGGCTATCGTTTGTAATTATGGCCTGTTATAAGCCCTTAACGGCTTATCAATGCAGTGACAGGTCTATAATTTGGCGGGAA
ATACCGGGTGCGGATGTAGTCCGTACCCTATCATTGCCTTGTGGTCAGTGTGTTGGTTGTCGCCTTGAACGCTCACGTCAGTGGGCGATTCGTTG
TATGCATGAGGCACAAATGCATACTAGTAATTGTTTTATTACTTTGACATATGCTCCAGAGCATTGTCCTAAGGATATGTCATTGGATTACAATGA
TTATCAGCTTTTTATGAAGCGGTTACGTAAGCGTTTTACTGGGAAAACGATACGTTTTTATATGGCAGGTGAATATGGTGAATCTTTTGATCGTCC
TCATTTCCATGCTTGTCTGTTTGGTCTTGATTTTCCGGATAAGAAAATATTTAAAAGAACGCAGACTGGCTCTATCCTCTACACGTCAGAGATTTTG
GAAGAATTGTGGCCGTTTGGCTATTCTACAATTGGTGATGTTACTTTTGAGTCTGCTGCTTATGTTGCAAGATATATTATGAAGAAGATTAATGG
GGTTACTGTCAATGAAAACCACGAA

>000147F|arrow

CAGCGAAGCTGAAAAAATGGGACTGGTAAAACCAGCCCAAACCGAGGTTTCAACCCCTGTTGGAACCTCGGAAGCACAGTTACCTACTTGATGT
AACTGTGCTAGGTGACACCAAACCACAAAAACACGATAAACAAGGACAGAAAAAATGATGCGTCGCAGACCAGCAAATAAGCAAAAGTCCGCT
AGGACTTTCCGTAAACATGCTTCACATACAAAACACGCAAATATGCGAAACTCGCCAATGCGTGGAGGCTGGAGACTCTAATAAAGTCCCCAGG
CACCTCACATGCCTTGTTATCACCCTCTCAAAGCATTTCAATGCTTTGACAAATCAATTGTTTTCGACGAAGTTCGGAAACATGACATCGTTCGATC
TTTAGACCTGCCCTGTGGGCAGTGCGTTGGATGCCGTCTAGAACGATCAAGACAATGGGCTATTCGGTGCATGCACGAAGCCCAATTGCATAAA
AACAACTCATTCATAACACTCACATATGACAATACACATCTCCCAAGCGATGGCTCTTTGGATCACAAAGACTTTCAACTGTTCCTTAAAAGACTT
AGAAAAACTCTCGCAAAAAGAGGACTTACAATCCGCTATTACATGGCTGGAGAATATGGTGAACTCTTCGCAAGACCCCACTTCCATGCCTGTAT
CTTCGGATACGACTTTCCTGATAAAAAATTATGGAAAAGGACTGCCTCTGGTTCTATGTTATATAGATCCGCAGAACTTGAAGCTCTCTGGCCATT
TGGTTATACCACCATTGGAGATGTTACTTTCGAATCAGCCGCCTACGTGGCTAGATACATAATGAAAAAACAAACAGGGAAAGATGCGGAATCT
CATTACAAACGCATACACCCTGAAACCGGCGAATATTTAGACTTAAAGCCGGAATATAATAAAATGTCTTTAAAACCGGGAATCGGTAAAGACTT
TTATATAAAATATACTTCGGATATATACCCGCAAGACTACGTAATACTTAGAGGTAAAAAGGTCAAACCACCAAAATACTATGACAAAATGTTTA
AAATTGACCAACCTTATGAGTATGACGAATTACTTTACATGCGGGAAAATAACGCTAAACTTAATTCCGAAGACAATACACCAGAACGACTATCT
GCAAAAGAACAAGTAACTATGGCAAAACTTCAACTATTAAAACGTAACCTCACTTAGGAAAATAATGAAACTTATCCTCGCTTCCGTAAAAGACC
GTGCTGCTGAAGCATATGCACGACCAATGTTCGTACCTTCCTCTGGAGTAGCTATACGCTCTTTTTCAGATGAAATTAATCGTTCTGATACTGAAA
ATCAACTTTTAATCACCCTGATGACTTCGATCTATATGAATTCGGAACTTTTGACGATTCAACTGGGTTATTCGATTTACATGAACAACCAAAACTC
CTATCATTAGGAAAACAAGTTAAACTTAAATAAAACAACCGAGGGGAAAAGAGATTTATCTTTCCCCCGGAACAACACTAAGGAAAAACATGCA
CCGCAATCAGTCAGTTAATACTCACCGCTTCGCGATGGTACCTAGAGCCGATATACCACGTAGTAAATTCGATGCTCAAAAAACACATAAAACGA
CTTTCGATGCGGGCTATTTAATTCCTGTATATGTTGATGAAGTGCTCCCTGGGGACACTTTCAACTTAAAAATGACGGCATTTGCCCGTCTAGCAA
CGCCTTTATATCCAATCATGGACAACATGATTATGGATTCTTTCTTTTTCTTTGTACCCAATCGCCTTATATGGAATAACTGGCAAAAATTTATGGG
TCAACAAGAAAATCCAACAGACTCAATATCTTATATTGTCCCAACTCAAACAAGCCCAACAGATGGTTATGCCGTAGGCAGCCTTCAAGACTATA

TGGGCTTACCAACAGTAGGCCAAATTGATACTGGCCGAACTATTACGCACTGTGCCTTTTGGCCACGTGCATACAATCTTATTTGGAACGAATGG
TTCCGAGATGAAAATTTACAAACAAGCGCAGTAGTTGATAAGGGCGATGGCCCTGATACTTCCTCAAACTATGTGCTAAAACGTCGTGGTAAAA
GACATGATTACTTTACGTCAGCATTACCATGGCCACAAAAAGGTGCGAGTGTCACCTTACCTTTAGGTACTACGGCTCCAATTAAATGGGATACC
ATTTCAGGAGACGCAACATCAAACGATAAATTTACGGTAATTCAAACAGATCCTGGAAATACGACTGCTTTAGCTAGATATGGCAACGCTTATGG
TGTTAATACTGCTGGTGTAGTAAATAACGTTTCTAATTTATATACCGACTTATCAGAAGCAACTGCTGCAACTGTCAATCAATTAAGACAGTCATT
TCAAATTCAAAAATTACTTGAAAGGGATGCACGTGGCGGAACACGATACACAGAAATTATCCGGAGTCACTTTGGAGTTATTTCCCCAGACGCCC
GTTTACAAAGGCCTGAATACCTTGGAGGCGGTTCAACACCAATTAATGTTAATCCGATTGCTCAAACGTCGGGAACAAACGCTTCTGGAACGACT
ACCCCTTTGGGCAACCTTGCTGCTATGGGTACTGCTCTCGCTCATAATCATGGATTTACTCAATCATTTACTGAGCATGGCGTTATTATTGGATTA
GTATCCATTAGAGCAGATCTTACTTATCAACAAGGATTAGACCGTATGTGGTCTAGATCTACACGATATGACTTTTATTTCCCAGCATTTGCTACTC
TAGGCGAACAATCTGTTTTGCAAAAAGAAATTTATGCAACAGGAGATACTGCAGCCGACAATACTGTTTTTGGATATCAAGAACGCTGGGCGGA
ATATCGTTACAAACCATCTAAAATTACTGGTTTGTTCAAATCAACATCGGCGGGCACGATCGATGGTTGGCATTTGGCTCAAAAATTTACCGCTGC
GCCTACTTTGAATAATACGTTTATTCAAGATACGCCTCCTGTATCACGTGTAGTAGCCGTTGGAGCAGCTGCAAATGGCCAACAATTCTTATTTGA
CTCATTTTTTGATGTCAAAATGGCAAGACCAATGCCAATGTATTCAGTACCTGGCTTAATAGACCATTTCTAATGGGACTATTTGACGGAATTGCC
GATTTAATCGGCCCTGCTATAGCTATAGGAGCTGCCCCTGCTACTGGGGGACTCTCCTTAGCTGCACTTGCACCTGCAGCAATAGGTGCAGCAGG
ACAATACTTTGGAACACAAAGTCAAAACGCAGCGAGTGCAGAACAAGCGAGTAATCAACAGAGATTTCAAGCTGAAATGTCTGGAACATCATAT
CAACGAGCAGTTGAAGATATGAAAAAAGCTGGGTTAAATCCCATGCTTGCGTATTCACAAGGCGGAGCCACAACACCAGCTGGAGCTATGGCCC
AGATGCAAAATGTTCTCGGTAATGCAACTACGTCCGGAACCCAAGCTTATCAAACGGTTGCTCAAGCAAATCAAGCTATTGCTCAATCTAAACAA
ATTGAAGCTCAAACAGAACTCACAAGTAATCAAACAGATAATGTACGTGCTGATACGTTAAACAAATTGGATGAAAATCCAAATATTAGAGCTCA
ATATAAACAAATACTTGCCGATACTTTCATGAAAAATGAAATAGGCAAAACATCAAGTGCTCAAGCTGCTCAAGCTTTGGCACAATCTCGTTATTC
AAACGAGTTAACAAAACTTGCTAAATCAGGGTCAGCTCCTAGTTCTAGCAAACCAATTTATCAAGACGTAAAAAACATCGCCAAAGATGCGTATA
GCGCATCTGGCGCAAAACGATACATCGATAACTATCGAGGTCAACCGATTCAACAAAATCGTACAAATAACCAACCACCAATGGAATGAAAATG
ACAAAGATTACAGCCCCATTTCTTCGTACTCCGTACAATTACGACACGATTGCTGCGTCAAATGAGTCAGGGCTGCATTGTGAGGATGCAACTCT
GACTCAGCAGCAATTTGCTGAAGAATGCGATATTAATAATATTATGGAAAAGTTTGGTATGACCGGACTTATTCCTCAAACTCCTTTAACGCCTCA
ATATGGCGACTTTAGTGGTGTCTATGACTACCACTCTGCTCTGAACCAGATTATGGCTTCAGACAACGAATTTATGGCTTTACCAGCCAATATTCG
TGAACGATTCGCTAATGATCCCGCGAATCTAATAGATTTTCTAGAAAATCCTGAAAATCG

>000187F|arrow

GAGAGTAATTTACCTAATACTCAAAATATTAATTTGAAATATCATGTTGTTAATTCATGGGAAAAACGTATTTTGAAGAATGATACTGGTCGTAAT
ATTGCTTTTAGTATTTATACTTGTGCACCTAAAAGGGCAGATTTTAAAAGCGAACTAGTTTTAAGTATTGGAGTTCCCGGGGCTCAACAAAATAAT
CAAGTCGCGCTTGTTGATCCTGTTACTCATTGGGGTGAAACTTTAGCACAAGATGCTACGAATTTGGTGAATATTAGTTCTTTAAGTAACTTAACT
TTGTTTGAATCTCCGACTAGACTTGCTGCTTGGAATAAGGCTTGGAATGTTGAAATTGTCAAAGTTGTTCTTGAACCAGGGCAAACGTATGAACA
TTTTATTCAAGGTCCAAAACATATGGATGTTAATATGGCTGATTTGTTTCAATCAGGTGGTTATCAAAGTATTCAAAAATGGATGCGATATTCGTT
TTATACAGTTCATATGGATCTTTTAGGTGCCAATGTTTCTACTGGTGTGTTTGCTGGTAGACCACGATTTGGGTCCAGTCTTACTTCTGGATTTGGT
TTGAAAGTTGAAAGACTAAGATATACTCAGTTTAGTATGCCAGAGCAAGTTGGGTTCAAACTTATTGCTGCTTCAGCTGGTCCAGGTCGTTATGG
TTTGGGTTCTCGAAGACCAACATACGGTAAAGCTGTTAATGATCCGATATTTACTGGTCCAGCGCTTGTTGTCAGAATGGATGAAGAAAATCCTA
CTGTAGTTGTTGGTACTGGTCTTTAATAAACTCGCTACTACATTAATTACTGCGACGAATAGGAGCAGTTGCACGCCTCCCGCAGGGTGCACCTA
TTTTCTGAGAATCGTCATCTAAGTATTACTTACTAGATGCGGTTCTCAGTTCTCATCGGTTCTCGTTGATGATTGGTTACATTTTGATGACCAATCA
TCATCTTGCAAATCACGTGGTCTGTCATCGATTACTATAAAAAGAAAGGGAATTCTCTTAATTTTTATTAATGAATATTGAATGGGAACAACTCCA
GAAGACAGGGTATATTCTGGTTGCTTACCATTCCTGACTACTGCTTCGTCACCTACCTCCCCCCTGGCTGCACTTACATTACAGGACAACTTGAAA
CTGCTCCAACGACTGGATATGTCCACTGGCAGCTGTTGGTCGCCACAAGCAAGAAGGCTTCTTTGGCCCAAATCAAACAAATCTTTGGAGCAGA
GTGTCATGGAGAACTCTCTCGTTCCTCTGCTGCACAACAATATTGTCAGAAAGAGGAGAGTCGAATCGAAGGTACCCAGTTCGAACTTGGTAGA
TGAATTATCTTAATAAAAGGAGTCAAACCATTTCAAAGGAATTCCAAAAGAGACTGGGAGTCCGTCTGGGTTTCAGCCAAATCCGGGGATTTGG
AGTCAATACCCGCGGACATTCGAGTGGTTAGTTATCGGACAATTCGAGCAATTGGTGCCGATTATTCAAAAGCTATTCCAATGGAACGAAATGCT
TTTATCTTCTGGGGTAAAACTGGAACAGGTAAAAGCAGAAGAGCGTGGGAGGAAGCAGGTATGGAAGCTTATTGTAAAGACCCACGTACTAAG
TTTTGGTGTGGTTACCAAGATGAATCGAACGTTGTGGTTGATGAATTTCGTGGTGGAATTGACATTGCCCATTTGTTACGATGGCTTGACAGGTA
CCCAGTCCGTGTGGAGGTTAAGGGAAGTTCACGAGTTTTAAAAGCTAAAACTTTTTGGTTTACTTCCAATACAAATCCACGTGATTGGTATCCAG
ATATAGATTATATGACATTGGAAGCTTTAATGCGACGCGTTACGGTTGTTGAGTTTGCCTAATAAATGTATGGAACTCCAAGAGGTATTAGTCGC
TGTCGTCGTCGTTCTCACTTTCGTCCAAGTGGTCGTGTCCCTTTATCAGTACCTAGGTCAAGATCTGCCCGTCGCCCCGCGCGTGCTAGAGTCCGA
ACGCGTGCACGAAGACCATCGCAACATCGTCGTCGTTAGTTAACCTAATAAATGTCATTAGTTCGTTATCAACAACAAAGACATCAATCCCATGAT
AATTCTGATTTTTGGAGAGATCAGGGTTGGAAAGCATTGAAATGGGCTTCTAAGAAAGCTAAACGAAAGTTAGAAGAAATGTGGCATGAGCGA
GGTTCTAAAAAGCGACGAAATCCACTTCCGCCAACTAAACAATATCATATGCGTGGTGATGTTAGTCGTGAAAGTGGTTTTTCTACTCGTGCCCC
CGGTGTCAAAAGAATCAAAAAAGGGAAACGTAATGTTGCATCCAAGTATGTGAAAGTCTCAAAGGCTTTCAAACTTAAAGTAGATAAGGTTATT

GAAAAGGAGCAGATTCATGGTAATTATACGGAGATTAGTTACGGTTATATGGATCTTCCTGTAAATGCCAATCAACAATCTAATTCTATTGTTGTT
AATGCTGATTATACAAAGTGGTCTTTTGATCCAGAGAATTGGTTACATTTACTGGGAGTGTTGTGGAAAGGTCAAACTTATTCACAAAGCGTACA
AGATTGGCAAGGAAGTTTGTTTGGTGGTATTCAAGCTGGTTTT

>000211F|arrow

TAAACTTTGGCCAATTGTTCCAAAGTAAACGATTAGGTACAAAGAAAAAGAAAGTATCAAGATGCAAGTTGTCCATCACAGGAAATAACGGTGT
GGCCAAACGTGCAAATGCAGTCATCTTTACACGATGAGTATCGCCAGGCAATACTTCATCACAATAAATAGGAACTAAAAAACCAGCATCAAAC
GTGGTTTTATGAGCATATTGAGTATTAAAACTAGAGCGGGGAATGTCCGCTTTAGGAACCATAGCAAATGAATGCGTACTTACTGACTTATTGCG
AAACATAAACATCTCCCGTAGTTCCGTACCACTCTTTCGAGTGATACGGTATAAAAAAAACCTTACTCGCCTTCGCGAATCATTACATCTTTTGCAC
GAGCAATCAACTTAGGGCTTTCCAGTAGTTCAAAAACACCAGTGGCATCGTCAAAAAGACCCAAATAGAACATATGAAAATCATCAGGATGTTT
ATACAACTGATTATCTTCGCTAGCTCGATTGACTTCATCCTGAAACTGACGTACTGCAACACCTTCAGATGCAACATAAGCTGGACGACCAAAAG
CATCTGCAGCAGTATCCTTAATAGAAACAATAACCATCTTCATAAAAACTCCTTAAATAGTACGTTTTAACAATGACAACTTAGCCAACGCAACTT
TTTCCTTAACAGCAAGTCGCTCAAGCGTGTTGTCCTCATGCCTAGATCGACCTTCCATCTCTCTGGCAAACTGAATCATATCGAATTCTTCAGGAAC
TTCAACTTAAATTTATTATCATAAAACCGTGGTGGACGGCACTTTTTGCCACGCACACAACGTGGTCTGACGTATAAACGTCTGACATGTACTTAT
CTAACCACGATTGCCCGATACCGGGCTTCAATGACATCTTATTAAATTCTGGCTTACGCTGAATTATCTCACCAGTCTCTAAATCACAATATTGATA
ATGGGCACCCGCATCAACCACTTCGTGGTTTTCATTGACAGTAACCCCATTAATCTTCTTCATAATATATCTTGCAACATAAGCAGCAGACTCAAA
AGTAACATCACCAATTGTAGAATAGCCAAACGGCCACAATTCTTCCAAAATCTCTGACGTGTAGAGGATAGAGCCAGTCTGCGTTCTTTTAAATA
TTTTCTTATCCGGAAAATCAAGACCAAACAGACAAGCATGGAAATGAGGACGATCAAAAGATTCACCATATTCACCTGCCATATAAAAACGTATC
GTTTTCCCAGTAAAACGCTTACGTAACCGCTTCATAAAAAGCTGATAATCATTGTAATCCAATGACATATCCTTAGGACAATGCTCTGGAGCATAT
GTCAAAGTAATAAAACAATTACTAGTATGCATTTGTGCCTCATGCATACAACGAATCGCCCACTGACGTGAGCGTTCAAGGCGACAACCAACACA
CTGACCACAAGGCAATGATAGGGTACGGACTACATCCGCACCCGGTATTTCCCGCCAAATTATAGACCTGTCACTGCATTGATAAGCCGTTAAGG
GCTTATAACAGGCCATAATTACAAACGATAGCCACCACGCTGGGGAGCGTGTCTCATATTAATTGACTTCGTCTTACTAGCAGTTCTGCGAAATG
ACTTTGCAGATTTATATTTGTTTACTGGCTTTCTTCGTAACATGATGAACTCCGTAGTTAAAATAGTGGTTTGGTGTCACCTAGCACAGTTACATCA
AGTAGAGTAACTGTGCTGGCCTCAGGATTTCATCCTTCGGCCTTAGGTGTTTCTGTAGAAACGATGGGTTCAACCACAGGTTGTCCATCAATAAG
ACCCAATTGAATCGCTTCATCACGATTCTGGTCGTTCTCAAGGAACTCCAATAATTTGACAGGATCATGGTCAAATCGGACTCTTAATTTCGCTGG
CAGAGCCATGAAATCGTCCATAGTTGCGTTAATTTGATTCAACGCAGAATGGTAATCAGTAACACCACTAAAATCGCCGTATTGAGGCGATACAG
GGGCCGTTGGAAGTTCCCCTGTAACCCCGAAACGTTCAATGATGACATTAATGTCACATTCATCTTTCATATGCTGTTGAGCAAGACTTGGGTCTT
GACACAGCAACGCATCGTTTACCGATGCTTCATCTTTATCATAGTTGTACGGATTACGTACAAATGGCAAATTCGCTTTACTCATTATTTGACTCCA
ATTCCCCAAGGGGTTAGTTGATTAGGATTATTTTTGAGTTTATCAAAAATTTCTTTCGAAGCATCGATACCCCTAGACCAAATAGCTGGGGATGG
AGCCAATAGCCTCTTAGTATTATATGACTGAGCGGATGTTAAATCCGCAGTCGTATTCTTTAAAGCAATATCAGCCAAAATACGTTTATTTTCGGC
TGTAATATTAGGAGCAGTAAGCAACTTATTAACAGTATCAGCCCTAGTATTAGCGGTACCAGCTTCAGTAGCTTCAGTCTGGGCGATAATCTGCT
TTTCTGTTTCTGATGCATTACGAATCTGTTGCATCATAGAAGCAGTATTAATAGCTGAATTAGTTGCATTACCTAAAACATTTTCCATAGTAGCAGT
TGAACCAGATGGGGTAGAAGCACCACCACGTTGATAGGCTAACATAGGAGATAAACCAGCAGCTTCTAAATCTTTAACAGCACGCTGGTAACTT
GTTCCACTCATATCGGCTTGAAAATCCATTTGCCTCTGAGCCATCTCCTGATTAGCTTTATTAGCAGAAGTAGAACCTAAATAAGAACCAACAGCG
CCCAAAGCAGTACCGACACCAGGAGTAAAGAAACTAGAAGCTGAAGACAGCTTAGAACCAACATTAGTAACCGCATCAAGTATTCCACCAAACA
TAAACTAACGCCCTTCGGTTGTTTCCTCACTACTCCTTACGGAGTAGTCGAGGTTATATAAAACATTAGAAATGATCAATTAGACCAGGTACGCTG
TACATAGGCATAGGTCTGGCCATCTGACAATCAAAGAAAGCATCCATTAAAAATTGCTGACCATTTGCTGCAGCTCCGACCGCCGTAGTACGAGC
AACTGGAGGGGTCTCTTGAATAAACGTTGAATTCAACGTAGGAAGAGAAGTAAATCGCTGTCCATAATGCCAAGCATCAATAGTGCCAGCAGAA
GTAGACCTAAAGAAACCAGTAATTTGAGAAGGTTTGTAACGATACTCCGCCCAACGTTCTTGATAACCAAATACATCATTGTCGGCTGCAGTACC
TTGAACATAAATTTCTTTGTTCAAAATAGCTTGCTCACCCAAAGTGGCAAATACTGGGAAATAAAAGTCATATCGTGTAGACCTAGACCACATCTT
AGGAAGACCCTGTTGATATGTGAGGTCAGCACGTACAGAAACTAATCCGATAATGTATCCATGTTCTTGAGCAGCATACGTAAAGCCGTGTCCCT
GAGCCAATGCAGTACCCATTGCAGCAAGGTTACCTTGCGGAGTAGCAGAACCAGTAACCGACGTTGCAGAAGTCTGAGCAATCGGATTAACATT
AACAAGGGTCGAACCTCCACCAATATATTCAGGACGTTGTAAACGATAATCCTGTGGAGTTACTCCAAAGTGAGCACGTAGTAACTCAGTATAAC
GAGTACCACCACGTGCATCGCGCTCCAATAACTTCTGAATCTGGAAAGATTGACGAAGTTGGTTAATAGTCGCAGCAGTAGCAGTAGACAAATC
AGCGTATAACTGATTAGTAGCAACACCAGCATTTGTACTATTAGACAAAGTATTAGAAGCTGAATTTAATTCTCTTAAAGCACCAACAGCAGTTTG
AAAAACACTATAACCAGCAGTACCATTGCCAGCTGGTATGTCAGAATAAATATTAGCACGTGTTCCTAACGGTAAAGAAACAGAAGCACCCTTCT
GTGGCCAAGGCAATGCACCAGTAAAATAATCTTTACGCTTACCACGTCTCAAAAGTGTATAGTTAGCAGGAACATCACCTGAATCACCAAGATTT
AACGTTACAGAATTCTGTAAGTTTTCATCTCTAAACCATTCGTTATAAATCTCATTATAAGCACGTAACGGCAAAACATTATGCGTTACTGTATTAG
CGCCAGTAATCTGACCAGCAGTAGGTAAACCAAAATAATCAAAAATTGAACAAACAGCATAACCACCAGCAGGACTAGTAATAGTAGGCACTAC
AAAAGAAATAGAATCACCAGGATTCGTTTGTTCACCCA

>000051F|arrow

CACAATCGCAAGCCGAAAAAACGGACGCGGATACAAATCTATCACGTGATCAGGCAACTATACTCGAGCAATACAGCTCGTGAAATTGCTCAGA
TGCCGGATACGCAAATTTGGTCAGCTTCGCGATGCCCAAATAGAGCAATTAAGGACATCAAGTGCATTACAAGCTGCACAACAGCGACAAGCGT
TAAGTCAAAGTGCATATACAGACCAATTAGAGCGATTAGCGCAAACTGATCGCGCCATCCAGTACTAAACCATTTATCAAGATGTTAAAGGCTAT
TTACATAGCCATATGATAAATATCAAAAATATCTACCATTTGGAAAAATGAAATGAAACAATCAAACTTAGAACCGCATACAACTATGACACGGA
TGCTGCGTCAAATGAGTCAGGGTTGGCTTGTGAGGAGCCAACTCTGGCTCAGCAGCATTATAAAGACGAATGCGATATAAATACTATCCTGGAA
CGTTTTAACGTTACAGGCCTATTACCTCAAAGTCCGCTGCCGCCTCAATATGGCGATTTCAGCGGAATTACTGACTATCATAGCGCCTTGAATAGG
TAATGAACGCTATGGAAGAATTTGATACTTACCGGCTCAAATTCGTGCTAGGTTCGAAAACGAACCAGCAAACCTGATTGAGTTCTTGCAAGACG
AGAAAAATCGACCAGAAGCCGAGAAACTCGGCCTGGTCGAAAGAGCCATTTCGGAAGAAATGCGATAAGCACAGTTACTCCACTTGATGTAACT
GTGCTAGGTGACACCAACACCAAAAATATCTGATAACGAGGCCAAAAATTATGCTTATAGAAAACAACACACAAGCGCAAAAGCGCTAAATCGT
TCCGTAGGAACACTTCAAAAACTAAAGCTGCAAATATGCAAAAAGCCCCGCAAGAGGGGGCTGGCGGCTCTAATAAAGCGCCAGGCTACCTCA
CATGGCCTGTTATCACCCACTGACTGCTTATTTAAGTAAGCATCAGACAAACTATAAGACCGGCAAATCTTATCGCCGTGTCGCATTCAAAGAATC
TGACGAGCATGATCGTCAGATTTCACTGCCCTGCGGCCAATGCGTTGGCTGCAGGCTAGAAAATCACGTCAATGGGCCATGCGCTGCATGCATG
AAGCCCAATTGCACGAAAAAAACTGCTTTATAAACCCTCACATACAACAATGAAAACCTTCCACAAACTGGATCGCTTGTCAAAAGCGACTTCCA
AAAGTTCCTTAAGCGCTTCAGAAAATCCATTGCACCTGCAAAATACGTTTACTACATGGCTGGAGAATACGGCACAAGTTTCGGCAGACCTCACT
TCCATGCCTGTATCTTCGGATACGATTTTCATGATAAGAAACTATTCAAAGGACTCCCTCTGGTTCTCTCATATATACATCCGACCACCTTGCAACC
CTCTGGCCACATGGTTATTCCTCCATTGGAGACGTTACATTCGAGTCAGCTGCTTACGTTGCTCGATATATTATGCAAAAATACAACGGCCAGATG
GAAGAAAACAAACATATAACAAAGGATGAGCATTACACATACTGTGATATAGAAACAGGGGAATTAATAAAGCTATTACCAGAATATAACAGA
GCCTTAAACCAGGCATTGGTGCTGAGTGGTACAAAAAATATCGTTCCGACGTATATCCCCATGACTATGTTGTAGTCAACGGAAAAAGGGTAAA
ACCCCCAAAATACTATGACAAAAAATATAAATCAGATTATCCATATGAATACGAAGAATTACTCCACAAACGTGAAACTTCTGCTAAACTCAACCA
CGAAGACAATACCTATGCCAGACTTGCCGTAAAGGAAAAAGTCACAAAGGCCAAACTTCAATTATTAAAACGTAACCTCACTTAGGAAATCCTCA
TGAAATTAGTACTCTGTACCGTTAAAGACCGCGCAGCAGATGCGTTTCGGTCGTCCAATGTTCGTCCGTTCTATCGGCGAAGCAATCCGGAGCTT
TAGCGACGAAGTCAATCGCCAGAGCGATGACAATCAACTTTATAACCATTCCGACGATTTCGACCTATTTGAATTAGGCGAATTCGACGATAATA
CGGGTTTGTTCCAATTACATGAACAACCCAACTTGTATCCTTAGGAAACAAGTCAAAATTACTGATAAAAACTAAGCGTAGAGTAAAAAGGGGA
AACCCCTTTTTCTCACGCAACTAGGCCTAGGAGCTCAAAAAATGCATCGTAACATCGTAGACGTCCATCAGTTCACAATGATTCCAAAGCGGATA
TCCCCCGCTCTACATTTGACTGTCAATCAACACATAAAACTACATTCGATGCTGGCTTCCTAGTCCCTGTACTCGTAGACGAAATGTTGCCAGCGA
TACATTCCGCTGCAACATGACCGCCTTTGCGCGATTGTCTACACCACTCTATATCCGATCATGGATAACATGCATCTGGATAGCTTCTTCTTTTGTG
CCAAATAGACTTATCTGGTCAAATTGGCAAAAATTTATGGGGCAGCAGGCAAATCCTGCGGACTCGATCTCGTACGTAGTGCCCCAACAAGTAA
CCCCAGCTGGTGGTTACGCTATTGGCAGCCTTCAAGATTATATGGGTCTGCCAACCGTAGCCAAGTAGTGCTGGTGGCACCGTAAGTCACTGTGC
CTTCTGGCCACGTGCTTACAACCTTATTTATAACGAATGGTTTCGGGACGAAAACCTTCAAAATTCAGTAGTTGTAGATACTGGCGATGGTCCAG
ATAACGTAGCCAACTACACATTATTACGACGTGGAAAACGTAAAGACTATTTCACGTCAGCATTACCTTTGGCCACAAAAGGGCGCAAGCGTTAC
TTTACCGCTTGGAACATCCGCCCCAATATTACGCACTAACATGCCCTGTTTCCAGACTGTATAACGCTGGAACATACATTACACAACCCCCGCCCA
GGCTATTAACGTAGGTGTTACTGGTCAAATTACTGGCGGTGCTGACGGCTTGGCAAATCATATGATCCTAATGGCGGTTTATATGCAGATTTTCA
GCTGCAACCGCTGCACAATTAATCAATTGCGTCAAAGCTTCCAGATTCAAAACTTTTAGAAAGGGACGCCCGTGCGGAACTCGATACACAGAATT
ATCCGCAGCCATTTCGGGGTCGTTAGCCCCGATGCGCGTCTCCAACGGCCTGAATACATTGGAGGCGGTTCAACACACATTAATATCAATCCAAT
CGCCCAGACGAATGGTACCGAGCTTCCGGACCACTACTCCTCTCGGTACACTTGGCGCTATGGGTACTGGGCTCGCTCACAATCATGGCTTTACT
TATTCAAGCACTGAACATGGTGTAATTATCGGTCTCTCGTTTCAGTACGAGCCGATTTAACATACCAACAAGGTATGCACCGCATGTGGAATCGT
TCCACACGTTATGATTTCTATTTCCCTGCTTTCGCCACTTTGGGCGAACAAGCAGTATTAAATGAAGAAATCTACGTACGAGCGATGCCCGATACA
GGAGTGTTTGGATACCAAGAACGTTGGGCAGAATATCGTTATATGCCAAGCCGAATTTCCAGTCTGTTCCGTAGTACGGCAGCTGGAACAATTG
ACGGCTGGCATTTAGCCCAACGGTTTACAACACTTCCAACTTTGAATAACACGTTTATTCAAGAAAATCCACCTGTCTCTCGAACCCTTGCGGTCG
GAGCAGCTGCCAACGGCCAGCAAATCATTTTTGATAGCTTTTTTGATATTAAAAAGCACGGCCAATGCCAATGTACTCTGTACCTGGCTTAATCG
ACCACTTCTAATGGCACTAGAAGCCGCTGCCTCAGGCGCCATCTGGCGCCGCTTTTGGACCTTACGGCTCCTTAATTGGAGCCGGAATAGGTGCG
GCCGCTAGTTATTTTGGTGGTCAAGAACAAACGCTGCCAGCGCACAACAAGCTGCAGCAATGATGCAATTCCAAGATGGTATGCGACGTACTGC
ATATCAAGACGCAGTAGCGGATCTTAAGGCTGCAGGTCTTAACCCTATGCTGGCTTATTCACAAGGCGGAGCCAAAGTCCAGCCTGGTGCGCAA
GCTCCAGTAGGAATCCACTAGGTGAGGCTGGAAATTCAGCCCGTGAAGCTGCCATGGCAGTCGCCAATTTTAAACAATTACAACTCAGATATCCT
GA

>000078F|arrow

TTGCCAGCGGCCATCACCAACAGTGTAGAAATTGCCAAACGTTGTAATTTGTCATTGACCTTGGGTAAGCCTCAGTTGCCAGATTTTCCAGTGCCT
AAAGGTATGACAAATTACAACGTTTGGCAATTTCTACACTGTTGGTGATGGCCGCTGGCAAGTCTGCAAAAAGTTTTTGCAGACTTGCCAGCGGC
CATCACCAACAGTGTAGAAATTGCCAAACGTTGTAATTTGTCATTGACCTTGGGTAAGCCTCAGTTGCCAGATTTTCCAGTGCCTAAAGGTATGA
CAAA

>000155F|arrow

TGCACGAGCAATCAACTTAGGCTTTCCGTAGTTCAAAAACACCAGTGGCATCGTCAAAAAGACCCAAATAGAACATATGAAAATCATCAGGATTT
ATACAACTGATTATCTTCGCTAGCTCGATTGACTTCATCCTGAAACTGACGTACTGCAACACCTTCAGATGCAACATAAGCTGGACGACCAAAAG
CATCTGCAGCAGTATCCTTAATAGAAACAATAACCATCTTCATAAAAACTCCTTAAATAGTACGTTTTAACAATGACAACTTAGCCAACGCAACTT
TTTCCTTAACAGCAAGTCGCTCAAGCGTGTTGTCCTCATGCCTAGATCGACCTTCCATCTCTCTGGCAAACTGAATCATATCGAATTCTTCAGGAA
ACTTCAACTTAAATTTATTATCATAAAACCGTGGTGGACGGCACTTTTTGCCACGCACCACAAACGTGGTCTGACGTATAAACGTCTGACATGTAC
TTATCTAACCACGATTGCCCGATACCGGGCTTCAATGACATCTTATTAAATTCTGGCTTACGCTGAATTATCTCACCAGTCTCTAATCACAATATTG
ATAATGGGCACCCGCATCAACCACTTCGTGGTTTTCATTGACAGTAACCCCATTAATCTTCTTCATAATATATCTTGCAACATAAGCAGCAGACTC
AAAAGTAACATCACCAATTGTAGAATAGCCAAACGGCCACAATTCTTCCAAAATCTCTGACGTGTAGAGGATAGAGCCAGTCTGCGTTCTTTTAA
ATATTTTCTTATCCGGAAAATCAAGACCAAACAGACAAGCATGGAAATGAGGACGATCAAAAGATTCACCATATTCACCTGCCATATAAAAACGT
ATCGTTTTTCCCAGTAAAACGCTTACGTAACCGCTTCATAAAAAGCTGATAATCATTGTAATCCAATGACATATCCTTAGGACAATGCTCTGGAGC
ATATGTCAAAGTAATAAAACAATTACTAGTATGCATTTGTGCCTCATGCATACAACGAATCGCCCACTGACGTGAGCGTTCAAGGCGACAACCAA
CACACTGACCACAAGGCAATGATAGGGTACGGACTACATCCGCACCCGGTATTTCCCGCCAAATTATAGACCTGTCACTGCATTGATAAGCCGTT
AAGGGCTTATAACAGGCCATAATTACAAACGATAGCCACCACGCTGGGGAGCGTGTCTCATATTAATTGACTTCGTCTTACTAGCAGTTCTGCGA
AATGACTTTGCAGATTTATATTTGTTTACTGGCTTTCTTCGTAACATGATGAACTCCGTAGTTAAAATAGTGGTTTGGTGTCACCTAGCACAGTTAC
ATCAAGTAGAGTAACTGTGCTGGCCTCAGGATTTCATCCTTCGGCCTTAGGTGTTTCTGTAGAAACGATGGGTTCAACCACAGGTTGTCCATCAA
TAAGACCCAATTGAATCGCTTCATCACGATTCTGGTCGTTCTCAAGGAACTCCAATAATTTGACAGGATCATGGTCAAATCGGACTCTTAATTTCG
CTGGCAGAGCCATGAAATCGTCCATAGTTGCGTTAATTTGATTCAACGCAGAATGGTAATCAGTAACACCACTAAAATCGCCGTATTGAGGCGAT
ACAGGGGCCGTTGGAAGTTCCCCTGTAACCCCGAAACGTTCAATGATGACATTAATGTCACATTCATCTTTCATATGCTGTTGAGCAAGACTTGG
GTCTTGACACAGCAACGCATCGTTTACCGATGCTTCATCTTTATCATAGTTGTACGGATTACGTACAAATGGCAAATTCGCTTTACTCATTATTTGA
CTCCAATTCCCCAAGGGGTTAGTTGATTAGGATTATTTTTGAGTTTATCAAAAATTTCTTTCGAAGCATCGATACCCCTAGACCAAATAGCTGGGG
ATGGAGCCAATAGCCTCTTAGTATTATATGACTGAGCGGATGTTAAATCCGCAGTCGTATTCTTTAAAGCAATATCAGCCAAAATACGTTTATTTT
CGGCTGTAATATTAGGAGCAGTAAGCAACTTATTAACAGTATCAGCCCTAGTATTAGCGGTACCAGCTTCAGTAGCTTCAGTCTGGGCGATAATC
TGCTTTTCTGTTTCTGATGCATTACGAATCTGTTGCATCATAGAAGCAGTATTAATAGCTGAATTAGTTGCATTACCTAAAACATTTTCCATAGTAG
CAGTTGAACCAGATGGGGTAGAAGCACCACCACGTTGATAGGCTAACATAGGAGATAAACCAGCAGCTTCTAAATCTTTAACAGCACGCTGGTA
ACTTGTTCCACTCATATCGGCTTGAAAATCCATTTGCCTCTGAGCCATCTCCTGATTAGCTTTATTAGCAGAAGTAGAACCTAAATAAGAACCAAC
AGCGCCCAAAGCAGTACCGACACCAGGAGTAAAGAAACTAGAAGCTGAAGACAGCTTAGAACCAACATTAGTAACCGCATCAAGTATTCCACCA
AACATAAACTAACGCCCTTCGGTTGTTTCCTCACTACTCCTTACGGAGTAGTCGAGGTTATATAAAACATTAGAAATGATCAATTAGACCAGGTAC
GCTGTACATAGGCATAGGTCTGGCCATCTGACAATCAAAGAAAGCATCCATTAAAAATTGCTGACCATTTGCTGCAGCTCCGACCGCCGTAGTAC
GAGCAACTGGAGGGGTCTCTTGAATAAACGTTGAATTCAACGTAGGAAGAGAAGTAAATCGCTGTCCATAATGCCAAGCATCAATAGTGCCAGC
AGAAGTAGACCTAAAGAAACCAGTAATTTGAGAAGGTTTGTAACGATACTCCGCCCAACGTTCTTGATAACCAAATACATCATTGTCGGCTGCAG
TACCTTGAACATAAATTTCTTTGTTCAAAATAGCTTGCTCACCCAAAGTGGCAAATACTGGGAAATAAAAGTCATATCGTGTAGACCTAGACCACA
TCTTAGGAAGACCCTGTTGATATGTGAGGTCAGCACGTACAGAAACTAATCCGATAATGTATCCATGTTCTTGAGCAGCATACGTAAAGCCGTGT
CCCTGAGCCAATGCAGTACCCATTGCAGCAAGGTTACCTTGCGGAGTAGCAGAACCAGTAACCGACGTTGCAGAAGTCTGAGCAATCGGATTAA
CATTAACAAGGGTCGAACCTCCACCAATATATTCAGGACGTTGTAAACGATAATCCTGTGGAGTTACTCCAAAGTGAGCACGTAGTAACTCAGTA
TAACGAGTACCACCACGTGCATCGCGCTCCAATAACTTCTGAATCTGGAAAGATTGACGAAGTTGGTTAATAGTCGCAGCAGTAGCAGTAGACA
AATCAGCGTATAACTGATTAGTAGCAACACCAGCATTTGTACTATTAGACAAAGTATTAGAAGCTGAATTTAATTCTCTTAAAGCACCAACAGCA
GTTTGAAAAACACTATAACCAGCAGTACCATTGCCAGCTGGTATGTCAGAATAAATATTAGCACGTGTTCCTAACGGTAAAGAAACAGAAGCAC
CCTTCTGTGGCCAAGGCAATGCACCAGTAAAATAATCTTTACGCTTACCACGTCTCAAAAGTGTATAGTTAGCAGGAACATCACCTGAATCACCA
AGATTTAACGTTACAGAATTCTGTAAGTTTTCATCTCTAAACCATTCGTTATAAATCTCATTATAAGCACGTAACGGCAAAACATTATGCGTTACTG
TATTAGCGCCAGTAATCTGACCAGCAGTAGGTAAACCAAAATAATCAAAAATTGAACAAACAGCATAACCACCAGCAGGACTAGTAATAGTAGG
CACTACAAAAGAAATAGAATCACCAGGATTCGTTTGTTCACCCATAAACTTTGGCCAATTGTTCCAAAGTAAACGATTAGGTACAAAGAAAAAGA
AAGTATCAAGATGCAAGTTGTCCATCACAGGAAATAACGGTGTGGCCAAACGTGCAAATGCAGTCATCTTTACACGATGAGTATCGCCAGGCAA
TACTTCATCACAATAAATAGGAACTAAAAAACCAGCATCAAACGTGGTTTTATGAGCATATTGAGTATTAAAACTAGAGCGGGGAATGTCCGCTT
TAGGAACCATAGCAAATGAATGCGTACTTACTGACTTATTGCGAAACATAAACATCTCCCGTAGTTCCGTACCACTCTTTCGAGTGATACGGTATA
AAAAAAACCTTACTCGCCTTCGCGAATCATTACATCTTT

>000012F|arrow

GTGAATCTTTTGATCGTCATTTCCATGCTTGTCTGTTTGGTCTTGATTTCCGGATAAGAAAATATTAAAAGAACGCAGACTGGCTCTATCCTCTACA
CGTCAGAGATTTTGGAAGAATTGTGGCCGTTTGGCTATTCTACAATTGGTGATGTTACTTTTGAGTCTGCTGCTTATGTTGCAAGATATATTATGA
AGAAGATTAATGGGGTTACTGTCAATGAAAACCACGAAGTGGTTGATGCGGGTGCCCATTATCAATATTGTGATTTAGAGACTGGTGAGATAAT
TCAGCGTAAGCCAGAATTTAATAAGATGTCATTGAAGCCCGGTATCGGGCAATCGTGGTTAGATAAGTACATGTCAGACGTTTATACGTCAGAC
CACGTTGTGGTGCGTGGCAAAAAGTGCCGTCCACCACGGTTTTATGATAATAAATTTAAGTTGAAGTTTCCTGAAGAATTCGATATGATTCAGTT

TGCCAGAGAGATGGAAGGTCGATCTAGGCATGAGGACAACACGCTTGAGCGACTTGCTGTTAAGGAAAAAGTTGCGTTGGCTAAGTTGTCATT
GTTAAAACGTACTATTTAAGGAGTTTTTATGAAGATGGTTATTGTTTCTATTAAGGATACTGCTGCAGATGCTTTTGGTCGTCCAGCTTATGTTGC
ATCTGAAGGTGTTGCAGTACGTCAGTTTCAGGATGAAGTCAATCGAGCTAGCGAAGATAATCAGTTGTATAAACATCCTGATGATTTTCATATGT
TCTATTTGGGTCTTTTTGACGATGCCACTGGTGTTTTGAACTACTGGAAAGCCCTAAGTTGATTGCTCGTGCAAAAGATGTAATGATTCGCGAAG
GCGAGTAAGGTTTTTTTATACCGTATCACTCGAAAGAGTGGTACGGAACTACGGGAGATGTTTATGTTTCGCAATAAGTCAGTACGCATTCATT
TGCTATGGTTCCTAAAGCGGACATTCCCCGCTCTAGTTTTAATACTCAATATGCTCATAAAACCACGTTTGATGCTGGTTTTTTAGTTCCTATTTATT
GTGATGAAGTATTGCCTGGCGATACTCATCGTGTAAAGATGACTGCATTTGCACGTTTGGCCACACCGTTATTTCCTGTGATGGACAACTTGCAT
CTTGATATTCTTTTTCTTTGTACCTAATCGTTTACTTTGGAACAATTGGCCAAAGTTTATGGGTGAACAAACGAATCCTGGTGATTCTATTTTTTGT
AGTGCTACTATTACTAGTCCTGCTGGTGGTTATGCTGTTGTTCAATTTTTGATTATTTTGGTTTACCTACTGCTGGTCAGATTACTGGCGCTAATAC
AGTAACGCATAATGTTTTGCCGTTACGTGCTTATAATGAGATTTATAACGAATGGTTTAGAGATGAAAACTTACAGAATTCTGTAACGTTAAATCT
TGGTGATTCAGGTGATGTTCCTGCTAACTATACACTTTTGAGACGTGGTAAGCGTAAAGATTATTTTACTGGTGCATTGCCTTGGCCACAGAAGG
GTGCTTCTGTTTCTTTACCGTTAGGAACACGTGCTAATATTTATTCTGACATACCAGCTGGCAATGGTACTGCTGGTTATAGTGTTTTTCAAACTGC
TGTTGGTGCTTTAAGAGAATTAAATTCAGCTTCTAATACTTTGTCTAATAGTACAAATGCTGGTGTTGCTACTAATCAGTTATACGCTGATTTGTCT
ACTGCTACTGCTGCGACTATTAACCAACTTCGTCAATCTTTCCAGATTCAGAAGTTATTGGAGCGCGATGCACGTGGTGGTACTCGTTATACTGAG
TTACTACGTGCTCACTTTGGAGTAACTCCACAGGATTATCGTTTACAACGTCCTGAATATATTGGTGGAGGTTCGACCCTTGTTAATGTTAATCCG
ATTGCTCAGACTTCTGCAACGTCGGTTACTGGTTCTGCTACTCCGCAAGGTAACCTTGCTGCAATGGGTACTGCATTGGCTCAGGGACACGGCTT
TACGTATGCTGCTCAAGAACATGGATACATTATCGGATTAGTTTCTGTACGTGCTGACCTCACATATCAACAGGGTCTTCCTAAGATGTGGTCTAG
GTCTACACGATATGACTTTTATTTCCCAGTATTTGCCACTTTGGGTGAGCAAGCTATTTTGAACAAAGAAATTTATGTTCAAGGTACTGCAGCCGA
CAATGATGTATTTGGTTATCAAGAACGTTGGGCGGAGTATCGTTACAAACCTTCTCAAATTACTGGTTTCTTTAGGTCTACTTCTGCTGGCACTAT
TGATGCTTGGCATTATGGACAGCGATTTACTTCTCTTCCTACGTTGAATTCAACGTTTATTCAAGAGACCCTCCAGTTGCTCGTACTACGGCGGTC
GGAGCTGCAGCAAATGGTCAGCAATTTTTAATGGATGCTTTCTTTGATTGTCAGATGGCCAGACCTATGCCTATGTACAGCGTACCTGGTCTAAT
TGATCATTTCTAATGTTTTTATAACCTCGACTACTCCGTAAGGAGTAGTGAGGAAACAACCGAAGGGCGTTAGTTTATGTTTGGTGGAATACTTG
ATGCGGTTACTGTTGGTTCTAAGCTGTCTTCAGCTTCTAGTTTTTACTCCTGGTGTCGGTACTGCTTTGGGCGGTTGGTTCTTATTTAGGTTCTACT
TCTGCTAATAAAGCTAATCAGGAGATGGCTCAGAGGCAAATGGATTTTCAAGCCGATATGAGTGGAACAAGTTACCAGCGTGCTGTTAAAGATT
TAGAAGCTGCTGGTTTATCTCCTATGTTAGCCTATCAACGTGGTGGTGCTTCTACCCCATCTGGTTCAACTGCTACTATGGAAAATGTTTTAGGTA
ATGCAACTAATTCAGCTATTAATACTGCTTCTATGATGCAACAGATTCGTAATGCATCAGAAACAGAAAAGCAGATTATCGCCCAGACTGAAGCT
ACTGAAGCTGGTACCGCTAATACTAGGGCTGATACTGTTAATAAGTTGCTTACTGCTCCTAATATTACAGCCGAAAATAAACGTATTTTGGCTGAT
ATTGCTTTAAAGAATACGACTGCGGATTTAACATCCGCTCAGTCATATAATACTAAGAGGCTATTGGCTCCATCCCAGCTATTTGGTCTAGGGGTA
TCGATGCTTCGAAAGAAATTTTGATAAACTCAAAAATAATCCTAATCAACTAACCCTTGGGGAATTGGAGTCAAATAATGAGTAAAGCGAATTTG
CCATTTGTACGTAATCCGTACAACTATGATAAAGATGAAGCATCGGTAAACGATGCGTTGCTGTGTCAAGACCCAAGTCTGCTCAACAGCATATG
AAAGATGAATGTGACATTAATGTCATCATTGAACGTTTCGGGGTTACAGGGGAACTTCCAACGGCCCCTGTATCGCCTCAATACGGCGATTTTAG
TGGTGTTACTGATTACCATTCTGCGTTGAATCAAATTAACGCAACTATGGACGATTTCATGGCTCTGCCAGCGAAATTAAGAGTCCGATTTGACCA
TGATCCTGTCAAATTATTGGAGTTCCTTGAGAACGACCAGAATCGTGATGAAGCGATTCAATTGGGTCTTATGATGGACAACCTGTGGTTGAACC
CATCGTTTCTACAGAAACACCTAAGGCCGAAGGATGAAATCCTGAGGCCAGCACAGTTACTCTACTTGATGTAACTGTGCTAGGTGACACCAAAC
CACTATTTTAACTACGGAGTTCATCATGTTACGAAGAAAGCCAGTAAACAAATATAAATCTGCAAAGTCATTTCGCAGAACTGCTAGTAAGACGA
AGTCAATTAATATGAGACACGCTCCCAGCGTGGTGGCTATCGTTTGTAATTATGGCCTGTTATAAGCCCTTAACGGCTTATCAATGCAGTGACAG
GTCTATAATTTGGCGGGAAATACCGGGTGCGGATGTAGTCCGTACCCTATCATTGCCTTGTGGTCAGTGTGTTGGTTGTCGCCTTGAACGCTCAC
GTCAGTGGGCGATTCGTTGTATGCATGAGGCACAAATGCATACTAGTAATTTTATTACTTTGACATATGCTCCAGAGCATTGTCTAAGGATATGTC
ATTGGATTACAATGATTATCAGCTTTTTATGAAGCGGTTACGTAAGCGTTTTACTGGGAAAACGATACGTTTTTATATGGCAGGTGAATATG

>000177F|arrow

CATAAACTTTGGGCCAATTGTTCCAAAGTAAACGATTAGGTACAAGAAAAAAGAAAGTATCAAGATGCAGTTGTCCATCACAGGAAATAACGGT
GGTGGCCAACGTGCAAATGCAGTCATCTTTACAACGATGAGTATCGCCCAGGCATACTTCATCACAATAATAGGAACTAAAAACCAGCATCAAAC
GTGGTTTTATGAGCATATTGAGTATTAAAACTAGAGCGGGGAATGTCCGCTTAGGAACCATAGCAAATGAATGCGTACTTACTGACTTATTGCGA
AACATAAACATCTCCCGTAGTTCCGTACCACTCTTTCGAGTGATACGGTATAAAAAAAAACCTTACTCGCCTTCGCGAATCATTACATCTGTTGCA
CGAGCAATCAACTTAGGGCTTTCCAGTAGTTCAAAAAACACCAGTGGCATCGTCAAAAAGACCCAAATAGAACATATGAAAATCATCAGGATGT
TTATACAACTGATGATCTTCGCTAGCTCGATTGACTTCAGCCTGAAACTGACGTACTGCAACACCTTCAGAGTGCAACATAAGCTGGACGACCAA
AAGCATCTGCAGCAGTACCTTAATAGAAACAATAACCATCTTCATAAAAACTCCTTAATAGTACGTTTTAACAATGACAACTTAGCCAACGCAACT
TTTTCCTTAACAGCAAGTCGCTCAAGCGTGTTGTCCTCATGCCTAGATCGACCTTCCATCTCTCTGGCAAACTGAATCATATCGAATTCTTCAGGAA
ACTTCAACTTAAATTTATTATCATAAAACCGTGGTGGACGGCACTTTTTGCCACGCACCACAAACGTGGTCTGACGTATAAACGTCTGACATGTAC
TTATCTAACCACGATTGCCCGATACCGGGCTTCAATGACATCTTATTAAATTCTGGCTTACGCTGATTATCTCACCAGTCTCTAAATCACAATATTG
ATAATGGGCACCCGCATCAACCACTTCGTGGTTTTCATTGACAGTAACCCATTAATCTTCTTCATAATATATCTTGCAACATAAGCAGCAGACTCA

AAAGTAACATCACCAATTGTAGAATAGCCAAACGGCCACAATTCTTCCAAAATCTCTGACGTGTAGAGGATAGAGCCAGTCTGCGTTCTTTTAAA
TATTTCTTATCCGGAAAATCAAGACCAAACAGACAAGCATGGAAATGAGGACGATCAAAAGATTCACCATATTCACCTGCCATATAAAAACGTAT
CGTTTTCCCAGTAAAACGCTTACGTAACCGCTTCATAAAAGCTGATAATCATTGTAATCCAATGACATATCCTTAGGACAATGCTCTGGAGCATAG
TCAAAGTAATAAAACAATTACTAGTATGCATTTGTGCCTCATGCATACAAACGAATCGCCCACTGGACGTGAGCGTTCAAGGCGACAACCACACA
CTGACCACAAGGCAAATGATAGGGTACGGACTACATCCGCACCCGGTATTTCCCGCCAAATTATAGACCTGTCACTGCATTGATAAGCCGTTAAG
GGCTTATAACAGGCCATAATTACAAACGATAGCCACCACGCTGGGGAGCGTGTCTCATATTAATTGACTTCGTCTTACTAGCAGTTCTGCGAAAT
GACTTTGCAGATTTATATTTGTTTACCTGGCTTTCTTCGTAACATGATGAACTCCGTAGTTAAAATAGTGGTTTGGTGTCACCTAGCACAGTTACAT
CAAGTAGAGTAACTGTGCTGGCCTCAGGATTTCATCCTTCGGCCTTAGGGTTTTCTGTAGAAACGATGGGTTCAACCACAGGTTGTCCATCAATA
AGACCCAATTGAATCGCTTCATCACGATTCTGGTCGTTCTCAAGGAACTCCAATAATTTGACAGGATCATGGTCAATCGGACTCTTAATTTCGCTG
GCAGAGCCATGAAATCGTCCATAGTTGCGTTAATTTGATTCAACGCAGAATGGTAATCAGTAACACCACTAAAATCGCCGTATTGAGGCGATACA
GGGGCCGTTGGAAGTTCCCCTGTAACCCCGAAAACGTTCAATGATGACATTAATGTCACATTCATCTTTCATATGCTGTTGAGCAAGACTTGGGT
CTTGACACAGCAACGCATCGTTTACCGATGCTTCATCTTTATCATAGTTGTACGGATTACGTACAAATGGCAAATTCGCTTTAACTCATTATTTGAC
TCCAATTCCCCAAGGGGTTAGTTGATTAGGATTATTTTTGAGTTGTATCAAAAATTTCTTTCGAAGCATCGATACCCCTAGACCAAATAGCTGGGG
ATGGAGCCAATAGCCTCTTAGTATTATATGACTGAGCGGATGTTAAATCCGCAGTCGTATTCTTTAAAGCAATATCAGCCAAAATACGTTTATTTT
CGGCTGTAATATTAGGAGCAGTAAGCAACTTATTAACAGTATCAGCCCTAGTATTAGCGGTACCAGCTTCAGTAGCTTCAGTCTGGGCGATAATC
TGCTTTTCTGTTTCTGATGCATTACGAATCTGTTGCATCATAGAAGCAGTATTAATAGCTGAATTAGTTGCATTACCTAAAACATTTCCATAGTAGC
AGTTGAACCAGATGGGGTAGAAGCACCACCACGTTGATAGGCTAACATAGGAGATAACCAGCAGCTTCTAAATCTTTAACAGCACGCTGGTAAC
TTGTTCCACTCATATCGGCTTGAAAATCCATTTGCCTCTGAGCCATCTCCTGATTAGCTTTATTAGCAGAAGTAGAACCTAAATAAGAACCAACAG
CGCCCAAAGCAGTACCGACACCAGGAGTAAAGAAACTAGAAGCTGAAGACAGCTTAGAACCAACATTAGTAACCGCATCAAGTATTCCACCAAA
CATAAACTAACGCCCTTCGGTTGTTTCCTCACTACTCCTTACGGAGTAGTCGAGGTTATATAAAACATTAGAAATGATCAATTAGACCAGGTACGC
TGTACATAGGCATAGGTCTGGCCATCTGACAATCAAAGAAAGCATCCATTAAAAATTGCTGACCATTTGCTGCAGCTCCGAACCGCCGTAGTACG
AGCAACTGGAGGGGTCTCTTGAATAAACGTTGAATTCAACGTAGGAAGAGAAGTAAATCGCTGTCCATAATGCCAAGCATCAATAGTGCCAGCA
GAAGTAGACCTAAAGAAACCAGTAATTTGAGAAGGTTTGTAACGATACTCCGCCCAACGTTCTTGATAACCAAATACATCATTGTCGGCTGCAGT
ACCTTGAACATAAATTTCTTTGTTCAAAATAGCTTGCTCACCCAAAGTGGCAAATACTGGGAAATAAAAGTCATATCGTGTAGACCTAGACCACAT
CTTAGGAAGACCCTGTTGATATGTGAGGTCAGCACGTACAGAAACTAATCCGATAATGTATCCATGTTCTTGAGCAGCATACGTAAAGCCGTGTC
CCTGAGCCAATGCAGTACCCATTGCAGCAAGGTTACCTTGCGGAGTAGCAGAACCAGTAACCGACGTTGCAGAAGTCTGAGCAATCGGATTAAC
ATTAACAAGGGTCGAACCTCCACCAATATATTCAGGACGTTGTAAACGATAATCCTGTGGAGTTACTCCAAAGTGAGCACGTAGTAACTCAGTAT
AACGAGTACCACCACGTGCATCGCGCTCCAATAACTTCTGAATCTGGAAAGATTGACGAAGTTGGTTAATAGTCGCAGCAGTAGCAGTAGACAA
ATCAGCGTATAACTGATTAGTAGCAACACCAGCATTTGTACTATTAGACAAAGTATTAGAAGCTGAATTTAATTCTCTTAAAGCACCAACAGCAGT
TTGAAAAACACTATAACCAGCAGTACCATTGCCAGCTGGTATGTCAGAATAAATATTAGCACGTGTTCCTAACGGTAAAGAAACAGAAGCACCCT
TCTGTGGCCAAGGCAATGCACCAGTAAAATAATCTTTACGCTTACCACGTCTCAAAAGTGTATAGTTAGCAGGAACATCACCTGAATCACCAAGA
TTTAACGTTACAGAATTCTGTAAGTTTTCATCTCTAAACCATTCGTTATAAATCTCATTATAAAGCACGTAACGGCAAAACATTATGCGTTACTGTA
TTAGCGCCAGTAATCTGACCAGCAGTAGGTAAACCAAAATAATCAAAAATTGAACAAACAGCATAACCACCAGCAGGACTAGTAATAGTAGGCA
CTACAAAGAAATAGAATCACCAGGATTCGTTTGTTCACC

>000000F|arrow

TCTAATTGATCATTTCTAATGTTTTATATAACCTCGACTACTCCGTAAGGAGTAGTGAGGAAACAACCGAAGGGCGTTAGTTTATGTTTGGTGGA
ATACTTGATGCGGTTACTAATGTTGGTTCTAAGCTGTCTTCAGCTTCTAGTTTCTTACTCCTGGTGTCGGTACTGCTTTGGGCGCTGTTGGTTCTTA
TTTAGGTTCTACTTCTGCTAATAAAGCTAATCAGGAGATGGCTCAGAGGCAAATGGATTTTCAAGCCGATATGAGTGGAACAAGTTACCAGCGT
GCTGTTAAAGATTTAGAAGCTGCTGGTTTATCTCCTATGTTAGCCTATCAACGTGGTGGTGCTTCTACCCCATCTGGTTCAACTGCTACTATGGAA
AATGTTTTAGGTAATGCAACTAATTCAGCTATTAATACTGCTTCTATGATGCAACAGATTCGTAATGCATCAGAAACAGAAAAGCAGATTATCGCC
CAGACTGAAGCTACTGAAGCTGGTACCGCTAATACTAGGGCTGATACTGTTAATAAGTTGCTTACTGCTCCTAATATTACAGCCGAAAATAAACG
TATTTTGGCTGATATTGCTTTAAAGAATACGACTGCGGATTTAACATCCGCTCAGTCATATAATACTAAGAGGCTATTGGCTCCATCCCCAGCTAT
TTGGTCTAGGGGTATCGATGCTTCGAAAGAAATTTTTGATAAACTCAAAAATAATCCTAATCAACTAACCCCTTGGGGAATTGGAGTCAAATAAT
GAGTAAAGCGAATTTGCCATTTGTACGTAATCCGTACAACTATGATAAAGATGAAGCATCGGTAAACGATGCGTTGCTGTGTCAAGACCCAAGT
CTTGCTCAACAGCATATGAAAGATGAATGTGACATTAATGTCATCATTGAACGTTTCGGGGTTACAGGGGAACTTCCAACGGCCCCTGTATCGCC
TCAATACGGCGATTTTAGTGGTGTTACTGATTACCATTCTGCGTTGAATCAAATTAACGCAACTATGGACGATTTCATGGCTCTGCCAGCGAAATT
AAGAGTCCGATTTGACCATGATCCTGTCAAATTATTGGAGTTCCTTGAGAACGACCAGAATCGTGATGAAGCGATTCAATTGGGTCTTATTGATG
GACAACCTGTGGTTGAACCCATCGTTTCTACAGAAACACCTAAGGCCGAAGGATGAAATCCTGAGGCCAGCACAGTTACTCTACTTGATGTAACT
GTGCTAGGTGACACCAAACCACTATTTTAACTACGGAGTTCATCATGTTACGAAGAAAGCCAGTAAACAAATATAAATCTGCAAAGTCATTTCGC
AGAACTGCTAGTAAGACGAAGTCAATTAATATGAGACACGCTCCCCAGCGTGGTGGCTATCGTTTGTAATTATGGCCTGTTATAAGCCCTTAACG
GCTTATCAATGCAGTGACAGGTCTATAATTTGGCGGGAAATACCGGGTGCGGATGTAGTCCGTACCCTATCATTGCCTTGTGGTCAGTGTGTTGG

TTGTCGCCTTGAACGCTCACGTCAGTGGGCGATTCGTTGTATGCATGAGGCACAAATGCATACTAGTAATTGTTTTATTACTTTGACATATGCTCC
AGAGCATTGTCCTAAGGATATGTCATTGGATTACAATGATTATCAGCTTTTTATGAAGCGGTTACGTAAGCGTTTTACTGGAAAACGATACGTT
TTTATATGGCAGGTGAATATGGTGAATCTTTTGATCGTCCTCATTTCCATGCTTGTCTGTTTGGTCTTGATTTTCCGGATAAGAAAATATTTAAAAG
AACGCAGACTGGCTCTATCCTCTACACGTCAGAGATTTTGGAAGAATTGTGGCCGTTTGGCTATTCTACAATTGGTGATGTTACTTTTGAGTCTGC
TGCTTATGTTGCAAGATATATTATGAAGAAGATTAATGGGGTTACTGTCAATGAAAACCACGAAGTGGTTGATGCGGGTGCCCATTATCAATATT
GTGATTTAGAGACTGGTGAGATAATTCAGCGTAAGCCAGAATTTAATAAGATGTCATTGAAGCCCGGTATCGGGCAATCGTGGTTAGATAAGTA
CATGTCAGACGTTTATACGTCAGACCACGTTGTGGTGCGTGGCAAAAAGTGCCGTCCACCACGGTTTTATGATAATAAATTTAAGTTGAAGTTTC
CTGAAGAATTCGATATGATTCAGTTTGCCAGAGAGATGGAAGGTCGATCTAGGCATGAGGACAACACGCTTGAGCGACTTGCTGTTAAGGAAA
AAGTTGCGTTGGCTAAGTTGTCATTGTTAAAACGTACTATTTAAGGAGTTTTTATGAAGATGGTTATTGTTTCTATTAAGGATACTGCTGCAGATG
CTTTTGGTCGTCCAGCTTATGTTGCATCTGAAGGTGTTGCAGTACGTCAGTTTCAGGATGAAGTCAATCGAGCTAGCGAAGATAATCAGTTGTAT
AAACATCCTGATGATTTTCATATGTTCTATTTGGGTCTTTTTGACGATGCCACTGGTGTTTTTGAACTACTGGAAAGCCCTAAGTTGATTGCTCGTG
CAAAAGATGTAATGATTCGCGAAGGCGAGTAAGGTTTTTTTATACCGTATCACTCGAAAGAGTGGTACGGAACTACGGGAGATGTTTATGTTTC
GCAATAAGTCAGTAAGTACGCATTCATTTGCTATGGTTCCTAAAGCGGACATTCCCCGCTCTAGTTTTAATACTCAATATGCTCATAAAACCACGT
TTGATGCTGGTTTTTTAGTTCCTATTTATTGTGATGAAGTATTGCCTGGCGATACTCATCGTGTAAAGATGACTGCATTTGCACGTTTGGCCACAC
CGTTATTTCCTGTGATGGACAACTTGCATCTTGATACTTTCTTTTTCTTTGTACCTAATCGTTTACTTTGGAACAATTGGCCAAAGTTTATGGGTGA
ACAAACGAATCCTGGTGATTCTATTTCTTTTGTAGTGCCTACTATTACTAGTCCTGCTGGTGGTTATGCTGTTTGTTCAATTTTTGATTATTTTGGTT
TACCTACTGCTGGTCAGATTACTGGCGCTAATACAGTAACGCATAATGTTTTGCCGTTACGTGCTTATAATGAGATTTATAACGAATGGTTTAGAG
ATGAAAACTTACAGAATTCTGTAACGTTAAATCTTGGTGATTCAGGTGATGTTCCTGCTAACTATACACTTTTGAGACGTGGTAAGCGTAAAGATT
ATTTTACTGGTGCATTGCCTTGGCCACAGAAGGGTGCTTCTGTTTCTTTACCGTTAGGAACACGTGCTAATATTTATTCTGACATACCAGCTGGCA
ATGGTACTGCTGGTTATAGTGTTTTTCAAACTGCTGTTGGTGCTTTAAGAGAATTAAATTCAGCTTCTAATACTTTGTCTAATAGTACAAATGCTG
GTGTTGCTACTAATCAGTTATACGCTGATTTGTCTACTGCTACTGCTGCGACTATTAACCAACTTCGTCAATCTTTCCAGATTCAGAAGTTATTGGA
GCGCGATGCACGTGGTGGTACTCGTTATACTGAGTTACTACGTGCTCACTTTGGAGTAACTCCACAGGATTATCGTTTACAACGTCCTGAATATAT
TGGTGGAGGTTCGACCCTTGTTAATGTTAATCCGATTGCTCAGACTTCTGCAACGTCGGTTACTGGTTCTGCTACTCCGCAAGGTAACCTTGCTGC
AATGGGTACTGCATTGGCTCAGGGACACGGCTTTACGTATGCTGCTCAAGAACATGGATACATTATCGGATTAGTTTCTGTACGTGCTGACCTCA
CATATCAACAGGGTCTTCCTAAGATGTGGTCTAGGTCTACACGATATGACTTTTATTTCCCAGTATTTGCCACTTTGGGTGAGCAAGCTATTTTGA
ACAAAGAAATTTATGTTCAAGGTACTGCAGCCGACAATGATGTATTTGGTTATCAAGAACGTTGGGCGGAGTATCGTTACAAACCTTCTCAAATT
ACTGGTTTCTTTAGGTCTACTTCTGCTGGCACTATTGATGCTTGGCATTATGGACAGCGATTTACTTCTCTTCCTACGTTGAATTCAACGTTTATTCA
AGAGACCCCTCCAGTTGCTCGTACTACGGCGGTCGGAGCTGCAGCAAATGGTCAGCAATTTTTAATGGATGCTTTCTTTGATTGTCAGATGGCCA
GACCTATGCCTATGTACAGCGTACCTGG

>000165F|arrow

TCAAGTCTCAATCCTATTGCGCAAAGATATATCTGTAAAATGAAATATGCTGAAGTGGTATCCAGTCCGCTCGGTGGTGGGACATATGGTCTTTA
CGCTCTCAATCTTAATTCCATCTTTGACCCCAACCGCACAGGTGTGGGGCATCAACCCTACGGGCACGATACATTTCAAACAATGTATAACCGTTA
CCGTGTAATCGCGTGCTCATACCGTATTGCCGTGTCATCTAGTGATGCGGCTGCTGTTCAGTTTGCGGTCCAACCAACAAATGAAGCGATAGCGC
CAGCAAACGTTAGCGAGGTTCGTGAAAACCCTCGCGCTCGGTATGCTTTACAAGGAGCAAGTGGTTCTCCTGTTAAATTTATAAGTGGTAAGGT
GTATTTACCAAGTCTCATGGGACGTAATAAGGCACAATATATGGCAGATGATAGGTATCAAGCCGTATTTGGTGCTAGTCCAAGTGAGATTGCA
TTGCTAAATATTTATTCAAGTAATTTGAGTGAATCAGCATCTGCTGCTACTCATTTTACAATATTGAACTGTCTTACACAATTGAATGTTTTGATGT
AAAGCATTTAACTCAGTCTTAGTAATTCCTCTAACACGGGGCCCTTGTGGCCCGCCCCAAGCGAAGCGCGGGAATCGATGTCCGGCGAGGTTCT
GCCCTCGCAGCGTAGCGAGAAGGCAGGTTCTGCGACGGACATTTCGATCGGAGCAACAGTGATTTACTAGAAAGTTCCCAAAGACGAGCCTACT
TAATAGGCTCGTCTAGGTCTAAAGTCTAAAGTCTAAAGTCTAAGTAGAATGGACTATTTTATTAGAATATCTAGAAGTTATGAAGAAGTAAGCGT
AATCATACCAGAATTTGATAAATGTTCAATAAGTCTCGTAATATATGAGCACGAGAAGGACGAAGAGGTCAATACAACTCATGTTCATATGTATT
TGGCAGGTTGTAGTGTGGGAACTGATACTCTTAAGAATTATGTTAAGAAAAAACTAGGAGATGTTAAGAAAACTGATTGGTCATTTAAGACATC
GTATGGAAAACCTATAAAACCTGTTGATAAGAAATGTATAACTTACATGTCCAAGGGTGGCTTAAACCCGTCTTATGTTAGAGGTATAGGGGAT
GAAGAAATTGTAACCCTGACCAGTGAGTGGGTTACTCCTAGTAACCTATCCTGGAATCTGTCTGAAGGTAAAATAAATATTTCTGATGGGACCCC
CCCAGAAGATGCTAAGAAGCCACCTACGTGGATGATTATGCAGCAAGTCGCTCATAGATGTGATGTTAATAACATCACCTCCGATGAAGACATTT
TAGATGAAATTGAAAAAACTATGAAAAAATACAATCATAAAACTAGTATTTATGATATGTTAAATTGGTTTGACAATGTCCGCTTTTATTCAGATA
AGAATAAAGGTCGTTTCAAAAATGAAGTTTTGGAAAAAATCAATTCTCGTCGTAGGGTTTAATTATTAACGTGTGATCTTGCGTTCAAATAAAAA
TCTAAGTAGTAAGTATAAATGGTCGCAAAAAGAACACGTATGCGTAAATCTCGTCCCCGGCGTAAGCGTGGTGGATATAAAACGAATAACAATT
TA

>000057F|arrow

CTAGCGAAGATAATCAGTTGTATAAACATCCTGATGATTTTCATATGTTCTATTTGGGTCTTTTTGACGATGCCACTGGTGTTTTTGAACTACTGGA
AAGCCCTAAGTTGATTGCTCGTGCAAAAGATGTAATGATTCGCGAAGGCGAGTAAGGTTTTTTTATACCGTATCACTCGAAAGAGTGGTACGG
AACTACGGGAGATGTTTATGTTTCGCAATAAGTCAGTAAGTACGCATTCATTTGCTATGGTTCCTAAAGCGGACATTCCCCGCTCTAGTTTTAATA
CTCAATATGCTCATAAAACCACGTTTGATGCTGGTTTTTTAGTTCCTATTTATTGTGATGAAGTATTGCCTGGCGATACTCATCGTGTAAAGATGA
CTGCATTTGCACGTTTGGCCACACCGTTATTTCCTGTGATGGACAACTTGCATCTTGATACTTTCTTTTTCTTTGTACCTAATCGTTTACTTTGGAAC
AATTGGCCAAAGTTTATGGGTGAACAAACGAATCCTGGTGATTCTATTTCTTTTGTAGTGCCTACTATTACTAGTCCTGCTGGTGGTTATGCTGTT
TGTTCAATTTTTGATTATTTTGGTTTACCTACTGCTGGTCAGATTACTGGCGCTAATACAGTAACGCATAATGTTTTGCCGTTACGTGCTTATAATG
AGATTTATAACGAATGGTTTAGAGATGAAAACTTACAGAATTCTGTAACGTTAAATCTTGGTGATTCAGGTGATGTTCCTGCTAACTATACACTTT
TGAGACGTGGTAAGCGTAAAGATTATTTTACTGGTGCATTGCCTTGGCCACAGAAGGGTGCTTCTGTTTCTTACCGTTAGGAACACGTGCTAATA
TTTATTCTGACATACCAGCTGGCAATGGTACTGCTGGTTATAGTGTTTTTCAAACTGCTGTTGGTGCTTTAAGAGAATTAAATTCAGCTTCTAATAC
TTTGTCTAATAGTACAAATGCTGGTGTTGCTACTAATCAGTTATACGCTGATTTGTCTACTGCTACTGCTGCGACTATTAACCAACTTCGTCAATCT
TTCCAGATTCAGAAGTTATTGGAGCGCGATGCACGTGGTGGTACTCGTTATACTGAGTTACTACGTGCTCACTTTGGAGTAACTCCACAGGATTA
TCGTTTACAACGTCCTGAATATATTGGTGGAGGTTCGACCCTTGTTAATGTTAATCCGATTGCTCAGACTTCTGCAACGTCGGTTACTGGTTCTGC
TACTCCGCAAGGTAACCTTGCTGCAATGGGTACTGCATTGGCTCAGGGACACGGCTTTACGTATGCTGCTCAAGAACATGGATACATTATCGGAT
TAGTTTCTGTACGTGCTGACCTCACATATCAACAGGGTCTTCCTAAGATGTGGTCTAGGTCTACACGATATGACTTTTATTTCCCAGTATTTGCCAC
TTTGGGTGAGCAAGCTATTTTGAACAAAGAAATTTATGTTCAAGGTACTGCAGCCGACAATGATGTATTTGGTTATCAAGAACGTTGGGCGGAG
TATCGTTACAAACCTTCTCAAATTACTGGTTTCTTTAGGTCTACTTCTGCTGGCACTATTGATGCTTGGCATTATGGACAGCGATTTACTTCTCTTCC
TACGTTGAATTCAACGTTTATTCAAGAGACCCCTCCAGTTGCTCGTACTACGGCGGTCGGAGCTGCAGCAAATGGTCAGCAATTTTTAATGGATG
CTTTCTTTGATTGTCAGATGGCCAGACCTATGCCTATGTACAGCGTACCTGGTCTAATTGATCATTTCTAATGTTTTATATAACCTCGACTACTCCG
TAAGGAGTAGTGAGGAAACAACCGAAGGGCGTTAGTTTATGTTTGGTGGAATACTTGATGCGGTTACTAATGTTGGTTCTAAGCTGTCTTCAGC
TTCTAGTTTCTTTACTCCTGGTGTCGGTACTGCTTTGGGCGCTGTTGGTTCTTATTTAGGTTCTACTTCTGCTAATAAAGCTAATCAGGAGATGGCT
CAGAGGCAAATGGATTTTCAAGCCGATATGAGTGGAACAAGTTACCAGCGTGCTGTTAAAGATTTAGAAGCTGCTGGTTTATCTCCTATGTTAGC
CTATCAACGTGGTGGTGCTTCTACCCCATCTGGTTCAACTGCTACTATGGAAAATGTTTTAGGTAATGCAACTAATTCAGCTATTAATACTGCTTCT
ATGATGCAACAGATTCGTAATGCATCAGAAACAGAAAAGCAGATTATCGCCCAGACTGAAGCTACTGAAGCTGGTACCGCTAATACTAGGGCTG
ATACTGTTAATAAGTTGCTTACTGCTCCTAATATTACAGCCGAAAATAAACGTATTTTGGCTGATATTGCTTTAAAGAATACGACTGCGGATTTAA
CATCCGCTCAGTCATATAATACTAAGAGGCTATTGGCTCCATCCCCAGCTATTTGGTCTAGGGGTATCGATGCTTCGAAAGAAATTTTTGATAAAC
TCAAAAATAATCCTAATCAACTAACCCCTTGGGGAATTGGAGTCAAATAATGAGTAAAGCGAATTTGCCATTTGTACGTAATCCGTACAACTATG
ATAAAGATGAAGCATCGGTAAACGATGCGTTGCTGTGTCAAGACCCAAGTCTTGCTCAACAGCATATGAAAGATGAATGTGACATTAATGTCAT
CATTGAACGTTTCGGGGTTACAGGGGAACTTCCAACGGCCCCTGTATCGCCTCAATACGGCGATTTAGTGGTGTTACTGATTACCATTCTGCGTT
GAATCAAATTAACGCAACTATGGACGATTTCATGGCTCTGCCAGCGAAATTAAGAGTCCGATTTGACCATGATCCTGTCAAATTATTGGAGTTCC
TTGAGAACGACCAGAATCGTGATGAAGCGATTCAATTGGGTCTTATTGATGGACAACCTGTGGTTGAACCCATCGTTTCTACAGAAACACCTAAG
GCCGAAGGATGAAATCCTGAGGCCAGCACAGTTACTCTACTTGATGTAACTGTGCTAGGTGACACCAAACCACTATTTTAACTACGGAGTTCATC
ATGTTACGAAGAAAGCCAGTAAACAAATATAAATCTGCAAAGTCATTTCGCAGAACTGCTAGTAAGACGAAGTCAATTAATATGAGACACGCTC
CCCAGCGTGGTGGCTATCGTTTGTAATTATGGCCTGTTATAAGCCCTTAACGGCTTATCAATGCAGTGACAGGTCTATAATTTGGCGGGAAATAC
CGGGTGCGGATGTAGTCCGTACCCTATCATTGCCTTGTGGTCAGTGTGTTGGTTGTCGCCTTGAACGCTCACGTCAGTGGGCGATTCGTTGTATG
CATGAGGCACAAATGCATACTAGTAATTGTTTTATTACTTTGACATATGCTCCAGAGCATTGTCCTAAGGATATGTCATTGGATTACAATGATTAT
CAGCTTTTTATGAAGCGGTTACGTAAGCGTTTTACTGGGAAAACGATACGTTTTTATATGGCAGGTGAATATGGTGAATCTTTTGATCGTCCTCAT
TTCCATGCTTGTCTGTTTGGTCTTGATTTTCCGGATAAGAAAATATTTAAAAGAACGCAGACTGGCTCTATCCTCTACACGTCAGAGATTTTGGAA
GAATTGTGGCCGTTTGGCTATTCTACAATTGGTGATGTTACTTTTGAGTCTGCTGCTTATGTTGCAAGATATATTATGAAGAAGATTAATGGGGTT
ACTGTCAATGAAAACCACGAAGTGGTTGATGCGGGTGCCCATTATCAATATTGTGATTTAGAGACTGGTGAGATAATTCAGCGTAAGCCAGAAT
TTAATAAGATGTCATTGAAGCCCGGTATCGGGCAATCGTGGTTAGATAAGTACATGTCAGACGTTTATACGTCAGACCACGTTGTGGTGCGTGG
CAAAAAGTGCCGTCCACCACGGTTTTATGATAATAAATTTAAGTTGAAGTTTCCTGAAGAATTCGATATGATTCAGTTTGCCAGAGAGATGGAAG
GTCGATCTAGGCATGAGGACAACACGCTTGAGCGACTTGCTGTTAAGGAAAAAGTTGCGTTGGCTAAGTTGTCATTGTTAAAACGTACTATTTAA
GGAGTTTTTATGAAGATGGTTATTGTTTCTATTAAGGATACTGCTGCAGATGCTTTTGGTCGTCCAGCTTATGTTGCATCTGAAGGTGTTGCAGTA
CGTCAGTTTCAGGATGAAGTCAATCGAG

>000153F|arrow

TTGTAAACGATAATCTTGTGGAGTTACACCAAAATGAGCACGTAACAATTCTGTATAGCGTGTACCACCTCGCGCATCGCGCTCTAACAAACGCT
GAATCTGGAAAGACTGACGTACTGGTTAATAGTTGCAGCCGTAGCATCACTTAAATCGGCATACAAACCAGTACCAGCAGTACCAGCGTTATTAC
TACGATACACAGCATGTGTAGTTGAATTAGCATAAATCTGCTTCAAAGCACCAGCACCGTCAACTAATGACAACGTTGTTGAATCATTAGTAACA
GACGTCTTAATAGGAGCAGACGTGCCTAAAGGCAACGTAACTGCATCGCCTTTCTGAGGCCAAGGCAAGGCACCAGTAAAATAATCCTTACGCT
TACCGCGTCGAACCATAGCATAATCACTCGGAGTATCAGGACCGTCACCCTTGTGAACGGTAACAGAATTTTGTAAATTCTCATCCCTAAACCACT

CGTTATAAATCAAATTATAAGCACGTAACGGTAACGCGTTATGCGTAACCGTATTAGTACCAGTAATCTGACCAGCCGTAGGCAAACCAAAATGA
TCAAAAATAGAACCTACTGCATAACCACCAGCAGTAGAAGTAATCTGTGGAACTACATACGAAATAGAATCACCTGGGTTCGCTTGCTCACCCAT
AAACTTAACCCAATTGTTCCAAACTAATCTATTTGGAACAAAGAAAAAGAAAGTATCTAAATGCAAATTGTCCATAACTGGAAACAAAGGCGTTG
CCAAACGGGCAAACATTGTAGCCTTTACATTGTGCATGTCCCCTGGGAGCACTTCATCACAATAAATAGGAACTAGATAACCACCATCAAAAGTA
GTTTTATGCGCATATTGCGTATCAAAACTAGAACGCGGAATTTCCGCTTTAGGAACCATAGCAAAACTATGTGAGCTTACTGACTTATTACGATGC
ATAACAATCTCCCGAAGTATTCCGAACCACTAGCAAGCTAGTGATCCGGCTTAAAAAAAACATTACTCGCCATCGCGAATCATAACATCCTTAGCT
CTAGAAATAAGCTTGGGAGAACCAAGCAAATCCATAGTACCAGAATTATCATCAAACGTACCAAAATAATATAACTGAAAATCATCAGGGTGTTT
ATATAACTGATTATCATCGCTAGCGCGATTAACTTCGTCCTGAAACTGACGAACAGCAACACCCTCAGATGCAACAAAAGCTGGACGAACCATAC
GCACCAGCTGCAGTATCTAAAATAGAAACAATAACATCTTCATAAAAACTCCTTTAAATCTTACGTTTTAAAAGCGATAACTTAGCCAAAGCGACT
TTTTCCTTTACAGCCAAACGCTCTAAAGTGTTATCTTCAAAATGTGAGCGACCTTCTAGCTCACGAGCATATTGTATACCATCAAATTCTTCTGGAA
ACAATACTTTAAAACTTATTATCATAAAACCGTGGTGGTCGGCACGTTTTGCCACGCACCACAACAGAGTCAGTCGTATAAAACGTCTGACATGA
CTTATCAAACCAAGCCTGACCAATGCCAGGCTTAAGAGACATCTTATTAAATTCAGGCTTACGCTGAATAATCTCACCAGTATCTAAATCACAATA
CTGATAATGCGCATCTGCATCAACCACTTCGTGGTTCTCATTAACGGTTTTACCGTTAATCTTCTTCATAATATATCGAGCAACATAAGCAGCAGAT
TCAAATTGACATCACCAATAGAACTATAGCCATACGGCCAAAGTTCTTCAAGTATCTTTGACGTATATAAGATAGACCCAGTCTGCGTTCTTTTGA
AAAACTTCTTATCTTCAAAATCAAGCCCAAAGATACAAGCATGGAAATGAGGACGATCGAAAGACTCACCATATTCACCTGCCATATAAAAACGA
ATAGTCTTGCCAGTATAGCGTTTTCTCAATCGTTTCATAAACAATTGAAAATCTTCATAATGCAAAGACATATCCTTAGGACAATGCTCTGGAGCA
TATGTCAAAGTAATAAAACAATTACTAGTATGCATTTGTGCCTCATGCATACATCTAACGGCCCACTGACGGGACCGTTCAAGGCGACAACCACA
CACTGACCACAAGGCAATGACAAGGTACGGACTACGTCCGCCCCTGGTATCTCCCGCCAAATAATAGACTTGTCAGCGCATTGATAAGCCGTTAA
CGGCTTATAACACGCCATAAATTACAGTCTAAAACCACCGCGCTGCGGTGAAGTACGCATATTAATGCTCTTGGTCTTGCTTACGCCACGACGAA
ACTTCTTAGCTGCGCCATGCTTGCTCATTGGTTTTCTATAAAGGCTCATAACATTGCACTCCGTAGTTAATAATGTGGTTTTGGTGTCACCTAGCAC
AGTTACATCAAGTAGAGTAACTGTGCTGCCATCCGCTTACGCGTCTGGCTTAGGTGTTTCTACTGCAGAAACGACGGGTTCAACCACAGGTTTAC
CGTCAATAAGACCAATCTGAATCGCTTCATCACGATTCTCTTCATTCTGTAAATAATTTAACAAAGCATTAGGATCGTTATCAAACTTAGCCCTAAT
CTTAGCTGGCAAAGCCATAAAAGCCTCATCAGAGGCACGAATAGCATTCAATGCGGTGTGATAGTCAGATACACCGCTAAAATCGCCATATGAT
GGCTCTAATGGCGCCTGGGGCAATTGCCCAGTAACGCCAAAACGCTCAACTATAATATTAATATCACATTCGTCCTCTCATGTGTTGTTGAGCCAA
ACTCGGATCTTTACATTCAAGACCAGTCTCTTGTGAAACAAGAGCCATATCATAATTGTACGGATTACGTACAAAAGGTAAATTCGTTTTACTCAT
TTTCTACGACCTTCCAAGGACCAAGGAAAAGACTTATTCTTTTCCAAAACTTATCAAAAATAGACTTACTACCTTTCTTAATATCGCGATACCAATA
TGGATCGCTAGACGGTGTTCGTAACTCTAAATTATTCTTAGCATCAATATCAGTCTTAATTGTTGATGCAGAATTAGCATTAACACGAGAACCAGC
CTCGGCCTGTTCCAAATAAAAACGAGCCGGTTCTTGTAAAACACGCTGAGAAGCTAAATCAGCTTCTTCAGCAACCTTCCTAGCAGTCTGAACAT
TTAATTCACGTTGAGATTTAGCAACTTCCATCTGCTCACGCAATAAATCACTCTCAACATCACGCTTAACAGCTCCACTCAAATCAGACTTAACTGG
AGAAGTAGAAGATGCAGTAGCACCACTCGGAACAGTAGAACCACCTTTAGAATATGCAAGCATAGGACTCAAACCAGCCTTATTCAAATCAGTA
ACCATACGCTGATATTGCGTATTGGACATATCTTCTTGAAAGCGTCTATTTGCTTGCGCTTCAGCAGTATTATATTTTTGCTCTTCATTCTGACTCCA
AGCAGAAGTAGCAAAATCAAATAAACCACCAGCAACTGAATCAAACATACCCATACTAACGCGCTCCGCTTGTTTGCTGACTACTGGTTTCCCAG
TAGTCCAGCTTATATTACATTAGAAATGGTCAATCAAGCCAGGTACAGAATACATCGGCATTGGACGAGCCATCTTACAATCAAAAAACGCATCC
ATCAAAAACTGCTGACCATTAGCAGATGCACCAACAGCAGTAGTACGATCAATAGGTGGCGTTTCTTGAATAAACGTAGAATTCAAAGTCGGCA
ACGAAGTGAACTTCTGAGCATAATGCCAAGGGTCAATCGTACCAGCTGAAGTCGACTTAAACAAACCTGTAATCTGAGAAGGTTTGTAACGGTA
CTCTGCCCAACGTTCCTGGTATCCAAATACATCATCATCAGTTGAAGTACCAGTAACATAGATTTCCTTATTAAGAACAGCCTGTTCACCTAAATG
AGCAAATACAGGGAAATAGAAATCATAACGTGTCTCACGAGACCACATCTTAGGTAAACCTTGCTGATATGTTAGATCAGCTCTTACGTTACCAA
ACCAATTATGTATCCATGTTCTTGAGCATGATACGTAAAACCATGTCCACTAGCCAACGCAGTACCCATTGCAGCCAAGTTACCAAGCGGAGTAG
CACCGCCAGAAATCGAAGTAGCAGACGTTTGAGCAATAGGATTAACGTTGACATAAGTAGAACCACCACCAATATATTCAGGACGTTGTAACGA
TAATCTTGTGGAGTTACACCAAAATGAGCACGTAACAATTCTGTATAGCGTGTACCACCTCGCGCATCGCGCTCTAACAAACGCTGAATCTGGAA
AGACTGACGTAACTGGTTAATAGTTGCAGCCGTAGCATCACTTAAATCGGCATACAAACCAGTACCAGCAGTACCAGCGTTATTACTACGATACA
CAGCATGTGTAGTTGAATTAGCATAAATCTGCTTCAAAGCACCAGCACCGTCAACTAATGACAACGTTGTTGAATCATTAGTAACAGACGTCTTA
ATAGGAGCAGACGTGCCTAAAGGCAACGTAACTGCATCGCCTTTCTGAGGCCAAGGCAAGGCACCAGTAAAATAATCCTTACGCTTACCGCGTC
GAACCATAGCATAATCACTCGGAGTATCAGGACCGTCACCCTTGTGAACGGTAACAGAATTTTGTAAATTCTCATCCCTAAACCACTCGTTATAAA
TCAAATTATAAGCACGTAACGGTAACGCGTTATGCGTAACCGTATTAGTACCAGTAATCTGACCAGCCGTAGGCAAACCAAAATGATCAAAATA
GAACCTACTGCATAACCACCAGCAGTAGAAGTAATCTGTGGAACTACATACGAAATAGAATCACCTGGGTTCGCTTGCTCACCCATAAACTTAAC
CCAATTGTTCCAAACTAATCTATTTGGAACAAAGAAAAAGAAAGTATCTAAATGCAAATTGTCCATAACTGGAAACAAAGGCGTTGCCAAACGG
GCAAACATTGTAGCCTTTACATTGTGCATGTCCCCTGGGAGCACTTCATCACAATAAATAGGAACTAGATAACCACCATCAAAAGTAGTTTTGAT
ACGCAATATGCGCATAAAACTACTTTTGATGGTGGTTATCTAGTTCCTATTTATTGTGATGAAGTGCTCCCAGGGGACATGCACAATGTAAAGGC
TACAATGTTTGCCCGTTGGGCAACGCCTTTGTTTCCAGTTATGGACAATTTGCATTTAGATACTTTCTTTTTCTTTGTTCCAAATAGATTAGTTTGGA
ACAATTGGGTTAAGTTTATGGGTGAGCAAGCGAACCCAGGTGATTCTATTTCGTATGTAGTTCCACAGATTACTTCTACTGCTGGTGGTTATGCA
GTAGGTTCTATTTTTGATCATTTTGGTTTGCCTACGGCTGGTCAGATTACTGGTACTAATACGGTTACGCATAACGCGTTACCGTTACGTGCTTAT

AATTTGATTTATAACGAGTGGTTTAGGGATGAGAATTTACAAAATTCTGTTACCGTTCACAAGGGTGACGGTCCTGATACTCCGAGTGATTATGC

TATGGTTCGACGCGGTAAGCGTAAGGATTATTTACTGGTGCCTTGCCTTGGCCTCAGAAAGGCGATGCAGTTACGTTGCCTTTAGGCACGTCTG

CTCCTATTAAGACGTCTGTTACTAATGATTCAACAACGTTGTCATTAGTTGACGGTGCTGGTGCTTTGAAGCAGATTTATGCTAATTCAACTACAC

ATGCTGTGTATCGTAGTAATAACGCTGGTACTGCTGGTACTGGTTTGTATGCCGATTTAAGTGATGCTACGGCTGCAACTATTAACCAGTTACGT

CAGTCTTTCCAGATTCAGCGTTTGTTAGAGCGCGATGCGCGAGGTGGTACACGCTATACAGAATTGTTACGTGCTCATTTTGGTGTAACTCCACA

AGAT

>000220F|arrow

CCAAACCACTATTTTAACTACGGAGTTCATCATGTTACGAAGACCAGTAAACAATATAAATCTGCAAAGTCATTTCGCAGAACTGCTAGTAAGAC

GAAATCAATTAATATGAGACACGCTCCCCAGCGTGGTGGCTATCGTTTGTAATTATGGCCTGTTATAAGCCCTTAACGGCTTATCAATGCAGTGA

CAGGTCTATAATTTGGCGGAAATACCGGTGCGGATGTAGTCCGTACCCTATCATTGCCTTGTGGTCAGTGTGTTGGTTGTCGCCTTGAACGCTCA

CGTCAGTGGGCGATTCGTTGTATGCATGAGGCACAAATGCATACTAGTAATTGTTTTATTACTTTGACATATGCTCCAGAGCATTGTCCTAAGGAT

ATGTCATTGGATTACAATGATTATCAGCTTTTTATGAAGCGGTTACGTAAGCGTTTTACTGGGAAAACGATACGTTTTTATATGGCAGGTGAATAT

GGTGAATCTTTTGATCGTCCTCATTTCCATGCTTGTCTGTTTGGTCTTGATTTTCCGGATAAGAAAATATTTAAAAGAACGCAGACTGGCTCTATCC

TCTACACGTCAGAGATTTTGGAAGAATTGTGGCCGTTTGGCTATTCTACAATTGGTGATGTTACTTTTGAGTCTGCTGCTTATGTTGCAAGATATA

TTATGAAGAAGATTAATGGGTTACTGTCAATGAAAACCACGAAGTGGTTGATGCGGGTGCCCATTATCAATATTGTGATTTAGAGACTGGTGAG

ATATTCAGCGTAAGCCAGAATTTAATAAGATGTCATTGAAGCCCGTATCGGGCAATCGTGGTTAGATAAGTACATGTCAACGTTTTACGTCAGAC

CACGTTGTGGTGCGTGGCAAAAAGTGCCGTCCACCACGGTTTTATGATAATAAATTTAAGTTGAAGTTTCCTGAAGAATTCGATATGATTCAGTT

TGCCAAGAGATGGAAGGTCGATCTAGGCATGAGGACAACACGCTTGAGCGACTTGCTGTTAAGGAAAAGTTGCGTTGGCTAAGTTGTCATTGTT

AAAACGTACTATTTAAGGAGTTTTTATGAAGATGGTTATTGTTTCTATTAAGGATACTGCTGCAGATGCTTTTGGTCGTCCAGCTTATGTTGCATC

TGAAGGTGTTGCAGTACGTCAGTTTCAGGATGAAGTCAATCGAGCTAGCGAAGATAATCAGTTGTATAAACATCCTGATGATTTTCATATGTTCT

ATTTGGGTCTTTTTGACGATGCCACTGGTGTTTTTGAACTACTGGAAAGCCCTAAGTTGATTGCTCGTGCAAAAGATGTAATGATTCGCGAAGGC

GAGTAAGGTTTTTTTATACCGTATCACTCGAAAGAGTGGTACGGAACTACGGGAGATGTTTATGTTTCGCAATAAGTCAGTAAGTACGCATTCA

TTTGCTATGGTTCCTAAAGCGGACATTCCCCGCTCTAGTTTTAATACTCAATATGCTCATAAAACCACGTTTGATGCTGGTTTTTTAGTTCCTATTTA

TTGTGATGAAGTATTGCCTGGCGATACTCATCGTGTAAAGATGACTGCATTTGCACGTTTGGCCACACCGTTATTTCCTGTGATGGACAACTTGCA

TCTTGATACTTTCTTTTTCTTTGTACCTAATCGTTTACTTTGGAACATTGGCCAAAGTTTATGGGTGAACAACGAATCCTGGTGATTCTATTTCTTTT

GTAGTGCCTACTATTACTAGTCCTGCTGGTGGTTATGCTGTTTGTTCAATTTTTGATTATTTTGGTTTACCTACTGCTGGTCAGATTACTGGCGCTA

ATACAGTAACGCATAATGTTTTGCCGTTACGTGCTTATAATGAGATTTATAACGAATGTTTAGAGATGAAAACTTACAGAATTCTGTAACGTTAAA

TCTTGGTGATTCAGGTGATGTTCCTGCTAACTATACACTTTTGAGACGTGGTAAGCGTAAAGATTATTTTACTGTGCATTGCCTTGGCCACAGAAG

GTGCTTCTGTTTCTTTACCGTTAGGAACACGTGCTAATATTTATTCTGACATACCAGCTGGCAATGGTACTGCTGGTTATAGTGTTTTTCAAACTGC

TGTTGGTGCTTTAAGAGAATTAAATTCAGCTTCTAATACTTTGTCTAATAGTACAAATGCTGGTGTTGCTACTAATCAGTTATACGCTGATTTGTCT

ACTGCTACTGCTGCGACTATTAACCAACTTCGTCAATCTTTCCAGATTCAGAAGTTATTGGAGCGCGATGCACGTGGTGGTACTCGTTATACTGAG

TTACTACGTGCTCACTTTGGAGTAACTCCACAGGATTATCGTTTACAACGTCCTGAATTATTGGTGGGTTCGACCCTTGTTAATGTTAATCCGATT

GCTCAGACTTCTGCAACGTCGGTTACTGGTTCTGCTACTCCGCAAGGTAACCTTGCTGCAATGGGTACTGCATTGGCTCAGGGACACGGCTTTAC

GTATGCTGCTCAAGAACATGGATACATTATCGGATTAGTTTCTGTACGTGCTGACCTCACATATCAACAGGGTCTTCCTAAGATGTGGTCTAGGTC

TACACGATATGACTTTTATTTCCCAGTATTTGCCACTTTGGGTGAGCAAGCTATTTTGAACAAAGAAATTTATGTTCAAGGTACTGCAGCCGACAA

TGATGTATTTGGTTATCAAGAACGTTGGGCGGAGTATCGTTACAAACCTTCTCAAATTACTGGTTTCTTTAGGTCTACTTCTGCTGGCACTATTGA

TGCTTGGCATTATGGACAGCGATTTACTTCTCTTCCTACGTTGAATTCAACGTTTATTCAAGAGACCCCTCCAGTTGCTCGTACTACGGCGGTCGG

AGCTGCAGCAAATGGTCAGCAATTTTTAATGGATGCTTTCTTTGATTGTCAGATGGCCAGACCTATGCCTATGTACAGCGTACCTGGTCTAATTGA

TCATTTCTAATGTTTTATATAACCTCGACTACTCCGTAAGGAGTAGTGAGGAAACAACCGAAGGCGTTAGTTTATGTTTGGTGGAATACTTGATG

CGGTTACTAATGTTGGTTCTAAGCTGTCTTCAGCTTCTAGTTTCTTTACTCCTGGTGTCGGTACTGCTTTGGGCGCTGTTGGTTCTTATTTAGGTTC

TACTTCTGCTAATAAAGCTAATCAGGAGATGGCTCAGAGGCAAATGGATTTTCAAGCCGATATGAGTGGAACAAGTTACCAGCGTGCTGTTAAA

GATTTAGAAGCTGCTGGTTTATCTCCTATGTTAGCCTATCAACGTGGTGGTGCTTCTACCCCATCTGGTTCAACTGCTACTATGGAAAATGTTTTA

GGTAATGCAACTAATTCAGCTATTAATACTGCTTCTATGATGCAACAGATTCGTAATGCATCAGAAACAGAAAAGCAGATTATCGCCCAGACTGA

AGCTACTGAAGCTGGTACCGCTAATACTAGGGCTGATACTGTTATAAGTTGCTTACTGCTCCTAATATTACAGCCGAAAATAAACGTATTTTGGCT

GATATTGCTTTAAAGAATACGACTGCGGATTTAACATCCGCTCAGTCATATAATACTAAGAGGCTATTGGCTCCATCCCCAGCTATTTGGTCTAGG

GTATCGATGCTTCGAAAGAAATTTTTGATAAACTCAAAATAATCCTAATCAACTAACCCCTTGGGGAATTGGAGTCAAATAATGAGTAAAGCGAA

TTTGCCATTTGTACGTAATCCGTACAACTATGATAAAGATGAAGCATCGGTAAACGATGCGTGCTGTGTCAAGACCCAAGTCTTGCTCAACAGCA

TATGAAAGATGAATGTGACATTAATGTCATCATTGAACGTTTCGGGTTACAGGGGAACTTCCAACGGCCCCTGTATCGCCTCAATACGGCGATTT

TAGTGGTGTTACTGATTACCATTCTGCGTTGAATCAAATTAACGCAACTATGGACGATTTCATGGCTCTGCCAGCGAAATTAAGAGTCCGATTTG

ACCATGATCCTGTCAAATTATTGGAGTTCCTTGAGAACGACCAGAATCGTGATGAAGCGATTCAATTGGGTCTTATTGATGGACAACCTGTGGTT

GAACCCATCGTTTCTACAGAAACACCTAAGGCCGAAGGATGAAATCCTGAGGCCAGCACAGTTACTCTACTTGATGTAACTGTGCTAGGTGACA

>000130F|arrow

CCTCTTAGTATTATATGACTGAGCGGATGTTAAATCCGCAGTCGTATTCTTTAAAGCAATATCAGCCAAAATACGTTTATTTTCGGCTGTAATATTA
GGAGCAGTAAGCAACTTATTAACAGTATCAGCCCTAGTATTAGCGGTACCAGCTTCAGTAGCTTCAGTCTGGGCGATAATCTGCTTTTCTGTTTCT
GATGCATTACGAATCTGTTGCATCATAGAAGCAGTATTAATAGCTGAATTAGTTGCATTACCTAAAACATTTTCCATAGTAGCAGTTGAACCAGAT
GGGGTAGAAGCACCACCACGTTGATAGGCTAACATAGGAGATAAACCAGCAGCTTCTAAATCTTTAACAGCACGCTGGTAACTTGTTCCACTCAT
ATCGGCTTGAAAATCCATTTGCCTCTGAGCCATCTCCTGATTAGCTTTATTAGCAGAAGTAGAACCTAAATAAGAACCAACAGCGCCCAAAGCAG
TACCGACACCAGGAGTAAAGAAACTAGAAGCTGAAGACAGCTTAGAACCAACATTAGTAACCGCATCAAGTATTCCACCAAACATAAACTAACG
CCCTTCGGTTGTTTCCTCACTACTCCTTACGGAGTAGTCGAGGTTATATAAAACATTAGAAATGATCAATTAGACCAGGTACGCTGTACATAGGCA
TAGGTCTGGCCATCTGACAATCAAAGAAAGCATCCATTAAAAATTGCTGACCATTTGCTGCAGCTCCGACCGCCGTAGTACGAGCAACTGGAGG
GGTCTCTTGAATAAACGTTGAATTCAACGTAGGAAGAGAAGTAAATCGCTGTCCATAATGCCAAGCATCAATAGTGCCAGCAGAAGTAGACCTA
AAGAAACCAGTAATTTGAGAAGGTTTGTAACGATACTCCGCCCAACGTTCTTGATAACCAAATACATCATTGTCGGCTGCAGTACCTTGAACATA
AATTTCTTTGTTCAAAATAGCTTGCTCACCCAAAGTGGCAAATACTGGGAAATAAAAGTCATATCGTGTAGACCTAGACCACATCTTAGGAAGAC
CCTGTTGATATGTGAGGTCAGCACGTACAGAAACTAATCCGATAATGTATCCATGTTCTTGAGCAGCATACGTAAAGCCGTGTCCCTGAGCCAAT
GCAGTACCCATTGCAGCAAGGTTACCTTGCGGAGTAGCAGAACCAGTAACCGACGTTGCAGAAGTCTGAGCAATCGGATTAACATTAACAAGG
GTCGAACCTCCACCAATATATTCAGGACGTTGTAAACGATAATCCTGTGGAGTTACTCCAAAGTGAGCACGTAGTAACTCAGTATAACGAGTACC
ACCACGTGCATCGCGCTCCAATAACTTCTGAATCTGGAAAGATTGACGAAGTTGGTTAATAGTCGCAGCAGTAGCAGTAGACAAATCAGCGTAT
AACTGATTAGTAGCAACACCAGCATTTGTACTATTAGACAAAGTATTAGAAGCTGAATTTAATTCTCTTAAAGCACCAACAGCAGTTTGAAAAAC
ACTATAACCAGCAGTACCATTGCCAGCTGGTATGTCAGAATAAATATTAGCACGTGTTCCTAACGGTAAAGAAACAGAAGCACCCTTCTGTGGCC
AAGGCAATGCACCAGTAAAATAATCTTTACGCTTACCACGTCTCAAAAGTGTATAGTTAGCAGGAACATCACCTGAATCACCAAGATTTAACGTT
ACAGAATTCTGTAAGTTTTCATCTCTAAACCATTCGTTATAAATCTCATTATAAGCACGTAACGGCAAAACATTATGCGTTACTGTATTAGCGCCA
GTAATCTGACCAGCAGTAGGTAAACCAAAATAATCAAAAATTGAACAAACAGCATAACCACCAGCAGGACTAGTAATAGTAGGCACTACAAAAG
AAATAGAATCACCAGGATTCGTTTGTTCACCCATAAACTTTGGCCAATTGTTCCAAAGTAAACGATTAGGTACAAAGAAAAAGAAAGTATCAAGA
TGCAAGTTGTCCATCACAGGAAATAACGGTGTGGCCAAACGTGCAAATGCAGTCATCTTTACACGATGAGTATCGCCAGGCAATACTTCATCACA
ATAAATAGGAACTAAAAAACCAGCATCAAACGTGGTTTTATGAGCATATTGAGTATTAAAACTAGAGCGGGGAATGTCCGCTTTAGGAACCATA
GCAAATGAATGCGTACTTACTGACTTATTGCGAAACATAAACATCTCCCGTAGTTCCGTACCACTCTTTCGAGTGATACGGTATAAAAAAAACCTT
ACTCGCCTTCGCGAATCATTACATCTTTTGCACGAGCAATCAACTTAGGGCTTTCCAGTAGTTCAAAAACACCAGTGGCATCGTCAAAAAGACCC
AAATAGAACATATGAAAATCATCAGGATGTTTATACAACTGATTATCTTCGCTAGCTCGATTGACTTCATCCTGAAACTGACGTACTGCAACACCT
TCAGATGCAACATAAGCTGGACGACCAAAAGCATCTGCAGCAGTATCCTTAATAGAAACAATAACCATCTTCATAAAAACTCCTTAAATAGTACG
TTTTAACAATGACAACTTAGCCAACGCAACTTTTTCCTTAACAGCAAGTCGCTCAAGCGTGTTGTCCTCATGCCTAGATCGACCTTCCATCTCTCTG
GCAAACTGAATCATATCGAATTCTTCAGGAAACTTCAACTTAAATTTATTATCATAAAACCGTGGTGGACGGCACTTTTTGCCACGCACCACAACG
TGGTCTGACGTATAAACGTCTGACATGTACTTATCTAACCACGATTGCCCGATACCGGGCTTCAATGACATCTTATTAAATTCTGGCTTACGCTGA
ATTATCTCACCAGTCTCTAAATCACAATATTGATAATGGGCACCCGCATCAACCACTTCGTGGTTTTCATTGACAGTAACCCCATTAATCTTCTTCA
TAATATATCTTGCAACATAAGCAGCAGACTCAAAAGTAACATCACCAATTGTAGAATAGCCAAACGGCCACAATTCTTCCAAAATCTCTGACGTG
TAGAGGATAGAGCCAGTCTGCGTTCTTTTAAATATTTTCTTATCCGGAAAATCAAGACCAAACAGACAAGCATGGAAATGAGGACGATCAAAAG
ATTCACCATATTCACCTGCCATATAAAAACGTATCGTTTTCCCAGTAAAACGCTTACGTAACCGCTTCATAAAAAGCTGATAATCATTGTAATCCAA
TGACATATCCTTAGGACAATGCTCTGGAGCATATGTCAAAGTAATAAAACAATTACTAGTATGCATTTGTGCCTCATGCATACAACGAATCGCCC
ACTGACGTGAGCGTTCAAGGCGACAACCAACACACTGACCACAAGGCAATGATAGGGTACGGACTACATCCGCACCCGGTATTTCCCGCCAAAT
TATAGACCTGTCACTGCATTGATAAGCCGTTAAGGGCTTATAACAGGCCATAATTACAAACGATAGCCACCACGCTGGGGAGCGTGTCTCATATT
AATTGACTTCGTCTTACTAGCAGTTCTGCGAAATGACTTTGCAGATTTATATTTGTTTACTGGCTTTCTTCGTAACATGATGAACTCCGTAGTTAAA
ATAGTGGTTTGGTGTCACCTAGCACAGTTACATCAAGTAGAGTAACTGTGCTGGCCTCAGGATTTCATCCTTCGGCCTTAGGTGTTTCTGTAGAA
ACGATGGGTTCAACCACAGGTTGTCCATCAATAAGACCCAATTGAATCGCTTCATCACGATTCTGGTCGTTCTCAAGGAACTCCAATAATTTGACA
GGATCATGGTCAAATCGGACTCTTAATTTCGCTGGCAGAGCCATGAAATCGTCCATAGTTGCGTTAATTTGATTCAACGCAGAATGGTAATCAGT
AACACCACTAAAATCGCCGTATTGAGGCGATACAGGGGCCGTTGGAAGTTCCCCTGTAACCCCGAAACGTTCAATGATGACATTAATGTCACATT
CATCTTTCATATGCTGTTGAGCAAGACTTGGGTCTTGACACAGCAACGCATCGTTTACCGATGCTTCATCTTTATCATAGTTGTACGGATTACGTA
CAAATGGCAAATTCGCTTTACTCATTATTTGACTCCAATTCCCCAAGGGGTTAGTTGATTAGGATTATTTTTGAGTTTATCAAAAATTTCTTTCGAA
GCATCGATACCCCTAGACCAAATAGCTGGGGATGGAGCCAATAG

>000068F|arrow

ACGTCAGCATTACCATGGCCACAAAAAGGTGCGAGTGTCACCTTACCTTTAGGTACTACGGCTCCAATTAAATGGGATACCATTTCAGGAGACGC
AACATCAAACGATAAATTTACGGTAATTCAAACAGATCCTGGAAATACGACTGCTTTAGCTAGATATGGCAACGCTTATGGTGTTAATACTGCTG
GTGTAGTAAATAACGTTTCTAATTTATATACCGACTTATCAGAAGCAACTGCTGCAACTGTCAATCAATTAAGACAGTCATTTCAAATTCAAAAAT
TACTTGAAAGGGATGCACGTGGCGGAACACGATACACAGAAATTATCCGGAGTCACTTTGGAGTTATTTCCCCAGACGCCCGTTTACCAAAGGC

CTGAATACCTTGGAGGCGGTTCAACACCAATTAATGTTAATCCGATTGCTCAAACGTCGGGAACAAACGCTTCTGGAACGACTACCCCTTTGGGC
AACCTTGCTGCTATGGGTACTGCTCTCGCTCATAATCATGGATTTACTCAATCATTTACTGAGCATGGCGTTATTATTGGATTAGTATCCATTAGA
GCAGATCTTACTTATCAACAAGGATTAGACCGTATGTGGTCTAGATCTACACGATATGACTTTTATTTCCCAGCATTTGCTACTCTAGGCGAACAA
TCTGTTTTGCAAAAAGAAATTTATGCAACAGGAGATACTGCAGCCGACAATACTGTTTTTGGATATCAAGAACGCTGGGCGGAATATCGTTACAA
ACCATCTAAAATTACTGGTTTGTTCAAATCAACATCGGCGGGCACGATCGATGGTTGGCATTTGGCTCAAAAATTTACCGCTGCGCCTACTTTGAA
TAATACGTTTATTCAAGATACGCCTCCTGTATCACGTGTAGTAGCCGTTGGAGCAGCTGCAAATGGCCAACAATTCTTATTTGACTCATTTTTTGAT
GTCAAAATGGCAAGACCAATGCCAATGTATTCAGTACCTGGCTTAATAGACCATTTCTAATGGGACTATTTGACGGAATTGCCGATTTAATCGGC
CCTGCTATAGCTATAGGAGCTGCCCCTGCTACTGGGGGACTCTCCTTAGCTGCACTTGCACCTGCAGCAATAGGTGCAGCAGGACAATACTTTGG
AACACAAAGTCAAAACGCAGCGAGTGCAGAACAAGCGAGTAATCAACAGAGATTTCAAGCTGAAATGTCTGGAACATCATATCAACGAGCAGT
TGAAGATATGAAAAAAGCTGGGTTAAATCCCATGCTTGCGTATTCACAAGGCGGAGCCACAACACCAGCTGGAGCTATGGCCCAGATGCAAAAT
GTTCTCGGTAATGCAACTACGTCCGGAACCCAAGCTTATCAAACGGTTGCGCAAGCAAATCAAGCTATTGCTCAATCTAAACAAATTGAAGCTCA
AACAGAACTCACAAGTAATCAAACAGATAATGTACGTGCTGATACGTTAAATAAATTGGATGAAAATCCAAATATTAGAGCTCAATATAAACAAT
ACTTGCCGATACTTTCATGAAAAATGAAATAGGCAAAACATCAAGTGCTCAAGCTGCTCAAGCTTTGGCACAATCTCGTTATTCAACGAGTTAAC
AAAACTTGCTAAATCAGGGTCAGCTCCTAGTTCTAGCAAACCAATTTATCAAGACGTAAAAACATCGCCAAAGATGCGTATAGCGCATCTGGCGC
AAAACGATACATCGATAACTATCGAGGTCAACCGATTCAACAAAATCGTACAAATAACCAACCACCAATGGAATGAAAATGACAAAGATTACAG
CCCCATTTCTTCGTACTCCGTACAATTACGACACGATTGCTGCGTCAAATGAGTCAGGGCTGCATTGTGAGGATGCAACTCTGACTCAGCAGCAA
TTTGCTGAAGAATGTGATATCAATAATATTATGGAAAAGTTCGGTATGACCGGACTTATTCCTCAAACTCCTTTAACGCCTCAATATGGCGACTTT
AGTGGTGTCTATGACTACCACTCTGCTCTGAACCAGATTATGGCTTCAGACAACGAATTTATGGCTTTACCAGCCAATATTCGTGAACGATTCGCT
AATGATCCCGCGAATCTAATAGATTTTCTAGAAAACCCTGAAAATCGCAGCGAAGCTGAAAAAATGGGACTGGTAAAACCAGCCCAACCGAGGT
TTCAACCCCTGTTGGAACCTCGGAAGCACAGTTACCTACTTGATGTAACTGTGCTAGGTGACACCAAACCACAAAAACACGATAACAAGGACAG
AAAAAATGATGCGTCGCAGACCAGCAAATAAGCAAAAGTCCGCTAGGACTTTCCGTAAACATGCTTCACATACAAAACACGCAAATATGCGAAA
CTCGCCAATGCGTGGAGGCTGGAGACTCTAATAAAGTCTTCAGGCACCTCACATGCCTTGTTATCACCCTCTCAAAGCATATCAATGCTTTGACAA
ATCAATTGTTTTCGACGAAGTTCGGAAACATGACATCGTTCGATCTTTAGACCTGCCCTGTGGGCAGTGCGTTGGATGCCGTCTAGAACGATCAA
GACAATGGGCTATTCGGTGCATGCACGAAGCCCAATTGCATAAAAACAACTCATTCATAACACTCACATATGACAATACACATCTCCCAAGCGAT
GGCTCTTTGGATCACAAAGACTTTCAATTGTTCCTTAAAAGACTTAGAAAAACTCTCGCAAAAAGAGGACTTACAATCCGCTATTACATGGCTGG
AGAATATGGTGAACTCTTCGCAAGACCCCACTTCCATGCCTGTATCTTCGGATACGACTTTCCTGATAAAAATTATGGAAAAGGACTGCCTCTGGT
TCTATGTTATATAGATCCGCAGAACTTGAAGCTCTCTGGCCATTTGTTATACCACCATTGGAGATGTTACTTTTGAATCAGCCGCCTACGTGGCTA
GATACATAATGAAAAAACAAACAGGGAAAGATGCGGAATCTCATTACAAACGCATACACCCTGAAACCGGCGAATATTTAGACTTAAAGCCGGA
ATATAATAAAATGTCTTTAAAACCGGGAATCGGTAAAGACTTTTATATAAAATATACTTCGGATATATACCCGCAAGACTACGTAATACTTAGAG
GTAAAAAGGTCAAACCACCAAAATACTATGACAAAATGTTTAAAATTGACCAACCTTATGAGTATGACGAATTACTTTACATGCGGGAAAATAAT
GCTAAATTTAATTCCGAAGACAATACACCAGAACGACTATCTGCAAAAGAACAAGTCACTATGGCAAAACTTCAACTATTAAAACGTAACCTTAC
TTAGGAAATAATGAAACTTATCCTCGCTTCCGTAAAAGACCGTGCTGCTGAAGCATATGCACGACCAATGTTCGTACCTTCCTCTGGAGTAGCTAT
ACGCTCTTTTCAGATGAAATTAATCGTTCTGATACTGAAAATCAACTCTTTAATCACCCTGATGATTTCGATCTATATGAATTCGGAACATTTGACG
ATTCAACTGGGTTATTCGATTTACATGAACAACCAAAACTCCTATCATTAGGAAAACAAGTTAAACTTAAATAAAACAACCGAGGGGAAAAGAG
ATTTATCTTTCCCCCGGAACAACACTAAGGAAAAACATGCACCGCAATCAGTCAGTTAATACTCACCGCTTCGCGATGGTACCTAGAGCCGATAT
ACCACGTAGTAAATTCGATGCTCAAAAAACACATAAAACGACTTTCGATGCGGGCTATCTAATTCCTGTATATGTTGATGAAGTGCTCCCTGGGG
ACACTTTCAACTTAAAAATGACGGCATTTGCCCGTCTAGCAACGCCTTTATATCCAATCATGGATAACATGATTATGGATTCTTTCTTTTTCTTTGTA
CCCAATCGCCTTATATGGAATAACTGGCAAAAATTTATGGGTCAACAAGAAAATCCAACAGACTCAATATCTTATATTGTCCCAACACAAACAAG
CCCAACAGATGGTTATGCCGTAGGCAGCCTTCAAGACTATATGGGCTTACCAACAGTAGGCCAAATTGATACTGGCCGAACTATTACGCACTGTG
CCTTTTGGCCACGTGCATACAATCTTATCTGGAACGAATGGTTCCGAGATGAAAATTTACAAACAAGCGCAGTAGTTGATAAGGGCGATGGCCC
TGATACTTCCTCAAACTATGTGCTAAAACGTCGTGGTAAAAGACATGATTACTTT

>000132F|arrow

ACTACGATACACAGCATGTGTAGTTGAATTAGCATAAATCTGCTTCAAAGCACCAGCACCGTCAACTAATGACAACGTTGTTGAATCATTAGTAA
CAGACGTCTTAATAGGAGCAGACGTGCCTAAAGGCAACGTAACTGCATCGCCTTTCTGAGGCCAAGGCAAGGCACCAGTAAAATAATCCTTACG
CTTACCGCGTCGAACCATAGCATAATCACTCGGAGTATCAGGACCGTCACCCTTGTGAACGGTAACAGAATTTTGTAAATTCTCATCCCTAAACCA
CTCGTTATAAATCAAATTATAAGCACGTAACGGTAACGCGTTATGCGTAACCGTATTAGTACCAGTAATCTGACCAGCCGTAGGCAAACCAAAAT
GATCAAAAATAGAACCTACTGCATAACCACCAGCAGTAGAAGTAATCTGTGGAACTACATACGAAATAGAATCACCTGGGTTCGCTTGCTCACCC
ATAAACTTAACCCAATTGTTCCAAACTAATCTATTTGGAACAAAGAAAAAGAAAGTATCTAAATGCAAATTGTCCATAACTGGAAACAAAGGCGT
TGCCAAACGGGCAAACATTGTAGCCTTTACATTGTGCATGTCCCCTGGGAGCACTTCATCACAATAAATAGGAACTAGATAACCACCATCAAAAG
TAGTTTTATGCGCATATTGCGTATCAAAACTAGAACGCGGAATTTCCGCTTTAGGAACCATAGCAAAACTATGTGAGCTTACTGACTTATTACGAT
GCATAACAATCTCCCGAAGTATTCCGAACCACTAGCAAGCTAGTGATCCGGCTTAAAAAAAAACATTACTCGCCATCGCGAATCATAACATCCTT

AGCTCTAGAAATAAGCTTGGGAGAACCAAGCAAATCCATAGTACCAGAATTATCATCAAACGTACCAAAATAATATAACTGAAAATCATCAGGG
TGTTTATATAACTGATTATCATCGCTAGCGCGATTAACTTCGTCCTGAAACTGACGAACAGCAACACCCTCAGATGCAACAAAGCTGGACGACC
ATACGCACCAGCTGCAGTATCTAAAATAGAAACAATAACCATCTTCATAAAAACTCCTTAAATCTTACGTTTTAAAAGCGATAACTTAGCCAAAGC
GACTTTTTCCTTTACAGCCAAACGCTCTAAAGTGTTATCTTCAAAATGTGAGCGACCTTCTAGCTCACGAGCATATTGTATACCATCAAATTCTTCT
GGAAACAATACTTTAAACTTATTATCATAAAACCGTGGTGGTCGGCACTTTTTGCCACGCACCACAACAGAGTCAGTCGTATAAACGTCTGACAT
GAACTTATCAAACCAAGCCTGACCAATGCCAGGCTTAAGAGACATCTTATTAAATTCAGGCTTACGCTGATAATCTCACCAGTATCTAAATCACAA
TACTGATAATGCGCATCTGCATCAACCACTTCGTGGTTCTCATTAACGGTTTTACCGTTAATCTTCTTCATAATATATCGAGCAACATAAGCAGCAG
ATTCAAAATTGACATCACCAATAGAACTATAGCCATACGGCCAAAGTTCTTCAAGTATCTTTGACGTATATAAGATAGACCCAGTCTGCGTTCTTT
TGAAAAACTTCTTATCTTCAAAATCAAGCCCAAAGATACAAGCATGGAAATGAGGACGATCGAAAGACTCACCATATTCACCTGCCATATAAAAA
CGAATAGTCTTGCCAGTATAGCGTTTCTCAATCGTTTCATAAACAATTGAAAATCTTCATAATGCAAAGACATATCCTTAGGACAATGCTCTGGAG
CATATGTCAAAGTAATAAAACAATTACTAGTATGCATTTGTGCCTCATGCATACATCTAACGGCCCACTGACGGGACCGTTCAAGGCGACAACCA
ACACACTGACCACAAGGCAATGACAAGGTACGGACTACGTCCGCCCCTGGTATCTCCCGCCAAATAATAGACTTGTCAGCGCATTGATAAGCCGT
TAACGGCTTATAACACGCCATAAATTACAGTCTAAAACCACCGCGCTGCGGTGAAGTACGCATATTAATGCTCTTGGTCTTGCTTACGCCACGAC
GAAACTTCTTAGCTGCGCCATGCTTGCTCATTGGTTTTCTATAAAGGCTCATAACATTGCACTCCGTAGTTAATAATGTGGTTTTGGTGTCACCTA
GCACAGTTACATCAAGTAGAGTAACTGTGCTGCCATCCGCTTACGCGTCTGGCTTAGGTGTTTCTACTGCAGAAACGACGGGTTCAACCACAGGT
TTACCGTCAATAAGACCAATCTGAATCGCTTCATCACGATTCTCTTCATTCTGTAAATAATTTAACAAAGCATTAGGATCGTTATCAAACTTAGCCC
TAATCTTAGCTGGCAAAGCCATAAAAGCCTCATCAGAGGCACGAATAGCATTCAATGCGGTGTGATAGTCAGATACACCGCTAAAATCGCCATAT
GATGGCTCTAATGGCGCCTGGGCAATTGCCCAGTAACGCCAAAACGCTCAACTATAATATTAATATCACATTCGTCTCTCATGTGTTGTTGAGCCA
AACTCGGATCTTTACATTCAAGACCAGTCTCTTGTGAAACAAGAGCCATATCATAATTGTACGGATTACGTACAAAAGGTAAATTCGTTTTACTCA
TTTTCTACGACCTTCCAAGGACCAAGGAAAAGACTTATTCTTTTCCAAAAACTTATCAAAAATAGACTTACTACCTTTCTTAATATCGCGATACCAA
TATGGATCGCTAGACGGTGTTCGTAACTCTAAATTATTCTTAGCATCAATATCAGTCTTAATTGTTGATGCAGAATTAGCATTAACACGAGAACCA
GCCTCGGCCTGTTCCAAATAAAAACGAGCCGGTTCTTGTAAAACACGCTGAGAAGCTAAATCAGCTTCTTCAGCAACCTTCCTAGCAGTCTGAAC
ATTTAATTCACGTTGAGATTTAGCAACTTCCATCTGCTCACGCAATAAATCACTCTCAACATCACGCTTAACAGCTCCACTCAAATCAGACTTAACT
GGAGAAGTAGAAGATGCAGTAGCACCACTCGGAACAGTAGAACCACCTTTAGAATATGCAAGCATAGGACTCAAACCAGCCTTATTCAAATCAG
TAACCATACGCTGATATTGCGTATTGGACATATCTTCTTGAAAGCGTCTATTTGCTTGCGCTTCAGCAGTATTATATTTTTGCTCTTCATTCTGACTC
CAAGCAGAAGTAGCAAAATCAAATAAACCACCAGCAACTGAATCAAACATACCCATACTAACGCGCTCCGCTTGTTTGCTGACTACTGGTTTCCC
AGTAGTCCAGCTTATATTACATTAGAAATGGTCAATCAAGCCAGGTACAGAATACATCGGCATTGGACGAGCCATCTTACAATCAAAAAACGCAT
CCATCAAAAACTGCTGACCATTAGCAGATGCACCAACAGCAGTAGTACGATCAATAGGTGGCGTTTCTTGAATAAACGTAGAATTCAAAGTCGG
CAACGAAGTGAACTTCTGAGCATAATGCCAAGGGTCAATCGTACCAGCTGAAGTCGACTTAAACAAACCTGTAATCTGAGAAGGTTTGTAACGG
TACTCTGCCCAACGTTCCTGGTATCCAAATACATCATCATCAGTTGAAGTACCAGTAACATAGATTTCCTTATTAAGAACAGCCTGTTCACCTAAAT
GAGCAAATACAGGGAAATAGAAATCATAACGTGTCTCACGAGACCACATCTTAGGTAAACCTTGCTGATATGTTAGATCAGCTCTTACGTTTACC
AAACCAATTATGTATCCATGTTCTTGAGCATGATACGTAAAACCATGTCCACTAGCCAACGCAGTACCCATTGCAGCCAAGTTACCAAGCGGAGT
AGCACCGCCAGAAATCGAAGTAGCAGACGTTTGAGCAATAGGATTAACGTTGACATAAGTAGAACCACCACCAATATATTCAGGACGTTGTAAA
CGATAATCTTGTGGAGTTACACCAAAATGAGCACGTAACAATTCTGTATAGCGTGTACCACCTCGCGCATCGCGCTCTAACAAACGCTGAATCTG
GAAAGACTGACGTAACTGGTTAATAGTTGCAGCCGTAGCATCACTTAAATCGGCATACAAACCAGTACCAGCAGTACCAGCGTTATT

>000084F|arrow

AGAGCGTTTGGCTGTAAAGGAAAAAGTCGCTTTGGCTAAGTTATCGCTTTTAAACGTCAGATTTAAAGGAGTTTTATGAAGATGGTTATGTTTCT
ATTTTAGATTACTGCGCTGGTGCGTAGGTCGTCCAGCTTTTTGTTGCATCTGAGGTGTTGCTGTTCGGCAGTTTCAGGACGAAGTAATCGCGCTA
GCGATGATAATCAGTTATATAAACACCCTGATGATTTTCAGTTATATATTTGGTACGTTTGATGATAATTCTGGTACTATGGATTTGCTTGGTTCTC
CCAAGCTTATTTCTAGAGCTAAGGATGTTATGATTCGCGATGGCGAGTAATGTTTTTTTAAGCGGATCACTAGCTTGCTAGTGGTTCGGAATACTT
CGGGAGATTGTTATGCATCGTAATAAGTCAGTAAGCTCACATAGTTTTGCTATGGTTCCTAAAGCGGAAATTCCGCGTTCTAGTTTTGATACGCA
ATATGCGCATAAAACTACTTTTGATGGTGGTTATCTAGTTCCTATTTATTGTGATGAAGTGCTCCCAGGGGACATGCACAATGTAAAGGCTACAA
TGTTTGCCCGTTTGGCAACGCCTTTGTTTCCAGTTATGGACAATTTGCATTTAGATACTTTCTTTTTCTTTGTTCCAAATAGATTAGTTTGGAACAAT
TGGGTTAAGTTTATGGGTGAGCAAGCGAACCCAGGTGATTCTATTTCGTATGTAGTTCCACAGATTACTTCTACTGCTGGTGGTTATGCAGTAGG
TTCTATTTTTGATCATTTTGGTTTGCCTACGGCTGGTCAGATTACTGGTACTAATACGGTTACGCATAACGCGTTACCGTTACGTGCTTATAATTTG
ATTTATAACGAGTGGTTTAGGGATGAGAATTTACAAAATTCTGTTACCGTTCACAAGGGTGACGGTCCTGATACTCCGAGTGATTATGCTATGGT
TCGACGCGGTAAGCGTAAGGATTATTTTACTGGTGCCTTGCCTTGGCCTCAGAAAGGCGATGCAGTTACGTTGCCTTTAGGCACGTCTGCTCCTA
TTAAGACGTCTGTTACTAATGATTCAACAACGTTGTCATTAGTTGACGGTGCTGGTGCTTTGAAGCAGATTTATGCTAATTCAACTACACATGCTG
TGTATCGTAGTAATAACGCTGGTACTGCTGGTACTGGTTTGTATGCCGATTTAAGTGATGCTACGGCTGCAACTATTAACCAGTTACGTCAGTCTT
TCCAGATTCAGCGTTTGTTAGAGCGCGATGCGCGAGGTGGTACACGCTATACAGAATTGTTACGTGCTCATTTTGGTGTAACTCCACAAGATTAT
CGTTTACAACGTCCTGAATATATGGTGGGGTTCTACTTATGTCAACGTTAATCCTATTGCTCAAACGTCTGCTACTTCGATTTCTGGCGGTGCTACT

CCGCTTGGTAACTTGGCTGCAATGGGTACTGCGTTGGCTAGTGGACATGGTTTTACGTATCATGCTCAAGAACATGGATACATAATTGGTTTGGT
AAACGTAAGAGCTGATCTAACATATCAGCAAGGTTTACCTAAGATGTGGTCTCGTGAGACACGTTATGATTTCTATTTCCCTGTATTTGCTCATTT
AGGTGAACAGGCTGTTCTTAATAAGGAAATCTATGTTACTGGTACTTCAACTGATGATGATGTATTTGGATACCAGGAACGTTGGGCAGAGTAC
CGTTACAAACCTTCTCAGATTACAGGTTTGTTTAAGTCGACTTCAGCTGGTACGATTGACCCTTGGCATTATGCTCAGAAGTTCACTTCGTTGCCG
ACTTTGAATTCTACGTTTATTCAAGAAACGCCACCTATTGATCGTACTACTGCTGTTGGTGCATCTGCTAATGGTCAGCAGTTTTTGATGGATGCG
TTTTTTTTGTAAGATGGCTCGTCCAATGCCGATGTATTCTGTACCTGGCTTGATTGACCATTTCTAATGTAATATAAGCTGGACTACTGGGAAACC
AGTAGTCAGCAAACAAGCGGAGCGCGTTAGTATGGGTATGTTTGATTCAGTTGCTGGTGGTTTATTTGATTTTGCTACTTCTGCTTGGAGTCAGA
ATGAAGAGCAAAAATATAATACTGCTGAAGCGCAAGCAAATAGACGCTTTCAAGAAGATATGTCCAATACGCAATATCAGCGTATGGTTACTGA
TTTGAATAAGGCTGGTTTGAGTCCTATGCTTGCATATTCTAAAGGTGGTTCTACTGTTCCGAGTGGTGCTACTGCATCTTCTCTTCTCCAGTTAAGT
CTGATTTGAGTGGAGCTGTTAAGCGTGATGTTGAGAGTGATTTATTGCGTGAGCAGATGGAAGTTGCTAAATCTCAACGTGAATTAAATGTTCA
GACTGCTAGGAAGGTTGCTGAAGAAGCTGATTTAGCTTCTCAGCGTGTTTTACAAGAACCGGCTCGTTTTTATTTGGAACAGGCCGAGGCTGGTT
CTCGTGTTAATGCTAATTCTGCATCAACAATTAAGACTGATATTGATGCTAAGAATAATTTAGAGTTACGAACACCGTCTAGCGATCCATATTGGT
ATCGCGATATTAAGAAAGGTAGTAAGTCTATTTTTGATAAGTTTTTGGAAAAGAATAAGTCTTTTCCTTGGTCCTTGGAAGGTCGTAGAAAATGA
GTAAAACGAATTTACCTTTTGTACGTAATCCGTACAATTATGATATGGCTCTTGTTTCACAAGAGACTGGTCTTGAATGTAAAGATCCGAGTTTGG
CTCAACAACACATGAGAGACGAATGTGATATTAATATTATTGAGCGTTTTGGCGTTACTGGGCAATTGCCCCAGGCGCCATTAGAGCCATCATAT
GGCGATTTTAGCGGTGTATCTGACTATCACACCGCATTGAATGCTATTCGTGCCTCTGATGAGGCTTTTATGGCTTTGCCAGCTAAGATTAGGGCT
AAGTTTGATAACGATCCTAATGCTTTGTTAAATTATTTACAGAATGAAGAGAATCGTGATGAAGCGATTCAGATTGGTCTTATTGACGGTAAACC
TGTGGTTGAACCCGTCGTTTCTGCAGTAGAAACCTAAGCCAGACGCGTAAGCGGATGGCAGCACAGTTACTCTACTTGATGTAACTGTGCTAGG
TGACACCAAAACCACATTATTAACTACGGAGTGCAATGTTATGAGCCTTTATAGAAAACCAATGAGCAAGCATGGCGCAGCTAAGAAGTTTCGTC
GTGGCGTAAGCAAGACCAAGAGCATTAATATGCGTACTTCACCGCAGCGCGGTGGTTTTAGACTGTAATTTATGGCGTGTTATAAGCCGTTAAC
GGCTTATCAATGCGCTGACAAGTCTATTATTTGGCGGGAGATACCAGGGGCGGACGTAGTCCTACCTTGTCATTGCCTTGTGGTCAGTGTGTTGG
TTGTCGCCTTGAACGGTCCCGTCAGTGGGCCGTTAGATGTATGCATGAGGCACAAATGCATACTAGTAATTGTTTTATTACTTTGACATATGCTCC
AGAGCATTGTCCTAAGGATATGTCTTTGCATTATGAAGATTTTCAATTGTTTATGAAACGATTGAGAAAACGCTATACTGGCAAGACTATTCGTTT
TTATATGGCAGGTGAATATGGTGAGTCTTTCGATCGTCCTCATTTCCATGCTTGTATCTTTGGGCTTGATTTTGAAGATAAGAAGTTTTTCAAAAG
AACGCAGACTGGGTCTATCTTATATACGTCAAAGATACTTGAAGAACTTTGGCCGTATGGCTATAGTTCTATTGGTGATGTCAATTTTGAATCTGC
TGCTTATGTTGCTCGATATATTATGAAGAAGATTAACGGTAAAACCGTTAATGAGAACCACGAAGTGGTTGATGCAGATGCGCATTATCAGTATT
GTGATTTAGATACTGGTGAGATTATTCAGCGTAAGCCTGAATTTAATAAGATGTCTCTTAAGCCTGGCATTGGTCAGGCTTGGTTTGATAAGTTC
ATGTCAGACGTTTATACGACTGACTCTGTTGTGGTGCGTGGCAAAAAGTGCCGACCACCACGGTTTTATGATAATAAGTTTAAAGTATTGTTTCC
AGAAGAATTTGATGGTATACAATATGCTCGTGAGCTAGAAGGTCGCTCACATTTTGAAGATAACACTTT

>000113F|arrow

CCGCTACTGCGTCTTGATATGCAGTACGTCGCATACCATCTTGGAATTGCATCATTGCTGCAGCTTGTTGTGCGCTGGCAGCGTTTTGTTCTTGAC
CACCAAAATAACTAGCGGCCGCACCTATTCCGGCTCCAATTAAGGAGCCGTAAGGTCCAAAAGCGGCGCCAGATGCGGCGCCTGAGGCAGCGG
CTTCTAGTGCCATTAGAAGTGGTCGATTAAGCCAGGTACAGAGTACATTGGCATTGGCCGTGCTTTTTTAATATCAAAAAAGCTATCAAAATGAT
TTGCTGGCCGTTGGCAGCTGCTCCGACCGCAAGGGTTCGAGAGACAGGTGGATTTTCTTGAATAAACGGTTATTTCAAAGTTGGAAGGTGTTGT
AAAACCGTTGGGCTAAATGCCAGCCGTCAATTGTTCCAGCTGCCGTACTACGGAACAGACTGGAAATTTCGGCTTGGCATATAACGATATTCTGC
CCAACGTTCTTGGTATCCAAACACTCCTGTATCGTTGGCATCGCCTCGTACGTAGATTTCTTCATTTAATACTGCTTGTTCGCCCAAAGTGGCGAA
AGCAGGGAAATAAAAATCATAACGTGTGGAACGATTCCACATGCGGTGCATACCTTGTTGGTATGTTAAATCGGCTCGTACTGAAACGAGACCG
ATAATTACACCATGTTCAGTGCTTGAATAAGTAAAGCCATGATTGTGAGCGAGCCCAGTACCCATAGCGCCAAGTGTACCGAGAGGAGTAGTGG
TCCCGGAAGCTCCGGTACCATTCGTCTGGGCGATTGGATTGATATTAATGTGTGTTGAACCGCCTCCAATGTATTCAGGCCGTTGGAGACGCGCA
TCGGGGCTAACGACCCCGAAATGGCTGCGGATAATTTCTGTGTATCGAGTTCCGCCACGGGCGTCCCTTTCTAAAAGTTTTTGAATCTGGAAGCT
TTGACGCAATTGATTAATTGTTGCAGCGGTTGCAGCTGATAAATCTGCATATAAACCGCCATTAGGATCATATGATTTTGCCAAGCCGTCAGCAC
CGCCAGTAATTTGACCAGTAACACCTACGTTAATAGCCTGGGCGGTTGCGTTTAATGTATTTGTTCCAGCGTTATACAGTCTGGAAACAGGCGCA
TTGTTAGTGCGTAATATTGGGGCGGATGTTCCAAGCGGTAAAGTAACGCTTGCGCCCTTTTGTGGCCAAGGTAATGCTGACGTGAAATAGTCTTT
ACGTTTTCCACGTCGTAATAATGTGTAGTTGGCTACGTTATCTGGACCATCGCCAGTATCTACAACTACTGAATTTTGAAGGTTTTCGTCCCGAAA
CCATTCGTTATAAATAAGGTTGTAAGCACGTGGCCAGAAGGCACAGTGACTTACGGTGCCACCAGCACCTACTTGGCCTACAGTTGGCAGACCC
ATATAATCTTGAAGGCTGCCAATAGCGTAACCACCAGCTGGGGTTACTTGTTGGGGCACTACGTACGAAATCGAGTCCGCAGGATTTGCCTGCT
GCCCCATAAATTTTTGCCAATTTGACCAGACAAGTCTATTTGGCACAAAGAAGAAGAAGCTATCCAAATGCATATTGTCCATGATCGGATAGAGT
GGTGTAGACAATCGCGCAAAGGCGGTCATGTTGCAGCGGAATGTATCGCCTGGCAACATTTCGTCTACGAGTACAGGGACTAGGAAGCCAGCA
TCGAATGTAGTTTTATGTGTTGATTGACAGTCAAATGTAGAGCGGGGGATATCCGCTTTTGGAATCATTGTGAACTGATGGACGTCTACCGACTT
GTTACGATGCATTTTTTTGAGCTCCTAGGCCTAGTTGCGTGAGAAAAAGGGGTTTCCCCCTTTTACTCTACGCTTAGTTTTTATCAGTAATTTTGA
CTTGTTTCCCTAAGGATACAAGTTTGGGTTGTTCATGTAATTGGAACAACCCGTATTATCGTCGAATTCGCCTAATTCAAATAGGTCGAAATCGTC

GGAATGGTTATAAAGTTGATTGTCATCGCTCTGGCGATTGACTTCGTCGCTAAAGCTCCGGATTGCTTCGCCGATAGAACGGACGAACATTGGAC
GACCGAACGCATCTGCTGCGCGGTCTTTAACGGTACAGAGTACTAATTTCATGAGGATTTCCTAAGTGAGGTTACGTTTTAATAATTGAAGTTTG
GCCTTTGTGACTTTTTCCTTTACGGCAAGTCTGGCATAGGTATGTCTTCGTGGTTGAGTTTAGCAGAAGTTTCACGTTTGTGGAGTAATTCTTCGT
ATTCATATGGATAATCTGATTTATATTTTTTGTCATAGTATTTGGGGGTTTTACCCTTTTTCCGTTGACTACAACGTAGTCATGGGGATATACGTCG
GAACGATATTTTTTGTACCACTCAGCACCAATGCCTGGTTTAAGGCTCATATTGTTATATTCTGGTAATAGCTTTATTAATTCCCCTGTTTCTATATC
ACAGTATGTGTAATGCTCATCCTTTGTTATATGTTTGTTTTCTTCCATCTGGCCGTTGTATTTTTGCATAATATATCGAGCAACGTAAGCAGCTGAC
TCGAATGTAACGTCTCCAATGGAGGAATAACCATGTGGCCAGAGGGTTGCAAGGTGGTCGGATGTATATATGAGAGAACCAGAGGGAGTCCTT
TTGAATAGTTTCTTATCATGAAAATCGTATCCGAAGATACAGGCATGGAAGTGAGGTCTGCCGAAACTTGTGCCGTATTCTCCAGCCATGTAGTA
ACGTAATTTTGCAGGTGCAATGGATTTTCTGAAGCGCTTAAGGAACTTTTGGAAGTCGCTTTGACAAGCGATCCAGTTTGTGGAAGGTTTTCATT
GTTGTATGTGAGGGTTATAAAGCAGTTTTTTTTCGTGCAATTGGGCTTCATGCATGCAACGCATGGCCATTGGACGTGATTTTTCTAGCCTGCAGC
CAACGCATTGGCCGCAGGGCAGTGAAATCTGACGATCATGCTCGTCAGATTCTTGAATGCGACACGGCGATAAGATTTGCCGTCTTATAGTTTGT
CTGATGCTTACTTAAATAAGCAGTCAGTGGGTGATAACAGGCCATGTGAGGTAGCCTGGCGCTTTATTAGAGCCGCCAGCCCCCTCTTGCGGGC
TTTTTGCATATTTGCAGCTTTAGTTTTTGAAGTGTTCCTACGAAACGATTTAGCGCTTTTGCGCTTGTTTGTTTGTTTTCTATAAAGCATAATTTTGG
CCTCGTTTATCAGATATTTTGGTGTTGGTGTCACCTAGCACAGTTTACATCAAGTGGAGTAACTGTGCTTATCGCCATTTCTTCCGAAATGGCTCTT
TCGACCAGGCCGAGTTTCTCGGCTTCTGGTCGATTTTTCTCGTCTTGCAAGAACTCAATCAGGTTTGCTGGTTCGTTTTCGAACCTAGCACGAATT
TGAGCCGGTAAGTTATCAAATTCTTCCATAGCGTTCATTACCTTATTCAAGGCGCTATGATAGTCAGTAATTCCGCTGAAATCGCCATATTGAGGC
GGCAGCGGACTTTGAGGTAATAGGCCTGTAACGTTAAAACGTTCCAGGATAGTATTTATATCGCATTCGTCTTTATAATGCTGCTGAGCCAGAGT
TGGCTCCTCACAAGCCAACCCTGACTCATTTGACGCAGCATCCGTGTCATAGTTGTATGCGGTTCTAAGTTTGATTGTTTTCATTTCATTTTCCAAA
TGGTAGATATTTTTGATATTTATCATATTGCTATGTAAATAGCCTTTAACATCTTGATAAATTGGTTTAGTACTGGATGGCGCTGATCCAGTTTGCG
CTAATCGCTCTAATTGGTCTGTATATGCACTTTGACTTAACGCTTGTCGCTGTTGTGCAGCTTGTAATGCACTTGATGTCCTTAATTGCTCTATTTG
GGCATCGCGAAGCTGACCAAATTTGCCGTATCCCGGCATCTGAGCAATTTCACGAGCTGTATTTGCTCGAGTATATGTTGCCTGATCACGTGATA
GATTTGTATCCGCGTCCGTTTTTTCGGCTTGCGATTGTGTCAGGATATTCTGAGTTTGTAATTGTTTAAAATTGGCGACTGCCATGGCAGCTTCAC
GGGCTGAATTTCCAGCCTCACCTAGTGGATTTCCTACTGGAGCTTGCGCACCAGGCTGGACTTTGGCTCCGCCTTGTGAATAAGCCAGCATAGGG
TTAAGACCTGCAGCCTTAAGAT

>000175F|arrow

GCGATTTTAGTGGTGTTACTGATTACCAGTCTGCGTTGAATCAATTAACGCAACTATGGACGATTTCATGGCTCTGCCAGCGAAATTAGAGTCCG
ATTTGACCATGATCCGTGTCAAGATTATTGGAGTTCCTTGAGAACGACCAGAATCGTGATGAAGCGATTCAATTGGGTCTTATTGATGGACAACC
TGTGGTTGAACCCATCGTTTTCTACAGAAACACCTAAGGCCGAGGATGAAATCCTGAGGCCAGCACAGTTACTCTACTTGATGTAACTGTGCTAG
GTGCACCAACCACTATTTTAACTACGGAGTTCATCATGTTACGAAGAAGAGGCCAGTAAACAAATATAATCTGCAAGAGGTCAGTTCGGGCAGA
GACTGCTAGTAGACAGTCAATTATATAAGCAACGCTACCCCAGCGGGTGGCTATCGTTGTGTAATTATGGCCTGTTATAAGCCCTTAACGGCTTA
TCAATGCAGTGACAGTCTATAATTTGGCGGGAAAATACCGGGTGCGGATGTAGTCCGTACCCTATCATTGCCTTGTGGTCAGTGTGTTGGTTGGT
CGCCTTGAACGCTCACGTCAGTGGGCGATTCGTTGTATGCATGAGGCACAAATGCATACTAGTAATTGTTTTATTACTTGGACATATGCTCCAGA
GCATTGTCCTAAGGATATGTCATTGGATTACAATGAGTATCAGCTTTTTATGAAGCGGTTACGTAAGCGTTTACTGGGAAAACGATACGTTTTTAT
ATGGCAGGTTGATATGGTGAATCTTTTGATCGTCCTCATTTCCATGCTTGTCTGTTTGGTCTTGAGTTTCCGGATAAGAAAATATTTAAAAGAACG
CAGACTGGCTCTATCCTCTACACGTCAGAGATTTGGAAGAATTGTGGCCGTTTGGCTATTCTACAATTGGTGATGTTACTTTTGAGTCTGCTGCTT
ATGTTGCAAGATATATTATGAAGAAGATTAATGGGGTTTACTGTCAATGAAAACCACGAAGTGGTTGATGCGGGTGCCCATTATCAATATTGTG
ATTTAGAGACTGGTGAGATATTCAGCGTAAGCCAGAATTAATAAGATGTCATTGAAGCCCGGTATCGGGCAATCGTGGTTAGATAAGTACATGT
CAGACGTTTATACGTCAGACCACGTTGTGGTGCGTGGCAAAAAGTGCCGTCCACCACGGTTTATGATAATAAATTTAAGTTGAAGTTTCCTGAAG
AATTCGATATGATTCAGGTTGCCAGAGAGATGGAAGGTCGATCTAGGCATGAGGACAACACGCTTGAGCGACTTGCTGTTAAGGAAAAAAGTT
GCGTTGGCTAAGTTGTCATTGTTAAAACGTACTATTTAAGGAGTTTTTATGAAGAATGGTTATTGTTTCTATTAAGGATACTGCTGCAGATGCTTT
GGTCGTCCAGCTTATGTTGCATCTGAAGGGTGTTGCAGTACGTCAGTTTCAGGATGAAGTCAATCGAGCTAGCGAAGATAATCAGTTGTATAAA
CATCCTGATGATTTCATATGTTCTATTTGGGTCTTTTTGACGATGCCACTGGTGTTTTTGAACTACTGGAAAGCCCTAAGTTGATTGCTCGTGCAAA
AGATGTAATGATTCGCGAAGGCGAGTAAGGTTTTTTTTATACCGTATCACTCGAAAGAGTGGTACGGAACTACGGGAGATGTTTATGTTTCGCA
ATAAGTCAGTAAGTACGCATTCATTTGCTATGGTTCCTAAAGCGGACATTCCCCGCTCTAGTTTTAATACTCAATATGCTCATAAAACCACGTTTG
ATGCTGGGTTTTTTAGTTCCTATTTATTGTGATGAAGTATTGCCTGGCGATACTCATCGTGTAAAGATGACTGCATTTGCACGTTGGCCACACCGT
TATTTCCTGTGATGGACAACTTGCATCTTGATACTTTCTTTTTCTTTGTACCTAATCGTTTACTTTGGAACAATTGGCCAAAGTTTATGGGTGAACA
AACGAATCCTGGTGATTCTATTTCTTTTGTAGTGCCTACTATTACTAGTCCTGCTGGTGGTTATGCTGTTTGTTCAATTTTTGATTATTTTGGTTTAC
CTACTGCTGGTCAGATTACTGGCGCTAATACAGTAACGCATAATGTTTTGCCGTTACGTGCTTATAATGAGATTTATAACGAATGGTTTAGAGAT
GAAAACTTACAGAATTCTGTAACGTTAAATCTTGGTGATTCAGGTGATGTTCCTGCTAACTATACACTTTTGAGACGTGGTAAGCGTAAAGATTAT
TTTACTGTGCATTGCCTTGGCCACAGAAGGGTGCTTCTGTTTCTTTACCGTTAGGAACACGTGCTAATATTTATTCTGACATACCAGCTGGCAATG
GTACTGCTGGTTTATAGTGTTTTTCAAACTGCTGTTGGTGCTTTAAGAGAATTAAATTCAGCTTCTAATACTTTGTCTAATAGTACAAATGCTGGTG

TTGCTACTAATCAGTTATACGCTGATTTGTCTACTGCTACTGCTGCGACTATTAACCAACTTCGTCAATCTTTCCAGATTCAGAAGTTATTGGAGCG
CGATGCACGTGGTGGTACTCGTTATACTGAGTTACTACGTGCTCACTTTGGAGTAACTCCACAGGATTATCGTTTACAACGTCCTGAATATATTGG
TGGAGGTTCGACCCTTGTTAATGTTAATCCGATTGCTCAGACTTCTGCAACGTCGGTTACTGGTTCTGCTACTCCGCAAGGTAACCTTGCTGCAAT
GGGTACTGCATTGGCTCAGGGACACGGCTTTACGTATGCTGCTCAAGAACATGGATACATTATCGGATTAGTTTCTGTACGTGCTGACCTCACAT
ATCAACAGGGTCTTCCTAAGATGTGGTCTAGGTCTACACGATATGACTTTTATTTCCCAGTATTTGCCACTTTGGGTGAGCAAGCTATTTTGAACA
AAGAATTTATGTTCAAGGTACTGCAGCCGACAATGATGTATTTGGTTATCAAGAACGTTGGGCGGAGTATCGTTACAAACCTTCTCAAATTACTG
GTTTCTTTAGGTCTACTTCTGCTGGCACTATTGATGCTTGGCATTATGGACAGCGATTTACTTCTCTTCCTACGTTGAATTCAACGTTTATTCAAGA
GACCCCTCCAGTTGCTCGTACTACGGCGGTCGGAGCTGCAGCAAATGGTCAGCAATTTTTAATGGATGCTTTCTTTGATTGTCAGATGGCCAGAC
CTATGCCTATGTACAGCGTACCTGGTCTAATTGATCATTTCTAATGTTTTATATAACCTCGACTACTCCGTAAGGAGTAGTGAGGAAACAACCGAA
GGGCGTTAGTTTATGTTTGGTGGAATACTTGATGCGGTTACTAATGTTGGTTCTAAGCTGTCTTCAGCTTCTAGTTTCTTTACTCCTGGTGTCGGT
ACTGCTTTGGGCGCTGTTGGTTCTTATTTAGGTTCTACTTCTGCTAATAAGCTAATCAGGAGATGGCTCAGAGGCAAATGGATTTTCAAGCCGAT
ATGAGTGGAACAAGTTACCAGCGTGCTGTTAAAGATTTAGAAGCTGCTGGTTTATCTCCTATGTTAGCCTATCAACGTGGTGGTGCTTCTACCCC
ATCTGGTTCAACTGCTACTATGGAAAATGTTTTAGGTAATGCAACTAATTCAGCTATTAATACTGCTTCTATGATGCAACAGATTCGTAATGCATC
AGAAACAGAAAAGCAGATTATCGCCCAGACTGAAGCTACTGAAGCTGGTACCGCTAATACTAGGGCTGATACTGTTAATAAGTTGCTTACTGCT
CCTAATATTACAGCCGAAAATAAACGTATTTTGGCTGATATTGCTTTAAAGAATACGACTGCGGATTTAACATCCGCTCAGTCATATAATACTAAG
AGGCTATTGGCTCCATCCCCAGCTATTGGTCTAGGGGTATCGATGCTTCGAAAGAAATTTTTGATAAACTCAAAAATAATCCTAATCAACTAACCC
CTTGGGGAATTGGAGTCAAATAATGAGTAAAGCGAATTTGCCATTTGTACGTAATCCGTACAACTATGATAAAGATGAAGCATCGGTAAACGAT
GCGTTGCTGTGTCAAGACCCAAGTCTTGCTCAACAGCATATGAAAGATGAATGTGACATTAATGTCATCATTGAACGTTTCGGGGTTACAGGGG
AACTTCCAACGGCCCCTGTATCGCCTCAATACG

>000178F|arrow

CGAGCCCAGTACCCATAGCGCCAAGTGTACCGAGAGGAGTAGTGGTCCCGGAAGCTCCGGTAACATTCGTCTGGGCGATTGGATTGATATTAAT
GTGTGTTGAACCGCCTCCAATGTATTCAGGCCGTTGGAGACGCGCATCGGGGCTAACGACCCCGAAATGGCTGCGGATAATTTCTGTGTATCGA
GTTCCGCCACGGGCGTCCCTTTCTAAAAGTTTTTGAATCTGGAAGCTTTGACGCAATTGATTAATTGTTGCAGCGGTTGCAGCTGATAAATCTGCA
TATAAACCGCCATTAGGATCATATGATTTTGCCAAGCCGTCAGCACCGCCAGTAATTTGACCAGTAACACCTACGTTAATAGCCTGGGCGGTTGC
GTTTAATGTATTTGTTCCAGCGTTATACAGTCTGAAACAGGCGCATTGTTAGTGCGTAATATTGGGGCGGATGTTCCAAGCGGTAAAGTAACGCT
TGCGCCCTTTTGTGGCCAAGGTAATGCTGACGTGAAATAGTCTTTACGTTTTCCACGTCGTAATAATGTGTAGTTGGCTACGTTATCTGGACCATC
GCCAGTATCTACAACTACTGAATTTTGAAGGTTTCGTCCCGAAACCATTCGTTATAAATAAGGTTGTAAGCACGTGGCCAGAAGGCACAGTGACT
TACGGTGCCACCAGCACCTACTTGGCCTACAGTTGGCAGACCCATATAATCTTGAAGGCTGCCAATAGCGTAACCACCAGCTGGGGTTACTTGTT
GGGGCACTACGTACGAAATCGAGTCCGCAGGATTTGCCTGCTGCCCCATAAATTTTTGCCAATTTGACCAGACAAGTCTATTTGGCACAAAGAAG
AAGAAGCTATCCAAATGCATATTGTCCATGATCGGATAGAGTGGTGTAGACAATCGCGCAAAGGCGGTCATGTTGCAGCGGAATGTATCGCCTG
GCAACATTTCGTCTACGAGTACAGGGACTAGAAAGCCAGCATCGAATGTAGTTTTATGTGTTGATTGACAGTCAAATGTAGAGCGGGGGATATC
CGCTTTTGGAATCATTGTGAACTGATGGACGTCTACCGACTTGTTACGATGCATTTTTTTGAGCTCCTAGGCCTAGTTGCGTGAGAAAAAGGGGT
TTCCCCCTTTTTACTCTACGCTTAGTTTTTATCAGTAATTTTGACTTGTTTCCCTAAGGATACAAGTTTGGGTTGTTCATGTAATTGGAACAAACCCG
TATTATCGTCGAATTCGCCTAATTCAAATAGGTCGAAATCGTCGGAATGGTTATAAAGTTGATTGTCATCGCTCTGGCGATTGACTTCGTCGCTAA
AGCTCCGGATTGCTTCGCCGATAGAACGGACGAACATTGGACGACCGAACGCATCTGCTGCGCGGTCTTTAACGGTACAGAGTACTAATTTCAT
GAGGATTTCCTAAGTGAGGTTACGTTTTAATAATTGAAGTTTGGCCTTTGTGACTTTTTCCTTTACGGCAAGTCTGGCATAGGTATTGTCTTCGTG
GTTGAGTTTAGCAGAAGTTTCACGTTGTGGAGTAATTCTTCGTATTCATATGGATAATCTGATTTATATTTTTTGTCATAGTATTTTGGGGGTTTTA
CCCTTTTTCCGTTGACTACAACGTAGTCATGGGGATATACGTCGGAACGATATTTTTTGTACCACTCAGCACCAATGCCTGGTTTAAGGCTCATAT
TGTTATATTTGGTAATAGCTTTATTAATTCCCCTGTTTCTATATCACAGTATGTGTAATGCTCATCCTTTGTTATATGTTTGTTTTCTTCCATCTGGCC
GTTGTATTTTTGCATAATATATCGAGCAACGTAAGCAGCTGACTCGAATGTAACGTCTCCAATGGAGGAATAACCATGTGGCCAGAGGGTTGCA
AGGTGGTCGGATGTATATATGAGAGAACCAGAGGGAGTCCTTTTTGAATAGGTTTCTTATCATGAAATCGTATCCGAAGATACAGGCATGGAAG
TGAGGTCTGCCGAAACTTGTGCCGTATTCTCCAGCCATGTAGTAACGTAATTTTGCAGGTGCAATGGATTTTCTGAAGCGCTTAAGGAACTTTTG
GAAGTCGCTTTTGACAAGCGATCCAGTTTGTGGAAGGTTTTCATTGTTGTATGTGAGGGTTATAAAGCAGTTTTTTCGTGCAATTGGGCTTCATG
CATGCAACGCATGGCCCATTGACGTGATTTTTCTAGCCTGCAGCCAACGCATTGGCCGCAGGGCAGTGAAATCTGACGATCATGCTCGTCAGATT
CTTTGAATGCGACACGGCGATAAGATTTGCCGGTCTTATAGTTTGTCTGATGCTTACTTAAATAAGCAGTCAGTGGGTGATAACAGGCCATGTGA
GGTAGCCTGGCGCTTTATTAGAGCCGCCAGCCCCCTCTTTGCGGGGCTTTTTGCATATTTGCAGCTTTAGTTTTTGAAGTGTTCCTACGAAACGAT
TTAGCGCTTTTGCGCTTGTTTGTTTGTTTTCTATAAAGCATAATTTTGGCCTCGTTTATCAGATATTTTGGTGTTGGTGTCACCTAGCACAGTTACAT
CAAGTGGAGTAACTGTGCTTATCGCCATTTCTTCCGAAATGGCTCTTTCGACCAGGCCGAGTTTCTCGGCTTCTGGTCGATTTTTCTCGTCTTGCAA
GAACTCAATCAGGTTTGCTGGTTCGTTTTCGAACCTAGCACGAATTTGAGCCGGTAAGTTATCAAATTCTTCCATAGCGTTCATTACCTTATTCAA
GGCGCTATGATAGTCAGTAATTCCGCTGAAATCGCCATATTGATGGCGGCAGCGGACTTTGAGGTAATAGGCCTGTAACGTTAAAACGTTCCAG
GATAGTATTTATATCGCATTCGTCTTTATAATGCTGCTGAGCCAGAGTTGGCTCCTCACAAGCCAACCCTGACTCATTTGACGCAGCATCCGTGTC

ATAGTTGTATGCGGTTCTAAGTTTGATTGTTTTCATTTCATTTTTCCAAATGGTAGATATTTTTGATATTTATCATATTGGCTATGTAAATAGCCTTT
AACATCTTGATAAATTGGTTTAGTACTGGATGGCGCTGATCCAGTTTGCGCTAATCGCTCTAATTGGTCTGTATATGCACTTTGACTTAACGCTTG
TCCGCTGTTGTGCAGCTTGTAATGCACTTGATGTCCTTAATTGCTCTATTTGGGCATCGCGAAGCTGACCAAATTTGCCGTATCCCGGCATCTGAG
CAATTTCACGAGCTGTATTTGCTCGAGTATATGTTGCCTGATCACGTGATAGATTTGTATCCGCGTCCGTTTTTTCGGCTTGCGATTGTGTCAGGA
TATTCTGAGTTTGTAATTGTTTAAAATTGGCGACTGCCATGGCAGCTTCACGGCTGAATTTCCAGCCTCACCTAGTGGATTTCCTACTGGAGCTTG
CGCACCAGGCTGGACTTTGGCTCCGCCTTGTGAATAAGCCAGCATAGGGTTAAGACCTGCAGCCTTAAGGATCCGCTACTGCGTCTTGATATGCA
GTACGTCGCATACCATCTTGGAATTGCATCATTGCTGCAGCTTGTTGTGCGCTGGCAGCGTTTTGTTCTTGACCACCAAAATAACTAGCGGCCGC
ACCTATTCCGGCTCCAATTAAGGAGCCGTAAGGTCCAAAAGCGGCGCCAGATGCGGCGCCTGAGGCAGCGGCTTCTAGTGCCATTAGAAGTGG
TCGATTAAGCCAGGTACAGAGTACATTGGCATTGGCCGTGCTTTTTAATATCAAAAAAGCTATCAAAAATGATTTGCTGGCCGTTGGCAGCTGC
TCCGACCGCAAGGGTTCGAGAGACAGGTGGATTTTCTTGAATAAACGTGTTATTCAAAGTTGGAAGTGTTGTAAACCGTTGGGCTAAATGCCAG
CCGTCAATTGTTCCAGCTGCCGTACTACGGAACAGACTGGAAATTCGGCTTGGCATATAACGATATTCTGCCCACGTTCTTGGTATCCAAACACTC
CTGTATCGTTGGCATCGCCTCGTACGTAGATTTCTTCATTTAATACTGCTTGTTCGCCCAAAGTGGCGAAAGCAGGGAAATAGAAATCATAACGT
GTGGAACGATTCCACATGCGGTGCATACCTTGTTGGTATGTTAAATCGGCTCGTACTGAAACGAGACCGATAATTACACCATGTTCAGTGCTTGA
ATAAGTAAAGCCATGATTGTGAG

>000146F|arrow

GGTAACTGTGCTTCCGAGGTTCCAACAGGGGTTGAAACCTCGGTTTGGGCTGGTTTTACCAGTCCCATTTTTTCAGCTTCGCTGCGATTTTCAGGG
TTTTCTAGAAAATCTATTAGATTCGCGGGATCATTAGCGAATCGTTCACGAATATTGGCTGGTAAAGCCATAAATTCGTTGTCTGAAGCCATAATC
TGGTTCAGAGCAGAGTGGTAGTCATAGACACCACTAAAGTCGCCATATTGAGGCGTTAAAGGAGTTTGAGGAATAAGTCCGGTCATACCGAACT
TTTCCATAATATTAGATATCACATTCTTCAGCAAATTGCTGCTGAGTCAGAGTTGCATCCTCACAATGCAGCCCTGACTCATTTGACGCAGCAATC
GTGTCGTAATTGTACGGAGTACGAAGAAATGGGGCTGTAATCTTTGTCATTTTCATTCCATTGGTGGTTGGTTATTTGTACGATTTTGTTGAATCG
GTTGACCTCGATAGTTATCGATGTATCGTTTTGCGCCAGATGCGCTATACGCATCTTTGGCGATGTTTTTTACGTCTTGATAAATTGGTTTGCTAG
AACTAGGAGCTGACCCTGATTTAGCAAGTTTTGTTAACTCGTTTGAATAACGAGATTGTGCCAAAGCTTGAGCAGCTTGAGCACTTGATGTTTTG
CCTATTTCATTTTTCATGAAAGTATCGGCAAGTATTTGTTTATATTGAGCTCTAATATTTGGATTTTCATCCAATTTATTTAACGTATCAGCACGTAC
ATTATCTGTTTGATTACTTGTGAGTTCTGTTTGAGCTTCAATTTGTTTAGATTGAGCAATAGCTTGATTTGCTTGCGCAACCGTTTGATAAGCTTGG
GTTCCGGACGTAGTTGCATTACCGAGAACATTTTGCATCTGGGCCATAGCTCCAGCTGGTGTTGTGGCTCCGCCTTGTGAATACGCAAGCATGGG
ATTTAACCCAGCTTTTTTCATATCTTCAACTGCTCGTTGATATGATGTTCCAGACATTTCAGCTTGAAATCTCTGTTGATTACTCGCTTGTTCTGCAC
TCGCTGCGTTTTGACTTTGTGTTCCAAAGTATTGTCCTGCTGCACCTATTGCTGCAGGTGCAAGTGCAGCTAAGGAGAGTCCCCCAGTAGCAGGG
GCAGCTCCTATAGCTATAGCAGGGCCGATTAAATCGGCAATTCCGTCAAATAGTCCCATTAGAAATGGTCTATTAAGCCAGGTACTGAATACATT
GGCATTGGTCTTGCCATTTTGACATCAAAAAATGAGTCAAATAAGAATTGTTGGCCATTTGCAGCTGCTCCAACGGCTACTACACGTGATACAGG
AGGCGTATCTTGAATAAACGTATTATTCAAAGTAGGCGCAGCGGTAAATTTTTGAGCCAAATGCCAACCATCGATCGTGCCCGCCGATGTTGATT
TGAACAAACCAGTAATTTTAGATGGTTTGTAACGATATTCCGCCCAGCGTTCTTGATATCCAAAAACAGTATTGTCGGCTGCAGTATCTTGTTGCA
TAAATTTCTTTTTGCAAAACAGATTGTTCGCCTAGAGTAGCAAATGCTGGGAAATAAAAGTCATATCGTGTAGATCTAGACCACATACGGTCTAA
TCCTTGTTGATAAGTAAGATCTGCTCTAATGGATACTAATCCAATAATAACGCCATGCTCAGTAAATGATTGAGTAAATCCATGATTATGAGCGA
GAGCAGTACCCATAGCAGCAAGGTTGCCCAAAGGGGTAGTCGTTCCAGAAGCGTTTGTTCCCGACGTTTGAGCAATCGGATTAACATTAATTGG
TGTTGAACCGCCTCCAAGGTATTCAGGCCTTTGTAAACGGGCGTCTGGGGAAATAACTCCAAAGTGACTCCGGATAATTTCTGTGTATCGTGTTC
CGCCACGTGCATCCCTTTCAAGTAATTTTGAATTTGAAATGACTGTCTTAATTGATTGACAGTTGCAGCAGTTGCTTCTGATAAGTCGGTATATAA
ATTAGAAACGTTATTTACTACACCAGCAGTATTAACACCATAAGCGTTGCCATATCTAGCTAAAGCAGTCGTATTTCCAGGATCTGTTTGAATTAC
CGTAAATTTATCGTTTGATGTTGCGTCTCCTGAAATGGTATCCCATTTAATTGGAGCCGTAGTACCTAAAGGTAAGGTGACACTCGCACCTTTTTG
TGGCCATGGTAATGCTGACGTAAAGTAATCATGTCTTTTACCACGACGTTTTAGCACATAGTTTGAGGAAGTATCAGGGCCATCGCCCTTATCAA
CTACTGCGCTTGTTTGTAAATTTTCATCTCGGAACCATTCGTTCCAGATAAGATTGTATGCACGTGGCCAAAAGGCACAGTGCGTAATAGTTCGG
CCAGTATCAATTTGGCCTACTGTTGGTAAGCCCATATAGTCTTGAAGGCTGCCTACGGCATAACCATCTGTTGGGCTTGTTTGTGTTGGGACAAT
ATAAGATATTGAGTCTGTTGGATTTTCTTGTTGACCCATAAATTTTTGCCAGTTATTCCATATAAGGCGATTGGGTACAAAGAAAAAGAAAGAAT
CCATAATCATGTTATCCATGATTGGATATAAAGGCGTTGCTAGACGGGCAAATGCCGTCATTTTTAAGTTGAAAGTGTCCCCAGGGAGCACTTCA
TCAACATATACAGGAATTAGATAGCCCGCATCGAAAGTCGTTTTATGTTTTTTGAGCATCGAATTTACTACGTGGTATATCGGCTCTAGGTACCAT
CGCGAAGCGGTGAGTATTAACTGACTGATTGCGGTGCATGTTTTTCCTTAGTGTTGTTCCGGGGGAAAGATAAATCTCTTTTCCCCTCGGTTGTTT
TATTTAAGTTTAACTTGTTTTCCTAATGATAGGAGTTTTGGTTGTTCATGTAAATCGAATAACCCAGTTGAATCGTCAAATGTTCCGAATTCATATA
GATCGAATCATCAGGGTGATTAAAGAGTTGATTTTCAGTATCAGAACGATTAATTTCATCTGAAAAAGAGCGTATAGCTACTCCAGAGGAAGGT
ACGAACATTGGTCGTGCATATGCTTCAGCAGCACGGTCTTTTACGGAAGCGAGGATAAGTTTCATTATTTTCCTAAGTAAGGTTACGTTTTAATAG
TTGAAGTTTTGCCATAGTGACTTGTTCTTTTGCAGATAGTCGTTCTGGTGTATTGTCTTCGGAATTAATTTAGCATTATTTTCCCGCATGTAAAGTA
ATTCGTCATACTCATAAGGTTGGTCAATTTTAAACATTTTGTCATAGTATTTGGTGGTTTGACCTTTTTACCTCTAAGTATTACGTAGTCTTGCGGG
TATATATCCGAAGTATTTTATATAAAAGTCTTTACCGATTCCCGGTTTTAAAGACATTTTATTATATTCCGGCTTTAAGTCTAAATATTCGCCGGTTT

CAGGGTGTATGCGTTTGTAATGAGATTCCGCATCTTTCCCTGTTTGTTTTTTCATTATGTATCTAGCCACGTAGGCGGCTGATTCAAAAGTAACAT
CTCCAATGGTGGTATAACCAAATGGCCAGAGAGCTTCAAGTTCTGCGGATCTATATAACATAGAACCAGAGGCAGTCCTTTTCCATAATTTTTTAT
CAGGAAAGTCGTATCCGAAGATACAGGCATGGAAGTGGGGTCTTGCGAAGAGTTCACCATATTCTCCAGCCATGTAATAGCGGATTGTAAGTCC
TCTTTTTGCGAGAGTTTTTCTAAGTCTTTTAAGGAACAATTGAAAGTCTTTGTGATCCAAAGAGCCATCGCTTGGGAGATGTGTATTGTCATATGT
GAGTGTTATGAATGAGTTGTTTTTATGCAATTGGGCTTCGTGCATGCACCGAATAGCCCATTGTCTTGATCGTTCTAGACGGCATCCAACGCACT
GCCCACAGGGCAGGTCTAAAGATCGAACGATGTCATGTTTCCGAACTTCGTCGAAAACAATTGATTTGTCAAAGCATTGATATGCTTTGAGAGG
GTGATAACAAGGCATGTGAGGTGCCTGAAGACTTTATTAGAGTCTCCAGCCTCCACGCATTGGCGAGTTTCGCATATTTGCGTGTTTTGTATGTG
AAGCATGTTTACGGAAAGTCCTAGCGGACTTTTGCTTATTTGCTGGTCTGCGACGCATCATTTTTTCTGTCCTTGTTTATCGTGTTTTTGTGGTTTG
GTGTCACCTAGCACAGTTACATCAAGTA

>000058F|arrow

CACGTGCATCGCGCTCCAATAACTTCTGAATCTGGAAAGATTGACGAAGTTGGTTAATAGTCGCAGCAGTAGCAGTAGACAAATCAGCGTATAA
CTGATTAGTAGCAACACCAGCATTTGTACTATTAGACAAAGTATTAGAAGCTGAATTTAATGCTCTTAAAGCACCAACAGCAGTTTGAAAAAACA
CTATAACCAGCAGTACCAATTGCCAGCTGGTATGTCAGAATAAATATTAGCACGTGTTCCTAACGGTAAAGAAACAGAAGCACCCTTCTGTGGCC
AAGGCAATGCACCAGTAAAATAATCTTTACGCTTACCACGTCTCAAAAGTGTATAGTTAGCAGGAACATCACCTGAATCACCAAGATTTAACGTT
ACAGAATTCTGTAAGTTTCATCTCTAAACCATTCGTTATAAATCTCATTATAAGCACGTAACGGCAAAAACATTATGCGTTACTGTATTAGCGCCA
GTAATCTGACCAGCAGTAGGTAAACAAAATAATCAAAATTGAACAAACAGCATAACCACCAGCAGACTAGTAATAGTAGGCACTACAAAAGAAA
TAGAATCACCAGGATTCGTTTGTTCACCCATAAACTTTGGCCAATTGTTCCAAAGTAAACGATTAGGTACAAAGAAAAGAAAGTATCAAGATGCA
AGTTGTCCATCACAGGAAATAACGGTGTGGCCAAACGTGCAAATGCAGTCATCTTTACACGATGAGTATCGCCAGGCAATACTTCATCACAATAA
ATAGGAACTAAAAACCAGCATCAAACGTGGTTTTATGAGCATATTGAGTATTAAAACTAGAGCGGGGAATGTCCGCTTTAGGAACCATAGCAAA
TGAATGCGTACTTACTGACTTATTGCGAAACATAAACATCTCCCGTAGTTCCGTACCACTCTTTCGAGTGATACGGTATAAAAAAACCTTACCGCC
TTCGCGAATCATTACATCTTTTGCACGAGCAATCAACTTAGGGCTTTCCAGTAGTTCAAAAACACCAGTGGCATCGTCAAAAAGACCCAATAGAA
CATATGAAAATCATCAGGATGTTTATACACTGATTATCTTCGCTAGCTCGATTGACTTCATCCTGAAACTGACGTACTGCAACACCTTCAGATGCA
ACATAAGCTGGACGACCAAAAGCATCTGCAGCAGTATCCTTAATAGAAACAATAACCATCTTCATAAAAACTCCTTAATAGTAACGTTTTAACAAT
GACAACTTAGCCACGCAACTTTTTCCTTAACAGCAAGTCGCTCAAGCGTGTTGTCCTCATGCCTAGATCGACCTTCCATCTCTCTGCAAACTGAATC
ATATCGAATTCTTCAGGAAACTTCAACTTAAATTTATTATCATAAAACCGTGGTGGACGGCACTTTTTGCCACGCACCACAACGTGGTCTGACGTA
TAAACGTCTGACATGTACTTATCTAACCACGATTGCCCGATACCGGGCTTCAATGACATCTTATTAAATTCTGGCTTACGCTGAATTATCTCACCAG
TCTCTAAATCACAATATTGATAATGGGCACCCGCATCAACCACTTCGTGGTTTTCATTGACAGTAACCCCATTAATCTTCTTCATAATATATCTTGC
AACATAAGCAGCAGACTCAAAAGTAACATCACCAATTGTAGAATAGCCAAACGGCCACAATTCTTCCAAAATCTCTGACGTGTAGAGGATAGAG
CCAGTCTGCGTTCTTTTAAATATTTTCTTATCCGGAAATCAAGACCAAACAGACAAGCATGGAAATGAGACGATCAAAAGATTCACCATATTCACC
TGCCATATAAAAACGTATCGTTTTCCCAGTAAAACGCTTACGTAACCGCTTCATAAAAAGCTGATAATCATTGTAATCCAATGACATATCCTTAGG
ACAATGCTCTGGAGCATATGTCAAAGTAATAAACAATTACTAGTATGCATTTGTGCCTCATGCATACAACGAATCGCCCACTGACGTGAGCGTTC
AAGGCGACAACCAACACACTGACCACAAGGCAATGATAGGGTACGGACTACATCCGCACCCGGTATTTCCCGCCAAATTATAGACCTGTCACTG
CATTGATAAGCCGTTAAGGGCTTATAACAGGCCATAATTACAAACGATAGCCACCACGCTGGGGAGCGTGTCTCATATTAATTGACTTCGTCTTA
CTAGCAGTTCTGCGAAATGACTTTGCAGATTTATATTTGTTTACTGGCTTTCTTCGTAACATGATGAACTCCGTAGTTAAAATAGTGGTTTGGTGT
CACCTAGCACAGTTACATCAAGTAGAGTAACTGTGCTGGCCTCAGGATTTCATCCTTCGGCCTTAGGTGTTTCTGTAGAAACGATGGGTTCAACC
ACAGGTTGTCCATCAATAAGACCCAATTGAATCGCTTCATCACGATTCTGGTCGTTCTCAAGGAACTCCAATAATTTGACAGGATCATGGTCAAAT
CGGACTCTTAATTTCGCTGGCAGAGCCATGAAATCGTCCATAGTTGCGTTAATTTGATTCAACGCAGAATGGTAATCAGTAACACCACTAAAATC
GCCGTATTGAGGCGATACAGGGGCCGTTGGAAGTTCCCCTGTAACCCCGAAACGTTCAATGATGACATTAATGTCACATTCATCTTTCATATGCT
GTTGAGCAAGACTTGGGTCTTGACACAGCAACGCATCGTTTACCGATGCTTCATCTTTATCATAGTTGTACGGATTACGTACAAATGGCAAATTC
GCTTTACTCATTATTTGACTCCAATTCCCCAAGGGGTTAGTTGATTAGGATTATTTTTGAGTTTATCAAAAATTTCTTTCGAAGCATCGATACCCCT
AGACCAAATAGCTGGGGATGGAGCCAATAGCCTCTTAGTATTATATGACTGAGCGGATGTTAAATCCGCAGTCGTATTCTTTAAAGCAATATCAG
CCAAAATACGTTTATTTTCGGCTGTAATATTAGGAGCAGTAAGCAACTTATTAACAGTATCAGCCCTAGTATTAGCGGTACCAGCTTCAGTAGCTT
CAGTCTGGGCGATAATCTGCTTTTCTGTTTCTGATGCATTACGAATCTGTTGCATCATAGAAGCAGTATTAATAGCTGAATGTTGCATTACCTAAA
ACATTTTCCATAGTAGCAGTTGAACCAGATGGGGTAGAAGCACCACCACGTTGATAGGCTAACATAGGAGATAAACCAGCAGCTTCTAAATCTTT
AACAGCACGCTGGTAACTTGTTCCACTCATATCGGCTTGAAAATCCATTTGCCTCTGAGCCATCTCCTGATTAGCTTTATTAGCAGAAGTAGAACC
TAAATAAGAACCAACAGCGCCCAAAGCAGTACCGACACCAGGAGTAAAGAAACTAGAAGCTGAAGACAGCTTAGAACCAACATTAGTAACCGC
ATCAAGTATTCCACCAAACATAAACTAACGCCCTTCGGTTGTTTCCTCACTACTCCTTACGGAGTAGTCGAGGTTATATAAAACATTAGAAATGAT
CAATTAGACCAGGTACGCTGTACATAGGCATAGGTCTGGCCATCTGACAATCAAAGAAAGCATCCATTAAAAATTGCTGACCATTTGCTGCAGCT
CCGACCGCCGTAGTACGAGCAACTGGAGGGTCTCTTGAATAAACGTTGAATTCACAGTAGGAAGAGAAGTAAATCGCTGTCCATAATGCCAAGC
ATCAATAGTGCCAGCGAAAGTAGACTACAGACAACCAGTAATTTGAGAAGGTTGTAAACGAATGACTCCGCCCAACGTTCTTGATAACCAATAC
ATCATTGTCGGCTGCAGTACCTTGAACATAAATTTCTTTGTTCAAAATAGCTTGCTCACCCAAAGTGGCAAAGTACTGGAAATAAAAGTCATATCG

TGTAGACCTAGACCACATCTTAGGGAAGACCCTGTTGATATGTGAGGTCAGCACGTACAGAAAACGTAATCCGATAATGTATCCATGTTCTGAGC
AGCTGACGTAAAGCCGTGTCCCTGAGACAATGCAGTACCCATTGCAGCAAGGTTACCTTGCGGAGTAGCAGAACCAGTAACCGACTTGCAGAAG
TCTGAGCAATCGGATTAACATTAACAAGGGTCGAACCTCCACCAATATATTCAGGACGTTGTAAACGATAATCCTGTGGAGTTACTCCAAAGTGA
GCACGTAGTAACTCAGTATAACGAGTACCAC

>000029F|arrow

GCTTCATCACGATTCTCTTCATTCTGTAAATAATTTAACAAAGCATTAGGATCGTTATCAAACTTAGCCCTAATCTTAGCTGGCAAAGCCATAAAA
GCCTCATCAGAGGCACGAATAGCATTCAATGCGGTGTGATAGTCAGATACACCGCTAAAATCGCCATATGATGGCTCTAATGGCGCCTGGGGCA
ATTGCCCAGTAACGCCAAAAACGCTCAACTATAATATTAATATCACATTCGTCTCTCATGTGTTGTTGAGCCAAACTCGGATCTTTACATTCAAGA
CCAGTCTCTTGTGAAACAAGAGCCATATCATAATTGTACGGATTACGTACAAAAGGTAAATTCGTTTTACTCATTTTCTACGACCTTCCAAGGACC
AAGGAAAAGACTTATTCTTTTCCAAAAACTTATCAAAATAGACTTACTACCTTTCTTAATATCGCGATACCAATATGGATCGCTAGACGGTGTTCG
TAACTCTAAATTATTCTTAGCATCAATATCAGTCTTAATTGTTGATGCAGAATTAGCATTAACACGAGAACCAGCCTCGGCCTGTTCCAAATAAAA
ACGAGCCGGTTCTTGTAAAACACGCTGAGAAGCTAAATCAGCTTCTTCAGCAACCTTCCTAGCAGTCTGAACATTTAATTCACGTTGAGATTTAGC
AACTTCCATCTGCTCACGCAATAAATCACTCTCAACATCACGCTTAACAGCTCCACTCAAATCAGACTTAACTGGAGAAGTAGAAGATGCAGTAG
CACCACTCGGAACAGTAGAACCACCTTTAGAATATGCAAGCATAGGACTCAAACCAGCCTTATTCAAATCAGTAACCATACGCTGATATTGCGTA
TTGGACATATCTTCTTGAAAGCGTCTATTTGCTTGCGCTTCAGCAGTATTATATTTTTGCTCTTCATTCTGACTCCAAGCAGAAGTAGCAAAATCAA
ATAAACCACCAGCAACTGAATCAAACATACCCATACTAACGCGCTCCGCTTGTTGCTGACTACTGGTTTCCCAGTAGTCCAGCTTATATTACATTA
GAAATGGTCAATCAAGCCAGGTACAGAATACATCGGCATTGGACGAGCCATCTTACAATCAAAAAACGCATCCATCAAAAACTGCTGACCATTA
GCAGATGCACCAACAGCAGTAGTACGATCAATAGGTGGCGTTTCTTGAATAAACGTAGAATTCAAAGTCGGCAACGAAGTGAACTTCTGAGCAT
AATGCCAAGGGTCAATCGTACCAGCTGAAGTCGACTTAAACAAACCTGTAATCTGAGAAGGTTTGTAACGGTACTCTGCCCAACGTTCCTGGTAT
CCAAATACATCATCATCAGTTGAAGTACCAGTAACATAGATTTCCTTATTAAGAACAGCCTGTTCACCTAAATGAGCAAATACAGGGAAATAGAA
ATCATAACGTGTCTCACGAGACCACATCTTAGGTAAACCTTGCTGATATGTTAGATCAGCTCTTACGTTTACCAAACCAATTATGTATCCATGTTCT
TGAGCATGATACGTAAAACCATGTCCACTAGCCAACGCAGTACCCATTGCAGCCAAGTTACCAAGCGGAGTAGCACCGCCAGAAATCGAAGTAG
CAGACGTTTGAGCAATAGGATTAACGTTGACATAAGTAGAACCACCACCAATATATTCAGGACGTTGTAAACGATAATCTTGTGGAGTTACACCA
AAATGAGCACGTAACAATTCTGTATAGCGTGTACCACCTCGCGCATCGCGCTCTAACAAACGCTGAATCTGGAAAGACTGACGTAACTGGTTAAT
AGTTGCAGCCGTAGCATCACTTAAATCGGCATACAAACCAGTACCAGCAGTACCAGCGTTATTACTACGATACACAGCATGTGTAGTTGAATTAG
CATAAATCTGCTTCAAAGCACCAGCACCGTCAACTAATGACAACGTTGTTGAATCATTAGTAACAGACGTCTTAATAGGAGCAGACGTGCCTAAA
GGCAACGTAACTGCATCGCCTTTCTGAGGCCAAGGCAAGGCACCAGTAAATAATCCTTACGCTTACCGCGTCGAACCATAGCATAATCACTCGGA
GTATCAGGACCGTCACCCTTGTGAACGGTAACAGAATTTTGTAAATTCTCATCCCTAAACCACTCGTTATAAATCAAATTATAAGCACGTAACGGT
AACGCGTTATGCGTAACCGTATTAGTACCAGTAATCTGACCAGCCGTAGGCAAACCAAAATGATCAAAAATAGAACCTACTGCATAACCACCAGC
AGTAGAAGTAATCTGTGGAACTACATACGAAATAGAATCACCTGGGTTCGCTTGCTCACCCATAAACTTAACCCAATTGTTCCAAACTAATCTATT
TGGAACAAAGAAAAAGAAAGTATCTAAATGCAAATTGTCCATAACTGGAAACAAAGGCGTTGCCAAACGGGCAAACATTGTAGCCTTTACATTG
TGCATGTCCCCTGGGAGCACTTCATCACAATAAATAGGAACTAGATAACCACCATCAAAAGTAGTTTTATGCGCATATTGCGTATCAAAACTAGA
ACGCGGAATTTCCGCTTTAGGAACCATAGCAAAACTATGTGAGCTTACTGACTTATTACGATGCATAACAATCTCCCGAAGTATTCCGAACCACTA
GCAAGCTAGTGATCCGGCTTAAAAAAAACATTACTCGCCATCGCGAATCATAACATCCTTAGCTCTAGAAATAAGCTTGGGAGAACCAAGCAAAT
CCATAGTACCAGAATTATCATCAAACGTACCAAAATAATATAACTGAAAATCATCAGGGTGTTTATATAACTGATTATCATCGCTAGCGCGATTAA
CTTCGTCCTGAAACTGACGAACAGCAACACCCTCAGATGCAACAAAAGCTGGACGACCATACGCACCAGCTGCAGTATCTAAAATAGAAACAAT
AACCATCTTCATAAAAACTCCTTTAAATCTTACGTTTTAAAAGCGATAACTTAGCCAAAGCGACTTTTTCCTTTACAGCCAAACGCTCTAAAGTGTT
ATCTTCAAAATGTGAGCGACCTTCTAGCTCACGAGCATATTGTATACCATCAAATTCTTCTGGAAACAATACTTTAAACTTATTATCATAAAACCGT
GGTGGTCGGCACTTTTTGCCACGCACCACAACAGAGTCAGTCGTATAAACGTCTGACATGAACTTATCAAACCAAGCCTGACCAATGCCAGGCTT
AAGAGACATCTTATTAAATTCAGGCTTACGCTGAATAATCTCACCAGTATCTAAATCACAATACTGATAATGCGCATCTGCATCAACCACTTCGTG
GTTCTCATTAACGGTTTTACCGTTAATCTTCTTCATAATATATCGAGCAACATAAGCAGCAGATTCAAAATTGACATCACCAATAGAACTATAGCC
ATACGGCCAAAGTTCTTCAAGTATCTTTGACGTATATAAGATAGACCCAGTCTGCGTTCTTTTGAAAAACTTCTTATCTTCAAAATCAAGCCCAAA
GATACAAGCATGGAAATGAGGACGATCGAAAGACTCACCATATTCACCTGCCATATAAAAACGAATAGTCTTGCCAGTATAGCGTTTTCTCAATC
GTTTCATAAACAATTGAAAATCTTCATAATGCAAAGACATATCCTTAGGACAATGCTCTGGAGCATATGTCAAAGTAATAAAACAATTACTAGTAT
GCATTTGTGCCTCATGCATACATCTAACGGCCCACTGACGGGACCGTTCAAGGCGACAACCAACACACTGACCACAAGGCAATGACAAGGTACG
GACTACGTCCGCCCCTGGTATCTCCCGCCAAATAATAGACTTGTCAGCGCATTGATAAGCCGTTAACGGCTTATAACACGCCATAAATTACAGTCT
AAAACCACCGCGCTGCGGTGAAGTACGCATATTAATGCTCTTGGTCTTGCTTACGCCACGACGAAACTTCTTAGCTGCGCCATGCTTGCTCATTG
GTTTTCTATAAAGGCTCATAACATTGCACTCCGTAGTTAATAATGTGGTTTTGGTGTCACCTAGCACAGTTACATCAAGTAGAGTAACTGTGCTGC
CATCCGCTTACGCGTCTGGCTTAGGTGTTTCTACTGCAGAAACGACGGGTTCAACCACAGGTTTACCGTCAATAAGACCAATCTGAATC

>000141F|arrow

GGACAACACGCTTGAGCGACTTGCTGTTAAGGAAAAGTTGCGTTGGCTAAGTTGTCATTGTTAAAACGTACTATTTAAGGAGTTTTTATGAAGAT
GGTTATTGTTTCTATTAAGGATACTGCTGCAGATGCTTTTGGTCGTCCAGCTTATGTTGCATCTGAAGGTGTTGCAGTACGTCAGTTTCAGGATGA
AGTCAATCGAGCTAGCGAAGATAATCAGTTGTATAAACATCCTGATGATTTTCATATGTTCTATTTGGGTCTTTTTGACGATGCCACTGGTGTTTTT
GAACTACTGGAAAGCCCTAAGTTGATTGCTCGTGCAAAAGATGTAATGATTCGCGAAGGCGAGTAAGGTTTTTTTTATACCGTATCACTCGAAAG
AGTGGTACGGAACTACGGGAGATGTTTATGTTTCGCAATAAGTCAGTAAGTACGCATTCATTTGCTATGGTTCCTAAAGCGGACATTCCCCGCTC
TAGTTTTAATACTCAATATGCTCATAAAACCACGTTTGATGCTGGTTTTTTAGTTCCTATTTATTGTGATGAAGTATTGCCTGGCGATACTCATCGT
GTAAAGATGACTGCATTTGCACGTTTGGCCACACCGTTATTTCCTGTGATGGACAACTTGCATCTTGATACTTTCTTTTTCTTTGTACCTAATCGTTT
ACTTTGGAACAATTGGCCAAAGTTTATGGGTGAACAAACGAATCCTGGTGATTCTATTTCTTTTGTAGTGCCTACTATTACTAGTCCTGCTGGTGG
TTATGCTGTTTGTTCAATTTTTGATTATTTTGGTTTACCTACTGCTGGTCAGATTACTGGCGCTAATACAGTAACGCATAATGTTTTGCCGTTACGT
GCTTATAATGAGATTTATAACGAATGGTTTAGAGATGAAAACTTACAGAATTCTGTAACGTTAAATCTTGGTGATTCAGGTGATGTTCCTGCTAAC
TATACACTTTTGAGACGTGGTAAGCGTAAAGATTATTTTACTGGTGCATTGCCTTGGCCACAGAAGGGTGCTTCTGTTTCTTTACCGTTAGGAACA
CGTGCTAATATTTATTCTGACATACCAGCTGGCAATGGTACTGCTGGTTATAGTGTTTTCAAACTGCTGTTGGTGCTTTAAGAGAATTAAATTCA
GCTTCTAATACTTTGTCTAATAGTACAAATGCTGGTGTTGCTACTAATCAGTTATACGCTGATTTGTCTACTGCTACTGCTGCGACTATTAACCAAC
TTCGTCAATCTTTCCAGATTCAGAAGTTATTGGAGCGCGATGCACGTGGTGGTACTCGTTATACTGAGTTACTACGTGCTCACTTTGGAGTAACTC
CACAGGATTATCGTTTACAACGTCCTGAATATATTGGTGGAGGTTCGACCCTTGTTAATGTTAATCCGATTGCTCAGACTTCTGCAACGTCGGTTA
CTGGTTCTGCTACTCCGCAAGGTAACCTTGCTGCAATGGGTACTGCATTGGCTCAGGGACACGGCTTTACGTATGCTGCTCAAGAACATGGATAC
ATTATCGGATTAGTTTCTGTACGTGCTGACCTCACATATCAACAGGGTCTTCCTAAGATGTGGTCTAGGTCTACACGATATGACTTTTATTTCCCA
GTATTTGCCACTTTGGGTGAGCAAGCTATTTTGAACAAAGAAATTTATGTTCAAGGTACTGCAGCCGACAATGATGTATTTGGTTATCAAGAACG
TTGGGCGGAGTATCGTTACAAACCTTCTCAAATTACTGGTTTCTTTAGGTCTACTTCTGCTGGCACTATTGATGCTTGGCATTATGGACAGCGATT
TACTTCTCTTCCTACGTTGAATTCAACGTTTATTCAAGAGACCCCTCCAGTTGCTCGTACTACGGCGGTCGGAGCTGCAGCAAATGGTCAGCAATT
TTTAATGGATGCTTTCTTTGATTGTCAGATGGCCAGACCTATGCCTATGTACAGCGTACCTGGTCTAATTGATCATTTCTAATGTTTTATATAACCT
CGACTACTCCGTAAGGAGTAGTGAGGAAACAACCGAAGGGCGTTAGTTTATGTTTGGTGGAATACTTGATGCGGTTACTAATGTTGGTTCTAAG
CTGTCTTCAGCTTCTAGTTTCTTTACTCCTGGTGTCGGTACTGCTTTGGGCGCTGTTGGTTCTTATTTAGGTTCTACTTCTGCTAATAAAGCTAATCA
GGAGATGGCTCAGAGGCAAATGGATTTTCAAGCCGATATGAGTGGAACAAGTTACCAGCGTGCTGTTAAAGATTTAGAAGCTGCTGGTTTATCT
CCTATGTTAGCCTATCAACGTGGTGGTGCTTCTACCCCATCTGGTTCAACTGCTACTATGGAAAATGTTTTAGGTAATGCAACTAATTCAGCTATT
AATACTGCTTCTATGATGCAACAGATTCGTAATGCATCAGAAACAGAAAAGCAGATTATCGCCCAGACTGAAGCTACTGAAGCTGGTACCGCTA
ATACTAGGGCTGATACTGTTAATAAGTTGCTTACTGCTCCTAATATTACAGCCGAAAATAAACGTATTTTGGCTGATATTGCTTTAAAGAATACGA
CTGCGGATTTAACATCCGCTCAGTCATATAATACTAAGAGGCTATTGGCTCCATCCCCAGCTATTTGGTCTAGGGGTATCGATGCTTCGAAAGAA
ATTTTTGATAAACTCAAAAATAATCCTAATCAACTAACCCCTTGGGGAATTGGAGTCAAATAATGAGTAAAGCGAATTTGCCATTTGTACGTAATC
CGTACAACTATGATAAAGATGAAGCATCGGTAAACGATGCGTTGCTGTGTCAAGACCCAAGTCTTGCTCAACAGCATATGAAAGATGAATGTGA
CATTAATGTCATCATTGAACGTTTCGGGGTTACAGGGGAACTTCCAACGGCCCCTGTATCGCCTCAATACGGCGATTTTAGTGGTGTTACTGATT
ACCATTCTGCGTTGAATCAAATTAACGCAACTATGGACGATTTCATGGCTCTGCCAGCGAAATTAAGAGTCCGATTTGACCATGATCCTGTCAAAT
TATTGGAGTTCCTTGAGAACGACCAGAATCGTGATGAAGCGATTCAATTGGGTCTTATTGATGGACAACCTGTGGTTGAACCCATCGTTTCTACA
GAACACCTAAGGCCGAAGGATGAAATCCTGAGGCCAGCACAGTTACTCTACTTGATGTAACTGTGCTAGGTGACACCAAACCACTATTTTAACTA
CGGAGTTCATCATGTTACGAAGAAAGCCAGTAAACAAATATAAATCTGCAAAGTCATTTCGCAGAACTGCTAGTAAGACGAAGTCAATTAATAT
GAGACACGCTCCCCAGCGTGGTGGCTATCGTTTGTAATTATGGCCTGTTATAAGCCCTTAACGGCTTATCAATGCAGTGACAGGTCTATAATTTG
GCGGGAAATACCGGGTGCGGATGTAGTCCGTACCCTATCATTGCCTTGTGGTCAGTGTGTTGGTTGTCGCCTTGAACGCTCACGTCAGTGGGCG
ATTCGTTGTATGCATGAGGCACAAATGCATACTAGTAATTGTTTTATTACTTTGACATATGCTCCAGAGCATTGTCCTAAGGATATGTCATTGGAT
TACAATGATTATCAGCTTTTTATGAAGCGGTTACGTAAGCGTTTTACTGGGAAAACGATACGTTTTTATATGGCAGGTGAATATGGTGAATCTTTT
GATCGTCCTCATTTCCATGCTTGTCTGTTTGGTCTTGATTTTCCGGATAAGAAAATATTTAAAAGAACGCAGACTGGCTCTATCCTCTACACGTCAG
AGATTTTGGAAGAATTGTGGCCGTTTGGCTATTCTACAATTGGTGATGTTACTTTTGAGTCTGCTGCTTATGTTGCAAGATATATTATGAAGAAGA
TTAATGGGGTTACTGTCAATGAAAACCACGAAGTGGTTGATGCGGGTGCCCATTATCAATATTGTGATTTAGAGACTGGTGAGATAATTCAGCG
TAAGCCAGAATTTAATAAGATGTCATTGAAGCCCGGTATCGGGCAATCGTGGTTAGATAAGTACATGTCAGACGTTTATACGTCAGACCACGTTG
TGGTGCGTGGCAAAAAGTGCCGTCCACCACGGTTTTATGATAATAAATTTAAGTTGAAGTTTCCTGAAGAATTCGATATGATTCAGTTTGCCAGA
GAGATGGAAGGTCGATCTAGGCATGA

>000028F|arrow

CCTACTGCATAACCACCAGCAGTAGAAGTAATCTGTGGAACTACATACGAAATAGAATCACCTGGGTTCGCTTGCTCACCCATAAACTTAACCCA
ATTGTTCCAAACTAATCTATTTGGAACAAAGAAAAAGAAAGTATCTAAATGCAAATTGTCCATAACTGGAAACAAAGGCGTTGCCAAACGGGCA
AACATTGTAGCCTTTACATTGTGCATGTCCCCTGGGAGCACTTCATCACAATAAATAGGAACTAGATAACCACCATCAAAAGTAGTTTTATGCGCA
TATTGCGTATCAAAACTAGAACGCGGAATTTCCGCTTTAGGAACCATAGCAAAACTATGTGAGCTTACTGACTTATTACGATGCATAACAATCTCC
CGAAGTATTCCGAACCACTAGCAAGCTAGTGATCCGGCTTAAAAAAAACATTACTCGCCATCGCGAATCATAACATCCTTAGCTCTAGAAATAAG

CTTGGGAGAACCAAGCAAATCCATAGTACCAGAATTATCATCAAACGTACCAAAATAATATAACTGAAAATCATCAGGGTGTTTATATAACTGAT
TATCATCGCTAGCGCGATTAACTTCGTCCTGAAACTGACGAACAGCAACACCCTCAGATGCAACAAAAGCTGGACGACCATACGCACCAGCTGC
AGTATCTAAATAGAAACAATAACCATCTTCATAAAAACTCCTTTAATCTTACGTTTAAAAGCGATAACTTAGCCAAAGCGACTTTTTCCTTTACAGC
CAAACGCTCTAAAGTGTTATCTTCAAAATGTGAGCGACCTTCTAGCTCACGAGCATATTGTATACCATCAAATTCTTCTGGAAACAATACTTTAAA
CTTATTATCATAAAACCGTGGTGGTCGGCACTTTTTGCCACGCACCACAACAGAGTCAGTCGTATAAACGTCTGACATGAACTTATCAAACCAAG
CCTGACCAATGCCAGGCTTAAGAGACATCTTATTAAATTCAGGCTTACGCTGAATAATCTCACCAGTATCTAAATCACAATACTGATAATGCGCAT
CTGCATCAACCACTTCGTGGTTCTCATTAACGGTTTTACCGTTAATCTTTCTTCATAATATATCGAGCAACATAAGCAGCAGATTCAAAATTGACAT
CACCAATAGAACTATAGCCATACGGCCAAAGTTCTTCAAGTATCTTTGACGTATATAAGATAGACCCAGTCTGCGTTCTTTTGAAAAACTTCTTAT
CTTCAAAATCAAGCCCAAAGATACAAGCATGGAAATGAGGACGATCGAAAGACTCACCATATTCACCTGCCATATAAAAACGAATAGTCTTGCC
AGTATAGCGTTTTCTCAATCGTTTCATAAACAATTGAAAATCTTCATAATGCAAAGACATATCCTTAGGACAATGCTCTGGAGCATATGTCAAAGT
AATAAAACAATTACTAGTATGCATTTGTGCCTCATGCATACATCTAACGGCCCACTGACGGGACCGTTCAAGGCGACAACCAACACACTGACCAC
AAGGCAATGACAAGGTACGGACTACGTCCGCCCCTGGTATCTCCCGCCAAATAATAGACTTGTCAGCGCATTGATAAGCCGTTAACGGCTTATAA
CACGCCATAAATTACAGTCTAAAACCACCGCGCTGCGGTGAAGTACGCATATTAATGCTCTTGGTCTTGCTTACGCCACGACGAAACTTCTTAGCT
GCGCCATGCTTGCTCATTGGTTTTCTATAAAGGCTCATAACATTGCACTCCGTAGTTAATAATGTGGTTTTGGTGTCACCTAGCACAGTTACATCA
AGTAGAGTAACTGTGCTGCCATCCGCTTACGCGTCTGGCTTAGGTGTTTCTACTGCAGAAACGACGGGTTCAACCACAGGTTTACCGTCAATAAG
ACCAATCTGAATCGCTTCATCACGATTCTCTTCATTCTGTAAATAATTTAACAAAGCATTAGGATCGTTATCAAACTTAGCCCTAATCTTAGCTGGC
AAAGCCATAAAAGCCTCATCAGAGGCACGAATAGCATTCAATGCGGTGTGATAGTCAGATACACCGCTAAAATCGCCATATGATGGCTCTAATG
GCGCCTGGGGCAATTGCCCAGTAACGCCAAAACGCTCAACTATAATATTAATATCACATTCGTCTCTCATGTGTTGTTGAGCCAAACTCGGATCTT
TACATTCAAGACCAGTCTCTTGTGAAACAAGAGCCATATCATAATTGTACGGATTACGTACAAAAGGTAAATTCGTTTACTCATTTTCTACGACC
TTCCAAGGACCAAGGAAAAGACTTATTCTTTTCCAAAAACTTATCAAAAATAGACTTACTACCTTTCTTAATATCGCGATACCAATATGGATCGCT
AGACGGTGTTCGTAACTCTAAATTATTCTTAGCATCAATATCAGTCTTAATTGTTGATGCAGAATTAGCATTAACACGAGAACCAGCCTCGGCCTG
TTCCAAATAAAAACGAGCCGGTTCTTGTAAAACACGCTGAGAAGCTAAATCAGCTTCTTCAGCAACCTTCCTAGCAGTCTGAACATTTAATTCACG
TTGAGATTTAGCAACTTCCATCTGCTCACGCAATAAATCACTCTCAACATCACGCTTAACAGCTCCACTCAAATCAGACTTAACTGGAGAAGTAGA
AGATGCAGTAGCACCACTCGGAACAGTAGAACCACCTTTAGAATATGCAAGCATAGGACTCAAACCAGCCTTATTCAAATCAGTAACCATACGCT
GATATTGCGTATTGGACATATCTTCTTGAAAGCGTCTATTTGCTTGCGCTTCAGCAGTATTATATTTTTGCTCTTCATTCTGACTCCAAGCAGAAGT
AGCAAAATCAAATAAACCACCAGCAACTGAATCAAACATACCCATACTAACGCGCTCCGCTTGTTTGCTGACTACTGGTTTCCCAGTAGTCCAGCT
TATATTACATTAGAAATGGTCAATCAAGCCAGGTACAGAATACATCGGCATTGGACGAGCCATCTTACAATCAAAAAACGCATCCATCAAAAACT
GCTGACCATTAGCAGATGCACCAACAGCAGTAGTACGATCAATAGGTGGCGTTTCTTGAATAAACGTAGAATTCAAAGTCGGCAACGAAGTGAA
CTTCTGAGCATAATGCCAAGGGTCAATCGTACCAGCTGAAGTCGACTTAAACAACCTGTAATCTGAGAAGGTTTGTAACGGTACTCTGCCCAACG
TTCCTGGTATCCAAATACATCATCATCAGTTGAAGTACCAGTAACATAGATTTCCTTATTAAGAACAGCCTGTTCACCTAAATGAGCAAATACAGG
GAAATAGAAATCATAACGTGTCTCACGAGACCACATCTTAGGTAAACCTTGCTGATATGTTAGATCAGCTCTTACGTTTACCAAACCAATTATGTA
TCCATGTTCTTGAGCATGATACGTAAAACCATGTCCACTAGCCAACGCAGTACCCATTGCAGCCAAGTTACCAAGCGGAGTAGCACCGCCAGAAA
TCGAAGTAGCAGACGTTTGAGCAATAGGATTAACGTTGACATAAGTAGAACCACCACCAATATATTCAGGACGTTGTAAACGATAATCTTGTGG
AGTTACACCAAAATGAGCACGTAACAATTCTGTATAGCGTGTACCACCTCGCGCATCGCGCTCTAACAAACGCTGAATCTGGAAAGACTGACGTA
ACTGGTTAATAGTTGCAGCCGTAGCATCACTTAAATCGGCATACAAACCAGTACCAGCAGTACCAGCGTTATTACTACGATACACAGCATGTGTA
GTTGAATTAGCATAAATCTGCTTCAAAGCACCAGCACCGTCAACTAATGACAACGTTGTTGAATCATTAGTAACAGACGTCTTAATAGGAGCAGA
CGTGCCTAAAGGCAACGTAACTGCATCGCCTTTCTGAGGCCAAGGCAAGGCACCAGTAAAATAATCCTTACGCTTACCGCGTCGAACCATAGCAT
AATCACTCGGAGTATCAGGACCGTCACCCTTGTGAACGGTAACAGAATTTTGTAAATTCTCATCCCTAAACCACTCGTTATAAATCAAATTATAAG
CACGTAACGGTAACGCGTTATGCGTAACCGTATTAGTACCAGTAATCTGACCAGCCGTAGGCAAACCAAAATGATCAAAAATAGAA

>000036F|arrow

TGTAAACGATAATCCTGTGGAGTTACTCCAAAGTGAGCACGTAGTAACTCAGTATAACGAGTACCACCACGTGCATCGCGCTCCAATAACTTCTG
AATCTGGAAAGATGACGAAGTTGGTTAATAGTCGCAGCAGTAGCAGTAGACAAATCAGCGTATAACTGATTAGTAGCAACACCAGCATTTGTAC
TATTAGACAAAGTATTAGAAGCTGAATTTAATTCTCTTAAAGCACCAACAGCAGTTTGAAAAAACACTATAACCAGCAGTACCATTGCCAGCTGG
TATGTCAGAATAAATATTAGCACGTGTTCCTAACGGTAAAGAAACAGAAGCACCCTTCTGTGGCCAAGGCAATGCACCAGTAAAATAATCTTTAC
GCTTACCCACGTCTCAAAAGTGTATAGTTAGCAGGACATCACCTGAATCACCAAGATTTAACGTTACAGAATTCTGTAAGTTTTCATCTCTAAACCA
TTCGTTATAAATTCATTATAAGCACGTAAACGGCAAACATTATGCGTTACTGTATTAGCGCCAGTAATCTGACCAGCAGTAGGTAAACCAAAATA
ATCAAAAATTGAACAAACAGCATAACCACCAGCAGGACTAGTAATAGTAGGCACTACAAAAGAAATAGAATCACCAGGATTCGTTGTTCACCCA
TAAACTTTGGCCAATTGTTCCAAAGTAAACGATTAGGTACAAAGAAAAGAAAGTATCAAGATGCAAGTGTCCATCACAGGAAATAACGGTGTGG
CCAAACGTGCAAATGCAGTCATCTTTACACGATGAGTATCGCCAGGCAATACTTCATCACAATAAATAGGAACTAAAAAACCAGCATAAACGTG
GTTTTATGAGCATATTGAGTATTAAAACTAGAGCGGGGAATGTCCGCTTTAGGAACCATAGCAAATGAATGCGTACTTACTGATTATTGCGAAAC
ATAAACATCTCCCGTAGTTCCGTACCATCTTTCGAGTGATACGGTATAAAAAAAACCTTACTCGCCTTCGCGAATCATTACATCTTTTGCACAATCA

ACTTAGGGCTTTCCAGTAGTTCAAAAACACAGTGGCATCGTCAAAAGACCCAAATAGAACATATGAAAATCATCAGGATGTTTATACAACTGATT
ATCTTCGCTAGCTCGATGACTTCATCCTGAAAATGACGTACTGCAACACCTTCAGATGCAACATAAGCTGGACGACCAAAAGCATCTGCAGCAGT
ATCCTTAATAGAAACAATAACCATCTTCATAAAAACTCTTAAATAGTACGTTTTAACAATGACAACTTAGCCAACGCAACTTTTTCCTTAACAGCAG
TCGCTCAAGCGTGTTGTCCTCATGCCTAGATCGACCTTCCATCTCTCTGGCAAACTGAATCATATCGAATTCTTCAGGAAACTTCAACTTAAATTTT
TATCAAAAACCGTGGTGGACGGCACTTTTTGCCACGCACCACAAACGTGGTCTGACGTATAAACGTCTGACATGTACTTATCTAACCACGATTGC
GATACCGGGCTTCAATGACATCTTATTAATTCTGGCTTACGCTGAATTATCTCACCAGTCTCTAAATCACAATATTGATAATGGGCACCCGCATCA
ACCACTTCGTGGTTTTCATGACAGTAACCCCATTAATCTTCTCATATATATCTTGCAACATAAGCAGCAGACTCAAAAGTAACATCACCAATTGTA
GAATAGCCAAACGGCCACAATTCTTCCAAAATCTCTGACGTGTAGAGGATAGAGCCAGTCTGCGTTCTTTTAAAATTTTCTTATCCGGAAAATCAA
GACCAAACAGACAAGCATGGAAATGAGGACGATCAAAGATCACCATATTCACCTGCCATATAAAAACGTATCGTTTTCCCAGTAAAACGCTTACG
TAACCGCTTCATAAAAAGCTGATAATCATTGTAATCCAATGACTATCCTTAGGACAATGCTCTGGAGCATATGTCAAAGTAATAAAACAATTACTA
GTATGCATTTGTGCCTCATGCATAAACGAATCGCCCACTGACGTGAGCGTTCAAGGCGACAACCAACACACTGACCACAAGGCAATGATAGGGT
ACGGACTACATCCGCACCCGGTATTTCCCGCCAAATTATAGACCTGTCACTGCATTGATAAGCCGTTAAGGGCTTATAACAGGCATAATTACAAA
CGATAGCCACCACGCTGGGGAGCGTGTCTCTATAATTGACTTCGTCTTACTAGCAGTTCTGCGAAATGACTTTGCAGATTTATATTTGTTTACTGG
CTTTTTCGTAACATGATGAACTCCGTAGTTAAAATAGTGGTTTGGTGTCACCTAGCACAGTTACATCAAGTAGAGTAACTGTGCTGGCCTCAGGA
TTTCATCTTCGGCCTTAGGTGTTTCTGTAGAAACGATGGGTTCAACCACAGGTTGTCCATCAATAAGACCAATTGAATCGCTTCATCACGATTCTG
GTCGTTCTCAAGGAACTCCAATAATTTGACAGGATCATGGTCAAATCGGACTCTTAATTTCGCTGGCAGAGCCATGAAACGTCCTAGTTGCGTTA
ATTTGATTCAACGCAGAATGGTAATCAGTAACACACTAAAATCGCCGTATTGAGGCGATACAGGGGCGTTGGAAGTTCCCCTGTAACCCCGAAA
CGTTCAATGATGACATTAATGTCACATTCATCTTTCATATGCTGTTGAGCAAGACTTGGGTTTGACACAGCAACGCATCGTTTACGATGCTTCATC
TTATCATAGTTGTACGGATTACGTACAAATGGCAAATTCGCTTATCATTATTTGACTCCAATTCCCAAGGGGTTAGTTGATTAGGATTATTTTGAG
TTTATCAAAAATTTCTTTCGAAGCATCGATACCCTAGACCAAATAGCTGGGGATGGAGCCAATAGCCTCTTAGTATTATATGACTGAGCGGATGT
TAAATCCGCAGTCGTATTTTTAAAGCAATATCAGCCAAAATACGTTTATTTTCGGCTGTAATATTAGGAGCAGTAAGCAACTTATTACACAGTATC
AGCCCTAGTATTAGCGGTACCAGCTTCAGTAGCTTCAGTCTGGGCGATAATCATTTTCTGTTTCTGATGCATTACGAATCTGTGCATCATAGAAGC
AGTATTAATAGCTGAATTAGTTGCATTACCTAAAACATTTTCCATAGTAGCAGTGAACCAGATGGGGTAGAAGCACCACCACGTTGATAGGCTAA
CATAGGAGATAAACCAGCAGCTTCTAAATCTTTAACAGCACGCTGGTAACTGTTCCACTCATATCGGCTTGAAAATCCATTTGCCTCTGAGCCATA
TCCTGATTAGCTTTATTAGCAGAAGTAACCTAAATAAGAACCAACAGCGCCCAAGCAGTACCGACACCAGGAGTAAAGAAACTAGAAGCTGAAG
ACAGCTTAGAACAACATTAGTAACCGCATCAAGTATTCCACCAAACATAAACTAACGCCCTTCGGTTGTTTCCTCACTATCCTACGGAGTAGTCGA
GGTTATATAAAACATTAGAAATGATCAATTAGACAGGTACGCTGTACATAGGCATAGGTCTGGCCATCTGACAATCAAAGAAAGCATCCATTAA
AAATTGCTGACCATTTGCTGCAGCTCGACCGCCGCAGTACGGCAACTGGAGGGGTCTCTTGAATAAACGTTGAATTCAACGTAGGAAGAGAAGT
AAATCGCTGTCCATAATGCCAAGCATCAATAGTGCCAGCAGAAGTAGACCTAAAGAAAACCAGTAATTGAGAAGGTTTGTAACGATACTCCGCC
CAACGTTCTTGATACCAAATACATCATTGTCGGCTGCAGTACCTTGAACATAAATTTCTTTGTTCAAAATAGCTTGCTCACCCAAAGTGGCAAATA
CTGGGAAATAAAAGTCATATCGTGTAGACCTAGACCACATCTTAGGAAGACCTGTGATATGTGAGGTCAGCACGTACAGAAAACTAATCCGATA
ATGTATCCATGTTCTTGAGCAGCATACGTAAAGCCGTGTCCCTGAGCCAATGCAGTACCCATTGCAGCAAGGTTACCTTGCGGAGTAGCAGAACC
AGTAACCGACGTTGCAGAAGTCTGAGCAATCGGATTAACATTAACAAGGGTCGAACCTCCACCAATATATTCAGGACGT

>000065F|arrow

ATTTGGGTCTTTTTGACGATGCCACTGGTGTTTTTGAACTACTGGAAAGCCCTAAGTTGATTGCTCGTGCAAAAGATGTAATGATTCGCGAAGGC
GAGTAAGGTTTTTTTTATACCGTATCACTCGAAAGAGTGGTACGGAACTACGGGAGATGTTTATGTTTCGCAATAAGTCAGTAAGTACGCATTCA
TTTGCTATGGTTCCTAAAGCGGACATTCCCCGCTCTAGTTTTAATACTCAATATGCTCATAAAACCACGTTTGATGCTGGTTTTTTAGTTCCTATTTA
TTGTGATGAAGTATTGCCTGGCGATACTCATCGTGTAAAGATGACTGCATTTGCACGTTTGGCCACACCGTTATTTCCTGTGATGGACAACTTGCA
TCTTGATACTTTCTTTTTCTTTGTACCTAATCGTTTACTTTGGAACAATTGGCCAAAGTTTATGGGTGAACAAACGAATCCTGGTGATTCTATTTCTT
TTGTAGTGCCTACTATTACTAGTCCTGCTGGTGGTTATGCTGTTTGTTCAATTTTTGATTATTTTGGTTTACCTACTGCTGGTCAGATTACTGGCGC
TAATACAGTAACGCATAATGTTTTGCCGTTACGTGCTTATAATGAGATTTATAACGAATGGTTTAGAGATGAAAACTTACAGAATTCTGTAACGTT
AAATCTTGGTGATTCAGGTGATGTTCCTGCTAACTATACACTTTTGAGACGTGGTAAGCGTAAAGATTATTTTACTGGTGCATTGCCTTGGCCACA
GAAGGGTGCTTCTGTTTCTTTACCGTTAGGAACACGTGCTAATATTTATTCTGACATACCAGCTGGCAATGGTACTGCTGGTTATAGTGTTTTTCA
AACTGCTGTTGGTGCTTTAAGAGAATTAAATTCAGCTTCTAATACTTTGTCTAATAGTACAAATGCTGGTGTTGCTACTAATCAGTTATACGCTGA
TTTGTCTACTGCTACTGCTGCGACTATTAACCAACTTCGTCAATCTTTCCAGATTCAGAAGTTATTGGAGCGCGATGCACGTGGTGGTACTCGTTA
TACTGAGTTACTACGTGCTCACTTTGGAGTAACTCCACAGGATTATCGTTTACAACGTCCTGAATATATTGGTGGAGGTTCGACCCTTGTTAATGT
TAATCCGATTGCTCAGACTTCTGCAACGTCGGTTACTGGTTCTGCTACTCCGCAAGGTAACCTTGCTGCAATGGGTACTGCATTGGCTCAGGGAC
ACGGCTTTACGTATGCTGCTCAAGAACATGGATACATTATCGGATTAGTTTCTGTACGTGCTGACCTCACATATCAACAGGGTCTTCCTAAGATGT
GGTCTAGGTCTACACGATATGACTTTTATTTCCCAGTATTTGCCACTTTGGGTGAGCAAGCTATTTTGAACAAAGAAATTTATGTTCAAGGTACTG
CAGCCGACAATGATGTATTTGGTTATCAAGAACGTTGGGCGGAGTATCGTTACAAACCTTCTCAAATTACTGGTTTCTTTAGGTCTACTTCTGCTG
GCACTATTGATGCTTGGCATTATGGACAGCGATTTACTTCTCTTCCTACGTTGAATTCAACGTTTATTCAAGAGACCCCTCCAGTTGCTCGTACTAC

GGCGGTCGGAGCTGCAGCAAATGGTCAGCAATTTTTAATGGATGCTTTCTTTGATTGTCAGATGGCCAGACCTATGCCTATGTACAGCGTACCTG
GTCTAATTGATCATTTCTAATGTTTTATATAACCTCGACTACTCCGTAAGGAGTAGTGAGGAAACAACCGAAGGGCGTTAGTTTATGTTTGGTGG
AATACTTGATGCGGTTACTAATGTTGGTTCTAAGCTGTCTTCAGCTTCTAGTTTCTTTACTCCTGGTGTCGGTACTGCTTTGGGCGCTGTTGGTTCT
TATTTAGGTTCTACTTCTGCTAATAAAGCTAATCAGGAGATGGCTCAGAGGCAAATGGATTTTCAAGCCGATATGAGTGGAACAAGTTACCAGC
GTGCTGTTAAAGATTTAGAAGCTGCTGGTTTATCTCCTATGTTAGCCTATCAACGTGGTGGTGCTTCTACCCCATCTGGTTCAACTGCTACTATGG
AAAATGTTTTAGGTAATGCAACTAATTCAGCTATTAATACTGCTTCTATGATGCAACAGATTCGTAATGCATCAGAAACAGAAAAGCAGATTATC
GCCCAGACTGAAGCTACTGAAGCTGGTACCGCTAATACTAGGGCTGATACTGTTAATAAGTTGCTTACTGCTCCTAATATTACAGCCGAAAATAA
ACGTATTTTGGCTGATATTGCTTTAAAGAATACGACTGCGGATTTAACATCCGCTCAGTCATATAATACTAAGAGGCTATTGGCTCCATCCCCAGC
TATTTGGTCTAGGGGTATCGATGCTTCGAAAGAAATTTTTGATAAACTCAAAAATAATCCTAATCAACTAACCCCTTGGGGAATTGGAGTCAAAT
AATGAGTAAAGCGAATTTGCCATTTGTACGTAATCCGTACAACTATGATAAAGATGAAGCATCGGTAAACGATGCGTTGCTGTGTCAAGACCCA
AGTCTTGCTCAACAGCATATGAAAGATGAATGTGACATTAATGTCATCATTGAACGTTTCGGGGTTACAGGGGAACTTCCAACGGCCCCTGTATC
GCCTCAATACGGCGATTTTAGTGGTGTTACTGATTACCATTCTGCGTTGAATCAAATTAACGCAACTATGGACGATTTCATGGCTCTGCCAGCGAA
ATTAAGAGTCCGATTTGACCATGATCCTGTCAAATTATTGGAGTTCCTTGAGAACGACCAGAATCGTGATGAAGCGATTCAATTGGGTCTTATTG
ATGGACAACCTGTGGTTGAACCCATCGTTTCTACAGAAACACCTAAGGCCGAAGGATGAAATCCTGAGGCCAGCACAGTTACTCTACTTGATGTA
ACTGTGCTAGGTGACACCAAACCACTATTTTAACTACGGAGTTCATCATGTTACGAAGAAAGCCAGTAAACAAATATAAATCTGCAAAGTCATTT
CGCAGAACTGCTAGTAAGACGAAGTCAATTAATATGAGACACGCTCCCCAGCGTGGTGGCTATCGTTTGTAATTATGGCCTGTTATAAGCCCTTA
ACGGCTTATCAATGCAGTGACAGGTCTATAATTTGGCGGGAAATACCGGGTGCGGATGTAGTCCGTACCCTATCATTGCCTTGTGGTCAGTGTGT
TGGTTGTCGCCTTGAACGCTCACGTCAGTGGGCGATTCGTTGTATGCATGAGGCACAAATGCATACTAGTAATTGTTTTATTACTTTGACATATGC
TCCAGAGCATTGTCCTAAGGATATGTCATTGGATTACAATGATTATCAGCTTTTTATGAAGCGGTTACGTAAGCGTTTTACTGGGAAAACGATAC
GTTTTTATATGGCAGGTGAATATGGTGAATCTTTTGATCGTCCTCATTTCCATGCTTGTCTGTTTGGTCTTGATTTTCCGGATAAGAAAATATTTAA
AAGAACGCAGACTGGCTCTATCCTCTACACGTCAGAGATTTTGGAAGAATTGTGGCCGTTTGGCTATTCTACAATTGGTGATGTTACTTTTGAGTC
TGCTGCTTATGTTGCAAGATATATTATGAAGAAGATTAATGGGGTTACTGTCAATGAAAACCACGAAGTGGTTGATGCGGGTGCCCATTATCAAT
ATTGTGATTTAGAGACTGGTGAGATAATTCAGCGTAAGCCAGAATTTAATAAGATGTCATTGAAGCCCGGTATCGGGCAATCGTGGTTAGATAA
GTACATGTCAGACGTTTATACGTCAGACCACGTTGTGGTGCGTGGCAAAAAGTGCCGTCCACCACGGTTTTATGATAATAAATTTAAGTTGAAGT
TTCCTGAAGAATTCGATATGATTCAGTTTGCCAGAGAGATGGAAGGTCGATCTAGGCATGAGGACAACACGCTTGAGCGACTTGCTGTTAAGGA
AAAAGTTGCGTTGGCTAAGTTGTCATTGTTAAAACGTACTATTTAAGGAGTTTTTATGAAGATGGTTATTGTTTCTATTAAGGATACTGCTGCAGA
TGCTTTTGGTCGTCCAGCTTATGTTGCATCTGAAGGTGTTGCAGTACGTCAGTTTCAGGATGAAGTCAATCGAGCTAGCGAAGATAATCAGTTGT
ATAAACATCCTGATGATTTTCATATGTTCT

>000108F|arrow

AGGTGGTCGGATGTATATATGAGAGAACCAGAGGGAGTCCTTTTGAATAGTTTCTTATCATGAAAATCGTATCCGAAGATACAGGCATGGAAGT
GAGGTCTGCCGAAACTTGTGCCGTATTCTCCAGCCATGTAGTAACGTAATTTTGCAGGTGCAATGGATTTTCTGAAGCGCTTAAGGAACTTTTGG
AAGTCGCTTTTGACAAGCGATCCAGTTTGTGGAAGGTTTTCATTGTTGTATGTGAGGGTTATAAAGCAGTTTTTTTCGTGCAATTGGGCTTCATGC
ATGCAGCGCATGGCCCATTGACGTGATTTTTCTAGCCTGCAGCCAACGCATTGGCCGCAGGGCAGTGAAATCTGACGATCATGCTCGTCAGATTC
TTTGAATGCGACACGGCGATAAGATTTGCCGGTCTTATAGTTTGTCTGATGCTTACTTAAATAAGCAGTCAGTGGGTGATAACAGGCCATGTGAG
GTAGCCTGGCGCTTTATTAGAGCCGCCAGCCCCCTCTTTGCGGGGCTTTTTGCATATTTGCAGCTTTAGTTTTTGAAGTGTTCCTACGGAACGATT
TAGCGCTTTTGCGCTTGTTTGTTTGTTTTCTATAAAGCATAATTTTTGGCCTCGTTTATCAGATATTTTTGGTGTTGGTGTCACCTAGCACAGTTACA
TCAAGTGGAGTAACTGTGCTTATCGCCATTTCTTCCGAAATGGCTCTTTCGACCAGGCCGAGTTTCTCGGCTTCTGGTCGATTTTTCTCGTCTTGCA
AGAACTCAATCAGGTTTGCTGGTTCGTTTTCGAACCTAGCACGAATTTGAGCCGGTAAGTTATCAAATTCTTCCATAGCGTTCATTACCTTATTCAA
GGCGCTATGATAGTCAGTAATTCCGCTGAAATCGCCATATTGAGGCGGCAGCGGACTTTGAGGTAATAGGCCTGTAACGTTAAAACGTTCCAGG
ATAGTATTTATATCGCATTCGTCTTTATAATGCTGCTGAGCCAGAGTTGGCTCCTCACAAGCCAACCCTGACTCATTTGACGCAGCATCCGTGTCA
TAGTTGTATGCGGTTCTAAGTTTGATTGTTTTCATTTCATTTTTCCAAATGGTAGATATTTTTGATATTTATCATATTGGCTATGTAAATAGCCTTTA
ACATCTTGATAAATTGGTTTAGTACTGGATGGCGCTGATCCAGTTTGCGCTAATCGCTCTAATTGGTCTGTATATGCACTTTGACTTAACGCTTGT
CGCTGTTGTGCAGCTTGTAATGCACTTGATGTCCTTAATTGCTCTATTTGGGCATCGCGAAGCTGACCAAATTTGCCGTATCCCGGCATCTGAGCA
ATTTCACGAGCTGTATTTGCTCGAGTATATGTTGCCTGATCACGTGATAGATTTGTATCCGCGTCCGTTTTTTTCGGCTTGCGATTGTGTCAGGAT
ATTCTGAGTTTGTAATTGTTTAAAATTGGCGACTGCCATGGCAGCTTCACGGGCTGAATTTCCAGCCTCACCTAGTGGATTTCCTACTGGAGCTTG
CGCACCAGGCTGGACTTTGGCTCCGCCTTGTGAATAAGCCAGCATAGGGTTAAGACCTGCAGCCTTAAGATCCGCTACTGCGTCTTGATATGCAG
TACGTCGCATACCATCTTGGAATTGCATCATTGCTGCAGCTTGTTGTGCGCTGGCAGCGTTTTGTTCTTGACCACCAAAATAACTAGCGGCCGCAC
CTATTCCGGCTCCAATTAAGGAGCCGTAAGGTCCAAAAGCGGCGCCAGATGCGGCGCCTGAGGCAGCGGCTTCTAGTGCCATTAGAAGTGGTC
GATTAAGCCAGGTACAGAGTACATTGGCATTGGCCGTGCTTTTTTAATATCAAAAAAGCTATCAAAAATGATTTGCTGGCCGTTGGCAGCTGCTC
CGACCGCAAGGGTTCGAGAGACAGGTGGATTTTCTTGAATAAACGTGTTATTCAAAGTTGGAAGTGTTGTAAACCGTTGGGCTAAATGCCAGCC
GTCAATTGTTCCAGCTGCCGTACTACGGAACAGACTGGAAATTCGGCTTGGCATATAACGATATTCTGCCCAACGTTCTTGGTATCCAAACACTCC

TGTATCGTTGGCATCGCCTCGTACGTAGATTTCTTCATTTAATACTGCTTGTTCGCCCAAAGTGGCGAAAGCAGGGAAATAGAAATCATAACGTG
TGGAACGATTCCACATGCGGTGCATACCTTGTTGGTATGTTAAATCGGCTCGTACTGAAACGAGACCGATAATTACACCATGTTCAGTGCTTGAA
TAAGTAAAGCCATGATTGTGAGCGAGCCCAGTACCCATAGCGCCAAGTGTACCGAGAGGAGTAGTGGTCCCGGAAGCTCCGGTACCATTCGTCT
GGGCGATTGGATTGATATTAATGTGTGTTGAACCGCCTCCAATGTATTCAGGCCGTTGGAGACGCGCATCGGGGCTAACGACCCCGAAATGGCT
GCGGATAATTTCTGTGTATCGAGTTCCGCCACGGGCGTCCCTTTCTAAAAGTTTTTGAATCTGGAAGCTTTGACGCAATTGATTAATTGTTGCAGC
GGTTGCAGCTGATAAATCTGCATATAAACCGCCATTAGGATCATATGATTTTGCCAAGCCGTCAGCACCGCCAGTAATTTGACCAGTAACACCTA
CGTTAATAGCCTGGGCGGTTGCGTTTAATGTATTTGTTCCAGCGTTATACAGTCTGGAAACAGGCGCATTGTTAGTGCGTAATATTGGGGCGGAT
GTTCCAAGCGGTAAAGTAACGCTTGCGCCCTTTTGTGGCCAAGGTAATGCTGACGTGAAATAGTCTTTACGTTTTCCACGTCGTAATAATGTGTA
GTTGGCTACGTTATCTGGACCATCGCCAGTATCTACAACTACTGAATTTTGAAGGTTTTCGTCCCGAAACCATTCGTTATAAATAAGGTTGTAAGC
ACGTGGCCAGAAGGCACAGTGACTTACGGTGCCACCAGCACCTACTTGGCCTACGGTTGGCAGACCCATATAATCTTGAAGGCTGCCAATAGCG
TAACCACCAGCTGGGGTTACTTGTTGGGGCACTACGTACGAGATCGAGTCCGCAGGATTTGCCTGCTGCCCCATAAATTTTTGCCAATTTGACCA
GATAAGTCTATTTGGCACAAAAAAGAAGAAGCTATCCAGATGCATGTTATCCATGATCGGATAGAGTGGTGTAGACAATCGCGCAAAGGCGGTC
ATGTTGCAGCGGAATGTATCGCCTGGCAACATTTCGTCTACGAGTACAGGGACTAGGAAGCCAGCATCGAATGTAGTTTTATGTGTTGATTGAC
AGTCAAATGTAGAGCGGGGGATATCCGCTTTTGGAATCATTGTGAACTGATGGACGTCTACCGACTTGTTACGATGCATTTTTTTGAGCTCCTAG
GCCTAGTTGCGTGAGAAAAAGGGGTTTCCCCCTTTTTACTCTACGCTTAGTTTTTATCAGTAATTTTGACTTGTTTCCCTAAGGATACAAGTTTGGG
TTGTTCATGTAATTGGAACAAACCCGTATTATCGTCGAATTCGCCTAATTCAAATAGGTCGAAATCGTCGGAATGGTTATAAAGTTGATTGTCATC
GCTCTGGCGATTGACTTCGTCGCTAAAGCTCCGGATTGCTTCGCCGATAGAACGGACGAACATTGGACGACCGAACGCATCTGCTGCGCGGTCT
TTAACGGTACAGAGTACTAATTTCATGAGGATTTCCTAAGTGAGGTTACGTTTTAATAATTGAAGTTTGGCCTTTGTGACTTTTTCCTTTACGGCA
AGTCTGGCATAGGTATTGTCTTCGTGGTTGAGTTTAGCAGAAGTTTCACGTTTGTGGAGTAATTCTTCGTATTCATATGGATAATCTGATTTATAT
TTTTTGTCATAGTATTTTGGGGGTTTTACCCTTTTTCCGTTGACTACAACATAGTCATGGGGATATACGTCGGAACGATATTTTTTGTACCACTCAG
CACCAATGCCTGGTTTAAGGCTCATATTGTTATATTCTGGTAATAGCTTTATTAATTCCCCTGTTTCTATATCACAGTATGTGTAATGCTCATCCTTT
GTTATATGTTTGTTTTCTTCCATCTGGCCGTTGTATTTTTGCATAATATATCGAGCAACGTAAGCAGCTGACTCGAATGTAACGTCTCCAATGGAG
GAATAACCATGTGGCCAGAGGGTTGCA

>000047F|arrow

TTCTGTACGTGCTGACCTCACATATCAACAGGGTCTTCCTAAGATGTGGTCTAGGTCTACACGATATGACTTTTATTTCCCAGTATTTGCCACTTTG
GGTGAGCAAGCTATTTTGAACAAAGAAATTTATGTTCAAGGTACTGCAGCCGACAATGATGTATTTGGTTATCAAGAACGTTGGGCGGAGTATC
GTTACAAACCTTCTCAAATTACTGGTTTCTTTAGGTCTACTTCTGCTGGCACTATTGATGCTTGGCATTATGGACAGCGATTTACTTCTCTTCCTAC
GTTGAATTCAACGTTTATTCAAGAGACCCCTCCAGTTGCTCGTACTACGGCGGTCGGAGCTGCAGCAAATGGTCAGCAATTTTTAATGGATGCTT
TCTTTGATTGTCAGATGGCCAGACCTATGCCTATGTACAGCGTACCTGGTCTAATTGATCATTTCTAATGTTTTATATAACCTCGACTACTCCGTAA
GGAGTAGTGAGGAAACAACCGAAGGGCGTTAGTTTATGTTTGGTGGAATACTTGATGCGGTTACTAATGTTGGTTCTAAGCTGTCTTCAGCTTCT
AGTTTCTTTACTCCTGGTGTCGGTACTGCTTTGGGCGCTGTTGGTTCTTATTTAGGTTCTACTTCTGCTAATAAAGCTAATCAGGAGATGGCTCAG
AGGCAAATGGATTTTCAAGCCGATATGAGTGGAACAAGTTACCAGCGTGCTGTTAAAGATTTAGAAGCTGCTGGTTTATCTCCTATGTTAGCCTA
TCAACGTGGTGGTGCTTCTACCCCATCTGGTTCAACTGCTACTATGGAAAATGTTTTAGGTAATGCAACTAATTCAGCTATTAATACTGCTTCTATG
ATGCAACAGATTCGTAATGCATCAGAAACAGAAAAGCAGATTATCGCCCAGACTGAAGCTACTGAAGCTGGTACCGCTAATACTAGGGCTGATA
CTGTTAATAAGTTGCTTACTGCTCCTAATATTACAGCCGAAAATAACGTATTTTGGCTGATATTGCTTTAAAGAATACGACTGCGGATTTAACATC
CGCTCAGTCATATAATACTAAGAGGCTATTGGCTCCATCCCCAGCTATTTGGTCTAGGGGTATCGATGCTTCGAAAGAAATTTTTGATAAACTCAA
AAATAATCCTAATCAACTAACCCCTTGGGGAATTGGAGTCAAATAATGAGTAAAGCGAATTTGCCATTTGTACGTAATCCGTACAACTATGATAA
AGATGAAGCATCGGTAAACGATGCGTTGCTGTGTCAAGACCCAAGTCTTGCTCAACAGCATATGAAAGATGAATGTGACATTAATGTCATCATT
GAACGTTTCGGGGTTACAGGGGAACTTCCAACGGCCCCTGTATCGCCTCAATACGGCGATTTTAGTGGTGTTACTGATTACCATTCTGCGTGAAT
CAAATTAACGCAACTATGGACGATTTCATGGCTCTGCCAGCGAAATTAAGAGTCCGATTTGACCATGATCCTGTCAAATTATTGGAGTTCCTTGA
GAACGACCAGAATCGTGATGAAGCGATTCAATTGGGTCTTATTGATGGACAACCTGTGGTTGAACCCATCGTTTCTACAGAAACACCTAAGGCC
GAAGGATGAAATCCTGAGGCCAGCACAGTACTCTACTTGATGTAACTGTGCTAGGTGACACCAACCACTATTTTAACTACGGAGTTCATCATGTT
ACGAAGAAAGCCAGTAAACAAATATAAATCTGCAAAGTCATTTCGCAGAACTGCTAGTAAGACGAAGTCAATTAATATGAGACACGCTCCCCAG
CGTGGTGGCTATCGTTTGTAATTATGGCCTGTTATAAGCCCTTAACGGCTTATCAATGCAGTGACAGGTCTATAATTTGGCGGGAAATACCGGGT
GCGGATGTAGTCCGTACCCTATCATTGCCTTGTGGTCAGTGTGTTGGTTGTCGCCTTGAACGCTCACGTCAGTGGGCGATTCGTTGTATGCATGA
GGCACAATGCATACTAGTAATTGTTTTATTACTTTGACATATGCTCCAGAGCATTGTCCTAAGGATATGTCATTGGATTACAATGATTATCAGCTTT
TTATGAAGCGGTTACGTAAGCGTTTTACTGGGAAAACGATACGTTTTTATATGGCAGGTGAATATGGTGAATCTTTTGATCGTCCTCATTTCCATG
CTTGTCATGTTTGGTCTTGATTTTCCGGATAAGAAAATATTTAAAAGAACGCAGACTGGCTCTATCCTCTACACGTCAGAGATTTTGGAAAGAATT
GTGGCCGTTTGGCTATTCTACAATTGGTGATGTTACTTTTGAGTCTGCTGCTTATGTTGCAAGATATATTATGAAGAAGATTAATGGGGTTACTGT
CAATGAAAACCACGAAGTGGTTGATGCGGGTGCCCATTATCAATATTGTGATTTAGAGACTGGTGAGATAATTCAGCGTAAGCCAGAATTTAAT
AAGATGTCATTGAAGCCCGGTATCGGGCCATCGTGGTTAGATAAGTACATGTCAGACGTTTATACGTCAGACCACGTTGTGGTGCGTGGCAAAA

AGTGCCGTCCACCACGGTTTTATGATAATAAATTTAAGTTGAAGTTTCCTGAAGAATTCGATATGATTCAGTTTGCCAGAGAGATGGAAGGTCGA
TCTAGGCATGAGGGAACAACACGCTTGAGCGAACTTGCTGTTAAGGAAAAGTTGCGTTGGCTAAGTGTCATTGTTAAACGTACATTTAAGGAGT
TTTTATGAAGGGTTATGTTTCTATAAGGATACTGCTGCAGATGCTTTTGGCTCGTCCAGCTTATGTTGCATCTGAAGGTGTTGCAGTACGTCAGT
TTCAGGATGAAGTCAATCGAGCTAGCGAAGATAATCAGTTGTCTAAACATCCTGATGATTTTCAATGTTCTATTTGGGTCTTTTTGACGGACGAGC
CACTGGTGTTTTTGAATACTGGAAAGCCCTAAGTTGATTGCTCGTGCAAAAGATGTAATGATTCGCGAAGGCGAGTAAGGTTTTTTTTATACCGT
ATCACTCGAAAGAGTGTACGGAACTACGGAGATGTTATGTTTCGCAATAAGTCAGTAAGTACCGCATTCATTTTGCTATGGTTCCTAAAGCGGAC
ATTTCCCCGCTCTAGTTTTAATACTCAATATGCTCATAAAACCACGTTTGATGCTGGTTTTTAGTTCCTATTGATTGTGATGAAAGTATTGCCTGGC
GATACTCATCGTGTAAAGATGACTGCATTTTGCACGTTTGGCACCACCGTATTTCCTGTGATGGACAACTTGCATCTTGATACTTTCTTTTTCTTTT
GTACCTAATCGTTTACTTTGAACAATTGGCCAAAGTTTTATGGGTGAACAAACGAATCCTGGTGATTCTATTTCTTTTTGTAGTGCCTACTATACTA
GTCCTGCTGGTGGTTATGCTGTTGTTCAATTTTTGATTATTTTGGTTACCTACTGCTGGTCAGATTACTGGCGCTAATACAGTAACGCATAATGTTT
TGCCGTTTACGTGCTTATAATGAGATTTATAACGAATGTTTAGAGATGAAAACTTACGAATTCTGTAACGTAAATCTTGGTGATCTCAGGTGATGT
TCTGCTAACTATACACTTTTGAGACGTGGTAAGCGTAAAGATTATTTTTACTGGTTGCATTGCCTTGGCCAAGAAGGGTGCTTCTGTTTCTTTACC
GTTAGGAAACACGTGCTACTAATTTATTCTGACATACCAGCTGCAATGTACTGCTGGTTATAGTGTTTTTCAAACTGCTGTTGGTGCTTTAAGAGA
ATTAAATTCAGCTTCTAATACTTTGTCTAATAGTACAAATGTGGTGTTGCTACTAATCAGTTATACGCTGATTTGTCTACTGCTACTGCTGCGACTA
TTAACCAACTTCGTCAATCTTTCCAGATTCAGAAGTTATTGGAGCGCGATGCACGTGGTGGTACTCGTTATACTGAGTTACTACGTGCTCACTTTG
GAGTAACTCCACAGGATTATCGTTTACAACGTCCTGAATATTGGTGGAGGTTCGACCCTTGTTAATGTTAATCCGATTGCTCAGACTTCTGCAACG
TCGGTTACTGGTTCTGCTACTCCGCACAAGGTAACCTTGCTGCAATGGGTACTGCATTGGCTCAGGGACACGGCTTTACGTATGCTGCTCAAGAA
CATGGATACATTATCGGATTAGT

>000013F|arrow

GGATACCATTTCAGGAGACGCAACATCAAACGATAAATTTACGGTAATTCAAACAGATCCTGGAAATACGACTGCTTTAGCTAGATATGGCAAC
GCTTATGGTGTTAATACTGCTGGTGTAGTAAATAACGTTTCTAATTTATATACCGACTTATCAGAAGCAACTGCTGCAACTGTCAATCAATTAAGA
CAGTCATTTCAAATTCAAAAATTACTTGAAAGGGATGCACGTGGCGGAACACGATACACAGAAATTATCCGGAGTCACTTTGGAGTTATTTCCCC
AGACGCCCGTTTACAAAGGCCTGAATACCTTGGAGGCGGTTCAACACCAATTAATGTTAATCCGATTGCTCAAACGTCGGGAACAAACGCTTCTG
GAACGACTACCCCTTTGGGCAACCTTGCTGCTATGGGTACTGCTCTCGCTCATAATCATGGATTTACTCAATCATTTACTGAGCATGGCGTTATTA
TTGGATTAGTATCCATTAGAGCAGATCTTACTTATCAACAAGGATTAGACCGTATGTGGTCTAGATCTACACGATATGACTTTTATTTCCCAGCAT
TTGCTACTCTAGGCGAACAATCTGTTTTGCAAAAAGAAATTTATGCAACAGGAGATACTGCAGCCGACAATACTGTTTTTGGATATCAAGAACGC
TGGGCGGAATATCGTTACAAACCATCTAAAATTACTGGTTTGTTCAAATCAACATCGGCGGGCACGATCGATGGTTGGCATTTGGCTCAAAAATT
TACCGCTGCGCCTACTTTGAATAATACGTTTATTCAAGATACGCCTCCTGTATCACGTGTAGTAGCCGTTGGAGCAGCTGCAAATGGCCAACAATT
CTTATTTGACTCATTTTTTGATGTCAAAATGGCAAGACCAATGCCAATGTATTCAGTACCTGGCTTAATAGACCATTTCTAATGGGACTATTTGAC
GGAATTGCCGATTTAATCGGCCCTGCTATAGCTATAGGAGCTGCCCCTGCTACTGGGGGACTCTCCTTAGCTGCACTTGCACCTGCAGCAATAGG
TGCAGCAGGACAATACTTTGGAACACAAAGTCAAAACGCAGCGAGTGCAGAACAAGCGAGTAATCAACAGAGATTTCAAGCTGAAATGTCTGG
AACATCATATCAACGAGCAGTTGAAGATATGAAAAAAGCTGGGTTAAATCCCATGCTTGCGTATTCACAAGGCGGAGCCACAACACCAGCTGGA
GCTATGGCCCAGATGCAAAATGTTCTCGGTAATGCAACTACGTCCGGAACCCAAGCTTATCAAACGGTTGCGCAAGCAAATCAAGCTATTGCTCA
ATCTAAACAAATTGAAGCTCAAACAGAACTCACAAGTAATCAAACAGATAATGTACGTGCTGATACGTTAAACAAATTGGATGAAAATCCAAATA
TTAGAGCTCAATATAAACAAATACTTGCCGATACTTTCATGAAAAATGAAATAGGCAAAACATCAAGTGCTCAAGCTGCTCAAGCTTTGGCACAA
TCTCGTTATTCAAACGAGTTAACAAAACTTGCTAAATCAGGGTCAGCTCCTAGTTCTAGCAAACCAATTTATCAAGACGTAAAAAACATCGCCAAA
GATGCGTATAGCGCATCTGGCGCAAAACGATACATCGATAACTATCGAGGTCAACCGATTCAACAAAATCGTACAAATAACCAACCACCAATGG
AATGAAAATGACAAAGATTACAGCCCCATTTCTTCGTACTCCGTACAATTACGACACGATTGCTGCGTCAAATGAGTCAGGGCTGCATTGTGAGG
ATGCAACTCTGACTCAGCAGCAATTTGCTGAAGAATGCGATATTAATAATATTATGGAAAAGTTCGGTATGACCGGACTTATTCCTCAAACTCCTT
TAACGCCTCAATATGGCGACTTTAGTGGTGTCTATGACTACCACTCTGCTCTGAACCAGATTATGGCTTCAGACAACGAATTTATGGCTTTACCAG
CCAATATTCGTGAACGATTCGCTAATGATCCCGCGAATCTAATAGATTTTCTAGAAAACCCTGAAAATCGCAGCGAAGCTGAAAAAATGGGACTG
GTAAAACCAGCCCAAACCGAGGTTTCAACCCCTGTTGGAACCTCGGAAGCACAGTTACCTACTTGATGTAACTGTGCTAGGTGACACCAAACCAC
AAAAACACGATAAACAAGGACAGAAAAAATGATGCGTCGCAGACCAGCAAATAAGCAAAAGTCCGCTAGGACTTTCCGTAAACATGCTTCACAT
ACAAAACACGCAAATATGCGAAACTCGCCAATGCGTGGAGGCTGGAGACTCTAATAAAGTCCCCAGGCACCTCACATGCCTTGTTATCACCCTCT
CAAAGCATTTCAATGCTTTGACAAATCAATTGTTTTCGACGAAGTTCGGAAACATGACATCGTTCGATCTTTAGACCTGCCCTGTGGGCAGTGCGT
TGGATGCCGTCTAGAACGATCAAGACAATGGGCTATTCGGTGCATGCACGAAGCCCAATTGCATAAAAACAACTCATTCATAACACTCACATATG
ACAATACACATCTCCCAAGCGATGGCTCTTTGGATCACAAAGACTTTCAACTGTTCCTTAAAAGACTTAGAAAAACTCTCGCAAAAAGAGGACTT
ACAATCCGCTATTACATGGCTGGAGAATATGGTGAACTCTTCGCAAGACCCCACTTCCATGCCTGTATCTTCGGATACGACTTTCCTGATAAAAAA
TTATGGAAAAGGACTGCCTCTGGTTCTATGTTATATAGATCCGCAGAACTTGAAGCTCTCTGGCCATTTGGTTATACCACCATTGGAGATGTTACT
TTCGAATCAGCCGCCTACGTGGCTAGATACATAATGAAAAAACAAACAGGGAAAGATGCGGAATCTCATTACAAACGCATACACCCTGAAACCG
GCGAATATTTAGACTTAAAGCCGGAATATAATAAAATGTCTTTAAAACCGGGAATCGGTAAAGACTTTTATATAAAATATACTTCGGATATATAC

CCGCAAGACTACGTAATACTTAGAGGTAAAAAGGTCAAACCACCAAAATACTATGACAAAATGTTTAAAATTGACCAACCTTATGAGTATGACGA
ATTACTTTACATGCGGGAAAATAACGCTAAACTTAATTCCGAAGACAATACACCAGAACGACTATCTGCAAAAGAACAAGTAACTATGGCAAAA
CTTCAACTATTAAAACGTAACCTCACTTAGGAAAATAATGAAACTTATCCTCGCTTCCGTAAAAGACCGTGCTGCTGAAGCATATGCACGACCAAT
GTTCGTACCTTCCTCTGGAGTAGCTATACGCTCTTTTTCAGATGAAATTAATCGTTCTGATACTGAAAATCAACTTTTTAATCACCCTGATGACTTC
GATCTATATGAATTCGGAACTTTGACGATTCAACTGGGTTATTCGATTTACATGAACAACCAAAACTCCTATCATTAGGAAAACAAGTTAAACTTA
AATAAAACAACCGAGGGGAAAAGAGATTTATCTTTCCCCCGGAACAACACTAAGGAAAAACATGCACCGCAATCAGTCAGTTAATACTCACCGC
TTCGCGATGGTACCTAGAGCCGATATACCACGTAGTAAATTCGATGCTCAAAAAACACATAAAACGACTTTCGATGCGGGCTATTTAATTCCTGT
ATATGTTGATGAAGTGCTCCCTGGGGACACTTTCAACTTAAAAATGACGGCATTTGCCCGTCTAGCAACGCCTTTATATCCAATCATGGACAACAT
GATTATGGATTCTTTCTTTTTCTTTGTACCCAATCGCCTTATATGGAATAACTGGCAAAAATTTATGGGTCAACAAGAAAATCCAACAGACTCAAT
ATCTTATATTGTCCCAACTCAAACAAGCCCAACAGATGGTTATGCCGTAGGCAGCCTTCAAGACTATATGGGCTTACCAACAGTAGGCCAAATTG
ATACTGGCCGAACTATTACGCACTGTGCCTTTTGGCCACGTGCATACAATCTTATTTGGAACGAATGGTTCCGAGATGAAAATTTACAAACAAGC
GCAGTAGTTGATAAGGGCGATGGCCCTGATACTTCCTCAAACTATGTGCTAAAACGTCGTGGTAAAAGACATGATTACTTTACGTCAGCATTACC
ATGGCCACAAAAAGGTGCGAGTGTCACCTTACCTTTAGGTACTACGGCTCCAATTAAATG

>000008F|arrow

CGTGGCTAGATACATAATGAAAAAACAAACAGGGAAAGATGCGGAATCTCATTACAAACGCATACACCCTGAAACCGGCGAATATTTAGACTTA
AAGCCGGAATATAATAAAATGTCTTTAAAACCGGGAATCGGTAAAGACTTTTATATAAAATACTTCGGGATATATACCCGCAAGACTACGTAATA
CTTAGAGGTAAAAAGGTCAAACCACCAAAATACTATGACAAAAATGTTTAAAATTGACCAACCTTATGAGTATGACGAATTACGTTACATGCGGG
AAAATAATGCTAAATTTAATTCCGAAGACAATACACCAGAACGACTATCGCAAAAGAACAAGTCACTATGGCAAAACTTCAACTATTAAAACGTA
ACCTTACTTAGGAAAATAATGAAACTTATCCTCGCGTCCGTAAAAGGACCGTGCTGCTGAAGCATATGCACGACCAATGTTCGTACCTTCCTCTG
GAGTAGCTATACGCTCTTGTTTCAGATGAAATTAATCGTTCTGATACTGAAAATCAACTCTTTAATCACCCTGATGATTTCGATCTATATGAATTCG
GAACATTGACGATTCAACTGGGTTATTCGATTTACATGAACAACCAAAACTCCTATCATTAGGAAAAACAAGTTAAACTTAAATAAACAACCGAG
GGGAAAAGAGATTTATCTTTCCCCCGGAACACACTAAGGAAAAACATGCACCGCAATCAGTCAGTTAATACTCACCGCTTCGCGATGGTACCTAG
AGCCGATATACCACGTAGTAAATTCGATGCTCAAAAAACACATAAAACGACTTTCGATGCGGGCTATCTAATTCCTGTATATGTTGATGAAGTGC
TCCCTGGGGACACTTTTCAACTTAAAAATGACGGCATTTGCCCGTCTAGCAACGCCTTTATATCCAATCATGGATAACATGATTATGGATTCTTTCT
TTTTCTTTGTACCCAATCGCCTTATATGGAATAACTGGCAAAAATTTATGGGTCAACAAGAAAATCCAACAGACTCAATATCTTATATTGTCCCAA
CACAAACAAGCCCAACAGATGGTTATGCCGTAGGCAGCCTTCAAGACTATATGGGCTTACCAACAGTAGGCCAAATTGATACTGGCCGAACTAT
TACGCACTGTGCCTTTTGGCCACGTGCATACAATCTTATCTGGAACGAATGGTTCCGAGATGAAAATTACAAACAAGCGCAGTAGTTGATAAGG
GCGATGGCCCTGATACTTCCTCAAACTATGTGGCTAAAACGTCGTGGTAAAAGACATGATTACTTTACGTCAGCATTACCATGGCCACAAAAAGG
TGCGAGTGTCACCTTACCTTTAGGTACTACGGCTCCAATTAAATGGGATACCATTTCAGGAGACGCAACATCAAACGATAAATTTACGGTAATTC
AAACAGATCCTGGAAATACGACTGCTTTAGCTAGATATGGCAACGCTTATGGGTTAATACTGCTGGTGTAGTAAATAACGTTTCTAATTTATATAC
CGACTTATCAGAAGCAACTGCTGCAACTGTCAATCAATTAAGACAGTCATTTCAAATTCAAAAATTACTTGAAAGGGATGCACGTGGCGGAACAC
GATACACAGAAATTATCCGGAGTCACTTTGGAGTTATTTCCCCAGACGCCCGTTTACAAAGGCCTGAATACCTTGGAGGCGGTTCAACACCAATT
AATGTTAATCCGATTGCTCAAACGTCGGGAACAAACGCTTCTGGAACGACTACCCCTTTGGGCAACCTTGCTGCTATGGGTACTGCTCTCGCTCAT
AATCATGGATTTACTCAATCATTTACTGAGCATGGCGTTATTATTGGATTAGTATCCATTAGAGCAGATCTTACTTATCAACAAGGATTAGACCGT
ATGTGGTCTAGATCTACACGATATGACTTTTATTTCCCAGCATTTGCTACTCTAGGCGAACAATCTGTTTTGCAAAAAGAAATTTATGCAACAGGA
GATACTGCAGCCGACAATACTGTTTTTGGATATCAAGAACGCTGGGCGGAATATCGTTACAAACCATCTAAATTACTGGTTTGTTCAAATCAACA
TCGGCGGGCACGATCGATGGTTGGCATTTGGCTCAAAAATTTACCGCTGCGCCTACTTTGAATAATACGTTTATTCAAGATACGCCTCCTGTATCA
CGTGTAGTAGCCGTTGGAGCAGCTGCAAATGGCCAACAATTCTTATTTGACTCATTTTTTGATGTCAAAATGGCAAGACCAATGCCAATGTATTC
AGTACCTGGCTTAATAGACCATTTCTAATGGGACTATTTGACGGAATTGCCGATTTAATCGGCCCTGCTATAGCTATAGGAGCTGCCCCTGCTACT
GGGGGACTCTCCTTAGCTGCACTTGCACCTGCAGCAATAGGTGCAGCAGGACAATACTTTGGAACACAAAGTCAAAACGCAGCGAGTGCAGAA
CAAGCGAGTAATCAACAGAGATTTCAAGCTGAAATGTCTGGAACATCATATCAACGAGCAGTTGAAGATATGAAAAAAGCTGGGTTAAATCCCA
TGCTTGCGTATTCACAAGGCGGAGCCACAACACCAGCTGGAGCTATGGCCCAGATGCAAAATGTTCTCGGTAATGCAACTACGTCCGGAACCCA
AGCTTATCAAACGGTTGCGCAAGCAAATCAAGCTATTGCTCAATCTAAACAAATTGAAGCTCAAACAGAACTCACAAGTAATCAAACAGATAATG
TACGTGCTGATACGTTAAATAAATTGGATGAAAATCCAAATATTAGAGCTCAATATAAACAAATACTTGCCGATACTTTCATGAAAAATGAAATA
GGCAAAACATCAAGTGCTCAAGCTGCTCAAGCTTTGGCACAATCTCGTTATTCAAACGAGTTAACAAAACTTGCTAAATCAGGGTCAGCTCCTAG
TTCTAGCAAACCAATTTATCAAGACGTAAAAAACATCGCCAAAGATGCGTATAGCGCATCTGGCGCAAAACGATACATCGATAACTATCGAGGTC
AACCGATTCAACAAAATCGTACAAATAACCAACCACCAATGGAATGAAAATGACAAAGATTACAGCCCCATTTCTTCGTACTCCGTACAATTACG
ACACGATTGCTGCGTCAAATGAGTCAGGGCTGCATTGTGAGGATGCAACTCTGACTCAGCAGCAATTTGCTGAAGAATGTGATATCAATAATATT
ATGGAAAAGTTCGGTATGACCGGACTTATTCCTCAAACTCCTTTAACGCCTCAATATGGCGACTTTAGTGGTGTCTATGACTACCACTCTGCTCTG
AACCAGATTATGGCTTCAGACAACGAATTTATGGCTTTACCAGCCAATATTCGTGAACGATTCGCTAATGATCCCGCGAATCTAATAGATTTTCTA
GAAAACCCTGAAAATCGCAGCGAAGCTGAAAAAATGGGACTGGTAAAACCAGCCCAAACCGAGGTTTCAACCCCTGTTGGAACCTCGGAAGCA

CAGTTACCTACTTGATGTAACTGTGCTAGGTGACACCAAACCACAAAAACACGATAAACAAGGACAGAAAAAATGATGCGTCGCAGACCAGCAA
ATAAGCAAAAGTCCGCTAGGACTTTCCGTAAACATGCTTCACATACAAAACACGCAAATATGCGAAACTCGCCAATGCGTGGAGGCTGGAGACT
CTAATAAAGTCTTCAGGCACCTCACATGCCTTGTTATCACCCTCTCAAAGCATATCAATGCTTTGACAAATCAATTGTTTTCGACGAAGTTCGGAA
ACATGACATCGTTCGATCTTTAGACCTGCCCTGTGGGCAGTGCGTTGGATGCCGTCTAGAACGATCAAGACAATGGGCTATTCGGTGCATGCAC
GAAGCCCAATTGCATAAAAACAACTCATTCATAACACTCACATATGACAATACACATCTCCCAAGCGATGGCTCTTTGGATCACAAAGACTTTCAA
TTGTTCCTTAAAAGACTTAGAAAAACTCTCGCAAAAAGAGGACTTACAATCCGCTATTACATGGCTGGAGAATATGGTGAACTCTTCGCAAGACC
CCACTTCCATGCCTGTATCTTCGGATACGACTTTCCTGATAAAAAATTATGGAAAAGGACTGCCTCTGGTTCTATGTTATATAGATCCGCAGAACT
TGAAGCTCTCTGGCCATTTGGTTATACCACCATTGGAGATGTTACTTTTGAATCAGCCGCCTA

>000190F|arrow

TATGTCAACGTTAATCCTATTGCTCAAACGTCTGCTACTTCGATTTCTGGCGGTGCTACTCCGCTTGGTAACTTGGCTGCAATGGGTACTGCGTTG
GCTAGTGGACATGGTTTTACGTATCATGCTCAAGAACATGGATACATAATTGGTTTGGTAAACGTAAGAGCTGATCTAACATATCAGCAAGGTTT
ACCTAAGATGTGGTCTCGTGAGACACGTTATGATTTCTATTTCCCTGTATTTGCTCATTTAGGTGAACAGGCTGTTCTTAATAAGGAAATCTATGT
TACTGGTACTTCAACTGATGATGATGTATTTGGATACCAGGAACGTTGGGCAGAGTACCGTTACAAACCTTCTCAGATTACAGGTTTGTTTAAGT
CGACTTCAGCTGGTACGATTGACCCTTGGCATTATGCTCAGAAGTTCACTTCGTTGCCGACTTTGAATTCTACGTTTATTCAAGAAACGCCACCTA
TTGATCGTACTACTGCTGTTGGTGCATCTGCTAATGGTCAGCAGTTTTTGATGGATGCGTTTTTTGATTGTAAGATGGCTCGTCCAATGCCGATGT
ATTCTGTACCTGGCTTGATTGACCATTTCTAATGTAATATAAGCTGGACTACTGGGAAACCAGTAGTCAGCAAACAAGCGGAGCGCGTTAGTATG
GGTATGTTTGATTCAGTTGCTGGTGGTTTATTTGATTTTGCTACTTCTGCTTGGAGTCAGAATGAAGAGCAAAAATATACTGCTGAAGCGCAAGC
AAATAGACGCTTTCAAGAAGATATGTCCAATACGCAATATCAGCGTATGGTTACTGATTTGAATAAGGCTGGTTTGAGTCCTATGCTTGCATATTC
TAAAGGTGGTTCTACTGTTCCGAGTGGTGCTACTGCATCTTCTACTTCTCCAGTTAAGTCTGATTTGAGTGGAGCTGTTAAGCGTGATGTTGAGA
GTGATTTATTGCGTGAGCAGATGGAAGTTGCTAAATCTCAACGTGAATTAAATGTTCAGACTGCTAGGAAGGTTGCTGAAGAAGCTGATTTAGC
TTCTCAGCGTGTTTTACAAGAACCGGCTCGTTTTTATTTGGAACAGGCCGAGGCTGGTTCTCGTGTTAATGCTAATTCTGCATCAACAATTAAGAC
TGATATTGATGCTAAGAATAATTTAGAGTTACGAACACCGTCTAGCGATCCATATTGGTATCGCGATATTAAGAAAGGTAGTAAGTCTATTTTGA
TAAGTTTTTGGAAAAGAATAAGTCTTTTCCTTGGTCCTTGGAAGGTCGTAGAAAATGAGTAAAACGAATTTACCTTTTGTACGTAATCCGTACAAT
TATGATATGGCTCTTGTTTCACAAGAGACTGGTCTTGAATGTAAAGATCCGAGTTTGGCTCAACAACACATGAGAGACGAATGTGATATTAATAT
TATAGTTGAGCGTTTTGGCGTTACTGGGCAATTGCCCCAGGCGCCATTAGAGCCATCATATGGCGATTTTAGCGGTGTATCTGACTATCACACCG
CATTGAATGCTATTCGTGCCTCTGATGAGGCTTTTATGGCTTTGCCAGCTAAGATTAGGGCTAAGTTTGATAACGATCCTAATGCTTTGTTAAATT
ATTTACAGAATGAAGAGAATCGTGATGAAGCGATTCAGATTGGTCTTATTGACGGTAAACCTGTGGTTGAACCCGTCGTTTCTGCAGTAGAAAC
ACCTAAGCCAGACGCGTAAGCGGATGGCAGCACAGTTACTCTACTTGATGTAACTGTGCTAGGTGACACCAAAACCACATTATTAACTACGGAG
TGCAATGTTATGAGCCTTTATAGAAAACCAATGAGCAAGCATGGCGCAGCTAAGAAGTTTCGTCGTGGCGTAAGCAAGACCAAGAGCATTAATA
TGCGTACTTCACCGCAGCGCGGTGGTTTTAGACTGTAATTTATGGCGTGTTATAAGCCGTTAACGGCTTATCAATGCGCTGACAAGTCTATTATTT
GGCGGGAGATACCAGGGGCGGACGTAGTCCGTACCTTGTCATTGCCTTGTGGTCAGTGTGTTGGTTGTCGCCTTGAACGGTCCCGTCAGTGGGC
CGTTAGATGTATGCATGAGGCACAAATGCATACTAGTAATTGTTTTATTACTTTGACATATGCTCCAGAGCATTGTCCTAAGGATATGTCTTTGCA
TTATGAAGATTTTCAATTGTTTATGAAACGATTGAGAAAACGCTATACTGGCAAGACTATTCGTTTTATATGGCAGGTGAATATGGTGAGTCTTTC
GATCGTCCTCATTTCCATGCTTGTATCTTTGGGCTTGATTTTGAAGATAAGAAGTTTTTCAAAAGAACGCAGACTGGGTCTATCTTATATACGTCA
AAGATACTTGAAGAACTTTGGCCGTATGGCTATAGTTCTATTGGTGATGTCAATTTTGAATCTGCTGCTTATGTTGCTCGATATATTATGAAGAAG
ATTAACGGTAAAACCGTTAATGAGAACCACGAAGTGGTTGATGCAGATGCGCATTATCAGTATTGTGATTTAGATACTGGTGAGATTATTCAGC
GTAAGCCTGAATTTAATAAGATGTCTCTTAAGCCTGGCATTGGTCAGGCTTGGTTTGATAAGTTCATGTCAGACGTTTATACGACTGACTCTGTTG
TGGTGCGTGGCAAAAAGTGCCGACCACCACGGTTTTATGATAATAAGTTTAAAGTATTGTTTCCAGAAGAATTTGATGGTATACAATATGCTCGT
GAGCTAGAAGGTCGCTCACATTTTGAAGATAACACTTTAGAGCGTTTGGCTGTAAAGGAAAAAGTCGCTTTGGCTAAGTTATCGCTTTTAAAACG
TAAGATTTAAAGGAGTTTTTATGAAGATGGTTATTGTTTCTATTTTAGATACTGCAGCTGGTGCGTATGGTCGTCCAGCTTTTGTTGCATCTGAGG
GTGTTGCTGTTCGTCAGTTTCAGGACGAAGTTAATCGCGCTAGCGATGATAATCAGTTATATAAACACCCTGATGATTTTCAGTTATATTATTTTG
GTACGTTTGATGATAATTCTGGTACTATGGATTTGCTTGGTTCTCCCAAGCTTATTTCTAGAGCTAAGGATGTTATGATTCGCGATGGCGAGTAAT
GTTTTTTTTAAGCCGGATCACTAGCTTGCTAGTGGTTCGGAATACTTCGGGAGATTGTTATGCATCGTAATAAGTCAGTAAGCTCACATAGTTTTG
CTATGGTTCCTAAAGCGGAAATTCCGCGTTCTAGTTTTGATACGCAATATGCGCATAAAACTACTTTTGATGGTGGTTATCTAGTTCCTATTTATTG
TGATGAAGTGCTCCCAGGGGACATGCACAATGTAAAGGCTACAATGTTTGCCCGTTTGGCAACGCCTTTGTTTCCAGTTATGGACAATTTGCATT
TAGATACTTTCTTTTTCTTTGTTCCAAATAGATTAGTTTGGAACAATTGGGTTAAGTTTATGGGTGAGCAAGCGAACCCAGGTGATTCTATTTCGT
ATGTAGTTCCACAGATTACTTCTACTGCTGGTGGTTATGCAGTAGGTTCTATTTTTGATCATTTTGGTTTGCCTACGGCTGGTCAGATTACTGGTAC
TAATACGGTTACGCATAACGCGTTACCGTTACGTGCTTATAATTTGATTTATAACGAGTGGTTTAGGGATGAGAATTTACAAAATTCTGTTACCGT
TCACAAGGGTGACGGTCCTGATACTCCGAGTGATTATGCTATGGTTCGACGCGGTAAGCGTAAGGATTATTTTACTGGTGCCTTGCCTTGGCCTC
AGAAAGGCGATGCAGTTACGTTGCCTTTAGGCACGTCTGCTCCTATTAAGACGTCTGTTACTAATGATTCAACAACGTTGTCATTAGTTGACGGT
GCTGGTGCTTTGAAGCAGATTTATGCTAATTCAACTACACATGCTGTGTATCGTAGTAATAACGCTGGTACTGCTGGTACTGGTTTGTATGCCGAT

TTAAGTGATGCTACGGCTGCAACTATTAACCAGTTACGTCAGTCTTTCCAGATTCAGCGTTTGTTAGAGCGCGATGCGCGAGGTGGTACACGCTA
TACAGAATTGTTACGTGCTCATTTTGGTGTAACTCCACAAGATTATCGTTTACAACGTCCTGAATATATTGGTGGTTCTACT

>232|arrow

GCCACAAGCGGGTAGTTGTATTAATAAAATTATTAAAGTTAATCATAAATTAAAGAGAATGACATTCAACCCCGATTCCTCATCTGTCAATGAATG
TACTAATCACCGAATGTATTGTGCTCTACAAACTGTAGCAGTTGATGGAACAACTAACACTGCTGGTACATCTGGAATTAATGTAATATATACTCA
TAAAATTCTTTTTAAAGATGCCTAATTCTAATGTACTACAAATAAGATTTATTTTATATACATCGGAAAAAAAAACACTATGCCACCCGCGGGGGT
TTGGGGGGGGCGGGTGCGACACCCCCCCATATAAGACATCACGCCGAAGGCGGTTTAACTTCATGCCCGAAGGGCATCATCTATTTGCGATAG
AGTCAAATAAGATTCCATTTCAGATTCCATTGAATTGAGGAGGCTACTTAATAGACTCCTCACCCTAATACCCTAAGAGCCTAAAGCCTAAAAACC
TATGTATATATAGAACCCTAAAATGAATATTGCTCTACGAATCACAATTGATGAACTCCCCGACTTTGACTGGTATGGTCTGTGTAAAAAAGGTA
GTTATATACCGGCACGAAGCAGATGCGAAAGGTGTCGGCAGACGCACTATCACGCGTTGATAGTAGGATATACGAAGAAGGAACAACATTCCG
AAATTACTTCGTTAAGAAATATAAATACTCTATGAAAACTACTGGTGTAGATGAAAATTTCATAACATATATGTCAAAAGGAAAATTAGACCCTCA
TTTTGTAATAGGAATGACGAGTGATGAAACACGAGCTATAAGGAAAAATGGGTGGAAATAAAACACACTGTTCTCCCCCTTGTCGTCGCTCATA
ATGGGCAAATAGTGAAACAAGTCCAGCCCGATAAGGAAAAAGAAATCAAATGGGATTGATAGAGATAATGCGTGCAGATTATAATATCGGTAT
GTCGCAAAGAGATGTATTAAATGCTAAGGAAAATATTATTACAAGAACATCAAGCAATAAGAATGTATACGATACTGGCATTATTATGATAGTGT
CGTAATTGTGGCAGACAAAAATACTTGGTTGGACTCTGCTGAAATAATCCTGAAAAACGCAACCGCGTATAAAGTTTAAAGAATAAAATATATTA
TCCATAGTATAACCCTGAAATGCCATCTCCGATACAACCAACGCAAGTATGCTCAAACGACCCAAGAAGAAAGCAAGAGAAGTGCAAAGTTAAG
CAAACCTGTAAAGAATGCTATTACTAAAATGATTACTGTTAAGCAAGAAACAAATGTTTCTCATTTGCCCCGAACCCTGCTTCGTTTAATCCCGTG
CTTCCTGCTTTGAATCTTGATAATGGTGCTATGATAGGACAAAGTACTGTCACACCAAACCCTCCGACGATAAGTCTGGATTCCCAATTTGCCACT
GGTGTAGATATGTGGTATAGAATTGGTAGTCGTATTGATATTAAAAGTTTTTCAACTATATTTCATTTTACTCTTAGAGACCAAGGATTGAAGAAT
CCGCAGTGGGTACGATTTTGGATATATCGTATTAAAAATGAAGATGGTCCAGTTGATTTAACAAACACAACCTCGCAAGAATGGTTCTTGCGCAA
TGGTACTACTACTGGATTTAGTGGAAATCTGTATGATTTACAACGCCCTGTCAATTTTAGACAAATAGAAGTATTCTATGACAAGATATTTATGTT
ACACGCAGAGCAACCTGGCACTGCGAACGGACAACC

>000224F|arrow

TTGAATGTCCCCTACCACTATACTAACTCTTAATCAATAAAAAACGTTTATTACGTATTTGTATAGTGGTATGGGATACCTCAAAAACAGAAACGC
CTATCAATTTTTGGACCAAGGCCCAAAAATCGGACATTACAAAAATTCGAGCCGTCCGATGTATCTATTTTACTCGCTTGGCCCTTGGCCAACCTC
CTAAAATGGTCCGCACTGGAATGCGATAATCTCTGGAATCAGAGGTCCTTTAGGCCGGTCGTCTTGGCCATACTGCGTATCCGGCCGGCGCTCTT
ATCCGGCCACAGGACTGATACATTTAAATAGGTTTAGGGAAATAGGTTTTTATTAGGTTAGGGAAAAACCCTTAAAAATCCCTAACCTAGGTATA
GGGTTCTATAGTACTGACAAGGTTACCAACTCGGTAGTAGTTGTCAACTTGGGCATATAACAGGCACAGCCGGTTTTTAGTTCAATTTCATATGC
AATACGAATACCGTTTTCAGCACCGGTAGCTACAGCTTGCAACTGTTTTTCGTATACTAAAACACGCTGTTTACCCATTTTTAAAGTATTATATACA
GACGATGTTTTGCCAATTAACATCATCAACGTATTAATTTGCATTTTACGTCGATACTTCAAACTAGACGTTTTCAAATGTCCAGGATCCAAAGTA
ATTTTACTGGACGTTTTCATTCCTTTAAGTTGACTTAACGGTGGTGGTTCATTCCAAGGTGACGCATTATATCCGTCAACTTTCAAATTCTGTTGAA
AGGTTGTCGGTGCAAAATAATACGTTTCTTCCACACTATAAAAATTATAAGGGCCGTCATAACTTCGGCCTTGCAAAGGCACATTATCAACTTCGT
CTGCTTCAGTATTAGCCGCCGCTGATACTGAACGATTCTGCACTTTAAGTTCAGACTGAATATACATATCTACAGTCATTCGCTTTAAATCCCATTT
TCCGATTTGTTCAGTGCCAACTACAGGAGTAGTGGCAAGCTGATCTCGATAGACCTCCAAATGGGTGAACTGTAAATCAGAGTTGGCATCAACC
AAAGCTTTCAACGGAGTTGATATACTATCCGCCAACTCTTGAACCGTATTATTAGTACCCGTCATAGTGACAACAAATTGTGTCACTGCGCCGGCT
GGATTAAGTTTGTACTTGAATACCAATACAACATTGCGACCATTTGAAGCATTAATATTCGTAGTATTCATATTCTCTACATTAATGTCAAAACATT
GCACCGCAATAGCTTTAGCCCATGCTAAAGACAAATCTGCAACAATTTGATCTGATCCGCACGTTGCTTGCTGAAACACAAACGTCTGATAAGTA
TTCAAATTATTAGATATCAAAACAGTTCCCGTTTCACGGGTTGTTATAATGCCCTTGCGGGCAAAATAATCAAACACATTTCTCTTACCGGTTGACT
TCTTAATTTTACCACTAGACTGGCCGGTTTGACTACGGCTCCGTCTAACCTTCTTAAAGTTCAACCTTCTTTTAACACTACTGAGTATAGTGTATCCT
CGACCCTTCTTTAGTATCTGTCTGCCTGTAGCAGACATCTTAGACATCTGTTTAACATTCTTGCGAGGAGTCTTCGGAGGAGTAGGATATGAAACC
GGGGGCATCTTGTTTGTTTTTTTAGAGAAAGAACCTACGATCTTTCTTCCGGTGTGATAGGCTGCCTGAGCATATCGCATATACTGCGCCGTTTTC
GCCATTTTGTACCCAATATGCCGCACTGCAGGAGCAAGGTCAGCCCCATGTAAACGATTGCCACCCATGACATATCCGAGGGCAGCTCTAGACA
AATGACCTGGGAGTTCTTCGTCATAAATCATGAATTTTTTTAAGAAACAAATAAAGGCTAGAAACTGACATTGTTATGGTAACCCAACCTGTGTCT
AGTGTCTAGCCGCTCCTAGTAAGTTATAGTCTATGTCTACGGGTAGACATTAAGTAATTACTAGGAGCGGCAAGACAAAAAAATTTAACCGAAG
GGAACGAGCCCCCCAGCAGACTGTTGTTTGTTCCTAACCGAAACAAAAATCAACAGTTCTTTTCTTCAAATTGATGCCACCTCGTCAATATCCAAA
AGCTAAATACTGGTTATTAACAATCCCACACGCTGACTATGTTACCTTTCAACCCAGATCCGTGGCATACATTCGAGGCCAACTTGAACGAGGTAT
TTTATTATATATTTGTTTTTTTTACTTATACTATTAGGTAACGCCAACGGATATCTCCACTGGCAGCTTCTGGCGGTCTTCGCCGACCAACAACGCC
TGGCCGCCGTTAAACGAATCTTTGGAGATTCCTGCCACGCCGAGCCCAGTCGCTCAGACGCAGCGGATGAGTATGTTTGGAAAGACGACACTGC
CGTCGCAGGTACCAGGTTCGAGCTGGGTCAACGGCCAGTTAAACGAAATTCGGCTGAAGACTGGGCCGCCATCAAGGATCACGCTAAACGTGG
AAGGCTCGATCTTGTACCGGACGATATATTCGTTAAGAATTATCGGACCCTCAGAACGATTGCTGCTGACTATGCTGAGCCTGTTGCAATCGAAC

GACAGGTTAAAGTTTACTGGGGTCCAACTGGAGTCGGGAAGTCTCGACGAGCATGGGCAGAAGCTGGTCTCGATGCGTATCCAAAATGTCCTCT CAGTAAATTCTGGGACGGATACCGAGCTCATGCACACGTTGTCATTGATGAGTACCGTGGAGGTATTAACATCAGTCACATCCTCCGATGGTTCG ACCGATACCCTGTTTGTGTGGACATTAAGGGATCGTCAACAGTCCTTTCTGCAACGCATATTTGGGTCACTTCCAACTTGCATCCAAGAGACTGGT ACGCTGATTTAGATGAAGGAACTAAAGCAGCACTTATGAGACGTTTGGAAATCA

>000144F|arrow

GGTTATTGTTTCTATTAAGGATACTGCTGCAGATGCTTTTGGTCGTCCAGCTTATGTTGCATCTGAAGGTGTTGCAGTACGTCAGTTTCAGGATGA AGTCAATCGAGCTAGCGAAGATAATCAGTTGTATAAACATCCTGATGATTTTCATATGTTCTATTTGGGTCTTTTTGACGATGCCACTGGTGTTTTT GAACTACTGGAAAGCCCTAAGTTGATTGCTCGTGCAAAAGATGTAATGATTCGCGAAGGCGAGTAAGGTTTTTTTTATACCGTATCACTCGAAAG AGTGGTACGGAACTACGGGAGATGTTTATGTTTCGCAATAAGTCAGTAAGTACGCATTCATTTGCTATGGTTCCTAAAGCGGACATTCCCCGCTC TAGTTTTAATACTCAATATGCTCATAAAACCACGTTTGATGCTGGTTTTTTAGTTCCTATTTATTGTGATGAAGTATTGCCTGGCGATACTCATCGT GTAAAGATGACTGCATTTGCACGTTTGGCCACACCGTTATTTCCTGTGATGGACAACTTGCATCTTGATACTTTCTTTTTCTTTGTACCTAATCGTTT ACTTTGGAACAATTGGCCAAAGTTTATGGGTGAACAAACGAATCCTGGTGATTCTATTTCTTTTGTAGTGCCTACTATTACTAGTCCTGCTGGTGG TTATGCTGTTTGTTCAATTTTTGATTATTTTGGTTTACCTACTGCTGGTCAGATTACTGGCGCTAATACAGTAACGCATAATGTTTTGCCGTTACGT GCTTATAATGAGATTTATAACGAATGGTTTAGAGATGAAAACTTACAGAATTCTGTAACGTTAAATCTTGGTGATTCAGGTGATGTTCCTGCTAAC TATACACTTTTGAGACGTGGTAAGCGTAAAGATTATTTTACTGGTGCATTGCCTTGGCCACAGAAGGGTGCTTCTGTTTCTTTACCGTTAGGAACA CGTGCTAATATTTATTCTGACATACCAGCTGGCAATGGTACTGCTGGTTATAGTGTTTTTCAAACTGCTGTTGGTGCTTTAAGAGAATTAAATTCA GCTTCTAATACTTTGTCTAATAGTACAAATGCTGGTGTTGCTACTAATCAGTTATACGCTGATTTGTCTACTGCTACTGCTGCGACTATTAACCAAC TTCGTCAATCTTTCCAGATTCAGAAGTTATTGGAGCGCGATGCACGTGGTGGTACTCGTTATACTGAGTTACTACGTGCTCACTTTGGAGTAACTC CACAGGATTATCGTTTACAACGTCCTGAATATATTGGTGGAGGTTCGACCCTTGTTAATGTTAATCCGATTGCTCAGACTTCTGCAACGTCGGTTA CTGGTTCTGCTACTCCGCAAGGTAACCTTGCTGCAATGGGTACTGCATTGGCTCAGGGACACGGCTTTACGTATGCTGCTCAAGAACATGGATAC ATTATCGGATTAGTTTCTGTACGTGCTGACCTCACATATCAACAGGGTCTTCCTAAGATGTGGTCTAGGTCTACACGATATGACTTTTATTTCCCA GTATTTGCCACTTTGGGTGAGCAAGCTATTTTGAACAAAGAAATTTATGTTCAAGGTACTGCAGCCGACAATGATGTATTTGGTTATCAAGAACG TTGGGCGGAGTATCGTTACAAACCTTCTCAAATTACTGGTTTCTTTAGGTCTACTTCTGCTGGCACTATTGATGCTTGGCATTATGGACAGCGATT TACTTCTCTTCCTACGTTGAATTCAACGTTTATTCAAGAGACCCCTCCAGTTGCTCGTACTACGGCGGTCGGAGCTGCAGCAAATGGTCAGCAATT TTTAATGGATGCTTTCTTTGATTGTCAGATGGCCAGACCTATGCCTATGTACAGCGTACCTGGTCTAATTGATCATTTCTAATGTTTTATATAACCT CGACTACTCCGTAAGGAGTAGTGAGGAAACAACCGAAGGGCGTTAGTTTATGTTTGGTGGAATACTTGATGCGGTTACTAATGTTGGTTCTAAG CTGTCTTCAGCTTCTAGTTTCTTTACTCCTGGTGTCGGTACTGCTTTGGGCGCTGTTGGTTCTTATTTAGGTTCTACTTCTGCTAATAAAGCTAATCA GGAGATGGCTCAGAGGCAAATGGATTTTCAAGCCGATATGAGTGGAACAAGTTACCAGCGTGCTGTTAAAGATTTAGAAGCTGCTGGTTTATCT CCTATGTTAGCCTATCAACGTGGTGGTGCTTCTACCCCATCTGGTTCAACTGCTACTATGGAAAATGTTTTAGGTAATGCAACTAATTCAGCTATT AATACTGCTTCTATGATGCAACAGATTCGTAATGCATCAGAAACAGAAAAGCAGATTATCGCCCAGACTGAAGCTACTGAAGCTGGTACCGCTA ATACTAGGGCTGATACTGTTAATAAGTTGCTTACTGCTCCTAATATTACAGCCGAAAATAAACGTATTTTGGCTGATATTGCTTTAAAGAATACGA CTGCGGATTTAACATCCGCTCAGTCATATAATACTAAGAGGCTATTGGCTCCATCCCCAGCTATTTGGTCTAGGGGTATCGATGCTTCGAAAGAA ATTTTTGATAAACTCAAAAATAATCCTAATCAACTAACCCCTTGGGGAATTGGAGTCAAATAATGAGTAAAGCGAATTTGCCATTTGTACGTAATC CGTACAACTATGATAAAGATGAAGCATCGGTAAACGATGCGTTGCTGTGTCAAGACCCAAGTCTTGCTCAACAGCATATGAAAGATGAATGTGA CATTAATGTCATCATTGAACGTTTCGGGGTTACAGGGGAACTTCCAACGGCCCCTGTATCGCCTCAATACGGCGATTTTAGTGGTGTTACTGATT ACCATTCTGCGTTGAATCAAATTAACGCAACTATGGACGATTTCATGGCTCTGCCAGCGAAATTAAGAGTCCGATTTGACCATGATCCTGTCAAAT TATTGGAGTTCCTTGAGAACGACCAGAATCGTGATGAAGCGATTCAATTGGGTCTTATTGATGGACAACCTGTGGTTGAACCCATCGTTTCTACA GAAACACCTAAGGCCGAAGGATGAAATCCTGAGGCCAGCACAGTTACTCTACTTGATGTAACTGTGCTAGGTGACACCAAACCACTATTTTAACT ACGGAGTTCATCATGTTACGAAGAAAGCCAGTAAACAAATATAAATCTGCAAAGTCATTTCGCAGAACTGCTAGTAAGACGAAGTCAATTAATAT GAGACACGCTCCCCAGCGTGGTGGCTATCGTTTGTAATTATGGCCTGTTATAAGCCCTTAACGGCTTATCAATGCAGTGACAGGTCTATAATTTG GCGGGAAATACCGGGTGCGGATGTAGTCCGTACCCTATCATTGCCTTGTGGTCAGTGTGTTGGTTGTCGCCTTGAACGCTCACGTCAGTGGGCG ATTCGTTGTATGCATGAGGCACAAATGCATACTAGTAATTGTTTTATTACTTTGACATATGCTCCAGAGCATTGTCCTAAGGATATGTCATTGGAT TACAATGATTATCAGCTTTTTATGAAGCGGTTACGTAAGCGTTTTACTGGGAAAACGATACGTTTTTATATGGCAGGTGAATATGGTGAATCTTTT GATCGTCCTCATTTCCATGCTTGTCTGTTTGGTCTTGATTTTCCGGATAAGAAAATATTTAAAAGAACGCAGACTGGCTCTATCCTCTACACGTCAG AGATTTTGGAAGAATTGTGGCCGTTTGGCTATTCTACAATTGGTGATGTTACTTTTGAGTCTGCTGCTTATGTTGCAAGATATATTATGAAGAAGA TTAATGGGGTTACTGTCAATGAAAACCACGAAGTGGTTGATGCGGGTGCCCATTATCAATATTGTGATTTAGAGACTGGTGAGATAATTCAGCG TAAGCCAGAATTTAATAAGATGTCATTGAAGCCCGGTATCGGGCAATCGTGGTTAGATAAGTACATGTCAGACGTTTATACGTCAGACCACGTTG TGGTGCGTGGCAAAAAGTGCCGTCCACCACGGTTTTATGATAATAAATTTAAGTTGAAGTTTCCTGAAGAATTCGATATGATTCAGTTTGCCAGA GAGATGGAAGGTCGATCTAGGCATGAGGACAACACGCTTGAGCGACTTGCTGTTAAGGAAAAAGTTGCGTTGGCTAAGTTGTCATTGTTAAAAC GTACTATTTAAGGAGTTTTTATGAAGAT

>000170F|arrow

GACAATATAAGATATTGAGTCTGTTGGATTTTCTTGTTGACCCATAAATTTTTGCCAGTTATTCCATATAAGGCGATTGGGTACAAAGAAAAAGA
AAGAATCCATAATCATGTTATCCATGATTGGATATAAAGGCGTTGCTAGACGGGCAAATGCCGTCATTTTTAAGTTGAAAGTGTCCCCAGGGAGC
ACTTCATCAACATATACAGGAATTAGATAGCCCGCATCGAAAGTCGTTTTATGTGTTTTTTGAGCATCGAATTTACTACGTGGTATATCGGCTCTA
GGTACCATCGCGAAGCGGTGAGTATTAACTGACTGATTGCGGTGCATGTTTTTCCTTAGTGTTGTTCCGGGGGAAAGATAAATCTCTTTTCCCCTC
GGTTGTTTTATTTAAGTTTAACTTGTTTTCCTAATGATAGGAGTTTTGGTTGTTCATGTAAATCGAATAACCCAGTTGAATCGTCAAATGTTCCGAA
TTCATATAGATCGAAATCATCAGGGTGATTAAAGAGTTGATTTTCAGTATCAGAACGATTAATTTCATCTGAAAAAGAGCGTATAGCTACTCCAG
AGGAAGGTACGAACATTGGTCGTGCATATGCTTCAGCAGCACGGTCTTTTACGGAAGCGAGGATAAGTTTCATTATTTTCCTAAGTAAGGTTACG
TTTTAATAGTTGAAGTTTTGCCATAGTGACTTGTTCTTTTGCAGATAGTCGTTCTGGTGTATTGTCTTCGGAATTAAATTTAGCATTATTTTCCCGCA
TGTAAAGTAATTCGTCATACTCATAAGGTTGGTCAATTTTAAACATTTTGTCATAGTATTTTGGTGGTTTGACCTTTTTACCTCTAAGTATTACGTA
GTCTTGCGGGTATATATCCGAAGTATATTTTATATAAAAGTCTTTACCGATTCCCGGTTTTAAAGACATTTTATTATATTCCGGCTTTAAGTCTAAA
TATTCGCCGGTTTCAGGGTGTATGCGTTTGTAATGAGATTCCGCATCTTTCCCTGTTTGTTTTTTCATTATGTATCTAGCCACGTAGGCGGCTGATT
CAAAAGTAACATCTCCAATGGTGGTATAACCAAATGGCCAGAGAGCTTCAAGTTCTGCGGATCTATATAACATAGAACCAGAGGCAGTCCTTTTC
CATAATTTTTTATCAGGAAAGTCGTATCCGAAGATACAGGCATGGAAGTGGGGTCTTGCGAAGAGTTCACCATATTCTCCAGCCATGTAATAGCG
GATTGTAAGTCCTCTTTTTGCGAGAGTTTTTCTAAGTCTTTTAAGGAACAATTGAAAGTCTTTGTGATCCAAAGAGCCATCGCTTGGGAGATGTGT
ATTGTCATATGTGAGTGTTATGAATGAGTTGTTTTTATGCAATTGGGCTTCGTGCATGCACCGAATAGCCCATTGTCTTGATCGTTCTAGACGGCA
TCCAACGCACTGCCCACAGGGCAGGTCTAAAGATCGAACGATGTCATGTTTCCGAACTTCGTCGAAAACAATTGATTTGTCAAAGCATTGATATG
CTTTGAGAGGGTGATAACAAGGCATGTGAGGTGCCTGAAGACTTTATTAGAGTCTCCAGCCTCCACGCATTGGCGAGTTTCGCATATTTGCGTGT
TTTGTATGTGAAGCATGTTTACGGAAAGTCCTAGCGGACTTTTGCTTATTTGCTGGTCTGCGACGCATCATTTTTTCTGTCCTTGTTTATCGTGTTT
TTGTGGTTTGGTGTCACCTAGCACAGTTACATCAAGTAGGTAACTGTGCTTCCGAGGTTCCAACAGGGGTTGAAACCTCGGTTTGGGCTGGTTTT
ACCAGTCCCATTTTTTCAGCTTCGCTGCGATTTTCAGGGTTTTCTAGAAAATCTATTAGATTCGCGGGATCATTAGCGAATCGTTCACGAATATTG
GCTGGTAAAGCCATAAATTCGTTGTCTGAAGCCATAATCTGGTTCAGAGCAGAGTGGTAGTCATAGACACCACTAAAGTCGCCATATTGAGGCG
TTAAAGGAGTTTGAGGAATAAGTCCGGTCATACCGAACTTTTCCATAATATTATTGATATCACATTCTTCAGCAAATTGCTGCTGAGTCAGAGTTG
CATCCTCACAATGCAGCCCTGACTCATTTGACGCAGCAATTTGCTGAAGAATGTGATATCAATAATATTATGGAAAAGTTCGGTATGACCGGACT
TATTCCTCAAACTCCTTTAACGCCTCAATATGGCGACTTTAGTGGTGTCTATGACTACCACTCTGCTCTGAACCAGATTATGGCTTCAGACAACGA
ATTTATGGCTTTACCAGCCAATATTCGTGAACGATTCGCTAATGATCCCGCGAATCTAATAGATTTTCTAGAAAACCCTGAAAATCGCAGCGAAG
CTGAAAAAATGGGACTGGTAAAACCAGCCCAAACCGAGGTTTCAACCCCTGTTGGAACCTCGGAAGCACAGTTACCTACTTGATGTAACTGTGC
TAGGTGACACCAAACCACAAAAACACGATAAACAAGGACAGAAAAAATGATGCGTCGCAGACCAGCAAATAAGCAAAAGTCCGCTAGGACTTT
CCGTAAACATGCTTCACATACAAAACACGCAAATATGCGAAACTCGCCAATGCGTGGAGGCTGGAGACTCTAATAAAGTCTTCAGGCACCTCAC
ATGCCTTGTTATCACCCTCTCAAAGCATATCAATGCTTTGACAAATCAATTGTTTTCGACGAAGTTCGGAAACATGACATCGTTCGATCTTTAGACC
TGCCCTGTGGGCAGTGCGTTGGATGCCGTCTAGAACGATCAAGACAATGGGCTATTCGGTGCATGCACGAAGCCCAATTGCATAAAAACAACTC
ATTCATAACACTCACATATGACAATACACATCTCCCAAGCGATGGCTCTTTGGATCACAAAGACTTTCAATTGTTCCTTAAAAGACTTAGAAAAAC
TCTCGCAAAAAGAGGACTTACAATCCGCTATTACATGGCTGGAGAATATGGTGAACTCTTCGCAAGACCCCACTTCCATGCCTGTATCTTCGGAT
ACGACTTTCCTGATAAAAAATTATGGAAAAGGACTGCCTCTGGTTCTATGTTATATAGATCCGCAGAACTTGAAGCTCTCTGGCCATTTGGTTATA
CCACCATTGGAGATGTTACTTTTGAATCAGCCGCCTACGTGGCTAGATACATAATGAAAAAACAAACAGGGAAAGATGCGGAATCTCATTACAA
ACGCATACACCCTGAAACCGGCGAATATTTAGACTTAAAGCCGGAATATAATAAAATGTCTTTAAAACCGGGAATCGGTAAAGACTTTTATATAA
AATATACTTCGGATATATACCCGCAAGACTACGTAATACTTAGAGGTAAAAAGGTCAAACCACCAAAATACTATGACAAAATGTTTAAAATTGAC
CAACCTTATGAGTATGACGAATTACTTTACATGCGGGAAAATAATGCTAAATTTAATTCCGAAGACAATACACCAGAACGACTATCTGCAAAAGA
ACAAGTCACTATGGCAAAACTTCAACTATTAAAACGTAACCTTACTTAGGAAAATAATGAAACTTATCCTCGCTTCCGTAAAAGACCGTGCTGCTG
AAGCATATGCACGACCAATGTTCGTACCTTCCTCTGGAGTAGCTATACGCTCTTTTTCAGATGAAATTAATCGTTCTGATACTGAAAATCAACTCTT
TAATCACCCTGATGATTTCGATCTATATGAATTCGGAACATTTGACGATTCAACTGGGTTATTCGATTTACATGAACAACCAAAACTCCTATCATTA
GGAAAACAAGTTAAACTTAAATAAAACAACCGAGGGGAAAAGAGATTTATCTTTCCCCCGGAACAACACTAAGGAAAAACATGCACCGCAATCA
GTCAGTTAATACTCACCGCTTCGCGATGGTACCTAGAGCCGATATACCACGTAGTAAATTCGATGCTCAAAAAACACATAAAACGACTTTCGATG
CGGGCTATCTAATTCCTGTATATGTTGATGAAGTGCTCCCTGGGGACACTTTCAACTTAAAAATGACGGCATTTGCCCGTCTAGCAACGCCTTTAT
ATCCAATCATGGATAACATGATTATGGATTCTTTCTTTTTCTTTGTACCCAATCGCCTTATATGGAATAACTGGCAAAAATTTATGGGTCAACAAGA
AAATCCAACAGACTCAATATCT

>000083F|arrow

TGAAGCATGTTTACGGAAAGTCCTAGCGGACTTTTGCTTATTTGCTGGTCTGCGACGCATCATTTTTTCTGTCCTTGTTTATCGTGTTTTTGTGGTT
GGTGTCACCTAGCACAGTTACATCAAGTAGGTAACTGTGCTTCCGAGGTTCCAACAGGGGTTGAAACCTCGGTTTGGGCTGGTTTTACCAGTCCC
ATTTTTTCAGCTTCGCTGCGATTTTCAGGGTTTTCTAGAAAATCTATTAGATTCGCGGGATCATTAGCGAATCGTTCACGAATATTGGCTGGTAAA
GCCATAAATTCGTTGTCTGAAGCCATAATCTGGTTCAGAGCAGAGTGGTAGTCATAGACACCACTAAAGTCGCCATATTGAGGCGTTAAAGGAG
TTTGAGGAATAAGTCCGGTCATACCGAACTTTTCCATAATATTATTGATATCACATTCTTCAGCAAATTGCTGCTGAGTCAGAGTTGCATCCTCAC

AATGCAGCCCTGACTCATTTGACGCAGCAATCGTGTCGTAATTGTACGGAGTACGAAGAAATGGGGCTGTAATCTTTGTCATTTTCATTCCATTG
GTGGTTGGTTATTTGTACGATTTTGTTGAATCGGTTGACCTCGATAGTTATCGATGTATCGTTTTGCGCCAGATGCGCTATACGCATCTTTGGCGA
TTTTTTACGTCTTGATAAATTGGTTTGCTAGAACTAGGAGCTGACCCTGATTTAGCAAGTTTTGTTAACTCGTTTGAATAACGAGATTGTGCCAAA
GCTTGAGCAGCTTGAGCACTTGATGTTTTGCCTATTTCATTTTTCATGAAAGTATCGGCAAGTATTTGTTTATATTGAGCTCTAATATTTGGATTTT
CATCCAATTTGTTTAACGTATCAGCACGTACATTATCTGTTTGATTACTTGTGAGTTCTGTTTGAGCTTCAATTTGTTTAGATTGAGCAATAGCTTG
ATTTGCTTGCGCAACCGTTTGATAAGCTTGGGTTCCGGACGTAGTTGCATTACCGAGAACATTTTGCATCTGGGCCATAGCTCCAGCTGGTGTTG
TGGCTCCGCCTTGTGAATACGCAAGCATGGGATTTAACCCAGCTTTTTTCATATCTTCAACTGCTCGTTGATATGATGTTCCAGACATTTCAGCTTG
AAATCTCTGTTGATTACTCGCTTGTTCTGCACTCGCTGCGTTTTGACTTTGTGTTCCAAAGTATTGTCCTGCTGCACCTATTGCTGCAGGTGCAAGT
GCAGCTAAGGAGAGTCCCCCAGTAGCAGGGGCAGCTCCTATAGCTATAGCAGGGCCGATTAAATCGGCAATTCCGTCAAATAGTCCCATTAGAA
ATGGTCTATTAAGCCAGGTACTGAATACATTGGCATTGGTCTTGCCATTTTGACATCAAAAAATGAGTCAAATAAGAATTGTTGGCCATTTGCAG
CTGCTCCAACGGCTACTACACGTGATACAGGAGGCGTATCTTGAATAAACGTATTATTCAAAGTAGGCGCAGCGGTAAATTTTTGAGCCAAATGC
CAACCATCGATCGTGCCCGCCGATGTTGATTTGAACAAACCAGTAATTTTAGATGGTTTGTAACGATATTCCGCCCAGCGTTCTTGATATCCAAAA
ACAGTATTGTCGGCTGCAGTATCTCCTGTTGCATAAATTTCTTTTTGCAAAACAGATTGTTCGCCTAGAGTAGCAAATGCTGGGAAATAAAAGTC
ATATCGTGTAGATCTAGACCACATACGGTCTAATCCTTGTTGATAAGTAAGATCTGCTCTAATGGATACTAATCCAATAATAACGCCATGCTCAGT
AAATGATTGAGTAAATCCATGATTATGAGCGAGAGCAGTACCCATAGCAGCAAGGTTGCCCAAAGGGGTAGTCGTTCCAGAAGCGTTTGTTCCC
GACGTTTGAGCAATCGGATTAACATTAATTGGTGTTGAACCGCCTCCAAGGTATTCAGGCCTTTGTAAACGGGCGTCTGGGGAAATAACTCCAA
AGTGACTCCGGATAATTTCTGTGTATCGTGTTCCGCCACGTGCATCCCTTTCAAGTAATTTTGAATTTGAAATGACTGTCTTAATTGATTGACAGTT
GCAGCAGTTGCTTCTGATAAGTCGGTATATAAATTAGAAACGTTATTTACTACACCAGCAGTATTAACACCATAAGCGTTGCCATATCTAGCTAAA
GCAGTCGTATTTCCAGGATCTGTTTGAATTACCGTAAATTTATCGTTTGATGTTGCGTCTCCTGAAATGGTATCCCATTTAATTGGAGCCGTAGTA
CCTAAAGGTAAGGTGACACTCGCACCTTTTTGTGGCCATGGTAATGCTGACGTAAAGTAATCATGTCTTTTACCACGACGTTTTAGCACATAGTTT
GAGGAAGTATCAGGGCCATCGCCCTTATCAACTACTGCGCTTGTTTGTAAATTTTCATCTCGGAACCATTCGTTCCAATAAGATTGTATGCACGTG
GCCAAAAGGCACAGTGCGTAATAGTTCGGCCAGTATCAATTTGGCCTACTGTTGGTAAGCCCATATAGTCTTGAAGGCTGCCTACGGCATAACCA
TCTGTTGGGCTTGTTTGTGTTGGGACAATATAAGATATTGAGTCTGTTGGATTTTCTTGTTGACCCATAAATTTTTGCCAGTTATTCCATATAAGGC
GATTGGGTACAAAGAAAAAGAAAGAATCCATAATCATGTTATCCATGATTGGATATAAAGGCGTTGCTAGACGGGCAAATGCCGTCATTTTTAA
GTTGAAAGTGTCCCCAGGGAGCACTTCATCAACATATACAGGAATTAGATAGCCCGCATCGAAAGTCGTTTTATGTGTTTTTGAGCATCGAATTT
ACTACGTGGTATATCGGCTCTAGGTACCATCGCGAAGCGGTGAGTATTAACTGACTGATTGCGGTGCATGTTTTTCCTTAGTGTTGTTCCGGGGG
AAAGATAAATCTCTTTTCCCCTCGGTTGTTTATTTAAGTTTAACTTGTTTTCCTAATGATAGGAGTTTTGGTTGTTCATGTAAATCGAATAACCCAG
TTGAATCGTCAAAGTTCCGAATTCATATAGATCGAATCATCAGGGTGATTAAAGAGTTGATTTTCAGTATCAGAACGATTAATTTCATCTGAAAAA
GAGCGTATAGCTACTCCAGAGGAAGGTACGAACATTGGTCGTGCATATGCTTCAGCAGCACGGTCTTTTACGGAAGCGAGGATAAGTTTCATTA
TTTTCCTAAGAGGTTACGTTTTAATAGTTGAAGTTTTGCCATAGTACTTGTTCTTTTGCAGATAGTCGTTCTGGTGTATTGTCTTCGGAATTAATTT
AGCATTATTTTCCCGCATGTAAAGTAATTCGTCATACTCATAAGGTTGGTCAATTTTAAACATTTTGTCATAGTATTTTGGTGGTTTGACCTTTTTAC
CTCTAAGTATTACGTAGTCTTGCGGGTATATATCCGAAGTATATTTTATTAAAAGTCTTTACCGATTCCCGGTTTTAAAGACATTTTATTATATTCC
GGCTTTAAGTCTAAATATTCGCCGGTTTCAGGGTGTATGCGTTTGTAATGAGATTCCGCATCTTTCCCTGTTTGTTTTTTCATTATGTATCTAGCCA
CGTAGGCGGCTGATTCAAAAGTAACATCTCCAATGGTGGTATAACCAAATGGCCAGAGAGCTTCAAGTTCTGCGGATCTATATAACATAGAACC
AGAGGCAGTCCTTTTCCATAATTTTTTATCAGGAAAGTCGTATCCGAAGATACAGGCATGGAAGTGGGGTCTTGCGAAGAGTTCACCATATTCTC
CAGCCATGTAATAGCGGATTGTAAGTCCTCTTTTTGCGAGAGTTTTTCTAAGTCTTTTAAGGAACAATTGAAAGTCTTTGTGATCCAAAGAGCCAT
CGCTTGGGAGATGTGTATTGTCATATGTGAGTGTTATGAATGAGTTGTTTTTATGCAATTGGGCTTCGTGCATGCACCGAATAGCCCATTGTCTTG
ATCGTTCTAGACGGCATCCAACGCACTGCCCACAGGGCAGGTCTAAAGATCGAACGATGTCATGTTTCCGAACTTCGTCGAAAACAATTGATTTG
TCAAAGCATTGATATGCTTTGAGAGGGTGATAACAAGGCATGTGAGGTGCCTGAAGACTTTATTAGAGTCTCCAGCCTCCACGCATTGGCGAGT
TTCGCATATTTGCGTGTTTTGTATG

>000006F|arrow

TGTAAACGATAATCCTGTGGAGTTACTCCAAAGTGAGCACGTAGTAACTCAGTATAACGAGTACCACCACGTGCATCGCGCTCCAATAACTTCTG
AATCTGGAAAGATGACGAAGTTGGTTAATAGTCGCAGCAGTAGCAGTAGACAAATCAGCGTATAACTGATTAGTAGCAACACCAGCATTTGTAC
TATTAGACAAAGTATTAGAAGCTGAATTTAATTCTCTTAAAGCACCAACAGCAGTTTGAAAAAACACTATAACCAGCAGTACCATTGCCAGCTGG
TATGTCAGAATAAATATTAGCACGTGTTCCTAACGGTAAAGAAACAGAAGCACCCTTCTGTGGCCAAGGCAATGCACCAGTAAAATAATCTTTAC
GCTTACCACGTCTCAAAAGTGTATAGTTAGCAGGACATCACCTGAATCACCAAGATTTAACGTTACAGAATTCTGTAAGTTTTCATCTCTAAACCA
TTCGTTATAAATTCATTATAAGCACGTAAACGGCAAACATTATGCGTTACTGTATTAGCGCCAGTAATCTGACCAGCAGTAGGTAAACCAAAATA
ATCAAAAATTGAACAAACAGCATAACCACCAGCAGGACTAGTAATAGTAGGCACTACAAAAGAAATAGAATCACCAGGATTCGTTGTTCACCCA
TAAACTTTGGCCAATTGTTCCAAAGTAAACGATTAGGTACAAAGAAAAGAAAGTATCAAGATGCAAGTGTCCATCACAGGAAATAACGGTGTGG
CCAAACGTGCAAATGCAGTCATCTTTACACGATGAGTATCGCCAGGCAATACTTCATCACAATAAATAGGAACTAAAAAACCAGCATAAACGTG
GTTTTATGAGCATATTGAGTATTAAAACTAGAGCGGGGAATGTCCGCTTTAGGAACCATAGCAAATGAATGCGTACTTACTGATTATTGCGAAAC

ATAAACATCTCCCGTAGTTCCGTACCATCTTTCGAGTGATACGGTATAAAAAAAACCTTACTCGCCTTCGCGAATCATTACATCTTTTGCACAATCA
ACTTAGGGCTTTCCAGTAGTTCAAAAACACAGTGGCATCGTCAAAAGACCCAAATAGAACATATGAAAATCATCAGGATGTTTATACAACTGATT
ATCTTCGCTAGCTCGATGACTTCATCCTGAAAATGACGTACTGCAACACCTTCAGATGCAACATAAGCTGGACGACCAAAAGCATCTGCAGCAGT
ATCCTTAATAGAAACAATAACCATCTTCATAAAAACTCTTAAATAGTACGTTTTAACAATGACAACTTAGCCAACGCAACTTTTTCCTTAACAGCAG
TCGCTCAAGCGTGTTGTCCTCATGCCTAGATCGACCTTCCATCTCTCTGGCAAACTGAATCATATCGAATTCTTCAGGAAACTTCAACTTAAATTTT
TATCAAAAACCGTGGTGGACGGCACTTTTTGCCACGCACCACAAACGTGGTCTGACGTATAAACGTCTGACATGTACTTATCTAACCACGATTGC
GATACCGGGCTTCAATGACATCTTATTAATTCTGGCTTACGCTGAATTATCTCACCAGTCTCTAAATCACAATATTGATAATGGGCACCCGCATCA
ACCACTTCGTGGTTTTCATGACAGTAACCCCATTAATCTTCTCATATATATCTTGCAACATAAGCAGCAGACTCAAAAGTAACATCACCAATTGTA
GAATAGCCAAACGGCCACAATTCTTCCAAAATCTCTGACGTGTAGAGGATAGAGCCAGTCTGCGTTCTTTTAAAATTTTCTTATCCGGAAAATCAA
GACCAAACAGACAAGCATGGAAATGAGGACGATCAAAGATCACCATATTCACCTGCCATATAAAAACGTATCGTTTTCCCAGTAAAACGCTTACG
TAACCGCTTCATAAAAAGCTGATAATCATTGTAATCCAATGACTATCCTTAGGACAATGCTCTGGAGCATATGTCAAAGTAATAAAACAATTACTA
GTATGCATTTGTGCCTCATGCATAAACGAATCGCCCACTGACGTGAGCGTTCAAGGCGACAACCAACACACTGACCACAAGGCAATGATAGGGT
ACGGACTACATCCGCACCCGGTATTTCCCGCCAAATTATAGACCTGTCACTGCATTGATAAGCCGTTAAGGGCTTATAACAGGCATAATTACAAA
CGATAGCCACCACGCTGGGGAGCGTGTCTCTATAATTGACTTCGTCTTACTAGCAGTTCTGCGAAATGACTTTGCAGATTTATATTTGTTTACTGG
CTTTTTCGTAACATGATGAACTCCGTAGTTAAAATAGTGGTTTGGTGTCACCTAGCACAGTTACATCAAGTAGAGTAACTGTGCTGGCCTCAGGA
TTTCATCTTCGGCCTTAGGTGTTTCTGTAGAAACGATGGGTTCAACCACAGGTTGTCCATCAATAAGACCAATTGAATCGCTTCATCACGATTCTG
GTCGTTCTCAAGGAACTCCAATAATTTGACAGGATCATGGTCAAATCGGACTCTTAATTTCGCTGGCAGAGCCATGAAACGTCCTAGTTGCGTTA
ATTTGATTCAACGCAGAATGGTAATCAGTAACACACTAAAATCGCCGTATTGAGGCGATACAGGGGCGTTGGAAGTTCCCCTGTAACCCCGAAA
CGTTCAATGATGACATTAATGTCACATTCATCTTTCATATGCTGTTGAGCAAGACTTGGGTTTGACACAGCAACGCATCGTTTACGATGCTTCATC
TTATCATAGTTGTACGGATTACGTACAAATGGCAAATTCGCTTATCATTATTTGACTCCAATTCCCAAGGGGTTAGTTGATTAGGATTATTTTGAG
TTTATCAAAAATTTCTTTCGAAGCATCGATACCCTAGACCAAATAGCTGGGGATGGAGCCAATAGCCTCTTAGTATTATATGACTGAGCGGATGT
TAAATCCGCAGTCGTATTTTTAAAGCAATATCAGCCAAAATACGTTTATTTTCGGCTGTAATATTAGGAGCAGTAAGCAACTTATTACACAGTATC
AGCCCTAGTATTAGCGGTACCAGCTTCAGTAGCTTCAGTCTGGGCGATAATCATTTTCTGTTTCTGATGCATTACGAATCTGTGCATCATAGAAGC
AGTATTAATAGCTGAATTAGTTGCATTACCTAAAACATTTTCCATAGTAGCAGTGAACCAGATGGGGTAGAAGCACCACCACGTTGATAGGCTAA
CATAGGAGATAAACCAGCAGCTTCTAAATCTTTAACAGCACGCTGGTAACTGTTCCACTCATATCGGCTTGAAAATCCATTTGCCTCTGAGCCATA
TCCTGATTAGCTTTATTAGCAGAAGTAACCTAAATAAGAACCAACAGCGCCCAAGCAGTACCGACACCAGGAGTAAAGAAACTAGAAGCTGAAG
ACAGCTTAGAACAACATTAGTAACCGCATCAAGTATTCCACCAAACATAAACTAACGCCCTTCGGTTGTTTCCTCACTATCCTACGGAGTAGTCGA
GGTTATATAAAACATTAGAAATGATCAATTAGACAGGTACGCTGTACATAGGCATAGGTCTGGCCATCTGACAATCAAAGAAAGCATCCATTAA
AAATTGCTGACCATTTGCTGCAGCTCGACCGCCGCAGTACGGCAACTGGAGGGGTCTCTTGAATAAACGTTGAATTCAACGTAGGAAGAGAAGT
AAATCGCTGTCCATAATGCCAAGCATCAATAGTGCCAGCAGAAGTAGACCTAAAGAAAACCAGTAATTGAGAAGGTTTGTAACGATACTCCGCC
CACGTTCTTGATAACCAAATACATCATTGTCGGCTGCAGTACCTTGACATAATTTCTTTGTTCAAAATAGCTTGCTCACCAAAGTGGCAAATACTG
GGAAATAAAAGTCATATCGTGTAGACCTAGACCCACATCTTAGGAAGACCATGTTGATATGTGAGGTCAGCACGTACAGAAACTAATCCGATAA
TGTATCCATGGTTCTTGAGCAGCATACGTAAAGCCGTGTCCCTGAGCCAATGCAGTAACCATTGCAGCAAGGTTACCTTGCGGAGTAGCAGAAA
CCAGTAAACCGACGTTGCAGAAGTCTGAGCAATCGGATTAACCTTAACAAGGGTCGAACCTCCAACCAATATATTCAGGACGT

>000089F|arrow

ACCAACCACCAATGGAATGAAAATGACAAAGATTACAGCCCCATTTCTTCGTACTCCGTACAATTACGACACGATTGCTGCGTCAAATGAGTCAG
GGCTGCATTGTGAGGATGCAACTCTGACTCAGCAGCAATTTGCTGAAGAATGTGATATCAATAATATTATGGAAAAGTTCGGTATGACCGGACT
TATTCCTCAAACTCCTTTAACGCCTCAATATGGCGACTTTAGTGGTGTCTATGACTACCACTCTGCTCTGAACCAGATTATGGCTTCAGACAACGA
ATTTATGGCTTTACCAGCCAATATTCGTGAACGATTCGCTAATGATCCCGCGAATCTAATAGATTTTCTAGAAAACCCTGAAAATCGCAGCGAAG
CTGAAAAAATGGGACTGGTAAAACCAGCCCAAACCGAGGTTTCAACCCCTGTTGGAACCTCGGAAGCACAGTTACCTACTTGATGTAACTGTGC
TAGGTGACACCAAACCACAAAAACACGATAAACAAGGACAGAAAAAATGATGCGTCGCAGACCAGCAAATAAGCAAAAGTCCGCTAGGACTTT
CCGTAAACATGCTTCACATACAAAACACGCAAATATGCGAAACTCGCCAATGCGTGGAGGCTGGAGACTCTAATAAAGTCAGGCACCTCACATG
CCTTGTTATCACCCTCTCAAAGCATCAATGCTTTGACAAATCAATGTTTTCGACGAAGTTCGGAAACATGACATCGTTCGATCTTTAGACCTGCCCT
GTGGGCAGTGCGTTGGATGCCGTCTAGAACGATCAAGACAATGGGCTATTCGGTGCATGCACGAAGCCCAATTGCATAAAAACAACTCATTCAT
AACACTCACATATGACAATACACATCTCCCAAGCGATGGCTCTTTGGATCACAAAGACTTTCAACTGTTCCTTAAAAGACTTAGAAAAACTCTCGC
AAAAAGAGGACTTACAATCCGCTATTACATGGCTGGAGAATATGGTGAACTCTTCGCAAGACCCACTTCCATGCCTGTATCTTCGGATACGACTT
TCCTGATAAAAATTATGGAAAAGGACTGCCTCTGGTTCTATGTTATATAGATCCGCAGAACTTGAAGCTCTCTGGCCATTTGGTTATACCACCATT
GGAGATGTTACTTTGAATCAGCCGCCTACGTGGCTAGATACATAATGAAAAAACAAACAGGGAAAGATGCGGAATCTCATTACAAACGCATACA
CCCTGAAACCGGCGAATATTTAGACTTAAAGCCGGAATATAATAAAATGTCTTTAAAACCGGGAATCGGTAAAGACTTTTATATAAAATATACTT
CGGATATATACCGCAAGACTACGTAATACTTAGAGGTAAAAAGGTCAAACCACCAAAATACTATGACAAAATGTTTAAAATTGACCAACCTTATG
AGTATGACGAATTACTTTACATGCGGGAAAATAATGCTAAATTTAATTCCGAAGACAATACACCAGAACGACTATCTGCAAAAGAACAAGTCACT

ATGGCAAAACTTCAACTATTAAAACGTAACCTTACTTAGGAAAATAATGAAACTTATCCTCGCTTCCGTAAAAGACCGTGCTGCTGAAGCATATG
CACGACCAATGTTCGTACCTTCCTCTGGAGTAGCTATACGCTCTTTTTCAGATGAAATTAATCGTTCTGATACTGAAAATCAACTTTTAATCACCCT
GATGATTCGATCTATATGAATTCGGAACATTTGACGATTCAACTGGGTTATTCGATTTACATGAACAACCAAAACTCCTATCATTAGGAAAACAAG
TTAAACTTAAATAAAACAACCGAGGGGAAAAGAGATTTATCTTTCCCCGGAACAACACTAAGGAAAAACATGCACCGCAATCAGTCAGTTAATA
CTCACCGCTTCGCGATGGTACCTAGAGCCGATATACCACGTAGTAAATTCGATGCTCAAAAAACACATAAAACGACTTTCGATGCGGGCTATCTA
ATTCCTGTATATGTTGATGAAGTGCTCCCTGGGGACACTTTCAACTTAAAAATGACGGCATTTGCCCGTCTAGCAACGCCTTTATATCCAATCATG
GACATGATTATGGATTCTTTCTTTTTCTTTGTACCCAATCGCCTTATATGGAATAACTGGCAAAAATTTATGGGTCAACAAGAAAATCCAACAGAC
TCAATATCTTATATTGTCCCAACACAAACAAGCCCAACAGATGGTTATGCCGTAGGCAGCCTTCAAGACTATATGGGCTTACCAACAGTAGGCCA
AATTGATACTGGCCGAACTATTACGCACTGTGCCTTTTGGCCACGTGCATACAATCTTATTGGAACGAATGGTTCCGAGATGAAAATTTACAAAC
AAGCGCAGTAGTTGATAAGGGCGATGGCCCTGATACTTCCTCAAACTATGTGCTAAAACGTCGTGGTAAAAGACATGATTACTTTACGTCAGCAT
TACCATGGCCACAAAAAGGTGCGAGTGTCACCTTACCTTTAGGTACTACGGCTCCAATTAAATGGGATACCATTTCAGGAGACGCAACATCAAAC
GATAAATTTACGGTAATTCAAACAGATCCTGGAAATACGACTGCTTTAGCTAGATATGGCAACGCTTATGGTGTTAATACTGCTGGTGTAGTAAA
TAACGTTTCTAATTTATATACCGACTTATCAGAAGCAACTGCTGCAACTGTCAATCAATTAAGACAGTCATTTCAAATTCAAAAATTACTTGAAAG
GGATGCACGTGGCGGAACACGATACACAGAAATTATCCGGAGTCACTTTGGAGTTATTTCCCCAGACGCCCGTTTACAAAGGCCTGAATACCTT
GGAGGCGGTTCAACACCAATTAATGTTAATCCGATTGCTCAAACGTCGGGAACAAACGCTTCTGGAACGACTACCCTTTGGGCAACCTTGCTGCT
ATGGGTACTGCTCTCGCTCATAATCATGGATTTACTCAATCATTTACTGAGCATGGCGTTATTATTGGATTAGTATCCATTAGAGCAGATCTTACTT
ATCAACAAGGATTAGACCGTATGTGGTCTAGATCTACACGATATGACTTTTATTTCCCAGCATTTGCTACTCTAGGCGAACAATCTGTTTTGCAAA
AAGAAATTTATGCAACAGGAGATACTGCAGCCGACAATACTGTTTTTGGATATCAAGAACGCTGGGCGGAATATCGTTACAAACCATCTAAAATT
ACTGGTTTGTTCAAATCAACATCGGCGGGCACGATCGATGGTTGGCATTTGGCTCAAAAATTTACCGCTGCGCCTACTTTGAATAATACGTTTATT
CAAGATACGCCTCCTGTATCACGTGTAGTAGCCGTTGGAGCAGCTGCAAATGGCCAACAATTCTTATTTGACTCATTTTTTGATGTCAAAATGGCA
AGACCAATGCCAATGTATTCAGTACCTGGCTTAATAGACCATTTCTAATGGGACTATTTGACGGAATTGCCGATTTAATCGGCCCTGCTATAGCTA
TAGGAGCTGCCCCTGCTACTGGGGGACTCTCCTTAGCTGCACTTGCACCTGCAGCAATAGGTGCAGCAGGACAATACTTTGGAACACAAAGTCA
AAACGCAGCGAGTGCAGAACAAGCGAGTAATCAACAGAGATTTCAAGCTGAAATGTCTGGAACATCATATCAACGAGCAGTTGAAGATATGAA
AAAAGCTGGGTTAAATCCCATGCTTGCGTATTCACAAGGCGGAGCCACAACACCAGCTGGAGCTATGGCCAGATGCAAAATGTTCTCGGTAATG
CAACTACGTCCGGAACCCAAGCTTATCAAACGGTTGCGCAAGCAAATCAAGCTATTGCTCAATCTAAACAAATTGAAGCTCAAACAGAACTCACA
AGTAATCAAACAGATAATGTACGTGCTGATACGTTAAACAAATTGGATGAAAATCCAAATATTAGAGCTCAATATAAAAAATACTTGCCGATACT
TTCATGAAAAATGAAATAGGCAAAACATCAAGTGCTCAAGCTGCTCAAGCTTTGGCACAATCTCGTTATTCAAACGAGTTAACAAAACTTGCTAA
ATCAGGGTCAGCTCCTAGTTCTAGCAAACCAATTTATCAAGACGTAAAAAACATCGCCAAAGATGCGTATAGCGCATCTGGCGCAAAACGATAC
ATCGATAACTATCGAGGTCAACCGATTCAACAAAATCGTACAAATA

>000133F|arrow

AGATCGACCTTCCATCTCTCTGGCAAACTGAATCATATCGAATTCTTCAGGAAACTTCAACTTAAATTTATTATCATAAAACCGTGGTGGACGGCA
CTTTTTGCCACGCACCACAACGTGGTCTGACGTATAAACGTCTGACATGTACTTATCTAACCACGATTGCCCGATACCGGGCTTCAATGACATCTT
ATTAAATTCTGGCTTACGCTGAATTATCTCACCAGTCTATAAATCACAATATTGATAATGGGCACCCGCATCAACAACTTCGTGGTTTTCATTGACA
GTAACCCCATTAATCTTCTTCATAATATATCTTGCAACATAAGCAGCAGACTCAAAAGTAACATCACCAATTGTAGAATAGCCAAACGGCCACAAT
TCTTCCAAAATCTCTGACGTGTAGAGGATAGAGCCAGTCTGCGTTCTTTTAAATATTTTCTTATCCGGAAAATCAAGACCAAACAGACAAGCATG
GAAATGAGGACGATCAAAAGATTCACCATATTCACCTGCCATTATACAAAACGTATCGTTTTCCAGTAAAACGCCTTACGTAACCGCTTCATAAAA
AAGCTGATAATCATTGTAATCCAATGACATATCCTTAGGACAATGCTCTGAAGCATATGTCAAAGTAATAAAACAATACTAGTATGCATTTTGTGC
CTCATGCATACCACCGAATCGCCCACTGACGTGAGCGTGTCAAGGCGACAACCAACCACCACTGACCCACAAGGCAATGATAGGGTACGGACTA
CATCCGCACCGGTATTTCCCGCCAAATTATAGACCTGTCACTGCATTGATAAGCCGTTAAGGGCTTATAACAGGCCATAATTACAAACGATAGCC
ACCACGCTGGGGAGCGTGTCTCATATTAATTGACTTCGTCTTACTAGCAGTTCTGCGAAATGACTTTGCAGATTTATATTTGTTTACTGGCTTTCTT
CGTAACATGATGAACTCCGTAGTTAAAATAGTGGTTTGGTGTCACCTAGCACAGTTACATCAAGTAGAGTAACTGTGCTGGCCTCAGGATTTCAT
CCTTCGGCCTTAGGTGTTTCTGTAGAAACGATGGGTTCAACCACAGGTTGTCCATCAATAAGACCCAATTGAATCGCTTCATCACGATTCTGGTCG
TTCTCAAGGAACTCCAATAATTTGACAGGATCATGGTCAAATCGGACTCTTAATTTCGCTGGCAGAGCCATGAAATCGTCCATAGTTGCGTTAATT
TGATTCAACGCAGAATGGTAATCAGTAACACCACTAAAATCGCCGTATTGAGGCGATACAGGGGCCGTTGGAAGTTCCCCTGTAACCCGAAACG
TTCAATGATGACATTAATGTCACATTCATCTTTCATATGCTGTTGAGCAAGACTTGGGTCTTGACACAGCAACGCATCGTTTACCGATGCTTCATCT
TTATCATAGTTGTACGGATTACGTACAAATGGCAAATTCGCTTTACTCATTATTTGACTCCAATTCCCCAAGGGGTTAGTTGATTAGGATTATTTTT
GAGTTTATCAAAAATTTCTTTCGAAGCATCGATACCCCTAGACCAAATAGCTGGGGATGGAGCCAATAGCCTCTTAGTATTATATGACTGAGCGG
ATGTTAAATCCGCAGTCGTATTCTTTAAAGCAATATCAGCCAAAATACGTTTATTTTCGGCTGTAATATTAGGAGCAGTAAGCAACTTATTAACAG
TATCAGCCCTAGTATTAGCGGTACCAGCTTCAGTAGCTTCAGTCTGGGCGATAATCTGCTTTTCTGTTTCTGATGCATTACGAATCTGTTGCATCAT
AGAAGCAGTATTAATAGCTGAATTAGTTGCATTACCTAAAACATTTTCCATAGTAGCAGTTGAACCAGATGGGGTAGAAGCACCACCACGTTGAT
AGGCTAACATAGGAGATAAACCAGCAGCTTCTAAATCTTTAACAGCACGCTGGTAACTTGTTCCACTCATATCGGCTTGAAAATCCATTTGCCTCT

GAGCCATCTCCTGATTAGCTTTATTAGCAGAAGTAGAACCTAAATAAGAACCAACAGCGCCCAAAGCAGTACCGACACCAGGAGTAAAGAAACT
AGAAGCTGAAGACAGCTTAGAACCAACATTAGTAACCGCATCAAGTATTCCACCAAACATAAACTAACGCCCTTCGGTTGTTTCCTCACTACTCCT
TACGGAGTAGTCGAGGTTATATAAAACATTAGAAATGATCAATTAGACCAGGTACGCTGTACATAGGCATAGGTCTGGCCATCTGACAATCAAA
GAAAGCATCCATTAAAAATTGCTGACCATTTGCTGCAGCTCCGACCGCCGTAGTACGAGCAACTGGAGGGGTCTCTTGAATAAACGTTGAATTCA
ACGTAGGAAGAGAAGTAAATCGCTGTCCATAATGCCAAGCATCAATAGTGCCAGCAGAAGTAGACCTAAAGAAACCAGTAATTTGAGAAGGTT
TGTAACGATACTCCGCCCAAACGTTCTTGATAACCAAATACATCATTGTCGGCTGCAGTACCTTGAACATAAATTTCTTTGTTCAAAATAGCTTGCT
CACCCAAAGTGGCAAATACTGGGAAATAAAAGTCATATCGTGTAGACCTAGACCACATCTTAGGAAGACCCTGTTGATATGTGAGGTCAGCACG
TACAGAAACTAATCCGATAATGTATCCATGTTCTTGAGCAGCATACGTAAAGCCGTGTCCCTGAGCCAATGCAGTACCCATTGCAGCAAGGTTAC
CTTGCGGAGTAGCAGAACCAGTAACCGACGTTGCAGAAGTCTGAGCAATCGGATTAACATTAACAAGGGTCGAACCTCCACCAATATATTCAGG
ACGTTGTAAACGATAATCCTGTGGAGTTACTCCAAAGTGAGCACGTAGTAACTCAGTATAACGAGTACCACCACGTGCATCGCGCTCCAATAACT
TCTGAATCTGGAAAGATTGACGAAGTTGGTTAATAGTCGCAGCAGTAGCAGTAGACAAATCAGCGTATAACTGATTAGTAGCAACACCAGCATT
TGTACTATTAGACAAAGTATTAGAAGCTGAATTTAATTCTCTTAAAGCACCAACAGCAGTTTGAAAAACACTATAACCAGCAGTACCATTGCCAG
CTGGTATGTCAGAATAAATATTAGCACGTGTTCCTAACGGTAAAGAAACAGAAGCACCCTTCTGTGGCCAAGGCAATGCACCAGTAAAATAATCT
TTACGCTTACCACGTCTCAAAAGTGTATAGTTAGCAGGAACATCACCTGAATCACCAAGATTTAACGTTACAGAATTCTGTAAGTTTTCATCTCTA
AACCATTCGTTATAAATCTCATTATAAGCACGTAACGGCAAAACATTATGCGTTACTGTATTAGCGCCAGTAATCTGACCAGCAGTAGGTAAACC
AAAATAATCAAAAATTGAACAAACAGCATAACCACCAGCAGGACTAGTAATAGTAGGCACTACAAAAGAAATAGAATCACCAGGATTCGTTTGT
TCACCCATAAACTTTGGCCAATTGTTCCAAAGTAAACGATTAGGTACAAAGAAAAAGAAAGTATCAAGATGCAAGTTGTCCATCACAGGAAATA
ACGGTGTGGCCAAACGTGCAAATGCAGTCATCTTTACACGATGAGTATCGCCAGGCAATACTTCATCACAATAAATAGGAACTAAAAAACCAGC
ATCAAACGTGGTTTTATGAGCATATTGAGTATTAAAACTAGAGCGGGGAATGTCCGCTTTAGGAACCATAGCAAATGAATGCGTACTTACTGACT
TATTGCGAAACATAAACATCTCCCGTAGTTCCGTACCACTCTTTCGAGTGATACGGTATAAAAAAACCTTACTCGCCTTCGCGAATCATTACATCTT
TTGCACGAGCAATCAACTTAGGGCTTTCCAGTAGTTCAAAAACACCAGTGGCATCGTCAAAAAGACCCAAATAGAACATATGAAAATCATCAGG
ATGTTTATACAACTGATTATCTTCGCTAGCTCGATTGACTTCATCCTGAAACTGACGTACTGCAACACCTTCAGATGCAACATAAGCTGGACGACC
AAAAGCATCTGCAGCAGTATCCTTAATAGAAACAATAACCATCTTCATAAAAACTCCTTAAATAGTACGTTTTAACAATGACAACTTAGCCAACGC
AACTTTTTCCTTAACAGCAAGTCGCTCAAGCGTGTTGTCCTCATGCCT

>000080F|arrow

TGACCTCGATAGTTATCGATGTATCGTTTTGCGCCAGATGCGCTATACGCATCTTTGGCGATGTTTTTACGTCTTGATAAATTGGTTTGCTAGAAC
TAGGAGCTGACCCTGATTTAGCAAGTTTTGTTAACTCGTTTGAATAACGAGATTGTGCCAAAGCTTGAGCAGCTTGAGCACTTGATGTTTTGCCT
ATTTCATTTTTCATGAAAGTATCGGCAAGTATTTGTTTATATTGAGCTCTAATATTTGGATTTTCATCCAATTTGTTTAACGTATCAGCACGTACATT
ATCTGTTTGATTACTTGTGAGTTCTGTTTGAGCTTCAATTTGTTTAGATTGAGCAATAGCTTGATTTGCTTGAGCAACCGTTTGATAAGCTTGGGTT
CCGGACGTAGTTGCATTACCGAGAACATTTTGCATCTGGGCCATAGCTCCAGCTGGTGTTGTGGCTCCGCCTTGTGAATACGCAAGCATGGGATT
TAACCCAGCTTTTTTCATATCTTCAACTGCTCGTTGATATGATGTTCCAGACATTTCAGCTTGAAATCTCTGTTGATTACTCGCTTGTTCTGCACTCG
CTGCGTTTTGACTTTGTGTTCCAAAGTATTGTCCTGCTGCACCTATTGCTGCAGGTGCAAGTGCAGCTAAGGAGAGTCCCCCAGTAGCAGGGGCA
GCTCCTATAGCTATAGCAGGGCCGATTAAATCGGCAATTCCGTCAAATAGTCCCATTAGAAATGGTCTATTAAGCCAGGTACTGAATACATTGGC
ATTGGTCTTGCCATTTTGACATCAAAAAATGAGTCAAATAAGAATTGTTGGCCATTTGCAGCTGCTCCAACGGCTACTACACGTGATACAGGAGG
CGTATCTTGAATAAACGTATTATTCAAAGTAGGCGCAGCGGTAAATTTTTGAGCCAAATGCCAACCATCGATCGTGCCCGCCGATGTTGATTTGA
ACAAACCAGTAATTTTAGATGGTTTGTAACGATATTCCGCCCAGCGTTCTTGATATCCAAAAACAGTATTGTCGGCTGCAGTATCTCCTGTTGCAT
AAATTTCTTTTTGCAAAACAGATTGTTCGCCTAGAGTAGCAAATGCTGGGAAATAAAAGTCATATCGTGTAGATCTAGACCACATACGGTCTAAT
CCTTGTTGATAAGTAAGATCTGCTCTAATGGATACTAATCCAATAATAACGCCATGCTCAGTAAATGATTGAGTAAATCCATGATTATGAGCGAG
AGCAGTACCCATAGCAGCAAGGTTGCCCAAAGGGGTAGTCGTTCCAGAAGCGTTTGTTCCCGACGTTTGAGCAATCGGATTAACATTAATTGGT
GTTGAACCGCCTCCAAGGTATTCAGGCCTTTGTAAACGGGCGTCTGGGGAAATAACTCCAAAGTGACTCCGGATAATTTCTGTGTATCGTGTTCC
GCCACGTGCATCCCTTTCAAGTAATTTTGAATTTGAAATGACTGTCTTAATTGATTGACAGTTGCAGCAGTTGCTTCTGATAAGTCGGTATATAAA
TTAGAAACGTTATTTACTACACCAGCAGTATTAACACCATAAGCGTTGCCATATCTAGCTAAAGCAGTCGTATTTCCAGGATCTGTTTGAATTACC
GTAAATTTATCGTTTGATGTTGCGTCTCCTGAAATGGTATCCCATTTAATTGGAGCCGTAGTACCTAAAGGTAAGGTGACACTCGCACCTTTTTGT
GGCCATGGTAATGCTGACGTAAAGTAATCATGTCTTTTACCACGACGTTTTAGCACATAGTTTGAGGAAGTATCAGGGCCATCGCCCTTATCAAC
TACTGCGCTTGTTTGTAAATTTTCATCTCGGAACCATTCGTTCCAAATAAGATTGTATGCACGTGGCCAAAAGGCACAGTGCGTAATAGTTCGGCC
AGTATCAATTTGGCCTACTGTTGGTAAGCCCATATAGTCTTGAAGGCTGCCTACGGCATAACCATCTGTTGGGCTTGTTTGAGTTGGGACAATAT
AAGATATTGAGTCTGTTGGATTTCTTGTTGACCCATAAATTTTTGCCAGTTATTCCATATAAGGCGATTGGGTACAAAGAAAAAGAAAGAATCCA
TAATCATGTTGTCCATGATTGGATATAAAGGCGTTGCTAGACGGGCAAATGCCGTCATTTTTAAGTTGAAAGTGTCCCCAGGGAGCACTTCATCA
ACATATACAGGAATTAAATAGCCCGCATCGAAAGTCGTTTTATGTGTTTTTGAGCATCGAATTTACTACGTGGTATATCGGCTCTAGGTACCATCG
CGAAGCGGTGAGTATTAACTGACTGATTGCGGTGCATGTTTTTCCTTAGTGTTGTTCCGGGGGAAAGATAAATCTCTTTTCCCCTCGGTTGTTTTA
TTTAAGTTTAACTTGTTTTCCTAATGATAGGAGTTTTGGTTGTTCATGTAAATCGAATAACCCAGTTGAATCGTCAAAAGTTCCGAATTCATATAGA

TCGAAGTCATCAGGGTGATTAAAAAGTTGATTTTCAGTATCAGAACGATTAATTTCATCTGAAAAAGAGCGTATAGCTACTCCAGAGGAAGGTA
CGAACATTGGTCGTGCATATGCTTCAGCAGCACGGTTTTACGGAAGCGAGGATAAGTTTCATTATTTTCCTAAGTGAGGTTACGTTTTAATAGTT
GAAGTTTTGCCATAGTTACTTGTTCTTTTGCAGATAGTCGTTCTGGTGTATTGTCTTCGGAATTAAGTTTAGCGTTATTTTCCCGCATGTAAAGTAA
TTCGTCATACTCATAAGGTTGGTCAATTTTAAACATTTTGTCATAGTATTTGGTGGTTTGACCTTTTTACCTCTAAGTATTACGTAGTCTTGCGGGT
ATATATCCGAAGTATATTTTATATAAAAGTCTTTACCGATTCCCGGTTTTAAAGACATTATATTCCGGCTTTAAGTCTAAATATTCGCCGGTTTCAG
GGTGTATGCGTTTGTAATGAGATTCCGCATCTTTCCCTGTTTGTTTTTTCATTATGTATCTAGCCACGTAGGCGGCTGATTCGAAAGTAACATCTCC
AATGGTGGTATAACCAAATGGCCAGAGAGCTTCAAGTTCTGCGGATCTATATAACATAGAACCAGAGGCAGTCCTTTTCCATAATTTTTATCAGG
AAAGTCGTATCCGAAGATACAGGCATGGAAGTGGGGTCTTGCGAAGAGTTCACCATATTCTCCAGCCATGTAATAGCGGATTGTAAGTCCTCTTT
TTGCGAGAGTTTTCTAAGTCTTTTAAGGAACAGTTGAAAGTCTTTGTGATCCAAAGAGCCATCGCTTGGGAGATGTGTATTGTCATATGTGAGTG
TTATGAATGAGTTGTTTTATGCAATTGGGCTTCGTGCATGCACCGAATAGCCCATTGTCTTGATCGTTCTAGACGGCATCCAACGCACTGCCCACA
GGGCAGGTCTAAAGATCGAACGATGTCATGTTTCCGAACTTCGTCGAAAACAATTGATTTGTCAAAGCATTGAAATGCTTTGAGAGGGTGATAA
CAAGGCATGTGAGGTGCCTGGGGACTTTATTAGAGTCTCCAGCCTCCACGCATTGGCGAGTTTCGCATATTTGCGTGTTTTGTATGTGAAGCATG
TTTACGGAAAGTCCTAGCGGACTTTTGCTTATTTGCTGGTCTGCGACGCATCATTTTTTCTGTCCTTGTTTATGTTTTTGTGGTTTGGTGTCACCTA
GCACAGTTACATCAAGTAGGTAACTGTGCTTCCGAGGTTCCAACAGGGGTTGAAACCTCGGTTTGGGCTGGTTTTACCAGTCCCATTTTTTCAGC
TTCGCTGCGATTTTCAGGGTTTTCTAGAAAATCTATTAGATTCGCGGGATCATTAGCGAATCGTTCACGAATATTGGCTGGTAAAGCCATAAATTC
GTTGTCTGAAGCCATAATCTGGTTCAGAGCAGAGTGGTAGTCATAGACACCACTAAAGTCGCCATATTGAGGCGTTAAAGGAGTTTGAGGAATA
AGTCCGGTCATACCAAACTTTTCCATAATATTATTAATATCGCATTCTTCAGCAAATTGCTGCTGAGTCAGAGTTGCATCCTCACAATGCAGCCCTG
ACTCATTTGACGCAGCAATCGTGTCGTAATTGTACGGAGTACGAAGAAATGGGGCTGTAATCTTTGTCATTTTCATTCCATTGGTGGTTGGTTATT
TGTACGATTTTGTTGAATCGGT

>000121F|arrow

TCAGCTTCTAATACTTTGTCTAATAGTACAAATGCTGGTGTTGCTACTAATCAGTTATACGCTGATTTGTCTACTGCTACTGCTGCGACTATTAACC
AACTTCGTCAATCTTTCCAGATTCAGAAGTTATTGGAGCGCGATGCACGTGGTGGTACTCGTTATACTGAGTTACTACGTGCTCACTTTGGAGTA
ACTCCACAGGATTATCGTTTACAACGTCCTGAATATATTGGTGGAGGTTCGACCCTTGTTAATGTTAATCCGATTGCTCAGACTTCTGCAACGTCG
GTTACTGGTTCTGCTACTCCGCAAGGTAACCTTGCTGCAATGGGTACTGCATTGGCTCAGGGACACGGCTTTACGTATGCTGCTCAAGAACATGG
ATACATTATCGGATTAGTTTCTGTACGTGCTGACCTCACATATCAACAGGGTCTTCCTAAGATGTGGTCTAGGTCTACACGATATGACTTTTATTTC
CCAGTATTTGCCACTTTGGGTGAGCAAGCTATTTTGAACAAAGAAATTTATGTTCAAGGTACTGCAGCCGACAATGATGTATTTGGTTATCAAGA
ACGTTGGGCGGAGTATCGTTACAAACCTTCTCAAATTACTGGTTTCTTTAGGTCTACTTCTGCTGGCACTATTGATGCTTGGCATTATGGACAGCG
ATTTACTTCTCTTCCTACGTTGAATTCAACGTTTATTCAAGAGACCCCTCCAGTTGCTCGTACTACGGCGGTCGGAGCTGCAGCAAATGGTCAGCA
ATTTTTAATGGATGCTTTCTTTGATTGTCAGATGGCCAGACCTATGCCTATGTACAGCGTACCTGGTCTAATTGATCATTTCTAATGTTTTATATAA
CCTCGACTACTCCGTAAGGAGTAGTGAGGAAACAACCGAAGGGCGTTAGTTTATGTTTGGTGGAATACTTGATGCGGTTACTAATGTTGGTTCTA
AGCTGTCTTCAGCTTCTAGTTTCTTTACTCCTGGTGTCGGTACTGCTTTGGGCGCTGTTGGTTCTTATTTAGGTTCTACTTCTGCTAATAAAGCTAA
TCAGGAGATGGCTCAGAGGCAAATGGATTTTCAAGCCGATATGAGTGGAACAAGTTACCAGCGTGCTGTTAAAGATTTAGAAGCTGCTGGTTTA
TCTCCTATGTTAGCCTATCAACGTGGTGGTGCTTCTACCCCATCTGGTTCAACTGCTACTATGGAAAATGTTTTAGGTAATGCAACTAATTCAGCTA
TTAATACTGCTTCTATGATGCAACAGATTCGTAATGCATCAGAAACAGAAAAGCAGATTATCGCCCAGACTGAAGCTACTGAAGCTGGTACCGCT
AATACTAGGGCTGATACTGTTAATAAGTTGCTTACTGCTCCTAATATTACAGCCGAAAATAAACGTATTTTGGCTGATATTGCTTTAAAGAATACG
ACTGCGGATTTAACATCCGCTCAGTCATATAATACTAAGAGGCTATTGGCTCCATCCCCAGCTATTTGGTCTAGGGGTATCGATGCTTCGAAAGA
AATTTTTGATAAACTCAAAAATAATCCTAATCAACTAACCCCTTGGGGAATTGGAGTCAAATAATGAGTAAAGCGAATTTGCCATTTGTACGTAAT
CCGTACAACTATGATAAAGATGAAGCATCGGTAAACGATGCGTTGCTGTGTCAAGACCCAAGTCTTGCTCAACAGCATATGAAAGATGAATGTG
ACATTAATGTCATCATTGAACGTTTCGGGGTTACAGGGGAACTTCCAACGGCCCCTGTATCGCCTCAATACGGCGATTTTAGTGGTGTTACTGAT
TACCATTCTGCGTTGAATCAAATTAACGCAACTATGGACGATTTCATGGCTCTGCCAGCGAAATTAAGAGTCCGATTTGACCATGATCCTGTCAAA
TTATTGGAGTTCCTTGAGAACGACCAGAATCGTGATGAAGCGATTCAATTGGGTCTTATTGATGGACAACCTGTGGTTGAACCCATCGTTTCTAC
AGAAACACCTAAGGCCGAAGGATGAAATCCTGAGGCCAGCACAGTTACTCTACTTGATGTAACTGTGCTAGGTGACACCAAACCACTATTTTAAC
TACGGAGTTCATCATGTTACGAAGAAAGCCAGTAAACAAATATAAATCTGCAAAGTCATTTCGCAGAACTGCTAGTAAGACGAAGTCAATTAATA
TGAGACACGCTCCCCAGCGTGGTGGCTATCGTTTGTAATTATGGCCTGTTATAAGCCCTTAACGGCTTATCAATGCAGTGACAGGTCTATAATTT
GGCGGGAAATACCGGGTGCGGATGTAGTCCGTACCCTATCATTGCCTTGTGGTCAGTGTGTTGGTTGTCGCCTTGAACGCTCACGTCAGTGGGC
GATTCGTTGTATGCATGAGGCACAAATGCATACTAGTAATTGTTTTATTACTTTGACATATGCTCCAGAGCATTGTCCTAAGGATATGTCATTGGA
TTACAATGATTATCAGCTTTTTATGAAGCGGTTACGTAAGCGTTTTACTGGGAAAACGATACGTTTTTATATGGCAGGTGAATATGGTGAATCTTT
TGATCGTCCTCATTTCCATGCTTGTCTGTTTGGTCTTGATTTTCCGGATAAGAAAATATTTAAAAGAACGCAGACTGGCTCTATCCTCTACACGTCA
GAGATTTTGGAAGAATTGTGGCCGTTTGGCTATTCTACAATTGGTGATGTTACTTTTGAGTCTGCTGCTTATGTTGCAAGATATATTATGAAGAAG
ATTAATGGGGTTACTGTCAATGAAAACCACGAAGTGGTTGATGCGGGTGCCCATTATCAATATTGTGATTTAGAGACTGGTGAGATAATTCAGC
GTAAGCCAGAATTTAATAAGATGTCATTGAAGCCCGGTATCGGGCAATCGTGGTTAGATAAGTACATGTCAGACGTTTATACGTCAGACCACGTT

GTGGTGCGTGGCAAAAAGTGCCGTCCACCACGGTTTTATGATAATAAATTTAAGTTGAAGTTTCCTGAAGAATTCGATATGATTCAGTTTGCCAG
AGAGATGGAAGGTCGATCTAGGCATGAGGACAACACGCTTGAGCGACTTGCTGTTAAGGAAAAAGTTGCGTTGGCTAAGTTGTCATTGTTAAA
ACGTACTATTTAAGGAGTTTTTATGAAGATGGTTATTGTTTCTATTAAGGATACTGCTGCAGATGCTTTTGGTCGTCCAGCTTATGTTGCATCTGA
AGGTGTTGCAGTACGTCAGTTTCAGGATGAAGTCAATCGAGCTAGCGAAGATAATCAGTTGTATAAACATCCTGATGATTTTCATATGTTCTATTT
GGGTCTTTTTGACGATGCCACTGGTGTTTTTGAACTACTGGAAAGCCCTAAGTTGATTGCTCGTGCAAAAGATGTAATGATTCGCGAAGGCGAGT
AAGGTTTTTTTATACCGTATCACTCGAAAGAGTGGTACGGAACTACGGGAGATGTTTATGTTTCGCAATAAGTCAGTAAGTACGCATTCATTTG
CTATGGTTCCTAAAGCGGACATTCCCCGCTCTAGTTTTAATACTCAATATGCTCATAAAACCACGTTTGATGCTGGTTTTTTAGTTCCTATTTATTGT
GATGAAGTATTGCCTGGCGATACTCATCGTGTAAAGATGACTGCATTTGCACGTTTGGCCACACCGTTATTTCCTGTGATGGACAACTTGCATCTT
GATACTTTCTTTTTCTTTGTACCTAATCGTTTACTTTGGAACAATTGGCCAAAGTTTATGGGTGAACAAACGAATCCTGGTGATTCTATTTCTTTTGT
AGTGCCTACTATTACTAGTCCTGCTGGTGGTTATGCTGTTTGTTCAATTTTTGATTATTTTGGTTTACCTACTGCTGGTCAGATTACTGGCGCTAAT
ACAGTAACGCATAATGTTTTGCCGTTACGTGCTTATAATGAGATTTATAACGAATGGTTTAGAGATGAAAACTTACAGAATTCTGTAACGTTAAAT
CTTGGTGATTCAGGTGATGTTCCTGCTAACTATACACTTTTGAGACGTGGTAAGCGTAAAGATTATTTTACTGGTGCATTGCCTTGGCCACAGAA
GGGTGCTTCTGTTTCTTTACCGTTAGGAACACGTGCTAATATTTATTCTGACATACCAGCTGGCAATGGTACTGCTGGTTATAGTGTTTTTCAAACT
GCTGTTGGTGCTTTAAGAGAATTAAAT

>000193F|arrow

CAGAAGTAGAACCTAAATAAGAACCAACAGCGCCCAAAGCAGTACCGACACCAGGAGTAAAGAAACTAGAAGCTGAAGACAGCTTAGAACCAA
CATTAGTAACCGCATCAAGTATTCCACCAAACATAAACTAACGCCCTTCGGTTGTTTCCTCACTACTCCTTACGGAGTAGTCGAGGTTATATAAAA
CATTAGAAATGATCAATTAGACCAGGTACGCTGTACATAGGCATAGGTCTGGCCATCTGACAATCAAGAAAGCATCCATTAAAAATTGCTGACCA
TTTGCTGCAGCTCCGACCGCCGTAGTACGAGCAACTGGAGGGGTCTCTTGAATAAACGTTGAATTCAACGTAGGAAGAGAAGTAAATCGCTGTC
CATAATGCCAAGCATCAATAGTGCCAGCAGAAGTAGACCTAAAGAAACCAGTAATTTGAGAAGGTTTGTAACGATACTCCGCCCAACGTTCTTG
ATAACCAAATACATCATTGTCGGCTGCAGTACCTTGAACATAAATTTCTTTGTTCAAAATAGCTTGCTCACCCAAAGTGGCAAATACTGGGAAATA
AAAGTCATATCGTGTAGACCTAGACCACATCTTAGGAAGACCCTGTTGATATGTGAGGTCAGCACGTACAGAAACTAATCCGATAATGTATCCAT
GTTCTTGAGCAGCATACGTAAAGCCGTGTCCCTGAGCCAATGCAGTACCCATTGCAGCAAGGTTACCTTGCGGAGTAGCAGAACCAGTAACCGA
CGTTGCAGAAGTCTGAGCAATCGGATTAACATTAACAAGGGTCGAACCTCCACCAATATATTCAGGACGTTGTAAACGATAATCCTGTGGAGTTA
CTCCAAAGTGAGCACGTAGTAACTCAGTATAACGAGTACCACCACGTGCATCGCGCTCCAATAACTTCTGAATCTGGAAAGATTGACGAAGTTG
GTTAATAGTCGCAGCAGTAGCAGTAGACAAATCAGCGTATAACTGATTAGTAGCAACACCAGCATTTGTACTATTAGACAAAGTATTAGAAGCT
GAATTTAATTCTCTTAAAGCACCAACAGCAGTTTGAAAAAACACTATAACCAGCAGTACCATTGCCAGCTGGTATGTCAGAATAAATATTAGCAC
GTGTTCCTAACGGTAAAGAAACAGAAGCACCCTTCTGTGGCCAAGGCAATGCACCAGTAAAATAATCTTTACGCTTACCACGTCTCAAAAGTGTA
TAGTTAGCAGGAACATCACCTGAATCACCAAGATTTAACGTTACAGAATTCTGTAAGTTTTCATCTCTAAACCATTCGTTATAAATCTCATTATAAG
CACGTAACGGCAAAACATTATGCGTTACTGTATTAGCGCCAGTAATCTGACCAGCAGTAGGTAAACCAAAATAATCAAAATTGAACAAACAGCA
TAACCACCAGCAGGACTAGTAATAGTAGGCACTACAAAAGAAATAGAATCACCAGGATTCGTTTGTTCACCCATAAACTTTGGCCAATTGTTCCA
AAGTAAACGATTAGGTACAAAGAAAAAGAAAGTATCAAGATGCAAGTTGTCCATCACAGGAAATAACGGTGTGGCCAAACGTGCAAATGCAGT
CATCTTTACACGATGAGTATCGCCAGGCAATACTTCATCACAATAAATAGGAACTAAAAAACCAGCATCAAACGTGGTTTTATGAGCATATTGAG
TATTAAAACTAGAGCGGGGAATGTCCGCTTTAGGAACCATAGCAAATGAATGCGTACTTACTGACTTATTGCGAAACATAAACATCTCCCGTAGT
TCCGTACCACTCTTTCGAGTGATACGGTATAAAAAAAACCTTACTCGCCTTCGCGAATCATTACATCTTTTGCACGAGCAATCAACTTAGGGCTTT
CCAGTAGTTCAAAAACACCAGTGGCATCGTCAAAAAGACCCAAATAGAACATATGAAAATCATCAGGATGTTTATACAACTGATTATCTTCGCTA
GCTCGATTGACTTCATCCTGAAACTGACGTACTGCAACACCTTCAGATGCAACATAAGCTGGACGACCAAAAGCATCTGCAGCAGTATCCTTAAT
AGAAACAATAACCATCTTCATAAAAACTCCTTAAATAGTACGTTTTAACAATGACAACTTAGCCAACGCAACTTTTTCCTTAACAGCAAGTCGCTC
AAGCGTGTTGTCCTCATGCCTAGATCGACCTTCCATCTCTCTGGCAAACTGAATCATATCGAATTCTTCAGGAAACTTCAACTTAAATTTATTATCA
TAAAACCGTGGTGGACGGCACTTTTTGCCACGCACCACAACGTGGTCTGACGTATAAACGTCTGACATGTACTTATCTAACCACGATTGCCCGAT
ACCGGGCTTCAATGACATCTTATTAAATTCTGGCTTACGCTGAATTATCTCACCAGTCTCTAAATCACAATATTGATAATGGGCACCCGCATCAAC
CACTTCGTGGTTTTCATTGACAGTAACCCCATTAATCTTCTTCATAATATATCTTGCAACATAAGCAGCAGACTCAAAAGTAACATCACCAATTGTA
GAATAGCCAAACGGCCACAATTCTTCCAAAATCTCTGACGTGTAGAGGATAGAGCCAGTCTGCGTTCTTTTAAATATTTTCTTATCCGGAAAATCA
AGACCAAACAGACAAGCATGGAAATGAGGACGATCAAAAGATTCACCATATTCACCTGCCATATAAAAACGTATCGTTTTCCCAGTAAAACGCTT
ACGTAACCGCTTCATAAAAAGCTGATAATCATTGTAATCCAATGACATATCCTTAGGACAATGCTCTGGAGCATATGTCAAAGTAATAAAACAAT
TACTAGTATGCATTTGTGCCTCATGCATACAACGAATCGCCCACTGACGTGAGCGTTCAAGGCGACAACCAACACACTGACCACAAGGCAATGAT
AGGGTACGGACTACATCCGCACCCGGTATTTCCCGCCAAATTATAGACCTGTCACTGCATTGATAAGCCGTTAAGGGCTTATAACAGGCCATAAT
TACAAACGATAGCCACCACGCTGGGGAGCGTGTCTCATATTAATTGACTTCGTCTTACTAGCAGTTCTGCGAAATGACTTTGCAGATTTATATTTG
TTTACTGGCTTTCTTCGTAACATGATGAACTCCGTAGTTAAAATAGTGGTTTGGTGTCACCTAGCACAGTTACATCAAGTAGAGTAACTGTGCTGG
CCTCAGGATTTCATCCTTCGGCCTTAGGTGTTTCTGTAGAAACGATGGGTTCAACCACAGGTTGTCCATCAATAAGACCCAATTGAATCGCTTCAT
CACGATTCTGGTCGTTCTCAAGGAACTCCAATAATTTGACAGGATCATGGTCAAATCGGACTCTTAATTTCGCTGGCAGAGCCATGAAATCGTCC

ATAGTTGCGTTAATTTGATTCAACGCAGAATGGTAATCAGTAACACCACTAAAATCGCCGTATTGAGGCGATACAGGGGCCGTTGGAAGTTCCC
CTGTAACCCCGAAACGTTCAATGATGACATTAATGTCACATTCATCTTTCATATGCTGTTGAGCAAGACTTGGGTCTTGACACAGCAACGCATCGT
TTACCGATGCTTCATCTTTATCATAGTTGTACGGATTACGTACAAATGGCAAATTCGCTTTACTCATTATTTGACTCCAATTCCCCAAGGGGTTAGT
TGATTAGGATTATTTTTGAGTTTATCAAAAATTTCTTTCGAAGCATCGATACCCCTAGACCAAATAGCTGGGGATGGAGCCAATAGCCTCTTAGTA
TTATATGACTGAGCGGATGTTAAATCCGCAGTCGTATTCTTTAAAGCAATATCAGCCAAAATACGTTTATTTTCGGCTGTAATATTAGGAGCAGTA
AGCAACTTATTAACAGTATCAGCCCTAGTATTAGCGGTACCAGCTTCAGTAGCTTCAGTCTGGGCGATAATCTGCTTTTCTGTTTCTGATGCATTA
CGAATCTGTTGCATCATAGAAGCAGTATTAATAGCTGAATTAGTTGCATTACCTAAAACATTTTCCATAGTAGCAGTTGAACCAGATGGGGTAGA
AGCACCACCACGTTGATAGGCTAACATAGGAGATAAACCAGCAGCTTCTAAATCTTTAACAGCACGCTGGTAACTTGTTCCACTCATATCGGCTT
GAAAATCCATTTGCCTCTGAGCCATCTCCTGATTAGCTTTATTAG

>000097F|arrow

TCAACGTGGTGGTGTTCTTACCCCTATCTGGTTCAACTGCTACTATGGAAAATGTTTTAGGTAATGCAACTAATTCAGCTATTAATACTGCTTCTAT
GATGCAACAGATTCGTAATGCATCAGAAACAGAAAAGCAGATTATCGCCCAGACTGAAGCTACTGAAGCTGGTACCGCTAATACTAGGGCTGAT
ACTGTTAATAAGTTGCTTACTGCTCCTAATATTACAGCCGAAAATAAACGTATTTTGGCTGATATTGCTTTAAAGAATACGACTGCGGATTAACAT
CCGCTCAGTCATATAATACTAAGAGGCTATTGGCTCCATCCCCAGCTATTTGGTCTAGGGGTATCGATGCTTCGAAAGAAATTTTGATAAACTCAA
AAATAATCCTAATCAACTAACCCCTTGGGGAATTGGAGTCAAATAATGAGTAAAGCGAATTTGCCATTTGTACGTAATCCGTACAACTATGATAA
AGATGAAGCATCGGTAAACGATGCGTTGCTGTGTCAAGACCAAGTCTTGCTCAACAGCATATGAAAGATGAATGTGACATTAATGTCATCATTG
AACGTTTCGGGGTTACAGGGGAACTTCCAACGGCCCCTGTATCGCCTCAATACGGCGATTTTAGTGGTGTTACTGATTACCATTCTGCGTTGAAT
CAAATTAACGCAACTATGGACGATTTCATGGCTCTGCCAGCGAAATTAAGAGTCCGATTTGACCATGATCCTGTCAAATTATTGGAGTTCCTTGA
GAACGACCAGAATCGTGATGAAGCGATTCAATTGGGTCTTATTGATGGACAACCTGTGGTTGAACCCATCGTTTCTACAGAAACACTAAGGCCG
AAGGATGAAATCCTGAGGCCAGCACAGTTACTCTACTTGATGTAACTGTGCTAGGTGACACCAAACCACTATTTTAACTACGGAGTTCATCATGT
TACGAAGAAAGCCAGTAAACAAATATAAATCTGCAAAGTCATTTCGCAGAACTGCTAGTAAGACGAAGTCAATTAATATGAGACACGCTCCCAG
CGTGGTGGCTATCGTTTGTAATTATGGCCTGTTATAAGCCCTTAACGGCTTATCAATGCAGTGACAGGTCTATAATTTGGCGGGAAATACCGGGT
GCGGATGTAGTCCGTACCCTATCATTGCCTTGTGGTCAGTGTGTTGGTTGTCGCCTTGAACGCTCACGTCAGTGGGCGATTCGTTGTATGCATGA
GGCACAAATGCATACTAGTAATTGTTTATTACTTTGACATATGCTCCAGAGCATTGTCCTAAGGATATGTCATTGGATTACAATGATTATCAGCTT
TTTATGAAGCGGTTACGTAAGCGTTTTACTGGGAAAACGATACGTTTTTATATGGCAGGTGAATATGGTGAATCTTTTGATCGTCCTCATTTCCAT
GCTTGTCTGTTTGGTCTTGATTTTCCGGATAAGAAAATATTTAAAAGAACGCAGACTGGCTCTATCCTCTACACGTCAGAGATTTGGAAGAATTGT
GGCCGTTTGGCTATTCTACAATTGGTGATGTTACTTTTGAGTCTGCTGCTTATGTTGCAAGATATATTATGAAGAAGATTAATGGGGTTACTGTCA
ATGAAAACCACGAAGTGGTTGATGCGGGTGCCCATTATCAATATTGTGATTTAGAGACTGGTGAGATAATTCAGCGTAAGCCAGAATTTAATAA
GATGTCATTGAAGCCCGGTATCGGGCAATCGTGGTTAGATAAGTACATGTCAGACGTTTATACGTCAGACCACGTTGTGGTGCGTGGCAAAAAG
TGCCGTCCACCACGGTTTTATGATAATAAATTTAAGTTGAAGAGCCAAGAATTCGATATGATTCAGTTTGCCAGAGAGATGGAAGGTCGATCTAG
GCATGAGGACAACACGCTTGAGCGACTTGCTGTAAGGAAAAAGTTGCGTTGGCTAAGTTGTCATTGTTAAAACGTACTATTTAAGGAGTTTTTAT
GAAGATGGTTATTGTTTCTATTAAGGATACTGCTGCAGATGCTTTTGGTCGTCCAGCTTATGTTGCATCTGAAGGTGTTGCAGTACGTCAGTTTCA
GGATGAAGTCAATCGAGCTAGCGAAGATAATCAGTTGTATAAACATCCTGATGATTTTCATATGTTCTATTTGGGTCTTTTTGACGATGCCACTGG
TGTTTTTGAACTACTGGAAAGCCCTAAGTTGATTGCTCGTGCAAAAGATGTAATGATTCGCGAAGGCGAGTAAGGTTTTTTTTATACCGTATCACT
CGAAAGAGTGGTACGGAACTACGGGAGATGTTTATGTTTCGCAATAAGTCAGTAAGTACGCATTCATTTGCTATGGTTCCTAAAGCGGACATTCC
CGCTCTAGTTTTAATACTCAATATGCTCATAAAACCACGTTTGATGCTGGTTTTTTAGTTCCTATTTATTGTGATGAAGTATTGCCTGGCGATACTC
ATCGTGTAAAGATGACTGCATTTGCACGTTTGGCCACACCGTTATTTCCTGTGATGGACAACTTGCATCTTGATACTTCTTTTTCTTTGTACCTAAT
CGTTTACTTTGGAACAATTGGCCAAAGTTTATGGGTGAACAAACGAATCCTGGTGATTCTATTTCTTTTGTAGTGCCTACTATTACTAGTCTGCTG
GTGGTTATGCTGTTTGTTCAATTTTTGATTATTTTGGTTTACCTACTGCTGGTCAGATTACTGGCGCTAATACAGTAACGCATAATGTTTTGCCGTT
ACGTGCTTATAATGAGATTTATAACGAATGGTTTAGAGATGAAAACTTACAGAATTCTGTAACGTTAAATCTTGGTGATTCAGGTGATGTTCCTG
CTAACTATACACTTTTGAGACGTGGTAAGCGTAAAGATTATTTTACTGGTGCATTGCCTTGGCCACAGAAGGGTGCTTCTGTTTCTTTACCGTTAG
GAACACGTGCTAATATTTATTCTGACATACCAGCTGGCAATGGTACTGCTGGTTATAGTGTTTTTCAAACTGCTGTTGGTGCTTTAAGAGAATTAA
ATTCAGCTTCTAATACTTTGTCTAATAGTACAAATGCTGGTGTTGTACTAATCAGTTATACGCTGATTTGTCTACTGCTACTGCTGCGACTATTAAC
CAACTTCGTCAATCTTTCCAGATTCAGAAGTTATTGGAGCGCGATGCACGTGGTGGTACTCGTTATACTGAGTTACTACGTGCTCACTTTGGAGTA
ACTCCACAGGATTATCGTTTACAACGTCCTGAATAATTGGTGGAGGTTCGACCCTTGTTAATGTTAATCCGATTGCTCAGACTTCTGCAACGTCGG
TTACTGGTTCTGCTACTCCGCAAGGTAACCTTGCTGCAATGGGTACTGCATTGGCTCAGGGACACGGCTTTACGTATGCTGCTCAAGAACATGGA
TACATTATCGGATTAGTTTCTGTACGTGCTGACCTCACATATCAACAGGGTCTTCCTAAGATGTGGTCTAGGTCTACACGATATGACTTTTATTTCC
CAGTATTTGCCACTTTGGGTGAGCAAGCTATTTTGAACAAAGAAATTTATGTTCAAGGTACTGCAGCCGACAATGATGTATTTGGTTATCAAGAA
CGTTGGGCGGAGTATCGTTACAAACCTTCTCAAATTACTGGTTTCTTTAGGTCTACTTCTGCTGGCACTATTGATGCTTGGCATTATGGACAGCGA
TTTACTTCTCTTCCTACGTTGAATTCAACGTTTATTCAAGAGACCCTCCAGTTGCTCGTACTACGGCGGTCGGAGCTGCAGCAAATGGTCAGCAAT
TTTTAATGGATGCTTTCTTTGATTGTCAGATGGCCAGACCTATGCCTATGTACAGCGTACCTGGTCTAATTGATCATTTCTAATGTTTTATATAACC

TCGACTACTCCGTAAGGAGTAGTGAGGAAACAACGAAGGGCGTTAGTTTATGTTTGGTGGAATACTTGATGCGGTTACTAATGTTGGTTCTAAG
CTGTCTTCAGCTTCTAGTTTCTTTACTCCTGGTGTCGGTACTGCTTTGGGCGCTGTTGGTTCTTATTTAGGTTCTACTTCTGCTAATAAAGCTAATCA
GGAGATGGCTCAGAGGCAAATGGATTTTCAAGCCGATATGAGTGGAACAAGTTACCAGCGTGCTGTTAAAGATTTAGAAGCTGCTGGTTTATCT
CCTATGTAGCCTA

>000023F|arrow

AGCTGAAATGTCTGGAACATCATATCAACGAGCAGTTGAAGATATGAAAAAAGCTGGGTTAAATCCCATGCTTGCGTATTCACAAGGCGGAGCC
ACAACACCAGCTGGAGCTATGGCCCAGATGCAAAATGTTCTCGGTAATGCAACTACGTCCGGAACCCAAGCTTATCAAACGGTTGCTCAAGCAA
ATCAAGCTATTGCTCAATCTAAACAAATTGAAGCTCAAACAGAACTCACAAGTAATCAAACAGATAATGTACGTGCTGATACGTTAAACAAATTG
GATGAAAATCCAAATATTAGAGCTCAATATAACAAATACTTGCCGATACTTTCATGAAAAATGAAATAGGCAAAACATCAAGTGCTCAAGCTGCT
CAAGCTTTGGCACAATCTCGTTATTCAAACGAGTTAACAAAACTTGCTAAATCAGGGTCAGCTCCTAGTTCTAGCAAACCAATTTATCAAGACGTA
AAAAATATCGCCAAAGATGCGTATAGCGCATCTGGCGCAAAACGATACATCGATAACTATCGAGGTCAACCGATTCAACAAAATCGTACAAATA
ACCAACCACCAATGGAATGAAAATGACAAAGATTACAGCCCCATTTCTTCGTACTCCGTACAATTACGACACGATTGCTGCGTCAAATGAGTCAG
GGCTGCATTGTGAGGATGCAACTCTGACTCAGCAGCAATTTGCTGAAGAATGCGATATTAATAATATTATGGAAAAGTTTGGTATGACCGGACTT
ATTCCTCAAACTCCTTTAACGCCTCAATATGGCGACTTTAGTGGTGTCTATGACTACCACTCTGCTCTGAACCAGATTATGGCTTCAGACAACGAA
TTTATGGCTTTAACCAGCCAATATTCGTGAACGATTCGCTAATGATCCCGCGAATCTAATAGATTTCTAGAAAACCCTGAAAATCGCAGCGAAGC
TGAAAAAATGGGACTGGTAAAACCAGCCCAAACCGAGGTTTCAACCCCTGTTGGAACCTCGGAAAGCACAGTTACCTACTTGATGTAACTGTGC
TAGGTGACACCAAACCACAAAAAACACGATAAACAAGGACAGAAAAAATGATGCGTCCAGACCAGCAAATAAGCAAAAGTCCGCTAGGACTTT
TCCGTAAACATGCTTCACATACAAAACACGCAAATATGCGAAACTCGCCAATGCGTGGAGGCTGGAGACTCTAATAAAGTCCCAGGCACCTCAC
ATGCCTGTTATCACCCTCTCAAAGCATTTCAATGCTTTGACAAATCAATTGTTTTCGACGAAGTTCGGAAACATGACATCGTTCGATCTTTAGACCT
GCCCTGTGGGCAGTGCGTGTGGATGCCGTCTAGAACGATCAAGACAATAGGGCTATTCGGTGCTGCACGAAGGCCCAATTGCATAAAAACAAC
TCATTCATAACACTCACATATGACAATACACATCTCCCAAGCGATGGCTCTTTGGATCACAAAGACTTTCAACTGTTCCTTAAAAGACTTAGAAAA
ACTCTCGCAAAAAGAGGACTTACAATCCGCTATTACATGGCTGGAGAATATGGTGAACTCTTCGCAAGACCCCACTTCCATGCCTGTATCTTCGG
ATACGACTTTCCTGATAAAAAATTATGGAAAAGGACTGCCTCTGGTTCTATGTTATATAGATCCGCAGAACTTGAAGCTCTCTGGCCATTTGGTTA
TACCACCATTGGAGATGTTACTTTCGAATCAGCCGCCTACGTGGCTAGATACATAATGAAAAAACAAACAGGGAAAGATGCGGAATCTCATTAC
AAACGCATACACCCTGAAACCGGCGAATATTTAGACTTAAAGCCGGAATATAATAAAATGTCTTTAAAACCGGGAATCGGTAAAGACTTTTATAT
AAAATATACTTCGGATATATACCCGCAAGACTACGTAATACTTAGAGGTAAAAAGGTCAAACCACCAAAACTATGACAAAATGTTTAAAATTGAC
CAACCTTATGAGTATGACGAATTACTTTACATGCGGGAAAATAACGCTAAACTTAATTCCGAAGACAATACACCAGAACGACTATCTGCAAAAGA
ACAAGTAATGGCAAAACTTCAACTATTAAAACGTAACCTCACTTAGGAAAATAATGAAACTTATCCTCGCTTCCGTAAAAGACCGTGCTGCTGAA
GCATATGCACGACCAATGTTCGTACCTTCCTCTGGAGTAGCTATACGCTCTTTTTCAGATGAAATTAATCGTTCTGATACTGAAAATCAACTTTTAA
TCACCCTGATGACTTCGATCTATATGAATTCGGAACTTTGACGATTCAACTGGGTTATTCGATTTACATGAACAACCAAAACTCCTATCATTAGGA
AAACAAGTTAAACTTAAATAAAACAACCGAGGGGAAAAGAGATTTATCTTTCCCCCGGAACAACACTAAGGAAAAACATGCACCGCAATCAGTC
AGTTAATACTCACCGCTTCGCGATGGTACCTAGAGCCGATATACCACGTAGTAAATTCGATGCTCAAAAAACACATAAAACGACTTTCGATGCGG
GCTATTTAATTCCTGTATATGTTGATGAAGTGCTCCCTGGGGACACTTTCAACTTAAAAATGACGGCATTTGCCCGTCTAGCAACGCCTTTATATC
CAATCATGGACAACATGATTATGGATTCTTTCTTTTTCTTTGTACCCAATCGCCTTATATGGAATAACTGGCAAAAATTTATGGGTCAACAAGAAA
ATCCAACAGACTCAATATCTTATATTGTCCCAACTCAAACAAGCCCAACAGATGGTTATGCCGTAGGCAGCCTTCAAGACTATATGGGCTTACCAA
CAGTAGGCCAAATTGATACTGGCCGAACTATTACGCACTGTGCCTTTTGGCCACGTGCATACAATCTTATTTGGAACGAATGGTTCCGAGATGAA
AATTTACAAACAAGCGCAGTAGTTGATAAGGGCGATGGCCCTGATACTTCCTCAAACTATGTGCTAAAACGTCGTGGTAAAAGACATGATTACTT
TACGTCAGCATTACCATGGCCACAAAAAGGTGCGAGTGTCACCTTACCTTTAGGTACTACGGCTCCAATTAAATGGGATACCATTTCAGGAGACG
CAACATCAAACGATAAATTTACGGTAATTCAAACAGATCCTGGAAATACGACTGCTTTAGCTAGATATGGCAACGCTTATGGTGTTAATACTGCT
GGTGTAGTAAATAACGTTTCTAATTTATATACCGACTTATCAGAAGCAACTGCTGCAACTGTCAATCAATTAAGACAGTCATTTCAAATTCAAAAA
TTACTTGAAAGGGATGCACGTGGCGGAACACGATACACAGAAATTATCCGGAGTCACTTTGGAGTTATTTCCCCAGACGCCCGTTTACAAAGGC
CTGAATACCTTGGAGGCGGTTCAACAATTAATGTTAATCCGATTGCTCAAACGTCGGGAACAAACGCTTCTGGAACGACTACCCCTTTGGGCAAC
CTTGCTGCTATGGGTACTGCTCTCGCTCATAATCATGGATTTACTCAATCATTTACTGAGCATGGCGTTATTATTGGATTAGTATCCATTAGAGCA
GATCTTACTTATCAACAAGGATTAGACCGTATGTGGTCTAGATCTACACGATATGACTTTTATTTCCCAGCATTTGCTACTCTAGGCGAACAATCT
GTTTTGCAAAAAGAAATTTATGCAACAGGAGATACTGCAGCCGACAATACTGTTTTTGGATATCAAGAACGCTGGGCGGAATATCGTTACAAAC
CATCTAAAATTACTGGTTTGTTCAAATCAACATCGGCGGGCACGATCGATGGTTGGCATTTGGCTCAAAATTTACCGCTGCGCCTACTTTGAATAA
TACGTTTATTCAAGATACGCCTCCTGTATCACGTGTAGTAGCCGTTGGAGCAGCTGCAAATGGCCAACAATTCTTATTTGACTCATTTTTTGATGT
CAAAATGGCAAGACCAATGCCAATGTATTCAGTACCTGGCTTAATAGACCATTTCTAATGGGACTATTTGACGGAATTGCCGATTTAATCGGCCC
TGCTATAGCTATAGGAGCTGCCCCTGCTACTGGGGGACTCTCCTTAGCTGCACTTGCACCTGCAGCAATAGGTGCAGCAGGACAATACTTTGGAA
CACAAAGTCAAAACGCAGCGAGTGCAGAACAAGCGAGTAATCAACAGAGATTTCA

>000045F|arrow

CAGCATTTGTACTATTAGACAAAGTATTAGAAGCTGAATTTAATTCTCTTAAAGCACCAACAGCAGTTTGAAAACACTATAACCAGCAGTACCATT
GCCAGCTGGTATGTCAGAATAAATATTAGCACGTGTTCCTAACGGTAAAGAAACAGAAGCACCCTTCTGTGGCCAAGGCAATGCACCAGTAAAA
TCTTTACGCTTACCACGTCTCAAAAGTGTATAGTTAGCAGGAACATCACCTGAATCACCAAGATTTAACGTTACAGAATTCTGTAAGTTTTCATCTC
TAAACCATTCGTTATAAATCTCATTATAAGCACGTAACGGCAAAACATTATGCGTTACTGTATTAGCGCCAGTAATCTGACCAGCAGTAGGTAAA
CCAAAATAATCAAAATTGAACAAACAGCATAACCACCAGCAGGACTAGTAATAGTAGGCACTACAAAAAATAGAATCACCAGGATTCGTTTGTT
CACCCATAAACTTTGGCCAATTGTTCCAAAGTAAACGATTAGGTACAAAGAAAAAGAAAGTATCAAGATGCAAGTTGTCCATCACAGGAAATAA
CGGTGTGGCCAAACGTGCAAATGCAGTCATCTTTACACGATGAGTATCGCCAGGCAATACTTCATCACAATAAATAGGAACTAAAAACCAGCATC
AAACGTGGTTTTATGAGCATATTGAGTATTAAAACTAGAGCGGGGAATGTCCGCTTTAGGAACCATAGCAATGAATGCGTACTTACTGACTTATT
GCGAAACATAAACATCTCCCGTAGTTCCGTACCACTCTTTCGAGTGATACGGTATAAAAAAACCTTACTCGCCTTCGCGAATCATTACATCTTTTG
CACGAGCAATCACTTAGGGCTTTCCAGTAGTTCAAAAACACCAGTGGCATCGTCAAAAGACCCAAGAACATATGAAAATCATCAGGATGTTTATA
CAACTGATTATCTTCGCTAGCTCGATTGACTTCATCCTGAAACTGACGTACTGCAACACCTTCAGATGCAACATAATGGACGACCAAAAGCATCTG
CAGCAGTATCCTTAATAGAAACAATAACCATCTTCATAAAACTCCTTAAATAGTACGTTTTAACAATGACAACTTAGCCAACGCAACTTTTTCCTTA
ACAGCAAGTCGCTCAAGCGTGTTGTCCTCATGCCTAGATCGACCTTCCATCTCTCTGGCAAACTGAATCATATCGAATTCTTCAGGAAACTTCAAC
TTAAATTTATTATCATAAAACCGTGGTGGACGGCACTTTTTGCCACGCACCACAACGTGGTCTGACGTATAAACGTCTGACATGTACTTATCTAAC
CACGATTGCCCGATACCGGGCTTCAATGACATCTTATTAAATTCTGGCTTACGCTGAATTATCTCACCATCTCTAAATCACAATATTGATAATGGG
CACCCGCATCAACCACTTCGTGGTTTTCATTGACAGTAACCCCATTAATCTTCTTCATAATATATCTTGCAACATAAGCAGCAGACTCAAAGTAACA
TCACCAATTGTAGAATAGCCAAACGGCCACAATTCTTCCAAAATCTCTGACGTGTAGAGGATAGAGCCAGTCTGCGTTCTTTTAAATATTTTCTTA
TCCGGAAAATCAAGACCAAACAGACAAGCATGGAAATGAGGACGATCAAAAGATTCACCATATTCACCTGCCATATAAAACGTATCGTTTTCCCA
GTAAAACGCTTACGTAACCGCTTCATAAAAAGCTGATAATCATTGTAATCCATGACATATCCTTAGGACAATGCTCTGGAGCATATGTCAAAGTA
ATAAAACAATTACTAGTATGCATTTGTGCCTCATGCATACAACGAATCGCCCACTGACGTGAGCGTTCAAGGCGACAACCAACACACTGACCACA
AGGCAATGATAGGTACGGACTACATCCGCACCCGTATTTCCCGCCAAATTATAGACCTGTCACTGCATTGATAAGCCGTTAAGGCTTATAACAGG
CCATAATTACAAACGATAGCCACCACGCTGGGGAGCGTGTCTCATATTAATTGACTTCGTCTTACTAGCAGTTCTGCGAAATGACTTTGCAGATTT
ATATTTTTTTGGCTTTCTTCGTAACATGATGAACTCCGTAGTTAAAATAGTGGTTTGGTGTCACCTAGCACAGTTACATCAAGTAGTAACTGTGCT
GGCCTCAGGATTTCATCCTTCGCCTTAGGTGTTTCTGTAGAAACGATGGGTTCAACCACAGGTTGTCCATCATAAGACCCAATTGAATCGCTTCAT
CACGATTCTGGTCGTTCTCAAGGAACTCCAATAATTTGACAGGATCATGGTCAAATCGGACTCTTAATTTCGCTGGCAGAGCCATGAAATCGTCC
ATAGTTTTAATTTGATTCAACGCAGAATGGTAATCAGTAACACCACTAAAATCGCCGTATTGAGGCGATACAGGGCCGTTGGAAGTTCCCCTGTA
ACCCCGAAACGTTCAATGATGACATTAATGTCACATTCATCTTTCATATGCTGTTGAGCAAGACTTGGGTCTTGACACAGCAACGCATCGTTTACC
GATGCTTCATCTTTATCATAGTTGTACGGATTACGTACAAATGGCAAATTCGCTTTACTCATTATTTGACTCCAATTCCCCAAGGGTTAGTTGATTA
GGATTATTTTTGAGTTTATCAAAATTTCTTTCGAAGCATCGATACCCCTAGACCAATAGCTGGGATGGAGCCAATAGCCTCTTAGTATTATATGAC
TGAGCGGATGTTAAATCCGCAGTCGTATTCTTTAAAGCAATATCAGCCAAAATACGTTTATTTTCGGCTGTAATATTAGGAGCAGTAAGCAACTT
ATTAACAGTATCAGCCCTAGTATTAGCGGTACCAGCTTCAGTAGCTTCAGTCTGGGCGATAATCTGCTTTTCTGTTTCTGATGCATTACGAATCTG
TTGCATCATAGAAGCAGTATTAATAGCTGAATTAGTTGCATTACCTAAAACATTTTCCATAGTAGCAGTTGAACCAGATGGGGTAGAAGCACCAC
CACGTTGATAGGCTAACATAGGAGATAACCAGCAGCTTCTAAATCTTTAACAGCACGCTGGTAACTTGTTCCACTCATATCGGCTTGAAAATCCAT
TTGCCTCTGAGCCATCTCCTGATTAGCTTTATTCAGAAGTAGAACCTAAATAAGAACCAACAGCGCCCAAAGCAGTACCGACACCAGAGTAAAGA
AACTAGAAGCTGAAGACAGCTTAGAACCACATTAGTAACCGCATCAAGTTCCACCAAACATAAACTAACGCCCTTCGGTTGTTTCCTCACTACTCC
TTACGGAGTAGTCGAGGTTATATAAAACATTAGAAATGATCAATTAGACCAGGTACGCTGTACATAGGCATAGGTCTGGCCATCTGACAATCAA
AGAAAGCATCCATTAAAAATTGCTGACCATTTGCTGCAGCTCCGACCGCCGTAGTACGAGCAACTGGAGGGGTCTCTTGAATAAACGTTGAATTC
ACGTAGGAAAGAAGTAAATCGCTGTCCATAATGCCAAGCATCAATAGTGCCAGCAGAAGTAGACCTAAAGAAACCAGTAATTTGAGAAGGTTT
GTAACGATACTCCGCCCAACGTTCTTGATAACCAAATACATCATTGTCGGCTGCAGTACCTTGAACATAAATTTCTTTGTTCAAAATAGCTTGCTCA
CCCAAAGTGGCAAATACTGGGAAATAAATCATATCGTGTAGACCTAGACCACATCTTAGGAAGACCCTGTTGATATGTGAGGTCAGCACGTACA
GAAACTAATCCGATAATGTATCCATGTTCTTGAGCAGCATACGTAAGCCGTGTCCCTGAGCCAATGCAGTACCCATTGCAGCAAGGTTACCTTGC
GGAGTAGCAGAACCAGTAACCGACGTTGCAGAAGTCTGAGCAATCGGATTAACATTAACAAGGGTCGAACCTCCACCAATATATTCAGGACGTT
GTAAACGATAATCCTGTGGAGTTACTCCAAAGTGAGCACGTAGTAACTCAGTATAACGAGTACCACCACGTGCATCGCGCTCCAATAACTTCTGA
ATCTGGAAAGATTGACGAAGTTGGTTAATAGTCGCAGCAGTAGCAGTAGACAAATCAGCGTATAACTGATTAGTAGCAACAC

>000004F|arrow

TTACGTCTTGATAAATTGGTTTGCTAGAACTAGGAGCTGACCCTGATTTAGCAAGTTTTGTTAACTCGTTTGAATAACGAGATTGTGCCAAAGCTT
GAGCAGCTTGAGCACTTGATGTTTTGCCTATTTCATTTTTCATGAAAGTATCGGCAAGTATTTGTTTATATTGAGCTCTAATATTTGGATTTTCATC
CAATTTATTTAATGTATCAGCACGTACAGTTGTCTGTTTGATTACTTGTAAGTTCTGTTTGAGCTTCAATTTGTTTAGATTGAGCAATAGCTTGGTT
TGCTTGCGCAACCGTTTGATAAGCTTGTGTTCCGGACGTAGTTGCATTACCGAGAACATTTTGCATCTGAGCCATAGCTCCAGCTGGTGTTGTGG
CTCCGCCTTGTGAATACGCAAGCATGGGATTTAACCCAGCTTTTTTCATATCTTCAACTGCTCGTTGATATGATGTTCCAGACATTTCAGCTTGAAA
TCTCTGTTGATTACTCGCTTGTTCTGCACTCGCTGCGTTTTGACTTTGTGTTCCAAAGTATTGTCCTGCTGCACCTATTGCTGCAGGTGCAAGTGCA

GCTAAGGAGAGTCCCCCAGTAGCAGGGGCAGCTCCTATAGCTATAGCAGGGCCGATTAAATCGGCAATTCCGTCAAATAGTCCCATTAGAAATG
GTCTATTAAGCCAGGTACTGAATACATTGGCATTGGTCTTGCCATTTTGACATCAAAAAATGAGTCAAATAAGAATTGTTGGCCATTTGCAGCTG
CTCCAACGGCTACTACACGTGATACAGGAGGCGTATCTTGAATAAACGTATTATTCAAAGTAGGCGCAGCGGTAAATTTTTGAGCCAAATGCCA
ACCATCGATCGTGCCCGCCGATGTTGATTTGAACAAACCAGTAATTTTAGATGGTTTGTAACGATATTCCGCCCAGCGTTCTTGATATCCAAAAAC
AGTATTGTCGGCTGCAGTATCTCCTGTTGCATAAATTTCTTTTTGCAAAACAGATTGTTCGCCTAGAGTAGCAAATGCTGGGAAATAAAAGTCATA
TCGTGTAGATCTAGACCACATACGGTCTAATCCTTGTTGATAAGTAAGATCTGCTCTAATGGATACTAATCCAATAATAACGCCATGCTCAGTAAA
TGATTGAGTAAATCCATGATTATGAGCGAGAGCAGTACCCATAGCAGCAAGGTTGCCCAAAGGGGTAGTCGTTCCAGAAGCGTTTGTTCCCGAC
GTTTGAGCAATCGGATTAACATTAATTGGTGTTGAACCGCCTCCAAGGTATTCAGGCCTTTGTAAACGGGCGTCTGGGGAAATAACTCCAAAGT
GACTCCGGATAATTTCTGTGTATCGTGTTCCGCCACGTGCATCCCTTTCAAGTAATTTTTGAATTTGAAATGACTGTCTTAATTGATTGACAGTTGC
AGCAGTTGCTTCTGATAAGTCGGTATATAAATTAGAAACGTTATTTACTACACCAGCAGTATTAACACCATAAGCGTTGCCATATCTAGCTAAAGC
AGTCGTATTTCCAGGATCTGTTTGAATTACCGTAAATTTATCGTTTGATGTTGCGTCTCCTGAAATGGTATCCCATTTAATTGGAGCCGTAGTACCT
AAAGGTAAGGTGACACTCGCACCTTTTTGTGGCCATGGTAATGCTGACGTAAAGTAATCATGTCTTTTACCACGACGTTTTAGCACATAGTTTGA
GGAAGTATCAGGGCCATCGCCCTTATCAACTACTGCGCTTGTTTGTAAATTTTCATCTCGGAACCATTCGTTCCAGATAAGATTGTATGCACGTGG
CCAAAAGGCACAGTGCGTAATAGTTCGGCCAGTATCAATTTGGCCTACTGTTGGTAAGCCCATATAGTCTTGAAGGCTGCCTACGGCATAACCAT
CTGTTGGGCTTGTTTGTGTTGGGACAATATAAGATATTGAGTCTGTTGGATTTTCTTGTTGACCCATAAATTTTTGCCAGTTATTCCATATAAGGC
GATTGGGTACAAAGAAAAGAAAGAATCCATAATCATGTTATCCATGATTGGATATAAAGGCGTTGCTAGACGGGCAAATGCCGTCATTTTTAAG
TTGAAAGTGTCCCCAGGGAGCACTTCATCAACATATACAGGAATTAAATAGCCCGCATCGAAAGTCGTTTTATGTGTTTTTTGAGCATCGAATTTA
CTACGTGGTATATCGGCTCTAGGTACCATCGCGAAGCGGTGAGTATTAACTGACTGATTGCGGTGCATGTTTTTCCTTAGTGTTGTTCCGGGGGA
AAGATAAATCTCTTTTCCCCTCGGTTGTTTTATTTAAGTTTAACTTGTTTTCCTAATGATAGGAGTTTTGGTTGTTCATGTAAATCGAATAACCCAGT
TGAATCGTCAAATGTTCCGAATTCATATAGATCGAAGTCATCAGGGTGATTAAAAAGTTGATTTTCAGTATCAGAACGATTAATTTCATCTGAAAA
AGAGCGTATAGCTACTCCAGAGGAAGGTACGAACATTGGTCGTGCATATGCTTCAGCAGCACGGTCTTTACGGAAGCGAGGATAAGTTTCATT
ATTTTCCTAAGTGAGGTTACGTTTTAATAGTTGAAGTTTTGCCATAGTGACTTGTTCTTTTGCAGATAGTCGTTCTGGTGTATTGTCTTCGGAATTA
AGTTTAGCATTATTTTCCCGCATGTAAAGTAATTCGTCATACTCATAAGGTTGGTCAATTTTAAACATTTTGTCATAGTATTTGGTGGTTTGACCT
TTTTACCTCTAAGTATTACGTAGTCTTGCGGGTATATATCCGAAGTATATTTTATATAAAAGTCTTTACCGATTCCCGGTTTTAAAGACATTTTATTA
TATTCCGGCTTTAAGTCTAAATATTCGCCGGTTTCAGGGTGTATGCGTTTGTAATGAGATTCCGCATCTTTCCCTGTTTGTTTTTTCATTATGTATCT
AGCCACGTAGGCGGCTGATTCGAAAGTAACATCTCCAATGGTGGTATAACCAAATGGCCAGAGAGCTTCAAGTTCTGCGGATCTATATAACATA
GAACCAGAGGCAGTCCTTTTCCATAATTTTTTATCAGGAAAGTCGTATCCGAAGATACAGGCATGGAAGTGGGGTCTTGCGAAGAGTTCACCAT
ATTCTCCAGCCATGTAATAGCGGATTGTAAGTCCTCTTTTTGCGAGAGTTTTTCTAAGTCTTTTAAGGAACAGTTGAAAGTCTTTGTGATCCAAAG
AGCCATCGCTTGGGAGATGTGTATTGTCATATGTGAGTGTTATGAATGAGTTGTTTTTATGCAATTGGGCTTCGTGCATGCACCGAATAGCCCAT
TGTCTTGATCGTTCTAGACGGCATCCAACGCACTGCCCACAGGGCAGGTCTAAAGATCGAACGATGTCATGTTTCCGAACTTCGTCGAAAACAAT
TGATTTGTCAAAGCATTGAAATGCTTTGAGAGGGTGATAACAAGGCATGTGAGGTGCCTGGGGACTTTATTAGAGTCTCCAGCCTCCACGCATT
GGCGAGTTTCGCATATTTGCGTGTTTTGTATGTGAAGCATGTTTACGGAAAGTCCTAGCGGACTTTTGCTTATTTGCTGGTCTGCGACGCATCATT
TTTTCTGTCCTTGTTTATCGTGTTTTTGTGGTTTGGTGTCACCTAGCACAGTTACATCAAGTAGGTAACTGTGCTTCCGAGGTTCCAACAGGGGTT
GAAACCTCGGTTTGGGCTGGTTTTACCAGTCCCATTTTTTCAGCTTCGCTGCGATTTTCAGGGTTTTCTAGAAAATCTATTAGATTCGCGGATCATT
AGCGAATCGTTCACGAATATTGGCTGGTAAAGCCATAAATTCGTTGTCTGAAGCCATAATCTGGTTCAGAGCAGAGTGGTAGTCATAGACACCA
CTAAAGTCGCCATATTGAGGCGTTAAAGGAGTTTGAGGAATAAGTCCGGTCATACCGAACTTTTCCATAATATTATTGATATCACATTCTTCAGCA
AATTGCTGCTGAGTCAGAGTTGCATCCTCACAATGCAGCCCTGACTCATTTGACGCAGCAATCGTGTCGTAATTGTACGGAGTACGAAGAAATG
GGGCTGTAATCTTTGTCATTTTCATTCCATTGGTGGTTGGTTATTTGTACGATTTTGTTGAATCGGTTGACCTCGATAGTTATCGATGTATCGTTTT
GCGCCAGATGCGCTATACGCATCTTTGGCGATGTTTT

>000076F|arrow

ACCTTGCGGAGTAGCAGAACCAGTAACCGACGTTGCAGAAGTCTGAGCAATCGGATTAACATTAACAAGGGTCGAACCTCCACCAATATATTCA
GGACGTTGTAAACGATAATCCTGTGGAGTTACTCCAAAGTGAGCACGTAGTAACTCAGTATAACGAGTACCACCACGTGCATCGCGCTCCAATA
ACTTCTGAATCTGGAAAGATTGACGAAGTTGGTTAATAGTCGCAGCAGTAGCAGTAGACAAATCAGCGTATAACTGATTAGTAGCAACACCAGC
ATTTGTACTATTAGACAAAGTATTAGAAGCTGAATTTAATTCTCTTAAAGCACCAACAGCAGTTTGAAAAACACTATAACCAGCAGTACCATTGCC
AGCTGGTATGTCAGAATAAATATTAGCAGTGTTCCTAACGGTAAAGAAACAGAAGCACCCTTCTGTGGCCAAGGCAATGCACCAGTAAAATAAT
CTTTACGCTTACCACGTCTCAAAAGTGTATAGTTAGCAGGAACATCACCTGAATCACCAAGATTTAACGTTACAGAATTCTGTAAGTTTTCATCTCT
AAACCATTCGTTATAAATCTCATTATAAGCACGTAACGGCAAAACATTATGCGTTACTGTATTAGCGCCAGTAATCTGACCAGCAGTAGGTAAAC
CAAAATAATCAAAAATGAACAAACAGCATAACCACCAGCAGGACTAGTAATAGTAGGCACTACAAAAGAAATAGAATCACCAGGATTCGTTTGT
TCACCCATAAACTTTGGCCAATTGTTCCAAAGTAAACGATTAGGTACAAAGAAAAAGAAAGTATCAAGATGCAAGTTGTCCATCACAGGAAATA
ACGGTGTGGCCAAACGTGCAAATGCAGTCATCTTTACACGATGAGTATCGCCAGGCAATACTTCATCACAATAAATAGGAACTAAAAAACCAGC
ATCAAACGTGGTTTTATGAGCATATTGAGTATTAAAACTAGAGCGGGGAATGTCCGCTTTAGGAACCATAGCAAATGAATGCGTACTTACTGACT

TATTGCGAAACATAAACATCTCCCGTAGTTCCGTACCACTCTTTCGAGTGATACGGTATAAAAAAAACCTTACTCGCCTTCGCGAATCATTACATC
TTTTGCACGAGCAATCAACTTAGGGCTTTCCAGTAGTTCAAAAACACCAGTGGCATCGTCAAAAAGACCCAAATAGAACATATGAAAATCATCAG
GATGTTTATACAACTGATTATCTTCGCTAGCTCGATTGACTTCATCCTGAAACTGACGTACTGCAACACCTTCAGATGCAACATAAGCTGGACGAC
CAAAAGCATCTGCAGCAGTATCCTTAATAGAAACAATAACCATCTTCATAAAAACTCCTTAAATAGTACGTTTTAACAATGACAACTTAGCCAACG
CAACTTTTTCCTTAACAGCAAGTCGCTCAAGCGTGTTGTCCTCATGCCTAGATCGACCTTCCATCTCTCTGGCAAACTGAATCATATCGAATTCTTC
AGGAAACTTCAACTTAAATTTATTATCATAAAACCGTGGTGGACGGCACTTTTGCCACGCACCACAACGTGGTCTGACGTATAAACGTCTGACAT
GTACTTATCTAACCACGATTGCCCGATACCGGGCTTCAATGACATCTTATTAAATTCTGGCTTACGCTGAATTATCTCACCAGTCTCTAAATCACAT
ATTGATAATGGGCACCCGCATCAACCACTTCGTGGTTTTCATTGACAGTAACCCCATTAATCTTCTTCATAATATATCTTGCAACATAAGCAGCAG
ACTCAAAAGTAACATCACCATTGTAGAATAGCCAAACGGCCACAATTCTTCCAAAATCTCTGACGTGTAGAGGATAGAGCCAGTCTGCGTTCTTT
TAAATATTTTCTTATCCGGAAAATCAAGACCAAACAGACAAGCATGGAAATGAGGACGATCAAAAGATTCACCATATTCACCTGCCATATAAAAA
CGTATCGTTTTCCCAGTAAAACGCTTACGTAACCGCTTCATAAAAAGCTGATAATCATTGTAATCCAATGACATATCCTTAGGACAATGCTCTGGA
GCATATGTCAAAGTAATAAAAAATTATAGTATGCATTTGTGCCTCATGCATACAACGAATCGCCCACTGACGTGAGCGTTCAAGGCGACAACCAA
CACACTGACCACAAGGCAATGATAGGGTACGGACTACATCCGCCCGGTATTTCCCGCCAAATTATAGACCTGTCACTGCATTGATAAGCCGTTAA
GGGCTTATAACAGGCCATAATTACAAACGATAGCCACCACGCTGGGGAGCGTGTCTCATATTAATTGACTTCGTCTTACTAGCAGTTCTGCGAAA
TGACTTTGCAGATTTATATTTGTTTACTGGCTTTCTTCGTAACATGATGAACTCCGTAGTTAAAATAGTGGTTTGGTGTCACCTAGCACAGTTACAT
CAAGTAGAGTAACTGTGCTGGCCTCAGGATTTCATCCTTCGGCCTTAGGTGTTTCTGTAGAAACGATGGGTTCAACCACAGGTTGTCCATCAATA
AGACCCAATTGAATCGCTTCATCACGATTCTGGTCGTTCTCAAGGAACTCCAATAATTTGACAGGATCATGGTCAAATCGGACTCTTAATTTCGCT
GGCAGAGCCATGAAATCGTCCATAGTTGCGTTAATTTGATCAACGCAGAATGGTAATCAGTAACACCACTAAAATCGCCGTATTGAGGCGATAC
AGGGGCCGTTGGAAGTTCCCCTGTAACCCCGAAACGTTCAATGATGACATTAATGTCACATCTTTCATATGCTGTGAGCAAGACTTGGGTCTTGA
CACAGCAACGCATCGTTTACCGATGCTTCATCTTTATCATAGTTGTACGGATTACGTACAAATGGCAAATTCGCTTTACTCATTATTTGACTCCAAT
TCCCCAAGGGGTTAGTTGATTAGGATTATTTTTGAGTTTATCAAAATTTCTTTCGAAGCATCGATACCCCTAGACCAAATAGCTGGGGATGGAGC
CAATAGCCTCTTAGTATTATATGACTGAGCGGATGTTAAATCCGCAGTCGTATTCTTTAAAGCAATATCAGCCAAAATACGTTTATTTTCGGCTGT
AATATTAGGAGCAGTAAGCAACTTATTAACAGTATCAGCCCTAGTATTAGCGGTACCAGCTTCAGTAGCTTCAGTCTGGGCGATAATCTGCTTTTC
TGTTTCTGATGCATTACGAATCTGTTGCATCATAGAAGCAGTATTAATAGCTGAATTAGTTGCATTACCTAAAACATTTTCCATAGTAGCAGTTGA
ACCAGATGGGGTAGAAGCACCACCACGTTGATAGGCTAACATAGGAGATAAACCAGCAGCTTCTAAATCTTTAACAGCACGCTGGTAACTTGTT
CCACTCATATCGGCTTGAAAATCCATTTGCCTCTGAGCCATCTCCTGATTAGCTTTATTAGCAGAAGTAGAACCTAAATAAGAACCAACAGCGCCC
AAAGCAGTACCGACACCAGGAGTAAAGAAACTAGAAGCTGAAGACAGCTTAGAACCAACATTAGTAACCGCATCAAGTATTCCACCAAACATAA
ACTAACGCCCTTCGGTTGTTTCCTCACTACTCCTTACGGAGTAGTCGAGGTTATATAAAACATTAGAAATGATCAATTAGACCAGGTACGCTGTAC
ATAGGCATAGGTCTGGCCATCTGACAATCAAAGAAAGCATCCATTAAAAATTGCTGACCATTTGCTGCAGCTCCGACCGCCGTAGTACGAGCAAC
TGGAGGGGTCTCTTGAAAAACGTTGAATTCAACGTAGGAAGAGAAGTAAATCGCTGTCCATAATGCCAAGCATCAATAGTGCCAGCAGAAGTA
GACCTAAAGAAACCAGTAATTTGAGAAGGTTTGTAACGATACTCCGCCCAACGTTCTTGATAACCAAATACATCATTGTCGGCTGCAGTACCTTG
AACATAAATTTCTTTGTTCAAAATAGCTTGCTCACCCAAAGTGGCAAATACTGGGAAATAAAAGTCATATCGTGTAGACCTAGACCACATCTTAG
GAAGACCCTGTTGATATGTGAGGTCAGCACGTACAGAAACTAATCCGATAATGTATCCATGTTCTTGAGCAGCATACGTAAAGCCGTGTCCCTGA
GCCATGCAGTACCCATTGCAGCAAGGTT

>000221F|arrow

GCTGAAAAAATGGGACTGGTAAAACCAGCCCAAACCGAGGTTTCAACCCTGTTGGAACCTCGGAAGCACAGTTACCTACTTGATGTAACTGTGC
TAGGTGACACCAAACCACAAAACACGATAACAAGGACAGAAAAAATGATGCGTCGCAGACCAGCAAATAAGCAAAAGTCCGCTAGGACTTTCC
GTAAACATGCTTCACATACAAAACACGCAAATATGCGAAACTCGCCAATGCGTGGAGGCTGGAGACTCTAATAAAGTCCCCAGGCACCTCACAT
GCCTTGTTATCACCCTCTCAAAGCATTTCAATGCTTTGACAAATCAATTGTTTTCGACGAAGTTCGGAAACATGACATCGTTCGATCTTTAGACCTG
CCCTGTGGGCAGGTGCGTTGGATGCCGTCTAGAACGATCAAGACAATGGGCTATTCGGTGCATGCACGAAGCCCAATTGCATAAAAACAACTCA
TTCATAACACTCACATATGACAATACACATCTCCCAAGCGATGGCTCTTTGGATCACAAAGACTTTCAACTGTTCCTTAAAAGACTTAGAAAAACT
CTCGCAAAAAGAGGACTTACAATCCGCTATTACATGGCTGGAGAATATGGTGAACTCTTCGCAAGACCCCACTTCCATGCCTGTATCTTCGGATA
CGACTTTCCTGATAAAAAATTATGGAAAAGGACTGCCTCTGGTTCTATGTTATATAGATCCGCAGAACTTGAAGCTCTCTGGCCATTTGGTTATAC
CACCATTGGAGATGTTACTTTCGAATCAGCCGCCTACGTGGCTAGATACATAATGAAAAAAACAAACAGGGAAAGATGCGGAATCTCATTACAA
ACGCATACACCCTGAAACCGGCGAATATTAGACTTAAAGCCGGAATATAATAAATGTCTTTAAAACCGGGAATCGGTAAAGACTTTTATATAAAA
TACTTCGGATATACCCGCAAGACTACGTTAATACTTAGAGTAAAAGTCAAACCACCAAAACTATGACAAAATGTTTAAAATTGACCAACCTTATG
AGTAATGACGAATTACTTTCAATGCAGGGAAAATAGACGCGTACAACTTAATTCCGGACAGACAAATACACCAAGAACGACTATCTGCAAAAGA
ACAAGATAAAATGGCAAAACTTCAACTCTTTACATAACGTACCTCACTGAGGAAATAATGAAAAACTTATCCTCGCTTCCGTAAAAGACCGTGCT
GCTTAAGCATATGCACGACCAATTTTTCGTACTTCCTTCCTCTGGAGTAGCTATACGCTCTTTTTTCAGATGAAACTTAATCGTTCTGATACTGAAG
AATACATAACTTTTTATCACCCTGATGACTTCGGTATCATATGAATTCGGAAACTTTGACCGATTCAAACTGGGGTTAATTCGGATTTACATGAAC
AACCAAAACTTCTATCATTAGGAAACAAGTGTAAACTTAATAAAACTAACCGCAGGGGAAAAGAGATTTATCTTTCCCCTGGAAACAACACTAAG

GAAAAACATGCACCGCAATCAGTCAGTAATACTCATCGCTTCGCGAATGGGTCCTAGAGCCGATATACCACGTAGTAAATTCGATGCTCCAAAAC
ACATAAACGACTTTCGATGCGGGGTCATATTCCGTAATGTTTATTAATTGTCTCCTGGGGACACTTTCACTTAAAAATGACGGCCATTTGCCCGTC
TAGCCAAACCGCCTTATATCCAATGCATTGGACACATGATTATGGATGTCTTTCGTTTTCTTTGTAACCAATTCGCCTATATGGAATACTGGCAAA
AATTATGGTTCAACAAGAAAATCCAAACAGACTCAATACTTATATTGTCCCAACTCAAACAAGCCCAACAGAATGGTTATGCCGTGCAGCCTTCA
AGACTATTTGCTGACCAACGGAGGCCAAATTGATACTGGCCGAACTATTCCGTCACTGTGCCTTTTGGCCACGTGCATACAATCTTAGTTTGAAC
GAATGGTTCCGAGAATGAAAATTTTACAAACAAGCGCAGTAGTTGATAAGTGGCGAGCGGCCCTGATACTTCCTCAAACTATGTGCTAAAACGT
CGTGGTAAAAGACATGATTACTTTACGTCAGCATTACCATGGCCACAAAAAGGTGCGAGTGTCACCTTACCTTTAGGTACTACGGCTCCAATTAA
ATGGGATACCATTTCAGGAGACGCACATCAAACGATAAATTTACGGTAATTCACACAGATCCTGGAAATACGACTGCTTTAGCTAGATATGGCAA
CGCTGTATGGTGTTAATACTGCTGGTGTAGTAATAACGTTTCTAATTTATATACCGACTTATCAGAAGCAACTGCTGCAACTGTCAATCAATTAAG
ACAGTCATTTCAAATTCAAAAATTACTTGAAAGGGATGCACGTGCGGAACACGATACACAGAAATTATCCGGAGTCACTTTGGAGTTATTTCCCC
AGACGCCCGGTTTACAAAGGCCGTGATACCTTGGAGGCGGTTCAACACCAATTAATGTTAATCCGATTGCTCAAACGTCGGGAACAAACGCTTTC
TGGACGAGCTACCCCTTGGGGCAACCTTGCTGCTATGGGTACTGCTCTCGCTCATAATCATTGGATTACTCATCATTTACTGAGCATGGCGTTATT
ATTGGATTAGTATCCATAGAGCAGATCTTACTTATCAACAAGGGATTAGACCGTATGTGGTCTAGATCTACACGATATGACTTTTATTTCCCAGCA
TTTGCTACTCTAGGCGAACATCTGTTTTGCAAAAAGAAATTTAGGCAACAGGAGATACTGCAGCCGACAATACTGTTTTTGGATATCAGAAACGC
TGGGCGGAATATCGTTACAAACCATCTAAAATTACTGGTTTGTTCAAATCAACATCGGCGGGCACGATCGATGGTTGGCATTGGCTCAAAATTTA
CCGCTGCGCCTACTTTGAATAATACGTTTATTCAAGATACGCCTCCTGTATCACGTGTAGTAGCCGTTGGAGCAGCTGCAAATGGCCAACAATTCT
TATTTGACTCATTTTTTGATGTCAAAATGGCAAGACCAATGCCAATGTATTCAGTACCTGGCTTAAATAGACCATTTCTAATGGACTATTTGACGG
AATTGCCGATTTAAATCGGCCCTGCTATAGCTATAGGAGCTGCCCCTGCTACTGGGGGACTCTCCTTAGCTGCACTTGCACCTGCAGCAATAGGT
GCAGCAGGACAATACTTTGGAACACAAAGTCAAAACGCAGCGAGTGCAGAACAAGCGGAGTAATCAACAGAGATTTCAAGCTGAAATGTCTGG
AACATCATATCAACGAGCAGTTGAAGATATGAAAAAAGCTGGGTTAAATCCCATGCTTGCGTATTCACAAGGCGGAGCCACAACACAGCTGGAG
CTATGGCCCAGATGCAAAATGTTCTCGGTAATGCAACTACGTCCGGAACCCAAGCTTATCAAACGGTTGCTCAAGCAAATCAAGCTATTGCTCAA
TCTAACAAATTGAAGCTCAAACGCCACTCACAAGTAATCAAACAGATAATGTACGTGCTGATACGTTTAAACAATTGATGAAAATCCAAATATTA
GAGCTCAATATAAACAAATACTTGCCGATACTTTCATGAAAAATGAAATAGGCAAAACATCAAGTGCTCAAGCTGCTCAAGCTTTGGCACAATCT
CGTTATTCAAACGAGTTAACAAAACTTGCTAAATCAGGGTCAGCTCCTAGTTCTAGCAAACCAATTTATCAAGACGTAAAAAACATCGCCAAAGA
TGCGTATAGCGCATCTGGCGCAAAACGATACATCGATAACTATCGAGGTCAACCGATTCAACAAAATCGTACAAATAACCAACCACCAATGGAA
TGAAAATGACAAAGATTACAGCCCCATTTCTTCGTACTCCGTACAATTACGACACGATTGCTGCGTCAAATGAGTCAGGGCTGCATTGTGAGGAT
GCAACTCTGACTCAGCAGCAATTTGCTGAAGAATGCGATATTAATAATATTATGGAAAAGTTTGGTATGACCGGACTTATTCCTCAAACTCCTTTA
TCGCCTCAATATGGCGACTTTAGTGGTGTCTATGACTACCACTCTGCTCTGAACCAGATTATGGCTTCAGACAACGAATTTATGGCTTTACCAGCC
AATATTCGTGAACGATTCGCTAATGATCCCGCGAATCTAATAGATTTTCTAGAAAATCCTGAAAATCGCAGCGAA

>000157F|arrow

AATCGCCCACTGACGTGAGCGTTCAAGGCGACAACCAACACACTGACCACAAGGCAATGATAGGGTACGGACTACATCCGCACCCGGTTTCCCG
CCAAATTATAGACCTGTCACTGCATTGATAAGCCGTTAAGGGCTTATAACAGGCCATAATTACAAACGATAGCCACCACGCTGGGGAGCGTGTCT
CATATTAATTGACTTCGTCTTACTAGCAGTTCTGCGAAATGACTTTGCAGATTATATTTGTTACTGGCTTTCTTCGTAACATGATGAACTCCGTAGT
TAAAATAGTGGTTTGGTGTCACCTAGCACAGTTACATCAAGTAGAGTAACTGTGCTGGCCTCAGGATTTCATCCTTCGGCCTTAGGTGTTTCTGTA
GAAACGATGGGTTCAACCACAGGTTGTCCATCAATAAGACCCAATTGAATCGCTTCATCACGATTCTGGTCGTTCTCAAGGAACTCCAATAATTG
ACAGGATCATGGTCAAATCGGACTCTTAATTTCGCTGGCAGAGCCATGAAATCGTCCATAGTTGCGTTAATTTGATTCAACGCAGAATGGTAATC
AGTAACACCACTAAAATCGCCGTATTGAGGCGATACAGGGGCCGTTGGAAGTTCCCCTGTAACCCCGAAACGTTCAATGATGACATTAATGTCAT
TCATCTTTCATATGCTGTTGAGCAAGACTTGGGTCTTGACACAGCAACGCATCGTTTACCGATGCTTCATCTTTATCATAGTTGTACGGATTACGT
ACAAATGGCAAATTCGCTTACTCATTATTTGACTCCAATTCCCCAAGGGGTTAGTTGATTAGGATTATTTTTGAGTTTATCAAAAATTTCTTTCGAA
GCATCGATACCCCTAGACCAAATAGCTGGGGATGGAGCAATAGCCTCTTAGTATTATATGACTGAGCGGATGTTAAATCCGCAGTCGTATTCTTT
AAAGCAATATCAGCCAAAATACGTTTATTTCGGCTGTAATATAGGAGCAGTAAGCAACTTATTAACAGTATCAGCCTAGTATTAGCGGTACCAGC
TTCAGTAGCTTCAGTCTGGGCGATAATCTGCTTTTCTGTTTCTGATGCATTACGAATCTGTTGCATCATAGAAGCAGTATTAATAGCTGAATAGTT
GCATTACCTAAAACATTTTCCATAGTAGCAGTTGAACCAGATGGGGTAGAAGCACCACCACGTTGATAGGCTAACATAGGAGATAAACCAGCAG
CTTCTAAATCTTTAACAGCACGCTGGTAACTTGTTCCACTCATATCGGCTTGAAAATCCATTTGCCTCTGAGCCATCTCCTGATTAGCTTTATTAGC
AGAAGTAGAACCTAAATAAGAACCAACAGCGCCCAAAGCAGTACCGACACCAGGAGTAAAGAAACTAGAAGCTGAAGACAGCTTAGAACCAAC
ATTAGTAACCGCATCAAGTATTCCACCAAACATAAACTAACGCCCTTCGGTTGTTTCCTCACTACTCCTTACGGAGTAGTCGAGGTTATATAAAAC
ATTAGAAATGATCAATTAGACCAGGTACGCTGTACATAGGCATAGGTCTGGCCATCTGACAATCAAAGAAAGCATCCATTAAAAATTGCTGACCA
TTTGCTGCAGCTCCGACCGCCGTAGTACGAGCAACTGGAGGGGTCTCTTGAATAAACGTTGAATTCAACGTAGGAAGAGAAGTAAATCGCTGTC
CATAATGCCAAGCATCAATAGTGCCAGAGAAGTAGACCTAAAGAAACCAGTAATTTGAGAAGGTTTGTAACGATACTCCGCCCAACGTTCTTGAT
AACCAAATACATCATTGTCGGCTGCAGTACCTTGAACATAAATTTCTTTGTTCAAAATAGCTTGCTCACCCAAAGTGGCAAATACTGGGAAATAAA
AGTCATATCGTGTAGACCTAGACCACATCTTAGGAAGACCCTGTTGATATGTGAGGTCAGCACGTACAAAACTAATCCGATAATGTATCCATGTC

TTGAGCAGCATACGTAAAGCCGTGTCCCTGAGCCAATGCAGTACCCATTGCAGCAAGGTTACCTTGCGGAGTAGCAGAACCAGTAACCGACGTT
GCAGAAGTCTGAGCAATCGGATTAACATTAACAAGGGTCGAACCTCCACCAATATATTCAGGACGTTGTAAACGATAATCTGTGGAGTTACTCCA
AAGTGAGCACGTAGTAATCAGTATAACGAGTACCACCACGTGCATCGCGCTCCAATAACTTCTGAATCTGGAAAGATTGACGAAGTTGGTTAAT
AGTCGCAGCAGTAGCAGTAGACAAATCAGCGTATAACTGATTAGTAGCAACACCAGCATTTGTACTATTAGACAAAGTATTAGAAGCTGAATTTA
ATTCTCTTAAAGCACCAACAGCAGTTTGAAAAACACTATAACCAGCAGTACCATTGCCAGCTGGTATGTCAGAATAAATATTAGCACGTGTTCCTA
ACGGTAAAGAAACAGAAGCACCCTTCTGTGGCCAAGGCAATGCACCAGTAAAATAATCTTTACGCTTACCACGTCTCAAAAGTGTATAGTTAGCA
GGAACATCACCTGAATCACCAAGATTTAACGTTACAGAATTCTGTAAGTTTTCATCTCTAAACCATTCGTTATAAATCTCATTATAAGCACGTAAC
GGCAAAACATTATGCGTTACTGTATTAGCGCCAGTAATCTGACCAGCAGTAGGTAAACCAAAATAATCAAAAATTGAACAAACAGCATAACCAC
CAGCAGGACTAGTAATAGTAGGCACTACAAAAGAAATAGAATCACCAGGATTCGTTTGTTCACCCATAAACTTTGGCCAATTGTTCCAAAGTAAA
CGATTAGGTACAAAGAAAAAGAAAGTATCAAGATGCAAGTTGTCCATCACAGGAAATAACGGTGTGGCCAAACGTGCAAATGCAGTCATCTTTA
CACGATGAGTATCGCCAGGCAATACTTCATCACAATAAATAGGAACTAAAAAACCAGCATCAAACGTGGTTTTATGAGCATATTGAGTATTAAAA
CTAGAGCGGGGAATGTCCGCTTAGGAACCATAGCAAATGAATGCGTACTTACTGACTTATTGCGAAACATAAACATCTCCCGTAGTTCCGTACCA
CTCTTTCGAGTGATACGGTATAAAAAAAACCTTACTCGCCTTCGCGAATCATTACATCTTTTGCACGAGCAATCAACTTAGGGCTTTCCAGTAGTT
CAAAAACACCAGTGGCATCGTCAAAAAGACCCAAATAGAACATATGAAAATCATCAGGATGTTTATACAACTGATTATCTTCGCTAGCTCGATTG
ACTTCATCCTGAAACTGACGTACTGCAACACCTTCAGATGCAAACATAAGCTGGACGACCAAAAGCATCTGCAGCAGTATCCTTAATAGAAACAA
TAACCATCTTCATAAAAAACTCCTTAAATAGTACGTTTTAACAATGACAACTTAGCCAACGCAACTTTTCCTTAACAGCAAGTCGCTCAAGCGTGTT
GTCTCATGCCTAGATCGACCTTCCATCTCTCTGGCAAACTGAATCATATCGAATTCTTCAGGAAACTTCAACTTAAATTTATTATCAAAAACCGTGG
TGGACGGCACTTTTTGCCACGCACCACAACGTGGTCTGACGTATAAACGTCTGACATGTACTTATCTAACCACGATTGCCCGATACCGGGCTTCA
ATGACATCTTATTAAATTCTGGCTTACGCTGAATTATCTCACCAGTCTCTAAATCACAATATTGATAATGGGCACCCGCATCAACCACTTCGTGGTT
TTCATTGACAGTAACCCATTAATCTTCTTCATAATATATCTTGCAACATAAGCAGCAGACTCAAAAGTAACATCACCAATTGTAGAATAGCCAAAC
GGCCACAATTCTTCCAAAATCTCTGACGTGTAGAGGATAGAGCCAGTCTGCGTTCTTTTAAATATTTTCTTATCCGGAAAATCAAGACCAAACAGA
CAAGCATGGAAATGAGGACGATCAAAAGATTCACCATATTCACCTGCCATATAAAAACGTATCGTTTTCCCAGTAAAACGCTTACGTAACCGCTT
CATAAAAAGCTGATAATCATTGTAATCCAATGACATATCCTTAGGACAATGCTCTGGAGCATATGTCAAAGTAATAAAACAATTACTAGTATGCA
TTTGTGCCTCATGCATACAACG

>000173F|arrow

TTAACGTTGACATAAGTAGAACCACCACCAATATATTCAGGACGTTGTAAACGATAATCTTGTGGAGTTACACCAAAATGAGCACGTAACAATTC
TGTATAGCGTGTACCACCTCGCGCATCGCGCTCTAACAAACGCTGAATCTGGAAAGACTGACGTAACTGGTTAATAGTTGCAGCCGTAGCATCAC
TTAAATCGGCATACAAACCAGTACCAGCAGTACCAGCGTTATTACTACGATACACAGCATGTGTAGTTGAATTAGCATAAATCTGCTTCAAAGCA
CCAGCACCGTCAACTAATGACAACGTTGTTGAATCATTAGTAACAGACGTCTTAATAGGAGCAGACGTGCCTAAAGGCAACGTAACTGCATCGC
CTTTCTGAGGCCAAGGCAAGGCACCAGTAAAATAATCCTTACGCTTACCGCGTCGAACCATAGCATAATCACTCGGAGTATCAGGACCGTCACCC
TTGTGAACGGTAACAGAATTTTGTAAATTCTCATCCCTAAACCACTCGTTATAAATCAAATTATAAGCACGTAACGGTAACGCGTTATGCGTAACC
GTATTAGTACCAGTAATCTGACCAGCCGTAGGCAAACCAAAATGATCAAAAATAGAACCTACTGCATAACCACCAGCAGTAGAAGTAATCTGTG
GAACTACATACGAAATAGAATCACCTGGGTTCGCTTGCTCACCCATAAACTTAACCCAATTGTTCCAAACTAATCTATTTGGAACAAAGAAAAAG
AAAGTATCTAAATGCAAATTGTCCATAACTGGAAACAAAGGCGTTGCCAAACGGGCAAACATTGTAGCCTTTACATTGTGCATGTCCCCTGGGA
GCACTTCATCACAATAAATAGGAACTAGATAACCACCATCAAAAGTAGTTTTATGCGCATATTGCGTATCAAAACTAGAACGCGGAATTTCCGCT
TTAGGAACCATAGCAAAACTATGTGAGCTTACTGACTTATTACGATGCATAACAATCTCCCGAAGTATTCCGAACCACTAGCAAGCTAGTGATCC
GGCTTAAAAAAAACATTACTCGCCATCGCGAATCATAACATCCTTAGCTCTAGAAATAAGCTTGGGAGAACCAAGCAAATCCATAGTACCAGAAT
TATCATCAAACGTACCAAAATAATATAACTGAAAATCATCAGGGTGTTTATATAACTGATTATCATCGCTAGCGCGATTAACTTCGTCCTGAAACT
GACGAACAGCAACACCCTCAGATGCAACAAAAGCTGGACGACCATACGCACCAGCTGCAGTATCTAAAATAGAAACAATAACCATCTTCATAAA
AACTCCTTTAAATCTTACGTTTTAAAAGCGATAACTTAGCCAAAGCGACTTTTTCCTTTACAGCCAAACGCTCTAAAGTGTTATCTTCAAAATGTGA
GCGACCTTCTAGCTCACGAGCATATTGTATACCATCAAATTCTTCTGGAAACAATACTTTAAACTTATTATCATAAAACCGTGGTGGTCGGCACTT
TTTGCCACGCACCACAACAGAGTCAGTCGTATAAACGTCTGACATGAACTTATCAAACCAAGCCTGACCAATGCCAGGCTTAAGAGACATCTTAT
TAAATTCAGGCTTACGCTGAATAATCTCACCAGTATCTAAATCACAATACTGATAATGCGCATCTGCATCAACCACTTCGTGGTTCTCATTAACGG
TTTTACCGTTAATCTTCTTCATAATATATCGAGCAACATAAGCAGCAGATTCAAAATTGACATCACCAATAGAACTATAGCCATACGGCCAAAGTT
CTTCAAGTATCTTTGACGTATATAAGATAGACCCAGTCTGCGTTCTTTTGAAAAACTTCTTATCTTCAAAATCAAGCCCAAAGATACAAGCATGGA
AATGAGGACGATCGAAAGACTCACCATATTCACCTGCCATATAAAAACGAATAGTCTTGCCAGTATAGCGTTTTCTCAATCGTTTCATAAACAATT
GAAAATCTTCATAATGCAAAGACATATCCTTAGGACAATGCTCTGGAGCATATGTCAAAGTAATAAAACAATTACTAGTATGCATTTGTGCCTCAT
GCATACATCTAACGGCCCACTGACGGGACCGTTCAAGGCGACAACCAACACACTGACCACAAGGCAATGACAAGGTACGGACTACGTCCGCCCC
TGGTATCTCCCGCCAAATAATAGACTTGTCAGCGCATTGATAAGCCGTTAACGGCTTATAACACGCCATAAATTACAGTCTAAAACCACCGCGCT
GCGGTGAAGTACGCATATTAATGCTCTTGGTCTTGCTTACGCCACGACGAAACTTCTTAGCTGCGCCATGCTTGCTCATTGGTTTTCTATAAAGGC
TCATAACATTGCACTCCGTAGTTAATAATGTGGTTTTGGTGTCACCTAGCACAGTTACATCAAGTAGAGTAACTGTGCTGCCATCCGCTTACGCGT

CTGGCTTAGGTGTTTCTACTGCAGAAACGACGGGTTCAACCACAGGTTTACCGTCAATAAGACCAATCTGAATCGCTTCATCACGATTCTCTTCAT
TCTGTAAATAATTTAACAAAGCATTAGGATCGTTATCAAACTTAGCCCTAATCTTAGCTGGCAAAGCCATAAAAGCCTCATCAGAGGCACGAATA
GCATTCAATGCGGTGTGATAGTCAGATACACCGCTAAAATCGCCATATGATGGCTCTAATGGCGCCTGGGGCAATTGCCCAGTAACGCCAAAAC
GCTCAACTATAATATTAATATCACATTCGTCTCTCATGTGTTGTTGAGCCAAACTCGGATCTTTACATTCAAGACCAGTCTCTTGTGAAACAAGAG
CCATATCATAATTGTACGGATTACGTACAAAAGGTAAATTCGTTTTACTCATTTTCTACGACCTTCCAAGGACCAAGGAAAAGACTTATTCTTTTCC
AAAAACTTATCAAAAATAGACTTACTACCTTTCTTAATATCGCGATACCAATATGGATCGCTAGACGGTGTTCGTAACTCTAAATTATTCTTAGCAT
CAATATCAGTCTTAATTGTTGATGCAGAATTAGCATTAACACGAGAACCAGCCTCGGCCTGTTCCAAATAAAAACGAGCCGGTTCTTGTAAAACA
CGCTGAGAAGCTAAATCAGCTTCTTCAGCAACCTTCCTAGCAGTCTGAACATTTAATTCACGTTGAGATTTAGCAACTTCCATCTGCTCACGCAAT
AAATCACTCTCAACATCACGCTTAACAGCTCCACTCAAATCAGACTTAACTGGAGAAGTAGAAGATGCAGTAGCACCACTCGGAACAGTAGAAC
CACCTTTAGAATATGCAAGCATAGGACTCAAACCAGCCTTATTCAAATCAGTAACCATACGCTGATATTGCGTATTGGACATATCTTCTTGAAAGC
GTCTATTTGCTTGCGCTTCAGCAGTATTATATTTTTGCTCTTCATTCTGACTCCAAGCAGAAGTAGCAAAATCAAATAAACCACCAGCAACTGAAT
CAAACATACCCATACTAACGCGCTCCGCTTGTTTGCTGACTACTGGTTTCCCAGTAGTCCAGCTTATATTACATTAGAAATGGTCAATCAAGCCAG
GTACAGAATACATCGGCATTGGACGAGCCATCTTACAATCAAAAAACGCATCCATCAAAAACTGCTGACCATTAGCAGATGCACCAACAGCAGT
AGTACGATCAATAGGTGGCGTTTCTTGAATAAACGTAGAATTCAAAGTCGGCAACGAAGTGAACTTCTGAGCATAATGCCAAGGGTCAATCGTA
CCAGCTGAAGTCGACTTAAACAAACCTGTAATCTGAGAAGGTTTGTAACGGTACTCTGCCCAACGTTCCTGGTATCCAAATACATCATCATCAGTT
GAAGTACCAGTAACATAGATTTCCTTATTAAGAACAGCCTGTTCACCTAAATGAGCAAATACAGGGAAATAGAAATCATAACGTGTCTCACGAGA
CCACATCTTAGGTAAACCTTGCTGATATGTTAGATCAGCTCTTACGTTTACCAAACCAATTATGTATCCATGTTCTTGAGCATGATACGTAAAACCA
TGTCCACTAGCCAACGCAGTACCCATTGCAGCCAAGTTACCAAGCGGAGTAGCACCGCCAGAAATCGAAGTAGCAGACGTTTGAGCAATAGGA

>000131F|arrow

ACCAACCTTATGAGTATGACGAATTACTTTACATGCGGGAAATAATGCTAAATTAATTCCGAAGACAATACACCAGAACGACTATCTGCAAAGAA
CAAGTCACTATGGCAAAACTTCAACTATTAAAACGTAACCTTAGGAAAATAATGAAACTTATCCTCGCTTCCGTAAAAGACCGTGCTGCTGAAGC
ATATGCACGACCAATGTTCGTACCTTCCTCTGGATAGCTATACGCTCTTTTTCAGATGAAATTATCGTTCTGATACTGAAAATCAACTCTTTAATCA
CCCTGATGATTCGATCTATATGAATTCGGAACATTTGACGATTCACTGGGTTATTCGATTTACATGAACAACCAAAACTCCTATCATTAGGAAAAC
AAGTTAAACTTAAATAAAACAACCGAGGGGAAAAGAGATTTATTTTCCCCCGGAACAACACTAAGGAAAAACATGCACCGCAATCAGTCAGTTA
ATACTCACCGCTTCGCGATGGTACCTAGAGCCGATATACCACGTAGTAAATTCGATGCTCAAAAACACTAAACGATTTCCGATGCGGGCTATCTA
TTCCTGTATATGTGTGATGAAGGCTCCCTGTGCCACTTTTCAACTTAAAAATGACCGCATTTGCCGTCTAGCAACGCCTTTATAGCCAAATCCTGG
ATAACATGATTATGGAGTCTTTCTTTTTCGTTGTACCCTCGCCTTATTGGAAACTCAAAATTTATTGGGCAACAGAAAATCCAACAGATTCAATATC
TTAAAATTGCCCAACACAAACAAGCCCAACAGATGGTATGCCGTAAGGCACCGTCAAGACTAGATGGGCTTACCAACAGTAGGCCAAATTAGAT
TACTGGCCGAACTATTACGCACTGTGCCTTTTGCCACGGCATACAATCTTATCTGAACGAATGGTTCCGAGATGAAAATTTACAAACAATCGCAG
TAGTTGAGTAAGGGCGATGGCCCTGATACTTCCTCAAACTATGTGCTAAAACGTCGTGGTAAAGACATGATTACTTTACGTCAGCATTACCATGG
CCACAAAAAGGTGCGAGTGTCACCTTACCTTTAGGTACTACGGCTCCAATTAAATGGGATACCATTTCAGGAGACGCAACATCAAACGATAAATT
TACGGTAATTCAAACAGATCCTGGAAAATACGACTGCTTTAGCTAGATATGGCAACGCTTATGGTGTTAATACTGCTGGTGTAGTAATAACGTTT
CTAATTTATATACCGACTTATCAGAAGCAACTGCTGCAACTGTCATCAATTAAGACAGTCATTTCAAATTCAAAATTACTTGAAGGGATGCACGTG
GCGGAACACGATACACAGAAATTATCCGGAGTCACTTTGGAGTTATTTCCCCAGACGCCCGTTTACAAAGGCCTGAATACCTTGGAGGCGGTTC
AACACCAATTAATGTTAATCCGATTGCTCAAACGTCGGGAACAAACGCTTCTGGAACGACTACCCCTTTGGGCAACCTTGCTGCTATGGTACTGC
TCTCGCTCATAATCATGGATTTACTCAATCATTTACTGAGCATGGCGTTATTATTGGATTAGTATCCATTAGAGCAGATCTTACTTATCAACAAGGA
TTAGACCGTATGTGGGTCTAGATCTACACGATATGACTTTTATTTCCCAGCATTTGCTACTCTAGGCGAACAATCTGTTTTGCAAAAAGAAATTTA
TGCAACAGGAGATACTGCAGCCGACAATACTGTTTTGGATATCAAGAACGCTGGGCGGAATATCGTTACAAACCATCTAAAATTACTGGTTTGTT
CAAATCAACATCGGCGGGCACGATCGATGGTTGGCATTTGGCTCAAAAATTTACCGCTGCGCCTACTTTGAATAATACGTTTATTCAAGATACGC
CTCCTGTATCACGTGTAGTAGCCGTTGGAGCAGCTGCAAATGGCCAACAATTCTTATTTGACTCATTTTTTGATGTCAAAATGGCAAGACCAATGC
CAATGTATTCAGTACCTGGCTTAATAGACCATTTCTAATGGGACTATTTGACGGAATTGCCGATTTAATCGGCCCTGCTATAGCTATAGGAGCTGC
CCCTGCTACTGGGGGACTCTCCTTAGCTGCACTTGCACCTGCAGCAATAGGTGCAGCAGGACAATACTTTGGAACACAAAGTCAAAACGCAGCG
AGTGCAGAACAAGCGAGTAATCAACAGAGATTTCAAGCTGAAATGTCTGGAACATCATATCAACGAGCAGTTGAAGATATGAAAAAAGCTGGG
TTAAATCCCATGCTTGCGTATTCACAAGGCGGAGCCACAACACCAGCTGGAGCTATGGCCCAGATGCAAAATGTTCTCGGTAATGCAACTACGTC
CGGAACCCAAGCTTATCAAACGGTTGCGCAAGCAAATCAAGCTATTGCTCAATCTAAACAAATTGAAGCTCAAACAGAACTCACAAGTAATCAAC
AGATAATGTACGTGCTGATACGTTAAATAAATTGGATGAAAATCCAAATATTAGAGCTCAATATAAACAAATACTTGCCGATACTTTCATGAAAA
ATGAAATAGGCAAAACATCAAGTGCTCAAGCTGCTCAAGCTTTGGCACAATCTCGTTATTCAAACGAGTTAACAAAACTTGCTAAATCAGGGTCA
GCTCCTAGTTCTAGCAAACCAATTTATCAAGACGTAAAAAACATCGCCAAAGATGCGTATAGCGCATCTGGCGCAAAACGATACATCGATAACTA
TCGAGGTCAACCGATTCAACAAAATCGTACAAATAACCAACCACCAATGGAATGAAAATGACAAAGATTACAGCCCCATTTCTTCGTACTCCGTA
CAATTACGACACGATTGCTGCGTCAAATGAGTCAGGGCTGCATTGTGAGGATGCAACTCTGACTCAGCAGCAATTTGCTGAAGAATGTGATATC
AATAATATTATGGAAAAGTTCGGTATGACCGGACTTATTCCTCAAACTCCTTTAACGCCTCAATATGGCGACTTTAGTGGTGTCTATGACTACCAC

TCTGCTCTGAACCAGATTATGGCTTCAGACAACGAATTTATGGCTTTACCAGCCAATATTCGTGAACGATTCGCTAATGATCCCGCGAATCTAATA
GATTTTCTAGAAAACCCTGAAAATCGCAGCGAAGCTGAAAAAATGGGACTGGTAAAACCAGCCCAAACCGAGGTTTCAACCCCTGTTGGAACCT
CGGAAGCACAGTTACCTACTTGATGTAACTGTGCTAGGTGACACCAAACCACAAAAACACGATAAACAAGGACAGAAAAAATGATGCGTCGCA
GACCAGCAAATAAGCAAAAGTCCGCTAGGACTTTCCGTAAACATGCTTCACATACAAAACACGCAAATATGCGAAACTCGCCAATGCGTGGAGG
CTGGAGACTCTAATAAAGTCTTCAGGCACCTCACATGCCTTGTTATCACCCTCTCAAAGCATATCAATGCTTTGACAAATCAATTGTTTTCGACGA
AGTTCGGAAACATGACATCGTTCGATCTTTAGACCTGCCCTGTGGGCAGTGCGTTGGATGCCGTCTAGAACGATCAAGACAATGGGCTATTCGG
TGCATGCACGAAGCCCAATTGCATAAAAACAACTCATTCATAACACTCACATATGACAATACACATCTCCCAAGCGATGGCTCTTTGGATCACAAA
GACTTTCAATTGTTCCTTAAAAGACTTAGAAAAACTCTCGCAAAAAGAGGACTTACAATCCGCTATTACATGGCTGGAGAATATGGTGAACTCTT
CGCAAGACCCCACTTCCATGCCTGTATCTTCGGATACGACTTTCCTGATAAAAAATTATGGAAAAGGACTGCCTCTGGTTCTATGTTATATAGATC
CGCAGAACTTGAAGCTCTCTGGCCATTTGGTTATACCACCATTGGAGATGTTACTTTTGAATCAGCCGCCTACGTGGCTAGATACATAATGAAAA
AACAAACAGGGAAAGATGCGGAATCTCATTACAAACGCATACACCCTGAAACCGGCGAATATTTAGACTTAAAGCCGGAATATAATAAAATGTC
TTTAAAACCGGAATCGGTAAAGACTTTTATATAAAATATACTTCGGATATATACCCGCAAGACTACGTAATACTTAGAGGTAAAAAGGTCAAACC
ACCAAAATACTATGACAAAATGTTTAAAATTG

>000218F|arrow

TGATAGCTTTTTTGATATTAAAAAAGCACGGCCAATGCCAATGTACTCTGTACCTGGCTTAATCGACACATTCTAATGGCACTAGAACCGCTGCCT
CAGCGCCGCATCTGGCGCCGCTTTTGGACCTTAACGGCTCCTTAATTGGAGCCGGAATAGGTCGGCCGCTAGTTATTTGTTGGTGTCAAGAACAA
AACGCTGCCAGCGCACAACAAGCTGCAGCATGATGCAATTCCAAGATGGTATGCGACGTACTGCATAGTCAAGACGCAGTAGCGGATCTTAGGC
TGCAGGTCTTAACCCTATGCTGGCTTATTCACAAGGCGAGCCAAAGTCAGCCTGGTGCGCAAGCTCCAGTAGGACAATCCACTAGGTGAGGCTG
GAAATTCAGCCCGTGAAGCTGCCATGGCAGTCGCCAATTTTAAACAATTACAAACTCAGAATATCCTGACACAATCGCAAGCCGAAAAAACCGG
ACGCGGATACAAATCTATCACGTGATCAGGCAACATATACTCGAGCAAATACAGCTCGTGAAATTGCTCAGGATGCCGGATACGGCAAATTTGG
TCAGCTTCGTGCGAGTGCCCAAATAGAGCATTAAGGACATCAAGTGCAGTTACAAGCTGCACAACAGCGACAAGCTTTAGTCAAGAGTGCATAT
ACAGGACCATTATAGCGATTACGCAAACTGATCAGCGCCATCCAGTACTAAACCAATTTATCAGAGTGTTACGGCTAATTTACATAGCCAATATG
ATAAATATCAAAAATATCTACCATTTGGGAAAAATGAAATGAAAACAATCAAACTTAGAACCGCATACACTATGAGCAGCGGATGGCTGCGTCA
AATGAGTCAGGGTTGGCTTGTGAGGAGCCAACTCTGGCTCAGCAGCATTATAAAGACGAATGCGATATAAATACTATCCTGGAACGTTTTAACG
TTACAGGCCTATTACCTCAAAGTCCGCTGCCGCCTCAATATGGCGATTTCAGCGGAATTACTGACTATCATAGCGCCTTGAATAAGGTAATGAAC
GCTATGGAAGAATTTGATAACTTACCGGCTCAAATTCGTGCTAGGTTCGAAAACGAACCAGCAAACCTGATTGAGTTCTTGCAAGACGAGAAAA
ATCGACCAGAAGCCGAGAAACTCGGCCTGGTCGAAAGAGCCATTTCGGAAGAAATGGCGATAAGCACAGTTACTCCACTTGATGTAACTGTGCT
AGGTGACACCAACACCAAAAATATCTGATAAACGAGGCCAAAAATTATGCTTTATAGAAAACAAACAAACAAGCGCAAAAGCGCTAAATCGTTC
CGTAGGAACACTTCAAAAACTAAAGCTGCAAATATGCAAAAAGCCCCGCAAAGAGGGGGGCTGGCGGCTCTAAATAAGCGCCAGGCTACCTCA
CATGGCCTGTTATCACCCACTGACTGCTTATTTAAGTAAGCATCAGACAAACTATAGACCGGCAAATCTTATCGCCGTGTCGCATTCAAAGAATCT
GACGAGCATGATCGTCAGATTTCACTGCCCTGCGGCCAATGCGTGGCTGCAGGCTAGAAAAATCACGTCAATGGGCCCATGCGTTAGCTGGCAT
GAAGCCCAATTGCACGAAAAAAAAACTGCTTTATAACCCTCACATACAACAATGAAAACCTGTCCACAAACTGGATCGCTGTCAAAAGCGACTTCC
AAAAGTTCCTTAAGCGCTTCAGAAAATCCATTGCACCTGCAAAATTACGTTACTACATGGCATGGAGAGAATACGGCACAAGTTTCGGCAGACCT
CAGCTTCCATGCCTGTATCTTCGGATACGATTTTCATGATAGAAAACTATTCAAAGACTCCCATCTGGTTTGCTCTCACTAATACATCCGACCCACC
TGGCAAACCCTCTGGCCCAACTATTGGTTATTTCCTCCATTGGAGGACGTTACATTCGAGTCAGCTGCTTACGTATGCTTCGATATATTATGCAAA
AATACAACTGGCCAGATGGAAGAAACAAACATATAACGAAAGGCATGAGCATTACACATACTGTGATATAGAATCCAGGGGAATTAATAAAGA
GCTCATTACCAGAATATAACCAATATGGGCCTAACCAGGCATTGGGCTGAGTGGTCACAAAAATAGCGTTTTCCGACGTATATCCCACATGACTA
CGTTTGAGTCAACGGAAAAAAAAAGGGTAAAAACCCCCAAAAAACTATGACAAAAAATATAAAATAGATTATCCATATGAATACCGAAGAATTAC
TCCACAAACGTGAAACTTCTGCTAACTCAACACGAAGACAATACCTATGCCAGACTTGCCGTAAAGGAAAAAGTCACAAAGGCCAAACTTCAATT
ATTAAAACGTAACCTCACTTAGAAATCCTCATGAAATTAGTACTCTGTACCGTTAAAGGACCGCGCAGCAGATGCGTTCGGTCGTCCAATGTTCG
TCCGTTCTATCGGCGAAGCAATCCGGAGCTTTAGCGACGAAGTCAATCGCCAGAGCGGATGACAATCAACTTTATAACCATTCCGACGATTTCGA
CCTATTTGATTAGGCGAATTCGACGATAATACGGGTTTGTTCCAATTACATGAACAACCCAAACTTGTATCCTTAGGGAAACAAGTCAAAATTACT
TGATAAAAACTAAGCGTAGAGGTTAAAAAGGGGGGAAACCCCTTTTTCTCACGCAACTAGGCCTAGGAGCTTCAAAAAAATGCATCGTAACAAG
TCGGTAGACGTCCATCAGTTCACAATGATTCCAAAAGCGGATATCCCCCGCTCTACATTTGACTGTCAATCAACACATAAAACCTACATTCGATGC
TGGCTTTCCTAGTCCCTGTACTCGTAGACGAATGTTTGCCAGGCGTACCATTCCGCTGCAACATGACCGCCTTTGCGCGATTGTCTACACCACTCT
ATCCGATCATGGATAACTATGCATCTGGATAGCTCTTCTTCTGTGCCAAATAGACTTGTCTGGTCAAATTGGCAAACAATTTATGGGGCAGCAGG
CAAATCCTGCGGACTCGATCTCGTACGTAGTGCCCCAACAAGTAACCCCAGCTGGTGGTTACGCTATTGGCAGCCTTCAAGATTATATGGGTCTG
CCAACCTGTAGGCCAAGTAGGTGCTGGTGGCACCGTAAGTCACTGTGCCTTCTGGCCACGTGCTTACAACCTTATTTATAACGAATGGTTTCGGG
ACGAAAACCTTCAAAATTCAGTAGTTGTAGATACTGGCGATGGTCCAGATAACGTAGCCAACTACACATTATTACGACGTGGAAAACGTAAAGA
CTATTTCACGTCAGCATTACCTTGGCCACAAAAGGGCGCAAGCGTTACTTTACCGCTTGGAACATCCGCCCCAATATTACGCACTAACAATGCGCC
TGTTTCCAGACTGTATAACGCTGGAACAAATACATTAAACGCAACCGCCCAGGCTCTTAAACGTAGGTGTTACTGGTCAAATTACTGGCGGTGCT

GACGGCTTGGCAAAATCATATGATCCTAATGGCGGTTTATATGCACGATTTATCAGCTGCAAACCGCCTGCATACAATTAATCAATTGCGTCAAA
GCTGTCCAGATTCAAAAACTTTTAGAAAGGGACGCCCGTGGCGGAACTCGATACACAGAAATTATCCGCAGCCATTTCGGGGTCGTTAGCCCCG
ATGCGCGTCTCCAACGGCCTGAATACATTGGAGGCGGTTCAACACACATTAATATCAATCCAATCGCCAGACGAATGGTACCGGAGCTTCCGGA
CCACTACTCCTCTCGGTACACTTGGCGCTATGGGTACTGGGCTCGCTCACAATCATGGCTTTAACTTATTCAAGCACTGAACATGGTGTAATTATC
GGTCTCGTTTCAGTACGAGCCGATTTAACATTACCAACAAGGTATGCACCGCATGTGGAATCGTTCCACACGTTATGATTTCTATTTCCCTGCTTC
GCCACTTTGGGCGAACAATGCAGTATTAAATGAAGAATAATCTACGTACGAGGCGATGCCAACGCTACAGGAGTGTTTGGATACCAAGAACGTT
GGGCAGAATATCGTTATATGCCAAGCCGAATTTTCCAGTCTGTTCCGTAGTACGGCAGCTGGAACAATGACGGCTGGCATTTAGCCCCAACGGTT
TACAACACTTCCAATTTGAATAACACGTTTTATTCAAGAAAATCCACCCTGTCTCTCGAACCCTTGCGGTCGGAGCAGCTGCCAACGGCAGCAAAT
CATTTT

>000072F|arrow

TATCTAAATGCAAATTGTCCATAACTGGAAACAAAGGCGTTGCCAAACGGGCAAACATTGTAGCCTTTACATTGTGCATGTCCCCTGGGAGCACT
TCATCACAATAAATAGGAACTAGATAACCACCATCAAAAGTAGTTTTATGCGCATATTGCGTATCAAAACTAGAACGCGGAATTTCCGCTTTAGG
AACCATAGCAAAACTATGTGAGCTTACTGACTTATTACGATGCATAACAATCTCCCGAAGTATTCCGAACCACTAGCAAGCTAGTGATCCGGCTT
AAAAAAAACATTACTCGCCATCGCGAATCATAACATCCTTAGCTCTAGAAATAAGCTTGGGAGAACCAAGCAAATCCATAGTACCAGAATTATCA
TCAAACGTACCAAAATAATATAACTGAAAATCATCAGGGTGTTTATATAACTGATTATCATCGCTAGCGCGATTAACTTCGTCCTGAAACTGACGA
ACAGCAACACCCTCAGATGCAACAAAAGCTGGACGACCATACGCACCAGCTGCAGTATCTAAAATAGAAACAATAACCATCTTCATAAAAACTCC
TTTAAATCTTACGTTTTAAAAGCGATAACTTAGCCAAAGCGACTTTTTCCTTTACAGCCAAACGCTCTAAAGTGTTATCTTCAAAATGTGAGCGAC
CTTCTAGCTCACGAGCATATTGTATACCATCAAATTCTTCTGGAAACAATACTTTAAACTTATTATCATAAAACCGTGGTGGTCGGCACTTTTTGCC
ACGCACCACAACAGAGTCAGTCGTATAAACGTCTGACATGAACTTATCAAACCAAGCCTGACCAATGCCAGGCTTAAGAGACATCTTATTAAATT
CAGGCTTACGCTGAATAATCTCACCAGTATCTAAATCACAATACTGATAATGCGCATCTGCATCAACCACTTCGTGGTTCTCATTAACGGTTTTACC
GTTAATCTTCTCATAATATATCGAGCAACATAAGCAGCAGATTCAAAATTGACATCACCAATAGAACTATAGCCATACGGCCAAAGTTCTTCAAGT
ATCTTTGACGTATATAAGATAGACCCAGTCTGCGTTCTTTTGAAAAACTTCTTATCTTCAAAATCAAGCCCAAAGATACAAGCATGGAAATGAGG
ACGATCGAAAGACTCACCATATTCACCTGCCATATAAAAACGAATAGTCTTGCCAGTATAGCGTTTTCTCAATCGTTTCATAAACAATTGAAAATC
TTCATAATGCAAAGACATATCCTTAGGACAATGCTCTGGAGCATATGTCAAAGTAATAAAACAATTACTAGTATGCATTTGTGCCTCATGCATACA
TCTAACGGCCCACTGACGGGACCGTTCAAGGCGACAACCAACACACTGACCACAAGGCAATGACAAGGTACGGACTACGTCCGCCCCTGGTATC
TCCCGCCAAATAATAGACTTGTCAGCGCATTGATAGAGCCGTTAACGGCTTATAACACGCCATAAATTACAGTCTAAAACCACCGCCGCTGCGGT
GAAGTACGCATATTAATGCTCTTGGTCTTGCTTGACGCCACGACCGAAACTTCTTAGCTGCGCCATGCTTGCTCATTGGTTTCTATAAAGGCTCA
TAACATTGCACTCCGTAGTTAATAATGTGGTTTTGTGTCACCTAGCACAGTTACATCAAGTAGAGTAACTGTGCTGCCATCCGCTTACGCGTCTGG
CTTAGGTGTTTCTACTGCAGAAAACGACGGGTTTCAACCACAGGTTTACCGTCAATAAGACCATCTGAATCGCTTCATCACGATTCTCTTCATTCT
GTAAATATTTAACAAAGCATTAGGATCGTTATCAAACTTAGCCCTAATCTTAGCTGGCAAAGCCATAAAAGCTCATCAGAGGCACGAATAGCATT
CAATGCGGTGTGATAGTCAGATACACCGCTAAAATCGCCATATGATGGCTCTAATGGCGCCTGGGGCAATTGCCCAGTAACGCCAAAACGCTCA
ACTATAATATTAATATCACATTCGTCTCTCATGTGTTGTTGAGCCAAACTCGGATCTTTACATTCAAGACCAGTCTCTTGTGAAACAAGAGCCATAT
CATAATTGTACGGATTACGTACAAAAGGTAAATTCGTTTTACTCATTTTCTACGACCTTCCAAGGACCAAGGAAAAGACTTATTCTTTTCCAAAAA
CTTATCAAAAATAGACTTACTACCTTTCTTAATATCGCGATACCAATATGGATCGCTAGACGGTGTTCGTAACTCTAAATTATTCTTAGCATCAATA
TCAGTCTTAATTGTTGATGCAGAATTAGCATTAACACGAGACCAGCCTCGGCCTGTTCCAAATAAAAACGAGCCGGTTCTTGTAAAACACGCTGA
AAAGCTAAATCAGCTTCTTCAGCAACCTTCCTAGCAGTCTGAACATTTAATTCACGTTGAGATTTAGCAACTTCCATCTGCTCACGCAATAATCACT
CTCAACATCACGCTTAACAGCTCCACTCAAATCAGACTTAACTGGAGAAGTAGAAGATGCAGTAGCACCACTCGGAACAGTAGAACCACCTTTAG
AATATGCAAGCATAGGACTCAAACCAGCCTTATTCAAATCAGTAACCATACGCTGATATTGCGTATTGGACATATCTTCTTGAAAGCGTCTATTTG
CTTGCGCTTCAGCAGTATTATATTTTTGCTCTTCATTCTGACTCCAAGCAGAAGTAGCAAAATCAAATAAACCACCAGCAACTGAATCAAACATAC
CCATACTAACGCGCTCCGCTTGTTTGCTGACTACTGGTTTCCCAGTAGTCCCAGCTTATATTACATTAGAAATGGTCAATCAAGCCAGGTACAGAA
TACATCGGCATTGGACGAGCCATCTTACAATCAAAAAACGCATCCATCAAAAACTGCTGACCATTAGCAGATGCACCAACAGCAGTAGTACGATC
AATAGGTGGCGTTTCTTGAATAAACGTAGAATTCAAGTCGGCAACGAAGTGAACTTCTGAGCATAATGCCAAGGGTCAATCGTACCAGCTGAAG
TCGACTTAACACAAACCTGTAATCTGAGAAGGTTTGTAACGGTACTCTGCCCAACGTTCCTGGTATCCAAATACATCATCATCAGTTGAAGTACCA
GTAACATAGATTTCCTTATTAAGAACAGCCTGTTCACCTAAATGAGCAAATACAGGGAAATAGAAATCATAACGTGTCTCACGAGACCACATCTT
AGGTAAACCTTGCTGATATGTTAGATCAGCTCTTACGTTTACCAAACCAATTATGTACCATGTTCTTGAGCATGATACGTAAAACCATGTCCACTA
GCCAACGCAGTACCCATTGCAGCCAAGTTACCAAGCGGAGTAGCACCGCCAGAAATCGAAGTAGCAGACGTTTGAGCAATAGGATTAACGTTG
ACATAAGTAGAACCACCACCAATATATTCAGGACGTTGTAAACGATAATCTTGTGGAGTTACACCAAATGAGCACGTAACAATTCTGTATAGCGT
GTACCACCTCGCGCATCGCGCTCTAACAAACGCTGAATCTGGAAAGACTGACGTAACTGGTTAATAGTTGCAGCCGTAGCATCACTTAAATCGGC
ATACAAACCAGTACCAGCAGTACCAGCGTTATTACTACGATACACAGCTGTGTAGTTGAATTAGCATAAATCTGCTTCAAAGCACCAGCACCGTC
AACTAATGACAACGTTGTTGAATCATTAGTAACAGACGTCTTAATAGGAGCAGACGTGCCTAAAGGCAACGTAACTGCATCGCCTTTCTGAGGCC
AAGGCAAGGCACCAGTAAAATAATCCTTACGCTTACCGCGTCGAACCATAGCATAATCACTCGGAGTATCAGGACCGTCACCCTTGTGAACGGT

AACAGAATTTTGTAAATTCTCATCCCTAAACCACTCGTTATAAATCAAATTATAAGCACGTAACGGTAACGCGTTATGCGTAACCGTATTAGTACC
AGTAATCTGACCAGCCGTAGGCAAACCAAAATGATCAAAAATAGAACCTACTGCATACCACCAGCAGTAGAAGTAATCTGTGGAACTACATACG
AAATAGAATCACCTGGGTTCGCTTGCTCACCCATAAACTTAACCCAATTGTTCCAAACTAATCTATTTGGAACAAAGAAAAAGAAAG

>000054F|arrow

ACTCGCCAATGCGTGGAGGCTGGAGACTCTAATAAAGTCTTCAGGCACCTCACATGCCTTGTTATCACCCTCTCAAAGCATATCAATGCTTTGACA
AATCAATTGTTTTCGACGAAGTTCGGAAACATGACATCGTTCGATCTTTAGACCTGCCCTGTGGGCAGTGCGTTGGATGCCGTCTAGAACGATCA
AGACAATGGGCTATTCGGTGCATGCACGAAGCCCAATTGCATAAAAACAAACTCATTCATAACACTCACATATGACAATACACATCTCCCAAGCG
ATGGCTCTTTGGATCACAAAGACTTTCAATTGTTCCTTAAAAGACTTAGAAAAACTCTCGCAAAAAGAGGACTTACAATCCGCTATTACATGGCTG
GAGAATATGGTGAACTCTTCGCAAGACCCCACTTCCATGCCTGTATCTTCGGATACGACTGTCCTGATAAAAAATTATGGAAAAGGACTGCCTCT
GGTTCTATGTTATATAGATCCGCAGAACTTGAAGCTCTCTGGCCATTTGGTTATACCACCATTGGAGATGTTACTTTTGAATCAGCCGCCTACGTG
GCTAGATACATAATGAAAAACAAACAGGGAAAGATGCGGAATCTCATTACAAACGCATACACCCTGAAACCGGCGAATATTTAGACTTAAAGCC
GGAATATAATAAAATGTCTTTAAAACCGGGAATCGGTAAAGACTTTTATATAAAATATACTTCGGATATATACCCGCAAGACTACGTAATACTTA
GAGGTAAAAAGGTCAAACCACCAAAATACTATGACAAAATGTTTAAAATTGACCAACCTTATGAGTATGACGAATTACTTTACATGCGGGAAAAT
AATGCCTAAATTAATTCCGAAGACAATACACCAGAACGACTATCTGCAAAAGAACAAGTCACTATGGCAAAACTTCAACTATTAAACGTACCTTA
CTTAGGAAAATAATGAAACTTATCCTCGCTTCCGTAAAGACCGTGCTGCTGAAGCATATGCACGACCAATGTTCGTACCTTCCTCTGGAGTAGCT
ATACGCTCTTTTCAGATGAAATTAATCGTTCTGATACTGAAAATCAACTCTTTAATCACCCTGATGATTTCGATCTATATGAATTCGGAACATTTGA
CGATTCAACTGGGTTATTCGATTTACATGAACAACCAAAACTCCTATCATTAGGAAAACAAGTTAAACTTAATAAAACAACCGAGGGGAAAAGA
GATTTATCTTTCCCCCGGAACAACACTAAGGAAAAACATGCACCGCAATCAGTCAGTTAATACTCACCGCTTCGCGATGGTACCTAGAGCCGATA
TACCACGTAGTAAATTCGATGCTCAAAAAACACATAAAACGACTTTCGATGCGGGCTATCTAATTCCTGTATATGTTGATGAAGTGCTCCCTGGG
GACACTTTCAACTTAAAAATGACGGCATTTGCCCGTCTAGCAACGCCTTTATATCCAATCATGGATAACATGATTATGGATTCTTTCTTTTTCTTTG
TACCCAATCGCCTTATATGGAATAACTGGCAAAAATTTATGGGTCAACAAGAAAATCCAACAGACTCAATATCTTATATTGTCCCAACACAAACAA
GCCCAACAGATGGTTATGCCGTAGGCAGCCTTCAAGACTATATGGGCTTACCAACAGTAGGCCAAATTGATACTGGCCGAACTATTACGCACTGT
GCCTTTTGGCCACGTGCATACAATCTTATCTGGAACGAATGGTTCCGAGATGAAAATTTACAAACAAGCGCAGTAGTTGATAAGGGCGATGGCC
CTGATACTTCCTCAAACTATGTGCTAAAACGTCGTGGTAAAAGACATGATTACTTTACGTCAGCATTACCATGGCCACAAAAAGGTGCGAGTGTC
ACCTTACCTTTAGGTACTACGGCTCCAATTAAATGGGATACCATTTCAGGAGACGCAACATCAAACGATAAATTTACGGTAATTCAAACAGATCCT
GGAAATACGACTGCTTTAGCTAGATATGGCAACGCTTATGGTGTTAATACTGCTGGTGTAGTAAATAACGTTTCTAATTTATATACCGACTTATCA
GAAGCAACTGCTGCAACTGTCAATCAATTAAGACAGTCATTTCAAATTCAAAAATTACTTGAAAGGGATGCACGTGGCGGAACACGATACACAG
AAATTATCCGGAGTCACTTTGGAGTTATTTCCCCAGACGCCCGTTTACAAAGGCCTGAATACCTTGGAGGCGGTTCAACACCAATTAATGTTAATC
CGATTGCTCAAACGTCGGGAACAAACGCTTCTGGAACGACTACCCCTTTGGGCAACCTTGCTGCTATGGGTACTGCTCTCGCTCATAATCATGGA
TTTACTCAATCATTTACTGAGCATGGCGTTATTATTGGATTAGTATCCATTAGAGCAGATCTTACTTATCAACAAGGATTAGACCGTATGTGGTCT
AGATCTACACGATATGACTTTTATTTCCCAGCATTTGCTACTCTAGGCGAACAATCTGTTTTGCAAAAAGAAATTTATGCAACAGGAGATACTGCA
GCCGACAATACTGTTTTTGGATATCAAGAACGCTGGGCGGAATATCGTTACAAACCATCTAAAATTACTGGTTTGTTCAAATCAACATCGGCGGG
CACGATCGATGGTTGGCATTTGGCTCAAAAATTTACCGCTGCGCCTACTTTGAATAATACGTTTATTCAAGATACGCCTCCTGTATCACGTGTAGT
AGCCGTTGGAGCAGCTGCAAATGGCCAACAATTCTTATTTGACTCATTTTTTGATGTCAAAATGGCAAGACCAATGCCAATGTATTCAGTACCTG
GCTTAATAGACCATTTCTAATGGGACTATTTGACGGAATTGCCGATTTAATCGGCCCTGCTATAGCTATAGGAGCTGCCCCTGCTACTGGGGGAC
TCTCCTTAGCTGCACTTGCACCTGCAGCAATAGGTGCAGCAGGACAATACTTTGGAACACAAAGTCAAAACGCAGCGAGTGCAGAACAAGCGAG
TAATCAACAGAGATTTCAAGCTGAAATGTCTGGAACATCATATCAACGAGCAGTTGAAGATATGAAAAAAGCTGGGTTAAATCCCATGCTTGCG
TATTCACAAGGCGGAGCCACAACACCAGCTGGAGCTATGGCCCAGATGCAAAATGTTCTCGGTAATGCAACTACGTCCGGAACCCAAGCTTATC
AAACGGTTGCGCAAGCAAATCAAGCTATTGCTCAATCTAAACAAATTGAAGCTCAAACAGAACTCACAAGTAATCAAACAGATAATGTACGTGCT
GATACGTTAAATAAATTGGATGAAAATCCAAATATTAGAGCTCAATATAAACAAATACTTGCCGATACTTTCATGAAAAATGAAATAGGCAAAAC
ATCAAGTGCTCAAGCTGCTCAAGCTTTGGCACAATCTCGTTATTCAAACGAGTTAACAAAACTTGCTAAATCAGGGTCAGCTCCTAGTTCTAGCAA
ACCAATTTATCAAGACGTAAAAAACATCGCCAAAGATGCGTATAGCGCATCTGGCGCAAAACGATACATCGATAACTATCGAGGTCAACCGATT
CAACAAAATCGTACAAATAACCAACCACCAATGGAATGAAAATGACAAAGATTACAGCCCCATTTCTTCGTACTCCGTACAATTACGACACGATT
GCTGCGTCAAATGAGTCAGGGCTGCATTGTGAGGATGCAACTCTGACTCAGCAGCAATTTGCTGAAGAATGTGATATCAATAATATTATGGAAA
AGTTCGGTATGACCGGACTTATTCCTCAAACTCCTTTAACGCCTCAATATGGCGACTTTAGTGGTGTCTATGACTACCACTCTGCTCTGAACCAGA
TTATGGCTTCAGACAACGAATTTATGGCTTTACCAGCCAATATTCGTGAACGATTCGCTAATGATCCCGCGAATCTAATAGATTTTCTAGAAAACC
CTGAAAATCGCAGCGAAGCTGAAAAAATGGGACTGGTAAAACCAGCCCAAACCGAGGTTTCAACCCCTGTTGGAACCTCGGAAGCACAGTTACC
TACTTGATGTAACTGTGCTAGGTGACACCAAACCACAAAAACACGATAAACAAGGACAGAAAAAATGATGCGTCGCAGACCAGCAAATAAGCA
AAAGTCCGCTAGGACTTTCCGTAAACATGCTTCACATACAAAACACGCAAATATGCGAA

>000103F|arrow

TGTAAACGATAATCCTGTGGAGTTACTCCAAAGTGAGCACGTAGTAACTCAGTATAACGAGTACCACCACGTGCATCGCGCTCCAATAACTTCTG
AATCTGGAAAGATGACGAAGTTGGTTAATAGTCGCAGCAGTAGCAGTAGACAAATCAGCGTATAACTGATTAGTAGCAACACCAGCATTTGTAC
TATTAGACAAAGTATTAGAAGCTGAATTTAATTCTCTTAAAGCACCAACAGCAGTTTGAAAAAACACTATAACCAGCAGTACCATTGCCAGCTGG
TATGTCAGAATAAATATTAGCACGTGTTCCTAACGGTAAAGAAACAGAAGCACCCTTCTGTGGCCAAGGCAATGCACCAGTAAAATAATCTTTAC
GCTTACCACGTCTCAAAAGTGTATAGTTAGCAGGACATCACCTGAATCACCAAGATTTAACGTTACAGAATTCTGTAAGTTTTCATCTCTAAACCA
TTCGTTATAAATTCATTATAAGCACGTAAACGGCAAACATTATGCGTTACTGTATTAGCGCCAGTAATCTGACCAGCAGTAGGTAAACCAAAATA
ATCAAAAATTGAACAAACAGCATAACCACCAGCAGGACTAGTAATAGTAGGCACTACAAAAGAAATAGAATCACCAGGATTCGTTGTTCACCCA
TAAACTTTGGCCAATTGTTCCAAAGTAAACGATTAGGTACAAAGAAAAGAAAGTATCAAGATGCAAGTGTCCATCACAGGAAATAACGGTGTGG
CCAAACGTGCAAATGCAGTCATCTTTACACGATGAGTATCGCCAGGCAATACTTCATCACAATAAATAGGAACTAAAAAACCAGCATAAACGTG
GTTTTATGAGCATATTGAGTATTAAAACTAGAGCGGGGAATGTCCGCTTTAGGAACCATAGCAAATGAATGCGTACTTACTGATTATTGCGAAAC
ATAAACATCTCCCGTAGTTCCGTACCATCTTTCGAGTGATACGGTATAAAAAAAACCTTACTCGCCTTCGCGAATCATTACATCTTTTGCACAATCA
ACTTAGGGCTTTCCAGTAGTTCAAAAACACAGTGGCATCGTCAAAAGACCCAAATAGAACATATGAAAATCATCAGGATGTTTATACAACTGATT
ATCTTCGCTAGCTCGATGACTTCATCCTGAAAATGACGTACTGCAACACCTTCAGATGCAACATAAGCTGGACGACCAAAAGCATCTGCAGCAGT
ATCCTTAATAGAAACAATAACCATCTTCATAAAAACTCTTAAATAGTACGTTTTAACAATGACAACTTAGCCAACGCAACTTTTTCCTTAACAGCAG
TCGCTCAAGCGTGTTGTCCTCATGCCTAGATCGACCTTCCATCTCTCTGGCAAACTGAATCATATCGAATTCTTCAGGAAACTTCAACTTAAATTTT
TATCAAAAACCGTGGTGGACGGCACTTTTTGCCACGCACCACAAACGTGGTCTGACGTATAAACGTCTGACATGTACTTATCTAACCACGATTGC
GATACCGGGCTTCAATGACATCTTATTAATTCTGGCTTACGCTGAATTATCTCACCAGTCTCTAAATCACAATATTGATAATGGGCACCCGCATCA
ACCACTTCGTGGTTTTCATGACAGTAACCCCATTAATCTTCTCATATATATCTTGCAACATAAGCAGCAGACTCAAAAGTAACATCACCAATTGTA
GAATAGCCAAACGGCCACAATTCTTCCAAAATCTCTGACGTGTAGAGGATAGAGCCAGTCTGCGTTCTTTTAAAATTTTCTTATCCGGAAAATCAA
GACCAAACAGACAAGCATGGAAATGAGGACGATCAAAGATCACCATATTCACCTGCCATATAAAAACGTATCGTTTTCCCAGTAAAACGCTTACG
TAACCGCTTCATAAAAAGCTGATAATCATTGTAATCCAATGACTATCCTTAGGACAATGCTCTGGAGCATATGTCAAAGTAATAAAACAATTACTA
GTATGCATTTGTGCCTCATGCATAAACGAATCGCCCACTGACGTGAGCGTTCAAGGCGACAACCAACACACTGACCACAAGGCAATGATAGGGT
ACGGACTACATCCGCACCCGGTATTTCCCGCCAAATTATAGACCTGTCACTGCATTGATAAGCCGTTAAGGGCTTATAACAGGCATAATTACAAA
CGATAGCCACCACGCTGGGGAGCGTGTCTCTATAATTGACTTCGTCTTACTAGCAGTTCTGCGAAATGACTTTGCAGATTTATATTTGTTTACTGG
CTTTTTCGTAACATGATGAACTCCGTAGTTAAAATAGTGGTTTGGTGTCACCTAGCACAGTTACATCAAGTAGAGTAACTGTGCTGGCCTCAGGA
TTTCATCTTCGGCCTTAGGTGTTTCTGTAGAAACGATGGGTTCAACCACAGGTTGTCCATCAATAAGACCAATTGAATCGCTTCATCACGATTCTG
GTCGTTCTCAAGGAACTCCAATAATTTGACAGGATCATGGTCAAATCGGACTCTTAATTTCGCTGGCAGAGCCATGAAACGTCCTAGTTGCGTTA
ATTTGATTCAACGCAGAATGGTAATCAGTAACACACTAAAATCGCCGTATTGAGGCGATACAGGGGCGTTGGAAGTTCCCCTGTAACCCCGAAA
CGTTCAATGATGACATTAATGTCACATTCATCTTTCATATGCTGTTGAGCAAGACTTGGGTTTGACACAGCAACGCATCGTTTACGATGCTTCATC
TTATCATAGTTGTACGGATTACGTACAAATGGCAAATTCGCTTATCATTATTTGACTCCAATTCCCAAGGGGTTAGTTGATTAGGATTATTTTGAG
TTTATCAAAAATTTCTTTCGAAGCATCGATACCCTAGACCAAATAGCTGGGGATGGAGCCAATAGCCTCTTAGTATTATATGACTGAGCGGATGT
TAAATCCGCAGTCGTATTTTTAAAGCAATATCAGCCAAAATACGTTTATTTTCGGCTGTAATATTAGGAGCAGTAAGCAACTTATTACACAGTATC
AGCCCTAGTATTAGCGGTACCAGCTTCAGTAGCTTCAGTCTGGGCGATAATCATTTTCTGTTTCTGATGCATTACGAATCTGTGCATCATAGAAGC
AGTATTAATAGCTGAATTAGTTGCATTACCTAAAACATTTTCCATAGTAGCAGTGAACCAGATGGGGTAGAAGCACCACCACGTTGATAGGCTAA
CATAGGAGATAAACCAGCAGCTTCTAAATCTTTAACAGCACGCTGGTAACTGTTCCACTCATATCGGCTTGAAAATCCATTTGCCTCTGAGCCATA
TCCTGATTAGCTTTATTAGCAGAAGTAACCTAAATAAGAACCAACAGCGCCCAAGCAGTACCGACACCAGGAGTAAAGAAACTAGAAGCTGAAG
ACAGCTTAGAACAACATTAGTAACCGCATCAAGTATTCCACCAAACATAAACTAACGCCCTTCGGTTGTTTCCTCACTATCCTACGGAGTAGTCGA
GGTTATATAAAACATTAGAAATGATCAATTAGACAGGTACGCTGTACATAGGCATAGGTCTGGCCATCTGACAATCAAAGAAAGCATCCATTAA
AAATTGCTGACCATTTGCTGCAGCTCGACCGCCGCAGTACGGCAACTGGAGGGGTCTCTTGAATAAACGTTGAATTCAACGTAGGAAGAGAAGT
AAATCGCTGTCCATAATGCCAAGCATCAATAGTGCCAGCAGAAGTAGACCTAAAGAAAACCAGTAATTGAGAAGGTTTGTAACGATACTCCGCC
CAACGTTCTTGATACCAAATACATCATTGTCGGCTGCAGTACCTTGAACATAAATTTCTTTGTTCAAAATAGCTTGCTCACCCAAAGTGGCAAATA
CTGGGAAATAAAAGTCATATCGTGTAGACCTAGACCACATCTTAGGAAGACCTGTGATATGTGAGGTCAGCACGTACAGAAAACTAATCCGATA
ATGTATCCATGTTCTTGAGCAGCATACGTAAAGCCGTGTCCCTGAGCCAATGCAGTACCCATTGCAGCAAGGTTACCTTGCGGAGTAGCAGAACC
AGTAACCGACGTTGCAGAAGTCTGAGCAATCGGATTAACATTAACAAGGGTCGAACCTCCACCAATATATTCAGGACGT

>000074F|arrow

CATATCTAGCTAAAGCAGTCGTATTTCCAGGATCTGTTTGAATTACCGTAAATTTATCGTTTGATGTTGCGTCTCCTGAAATGGTATCCCATTTAAT
TGGAGCCGTAGTACCTAAAGGTAAGGTGACACTCGCACCTTTTTGTGGCCATGGTAATGCTGACGTAAAGTAATCATGTCTTTTACCACGACGTT
TTAGCACATAGTTTGAGGAAGTATCAGGGCCATCGCCCTTATCAACTACTGCGCTTGTTTGTAAATTTTCATCTCGGAACCATTCGTTCCAGATAA
GATTGTATGCACGTGGCCAAAAGGCACAGTGCGTAATAGTTCGGCCAGTATCAATTTGGCCTACTGTTGGTAAGCCCATATAGTCTTGAAGGCT
GCCTACGGCATAACCATCTGTTGGGCTTGTTTGTGTTGGGACAATATAAGATATTGAGTCTGTTGGATTTTCTTGTTGACCCATAAATTTTTGCCA
GTTATTCCATATAAGGCGATTGGGTACAAAGAAAAAGAAAGAATCCATAATCATGTTATCCATGATTGGATATAAAGGCGTTGCTAGACGGGCA

AATGCCGTCATTTTTAAGTTGAAAGTGTCCCCAGGGAGCACTTCATCAACATATACAGGAATTAGATAGCCCGCATCGAAAGTCGTTTTATGTGT
TTTTTGAGCATCGAATTTACTACGTGGTATATCGGCTCTAGGTACCATCGCGAAGCGGTGAGTATTAACTGACTGATTGCGGTGCATGTTTTTCCT
TAGTGTTGTTCCGGGGGAAAGATAAATCTCTTTTCCCCTCGGTTGTTTTATTTAAGTTTAACTTGTTTTCCTAATGATAGGAGTTTTGGTTGTTCAT
GTAAATCGAATAACCCAGTTGAATCGTCAAATGTTCCGAATTCATATAGATCGAAATCATCAGGGTGATTAAAGAGTTGATTTTCAGTATCAGAA
CGATTAATTTCATCTGAAAAAGAGCGTATAGCTACTCCAGAGGAAGGTACGAACATTGGTCGTGCATATGCTTCAGCAGCACGGTCTTTTACGGA
AGCGAGGATAAGTTTCATTATTTTCCTAAGTAAGGTTACGTTTTAATAGTTGAAGTTTTGCCATAGTGACTTGTTCTTTTGCAGATAGTCGTTCTG
GTGTATTGTCTTCGGAATTAAATTTAGCATTATTTTCCCGCATGTAAAGTAATTCGTCATACTCATAAGGTTGGTCAATTTTAAACATTTTGTCATA
GTATTTTGGTGGTTTGACCTTTTTACCTCTAAGTATTACGTAGTCTTGCGGGTATATATCCGAAGTATATTTTATATAAAAGTCTTTACCGATTCCC
GGTTTTAAAGACATTTTATTATATTCCGGCTTTAAGTCTAAATATTCGCCGGTTTCAGGGTGTATGCGTTTGTAATGAGATTCCGCATCTTTCCCTG
TTTGTTTTTTCATTATGTATCTAGCCACGTAGGCGGCTGATTCAAAAGTAACATCTCCAATGGTGGTATAACCAAATGGCCAGAGAGCTTCAAGTT
CTGCGGATCTATATAACATAGAACCAGAGGCAGTCCTTTTCCATAATTTTTTATCAGGAAAGTCGTATCCGAAGATACAGGCATGGAAGTGGGGT
CTTGCGAAGAGTTCACCATATTCTCCAGCCATGTAATAGCGGATTGTAAGTCCTCTTTTTGCGAGAGTTTTTCTAAGTCTTTTAAGGAACAATTGA
AAGTCTTTGTGATCCAAAGAGCCATCGCTTGGGAGATGTGTATTGTCATATGTGAGTGTTATGAATGAGTTGTTTTTATGCAATTGGGCTTCGTG
CATGCACCGAATAGCCCATTGTCTTGATCGTTCTAGACGGCATCCAACGCACTGCCCACAGGGCAGGTCTAAAGATCGAACGATGTCATGTTTCC
GAACTTCGTCGAAAACAATTGATTTGTCAAAGCATTGATATGCTTTGAGAGGGTGATAACAAGGCATGTGAGGTGCCTGAAGACTTTATTAGAG
TCTCCAGCCTCCACGCATTGGCGAGTTTCGCATATTTGCGTGTTTTGTATGTGAAGCATGTTTACGGAAAGTCCTAGCGGACTTTTGCTTATTTGC
TGGTCTGCGACGCATCATTTTTTCTGTCCTTGTTTATCGTGTTTTTGTGGTTTGGTGTCACCTAGCACAGTTACATCAAGTAGGTAACTGTGCTTCC
GAGGTTCCAACAGGGGTTGAAACCTCGGTTTGGGCTGGTTTTACCAGTCCCATTTTTTCAGCTTCGCTGCGATTTTCAGGGTTTTCTAGAAAATCT
ATTAGATTCGCGGGATCATTAGCGAATCGTTCACGAATATTGGCTGGTAAAGCCATAAATTCGTTGTCTGAAGCCATAATCTGGTTCAGAGCAGA
GTGGTAGTCATAGACACCACTAAAGTCGCCATATTGAGGCGTTAAAGGAGTTTGAGGAATAAGTCCGGTCATACCGAACTTTTCCATAATATTAT
TGATATCACATTCTTCAGCAAATTGCTGCTGAGTCAGAGTTGCATCCTCACAATGCAGCCCTGACTCATTTGACGCAGCAATCGTGTCGTAATTGT
ACGGAGTACGAAGAAATGGGGCTGTAATCTTTGTCATTTTCATTCCATTGGTGGTTGGTTATTTGTACGATTTTGTTGAATCGGTTGACCTCGATA
GTTATCGATGTATCGTTTTGCGCCAGATGCGCTATACGCATCTTTGGCGATGTTTTTTACGTCTTGATAAATTGGTTTGCTAGAACTAGGAGCTGA
CCCTGATTTAGCAAGTTTTGTTAACTCGTTTGAATAACGAGATTGTGCCAAAGCTTGAGCAGCTTGAGCACTTGATGTTTTGCCTATTTCATTTTTC
ATGAAAGTATCGGCAAGTATTTGTTTATATTGAGCTCTAATATTTGGATTTTCATCCAATTTATTTAACGTATCAGCACGTACATTATCTGTTTGAT
TACTTGTGAGTTCTGTTTGAGCTTCAATTTGTTTAGATTGAGCAATAGCTTGATTTGCTTGCGCAACCGTTTGATAAGCTTGGGTTCCGGACGTAG
TTGCATTACCGAGAACATTTTGCATCTGGGCCATAGCTCCAGCTGGTGTTGTGGCTCCGCCTTGTGAATACGCAAGCATGGGATTTAACCCAGCT
TTTTTCATATCTTCAACTGCTCGTTGATATGATGTTCCAGACATTTCAGCTTGAAATCTCTGTTGATTACTCGCTTGTTCTGCACTCGCTGCGTTTTG
ACTTTGTGTTCCAAAGTATTGTCCTGCTGCACCTATTGCTGCAGGTGCAAGTGCAGCTAAGGAGAGTCCCCCAGTAGCAGGGGCAGCTCCTATAG
CTATAGCAGGGCCGATTAAATCGGCAATTCCGTCAAATAGTCCCATTAGAAATGGTCTATTAAGCCAGGTACTGAATACATTGGCATTGGTCTTG
CCATTTTGACATCAAAAAATGAGTCAAATAAGAATTGTTGGCCATTTGCAGCTGCTCCAACGGCTACTACACGTGATACAGGAGGCGTATCTTGA
ATAAACGTATTATTCAAAGTAGGCGCAGCGGTAAATTTTTGAGCCAAATGCCAACCATCGATCGTGCCCGCCGATGTTGATTTGAACAAACCAGT
AATTTTAGATGGTTTGTAACGATATTCCGCCCAGCGTTCTTGATATCCAAAAACAGTATTGTCGGCTGCAGTATCTCCTGTTGCATAAATTTCTTTT
TGCAAAACAGATTGTTCGCCTAGAGTAGCAAATGCTGGGAAATAAAAGTCATATCGTGTAGATCTAGACCACATACGGTCTAATCCTTGTTGATA
AGTAAGATCTGCTCTAATGGATACTAATCCAATAATAACGCCATGCTCAGTAAATGATTGAGTAAATCCATGATTATGAGCGAGAGCAGTACCCA
TAGCAGCAAGGTTGCCCAAAGGGGTAGTCGTTCCAGAAGCGTTTGTTCCCGACGTTTGAGCAATCGGATTAACATTAATTGGTGTTGAACCGCCT
CCAAGGTATTCAGGCCTTTGTAAACGGGCGTCTGGGGAAATAACTCCAAAGTGACTCCGGATAATTTCTGTGTATCGTGTTCCGCCACGTGCATC
CCTTTCAAGTAATTTTTGAATTTGAAATGACTGTCTTAATTGATTGACAGTTGCAGCAGTTGCTTCTGATAAGTCGGTATATAAATTAGAAACGTT
ATTTACTACACCAGCAGTATTAACACCATAAGCGTTGC

>000163F|arrow

GAAGTTTTGCCATAGTTACTTGTTCTTTTGCAGATAGTCGTTCTGGTGTATTGTCTTCGGAATTAAGTTTAGCGTTATTTTCCCGCATGTAAAGTAA
TTCGTCATACTCATAAGGTTGGTCAATTTTAAACATTTTGTCATAGTATTTTGGTGGTTTGACCTTTTTACCTCTAAGTATTACGTAGTCTTGCGGG
TATATATCCGAAGTATATTTTATATAAAAGTCTTTACCGATTCCCGGTTTTAAAGACATTTATTATATTCCGGCTTTAAGTCTAAATATTCGCCGGTT
TCAGGGTGTATGCGTTTGTAATGAGATTCCGCATCTTTCCCTGTTTGTTTTTTCATTATGTATCTAGCCACGTAGGCGGCTGATTCGAAAGTAACA
TCTCCAATGGTGGTATAACCAAATGGCCAGAGAGCTTCAAGTTCTGCGGATCTATATAACATAGAACCAGAGGCAGTCCTTTTCCATAATTTTTTA
TCAGGAAAGTCGTATCCGAAGATACAGGCATGGAAGTGGGGTCTTGCGAAGAGTTCACCATATTCTCCAGCCATGTAATAGCGGATTGTAAGTC
CTCTTTTTGCGAGAGTTTTTCTAAGTCTTTTAAGGAACAGTTGAAAGTCTTTGTGATCCAAAGAGCCATCGCTTGGGAGATGTGTATTGTCATATG
TGAGTGTTATGAATGAGTTGTTTTTATGCAATTGGGCTTCGTGCATGCACCGAATAGCCCATTGTCTTGATCGTTCTAGACGGCATCCAACGCACT
GCCCACAGGGCAGGTCTAAAGATCGAACGATGTCATGTTTCCGAACTTCGTCGAAAACAATTGATTTGTCAAAGCATTGAAATGCTTTGAGAGG
GTGATAACAAGGCATGTGAGGTGCCTGGGGACTTATTAGAGTCTCCAGCCTCCACGCATTGGCGAGTTTCGCATATTTGCGTGTTTTGTATGTGA
AGCATGTTTACGGAAAGTCCTAGCGGACTTTTGCTTATTTGCTGGTCTGCGACGCATCATTTTTTCTGTCCTTGTTTATCGTGTTTTTGTGGTTTGG

TGTCACCTAGCACAGTTACATCAAGTAGGTAACTGTGCTTCCGAGGTTCCAACAGGGGTTGAAACCTCGGTTTGGGCTGGTTTTACCAGTCCCAT
TTTTTCAGCTTCGCTGCGATTTTCAGGGTTTTCTAGAAAATCTATTAGATTCGCGGGATCATTAGCGAATCGTTCACGAATATTGGCTGGTAAAGC
CATAAATTCGTTGTCTGAAGCCATAATCTGGTTCAGAGCAGAGTGGTAGTCATAGACACCACTAAAGTCGCCATATTGAGGCGTTAAAGGAGTTT
GAGGAATAAGTCCGGTCATACCAAACTTTTCCATAATATTATTAATATCGCATTCTTCAGCAAATTGCTGCTGAGTCAGAGTTGCATCCTCACAAT
GCAGCCCTGACTCATTTGACGCAGCAATCGTGTCGTAATTGTACGGAGTACGAAGAAATGGGGCTGTAATCTTTGTCATTTTCATTCCATTGGTG
GTTGGTTATTTGTACGATTTTGTTGAATCGGTTGACCTCGATAGTTATCGATGTATCGTTTTGCGCCAGATGCGCTATACGCATCTTTGGCGATGT
TTTTTACGTCTTGATAAATTGGTTTGCTAGAACTAGGAGCTGACCCTGATTTAGCAAGTTTTGTTAACTCGTTTGAATAACGAGATTGTGCCAAAG
CTTGAGCAGCTTGAGCACTTGATGTTTTGCCTATTTCATTTTTCATGAAAGTATCGGCAAGTATTTGTTTATATTGAGCTCTAATATTTGGATTTTC
ATCCAATTTGTTTAACGTATCAGCACGTACATTATCTGTTTGATTACTTGTGAGTTCTGTTTGAGCTTCAATTTGTTTAGATTGAGCAATAGCTTGA
TTTGCTTGAGCAACCGTTTGATAAGCTTGGGTTCCGGACGTAGTTGCATTACCGAGAACATTTTGCATCTGGGCCATAGCTCCAGCTGGTGTTGT
GGCTCCGCCTTGTGAATACGCAAGCATGGGATTTAACCCAGCTTTTTTCATATCTTCAACTGCTCGTTGATATGATGTTCCAGACATTTCAGCTTG
AAATCTCTGTTGATTACTCGCTTGTTCTGCACTCGCTGCGTTTTGACTTTGTGTTCCAAAGTATTGTCCTGCTGCACCTATTGCTGCAGGTGCAAGT
GCAGCTAAGGAGAGTCCCCCAGTAGCAGGGGCAGCTCCTATAGCTATAGCAGGGCCGATTAAATCGGCAATTCCGTCAAATAGTCCCATTAGAA
ATGGTCTATTAAGCCAGGTACTGAATACATTGGCATTGGTCTTGCCATTTTGACATCAAAAAATGAGTCAAATAAGAATTGTTGGCCATTTGCAG
CTGCTCCAACGGCTACTACACGTGATACAGGAGGCGTATCTTGAATAAACGTATTATTCAAAGTAGGCGCAGCGGTAAATTTTTGAGCCAAATGC
CAACCATCGATCGTGCCCGCCGATGTTGATTTGAACAAACCAGTAATTTTAGATGGTTTGTAACGATATTCCGCCCAGCGTTCTTGATATCCAAAA
ACAGTATTGTCGGCTGCAGTATCTCCTGTTGCATAAATTTCTTTTTGCAAAACAGATTGTTCGCCTAGAGTAGCAAATGCTGGGAAATAAAAGTC
ATATCGTGTAGATCTAGACCACATACGGTCTAATCCTTGTTGATAAGTAAGATCTGCTCTAATGGATACTAATCCAATAATAACGCCATGCTCAGT
AAATGATTGAGTAAATCCATGATTATGAGCGAGAGCAGTACCCATAGCAGCAAGGTTGCCCAAAGGGGTAGTCGTTCCAGAAGCGTTTGTTCCC
GACGTTTGAGCAATCGGATTAACATTAATTGGTGTTGAACCGCCTCCAAGGTATTCAGGCCTTTGTAAACGGGCGTCTGGGGAAATAACTCCAA
AGTGACTCCGGATAATTTCTGTGTATCGTGTTCCGCCACGTGCATCCCTTTCAAGTAATTTTTGAATTTGAAATGACTGTCTTAATTGATTGACAGT
TGCAGCAGTTGCTTCTGATAAGTCGGTATATAAATTAGAAACGTTATTTACTACACCAGCAGTATTAACACCATAAGCGTTGCCATATCTAGCTAA
AGCAGTCGTATTTCCAGGATCTGTTTGAATTACCGTAAATTTATCGTTTGATGTTGCGTCTCCTGAAATGGTATCCCATTTAATTGGAGCCGTAGT
ACCTAAAGGTAAGGTGACACTCGCACCTTTTTGTGGCCATGGTAATGCTGACGTAAAGTAATCATGTCTTTTACCACGACGTTTTAGCACATAGTT
TGAGGAAGTATCAGGGCCATCGCCCTTATCAACTACTGCGCTTGTTTGTAAATTTTCATCTCGGAACCATTCGTTCCAAATAAGATTGTATGCACG
TGGCCAAAAGGCACAGTGCGTAATAGTTCGGCCAGTATCAATTTGGCCTACTGTTGGTAAGCCCATATAGTCTTGAAGGCTGCCTACGGCATAAC
CATCTGTTGGGCTTGTTTGAGTTGGGACAATATAAGATATTGAGTCTGTTGGATTTTCTTGTTGACCCATAAATTTTTGCCAGTTATTCCATATAAG
GCGATTGGGTACAAAGAAAAAGAAAGAATCCATAATCATGTTGTCCATGATTGGATATAAAGGCGTTGCTAGACGGGCAAATGCCGTCATTTTT
AAGTTGAAAGTGTCCCCAGGGAGCACTTCATCAACATATACAGGAATTAAATAGCCCGCATCGAAAGTCGTTTTATGTGTTTTTTGAGCATCGAA
TTTACTACGTGGTATATCGGCTCTAGGTACCATCGCGAAGCGAGTGAGTATTAACTGACTGATTGCGGTGCATGTTTTTCCTTAGTGTTGTTCCGG
GGGAAAGATAAATCTCTTTTCCCCTCGGTTGTTTTATTTAAGTTTAACTTGTTTTCCTAATGATAGGAGTTTTGGTTGTTCATGTAAATCGAATAAC
CCAGTTGAATCGTCAAAAGTTCCGAATTCATATAGATCGAAGTCATCAGGGTGATTAAAAAGTTGATTTTCAGTATCAGAACGATTAATTTCATCT
GAAAAAGAGCGTATAGCTACTCCAGAGGAAGGTACGAACATTGGTCGTGCATATGCTTCAGCAGCACGGTCTTTTACGGAAGCGAGGATAAGT
TTCATTATTTTCCTAAGTGAGGTTACGTTTTAATAGTT

>000090F|arrow

GGTAACTGTGCTTCCGAGGTTCCAACAGGGGTTGAAACCTCGGTTTGGGCTGGTTTTACCAGTCCCATTTTTTCAGCTTCGCTGCGATTTTCAGGG
TTTTCTAGAAAATCTATTAGATTCGCGGGATCATTAGCGAATCGTTCACGAATATTGGCTGGTAAAGCCATAAATTCGTTGTCTGAAGCCATAATC
TGGTTCAGAGCAGAGTGGTAGTCATAGACACCACTAAAGTCGCCATATTGAGGCGTTAAAGGAGTTTGAGGAATAAGTCCGGTCATACCGAACT
TTTCCATAATATTAGATATCACATTCTTCAGCAAATTGCTGCTGAGTCAGAGTTGCATCCTCACAATGCAGCCCTGACTCATTTGACGCAGCAATC
GTGTCGTAATTGTACGGAGTACGAAGAAATGGGGCTGTAATCTTTGTCATTTTCATTCCATTGGTGGTTGGTTATTTGTACGATTTTGTTGAATCG
GTTGACCTCGATAGTTATCGATGTATCGTTTTGCGCCAGATGCGCTATACGCATCTTTGGCGATGTTTTTTACGTCTTGATAAATTGGTTTGCTAG
AACTAGGAGCTGACCCTGATTTAGCAAGTTTTGTTAACTCGTTTGAATAACGAGATTGTGCCAAAGCTTGAGCAGCTTGAGCACTTGATGTTTTG
CCTATTTCATTTTTCATGAAAGTATCGGCAAGTATTTGTTTATATTGAGCTCTAATATTTGGATTTTCATCCAATTTATTTAACGTATCAGCACGTAC
ATTATCTGTTTGATTACTTGTGAGTTCTGTTTGAGCTTCAATTTGTTTAGATTGAGCAATAGCTTGATTTGCTTGCGCAACCGTTTGATAAGCTTGG
GTTCCGGACGTAGTTGCATTACCGAGAACATTTTGCATCTGGGCCATAGCTCCAGCTGGTGTTGTGGCTCCGCCTTGTGAATACGCAAGCATGGG
ATTTAACCCAGCTTTTTTCATATCTTCAACTGCTCGTTGATATGATGTTCCAGACATTTCAGCTTGAAATCTCTGTTGATTACTCGCTTGTTCTGCAC
TCGCTGCGTTTTGACTTTGTGTTCCAAAGTATTGTCCTGCTGCACCTATTGCTGCAGGTGCAAGTGCAGCTAAGGAGAGTCCCCCAGTAGCAGGG
GCAGCTCCTATAGCTATAGCAGGGCCGATTAAATCGGCAATTCCGTCAAATAGTCCCATTAGAAATGGTCTATTAAGCCAGGTACTGAATACATT
GGCATTGGTCTTGCCATTTTGACATCAAAAAATGAGTCAAATAAGAATTGTTGGCCATTTGCAGCTGCTCCAACGGCTACTACACGTGATACAGG
AGGCGTATCTTGAATAAACGTATTATTCAAAGTAGGCGCAGCGGTAAATTTTTGAGCCAAATGCCAACCATCGATCGTGCCCGCCGATGTTGATT
TGAACAAACCAGTAATTTTAGATGGTTTGTAACGATATTCCGCCCAGCGTTCTTGATATCCAAAAACAGTATTGTCGGCTGCAGTATCTTGTTGCA

TAAATTTCTTTTTGCAAAACAGATTGTTCGCCTAGAGTAGCAAATGCTGGGAAATAAAAGTCATATCGTGTAGATCTAGACCACATACGGTCTAA
TCCTTGTTGATAAGTAAGATCTGCTCTAATGGATACTAATCCAATAATAACGCCATGCTCAGTAAATGATTGAGTAAATCCATGATTATGAGCGA
GAGCAGTACCCATAGCAGCAAGGTTGCCCAAAGGGGTAGTCGTTCCAGAAGCGTTTGTTCCCGACGTTTGAGCAATCGGATTAACATTAATTGG
TGTTGAACCGCCTCCAAGGTATTCAGGCCTTTGTAAACGGGCGTCTGGGGAAATAACTCCAAAGTGACTCCGGATAATTTCTGTGTATCGTGTTC
CGCCACGTGCATCCCTTTCAAGTAATTTTGAATTTGAAATGACTGTCTTAATTGATTGACAGTTGCAGCAGTTGCTTCTGATAAGTCGGTATATAA
ATTAGAAACGTTATTTACTACACCAGCAGTATTAACACCATAAGCGTTGCCATATCTAGCTAAAGCAGTCGTATTTCCAGGATCTGTTTGAATTAC
CGTAAATTTATCGTTTGATGTTGCGTCTCCTGAAATGGTATCCCATTTAATTGGAGCCGTAGTACCTAAAGGTAAGGTGACACTCGCACCTTTTTG
TGGCCATGGTAATGCTGACGTAAAGTAATCATGTCTTTTACCACGACGTTTTAGCACATAGTTTGAGGAAGTATCAGGGCCATCGCCCTTATCAA
CTACTGCGCTTGTTTGTAAATTTTCATCTCGGAACCATTCGTTCCAGATAAGATTGTATGCACGTGGCCAAAAGGCACAGTGCGTAATAGTTCGG
CCAGTATCAATTTGGCCTACTGTTGGTAAGCCCATATAGTCTTGAAGGCTGCCTACGGCATAACCATCTGTTGGGCTTGTTTGTGTTGGGACAAT
ATAAGATATTGAGTCTGTTGGATTTTCTTGTTGACCCATAAATTTTTGCCAGTTATTCCATATAAGGCGATTGGGTACAAAGAAAAAGAAAGAAT
CCATAATCATGTTATCCATGATTGGATATAAAGGCGTTGCTAGACGGGCAAATGCCGTCATTTTTAAGTTGAAAGTGTCCCCAGGGAGCACTTCA
TCAACATATACAGGAATTAGATAGCCCGCATCGAAAGTCGTTTTATGTTTTTTGAGCATCGAATTTACTACGTGGTATATCGGCTCTAGGTACCAT
CGCGAAGCGGTGAGTATTAACTGACTGATTGCGGTGCATGTTTTTCCTTAGTGTTGTTCCGGGGGAAAGATAAATCTCTTTTCCCCTCGGTTGTTT
TATTTAAGTTTAACTTGTTTTCCTAATGATAGGAGTTTTGGTTGTTCATGTAAATCGAATAACCCAGTTGAATCGTCAAATGTTCCGAATTCATATA
GATCGAATCATCAGGGTGATTAAAGAGTTGATTTTCAGTATCAGAACGATTAATTTCATCTGAAAAAGAGCGTATAGCTACTCCAGAGGAAGGT
ACGAACATTGGTCGTGCATATGCTTCAGCAGCACGGTCTTTTACGGAAGCGAGGATAAGTTTCATTATTTTCCTAAGTAAGGTTACGTTTTAATAG
TTGAAGTTTTGCCATAGTGACTTGTTCTTTTGCAGATAGTCGTTCTGGTGTATTGTCTTCGGAATTAATTTAGCATTATTTTCCCGCATGTAAAGTA
ATTCGTCATACTCATAAGGTTGGTCAATTTTAAACATTTTGTCATAGTATTTGGTGGTTTGACCTTTTTACCTCTAAGTATTACGTAGTCTTGCGGG
TATATATCCGAAGTATTTTATATAAAAGTCTTTACCGATTCCCGGTTTTAAAGACATTTTATTATATTCCGGCTTTAAGTCTAAATATTCGCCGGTTT
CAGGGTGTATGCGTTTGTAATGAGATTCCGCATCTTTCCCTGTTTGTTTTTTCATTATGTATCTAGCCACGTAGGCGGCTGATTCAAAAGTAACAT
CTCCAATGGTGGTATAACCAAATGGCCAGAGAGCTTCAAGTTCTGCGGATCTATATAACATAGAACCAGAGGCAGTCCTTTTCCATAATTTTTTAT
CAGGAAAGTCGTATCCGAAGATACAGGCATGGAAGTGGGGTCTTGCGAAGAGTTCACCATATTCTCCAGCCATGTAATAGCGGATTGTAAGTCC
TCTTTTTGCGAGAGTTTTTCTAAGTCTTTTAAGGAACAATTGAAAGTCTTTGTGATCCAAAGAGCCATCGCTTGGGAGATGTGTATTGTCATATGT
GAGTGTTATGAATGAGTTGTTTTTATGCAATTGGGCTTCGTGCATGCACCGAATAGCCCATTGTCTTGATCGTTCTAGACGGCATCCAACGCACT
GCCCACAGGGCAGGTCTAAAGATCGAACGATGTCATGTTTCCGAACTTCGTCGAAAACAATTGATTTGTCAAAGCATTGATATGCTTTGAGAGG
GTGATAACAAGGCATGTGAGGTGCCTGAAGACTTTATTAGAGTCTCCAGCCTCCACGCATTGGCGAGTTTCGCATATTTGCGTGTTTTGTATGTG
AAGCATGTTTACGGAAAGTCCTAGCGGACTTTTGCTTATTTGCTGGTCTGCGACGCATCATTTTTTCTGTCCTTGTTTATCGTGTTTTTGTGGTTTG
GTGTCACCTAGCACAGTTACATCAAGTA

>000110F|arrow

CATATGATGGCTCTAATGGCGCCTGGGGCAATTGCCCAGTAACGCCAAAACGCTCAACTATAATATTAATATCACATTCGTCTCTCATGTGTTGTT
GAGCCAAACTCGGATCTTTACATTCAAGACCAGTCTCTTGTGAAACAAGAGCCATATCATAATTGTACGGATTACGTACAAAAGGTAAATTCGTT
TTACTCATTTTCTACGACCTTCCAAGGACCAAGGAAAAGACTTATTCTTTTCCAAAAACTTATCAAAAATAGACTTACTACCTTTCTTAATATCGCG
ATACCAATATGGATCGCTAGACGGTGTTCGTAACTCTAAATTATTCTTAGCATCAATATCAGTCTTAATTGTTGATGCAGAATTAGCATTAACACG
AGAACCAGCCTCGGCCTGTTCCAAATAAAAACGAGCCGGTTCTTGTAAAACACGCTGAGAAGCTAAATCAGCTTCTTCAGCAACCTTCCTAGCAG
TCTGAACATTTAATTCACGTTGAGATTTAGCAACTTCCATCTGCTCACGCAATAAATCACTCTCAACATCACGCTTAACAGCTCCACTCAAATCAGA
CTTAACTGGAGAAGTAGAAGATGCAGTAGCACCACTCGGAACAGTAGAACCACCTTTAGAATATGCAAGCATAGGACTCAAACCAGCCTTATTC
AAATCAGTAACCATACGCTGATATTGCGTATTGGACATATCTTCTTGAAAGCGTCTATTTGCTTGCGCTTCAGCAGTATTATATTTTTGCTCTTCAT
TCTGACTCCAAGCAGAAGTAGCAAAATCAAATAAACCACCAGCAACTGAATCAAACATACCCATACTAACGCGCTCCGCTTGTTTGCTGACTACT
GGTTTCCCAGTAGTCCAGCTTATATTACATTAGAAATGGTCAATCAAGCCAGGTACAGAATACATCGGCATTGGACGAGCCATCTTACAATCAAA
AAACGCATCCATCAAAAACTGCTGACCATTAGCAGATGCACCAACAGCAGTAGTACGATCAATAGGTGGCGTTTCTTGAATAAACGTAGAATTCA
AAGTCGGCAACGAAGTGAACTTCTGAGCATAATGCCAAGGGTCAATCGTACCAGCTGAAGTCGACTTAAACAAACCTGTAATCTGAGAAGGTTT
GTAACGGTACTCTGCCCAACGTTCCTGGTATCCAAATACATCATCATCAGTTGAAGTACCAGTAACATAGATTTCCTTATTAAGAACAGCCTGTTC
ACCTAAATGAGCAAATACAGGGAAATAGAAATCATAACGTGTCTCACGAGACCACATCTTAGGTAAACCTTGCTGATATGTTAGATCAGCTCTTA
CGTTTACCAAACCAATTATGTATCCATGTTCTTGAGCATGATACGTAAAACCATGTCCACTAGCCAACGCAGTACCCATTGCAGCCAAGTTACCAA
GCGGAGTAGCACCGCCAGAAATCGAAGTAGCAGACGTTTGAGCAATAGGATTAACGTTGACATAAGTAGAACCACCACCAATATATTCAGGAC
GTTGTAAACGATAATCTTGTGGAGTTACACCAAAATGAGCACGTAACAATTCTGTATAGCGTGTACCACCTCGCGCATCGCGCTCTAACAAACGC
TGAATCTGGAAAGACTGACGTAACTGGTTAATAGTTGCAGCCGTAGCATCACTTAAATCGGCATACAAACCAGTACCAGCAGTACCAGCGTTATT
ACTACGATACACAGCATGTGTAGTTGAATTAGCATAAATCTGCTTCAAAGCACCAGCACCGTCAACTAATGACAACGTTGTTGAATCATTAGTAA
CAGACGTCTTAATAGGAGCAGACGTGCCTAAAGGCAACGTAACTGCATCGCCTTTCTGAGGCCAAGGCAAGGCACCAGTAAAATAATCCTTACG
CTTACCGCGTCGAACCATAGCATAATCACTCGGAGTATCAGGACCGTCACCCTTGTGAACGGTAACAGAATTTTGTAAATTCTCATCCCTAAACCA

CTCGTTATAAATCAAATTATAAGCACGTAACGGTAACGCGTTATGCGTAACCGTATTAGTACCAGTAATCTGACCAGCCGTAGGCAAACCAAAAT
GATCAAAAATAGAACCTACTGCATAACCACCAGCAGTAGAAGTAATCTGTGGAACTACATACGAAATAGAATCACCTGGGTTCGCTTGCTCACCC
ATAAACTTAACCCAATTGTTCCAAACTAATCTATTTGGAACAAAGAAAAAGAAAGTATCTAAATGCAAATTGTCCATAACTGGAAACAAAGGCGT
TGCCAAACGGGCAAACATTGTAGCCTTTACATTGTGCATGTCCCCTGGGAGCACTTCATCACAATAAATAGGAACTAGATAACCACCATCAAAAG
TAGTTTTATGCGCATATTGCGTATCAAAACTAGAACGCGGAATTTCCGCTTTAGGAACCATAGCAAAACTATGTGAGCTTACTGACTTATTACGAT
GCATAACAATCTCCCGAAGTATTCCGAACCACTAGCAAGCTAGTGATCCGGCTTAAAAAAAACATTACTCGCCATCGCGAATCATAACATCCTTA
GCTCTAGAAATAAGCTTGGGAGAACCAAGCAAATCCATAGTACCAGAATTATCATCAAACGTACCAAAATAATATAACTGAAAATCATCAGGGT
GTTTATATAACTGATTATCATCGCTAGCGCGATTAACTTCGTCCTGAAACTGACGAACAGCAACACCCTCAGATGCAACAAAAGCTGGACGACCA
TACGCACCAGCTGCAGTATCTAAAATAGAAACAATAACCATCTTCATAAAAACTCCTTTAAATCTTACGTTTTAAAAGCGATAACTTAGCCAAAGC
GACTTTTTCCTTTACAGCCAAACGCTCTAAAGTGTTATCTTCAAAATGTGAGCGACCTTCTAGCTCACGAGCATATTGTATACCATCAAATTCTTCT
GGAAACAATACTTTAAACTTATTATCATAAAACCGTGGTGGTCGGCACTTTTTGCCACGCACCACAACAGAGTCAGTCGTATAAACGTCTGACAT
GAACTTATCAAACCAAGCCTGACCAATGCCAGGCTTAAGAGACATCTTATTAAATTCAGGCTTACGCTGAATAATCTCACCAGTATCTAAATCACA
ATACTGATAATGCGCATCTGCATCAACCACTTCGTGGTTCTCATTAACGGTTTTACCGTTAATCTTCTTCATAATATATCGAGCAACATAAGCAGCA
GATTCAAAATTGACATCACCAATAGAACTATAGCCATACGGCCAAAGTTCTTCAAGTATCTTTGACGTATATAAGATAGACCCAGTCTGCGTTCTT
TTGAAAAACTTCTTATCTTCAAAATCAAGCCCAAAGATACAAGCATGGAAATGAGGACGATCGAAAGACTCACCATATTCACCTGCCATATAAAA
ACGAATAGTCTTGCCAGTATAGCGTTTTCTCAATCGTTTCATAAACAATTGAAAATCTTCATAATGCAAAGACATATCCTTAGGACAATGCTCTGG
AGCATATGTCAAAGTAATAAAACAATTACTAGTATGCATTTGTGCCTCATGCATACATCTAACGGCCCACTGACGGGACCGTTCAAGGCGACAAC
CAACACACTGACCACAAGGCAATGACAAGGTACGGACTACGTCCGCCCCTGGTATCTCCCGCCAAATAATAGACTTGTCAGCGCATTGATAAGCC
GTTAACGGCTTATAACACGCCATAAATTACAGTCTAAAACCACCGCGCTGCGGTGAAGTACGCATATTAATGCTCTTGGTCTTGCTTACGCCACG
ACGAAACTTCTTAGCTGCGCCATGCTTGCTCATTGGTTTTCTATAAAGGCTCATAACATTGCACTCCGTAGTTAATAATGTGGTTTTGGTGTCACCT
AGCACAGTTACATCAAGTAGAGTAACTGTGCTGCCATCCGCTTACGCGTCTGGCTTAGGTGTTTCTACTGCAGAAACGACGGGTTCAACCACAGG
TTTACCGTCAATAAGACCAATCTGAATCGCTTCATCACGATTCTCTTCATTCTGTAAATAATTTAACAAAGCATTAGGATCGTTATCAAACTTAGCC
CTAATCTTAGCTGGCAAAGCCATAAAAGCCTCATCAGAGGCACGAATAGCATTCAATGCGGTGTGATAGTCAGATACACCGCTAAAATCGC

>000202F|arrow

CTCGGAAGCACAGTTACCTACTTGATGTAACTGTGCTAGGTGACACCAAACCACAAAAACACGATAAACAAGGACAGAAAAAATGCGTCGCAGA
CCAGCAAATAAGCAAAAGTCCGCTAGGACTTTCCGTAAACATGCTTCACATACAAAACACGCAAATATGCGAAACTCGCCAATGCGTGGAGGCT
GGAGACTCTAATAAAGTCTTCAGGCACCTCACATGCCTTGTTATCACCCTCTCAAAGCATATCAATGCTTTGACAAATCAATTGTTTTCGACGAAG
TTCGGAAACATGACATCGTTCGATCTTTAGACCTGCCCTGTGGGCAGTGCGTTGGATGCCGTCTAGAACGATCAAGACAATGGGCTATTCGGTG
CATGCACGAAGCCCAATTGCATAAAAACAACTCATTCATACACTCACATATGACAATACACATCTCCCAAGCGATGGCTCTTTGGATCACAAAGA
CTTTCAATTGTTCCTTAAAAGACTTAGAAAAACTCTCGCAAAAGAGGACTTACAATCCGCTATTACATGCTGGAGAATATGGTGAACTCTTCGCAA
GACCCCACTTCCATGCCTGTATCTTCGGATACGACTTTCCTGATAAAAATTATGGAAAAGGACTGCCTCTGGTTCTATGTTATATAGATCCGCAGA
ACTTGAAGCTCTCTGGCCATTTGGTTATACCACCATTGGAGATGTTACTTTTGAATCAGCCGCCTACGTGGCTAGATACATAATGAAAAAACAAA
CAGGGAAAGATGCGGAATCTCATTACAAACGCATACACCCTGAAACCGGCGAATATTTAGACTTAAAGCCGGAATATAATAAAATGTCTTTAAA
ACCGGGAATCGGTAAAGACTTTTATATAAATATACTTCGGATATACCCGCAAGACTACGTAATACTTAGAGGTAAAAGGTCAAACCACCAAAAC
TATGACAAAATGTTTAAAATTGACCAACCTTATGATATGACGAATTACTTTACATGCGGGAAAATAATGCTAAATTAATTCCGAAGACAATACACC
AGAACGACTATCTGCAAAAGAACAAGTCACTATGGCAAAACTTCAACTATTAAAACGTAACCTTACTTAGGAAAATAATGAAACTTATCCTCGCT
TCCGTAAAAGACCGTGCTGCTGAAGCATATGCACGACCAATGTTCGTACCTTCCTCTGGAGTAGCTATACGCTCTTTTTCAGATGAAATTAATCGT
TCTGATACTGAAAATCAACTCTTTAATCACCCTGATGATTTCGATCTATATGAATTCGGAACATTTGACGATTCAACTGGGTTATTCGATTTACATG
AACAACCAAAACTCCTATCATTAGGAAAACAAGTTAAACTTAAATAAAACAACCGAGGGGAAAAGAGATTTATCTTTCCCCCGGAACAACACTAA
GGAAAAACATGCACCGCAATCAGTCAGTTAATACTCACCGCTTCGCGATGGTACCTAGAGCCGATATACCACGTAGTAAATTCGATGCTCAAAAA
ACACATAAAACGACTTTCGATGCGGGCTATCTAATTCCTGTATATGTTGATGAAGTGCTCCCTGGGGACACTTTCAACTTAAAAATGACGGCATTT
GCCCGTCTAGCAACGCCTTTATATCCAATCATGGATAACATGATTATGGATTCTTTCTTTTTCTTTGTACCCAATCGCCTTATATGGAATAACTGGC
AAAAATTTATGGGTCAACAAGAAAATCCAACAGACTCAATATCTTATATTGTCCCAACACAAACAAGCCCAACAGATGGTTATGCCGTAGGCAGC
CTTCAAGACTATATGGGCTTACCAACAGTAGGCCAAATTGATACTGGCCGAACTATTACGCACTGTGCCTTTTGGCCACGTGCATACAATCTTATC
TGGAACGAATGGTTCCGAGATGAAAATTTACAAACAAGCGCAGTAGTTGATAAGGGCGATGGCCCTGATACTTCCTCAAACTATGTGCTAAAAC
GTCGTGGTAAAAGACATGATTACTTTACGTCAGCATTACCATGGCCACAAAAAGGTGCGAGTGTCACCTTACCTTTAGGTACTACGGCTCCAATT
AAATGGGATACCATTTCAGGAGACGCAACATCAAACGATAAATTTACGGTAATTCAAACAGATCCTGGAAATACGACTGCTTTAGCTAGATATG
GCAACGCTTATGGTGTTAATACTGCTGGTGTAGTAAATAACGTTTCTAATTTATATACCGACTTATCAGAAGCAACTGCTGCAACTGTCAATCAAT
TAAGACAGTCATTTCAAATTCAAAATTACTTGAAAGGGATGCACGTGGCGGAACACGATACACAGAAATTATCCGGAGTCACTTTGGAGTTATTT
CCCCAGACGCCCGTTTACAAAGGCCTGAATACCTTGGAGGCGGTTCAACACCAATTAATGTTAATCCGATTGCTCAAACGTCGGGAACAAACGCT
TCTGGAACGACTACCCCTTTGGGCAACCTTGCTGCTATGGGTACTGCTCTCGCTCATAATCATGGATTTACTCAATCATTTACTGAGCATGGCGTT

ATTATTGGATTAGTATCCATTAGAGCAGATCTTACTTATCAACAAGGATTAGACCGTATGTGGTCTAGATCTACACGATATGACTTTTATTTCCCA
GCATTTGCTACTCTAGGCGAACAATCTGTTTTGCAAAAAGAAATTTATGCAACAGGAGATACTGCAGCCGACAATACTGTTTTTGGATATCAAGA
ACGCTGGGCGGAATATCGTTACAAACCATCTAAAATTACTGGTTTGTTCAAATCAACATCGGCGGGCACGATCGATGGTTGGCATTTGGCTCAAA
AATTTACCGCTGCGCCTACTTTGAATAATACGTTTATTCAAGATACGCCTCCTGTATCACGTGTAGTAGCCGTTGGAGCAGCTGCAAATGGCCAAC
AATTCTTATTTGACTCATTTTTTGATGTCAAAATGGCAAGACCAATGCCAATGTATTCAGTACCTGGCTTAATAGACCATTTCTAATGGGACTATTT
GACGGAATTGCCGATTTAATCGGCCCTGCTATAGCTATAGGAGCTGCCCCTGCTACTGGGGGACTCTCCTTAGCTGCACTTGCACCTGCAGCAAT
AGTGCAGCAGGACAATACTTTGGAACACAACAAGTCAAACGCAGCGAGTGCAGAACAAGCGAGTAATCAACAGAGATTTCAAGCTGAAATGTC
TGGAACATCATATCAACGAGCAGTTGAAGATATGAAAAAAGCTGGGTTAAATCCCATGCTTGCGTATTCACAAGGCGGAGCCACAACACCAGCT
GGAGCTATGGCCCAGATGCAAAATGTTCTCGGTAATGCAACTACGTCCGGAACCCAAGCTTATCAAACGGTTGCGCAAGCAAATCAAGCTATTG
CTCAATCTAAACAAATTGAAGCTCAAACAGAACTCACAAGTAATCAAACAGATAATGTACGTGCTGATACGTTAAATAAATTGGATGAAAATCCA
AATATTAGAGCTCAATATAAACAAATACTTGCCGATACTTTCATGAAAAATGAAATAGGCAAAACATCAAGTGCTCAAGCTGCTCAAGCTTTGGC
ACAATCTCGTTATTCAAACGAGTTAACAAAACTTGCTAAATCAGGGTCAGCTCCTAGTTCTAGCAAACCAATTTATCAAGACGTAAAAAACATCGC
CAAAGATGCGTATAGCGCATCTGGCGCAAAACGATACATCGATAACTATCGAGGTCAACCGATTCAACAAAATCGTACAAATAACCAACCACCA
ATGGAATGAAAATGACAAAGATTACAGCCCCATTTCTTCGTACTCCGTACAATTACGACACGATTGCTGCGTCAAATGAGTCAGGGCTGCATTGT
GAGGATGCAACTCTGACTCAGCAGCAATTTGCTGAAGAATGTGATATCAATAATATTATGGAAAAGTTCGGTATGACCGGACTTATTCCTCAAAC
TCCTTTAACGCCTCAATATGGCGACTTTAGTGGTGTCTATGACTACCACTCTGCTCTGAACCAGATTATGGCTTCAGACAACGAATTTATGGCTTT
ACCAGCCAATATTCGTGAACGATTCGCTAATGATCCCGCGAATCTAATAGATTTTCTAGAAAACCCTGAAAATCGCAGCGAAGCTGAAAAAATGG
GACTGGTAAAACCAGCCCAAACCGAGGTTTCAACCCCTGTTGGAAC

>000120F|arrow

GGAACTTCCAACGGCCCCTGTATCGCCTCAATACGGCGATTTTAGTGGTGTTACTGATTACCATTCTGCGTTGAATCAAATTAACGCAACTATGGA
CGATTTCATGGCTCTGCCAGCGAAATTAAGAGTCCGATTTGACCATGATCCTGTCAAATTATTGGAGTTCCTTGAGAACGACCAGAATCGTGATG
AAGCGATTCAATTGGGTCTTATTGATGGACAACCTGTGGTTGAACCCATCGTTTCTACAGAAACACCTAAGGCCGAAGGATGAAATCCTGAGGC
CAGCACAGTTACTCTACTTGATGTAACTGTGCTAGGTGACACCAAACCACTATTTTAACTACGGAGTTCATCATGTTACGAAGAAAGCCAGTAAA
CAAATATAAATCTGCAAAGTCATTTCGCAGAACTGCTAGTAAGACGAAGTCATTAATATGAGACACGCTCCCCAGCGTGGTGGCTATCGTTTGTA
ATTATGGCCTGTTATAAGCCCTTAACGGCTTATATCATGCAGTGACAGTCTATAATTTGGCGGGAAATACCGGGTGCGGATGTAGTCCGTACCCT
ATCATTGCCTTGTGGTCAGTGTTTGGTTGTCGCCTTGAACGCTCACGTCAGTGGGCGATTCGTTGTATGCATGAGGCACAAATGCATACTAGTAA
TTGTTTTATTACTTTGACATATGCTCCAGAGCATTGTCCTAAGGATATGTCATTGGATTACAATGATTATCAGCTTTTTATGAAGCGGTTACGTAAG
CGTTTTACTGGAAAACGATACGTTTTTATATGGCAGGTGAATATGGTGAATCTTTTGATCGTCCTCATTTCCATGCTTGTCTGTTTGGTCTTGATTT
TCCGGATAAGAAAATATTTAAAAGAACGCAGACTGGCTCTATCCTCTACACGTCAGAGATTTTGGAAGAATTGTGGCCGTTTGGCTATTCTACAA
TTGGTGATGTTACTTTTGAGTCTGCTGCTTATGTTGCAAGATATATTATGAAGAATTAATGGGGTTACTGTCAAAACCACGAAGTGGTTGATGCG
GGTGCCCATTATCAATATTGTGATTTAGAGACTGGTGAGATAATTCAGCGTAAGCCAGAATTTAATAAGATGTCATTGAAGCCCGGTATCGGGC
AATCGTGGTTAGATAAGTACATGTCAGACGTTTATACGTCAGACCACGTTTGGTGCGTGGCAAAAAGTGCCGTCCACCACGGTTTTATGATAATA
AATTTAAGTTGAAGTTTCCTGAAGAATTCGATATGATTCAGTTTGCCAGAGAGATGGAAGGTCGATCTAGGCATGAGGACAACACGCTTGAGCG
ACTTGCTGTTAAGGAAAAAGTTGCGTTGGCTAAGTTGTCATTGTTAAAACGTACTATTTAAGGAGTTTTTATGAAGATGGTTATTGTTTCTATTAA
GGATACTGCTGCAGATGCTTTTGGTCGTCCAGCTTATGTTGCATCTGAAGGTGTTGCAGTACGTCAGTTTCAGGATGAAGTCAATCGAGCTAGCG
AAGATAATCAGTTGTATAAACATCCTGATGATTTTCATATGTTCTATTTGGGTCTTTTTGACGATGCCACTGGTGTTTTTGAACTACTGGAAAGCCC
TAAGTTGATTGCTCGTGCAAAAGATGTAATGATTCGCGAAGGCGAGTAAGGTTTTTTTTATACCGTATCACTCGAAAGAGTGGTACGGAACTAC
GGAGATGTTTATGTTTCGCAATAAGTCAGTAAGTACGCATTCATTTGCTATGGTTCCTAAAGCGGACATTCCCCGCTCTAGTTTTAATACTCATAT
GCTCATAAAACGTTTGATGCTGGTTTTTTAGTTCCTATTTATTGTGATGAAGTATTGCCTGGCGATACTCATCGTGTAAAGATGACTGCATTTGCA
CGTTTGGCCACACCGTTATTTCCTGTGATGGACAACTTGCATCTTGATACTTTCTTTTTCTTTGTACCTAATCGTTTACTTTGGAACAATTGGCCAAA
GTTTATGGGTGAACAAACGAATCCTGGTGATTCTATTTCTTTTGTAGTGCCTACTATTACTAGTCCTGCTGGTGGTTATGCTGTTTGTTCAATTTTT
GATTATTTTGGTTTACCTACTGCTGGTCAGATTACTGGCGCTAATACAGTAACGCATAATGTTTTGCCGTTACGTGCTTATAATGAGATTTATAAC
GAATGGTTTAGAGATGAAAACTTACAGAATTCTGTAACGTTAAATCTTGGTGATTCAGGTGATGTTCCTGCTAACTATACACTTTTGAGACGTGG
TAAGCGTAAAGATTATTTTACTGGTGCATTGCCTTGGCCACAGAAGGGTGCTTCTGTTTCTTTACCGTTAGGAACACGTGCTAATATTTATTCTGA
CATACCAGCTGGCAATGGTACTGCTGGTTATAGTGTTTTTCAAACTGCTGTTGGTGCTTTAAGAGAATTAAATTCAGCTTCTAATACTTTGTCTAAT
AGTACAAATGCTGGTGTTGCTACTAATCAGTTATACGCTGATTTGTCTACTGCTACTGCTGCGACTATTAACCAACTTCGTCAATCTTTCCAGATTC
AGAAGTTATTGGAGCGCGATGCACGTGGTGGTACTCGTTATACTGAGTTACTACGTGCTCACTTTGGAGTAACTCCACAGGATTATCGTTTACAA
CGTCCTGAATATATTGGTGGAGGTTCGACCCTTGTTAATGTTAATCCGATTGCTCAGACTTCTGCAACGTCGGTTACTGGTTCTGCTACTCCGCAA
GGTAACCTTGCTGCAATGGGTACTGCATTGGCTCAGGGACACGGCTTTACGTATGCTGCTCAAGAACATGGATACATTATCGGATTAGTTTCTGT
ACGTGCTGACCTCACATATCAACAGGGTCTTCCTAAGATGTGGTCTAGGTCTACACGATATGACTTTTATTTCCCAGTATTTGCCACTTTGGGTGA
GCAAGCTATTTTGAACAAAGAAATTTATGTTCAAGGTACTGCAGCCGACAATGATGTATTTGGTTATCAAGAACGTTGGGCGGAGTATCGTTACA

AACCTTCTCAAATTACTGGTTTCTTTAGGTCTACTTCTGCTGGCACTATTGATGCTTGGCATTATGGACAGCGATTTACTTCTCTTCCTACGTTGAAT
TCAACGTTTATTCAAGAGACCCCTCCAGTTGCTCGTACTACGGCGGTCGGAGCTGCAGCAAATGGTCAGCAATTTTTAATGGATGCTTTCTTTGAT
TGTCAGATGGCCAGACCTATGCCTATGTACAGCGTACCTGGTCTAATTGATCATTTCTAATGTTTATATAACCTCGACTACTCCGTAAGGTAGTG
AGGAAACAACCGAAGGGCGTTAGTTTATGTTTGGTGGAATACTTGATGCGGTTACTAATGTTGGTTCTAAGCTGTCTTCAGCTTCTAGTTTCTTTA
CTCCTGGTGTCGGTACTGCTTTGGGCGCTGTTGGTTCTTATTTAGGTTCTACTTCTGCTAATAAAGCTAATCAGGAGATGGCTCAGAGGCAAATG
GATTTTCAAGCCGATATGAGTGGAACAAGTTACCAGCGTGCTGTTAAAGATTTAGAAGCTGCTGGTTTATCTCCTATGTTAGCCTATCAACGTGG
TGGTGCTTCTACCCCATCTGGTTCAACTGCTACTATGGAAAATGTTTTAGGTAATGCAACTAATTCAGCTATTAATACTGCTTCTATGATGCAACA
GATTCGTAATGCATCAGAAACAGAAAAGCATTATCGCCCAGACTGAAGCTACTGAAGCTGGTACCGCTAATACTAGGGCTGATACTGTTAATAA
GTTGCTTACTGCTCCTAATATTACAGCCGAAAATAAACGTATTTTGGCTGATATTGCTTTAAAGAATACGACTGCGGATTTAACATCCGCTCAGTC
ATATAATACTAAGAGGCTATTGGCTCCATCCCCAGCTATTTGGTCTAGGGTATCGATGCTTCGAAAGAAATTTTTGATAAACTCAAAAATAATCCT
AATCAACTAACCCCTTGGGGAATTGGAGTCAAATAATGAGTAAAGCGAATTTGCCATTTGTACGTAATCCGTACAACTATGATAAAGATGAAGCA
TCGGTAAACGATGCGTTGCTGTGTCAAGACCCAAGTCTTGCTCAACAGCATATGAAAGATGAATGTGACATTAATGTCATCATTGAACGTTTCGG
GGTTACAG

>000093F|arrow

CAAACTGCTGTTGGTGCTTTAAGAGAATTAATTCAGCTTCTAATACTTTGTCTAATAGTACAAATGCTGGTGTTGCTACTAATCAGTTATACGCTG
ATTTGTCTACTGCTACTGCTGCGACTATTAACCAACTTCGTCAATCTTTCCAGATTCAGAAGTTATTGGAGCGCGATGCACGTGGTGGTACTCGTT
ATACTGAGTTACTACGTGCTCACTTTGGAGTAACTCCACAGGATTATCGTTTACAACGTCCTGAATATATTGGTGGAGGTTCGACCCTTGTTAATG
TTAATCCGATTGCTCAGACTTCTGCAACGTCGGTTACTGGTTCTGCTACTCCGCAAGGTAACCTTGCTGCAATGGGTACTGCATTGGCTCAGGGA
CACGGCTTTACGTATGCTGCTCAAGAACATGGATACATTATCGGATTAGTTTCTGTACGTGCTGACCTCACATATCAACAGGGTCTTCCTAAGATG
TGGTCTAGGTCTACACGATATGACTTTTATTTCCCAGTATTTGCCACTTTGGGTGAGCAAGCTATTTTGAACAAAGAAATTTATGTTCAAGGTACT
GCAGCCGACAATGATGTATTTGGTTATCAAGAACGTTGGGCGGAGTATCGTTACAAACCTTCTCAAATTACTGGTTTCTTTAGGTCTACTTCTGCT
GGCACTATTGATGCTTGGCATTATGGACAGCGATTTACTTCTCTTCCTACGTTGAATTCAACGTTTATTCAAGAGACCCCTCCAGTTGCTCGTACTA
CGGCGGTCGGAGCTGCAGCAAATGGTCAGCAATTTTAATGGATGCTTTCTTTGATTGTCAGATGGCCAGACCTATGCCTATGTACAGCGTACCTG
GTCTAATTGATCATTTCTAATGTTTATATAACCTCGACTACTCCGTAAGGAGTAGTGAGGAAACAACCGAAGGGCGTTAGTTTATGTTTGGTGGA
ATACTTGATGCGGTTACTAATGTTGGTTCTAAGCTGTCTTCAGCTTCTAGTTTCTTTACTCCTGGTGTCGGTACTGCTTTGGGCGCTGTTGGTTCTT
ATTTAGGTTCTACTTCTGCTAATAAAGCTAATCAGGAGATGGCTCAGAGGCAAATGGATTTTCAAGCCGATATGAGTGGAACAAGTTACCAGCG
TGCTGTTAAAGATTTAGAAGCTGCTGGTTTATCTCCTATGTTAGCCTATCAACGTGGTGGTGCTTCTACCCCATCTGGTTCAACTGCTACTATGGA
AAATGTTTTAGGTAATGCAACTAATTCAGCTATTAATACTGCTTCTATGATGCAACAGATTCGTAATGCATCAGAAACAGAAAAGCAGATTATCG
CCCAGACTGAAGCTACTGAAGCTGGTACCGCTAATACTAGGGCTGATACTGTTAATAAGTTGCTTACTGCTCCTAATATTACAGCCGAAAATAAA
CGTATTTTGGCTGATATTGCTTTAAAGAATACGACTGCGGATTTAACATCCGCTCAGTCATATAATACTAAGAGGCTATTGGCTCCATCCCCAGCT
ATTTGGTCTAGGGGTATCGATGCTTCGAAAGAAATTTTTGATAAACTCAAAAATAATCCTAATCAACTAACCCCTTGGGGAATTGGAGTCAAATA
ATGAGTAAAGCGAATTTGCCATTTGTACGTAATCCGTACAACTATGATAAAGATGAAGCATCGGTAAACGATGCGTTGCTGTGTCAAGACCCAA
GTCTTGCTCAACAGCATATGAAAGATGAATGTGACATTAATGTCATCATTGAACGTTTCGGGGTTACAGGGGAACTTCCAACGGCCCCTGTATCG
CCTCAATACGGCGATTTTAGTGGTGTTACTGATTACCATTCTGCGTTGAATCAAATTAACGCAACTATGGACGATTTCATGGCTCTGCCAGCGAAA
TTAAGAGTCCGATTTGACCATGATCCTGTCAAATTATTGGAGTTCCTTGAGAACGACCAGAATCGTGATGAAGCGATTCAATTGGGTCTTATTGA
TGGACAACCTGTGGTTGAACCCATCGTTTCTACAGAAACACCTAAGGCCGAAGGATGAAATCCTGAGGCCAGCACAGTTACTCTACTTGATGTAA
CTGTGCTAGGTGACACCAAACCACTATTTTAACTACGGAGTTCATCATGTTACGAAGAAAGCCAGTAAACAATATAAATCTGCAAAGTCATTTCG
CAGAACTGCTAGTAAGACGAAGTCAATTAATATGAGACACGCTCCCCAGCGTGGTGGCTATCGTTTGTAATTATGGCCTGTTATAAGCCCTTAAC
GGCTTATCAATGCAGTGACAGGTCTATAATTTGGCGGGAAATACCGGGTGCGGATGTAGTCCGTACCCTATCATTGCCTTGTGGTCAGTGTGTTG
GTTGTCGCCTTGAACGCTCACGTCAGTGGGCGATTCGTTGTATGCATGAGGCACAAATGCATACTAGTAATTGTTTTATTACTTTGACATATGCTC
CAGAGCATTGTCCTAAGGATATGTCATTGGATTACAATGATTATCAGCTTTTTATGAAGCGGTTACGTAAGCGTTTTACTGGGAAAACGATACGT
TTTTATATGGCAGGTGAATATGGTGAATCTTTTGATCGTCCTCATTTCCATGCTTGTCTGTTTGGTCTTGATTTTCCGGATAAGAAAATATTTAAAA
GAACGCAGACTGGCTCTATCCTCTACACGTCAGAGATTTTGGAAGAATTGTGGCCGTTTGGCTATTCTACATTGGTGATGTTACTTTTGAGTCTGC
TGCTTATGTTGCAAGATATATTATGAAGAAGATTAATGGGGTTACTGTCAATGAAAACCACGAAGTGGTTGATGCGGGTGCCCATTATCAATATT
GTGATTTAGAGACTGGTGAGATAATTCAGCGTAAGCCAGAATTTAATAAGATGTCATTGAAGCCCGGTATCGGGCAATCGTGGTTAGATAAGTA
CATGTCAGACGTTTATACGTCAGACCACGTTGTGGTGCGTGGCAAAAAGTGCCGTCCACCACGGTTTTATGATAATAAATTTAAGTTGAAGTTTC
CTGAAGAATTCGATATGATTCAGTTTGCCAGAGAGATGGAAGGTCGATCTAGGCATGAGGACAACACGCTTGAGCGACTTGCTGTTAAGGAAA
AAGTTGCGTTGGCTAGTTGTCATTGTTAAAACGTACTATTTAAGGAGTTTTTATGAAGATGGTTATTGTTTCTATTAAGGATACTGCTGCAGATGC
TTTTGGTCGTCCAGCTTATGTTGCATCTGAAGGTGTTGCAGTACGTCAGTTTCAGGATGAAGTCAATCGAGCTAGCGAAGATAATCAGTTGTATA
AACATCCTGATGATTTTCATATGTTCTATTTGGGTCTTTTTGACGATGCCACTGGTGTTTTTGAACTACTGGAAAGCCCTAAGTTGATTGCTCGTGC
AAAGATGTAATGATTCGCGAAGGCGAGTAAGGTTTTTTTATACCGTATCACTCGAAAGAGTGGTACGGAACTACGGGAGATGTTTATGTTTCG

CAATAAGTCAGTAAGTACGCATTCATTGCTATGGTTCCTAAAGCGGACATTCCCCGCTCTAGTTTTAATACTCAATATGCTCATAAAACCACGTTT
GATGCTGGTTTTTTAGTTCCTATTTATTGTGATGAAGTATTGCCTGGCGATACTCATCGTGTAAAGATGACTGCATTTGCACGTTTGGCCACACCG
TTATTTCCTGTGATGGACAACTTGCATCTTGATACTTTCTTTTTCTTTGTACCTAATCGTTTACTTTGGAACAATTGGCCAAAGTTTATGGGTGAAC
AAACGAATCCTGGTGATTCTATTTCTTTTGTAGTGCCTACTATTACTAGTCCTGCTGGTGGTTATGCTGTTTGTTCAATTTTTGATTATTTTGGTTTA
CCTACTGCTGGTCAGATTACTGGCGCTAATACAGTAACGCATAATGTTTTGCCGTTACGTGCTTATAATGAGATTTATAACGAATGGTTTAGAGAT
GAAAACTTACAGAATTCTGTAACGTTAAATCTTGGTGATTCAGGTGATGTTCCTGCTAACTATACACTTTGAGACGTGGTAAGCGTAAAGATTATT
TTACTGGTGCATTGCCTTGGCCACAGAAGGGTGCTTCTGTTTCTTTACCGTTAGGAACACGTGCTAATATTTATTCTGACATACCAGCTGGCAATG
GTACTGCTGGTTATAGTGTTTTT

>000011F|arrow

CGTTAATTTGATTCAACGCAGAATGGTAATCAGTAACCACTAAAATCGCCGTATTGAGGCGATACAGGGGCCGTGGAAGTTCCCCTGTAACCCC
GAACGTTCAATGATGACATTAATGTCACATTCATCTTTCATATGCTGTGAGCAAGACTTGGGTCTTGACACAGCAACGCATCGTTTACCGATGCTT
CATCTTTATCATAGTTTACGATTACGTACAAATGGCAAATTCGCTTTACTCATTATTTGACTCCAATTCCCCAAGGGGTTAGTTGATTAGGATTATT
TTTGAGTTTATCAAAAATTTCTTCGAAGCATCGATACCCCTAGACCAATAGCTGGGGATGGAGCCAATAGCCTCTAGTATTATCTGAGCGGATGT
TAAATCCGCAGTCGTATTCTTTAAAGCAATATCAGCCAAAATACTTTATTTTCGGCTGTAATATTGGAGCAGTAAGCACTTATTAACAGTATCACC
CTATTAGCGGTACCAGCTTCATAGCTTCAGTCTGGGCGATAATCTGCTTTTCTGTTTCTGATGCATTACGAATCTGTTGCATCATGAAGCAGTATT
AATAGCTGATTAGTTGCATTACCTAAAACATTTTCCATAGTAGCAGTTGAACCAGATGGGTAGAAGCACCACCACGTTGATAGGCTAACATAGGA
TAAACCAGCAGCTTCTAAATCTTTAACAGCACGCTGGTAACTTGTTCCACTCATATCGCTTGAAAATCCATTTGCCTCTGAGCCATCTCCTGATTAG
CTTATTAGCAGAAGTAAACCTAAATAAGAACCAACAGCGCCCAAAGCAGTACCGACACCAGAGTAAAAGACTAGAAGCTGAAGACAGCTTAGA
ACCAACATTAGTAACCGCATCAAGTATTCCACCAAACATAAACTAACGCCCTTCGGTTGTTTCCTCACTACTCCTTACGGAGTAGTCGAGGTTATA
TAAAACATTAGAATGATCAATTAGACCAGGTACGCTGTACATAGGCATAGTCTGGCCATCTGACAATCAAAGAAAGCATCCTTAAAATTGCTGAC
CATTTGCTGCAGCTCCGACCGCCGTAGTACGAGCAACTGGAGGGTCTCTTGAATAAACGTTGAATTCACGTAGGAAGAGAAGTAAATCGCTGTC
CATAATGCCAAGCATCAATAGTGCCAGCAGAAGTAGACCTAAAGAACCAGTAATTTGAGAAGGTTTGTAACGATACTCCGCCCAACGTTCTTGAT
AACCAATACATCATTGTCGGCTGCAGTACCTTGAACATAAATTTCTTTGTTCAAAATAGCTTGCTCACCCAAAGTGGCAAATACTGGGAAATAAAA
GTCATATCGTGTAGACCTAGACCACATCTAGGAAGACCCTGTTGATATGTGAGGTCAGCACGTACAGAAACTAATCCGATAATGTATCCATGTTC
TTGAGCAGCATACGTAAAGCCGTGTCCCTGAGCCAATGCAGTACCCATTGCAGCAAGGTTACCTTGCGGAGTAGCAACCAGTAACCGACGTTGC
AGAAGTCTGAGCAATCGGATTAACATTAACAAGGGTCGAACCTCCACCAATAATTCAGACGTTGTAAACGATAATCCTGTGGAGTTACTCCAAAG
TGAGCACGTAGTAACTCAGTATACGAGTACCACCACGTGCATCGCGCTCCAATAACTTCTGAATCTGGAAAGATTGACGAAGTTGGTTAATAGTC
GCAGCAGTAGCGTAGACAAATCAGCGTATAACTGATTAGTAGCAACACCACATTTGTACTATTAGACAAAGTATTAGAAGCTGAATTTAATTCTC
TTAAAGCACCAACAGCATTTGAAAAACACTATAACCAGCAGTACCATTGCCAGCTGGTATGTCAGAATAAATACACGTGTTCCTAACGGTAAGAA
ACAGAGCACCCTTCTGTGGCCAAGGCAATGCACCAGTAAATAATCTTTACGCTTACCACGTCTCAAAAGTGTATAGTTAGCAGGAACATCACCTG
AATCACCAAGATTTAACGTTACAGAATTCTGTAGTTTTCATCTCTAAACCATTCGTTATAAATCTCATTATAAGCACGTAACGGCAAAACATTATGC
GTTACTGTATTAGCGCCAGTAATCTGACCAGCAGTAGGTAAACCAAAATAATCAAAATTGAACAAACAGCATAACCACAGCACTAGTAATAGTA
GGCACTACAAAGAAATAGAATCACCAGGATTCGTTTGTTCACCCATAAACTTTGGCCAATTGTTCCAAAGTAAACGATTAGGTACAAAGAAAAAG
AAAGTATCAAGATGCAAGTTGTCCATCACAGGAATACGGTGTGGCCAAACGTGCAAATGCAGTCATCTTTACACGATGAGTATCGCCAGGCAAT
ACTTCATCACAATAAATAGGAACTAAAAACCAGCATCAAACGTGGTTTTATGACATATTGAGTATTAAAACTAGAGCGGGGAATGTCCGCTTTAG
GAACCATAGCAAATGAATGCGTACTTACTGACTTATTGCGAACATAACATCTCCCGTAGTTCCGTACCACTCTTTCGAGTGATACGGTATAAAAAA
AACCTTACTCGCCTTCGCGAATCATTACATCTTTTGCACGAGCAATCAACTTAGGCTTTCCAGTAGTTCAAAAACACCAGTGGCCTCGTCAAAAAG
ACCAAATAGAACATATGAAAATCATCAGGATGTTTATACAACTGATTATCTTCGCTAGCTCGATTGACTTCATCCTGAAGCTGACGTACTGCAACA
CCTTCAGATGCAACATAAGCTGGACGACCAAAAGCATCTGCAGCAGTATCCTTAATAGAAACAATAACCATCTTCATAAAAACTCCTTAAATAGT
ACGTTTTAACAATGACAACTTAGCCAACGCAACTTTTCCTTAACAGCAAGTCGCTCAAGCGGTGTTGTCCTCATGCCTAGATCGACCTTCCATCTCT
CTGGCAAACTGAATCATATCGAATTCGTCAGGGAAACTTCAACTTAAATTTATTATCATAAACCGTGGTGGACGGCACTTTTTGCCACGCACCACA
ACGTGGTCTGACGTATAACGTCTGACATGTACTTATCTAACCACGATTGCCCGATACCGGGCTTCAATGACATCTTATTAAATTCTGGCTTACGCT
GAATTATCTCACCAGTCTCTAATCACAATATTGATAATGGGCACCCGCATCAACCACTTCGTGGTTTTCATTGACAGTAACCCCATTAATCTTCTTC
ATAATATATCTTGCAACATAAGCAGCAGACTCAAAAGTAACATCACCAATTGTAGAATAGCCAAACGGCCACAATTCTTCCAAAATCTCTGACGT
GTAGAGGATAGAGCCAGTCTGCGTTCTTTTAAATATTTTCTTATCCGGAAAATCAAGACCAAACAGACAAGCATGGAAATGAGGACGATCAAAA
GATTCACCATATTCACCTGCCATATAAAAACGTAATCGTTTTCCCAGTAAACGCTTACGTAACCGCTTCATAAAAAGCTGATAATCATTGTAATCCA
ATGACATATCCTTAGGACAATGCTCTGGAGCATATGTCAAGTAATAAAACATTACTAGTATGCATTTGTGCCTCATGCATACAACGAATCGCCCAC
TGACGTGAGCGTTCAAGGCGACAACCAACACACTGACCACAAGGCAATGATAGGGTACGGACTACATCCGCACCCGGTATTTCCCGCCAAATTA
TAGACCTGTCACTGCATTGATAAGCCGTTAAGGGCTTATACAGGCCATAATTACAAACGATAGCCACCACGCTGGGGAGCGTGTCTCATATTAAT
TGACTTCGTCCTTACTAGCAGTTCTGCGAAATGACTTTGCAGATTTATATTTGTTTACTGGCTTTCTTCGTAACATGATGAACTCCGTAGTTAAAAT
AGTGGTTTGGTGTCACCTAGCACAGTTACATCAAGTAGAGTAACTGTGCTGGCCTCAGGATTTCATCCTTCGGCCTTAGGTGTTTCTGTAGAAAC

GATGGGTTCAACCACAGGTTGTCCATCAATAAGACCCAATTGAATCGCTTCATCACGATTCTGGTCGTTCTCAAGGAACTCCAATAATTTGACAG
GATCATGGTCAAATCGGACTCTTAATTTCGCTGGCAGAGCCATGAAATCGTCCATAGTTG

>000154F|arrow

ATTATGGAAAAGTTTGGTATGACCGGACTTATTCCTCAAACTCCTTTAACGCCTCAATATGGCGACTTTAGTGGTGTCTATGACTACCACTCTGCT
CTGAACCAGATTATGGCTTCAGACAACGAATTTATGGCTTTACCAGCCAATATTCGTGAACGATTCGCTAATGATCCCGCGAATCTAATAGATTTT
CTAGAAAACCCTGAAAATCGCAGCGAAGCTGAAAAAATGGGACTGGTAAAACCAGCCCAAACCGAGGTTTCAACCCCTGTTGGAACCTCGGAA
GCACAGTTACCTACTTGATGTAACTGTGCTAGGTGACACCAAACCACAAAAACACGATAAACAAGGACAGAAAAAATGATGCGTCGCAGACCAG
CAAATAAGCAAAAGTCCGCTAGGACTTTCCGTAAACATGCTTCACATACAAAACACGCAAATATGCGAAACTCGCCAATGCGTGGAGGCTGGAG
ACTCTAATAAAGTCCCCAGGCACCTCACATGCCTTGTTATCACCCTCTCAAAGCATTTCAATGCTTTGACAAATCAATTGTTTTCGACGAAGTTCGG
AAACATGACATCGTTCGATCTTTAGACCTGCCCTGTGGGCAGTGCGTTGGATGCCGTCTAGAACGATCAAGACAATGGGCTATTCGGTGCATGC
ACGAAGCCCAATTGCATAAAAACAACTCATTCATAACACTCACATATGACAATACACATCTCCCAAGCGATGGCTCTTTGGATCACAAAGACTTTC
AACTGTTCCTTAAAAGACTTAGAAAAACTCTCGCAAAAAGAGGACTTACAATCCGCTATTACATGGCTGGAGAATATGGTGAACTCTTCGCAAGA
CCCCACTTCCATGCCTGTATCTTCGGATACGACTTTCCTGATAAAAA

>000135F|arrow

ATTTGTTTACTGGCTTTCTTCGTAACATGATGAACTCCGTAGTTAAAATAGTGGTTTGGTGTCACCTAGCACAGTTACATCAAGTAGAGTAACTGT
GCTGGCCTCAGGATTTCATCCTTCGGCCTTAGGTGTTTCTGTAGAAACGATGGGTTCAACCACAGGTTGTCCATCAATAAGACCCAATTGAATCG
CTTCATCACGATTCTGGTCGTTCTCAAGGAACTCCAATAATTTGACAGGATCATGGTCAAATCGGACTCTTAATTTCGCTGGCAGAGCCATGAAAT
CGTCCATAGTTGCGTTAATTTGATTCAACGCAGAATGGTAATCAGTAACACCACTAAAATCGCCGTATTGAGGCGATACAGGGGCCGTTGGAAG
TTCCCCTGTAACCCCGAAACGTTCAATGATGACATTAATGTCACATTCATCTTTCATATGCTGTTGAGCAAGACTTGGGTCTTGACACAGCAACGC
ATCGTTTACCGATGCTTCATCTTTATCATAGTTGTACGGATTACGTACAAATGGCAAATTCGCTTTACTCATTATTTGACTCCAATTCCCCAAGGGG
TTAGTTGATTAGGATTATTTTTGAGTTTATCAAAAATTTCTTTCGAAGCATCGATACCCCTAGACCAAATAGCTGGGGATGGAGCCAATAGCCTCT
TAGTATTATATGACTGAGCGGATGTTAAATCCGCAGTCGTATTCTTTAAAGCAATATCAGCCAAAATACGTTTATTTTCGGCTGTAATATTAGGAG
CAGTAAGCAACTTATTAACAGTATCAGCCCTAGTATTAGCGGTACCAGCTTCAGTAGCTTCAGTCTGGGCGATAATCTGCTTTTCTGTTTCTGATG
CATTACGAATCTGTTGCATCATAGAAGCAGTATTAATAGCTGAATTAGTTGCATTACCTAAAACATTTTCCATAGTAGCAGTTGAACCAGATGGG
GTAGAAGCACCACCACGTTGATAGGCTAACATAGGAGATAAACCAGCAGCTTCTAAATCTTTAACAGCACGCTGGTAACTTGTTCCACTCATATC
GGCTTGAAAATCCATTTGCCTCTGAGCCATCTCCTGATTAGCTTTATTAGCAGAAGTAGAACCTAAATAAGAACCAACAGCGCCCAAAGCAGTAC
CGACACCAGGAGTAAAGAAACTAGAAGCTGAAGACAGCTTAGAACCAACATTAGTAACCGCATCAAGTATTCCACCAAACATAAACTAACGCCC
TTCGGTTGTTTCCTCACTACTCCTTACGGAGTAGTCGAGGTTATATAAAACATTAGAAATGATCAATTAGACCAGGTACGCTGTACATAGGCATA
GGTCTGGCCATCTGACAATCAAAGAAAGCATCCATTAAAAATTGCTGACCATTTGCTGCAGCTCCGACCGCCGTAGTACGAGCAACTGGAGGGG
TCTCTTGAATAAACGTTGAATTCAACGTAGGAAGAGAAGTAAATCGCTGTCCATAATGCCAAGCATCAATAGTGCCAGCAGAAGTAGACCTAAA
GAAACCAGTAATTTGAGAAGGTTTGTAACGATACTCCGCCCAACGTTCTTGATAACCAAATACATCATTGTCGGCTGCAGTACCTTGAACATAAA
TTTCTTTGTTCAAAATAGCTTGCTCACCCAAAGTGGCAAATACTGGGAAATAAAAGTCATATCGTGTAGACCTAGACCACATCTTAGGAAGACCC
TGTTGATATGTGAGGTCAGCACGTACAGAAACTAATCCGATAATGTATCCATGTTCTTGAGCAGCATACGTAAAGCCGTGTCCCTGAGCCAATGC
AGTACCCATTGCAGCAAGGTTACCTTGCGGAGTAGCAGAACCAGTAACCGACGTTGCAGAAGTCTGAGCAATCGGATTAACATTAACAAGGGTC
GAACCTCCACCAATATATTCAGGACGTTGTAAACGATAATCCTGTGGAGTTACTCCAAAGTGAGCACGTAGTAACTCAGTATAACGAGTACCACC
ACGTGCATCGCGCTCCAATAACTTCTGAATCTGGAAAGATTGACGAAGTTGGTTAATAGTCGCAGCAGTAGCAGTAGACAAATCAGCGTATAAC
TGATTAGTAGCAACACCAGCATTTGTACTATTAGACAAAGTATTAGAAGCTGAATTTAATTCTCTTAAAGCACCAACAGCAGTTTGAAAAACACTA
TAACCAGCAGTACCATTGCCAGCTGGTATGTCAGAATAAATATTAGCACGTGTTCCTAACGGTAAAGAAACAGAAGCACCCTTCTGTGGCCAAG
GCAATGCACCAGTAAAATAATCTTTACGCTTACCACGTCTCAAAAGTGTATAGTTAGCAGGAACATCACCTGAATCACCAAGATTTAACGTTACA
GAATTCTGTAAGTTTTCATCTCTAAACCATTCGTTATAAATCTCATTATAAGCACGTAACGGCAAAACATTATGCGTTACTGTATTAGCGCCAGTA
ATCTGACCAGCAGTAGGTAAACCAAAATAATCAAAAATTGAACAAACAGCATAACCACCAGCAGGACTAGTAATAGTAGGCACTACAAAAGAAA
TAGAATCACCAGGATTCGTTTGTTCACCCATAAACTTTGGCCAATTGTTCCAAAGTAAACGATTAGGTACAAAGAAAAAGAAAGTATCAAGATGC
AAGTTGTCCATCACAGGAAATAACGGTGTGGCCAAACGTGCAAATGCAGTCATCTTTACACGATGAGTATCGCCAGGCAATACTTCATCACAATA
AATAGGAACTAAAAAACCAGCATCAAACGTGGTTTTATGAGCATATTGAGTATTAAAACTAGAGCGGGGAATGTCCGCTTTAGGAACCATAGCA
AATGAATGCGTACTTACTGACTTATTGCGAAACATAAACATCTCCCGTAGTTCCGTACCACTCTTTCGAGTGATACGGTATAAAAAAAAACCTTACT
CGCCTTCGCGAATCATTACATCTTTTGCACGAGCAATCAACTTAGGGCTTTCCAGTAGTTCAAAAACACCAGTGGCATCGTCAAAAAGACCCAAA
TAGAACATATGAAAATCATCAGGATGTTTATACAACTGATTATCTTCGCTAGCTCGATTGACTTCATCCTGAAACTGACGTACTGCAACACCTTCA
GATGCAACATAAGCTGGACGACCAAAAGCATCTGCAGCAGTATCCTTAATAGAAACAATAACCATCTTCATAAAAACTCCTTAAATAGTACGTTT
TAACAATGACAACTTAGCCAACGCAACTTTTTCCTTAACAGCAAGTCGCTCAAGCGTGTTGTCCTCATGCCTAGATCGACCTTCCATCTCTCTGGC
AAACTGAATCATATCGAATTCTTCAGGAAACTTCAACTTAAATTTATTATCATAAAACCGTGGTGGACGGCACTTTTTGCCACGCACCACAACGTG

GTCTGACGTATAAACGTCTGACATGTACTTATCTAACCACGATTGCCCGATACCGGGCTTCAATGACATCTTATTAAATTCTGGCTTACGCTGAAT
TATCTCACCAGTCTCTAAATCACAATATTGATAATGGGCACCCGCATCAACCACTTCGTGGTTTCATTGACAGTAACCCCATTAATCTTCTTCATA
ATATATCTTGCAACATAAGCAGCAGACTCAAAAGTAACATCACCAATTGTAGAATAGCCAAACGGCCACAATTCTTCCAAAATCTCTGACGTGTA
GAGGATAGAGCCAGTCTGCGTTCTTTTAAATATTTTCTTATCCGGAAAATCAAGACCAAACAGACAAGCATGGAAATGAGGACGATCAAAAGAT
TCACCATATTCACCTGCCATATAAAAACGTATCGTTTTCCCAGTAAAACGCTTACGTAACCGCTTCATAAAAAGCTGATAATCATTGTAATCCAATG
ACATATCCTTAGGACAATGCTCTGGAGCATATGTCAAAGTAATAAAACAATTACTAGTATGCATTTGTGCCTCATGCATACAACGAATCGCCCACT
GACGTGAGCGTTCAAGGCGACAACCAACACACTGACCACAAGGCAATGATAGGGTACGGACTACATCCGCACCCGGTATTTCCCGCCAAATTAT
AGACCTGTCACTGCATTGATAAGCCGTTAAGGGCTTATAACAGGCCATAATTACAAACGATAGCCACCACGCTGGGGAGCGTGTCTCATATTAAT
TGACTTCGTCTTACTAGCAGTTCTGCGAAATGACTTTGCAGATTTAT

>000056F|arrow

TGGTCTAGGTCTACACGATATGACTTTTATTTCCCAGTATTTGCCACTTTGGGTGAGCAAGCTATTTTGAACAAAGAAATTTATGTTCAAGGTACT
GCAGCCGACAATGATGTATTTGGTTATCAAGAACGTTGGGCGGAGTATCGTTACAAACCTTCTCAAATTACTGGTTTCTTTAGGTCTACTTCTGCT
GGCACTATTGATGCTTGGCATTATGGACAGCGATTTACTTCTCTTCCTACGTTGAATTCAACGTTTATTCAAGAGACCCCTCCAGTTGCTCGTACTA
CGGCGGTCGGAGCTGCAGCAAATGGTCAGCAATTTTTAATGGATGCTTTCTTTGATTGTCAGATGGCCAGACCTATGCCTATGTACAGCGTACCT
GGTCTAATTGATCATTTCTAATGTTTTATATAACCTCGACTACTCCGTAAGGAGTAGTGAGGAAACAACCGAAGGGCGTTAGTTTATGTTTGGTG
GAATACTTGATGCGGTTACTAATGTTGGTTCTAAGCTGTCTTCAGCTTCTAGTTTCTTTACTCCTGGTGTCGGTACTGCTTTGGGCGCTGTTGGTTC
TTATTTAGGTTCTACTTCTGCTAATAAAGCTAATCAGGAGATGGCTCAGAGGCAAATGGATTTTCAAGCCGATATGAGTGGAACAAGTTACCAGC
GTGCTGTTAAAGATTTAGAAGCTGCTGGTTTATCTCCTATGTTAGCCTATCAACGTGGTGGTGCTTCTACCCCATCTGGTTCAACTGCTACTATGG
AAAATGTTTTAGGTAATGCAACTAATTCAGCTATTAATACTGCTTCTATGATGCAACAGATTCGTAATGCATCAGAAACAGAAAAGCAGATTATC
GCCCAGACTGAAGCTACTGAAGCTGGTACCGCTAATACTAGGGCTGATACTGTTAATAAGTTGCTTACTGCTCCTAATATTACAGCCGAAAATAA
ACGTATTTTGGCTGATATTGCTTTAAAGAATACGACTGCGGATTTAACATCCGCTCAGTCATATAATACTAAGAGGCTATTGGCTCCATCCCCAGC
TATTTGGTCTAGGGGTATCGATGCTTCGAAAGAAATTTTTGATAAACTCAAAAATAATCCTAATCAACTAACCCCTTGGGGAATTGGAGTCAAAT
AATGAGTAAAGCGAATTTGCCATTTGTACGTAATCCGTACAACTATGATAAAGATGAAGCATCGGTAAACGATGCGTTGCTGTGTCAAGACCCA
AGTCTTGCTCAACAGCATATGAAAGATGAATGTGACATTAATGTCATCATTGAACGTTTCGGGGTTACAGGGGAACTTCCAACGGCCCCTGTATC
GCCTCAATACGGCGATTTTAGTGGTGTTACTGATTACCATTCTGCGTTGAATCAAATTAACGCAACTATGGACGATTTCATGGCTCTGCCAGCGAA
ATTAAGAGTCCGATTTGACCATGATCCTGTCAAATTATTGGAGTTCCTTGAGAACGACCAGAATCGTGATGAAGCGATTCAATTGGGTCTTATTG
ATGGACAACCTGTGGTTGAACCCATCGTTTCTACAGAAACACCTAAGGCCGAAGGATGAAATCCTGAGGCCAGCACAGTTACTCTACTTGATGTA
ACTGTGCTAGGTGACACCAAACCACTATTTTAACTACGGAGTTCATCATGTTACGAAGAAAGCCAGTAAACAAATATAAATCTGCAAAGTCATTT
CGCAGAACTGCTAGTAAGACGAAGTCAATTAATATGAGACACGCTCCCCAGCGTGGTGGCTATCGTTTGTAATTATGGCCTGTTATAAGCCCTTA
ACGGCTTATCAATGCAGTGACAGGTCTATAATTTGGCGGGAAATACCGGGTGCGGATGTAGTCCGTACCCTATCATTGCCTTGTGGTCAGTGTGT
TGGTTGTCGCCTTGAACGCTCACGTCAGTGGGCGATTCGTTGTATGCATGAGGCACAAATGCATACTAGTAATTGTTTTATTACTTTGACATATGC
TCCAGAGCATTGTCCTAAGGATATGTCATTGGATTACAATGATTATCAGCTTTTTATGAAGCGGTTACGTAAGCGTTTTACTGGGAAAACGATAC
GTTTTTATATGGCAGGTGAATATGGTGAATCTTTTGATCGTCCTCATTTCCATGCTTGTCTGTTTGGTCTTGATTTTCCGGATAAGAAAATATTTAA
AAGAACGCAGACTGGCTCTATCCTCTACACGTCAGAGATTTTGGAAGAATTGTGGCCGTTTGGCTATTCTACAATTGGTGATGTTACTTTTGAGTC
TGCTGCTTATGTTGCAAGATATATTATGAAGAAGATTAATGGGGTTACTGTCAATGAAAACCACGAAGTGGTTGATGCGGGTGCCCATTATCAAT
ATTGTGATTTAGAGACTGGTGAGATAATTCAGCGTAAGCCAGAATTTAATAAGATGTCATTGAAGCCCGGTATCGGGCAATCGTGGTTAGATAA
GTACATGTCAGACGTTTATACGTCAGACCACGTTGTGGTGCGTGGCAAAAAGTGCCGTCCACCACGGTTTTATGATAATAAATTTAAGTTGAAGT
TTCCTGAAGAATTCGATATGATTCAGTTTGCCAGAGAGATGGAAGGTCGATCTAGGCATGAGGACAACACGCTTGAGCGACTTGCTGTTAAGGA
AAAAGTTGCGTTGGCTAAGTTGTCATTGTTAAAACGTACTATTTAAGGAGTTTTTATGAAGATGGTTATTGTTTCTATTAAGGATACTGCTGCAGA
TGCTTTTGGTCGTCCAGCTTATGTTGCATCTGAAGGTGTTGCAGTACGTCAGTTTCAGGATGAAGTCAATCGAGCTAGCGAAGATAATCAGTTGT
ATAAACATCCTGATGATTTTCATATGTTCTATTTGGGTCTTTTTGACGATGCCACTGGTGTTTTTGAACTACTGGAAAGCCCTAAGTTGATTGCTCG
TGCAAAAGATGTAATGATTCGCGAAGGCGAGTAAGGTTTTTTTTATACCGTATCACTCGAAAGAGTGGTACGGAACTACGGGAGATGTTTATGT
TTCGCAATAAGTCAGTAAGTACGCATTCATTTGCTATGGTTCCTAAAGCGGACATTCCCCGCTCTAGTTTTAATACTCAATATGCTCATAAAACCAC
GTTTGATGCTGGTTTTTTAGTTCCTATTTATTGTGATGAAGTATTGCCTGGCGATACTCATCGTGTAAAGATGACTGCATTTGCACGTTTGGCCAC
ACCGTTATTTCCTGTGATGGACAACTTGCATCTTGATACTTTCTTTTTCTTTGTACCTAATCGTTTACTTTGGAACAATTGGCCAAAGTTTATGGGT
GAACAAACGAATCCTGGTGATTCTATTTCTTTTGTAGTGCCTACTATTACTAGTCCTGCTGGTGGTTATGCTGTTTGTTCAATTTTTGATTATTTTGG
TTTACCTACTGCTGGTCAGATTACTGGCGCTAATACAGTAACGCATAATGTTTTGCCGTTACGTGCTTATAATGAGATTTATAACGAATGGTTTAG
AGATGAAAACTTACAGAATTCTGTAACGTTAAATCTTGGTGATTCAGGTGATGTTCCTGCTAACTATACACTTTTGAGACGTGGTAAGCGTAAAG
ATTATTTTACTGGTGCATTGCCTTGGCCACAGAAGGGTGCTTCTGTTTCTTTACCGTTAGGAACACGTGCTAATATTTATTCTGACATACCAGCTG
GCAATGGTACTGCTGGTTATAGTGTTTTTCAAACTGCTGTTGGTGCTTTAAGAGAATTAAATTCAGCTTCTAATACTTTGTCTAATAGTACAAATG
CTGGTGTTGCTACTAATCAGTTATACGCTGATTTGTCTACTGCTACTGCTGCGACTATTAACCAACTTCGTCAATCTTTCCAGATTCAGAAGTTATT

GGAGCGCGATGCACGTGGTGGTACTCGTTATACTGAGTTACTACGTGCTCACTTTGGAGTAACTCCACAGGATTATCGTTTACAACGTCCTGAAT
ATATTGGTGGAGGTTCGACCCTTGTTAATGTTAATCCGATTGCTCAGACTTCTGCAACGTCGGTTACTGGTTCTGCTACTCCGCAAGGTAACCTTG
CTGCAATGGGTACTGCATTGGCTCAGGGACACGGCTTTACGTATGCTGCTCAAGAACATGGATACATTATCGGATTAGTTTCTGTACGTGCTGAC
CTCACATATCAACAGGGTCTTCCTAAGATG

>000112F|arrow

AAATGATCAATTAGACCAGGTACGCTGTACATAGGCATAGGTCTGGCCATCTGACAATCAAAGAAAGCATCCATTAAAAATTGCTGACCATTTGC
TGCAGCTCCGACCGCCGTAGTACGAGCAACTGGAGGGGTCTCTTGAATAAACGTTGAATTCAACGTAGGAAGAGAAGTAAATCGCTGTCCATAA
TGCCAAGCATCAATAGTGCCAGCAGAAGTAGACCTAAAGAAACCAGTAATTTGAGAAGGTTTGTAACGATACTCCGCCCAACGTTCTTGATAACC
AAATACATCATTGTCGGCTGCAGTACCTTGAACATAAATTTCTTTGTTCAAAATAGCTTGCTCACCCAAAGTGGCAAATACTGGGAAATAAAAGT
CATATCGTGTAGACCTAGACCACATCTTAGGAAGACCCTGTTGATATGTGAGGTCAGCACGTACAGAAACTAATCCGATAATGTATCCATGTTCT
TGAGCAGCATACGTAAAGCCGTGTCCCTGAGCCAATGCAGTACCCATTGCAGCAAGGTTACCTTGCGGAGTAGCAGAACCAGTAACCGACGTTG
CAGAAGTCTGAGCAATCGGATTAACATTAACAAGGGTCGAACCTCCACCAATATATTCAGGACGTTGTAAACGATAATCCTGTGGAGTTACTCCA
AAGTGAGCACGTAGTAACTCAGTATAACGAGTACCACCACGTGCATCGCGCTCCAATAACTTCTGAATCTGGAAAGATTGACGAAGTTGGTTAA
TAGTCGCAGCAGTAGCAGTAGACAAATCAGCGTATAACTGATTAGTAGCAACACCAGCATTTGTACTATTAGACAAAGTATTAGAAGCTGAATTT
AATTCTCTTAAAGCACCAACAGCAGTTTGAAAAACACTATAACCAGCAGTACCATTGCCAGCTGGTATGTCAGAATAAATATTAGCACGTGTTCCT
AACGGTAAAGAAACAGAAGCACCCTTCTGTGGCCAAGGCAATGCACCAGTAAAATAATCTTTACGCTTACCACGTCTCAAAAGTGTATAGTTAGC
AGGAACATCACCTGAATCACCAAGATTTAACGTTACAGAATTCTGTAAGTTTTCATCTCTAAACCATTCGTTATAAATCTCATTATAAGCACGTAAC
GGCAAAACATTATGCGTTACTGTATTAGCGCCAGTAATCTGACCAGCAGTAGGTAAACCAAAATAATCAAAAATTGAACAAACAGCATAACCAC
CAGCAGGACTAGTAATAGTAGGCACTACAAAAGAAATAGAATCACCAGGATTCGTTTGTTCACCCATAAACTTTGGCCAATTGTTCCAAAGTAAA
CGATTAGGTACAAAGAAAAAGAAAGTATCAAGATGCAAGTTGTCCATCACAGGAAATAACGGTGTGGCCAAACGTGCAAATGCAGTCATCTTTA
CACGATGAGTATCGCCAGGCAATACTTCATCACAATAAATAGGAACTAAAAAACCAGCATCAAACGTGGTTTTATGAGCATATTGAGTATTAAAA
CTAGAGCGGGGAATGTCCGCTTTAGGAACCATAGCAAATGAATGCGTACTTACTGACTTATTGCGAAACATAAACATCTCCCGTAGTTCCGTACC
ACTCTTTCGAGTGATACGGTATAAAAAAAACCTTACTCGCCTTCGCGAATCATTACATCTTTTGCACGAGCAATCAACTTAGGGCTTTCCAGTAGT
TCAAAAACACCAGTGGCATCGTCAAAAAGACCCAAATAGAACATATGAAAATCATCAGGATGTTTATACAACTGATTATCTTCGCTAGCTCGATT
GACTTCATCCTGAAACTGACGTACTGCAACACCTTCAGATGCAACATAAGCTGGACGACCAAAAGCATCTGCAGCAGTATCCTTAATAGAAACAA
TAACCATCTTCATAAAAACTCCTTAAATAGTACGTTTTAACAATGACAACTTAGCCAACGCAACTTTTTCCTTAACAGCAAGTCGCTCAAGCGTGTT
GTCCTCATGCCTAGATCGACCTTCCATCTCTCTGGCAAACTGAATCATATCGAATTCTTCAGGAAACTTCAACTTAAATTTATTATCATAAAACCGT
GGTGGACGGCACTTTTTGCCACGCACCACAACGTGGTCTGACGTATAAACGTCTGACATGTACTTATCTAACCACGATTGCCCGATACCGGGCTT
CAATGACATCTTATTAAATTCTGGCTTACGCTGAATTATCTCACCAGTCTCTAAATCACAATATTGATAATGGGCACCCGCATCAACCACTTCGTG
GTTTTCATTGACAGTAACCCCATTAATCTTCTTCATAATATATCTTGCAACATAAGCAGCAGACTCAAAAGTAACATCACCAATTGTAGAATAGCC
AAACGGCCACAATTCTTCCAAAATCTCTGACGTGTAGAGGATAGAGCCAGTCTGCGTTCTTTTAAATATTTTCTTATCCGGAAAATCAAGACCAAA
CAGACAAGCATGGAAATGAGGACGATCAAAAGATTCACCATATTCACCTGCCATATAAAAACGTATCGTTTTCCCAGTAAAACGCTTACGTAACC
GCTTCATAAAAAGCTGATAATCATTGTAATCCAATGACATATCCTTAGGACAATGCTCTGGAGCATATGTCAAAGTAATAAAACAATTACTAGTAT
GCATTTGTGCCTCATGCATACAACGAATCGCCCACTGACGTGAGCGTTCAAGGCGACAACCAACACACTGACCACAAGGCAATGATAGGGTACG
GACTACATCCGCACCCGGTATTTCCCGCCAAATTATAGACCTGTCACTGCATTGATAAGCCGTTAAGGGCTTATAACAGGCCATAATTACAAACG
ATAGCCACCACGCTGGGGAGCGTGTCTCATATTAATTGACTTCGTCTTACTAGCAGTTCTGCGAAATGACTTTGCAGATTTATATTTGTTTACTGG
CTTTCTTCGTAACATGATGAACTCCGTAGTTAAAATAGTGGTTTGGTGTCACCTAGCACAGTTACATCAAGTAGAGTAACTGTGCTGGCCTCAGG
ATTTCATCCTTCGGCCTTAGGTGTTTCTGTAGAAACGATGGGTTCAACCACAGGTTGTCCATCAATAAGACCCAATTGAATCGCTTCATCACGATT
CTGGTCGTTCTCAAGGAACTCCAATAATTTGACAGGATCATGGTCAAATCGGACTCTTAATTTCGCTGGCAGAGCCATGAAATCGTCCATAGTTG
CGTTAATTTGATTCAACGCAGAATGGTAATCAGTAACACCACTAAAATCGCCGTATTGAGGCGATACAGGGGCCGTTGGAAGTTCCCCTGTAACC
CCGAAACGTTCAATGATGACATTAATGTCACATTCATCTTTCATATGCTGTTGAGCAAGACTTGGGTCTTGACACAGCAACGCATCGTTTACCGAT
GCTTCATCTTTATCATAGTTGTACGGATTACGTACAAATGGCAAATTCGCTTTACTCATTATTTGACTCCAATTCCCCAAGGGGTTAGTTGATTAGG
ATTATTTTTGAGTTTATCAAAAATTTCTTTCGAAGCATCGATACCCCTAGACCAAATAGCTGGGGATGGAGCCAATAGCCTCTTAGTATTATATGA
CTGAGCGGATGTTAAATCCGCAGTCGTATTCTTTAAAGCAATATCAGCCAAAATACGTTTATTTTCGGCTGTAATATTAGGAGCAGTAAGCAACTT
ATTAACAGTATCAGCCCTAGTATTAGCGGTACCAGCTTCAGTAGCTTCAGTCTGGGCGATAATCTGCTTTTCTGTTTCTGATGCATTACGAATCTG
TTGCATCATAGAAGCAGTATTAATAGCTGAATTAGTTGCATTACCTAAAACATTTTCCATAGTAGCAGTTGAACCAGATGGGGTAGAAGCACCAC
CACGTTGATAGGCTAACATAGGAGATAAACCAGCAGCTTCTAAATCTTTAACAGCACGCTGGTAACTTGTTCCACTCATATCGGCTTGAAAATCC
ATTTGCCTCTGAGCCATCTCCTGATTAGCTTTATTAGCAGAAGTAGAACCTAAATAAGAACCAACAGCGCCCAAAGCAGTACCGACACCAGGAGT
AAAGAAACTAGAAGCTGAAGACAGCTTAGAACCAACATTAGTAACCGCATCAAGTATTCCACCAAACATAAACTAACGCCCTTCGGTTGTTTCCT
CACTACTCCTTACGGAGTAGTCGAGGTTATATAAAACATTAG

>000034F|arrow

TTGCTCAGACTTCTGCAACGTCGGTTACTGGTTCTGCTACTCCGCAAGGTAACCTTGCTGCAATGGGTACTGCATTGGCTCAGGGACACGGCTTT
ACGTATGCTGCTCAAGAAACATGGATACATTATCGGATTAGTTTCTGTACGTGCTGACCTCACATATCAACAGGGTCTTCCTAAGATGTGGTCTA
GGTCTACACGATATGACTTTTATTTCCCAGTATTTGCCACTTTGGGTGAGCAAGCTATTTTGAACAAAGAAATTTATGTTCAAGGTACTGCAGCCG
ACAATGATGTATTTGGTTATCAAGAACGTTGGGCGGAGTATCGTTACAAACCTTCTCAAATTACTGGTTTCTTTAGGTCTACTTCTGCTGGCACTA
TTGATGCTTGGCATTATGGACAGCGATTTACTTCTCTTCCTACGTTGAATTCAACGTTTATTCAAGAGACCCCTCCAGTTGCTCGTACTACGGCGG
TCGGAGCTGCAGCAAATGGTCAGCAATTTTTAATGGATGCTTTCTTTGATTGTCAGATGGCCAGACCTATGCCTATGTACAGCGTACCTGGTCTA
ATTGATCATTTCTAATGTTTTATATATACCATCGACTACTCCGTAACATGAGTAGTGTAGAAACAACCGAGGGCTTTAGTTTATGTTTGGTGGAAT
ACTGATCGTACTAATGTGTTCTAAGCTTCTCACTTCTAGTTTGTACGCCTGGTGTCCGGTACTGCTTTGGGGCGCTGTTGGTCTTATTTTAGGGTTC
GACGTCTCTATAAAACTATCAAGGAGCTGGCTCAGAGCAAATGGATTTCAAGCCGAATGATTGGAACCTTACCAGCGTGCTGTTAAAAGAATGT
TATATCTGCCTGTTTATCGCCTATGTTGCCTAGCAACGTGGGGTGTGCGTCTACCCATCGGTGCAACTGCTACTAGAAAATGTTTTAGGTAATGCA
ACTATTCAGCTATTAAATAACTGCTTCTATGAGCAACAGATCGTATGCATCAGAACAGAAGCGATTATCTCCCAGACTGAGCTACTGAAGCTGGT
ACCGCAATCCTCGGGGCTGATACTGTTAATACTTGCTTTACTGCTCCTAATATTACAGCCGAAAATAAACGTATTTTGGCTGATATTGCTTATAAA
GAATACGACGCGGGATTTAACAGCCGCTCAGTCATATAATACTAAGAGGCTTATTGGCTCCATCCCAGCTATTTGGTCTAGGGGGTATCGATGGC
TTCGAAAGAAATTTTTGATAAACTCAAAAATAATCCTAATCAACTAACCCCTTGGGGAATTGGAGTCAAATAATGAGTAAAGCGAATTTGCCATT
TGTACGTAATCCGTACAACTATGATAAAGATGAAGCATCGGGGTAACGATGCGTTGCTGTGCAAGACCCAAGTCTTGCTCAACAGCATATGAAG
ATGAATGTGACATTAATGTCATCATTGAACGTTTCGGGTTACAGGGGAACTTCCACGGCCCTGTATCGCCTCAATACGGCGATTTTAGTGGTGTT
ACTGATTACCATTCTGCGTTGAATCAAATTAACGCACTATGGACGATTTCATGGCTCTGCCAGCGAAATTAAGAGTCCGATTTGACCATGATCCTG
TCAAATTATTGGAGTTCCTTGAGAACGACCAGAAGCGTGATGAAGCGATTCAATTGGGTCTTATTGATGGACAACCTGTGGTTGAACCCATCGAT
TTCTACAGAACACCTAAGGCCGAAGTATGAATCCTGAGGCCAGCACAGTTACTCTACTTGATGTAACTGTGCTAGTGACACCAAACCACTATTTA
ACTACGGAGTTCATCATGTTACAAGAAAGCCAGTAAACAATAGAAAATCTGCAAAAGGTCCATTTCGCAGAACTGCTAGTAAGACGAAGTCAAT
TAATATGAGACACGCTCCCAGCGGGGTGGGCTATCGTTTTGTATTATGGCCTGTTATAAGCCCTTAACGGCTTATCAATGCAGTGACAGGTCTAT
AATTTGGCGGGAAAATACCGGGTGCGGATGTAGTCCGTACCCTATCATTGCCTTGTGGTCAGTGTGTTGGTTGTCGCCTGAACGCTCACGCAGT
GGCGATTCGGTGTATGCAGGAGGCACAAATGCATACTATAATTGTTTTATTACTTTGACATATGCTCCAGCATTGTCCTAAGGATATGTCATTGGA
TTACAATGATTATCAGCTTTTTATGAAGCGGTTACGTAAGCGTTTTACTGGGAAAACGATACGTTTTTATATGGCAGTGAATATGGTGATCTTTTG
ATCGTCCTCATTTCCATGCTTGTCTGTTTGGTCTTGATTTTCCGGATAAAATATTTAAAAGAACGCAGACTGGCTCTATCCTCTACACGTCAGAGAT
TTTGGAAGAATTGTGGCCGTTTGGCTATTCTACAATTGGTGATGTTACTTTTGAGTCTGCTGCTTATGTTGCAAGATATATTATGAAGAGATTAAT
GGGTTACTGTCAATGAAAACCACGAAGTGGTTGATGCGGGTGCCCATTATCAATTGTGATTAGAGACTGGTGAGATAATTCAGCGTAAGCCAGA
ATTTAATAGATGTCATTGAAGCCCGGTATCGGGCAATCGTGGTTAGATAAGTACATGTCAGACGTTTATACGTCAGACCACGTTGTGGTGCGTG
GCAAAAAGTGCCGTCCACCACGGTTTTATGATAATAAATTTAAGTTGAAGTTTCCTGAAGAATTCGATATGATTCAGTTTGCCAGAGAGATGGAA
GGTCGATCTAGGCATGAGGACAACACGCTTGAGCGACTTGCTGTTAAGGAAAAAGTTGCGTTGGCTAAGTTGTCATTGTTAAAACGTACTATTTA
AGGAGTTTTTATGAAGATGGTTATTGTTTCTATTAAGGATACTGCTGCAGATGCTTTTGGTCGTCCAGCTTATGTTGCATCTGAAGGTGTTGCAGT
ACGTCAGTTTCAGGATGAAGTCAATCGAGCTAGCGAAGATAATCAGTTGTATAAACATCCTGATGATTTTCATATGTTCTATTTGGGTCTTTTTGA
CGATGCCACTGGTGTTTTTGAACTACTGGAAAGCCCTAAGTTGATTGCTCGTGCAAAAGATGTAATGATTCGCGAAGGCGAGTAAGGTTTTTTT
ATACCGTATCACTCGAAAGAGTGGTACGGAACTACGGGAGATGTTTATGTTTCGCAATAAGTCAGTAAGTACGCATTCATTTGCTATGGTTCCTA
AAGCGGACATTCCCCGCTCTAGTTTTAATACTCAATATGCTCATAAAACCACGTTTGATGCTGGTTTGTTAGTTCCTATTTATTGTGATGAAGTATT
GCCTGGCGATACTCATCGTGTAAAGATGACTGCATTTGCAACGTTTGGCCACACCGTTATTTCCTGTGATGGACAACTTGCATCTTGATACTTTCT
TTTTCTTTGTACCTAATCGTTTACTTTGGAACAATTGGCCAAAGTTTATGGGTGAACAAACGAATCCTGGTGATTCTATTTCTTTTGTAGTGCCTAC
TATTACTAGTCCTGCTGGTGGTTATGCTGTTTGTTCAATTTTTGATTATTTTGGTTTACCTACTGCTGGTCAGATTACTGGCGCTAATACAGTAACG
CATAATGTTTTGCCGTTACGTGCTTATAATGAGATTTATAACGAATGGTTTAGAGATGAAAACTTACAGAATTCTGTAACGTTAAATCTTGGTGAT
TCAGGTGATGTTCCTGCTAACTATACACTTTTGAGACGTGGTAAGCGTAAGATTATTTTACTGGTGCATTGCCTTGGCCACAGAAGGGTGCTTCT
GTTTCTTTACCGTTAGGAACACGTGCTAATATTTATTCTGACATACCAGCTGGCAATGGTACTGCTGGTTATAGTGTTTTTCAAACTGCTGTTGGT
GCTTTAAGAGAATTAAATTCAGCTTCTAATACTTTGTCTAATAGTACAAATGCTGGTGTTGCTACTAATCAGTTATACGCTGATTTGTCTACTGCTA
CTGCTGCGACTATTAACCAACTTCGTCAATCTTTCCAGATTCAGAAGTTATTGGAGCGCGATGCACGTGTGGTACTCGTTATACTGAGTTACTACG
TGCTCACTTTGGAGTAACTCCACAGGATTATCGTTTACAACGTCCTGATATATTGGTGGAGGTTCGACCCTTGTTAATGTTAATCCGA

>000185F|arrow

CGATGTTTTTTACGTCTTGATAAATTGGTTTGCTAGAACTAGGAGCTGACCTGATTTAGCAAGTTTTGTTAACTCGTTTGAATAACGAGATTGTGC
CAAAGCTTGAGCAGCTTGAGCACTTGATGTTTTGCCTATTTCATTTTTCATGAAAGTATCGGCAAGTATTTGTTTATATTGAGCTCTAATATTTGGA
TTTTCATCCAATTTATTTAACGTATCAGCACGTACATTATCTGTTTGATTACTTGTGAGTTCTGTTTGAGCTTCAATTTGTTTAGATTGAGCAATAGC
TTGATTTGCTTGCGCAACCGTTTGATAAGCTTGGGTTCCGGACGTAGTTGCATTACCGAGAACATTTTGCATCTGGGCCATAGCTCCAGCTGGTG
TTGTGGCTCCGCCTTGTGAATACGCAAGCATGGGATTTAACCCAGCTTTTTTCATATCTTCAACTGCTCGTTGATATGATGTTCCAGACATTTCAGC
TTGAAATCTCTGTTGATTACTCGCTTGTTCTGCACTCGCTGCGTTTTGACTTTGTGTTCCAAAGTATTGTCCTGCTGCACCTATTGCTGCAGGTGCA

AGTGCAGCTAAGGAGAGTCCCCCAGTAGCAGGGGCAGCTCCTATAGCTATAGCAGGGCCGATTAAATCGGCAATTCCGTCAAATAGTCCCATTA
GAAATGGTCTATTAAGCCAGGTACTGAATACATTGGCATTGGTCTTGCCATTTTGACATCAAAAAATGAGTCAAATAAGAATTGTTGGCCATTTG
CAGCTGCTCCAACGGCTACTACACGTGATACAGGAGGCGTATCTTGAATAAACGTATTATTCAAAGTAGGCGCAGCGGTAAATTTTTGAGCCAA
ATGCCAACCATCGATCGTGCCCGCCGATGTTGATTTGAACAAACCAGTAATTTTAGATGGTTTGTAACGATATTCCGCCCAGCGTTCTTGATATCC
AAAAACAGTATTGTCGGCTGCAGTATCTCCTGTTGCATAAATTTTTTTTGCAAAACAGATTGTTCGCCTAGAGTAGCAAATGCTGGGAAATAAAA
GTCATATCGTGTAGATCTAGACCACATACGGTCTAATCCTTGTTGATAAGTAAGATCTGCTCTAATGGATACTAATCCAATAATAACGCCATGCTC
AGTAAATGATTGAGTAAATCCATGATTATGAGCGAGAGCAGTACCCATAGCAGCAAGGTTGCCCAAAGGGGTAGTCGTTCCAGAAGCGTTTGTT
CCCGACGTTTGAGCAATCGGATTAACATTAATTGGTGTTGAACCGCCTCCAAGGTATTCAGGCCTTTGTAAACGGGCGTCGGGGAAATAACTCCA
AAGTGACTCCGGATAATTTCTGTGTATCGTGTTCCGCCACGTGCATCCCTTTCAAGTAATTTTTGAATTTGAAATGACTGTCTTAATTGATTGACAG
TTGCAGCAGTTGCTTCTGATAAGTCGGTATATAAATTAGAAACGTTATTTACTACACCAGCAGTATTAACACCATAAGCGTTGCCATATCTAGCTA
AAGCAGTCGTATTTCCAGGATCTGTTTGAATTACCGTAAATTTATCGTTTGATGTTGCGTCTCCTGAAATGGTATCCCATTTAATTGGAGCCGTAG
TACCTAAAGGTAAGGTGACACTCGCACCTTTTTGTGGCCATGGTAATGCTGACGTAAAGTAATCATGTCTTTTACCACGACGTTTTAGCACATAGT
TTGAGGAAGTATCAGGGCCATCGCCCTTATCAACTACTGCGCTTGTTTGTAAATTTTCATCTCGGAACCATTCGTTCCAGATAAGATTGTATGCAC
GTGGCCAAAAGGCACAGTGCGTAATAGTTCGGCCAGTATCAATTTGGCCTACTGTTGGTAAGCCCATATAGTCTTGAAGGCTGCCTACGGCATA
ACCATCTGTTGGGCTTGTTTGTGTTGGGACAATATAAGATATTGAGTCTGTTGGATTTTCTTGTTGACCCATAAATTTTTGCCAGTTATTCCATATA
AGGCGATTGGGTACAAAGAAAAAGAAAGAATCCATAATCATGTTATCCATGATTGGATATAAAGGCGTTGCTAGACGGGCAAATGCCGTCATTT
TTAAGTTGAAAGTGTCCCAGGGAGCACTTCATCAACATATACAGGAATTAGATAGCCCGCATCGAAAGTCGTTTTATGTGTTTTTGAGCATCGAA
TTTACTACGTGGTATATCGGCTCTAGGTACCATCGCGAAGCGGTGAGTATTAACTGACTGATTGCGGTGCATGTTTTTCCTTAGTGTTGTTCCGGG
GGAAAGATAAATCTCTTTTCCCCTCGGTTGTTTTATTTAAGTTTAACTTGTTTTCCTAATGATAGGAGTTTTGGTTGTTCATGTAAATCGAATAACC
CAGTTGAATCGTCAAATGTTCCGAATTCATATAGATCGAATCATCAGGGTGATTAAAGAGTTGATTTTCAGTATCAGAACGATTAATTTCATCTGA
AAAAGAGCGTATAGCTACTCCAGAGGAAGGTACGAACATTGGTCGTGCATATGCTTCAGCAGCACGGTCTTTTACGGAAGCGAGGATAAGTTTC
ATTATTTCCTAAGTAAGGTTACGTTTTAATAGTTGAAGTTTTGCCATAGTGACTTGTTCTTTTGCAGATAGTCGTTCTGGTGTATTGTCTTCGGAAT
TAAATTTAGCTTATTTTCCCGCATGTAAAGTAATTCGTCATACTCATAAGGTTGGTCAATTTTAAACATTTTGTCATAGTATTTTGGTGGTTTGACC
TTTTACCTCTAAGTATTACGTAGTCTTGCGGGTATATATCCGAAGTATATTTATATAAAAGTCTTTACCGATTCCCGGTTTTAAAGACATTTTATTAT
ATTCCGGCTTTAAGTCTAAATATTCGCCGGTTTCAGGGTGTATGCGTTTGTAATGAGATTCCGCATCTTTCCCTGTTTGTTTTTTCAATGTATCTAG
CCACGTAGGCGGCTGATTCAAAAGTAACATCTCCAATGGTGGTATAACCAAATGGCCAGAGAGCTTCAAGTTCTGCGGATCTATATAACATAGA
ACCAGAGGCAGTCCTTTTCCATAATTTTTTATCAGGAAAGTCGTATCCGAAGATACAGGCATGGAAGTGGGGTCTTGCGAAGAGTTCACCATATT
CTCCAGCCATGTAATAGCGGATTGTAAGTCCTCTTTTGCGAGAGTTTTTCTAAGTCTTTTAAGGAACAATTGAAAGTCTTTGTGATCCAAAGAGCC
ATCGCTTGGGAGATGTGTATTGTCATATGTGAGTGTTATGAATGAGTTGTTTTATGCAATTGGGCTTCGTGCATGCACCGAATAGCCCATTGTCTT
GATCGTTCTAGACGGCATCCAACGCACTGCCCACAGGGCAGGTCTAAAGATCGAACGATGTCATGTTTCCGAACTTCGTCGAAAACAATTGATTT
GTCAAAGCATTGATATGCTTTGAGAGGGTGATAACAAGGCATGTGAGGTGCCTGAAGACTTTATTAGAGTCTCCAGCCTCCACGCATTGGCGAG
TTTCGCATATTTGCGTGTTTTGTATGTGAAGCATGTTTACGGAAAGTCCTAGCGGACTTTTGCTTATTTGCTGGTCTGCGACGCATCATTTTTCTGT
CCTTGTTTATCGTGTTTTGTGGTTTGGTGTCACCTAGCACAGTTACATCAAGTAGGTAACTGTGCTTCCGAGGTTCCAACAGGGGTTGAAACCTCG
GTTTGGGCTGGTTTTACCAGTCCCATTTTTTCAGCTTCGCTGCGATTTTCAGGGTTTTCTAGAAAATCTATTAGATTCGCGGGATCATTAGCGAATC
GTTCACGAATATTGGCTGGTAAAGCCATAAATTCGTTGTCTGAAGCCATAATCTGGTTCAGAGCAGAGTGGTAGTCATAGACACCACTAAAGTC
GCCATATTGAGGCGTTAAAGGAGTTTGAGGAATAAGTCCGGTCATACCGAACTTTTCCATAATATTATTGATATCACATTCTTCAGCAAATTGCTG
CTGAGTCAGAGTTGCATCCTCACAATGCAGCCCTGACTCATTTGACGCAGCAATCGTGTCGTAATTGTACGGAGTACGAAGAAATGGGGCTGTA
ATCTTTGTCATTTTCATTCCATTGGTGGTTGGTTATTTGTACGATTTTGTTGAATCGGTTGACCTCGATAGTTATCGATGTATCGTTTTGCGCCAGA
TGCGCTATACGCATCTTTGG

>000124F|arrow

ACCAACCACCAATGGAATGAAAATGACAAAGATTACAGCCCCATTTCTTCGTACTCCGTACAATTACGACACGATTGCTGCGTCAAATGAGTCAG
GGCTGCATTGTGAGGATGCAACTCTGACTCAGCAGCAATTTGCTGAAGAATGTGATATCAATAATATTATGGAAAAGTTCGGTATGACCGGACT
TATTCCTCAAACTCCTTTAACGCCTCAATATGGCGACTTTAGTGGTGTCTATGACTACCACTCTGCTCTGAACCAGATTATGGCTTCAGACAACGA
ATTTATGGCTTTACCAGCCAATATTCGTGAACGATTCGCTAATGATCCCGCGAATCTAATAGATTTTCTAGAAAACCCTGAAAATCGCAGCGAAG
CTGAAAAAAATGGGACTGGTAAAACCAGCCCAAACCGAGGTTTCAACCCCTGTTGGAACCTCGGAAGCACAGTTACCTACTTGATGTAACTGTGC
TAGGTGACACCAAACCACAAAAACACGATAAACAAGGACAGAAAAAATGATGCGTCGCAGACCAGCAAATAAGCAAAAGTCCGCTAGGACTTT
CCGTAAACATGCTTCACATACAAAACACGCAAATATGCGAAACTCGCCAATGCGTGGAGGCTGGAGACTCTAATAAAGTCAGGCACCTCACATG
CCTTGTTATCACCCTCTCAAAGCATCAATGCTTTGACAAATCAATGTTTTCGACGAAGTTCGGAAACATGACATCGTTCGATCTTTAGACCTGCCCT
GTGGGCAGTGCGTTGGATGCCGTCTAGAACGATCAAGACAATGGGCTATTCGGTGCATGCACGAAGCCCAATTGCATAAAAACAACTCATTCAT
AACACTCACATATGACAATACACATCTCCCAAGCGATGGCTCTTTGGATCACAAAGACTTTCAACTGTTCCTTAAAAGACTTAGAAAAACTCTCGC
AAAAAGAGGACTTACAATCCGCTATTACATGGCTGGAGAATATGGTGAACTCTTCGCAAGACCCACTTCCATGCCTGTATCTTCGGATACGACTT

TCCTGATAAAAATTATGGAAAAGGACTGCCTCTGGTTCTATGTTATATAGATCCGCAGAACTTGAAGCTCTCTGGCCATTTGGTTATACCACCATT
GGAGATGTTACTTTGAATCAGCCGCCTACGTGGCTAGATACATAATGAAAAAACAAACAGGGAAAGATGCGGAATCTCATTACAAACGCATACA
CCCTGAAACCGGCGAATATTTAGACTTAAAGCCGGAATATAATAAAATGTCTTTAAAACCGGGAATCGGTAAAGACTTTTATATAAAATATACTT
CGGATATATACCGCAAGACTACGTAATACTTAGAGGTAAAAAGGTCAAACCACCAAAATACTATGACAAAATGTTTAAAATTGACCAACCTTATG
AGTATGACGAATTACTTTACATGCGGGAAAATAATGCTAAATTTAATTCCGAAGACAATACACCAGAACGACTATCTGCAAAAGAACAAGTCACT
ATGGCAAAACTTCAACTATTAAAACGTAACCTTACTTAGGAAAATAATGAAACTTATCCTCGCTTCCGTAAAAGACCGTGCTGCTGAAGCATATG
CACGACCAATGTTCGTACCTTCCTCTGGAGTAGCTATACGCTCTTTTTCAGATGAAATTAATCGTTCTGATACTGAAAATCAACTTTTAATCACCCT
GATGATTCGATCTATATGAATTCGGAACATTTGACGATTCAACTGGGTTATTCGATTTACATGAACAACCAAAACTCCTATCATTAGGAAAACAAG
TTAAACTTAAATAAAACAACCGAGGGGAAAAGAGATTTATCTTTCCCCGGAACAACACTAAGGAAAAACATGCACCGCAATCAGTCAGTTAATA
CTCACCGCTTCGCGATGGTACCTAGAGCCGATATACCACGTAGTAAATTCGATGCTCAAAAAACACATAAAACGACTTTCGATGCGGGCTATCTA
ATTCCTGTATATGTTGATGAAGTGCTCCCTGGGGACACTTTCAACTTAAAAATGACGGCATTTGCCCGTCTAGCAACGCCTTTATATCCAATCATG
GACATGATTATGGATTCTTTCTTTTTCTTTGTACCCAATCGCCTTATATGGAATAACTGGCAAAAATTTATGGGTCAACAAGAAAATCCAACAGAC
TCAATATCTTATATTGTCCCAACACAAACAAGCCCAACAGATGGTTATGCCGTAGGCAGCCTTCAAGACTATATGGGCTTACCAACAGTAGGCCA
AATTGATACTGGCCGAACTATTACGCACTGTGCCTTTTGGCCACGTGCATACAATCTTATTGGAACGAATGGTTCCGAGATGAAAATTTACAAAC
AAGCGCAGTAGTTGATAAGGGCGATGGCCCTGATACTTCCTCAAACTATGTGCTAAAACGTCGTGGTAAAAGACATGATTACTTTACGTCAGCAT
TACCATGGCCACAAAAAGGTGCGAGTGTCACCTTACCTTTAGGTACTACGGCTCCAATTAAATGGGATACCATTTCAGGAGACGCAACATCAAAC
GATAAATTTACGGTAATTCAAACAGATCCTGGAAATACGACTGCTTTAGCTAGATATGGCAACGCTTATGGTGTTAATACTGCTGGTGTAGTAAA
TAACGTTTCTAATTTATATACCGACTTATCAGAAGCAACTGCTGCAACTGTCAATCAATTAAGACAGTCATTTCAAATTCAAAAATTACTTGAAAG
GGATGCACGTGGCGGAACACGATACACAGAAATTATCCGGAGTCACTTTGGAGTTATTTCCCCAGACGCCCGTTTACAAAGGCCTGAATACCTT
GGAGGCGGTTCAACACCAATTAATGTTAATCCGATTGCTCAAACGTCGGGAACAAACGCTTCTGGAACGACTACCCTTTGGGCAACCTTGCTGCT
ATGGGTACTGCTCTCGCTCATAATCATGGATTTACTCAATCATTTACTGAGCATGGCGTTATTATTGGATTAGTATCCATTAGAGCAGATCTTACTT
ATCAACAAGGATTAGACCGTATGTGGTCTAGATCTACACGATATGACTTTTATTTCCCAGCATTTGCTACTCTAGGCGAACAATCTGTTTTGCAAA
AAGAAATTTATGCAACAGGAGATACTGCAGCCGACAATACTGTTTTTGGATATCAAGAACGCTGGGCGGAATATCGTTACAAACCATCTAAAATT
ACTGGTTTGTTCAAATCAACATCGGCGGGCACGATCGATGGTTGGCATTTGGCTCAAAAATTTACCGCTGCGCCTACTTTGAATAATACGTTTATT
CAAGATACGCCTCCTGTATCACGTGTAGTAGCCGTTGGAGCAGCTGCAAATGGCCAACAATTCTTATTTGACTCATTTTTTGATGTCAAAATGGCA
AGACCAATGCCAATGTATTCAGTACCTGGCTTAATAGACCATTTCTAATGGGACTATTTGACGGAATTGCCGATTTAATCGGCCCTGCTATAGCTA
TAGGAGCTGCCCCTGCTACTGGGGGACTCTCCTTAGCTGCACTTGCACCTGCAGCAATAGGTGCAGCAGGACAATACTTTGGAACACAAAGTCA
AAACGCAGCGAGTGCAGAACAAGCGAGTAATCAACAGAGATTTCAAGCTGAAATGTCTGGAACATCATATCAACGAGCAGTTGAAGATATGAA
AAAAGCTGGGTTAAATCCCATGCTTGCGTATTCACAAGGCGGAGCCACAACACCAGCTGGAGCTATGGCCAGATGCAAAATGTTCTCGGTAATG
CAACTACGTCCGGAACCCAAGCTTATCAAACGGTTGCGCAAGCAAATCAAGCTATTGCTCAATCTAAACAAATTGAAGCTCAAACAGAACTCACA
AGTAATCAAACAGATAATGTACGTGCTGATACGTTAAACAAATTGGATGAAAATCCAAATATTAGAGCTCAATATAAAAAATACTTGCCGATACT
TTCATGAAAAATGAAATAGGCAAAACATCAAGTGCTCAAGCTGCTCAAGCTTTGGCACAATCTCGTTATTCAAACGAGTTAACAAAACTTGCTAA
ATCAGGGTCAGCTCCTAGTTCTAGCAAACCAATTTATCAAGACGTAAAAAACATCGCCAAAGATGCGTATAGCGCATCTGGCGCAAAACGATAC
ATCGATAACTATCGAGGTCAACCGATTCAACAAAATCGTACAAATA

>000150F|arrow

GAAACTCGCCAATGCGTGGAGGCTGGAGGACTCTAATAAAGTCCCAGGCACCTCACATGCCTTGTTATCACCCTCTCAAAGCATTCAATGCTTTG
ACAAATCAATTGTTTTCGACGAAGTTCGGAAACATGACATCGTTCGATCTTTAGACCTGCCTGTGGCAGTGCGTTGGATGCCGTCTAGAACGATC
AAGACAATGGGCTATTCGGTGCATGCACGAAGCCCAATTGCATAAAAACAACTCATTCATAACACTCACATATGACAATACACATCTCCCAAGCG
ATGGCTCTTTGGATCACAAAGACTTTCAATTGTTCCTTAAAAGACTTAGAAAAACTCTCGCAAAAAGAGGACTTACATCCGCTATTACATGGCTG
GAGAATATGGTGAACTCTTCGCAAGACCCCACTTCCATGCCTGTATCTTCGGATACGACTTTCCTGATAAAAAATTATGGAAAAGGACTGCCTCT
GGTTCTATGTTATATAGATCCGCAGAACTTGAAGCTCTCTGGCCATTTGGTTATACCACCATTGGAGATGTTACTTTCGAATCAGCCGCCTACGTG
GCTAGATACATAATGAAAAAACAAACAGGGAAAGATGCGGAATCTCATTACAAACGCATACACCCTGAAACCGGCGAATATTTAGACTTAAAGC
CGGAATATAATAAAATGTCTTTAAAACCGGGAATCGGTAAAGACTTTTATATAAAATATACTTCGGATATATACCCGCAAGACTACGTAATACTTA
GAGGTAAAAAGGTCAAACCACCAAAATACTATGACAAAATGTTTAAAATTGACCAACCTTATGAGTATGACGAATTACTTTACATGCGGGAAAAT
AATGCTAAACTTAATTCCGAAGACAATACACCAGAACGACTATCTGCAAAAGAACAAGTCACTATGGCAAAACTTCAACTATTAAACGTAACCTT
ACTTAGGAAAATAATGAAACTTATCCTCGCTTCCGTAAAAGACCGTGCTGCTGAAGCATATGCACGACCAATGTTCGTACCTTCCTCTGGAGTAG
CTATACGCTCTTTTTCAGATGAAATTAATCGTTCTGATACTGAAAATCAACTCTTTAATCACCCTGATGACTTCGATCTATATGAATTCGGAACATT
TGACGATTCAACTGGGTTATTCGATTTACATGAACAACCAAAACTCCTATCATTAGGAAAACAGTTAAACTTAAATAAAACAACCGAGGGGAAAA
GAGATTTATCTTTCCCCCGGAACAACACTAAGGAAAAACATGCACCGCAATCAGTCAGTTAATACTCACCGCTTCGCGATGGTACCTAGAGCCGA
TATACCACGTAGTAAATTCGATGCTCAAAAAACACATAAAACGACTTTCGATGCGGGCTATTAATTCCTGTATATGTTGATGAAGTGCTCCCTGG
GGACACTTTCAACTTAAAAATGACGGCATTTGCCCGTCTAGCAACGCCTTTATATCCAATCATGGATAACATGATTATGGATTCTTTCTTTTTCTTT

GTACCCAATCGCCTTATATGGAATAACTGGCAAAAATTTATGGGTCAACAAGAAAATCCAACAGACTCAATATCTTATATTGTCCCAACACAACA
AGCCCAACAGATGGTTATGCCGTAGGCAGCCTTCAAGACTATATGGGCTTACCAACAGTAGGCCAAATTGATACTGGCCGAACTATTACGCACT
GTGCCTTTTGGCCACGTGCATACAATCTTATCTGGAACGAATGGTTCCGAGATGAAAATTTACAAACAAGCGCAGTAGTTGATAAGGGCGATGG
CCCTGATACTTCCTCAAACTATGTGCTAAAACGTCGTGGTAAAAGACATGATTACTTTACGTCAGCATTACCATGGCCACAAAAAGGTGCGAGTG
TCACCTTACCTTTAGGTACTACGGCTCCAATTAAATGGGATACCATTTCAGGAGACGCAACATCAAACGATAAATTTACGGTAATTCAAACAGATC
CTGGAAATACGACTGCTTTAGCTAGATATGGCAACGCTTATGGTGTTAATACTGCTGGTGTAGTAAATAACGTTTCTAATTTATATACCGACTTAT
CAGAAGCAACTGCTGCAACTGTCAATCAATTAAGACAGTCATTTCAAATTCAAAAATTACTTGAAAGGGATGCACGTGGCGGAACACGATACAC
AGAAATTATCCGGAGTCACTTTGGAGTTATTTCCCCAGACGCCCGTTTACAAAGGCCTGAATACCTTGGAGGCGGTTCAACACCAATTAATGTTA
ATCCGATTGCTCAAACGTCGGGAACAAACGCTTCTGGAACGACTACCCCTTTGGGCAACCTTGCTGCTATGGGTACTGCTCTCGCTCATAATCAT
GGATTTACTCAATCATTTACTGAGCATGGCGTTATTATTGGATTAGTATCCATTAGAGCAGATCTTACTTATCAACAAGGATTAGACCGTATGTGG
TCTAGATCTACACGATATGACTTTTATTTCCCAGCATTTGCTACTCTAGGCGAACAATCTGTTTTGCAAAAAGAAATTTATGCAACAGGAGATACT
GCAGCCGACAATACTGTTTTTGGATATCAAGAACGCTGGGCGGAATATCGTTACAAACCATCTAAAATTACTGGTTTGTTCAAATCAACATCGGC
GGGCACGATCGATGGTTGGCATTTGGCTCAAAAATTTACCGCTGCGCCTACTTTGAATAATACGTTTATTCAAGATACGCCTCCTGTATCACGTGT
AGTAGCCGTTGGAGCAGCTGCAAATGGCCAACAATTCTTATTTGACTCATTTTTTGATGTCAAAATGGCAAGACCAATGCCAATGTATTCAGTAC
CTGGCTTAATAGACCATTTCTAATGGGACTATTTGACGGAATTGCCGATTTAATCGGCCCTGCTATAGCTATAGGAGCTGCCCCTGCTACTGGGG
GACTCTCCTTAGCTGCACTTGCACCTGCAGCAATAGGTGCAGCAGGACAATACTTTGGAACACAAAGTCAAAACGCAGCGAGTGCAGAACAAGC
GAGTAATCAACAGAGATTTCAAGCTGAAATGTCTGGAACATCATATCAACGAGCAGTTGAAGATATGAAAAAAGCTGGGTTAAATCCCATGCTT
GCGTATTCACAAGGCGGAGCCACAACACCAGCTGGAGCTATGGCCCAGATGCAAAATGTTCTCGGTAATGCAACTACGTCCGGAACCCAAGCTT
ATCAAACGGTTGCTCAAGCAAATCAAGCTATTGCTCAATCTAAACAAATTGAAGCTCAAACAGAACTCACAAGTAATCAAACAGATAATGTACGT
GCTGATACGTTAAACAAATTGGATGAAAATCCAAATATTAGAGCTCAATATAAACAAATACTTGCCGATACTTTCATGAAAAATGAAATAGGCAA
AACATCAAGTGCTCAAGCTGCTCAAGCTTTGGCACAATCTCGTTATTCAAACGAGTTAACAAAACTTGCTAAATCAGGGTCAGCTCCTAGTTCTAG
CAAACCAATTTATCAAGACGTAAAAAACATCGCCAAAGATGCGTATAGCGCATCTGGCGCAAAACGATACATCGATAACTATCGAGGTCAACCG
ATTCAACAAAATCGTACAAATAACCAACCACCAATGGAATGAAAATGACAAAGATTACAGCCCCATTTCTTCGTACTCCGTACAATTACGACACG
ATTGCTGCGTCAAATGAGTCAGGGCTGCATTGTGAGGATGCAACTCTGACTCAGCAGCAATTTGCTGAAGAATGCGATATTAATAATATTATGG
AAAAGTTTGGTATGACCGGACTTATTCCTCAAACTCCTTTAACGCCTCAATATGGCGACTTTAGTGGTGTCTATGACTACCACTCTGCTCTGAACC
AGATTATGGCTTCAGACAACGAATTTATGGCTTTACCAGCCAATATTCGTGAACGATTCGCTAATGATCCCGCGAATCTAATAGATTTTCTAGAAA
ACCCTGAAAATCGCAGCGAAGCTGAAAAAATGGGACTGGTAAAACCAGCCCAAACCGAGGTTTCAACCCCTGTTGGAACCTCGGAAGCACAGTT
ACCTACTTGATGTAACTGTGCTAGGTGACACCAAACCACAAAAACACGATAAACAAGGACAGAAAAAATGATGCGTCGCAGACCAGCAAATAA
GCAAAAGTCCGCTAGGACTTTCCGTAAACATGCTTCACATACAAAACACGCAAATATGC

>000067F|arrow

TAATAGTTCGGCCAGTATCAATTTGGCCTACTGTTGGTAAGCCCATATAGTCTTGAAGGCTGCCTACGGCATAACCATCTGTTGGGCTTGTTTGTG
TTGGGACAATATAAGATATTGAGTCTGTTGGATTTTCTTGTTGACCCATAAATTTTTGCCAGTTATTCCATATAAGGCGATTGGGTACAAAGAAAA
AGAAAGAATCCATAATCATGTTATCCATGATTGGATATAAAGGCGTTGCTAGACGGGCAAATGCCGTCATTTTTAAGTTGAAAGTGTCCCCAGG
GAGCACTTCATCAACATATACAGGAATTAGATAGCCCGCATCGAAAGTCGTTTTATGTGTTTTTTGAGCATCGAATTTACTACGTGGTATATCGGC
TCTAGGTACCATCGCGAAGCGGTGAGTATTAACTGACTGATTGCGGTGCATGTTTTTCCTTAGTGTGTTCCGGGGGAAAGATAAATCTCTTTTCC
CCTCGGTTGTTTTATTTAAGTTTAACTTGTTTTCCTAATGATAGGAGTTTTGGTTGTTCATGTAAATCGAATAACCCAGTGAATCGTCAAATGTTCC
GAATTCATATAGATCGAAATCATCAGGGTGATTAAAGAGTTGATTTTCAGTATCAGAACGATTAGTTCATCTGAAAAAGAGCGTATAGCTACTCC
AGAGAAGGTACGAACATTGGCGTGCATATGCTTCAGCAGCACGGTCTTTACGGAAGCGAGGATAAGTTTCATTATTTCCTAAGTAAGGTTACGT
TTTAATAGTTGAAGTTTGCCATAGTGACTTGTTCTTTTGCAGATAGTCGTTCTGGTGTATTGTCTTCGGAATTAAATTTAGCATTATTTGCCCGCAT
GTAAAGTAATTCGTCATACTCATAAGGGTTGGTCAATTTAAACATTTTTGTCATAGTATTTTGGTGGTTTGACCTTTTTACCTCTAAGTATTACGTA
GTCTTGCGGGTATATATCCGAAGTATATTTTATATAAAAGTCTTTACCGATTCCCGGTTTTAAGACATTTTATTATATCCGGCTTTAAGTCTAAATA
TTCGCCGGGTTTCAGGTGTATGCGTTTGTAATGAGATTTCCGCATCTTTCCCTGTTTGTTTTTTCATTATGTATCTAGCCACGTAGGCGGCTGATTC
AAAAGTAACATCTCCAATGGTGGTATAACCAAATGGCCAGAGAGCTTCAAGTTCTGCGGATCTATATAATAGGAACCAGAGGCAGTCCTTTTCCA
TAATTTTTTATCAGGAAAGTCGTATCCGAGATACAGGCATGGAAGTGGGGTCTTGCGAAGAGTTCACCCATATTCTCCAGCCATGTAATAGCGGA
TTGTAAGTCCTCTTTGCGAGAGTTTTTCTAAGTCTTTTAAGGAACAATTGAAAGTCTTTGTGATCCAAAGAGCCATCGCTTGGGAGATGTGTATTG
TCATATGTGAGTGTATGAGTTGTTTTTATGCAATTGGGCTTCGTGCATGCACCGAATAGCCCATTGTCTTGATCGTTCTAGACGGCATCCAACGCA
CTGCCCACAGGGCAGGTCTAAAGATCGAACGATGTCATGTTTCCGAACTTCGTCGAAAACAATTGATTTGTCAAAGCATTGAATGCTTTGAGAGG
GTGATAACAAGGCATGTGAGGTGCCTGAAGACTTTATTAGAGTCTCCAGCCTCCACGCATTGGCGAGTTTCGCATATTTGCGTGTTTTGTATGTG
AAGCATGTTTACGGAAAGTCCTAGCGGACTTTTGCTTATTTGCTGGTCTGCGACGCATCATTTTTCTGTCCTTGTTTATCGTGTTTTTGTGGTTTGG
TGTCACCTAGCACAGTTACATCAAGTAGGTAACTGTGCTTCCGAGGTTCCAACAGGGGTTGAAACCTCGGTTTGGGCTGGTTTTACCAGTCCCAT
TTTTTCAGCTTCGCTGCGATTTTCAGGGTTTTCTAGAAAATCTATTAGATTCGCGGGATCATTAGCGAATCGTTCACGAATATTGGCTGGTAAAGC

CATAAATTCGTTTGTCTGAAGCCATAATCTGGTTCAGAGCAGAGTGGTAGTCATAGACACCACTAAAGTCGCCATATTGAGGCGTTTAAAGGAGT
TTGAGGAATAAGTCCGGTCATACCGAACTTTCCATAATATTATTGATATCACATTCTTCAGCAAATTGCTGCTGAGTCAGAGTTGCATCCTCACAA
TGCAGCCTGACTCATTTGACGCAGCAATCGGTCGTAATTGTACGGAGTACGAAGAAATGGGGCTGTAATCTTGTCATTTCATTCCATTGGTGGTT
GGTTATTTGTACGATTTTGTTGAATCGGTTGACCTCGATAGTTATCGATGTATCGTTTTGCGCCAGATGCGCTATACGCATCTTTGGCGATGTTTTT
TACGTCTTGATAAATTGGTTTGCTAGAACTAGGAGCTGACCCTGATTTAGCAAGTTTTGTTAACTCGTTTGAATAACGAGATTGTGCCAAAGCTTG
AGCAGCTTGAGCACTTGATGTTTTGCCTATTTCATTTTTCATGAAAGTATCGGCAAGTATTTGTTTATATGAGCTCTAATATTTGGATTTTCATCCA
ATTTATTTAACGTATCAGCACGTACATTATCTGTTTGATTACTTGTGAGTTCTGTTTGAGCTTCAATTTGTTTAGATTGAGCAATAGCTTGATTTGCT
TGCGCAACCGTTTGATAAGCTTGGGTTCCGGACGTAGTTGCATTACCGAGAACATTTTGCATCTGGGCCATAGCTCCAGCTGGTGTTGTGGCTCC
GCCTTGTGAATACGCAAGCATGGGATTTAACCCAGCTTTTTCATATCTTCAACTGCTCGTTGATATGATGTTCCAGACATTTCAGCTTGAAATCTCT
GTTGATTACTCGCTTGTTCTGCACTCGCTGCGTTTTGACTTGTGTTCCAAAGTATTGTCCTGCTGCACCTATTGCTGCAGGTGCAAGTGCAGCTAA
GGAGAGTCCCCCAGTAGCAGGGGCAGCTCCTATAGCTATAGCAGGGCCGATTAAATCGGCAATTCCGTCAAATAGTCCCATTAGAAATGGTCTA
TTAAGCCAGGTACTGAATACATTGGCATTGGTCTTGCCATTTTGACATCAAAAAATGAGTCAAATAAGAATTGTTGGCCATTTGCAGCTGCTCCA
ACGGCTACTACACGTGATACAGGAGGCGTATCTTGAATAAACGTATTATTCAAAGTAGGCGCAGCGGTAAATTTTTGAGCCAAATGCCAACCAT
CGATCGTGCCCGCCGATGTTGATTTGAACAAACCAGTAATTTTAGATGGTTTGTAACGATATTCCGCCCAGCGTTCTTGATATCCAAAAACAGTAT
TGTCGCTGCAGTATCTCCTGTTGCATAAATTTCTTTTTGCAAAACAGATTGTTCGCCTAGAGTAGCAAATGCTGGGAAATAAAAGTCATATCGTGT
AGATCTAGACCACATACGGTCTAATCCTTGTTGATAAGTAAGATCTGCTCTAATGGATACTAATCCAATAATAACGCCATGCTCAGTAAATGATTG
AGTAAATCCATGATTATGAGCGAGAGCAGTACCCATAGCAGCAAGGTTGCCCAAAGGGGTAGTCGTTCCAGAAGCGTTTGTTCCCGACGTTTGA
GCAATCGGATTAACATTAATTGGTGTTGAACCGCCTCCAAGGTATTCAGGCCTTTGTAAACGGGCGTCTGGGGAAATAACTCCAAAGTGACTCC
GGATAATTTCTGTGTATCGTGTTCCGCCACGTGCATCCCTTTCAAGTAATTTTTGAATTTGAAATGACCTGTCTTAATTGATTGACAGTTGCAGCA
GTTGCTTCTGATAAGTCGGTATATAAATTAGAAACGTTATTTACTACACCAGCAGTATTAACACCATAAGCGTTGCCATATCTAGCTAAAGCAGTC
GTATTTCCAGGATCTGTTTGAATTACCGTAAATTTATCGTTTGATTGTTGCGTCTCCTGAAATGGTATCCCATTTAATTGGAGCCGTAGTACCTAAG
GTAAGGTGACACTCGCACCTTTTTGTGGCCATGGTAATGCTGACGTAAAGTAATCATGTCTTTTACCACGACGTTTTAGCACATAGTTTGAGGAA
GTATCAGGGCCATCGCCCTTATCAACTACTGCGCTTGTTTGTAAATTTCATCTCGGAACCATTCGTTCCAGATAAGATTGTATGCACGTGGCCAAA
AGGCACAGTGCG

>000197F|arrow

CAGCATTTGTACTATTAGACAAAGTATTAGAAGCTGAATTTAATTCTCTTAAAGCACCAACAGCAGTTTGAAAACACTATAACCAGCAGTACCATT
GCCAGCTGGTATGTCAGAATAAATATTAGCACGTGTTCCTAACGGTAAAGAAACAGAAGCACCCTTCTGTGGCCAAGGCAATGCACCAGTAAAA
TCTTTACGCTTACCACGTCTCAAAAGTGTATAGTTAGCAGGAACATCACCTGAATCACCAAGATTTAACGTTACAGAATTCTGTAAGTTTTCATCTC
TAAACCATTCGTTATAAATCTCATTATAAGCACGTAACGGCAAAACATTATGCGTTACTGTATTAGCGCCAGTAATCTGACCAGCAGTAGGTAAA
CCAAAATAATCAAAATTGAACAAACAGCATAACCACCAGCAGGACTAGTAATAGTAGGCACTACAAAAAATAGAATCACCAGGATTCGTTTGTT
CACCCATAAACTTTGGCCAATTGTTCCAAAGTAAACGATTAGGTACAAAGAAAAAGAAAGTATCAAGATGCAAGTTGTCCATCACAGGAAATAA
CGGTGTGGCCAAACGTGCAAATGCAGTCATCTTTACACGATGAGTATCGCCAGGCAATACTTCATCACAATAAATAGGAACTAAAAACCAGCATC
AAACGTGGTTTTATGAGCATATTGAGTATTAAAACTAGAGCGGGGAATGTCCGCTTTAGGAACCATAGCAATGAATGCGTACTTACTGACTTATT
GCGAAACATAAACATCTCCCGTAGTTCCGTACCACTCTTTCGAGTGATACGGTATAAAAAAACCTTACTCGCCTTCGCGAATCATTACATCTTTTG
CACGAGCAATCACTTAGGGCTTTCCAGTAGTTCAAAAACACCAGTGGCATCGTCAAAAGACCCAAGAACATATGAAAATCATCAGGATGTTTATA
CAACTGATTATCTTCGCTAGCTCGATTGACTTCATCCTGAAACTGACGTACTGCAACACCTTCAGATGCAACATAATGGACGACCAAAAGCATCTG
CAGCAGTATCCTTAATAGAAACAATAACCATCTTCATAAAACTCCTTAAATAGTACGTTTTAACAATGACAACTTAGCCAACGCAACTTTTTCCTTA
ACAGCAAGTCGCTCAAGCGTGTTGTCCTCATGCCTAGATCGACCTTCCATCTCTCTGGCAAACTGAATCATATCGAATTCTTCAGGAAACTTCAAC
TTAAATTTATTATCATAAAACCGTGGTGGACGGCACTTTTTGCCACGCACCACAACGTGGTCTGACGTATAAACGTCTGACATGTACTTATCTAAC
CACGATTGCCCGATACCGGGCTTCAATGACATCTTATTAAATTCTGGCTTACGCTGAATTATCTCACCATCTCTAAATCACAATATTGATAATGGG
CACCCGCATCAACCACTTCGTGGTTTTCATTGACAGTAACCCCATTAATCTTCTTCATAATATATCTTGCAACATAAGCAGCAGACTCAAAGTAACA
TCACCAATTGTAGAATAGCCAAACGGCCACAATTCTTCCAAAATCTCTGACGTGTAGAGGATAGAGCCAGTCTGCGTTCTTTTAAATATTTTCTTA
TCCGGAAAATCAAGACCAAACAGACAAGCATGGAAATGAGGACGATCAAAAGATTCACCATATTCACCTGCCATATAAAACGTATCGTTTTCCCA
GTAAAACGCTTACGTAACCGCTTCATAAAAAGCTGATAATCATTGTAATCCATGACATATCCTTAGGACAATGCTCTGGAGCATATGTCAAAGTA
ATAAAACAATTACTAGTATGCATTTGTGCCTCATGCATACAACGAATCGCCCACTGACGTGAGCGTTCAAGGCGACAACCAACACACTGACCACA
AGGCAATGATAGGTACGGACTACATCCGCACCCGTATTTCCCGCCAAATTATAGACCTGTCACTGCATTGATAAGCCGTTAAGGCTTATAACAGG
CCATAATTACAAACGATAGCCACCACGCTGGGGAGCGTGTCTCATATTAATTGACTTCGTCTTACTAGCAGTTCTGCGAAATGACTTTGCAGATTT
ATATTTTTTTGGCTTTCTTCGTAACATGATGAACTCCGTAGTTAAAATAGTGGTTTGGTGTCACCTAGCACAGTTACATCAAGTAGTAACTGTGCT
GGCCTCAGGATTTCATCCTTCGCCTTAGGTGTTTCTGTAGAAACGATGGGTTCAACCACAGGTTGTCCATCATAAGACCCAATTGAATCGCTTCAT
CACGATTCTGGTCGTTCTCAAGGAACTCCAATAATTTGACAGGATCATGGTCAAATCGGACTCTTAATTTCGCTGGCAGAGCCATGAAATCGTCC
ATAGTTTTAATTTGATTCAACGCAGAATGGTAATCAGTAACACCACTAAAATCGCCGTATTGAGGCGATACAGGGCCGTTGGAAGTTCCCCTGTA

ACCCCGAAACGTTCAATGATGACATTAATGTCACATTCATCTTTCATATGCTGTTGAGCAAGACTTGGGTCTTGACACAGCAACGCATCGTTTACC
GATGCTTCATCTTTATCATAGTTGTACGGATTACGTACAAATGGCAAATTCGCTTTACTCATTATTTGACTCCAATTCCCCAAGGGTTAGTTGATTA
GGATTATTTTTGAGTTTATCAAAATTTCTTTCGAAGCATCGATACCCCTAGACCAATAGCTGGGATGGAGCCAATAGCCTCTTAGTATTATATGAC
TGAGCGGATGTTAAATCCGCAGTCGTATTCTTTAAAGCAATATCAGCCAAAATACGTTTATTTTCGGCTGTAATATTAGGAGCAGTAAGCAACTT
ATTAACAGTATCAGCCCTAGTATTAGCGGTACCAGCTTCAGTAGCTTCAGTCTGGGCGATAATCTGCTTTTCTGTTTCTGATGCATTACGAATCTG
TTGCATCATAGAAGCAGTATTAATAGCTGAATTAGTTGCATTACCTAAAACATTTTCCATAGTAGCAGTTGAACCAGATGGGGTAGAAGCACCAC
CACGTTGATAGGCTAACATAGGAGATAACCAGCAGCTTCTAAATCTTTAACAGCACGCTGGTAACTTGTTCCACTCATATCGGCTTGAAAATCCAT
TTGCCTCTGAGCCATCTCCTGATTAGCTTTATTCAGAAGTAGAACCTAAATAAGAACCAACAGCGCCCAAAGCAGTACCGACACCAGAGTAAAGA
AACTAGAAGCTGAAGACAGCTTAGAACCACATTAGTAACCGCATCAAGTTCCACCAAACATAAACTAACGCCCTTCGGTTGTTTCCTCACTACTCC
TTACGGAGTAGTCGAGGTTATATAAAACATTAGAAATGATCAATTAGACCAGGTACGCTGTACATAGGCATAGGTCTGGCCATCTGACAATCAA
AGAAAGCATCCATTAAAAATTGCTGACCATTTGCTGCAGCTCCGACCGCCGTAGTACGAGCAACTGGAGGGGTCTCTTGAATAAACGTTGAATTC
ACGTAGGAAAGAAGTAAATCGCTGTCCATAATGCCAAGCATCAATAGTGCCAGCAGAAGTAGACCTAAAGAAACCAGTAATTTGAGAAGGTTT
GTAACGATACTCCGCCCAACGTTCTTGATAACCAAATACATCATTGTCGGCTGCAGTACCTTGAACATAAATTTCTTTGTTCAAAATAGCTTGCTCA
CCCAAAGTGGCAAATACTGGGAAATAAATCATATCGTGTAGACCTAGACCACATCTTAGGAAGACCCTGTTGATATGTGAGGTCAGCACGTACA
GAAACTAATCCGATAATGTATCCATGTTCTTGAGCAGCATACGTAAGCCGTGTCCCTGAGCCAATGCAGTACCCATTGCAGCAAGGTTACCTTGC
GGAGTAGCAGAACCAGTAACCGACGTTGCAGAAGTCTGAGCAATCGGATTAACATTAACAAGGGTCGAACCTCCACCAATATATTCAGGACGTT
GTAAACGATAATCCTGTGGAGTTACTCCAAAGTGAGCACGTAGTAACTCAGTATAACGAGTACCACCACGTGCATCGCGCTCCAATAACTTCTGA
ATCTGGAAAGATTGACGAAGTTGGTTAATAGTCGCAGCAGTAGCAGTAGACAAATCAGCGTATAACTGATTAGTAGCAACAC

>000161F|arrow

CTTTGGAACACAAAGTCAAAACGCAGCGAGTGCAGAACAAGCGAGTAATCAACAGAGATTTCAAGCTGAAATGTCTGGAACATCATATCAACGA
GCAGTTGAAGATATGAAAAAAGCTGGGTTAAATCCCATGCTTGCGTATTCACAAGGCGGAGCCACAACACCAGCTGGAGCTATGGCCCAGATGC
AAAATGTTCTCGGTAATGCAACTACGTCCGGAACCCAAGCTTATCAACGGTTGCGCAAGCAAATCAAGCTATTGCTCAATCTAAACAAATTGAAG
CTCAAACAGAACTCACAAGTAATCAAACAGATAATGTACGTGCTGATACGTTAAAAAATTGGATGAAAATCCAAATATTAGAGCTCAATATAAAC
AAATACTGCCGATACTTTCATGAAAAATGAAATAGGCAAAACATCAAGTGCTCAAGCTGCTCAAGCTTTGGCACAATCTCGTTATTCAAACGAGT
TAACAAAACTTGCTAAATCAGGGTCAGCTCCTAGTTCTAGCAAACCAATTTATCAAGACGTAAAAAACATCGCCAAAGATGCGTATAGCGCATCT
GGCGCAAAACGATACATCGATAACTATCGAGGTCAACCGATTCAACAAAATCGTACAAATAACCAACCACCAATGGAATGAAAATGACAAAGAT
TACAGCCCCATTTCTTCGTACTCCGTACAATTACGACACGATTGCTGCGTCAAATGAGTCAGGGCTGCATTGTGAGGATGCAACTCTGACTCAGC
AGCAATTTGCTGAAGAATGCGATTAATAATATTATGGAAAAGTTTGGTATGACCGGACTTATTCCTCAAACTCCTTTAACGCCTCAATATGGCGAC
TTTAGTGGTGTCTATGACTACCACTCTGCTCTGAACCAGATTATGGCTTCAGACAACGAATTTATGGCTTTACCAGCCAATATTCGTGAACGATTC
GCTAATGATCCCGCGAATCTAATAGATTTTCTAGAAAACCTGAAAATCGCAGCGAAGCTGAAAAAATGGGACTGGTAAAACCAGCCCAAACCGA
GGTTTCAACCCCTGTTGGAACCTCGGAAGCACAGTTACCTACTTGATGTAACTGTGCTAGGTGACACCAAACCACAAAAACACGATAAACAAGG
ACAGAAAAAAATGATGCGTCGCAGACCAGCAAATAAGCAAAAGTCCGCTAGGACTTTCCGTAAACATGCTTCACATACAAAACACGCAAATATGC
GAAACTCGCCAATGCGTGGAGGCTGGAGACTCTAATAAAGTCCAGGCACCTCACATGCCTTGTTATCACCCTCTCAAAGCATTTCAATGCTTTGA
CAAATCAATTGTTTTCGACGAAGTTCGGAAACATGACATCGTTCGATCTTTAGACCTGCCCTGTGGGCAGTGCGTTGGATGCCGTCTAGAACGAT
CAAGACAATGGGCTATTCGGTGCATGCACGAAGCCCAATTGCATAAAAACAACTCATTCATAACACTCACATATGACAATACACATCTCCCAAGC
GATGGCTCTTTGGATCACAAAGACTTTAAACGTTCCTTAAAAGACTTAGAAAAACTCTCGCAAAAAGAGGACTTACAATCCGCTATTACATGGCT
GGAGAATATGGTGAACTCTTCGCAAGACCCCACTTCCATGCCTGTATCTTCGGATACGACTTTCCTGATAAAAAATTATGGAAAAGGACTGCCTC
TGGTTCTATGTTATATAGATCCGCAGAACTTGAAGCTCTCTGGCCATTTGGTTATACCACCATTGGAGATGTTACTTTCGAATCAGCCGCCTACGT
GGCTAGATACATAATGAAAAAACAAACAGGGAAAGATGCGGAATCTCATTACAAACGCATACACCCTGAAACCGGCGAATATTTAGACTTAAAG
CCGGAATATAATAAAATGTCTTTAAAACCGGGAATCGGTAAAGACTTTTATATATATACTTCGGATATATACCCGCAAGACTACGTAATACTTAGA
GGTAAAAAGGTCAAACCAAAAATACTATGACAAAATGTTTAAAATTGACCAACCTTATGAGTATGACGAATTACTTTACATGCGGGAAAATAATG
CTAAACTTAATTCCGAAGACAATACACCAGAACGACTATCTGCAAAAGAACAAGTAACTATGGCAAAACTTCAACTATTAAAACGTAACCTCACT
TAGGAAAATAATGAAACTTATCCTCGCTTCCGTAAAAGACCGTGCTGCTGAAGCATATGCACGACCAATGTTCGTACCTTCTCTGGAGTAGCTAT
ACGCTCTTTTTCAGATGAAATTAATCGTTCTGATACTGAAAATCAACTTTTAATCACCCTGATGACTTCGATCTATATGAATTCGGAACTTTGACGA
TTCAACTGGGTTATTCGATTTACATGAACAACCAAAACTCCTATCATTAGGAAAACAAGTTAAACTTAAATAAAACAACCGAGGGGAAAAGAGAT
TTATCTTTCCCCGGAACAACACTAAGGAAAAACATGCACCGCAATCAGTCAGTTAATACTCACCGCTTCGCGATGGTACCTAGAGCCGATATACC
ACGTAGTAAATTCGATGCTCAAAAAACACATAAAACGACTTTCGATGCGGGCTATTATTCCTGTATATGTTGATGAAGTGCTCCCTGGGGACACT
TTCAACTTAAAAATGACGGCATTTGCCCGTCTAGCAACGCCTTTATATCCAATCATGGACAACATGATTATGGATTCTTTCTTTTTCTTGTACCCAA
TCGCCTTATATGGAATAACTGGCAAAAATTTATGGGTCAACAAGAAAATCCAACAGACTCAATATCTTATATTGTCCCAACAAAACAAGCCCAAC
AGATGGTTATGCCGTAGGCAGCCTCAAGACTATATGGGCTTACCAACAGTAGGCCAAATTGATACTGGCCGAACTATTACGCACTGTGCCTTTTG
GCCACGTGCATACAATCTTATTGGAACGAATGGTTCCGAGATGAAAATTTACAAACAAGCGCAGTAGTTGATAAGGGCGATGGCCCTGATACTT

CCTCAAACTATGTGCTAAAACGTCGTGGTAAAAGACATGATTACTTTACGTCAGCATTACCATGGCCACAAAAAGGTGCGAGTGTCACCTTACCT
TTAGGTACTACGGCTCCAATTAAATGGGATACCATTTCAGGAGACGCAACATCAAACGATAAATTTACGGTAATTCAAACAGATCCTGGAAATAC
GACTGCTTTAGCTAGATATGGCAACGCTTATGGTGTTAATACTGCTGGTGTAGTAAATAACGTTTCTAATTTATATACCGACTTATCAGAAGCAAC
TGCTGCAACTGTCAATCAATTAAGACAGTCATTTCAAATTCAAAAATTACTTGAAAGGGATGCACGTGGCGGAACACGATACACAGAAATTATCC
GGAGTCACTTTGGAGTTATTTCCCCAGACGCCCGTTTACAAAGGCCTGAATACCTTGGAGGCGGTTCAACACCAATTAATGTTAATCCGATTGCT
CAAACGTCGGGACAAACGCTTCTGGAACGACTACCCCTTTGGGCAACCTTGCTGCTATGGGTACTGCTCTCGCTATAATCATGGATTTACTCAATC
ATTTACTGAGCATGGCGTTATTATTGGATTAGTATCCATTAGAGCAGATCTTACTTATCAACAAGGATTAGACCGTATGTGGTCTAGATCTACACG
ATATGACTTTTATTTCCCAGCATTTGCTACTCTAGGCGAACAATCTGTTTTGCAAAAAGAAATTTATGCAACAGGAGATACTGCAGCCGACAATAC
TGTTTTTGGATATCAAGAACGCTGGGCGGAATATCGTTACAAACCATCTAAAATTACTGGTTTGTTCAAATCAACATCGGCGGGCACGATCGATG
GTTGGCATTTGGCTCAAAAATTTACCGCTGCGCCTACTTTGAATAATACGTTTATTCAAGATACGCCTCCTGTATCACGTGTAGTAGCCGTTGGAG
CAGCTGCAAATGGCCAACAATTCTTATTTGACTCATTTTTTGATGTCAAAATGGCAAGACCAATGCCAATGTATTCAGTACCTGGCTTAATAGACC
ATTTCTAATGGGACTATTTGACGGAATTGCCGATTTAATCGGCCCTGCTATAGCTATAGGAGCTGCCCCTGCTACTGGGGGACTCTCCTTAGCTG
CACTTGCACCTGCAGCAATAGGTGCAGCAGGACAATA

>000223F|arrow

GAATTTTTCATTTATGAAAAAGGGAGAGGGCGTCGGGCAAGCCCTTTCTCGAGTGTATGATGATAATATAAAAAAAAAAAAAGTTTTTTTTTTT
TATATTATTATTATACCGAGCATTAATAGTATATTGTCCATAATTACTATCCATTCCTAATATTAGTACATATTATCAGTAAAAACTAATAATATATA
TTAATCAGAATCACTATCATCCCAATATATACTATTATTATCATTTAATCCATTATATCTATTATTATCCCAATCCATATGTACTCTACTATTATTATT
AGACAATGGAGTAGGAGGTATTTTATTAGGATTAACCGCTTTAGAATTAACCGCTTTAGCATTCTTAAGTGGTTGTAAATCTTTGTTATCATCAAT
AATCCAAATACGCCATCTATCAGCAGACATTAAACCAAAATCAATAAGTTCATTACAAAATACCCAAACTCTAGGTGGATGAAATTCCCAATCTTT
AAATTTGTTTCTAGTATCGTAAACATATCCAGATTTAATTTGTTCGAAAGCACACATAAATCCATTCATTTTATTTTTAGTTAAAGCACGCGGTAAG
TCAAAAATAATAATTTTAGGATCTAAACAGTTTTTAGCTTCTAATATATCACAACAACTAGCAACAATTCTATCACAATCATTAATAGTAGGCATAC
TAATACCAAATTTACCTATTCTACAGAATGTAGAAATAGTAGATTTACCTTTATCACCATCAGGGTCATAAACTAGATCAATAATTCTAGAATTTCT
AAATTTCTTTTGTCCAGATGTTAATATTTTTTTTTGCATAGGATATAAATCATTATATCTTATTTTATCTAAAGTATATGGTTTAAATATAGTAGGAT
CATCTAAAAATTGATTGTAATAAATTTCGTCATCCATTAAATCATCATCATAAGACATAACATCCGTCCATGGACCTTCTAATCTACTTTGTACTTTT
AATACATAAAACATTGAACCTTTTCTATGCTCTACTTCAGTAGTTGGTTTTAAGTAATTAGGTACATTGATATTAAAAGATTTAAATAATTTTTTTA
AAGGTTCTTCAGTTATTTTTTTTGTTAGATTCATTCTACCTTGCCAATGTATATAACCACCATCACCTTCTTCAAGTTGAAAGCACCATTTTTTACAA
TAGTGTTTTAAAAATTTAATTAATTCAATTTTAGTAATCCATTCTTTGTTGTATCTAATTTCCCATGTTGCACAAGAATTCATATATATAATAATAAT
AGAAAAAAATTATTTTTAAATGATTTTTCCAAAGGTTATAAACAGTAAATATTCAAAATACGATAAGTCAACATAATAGTTAAAAACACTTTCCAG
ACTTTCCAAACGTTTGCCGGAATAAAATCAATAAGAATAAACACTTTCCAGAAATTCCTGAGTTTCTTTAAGGAATATAAGGTGTAGATATTTTGA
AAAAATATCTGTTTTTCCAGAGCGAACGCTCGCCACCTTATATTCCGACCGTCAGTCAACAACGGTACGGGATGTGAAGAGTTGGAGTATTCACT
ATGAAATAGTGCATTCTCCAAACTCTGAACGATTACAAATAGTCAGTTAACAATTCAAATTAAATAGTCAGTTAACAATCCTAAAATTAAATAGTA
AAAATCTTCCAATGATTCTTATAATTAGGTGATTATTTGTAACATTACTATGAAAAATATGTCACGACATATTTTTTATATTGAATTATATTTTATTA
AGTTTTTTACGACTAGAAAACAACGGTTG

>000205F|arrow

CAAAATGTTTAAAATTGACCAACCTTATGAGTATGACGAATTACTTTACATGCGGGAAAATAACGCTAAACTTAATTCCGAAGACAATACACCAG
AACGACTATCTGCAAAAGAACAAGTAACTATGGCAAAACTTCAACTATTAAAACGTAACCTCACTTAGGAAAATAATGAAACTTATCCTCGCTTCC
GTAAAAGACCGTGCTGCTGAAGCATATGCACGACCAATGTTCGTACCTTCCTCTGGAGTAGCTATACGCTCTTTTCAGATGAAATTAATCGTTCTG
ATACTGAAAATCAACTTTTTAATCACCCTGATGACTTCGATCTATATGAATTCGGAACTTTTGACGATTCAACTGGGTTATTCGATTTACATGAACA
ACCAAAACTTCTATCATTAGGAAAACAAGTTAAACTTAAATAAAACAACCGAGGGGAAAAGAGATTTATCTTTCCCCCGGAACAACACTAAGGA
AAAACATGCACCGCAATCAGTCAGTTAATACTCACCGCTTCGCGATGGTACCTAGAGCCGATATACCACGTAGTAAATTCGATGCTCAAAAAACA
CATAAAACGACTTTCGATGCGGGCTATTTAATTCCTGTATATGTTGATGAAGTGCTCCCTGGGGACACTTTCAACTTAAAAATGACGGCATTTGCC
CGTCTAGCAACGCCTTTATATCCAATCATGGACAACATGATTATGGATTCTTTCTTTTTCTTTGTACCCAATCGCCTTATATGGAATAACTGGCAAA
AATTTATGGGTCAACAAGAAAATCCAACAGACTCAATATCTTATATTGTCCCAACTCAAACAAGCCCAACAGATGGTTATGCCGTAGGCAGCCTT
CAAGACTATATGGGCTTACCAACAGTAGGCCAAATTGATACTGGCCGAACTATTACGCACTGTGCCTTTTGGCCACGTGCATACAATCTTATTTG
GAACGAATGGTTCCGAGATGAAAATTTACAAACAAGCGCAGTAGTTGATAAGGGCGATGGCCCTGATACTTCCTCAAACTATGTGCTAAAACGT
CGTGGTAAAAGACATGATTACTTTACGTCAGCATTACCATGGCCACAAAAAGGTGCGAGTGTCACCTTACCTTTAGGTACTACGGCTCCAATTAA
ATGGGATACCATTTCAGGAGACGCAACATCAAACGATAAATTTACGGTAATTCAAACAGATCCTGGAAATACGACTGCTTTAGCTAGATATGGCA
ACGCTTATGGTGTTAATACTGCTGGTGTAGTAAATAACGTTTCTAATTTATATACCGACTTATCAGAAGCAACTGCTGCAACTGTCAATCAATTAA
GACAGTCATTTCAAATTCAAAAATTACTTGAAAGGGATGCACGTGGCGGAACACGATACACAGAAATTATCCGGAGTCACTTTGGAGTTATTTCC
CCAGACGCCCGTTTACAAAGGCCTGAATACCTTGGAGGCGGTTCAACACCAATTAATGTTAATCCGATTGCTCAAACGTCGGGAACAAACGCTTC

TGGAACGACTACCCCTTTGGGCAACCTTGCTGCTATGGGTACTGCTCTCGCTCATAATCATGGATTTACTCAATCATTTACTGAGCATGGCGTTAT
TATTGGATTAGTATCCATTAGAGCAGATCTTACTTATCAACAAGGATTAGACCGTATGTGGTCTAGATCTACACGATATGACTTTTATTTCCCAGC
ATTTGCTACTCTAGGCGAACAATCTGTTTTGCAAAAAGAAATTTATGCAACAGGAGATACTGCAGCCGACAATACTGTTTTTGGATATCAAGAAC
GCTGGGCGGAATATCGTTACAAACCATCTAAAATTACTGGTTTGTTCAAATCAACATCGGCGGGCACGATCGATGGTTGGCATTTGGCTCAAAAA
TTTACCGCTGCGCCTACTTTGAATAATACGTTTATTCAAGATACGCCTCCTGTATCACGTGTAGTAGCCGTTGGAGCAGCTGCAAATGGCCAACAA
TTCTTATTTGACTCATTTTTTGATGTCAAAATGGCAAGACCAATGCCAATGTATTCAGTACCTGGCTTAATAGACCATTTCTAATGGGACTATTTGA
CGGAATTGCCGATTTAATCGGCCCTGCTATAGCTATAGGAGCTGCCCCTGCTACTGGGGGACTCTCCTTAGCTGCACTTGCACCTGCAGCAATAG
GTGCAGCAGGACAATACTTTGGAACACAAAGTCAAAACGCAGCGAGTGCAGAACAAGCGAGTAATCAACAGAGATTTCAAGCTGAAATGTCTG
GAACATCATATCAACGAGCAGTTGAAGATATGAAAAAAGCTGGGTTAAATCCCATGCTTGCGTATTCACAAGGCGGAGCCACAACACCAGCTGG
AGCTATGGCCCAGATGCAAAATGTTCTCGGTAATGCAACTACGTCCGGAACCCAAGCTTATCAAACGGTTGCTCAAGCAAATCAAGCTATTGCTC
AATCTAAACAAATTGAAGCTCAAACAGAACTCACAAGTAATCAAACAGATAATGTACGTGCTGATACGTTAAACAAATTGGATGAAAATCCAAAT
ATTAGAGCTCAATATAAACAAATACTTGCCGATACTTTCATGAAAAATGAAATAGGCAAAACATCAAGTGCTCAAGCTGCTCAAGCTTTGGCACA
ATCTCGTTATTCAAACGAGTTAACAAAACTTGCTAAATCAGGGTCAGCTCCTAGTTCTAGCAAACCAATTTATCAAGACGTAAAAAACATCGCCAA
AGATGCGTATAGCGCATCTGGCGCAAAACGATACATCGATAACTATCGAGGTCAACCGATTCAACAAAATCGTACAAATAACCAACCACCAATG
GAATGAAAATGACAAAGATTACAGCCCCATTTCTTCGTACTCCGTACAATTACGACACGATTGCTGCGTCAAATGAGTCAGGGCTGCATTGTGAG
GATGCAACTCTGACTCAGCAGCAATTTGCTGAAGAATGCGATATTAATAATATTATGGAAAAGTTTGGTATGACCGGACTTATTCCTCAAACTCCT
TTAATCGCCTCAATATGGCGACTTTAGTGGTGTCTATGACTACCACTCTGCTCTGAACCAGATTATGGCTTCAGACAACGAATTTATGGCTTTACC
AGCCAATATTCGTGAACGATTCGCTAATGATCCCGCGAATCTAATAGATTTTCTAGAAAATCCTGAAAATCGCAGCGAAGCTGAAAAAAATGGGA
CTGGTAAAACCAGCCCAAACCGAGGTTTCAACCCCTGTTGGAACCTCGGAAGCACAGTTACCTACTTGATGTAACTGTGCTAGGTGACACCAAAC
CACAAAAACACGATAAACAAGGACAGAAAAAATGATGCGTCGCAGACCAGCAAATAAGCAAAAGTCCGCTAGGACTTTCCGTAAACATGCTTCA
CATACAAAACACGCAAATATGCGAAACTCGCCAATGCGTGGAGGCTGGAGACTCTAATAAAGTCCCCAGGCACCTCACATGCCTTGTTATCACCC
TCTCAAAGCATTTCAATGCTTTGACAAATCAATTGTTTTCGACGAAGTTCGGAAACATGACATCGTTCGATCTTTAGACCTGCCCTGTGGGCAGTG
CGTTGGATGCCGTCTAGAACGATCAAGACAATGGGCTATTCGGTGCATGCACGAAGCCCAATTGCATAAAAACAACTCATTCATAACACTCACAT
ATGACAATACACATCTCCCAAGCGATGGCTCTTTGGATCACAAAGACTTTCAACTGTTCCTTAAAAGACTTAGAAAAACTCTCGCAAAAAGAGGA
CTTACAATCCGCTATTACATGGCTGGAGAATATGGTGAACTCTTCGCAAGACCCCACTTCCATGCCTGTATCTTCGGATACGACTTTCCTGATAAA
AAATTATGGAAAAGGACTGCCTCTGGTTCTATGTTATATAGATCCGCAGAACTTGAAGCTCTCTGGCCATTTGGTTATACCACCATTGGAGATGTT
ACTTTCGAATCAGCCGCCTACGTGGCTAGATACATAATGAAAAAACAAACAGGGAAAGATGCGGAATCTCATTACAAACGCATACACCCTGAAA
CCGGCGAATATTTAGACTTAAAGCCGGAATATAATAAAATGTCTTTAAAACCGGGAATCGGTAAAGACTTTTATATAAAATATACTTCGGATATA
TACCCGCAAGACTACGTAATACTTAGAGGTAAAAAGGTCAAACCACCAAAATACTATGA

>000122F|arrow

TTCTATATCACAGTATGTGTAATGCTCATCCTTTGTTATATGTTTGTTTTCTTCCATCTGGCCGTTGTATTTTTGCATAATATATCGAGCAACGTAAG
CAGCTGACTCGAATGTAACGTCTCCAATGGAGGAATAACCATGTGGCCAGAGGGTTGCAAGGTGGTCGGATGTATATATGAGAGAACCAGAGG
GAGTCCTTTTGAATAGTTTCTTATCATGAAAATCGTATCCGAAGATACAGGCATGGAAGTGAGGTCTGCCGAAACTTGTGCCGTATTCTCCAGCC
ATGTAGTAACGTAATTTTGCAGGTGCAATGGATTTTCTGAAGCGCTTAAGGAACTTTTGGAAGTCGCTTTTGACAAGCGATCCAGTTTGTGGAAG
GTTTTCATTGTTGTATGTGAGGGTTATAAAGCAGTTTTTTTTCGTGCAATTGGGCTTCATGCATGCAGCGCATGGCCCATTGACGTGATTTTTCTA
GCCTGCAGCCAACGCATTGGCCGCAGGGCAGTGAAATCTGACGATCATGCTCGTCAGATTCTTTGAATGCGACACGGCGATAAGATTTGCCGGT
CTTATAGTTTGTCTGATGCTTACTTAAATAAGCAGTCAGTGGGTGATAACAGGCCATGTGAGGTAGCCTGGCGCTTTATTAGAGCCGCCAGCCCC
CTCTTTGCGGGGCTTTTTGCATATTTGCAGCTTTAGTTTTTGAAGTGTTCCTACGGAACGATTTAGCGCTTTTGCGCTTGTTTGTTTGTTTTCTATAA
AGCATAATTTTTGGCCTCGTTTATCAGATATTTTTGGTGTTGGTGTCACCTAGCACAGTTACATCAAGTGGAGTAACTGTGCTTATCGCCATTCTTC
CGAAATGGCTCTTTCGACCAGGCCGAGTTTCTCGGCTTCTGGTCGATTTTTCTCGTCTTGCAAGAACTCAATCAGGTTTGCTGGTTCGTTTTCGAA
CCTAGCACGAATTTGAGCCGGTAAGTTATCAAATTCTTCCATAGCGTTCATTACCTTATTCAAGGCGCTATGATAGTCAGTAATTCCGCTGAAATC
GCCATATTGAGGCGGCAGCGGACTTTGAGGTAATAGGCCTGTAACGTTAAAACGTTCCAGGATAGTATTATATCGCATTCGTCTTTATAATGCTG
CTGAGCCAGAGTTGGCTCCTCACAAGCCAACCCTGACTCATTTGACGCAGCATCCGTGTCATAGTTGTATGCGGTTCTAAGTTTGATTGTTTTCAT
TTCATTTTTCCAAATGGTAGATATTTTTGATATTTATCATATTGGCTATGTAAATAGCCTTTAACATCTTGATAAATTGGTTTAGTACTGGATGGCG
CTGATCCAGTTTGCGCTAATCGCTCTAATTGGTCTGTATATGCACTTTGACTTAACGCTTGTCGCTGTTGTGCAGCTTGTAATGCACTTGATGTCCT
TAATTGCTCTATTTGGGCATCGCGAAGCTGACCAAATTTGCCGTATCCCGGCATCTGAGCAATTTCACGAGCTGTATTTGCTCGAGTATATGTTGC
CTGATCACGTGATAGATTTGTATCCGCGTCCGTTTTTTCGGCTTGCGATTGTGTCAGGATATTCTGAGTTTGTAATTGTTTAAAATTGGCGACTGC
CATGGCAGCTTCACGGGCTGAATTTCCAGCCTCACCTAGTGGATTTCCTACTGGAGCTTGCGCACCAGGCTGGACTTTGGCTCCGCCTTGTGAAT
AAGCCAGCATAGGGTTAAGACCTGCAGCCTTAAGATCCGCTACTGCGTCTTGATATGCAGTACGTCGCATACCATCTTGGAATTGCATCATTGCT
GCAGCTTGTTGTGCGCTGGCAGCGTTTTGTTCTTGACCACCAAAATAACTAGCGGCCGCACCTATTCCGGCTCCAATTAAGGAGCCGTAAGGTCC
AAAAGCGGCGCCAGATGCGGCGCCTGAGGCAGCGGCTTCTAGTGCCATTAGAAGTGGTCGATTAAGCCAGGTACAGAGTACATTGGCATTGGC

CGTGCTTTTTTAATATCAAAAAAGCTATCAAAAATGATTTGCTGGCCGTTGGCAGCTGCTCCGACCGCAAGGGTTCGAGAGACAGGTGGATTTTC
TTGAATAAACGTGTTATTCAAAGTTGGAAGTGTTGTAAACCGTTGGGCTAAATGCCAGCCGTCAATTGTTCCAGCTGCCGTACTACGGAACAGAC
TGGAAATTCGGCTTGGCATATAACGATATTCTGCCCAACGTTCTTGGTATCCAAACACTCCTGTATCGTTGGCATCGCCTCGTACGTAGATTTCTT
CATTTAATACTGCTTGTTCGCCCAAAGTGGCGAAAGCAGGGAAATAGAAATCATAACGTGTGGAACGATTCCACATGCGGTGCATACCTTGTTG
GTATGTTAAATCGGCTCGTACTGAAACGAGACCGATAATTACACCATGTTCAGTGCTTGAATAAGTAAAGCCATGATTGTGAGCGAGCCCAGTA
CCCATAGCGCCAAGTGTACCGAGAGGAGTAGTGGTCCCGGAAGCTCCGGTACCATTCGTCTGGGCGATTGGATTGATATTAATGTGTGTTGAAC
CGCCTCCAATGTATTCAGGCCGTTGGAGACGCGCATCGGGGCTAACGACCCCGAAATGGCTGCGGATAATTTCTGTGTATCGAGTTCCGCCACG
GGCGTCCCTTTCTAAAAGTTTTTGAATCTGGAAGCTTTGACGCAATTGATTAATTGTTGCAGCGGTTGCAGCTGATAAATCTGCATATAAACCGCC
ATTAGGATCATATGATTTTGCCAAGCCGTCAGCACCGCCAGTAATTTGACCAGTAACACCTACGTTAATAGCCTGGGCGGTTGCGTTTAATGTATT
TGTTCCAGCGTTATACAGTCTGGAAACAGGCGCATTGTTAGTGCGTAATATTGGGGCGGATGTTCCAAGCGGTAAAGTAACGCTTGCGCCCTTTT
GTGGCCAAGGTAATGCTGACGTGAAATAGTCTTTACGTTTTCCACGTCGTAATAATGTGTAGTTGGCTACGTTATCTGGACCATCGCCAGTATCT
ACAACTACTGAATTTTGAAGGTTTTCGTCCCGAAACCATTCGTTATAAATAAGGTTGTAAGCACGTGGCCAGAAGGCACAGTGACTTACGGTGCC
ACCAGCACCTACTTGGCCTACAGTTGGCAGACCCATATAATCTTGAAGGCTGCCAATAGCGTAACCACCAGCTGGGGTTACTTGTTGGGGCACTA
CGTACGAAATCGAGTCCGCAGGATTTGCCTGCTGCCCCATAAATTTTTGCCAATTTGACCAGACAAGTCTATTTGGCACAAAGAAGAAGAAGCTA
TCCAAATGCATATTGTCCATGATCGGATAGAGTGGTGTAGACAATCGCGCAAAGGCGGTCATGTTGCAGCGGAATGTATCGCCTGGCAACATTT
CGTCTACGAGTACAGGGACTAGGAAGCCAGCATCGAATGTAGTTTTATGTGTTGATTGACAGTCAAATGTAGAGCGGGGGATATCCGCTTTTGG
AATCATTGTGAACTGATGGACGTCTACCGACTTGTTACGATGCATTTTTTTGAGCTCCTAGGCCTAGTTGCGTGAGAAAAAGGGGTTTCCCCCTTT
TTACTCTACGCTTAGTTTTTATCAGTAATTTTGACTTGTTTCCCTAAGGATACAAGTTTGGGTTGTTCATGTAATTGGAACAAACCCGTATTATCGT
CGAATTCGCCTAATTCAAATAGGTCGAAATCGTCGGAATGGTTATAAAGTTGATTGTCATCGCTCTGGCGATTGACTTCGTCGCTAAAGCTCCGG
ATTGCTTCGCCGATAGAACGGACGAACATTGGACGACCGAACGCATCTGCTGCGCGGTCTTTAACGGTACAGAGTACTAATTTCATGAGGATTTC
CTAAGTGAGGTTACGTTTTAATAATTGAAGTTTGGCCTTTGTGACTTTTTCCTTTACGGCAAGTCTGGCATAGGTATTGTCTTCGTGGTTGAGTTTA
GCAGAAGTTTCACGTTTGTGGAGTAATTCTTCGTATTCATATGGATAATCTGATTTATATTTTTTGTCATAGTATTTTGGGGGTTTTACCCTTTTTCC
GTTGACTACAACGTAGTCATGGGGATATACGTCGGAACGATATTTTTTGTACCACTCAGCACCAATGCCTGGTTTAAGGCTCATATTGTTATATTC
TGGTAATAGCTTTATTAATTCCCCTGT

>000031F|arrow

ACCTTGCGGAGTAGCAGAACCAGTAACCGACGTTGCAGAAGTCTGAGCAATCGGATTAACATTAACAAGGGTCGAACCTCCACCAATATATTCA
GGACGTTGTAAACGATAATCCTGTGGAGTTACTCCAAAGTGAGCACGTAGTAACTCAGTATAACGAGTACCACCACGTGCATCGCGCTCCAATA
ACTTCTGAATCTGGAAAGATTGACGAAGTTGGTTAATAGTCGCAGCAGTAGCAGTAGACAAATCAGCGTATAACTGATTAGTAGCAACACCAGC
ATTTGTACTATTAGACAAAGTATTAGAAGCTGAATTTAATTCTCTTAAAGCACCAACAGCAGTTTGAAAAACACTATAACCAGCAGTACCATTGCC
AGCTGGTATGTCAGAATAAATATTAGCACGTGTTCCTAACGGTAAAGAACAGAAGCACCCTTCTGTGGCCAAGGCAATGCACCAGTAAAATAAT
CTTTACGCTTACCACGTCTCAAAAGTGTATAGTTAGCAGGAACATCACCTGAATCACCAAGATTTAACGTTACAGAATTCTGTAAGTTTTCATCTCT
AAACCATTCGTTATAAATCTCATTATAAGCACGTAACGGCAAAACATTATGCGTTACTGTATTAGCGCCAGTAATCTGACCAGCAGTAGGTAAAC
CAAAATAATCAAAAATTGAACAAACAGCATAACCACCAGCAGGACTAGTAATAGTAGGCACTACAAAAGAAATAGAATCACCAGGATTCGTTTG
TTCACCCATAAACTTTGGCCAATTGTTCCAAAGTAAACGATTAGGTACAAAGAAAAAGAAAGTATCAAGATGCAAGTTGTCCATCACAGGAAATA
ACGGTGTGGCCAAACGTGCAAATGCAGTCATCTTTACACGATGAGTATCGCCAGGCAATACTTCATCACAATAAATAGGAACTAAAAAACCAGC
ATCAAACGTGGTTTTATGAGCATATTGAGTATTAAAACTAGAGCGGGGAATGTCCGCTTTAGGAACCATAGCAAATGAATGCGTACTTACTGACT
TATTGCGAAACATAAACATCTCCCGTAGTTCCGTACCACTCTTTCGAGTGATACGGTATAAAAAAAACCTTACTCGCCTTCGCGAATCATTACATC
TTTTGCACGAGCAATCAACTTAGGGCTTTCCAGTAGTTCAAAAACACCAGTGGCATCGTCAAAAAGACCCAAATAGAACATATGAAAATCATCAG
GATGTTTATACAACTGATTATCTTCGCTAGCTCGATTGACTTCATCCTGAAACTGACGTACTGCAACACCTTCAGATGCAACATAAGCTGGACGAC
CAAAAGCATCTGCAGCAGTATCCTTAATAGAAACAATAACCATCTTCATAAAAACTCCTTAAATAGTACGTTTTAACAATGACAACTTAGCCAACG
CAACTTTTTCCTTAACAGCAAGTCGCTCAAGCGTGTTGTCCTCATGCCTAGATCGACCTTCCATCTCTCTGGCAAACTGAATCATATCGAATTCTTC
AGGAAACTTCAACTTAAATTTATTATCATAAAACCGTGGTGGACGGCACTTTTTGCCACGCACCACAACGTGGTCTGACGTATAAACGTCTGACA
TGTACTTATCTAACCACGATTGCCCGATACCGGGCTTCAATGACATCTTATTAAATTCTGGCTTACGCTGAATTATCTCACCAGTCTCTAAATCACA
ATATTGATAATGGGCACCCGCATCAACCACTTCGTGGTTTTCATTGACAGTAACCCCATTAATCTTCTTCATAATATATCTTGCAACATAAGCAGCA
GACTCAAAAGTAACATCACCAATTGTAGAATAGCCAAACGGCCACAATTCTTCCAAAATCTCTGACGTGTAGAGGATAGAGCCAGTCTGCGTTCT
TTTAAATATTTTCTTATCCGGAAAATCAAGACCAAACAGACAAGCATGGAAATGAGGACGATCAAAAGATTCACCATATTCACCTGCCATATAAA
AACGTATCGTTTTCCCAGTAAAACGCTTACGTAACCGCTTCATAAAAAGCTGATAATCATTGTAATCCAATGACATATCCTTAGGACAATGCTCTG
GAGCATATGTCAAAGTAATAAAACAATTACTAGTATGCATTTGTGCCTCATGCATACAACGAATCGCCCACTGACGTGAGCGTTCAAGGCGACAA
CCAACACACTGACCACAAGGCAATGATAGGGTACGGACTACATCCGCACCCGGTATTTCCCGCCAAATTATAGACCTGTCACTGCATTGATAAGC
CGTTAAGGGCTTATAACAGGCCATAATTACAAACGATAGCCACCACGCTGGGGAGCGTGTCTCATATTAATTGACTTCGTCTTACTAGCAGTTCT
GCGAAATGACTTTGCAGATTTATATTTGTTTACTGGCTTTCTTCGTAACATGATGAACTCCGTAGTTAAAATAGTGGTTTGGTGTCACCTAGCACA

GTTACATCAAGTAGAGTAACTGTGCTGGCCTCAGGATTTCATCCTTCGGCCTTAGGTGTTTCTGTAGAAACGATGGGTTCAACCACAGGTTGTCC
ATCAATAAGACCCAATTGAATCGCTTCATCACGATTCTGGTCGTTCTCAAGGAACTCCAATAATTTGACAGGATCATGGTCAAATCGGACTCTTAA
TTTCGCTGGCAGAGCCATGAAATCGTCCATAGTTGCGTTAATTTGATTCAACGCAGAATGGTAATCAGTAACACCACTAAAATCGCCGTATTGAG
GCGATACAGGGGCCGTTGGAAGTTCCCCTGTAACCCCGAAACGTTCAATGATGACATTAATGTCACATTCATCTTTCATATGCTGTTGAGCAAGA
CTTGGGTCTTGACACAGCAACGCATCGTTTACCGATGCTTCATCTTTATCATAGTTGTACGGATTACGTACAAATGGCAAATTCGCTTTACTCATTA
TTTGACTCCAATTCCCCAAGGGGTTAGTTGATTAGGATTATTTTTGAGTTTATCAAAAATTTCTTTCGAAGCATCGATACCCCTAGACCAAATAGCT
GGGGATGGAGCCAATAGCCTCTTAGTATTATATGACTGAGCGGATGTTAAATCCGCAGTCGTATTCTTTAAAGCAATATCAGCCAAAATACGTTT
ATTTTCGGCTGTAATATTAGGAGCAGTAAGCAACTTATTAACAGTATCAGCCCTAGTATTAGCGGTACCAGCTTCAGTAGCTTCAGTCTGGGCGA
TAATCTGCTTTTCTGTTTCTGATGCATTACGAATCTGTTGCATCATAGAAGCAGTATTAATAGCTGAATTAGTTGCATTACCTAAAACATTTTCCAT
AGTAGCAGTTGAACCAGATGGGGTAGAAGCACCACCACGTTGATAGGCTAACATAGGAGATAAACCAGCAGCTTCTAAATCTTTAACAGCACGC
TGGTAACTTGTTCCACTCATATCGGCTTGAAAATCCATTTGCCTCTGAGCCATCTCCTGATTAGCTTTATTAGCAGAAGTAGAACCTAAATAAGAA
CCAACAGCGCCCAAAGCAGTACCGACACCAGGAGTAAAGAAACTAGAAGCTGAAGACAGCTTAGAACCAACATTAGTAACCGCATCAAGTATTC
CACCAAACATAAACTAACGCCCTTCGGTTGTTTCCTCACTACTCCTTACGGAGTAGTCGAGGTTATATAAAACATTAGAAATGATCAATTAGACCA
GGTACGCTGTACATAGGCATAGGTCTGGCCATCTGACAATCAAAGAAAGCATCCATTAAAAATTGCTGACCATTTGCTGCAGCTCCGACCGCCGT
AGTACGAGCAACTGGAGGGGTCTCTTGAATAAACGTTGAATTCAACGTAGGAAGAGAAGTAAATCGCTGTCCATAATGCCAAGCATCAATAGTG
CCAGCAGAAGTAGACCTAAAGAAACCAGTAATTTGAGAAGGTTTGTAACGATACTCCGCCCAACGTTCTTGATAACCAAATACATCATTGTCGGC
TGCAGTACCTTGAACATAAATTTCTTTGTTCAAAATAGCTTGCTCACCCAAAGTGGCAAATACTGGGAAATAAAAGTCATATCGTGTAGACCTAG
ACCACATCTTAGGAAGACCCTGTTGATATGTGAGGTCAGCACGTACAGAAACTAATCCGATAATGTATCCATGTTCTTGAGCAGCATACGTAAAG
CCGTGTCCCTGAGCCAATGCAGTACCCATTGCAGCAAGGTT

>000033F|arrow

GCGAGGTGGTACACGCTATACAGAATTGTTACGTGCTCATTTTGGTGTAACTCCACAAGATTATCGTTTACAACGTCCTGAATATATTGGTGGTG
GTTCTACTTATGTCAACGTTAATCCTATTGCTCAAACGTCTGCTACTTCGATTTCTGGCGGTGCTACTCCGCTTGGTAACTTGGCTGCAATGGGTAC
TGCGTTGGCTAGTGGACATGGTTTTACGTATCATGCTCAAGAACATGGATACATAATTGGTTTGGTAAACGTAAGAGCTGATCTAACATATCAGC
AAGGTTTACCTAAGATGTGGTCTCGTGAGACACGTTATGATTTCTATTTCCCTGTATTTGCTCATTTAGGTGAACAGGCTGTTCTTAATAAGGAAA
TCTATGTTACTGGTACTTCAACTGATGATGATGTATTTGGATACCAGGAACGTTGGGCAGAGTACCGTTACAAACCTTCTCAGATTACAGGTTTGT
TTAAGTCGACTTCAGCTGGTACGATTGACCCTTGGCATTATGCTCAGAAGTTCACTTCGTTGCCGACTTTGAATTCTACGTTTATTCAAGAAACGC
CACCTATTGATCGTACTACTGCTGTTGGTGCATCTGCTAATGGTCAGCAGTTTTTGATGGATGCGTTTTTTGATTGTAAGATGGCTCGTCCAATGC
CGATGTATTCTGTACCTGGCTTGATTGACCATTTCTAATGTAATATAAGCTGGACTACTGGGAAACCAGTAGTCAGCAAACAAGCGGAGCGCGTT
AGTATGGGTATGTTTGATTCAGTTGCTGGTGGTTTATTTGATTTTGCTACTTCTGCTTGGAGTCAGAATGAAGAGCAAAAATATAATACTGCTGA
AGCGCAAGCAAATAGACGCTTTCAAGAAGATATGTCCAATACGCAATATCAGCGTATGGTTACTGATTTGAATAAGGCTGGTTTGAGTCCTATGC
TTGCATATTCTAAAGGTGGTTCTACTGTTCCGAGTGGTGCTACTGCATCTTCTACTTCTCCAGTTAAGTCTGATTTGAGTGGAGCTGTTAAGCGTG
ATGTTGAGAGTGATTTATTGCGTGAGCAGATGGAAGTTGCTAAATCTCAACGTGAATTAAATGTTCAGACTGCTAGGAAGGTTGCTGAAGAAGC
TGATTTAGCTTCTCAGCGTGTTTTACAAGAACCGGCTCGTTTTTATTTGGAACAGGCCGAGGCTGGTTCTCGTGTTAATGCTAATTCTGCATCAAC
AATTAAGACTGATATTGATGCTAAGAATAATTTAGAGTTACGAACACCGTCTAGCGATCCATATTGGTATCGCGATATTAAGAAAGGTAGTAAGT
CTATTTTTGATAAGTTTTTGGAAAAGAATAAGTCTTTTCCTTGGTCCTTGGAAGGTCGTAGAAAATGAGTAAAACGAATTTACCTTTTGTACGTAA
TCCGTACAATTATGATATGGCTCTTGTTTCACAAGAGACTGGTCTTGAATGTAAAGATCCGAGTTTGGCTCAACAACACATGAGAGACGAATGTG
ATATTAATATTATAGTTGAGCGTTTTGGCGTTACTGGGCAATTGCCCCAGGCGCCATTAGAGCCATCATATGGCGATTTTAGCGGTGTATCTGAC
TATCACACCGCATTGAATGCTATTCGTGCCTCTGATGAGGCTTTTATGGCTTTGCCAGCTAAGATTAGGGCTAAGTTTGATAACGATCCTAATGCT
TTGTTAAATTATTTACAGAATGAAGAGAATCGTGATGAAGCGATTCAGATTGGTCTTATTGACGGTAAACCTGTGGTTGAACCCGTCGTTTCTGC
AGTAGAAACACCTAAGCCAGACGCGTAAGCGGATGGCAGCACAGTTACTCTACTTGATGTAACTGTGCTAGGTGACACCAAAACCACATTATTA
ACTACGGAGTGCAATGTTATGAGCCTTTATAGAAAACCAATGAGCAAGCATGGCGCAGCTAAGAAGTTTCGTCGTGGCGTAAGCAAGACCAAG
AGCATTAATATGCGTACTTCACCGCAGCGCGGTGGTTTTAGACTGTAATTTATGGCGTGTTATAAGCCGTTAACGGCTTATCAATGCGCTGACAA
GTCTATTATTTGGCGGGAGATACCAGGGGCGGACGTAGTCCGTACCTTGTCATTGCCTTGTGGTCAGTGTGTTGGTTGTCGCCTTGAACGGTCCC
GTCAGTGGGCCGTTAGATGTATGCATGAGGCACAAATGCATACTAGTAATTGTTTTATTACTTTGACATATGCTCCAGAGCATTGTCCTAAGGAT
ATGTCTTTGCATTATGAAGATTTTCAATTGTTTATGAAACGATTGAGAAAACGCTATACTGGCAAGACTATTCGTTTTTATATGGCAGGTGAATAT
GGTGAGTCTTTCGATCGTCCTCATTTCCATGCTTGTATCTTTGGGCTTGATTTTGAAGATAAGAAGTTTTTCAAAAGAACGCAGACTGGGTCTATC
TTATATACGTCAAAGATACTTGAAGAACTTTGGCCGTATGGCTATAGTTCTATTGGTGATGTCAATTTTGAATCTGCTGCTTATGTTGCTCGATAT
ATTATGAAGAAGATTAACGGTAAAACCGTTAATGAGAACCACGAAGTGGTTGATGCAGATGCGCATTATCAGTATTGTGATTTAGATACTGGTG
AGATTATTCAGCGTAAGCCTGAATTTAATAAGATGTCTCTTAAGCCTGGCATTGGTCAGGCTTGGTTTGATAAGTTCATGTCAGACGTTTATACGA
CTGACTCTGTTGTGGTGCGTGGCAAAAAGTGCCGACCACCACGGTTTTATGATAATAAGTTTAAAGTATTGTTTCCAGAAGAATTTGATGGTATA
CAATATGCTCGTGAGCTAGAAGGTCGCTCACATTTTGAAGATAACACTTTAGAGCGTTTGGCTGTAAAGGAAAAAGTCGCTTTGGCTAAGTTATC

GCTTTTAAAACGTAAGATTTAAAGGAGTTTTTATGAAGATGGTTATTGTTTCTATTTTAGATACTGCAGCTGGTGCGTATGGTCGTCCAGCTTTTG
TTGCATCTGAGGGTGTTGCTGTTCGTCAGTTTCAGGACGAAGTTAATCGCGCTAGCGATGATAATCAGTTATATAAACACCCTGATGATTTTCAG
TTATATTATTTTGGTACGTTTGATGATAATTCTGGTACTATGGATTTGCTTGGTTCTCCCAAGCTTATTTCTAGAGCTAAGGATGTTATGATTCGCG
ATGGCGAGTAATGTTTTTTTTAAGCCGGATCACTAGCTTGCTAGTGGTTCGGAATACTTCGGGAGATTGTTATGCATCGTAATAAGTCAGTAAGC
TCACATAGTTTTGCTATGGTTCCTAAAGCGGAAATTCCGCGTTCTAGTTTTGATACGCAATATGCGCATAAAACTACTTTTGATGGTGGTTATCTA
GTTCCTATTTATTGTGATGAAGTGCTCCCAGGGGACATGCACAATGTAAAGGCTACAATGTTTGCCCGTTTGGCAACGCCTTTGTTTCCAGTTATG
GACAATTTGCATTTAGATACTTTCTTTTTCTTTGTTCCAAATAGATTAGTTTGGAACAATTGGGTTAAGTTTATGGGTGAGCAAGCGAACCCAGGT
GATTCTATTTCGTATGTAGTTCCACAGATTACTTCTACTGCTGGTGGTTATGCAGTAGGTTCTATTTTTGATCATTTTGGTTTGCCTACGGCTGGTC
AGATTACTGGTACTAATACGGTTACGCATAACGCGTTACCGTTACGTGCTTATAATTTGATTTATAACGAGTGGTTTAGGGATGAGAATTTACAA
AATTCTGTTACCGTTCACAAGGGTGACGGTCCTGATACTCCGAGTGATTATGCTATGGTTCGACGCGGTAAGCGTAAGGATTATTTTACTGGTGC
CTTGCCTTGGCCTCAGAAAGGCGATGCAGTTACGTTGCCTTTAGGCACGTCTGCTCCTATTAAGACGTCTGTTACTAATGATTCAACAACGTTGTC
ATTAGTTGACGGTGCTGGTGCTTTGAAGCAGATTTATGCTAATTCAACTACACATGCTGTGTATCGTAGTAATAACGCTGGTACTGCTGGTACTG
GTTTGTATGCCGATTTAAGTGATGCTACGGCTGCAACTATTAACCAGTTACGTCAGTCTTTCCAGATTCAGCGTTTGTTAGAGCGCGATGC

>000104F|arrow

GACCTTCCAAGGACCAAGGAAAAGACTTATTCTTTTCCAAAAACTTATCAAAAATAGACTTACTACCTTTCTTAATATCGCGATACCAATATGGAT
CGCTAGACGGTGTTCGTAACTCTAAATTATTCTTAGCATCAATATCAGTCTTAATTGTTGATGCAGAATTAGCATTAACACGAGAACCAGCCTCGG
CCTGTTCCAAATAAAAACGAGCCGGTTCTTGTAAAACACGCTGAGAAGCTAAATCAGCTTCTTCAGCAACCTTCCTAGCAGTCTGAACATTTAATT
CACGTTGAGATTTAGCAACTTCCATCTGCTCACGCAATAAATCACTCTCAACATCACGCTTAACAGCTCCACTCAAATCAGACTTAACTGGAGAAG
TAGAAGATGCAGTAGCACCACTCGGAACAGTAGAACCACCTTTAGAATATGCAAGCATAGGACTCAAACCAGCCTTATTCAAATCAGTAACCATA
CGCTGATATTGCGTATTGGACATATCTTCTTGAAAGCGTCTATTTGCTTGCGCTTCAGCAGTATTATATTTTTGCTCTTCATTCTGACTCCAAGCAG
AAGTAGCAAAATCAAATAAACCACCAGCAACTGAATCAAACATACCCATACTAACGCGCTCCGCTTGTTTGCTGACTACTGGTTTCCCAGTAGTCC
AGCTTATATTACATTAGAAATGGTCAATCAAGCCAGGTACAGAATACATCGGCATTGGACGAGCCATCTTACAATCAAAAAACGCATCCATCAAA
AACTGCTGACCATTAGCAGATGCACCAACAGCAGTAGTACGATCAATAGGTGGCGTTTCTTGAATAAACGTAGAATTCAAAGTCGGCAACGAAG
TGAACTTCTGAGCATAATGCCAAGGGTCAATCGTACCAGCTGAAGTCGACTTAAACAAACCTGTAATCTGAGAAGGTTTGTAACGGTACTCTGCC
CAACGTTCCTGGTATCCAAATACATCATCATCAGTTGAAGTACCAGTAACATAGATTTCCTTATTAAGAACAGCCTGTTCACCTAAATGAGCAAAT
ACAGGGAAATAGAAATCATAACGTGTCTCACGAGACCACATCTTAGGTAAACCTTGCTGATATGTTAGATCAGCTCTTACGTTTACCAAACCAAT
TATGTATCCATGTTCTTGAGCATGATACGTAAAACCATGTCCACTAGCCAACGCAGTACCCATTGCAGCCAAGTTACCAAGCGGAGTAGCACCGC
CAGAAATCGAAGTAGCAGACGTTTGAGCAATAGGATTAACGTTGACATAAGTAGAACCACCACCAATATATTCAGGACGTTGTAAACGATAATC
TTGTGGAGTTACACCAAAATGAGCACGTAACAATTCTGTATAGCGTGTACCACCTCGCGCATCGCGCTCTAACAAACGCTGAATCTGGAAAGACT
GACGTAACTGGTTAATAGTTGCAGCCGTAGCATCACTTAAATCGGCATACAAACCAGTACCAGCAGTACCAGCGTTATTACTACGATACACAGCA
TGTGTAGTTGAATTAGCATAAATCTGCTTCAAAGCACCAGCACCGTCAACTAATGACAACGTTGTTGAATCATTAGTAACAGACGTCTTAATAGG
AGCAGACGTGCCTAAAGGCAACGTAACTGCATCGCCTTTCTGAGGCCAAGGCAAGGCACCAGTAAAATAATCCTTACGCTTACCGCGTCGAACC
ATAGCATAATCACTCGGAGTATCAGGACCGTCACCCTTGTGAACGGTAACAGAATTTTGTAAATTCTCATCCCTAAACCACTCGTTATAAATCAAA
TTATAAGCACGTAACGGTAACGCGTTATGCGTAACCGTATTAGTACCAGTAATCTGACCAGCCGTAGGCAAACCAAAATGATCAAAAATAGAAC
CTACTGCATAACCACCAGCAGTAGAAGTAATCTGTGGAACTACATACGAAATAGAATCACCTGGGTTCGCTTGCTCACCCATAAACTTAACCCAA
TTGTTCCAAACTAATCTATTTGGAACAAAGAAAAAGAAAGTATCTAAATGCAAATTGTCCATAACTGGAAACAAAGGCGTTGCCAAACGGGCAA
ACATTGTAGCCTTTACATTGTGCATGTCCCCTGGGAGCACTTCATCACAATAAATAGGAACTAGATAACCACCATCAAAAGTAGTTTTATGCGCAT
ATTGCGTATCAAAACTAGAACGCGGAATTTCCGCTTTAGGAACCATAGCAAAACTATGTGAGCTTACTGACTTATTACGATGCATAACAATCTCCC
GAAGTATTCCGAACCACTAGCAAGCTAGTGATCCGGCTTAAAAAAAACATTACTCGCCATCGCGAATCATAACATCCTTAGCTCTAGAAATAAGC
TTGGGAGAACCAAGCAAATCCATAGTACCAGAATTATCATCAAACGTACCAAAATAATATAACTGAAAATCATCAGGGTGTTTATATAACTGATT
ATCATCGCTAGCGCGATTAACTTCGTCCTGAAACTGACGAACAGCAACACCCTCAGATGCAACAAAAGCTGGACGACCATACGCACCAGCTGCA
GTATCTAAAATAGAAACAATAACCATCTTCATAAAAACTCCTTTAAATCTTACGTTTTAAAAGCGATAACTTAGCCAAAGCGACTTTTTCCTTTACA
GCCAAACGCTCTAAAGTGTTATCTTCAAAATGTGAGCGACCTTCTAGCTCACGAGCATATTGTATACCATCAAATTCTTCTGGAAACAATACTTTA
AACTTATTATCATAAAACCGTGGTGGTCGGCACTTTTTGCCACGCACCACAACAGAGTCAGTCGTATAAACGTCTGACATGAACTTATCAAACCA
AGCCTGACCAATGCCAGGCTTAAGAGACATCTTATTAAATTCAGGCTTACGCTGAATAATCTCACCAGTATCTAAATCACAATACTGATAATGCGC
ATCTGCATCAACCACTTCGTGGTTCTCATTAACGGTTTTACCGTTAATCTTCTTCATAATATATCGAGCAACATAAGCAGCAGATTCAAAATTGACA
TCACCAATAGAACTATAGCCATACGGCCAAAGTTCTTCAAGTATCTTTGACGTATATAAGATAGACCCAGTCTGCGTTCTTTTGAAAAACTTCTTA
TCTTCAAAATCAAGCCCAAAGATACAAGCATGGAAATGAGGACGATCGAAAGACTCACCATATTCACCTGCCATATAAAAACGAATAGTCTTGCC
AGTATAGCGTTTTCTCAATCGTTTCATAAACAATTGAAAATCTTCATAATGCAAAGACATATCCTTAGGACAATGCTCTGGAGCATATGTCAAAGT
AATAAAACAATTACTAGTATGCATTTGTGCCTCATGCATACATCTAACGGCCCACTGACGGGACCGTTCAAGGCGACAACCAACACACTGACCAC
AAGGCAATGACAAGGTACGGACTACGTCCGCCCCTGGTATCTCCCGCCAAATAATAGACTTGTCAGCGCATTGATAAGCCGTTAACGGCTTATAA

CACGCCATAAATTACAGTCTAAAACCACCGCGCTGCGGTGAAGTACGCATATTAATGCTCTTGGTCTTGCTTACGCCACGACGAAACTTCTTAGCT
GCGCCATGCTTGCTCATTGGTTTTCTATAAAGGCTCATAACATTGCACTCCGTAGTTAATAATGTGGTTTTGGTGTCACCTAGCACAGTTACATCA
AGTAGAGTAACTGTGCTGCCATCCGCTTACGCGTCTGGCTTAGGTGTTTCTACTGCAGAAACGACGGGTTCAACCACAGGTTTACCGTCAATAAG
ACCAATCTGAATCGCTTCATCACGATTCTCTTCATTCTGTAAATAATTTAACAAAGCATTAGGATCGTTATCAAACTTAGCCCTAATCTTAGCTGGC
AAAGCCATAAAAGCCTCATCAGAGGCACGAATAGCATTCAATGCGGTGTGATAGTCAGATACACCGCTAAAATCGCCATATGATGGCTCTAATG
GCGCCTGGGGCAATTGCCCAGTAACGCCAAAACGCTCAACTATAATATTAATATCACATTCGTCTCTCATGTGTTGTTGAGCCAAACTCGGATCTT
TACATTCAAGACCAGTCTCTTGTGAAACAAGAGCCATATCATAATTGTACGGATTACGTACAAAAGGTAAATTCGTTTTACTCATTTTCTAC

>000167F|arrow

ATATTTTCTTATCCGGAAAATCAAGACCAAACAGACAAGCATGGAAATGAGGACGATCAAAAGATTCACCATATTCACCTGCCATATAAAAACGT
ATCGTTTTCCCAGTAAAACGCTTACGTAACCGCTTCATAAAAGCTGATAATCATTGTAATCCAATGACATATCCTTAGGACAATGCTCTGGAGCAT
ATGTCAAAGTAATAAAACAATTACTAGTATGCATTTGTGCCTCATGCATACAACGAATCGCCCACTGACGTGAGCGTTCAAGGCGACAACCAACA
CACTGACCACAAGGCAATGATAGGGTACGGACTACATCCGCACCCGGTATTTCCCGCCAAATTATAGACCTGTCACTGCATTGATAAGCCGTTAA
GGGCTTATAACAGGCCATAATTACAAACGATAGCCACCACGCTGGGGAGCGTGTCTCATATTAATTGACTTCGTCTTACTAGCAGTTCTGCGAAA
TGACTTTGCAGATTTATATTTGTTTACTGGCTTTCTTCGTAACATGATGAACTCCGTAGTTAAAATAGTGGTTTGGTGTCACCTAGCACAGTTACAT
CAAGTAGAGTAACTGTGCTGGCCTCAGGATTTCATCCTTCGGCCTTAGGTGTTTCTGTAGAAACGATGGGTTCAACCACAGGTTGTCCATCAATA
AGACCCAATTGAATCGCTTCATCACGATTCTGGTCGTTCTCAAGGAACTCCAATAATTTGACAGGATCATGGTCAAATCGGACTCTTAATTTCGCT
GGCAGAGCCATGAAATCGTCCATAGTTGCGTTAATTTGATTCAACGCAGAATGGTAATCAGTAACACCACTAAAATCGCCGTATTGAGGCGATA
CAGGGGCCGTTGGAAGTTCCCCTGTAACCCCGAAACGTTCAATGATGACATTAATGTCACATTCATCTTTCATATGCTGTTGAGCAAGACTTGGG
TCTTGACACAGCAACGCATCGTTTACCGATGCTTCATCTTTATCATAGTTGTACGGATTACGTACAAATGGCAAATTCGCTTTACTCATTATTTGAC
TCCAATTCCCCAAGGGGTTAGTTGATTAGGATTATTTTTGAGTTTATCAAAAATTTCTTTCGAAGCATCGATACCCCTAGACCAAATAGCTGGGGA
TGGAGCCAATAGCCTCTTAGTATTATATGACTGAGCGGATGTTAAATCCGCAGTCGTATTCTTTAAAGCAATATCAGCCAAAATACGTTTATTTTC
GGCTGTAATATTAGGAGCAGTAAGCAACTTATTAACAGTATCAGCCCTAGTATTAGCGGTACCAGCTTCAGTAGCTTCAGTCTGGGCGATAATCT
GCTTTTCTGTTTCTGATGCATTACGAATCTGTTGCATCATAGAAGCAGTATTAATAGCTGAATTAGTTGCATTACCTAAAACATTTTCCATAGTAGC
AGTTGAACCAGATGGGGTAGAAGCACCACCACGTTGATAGGCTAACATAGGAGATAAACCAGCAGCTTCTAAATCTTTAACAGCACGCTGGTAA
CTTGTTCCACTCATATCGGCTTGAAAATCCATTTGCCTCTGAGCCATCTCCTGATTAGCTTTATTAGCAGAAGTAGAACCTAAATAAGAACCAACA
GCGCCCAAAGCAGTACCGACACCAGGAGTAAAGAAACTAGAAGCTGAAGACAGCTTAGAACCAACATTAGTAACCGCATCAAGTATTCCACCAA
ACATAAACTAACGCCCTTCGGTTGTTTCCTCACTACTCCTTACGGAGTAGTCGAGGTTATATAAAACATTAGAAATGATCAATTAGACCAGGTACG
CTGTACATAGGCATAGGTCTGGCCATCTGACAATCAAAGAAAGCATCCATTAAAAATTGCTGACCATTTGCTGCAGCTCCGACCGCCGTAGTACG
AGCAACTGGAGGGGTCTCTTGAATAAACGTTGAATTCAACGTAGGAAGAGAAGTAAATCGCTGTCCATAATGCCAAGCATCAATAGTGCCAGCA
GAAGTAGACCTAAAGAAACCAGTAATTTGAGAAGGTTTGTAACGATACTCCGCCCAACGTTCTTGATAACCAAATACATCATTGTCGGCTGCAGT
ACCTTGAACATAAATTTCTTTGTTCAAAATAGCTTGCTCACCCAAAGTGGCAAATACTGGGAAATAAAAGTCATATCGTGTAGACCTAGACCACAT
CTTAGGAAGACCCTGTTGATATGTGAGGTCAGCACGTACAGAAACTAATCCGATAATGTATCCATGTTCTTGAGCAGCATACGTAAAGCCGTGTC
CCTGAGCCAATGCAGTACCCATTGCAGCAAGGTTACCTTGCGGAGTAGCAGAACCAGTAACCGACGTTGCAGAAGTCTGAGCAATCGGATTAAC
ATTAACAAGGGTCGAACCTCCACCAATATATTCAGGACGTTGTAAACGATAATCCTGTGGAGTTACTCCAAAGTGAGCACGTAGTAACTCAGTAT
AACGAGTACCACCACGTGCATCGCGCTCCAATAACTTCTGAATCTGGAAAGATTGACGAAGTTGGTTAATAGTCGCAGCAGTAGCAGTAGACAA
ATCAGCGTATAACTGATTAGTAGCAACACCAGCATTTGTACTATTAGACAAAGTATTAGAAGCTGAATTTAATTCTCTTAAAGCACCAACAGCAGT
TTGAAAAACACTATAACCAGCAGTACCATTGCCAGCTGGTATGTCAGAATAAATATTAGCACGTGTTCCTAACGGTAAAGAAACAGAAGCACCCT
TCTGTGGCCAAGGCAATGCACCAGTAAAATAATCTTTACGCTTACCACGTCTCAAAAGTGTATAGTTAGCAGGAACATCACCTGAATCACCAAGA
TTTAACGTTACAGAATTCTGTAAGTTTTCATCTCTAAACCATTCGTTATAAATCTCATTATAAGCACGTAACGGCAAAACATTATGCGTTACTGTAT
TAGCGCCAGTAATCTGACCAGCAGTAGGTAAACCAAAATAATCAAAAATTGAACAAACAGCATAACCACCAGCAGGACTAGTAATAGTAGGCAC
TACAAAAGAAATAGAATCACCAGGATTCGTTTGTTCACCCATAAACTTTGGCCAATTGTTCCAAAGTAAACGATTAGGTACAAAGAAAAAGAAA
GTATCAAGATGCAAGTTGTCCATCACAGGAAATAACGGTGTGGCCAAACGTGCAAATGCAGTCATCTTTACACGATGAGTATCGCCAGGCAATA
CTTCATCACAATAAATAGGAACTAAAAAACCAGCATCAAACGTGGTTTTATGAGCATATTGAGTATTAAAACTAGAGCGGGGAATGTCCGCTTTA
GGAACCATAGCAAATGAATGCGTACTTACTGACTTATTGCGAAACATAAACATCTCCCGTAGTTCCGTACCACTCTTTCGAGTGATACGGTATAA
AAAAACCTTACTCGCCTTCGCGAATCATTACATCTTTTGCACGAGCAATCAACTTAGGGCTTTCCAGTAGTTCAAAAACACCAGTGGCATCGTCAA
AAAGACCCAAATAGAACATATGAAAATCATCAGGATGTTTATACAACTGATTATCTTCGCTAGCTCGATTGACTTCATCCTGAAACTGACGTACTG
CAACACCTTCAGATGCAACATAAGCTGGACGACCAAAAGCATCTGCAGCAGTATCCTTAATAGAAACAATAACCATCTTCATAAAAACTCCTTAA
ATAGTACGTTTTAACAATGACAACTTAGCCAACGCAACTTTTTCCTTAACAGCAAGTCGCTCAAGCGTGTTGTCCTCATGCCTAGATCGACCTTCC
ATCTCTCTGGCAAACTGAATCATATCGAATTCTTCAGGAAACTTCAACTTAAATTTATTATCATAAAACCGTGGTGGACGGCACTTTTTGCCACGC
ACCACAACGTGGTCTGACGTATAAACGTCTGACATGTACTTATCTAACCACGATTGCCCGATACCGGGCTTCAATGACATCTTATTAAATTCTGGC
TTACGCTGAATTATCTCACCAGTCTCTAAATCACAATATTGATAATGGGCACCCGCATCAACCACTTCGTGGTTTTCATTGACAGTAACCCCATTAA

TCTTCTTCATAATATATCTTGCAACATAAGCAGCAGACTCAAAAGTAACATCACCAATTGTAGAATAGCCAAACGGCCACAATTCTTCCAAAATCT
CTGACGTGTAGAGGATAGAGCCAGTCTGCGTTCTTTTAA

>000049F|arrow

ATGATGCAATTCCAAGATGGTATGCGACGTACTGCATATCAAGACGCAGTAGCGGATCTTAAGGCTGCAGGTCTTAACCCTATGCTGGCTTATTC
ACAAGGCGGAGCCAAAGTCCAGCCTGGTGCGCAAGCTCCAGTAGGAAATCCACTTAGGTGAGGCTGGAAATTCAGCCCGTGAAGCTGCCATGG
CAGTCGCCAATTTAAACAATTACAAACTCAGAATATCCTGACACAATCGCAAGCCGAAAAAACGGACGCGGATACAAATCTATCACGTGATCAG
GCAACATATACTCGAGCAAATACAGCTCGTGAAATTGCTCAGATGCCGGGATACGGCAAATTTGGTCAGCTTCGCGATGCCCAAATAGAGCAAT
TAAGGACATCAAGTGCATTACAAGCTGCACAACAGCGACAAGCGTTAAGTCAAAGTGCATATACAGACCAATTAGAGCGATTAGCGCAAACTGG
ATCAGCGCCATCCAGTACTAAACCAATTTATCAAGATGTTAAAGGCTATTTACATAGCCAATATGATAAATATCAAAAATATCTACCATTTGGAAA
AATGAAATGAAAACAATCAAACTTAGAACCGCATACAACTATGACACGGATGCTGCGTCAAATGAGTCAGGGTTGGCTTGTGAGGAGCCAACTC
TGGCTCAGCAGCATTATAAAGACGAATGCGATATTAATACTATTCTGGAACGTTTTAACGTTACAGGCTTATTACCTCAAAGTCCGCTGCCGCCTC
AATATGGCGATTTCAGCGGAATTACTGACTACCATAGCGCTTTGAACAAGGTAATGAACGCTATGGAAGAATTTGATAACCTACCGGCTCAAATT
CGTGCTAGGTTCGAAAACGAACCAGCAAACCTGATTGAGTTCTTGCAAGACGAGAAAAATCGACCAGAAGCCGAGAAACTCGGCTTGGTCGAA
AGAGCCATTTCGGAAGAAATGGCGATAAGCACAGTTACTCCACTTGATGTAACTGTGCTAGGTGACACCAACACCAAAAATATCTGATAAACGA
GGCCAAAAATTATGCTTTATAGAAAACAAACAAACAAGCGCAAAAGCGCTAAATCGTTCCGTAGGAACACTTCAAAAACTAAAGCTGCAAATAT
GCAAAAAGCCCCGCAAAGAGGGGGCTGGCGGCTCTAATAAAGCGCCAGGCTACCTCACATGGCCTGTTATCACCCACTGACTGCTTATTTAAGT
AAGCATCAGACAAACTATAAGACCGGCAAATCTTATCGCCGTGTCGCATTTAAAGAATCTGACGAGCATGATCGTCAGATTTCACTGCCCTGCGG
CCAATGCGTTGGCTGCAGGCTAGAAAAATCACGTCAATGGGCCATGCGCTGTATGCATGAAGCCCAATTGCACGAAAAAAATTGCTTTATAACC
CTCACATACAACAATGAAAACCTTCCACAAACTGGATCGCTTGTCAAAAGCGACTTCCAAAAATTCCTCAAACGCTTTAGAAAATCCATTGCACCT
GCAAAATTACGTTACTACATGGCTGGAGAATACGGCACAAGTTTCGGCAGACCTCACTTCCATGCCTGTATCTTCGGATACGATTTTCATGATAA
GAAACTATTCAAAAGGACTCCCTCTGGTTCTCTCATATATACATCCGACCACCTTGCAACCCTCTGGCCACATGGTTATTCCTCCATTGGAGACGTT
ACATTCGAGTCAGCTGCTTACGTTGCTCGATATATTATGCAAAAATACAACGGCCAGATGGAAGAAAACAAACATATAACAAAGGATGAGCACT
ACACATACTGTGATATAGAAACAGGGGAGTTAATAAAGCTCTTACCTGAATACAATAATATGAGCCTTAAACCGGGTATCGGTGCTGAGTGGTA
CAAAAAATACCGTTCCGACGTGTATCCTCATGACTATGTTGTAGTCAATGGAAAAAGGGTAAAACCCCCAAAATACTATGACAAAAAATATAAAT
CAGATTATCCATACGAATACGAAGAATTACTCCACAAACGTGAAACTTCTGCTAAACTCAACCACGAAGACAATACCTATGCTAGACTTGCCGTG
AAGGAAAAAGTCACAAAGGCTAAACTTCAATTATTAAAACGTAACCTCACTTAGGAAATCCTCATGAAATTAGTTCTCTGTACTGTTAAAGACCG
CGCAGCAGATGCGTTCGGTCGCCCAATGTTCGTCCGTTCCATCGGCGAAGCAATCCGGAGCTTTAGCGACGAAGTCAATCGCCAGAGCGATGAC
AATCAACTTTACAACCATTCCGACGATTTCGACCTATTTGAATTAGGCGAATTCGACGATAATACGGGTTTGTTCCAATTACATGAACAACCCAAA
CTTGTATCCTTAGGGAAACAAGTCAAAATTACTGATAAAAATTAAGCGTAGAGTAAAAAGGGGGAAACCCCTTTTTCTCACGCAACTAGGCCTAG
GAGCTCAATAAAATGCATCGTAACAAGTCGGTAGACGTCCATCAGTTCACAATGATTCCAAAAGCGGATATCCCCCGCTCTACATTTGACTGCCA
ATCAACACACAAAACTACATTCGATGCTGGCTTCCTAGTCCCTGTACTCGTAGACGAAATGTTGCCAGGCGATACATTCCGCTGCAACATGACCG
CGTTTGCGCGATTGTCTACACCACTCTATCCGATCATGGATAACATGCATCTGGATAGCTTCTTCTTTTTTGTGCCAAATAGACTTATCTGGTCAAA
TTGGCAAAAATTTATGGGGCAGCAGGCAAATCCTGCGGACTCGATCTCGTACGTAGTGCCCCAACAAGTAACCCCAGCTGGTGGTTACGCTATT
GGCAGCCTTCAAGATTATATGGGTCTGCCAACCGTAGGCCAAGTAGGTGCTGGTGGCACCGTAAGTCACTGTGCCTTCTGGCCACGTGCTTACA
ACCTTATTTATAACGAATGGTTTCGGGACGAAAACCTTCAAAATTCAGTAGTTGTAGATACTGGCGATGGTCCAGATAACGTAGCCAACTACACA
TTATTACGACGTGGAAAACGTAAAGACTATTTCACGTCAGCATTACCTTGGCCACAAAAGGGCGCAAGCGTTACTTTACCGCTTGGAACATCCGC
CCCAATATTACGCACTAACAATGCGCCTGTTTCCAGACTGTATAACGCTGGAACAAATACATTAAACGCAACCGCCCAGGCTATTAACGTAGGTG
TTACTGGTCAAATTACTGGCGGTGCTGACGGCTTGGCAAAATCTTATGATCCTAATGGCGGTTTATATGCAGATTTATCAGCTGCAACCGCTGCA
ACAATTAATCAATTGCGTCAAAGCTTTCAGATACAAAAACTTTTAGAAAGGGACGCCCGTGGCGGAACTCGATATACAGAAATTATCCGCAGCCA
TTTCGGGGTCGTCAGCCCCGATGCGCGTCTCCAACGGCCTGAATACATTGGAGGCGGTTCAACACACATTAACATCAATCCAATCGCCCAGACGA
ATGGTACCGGAGCTTCCGGGACCACTACTCCTCTCGGTACACTTGGCGCTATGGGTACTGGGCTCGCTCACAATCATGGCTTTACTTATTCAAGC
ACTGAACATGGTGTAATTATCGGTCTCGTTTCAGTACGAGCCGATTTAACATACCAACAAGGTATGCACCGCATGTGGAATCGTTCCACACGTTA
TGATTTTTATTTCCCTGCTTTCGCCACTTTGGGCGAACAAGCAGTATTAAACGAAGAAATCTACGTACGAGGCGATGCCAACGACACAGGAGTGT
TTGGATACCAAGAACGTTGGGCAGAATATCGTTATATGCCAAGCCGAATTTCCAGTCTGTTCCGTAGTACGGCAGCTGGAACAATCGACGGCTG
GCATTTAGCCCAACGGTTTACAACTCTTCCAACTTTGAATAACACGTTTATTCAAGAAAATCCACCTGTCTCTCGAACCCTTGCGGTCGGAGCAGC
TGCCAACGGTCAGCAAATCATTTTTGATAGCTTTTTTGATATTAAAAAAGCACGGCCAATGCCAATGTACTCTGTACCTGGCTTAATCGACCACTT
CTAATGGCACTAGAAGCCGCTGCCTCAGGCGCCGCATCTGGCGCCGCTTTTGGACCTTACGGCTCCTTAATTGGAGCCGGAATAGGTGCGGCCG
CTAGTTATTTTGGTGGTCAAGAACAAAACGCTGCTAGCGCACAACAAGCTGCAGCA

>000053F|arrow

TTGTAAACGATAATCTTGTGGAGTTACACCAAAATGAGCACGTAACAATTCTGTATAGCGTGTACCACCTCGCGCATCGCGCTCTAACAAACGCT
GAATCTGGAAAGACTGACGTAACTGGTTAATAGTTGCAGCCGTAGCATCACTTAAATCGGCATACAAACCAGTACCAGCAGTACCAGCGTTATTA
CTACGATACACAGCATGTGTAGTTGAATTAGCATAAATCTGCTTCAAAGCACCAGCACCGTCAACTAATGACAACGTTGTTGAATCATTAGTAAC
AGACGTCTTAATAGGAGCAGACGTGCCTAAAGGCAACGTAACTGCATCGCCTTTCTGAGGCCAAGGCAAGGCACCAGTAAAATAATCCTTACGC
TTACCGCGTCGAACCATAGCATAATCACTCGGAGTATCAGGACCGTCACCCTTGTGAACGGTAACAGAATTTTGTAAATTCTCATCCCTAAACCAC
TCGTTATAAATCAAATTATAAGCACGTAACGGTAACGCGTTATGCGTAACCGTATTAGTACCAGTAATCTGACCAGCCGTAGGCAAACCAAAATG
ATCAAAAATAGAACCTACTGCATAACCACCAGCAGTAGAAGTAATCTGTGGAACTACATACGAAATAGAATCACCTGGGTTCGCTTGCTCACCCA
TAAACTTAACCCAATTGTTCCAAACTAATCTATTTGGAACAAAGAAAAAGAAAGTATCTAAATGCAAATTGTCCATAACTGGAAACAAAGGCGTT
GCCAAACGGGCAAACATTGTAGCCTTTACATTGTGCATGTCCCCTGGGAGCACTTCATCACAATAAATAGGAACTAGATAACCACCATCAAAAGT
AGTTTTATGCGCATATTGCGTATCAAAACTAGAACGCGGAATTTCCGCTTTAGGAACCATAGCAAAACTATGTGAGCTTACTGACTTATTACGAT
GCATAACAATCTCCCGAAGTATTCCGAACCACTAGCAAGCTAGTGATCCGGCTTAAAAAAAACATTACTCGCCATCGCGAATCATAACATCCTTA
GCTCTAGAAATAAGCTTGGGAGAACCAAGCAAATCCATAGTACCAGAATTATCATCAAACGTACCAAAATAATATAACTGAAAATCATCAGGGT
GTTTATATAACTGATTATCATCGCTAGCGCGATTAACTTCGTCCTGAAACTGACGAACAGCAACACCCTCAGATGCAACAAAAGCTGGACGACCA
TACGCACCAGCTGCAGTATCTAAAATAGAACAATAACCATCTTCATAAAAACTCCTTTAAATCTTACGTTTTAAAAGCGATAACTTAGCCAAAGCG
ACTTTTTCCTTTACAGCCAAACGCTCTAAAGTGTTATCTTCAAAATGTGAGCGACCTTCTAGCTCACGAGCATATTGTATACCATCAAATTCTTCTG
GAAACAATACTTTAAACTTATTATCATAAAACCGTGGTCGGCACTTTTTGCCACGCACCACAACAGAGTCAGTCGTATAAACGTCTGACATGAACT
TATCAAACCAAGCCTGACCAATGCCAGGCTTAAGAGACATCTTATTAAATTCAGGCTTACGCTGAATAATCTCACCAGTATCTAAATCACAATACT
GATAATGCGCATCTGCATCAACCACTTCGTGGTTCTCATTAACGGTTTTACCGTTAATCTTCTTCATAATATATCGAGCAACATAAGCAGCAGATTC
AAAATTGACATCACCAATAGAACTATAGCCATACGGCCAAAGTTCTTCAAGTATCTTTGACGTATATAAGATAGACCCAGTCTGCGTTCTTTTGAA
AAACTTCTTATCTTCAAAATCAAGCCCAAAGATACAAGCATGGAAATGAGGACGATCGAAAGACTCACCATATTCACCTGCCATATAAAAACGAA
TAGTCTTGCCAGTATAGCGTTTTCTCAATCGTTTCATAAACAATTGAAAATCTTCATAATGCAAAGACATATCCTTAGGACAATGCTCTGGAGCAT
ATGTCAAAGTAATAAAACAATTACTAGTATGCATTTGTGCCTCATGCATACATCTAACGGCCCACTGACGGGACCGTTCAAGGCGACAACCAACA
CACTGACCACAAGGCAATGACAAGGTACGGACTACGTCCGCCCCTGGTATCTCCCGCCAAATAATAGACTTGTCAGCGCATTGATAAGCCGTTAA
CGGCTTATAACACGCCATAAATTACAGTCTAAAACCACCGCGCTGCGGTGAAGTACGCATATTAATGCTCTTGGTCTTGCTTACGCCACGACGAA
ACTTCTTAGCTGCGCCATGCTTGCTCATTGGTTTTCTATAAAGGCTCATAACATTGCACTCCGTAGTTTAATGTGGTTTTGGTGTCACCTAGCACAG
TTACATCAAGTAGAGTAACTGTGCTGCCATCCGCTTACGCGTCTGGCTTAGGTGTTTCTACTGCAGAAACGACGGGTTCAACCACAGGTTTACCG
TCAATAAGACCAATCTGAATCGCTTCATCACGATTCTCTTCATTCTGTAAATAATTTAACAAAGCATTAGGATCGTTATCAAACTTAGCCCTAATCT
TAGCTGGCAAAGCCATAAAAGCCTCATCAGAGGCACGAATAGCATTCAATGCGGTGTGATAGTCAGATACACCGCTAAAATCGCCATATGATGG
CTCTAATGGCGCCTGGGGCAATTGCCCAGTAACGCCAAAACGCTCAACTATAATATTAATATCACATTCGTCTCTCATGTGTTGTTGAGCCAAACT
CGGATCTTTACATTCAAGACCAGTCTCTTGTGAAACAAGAGCCATATCATAATTGTACGGATTACGTACAAAAGGTAAATTCGTTTTACTCATTTT
CTACGACCTTCCAAGGACCAAGGAAAAGACTTATTCTTTTCCAAAAACTTATCAAAAATAGACTTACTACCTTTCTTAATATCGCGATACCAATATG
GATCGCTAGACGGTGTTCGTAACTCTAAATTATTCTTAGCATCAATATCAGTCTTAATTGTTGATGCAGAATTAGCATTAACACGAGAACCAGCCT
CGGCCTGTTCCAAATAAAAACGAGCCGGTTCTTGTAAAACACGCTGAGAAGCTAAATCAGCTTCTTCAGCAACCTTCCTAGCAGTCTGAACATTT
AATTCACGTTGAGATTTAGCAACTTCCATCTGCTCACGCAATAAATCACTCTCAACATCACGCTTAACAGCTCCACTCAAATCAGACTTAACTGGA
GAAGTAGAAGATGCAGTAGCACCACTCGGAACAGTAGAACCACCTTTAGAATATGCAAGCATAGGACTCAAACCAGCCTTATTCAAATCAGTAA
CCATACGCTGATATTGCGTATTGGACATATCTTCTTGAAAGCGTCTATTTGCTTGCGCTTCAGCAGTATTATATTTTTGCTCTTCATTCTGACTCCAA
GCAGAAGTAGCAAAATCAAATAAACCACCAGCAACTGAATCAAACATACCCATACTAACGCGCTCCGCTTGTTTGCTGACTACTGGTTTCCCAGT
AGTCCAGCTTATATTACATTAGAAATGGTCAATCAAGCCAGGTACAGAATACATCGGCATTGGACGAGCCATCTTACAATCAAAAAACGCATCCA
TCAAAAACTGCTGACCATTAGCAGATGCACCAACAGCAGTAGTACGATCAATAGGTGGCGTTTCTTGAATAAACGTAGAATTCAAAGTCGGCAA
CGAAGTGAACTTCTGAGCATAATGCCAAGGGTCAATCGTACCAGCTGAAGTCGACTTAAACAAACCTGTAATCTGAGAAGGTTTGTAACGGTAC
TCTGCCCAACGTTCCTGGTATCCAAATACATCATCATCAGTTGAAGTACCAGTAACATAGATTTCCTTATTAAGAACAGCCTGTTCACCTAAATGA
GCAAATACAGGGAAATAGAAATCATAACGTGTCTCACGAGACCACATCTTAGGTAAACCTTGCTGATATGTTAGATCAGCTCTTACGTTTACCAA
ACCAATTATGTATCCATGTTCTTGAGCATGATACGTAAAACCATGTCCACTAGCCAACGCAGTACCCATTGCAGCCAAGTTACCAAGCGGAGTAG
CACCGCCAGAAATCGAAGTAGCAGACGTTTGAGCAATAGGATTAACGTTGACATAAGTAGAACCACCACCAATATATTCAGGACG

>000069F|arrow

TCAGGATTTTCTAGAAAAATATTAGATTCGCGGGATCATTAGCGAATCGTTCACGAATATTGGCTGGAAAGCCATAAATTCGTTGTCTGAAGACA
TAATCTGGTTCAGAGCAGAGTGGTAGTCATAGACACCACTAAAGTCGCCATATGAGGCGTTAAAGGAGTTTGAGGAATAAGTCCGGTCATACCA
AACTTTCCCATAATATATTAATATCGCCTTCTTCGCAAATTGCTGCTGAGGTTCGAGTTTCATACCTAACAATGCAGCAAACCCTGGAACGTCATT
GACGCAGCAATCTTGTCGTAATTGTACGATACGAAGAAAATGTTGGCTGTAACTCTTTGTCATTTTTCCATTCCAGTTGGTGGTGGTTATTTGTAC
GATTTTTGTGTGAAATCGGTGGACCCCTCGATAGTTACTCGATGTATCGTTTGCGCCAGATGCGCTATACGCATCTTGGCGATAGTTTTTTTAACG
TCTTGATACATTGGTTTGCTAGAACTAGGAGCTGACCCTGATTTAGCAAGTTTTGTTAACTCGTTTGAATAACGAGATTGTGCCAAAGCTTGAGCA

GCTTGAGCACTTGATGTTTTGCCTATTTCATTTTTCATGAAAGTATCGGCAAGTATTTGTTTATATTGAGCTCTAATATTTGGATTTTCATCCAATTT
GTTTAACGTATCAGCACGTACATTATCTGTTTGATTACTTGTGAGTTCTGTTTGAGCTTCAATTTGTTTAGATTGAGCAATAGCTTGATTTGCTTGA
GCAACCGTTTGATAAGCTTGGGTTCCGGACGTAGTTGCATTACCGAGAACATTTTGCATCTGGGCCATAGCTCCAGCTGGTGTTGTGGCTCCGCC
TTGTGAATACGCAAGCATGGGATTTAACCCAGCTTTTTCATATCTTCAACTGCTCGTTGATATGATGTTCCAGACATTTCAGCTTGAAATCTCTGT
TGATTACTCGCTTGTTCTGCACTCGCTGCGTTTTGACTTTGTGTTCCAAAGTATTGTCCTGCTGCACCTATTGCTGCAGGTGCAAGTGCAGCTAAG
GAGAGTCCCCCAGTAGCAGGGGCAGCTCCTATAGCTATAGCAGGGCCGATTAAATCGGCAATTCCGTCAAATAGTCCCATTAGAAATGGTCTAT
TAAGCCAGGTACTGAATACATTGGCATGGTCTTGCCATTTTGACATCAAAAAATGAGTCAAATAAGAATTGTTGGCCATTTGCAGCTGCTCCAAC
GGCTACTACACGTGATACAGGAGGCGTATCTTGAATAAACGTATTATTCAAAGTAGGCGCAGCGGTAAATTTTTGAGCCAATGCCAACCATCGA
TCGTGCCCGCCGATGTTGATTTGAACAAACCAGTAATTTTAGATGGTTTGTAACGATATTCCGCCCAGCGTTCTTGATATCCAAAACAGTATTGTC
GGCTGCAGTATCTCCTGTTGCATAAATTTCTTTTTGCAAAACAGATTGTTCGCCTAGAGTAGCAAATGCTGGGAAATAAAAGTCATATCGTGTAG
ATCTAGACCACATACGGTCTAATCCTTGTTGATAAGTAAGATCTGCTCTAATGGATACTAATCCAATAATAACGCCATGCTCAGTAATGATTGAGT
AAATCCATGATTATGAGCGAGAGCAGTACCCATAGCAGCAAGGTTGCCCAAAGGGGTAGTCGTTCCAGAAGCGTTTGTTCCCGACGTTTGAGCA
ATCGGATTAACATTAATTGGTGTTGAACCGCCTCCAAGGTATTCAGGCCTTTGTAAACGGGCGTCTGGGGAAATAACTCCAAAGTGACTCCGGAT
AATTTCTGTGTATCGTGTTCCGCCACGTGCATCCCTTTCAAGTAATTTTTGAATTTGAAATGACTGTCTTAATTGATTGACAGTTGCAGCAGTTGCT
TCTGATAAGTCGGTATATAAATTAGAAACGTTATTTACTACACCAGCAGTATTAACACCATAAGCGTTGCCATATCTAGCTAAAGCAGTCGTATTT
CCAGGATCTGTTTGAATTACCGTAAATTTATCGTTTGATGTTGCGTCTCCTGAAATGGTATCCCATTTAATTGGAGCCGTAGTACCTAAAGGTAAG
GTGACACTCGCACCTTTTTGTGGCCATGGTAATGCTGACGTAAAGTAATCATGTCTTTTACCACGACGTTTTAGCACATAGTTTGAGGAAGTATCA
GGGCCATCGCCCTTATCAACTACTGCGCTTGTTTGTAAATTTTCATCTCGGAACCATTCGTTCCAAATAAGATTGTATGCACGTGGCCAAAAGGCA
CAGTGCGTAATAGTTCGGCCAGTATCAATTTGGCCTACTGTTGGTAAGCCCATATAGTCTTGAAGGCTGCCTACGGCATAACCATCTGTTGGGCT
TGTTTGAGTTGGGACAATATAAGATATTGAGTCTGTGGATTTTCTTGTTGACCCATAAATTTTTGCCAGTTATTCCATATAAGGCGATTGGGTACA
AAGAAAAAGAAAGAATCCATAATCATGTTGTCCATGATTGGATATAAAGGCGTTGCTAGACGGGCAAATGCCGTCATTTTTAAGTTGAAAGTGT
CCCCAGGGAGCACTTCATCAACATATACAGGAATTAAATAGCCCGCATCGAAAGTCGTTTTATGTGTTTTTTGAGCATCGAATTTACTACGTGGTA
TATCGGCTCTAGGTACCATCGCGAAGCGATGAGTATTAACTGACTGATTGCGGTGCATGTTTTTCCTTAGTGTTGTTCCGGGGGAAAGATAAATC
TCTTTTCCCCTCGGTTGTTTTATTTAAGTTTAACTTGTTTTCCTAATGATAGAAGTTTTGGTTGTTCATGTAAATCGAATAACCCAGTTGAATCGTCA
AAAGTTCCGAATTCATATAGATCGAAGTCATCAGGGTGATTTAAAAGTTGATTTTCAGTATCAGAACGATTAATTTCATCTGAAAAAGAGCGTAT
AGCTACTCCAGAGGAAGGTACGAACATTGGTCGTGCATATGCTTCAGCAGCACGGTCTTTTACGGAAGCGAGGATAAGTTTCATTATTTTCCTAA
GTGAGGTTACGTTTTAATAGTTGAAGTTTTGCCATAGTTACTTGTTCTTTTGCAGATAGTCGTTCTGGTGTATTGTCTTCGGAATTAAGTTTAGCGT
TATTTTCCCGCATGTAAAGTAATTCGTCATACTCATAAGGTTGGTCAATTTTAAACATTTTGTCATAGTATTTTGGTGGTTTGACCTTTTTACCTCTA
AGTATTACGTAGTCTTGCGGGTATATATCCGAAGTATATTTTATATAAAAGTCTTTACCGATTCCCGGTTTTAAAGACATTTTATTATATTCCGGCT
TTAAGTCTAAATATTCGCCGGTTTCAGGGTGTATGCGTTTGTAATGAGATTCCGCATCTTTCCCTGTTTGTTTTTTCATTATGTATCTAGCCACGTA
GGCGGCTGATTCGAAAGTAACATCTCCAATGGTGGTATAACCAAATGGCCAGAGAGCTTCAAGTTCTGCGGATCTATATAACATAGAACCAGAG
GCAGTCCTTTTCCATAATTTTTTATCAGGAAAGTCGTATCCGAAGATACAGGCATGGAAGTGGGGTCTTGCGAAGAGTTCACCATATTCTCCAGC
CATGTAATAGCGGATTGTAAGTCCTCTTTTTGCGAGAGTTTTTCTAAGTCTTTTAAGGAACAGTTGAAAGTCTTTGTGATCCAAAGAGCCATCGCT
TGGGAGATGTGTATTGTCATATGTGAGTGTTATGAATGAGTTGTTTTTATGCAATTGGGCTTCGTGCATGCACCGAATAGCCCATTGTCTTGATC
GTTCTAGACGGCATCCAACGCACTGCCCACAGGGCAGGTCTAAAGATCGAACGATGTCATGTTTCCGAACTTCGTCGAAAACAATTGATTTGTCA
AAGCATTGAAATGCTTTGAGAGGGTGATAACAAGGCATGTGAGGTGCCTGGGGACTTTATTAGAGTCTCCAGCCTCCACGCATTGGCGAGTTTC
GCATATTTGCGTGTTTTGTATGTGAAGCATGTTTACGGAAAGTCCTAGCGGACTTTTGCTTATTTGCTGGTCTGCGACGCATCATTTTTCTGTCCTT
GTTTATCGTGTTTTTGTGGTTTGGTGTCACCTAGCACAGTTACATCAAGTAGGTAACTGTGCTTCCGAGGTTCCAACAGGGGTTGAAACCTCGGT
TTGGGCTGGTTTTACCAGTCCCATTTTTTCAGCTTCGCTGCGATTT

>000152F|arrow

CATAAACTTTGGCCAATTGTTCCAAAGTAAACGATTAGGTACAAAGAAAAAGAAAGTATCAAGATGCAAGTTGTCCATCACAGGAAATAACGGT
GTGGCCAAACGTGCAAATGCAGTCATCTTTACACGATGAGTATCGCCAGGCAATACTTCATCACAATAAATAGGAACTAAAAAACCAGCATCAA
ACGTGGTTTTATGAGCATATTGAGTATTAAAACTAGAGCGGGGAATGTCCGCTTAGGAACCATAGCAAATGAATGCGTACTTACTGACTTATTGC
GAAACATAAACATCTCCCGTAGTTCCGTACCACTCTTTCGAGTGATACGGTATAAAAAAAAACCTTACTCGCCTTCGCGAATCATTACATCTTTGC
ACGAGCAATCAACTTAGGGCTTTCCAGTAGTTCAAAAACACCAGTGGCATCGTCAAAAAGACCCAAATAGAACATATGAAAATCATCAGGATGT
TTATACAACTGATTATCTTCGCTAGCTCGATTGACTTCATCCTGAAACTGACGTACTGCAACACCTTCAGATGCAACATAAGCTGGACGACCAAAA
GCATCTGCAGCAGTATCCTTATAGAAACAATAACCATCTTCATAAAAACTCCTTAATAGTACGTTTAAACAATGACAACTTAGCCAACGCAACTTT
TTCCTTAACAGCAAGTCGCTCAAGCGTGTTGTCCTCATGCCTAGATCGACCTTCCATCTCTCTGGCAAACTGATCATATCGAATTCTTCAGGAAACT
TCAACTTAAATTTAGTATCATAAAACCGTGGTGGACGGCACTTTTTGCCACGCACCACAACGTGGTCTGACGTATAAACGTCTGACATGTACTATC
TAACCACGATTGCCCGATACCGGGCTTCAATGACATCTTATTAAATTCTGGCTTACGCTGAATTATCTCACCAGTCTCTAATCACAATATTGATAAT
GGGCACCCGCATCAACCACTTCGTGGTTTTCATTGACAGTAACCCCATTAATCTTCTTCATAATATATCTTGCAACATAAGCAGCAGACTCAAAAG

TAACATCACCAATTGTAGAATAGCCAAACGGCCACAATTCTTCCAAAATCTCTGACGTGTAGAGGATAGAGCCAGTCTGCGTTCTTTTAAATATTT
CTTATCCGGAAAATCAAGACCAAACAGACAAGCATGGAAATGAGGACGATCAAAAGATTCACCATATTCACCTGCCATATAAAAACGTATCGTTT
TTCCCAGTAAAACGCTTACGTAACCGCTTCATAAAAAGCTGATAATCATTGTAATCCAATGACATATCCTTAGGACAATGCTCTGGAGCATATGTC
AAAGTAATAAAACAATTACTAGTATGCATTTGTGCCTCATGCATACAACGAATCGCCCACTGACGTGAGCGTTCAAGGCGACAACCAACACACTG
ACCACAAGGCAATGATAGGGTACGGACTACATCCGCACCCGGTATTTCCCGCCAAATTATAGACCTGTCACTGCATTGATAAGCCGTTAAGGGCT
TATAACAGGCCATAATTACAAACGATAGCCACCACGCTGGGGAGCGTGTCTCATATTAATTGACTTCGTCTTACTAGCAGTTCTGCGAAATGACT
TTGCAGATTTATATTTGTTTACTGGCTTTCTTCGTACATGATGAACTCCGTAGTTAAAATAGTGGTTTGGTGTCACCTAGCACAGTTACATCAAGTA
GAGTAACTGTGCTGGCCTCAGGATTTCATCCTTCGGCCTTAGGTGTTTCTGTAGAAACGATGGGTTCAACCACAGGTTGTCCATCAATAAGACCC
AATTGAATCGCTTCATCACGATTCTGGTCGTTCTCAAGGAACTCCAATAATTTGACAGGATCATGGTCAAATCGGACTCTTAATTTCGCTGGCAGA
GCCATGAAATCGTCCATAGTTGCGTTAATTTGATTCAACGCAGAATGGTAATCAGTAACACCACTAAAATCGCCGTATTGAGGCGATACAGGGG
CCGTTGGAAGTTCCCCTGTAACCCCGAAACGTTCAATGATGACATTAATGTCACATTCATCTTTCATATGCTGTTGAGCAAGACTTGGGTCTTGAC
ACAGCAACGCATCGTTTACCGATGCTTTCATCTTTATCATAGTTGTACGGATTACGTACAAATGGCAAATTCGCTTTACTCATTATTTGACTCCAAT
TCCCCAAGGGGTTAGTTTGATTAGGATTATTTTGAGTTTATCAAAAATTTCTTTCGAAGCATCGATACCCCTAGACCAAATAGCTGGGGATGGAG
CCAATAGCCTCTTAGTATTATATGACTGAGCGGATGTTAAATCCGCAGTCGTATTCTTTAAAGCAATATCAGCCAAAATACGTTTATTTTCGGCTG
TAATATTAGGAGCAGTAAGCAACTTATTAACAGTATCAGCCCTAGTATTAGCGGTACCAGCTTCAGTAGCTTCAGTCTGGGCGATAATCTGCTTTT
CTGTTTCTGATGCATTACGAATCTGTTGCATCATAGAAGCAGTATTAATAGCTGAATTAGTTGCATTACCTAAAACATTTTCCATAGTAGCAGTTG
AACCAGATGGGGTAGAAGCACCACCACGTTGATAGGCTAACATAGGAGATAAACCAGCAGCTTCTAAATCTTTAAACAGCACGCTGGTAACTTG
TTCCACTCATATCGGCTTGAAAATCCATTTGCCTCTGAGCCATCTCCTGATTAGCTTTATTAGCAGAAGTAGAACCTAAATAAGAACCAACAGCGC
CCAAAGCAGTACCGACACCAGGAGTAAAGAAACTAGAAGCTGAAGACAGCTTAGAACCAACATTAGTAACCGCATCAAGTATTCCACCAAACAT
AAACTAACGCCCTTCGGTTGTTTCCTCACTACTCCTTACGGAGTAGTCGAGGTTATATAAAACATTAGAAATGATCAATTAGACCAGGTACGCTGT
ACATAGGCATAGGTCTGGCCATCTGACAATCAAAGAAAGCATCCATTAAAAATTGCTGACCATTTGCTGCAGCTCCGACCGCCGTAGTACGAGCA
ACTGGAGGGGTCTCTTGAATAAACGTTGAATTCAACGTAGGAAGAGAAGTAAATCGCTGTCCATAATGCCAAGCATCAATAGTGCCAGCAGAAG
TAGACCTAAAGAAACCAGTAATTTGAGAAGGTTTGTAACGATACTCCGCCCAACGTTCTTGATAACCAAATACATCATTGTCGGCTGCAGTACCT
TGAACATAAATTTCTTTGTTCAAAATAGCTTGCTCACCCAAAGTGGCAAATACTGGGAAATAAAAGTCATATCGTGTAGACCTAGACCACATCTTA
GGAAGACCCTGTTGATATGTGAGGTCAGCACGTACAGAAACTAATCCGATAATGTATCCATGTTCTTGAGCAGCATACGTAAAGCCGTGTCCCTG
AGCCAATGCAGTACCCATTGCAGCAAGGTTACCTTGCGGAGTAGCAGAACCAGTAACCGACGTTGCAGAAGTCTGAGCAATCGGATTAACATTA
ACAAGGGTCGAACCTCCACCAATATATTCAGGACGTTGTAAACGATAATCCTGTGGAGTTACTCCAAAGTGAGCACGTAGTAACTCAGTATAACG
AGTACCACCACGTGCATCGCGCTCCAATAACTTCTGAATCTGGAAAGATTGACGAAGTTGGTTAATAGTCGCAGCAGTAGCAGTAGACAAATCA
GCGTATAACTGATTAGTAGCAACACCAGCATTTGTACTATTAGACAAAGTATTAGAAGCTGAATTTAATTCTCTTAAAGCACCAACAGCAGTTTGA
AAAACACTATAACCAGCAGTACCATTGCCAGCTGGTATGTCAGAATAAATATTAGCACGTGTTCCTAACGGTAAAGAAACAGAAGCACCCTTCTG
TGGCCAAGGCAATGCACCAGTAAAATAATCTTTACGCTTACCACGTCTCAAAAGTGTATAGTTAGCAGGAACATCACCTGAATCACCAAGATTTA
ACGTTACAGAATTCTGTAAGTTTTCATCTCTAAACCATTCGTTATAAATCTCATTATAAGCACGTAACGGCAAAACATTATGCGTTACTGTATTAGC
GCCAGTAATCTGACCAGCAGTAGGTAAACCAAAATAATCAAAATTGAACAAACAGCATAACCACCAGCAGGACTAGTAATAGTAGGCACTACAA
AAGAAATAGAATCACCAGGATTCGTTTGTTCACC

>000091F|arrow

GGAAAGATGCGGATCTCATTACAACGCATACACCCTGAAACCGGCGAATATTTAGACTTAAAGCCGGAATATAATAAATGTCTTTAAAACCGGG
AATCGGTAAAGACTTTTATATAAATATACTTCGGATATATACCCGCAAGACTACGTAATACTTAGAGGTAAAAGGTCAAACCACCAAAAACTATG
ACAATGAAAATGTTTATTGACCACCTTATGATATGACGAATTACTTTACATGCGGGAAATAACGCTAAATTTAATTCCGAAGACAATACCCAGAA
CGACTATCTGCAAAGAACAAGTCACTATGGCAAAACTTCAACTATTAAAACGTAACTCACTTAGGAAAATAATGAACTTATCCTCGCTTCCGTAAA
ACCGTGCTGCTGAAGCATATGCACGACCAATGTTCGTACCTTCCTCTGGAGTAGCTATACGCTCTTTTTCAGATGAAATTAATCGTTCTGATACTG
AAAATCAACTTTTTAATCACCCTGATGATTCGATCTATATGAATTCGGAACATTTGACGATTCAACTGGGTTATTCGATTTACATGAACAACCAAA
ACTCCTATCATTAGGAAAACAAGTTAAACTTAAATAAAACACCGAGGGGAAAGAGATTTTCTTTCCCCCAACACTAAGGAAAAACATGCACCGCA
ATCAGTCAGTTAATACTCACCGCTTCGCGATGGTACCTAGAGCCGATATACCACGTAGTAAATTCGATGCTCAAAAAACATAAAACGACTTTCGA
TGCGGGCTATTAATTCCTGTATATGTTGATGAAGTGCTCCCTGGGGACTTTCAACTTAAAAATGACGGCATTTGCCCGTCTAGCACGCCTTTATAT
CCAATCATGGACAACATGATTATGGATTCTTTCTTTTTCTTTGTACCCAATCGCCTTATATGGAATAACTGGCAAAAATTTATGGGTCAACAAGAA
ATCCAACAGACTCAATATCTTATATTGTCCCACCAAACAAGCCCAACGATGGTTATGCCGGAAGGCAGCCGTCAAGACGATATGGCTGACCAACA
GTAGGCCAAGATTGATACTGGCGAACTATTACGCACTGTGCCTTGTGGCCAAGTGGCGCCAATCGTATTTGGAACGAATGGTTCCGAGATGAAA
AGTGGCAAACAAGCGCAGTAAGTTGAGAAGGCGATGGCCCGGAGACTTCCATCAAACGAGGTGCTAAAACGTTCCGGTGTTAAAAAGACTGTA
TTACTTTACGTCAGCATTACCATGGCCACAAAAGGTGCGAGTGTCACCTTACCTTTAGGTACTACGGCTCCAATTAAATGGGATACCATTTCAGGA
GACGCAACATCAACGTAAATGTACGGTAAGTTCAAACAGATCCTGGAAAATACAGCTGCTTTAGCTAGATATGGCAAACGCTGATGGTGTTAAT
ACTGCGGTGTAGTAAGAACGTTTCTAATTTATATACCGACTTATCAGAAGCAACTGCTGCAACTGTCAATCAATTAAGACAGTCATTTCAAATTCA

AAAATTACTTGAAAGGGATGCACGGGCGGAACACGATACACAGAAATTATCCGGAGTCACTTTGGAGTTATTTCCCCAGACGCCCGTTTACAAA
GGCCTGAATACCTTGGAGGCGGTTCAACACCAATTAATGTTAATCCGATTGCTCAAACGTCGGGAACAACGCTTCTGGAACGACTACCCCTTTGG
GCAACCTTGCTGCTATGGTACTGCTCTCGCTCATAATCATGGATTTACTCAATCATTTACTGAGCATGGCGTTATTATTGGATTAGTATCCATTAGA
GCAGATCTTACTTATCAACAAGGATTAGACCGTATGTGGTCTAGATCTACACGATATGACTTTTATTTCCCAGCATTTGCTACTCTAGGCGAACAA
TCTGTTTGCAAAAAGAAATTTATGCACAGGAGATACTGCAGCCGACAATACTGTTTTTGGATATCAAGAACGCTGGGCGGAATATCGTTACAAAC
CATCTAAATTACTGGTTTGTTCAAATCAACATCGGCGGGCACGATCGATGGTTGGCATTTGGCTCAAAATTTACCGCTGCGCCTACTTTGAATAAT
ACGTTTATTCAAGATACGCCTCCTGTATCACGTGTAGTAGCCGTTGGAGCAGCTGCAAATGGCCAACAATTCTTATTTGACTCATTTTTTGATGTC
AAAATGGCAAGACCAATGCCAATGTATTCAGTACCTGGCTTAATAGACCATTTCTAATGGGACTATTTGACGGAATTGCCGATTTAATCGGCCCT
GCTATAGCTATAGGAGCTGCCCCTGCTACTGGGGACTCTCCTTAGCTGCACTTGCACCTGCAGCAATAGGTGCAGCAGGACAATACTTTGGAACA
CAAAGTCAAAACGCAGCGAGTGCAGAACAAGCGAGTAATCAACAGAGATTTCAAGCTGAAATGTCTGGAACATCATATCAACGAGCAGTTGAA
GATATGAAAAAAGCTGGGTTAAATCCCATGCTTGCGTATTCACAAGGCGGAGCCACAACACCAGCTGGAGCTATGGCCCAGATGCAAATGTTCT
CGGTAATGCACTACGTCCGGAACCCAAGCTTATCAAACGGTTGCGCAAGCAAATCAAGCTATTGCTCAATCTAAACAAATTGAAGCTCAAACAGA
ACTCACAAGTAATCAAACAGATAATGTACGTGCTGATACGTTAATACATTGGATGAAAATCCAAATATTAGAGCTCAATATAAACAAATACTTGC
CGATACTTTCATGAAAAATGAATAGGCAAAACATCAAGTGCTCAAGCTGCTCAAGCTTTGGCACAATCTCGTTATTCAAACGAGTTAACAAAACT
TGCTAATCAGGTCAGCTCCTAGTTCTAGCAAACCAATTTATCAAGACGTAAAAAACATCGCCAAAGATGCGTATAGCGCATCTGGCGCAAAACGA
TACATCGATAACTATCGAGGTCAACCGATTCAACAAAATCGTACAAATAACCAACCACCAATGGAATGAAAATGCAAAGATTACAGCCCCATTTC
TTCGTACTCCGGACAATTACGACACGATTAGCTGCGTCAAATGAGTCAGGGCTGGCATGGGGATGATGCAACTCTGACTCAGTCAGCAATTTGC
TGAAGAAGGTGATTCAATAAGATTATGGAAAGTTCGTATGACCGGACTTATTCACTCAACGCCTTAAACGCCGCATATGGCGACTTTAGTGGGG
TCCTATGAAACTACCACTCCTGCTCTGAACCAAGATTATGGGCTTCATACAACGAATTTAGGGGCTTTACCAGCCAATAGTCGTGGAACGATTCG
CTAATGATCCCTGCGAATCTCATAGCATTCTAGAAAACTCCCTGAATCGCAGCCGAAGCTGAAAAAAGGGGACTGGGGGTAAAGACCACCCAAA
CCGAGTTACACCCCTGTTGGAACCTCGGAAGCACAGTTACCCGACTTGATGTAACTGTGCGAGGTGACACCAAACCACAAAAACAACGATAAAC
AAAGGACAGAAAAAAGGATGCGCTCGCCAGACCACAAAATAAGCAAAAGTCCGCTAGGACTTTCCGTAAACATGCTTCACATACAAAACCCAAA
TAGCGAAACGCGCCATGCGTGGAGGCTGGAGACTCTAATAAAGTCGTCAGGCACCTCACATGCCTTGTTATCACCTCATCAAAGCATATCATGCT
TTGACAAATCAAGTGTTTTTCGACGAAGTTCGGGAAACAGTACATCGTTCGATCTGTAGACCTGCCCTGTGGGCAGTGCGTTTAGTGCCGTCCTA
GAACGATCAAGACAATGGGCTATTCGTGCATGCACGAAGCCCAAGTTGCATAAAAACACGCATTCATAACACTCACTATGACAAATACACATCTC
CCAAGCGATGGCTCTTTGGATCACAAAGACGTTCAATTGTTCCTTAAAAGACTTAGAAAAAACTCTCGCAAAAGAGGACTTACAATCCGCTATTA
CATGGCTGGAGAATATGGTGAACTCTTCGCAAGACCCCACTTCCATGCCTGTATCTTCGGATACGACTTTCCTGATAAAAAATTATGGAAAAGGA
CTGCCTCTGGTTCTATGTTATATAGATCCGCAAACTTGAAGCTCTCTGCCATTTGGTTATACCACCATTGGAGATGTTACTTTTGAATCAGCCGCCT
ACGTGGCTAGATACATAATGAAAAAACAAACAG

>000010F|arrow

AATCGAATAACCCAGTTGAATCGTCAAATGTTCCGAATTCATATAGATCGAAGTCATCAGGGTGATTAAAAAGTTGATTTTCAGTATCAGAACGA
TTAATTTCATCTGAAAAAGAGCGTATAGCTACTCCAGAGGAAGGTACGAACATTGGTCGTGCATATGCTTCAGCAGCACGGTCTTTTACGGAAGC
GAGGATAAGTTTCATTATTTTCCTAAGTGAGGTTACGTTTTAATAGTTGAAGTTTTGCCATAGTTACTTGTTCTTTTGCAGATAGTCGTTCTGGTGT
ATTGTCTTCGGAATTAAGTTTAGCATTATTTTCCCGCATGTAAAGTAATTCGTCATACTCATAAGGTGGTCAATTTTAAACATTTTGTCATAGTATT
TTGGTGGTTTGACCTTTTTACCTCTAAGTATTACGTAGTCTTGCGGGTATATATCCGAAGTATATTTTATATAAAAGTCTTTACCGATTCCCGGTTT
TAAAGACATTTTATTATATTCCGGCTTTAAGTCTAAATATTCGCCGGTTTCAGGGTGTATGCGTTTGTAATGAGATTCCGCATCTTTCCCTGTTTGT
TTTTTCATTATGTATCTAGCCACGTAGGCGGCTGATTCGAAAGTAACATCTCCAATGGTGGTATAACCAAATGGCCAGAGAGCTTCAAGTTCTGC
GGATCTATATAACATAGAACCAGAGGCAGTCCTTTTCCATAATTTTTTATCAGGAAAGTCGTATCCGAAGATACAGGCATGGAAGTGGGGTCTTG
CGAAGAGTTCACCATATTCTCCAGCCATGTAATAGCGGATTGTAAGTCCTCTTTTTGCGAGAGTTTTTCTAAGTCTTTTAAGGAACAGTTGAAAGT
CTTTGTGATCCAAAGAGCCATCGCTTGGGAGATGTGTATTGTCATATGTGAGTGTTATGAATGAGTTGTTTTTATGCAATTGGGCTTCGTGCATG
CACCGAATAGCCCATTGTCTTGATCGTTCTAGACGGCATCCAACGCACTGCCCACAGGGCAGGTCTAAAGATCGAACGATGTCATGTTTCCGAAC
TTCGTCGAAAAACAATTGATTTGTCAAAGCATTGAAATGCTTTGAGAGGGTGATAACAAGGCATGTGAGGTGCCTGGGGACTTTATTAGAGTCTC
CAGCCTCCACGCATTGGCGAGTTTCGCATATTTGCGTGTTTTGTATGTGAAGCATGTTTACGGAAAGTCCTAGCGGACTTTTGCTTATTTGCTGGT
CTGCGACGCATCATTTTTTCTGTCCTTGTTTATCGTGTTTTTGTGGTTTGGTGTCACCTAGCACAGTTACATCAAGTAGGTAACTGTGCTTCCGAGG
TTCCAACAGGGGTTGAAACCTCGGTTTGGGCTGGTTTTACCAGTCCCATTTTTTCAGCTTCGCTGCGATTTTCAGGGTTTTCTAGAAAATCTATTA
GATTCGCGGGATCATTAGCGAATCGTTCACGAATATTGGCTGGTAAAGCCATAAATTCGTTGTCTGAAGCCATAATCTGGTTCAGAGCAGAGTG
GTAGTCATAGACACCACTAAAGTCGCCATATTGAGGCGTTAAAGGAGTTTGAGGAATAAGTCCGGTCATACCGAACTTTTCCATAATATTATTAA
TATCGCATTCTTCAGCAAATTGCTGCTGAGTCAGAGTTGCATCCTCACAATGCAGCCCTGACTCATTTGACGCAGCAATCGTGTCGTAATTGTACG
GAGTACGAAGAAATGGGGCTGTAATCTTTGTCATTTTCATTCCATTGGTGGTTGGTTATTTGTACGATTTTGTTGAATCGGTTGACCTCGATAGTT
ATCGATGTATCGTTTTGCGCCAGATGCGCTATACGCATCTTTGGCGATGTTTTTTACGTCTTGATAAATTGGTTTGCTAGAACTAGGAGCTGACCC
TGATTTAGCAAGTTTTGTTAACTCGTTTGAATAACGAGATTGTGCCAAAGCTTGAGCAGCTTGAGCACTTGATGTTTTGCCTATTTCATTTTTCATG

AAAGTATCGGCAAGTATTTGTTTATATTGAGCTCTAATATTTGGATTTTCATCCAATTTGTTTAACGTATCAGCACGTACATTATCTGTTTGATTACT
TGTGAGTTCTGTTTGAGCTTCAATTTGTTTAGATTGAGCAATAGCTTGATTTGCTTGCGCAACCGTTTGATAAGCTTGGGTTCCGGACGTAGTTGC
ATTACCGAGAACATTTTGCATCTGGGCCATAGCTCCAGCTGGTGTTGTGGCTCCGCCTTGTGAATACGCAAGCATGGGATTTAACCCAGCTTTTTT
CATATCTTCAACTGCTCGTTGATATGATGTTCCAGACATTTCAGCTTGAAATCTCTGTTGATTACTCGCTTGTTCTGCACTCGCTGCGTTTTGACTTT
GTGTTCCAAAGTATTGTCCTGCTGCACCTATTGCTGCAGGTGCAAGTGCAGCTAAGGAGAGTCCCCCAGTAGCAGGGGCAGCTCCTATAGCTAT
AGCAGGGCCGATTAAATCGGCAATTCCGTCAAATAGTCCCATTAGAAATGGTCTATTAAGCCAGGTACTGAATACATTGGCATTGGTCTTGCCAT
TTTGACATCAAAAAATGAGTCAAATAAGAATTGTTGGCCATTTGCAGCTGCTCCAACGGCTACTACACGTGATACAGGAGGCGTATCTTGAATAA
ACGTATTATTCAAAGTAGGCGCAGCGGTAAATTTTTGAGCCAAATGCCAACCATCGATCGTGCCCGCCGATGTTGATTTGAACAAACCAGTAATT
TTAGATGGTTTGTAACGATATTCCGCCCAGCGTTCTTGATATCCAAAAACAGTATTGTCGGCTGCAGTATCTCCTGTTGCATAAATTTCTTTTTGCA
AAACAGATTGTTCGCCTAGAGTAGCAAATGCTGGGAAATAAAAGTCATATCGTGTAGATCTAGACCACATACGGTCTAATCCTTGTTGATAAGTA
AGATCTGCTCTAATGGATACTAATCCAATAATAACGCCATGCTCAGTAAATGATTGAGTAAATCCATGATTATGAGCGAGAGCAGTACCCATAGC
AGCAAGGTTGCCCAAAGGGGTAGTCGTTCCAGAAGCGTTTGTTCCCGACGTTTGAGCAATCGGATTAACATTAATTGGTGTTGAACCGCCTCCAA
GGTATTCAGGCCTTTGTAAACGGGCGTCTGGGGAAATAACTCCAAAGTGACTCCGGATAATTTCTGTGTATCGTGTTCCGCCACGTGCATCCCTT
TCAAGTAATTTTTGAATTTGAAATGACTGTCTTAATTGATTGACAGTTGCAGCAGTTGCTTCTGATAAGTCGGTATATAAATTAGAAACGTTATTT
ACTACACCAGCAGTATTAACACCATAAGCGTTGCCATATCTAGCTAAAGCAGTCGTATTTCCAGGATCTGTTTGAATTACCGTAAATTTATCGTTT
GATGTTGCGTCTCCTGAAATGGTATCCCATTTAATTGGAGCCGTAGTACCTAAAGGTAAGGTGACACTCGCACCTTTTTGTGGCCATGGTAATGC
TGACGTAAAGTAATCATGTCTTTTACCACGACGTTTTAGCACATAGTTTGAGGAAGTATCAGGGCCATCGCCCTTATCAACTACTGCGCTTGTTTG
TAAATTTTCATCTCGGAACCATTCGTTCCAAATAAGATTGTATGCACGTGGCCAAAAGGCACAGTGCGTAATAGTTCGGCCAGTATCAATTTGGC
CTACTGTTGGTAAGCCCATATAGTCTTGAAGGCTGCCTACGGCATAACCATCTGTTGGGCTTGTTTGAGTTGGGACAATATAAGATATTGAGTCT
GTTGGATTTTCTTGTTGACCCATAAATTTTTGCCAGTTATTCCATATAAGGCGATTGGGTACAAAGAAAAAGAAAGAATCCATAATCATGTTGTCC
ATGATTGGATATAAAGGCGTTGCTAGACGGGCAAATGCCGTCATTTTTAAGTTGAAAGTGTCCCCAGGGAGCACTTCATCAACATATACAGGAA
TTAAATAGCCCGCATCGAAAGTCGTTTTATGTGTTTTTTGAGCATCGAATTTACTACGTGGTATATCGGCTCTAGGTACCATCGCGAAGCGGTGA
GTATTAACTGACTGATTGCGGTGCATGTTTTTCCTTAGTGTTGTTCCGGGGGAAAGATAAATCTCTTTTCCCCTCGGTTGTTTTATTTAAGTTTAAC
TTGTTTTCCTAATGATAGGAGTTTTGGTTGTTCATGTA

>000024F|arrow

TTTTCCGTTGACTACAACTAGTCATGGGGATATACGTCGGAACGATATTTTTTGTACCACTCAGCACCAATGCCTGGTTTAAGGCTCATATTGTTA
TATTCTGGTAATAGCTTTATTAATTCCCCTGTTTCTATATCACAGTATGTGTAATGCTCATCCTTTGTTATATGTTTGTTTTCTTCCATCTGGCCGTTG
TATTTTTGCATAATATATCGAGCAACGTAAGCAGCTGACTCGAATGTAACGTCTCCAATGGAGGAATAACCATGTGGCCAGAGGGTTGCAAGGT
GGTCGGATGTATATATGAGAGAACCAGAGGGAGTCCTTTTGAATAGTTTCTTATCATGAAAATCGTATCCGAAGATACAGGCATGGAAGTGAGG
TCTGCCGAAACTTGTGCCGTATTCTCCAGCCATGTAGTAACGTAATTTTGCAGGTGCAATGGATTTTCTGAAGCGCTTAAGGAACTTTTGGAAGT
CGCTTTTGACAAGCGATCCAGTTTGTGGAAGGTTTTCATTGTTGTATGTGGGGTTATAAAGCAGTTTTTTTCGTGCAATTGGGCTTCATGCATGCA
GCGCATGGCCCATTGACGTGATTTTTCTAGCCTGCAGCCAACGCATTGGCCGCAGGGCAGTGAAATCTGACGATCATGCTCGTCAGATTCTTTGA
ATGCGACACGGCGATAAGATTTGCCGGTCTTATAGTTTGTCTGATGCTTACTTAAATAAGCAGTCAGTGGGTGATAACAGGCCATGTGAGGTAG
CCTGGCGCTTTATTAGAGCCGCCAGCCCCCTCTTTGCGGGGCTTTTTGCATATTTGCAGCTTTAGTTTTTGAAGTGTTCCTACGGAACGATTTAGC
GCTTTTGCGCTTGTTTGTTTGTTTTCTATAAAGCATAATTTTTGGCCTCGTTTATCAGATATTTTTGGTGTTGGTGTCACCTAGCACAGTTACATCAA
GTGGAGTAACTGTGCTTATCGCCATTTCTTCCGAAATGGCTCTTTCGACCAGGCCGAGTTTCTCGGCTTCTGGTCGATTTTTCTCGTCTTGCAAGA
ACTCAATCAGGTTTGCTGGTTCGTTTTCGAACCTAGCACGAATTTGAGCCGGTAAGTTATCAAATTCTTCCATAGCGTTCATTACCTTATTCAAGGC
GCTATGATAGTCAGTAATTCCGCTGAAATCGCCATATTGAGGCGGCAGCGGACTTTGAGGTAATAGGCCTGTAACGTTAAAACGTTCCAGGATA
GTATTTATATCGCATTCGTCTTTATAATGCTGCTGAGCCAGAGTTGGCTCCTCACAAGCCAACCCTGACTCATTTGACGCAGCATCCGTGTCATAG
TTGTATGCGGTTCTAAGTTTGATTTTTCATTTCATTTTTCCAAATGGTAGATATTTTTGATATTTATCATATTGGCTATGTAAATAGCCTTTAACATC
TTGATAAATTGGTTTAGTACTGGATGGCGCTGATCCAGTTTGCGCTAATCGCTCTAATTGGTCTGTATATGCACTTTGACTTAACGCTTGTCGCTG
TTGTGCAGCTTGTAATGCACTTGATGTCCTTAATTGCTCTATTTGGGCATCGCGAAGCTGACCAAATTTGCCGTATCCCGGCATCTGAGCAATTTC
ACGAGCTGTATTTGCTCGAGTATATGTTGCCTGATCACGTGATAGATTTGTATCCGCGTCCGTTTTTCGGCTTGCGATTGTGTCAGGATATTCTGA
GTTTGTAATTGTTTAAAATTGGCGACTGCCATGGCAGCTTCACGGGCTGAATTTCCAGCCTCACCTAGTGGATTTCCTACTGGAGCTTGCGCACCA
GGCTGGACTTTGGCTCCGCCTTGTGAATAAGCCAGCATAGGGTTAAGACCTGCAGCCTTAAGATCCGCTACTGCGTCTTGATATGCAGTACGTCG
CATACCATCTTGGAATTGCATCATTGCTGCAGCTTGTTGTGCGCTGGCAGCGTTTTGTTCTTGACCACCAAAATAACTAGCGGCCGCACCTATTCC
GGCTCCAATTAAGGAGCCGTAAGGTCCAAAAGCGGCGCCAGATGCGGCGCCTGAGGCAGCGGCTTCTAGTGCCATTAGAAGTGGTCGATTAAG
CCAGGTACAGAGTACATTGGCATTGGCCGTGCTTTTTTAATATCAAAAAAGCTATCAAAATGATTTGCTGGCCGTTGGCAGCTGCTCCGACCGCA
AGGGTTCGAGAGACAGGTGGATTTTCTTGAATAAACGTGTTATTCAAAGTTGGAAGTGTTGTAAACCGTTGGGCTAAATGCCAGCCGTCAATTG
TTCCAGCTGCCGTACTACGGAACAGACTGGAAATTCGGCTTGGCATATAACGATATTCTGCCCAACGTTCTTGGTATCCAAACACTCCTGTATCGT
TGGCATCGCCTCGTACGTAGATTTCTTCATTTAATACTGCTTGTTCGCCCAAAGTGGCGAAAGCAGGGAAATAGAAATCATAACGTGTGGAACGA

TTCCACATGCGGTGCATACCTTGTTGGTATGTTAAATCGGCTCGTACTGAAACGAGACCGATAATTACACCATGTTCAGTGCTTGAATAAGTAAA
GCCATGATTGTGAGCGAGCCCAGTACCCATAGCGCCAAGTGTACCGAGAGGAGTAGTGGTCCCGGAAGCTCCGGTACCATTCGTCTGGGCGAT
TGGATTGATATTAATGTGTGTTGAACCGCCTCCAATGTATTCAGGCCGTTGGAGACGCGCATCGGGGCTAACGACCCCGAAATGGCTGCGGATA
ATTTCTGTGTATCGAGTTCCGCCACGGGCGTCCCTTTCTAAAAGTTTTTGAATCTGGAAGCTTTGACGCAATTGATTAATTGTTGCAGCGGTTGCA
GCTGATAAATCTGCATATAAACCGCCATTAGGATCATATGATTTTGCCAAGCCGTCAGCACCGCCAGTAATTTGACCAGTAACACCTACGTTAATA
GCCTGGGCGGTTGCGTTTAATGTATTTGTTCCAGCGTTATACAGTCTGGAAACAGGCGCATTGTTAGTGCGTATATTGGGGCGGATGTTCCAAGC
GGTAAAGTAACGCTTGCGCCCTTTTGTGGCCAAGGTAATGCTGACGTGAAATAGTCTTTACGTTTTCCACGTCGTAATAATGTGTAGTTGGCTAC
GTTATCTGGACCATCGCCAGTATCTACAACTACTGAATTTTGAAGGTTTTCGTCCCGAAACCATTCGTTATAAATAAGGTTGTAAGCACGTGGCCA
GAAGGCACAGTGACTTACGGTGCCACCAGCACCTACTTGGCCTACGGTTGGCAGACCCATATAATCTTGAAGGCTGCCAATAGCGTAACCACCA
GCTGGGGTTACTTGTTGGGGCACTACGTACGAGATCGAGTCCGCAGGATTTGCCTGCTGCCCCATAAATTTTTGCCAATTTGACCAGATAAGTCT
ATTTGGCACAAAAAGAAGAAGCTATCCAAATGCATGTATCCATGATCGGATAGAGTGGTGTAGACAATCGCGCAAAGGCGGTCATGTTGCAGC
GGAATGTATCGCCTGGCAACATTTCGTCTACGAGTACAGGGACTAGGAAGCCAGCATCGAATGTAGTTTTATGTGTTGATTGACAGTCAAATGT
AGAGCGGGGGATATCCGCTTTTGGAATCATTGTGAACTGATGGACGTCTACCGACTTGTTACGATGCATTTTTTTGAGCTCCTAGGCCTAGTTGC
GTGAGAAAAAGGGGTTTCCCCCTTTTTACTCTACGCTTAGTTTTTATCAGTAATTTTGACTTGTTTCCCTAAGGATACAAGTTTGGGTTGTTCATGT
AATTGGAACAAACCCGTATTATCGTCGAATTCGCCTAATTCAAATAGGTCGAAATCGTCGGAATGGTTATAAAGTTGATTGTCATCGCTCTGGCG
ATTGACTTCGTCGCTAAAGCTCCGGATTGCTTCGCCGATAGAACGGACGAACATTGGACGACCGAACGCATCTGCTGCGCGGTCTTTAACGGTA
CAGAGTACTAATTTCATGAGGATTTCCTAAGTGAGGTTACGTTTTAATAATTGAAGTTTGGCCTTTGTGACTTTTTCCTTTACGGCAAGTCTGGCA
TAGGTATTGTCTTCGTGGTTGAGTTTAGCAGAAGTTTCACGTTTGTGGAGTAATTCTTCGTATTCATATGGATAATCTGATTTTATTTTTTGTCATA
GTATTTTGGGGGTTTTACCCT

>000085F|arrow

GCGGAGTATCGTTACAAACCTTCTCAAATTACTGGTTTCTTTAGGTCTACTTCTGCTGGCACTATTGATGCTTGGCATTATGGACAGCGATTTACTT
CTCTTCCTACGTTGAATTCAACGTTTATTCAAGAGACCCCTCCAGTTGCTCGTACTACGGCGGTCGGAGCTGCAGCAAATGGTCAGCAATTTTTAA
TGGATGCTTTCTTTGATTGTCAGATGGCCAGACCTATGCCTATGTACAGCGTACCTGGTCTAATTGATCATTTCTAATGTTTTATATAACCTCGACT
ACTCCGTAAGGAGTAGTGAGGAAACAACCGAAGGGCGTTAGTTTATGTTTGGTGGAATACTTGATGCGGTTACTAATGTTGGTTCTAAGCTGTC
TTCAGCTTCTAGTTTCTTTACTCCTGGTGTCGGTACTGCTTTGGGCGCTGTTGGTTCTTATTTAGGTTCTACTTCTGCTAATAAAGCTAATCAGGAG
ATGGCTCAGAGGCAAATGGATTTTCAAGCCGATATGAGTGGAACAAGTTACCAGCGTGCTGTTAAAGATTTAGAAGCTGCTGGTTTATCTCCTAT
GTTAGCCTATCAACGTGGTGGTGCTTCTACCCCATCTGGTTCAACTGCTACTATGGAAAATGTTTTAGGTAATGCAACTAATTCAGCTATTAATAC
TGCTTCTATGATGCAACAGATTCGTAATGCATCAGAAACAGAAAAGCAGATTATCGCCCAGACTGAAGCTACTGAAGCTGGTACCGCTAATACTA
GGGCTGATACTGTTAATAAGTTGCTTACTGCTCCTAATATTACAGCCGAAAATAAACGTATTTTGGCTGATATTGCTTTAAAGAATACGACTGCGG
ATTTAACATCCGCTCAGTCATATAATACTAAGAGGCTATTGGCTCCATCCCCAGCTATTTGGTCTAGGGGTATCGATGCTTCGAAAGAAATTTTTG
ATAAACTCAAAAATAATCCTAATCAACTAACCCCTTGGGGAATTGGAGTCAAATAATGAGTAAAGCGAATTTGCCATTTGTACGTAATCCGTACA
ACTATGATAAAGATGAAGCATCGGTAAACGATGCGTTGCTGTGTCAAGACCCAAGTCTTGCTCAACAGCATATGAAAGATGAATGTGACATTAA
TGTCATCATTGAACGTTTCGGGGTTACAGGGGAACTTCCAACGGCCCCTGTATCGCCTCAATACGGCGATTTTAGTGGTGTTACTGATTACCATTC
TGCGTTGAATCAAATTAACGCAACTATGGACGATTTCATGGCTCTGCCAGCGAAATTAAGAGTCCGATTTGACCATGATCCTGTCAAATTATTGG
AGTTCCTTGAGAACGACCAGAATCGTGATGAAGCGATTCAATTGGGTCTTATTGATGGACAACCTGTGGTTGAACCCATCGTTTCTACAGAAACA
CCTAAGGCCGAAGGATGAAATCCTGAGGCCAGCACAGTTACTCTACTTGATGTAACTGTGCTAGGTGACACCAAACCACTATTTTAACTACGGAG
TTCATCATGTTACGAAGAAAGCCAGTAAACAAATATAAATCTGCAAAGTCATTTCGCAGAACTGCTAGTAAGACGAAGTCAATTAATATGAGACA
CGCTCCCCAGCGTGGTGGCTATCGTTTGTAATTATGGCCTGTTATAAGCCCTTAACGGCTTATCAATGCAGTGACAGGTCTATAATTTGGCGGGA
AATACCGGGTGCGGATGTAGTCCGTACCCTATCATTGCCTTGTGGTCAGTGTGTTGGTTGTCGCCTTGAACGCTCACGTCAGTGGGCGATTCGTT
GTATGCATGAGGCACAAATGCATACTAGTAATTGTTTTATTACTTTGACATATGCTCCAGAGCATTGTCCTAAGGATATGTCATTGGATTACAATG
ATTATCAGCTTTTTATGAAGCGGTTACGTAAGCGTTTTACTGGGAAAACGATACGTTTTTATATGGCAGGTGAATATGGTGAATCTTTTGATCGTC
CTCATTTCCATGCTTGTCTGTTTGGTCTTGATTTTCCGGATAAGAAAATATTTAAAAGAACGCAGACTGGCTCTATCCTCTACACGTCAGAGATTTT
GGAAGAATTGTGGCCGTTTGGCTATTCTACAATTGGTGATGTTACTTTTGAGTCTGCTGCTTATGTTGCAAGATATATTATGAAGAAGATTAATG
GGGTTACTGTCAATGAAAACCACGAAGTGGTTGATGCGGGTGCCCATTATCAATATTGTGATTTAGAGACTGGTGAGATAATTCAGCGTAAGCC
AGAATTTAATAAGATGTCATTGAAGCCCGGTATCGGGCAATCGTGGTTAGATAAGTACATGTCAGACGTTTATACGTCAGACCACGTTGTGGTG
CGTGGCAAAAAGTGCCGTCCACCACGGTTTTATGATAATAAATTTAAGTTGAAGTTTCCTGAAGAATTCGATATGATTCAGTTTGCCAGAGAGAT
GGAAGGTCGATCTAGGCATGAGGACAACACGCTTGAGCGACTTGCTGTTAAGGAAAAAGTTGCGTTGGCTAAGTTGTCATTGTTAAAACGTACT
ATTTAAGGAGTTTTTATGAAGATGGTTATTGTTTCTATTAAGGATACTGCTGCAGATGCTTTTGGTCGTCCAGCTTATGTTGCATCTGAAGGTGTT
GCAGTACGTCAGTTTCAGGATGAAGTCAATCGAGCTAGCGAAGATAATCAGTTGTATAAACATCCTGATGATTTTCATATGTTCTATTTGGGTCTT
TTTGACGATGCCACTGGTGTTTTTGAACTACTGGAAAGCCCTAAGTTGATTGCTCGTGCAAAAGATGTAATGATTCGCGAAGGCGAGTAAGGTTT
TTTTATACCGTATCACTCGAAAGAGTGGTACGGAACTACGGGAGATGTTTATGTTTCGCAATAAGTCAGTAAGTACGCATTCATTTGCTATGGTTC

CTAAAGCGGACATTCCCCGCTCTAGTTTTAATACTCAATATGCTCATAAAACCACGTTTGATGCTGGTTTTTTAGTTCCTATTTATTGTGATGAAGT
ATTGCCTGGCGATACTCATCGTGTAAAGATGACTGCATTTGCACGTTTGGCCACACCGTTATTTCCTGTGATGGACAACTTGCATCTTGATACTTT
CTTTTTCTTTGTACCTAATCGTTTACTTTGGAACAATTGGCCAAAGTTTATGGGTGAACAAACGAATCCTGGTGATTCTATTTCTTTTGTAGTGCCT
ACTATTACTAGTCCTGCTGGTGGTTATGCTGTTTGTTCAATTTTTGATTATTTTGGTTTACCTACTGCTGGTCAGATTACTGGCGCTAATACAGTAA
CGCATAATGTTTTGCCGTTACGTGCTTATAATGAGATTTATAACGAATGGTTTAGAGATGAAAACTTACAGAATTCTGTAACGTTAAATCTTGGTG
ATTCAGGTGATGTTCCTGCTAACTATACACTTTTGAGACGTGGTAAGCGTAAAGATTATTTTACTGGTGCATTGCCTTGGCCACAGAAGGGTGCT
TCTGTTTCTTTACCGTTAGGAACACGTGCTAATATTTATTCTGACATACCAGCTGGCAATGGTACTGCTGGTTATAGTGTTTTTCAAACTGCTGTTG
GTGCTTTAAGAGAATTAAATTCAGCTTCTAATACTTTGTCTAATAGTACAAATGCTGGTGTTGCTACTAATCAGTTATACGCTGATTTGTCTACTGC
TACTGCTGCGACTATTAACCAACTTCGTCAATCTTTCCAGATTCAGAAGTTATTGGAGCGCGATGCACGTGGTGGTACTCGTTATACTGAGTTACT
ACGTGCTCACTTTGGAGTAACTCCACAGGATTATCGTTTACAACGTCCTGAATATATTGGTGGAGGTTCGACCCTTGTTAATGTTAATCCGATTGC
TCAGACTTCTGCAACGTCGGTTACTGGTTCTGCTACTCCGCAAGGTAACCTTGCTGCAATGGGTACTGCATTGGCTCAGGGACACGGCTTTACGT
ATGCTGCTCAAGAACATGGATACATTATCGGATTAGTTTCTGTACGTGCTGACCTCACATATCAACAGGGTCTTCCTAAGATGTGGTCTAGGTCTA
CACGATATGACTTTTATTTCCCAGTATTTGCCACTTTGGGTGAGCAAGCTATTTTGAACAAAGAAATTTATGTTCAAGGTACTGCAGCCGACAATG
ATGTATTTGGTTATCAAGAACGTTGG

>000079F|arrow

CACAATCGCAAGCCGAAAAAACGGACGCGGATACAAATCTATCACGTGATCAGGCAACTATACTCGAGCAATACAGCTCGTGAAATTGCTCAGA
TGCCGGATACGCAAATTTGGTCAGCTTCGCGATGCCCAAATAGAGCAATTAAGGACATCAAGTGCATTACAAGCTGCACAACAGCGACAAGCGT
TAAGTCAAAGTGCATATACAGACCAATTAGAGCGATTAGCGCAAACTGATCGCGCCATCCAGTACTAAACCATTTATCAAGATGTTAAAGGCTAT
TTACATAGCCATATGATAAATATCAAAAATATCTACCATTTGGAAAAATGAAATGAAACAATCAAACTTAGAACCGCATACAACTATGACACGGA
TGCTGCGTCAAATGAGTCAGGGTTGGCTTGTGAGGAGCCAACTCTGGCTCAGCAGCATTATAAAGACGAATGCGATATAAATACTATCCTGGAA
CGTTTTAACGTTACAGGCCTATTACCTCAAAGTCCGCTGCCGCCTCAATATGGCGATTTCAGCGGAATTACTGACTATCATAGCGCCTTGAATAGG
TAATGAACGCTATGGAAGAATTTGATACTTACCGGCTCAAATTCGTGCTAGGTTCGAAAACGAACCAGCAAACCTGATTGAGTTCTTGCAAGACG
AGAAAAATCGACCAGAAGCCGAGAAACTCGGCCTGGTCGAAAGAGCCATTTCGGAAGAAATGCGATAAGCACAGTTACTCCACTTGATGTAACT
GTGCTAGGTGACACCAACACCAAAAATATCTGATAACGAGGCCAAAAATTATGCTTATAGAAAACAACACACAAGCGCAAAAGCGCTAAATCGT
TCCGTAGGAACACTTCAAAAACTAAAGCTGCAAATATGCAAAAAGCCCCGCAAGAGGGGGCTGGCGGCTCTAATAAAGCGCCAGGCTACCTCA
CATGGCCTGTTATCACCCACTGACTGCTTATTTAAGTAAGCATCAGACAAACTATAAGACCGGCAAATCTTATCGCCGTGTCGCATTCAAAGAATC
TGACGAGCATGATCGTCAGATTTCACTGCCCTGCGGCCAATGCGTTGGCTGCAGGCTAGAAAATCACGTCAATGGGCCATGCGCTGCATGCATG
AAGCCCAATTGCACGAAAAAAACTGCTTTATAAACCCTCACATACAACAATGAAAACCTTCCACAAACTGGATCGCTTGTCAAAAGCGACTTCCA
AAAGTTCCTTAAGCGCTTCAGAAAATCCATTGCACCTGCAAAATACGTTTACTACATGGCTGGAGAATACGGCACAAGTTTCGGCAGACCTCACT
TCCATGCCTGTATCTTCGGATACGATTTTCATGATAAGAAACTATTCAAAGGACTCCCTCTGGTTCTCTCATATATACATCCGACCACCTTGCAACC
CTCTGGCCACATGGTTATTCCTCCATTGGAGACGTTACATTCGAGTCAGCTGCTTACGTTGCTCGATATATTATGCAAAAATACAACGGCCAGATG
GAAGAAAACAAACATATAACAAAGGATGAGCATTACACATACTGTGATATAGAAACAGGGGAATTAATAAAGCTATTACCAGAATATAACAGA
GCCTTAAACCAGGCATTGGTGCTGAGTGGTACAAAAAATATCGTTCCGACGTATATCCCCATGACTATGTTGTAGTCAACGGAAAAAGGGTAAA
ACCCCCAAAATACTATGACAAAAAATATAAATCAGATTATCCATATGAATACGAAGAATTACTCCACAAACGTGAAACTTCTGCTAAACTCAACCA
CGAAGACAATACCTATGCCAGACTTGCCGTAAAGGAAAAAGTCACAAAGGCCAAACTTCAATTATTAAAACGTAACCTCACTTAGGAAATCCTCA
TGAAATTAGTACTCTGTACCGTTAAAGACCGCGCAGCAGATGCGTTTCGGTCGTCCAATGTTCGTCCGTTCTATCGGCGAAGCAATCCGGAGCTT
TAGCGACGAAGTCAATCGCCAGAGCGATGACAATCAACTTTATAACCATTCCGACGATTTCGACCTATTTGAATTAGGCGAATTCGACGATAATA
CGGGTTTGTTCCAATTACATGAACAACCCAACTTGTATCCTTAGGAAACAAGTCAAAATTACTGATAAAAACTAAGCGTAGAGTAAAAAGGGGA
AACCCCTTTTTCTCACGCAACTAGGCCTAGGAGCTCAAAAAATGCATCGTAACATCGTAGACGTCCATCAGTTCACAATGATTCCAAAGCGGATA
TCCCCCGCTCTACATTTGACTGTCAATCAACACATAAAACTACATTCGATGCTGGCTTCCTAGTCCCTGTACTCGTAGACGAAATGTTGCCAGCGA
TACATTCCGCTGCAACATGACCGCCTTTGCGCGATTGTCTACACCACTCTATATCCGATCATGGATAACATGCATCTGGATAGCTTCTTCTTTTGTG
CCAAATAGACTTATCTGGTCAAATTGGCAAAAATTTATGGGGCAGCAGGCAAATCCTGCGGACTCGATCTCGTACGTAGTGCCCCAACAAGTAA
CCCCAGCTGGTGGTTACGCTATTGGCAGCCTTCAAGATTATATGGGTCTGCCAACCGTAGCCAAGTAGTGCTGGTGGCACCGTAAGTCACTGTGC
CTTCTGGCCACGTGCTTACAACCTTATTTATAACGAATGGTTTCGGGACGAAAACCTTCAAAATTCAGTAGTTGTAGATACTGGCGATGGTCCAG
ATAACGTAGCCAACTACACATTATTACGACGTGGAAAACGTAAAGACTATTTCACGTCAGCATTACCTTTGGCCACAAAAGGGCGCAAGCGTTAC
TTTACCGCTTGGAACATCCGCCCCAATATTACGCACTAACATGCCCTGTTTCCAGACTGTATAACGCTGGAACATACATTACACAACCCCCGCCCA
GGCTATTAACGTAGGTGTTACTGGTCAAATTACTGGCGGTGCTGACGGCTTGGCAAATCATATGATCCTAATGGCGGTTTATATGCAGATTTTCA
GCTGCAACCGCTGCACAATTAATCAATTGCGTCAAAGCTTCCAGATTCAAAACTTTTAGAAAGGGACGCCCGTGCGGAACTCGATACACAGAATT
ATCCGCAGCCATTTCGGGGTCGTTAGCCCCGATGCGCGTCTCCAACGGCCTGAATACATTGGAGGCGGTTCAACACACATTAATATCAATCCAAT
CGCCCAGACGAATGGTACCGAGCTTCCGGACCACTACTCCTCTCGGTACACTTGGCGCTATGGGTACTGGGCTCGCTCACAATCATGGCTTTACT
TATTCAAGCACTGAACATGGTGTAATTATCGGTCTCTCGTTTCAGTACGAGCCGATTTAACATACCAACAAGGTATGCACCGCATGTGGAATCGT

TCCACACGTTATGATTTCTATTTCCCTGCTTTCGCCACTTTGGGCGAACAAGCAGTATTAAATGAAGAAATCTACGTACGAGCGATGCCCGATACA
GGAGTGTTTGGATACCAAGAACGTTGGGCAGAATATCGTTATATGCCAAGCCGAATTTCCAGTCTGTTCCGTAGTACGGCAGCTGGAACAATTG
ACGGCTGGCATTTAGCCCAACGGTTTACAACACTTCCAACTTTGAATAACACGTTTATTCAAGAAAATCCACCTGTCTCTCGAACCCTTGCGGTCG
GAGCAGCTGCCAACGGCCAGCAAATCATTTTTGATAGCTTTTTTGATATTAAAAAGCACGGCCAATGCCAATGTACTCTGTACCTGGCTTAATCG
ACCACTTCTAATGGCACTAGAAGCCGCTGCCTCAGGCGCCATCTGGCGCCGCTTTTGGACCTTACGGCTCCTTAATTGGAGCCGGAATAGGTGCG
GCCGCTAGTTATTTTGGTGGTCAAGAACAAACGCTGCCAGCGCACAACAAGCTGCAGCAATGATGCAATTCCAAGATGGTATGCGACGTACTGC
ATATCAAGACGCAGTAGCGGATCTTAAGGCTGCAGGTCTTAACCCTATGCTGGCTTATTCACAAGGCGGAGCCAAAGTCCAGCCTGGTGCGCAA
GCTCCAGTAGGAATCCACTAGGTGAGGCTGGAAATTCAGCCCGTGAAGCTGCCATGGCAGTCGCCAATTTTAAACAATTACAACTCAGATATCCT
GA

>000064F|arrow

TCTAATTGATCATTTCTAATGTTTTATATAACCTCGACTACTCCGTAAGGAGTAGTGAGGAAACAACCGAAGGGCGTTAGTTTATGTTTGGTGGA
ATACTTGATGCGGTTACTAATGTTGGTTCTAAGCTGTCTTCAGCTTCTAGTTTCTTTACTCCTGGTGTCGGTACTGCTTTGGGCGCTGTTGGTTCTT
ATTTAGGTTCTACTTCTGCTAATAAAGCTAATCAGGAGATGGCTCAGAGGCAAATGGATTTTCAAGCCGATATGATGGAACAAGTTACCAGCGT
GCTGTTAAAGATTTAGAAGCTGCTGGTTTATCTCCTATGTTAGCCTATCAACGTGGTGGTGCTTCTACCCCATCTGGTTCAACTGCTACTATGGAA
AATGTTTTAGGTAATGCAACTAATTCAGCTATTAATACTGCTTCTATGATGCAACAGATTCGTAATGCATCAGAAACAGAAAAGCAGATTATCGCC
CAGACTGAAGCTACTGAAGCTGGTACCGCTAATACTAGGGCTGATACTGTTAATAAGTTGCTTACTGCTCCTAATATTACAGCCGAAAATAACGT
ATTTTGGCTGATATTGCTTAAAGAATACGACTGCGGATTTAACATCCGCTCAGTCATATAATACTAAGAGGCTATTGGCTCCATCCCCAGCTATTT
GGTCTAGGGTATCGATGCTTCGAAAGAAATTTTTGATAAACTCAAAAATAATCCTAATCAACTAACCCCTTGGGGAATTGGAGTCAATAATGAGT
AAAGCGAATTTGCCATTTGTACGTAATCCGTACAACTATGATAAAGATGAAAGCATCGGTAAACGATGCGTTGCTGTGTCAAGACCCAAGTCTTG
CTCAACAGCATATGAAAGATGAATGTGACATTAATGTCATCAATTGAACGTTTCGGGGTTACAGGGGAACTTCCAACGGCCCCTGTATCGCCTCA
ATACGGCGATTTAGTGGTGTTACTGATTACCATTCTGCGTTGAATCAAATTAACGCAACTATGGACGATTTCATGGCTCTGCCAGCGAAATTAAG
AGTCCGATTTGACCATGATCCTGTCAATTATTGGAGTTCCTTGAGAACGACCAAATCGTGATGAGCGATTCAATTGGGTCTTATTGATGGACAAC
CTGTGGTTGAACCCATCGTTCTACAGAAACCCTAAGGCCGAAGGATGAAATCCTGAGGCCAGCACAGTTACTCTACTTGATGTAACTGTGCTAGG
TGACACCAAACCACTATTTTAACTACGGAGTTCATCATGTTACGAAGAAAGCCAGTAAACAATAATAAATCTGCAAGGTCATTTCGCAGAACTGC
TAGTAAGACGAAGTCAATTAATATGAGACACGCTCCCCAGCGTGGTGGCTATCGTTTGAATTATGGCCTGTATAAGCCCTTAACGCTTATCATGC
AGTGACAGGTCTATAATTTGGCGGGAAATACCGGGTGCGGATGTAGTCCGTACCCTATCATTGCCTTGTGGTCAGTGTGTTGGTTGTCGCCTGA
ACGCTCACGTCATGGGCGATTCGTTGTATGCATGAGGCACAAATGCATACTAGTAATTGTTTTATTACTTTGACATATGCTCCAGAGCATTGTCCT
AAGGATATGTCATTGGATTACAATGATTATCACTTTTTATGAGCGTTACGTAAGCGTTTTACTGGGAAAACGATACGTTTTTATATGGCAGGTGA
ATATGGTGAATCTTTTGATCGTCCTCATTTCCATGCTTGTCTTTTGGTCTTGATTTTCTCGATAAGAAAATATTTAAAAGAACGCAGACTGGCTCTA
TCCTCTACACGTCAGAGATTTTGGAAGAATTGTGGCCGTTTGGCTATTCTACAATTGGTGATGTTACTTTGAGTCGGCTGCTTATGTTGCAAGATA
TATTATGAAGAAGATTAATGGTGGTTACTGTCAATGAAAACCACGAAGTGGTTGATGCGGGTGCCCATTATCAATATTGTGATTTAGAGACTGGT
GAGATAATTCAGCGTAAGCCAGAATTTAATAAGAGTCATTGAAGCCCGGTATCGGCAATCGTGGTTAGATAAGTACATGTCAGACGTTTATAC
GTCAGACCACGTTGTGGTGCGTGGCAAAAAGTGCCGTCCACCACGGTTTTATGATAATAAATTTAAGTTGAAGTTTCCTGAAGAATTCGATATGA
TTCAGTTTGCCAGAGAGATGGAGGTCGATCTAGGCATGAGGACAACACGCTTGAGCGACTTGCTGTTAAGGAAAAAGTTGCGTTGGCTAAGTT
GTCATTGTTAAACGTACTATTTAAGGAGTTTTTATGAAGATGGTTATTGTTCTATTAAGGATACTGCTGCAGATGCTTTTGGTCGTCCAGCTTATG
TTGCATCTGAAGGTGTTGCAGTACGTCAGTTTCAGGATGAAGTCAATCGAGCTAGCGAAGATAATCAGTTGTATAAACATCCTGATGATTTTCAT
ATGTTCTATTTGGGTCTTTTTGACGATGCCACTGGTGTTTTGAACTACTGGAAAGCCCTAAGTTGATTGCTCGTGCAAAAGATGTAATGATTCGCG
AAGGCGAGTAAGGTTTTTTTATACCGTATCACTCGAAAGAGTGGTACGGAACTACGGGAGATGTTATGTTTCGCAATAAGTCAGTAAGTACGC
ATTCATTTGCTATGGGTTCCTAAAGCGGACATTCCCCGCTCTAGTTTTAATACTCAATATGCTCATAAAACCACGTTGATGCTGGTTTTTTAGTTCC
TATTTATTGTGATGAAGTATTGCCTGGCGATACTCATCGTGTAAAGATGACTGCATTTGCACGTTTGGCCACACCGTTATTTCCTGTGATGGACAA
CTTGCATCTTGATACTTTCTTTTTCTTTGTACCTAATCGTTTACTTGGAACAATTGGCAAAGTTTATGGTGAACAAACGAATCCTGGTGATTCTATT
CTTTTGTAGTGCCTACTATTACTAGTCCTGCTGGTGGTTATGCTGTTTGTTCAATTTTGGATTATTTTGGTTTACCTACTGCTGGTCAGATTACTGG
CGCTAATACAGTAACGCATAATGTTTTGCCGTTACGTGCTTATAATGAGATTTATAACGAATGGTTTAGAGATGAAAACTTACAGAATTCTGTAAC
GTTAAATCTTGGTGATTCAGGTGATGTTCCTGCTAACTATACACTTTTGAGACGTGGTAAGCGTAAAGATTATTTTACTGGTGCATTGCCTTGGCC
ACAGAAGGGTGCTTCTGTTTCTTTACCGTTAGGAACACGTGCTAATATTTATTCTGACATACCAGCTGGCAATGGTACTGCTGGTTATAGTGTTTT
TCAAACTGCTGTTGGTGCTTTAAGAGAATTAAATTCAGCTTCTAATACTTTGTCTAATAGTACAAATGCTGGTGTTGCTACTAATCAGTTATACGCT
GATTTGTCTACTGCTACTGCTGCGACTATTAACCAACTTCGTCAATCTTTCCAGATTCAGAAGTTATTGGAGCGCGATGCACGTGGTGGTACTCGT
TATACTGAGTTACTACGTGCTCACTTTGGAGTAACTCCACAGGATTATCGTTTACAACGTCCTGAATATATTGGTGGAGGTTCGACCCTTGTTAAT
GTTAATCCGATTGCTCAGACTTCTGCAACGTCGGTTACTGGTTCTGCTACTCCGCAAGGTACCTTGCTGCAATGGGTACTGCATTGGCTCAGGGA
CACGGCTTTACGTATGCTGCTCAAGAACATGGATACATTATCGGATTAGTTTCTGTACGTGCTGACCTCACATATCAACAGGGTCTTCCTAAGATG
TGGTCTAGGTCTACACGATATGACTTTTATTTCCCAGTATTTGCCACTTTGGGTGAGCAAGCTATTTTGAACAAAGAAATTTATGTTCAAGGTACT

GCAGCCGACAATGATGTATTTGGTTATCAAGAACGTTGGGCGGAGTATCGTTACAAACCTTCTCAAATTACTGGTTTCTTTAGGTCTACCTTCTGC
TGGCACTATTGATGCTTGGCATTATGGACAGCGATTTACTTCTCTTCCTACGTTGAATTCAACGTTTATTCAAGAGACCCCTCCAGTTGCTCGTACT
ACGGCGGTCGGAGCTGCAGCAAATGGTCAGCAATTTTAATGGATGCTTTCTTTGATTGTCAGATGGCCAGACCTATGCCTATGTACAGCGTACCT
GG

>000225F|arrow

TCGTATTAAAAATGAAGATGGTCCTGTTGATTTAACAAACACTACATCACAAGAATGGTTCTTGCGTAATGGTACTTCTACTGGATTTGCTGGAAA
TCTGGTTGATTTACAACGCCCTGTCAATTTTAGACAAATTGAGGTATTCTATGATAAGATATTTATGTTACACGCAGAGCAACCTGGCACTGCGAA
CGGACAACCGCCACAAGCGGGTAGTTGTATTAATAAAATTATTAAAGTTAATCATAAATTAAAACGAATGACATTCAACCCCGATTCCTCATCTGT
CAATGAATGTACTAATCACCGAATGTATTGTGCTCTACAAACTGTAGCAGTTGATGGAACAACTAACACTGCTGGTACATCTGGAATTAATGTAA
TATATACTCATAAAATTCTTTTTAAAGATGCCTAATTCTAATGTACTACAAATAAGATTTATTTTATATACATCGGAAAAAAAACACTATGCCACCC
GCGGGGGTTTGGGGGGGGCGGGTGCGACACCCCCCCCATATAAGACATCACGCCGAAGGCGGTTTAACTTCATGCCCGAAGGGCATCATCTAT
TTGCGATAGAGTCAAATAAGATTCCATTTCAGATTCCATTGAATTGAGGAGGCTACTTTATAGACTCCTCACCCTAATACCCTAAAGAGCCTAAAG
CCTAAAAACCTATGTATATGTAGAACCCTAAAATGAATATTGCCCTACGAATCACAATTGATGAGCTTCCCAACTTTGACTGGTACGGATTATGTA
AAAAGGTAGTTATATACCGACACGAGGCAGATGCGAAAGTGTCGCAGACGCATTATCACGCGTTGATAGTAGGATATACGAAGAAGGAACAAA
CATTCCGAAATTACTTCGTTAAGAAATATAAATACTCAATGAAAACAACTGGTGTTGATGAAAATTTCATAACATATATGTCAAAAGGAAAACTT
GAACCTCATTTTGTAATAGGAATGACGAGTGATGAAATCACGAGCTACAAGGAAAAATGGGTGGAAATAAAACACACTGTATCTCCCCCTTTGG
TCGTCGCCCATAATGGGAAATTGGTGAAACAAGTTCCCGACGATAAGAAAAAGAAATCCAAATGGGATTTAATAGAGATAATGCGAGCAGATT
ATAATATCGGTATGTCGCAGAGAGATGTGTTAAAAATGATAAGAAAAATATTATTACAAGAACATCAAGCAATAGGAATGTATAAGGTACTGGA
TTATTATGATAGTGTAGTAATGGTGGCAGACAAAAATACTTGGTTGGACTCTGCTGAAATAATCCTGAATAAACGCAACCGCGTATAAAGTTTAA
AGAATAAAATATATATACATAGTATAACCCTGAAATGCCTCTCCGATACAAACCACGCAAGTATGCCAAACGACCCAAGAAGAAAGCAAAGAAA
GTAGCAAAGTTAAGCAAACCTGTTAAGAATGCTATTAATAAAATGATTGCTGTTAAGCAAGAAACAAAATGTTTCTCATTTGCCCCGAACCCTGC
TTCGTTTAATCCTGTGCTTCCTGCTTTGAATCTTGATAATGGTGCTATGATAGGACAAAGTACTGTCACACCAAACCCTCCGACGATAAGTCTGGA
TTCCCAATTTGCCACTGGTGTAGATATGTGGTATAGAATTGGTAGTCGTATTGATATTAAAAGTTTTTCAACTATATTTCATTTTACTCTTAGAGAC
CAAGGATTGAAGAATCCGCAGTGGGTACGATTTTGGATATA

>000216F|arrow

GCGATTTTAGTGGTGTTACTGATTACCATTCTGCGTTGAATCAAATTAACGCAACTATGGACGATTTCATGGCTCTGCCAGCGAAATTAAGAGTCC
GATTTGACCATGATCCTGTCAAATTATTGGAGTTCCTTGAGAACGACCAGAATCGTGATGAAGCGATTCAATTGGGTCTTATTGATGGACAACCT
GTGGTTGAACCCATCGTTTCTACAGAAACACCTAAGGCCGAAGGATGAAATCCTGAGGCCAGCACAGTTACTCTACTTGATGTAACTGTGCTAG
GTGACACCAAACCACTATTTTAACTACGGAGTTCATCATGTTACGAAGAAAGCCAGTAAACAAATATAAATCTGCAAAGTCATTTCGCAGAACTG
CTAGTAAGACGAAGTCAATTAATATGAGACACGCTCCCCAGCGTGGTGGCTATCGTTTGTAATTATGGCCTGTTATAAGCCCTTAACGGCTTATC
AATGCAGTGACAGGTCTATAATTTGGCGGGAAATACCGGGTGCGGATGTAGTCCGTACCCTATCATTGCCTTGTGGTCAGTGTGTTGGTTGTCG
CCTTGAACGCTCACGTCAGTGGGCGATTCGTTGTATGCATGAGGCACAAATGCATACTAGTAATTGTTTTATTACTTTGACATATGCTCCAGAGCA
TTGTCCTAAGGATATGTCATTGGATTACAATGATTATCAGCTTTTTATGAAGCGGTTACGTAAGCGTTTTACTGGGAAAACGATACGTTTTTATAT
GGCAGGTGAATATGGTGAATCTTTTGATCGTCCTCATTTCCATGCTTGTCTGTTTGGTCTTGATTTTCCGGATAAGAAAATATTTAAAAGAACGCA
GACTGGCTCTATCCTCTACACGTCAGAGATTTTGGAAGAATTGTGGCCGTTTGGCTATTCTACAATTGGTGATGTTACTTTTGAGTCTGCTGCTTA
TGTTGCAAGATATATTATGAAGAAGATTAATGGGGTTACTGTCAATGAAAACCACGAAGTGGTTGATGCGGGTGCCCATTATCAATATTGTGATT
TAGAGACTGGTGAGATAATTCAGCGTAAGCCAGAATTTAATAAGATGTCATTGAAGCCCGGTATCGGGCAATCGTGGTTAGATAAGTACATGTC
AGACGTTTATACGTCAGACCACGTTGTGGTGCGTGGCAAAAGTGCCGTCCACCACGGTTTTATGATAATAAATTTAAGTTGAAGTTTCCTGAAGA
ATTCGATATGATTCAGTTTGCCAGAGAGATGGAAGGTCGATCTAGGCATGAGGACAACACGCTTGAGCGACTTGCTGTTAAGGAAAAAGTTGC
GTTGGCTAAGTTGTCATTGTTAAAACGTACTATTTAAGGAGTTTTTATGAATGGTTATTGTTTCTATTAAGGATACTGCTGCAGATGCTTTTGGTC
GTCCAGCTTATGTTGCATCTGAAGGTGTTGCAGTACGTCAGTTTCAGGATGAAGTCAATCGAGCTAGCGAAGATAATCAGTTGTATAAACATCCT
GATGATTTTCATATGTTCTATTTGGGTCTTTTTGACGATGCCACTGGTGTTTTTGAACTACTGGAAAGCCCTAAGTTGATTGCTCGTGCAAAAGAT
GTAATGATTCGCGAAGGCGAGTAAGGTTTTTTTTATACCGTATCACTCGAAAGAGTGGTACGGAACTACGGGAGATGTTTATGTTTCGCAATAA
GTCAGTAAGTACGCATTCATTTGCTATGGTTCCTAAAGCGGACATTCCCCGCTCTAGTTTTAATACTCAATATGCTCATAAAACCACGTTTGATGCT
GGTTTTTTAGTTCCTATTTATTGTGATGAAGTATTGCCTGGCGATACTCATCGTGTAAAGATGACTGCATTTGCACGTTTGGCCCACCGTTATTTCC
TGTGATGGACAACTTGCATCTTGATACTTTCTTTTTCTTTGTACCTAATCGTTTACTTTGGAACAATTGGCCAAAGTTTATGGGTGAACAAACGAAT
CCTGGTGATTCTATTTCTTTTGTAGTGCCTACTATTACTAGTCCTGCTGGTGGTTATGCTGTTTGTTCAATTTTTGATTATTTTGGTTTACCTACTGCT
GGTCAGATTACTGGCGCTAATACAGTAACGCATAATGTTTTGCCGTTACGTGCTTATAATGAGATTTATAACGAATGGTTTAGAGATGAAAACTT
ACAGAATTCTGTAACGTTAAATCTTGGTGATTCAGGTGATGTTCCTGCTAACTATACACTTTTGAGACGTGGTAAGCGTAAAGATTATTTTACTGG
TGCATTGCCTTGGCCACAGAAGGGTGCTTCTGTTTCTTTACCGTTAGGAACACGTGCTAATATTTATTCTGACATACCAGCTGGCAATGGTACTGC

TGGTTATAGTGTTTTTCAAACTGCTGTTGGTGCTTTAAGAGAATTAAATTCAGCTTCTAATACTTTGTCTAATAGTACAAATGCTGGTGTTGCTACT
ACAGTTATACGCTGATTTGTCTACTGCTACTGCTGCGACTATTAACCAACTTCGTCAATCTTTCCAGATTCAGAGTTATTGGAGCGCGATGCACGT
GGTGGTACTCGTTATACTGAGTTACTACGTGCTCACTTTGGAGTAACTCCACAGGATTATCGTTTACAACGTCCTGAATATATTGGTGGAGGTTC
GACCCTTGTTAATGTTAATCCGATTGCTCAGACTTCTGCAACGTCGGTTACTGGTTCTGCTACTCCGCAAGGTAACCTTGCTGCAATGGGTACTGC
ATTGGCTCAGGGACACGGCTTTACGTATGCTGCTCAAGAACATGGATACATTATCGGATTAGTTTCTGTACGTGCTGACCTCACATATCAACAGG
GTCTTCCTAAGATGTGGTCTAGGTCTACACGATATGACTTTTATTTCCCAGTATTTGCCACTTTGGGTGAGCAAGCTATTTTGAACAAAGAATTTA
TGTTCAAGGTACTGCAGCCGACAATGATGTATTTGGTTATCAAGAACGTTGGGCGGAGTATCGTTACAAACCTTCTCAAATTACTGGTTTCTTTAG
GTCTACTTCTGCTGGCACTATTGATGCTTGGCATTATGGACAGCGATTTACTTCTCTTCCTACGTTGAATTCAACGTTTATTCAAGAGACCCCTCCA
GTTGCTCGTACTACGGCGGTCGGAGCTGCAGCAAATGGTCAGCAATTTTTAATGGATGCTTTCTTTGATTGTCAGATGGCCAGACCTATGCCTAT
GTACAGCGTACCTGGTCTAATTGATCATTTCTAATGTTTTATATAACCTCGACTACTCCGTAAGGAGTAGTGAGGAAACAACCGAAGGGCGTTAG
TTTATGTTTGGTGGAATACTTGATGCGGTTACTAATGTTGGTTCTAAGCTGTCTTCAGCTTCTAGTTTCTTTACTCCTGGTCGTCTGGTACTGCTTT
GGGCGCTGTTGGTTCTTATTTAGGTTCTACTTCTGCTAATAAAGCTAATCAGAGATGGCTCAGAGGCAAATGGATTTTCAAGCCGATATGAGTGG
AACAAGTTACCAGCGTGCTGTTAAAGATTTAGAAGCTGCTGGTTTATCTCCTATGTTAGCCTATCAACGTGGTGGTGCTTCTACCCCATCTGGTTC
AACTGCTACTATGGAAAATGTTTTAGGTAATGCAACTAATTCAGCTATTAATACTGCTTCTATGATGCAACAGATTCGTAATGCATCAGAAACAGA
AAAGCAGATTATCGCCCAGACTGAAGCTACTGAAGCTGGTACCGCTAATACTAGGGCTGATACTGTTAATAAGTTGCTTACTGCTCCTAATATTA
CAGCCGAAAATAAACGTATTTTGGCTGATATTGCTTTAAAGAATACGACTGCGGATTTAACATCCGCTCAGTCATATAATACTAAGAGGCTATTG
GCTCCATCCCCAGCTATTTGGTCTAGGGTATCGATGCTTCGAAAGAAATTTTTGATAAACTCAAAAATAATCCTAATCAACTAACCCCTTGGGGAA
TTGGAGTCAAATAATGAGTAAAGCGAATTTGCCATTTGTACGTAATCCGTACAACTATGATAAAGATGAAGCATCGGTAAACGATGCGTTGCTGT
GTCAAGACCCAAGTCTTGCTCAACAGCATATGAAAGATGAATGTGACATTAATGTCATCATTGAACGTTTCGGGTTACAGGGGAACTTCCAACG
GCCCCTGTATCGCCTCAATACG

>000162F|arrow

CGCAGACTGGCTCTATCCTCTACACGTCAGAGATTTTGGAAGAATTGTGGCCGTTTGGCTATTCTACAATTGGTGATGTTACTTTTGAGTCTGCTG
CTTATGTTGCAAGATATATTATGAAGAAGATTAATGGGGTTACTGTCAATGAAAACCACGAAGTGGTTGATGCGGGTGCCCATTATCAATATTGT
GATTTAGAGACTGGTGAGATAATTCAGCGTAAGCCAGAATTTAATAAGATGTCATTGAAGCCCGGTATCGGGCAATCGTGGTTAGATAAGTACA
TGTCAGACGTTTATACGTCAGACCACGTTGTGGTGCGTGGCAAAAAGTGCCGTCCACCACGGTTTTATGATAATAAATTTAAGTTGAAGTTTCCT
GAAGAATTCGATATGATTCAGTTTGCCAGAGAGATGGAAGGTCGATCTAGGCATGAGGACAACACGCTTGAGCGACTTGCTGTTAAGGAAAAA
GTTGCGTTGGCTAAGTTGTCATTGTTAAAACGTACTATTTAAGGAGTTTTTATGAAGATGGTTATTGTTTCTATTAAGGATACTGCTGCAGATGCT
TTTGGTCGTCCAGCTTATGTTGCATCTGAAGGTGTTGCAGTACGTCAGTTTCAGGATGAAGTCAATCGAGCTAGCGAAGATAATCAGTTGTATAA
ACATCCTGATGATTTTCATATGTTCTATTTGGGTCTTTTTGACGATGCCACTGGTGTTTTTGAACTACTGGAAAGCCCTAAGTTGATTGCTCGTGCA
AAAGATGTAATGATTCGCGAAGGCGAGTAAGGTTTTTTTTATACCGTATCACTCGAAAGAGTGGTACGGAACTACGGGAGATGTTTATGTTTCG
CAATAAGTCAGTAAGTACGCATTCATTTGCTATGGTTCCTAAAGCGGACATTCCCCGCTCTAGTTTTAATACTCAATATGCTCATAAAACCACGTTT
GATGCTGGTTTTTTAGTTCCTATTTATTGTGATGAAGTATTGCCTGGCGATACTCATCGTGTAAAGATGACTGCATTTGCACGTTTGGCCACACCG
TTATTTCCTGTGATGGACAACTTGCATCTTGATACTTTCTTTTTCTTTGTACCTAATCGTTTACTTTGGAACAATTGGCCAAAGTTTATGGGTGAAC
AAACGAATCCTGGTGATTCTATTTCTTTTGTAGTGCCTACTATTACTAGTCCTGCTGGTGGTTATGCTGTTTGTTCAATTTTTGATTATTTTGGTTTA
CCTACTGCTGGTCAGATTACTGGCGCTAATACAGTAACGCATAATGTTTTGCCGTTACGTGCTTATAATGAGATTTATAACGAATGGTTTAGAGAT
GAAAACTTACAGAATTCTGTAACGTTAAATCTTGGTGATTCAGGTGATGTTCCTGCTAACTATACACTTTTGAGACGTGGTAAGCGTAAAGATTAT
TTTACTGGTGCATTGCCTTGGCCACAGAAGGGTGCTTCTGTTTCTTTACCGTTAGGAACACGTGCTAATATTTATTCTGACATACCAGCTGGCAAT
GGTACTGCTGGTTATAGTGTTTTTCAAACTGCTGTTGGTGCTTTAAGAGAATTAAATTCAGCTTCTAATACTTTGTCTAATAGTACAAATGCTGGT
GTTGCTACTAATCAGTTATACGCTGATTTGTCTACTGCTACTGCTGCGACTATTAACCAACTTCGTCAATCTTTCCAGATTCAGAAGTTATTGGAGC
GCGATGCACGTGGTGGTACTCGTTATACTGAGTTACTACGTGCTCACTTTGGAGTAACTCCACAGGATTATCGTTTACAACGTCCTGAATATATTG
GTGGAGGTTCGACCCTTGTTAATGTTAATCCGATTGCTCAGACTTCTGCAACGTCGGTTACTGGTTCTGCTACTCCGCAAGGTAACCTTGCTGCAA
TGGGTACTGCATTGGCTCAGGGACACGGCTTTACGTATGCTGCTCAAGAACATGGATACATTATCGGATTAGTTTCTGTACGTGCTGACCTCACA
TATCAACAGGGTCTTCCTAAGATGTGGTCTAGGTCTACACGATATGACTTTTATTTCCCAGTATTTGCCACTTTGGGTGAGCAAGCTATTTTGAAC
AAAGAATTTATGTTCAAGGTACTGCAGCCGACAATGATGTATTTGGTTATCAAGAACGTTGGGCGGAGTATCGTTACAAACCTTCTCAAATTAC
TGGTTTCTTTAGGTCTACTTCTGCTGGCACTATTGATGCTTGGCATTATGGACAGCGATTTACTTCTCTTCCTACGTTGAATTCAACGTTTATTCAA
GAGACCCCTCCAGTTGCTCGTACTACGGCGGTCGGAGCTGCAGCAAATGGTCAGCAATTTTTAATGGATGCTTTCTTTGATTGTCAGATGGCCAG
ACCTATGCCTATGTACAGCGTACCTGGTCTAATTGATCATTTCTAATGTTTTATATAACCTCGACTACTCCGTAAGGAGTAGTGAGGAAACAACCG
AAGGGCGTTAGTTTATGTTTGGTGGAATACTTGATGCGGTTACTAATGTTGGTTCTAAGCTGTCTTCAGCTTCTAGTTTCTTTACTCCTGGTGTCG
GTACTGCTTTGGGCGCTGTTGGTTCTTATTTAGGTTCTACTTCTGCTAATAAAGCTAATCAGGAGATGGCTCAGAGGCAAATGGATTTTCAAGCC
GATATGAGTGGAACAAGTTACCAGCGTGCTGTTAAAGATTTAGAAGCTGCTGGTTTATCTCCTATGTTAGCCTATCAACGTGGTGGTGCTTCTAC
CCCATCTGGTTCAACTGCTACTATGGAAAATGTTTTAGGTAATGCAACTAATTCAGCTATTAATACTGCTTCTATGATGCAACAGATTCGTAATGC

ATCAGAAACAGAAAAGCAGATTATCGCCCAGACTGAAGCTACTGAAGCTGGTACCGCTAATACTAGGGCTGATACTGTTAATAAGTTGCTTACT
GCTCCTAATATTACAGCCGAAAATAAACGTATTTTGGCTGATATTGCTTTAAAGAATACGACTGCGGATTTAACATCCGCTCAGTCATATAATACT
AAGAGGCTATTGGCTCCATCCCCAGCTATTTGGTCTAGGGGTATCGATGCTTCGAAAGAAATTTTTGATAAACTCAAAAATAATCCTAATCAACTA
ACCCCTTGGGGAATTGGAGTCAAATAATGAGTAAAGCGAATTTGCCATTTGTACGTAATCCGTACAACTATGATAAAGATGAAGCATCGGTAAA
CGATGCGTTGCTGTGTCAAGACCCAAGTCTTGCTCAACAGCATATGAAAGATGAATGTGACATTAATGTCATCATTGAACGTTTCGGGGTTACAG
GGGAACTTCCAACGGCCCCTGTATCGCCTCAATACGGCGATTTTAGTGGTGTTACTGATTACCATTCTGCGTTGAATCAAATTAACGCAACTATGG
ACGATTTCATGGCTCTGCCAGCGAAATTAAGAGTCCGATTTGACCATGATCCTGTCAAATTATTGGAGTTCCTTGAGAACGACCAGAATCGTGAT
GAAGCGATTCAATTGGGTCTTATTGATGGACAACCTGTGGTTGAACCCATCGTTTCTACAGAAACACCTAAGGCCGAAGGATGAAATCCTGAGG
CCAGCACAGTTACTCTACTTGATGTAACTGTGCTAGGTGACACCAAACCACTATTTTAACTACGGAGTTCATCATGTTACGAAGAAAGCCAGTAA
ACAAATATAAATCTGCAAAGTCATTTCGCAGAACTGCTAGTAAGACGAAGTCAATTAATATGAGACACGCTCCCCAGCGTGGTGGCTATCGTTTG
TAATTATGGCCTGTTATAAGCCCTTAACGGCTTATCAATGCAGTGACAGGTCTATAATTTGGCGGGAAATACCGGGTGCGGATGTAGTCCGTACC
CTATCATTGCCTTGTGGTCAGTGTGTTGGTTGTCGCCTTGAACGCTCACGTCAGTGGGCGATTCGTTGTATGCATGAGGCACAAATGCATACTAG
TAATTGTTTTATTACTTTGACATATGCTCCAGAGCATTGTCCTAAGGATATGTCATTGGATTACAATGATTATCAGCTTTTTATGAAGCGGTTACGT
AAGCGTTTTACTGGGAAAACGATACGTTTTTATATGGCAGGTGAATATGGTGAATCTTTTGATCGTCCTCATTTCCATGCTTGTCTGTTTGGTCTT
GATTTTCCGGATAAGAAAATATTTAAAAGAA

>000099F|arrow

CCGCTATTACATGGCTGGAGAATATGGTGAACTCTTCGCAAGACCCCACTTCCATGCCTGTATCTCGACTTACTTCCTGATAAAAAATTATGGAAA
AGGACTGCCCTGGTTCTATGTTATATAGATCCGCAGAACTTGAAGCTCTCTGGCCATTTGGTTATACCACCATTGGAGATGTTACTTTCGAATCAG
CCGCCTACGTGGCTAGATACATAATGAAAAAACAAACAGGAAAGATGCGGAATCTCATTACAAACGCATACACCCTGAAACCGGCGAATATTTA
GACTTAAAGCCGGAATATAATAAATGTCTTTAAAACCGGAATCGGTAAAGACTTTTATATAAAATATACTTCGGATATATACCCGCAAGACTACG
TAATACTTAGAGGTAAAAGGTCAAACCACCAAAATACTATGACAAATGTTTAAAATTGACCAACCTTATGAGTATGACGAATTACTTTACATGCG
GGAAAATAACGCTAAACTTAATTCCGAAGACATACACCGAACGACTATCTGCAAAAGAACAAGTAACTATGGCAAAACTTCAACTATTAAAACGT
AACCTCACTTAGGAAAATAATGAAACTTATCCTCGCTTCCGTAAAAGACCGTGCTGCTGAAGCATATGCACGACCAATGTTCGTACCTTCCTCTGG
ATAGCTATACGCTCTTTTTCAGATGAAATTAATCGTTCTGATACTGAAATCAACTTTTTTATTAATCACCCTGATGACTTCGATCTATATGAATTCG
GAACTTTTGACGATTCAACTGGGTTATTCGATTTACATGAACAACCAAAACTCTATCATTAGGAAAACAAGTTAAACTTAAATAAAACAACCGAG
GGGAAAGGATTTATCTTTCCCCCGGAACAACACTAAGGAAAAACATGCACCGCAATCAGTCAGTTAATACTCACCGCTTCGCGATGGTACCTAGA
GCCGATATACCACGTAGTAAATTCGATGCTCAAAAACACATAAAACGACTTTCGATGCGCTCCCTGGGACACTTTCACTTAAAAATGACGGCATT
TGCCCGTCTAGCAACGCCTTTATATCCAATCATGGACAACATGATTATGGATTCTTTCTTTTTCTTTGTACCCCAATCGCCTTATATTGGAATAACT
GGCAAAAATGTATGGGTCAACAAGAAAATCCAACAGACTCAATATCTTATATTGTCCCAACTCAAACAAGCCCAACAGATGGTTATGCCGTAGGC
AGCCTTCAAGACATATATGGGCTTACCAACAGTAGGCCAAATTGATACTGGCCGAACTATTACGCACTGTGCCTTTTGGCCACGTGCATACAATC
TTATTTGGAACGAATGGTTCCGAGATGAAAATTTACAAACAAGCGCAAGTAGTTGATAAGGCGATGGCCCTGATACTTCCTCAAACTATGTTCTA
AAACGTCGTGGTAAAGAGACATGATTACTTTACGTCAGCATTACCATGGCCACAAAAAGGTGCGAGTGTCACCTTACACTTTAGGTACTGACGG
CTCCAATTAAATTGGGATACCATTTCAGGAGACGCAACATCAAACCGATAAATTTACGGTATTCAAACAGATCCTGGAAATACGACTGCTTTAGC
TAGATATGGCAACAGCTGATGGTGTTATACTGCGGGTGTAGTAAATAACGTTTCGAATTTATATACCGACTTATCAGAAGCACTGCTGCAACTGT
CCAATCAATTAAGACAGTCCTTTCAAATTCAAAAATTACTTGAAAGGGATGCACGTGGCGGAACACGATACACAGAAATTATCCGGAGTCACTTT
GGAGTTATTTCCCAGACGCCCGTTTACAAAGGCCTGAATACCTTGGAGGCGGTTCAACACCAATTAATGTTAATCCGATTGCTCAAACGTCGGGA
ACAAACGCTTCTGGAACGACTACCCCTTTGGGCAAACCTTGCTGCTATGGGTACTGCTCTCGCTCATAATCATGGATTTACTCAATACATTTACTG
AGCATGGCGGTTATTATTGGATTAGTATCCATTATAGCAGATCTTACTTATCAACAGGATTAGACCGTATGTGGTCTAGATCTACACGATATGACT
TTTATTTCCCAGCATTTGCTACTCTAAGGCGAACAATCTGTTTTTGCAAAAAGAAATTTATGCAACAGGAGATACTGCAGCCGACAATACTGTTTT
TGGATATCAAGAACGCTGGGCGGGAATATCGTTACAAACCATCTAAAATTACTGGTTTGTTCAAATCACATCGGCGGGCACGATCGATGGTTGG
CATTTGGCTCAAAACATTACCGCTGCGCCTACTTTGAATAATACGTTTATTCAAGATACGCCTCCTGTATCACGTGTAGTAGCCGTTTGGAGCAGC
TGCAAATGGCCAACAATTCTTATTTGACTCATTTTTTGATGTCAAAATGGCAAGACCAATGCCAATGTATTCAGTACCTGGCTTAATAGACCATTT
CTAATGGGACTATTTGACGGAATTGCCGATTTAATCGGCCCTGCTATAGCTATAGGAGCTGCCCCTGCTACTGGGGGACTCTCCTTAGCTGCACT
TGCACCTGCAGCAATAGGTGCAGCAGGACAATACTTTGGAACACAAAGTCAAAACGCAGCGAGTGCAGAACAAGCGAGTAATCAACAGAGATT
TCAAGCTGAAATGTCTGGAACATCATATCAACGAGCAGTTGAAGATATGAAAAAAGCTGGGTTAAATCCCATGCTTGCGTATTCACAAGGCGGA
GCCACAACACCAGCTGGAGCTATGGCCCAGATGCAAAATGTTCTCGGTAATGCAACTACGTCCGGAACCCAAGCTTATCAAACGGTTGCTCAAG
CAAATCAAGCTATTGCTCAATCTAAACAAATTGAAGCTCAAACAGAACTCACAAGTAATCAAACAGATAATGTACGTGCTGATACGTTAAACAAA
TTGGATGAAAATCCAAATATTAGAGCTCAATATAAACAAATACTTGCCGATACTTTCATGAAAAATGAAATAGGCAAAACATCAAGTGCTCAAGC
TGCTCAAGCTTTGGCACAATCTCGTTATTCAAACGAGTTAACAAAACTTGCTAAATCAGGGTCAGCTCCTAGTTCTAGCAAACCAATTTATCAAGA
CGTAAAAAATATCGCCAAAGATGCGTATAGCGCATCTGGCGCAAAACGATACATCGATAACTATCGAGGTCAACCGATTCAACAAAATCGTACA
AATAACCAACCACCAATGGAATGAAAATGACAAAGATTACAGCCCCATTTCTTCGTACTCCGTACAATTACGACACGATTGCTGCGTCAAATGAG

TCAGGGCTGCATTGTGAGGATGCAACTCTGACTCAGCAGCAATTTGCTGAAGAATGCGATATTAATAATATTATGGAAAAGTTTGGTATGACCG
GACTTATTCCTCAACTCCTTTAACGCCTCAATATGGCGACTTTAGTGGTGTCTATGACTACCACTCTGCTCTGAACCAGATTATGGCTTCAGACAAC
GAATTTATGGCTTTACCAGCCAATATTCGTGAACGATTCGCTAATGATCCCGCGAATCTAATAGATTTTCTAGAAAATCCTGAAAATCGCAGCGA
AGCTGAAAAAATGGGACTGGTAAAACCAGCCCAAACCGAGGTTTCAACCCCTGTTGGAACCTCGGAAGCACAGTTACCTACTTGATGTAACTGT
GCTAGGTGACACCAAACCACAAAAACACGATAAACAAGGACAGAAAAAATGATGCGTCGCAGACCAGCAAATAAGCAAAAGTCCGCTAGGACT
TTCCGTAAACATGCTTCACATACAAAACACGCAAATATGCGAAACTCGCCAATGCGTGGAGGCTGGAGACTCTAATAAAGTCCCCAGGCACCTCA
CATGCCTTGTTATCACCCTCTCAAGCATTTCAATGCTTTGACAAATCAATTGTTTTCGACGAAGTTCGGAAACATGACATCGTTCGATCTTTAGACC
TGCCCTGTGGGCAGTGCGTTGGATGCCGTCTAGAACGATCAAGACAATGGGCTATTCGGTGCATGCACGAAGCCCAATTGCATAAAAACAACTC
ATTCATAACACTCACATATGACAATACACATCTCCCAAGCGATGGCTCTTTGGATCACAAAGACTTTCAACTGTTCCTTAAAAGACTTAGAAAAAC
TCTCGCAAAAAGAGGACTTACAAT

>000060F|arrow

TTTGTTGAATCGGTTGACCTCGATAGTTATCGATGTATCGTTTTGCGCCAGATGCGCTATACGCATCTTTGGCGATGTTTTTTACGTCTTGATAAAT
TGGTTTGCTAGAACTAGGAGCTGACCCTGATTTAGCAAGTTTTGTTAACTCGTTTGAATAACGAGATTGTGCCAAAGCTTGAGCAGCTTGAGCAC
TTGATGTTTTGCCTATTTCATTTTTCATGAAAGTATCGGCAAGTATTTGTTTATATTGAGCTCTAATATTTGGATTTTCATCCAATTTGTTTAACGTA
TCAGCACGTACATTATCTGTTTGATTACTTGTGAGTTCTGTTTGAGCTTCAATTTGTTTAGATTGAGCAATAGCTTGATTTGCTTGCGCAACCGTTT
GATAAGCTTGGGTTCCGGACGTAGTTGCATTACCGAGAACATTTTGCATCTGGGCCATAGCTCCAGCTGGTGTTGTGGCTCCGCCTTGTGAATAC
GCAAGCATGGGATTTAACCCAGCTTTTTTCATATCTTCAACTGCTCGTTGATATGATGTTCCAGACATTTCAGCTTGAAATCTCTGTTGATTACTCG
CTTGTTCTGCACTCGCTGCGTTTTGACTTTGTGTTCCAAAGTATTGTCCTGCTGCACCTATTGCTGCAGGTGCAAGTGCAGCTAAGGAGAGTCCCC
CAGTAGCAGGGGCAGCTCCTATAGCTATAGCAGGGCCGATTAAATCGGCAATTCCGTCAAATAGTCCCATTAGAAATGGTCTATTAAGCCAGGT
ACTGAATACATTGGCATTGGTCTTGCCATTTTGACATCAAAAAATGAGTCAAATAAGAATTGTTGGCCATTTGCAGCTGCTCCAACGGCTACTACA
CGTGATACAGGAGGCGTATCTTGAATAAACGTATTATTCAAAGTAGGCGCAGCGGTAAATTTTTGAGCCAAATGCCAACCATCGATCGTGCCCG
CCGATGTTGATTTGAACAAACCAGTAATTTTAGATGGTTTGTAACGATATTCCGCCCAGCGTTCTTGATATCCAAAAACAGTATTGTCGGCTGCAG
TATCTCCTGTTGCATAAATTTCTTTTTGCAAAACAGATTGTTCGCCTAGAGTAGCAAATGCTGGGAAATAAAAGTCATATCGTGTAGATCTAGACC
ACATACGGTCTAATCCTTGTTGATAAGTAAGATCTGCTCTAATGGATACTAATCCAATAATAACGCCATGCTCAGTAAATGATTGAGTAAATCCAT
GATTATGAGCGAGAGCAGTACCCATAGCAGCAAGGTTGCCCAAAGGGGTAGTCGTTCCAGAAGCGTTTGTTCCCGACGTTTGAGCAATCGGATT
AACATTAATTGGTGTTGAACCGCCTCCAAGGTATTCAGGCCTTTGTAAACGGGCGTCTGGGGAAATAACTCCAAAGTGACTCCGGATAATTTCTG
TGTATCGTGTTCCGCCACGTGCATCCCTTTCAAGTAATTTTTGAATTTGAAATGACTGTCTTAATTGATTGACAGTTGCAGCAGTTGCTTCTGATAA
GTCGGTATATAAATTAGAAACGTTATTTACTACACCAGCAGTATTAACACCATAAGCGTTGCCATATCTAGCTAAAGCAGTCGTATTTCCAGGATC
TGTTTGAATTACCGTAAATTTATCGTTTGATGTTGCGTCTCCTGAAATGGTATCCCATTTAATTGGAGCCGTAGTACCTAAAGGTAAGGTGACACT
CGCACCTTTTTGTGGCCATGGTAATGCTGACGTAAAGTAATCATGTCTTTTACCACGACGTTTTAGCACATAGTTTGAGGAAGTATCAGGGCCATC
GCCCTTATCAACTACTGCGCTTGTTTGTAAATTTTCATCTCGGAACCATTCGTTCCAAATAAGATTGTATGCACGTGGCCAAAAGGCACAGTGCGT
AATAGTTCGGCCAGTATCAATTTGGCCTACTGTTGGTAAGCCCATATAGTCTTGAAGGCTGCCTACGGCATAACCATCTGTTGGGCTTGTTTGAG
TTGGGACAATATAAGATATTGAGTCTGTTGGATTTTCTTGTTGACCCATAAATTTTTGCCAGTTATTCCATATAAGGCGATTGGGTACAAAGAAAA
AGAAAGAATCCATAATCATGTTGTCCATGATTGGATATAAAGGCGTTGCTAGACGGGCAAATGCCGTCATTTTTAAGTTGAAAGTGTCCCCAGG
GAGCACTTCATCAACATATACAGGAATTAAATAGCCCGCATCGAAAGTCGTTTTATGTGTTTTTTGAGCATCGAATTTACTACGTGGTATATCGGC
TCTAGGTACCATCGCGAAGCGGTGAGTATTAACTGACTGATTGCGGTGCATGTTTTTCCTTAGTGTTGTTCCGGGGGAAAGATAAATCTCTTTTCC
CCTCGGTTGTTTTATTTAAGTTTAACTTGTTTTCCTAATGATAGGAGTTTTGGTTGTTCATGTAAATCGAATAACCCAGTTGAATCGTCAAAAGTTC
CGAATTCATATAGATCGAAGTCATCAGGGTGATTAAAAAGTTGATTTTCAGTATCAGAACGATTAATTTCATCTGAAAAAGAGCGTATAGCTACT
CCAGAGGAAGGTACGAACATTGGTCGTGCATATGCTTCAGCAGCACGGTCTTTTACGGAAGCGAGGATAAGTTTCATTATTTTCCTAAGTGAGG
TTACGTTTTAATAGTTGAAGTTTTGCCATAGTTACTTGTTCTTTTGCAGATAGTCGTTCTGGTGTATTGTCTTCGGAATTAAGTTTAGCAGTTATTTT
CCCGCATGTAAAGTAATTCGTCATACTCATAAGGTTGGTCAATTTTAAACATTTTGTCATAGTATTTTGGTGGTTTGACCTTTTTACCTCTAAGTAT
TACGTAGTCTTGCGGGTATATATCCGAAGTATATTTTATATAAAAGTCTTTACCGATTCCCGGTTTTAAAGACATTTTATTATATTCCGGCTTTAAG
TCTAAATATTCGCCGGTTTCAGGGTGTATGCGTTTGTAATGAGATTCCGCATCTTTCCCTGTTTGTTTTTCATTATGTATCTAGCCACGTAGGCGGC
TGATTCGAAAGTAACATCTCCAATGGTGGTATAACCAAATGGCCAGAGAGCTTCAAGTTCTGCGGATCTATATAACATAGAACCAGAGGCAGTC
CTTTTCCATAATTTTTTATCAGGAAAGTCGTATCCGAAGATACAGGCATGGAAGTGGGGTCTTGCGAAGAGTTCACCATATTCTCCAGCCATGTA
ATAGCGGATTGTAAGTCCTCTTTTTGCGAGAGTTTTTCTAAGTCTTTTAAGGAACAGTTGAAAGTCTTTGTGATCCAAAGAGCCATCGCTTGGGA
GATGTGTATTGTCATATGTGAGTGTTATGAATGAGTTGTTTTTATGCAATTGGGCTTCGTGCATGCACCGAATAGCCCATTGTCTTGATCGTTCTA
GACGGCATCCAACGCACTGCCCACAGGGCAGGTCTAAAGATCGAACGATGTCATGTTTCCGAACTTCGTCGAAAACAATTGATTTGTCAAAGCA
TTGAAATGCTTTGAGAGGGTGATAACAAGGCATGTGAGGTGCCTGGGGACTTTATTAGAGTCTCCAGCCTCCACGCATTGGCGAGTTTCGCATA
TTTGCGTGTTTTGTATGTGAAGCATGTTTACGGAAAGTCCTAGCGGACTTTTGCTTATTTGCTGGTCTGCGACGCATCATTTTTTCTGTCCTTGTTT
ATCGTGTTTTTGTGGTTTGGTGTCACCTAGCACAGTTACATCAAGTAGGTAACTGTGCTTCCGAGGTTCCAACAGGGGTTGAAACCTCGGTTTGG

GCTGGTTTTACCAGTCCCATTTTTTCAGCTTCGCTGCGATTTTCAGGGTTTTCTAGAAAATCTATTAGATTCGCGGGATCATTAGCGAATCGTTCAC
GAATATTGGCTGGTAAAGCCATAAATTCGTTGTCTGAAGCCATAATCTGGTTCAGAGCAGAGTGGTAGTCATAGACACCACTAAAGTCGCCATAT
TGAGGCGTTAAAGGAGTTTGAGGAATAAGTCCGGTCATACCGAACTTTTCCATAATATTATTAATATCGCATTCTTCAGCAAATTGCTGCTGAGTC
AGAGTTGCATCCTCACAATGCAGCCCTGACTCATTTGACGCAGCAATCGTGTCGTAATTGTACGGAGTACGAAGAAATGGGGCTGTAATCTTTGT
CATTTTCATTCCATTGGTGGTTGGTTATTTGTACGAT

>000070F|arrow

TCAACGTGGTGGTGTTCTTACCCCTATCTGGTTCAACTGCTACTATGGAAAATGTTTTAGGTAATGCAACTAATTCAGCTATTAATACTGCTTCTAT
GATGCAACAGATTCGTAATGCATCAGAAACAGAAAAGCAGATTATCGCCCAGACTGAAGCTACTGAAGCTGGTACCGCTAATACTAGGGCTGAT
ACTGTTAATAAGTTGCTTACTGCTCCTAATATTACAGCCGAAAATAAACGTATTTTGGCTGATATTGCTTTAAAGAATACGACTGCGGATTAACAT
CCGCTCAGTCATATAATACTAAGAGGCTATTGGCTCCATCCCCAGCTATTTGGTCTAGGGGTATCGATGCTTCGAAAGAAATTTTGATAAACTCAA
AAATAATCCTAATCAACTAACCCCTTGGGGAATTGGAGTCAAATAATGAGTAAAGCGAATTTGCCATTTGTACGTAATCCGTACAACTATGATAA
AGATGAAGCATCGGTAAACGATGCGTTGCTGTGTCAAGACCAAGTCTTGCTCAACAGCATATGAAAGATGAATGTGACATTAATGTCATCATTG
AACGTTTCGGGGTTACAGGGGAACTTCCAACGGCCCCTGTATCGCCTCAATACGGCGATTTTAGTGGTGTTACTGATTACCATTCTGCGTTGAAT
CAAATTAACGCAACTATGGACGATTTCATGGCTCTGCCAGCGAAATTAAGAGTCCGATTTGACCATGATCCTGTCAAATTATTGGAGTTCCTTGA
GAACGACCAGAATCGTGATGAAGCGATTCAATTGGGTCTTATTGATGGACAACCTGTGGTTGAACCCATCGTTTCTACAGAAACACTAAGGCCG
AAGGATGAAATCCTGAGGCCAGCACAGTTACTCTACTTGATGTAACTGTGCTAGGTGACACCAAACCACTATTTTAACTACGGAGTTCATCATGT
TACGAAGAAAGCCAGTAAACAAATATAAATCTGCAAAGTCATTTCGCAGAACTGCTAGTAAGACGAAGTCAATTAATATGAGACACGCTCCCAG
CGTGGTGGCTATCGTTTGTAATTATGGCCTGTTATAAGCCCTTAACGGCTTATCAATGCAGTGACAGGTCTATAATTTGGCGGGAAATACCGGGT
GCGGATGTAGTCCGTACCCTATCATTGCCTTGTGGTCAGTGTGTTGGTTGTCGCCTTGAACGCTCACGTCAGTGGGCGATTCGTTGTATGCATGA
GGCACAAATGCATACTAGTAATTGTTTATTACTTTGACATATGCTCCAGAGCATTGTCCTAAGGATATGTCATTGGATTACAATGATTATCAGCTT
TTTATGAAGCGGTTACGTAAGCGTTTTACTGGGAAAACGATACGTTTTTATATGGCAGGTGAATATGGTGAATCTTTTGATCGTCCTCATTTCCAT
GCTTGTCTGTTTGGTCTTGATTTTCCGGATAAGAAAATATTTAAAAGAACGCAGACTGGCTCTATCCTCTACACGTCAGAGATTTGGAAGAATTGT
GGCCGTTTGGCTATTCTACAATTGGTGATGTTACTTTTGAGTCTGCTGCTTATGTTGCAAGATATATTATGAAGAAGATTAATGGGGTTACTGTCA
ATGAAAACCACGAAGTGGTTGATGCGGGTGCCCATTATCAATATTGTGATTTAGAGACTGGTGAGATAATTCAGCGTAAGCCAGAATTTAATAA
GATGTCATTGAAGCCCGGTATCGGGCAATCGTGGTTAGATAAGTACATGTCAGACGTTTATACGTCAGACCACGTTGTGGTGCGTGGCAAAAAG
TGCCGTCCACCACGGTTTTATGATAATAAATTTAAGTTGAAGAGCCAAGAATTCGATATGATTCAGTTTGCCAGAGAGATGGAAGGTCGATCTAG
GCATGAGGACAACACGCTTGAGCGACTTGCTGTAAGGAAAAAGTTGCGTTGGCTAAGTTGTCATTGTTAAAACGTACTATTTAAGGAGTTTTTAT
GAAGATGGTTATTGTTTCTATTAAGGATACTGCTGCAGATGCTTTTGGTCGTCCAGCTTATGTTGCATCTGAAGGTGTTGCAGTACGTCAGTTTCA
GGATGAAGTCAATCGAGCTAGCGAAGATAATCAGTTGTATAAACATCCTGATGATTTTCATATGTTCTATTTGGGTCTTTTTGACGATGCCACTGG
TGTTTTTGAACTACTGGAAAGCCCTAAGTTGATTGCTCGTGCAAAAGATGTAATGATTCGCGAAGGCGAGTAAGGTTTTTTTTATACCGTATCACT
CGAAAGAGTGGTACGGAACTACGGGAGATGTTTATGTTTCGCAATAAGTCAGTAAGTACGCATTCATTTGCTATGGTTCCTAAAGCGGACATTCC
CGCTCTAGTTTTAATACTCAATATGCTCATAAAACCACGTTTGATGCTGGTTTTTTAGTTCCTATTTATTGTGATGAAGTATTGCCTGGCGATACTC
ATCGTGTAAAGATGACTGCATTTGCACGTTTGGCCACACCGTTATTTCCTGTGATGGACAACTTGCATCTTGATACTTCTTTTTCTTTGTACCTAAT
CGTTTACTTTGGAACAATTGGCCAAAGTTTATGGGTGAACAAACGAATCCTGGTGATTCTATTTCTTTTGTAGTGCCTACTATTACTAGTCTGCTG
GTGGTTATGCTGTTTGTTCAATTTTTGATTATTTTGGTTTACCTACTGCTGGTCAGATTACTGGCGCTAATCACGTAACGCATAATGTTTTGCCGTG
ACGTGCTTATAATGCGATTTATAACGATATGGTTTAGAGATGCAAACTTACAGAATTCTGTACCAGTTACAATCTGTGGTGATTCAGGTGCTGTTC
TGCTAAACTATACACTTTTGAGACGTGGTAAGCGTAAAGATTATTTTACTGGTGCATGCCTGGCCACAGAAGGGTGCTTCTGTTTCTTTACCGTTA
GGATAACACGTGCTAATATTATTGCTGACCATACCAGCTGGCAATGGTACTGTGGTTATAGTGTTTTTCAAACTGCTGTTGGTGCTTAAGAGAATT
AAATTCAGCTTCTAATACTTTGTCTAATAGTTACAAATGCTGGTGTTGCTACTAATCAGTTATACGCTGATTTGTCTACTGCTACTGCTGCGAACTA
TTAACCAACTTCGTCAATCTTTCCAGATTCAGAAGTTATTGGAGCGCGATGCACGTGGTGGTACTCGTTATACTGAGTTACTACGTGCTCACTTTG
AGTAACTCCACAGGATTATCGTTTACAACGTCCTGAATATATTGGTGGAGGTTCGACCCTTGTTAATTTAATCCGATTGCTCAGACTCTGCCAACG
TCGGTTACTGGTTCTGCTACTCCGCAAGGTAACCTTGCTGCAATGGGTACTGCATTGGCTCAGGGACACGGCTTTACGTATGCTGCTCAAGAACA
TGGATACATTATCGGATAGTTCTGTACGTGCTGACCTCACATATCAACAGGGTCTTCCTAAGATGTGGTCTAGGTCTACACGATATGACTTTTATT
TCCAGTATTTGCCACTTTGGGTGAGCAAGCTATTTTGAACAAAGAAATTTATGTTCAAGGTTACTGCAGCCGACAATGATGTATTTGGTTATCAAG
AACGTTGGGCGGAGTATCGTACAAACCTTCTCAAATTAATGGTTTCTTTAGGTCTACTTCTGCTGGCACTATTGATGCTTGGCCTTATGGACAGCG
ATTTACTTCTCTTCCTACGTTGAATTCAACGTTTATTCAAGAGACCCCTCCAGTTGCTCGTACTACGGCGGTCGGCAGCTGCAGCAAATGGTCAGC
AATTTTAATGGATGCTTTCTTTGATTGTCAGATGGCCAGACCTATGCCTATGTACAGAGTACCTGGTCTAATGATCATTTCTAATGTTTTATATAAC
TCGACTACTCCGTAAGGAGTAGTGAGGGGAAACAACGAAGGGCGTTAGTTTATGTTTGGTGGATACTTGATGCGGTTACTAATGTTGGTTCTAA
GCTGTCTTCAGCTTCTAGTTTCTTTACTCCTGTGTCGGTACTGCTTTGGGCGCTGTTGGTTTCTTATTTAGGTTCTACTTCTGCTAATAAAGCTAATC
AGGAGATGGCTCAGAGGCAAATGGATTTTCAAGCCGATATGAGTGGAACAAGTTACCAGCTGTGCTGTTAAAGATTTAGAAGCTGCTGGTTTAT
CTCCTATGTTAGCCTA

>000119F|arrow

AGAGCGATTAGCGCAAACTGGATCAGCGCCATCCAGTACTAAACCAATTTATCAAGATGTTAAAGGCTATTTACATAGCCAATATGATAAATATC
AAAAATATCTACCATTTGGAAAAATGAAATGAAAACAATCAAACTTAGAACCGCATACAACTATGACACGGATGCTGCGTCAAATGAGTCAGGG
TTGGCTTGTGAGGAGCCAACTCTGGCTCAGCAGCATTATAAAGACGAATGCGATATAAATACTATCCTGGAACGTTTTAACGTTACAGGCCTATT
ACCTCAAAGTCCGCTGCCGCCTCATATGGCGATTTCAGCGGAATTACTGACTATCATAGCGCCTTGAATAAGGTAATGAACGCTATGGAAGAATT
TGATAACTTACCGGCTCAAATTCGTGCTAGGTTCGAAAACGAACCAGCAAACCTGATTGAGTTCTTGCAAGACGAGAAAAATCGACCAGAAGCC
GAGAAACTCGGCCTGGTCGAAAGAGCCATTTCGGAAGAAATGGCGATAAGCACAGTTACTCCACTTGATGTAACTGTGCTAGGTGACACCAACA
CCAAAAATATCTGATAAACGAGGCCAAAAATTATGCTTTATAGAAAACAAACAAACAAGCGCAAAAGCGCTAAATCGTTTCCGTAGGAACACTT
CAAAAACTAAAGCTGCAAATATGCAAAAAGCCCCGCAAAGAGGGGGCTGGCGGCTCTAATAAAGCGCCAGGCTACCTCACATGGCCTGTTATCA
CCCACTGACTGCTTATTTAAGTAAGCATCAGACAAACTATAAGACCGGCAAATCTTATCGCCGTGTCGCATTCAAAGAATCTGACGAGCATGATC
GTCAGATTTCACTGCCCTGCGGCCAATGCGTTGGCTGCAGGCTAGAAAAATCACGTCAATGGGCCATGCGTTGCATGCATGAAGCCCAATTGCA
CGAAAAAAAACTGCTTTATAACCCTCACATACAACAATGAAAACCTTCCACAAACTGGATCGCTTGTCAAAAGCGACTTCCAAAAGTTCCTTAAGC
GCTTCAGAAAATCCATTGCACCTGCAAAATTACGTTACTACATGGCTGGAGAATACGGCACAAGTTTCGGCAGACCTCACTTCCATGCCTGTATCT
TCGGATACGATTTTCATGATAAGAAACTATTCAAAAGGACTCCCTCTGGTTCTCTCATATATACATCCGACCACCTTGCAACCCTCTGGCCACATG
GTTATTCCTCCATTGGAGACGTTACATTCGAGTCAGCTGCTTACGTTGCTCGATATATTATGCAAAAATACAACGGCCAGATGGAAGAAAACAAA
CATATAACAAAGGATGAGCATTACACATACTGTGATATAGAAACAGGGGAATTAATAAAGCTATTACCAGAATATAACAATATGAGCCTTAAAC
CAGGCATTGGTGCTGAGTGGTACAAAAAATATCGTTCCGACGTATATCCCCATGACTACGTTGTAGTCAACGGAAAAAGGGTAAAACCCCCAAA
ATACTATGACAAAAAATATAAATCAGATTATCCATATGAATACGAAGAATTACTCCACAAACGTGAAACTTCTGCTAAACTCAACCACGAAGACA
ATACCTATGCCAGACTTGCCGTAAAGGAAAAAGTCACAAAGGCCAAACTTCAATTATTAAAACGTAACCTCACTTAGGAAATCCTCATGAAATTA
GTACTCTGTACCGTTAAAGACCGCGCAGCAGATGCGTTCGGTCGTCCAATGTTCGTCCGTTCTATCGGCGAAGCAATCCGGAGCTTTAGCGACGA
AGTCAATCGCCAGAGCGATGACAATCAACTTTATAACCATTCCGACGATTTCGACCTATTGAATTAGGCGAATTCGACGATAATACGGGTTTGTT
CCAATTACATGAACAACCCAAACTTGTATCCTTAGGGAAACAAGTCAAAATTACTGATAAAAACTAAGCGTAGAGTAAAAGGGGGAAACCCCT
TTTTCTCACGCAACTAGGCCTAGGAGCTCAAAAAAAAATGCATCGTAACAAGTCGGTAGACGTCCATCAGTTCACAATGATTCCAAAAGCGGATAT
CCCCCGCTCTACATTTGACTGTCAATCAACACATAAACTACATTCGATGCTGGCTTCCTAGTCCCTGTACTCGTAGACGAAATGTTGCCAGGCGAT
ACATTCCGCTGCAACATGACCGCCTTTGCGCGATTGTCTACACCACTCTATCCGATCATGGACAATATGCATTTGGATAGCTTCTTCTTCTTTGTGC
CAAATAGACTTGTCTGGTCAAATTGGCAAAAATTTATGGGGCAGCAGGCAAATCCTGCGGACTCGATTTCGTACGTAGTGCCCCAACAAGTAAC
CCCAGCTGGTGGGTACGCTATTGGCAGCCTTCAAGATTATATGGGTCTGCCAACGTAGGCCAAGTAGGTGCTGGTGGCACCGTAAGTCACTGTG
CCTTCTGGCCACGTGCTTACAACCTTATTTATAACGAATGGTTTCGGGACGAAACCTTCAAAATTCAGTAGTTGTAGATACTGGCGATGGTCCAG
ATAACGTAGCCAACTACACATTATTACGACGTGGAAAACGTAAAGACTATTTCACGTCAGCATTACCTTGGCCACAAAAGGGCGCAAGCGTTACT
TTACCGCTTGGAACATCCGCCCCAATATTACGCACTAACAATGCGCCTGTTTCCAGACTGTATAACGCTGGAACAAATACATTAAACGCAACCGCC
CAGGCTATTAACGTAGGTGTTACTGGTCAAATTACTGGCGGTGCTGACGGCTTGGCAAAATCATATGATCCTAATGGCGGTTTATATGCAGATTT
ATCAGCTGCAACCGCTGCAACAATTAATCAATTGCGTCAAAGCTTCCAGATTCAAAAACTTTTAGAAAGGGACGCCCGTGGCGGAACTCGATACA
CAGAAATTATCCGCAGCCATTTCGGGGTCGTTAGCCCCGATGCGCGTCTCCAACGGCCTGAATACATTGGAGGCGGTTCAACACACATTAATATC
AATCCAATCGCCCAGACGAATGGTACCGGAGCTTCCGGGACCACTACTCCTCTCGGTACACTTGGCGCTATGGGTACTGGGCTCGCTCACAATCA
TGGCTTTACTTATTCAAGCACTGAACATGGTGTAATTATCGGTCTCGTTTCAGTACGAGCCGATTTAACATACCAACAAGGTATGCACCGCATGTG
GAATCGTTCCACACGTTATGATTTCTATTTCCCTGCTTTCGCCACTTTGGGCGAACAAGCAGTATTAAATGAAGAAATCTACGTACGAGGCGATGC
CAACGATACAGGAGTGTTTGGATACCAAGAACGTTGGGCAGAATATCGTTATATGCCAAGCCGAATTTCCAGTCTGTTCCGTAGTACGGCAGCT
GGAACAATTGACGGCTGGCATTTAGCCCAACGGTTTACAACACTTCCAACTTTGAATAACACGTTTATTCAAGAAAATCCACCTGTCTCTCGAACC
CTTGCGGTCGGAGCAGCTGCCAACGGCCAGCAAATCATTTTTGATAGCTTTTTTGATATTAAAAAAGCACGGCCAATGCCAATGTACTCTGTACC
TGGCTTAATCGACCACTTCTAATGGCACTAGAAGCCGCTGCCTCAGGCGCCGCATCTGGCGCCGCTTTTGGACCTTACGGCTCCTTAATTGGAGC
CGGAATAGGTGCGGCCGCTAGTTATTTTGGTGGTCAAGAACAAAACGCTGCCAGCGCACAACAAGCTGCAGCAATGATGCAATTCCAAGATGG
TATGCGACGTACTGCATATCAAGACGCAGTAGCGGATCTTAAGGCTGCAGGTCTTAACCCTATGCTGGCTTATTCACAAGGCGGAGCCAAAGTC
CAGCCTGGTGCGCAAGCTCCAGTAGGAAATCCACTAGGTGAGGCTGGAAATTCAGCCCGTGAAGCTGCCATGGCAGTCGCCAATTTTAAACAAT
TACAAACTCAGAATATCCTGACACAATCGCAAGCCGAAAAAACGGACGCGGATACAAATCTATCACGTGATCAGGCAACATATACTCGAGCAAA
TACAGCTCGTGAAATTGCTCAGATGCCGGGATACGGCAAATTTGGTCAGCTTCGCGATGCCCAAATAGAGCAATTAAGGACATCAAGTGCATTA
CAAGCTGCACAACAGCGACAAGCGTTAAGTCAAAGTGCATATACAGACCAATT

>000212F|arrow

AGCTGAAATGTCTGGAACATCATATCAACGAGCAGTTGAAGATATGAAAAAAGCTGGGTTAAATCCCATGCTTGCGTATTCACAAGGCGGAGCC
ACAACACCAGCTGGAGCTATGGCCCAGATGCAAAATGTTCTCGGTAATGCAACTACGTCCGGAACCCAAGCTTATCAAACGGTTGCTCAAGCAA
ATCAAGCTATTGCTCAATCTAAACAAATTGAAGCTCAAACAGAACTCACAAGTAATCAAACAGATAATGTACGTGCTGATACGTTAAACAAATTG
GATGAAAATCCAAATATTAGAGCTCAATATAAACAAATACTTGCCGATACTTTCATGAAAAATGAAATAGGCAAAACATCAAGTGCTCAAGCTGC

TCAAGCTTTGGCACAATCTCGTTATTCAAACGAGTTAACAAAACTTGCTAAATCAGGGTCAGCTCCTAGTTCTAGCAAACCAATTTATCAAGACGT
AAAAAATATCGCCAAAGATGCGTATAGCGCATCTGGCGCAAAACGATACATCGATAACTATCGAGGTCAACCGATTCAACAAAATCGTACAAAT
AACCAACCACCAATGGAATGAAAATGACAAAGATTACAGCCCCATTTCTTCGTACTCCGTACAATTACGACACGATTGCTGCGTCAAATGAGTCA
GGGCTGCATTGTGAGGATGCAACTCTGACTCAGCAGCAATTTGCTGAAGAATGCGATATAATATATTATGGAAAAGTTTGGTATGACCGGACTT
ATTCCTCAAACTCCTTTAACGCCTCAATATGGCGACTTTAGTGGTGTCTATGACTACCACTCTGCTCTGAACCAGATTATGGCTTCAGACAACGAA
TTTATGGCTTTACCAGCCAATATTCGTGAACGATTCGCTAATGATCCCGCGAATCTAATAGATTTTCTAGAAAACCCTGAAAATCGCAGCGAAGCT
GAAAAAATGGGACTGGTAAAACCAGCCCAAACCGAGGTTTCAACCCCTGTTGGAACCTCGGAAGCACAGTTACCTACTTGATGTAACTGTGCTA
GGTGACACCAAACCACAAAAACACGATAAACAAGGACAGAAAAAATGATGCGTCGCAGACCAGCAAATAAGCAAAAGTCCGCTAGGACTTTCC
GTAAACATGCTTCACATACAAAACACGCAAATATGCGAAACTCGCCAATGCGTGGAGGCTGGAGACTCTAATAAAGTCCCCAGGCACCTCACAT
GCCTTGTTATCACCCTCTCAAAGCATTTCAATGCTTTGACAAATCAATTGTTTTCGACGAAGTTCGGAAACATGACATCGTTCGATCTTTAGACCTG
CCCTGTGGGCAGTGCGTTGGATGCCGTCTAGAACGATCAAGACAATGGGCTATTCGGTGCATGCACGAAGCCCAATTGCATAAAAACAACTCAT
TCATAACACTCACATATGACAATACACATCTCCCAAGCGATGGCTCTTTGGATCACAAAGACTTTCAACTGTTCCTTAAAACTTAGAAAAACTCTC
GCAAAAAGAGGACTTACAATCCGCTATTACATGGCTGGAGAATATGGTGAACTCTTCGCAAGACCCCACTTCCATGCCTGTATCTTCGGATACGA
CTTTCCTGATAAAAAATGGAAAAGGACTGCCTCTGGTTCTATGTTATATAGATCCGCAGAACTTGAAGCTCTCTGGCCATTTGGTTATACCACCAT
TGGAGATGTTACTTTCGAATCAGCCGCCTACGTGGCTAGATACATAATGAAAAACAAACAGGGAAAGATGCGGAATCTCATTACAAACGCATAC
ACCCTGAAACCGGCGAATATTTAGACTTAAAGCCGGAATATAATAAAATGTCTTTAAAACCGGGAATCGGTAAAGACTTTTATATAAAATATACT
TCGGATATATACCCGCAAGACTACGTAATACTTAGAGGTAAAAAGGTCAAACCACCAAAATACTATGACAAAATGTTTAAAATTGACCAACCTTA
TGAGTATGACGAATTACTTTACATGCGGGAAAATAACGCTAAACTTAATTCCGAAGACAATACACCAGAACGACTATCTGCAAAAGAACAAGTA
CTATGGCAAAACTTCAACTATTAAAACGTAACCTCTTAGGAAAATAATGAAACTTATCCTCGCTTCCGTAAAAGACCGTGCTGCTGAAGCATATGC
ACGACCAATGTTCGTACCTTCCTCTGGAGTAGCTATACGCTCTTTTTCAGATGAAATTAATCGTTCTGATACTGAAAATCAACTTTTTAATCACCCT
GATGACTTCGATCTATATGAATTCGGAACTTTGACGATTCAACTGGGTTATTCGATTTACATGAACAACCAAAACTCCTATCATTAGGAAAACAAG
TTAAACTTAAATAAAACAACCGAGGGGAAAAGAGATTTATCTTTCCCCCGGAACAACACTAAGGAAAAACATGCACCGCAATCAGTCAGTTAAT
ACTCACCGCTTCGCGATGGTACCTAGAGCCGATATACCACGTAGTAAATTCGATGCTCAAAAACACATAAACGACTTTCGATGCGGGCTATTTAA
TTCCTGTATATGTTGATGAAGTGCTCCCTGGGGACACTTTCAACTTAAAAATGACGGCATTTGCCCGTCTAGCAACGCCTTTATATCCAATCATGG
ACAACATGATTATGGATTCTTTCTTTTTCTTTGTACCCAATCGCCTTATATGGAATAACTGGCAAAAATTTATGGGTCAACAAGAAATCCAACAGA
CTCAATATCTTATATTGTCCCAACTCAAACAAGCCCAACAGATGGTTATGCCGTAGGCAGCCTTCAAGACTATATGGGCTTACCAACAGTAGGCC
AAATTGATACTGGCCGAACTATTACGCACTGTGCCTTTTGGCCACGTGCATACAATCTTATTTGGAACGAATGGTTCCGAGATGAAAATTTACAA
ACAAGCGCAGTAGTTGATAAGGGCGATGGCCCTGATACTTCCTCAAACTATGTGCTAAAACGTCGTGGTAAAAGACATGATTACTTTACGTCAGC
ATTACCATGGCCACAAAAAGGTGCGAGTGTCACCTTACCTTTAGGTACTACGGCTCCAATTAAATGGGATACCATTTCAGGAGACGCAACATCAA
ACGATAAATTTACGGTAATTCAAACAGATCCTGGAAATACGACTGCTTTAGCTAGATATGGCAACGCTTATGGTGTTAATACTGCTGGTGTAGTA
AATAACGTTTCTAATTTATATACCGACTTATCAGAAGCAACTGCTGCAACTGTCAATCAATTAAGACAGTCATTTCAAATTCAAAATTACTTGAAA
GGGATGCACGTGGCGGAACACGATACACAGAAATTATCCGGAGTCACTTTGGAGTTATTTCCCCAGACGCCCGTTTACAAAGGCCTGAATACCT
TGGAGGCGGTTCAACACCAATTAATGTTAATCCGATTGCTCAAACGTCGGGAACAAACGCTTCTGGAACGACTACCCCTTTGGGCAACCTTGCTG
CTATGGGTACTGCTCTCGCTCATAATCATGGATTTACTCAATCATTTACTGAGCATGGCGTTATTATTGGATTAGTATCCATTAGAGCAGATCTTAC
TTATCAACAAGGATTAGACCGTATGTGGTCTAGATCTACACGATATGACTTTTATTTCCCAGCATTTGCTACTCTAGGCGAACAATCTGTTTTGCA
AAAAGAAATTTATGCAACAGGAGATACTGCAGCCGACAATACTGTTTTTGGATATCAAGAACGCTGGGCGGAATATCGTTACAAACCATCTAAA
ATTACTGGTTTGTTCAAATCAACATCGGCGGGCACGATCGATGGTTGGCATTTGGCTCAAAAATTTACCGCTGCGCCTACTTTGAATAATACGTTT
ATTCAAGATACGCCTCCTGTATCACGTGTAGTAGCCGTTGGAGCAGCTGCAAATGGCCAACAATTCTTATTTGACTCATTTTTTGATGTCAAAATG
GCAAGACCAATGCCAATGTATTCAGTACCTGGCTTAATAGACCATTTCTAATGGGACTATTTGACGGAATTGCCGATTTAATCGGCCCTGCTATA
GCTATAGGAGCTGCCCCTGCTACTGGGGGACTCTCCTTAGCTGCACTTGCACCTGCAGCAATAGGTGCAGCAGGACAATACTTTGGAACACAAA
GTCAAAACGCAGCGAGTGCAGAACAAGCGAGTAATCAACAGAGATTTCA

>000183F|arrow

ATCAAATGATTTCCATAAGGCCAAGCCTTTAAAACGGAATCAGAAGTGAAGGTACGATCACCACCAACAGCGCGACCAAAAGCGCGACGGTCA
GAAGAAAAATCAACTCCAAACAACGCAATATGAAAATGCGGGCGTTTAGTTTGATCTCCGTACTCACCAGAAGCCACATAACGAAACTTGAAAC
CCGCCTTACGCAAACGCTTAAAAAAGCGTTGCAGGTCCTCTTTAAAAAGTTGACCATGTTCGGGAAGCCAAGCATCGTTATACGTGAGGTTCAGC
ATACAAGACACCTGATGCATCTGTTGCTCGTGAGTGATACGGATGGCCCATTCTCTCGAATAAGCCAGCCTGCACTCTATACACTGTCCGCACTTG
ATAGGGCCGTGTTGAGGGTGTGACCAAAGAGTTGAGCACACCTAAGCCTTAAAGACGGATACCACCACGCATAGGACCAGCAGTGATGTTGAT
CAACTTGGTGGTCTTGACGTTGCGCTTAAAGCTAGAAGCACTAGCTTGCTTGTTAGCATTGTGACGATGCAAAGGTTTCATGATGAACTCCATTA
GAACAGAAAACAAAAAAGGTGTCAATAGGCACAGTTACATCAAGTAGCGAACTGTGCCAAAAGCAGCTTTAAGCTGCAGGAGCCGCAGGAGC
GGCATCAACAACGGAAGGACTCTCCTTGACTTGCGGTAGAGCAAGACCAAGGCGAACCGCCTCACCAGTGTTGTCAGGATTAGAAAAAAATTCC
AAAAATTCTTGCGGAGAATTGTGGAAACGAGCACGGACCTTAGCGTCCATGCGCATGAAATTTTCGTCAGCTTGACGAACAACATTCATAGCAG

ATTGAAATCAAAAACACCCTCATAGTCAACATACTGAGGCATAGAAGCTGGCTCAGGTAAATGACCAGTCTTCATAAAACGATCAACAATAGTGT
TGATATCAGATTCCTCTTTAAACTGCTGTTGAGTCAAAGACGAATCCATACACTTAAGCCCTGTCTCATTAGAGCGGGCATCGTGATTATCATAAG
CAGAAATAAACTTCATAAAAAACTCCTAACGTTTAGACAACATACGGAAACAGAAATCAAGGAATCGATCATGGGCTTATATTGCCCAAAATCTT
TACCAAAACTCTCAGCTCTATTAATTGCATCCTTATCAAGTTGCAACAAATCGCCTTCGAGCATAGTCTTAACAGTCAACCAAGTAAGCTGCTTAG
CACGCTGCTGTTCAGTCAAAGACTGAAAGGCAGTGAGCTTAGAAGCAGCATCAAGCTGCTTAGCAGCAGCAATCAAACGATCACCCTCAAGAGG
AATATTCTTAATTTCGGCAACAATCTTAGCAGATTGTGTCTCCATCATAGAAATATGAGAACGCTTTTCGTCAGCAGTAGCACCTGCAAGCCCAGT
CTGGGCCTCAAGCAAATAACGCTGAGCACGCTTGTTAATAGTATCAGCACCAACGTTTTCAACATCGGCTGAAGCCTTAGAAACACGAGAAGAA
GACTCAGCAGAAGACAAACCAGCAGAAACAGCATTCTGCATAGGCATAACAGTACCAGGAGGCGTAGAAGCACCACCGCCCTTAACGTAAGCA
AGCATAGGATTAAGGCCAGCAGCCTCCAAATCCTTAACTTGACGTTGATAAGCGGTATTACTCATACGCTCCTGAAAAGCTGTATTCTCAGCAGC
AAGCTCACGGTTCTGGGCATTGGTATCTTGTTGACCAAGAAAACCAGCGACACCAGAAGCAACGGAAGCAATAGGGGCAGTAAGCCAATCGAG
CATAATGAACCTTTCGGCTCATGCGGTGTAAACCGCACGAGCTTAAAAATGATCGATAAGACCAGGTACAGAGTACATAGGCAAAGGACGAGC
AGCATTAATATTAAAGAAGGCATCGAGAAGAAGCTGCTGACCATTTGCACCAGTACCAACAGCCAAATTGCGAGCAAGAGGCGGAGTGTCCTG
GATAAAAGTAGAATTAAGAGTAGGCAAAGAAGTAAACTTCTGTGCATAATGCCACGGATCAATAGTACCCGCAGCAGTAGAACGGAAAAGACC
GGTAATCTCAGAAGGGTTGTAACGATACTCAGCCCAACGCTCCTGATAACCGAAAACTTGGGAATCAGTAGAACCACCAGTTACATAAATTTCCT
TGTTCAAAACAGCTTGTTCACCAAGCATAGCAAACACAGGAAAATAAAAATCGTAACGAGTAGAACGCGACCAATGTCGGCGAAGACCTTGCTG
ATAAGTAAGATCAGCGCGAACAGAAACAACACCAATCACATAACCGTGTTCCACGAATGATTGAGTGAAACCATGGCCTTTAGCCATGTAAGTA
CCCATAGCAGCCAAATTACCTTGCGGTGTAGTCTGTCCGGAAGTACCGGTACCAGACGTTTGAGCAATGGGAGAAATATTTATAGGTGTTGAAC
CACCACCCAAATATTCAGGACGCTGTAAACGAGCGTCGGGCGAAGTAACGCCAAAATGAGATCTCAAAATCTCGGTATAACGAGTACCACCACG
AGCATCACGCTCTAGCAGCTTTTGAATCTGAAAACTCTGACGCAACTGGTTGATAGTTGCAGCAGTAGCCTGGCTAAGATCAGCATAAAGACCTG
AAGCACCAGAAGTAACAACACCATAAGCTTGATTAGCACCAGCAACAGTAACAGTGTTATCAGGCAAAGCCTTGTTATAACCACCAGCCTCTGCA
TACATAGCACCACCAGACTGACGCAAACCATATGTTGTAGTGCCATTAGTAGTAAGGCCAAGAACCTTACCATCACCATAAACAGGCGCAGACG
TACCCAAAGGCAAACTTACAGCATTATTGCCTTTTTGGGGCCAAGGCAACGAGCCAGTGAAATAATCATGACGCTTGCCACGTCGAAGGATTGC
GTAGTTAGTAGAGGCGGAGGCGTCAGGGCCATCGCCTTTGTTAACCACAACGGAATTCTGAAGGTTTTCATCACGAAACCATTGATTATAAATTA
AATTGTAGGCACGGGCAGGTAACGCCGAATGTGAAACCGTATTACCAGCGGTAACTTGGCCAACAGTCGGTAAACCAAGGTAGTCCTGTAAGG
ACCCGATAGCGTATCCACCAGCTGGGGATACTTGTTGAGGGATAGTGTAAGAAATGGAATCGGCAGGATTATCCTGCTCTCCCATAAACTTAAC
CCAATTGCTCCAAACCAAACGATTAGGAACAAAGAAAAAGAACGAGTCCAGATGGAGATTATCCATAACTGGAAAGATGGGGGTCGCAAGACG
ACCGAACATAGTGACGTTGACATTAAAAGTATCACCGGGCAAGACCTCCTCACACATGATAGGAACAATCAGACCACTGTCAAAAGTAGTCTTA
AGAGTTTTCTGCATAGCGAATCGGGAACGGGGAATGTCAGAACGGGGAACCATTGCAAAATTATGAGCATCAACCGATTTATTGTGGAACATAA
AAAACTCCAAAAAGAAAAAAGCACCCCCGAAGGGGTGCAAGGGTCAGACAGACTGCAAAACATCCTTGGCACGAACCAAGACCTGGGGAACA
GAAATAGAAAATTCGCCGGTATTATCGTTAAACTCACCGAGCAAATAAAGATCGAAGTCATCAGGATGCTTATTCAGCTGATTATCAGCAGCAGC
ACGATTGACTTCGTCAGTAAAATCGCGAATGGCAACATTGCGATGGGGAACAAAAAAAGGACGATTAAAGACTTCAGCTGCGCGATCTTTAACA
CAAACAACAAATAAATGCATGATATGACCTTAAATATTTATAAATTACGTTTAGATAAATTAGAACGAGAAGTAGAAACAAGAGAACGAGAAAC
CTTACGAACAGGAAGATTCTCAAACGCTATACGCTCAACTTCCATCTCGGCTCGCGCCGAAGAACGATACTGCATGTCCAGGGCTAAATCGGACC
CAACCTCCTTCAATAAAGTTTTGTAAAAACGGGGAACAGGAGCTTTACTACCTTGTAAGGTAATGACAGAAGCATGCGGAAAAACATCCGACAT
AAAAAAATCCCGAAACCAAGAACGACCAATTCCTTTAGACATAAGCATAAACTCGGGATTAGGCAACACAACTTCACCAGTAACATCATCAATAA
ACAATGGCTCAGGTTGTTGCAAGCCCTTAATCTTTTTCAAGATATAGCGGGCAATGTATGCAGCGGACTCAAAATTAAGAGTACCA

>000117F|arrow

TTCAAAATTCAGTAGTTGTAGATACTGGCGATGGTCCAGATAACGTAGCCAACTACACATTATTACGACGTGGAAAACGTAAAGACTATTTCACG
TCAGCATTACCTTGGCCACAAAAGGGCGCAAGCGTTACTTTACCGCTTGGAACATCCGCCCCAATATTACGCACTAACAATGCGCCTGTTTCCAG
ACTGTATAACGCTGGAACAAATACATTAAACGCAACCGCCCAGGCTATTAACGTAGGTGTTACTGGTCAAATTACTGGCGGTGCTGACGGCTTG
GCAAAATCATATGATCCTAATGGCGGTTTATATGCAGATTTATCAGCTGCAACCGCTGCAACAATTAATCAATTGCGTCAAAGCTTCCAGATTCAA
AAACTTTTAGAAAGGGACGCCCGTGGCGGAACTCGATACACAGAAATTATCCGCAGCCATTTCGGGGTCGTTAGCCCCGATGCGCGTCTCCAAC
GGCCTGAATACATTGGAGGCGGTTCAACACACATTAATATCAATCCAATCGCCCAGACGAATGGTACCGGAGCTTCCGGGACCACTACTCCTCTC
GGTACACTTGGCGCTATGGGTACTGGGCTCGCTCACAATCATGGCTTTACTTATTCAAGCACTGAACATGGTGTAATTATCGGTCTCGTTTCAGTA
CGAGCCGATTTAACATACCAACAAGGTATGCACCGCATGTGGAATCGTTCCACACGTTATGATTTTTTTCCCTGCTTTCGCCACTTTGGGCGAACA
AGCAGTATTAAATGAAGAAATCTACGTACGAGGCGATGCCAACGATACAGGAGTGTTTGGATACCAAGAACGTTGGGCAGAATATCGTTATATG
CCAAGCCGAATTTCCAGTCTGTTCCGTAGTACGGCAGCTGGAACAATTGACGGCTGGCATTTAGCCCAACGGTTTACAACACTTCCAACTTTGAA
TAACACGTTTATTCAAGAAAATCCACCTGTCTCTCGAACCCTTGCGGTCGGAGCAGCTGCCAACGGCCAGCAAATCATTTTTGATAGCTTTTTTGA
TATTAAAAAAGCACGGCCAATGCCAATGTACTCTGTACCTGGCTTAATCGACCACTTCTAATGGCACTAGAAGCCGCTGCCTCAGGCGCCGCATC
TGGCGCCGCTTTTGGACCTTACGGCTCCTTAATTGGAGCCGGAATAGGTGCGGCCGCTAGTTATTTTGGTGGTCAAGAACAAAACGCTGCCAGC
GCACAACAAGCTGCAGCAATGATGCAATTCCAAGATGGTATGCGACGTACTGCATATCAAGACGCAGTAGCGGATCTTAAGGCTGCAGGTCTTA

ACCCTAGGCCTGGCTGTATGTCACAATGCGAGACCAAAGTCCAGGACTGGTGCGCAAGCTCCAGTAGGAAATCCCCTAGGTGACGCTAGGAAA
TTCAGCCCTGTGAAGCTGCCATGGCAGTCCGCCAATTTTAAACAATTACAATCAGAACTATCCTGACACAATCGCAAAGCCGAAAAAACGGACGC
GGATACACATCTATTCACGTGAGACAGGCCAACATATACTCGAGCAAATTACAGTCTCGTGAAAATTGCTCAGATTGCCGAAGGGATAACGGCC
ATTGTGGTCAGCTACGCGATGCCCAAAGTAGAGCAATTAAGGACATCAAGTTGCAAGTACAAGCTGCACAACAGCGACAAGCGTTAAGTCAAGT
GGCATATACAGACCAATTAGAGCGAAGTAGCGCAAACTGGATCAGCGCCATCCAGTAACTAAACCACTTATCAAGATGTTAAAGGCTATTTCAC
AATAGCCAATATGATAAAATATCAAAAAGTATCTACCATTTGGTGAAAAATGTAAATGAAAACATCAAACTTAGAACCGCATACAACTATGACCA
CGGATGCTGCGTCAAATGAGTCAGGGTTGGCTTGTGAGGAGCCAACTCTGGCTCAGCAGCATTATAAAGACGAATGCGATATAATACTATCCTG
GAATCGTTTAACGTTACAGGCCTATTACCTCAAAGTCCCGCTGCCGCCTCAATATGGCGATTTCAGCGGAATTACTGACTATCATAGCGCCTTGAA
TAAGGTAATGAACGCTATGGAAGAATTTGATAACTTACCGGCTCAAATTCGTGCTAGGTTCGAAAACGAACCAGCAAACCTGATTGAGTTCTTGC
AAGACGAGAAAAATCGACCAGAAGCCGAGAAACTCGGCCTGGTCGAAAGAGCCATTTCGGAAGAAATGGCGATAAGCACAGTTACTCCACTTG
ATGTAACTGTGCTAGGTGACACCAACACCAAAAATATCTGATAAACGAGGCCAAAAATTATGCTTTATAGAAAACAAACAAACAAGCGCAAAAG
CGCTAAATCGTTCCGTAGGAACACTTCAAAAACTAAAGCTGCAAATATGCAAAAAGCCCCGCAAAGAGGGGGCTGGCGGCTCTAATAAAGCGC
CAGGCTACCTCACATGGCCTGTTATCACCCACTGACTGCTTATTTAAGTAAGCATCAGACAAACTATAAGACCGGCAAATCTTATCGCCGTGTCGC
ATTCAAAGAATCTGACGAGCATGATCGTCAGATTTCACTGCCCTGCGGCCAATGCGTTGGCTGCAGGCTAGAAAAATCACGTCAATGGGCCATG
CGTTGCATGCATGAAGCCCAATTGCACGAAAAAAACTGCTTTATAACCCTCACATACAACAATGAAAACCTTCCACAAACTGGATCGCTTGTCAA
AAGCGACTTCCAAAAGTTCCTTAAGCGCTTCAGAAAATCCATTGCACCTGCAAAATTACGTTACTACATGGCTGGAGAATACGGCACAAGTTTCG
GCAGACCTCACTTCCATGCCTGTATCTTCGGATACGATTTTCATGATAAGAAACTATTCAAAAGGACTCCCTCTGGTTCTCTCATATATACATCCGA
CCACCTTGCAACCCTCTGGCCACATGGTTATTCCTCCATTGGAGACGTTACATTCGAGTCAGCTGCTTACGTTGCTCGATATATTATGCAAAAATA
CAACGGCCAGATGGAAGAAAACAAACATATAACAAAGGATGAGCATTACACATACTGTGATATAGAAACAGGGGAATTAATAAAGCTATTACC
AGAATATAACAATATGAGCCTTAAACCAGGCATTGGTGCTGAGTGGTACAAAAAATATCGTTCCGACGTATATCCCCATGACTACGTTGTAGTCA
ACGGAAAAAGGGTAAAACCCCCAAAATACTATGACAAAAAATATAAATCAGATTATCCATATGAATACGAAGAATTACTCCACAAACGTGAAAC
TTCTGCTAAACTCAACCACGAAGACAATACCTATGCCAGACTTGCCGTAAAGGAAAAAGTCACAAAGGCCAAACTTCAATTATTAAAACGTAACC
TCACTTAGGAAATCCTCATGAAATTAGTACTCTGTACCGTTAAAGACCGCGCAGCAGATGCGTTCGGTCGTCCAATGTTCGTCCGTTCTATCGGC
GAAGCAATCCGGAGCTTTAGCGACGAAGTCAATCGCCAGAGCGATGACAATCAACTTTATAACCATTCCGACGATTTCGACCTATTTGAATTAGG
CGAATTCGACGATAATACGGGTTTGTTCCAATTACATGAACAACCCAAACTTGTATCCTTAGGGAAACAAGTCAAAATTACTGATAAAAACTAAG
CGTAGAGTAAAAAGGGGGAAACCCCTTTTTCTCACGCAACTAGGCCTAGGAGCTCAAAAAAATGCATCGTAACAAGTCGGTAGACGTCCATCAG
TTCACAATGATTCCAAAAGCGGATATCCCCCGCTCTACATTTGACTGTCAATCAACACATAAAACTACATTCGATGCTGGCTTTCTAGTCCCTGTAC
TCGTAGACGAAATGTTGCCAGGCGATACATTCCGCTGCAACATGACCGCCTTTGCGCGATTGTCTACACCACTCTATCCGATCATGGACAATATG
CATTTGGATAGCTTCTTCTTCTTTGTGCCAAATAGACTTGTCTGGTCAAATTGGCAAAAATTTATGGGGCAGCAGGCAAATCCTGCGGACTCGATT
TCGTACGTAGTGCCCCAACAAGTAACCCCAGCTGGTGGTTACGCTATTGGCAGCCTTCAAGATTATATGGGTCTGCCAACTGTAGGCCAAGTAG
GTGCTGGTGGCACCGTAAGTCACTGTGCCTTCTGGCCACGTGCTTACAACCTTATTTATAACGAATGGTTTCGGGACGAAAACC

>000032F|arrow

ACTACGATACACAGCATGTGTAGTTGAATTAGCATAAATCTGCTTCAAAGCACCAGCACCGTCAACTAATGACAACGTTGTTGAATCATTAGTAA
CAGACGTCTTAATAGGAGCAGACGTGCCTAAAGGCAACGTAACTGCATCGCCTTTCTGAGGCCAAGGCAAGGCACCAGTAAAATAATCCTTACG
CTTACCGCGTCGAACCATAGCATAATCACTCGGAGTATCAGGACCGTCACCCTTGTGAACGGTAACAGAATTTTGTAAATTCTCATCCCTAAACCA
CTCGTTATAAATCAAATTATAAGCACGTAACGGTAACGCGTTATGCGTAACCGTATTAGTACCAGTAATCTGACCAGCCGTAGGCAAACCAAAAT
GATCAAAAATAGACCTACTGCATAACCACCAGCAGTAGAAGTAATCTGTGGAACTACATACGAAATAGAATCACCTGGGTTCGCTTGCTCACCCA
TAAACTTAACCCAATTGTTCCAAACTAATCTATTTGGAACAAAGAAAAAGAAAGTATCTAAATGCAAATTGTCCATAACTGGAAACAAAGGCGTT
GCCAAACGGGCAAACATTGTAGCCTTTACATTGTGCATGTCCCCTGGGAGCACTTCATCACAATAAATAGGAACTAGATAACCACCATCAAAAGT
AGTTTTATGCGCATATTGCGTATCAAAACTAGAACGCGGAATTTCCGCTTTAGGAACCATAGCAAAACTATGTGAGCTTACTGACTTATTACGAT
GCATAACAATCTCCCGAAGTATTCCGAACCACTAGCAAGCTAGTGATCCGGCTTAAAAAAACATTACTCGCCATCGCGAATCATAACATCCTTAG
CTCTAGAAATAAGCTTGGGAGAACCAAGCAAATCCATAGTACCAGAATTATCATCAAACGTACCAAAATAATATAACTGAAAATCATCAGGGTTT
TATATAACTGATTATCATCGCTAGCGCGATTAACTTCGTCCTGAAACTGACGAACAGCAACACCCTCAGATGCAACAAAAGCTGGACGACCATAC
GCACCAGCTGCAGTATCTAAAATAGAAACAATAACCATCTTCATAAAACTCCTTTAAATCTTACGTTTTAAAAGCGATAACTTAGCCAAAGCGACT
TTTTCCTTTACAGCCAAACGCTCTAAAGTGTTATCTTCAAAATGTGAGCGACCTTCTAGCTCACGAGCATATTGTATACCATCAAATTCTTCTGGAA
ACAATACTTTAAACTTATTATCATAAAACCGTGGTGGTCGGCACTTTTTGCCACGCACCACAACAGAGTCAGTCGTATAAACGTCTGACATGAACT
TATCAAACCAAGCCTGACCAATGCCAGGCTTAAGAGACATCTTATTAAATTCAGCTTACGCTGATAATCTCACCAGTATCTAAAATCACAATACTG
ATAATGCGCATCTGCATCAACCACTTCGTGGTTTCTCATTAACGGTTTTACCGTTAATCTTCTTCATAATATACGAGCAACATAAGCAGCAGATTCA
AATTGACATCCACCAATAGAATTATAGCCTATACGGCCAAGTTCTCAGTATCTTTGACGTATATAAGATAGACCCAGTCTGCGTTCTTTTGAAAAC
TTCTTATCTTCAAAATCAAGCCCAAAGATACAAGCATGGAAATGAGGACGATCGAAAGACTCACCATATTCACCTGCCATATAAAAACGAATAGT
CTTGCCAGTATAGCGTTTTTCTCAATCGTTTCATAACACAATGAAAATCTTCATAATGCAAAGACATATCCTTAGGACAATGCTCTGAGCATATGT

CAAAGTAATAAACAATTACTAGTATGGTTTGTGCCTCATGCATACCTCTAACGGCCCACTGGACGGGACCGTTCAAGGCGACAAACCAACACACT
GACCACAAGGCAATGACACAGGTCCGGACTACGTCCGCCCCTGGTACTCTCCCGCCAAATAATAGACTTGTCAGCGCATTGATAAGCCGTTAAC
GGCTTATAACACGCCATAAATTACAGTCTAAAACCACCGCGCTGCGGTGAAGTACGCATATTAATGCTCTTGGTCTTGCTTACGCACGACGAAAC
TTCTTAGCTGCGCCAGCTTGCTCATTGGTTTTCTATAAAGGCTCATAACATTGCACTCCGTAGTTAATAATGTGGTTTGTGTGTCACTAGCACAGTT
ACATCAAGTAGAGTAACTGTGCTGCCATCCGCTTACGCGTCTGGCTTAGGTGTTCTACTGCAGAAACGACGGTTCAACCACAGGTTTACCGTCAC
ATAAGACCAATCTGAATCGCTCTCATCACGATTCTATTCATTCTGTAAATAATTTAACAAAGCATTAGGATCGTTATCAAACTTAGCCCTAATCTTA
GCTGGCAAAGCCATAAAAGCCTCATCAAGGCACGAATAGCATTCATGCGTGTGATAGTCAGATACACCGCTAAAATCGCCATATGATGGCTCTA
ATGGCGCCTGGGGGCAATTGCCCAGTAACGCCAAAACGCTCAACTATAATATTAATATCACATCGTCTCTCAGGTGTTGTTGAGCCAAACTACTC
GGATCTTTACATTCAAGACCAGTCTCTGTGAAACAAGAGCCATATCATAATTGTACGGATTACGTACAAAAGGTAAATTCGTTTTACTCATTTTCT
ACGACCTTCCAAGGACCAAGGAAAAGACTTATTCTTTTCCAAAACTTATCAAAATAGACTTACTACCTTTCTTAATATCGCGATACCAATATGGAT
CGCTAGACGGTGTTCGTAACTCTAAATTATTCTTAGCATCAATATCAGTCTTAATTGTTGATGCAGAATTAGCATTAACACGAGAACCAGCCTCGG
CCTGTTCCAAATAAAAACGAGCCGGTTCTTGTAAAACGCTGAGAAGCTAAATCAGCTTCTTCAGCAACCTTCCTAGCAGTCTGAACATTTAATTCA
CGTTGAGATTTAGCAACTTCCATCTGCTCACGCAATAAATCACTCTCAACATCACGCTTAACAGCTCCACTCAAATCAGACTTAACTGGAGAAGTA
GAAGATGCAGTAGCACCACTCGGAACAGTAGAACCACCTTTAGAATATGCAAGCATAGGACTCAAACCAGCCTTATTCAAATCAGTAACCATAC
GCTGATATTGCGTATTGGACATATCTTCTTGAAAGCGTCTATTTGCTTGCGCTTCAGCAGTATTATATTTTTGCTCTTCATTCTGACTCCAAGCAGA
AGTAGCAAAATCAAATAAACCACCAGCAACTGAATCAAACATACCCATACTAACGCGCTCCGCTTGTTTGCTGACTACTGGTTTCCCAGTAGTCCA
GCTTATATTACATTAGAAATGGTCAATCAAGCCAGGTACAGAATACATCGGCATTGGACGAGCCATCTTACAATCAAAAAACGCATCCATCAAAA
ACTGCTGACCATTAGCAGATGCACCAACAGCAGTAGTACGATCAATAGGTGGCGTTTCTTGAATAAACGTAGAATTCAAAGTCGGCAACGAAGT
GAACTTCTGAGCATAATGCCAAGGGTCAATCGTACCAGCTGAAGTCGACTTAAACAAACCTGTAATCTGAGAAGGTTTGTAACGGTACTCTGCCC
AACGTTCCTGGTATCCAAATACATCATCATCAGTTGAAGTACCAGTAACATAGATTTCCTTATTAAGAACAGCCTGTTCACCTAAATGAGCAAATA
CAGGGAAATAGAAATCATAACGTGTCTCACGAGACCACATCTTAGGTAAACCTTGCTGATATGTTAGATCAGCTCTTACGTTTACCAAACCAATT
ATGTATCCATGTTCTTGAGCATGATACGTAAAACCATGTCCACTAGCCAACGCAGTACCCATTGCAGCCAAGTTACCAAGCGGAGTAGCACCGCC
AGAAATCGAAGTAGCAGACGTTTGAGCAATAGGATTAACGTTGACATAAGTAGAACCACCACCAATATATTCAGGACGTTGTAAACGATAATCT
TGTGGAGTTACACCAAAATGAGCACGTAACAATTCTGTATAGCGTGTACCACCTCGCGCATCGCGCTCTAACAAACGCTGAATCTGGAAAGACT
GACGTAACTGGTTAATAGTTGCAGCCGTAGCATCACTTAAATCGGCATACAAACCAGTACCAGCAGTACCAGCGTTATT

>000128F|arrow

AGTATCAAGATGCAAGTTGTCCCATCACAGGAAATAACGGTGTGGCCAAACGTGCAAATGCAGTCATCTTTACACGATGAGTATCGCCAGGCAA
TACTTCATCACAATAAATAGGAACTAAAAACCAGCATCAAACGTGGTTTTATGAGCATATTGAGTATTAAAACTAGAGCGGGGAATGTCCGCTTT
AGGAACCATAGCAAATGAATGCGTACTTACTGACTTATTGCGAAACATAAACATCTCCCGTAGTTCCGTACCACTCTTTCGAGTGATACGGTATA
AAAAAACCTTACTCGCCTTCGCGAATCATTACATCTTTTGCACGAGCAATCAACTTAGGGCTTTCCAGTAGTTCAAAAACACCAGTGGCATCGTCA
AAAGACCCAAATAGAACATATGAAAATCATCAGGATGTTTATACAACTGATTATCTTCGCTAGCTCGATTGACTTCATCCTGAAACTGACGTACTG
CACACCTTCAGATGCAACATAAGCTGGACGACCAAAAGCATCTGCAGCAGTATCCTTAATAGAAACAATAACCATCTTCATAAAAACTCCTTAAA
TAGTACGTTTTAACAATGACAACTTAGCCAACGCAACTTTTTCCTTAACAGCAAGTCGCTCAAGCGTGGTCCTCATGCCTAGATCGACCTTCCATC
TCTCTGGCAAACTGAATCATATCGAATTCTTCAGGAAACTTCAACTTAAATTTATTATCATAAAACCGTGGTGGACGGCACTTTTTGCCACGCACC
ACAACGTGGTCTGACGTATAACGTGACATGTACTTATCTAACCACGATTGCCCGATACCGGGCTTCAATGACATCTTATTAAATCTGGCTTACGCT
GAATTATCTCACCAGTCTCTAAATCACAATATGAGAAGGCACCCGCATCAACCACTTCGTGGTTTTCATTGACAGTAAACCCCATTAATCTCTTCAT
AATATATTGCAACATAAGCAGCAGACTCAAAGTACATCACCAATTGTAGAATAGCAAACGGCCACAATTCTTCCTCTCTGACGTGTAGAGGATAG
AGCCAGTCTGCGTTCTTTTAAATATTTTCTTATCCGGAAAATCAAGACCAAACAGAAGCATGGAAATGAGGACGATCAAAGATTCACCATATTCA
CCTGCCATATAAAAACGTATCGTTTTCCAGTAAAACGCTTACGTAACCGCTTCATAAAAAGCTGATAATCATGTAATCCAATGACATATCCTTAGG
ACAATGCTCTGGAGCATATGTCAAGTAATAAAACAATTACTAGATGCTTGTGCCTCATGCATCCAACGAATCGCCCACTGACGTGAGCGTTCAAG
GCGACAACCAACACATGACCACAAGGCAATGATAGGGTACGGACTACATCCGCACCCGGTATTTCCCGCCAAATTATAGACCTTCACTGCATTGA
TAGCCGTTAAGGGCTTATAACAGGCCATAATTACAAACGATAGCCACCACGCTGGGGAGCGTGTCTCATATTAATTGACTTCGTCTTACTAGCAG
TTCTGCGAAATGACTTTGCAGATTATATTGTTTACTGGCTTCTTCGTAACATGATGAACTCCGTAGTTAAAATAGTGGTTTGGTGTCACCTAGCAC
AGTTACATCAGTAGATAACTGTGCTGGCCTCAGGATTTCATCCTTCGGCCTTAGGTGTTTCTGTAGAAACGATGGGTTCAACCACAGGTTGTCCA
TCAATAAGACCCAATTGAATCGCTTCATCACGATTCTGGTCGTTCTCAAGGAACTCCAATAATTTGACAGGATCATGGTCAAATCGGACTCTTAAT
TTCGCTGGCAGAGCCATGAAATCGTCCATAGTTGCGTTAATTTGATTCAACGCAGAATTGTAATCAGTAACACCACTAAAATCGCCGTATTGAGG
CGATACAGGGCCGTTGGAAGTTCCCCTGTACCCCGAAACGTTCAATGATGACATTAATGTCACATTCATCTTTCATATGCTGTTGAGCAAGACTTG
GGTCTTGACACAGCAACGCATCGTTACCGATGCTTCATCTTTATCATAGTTGTACGGATTACGTACAAATGGCAAATTCGCTTTACTCATTATTTGA
CTCCAATTCCCCAAGGGGTAGTTGATTAGGATTATTTTTGAGTTTATCAAAAATTTCTTTCGAAGCATCGATACCCTAGACCAAATAGCTGGGGAT
GGAGCCAATAGCTCTTAGTATTATATGACTGAGCGGTGTTAAATCCGCAGTCGTATTCTTTAAAGCAATATCAGCCAAAATACGTTTATTTTCGGC
TGTAATATTAGGAGCAGTAAGCAACTTATTAACAGTATCAGCCCTAGTATTAGCGGTACCAGCTTCAGTAGCTTCAGTCTGGGCGATAATCTGCT

TTTCTGTTTCTGATGCATTACGAATCTGTTGCATCATAGAAGCAGTATTAATAGCTGAATTAGTTGCATTACCTAAAACATTTTCCATAGTAGCAGT
GAACCAGATGGGGTAGAAGCACCACCACGTTGATAGGCTAACATAGGAGATAAACCAGCAGCTTCTAAATCTTTAACAGCACGCTGGTAACTTG
TTCCACTCATATCGGCTTGAAATCCATTGCCTCTGAGCCATCTCCTGATTAGCTTATTAGCAGTAGAACCTAAATAAGAACCAACAGCGCCCAAAG
CAGTACCGACACCAGGAGTAAAGAAACTAGAAGCTGAAGAGCTTAGAACCAACATTAGTAACCGCATCAAGTATTCCACCAAACATAACAAAAC
GCCCTTCGGTTGTTTCCTCACTACTCCTTACGGAGTAGTCGAGGTTATATAAAACATTAGAAATGATCAATTAGACCAGGTACGCTGTACATAGG
CATAGGTCTGGCCATCTGACAATCAAGAAAGCATCCATTAAAAATTGCTGACCATTTGCTGCAGCTCCGACCGCCGTAGTACGAGCAACTGGAG
GGGTCTCTTGAATAAACGTTGAATTCAACGTAGGAAGAGAAGTAAATCGCTGTCCATAATGCCAAGCATCAATAGTGCCAGCAGAAGTAGACCT
AAAGAAACCAGTAATTTGAGAAGGTTTGTAACGATACTCCGCCCAACGTTCTTGATAACCAAATACATCATTGTCGGCTGCAGTACCTTGAACAT
AATTTCTTTGTTCAAAATAGCTTGCTCACCCAAAGTGGCAAATACTGGGAATAAAAGTCATATCGTGTAGACCTAGACCACATCTTAGGAAGACC
CATGTTGATATGTGAGTGTCAGCACGTACAGAAACTAATCCGATAATGTATCCATGTTCTTGAGCAGCATACGTAAAAGCCGTGTCCCTGAGCCA
TGCAGTACCCATTGCAGCAAGGTTACCTTGCGGAGTAGCAGAACCAGTAACCGACGTTGCAGAAGTCTGAGCAATCGGATTAACATTAACAAGG
GTCGAACCTCCACCAATATTCAGGACGTTGTAAACGATAATCCTGTGGAGTTACTCCAAAGTGAGCACGTAGTAACTCAGTATAACGAGTACCAC
CACGTGCATCGCGCTCCAATAACTTCTGAATCTGGAAAGATTGACGAAGTTGGTTAATAGTCGCAGCAGTAGCAGTAGACAAATCAGCGTATAA
CTGATTAGTAGCAACACCAGCATTTGTACTATTAGACAAAGTATTAGAAGCTGAATTTAATCTCTTAAAGCACCAACAGCAGTTTGAAAAACACT
ATAACCAGCAGTACCATTGCCAGCTGGTATGTCAGAATAAATATTAGCACGTGTTCCTAACGGTAAAGAAACAGAAGCACCCTTCTGTGGCCAA
GGCAATGCACCAGTAAAATAATCTTTACGCTTACCACGTCTCAAAGTGTATAGTTAGCAGGAACATCACCTTGAGAATAACCAAGATATTAACGT
TACAGAATTCCTGTAAAGATCGTTTCATCTCTAATACTCCTTCGTATATTAAATCTCATTATAATAGCGCCCTGATACGGGCAAAACCATTATGCGT
ACTAGTATAGACGCAGTAATCTGACACAGCAGATAGGTAAACAAAATATCAAAATGGACACACATGCCAGAACCACCAGCACGGACTAGTAATA
GTAGGCACTACAAAAGAAAAGTGAGAAATCACCAGGATTCGTAGTTCACCCCATAAAACTTTTGGGCCAATTGTTCCAAAGTAAACGGATAGGT
ACAAAGAAAAGAA

>000123F|arrow

TTCAAAATTCAGTAGTTGTAGATACTGGCGATGGTCCAGATAACGTAGCCAACTACACATTATTACGACGTGGAAAACGTAAAGACTATTTCACG
TCAGCATTACCTTGGCCACAAAAGGGCGCAAGCGTTACTTTACCGCTTGGAACATCCGCCCCAATATTACGCACTAACAATGCGCCTGTTTCCAG
ACTGTATAACGCTGGAACAAATACATTAAACGCAACCGCCCAGGCTATTAACGTAGGTGTTACTGGTCAAATTACTGGCGGTGCTGACGGCTTG
GCAAAATCATATGATCCTAATGGCGGTTTATATGCAGATTTATCAGCTGCAACCGCTGCAACAATTAATCAATTGCGTCAAAGCTTCCAGATTCAA
AAACTTTTAGAAAGGGACGCCCGTGGCGGAACTCGATACACAGAAATTATCCGCAGCCATTTCGGGGTCGTTAGCCCCGATGCGCGTCTCCAAC
GGCCTGAATACATTGGAGGCGGTTCAACACACATTAATATCAATCCAATCGCCCAGACGAATGGTACCGGAGCTTCCGGGACCACTACTCCTCTC
GGTACACTTGGCGCTATGGGTACTGGGCTCGCTCACAATCATGGCTTTACTTATTCAAGCACTGAACATGGTGTAATTATCGGTCTCGTTTCAGTA
CGAGCCGATTTAACATACCAACAAGGTATGCACCGCATGTGGAATCGTTCCACACGTTATGATTTTTTTCCCTGCTTTCGCCACTTTGGGCGAACA
AGCAGTATTAAATGAAGAAATCTACGTACGAGGCGATGCCAACGATACAGGAGTGTTTGGATACCAAGAACGTTGGGCAGAATATCGTTATATG
CCAAGCCGAATTTCCAGTCTGTTCCGTAGTACGGCAGCTGGAACAATTGACGGCTGGCATTTAGCCCAACGGTTTACAACACTTCCAACTTTGAA
TAACACGTTTATTCAAGAAAATCCACCTGTCTCTCGAACCCTTGCGGTCGGAGCAGCTGCCAACGGCCAGCAAATCATTTTTGATAGCTTTTTTGA
TATTAAAAAAGCACGGCCAATGCCAATGTACTCTGTACCTGGCTTAATCGACCACTTCTAATGGCACTAGAAGCCGCTGCCTCAGGCGCCGCATC
TGGCGCCGCTTTTGGACCTTACGGCTCCTTAATTGGAGCCGGAATAGGTGCGGCCGCTAGTTATTTGGTGGTCAAGAACAAAACGCTGCCAGC
GCACAACAAGCTGCAGCAATGATGCAATTCCAAGATGGTATGCGACGTACTGCATATCAAGACGCAGTAGCGGATCTTAAGGCTGCAGGTCTTA
ACCCTATGCTGGCTTATTCACAAGGCGGAGCCAAAGTCCAGCCTGGTGCGCAAGCTCCAGTAGGAAATCCACTAGGTGAGGCTGGAAATTCAGC
CCGTGAAGCTGCCATGGCAGTCGCCAATTTTAAACAATTACAAACTCAGAATATCCTGACACAATCGCAAGCCGAAAAAAACGGACGCGGATACA
AATCTATCACGTGATCAGGCAACATATACTCGAGCAAATACAGCTCGTGAAATTGCTCAGATGCCGGGATACGGCAAATTTGGTCAGCTTCGCG
ATGCCCAAATAGAGCAATTAAGGACATCAAGTGCATTACAAGCTGCACAACAGCGACAAGCGTTAAGTCAAAGTGCATATACAGACCAATTAGA
GCGATTAGCGCAAACTGGATCAGCGCCATCCAGTACTACCAATTATCAATCAGATGTTAAAGGCTATTTACATAGCCAATATGATAAATATCAAA
AATATCTACCATTTGGAAAAATGAAATGAAAACAATCAAACTTAGAACCGCATACAACTATGACACGGATGCTGCGTCAAATGAGTCAGGGTTG
GCTTGTGAGGAGCCAACTCTGGCTCAGCAGCATTATAAAGACGAATGCGATATAAATACTATCCTGGAACGTTTTAACGTTACAGGCCTATTACC
TCAAAGTCCGCTGCCGCCTCAATATGGCGATTTCAGCGGAATTACTGACTATCATAGCGCCTTGAATAAGGTAATGAACGCTATGGAAGAATTTG
ATAACTTACCGGCTCAAATTCGTGCTAGGTTCGAAAACGAACCAGCAAACCTGATTGAGTTCTTGCAAGACGAGAAAAATCGACCAGAAGCCGA
GAAACTCGGCCTGGTCGAAAGAGCCATTTCGGAAGAAATGGCGATAAGCACAGTTACTCCACTTGATGTAACTGTGCTAGGTGACACCAACACC
AAAAATATCTGATAAACGAGGCCAAAAATTATGCTTTATAGAAAACAAACAAACAAGCGCAAAAGCGCTAAATCGTTCCGTAGGAACACTTCAA
AAACTAAAGCTGCAAATATGCAAAAAGCCCCGCAAAGAGGGGGCTGGCGGCTCTAATAAAGCGCCAGGCTACCTCACATGGCCTGTTATCACCC
ACTGACTGCTTATTTAAGTAAGCATCAGACAAACTATAAGACCGGCAAATCTTATCGCCGTGTCGCATTCAAAGAATCTGACGAGCATGATCGTC
AGATTTCACTGCCCTGCGGCCAATGCGTTGGCTGCAGGCTAGAAAAATCACGTCAATGGGCCATGCGTGCATGCATGAAGCCCAATTGCACGAA
AAAAACTGCTTTATAACCCTCACATACAACAATGAAAACCTTCCACAAACTGGATCGCTTGTCAAAAGCGACTTCCAAAAGTTCCTTAAGCGCTTC
AGAAAATCCATTGCACCTGCAAAATTACGTTACTACATGGCTGGAGAATACGGCACAAGTTTCGGCAGACCTCACTTCCATGCCTGTATCTTCGG

ATACGATTTTCATGATAAGAAACTATTCAAAAGGACTCCCTCTGGTTCTCTCATATATACATCCGACCACCTTGCAACCCTCTGGCCACATGGTTAT
TCCTCCATTGGAGACGTTACATTCGAGTCAGCTGCTTACGTTGCTCGATATATTATGCAAAAATACAACGGCCAGATGGAAGAAAACAAACATAT
AACAAAGGATGAGCATTACACATACTGTGATATAGAAACAGGGGAATTAATAAAGCTATTACCAGAATATAACAATATGAGCCTTAAACCAGGC
ATTGGTGCTGAGTGGTACAAAAATATCGTTCCGACGTATATCCCCATGACTACGTTGTAGTCAACGGAAAAAGGGTAAAACCCCCAAAATACTAT
GACAAAAAATATAAATCAGATTATCCATATGAATACGAAGAATTACTCCACAAACGTGAAACTTCTGCTAAACTCAACCACGAAGACAATACCTA
TGCCAGACTTGCCGTAAAGGAAAAAGTCACAAAGGCCAAACTTCAATTATTAAAACGTAACCTCACTTAGGAAATCCTCATGAAATTAGTACTCT
GTACCGTTAAAGACCGCGCAGCAGATGCGTTCGGTCGTCCAATGTTCGTCCGTTCTATCGGCGAAGCAATCCGGAGCTTTAGCGACGAAGTCAA
TCGCCAGAGCGATGACAATCAACTTTATAACCATTCCGACGATTTCGACCTATTTGAATTAGGCGAATTCGACGATAATACGGGTTTGTTCCAATT
ACATGAACAACCCAAACTTGTATCCTTAGGGAAACAAGTCAAAATTACTGATAAAAACTAAGCGTAGAGTAAAAAGGGGGAAACCCCTTTTTCTC
ACGCAACTAGGCCTAGGAGCTCAAAAAAATGCATCGTAACAAGTCGGTAGACGTCCATCAGTTCACAATGATTCCAAAAGCGGATATCCCCCGC
TCTACATTTGACTGTCAATCAACACATAAAACTACATTCGATGCTGGCTTCCTAGTCCCTGTACTCGTAGACGAAATGTTGCCAGGCGATACATTC
CGCTGCAACATGACCGCCTTTGCGCGATTGTCTACACCACTCTATCCGATCATGGACATATGCATTTGGATAGCTTCTTCTTCTTTGTGCCAAATAG
ACTTGTCTGGTCAAATTGGCAAAAATTTATGGGGCAGCAGGCAAATCCTGCGGACTCGATTCGTACGTAGTGCCCCAACAAGTAACCCCAGCTG
GTGGTTACGCTATTGGCAGCCTTCAAGATTATATGGGTCTGCCAACTGTAGGCCAAGTAGGTGCTGGTGGCACCGTAAGTCACTGTGCCTTCTG
GCCACGTGCTTACAACCTTATTTATAACGAATGGTTTCGGGACGAAAACC

>000087F|arrow

GTTGTTTTTATGCAATTGGGCTTCGTGCATGCACCGAATAGCCCATTGTCTTGATCGTTCTAGACGGCATCCAACGCACTGCCCACAGGGCAGGT
CTAAAGATCGCGAACGATGTCATGTTTCCGAACTTCGTCGAAAACAATTGATTTGTCAAAGCATTGAAATGCTTTGAGAGGGTGATAACAAGGC
ATGTGAGGTGCCTGGGGACTTTATTAGAGTCTCCAGCCTCCACGCATTGGCGAGTTTCGCATATTTGCGTGTTTTGTATGTGAAGCATGTTTACG
GAAAGTCCTAGCGGACTTTTGCTTTGCTGGTCTGCGACGCATCATTTTTTCTGTCCTTGTTATCGTGTTTTTGTGGTTTGGTGTCACCTAGCACAGT
TTACATCAGTAGGATAACTGTGCTCCGAGGTTCCACAGGGGTGAAACCTCGGTTTGGGCTGGTTTTACAGTCCATTTTTTCAGCTTCGCTGCGATT
TTCAGGTTTTCTAGAAATCTATTAGATTCGCGGATCATTAGCGAAATCGTTCACGTAATTGGCTGGTAAAGCCATAAATTCGTTGTCTGAAGCCAT
AATCTGGTTCAGAGCAGAGTGGTAGTCATAGACACACTAAAGTCGCCATATTGAGGCAGTATAAAGGAGTTTGAGGAATAAGTCCGGTCATACA
AACTTTTCCATACTATATTGAATCGCATTCTCAGCAAATTGCTGCTGAGTCAGAGTTGCATCCGCACAATGCAGCCCTGACTCATTTGACGCAGCA
ATCGATGTCGTAATTGTACGGAGTACGAAGAAATGGGGCTGTAATCTTTGTCCATTTTCATTCTCATTGGTGGTTGGTTATTTGTACGATTTGTTG
AATACGGTTGACCTCGATAGTTATCGATGTATCGTTTTGCGCCAGATGCGCTATACGCATCTTTGGCGATGTTTTTTACGTCTTGATAAATTGGTTT
GCTAGAACTAGGAGCTGACCTGATTTAGCAAGTTTTGTTAACTCGTTTGAATAACGAGATTGTGCCAAGCTTGAGCAGCTTGAGCACTTGATGTT
TTGCCTATTTCATTTTTCATGAAAGTATCGGCAAGTATTTGTTTATATTGAGCTCTAATATTTGGATTTTCATCCAATTTGTTTAACGTATCAGCACG
TACATTATCTGTTTGATTACTTGTGAGTTCTGTTTGAGCTTCAATTTGTTAGATTGAGCAATAGCTTGATTTGCTTGAGCAACCGTTTGATAAGCTT
GGGTTCCGGACGTAGTTGCATTACCGAGAACATTTGCATCTGGGCCATAGCTCCAGCTGGTGTTGTGGCTCCGCCTTGTGAATACGCAAGCATG
GGATTTAACCCAGCTTTTTTCATATCTTCAACTGCTCGTTGATATGATGTTCCAGACATTTCAGCTTGAAATCTCTGTTGATTACCTCGCTTGTTCTG
CACTCGCTGCGTTTTTGACTTTGTGTTCCAAAGTATTGTCCTGCTGCACCTATTGCTGCAGGTGCAAGTGCAGCTAAGGAGAGTCCCCCAGTAGC
AGGGGCAGCTCCTATAGCTATAGCAGGCCGATTAAATCGGCAATTCCGTCAACTAGTCCCATTAGAAATGGTCTATTAAGCCAGGTACTGAATAC
ATTGGCATTGGTCTTGCCATTTTGACATCAAAAAATGAGTCAAATAAGAATTGTTGGCCATTTGCAGCTGCTCCAACGGCTACTACACGTGATAC
AGGAGGCGTATCTTGAATAAACGTATTATTCAAAGTAGGCGCAGCGGTAAATTTTTGAGCCAAATGCCAACCATCGATCGTGCCGCCGATGTTG
ATTTGAACAAACCAGTAATTTTAGATGGTTTGTAACGATATTCCGCCCAGCGTTCTTGATATCCAAAACAGTATTGTCGGCTGCAGTATCTCCTGT
TGCATAAATTTCTTTTTGCAAAACAGATTGTTCGCCTAGAGTAGCAAATGCTGGGAAATAAAAGTCCTATCGTGTAGATCTAGACCACATACGGT
CTAATCCTTGTTGATAAGTAAGATCTGCTCTAATGGATACTAATCCAATAATAACGCCATGCTCAGTAAATGATTGAGTAAATCCATGATTATGAG
CGAGAGCAGTACCCATAGCAGCAAGGTTGCCCAAAGGGGTAGTCGTTCCAGAAGCGTTTGTTCCCGACGTTTGAGCAATCGGATTAACATTAAT
TGGTGTTGAACCGCCTCCAAGGTATTCAGGCCTTTGTAAACGGGCGTCTGGGGAAATAACTCCAAAGTGACTCCGGATAATTTCTGTGTATCGTG
TTCCGCCACGTGCATCCCTTTCAAGTAATTTTTGAATTTGAAATGACTGTCTTAATTGATTGACAGTTGCAGCAGTTGCTTCTGATAAGTCGGTATA
TAAATTAGAAACGTTATTTACTACACCAGCAGTATTAACACCATAAGCGTTGCCATATCTAGCTAAAGCAGTCGTATTTCCAGGATCTGTTTGAAT
TACCGTAAATTTATCGTTTGATGTTGCGTCTCCTGAAATGGTATCCCATTTAATTGGAGCCGTAGTACCTAAAGGTAAGGTGACACTCGCACCTTT
TTGTGGCCATGGTAATGCTGACGTAAAGTAATCATGTCTTTTACCACGACGTTTTAGCACATAGTTTGAGGAAGTATCAGGGCCATCGCCCTTATC
AACTACTGCGCTTGTTTGTAAATTTTCATCTCGGAACCATTCGTTCCAAATAAGATTGTATGCACGTGGCCAAAAGGCACAGTGCGTAATAGTTCG
GCCAGTATCAATTTGGCCTACTGTTGGTAAGCCCATATAGTCTTGAAGGCTGCCTACGGCATAACCATCTGTTGGGCTTGTTTGAGTTGGGACAA
TATAAGATATTGAGTCTGTTGGATTTCTTGTTGACCCATAAATTTTTGCCAGTTATTCCATATAAGGCGATTGGGTACAAAGAAAAAGAAAGAAT
CCATAATCATGTTGTCCATGATTGGATATAAAGGCGTTGCTAGACGGGCAAATGCCGTCATTTTTAAGTTGAAAGTGTCCCCAGGGAGCACTTCA
TCAACATATACAGGAATTAAATAGCCCGCATCGAAAGTCGTTTTATGTGTTTTTTGAGCATCGAATTTACTACGTGGTATATCGGCTCTAGGTACC
ATCGCGAAGCGGTGAGTATTAACTGACTGATTGCGGTGCATGTTTTTCCTTAGTGTTGTTCCGGGGGAAAGATAAATCTCTTTTCCCCTCGGTTGT
TTTATTTAAGTTTAACTTGTTTTCCTAATGATAGGAGTTTTGGTTGTTCATGTAAATCGAATAACCCAGTTGAATCGTCAAAAGTTCCGAATTCATA

TAGATCGAAGTCATCAGGGTGATTAAAAAGTTGATTTTCAGTATCAGAACGATTAATTTCATCTGAAAAAGAGCGTATAGCTACTCCAGAGGAA
GGTACGAACATTGGTCGTGCATATGCTTCAGCAGCACGGTCTTTTACGGAAGCGAGGATAAGTTTCATTATTTTCCTAAGTGAGGTTACGTTTTA
ATAGTTGAAGTTTTGCCATAGTTACTTGTTCTTTTGCAGATAGTCGTTCTGGTGTATTGTCTTCGGAATTAAGTTTAGCGTTATTTTCCCGCATGTA
AAGTAATTCGTCATACTCATAAGGTTGGTCAATTTTAAACATTTTGTCATAGTATTTTGGTGGTTTGACCTTTTTACCTCTAAGTATTACGTAGTCTT
GCGGGTATATATCCGAAGTATATTTTATATAAAAGTCTTTACCGATTCCCGGTTTTAAAGACATTTTATTATATTCCGGCTTTAAGTCTAAATATTC
GCCGGTTTCAGGGTGTATGCGTTTGTAATGAGATTCCGCATCTTTCCCTGTTTGTTTTTTCATTATGTATCTAGCCACGTAGGCGGCTGATTCGAA
AGTAACATCTCCAATGGTGGTATAACCAAATGGCCAGAGAGCTTCAAGTTCTGCGGATCTATATAACATAGAACCAGAGGCAGTCCTTTTCCATA
ATTTTTTATCAGGAAAGTCGTATCCGAAGATACAGGCATGGAAGTGGGGTCTTGCGAAGAGTTCACCATATTCTCCAGCCATGTAATAGCGGATT
GTAAGTCCTCTTTTTGCGAGAGTTTTTCTAAGTCTTTTAAGGAACAGTTGAAAGTCTTTGTGATCCAAAGAGCCATCGCTTGGGAGATGTGTATTG
TCATATGTGAGTGTTATGAATGA

>000129F|arrow

ACTCTACTTGATGTAACTGTGCTAGGTGACACCAAACCACTATTTTAACTACGGAGTTCATCATGTTACGAAGAAAGCCAGTAAACAAATATAAA
TCTGCAAAGTCATTTCGCAGAACTGCTAGTAAGACGAAGTCAATTAATATGAGACACGCTCCCCAGCGTGGTGGCTATCGTTTGTAATTATGGCC
TGTTATAAGCCCTTAACGGCTTATCAATGCAGTGACAGGTCTATAATTTGGCGGGAAATACCGGGTGCGGATGTAGTCCGTACCCTATCATTGCC
TTGTGGTCAGTGTGTTGGTTGTCGCCTTGAACGCTCACGTCAGTGGGCGATTCGTTGTATGCATGAGGCACAAATGCATACTAGTAATTGTTTTA
TTACTTTGACATATGCTCCAGAGCATTGTCCTAAGGATATGTCATTGGATTACAATGATTATCAGCTTTTTATGAAGCGGTTACGTAAGCGTTTTA
CTGGGAAAACGATACGTTTTTATATGGCAGGTGAATATGGTGAATCTTTTGATCGTCCTCATTTCCATGCTTGTCTGTTTGGTCTTGATTTTCCGGA
TAAGAAAATATTAAAAGAACGCAGACTGGCTCTATCCTCTACACGTCAGAGATTTTGGAAGAATTGTGGCCGTTTGGCTATTCTACAATTGGTGA
TGTTACTTTTGAGTCTGCTGCTTATGTTGCAAGATATATTATGAAGAAGATTAATGGGGTTACTGTCAATGAAACCACGAAGTGGTTGATGCGGG
TGCCCATTATCAATATTGTGATTTAGAGACTGGTGAGATAATTCAGCGTAAGCCAGAATTTAATAAGATGTCATTGAAGCCCGGTATCGGGCAAT
CGTGGTTAGATAAGTACATGTCAGACGTTTATACGTCAGACCACGTTGTGGTGCGTGGCAAAAAGTGCCGTCCACCACGGTTTTATGATAATAAA
TTTAAGTTGAAGTTTCCTGAAGAATTCGATATGATTCAGTTTGCCAGAGAGATGGAAGGTCGATCTAGGCATGAGGACAACACGCTTGAGCGAC
TTGCTGTTAAGGAAAAAGTTGCGTTGGCTAAGTTGTCATTGTTAAAACGTACTATTTAAGGAGTTTTTATGAAGATGGTTATTGTTTCTATTAAGG
ATACTGCTGCAGATGCTTTTGGTCGTCCAGCTTATGTTGCATCTGAAGGTGTTGCAGTACGTCAGTTTCAGGATGAAGTCAATCGAGCTAGCGAA
GATAATCAGTTGTATAAACATCCTGATGATTTTCATATGTTCTATTTGGGTCTTTTTGACGATGCCACTGGTGTTTTTGAACTACTGGAAAGCCCTA
AGTTGATTGCTCGTGCAAAAGATGTAATGATTCGCGAAGGCGAGTAAGGTTTTTTTATACCGTATCACTCGAAAGAGTGGTACGGAACTACGGG
AGATGTTTATGTTTCGCAATAAGTCAGTAAGTACGCATTCATTTGCTATGGTTCCTAAAGCGGACATTCCCCGCTCTAGTTTTAATACTCAATATGC
TCATAAAACCACGTTTGATGCTGGTTTTTAGTTCCTATTTATTGTGATGAAGTATTGCCTGGCGATACTCATCGTGTAAAGATGACTGCATTTGCA
CGTTTGGCCACACCGTTATTTCCTGTGATGGACAACTTGCATCTTGATACTTTCTTTTTCTTTGTACCTAATCGTTTACTTTGGAACAATTGGCCAAA
GTTTATGGGTGAACAAACGAATCCTGGTGATTCTATTTCTTTTGTAGTGCCTACTATTACTAGTCCTGCTGGTGGTTATGCTGTTTGTTCAATTTTT
GATTATTTTGGTTTACCTACTGCTGGTCAGATTACTGGCGCTAATACAGTAACGCATAATGTTTTGCCGTTACGTGCTTATAATGAGATTTATAAC
GAATGGTTTAGAGATGAAAACTTACAGAATTCTGTAACGTTAAATCTTGGTGATTCAGGTGATGTTCCTGCTAACTATACACTTTTGAGACGTGG
TAAGCGTAAAGATTATTTTACTGGTGCATTGCCTTGGCCACAGAAGGGTGCTTCTGTTTCTTTACCGTTAGGAACACGTGCTAATATTTATTCTGA
CATACCAGCTGGCAATGGTACTGCTGGTTATAGTGTTTTTCAAACTGCTGTTGGTGCTTTAAGAGAATTAAATTCAGCTTCTAATACTTTGTCTAAT
AGTACAAATGCTGGTGTTGCTACTAATCAGTTATACGCTGATTTGTCTACTGCTACTGCTGCGACTATTAACCAACTTCGTCAATCTTTCCAGATTC
AGAAGTTATTGGAGCGCGATGCACGTGGTGGTACTCGTTATACTGAGTTACTACGTGCTCACTTTGGAGTAACTCCACAGGATTATCGTTTACAA
CGTCCTGAATATATTGGTGGAGGTTCGACCCTTGTTAATGTTAATCCGATTGCTCAGACTTCTGCAACGTCGGTTACTGGTTCTGCTACTCCGCAA
GGTAACCTTGCTGCAATGGGTACTGCATTGGCTCAGGGACACGGCTTTACGTATGCTGCTCAAGAACATGGATACATTATCGGATTAGTTTCTGT
ACGTGCTGACCTCACATATCAACAGGGTCTTCCTAAGATGTGGTCTAGGTCTACACGATATGACTTTTATTTCCCAGTATTTGCCACTTTGGGTGA
GCAAGCTATTTTGAACAAAGAAATTTATGTTCAAGGTACTGCAGCCGACAATGATGTATTTGGTTATCAAGAACGTTGGGCGGAGTATCGTTACA
AACCTTCTCAAATTACTGGTTTCTTTAGGTCTACTTCTGCTGGCACTATTGATGCTTGGCATTATGGACAGCGATTTACTTCTCTTCCTACGTTGAAT
TCAACGTTTATTCAAGAGACCCCTCCAGTTGCTCGTACTACGGCGGTCGGAGCTGCAGCAAATGGTCAGCAATTTTTAATGGATGCTTTCTTTGAT
TGTCAGATGGCCAGACCTATGCCTATGTACAGCGTACCTGGTCTAATTGATCATTTCTAATGTTTTATATAACCTCGACTACTCCGTAGGAGTAGT
GAGGAAACAACCGAAGGGCGTTAGTTTATGTTTGGTGGAATACTTGATGCGGTTACTAATGTTGGTTCTAAGCTGTCTTCAGCTTCTAGTTTCTTT
ACTCCTGGTGTCGGTACTGCTTTGGCGCTGTTGGTTCTTATTTAGGTTCTACTTCTGCTAATAAGCTAATCAGGAGATGGCTCAGAGGCAAATGG
ATTTTCAAGCCGATATGAGTGGAACAAGTTACCAGCGTGCTGTTAAAAGATTTAGAAGCTGCTGGTTTATCTCCTATGTTAGCCTATCAACGTGG
TGGTGCTTCTACCCCATCTGGTTCAACTGCTACTATGGAAAATGTTTTAGGTAATGCAACTAATTCAGCTATTAATACTGCTTCTATGATGCAACA
GATTCGTAATGCATCAGAAACGAAAAGCAGATTATCGCCCAGACTGAAGCTACTGAAGCTGGTACCGCTAATACTAGGGCTGATACTGTTAATA
AGTTGCTTACTGCTCCTAATATTACAGCCGAAAATAAACGTATTTGGCTGATATTGCTTTAAAGAATACGACTGCGGATTTAACATCCGCTCAGTC
ATATAATACTAAGAGGCTATTGGCTCCATCCCCAGCTATTTGGTCTAGGGGTATCGATGCTTCGAAAGAAATTTTTGATAAACTCAAAAATAATCC
TAATCAACTAACCCCTTGGGGAATTGGAGTCAAATAATGAGTAAAGCGAATTTGCCATTTGTACGTATCCGTACAACTATGATAAAGATGAAGCA

TCGGTAAACGATGCGTTGCTGTGTCAAGACCCAAGTCTTGCTCAACAGCATATGAAAGATGAATGTGACATTAATGTCATCATTGAACGTTTCGG
GGTTACAGGGGAACTTCCAACGGCCCCTGTATCGCCTCAATACGGCGATTTAGTGGTGTTACTGATTACCATTCTGCGTTGAATCAAATTAACGC
AACTATGGACGATTCATGGCTCTGCCAGCGAAATTAAGAGTCCGATTTGACCATGATCCTGTCAAATTATTGGAGTTCCTTGAGACGACCAGATC
GTGATGAAGCGATTCAATTGGGTCTTATTGATGGACAACCTGTGGTTGAACCCATCGTTTCTACAGAAACACCTAAGGCCGAAGGATGAAATCCT
GAGGCCAGCACAGTT

>000188F|arrow

CAGCTTCAGTAGCTTCAGTCTGGGCGATAATCTGCTTTTCTGTTTCTGATGCATTACGAATCTGTTGCATCATAGAAGCAGTATTAATAGCTGAAT
TAGTTGCATTACCTAAAACATTTTCCATAGTAGCAGTTGAACCAGATGGGGTAGAAGCACCACCACGTTGATAGGCTAACATAGGAGATAAACC
AGCAGCTTCTAAATCTTTAACAGCACGCTGGTAACTTGTTCCACTCATATCGGCTTGAAAATCCATTTGCCTCTGAGCCATCTCCTGATTAGCTTTA
TTAGCAGAAGTAGAACCTAAATAAGAACCAACAGCGCCCAAAGCAGTACCGACACCAGGAGTAAAGAAACTAGAAGCTGAAGACAGCTTAGAA
CCAACATTAGTAACCGCATCAAGTATTCCACCAAACATAAACTAACGCCCTTCGGTTGTTTCCTCACTACTCCTTACGGAGTAGTCGAGGTTATAT
AAAACATTAGAAATGATCAATTAGACCAGGTACGCTGTACATAGGCATAGGTCTGGCCATCTGACAATCAAGAAAGCATCCATTAAAAATTGCT
GACCATTTGCTGCAGCTCCGACCGCCGTAGTACGAGCAACTGGAGGGGTCTCTTGAATAAACGTTGAATTCAACGTAGGAAGAGAAGTAAATCG
CTGTCCATAATGCCAAGCATCAATAGTGCCAGCAGAAGTAGACCTAAAGAAACCAGTAATTTGAGAAGGTTTGTAACGATACTCCGCCCAACGTT
CTTGATAACCAAATACATCATTGTCGCTGCAGTACCTTGAACATAAATTTCTTTGTTCAAAATAGCTTGCTCACCCAAAGTGGCAAATACTGGGAA
ATAAAAGTCATATCGTGTAGACCTAGACCACATCTTAGGAAGACCCTGTTGATATGTGAGGTCAGCACGTACAGAAACTAATCCGATAATGTATC
CATGTTCTTGAGCAGCATACGTAAAGCCGTGTCCCTGAGCCAATGCAGTACCCATTGCAGCAAGGTTACCTTGCGGAGTAGCAGAACCAGTAAC
CGACGTTGCAGAAGTCTGAGCAATCGGATTAACATTAACAAGGGTCGAACCTCCACCAATATATTCAGGACGTTGTAAACGATAATCCTGTGGA
GTTACTCCAAAGTGAGCACGTAGTAACTCAGTATAACGAGTACCACCACGTGCATCGCGCTCCAATAACTTCTGAATCTGGAAAGATTGACGAAG
TTGGTTAATAGTCGCAGCAGTAGCAGTAGACAAATCAGCGTATAACTGATTAGTAGCAACACCAGCATTTGTACTATTAGACAAAGTATTAGAAG
CTGAATTTAATTCTCTTAAAGCACCAACAGCAGTTTGAAAAACACTATAACCAGCAGTACCATTGCCAGCTGGTATGTCAGAATAAATATTAGCAC
GTGTTCCTAACGGTAAAGAAACAGAAGCACCCTTCTGTGGCCAAGGCAATGCACCAGTAAAATAATCTTTACGCTTACCACGTCTCAAAAGTGTA
TAGTTAGCAGGAACATCACCTGAATCACCAAGATTTAACGTTACAGAATTCTGTAAGTTTTCATCTCTAAACCATTCGTTATAAATCTCATTATAAG
CACGTAACGGCAAAACATTATGCGTTACTGTATTAGCGCCAGTAATCTGACCAGCAGTAGGTAAACCAAAATAATCAAAAATTGAACAAACAGC
ATAACCACCAGCAGGACTAGTAATAGTAGGCACTACAAAAGAAATAGAATCACCAGGATTCGTTTGTTCACCCATAAACTTTGGCCAATTGTTCC
AAAGTAAACGATTAGGTACAAAGAAAAAGAAAGTATCAAGATGCAAGTTGTCCATCACAGGAAATAACGGTGTGGCCAAACGTGCAAATGCAG
TCATCTTTACACGATGAGTATCGCCAGGCAATACTTCATCACAATAAATAGGAACTAAAAAACCAGCATCAAACGTGGTTTTATGAGCATATTGA
GTATTAAAACTAGAGCGGGGAATGTCCGCTTTAGGAACCATAGCAAATGAATGCGTACTTACTGACTTATTGCGAAACATAAACATCTCCCGTAG
TTCCGTACCACTCTTTCGAGTGATACGGTATAAAAAAAAACCTTACTCGCCTTCGCGAATCATTACATCTTTTGCACGAGCAATCAACTTAGGGCT
TTCCAGTAGTTCAAAAACACCAGTGGCATCGTCAAAAAGACCCAAATAGAACATATGAAAATCATCAGGATGTTTATACAACTGATTATCTTCGC
TAGCTCGATTGACTTCATCCTGAAACTGACGTACTGCAACACCTTCAGATGCAACATAAGCTGGACGACCAAAAGCATCTGCAGCAGTATCCTTA
ATAGAAACAATAACCATCTTCATAAAAACTCCTTAAATAGTACGTTTTAACAATGACAACTTAGCCAACGCAACTTTTTCCTTAACAGCAAGTCGC
TCAAGCGTGTTGTCCTCATGCCTAGATCGACCTTCCATCTCTCTGGCAAACTGAATCATATCGAATTCTTCAGGAAACTTCAACTTAAATTTATTAT
CATAAAACCGTGGTGGACGGCACTTTTTGCCACGCACCACAACGTGGTCTGACGTATAAACGTCTGACATGTACTTATCTAACCACGATTGCCCG
ATACCGGGCTTCAATGACATCTTATTAAATTCTGGCTTACGCTGAATTATCTCACCAGTCTCTAAATCACAATATTGATAATGGGCACCCGCATCA
ACCACTTCGTGGTTTTCATTGACAGTAACCCCATTAATCTTCTTCATAATATATCTTGCAACATAAGCAGCAGACTCAAAGTAACATCACCAATTG
TAGAATAGCCAAACGGCCACAATTCTTCCAAAATCTCTGACGTGTAGAGGATAGAGCCAGTCTGCGTTCTTTTAAATATTTTCTTATCCGGAAAAT
CAAGACCAAACAGACAAGCATGGAAATGAGGACGATCAAAAGATTCACCATATTCACCTGCCATATAAAAACGTATCGTTTTCCCAGTAAAACG
CTTACGTAACCGCTTCATAAAAAGCTGATAATCATTGTAATCCAATGACATATCCTTAGGACAATGCTCTGGAGCATATGTCAAAGTAATAAAACA
ATTACTAGTATGCATTTGTGCCTCATGCATACAACGAATCGCCCACTGACGTGAGCGTTCAAGGCGACAACCAACACACTGACCACAAGGCAATG
ATAGGGTACGGACTACATCCGCACCCGGTATTTCCCGCCAAATTATAGACCTGTCACTGCATTGATAAGCCGTTAAGGGCTTATAACAGGCCATA
ATTACAAACGATAGCCACCACGCTGGGGAGCGTGTCTCATATTAATTGACTTCGTCTTACTAGCAGTTCTGCGAAATGACTTTGCAGATTTATATT
TGTTTACTGGCTTTCTTCGTAACATGATGAACTCCGTAGTTAAAATAGTGGTTTGGTGTCACCTAGCACAGTTACATCAAGTAGAGTAACTGTGCT
GGCCTCAGGATTTCATCCTTCGGCCTTAGGTGTTTCTGTAGAAACGATGGGTTCAACCACAGGTTGTCCATCAATAAGACCCAATTGAATCGCTTC
ATCACGATTCTGGTCGTTCTCAAGGAACTCCAATAATTTGACAGGATCATGGTCAAATCGGACTCTTAATTTCGCTGGCAGAGCCATGAAATCGT
CCATAGTTGCGTTAATTTGATTCAACGCAGAATGGTAATCAGTAACACCACTAAAATCGCCGTATTGAGGCGATACAGGGGCCGTTGGAAGTTC
CCCTGTAACCCCGAAACGTTCAATGATGACATTAATGTCACATTCATCTTTCATATGCTGTTGAGCAAGACTTGGGTCTTGACACAGCAACGCATC
GTTTACCGATGCTTCATCTTTATCATAGTTGTACGGATTACGTACAAATGGCAAATTCGCTTTACTCATTATTTGACTCCAATTCCCCAAGGGGTTA
GTTGATTAGGATTATTTTTGAGTTTATCAAAAATTTCTTTCGAAGCATCGATACCCCTAGACCAAATAGCTGGGGATGGAGCCAATAGCCTCTTAG
TATTATATGACTGAGCGGATGTTAAATCCGCAGTCGTATTCTTTAAAGCAATATCAGCCAAAATACGTTTATTTTCGGCTGTAATATTAGGAGCAG
TAAGCAACTTATTAACAGTATCAGCCCTAGTATTAGCGGTAC

>000030F|arrow

TTTTCCGTTGACTACAACTAGTCATGGGGATATACGTCGGAACGATATTTTTTGTACCACTCAGCACCAATGCCTGGTTTAAGGCTCATATTGTTA
TATTCTGGTAATAGCTTTATTAATTCCCCTGTTTCTATATCACAGTATGTGTAATGCTCATCCTTTGTTATATGTTTGTTTCTTCCATCTGGCCGTTG
TATTTTTGCATAATATATCGAGCAACGTAAGCAGCTGACTCGAATGTAACGTCTCCAATGGAGGAATAACCATGTGGCCAGAGGGTTGCAAGGT
GGTCGGATGTATATATGAGAGAACCAGAGGGAGTCCTTTTGAATAGTTTCTTATCATGAAAATCGTATCCGAAGATACAGGCATGGAAGTGAGG
TCTGCCGAAACTTGTGCCGTATTCTCCAGCCATGTAGTAACGTAATTTTGCAGGTGCAATGGATTTTCTGAAGCGCTTAAGGAACTTTTGGAAGT
CGCTTTTGACAAGCGATCCAGTTTGTGGAAGGTTTTCATTGTTGTATGTGGGGTTATAAAGCAGTTTTTTTCGTGCAATTGGGCTTCATGCATGCA
GCGCATGGCCCATTGACGTGATTTTTCTAGCCTGCAGCCAACGCATTGGCCGCAGGGCAGTGAAATCTGACGATCATGCTCGTCAGATTCTTTGA
ATGCGACACGGCGATAAGATTTGCCGGTCTTATAGTTTGTCTGATGCTTACTTAAATAAGCAGTCAGTGGGTGATAACAGGCCATGTGAGGTAG
CCTGGCGCTTTATTAGAGCCGCCAGCCCCCTCTTTGCGGGGCTTTTTGCATATTTGCAGCTTTAGTTTTTGAAGTGTTCCTACGGAACGATTTAGC
GCTTTTGCGCTTGTTTGTTTGTTTTCTATAAAGCATAATTTTTGGCCTCGTTTATCAGATATTTTTGGTGTTGGTGTCACCTAGCACAGTTACATCAA
GTGGAGTAACTGTGCTTATCGCCATTTCTTCCGAAATGGCTCTTTCGACCAGGCCGAGTTTCTCGGCTTCTGGTCGATTTTTCTCGTCTTGCAAGA
ACTCAATCAGGTTTGCTGGTTCGTTTTCGAACCTAGCACGAATTTGAGCCGGTAAGTTATCAAATTCTTCCATAGCGTTCATTACCTTATTCAAGGC
GCTATGATAGTCAGTAATTCCGCTGAAATCGCCATATTGAGGCGGCAGCGGACTTTGAGGTAATAGGCCTGTAACGTTAAAACGTTCCAGGATA
GTATTTATATCGCATTCGTCTTTATAATGCTGCTGAGCCAGAGTTGGCTCCTCACAAGCCAACCCTGACTCATTTGACGCAGCATCCGTGTCATAG
TTGTATGCGGTTCTAAGTTTGATTTTTCATTTCATTTTTCCAAATGGTAGATATTTTTGATATTTATCATATTGGCTATGTAAATAGCCTTTAACATC
TTGATAAATTGGTTTAGTACTGGATGGCGCTGATCCAGTTTGCGCTAATCGCTCTAATTGGTCTGTATATGCACTTTGACTTAACGCTTGTCGCTG
TTGTGCAGCTTGTAATGCACTTGATGTCCTTAATTGCTCTATTTGGGCATCGCGAAGCTGACCAAATTTGCCGTATCCCGGCATCTGAGCAATTTC
ACGAGCTGTATTTGCTCGAGTATATGTTGCCTGATCACGTGATAGATTTGTATCCGCGTCCGTTTTTCGGCTTGCGATTGTGTCAGGATATTCTGA
GTTTGTAATTGTTTAAAATTGGCGACTGCCATGGCAGCTTCACGGGCTGAATTTCCAGCCTCACCTAGTGGATTTCCTACTGGAGCTTGCGCACCA
GGCTGGACTTTGGCTCCGCCTTGTGAATAAGCCAGCATAGGGTTAAGACCTGCAGCCTTAAGATCCGCTACTGCGTCTTGATATGCAGTACGTCG
CATACCATCTTGGAATTGCATCATTGCTGCAGCTTGTTGTGCGCTGGCAGCGTTTTGTTCTTGACCACCAAAATAACTAGCGGCCGCACCTATTCC
GGCTCCAATTAAGGAGCCGTAAGGTCCAAAAGCGGCGCCAGATGCGGCGCCTGAGGCAGCGGCTTCTAGTGCCATTAGAAGTGGTCGATTAAG
CCAGGTACAGAGTACATTGGCATTGGCCGTGCTTTTTTAATATCAAAAAAGCTATCAAAATGATTTGCTGGCCGTTGGCAGCTGCTCCGACCGCA
AGGGTTCGAGAGACAGGTGGATTTTCTTGAATAAACGTGTTATTCAAAGTTGGAAGTGTTGTAAACCGTTGGGCTAAATGCCAGCCGTCAATTG
TTCCAGCTGCCGTACTACGGAACAGACTGGAAATTCGGCTTGGCATATAACGATATTCTGCCCAACGTTCTTGGTATCCAAACACTCCTGTATCGT
TGGCATCGCCTCGTACGTAGATTTCTTCATTTAATACTGCTTGTTCGCCCAAAGTGGCGAAAGCAGGGAAATAGAAATCATAACGTGTGGAACGA
TTCCACATGCGGTGCATACCTTGTTGGTATGTTAAATCGGCTCGTACTGAAACGAGACCGATAATTACACCATGTTCAGTGCTTGAATAAGTAAA
GCCATGATTGTGAGCGAGCCCAGTACCCATAGCGCCAAGTGTACCGAGAGGAGTAGTGGTCCCGGAAGCTCCGGTACCATTCGTCTGGGCGAT
TGGATTGATATTAATGTGTGTTGAACCGCCTCCAATGTATTCAGGCCGTTGGAGACGCGCATCGGGGCTAACGACCCCGAAATGGCTGCGGATA
ATTTCTGTGTATCGAGTTCCGCCACGGGCGTCCCTTTCTAAAAGTTTTTGAATCTGGAAGCTTTGACGCAATTGATTAATTGTTGCAGCGGTTGCA
GCTGATAAATCTGCATATAAACCGCCATTAGGATCATATGATTTTGCCAAGCCGTCAGCACCGCCAGTAATTTGACCAGTAACACCTACGTTAATA
GCCTGGGCGGTTGCGTTTAATGTATTTGTTCCAGCGTTATACAGTCTGGAAACAGGCGCATTGTTAGTGCGTATATTGGGGCGGATGTTCCAAGC
GGTAAAGTAACGCTTGCGCCCTTTTGTGGCCAAGGTAATGCTGACGTGAAATAGTCTTTACGTTTTCCACGTCGTAATAATGTGTAGTTGGCTAC
GTTATCTGGACCATCGCCAGTATCTACAACTACTGAATTTTGAAGGTTTTCGTCCCGAAACCATTCGTTATAAATAAGGTTGTAAGCACGTGGCCA
GAAGGCACAGTGACTTACGGTGCCACCAGCACCTACTTGGCCTACGGTTGGCAGACCCATATAATCTTGAAGGCTGCCAATAGCGTAACCACCA
GCTGGGGTTACTTGTTGGGGCACTACGTACGAGATCGAGTCCGCAGGATTTGCCTGCTGCCCCATAAATTTTTGCCAATTTGACCAGATAAGTCT
ATTTGGCACAAAAAGAAGAAGCTATCCAAATGCATGTATCCATGATCGGATAGAGTGGTGTAGACAATCGCGCAAAGGCGGTCATGTTGCAGC
GGAATGTATCGCCTGGCAACATTTCGTCTACGAGTACAGGGACTAGGAAGCCAGCATCGAATGTAGTTTTATGTGTTGATTGACAGTCAAATGT
AGAGCGGGGGATATCCGCTTTTGGAATCATTGTGAACTGATGGACGTCTACCGACTTGTTACGATGCATTTTTTTGAGCTCCTAGGCCTAGTTGC
GTGAGAAAAAGGGGTTTCCCCCTTTTTACTCTACGCTTAGTTTTTATCAGTAATTTTGACTTGTTTCCCTAAGGATACAAGTTTGGGTTGTTCATGT
AATTGGAACAAACCCGTATTATCGTCGAATTCGCCTAATTCAAATAGGTCGAAATCGTCGGAATGGTTATAAAGTTGATTGTCATCGCTCTGGCG
ATTGACTTCGTCGCTAAAGCTCCGGATTGCTTCGCCGATAGAACGGACGAACATTGGACGACCGAACGCATCTGCTGCGCGGTCTTTAACGGTA
CAGAGTACTAATTTCATGAGGATTTCCTAAGTGAGGTTACGTTTTAATAATTGAAGTTTGGCCTTTGTGACTTTTTCCTTTACGGCAAGTCTGGCA
TAGGTATTGTCTTCGTGGTTGAGTTTAGCAGAAGTTTCACGTTTGTGGAGTAATTCTTCGTATTCATATGGATAATCTGATTTTATTTTTTGTCATA
GTATTTTGGGGGTTTTACCCT

>000086F|arrow

CACGTGCATCGCGCTCCAATAACTTCTGAATCTGGAAAGATTGACGAAGTTGGTTAATAGTCGCAGCAGTAGCAGTAGACAAATCAGCGTATAA
CTGATTAGTAGCAACCAGCATTTGTACTATTAGACAAAGTATTAGAAGCTGAATTTAATTCTCTTAAAGCACCAACAGCAGTTTGAAAAACACTAT
AACCAGCAGTACCATTGCCAGCTGGTATGTCAGAATAATATTAGCACGTGTTCCTAACGGTAAAGAAACAGAAGCACCCTTCTGTGGCCAAGGC
AATGCACCAGTAAAATAATCTTTACGCTTACCACGTCTCAAAAGTGTATAGTTAGCAGGAACATCACCTGAATCACCAAAGATTTAACGTTACAG

AATTCTGTAAGTTTTCATCTCTAAACCATTCGTTATAAATCTCATTATAAGCACGTAACGGCAAAACATTATGCGTTACTGTATTAGCGCCAGTAAT
CTGACCAGCAGTAGGTAAACCAAAATAATCAAAATTGAACAAACAGCATAACCACCAGCAGGACTAGTAATAGTAGGCACTACAAAAGAAATA
GAATCACCAGGATTCGTTTGTTCACCCATAAACTTTGGCCAATTGTTCCAAAGTAACGATTAGGTACAAAGAAAAAAAGTATCAAGATGCAAGTT
GTCCATCACAGGAAATAACGGTGTGGCCAAACGTGCAAATGCAGTCATCTTTACACGATAGTATCGCCAGGCAATACTTCATCACAATAAATAGG
AACTAAAAACCAGCATCAAACGTGGTTTTATGAGCATATTGAGTATTAAAACTAGAGCGGGGAATGTCCGCTTTAGGAACCATAGCAAATGAAT
GCGTACTTACTGACTTATTGCGAAACATAAACATCTCCCGTAGTTCCGTACCACTCTTTCGAGTGATACGGTATAAAAAAAACCTTACTCGCCTTC
GCGAATCATTACATCTTTTGCACGAGCAATCAACTTAGGGCTTTCCAGTAGTTCAAAAACACCAGTGGCATCGTCAAAAAGACCCAAATAGAACA
TATGAAAATCATCAGGATGTTTATACAACTGATTATCTTCGCTAGCTCGATTGACTTCATCCTGAAACTGACGTACTGCAACACCTTCAGATGCAA
CATAAGCTGGACGACCAAAAGCATCTGCAGCAGTATCCTTAATAGAAACAATAACCATCTTCATAAAAACTCCTTAAATAGTACGTTTTAACAATG
ACAACTTAGCCAACGCAACTTTTTCCTTAACAGCAAGTCGCTCAAGCGTGTTGTCCTCATGCCTAGATCGACCTTCCATCTCTCTGGCAAACTGAA
TCATATCGAATTCTTCAGGAAACTTCAACTTAAATTTATTATCATAAAACCGTGGTGGACGGCACTTTTTGCCACGCACCACAACGTGGTCTGACG
TATAAACGTCTGACATGTACTTATCTAACCACGATTGCCCGATACCGGGCTTCAATGACATCTTATTAAATTCTGGCTTACGCTGAATTATCTCACC
AGTCTCTAAATCACAATATTGATAATGGGCACCCGCATCAACCACTTCGTGGTTTTCATTGACAGTAACCCCATTAATCTTCTTCATAATATATCTT
GCAACATAAGCAGCAGACTCAAAAGTAACATCACCAATTGTAGAATAGCCAAACGGCCACAATTCTTCCAAAATCTCTGACGTGTAGAGGATAG
AGCCAGTCTGCGTTCTTTTAAATATTTTCTTATCCGGAAATCAAGACCAAACAGACAAGCATGGAAATGAGGACGATCAAAAGATTCACCATATT
CACCTGCCATATAAAAACGTATCGTTTTCCCAGTAAAACGCTTACGTAACCGCTTCATAAAAAGCTGATAATCATTGTAATCCAATGACATATCCTT
AGGACAATGCTCTGGAGCATATGTCAAAGTAATAAACAATTACTAGTATGCATTTGTGCCTCATGCATACAACGAATCGCCCACTGACGTGAGCG
TTCAAGGCGACAACCAACACACTGACCACAAGGCAATGATAGGGTACGGACTACATCCGCACCCGGTATTTCCCGCCAAATTATAGACCTGTCAC
TGCATTGATAAGCCGTTAAGGGCTTATAACAGGCCATAATTACAAACGATAGCCACCACGCTGGGGAGCGTGTCTCATATTAATTGACTTCGTCT
TACTAGCAGTTCTGCGAAATGACTTTGCAGATTTATATTTGTTTACTGGCTTTCTTCGTAACATGATGAACTCCGTAGTTAAAATAGTGGTTTGGT
GTCACCTAGCACAGTTACATCAAGTAGAGTAACTGTGCTGGCCTCAGGATTTCATCCTTCGGCCTTAGGTGTTTCTGTAGAAACGATGGGTTCAA
CCACAGGTTGTCCATCAATAAGACCCAATTGAATCGCTTCATCACGATTCTGGTCGTTCTCAAGGAACTCCAATAATTTGACAGGATCATGGTCAA
ATCGGACTCTTAATTTCGCTGGCAGAGCCATGAAATCGTCCATAGTTGCGTTAATTTGATTCAACGCAGAATGGTAATCAGTAACACCACTAAAA
TCGCCGTATTGAGGCGATACAGGGGCCGTTGGAAGTTCCCCTGTAACCCCGAAACGTTCAATGATGACATTAATGTCACATTCATCTTTCATATG
CTGTTGAGCAAGACTTGGGTCTTGACACAGCAACGCATCGTTTACCGATGCTTCATCTTTATCATAGTTGTACGGATTACGTACAAATGGCAAATT
CGCTTTACTCATTATTTGACTCCAATTCCCCAAGGGGTTAGTTGATTAGGATTATTTTTGAGTTTATCAAAAATTTCTTTCGAAGCATCGATACCCC
TAGACCAAATAGCTGGGGATGGAGCCAATAGCCTCTTAGTATTATATGACTGAGCGGATGTTAAATCCGCAGTCGTATTCTTTAAAGCAATATCA
GCCAAAATACGTTTATTTTCGGCTGTAATATTAGGAGCAGTAAGCAACTTATTAACAGTATCAGCCCTAGTATTAGCGGTACCAGCTTCAGTAGCT
TCAGTCTGGGCGATAATCTGCTTTTCTGTTTCTGATGCATTACGAATCTGTTGCATCATAGAAGCAGTATTAATAGCTGAATGTTGCATTACCTAA
AACATTTTCCATAGTAGCAGTTGAACCAGATGGGGTAGAAGCACCACCACGTTGATAGGCTAACATAGGAGATAAACCAGCAGCTTCTAAATCT
TTAACAGCACGCTGGTAACTTGTTCCACTCATATCGGCTTGAAAATCCATTTGCCTCTGAGCCATCTCCTGATTAGCTTTATTAGCAGAAGTAGAA
CCTAAATAAGAACCAACAGCGCCCAAAGCAGTACCGACACCAGGAGTAAAGAAACTAGAAGCTGAAGACAGCTTAGAACCAACATTAGTAACC
GCATCAAGTATTCCACCAAACATAAACTAACGCCCTTCGGTTGTTTCCTCACTACTCCTTACGGAGTAGTCGAGGTTATATAAAACATTAGAAATG
ATCAATTAGACCAGGTACGCTGTACATAGGCATAGGTCTGGCCATCTGACAATCAAAAAGCATCCATTAAAAATTGCTGACCATTTGCTGCAGCT
CCGACCGCCGTAGTACGAGCAACTGGAGGGTCTCTTGAATAAACGTTGAATTCAACGTAGGAAGAGAAGTAAATCGCTGTCCATAATGCCAAGC
ATCAATAGTGCCAGCAGAAGTAGACCTAAAGACCAGTAATTTGAGAAGGTTTGTAACGATACTCCGCCCAACGTTCTTGATAACCAAATACATCA
TTGTCGGCTGCAGTACCTTGAACATAAATTTCTTTGTTCAAAATAGCTTGCTCACCCAAAGTGGCAAATACTGGGAAATAAAAGTCATATCGTGTA
GACCTAGACCACATCTTAGGAAGACCCTGTTGATATGTGAGGTCAGCACGTACAGAAACTAATCCGATAATGTATCCATGTTCTTGAGCAGCATA
CGTAAAGCCGTGTCCCTGAGCCAATGCAGTACCCATTGCAGCAAGGTTACCTTGCGGAGTAGCAGAACCAGTAACCGACGTTGCAGAAGTCTGA
GCAATCGGATTAACATTAACAAGGGTCGAACCTCCACCAATATATTCAGGACGTTGTAAACGATAATCCTGTGGAGTTACTCCAAAGTGAGCACG
TAGTAACTCAGTATAACGAGTACCAC

>000111F|arrow

TAGGGGTATCGATGCTTCGAAAGAAATTTTTGATAAACTCAAAAATAATCCTAATCAACTAACCCCTTGGGGAATTGGAGTCAAATAATGAGTAA
AGCGAATTTGCCATTTGTACGTAATCCGTACAACTATGATAAAGATGAAGCATCGGTAAACGATGCGTTGCTGTGTCAAGACCCAAGTCTTGCTC
AACAGCATATGAAAGATGAATGTGACATTAATGTCATCATTGAACGTTTCGGGGTTACAGGGGAACTTCCAACGGCCCCTGTATCGCCTCAATAC
GGCGATTTTAGTGGTGTTACTGATTACCATTCTGCGTTGAATCAAATTAACGCAACTATGGACGATTTCATGGCTCTGCCAGCGAAATTAAGAGT
CCGATTTGACCATGATCCTGTCAAATTATTGGAGTTCCTTGAGAACGACCAGAATCGTGATGAAGCGATTCAATTGGGTCTTATTGATGGACAAC
CTGTGGTTGAACCCATCGTTTCTACAGAAACACCTAAGGCCGAAGGATGAAATCCTGAGGCCAGCACAGTTACTCTACTTGATGTAACTGTGCTA
GGTGACACCAAACCACTATTTTAACTACGGAGTTCATCATGTTACGAAGAAAGCCAGTAAACAAATATAAATCTGCAAAGTCATTTCGCAGAACT
GCTAGTAAGACGAAGTCAATTAATATGAGACACGCTCCCCAGCGTGGTGGCTATCGTTTGTAATTATGGCCTGTTATAAGCCCTTAACGGCTTAT
CAATGCAGTGACAGGTCTATAATTTGGCGGGAAATACCGGGTGCGGATGTAGTCCGTACCCTATCATTGCCTTGTGGTCAGTGTGTTGGTTGTC

GCCTTGAACGCTCACGTCAGTGGGCGATTCGTTGTATGCATGAGGCACAAATGCATACTAGTAATTGTTTTATTACTTTGACATATGCTCCAGAGC
ATTGTCCTAAGGATATGTCATTGGATTACAATGATTATCAGCTTTTTATGAAGCGGTTACGTAAGCGTTTTACTGGGAAAACGATACGTTTTTATA
TGGCAGGTGAATATGGTGAATCTTTTGATCGTCCTCATTTCCATGCTTGTCTGTTTGGTCTTGATTTTCCGGATAAGAAAATATTTAAAAGAACGC
AGACTGGCTCTATCCTCTACACGTCAGAGATTTTGGAAGAATTGTGGCCGTTTGGCTATTCTACAATTGGTGATGTTACTTTTGAGTCTGCTGCTT
ATGTTGCAAGATATATTATGAAGAAGATTAATGGGGTTACTGTCAATGAAAACCACGAAGTGGTTGATGCGGGTGCCCATTATCAATATTGTGA
TTTAGAGACTGGTGAGATAATTCAGCGTAAGCCAGAATTTAATAAGATGTCATTGAAGCCCGGTATCGGGCAATCGTGGTTAGATAAGTACATG
TCAGACGTTTATACGTCAGACCACGTTGTGGTGCGTGGCAAAAAGTGCCGTCCACCACGGTTTTATGATAATAAATTTAAGTTGAAGTTTCCTGA
AGAATTCGATATGATTCAGTTTGCCAGAGAGATGGAAGGTCGATCTAGGCATGAGGACAACACGCTTGAGCGACTTGCTGTTAAGGAAAAAGT
TGCGTTGGCTAAGTTGTCATTGTTAAAACGTACTATTTAAAGGAGTTTTTATGAAGATGGTTTATTGTTTCTATTAAGGATACTGCTGCAGATGCT
TTTGGTCGTCCAGCTTATGTTGCATCTGAAGGTGTTTGCAGTACGTCAGTTTCAGGATGAAGTCAATCGAGCTAGCGAAGATAATCAGTTGTATA
AACATCCTGATGATTTTCATATGTTCTATTTGGGTCTTTTGACGATGCCACTGGTGTTTTTGAACTACTGGAAAGCCTAAGTTGATTGCTCGTGCA
AAAGATTAATGATTCGCGAAGGCGAGTAAGTTTTGTTTATACCGTAACCACTCTTTCGAAGTGATACGGTATAAAAAAAACCTTACTCGCCTTCG
CGAATTCATTACATCTTTTGCACGAGCAATCAACTTAGGCTTTCCAGTAGTTCAAAAACACCAGTGGCATCGTCAAAAAGACCCAATAGAACATA
GAAATCATCAGGATGTTTATACAACTGATTATCTTCGCTAGCTCGATTGACTTCATCCTGAAACTGACGTACTGCAACACCTTCAGATGCAACATA
AGCTGGACGACCAAAAGCATCTGCAGCAGTATCCTTAATAGAAACAATAACCATCTTCATAAAAACTCCTTAAATAGTACGTTTTAACAATGACA
ACTTAGCCAACGCAACTTTTTCCTTAACAGCAAGTCGCTCAAGCGTGTTGTCCTCATGCCTAGATCGACCTTCCATCTCTCTGGCAAACTGAATCAT
ATCGAATTCTTCAGGAAACTTCAACTTAAATTTATTATCATAAAACCGTGGTGGACGGCACTTTTTGCCACGCACCACAACGTGGTCTGACGTATA
AACGTCTGACATGTACTTATCTAACCACGATTGCCCGATACCGGGCTTCAATGACATCTTATTAAATTCTGGCTTACGCTGAATTATCTCACCAGTC
TCTAAATCACAATATTGATAATGGGCACCCGCATCAACCACTTCGTGGTTTTCATTGACAGTAACCCCATTAATCTTCTTCATAATATATCTTGCAA
CATAAGCAGCAGACTCAAAAGTAACATCACCAATTGTAGAATAGCCAAACGGCCACAATTCTTCCAAAATCTCTGACGTGTAGAGGATAGAGCC
AGTCTGCGTTCTTTTAAATATTTTCTTATCCGGAAAATCAAGACCAAACAGACAAGCATGGAAATGAGGACGATCAAAAGATTCACCATATTCAC
CTGCCATATAAAAACGTATCGTTTTCCCAGTAAAACGCTTACGTAACCGCTTCATAAAAAGCTGATAATCATTGTAATCCAATGACATATCCTTAG
GACAATGCTCTGGAGCATATGTCAAAGTAATAAAACAATTACTAGTATGCATTTGTGCCTCATGCATACAACGAATCGCCCACTGACGTGAGCGT
TCAAGGCGACAACCAACACACTGACCACAAGGCAATGATAGGGTACGGACTACATCCGCACCCGGTATTTCCCGCCAAATTATAGACCTGTCACT
GCATTGATAAGCCGTTAAGGGCTTATAACAGGCCATAATTACAAACGATAGCCACCACGCTGGGGAGCGTGTCTCATATTAATTGACTTCGTCTT
ACTAGCAGTTCTGCGAAATGACTTTGCAGATTTATATTTGTTTACTGGCTTTCTTCGTAACATGATGAACTCCGTAGTTAAAATAGTGGTTTGGTG
TCACCTAGCACAGTTACATCAAGTAGAGTAACTGTGCTGGCCTCAGGATTTCATCCTTCGGCCTTAGGTGTTTCTGTAGAAACGATGGGTTCAAC
CACAGGTTGTCCATCAATAAGACCCAATTGAATCGCTTCATCACGATTCTGGTCGTTCTCAAGGAACTCCAATAATTTGACAGGATCATGGTCAAA
TCGGACTCTTAATTTCGCTGGCAGAGCCATGAAATCGTCCATAGTTGCGTTAATTTGATTCAACGCAGAATGGTAATCAGTAACACCACTAAAAT
CGCCGTATTGAGGCGATACAGGGGCCGTTGGAAGTTCCCCTGTAACCCCGAAACGTTCAATGATGACATTAATGTCACATTCATCTTTCATATGC
TGTTGAGCAAGACTTGGGTCTTGACACAGCAACGCATCGTTTACCGATGCTTCATCTTTATCATAGTTGTACGGATTACGTACAAATGGCAAATTC
GCTTTACTCATTATTTGACTCCAATTCCCCAAGGGGTTAGTTGATTAGGATTATTTTTGAGTTTATCAAAAATTTCTTTCGAAGCA

>000214F|arrow

GCGGTGCATGTTTTTCCTTAGTGTTGTTCCGGGGGAAAGATAAATCTCTTTTCCCCTCGGTGTTTTATTTAAGTTTAACTTGTTTTCCTAATGATAG
GAGTTTTGGTTGTTCATGTAAATCGAATAACCCAGTTGAATCGTCAAATGTTCCGAATTCATATAGATCGAAATCATCAGGGTGATTAAAGAGTT
GATTTTCAGTATCAGAACGATTAATTTCATCTGAAAAAGAGCGTATAGCTACTCCAGAGGAAGGTACGAACATTGGTCGTGCATATGCTTCAGCA
GCACGGTCTTTTACGGAAGCGAGGATAAGTTTCATTATTTTCCTAAGTAAGGTTACGTTTTAATAGTTGAAGTTTTGCCATAGTGACTTGTTCTTTT
GCAGATAGTCGTTCTGGTGTATTGTCTTCGGAATTAAATTTAGCATTATTTTCCCGCATGTAAAGTAATTCGTCATACTCATAAGGTTGGTCAATTT
TAAACATTTTGTCATAGTATTTTGGTGGTTTGACCTTTTTACCTCTAAGTATTACGTAGTCTTGCGGGTATATATCCGAAGTATATTTTATATAAAA
GTCTTTACCGATTCCCGGTTTTAAAGACATTTTATTATATTCCGGCTTTAAGTCTAAATATTCGCCGGTTTCAGGGTGTATGCGTTTGTAATGAGAT
TCCGCATCTTTCCCTGTTTGTTTTTTCATTATGTATCTAGCCACGTAGGCGGCTGATTCAAAAGTAACATCTCCAATGGTGGTATAACCAAATGGCC
AGAGAGCTTCAAGTTCTGCGGATCTATATAACATAGAACCAGAGGCAGTCCTTTTCCATAATTTTTATCAGGAAAGTCGTATCCGAAGATACAGG
CATGGAAGTGGGGTCTTGCGAAGAGTTCACCATATTCTCCAGCCATGTAATAGCGGATTGTAAGTCCTCTTTTTGCGAGAGTTTTTCTAAGTCTTT
TAAGGAACAATGAAAGTCTTTGTGATCCAAAGAGCCATCGCTTGGGAGATGTGTATTGTCATATGTGAGTGTTATGAATGAGTTGTTTTTATGCA
ATTGGGCTTCGTGCATGCACCGAATAGCCCATTGTCTTGATCGTTCTAGACGGCATCCAACGCACTGCCCACAGGGCAGGTCTAAAGATCGAAC
GATGTCATGTTTCCGAACTTCGTCGAAAACAATTGATTTGTCAAAGCATTGATATGCTTTGAGAGGGTGATAACAAGGCATGTGAGGTGCCTGA
AGACTTTATTAGAGTCTCCAGCCTCCACGCATTGGCGAGTTTCGCATATTTGCGTGTTTTGTATGTGAAGCATGTTTACGGAAAGTCCTAGCGGA
CTTTTGCTTATTTGCTGGTCTGCGACGCATCATTTTTTCTGTCCTTGTTTATCGTGTTTTTGTGGTTTGGTGTCACCTAGCACAGTTACATCAAGTAG
GTAACTGTGCTTCCGAGGTTCCAACAGGGGTTGAAACCTCGGTTTGGGCTGGTTTTACCAGTCCCATTTTTTCAGCTTCGCTGCGATTTTCAGGGT
TTTCTAGAAAATCTATTAGATTCGCGGGATCATTAGCGAATCGTTCACGAATATTGGCTGGTAAAGCCATAAATTCGTTGTCTGAAGCCATAATCT
GGTTCAGAGCAGAGTGGTAGTCATAGACACCACTAAAGTCGCCATATTGAGGCGTTAAAGGAGTTTGAGGAATAAGTCCGGTCATACCGAACTT

TTCCATAATATTATTGATATCACATTCTTCAGCAAATTGCTGCTGAGTCAGAGTTGCATCCTCACAATGCAGCCCTGACTCATTTGACGCAGCAATC
GTGTCGTAATTGTACGGAGTACGAAGAAATGGGGCTGTAATCTTTGTCATTTTCATTCCATTGGTGGTTGGTTATTTGTACGATTTTGTTGAATCG
GTTGACCTCGATAGTTATCGATGTATCGTTTTGCGCCAGATGCGCTATACGCATCTTTGGCGATGTTTTTTACGTCTTGATAAATTGGTTTGCTAG
AACTAGGAGCTGACCCTGATTTAGCAAGTTTTGTTAACTCGTTTGAATAACGAGATTGTGCCAAAGCTTGAGCAGCTTGAGCACTTGATGTTTTG
CCTATTTCATTTTTCATGAAAGTATCGGCAAGTATTTGTTTATATTGAGCTCTAATATTTGGATTTTCATCCAATTTATTTAACGTATCAGCACGTAC
ATTATCTGTTTGATTACTTGTGAGTTCTGTTTGAGCTTCAATTTGTTTAGATTGAGCAATAGCTTGATTTGCTTGCGCAACCGTTTGATAAGCTTGG
GTTCCGGACGTAGTTGCATTACCGAGAACATTTTGCATCTGGGCCATAGCTCCAGCTGGTGTTGTGGCTCCGCCTGTGAATACGCAAGCATGGG
ATTTAACCCAGCTTTTTTTCATATCTTCAACTGCTCGTTGATATGATGTTCCAGACATTTCAGCTTGAAATCTCTGTTGATTACTCGCTTGTTCTGCA
CTCGCTGCGTTTTGACTTTGTGTTCCAAAGTATTGTCCTGCTGCACCTATTGCTGCAGGTGCAAGTGCAGCTAAGGAGAGTCCCCCAGTAGCAGG
GGCAGCTCCTATAGCTATAGCAGGGCCGATTAAATCGGCAATTCCGTCAAATAGTCCCATTAGAAATGGTCTATTAAGCCAGGTACTGAATACAT
TGGCATTGGTCTTGCCATTTTGACATCAAAAAATGAGTCAAATAAGAATTGTTGGCCATTTGCAGCTGCTCCAACGGCTACTACACGTGATACAG
GAGGCGTATCTTGAATAAACGTATTATTCAAAGTAGGCGCAGCGGTAAATTTTTGAGCCAAATGCCAACCATCGATCGTGCCCGCCGATGTTGAT
TTGAACAAACCAGTAATTTTAGATGGTTTGTAACGATATTCCGCCCAGCGTTCTTGATATCCAAAAACAGTATTGTCGGCTGCAGTATCTCCTGTT
GCATAAATTTCTTTTTGCAAAACAGATTGTTCGCCTAGAGTAGCAAATGCTGGGAAATAAAAGTCATATCGTGTAGATCTAGACCACATACGGTC
TAATCCTTGTTGATAAGTAAGATCTGCTCTAATGGATACTAATCCAATAATAACGCCATGCTCAGTAAATGATTGAGTAAATCCATGATTATGAGC
GAGAGCAGTACCCATAGCAGCAAGGTTGCCCAAAGGGGTAGTCGTTCCAGAAGCGTTTGTTCCCGACGTTTGAGCAATCGGATTAACATTAATT
GGTGTTGAACCGCCTCCAAGGTATTCAGGCCTTTGTAAACGGGCGTCTGGGGAAATAACTCCAAAGTGACTCCGGATAATTTCTGTGTATCGTGT
TCCGCCACGTGCATCCCTTTCAAGTAATTTTTGAATTTGAAATGACTGTCTTAATTGATTGACAGTTGCAGCAGTTGCTTCTGATAAGTCGGTATAT
AAATTAGAAACGTTATTTACTACACCAGCAGTATTAACACCATAAGCGTTGCCATATCTAGCTAAAGCAGTCGTATTTCCAGGATCTGTTTGAATT
ACCGTAAATTTATCGTTTGATGTTGCGTCTCCTGAAATGGTATCCCATTTAATTGGAGCCGTAGTACCTAAAGGTAAGGTGACACTCGCACCTTTT
TGTGGCCATGGTAATGCTGACGTAAAGTAATCATGTCTTTTACCACGACGTTTTAGCACATAGTTTGAGGAAGTATCAGGGCCATCGCCCTTATC
AACTACTGCGCTTGTTTGTAAATTTTCATCTCGGAACCATTCGTTCCAGATAAGATTGTATGCACGTGGCCAAAAGGCACAGTGCGTAATAGTTC
GGCCAGTATCAATTTGGCCTACTGTTGGTAAGCCCATATAGTCTTGAAGGCTGCCTACGGCATAACCATCTGTTGGGCTTGTTTGTGTTGGGACA
ATATAAGATATTGAGTCTGTTGGATTTTCTTGTTGACCCATAAATTTTTGCCAGTTATTCCATATAAGGCGATTGGGTACAAAGAAAAAGAAAGA
ATCCATAATCATGTTATCCATGATTGGATATAAAGGCGTTGCTAGACGGGCAAATGCCGTCATTTTTAAGTTGAAAGTGTCCCCAGGGAGCACTT
CATCAACATATACAGGAATTAGATAGCCCGCATCGAAAGTCGTTTTATGTGTTTTTTGAGCATCGAATTTACTACGTGGTATATCGGCTCTAGGTA
CCATCGCGAAGCGGTGAGTATTAACTGACTGATT

>000105F|arrow

ACTTTTTGCCACGCACCACAACGTGGTCTGACGTATAAACGTCTGACATGTACTTATCTAACCACGATTGCCCGATACCGGGCTTCAATGACATCT
TATTAAATTCTGGCTTACGCTGAATTATCTCACCAGTCTCTAAATCACAATATTGATAATGGGCACCCGCATCAACCACTTCGTGGTTTTCATTGAC
AGTAACCCCATTAATCTTCTTCATAATATATCTTGCAACATAAGCAGCAGACTCAAAAGTAACATCACCAATTGTAGAATAGCCAAACGGCCACAA
TTCTTCCAAAATCTCTGACGTGTAGAGGATAGAGCCAGTCTGCGTTCTTTTAAATATTTTCTTATCCGGAAAATCAAGACCAAACAGACAAGCATG
GAAATGAGGACGATCAAAAGATTCACCATATTCACCTGCCATATAAAAACGTATCGTTTTCCCAGTAAAACGCTTACGTAACCGCTTCATAAAAA
GCTGATAATCATTGTAATCCAATGACATATCCTTAGGACAATGCTCTGGAGCATATGTCAAAGTAATAAAACAATTACTAGTATGCATTTGTGCCT
CATGCATACAACGAATCGCCCACTGACGTGAGCGTTCAAGGCGACAACCAACACACTGACCACAAGGCAATGATAGGGTACGGACTACATCCGC
ACCCGGTATTTCCCGCCAAATTATAGACCTGTCACTGCATTGATAAGCCGTTAAGGGCTTATAACAGGCCATAATTACAAACGATAGCCACCACG
CTGGGGAGCGTGTCTCATATTAATTGACTTCGTCTTACTAGCAGTTCTGCGAAATGACTTTGCAGATTTATATTTGTTTACTGGCTTTCTTCGTAAC
ATGATGAACTCCGTAGTTAAAATAGTGGTTTGGTGTCACCTAGCACAGTTACATCAAGTAGAGTAACTGTGCTGGCCTCAGGATTTCATCCTTCG
GCCTTAGGTGTTTCTGTAGAAACGATGGGTTCAACCACAGGTTGTCCATCAATAAGACCCAATTGAATCGCTTCATCACGATTCTGGTCGTTCTCA
AGGAACTCCAATAATTTGACAGGATCATGGTCAAATCGGACTCTTAATTTCGCTGGCAGAGCCATGAAATCGTCCATAGTTGCGTTAATTTGATT
CAACGCAGAATGGTAATCAGTAACACCACTAAAATCGCCGTATTGAGGCGATACAGGGGCCGTTGGAAGTTCCCCTGTAACCCCGAAACGTTCA
ATGATGACATTAATGTCACATTCATCTTTCATATGCTGTTGAGCAAGACTTGGGTCTTGACACAGCAACGCATCGTTTACCGATGCTTCATCTTTAT
CATAGTTGTACGGATTACGTACAAATGGCAAATTCGCTTTACTCATTATTTGACTCCAATTCCCCAAGGGGTTAGTTGATTAGGATTATTTTTGAG
TTTATCAAAAATTTCTTTCGAAGCATCGATACCCCTAGACCAAATAGCTGGGGATGGAGCCAATAGCCTCTTAGTATTATATGACTGAGCGGATG
TTAAATCCGCAGTCGTATTCTTTAAAGCAATATCAGCCAAAATACGTTTATTTTCGGCTGTAATATTAGGAGCAGTAAGCAACTTATTAACAGTAT
CAGCCCTAGTATTAGCGGTACCAGCTTCAGTAGCTTCAGTCTGGGCGATAATCTGCTTTTCTGTTTCTGATGCATTACGAATCTGTTGCATCATAG
AAGCAGTATTAATAGCTGAATTAGTTGCATTACCTAAAACATTTTCCATAGTAGCAGTTGAACCAGATGGGGTAGAAGCACCACCACGTTGATAG
GCTAACATAGGAGATAAACCAGCAGCTTCTAAATCTTTAACAGCACGCTGGTAACTTGTTCCACTCATATCGGCTTGAAAATCCATTTGCCTCTGA
GCCATCTCCTGATTAGCTTTATTAGCAGAAGTAGAACCTAAATAAGAACCAACAGCGCCCAAAGCAGTACCGACACCAGGAGTAAAGAAACTAG
AAGCTGAAGACAGCTTAGAACCAACATTAGTAACCGCATCAAGTATTCCACCAAACATAAACTAACGCCCTTCGGTTGTTTCCTCACTACTCCTTA
CGGAGTAGTCGAGGTTATATAAAACATTAGAAATGATCAATTAGACCAGGTACGCTGTACATAGGCATAGGTCTGGCCATCTGACAATCAAAGA

AAGCATCCATTAAAAATTGCTGACCATTTGCTGCAGCTCCGACCGCCGTAGTACGAGCAACTGGAGGGGTCTCTTGAATAAACGTTGAATTCAAC
GTAGGAAGAGAAGTAAATCGCTGTCCATAATGCCAAGCATCAATAGTGCCAGCAGAAGTAGACCTAAAGAAACCAGTAATTTGAGAAGGTTTG
TAACGATACTCCGCCCAACGTTCTTGATAACCAAATACATCATTGTCGGCTGCAGTACCTTGAACATAAATTTCTTTGTTCAAAATAGCTTGCTCAC
CCAAAGTGGCAAATACTGGGAAATAAAAGTCATATCGTGTAGACCTAGACCACATCTTAGGAAGACCCTGTTGATATGTGAGGTCAGCACGTAC
AGAAACTAATCCGATAATGTATCCATGTTCTTGAGCAGCATACGTAAAGCCGTGTCCCTGAGCCAATGCAGTACCCATTGCAGCAAGGTTACCTT
GCGGAGTAGCAGAACCAGTAACCGACGTTGCAGAAGTCTGAGCAATCGGATTAACATTAACAAGGGTCGAACCTCCACCAATATATTCAGGACG
TTGTAAACGATAATCCTGTGGAGTTACTCCAAAGTGAGCACGTAGTAACTCAGTATAACGAGTACCACCACGTGCATCGCGCTCCAATAACTTCT
GAATCTGGAAAGATTGACGAAGTTGGTTAATAGTCGCAGCAGTAGCAGTAGACAAATCAGCGTATAACTGATTAGTAGCAACACCAGCATTTGT
ACTATTAGACAAAGTATTAGAAGCTGAATTTAATTCTCTTAAAGCACCAACAGCAGTTTGAAAAACACTATAACCAGCAGTACCATTGCCAGCTG
GTATGTCAGAATAAATATTAGCACGTGTTCCTAACGGTAAAGAAACAGAAGCACCCTTCTGTGGCCAAGGCAATGCACCAGTAAAATAATCTTTA
CGCTTACCACGTCTCAAAAGTGTATAGTTAGCAGGAACATCACCTGAATCACCAAGATTTAACGTTACAGAATTCTGTAAGTTTTCATCTCTAAAC
CATTCGTTATAAATCTCATTATAAGCACGTAACGGCAAAACATTATGCGTTACTGTATTAGCGCCAGTAATCTGACCAGCAGTAGGTAAACCAAA
ATAATCAAAAATTGAACAAACAGCATAACCACCAGCAGGACTAGTAATAGTAGGCACTACAAAAGAAATAGAATCACCAGGATTCGTTTGTTCA
CCCATAAACTTTGGCCAATTGTTCCAAAGTAAACGATTAGGTACAAAGAAAAAGAAAGTATCAAGATGCAAGTTGTCCATCACAGGAAATAACG
GTGTGGCCAAACGTGCAAATGCAGTCATCTTTACACGATGAGTATCGCCAGGCAATACTTCATCACAATAAATAGGAACTAAAAAACCAGCATCA
AACGTGGTTTTATGAGCATATTGAGTATTAAAACTAGAGCGGGGAATGTCCGCTTTAGGAACCATAGCAAATGAATGCGTACTTACTGACTTATT
GCGAAACATAAACATCTCCCGTAGTTCCGTACCACTCTTTCGAGTGATACGGTATAAAAAAAACCTTACTCGCCTTCGCGAATCATTACATCTTTT
GCACGAGCAATCAACTTAGGGCTTTCCAGTAGTTCAAAAACACCAGTGGCATCGTCAAAAAGACCCAAATAGAACATATGAAAATCATCAGGAT
GTTTATACAACTGATTATCTTCGCTAGCTCGATTGACTTCATCCTGAAACTGACGTACTGCAACACCTTCAGATGCAACATAAGCTGGACGACCAA
AAGCATCTGCAGCAGTATCCTTAATAGAAACAATAACCATCTTCATAAAAACTCCTTAAATAGTACGTTTTAACAATGACAACTTAGCCAACGCAA
CTTTTTCCTTAACAGCAAGTCGCTCAAGCGTGTTGTCCTCATGCCTAGATCGACCTTCCATCTCTCTGGCAAACTGAATCATATCGAATTCTTCAGG
AAACTTCAACTTAAATTTATTATCATAAAACCGTGGTGGACGGC

>000136F|arrow

TAATTGTTTATAATAAAGGTTAAATAAGCTATTAATACTCATTGTCGTTCATATCCATATCCTCCATATCGCCGGCTAATACTGACAAACTATCTGT
GCCTGTCATTATTCCCTGATCTGTGTAACCCATATCGCCGCTTTCGTCGTCGCCTGCCAATATGCTTAAATTGTCTGTGCCGCTTACATAGTCTACT
GAATATTCATCTGCGCCAACTACTCCTGCTATTGCAGATACTACGCCAAATTGTGATGCTAATGTAACGCCGCCGTTTACAATCATACCTGTTCCG
ATACCGGCAACAAATTTATTTTTTACAAATTTTGGTAAAAATATACCTGCTGCAATCTGTCCGGCTGCAACTAATTTTGGATTTACTTTATCCTGTA
GTTTATTTTGTACCAATCTGCCTGCAACTGCTCCTGCAACCATTGCAAGAACGTTTGTCATACCGCCGCCAACGCCTGACATACTGTGTCTGCGTC
TACGTGTATGCTTTTTTTTGTGTGATCTTCTTTTTGCCATTGTATTGTTTTTTTTATTGTTTTAAAATTACTTTCCGGTTACGTCGTGATCTTTTGCAA
ATAACCCTAATAATAAGGCGCCTATACCTGCCAATATATGCAATATGTTTTTTTGTTCAACGCCTTCAATTATTGTTGGAATACCTGCAACCGATCC
AAACAATGTTGTTTTGAGATTTCTGAATATATTTTTCATAACTGATTTTTTTTTGTTTGTTCTATCAAAAAATTTACTTTTTCCTCCAGGCGCGCCAAT
CTTTCTGATAGATCGCTAAACTCTTTTACTTTGTCCTGGTGACTATCAATCTTATTTTTATACACTCCAACTAATATTGCAACTGTTACAAATAATTG
TATGTAAATAAAATACTCCTGCATTAAAACAATTTTATTTTTTTAGTATCTGAAATAAGTATTTTAATCAATGCCATTTCTTTTTTGATTTTTTGTTTT
TCTTTTGTATTGCTTGAACTAATATAATCCTGCGCTAATTTGCCATAAATATTTTGCCAAATTTTAAGCATATCAAAACTACTTTTTGTTGTACCACT
TACAACGCTAATTTTTACATTATGGCTTTTGCTGTCCTTATGATAACTTTTTGTAGGTGTTTTTTTCTTTACTTTTCCTATTGTTACTGTTGCCGGTAA
TTTCATAGGGAAATGTTTGCTGCCTGTTGTTTTACCTTTTGCACCTTTGTTTAATATATCAACAAACGCCTTTAATTTATCTGTTTTTGGCGCCGGCG
TTAATTCTTTTTTTATAACTTTTGCCGTTTGTAATGGCGTTAATTTTTTATCAAAACTATCTGAAATAATGTTCAATAAATTTTGATCATTATTTACAA
GTTGCTGCGTAAAATTTAATGATTTTTTTAAAACTTTTGCCAAACTTTTTATTGTGGCAATTTCAAATTTTGCATAATCGCCGGCGGCTCCTATTTTA
GAACGTTTTACAACTTTACCACTTGCAGCTTTTATTGCGTCCGTCCATTTAGCAAACTTTCCTGTTTTATACAGCTGCTTTGCTTTTGTCGTGATCTG
TTTTAATTTATTGCTCATTTAAACAATCTTTTGTTTGATAGTAAAAAATAACCTGCCAACGCAATTCCTCCATACAATAAATATGGATTAATGGCTC
CAACTGTTTCCTGTTCCTGTTCCGGCTGTTCCTGCTGATCTGCAGGCGCCTCCATTCCTGGTTCCTCTATTTCCTCCGATTTGCCTCCCATAAATTTA
AGTAATGCAATAATAATTGGCGTCGCTGATGTTATTAATGCAGAAACTGCAACTCCAATACTTGCACCTGGATGCTTACGCTTATATTTTGCTACA
GCTTTATTAACTGTCGTTTCCAATAATTTTGCATTGCCGCCTAATTGGCACCATTTAGCCAATAATGCAGGACGGCGTTTAGGATCATTTAACGTTT
TATATAATTTTAATGCAAACTTTTTAACATTCAAACGTACCAACGCCAAAAACGCGCGACGCGGCACGCTTAACGCGATTTTTGCCGCTGTTCTGC
CTTTACAACCTTCGCCGCCACTTCTTGCAGATCTGCGCTGCGTCCTAGCAGCTTTGCGCTGTGTCCGTGTTTTTCTTGCTCCTATGCCGCTAATACT
ATATAAACTCATATTCTTATCAATTTTATTAATATACGGTTTGTGTTCGTTCCAGGTGTCTAATACGGGATCGCACCAATACTCAATACCGTTAATT
TTAACCACAATAAACACGTGATGCGGGACGCTGCCAAATAACTTATAATTTGCAAACCTGTAACAAAAATCAATCGGCTTGCCTGATCTTTTTAAA
CTGTCCAATACTCCTGCCTGGAATAAACTGTAATGTTTACAATCATTACCGCCGTCTGCAAATTTACCTGTTGCCAATATTGCTGCAGGCGATTTAA
CACTTTGCCTGGTGTCCGGCTCAATGCTATATTTTACATTTTTCTTTAAAAAATCAAATAAATATTTGCAGGTGTTTTTTACATTTCCTTTCCAAAAA
TGATTTGCAATTTTATCGTAATCAGATTTGTATTTATTATGTGTGTCAATGATCTTTTTTATTATGTCTGTAGTCTTTTGATTTTCTACTAACATATTT
CGTTTGTTGGCAAACGGTGTTAATTTTCCCAATAAATAACGCGCGTTCATTTATACTACGTTTACTTTTTGAGAAATTGGAATAATAATGCCGCCG

CTGTTGACTGATCCCGTAAACTCAACGTTTATTTTTTTAGTTTTATTTTTAAACAAACTTATTAATGTTGTTAACGCATTAAAAATTGGCGTTTTTAT
ATTTACGGTATATATACTTTCTGATTGTCCTGGAATACTTAAACTATCTGTTTGAGATAAACTACTCAATAACTGTCCATTTAATAAAATATCGCCT
GCAATGCTGTCAATAGTTAACACGCTGCTTGTTGGATTTACAATTCTAAATACTGCCTGTACTGTTGGGAAATTTAATCCCTTTGCTTTTTTTAATG
CTACTGTTTGAAAATATACGCGCAAATTTTTTGCAGCTGATTTTTTGGATAAGATATACAGCAAAATTGCTGCTCCAACGCCTACAAATAAAAATG
ATTTTTTCATTGTTCCTGAATATACAAAAAATAATTGAAAAAATCAAAAAAGAATTGAAAAAACGCCTAAAAAACACATTTTAGCTTGCAGGCGCT
TGTGGGCGCCTGCAAGCTAATATATATTTTTGAATATTAGTACAAATGTGGATAAATTAGCTTATTTAACTTTTAATAATATATTTACGTATCTTGT
GCTAAATTCTTCATAGGGTTTATAGTTTTTCGTGTAATGGATAAAATTCGGCGGCTTATTTTTATTCGCTGCCTTTGTTTTTCATTCAGTTTTGTAAA
TTTTGTTTTTATGTTTAATTCGTCGGCAGCTTATTTTTATTCGCTGCCTTTTTTTTCAATTTTATTTTTATATTTGTACTTCCCGCGTTTGTTTGCATAT
CCGTTGGAAACAAAAACAGCCGGCATATTTTGCCGGCTGTTTGAATTTTAAAATTAAAATTTATGAATATTTTTTTATTATTAATGCAGTTGTTTTT
TATTATTATACCAGGTGTATTGTTTGCAACCTGGATCGTTCATAAATTAATTTTATTCGTTTTTGAATTTAATGAGTGGATTACAAATAAACGCGCT
TAACAAACAACTTTGTTTCTTTATCATAGTAATTAATGTAACGGATCTGTTTACTCCTGACAAACTTCTCAAACTCGTTTGTTTTGCTTTCTTTAATC
ATTCTGTATTTATACCACTTTCCGAAATTGTCGCAACACAATGCTGTTAAATAATTATTAGTCATTTTTTACAATTTTTAATATGTATAATCAATGAT
ATTATTTGTATAATTAAAAAAATAATAGAATAAGGATATTTTACAAAAACTGCTATAGGCGACGCTGAAACAATAAAAACAATATATTTATAATTT
TCCATTCTATTAATTTTGTGATGTTGGCAATATTGTTTCCTGTAACTGCAATTCCTGGCGTTCTATTGATCTGATCAACGCGCGCTGTGCGCAAATG
GCAATTAATAACGCCTCCGTAATTTCTTCGCTAAAAATAGCTATATTATTGGACGCCTGCAGCTGATATATTAACTCCAATAAATTAATATTTTCTC
TTAAAATTCCTTTTTGTGATTTTGTCATTTTTTTATGTTTTTTAGTTTTATGTAATAAAGATCTATAACCTCGCCATATGGCGAGGTTTTTTTAAAAT
AAAAAAATCGCTAATCATATTAGACAATTCTAATTTGTTAAATTGATTAAAATAATGCGGATAATAACCCGGTTTAATTAATGTAGTTAATAATTT
ATCGCCGTGATAAATACAGGCAAATTTGCAATGTTTTTTGGTTATGTCTTTAGCAACTGAATAACAATCGTCGTAACATTCAAACACGTGATCTAC
TATGTAGTTAGGCGCCTTAATTATATACTGCATTAGATTAATTTTAAAGTATTATTATTTGTTTCGTCCTCGTCAATCTCTATTGTTTTTTCGTAACCC
GTGCATGTCATTACATACTCCTGGACGGATCCGGCGGCTAATGTGTATTTTACAAAAGTAGCATGTGCGCAACTGCTACTGCAGGGGATCCTCAT
AATTGCCAACCCGCCTAAATTATTTTGATGTGGTATAGCCGGCTGAAATGGGCAAATACATTCCTTTCCGTCCTTACATAACACCTTTCCGTGTGTC
TGATCTTCTTTTACTGTGTACTTCATTTTTTATTTTTTTTGGTTAATTAAAAACTTTGTTTAAATTGCTATCAAATAAGCTATTCAACAGGCGCCTGTA
GTATCAATTTTGATCCAGGCGCTTTACTTTTTCAGTCAGGATCCTGTCTATTATTATTTTAATAATGTAATCCTGTAAATTCATTTTTTTATCTGCTGC
AAATAATTTAAGATCGTTAATTTCTTGCTCATAAAGTTTAATATTTACTGCAGCTATTTTAAATTTGTTCATTTTCATTTTTTAAAATTTATTGTTAAT
TTGTAGATCAATTATTTCCTGGTATAGCATATAATAAGCTTCGTAAAAGTGCATATATTTTTTCTTTTGGTTTGTGTCTATTTCAGTAAAATACAAAT
TGAAAAAAAAATCCCGTCTTGATTTTATGTATTCTAAATAATTCATAGTTTTAAAATTTTATTAGGTTTTCAATTAATAATGCAATAACAATGATAA
TAAATAACATTAAATAATCTTTCTGTTTTTGTGTCATTTTGATTTTATTTTAGTTGAATAAATGATTTATTATTTTCGTCGTAACATAATTCTATCGGC
TCAAACCAACCTGCAGAACGTAGCAATTTTGGTGATAGTATAAAATTACCTTCTTTTGTTTTTTCAACCTCCAACGTACTTTGCGCGTAACGATCTG
AACTGCTGCCAATGTGTCCAATGCTCATTTTATCTTTTTTGGATAAATGCAGGACGGTTATTATTAATACGTCGTGTTTCTTTGCTAATTTCCTGAT
GTATTTTATTACTTGCTTTGCCTCAATTTCATTGTTCATATTTTCTATCGCATCCAATAATCCGTCCAGGATCAAAACTGCGCAATCCGGCGTTTGTG
TTAAATACACGTGCATTAATTTTAAAATTGTCCGGCTGTTTTGTTCAACTAAACTAAACACGTCCAAATTTTTATAAATATTATCTGTTTTGGCTAAC
CTGGCAATGTTGCGCGCCTTTGTCTGAAAATCAAAACTACTTTGCTCTGTGTCAAACAAACAGATCCTGTATTTATTTTCAGCATTATAAGTTAAAA
TATTAATTCCGAATATTTCACGTTTTGAAATACCGGCGGCGATAATTGCGCTGATGAAATTTGATTTACCTGATTTGGCGGCGCCAACTAATGAAA
CAAAATTGCCTGTTGATCCAATACATTTATTTTCAATAACAAAATAAATTTTGTCATTTGGTATTGGTGTACTTGCATCAATACGGCATTGTTGTAA
AATATCGGAAAGTGAAAGTTTTTGCATATCCTTTTATATATTCTTTCCTGAATACGTCGTAAAATAATGAATAAATTATTTATCCAACAAATTTATTT
TTTACGGTAAAATAAAAAACCTATAAACAAACCTATAATAATAGGCGTTGAAATAATTAAACTACCTTTTTTTTTTACTACTTCTTTAATAACCTCT
GTTATATCTTGTTTTATTTTAGGAAATAGTTTTGTCAACTTAGTTGCGCTATCTCTGACTAATCCGCTTTTACTTGTTGCGCGCGCGTTTATAATTTT
TTTGTTTATTTCTGTTGTGTCTGTTACGCCTTTTTTAATTTCCTGGTTAAAAATGTTATTATAATAATTGCTATAAACAATACCATTCCAGGCGCTAT
AAACATAATTCAACGCTAATTTATTGTCATTAATAACTAATTGTTTTCCTTTTTCGTCTAATTTGCTCCAAACATATTTTAAAAAATATTTGTACATCA
TTTTAACGCAAAGATCTTGTAAAATTTTTCTATTATTTCCTCCAAAACTATTATAAAAAAACTTATTACGTGCGTCTAATTTATCTAATGTTTCCCAA
AATTTTTTAGCGTCGTCATTTGCATATTGATAAACTCCGCTATAAATATATTTTAAATTGTCCTGTACATTATCTGTTTTCCTGGTACTACTATACCA
CATATTCCAACCTGCGTGCCTATCCAATCCGAATAAAGTTTCGCCACTTCTTCCATAATCGGCATTAATAAACGCTTGTGCGCTTATTTTTTTTCCGT
TTGACATTGTAGATCCTTTTATATAAAAATCCGGGTGATAATAACCGCCTTCCAAATTATCTATAATGAAATTTGCTATGTATTGAAATTTATTAAA
TGGATAATCCATATTAAAAATTAAATATGTGAAATATAAAAAGTATTGTTTGTCTGTAAATACATTACGTAAATTAATCCAGGTTGATTAATATTG
CTTTGTCCTTTTATCGTTGCTCCTCCTGCTGTTGTAAATGTTGCAGATATATCAAACAAAAAAACGCCTGTTATTCTATCTTTCCAATTTGAAGGATT
TAAAACAATACTGATTGCACCTGTTACAACTCTATGAATACAATTAAAATTAACTGTTGGCGTTGAAGGCGTTGAATTAATCAAACCAATGTAACG
CGGATCCTGCAGCTGTATTGTTCCGTCGCTTTCCGGTAAATAACATTTTTGGTTATTATATGCAGGATTTGGATATAATAGCTGTGTACTTGACGC
TAATTGAAAATTTATATATGTAGCTCTAATTTCTAAATTTGTACTTATTGCAGGTATTTGAAAAAAAAGGTTTAACGGTGTTATTGCTATACCTTCA
TTAGTTGTTACATCGTACGTAGAAAATGCAGATGATCCAGGATTGTTAATTATTGTTTGAACGTCACTACCTATATCATTTAATATTACAGAAATAT
CATTTGAAATATTTCCCGCTGTTAATACTTGTTGCAGATTTGGTATTGATCCGGCACCGCCTAATGCAAACCAACCTCCCGCCTGTGATCTGTATAT
AATTAATCCGACCGTATCAATAAAAATCGTACCTGTTGCAACGTCTGTTGCTGCGGGTATATTGGCGTAATCGTCTGCAATTATTCCAGGCGTCGC
GCTAATATTGGAAACGCCGTTATTTACTGTTATACTCATAATTTTATTTATTATTTGCAACTGTGATCCAGGCGCCGCCGGTACTAACTGTAAATAT

TTGCGCTGTATCTGTGCTTATATAAATTGTTCCTGCAGCTAAATTGGCTGCAGCCGGACGCGACGCAAAAACGCCTGTTATTATTCCAGGCGTTCC
ACTTATATTTTGAACTGAATTGCTAATTGTTATTGCCATAATTTAAACATATACTTTGCGAAATACTATTAATTTGTTGGCTCCTGCGCTACTAAAA
AAATATGTGTAAACTGTTCTGTCAATTTCTCCGGCATTTGCTGACAATGTTATACTGCTGCCTGGATTAATAACCAACGCGCTGTTTAATGTTACGT
TTGATGTGCCGTTATTAAAAAATGTTATATCCGCGCAATCGCTGCGAACTGATCCAACCGGATCAACTAAATTTAAAACGTCAATTTTATAATTTT
GCATCAATATATTTTTTTTGATTAATACGCAACGCGGCGCCGGAAACCCAACAACCTGCTCAAACAATATTATTTTTAAAACGCCGGTTAAATACT
CATTTTCATTTCCTTTAATTATATGCTGATCGTTTGGCTGTATTGTATTACTTGTATTTTCCCAACTTATTAAATTACTCGCTGTATTGTTACAAATAA
TAATTGTATTATAACCTTTAATATTTACGTTTGTGTCCTCTTGCGCTGCACCTGGTACAACTTTTATAATATTTTGAAACTCGTTTACGTATTCTTTCA
TAAAGGTTAAATAAGCTGATTAATCCTGCGGAAACGGTTTAAAACTGCTTTGCGTCGGCTCAATAATTATCTGTGCTTTTTTCTTTCCTTTACGCTT
TAATTTTCTTGCTGTTTTTTTATCTAAACGTTTCTTTTTTCTTTCTGCAGGTGTTTTTTTACCTTTTGTAAATTCTATATTTTTTTGTCCTGCAGGTGTTT
TTATTAAAACTTTTATATCCTTATATCCCTGTGCTATTTTTTTTGCTGTAGATATTGCCTGATCAACGCTGCCGCCTGTAGTTTTTACGTCAATAACT
GCATCCGGTGTTTTAATTTCTTTATCTGTTAACTCCTGTCCTTCATCTGATCCAGGCGCCGCCGATCCAAATAATTTGTCTCTGAAATAATACGCGG
CAATCGCTGCAGCACCTATTAAGTATATTGGTTTAATCTTTGCCATTATTTTTTTAATATTTTTGGTAATAAATAACCGGCTGCATATATTCCGACG
CCGATATATAATAAAGTTTTTAACGTTTTTCCGCCAGCTGCAAACATATCTGTTAACTTGTCAACCGGTGTTTTATTTTCCTCTGTTTTTTCCGCTTG
TATTTCTACGTCACTTTTGGCGCCTTGCTCCTGTAATTTTGATAAACTAAAACGTTTGTCGTTTTTAAAAATTACGGCATAATATTTTTTGTCGCGTC
CTAAAAAAGTAAAGTATGTATTTGTCCTGGTGGCGTATTTAATACCGTATTGGTTTGTATATTCCTGTACCGGTAATAAAAAACTATCTAAAACAA
AACTGTATCCTGCAGGTAGTTTATTACTTGTTATTGCTTTTGCTTTATCGCCAAATTTGTTGATGTCCTCAACTCTGTAAAATTCTAAAGGACGCGT
TACATAAAACGTTTTGTTTAACAACTGTCCTGGTGAAAATATCGGCATAATATTATAACATTGAAATTAACATAGTGATCCTATCTGCCGGCATTG
CTGCCAACTTTTGCAGGTGTTCCAATTTAACACCCTTTGCAAATAATATATTTATTGTTTCCTGCAATGTCTGATCCGGATTAATACCGGCTAAATT
GCTAACTGTCGGCTGTACCGGCGGCAATATTCTATTAATTAACTGTCCTATTATTTCAGGCGTAAAAAATTTACCTATGCCGTTCATTCCGTCCGGC
TCCTGTTCGTCGTCCTCCTCTATTCTTTCCGCCTGCAGCGCTGATATTTCAGATCGTAACTTATTTACTTCGCTGTGCATTGCTGCAATGGTGTAAG
CGTTTGGATCTGCAGGCAATCCGGCAACTCCAGGTAACTCCAAACATTGAAACGCAATAACGCAATAATGTTCTGATTTGCGCGTATAATTTTTTT
CTTTTTTGTTATGCAATATTAATTCGTGAACGTGTGTAATATTGCGCTTTATAGATCTTTCAATTTCTTCCGTCAATTTTTCTGCAACGTCCTCCAGC
TGATCGCCTTCCGTATAATCGCAAATTAATTTTGTTCCAATCCATAAACTGAAATAAGGCGCCTTTGCTTTCTTTTGAAACGCATCCAGCACCTCAT
CCAATCCAATATATCTTGCTTGATAACTCATTTTTTTACTTAAAGTAAATGTTAAATAAAATTGATATTGGCGCTGATGTAGCAACCAATGCAACCG
GGAAAGTTACATAACATTTTTCCCAATAAATTACCTGTCCGTTTAATTCATACATTTGTCTAACAAAAGGATCATTGGCGGCATTTTGCACCCTGTG
CATTAATGTAAATGGTACCTGTTGGATCCATTCGCCAACGTTTGCAGGATTACCAACGTCATTTAAGTATAACGTTAAATAAGCTGTTTTCATTTG
CGCATTTGTTAACGGCGTCAAATTTGTTGGTGACAATGTCATATCGCCAACGTTTAATACTTCAATGCCGTATATCTGTTTTTGACGCAAATACGG
CTGATCATTTAGCTGCACCTTTGTTAAACTTGCACCCTGTGGGTAAACTTGTTCAACTAACTGTGATTTAAAAACTTTAGGCATTTTTTAAAATTTT
AAAATGATCCTGGACGTGTTTGCCAGGATCATTAATAAATAAAATTGATTACGAAACGGATGTGACATTTTGCGCCAAAATTCCCGAAAGTAAAC
AGGTAACAACTGTTGTGTTACTTGCTTGCAATGTTCCAACGGCTGCAGGTAGGTTAATGGTAAAGTTATTATTTTTACTTCCGATCAAAACCCAAT
TTGGCTCACACGCAAATGCTGTTGCATTAATACCGTCAAACGCATCTGAAATGCTGCCCGGTGTAGCCGTTGCAATAGCCAAAATAGATTGTTGG
TAAGTTGGTACGCATTTATGGCGTTCCATTGGTAATGCAGGCGAAATTACGCGGTTATTTACAGTCAAGTTATACGTTGACTGATAAAAAATTATTC
AATGCTGCAGATGCTCCTGCTGTTGAAAATCTGTAAGGATTGTTCCAGGTAATTGGATCGTAATCCGTTGCAGTTGCACTGCTCGCCAATCCTAA
AAAGAACTGTATTGATCCACAATAGAACGCATCCTGCAAATTTAATCGCTGCTCTGTGGCTCTTACAACTCCGTTGCTGTTTGTGCTGCTGTCATTT
ACTAATACTCCCAAACGGTAAGATGTAACGTTTGTTGATAATGTACACTCCAGGCGTAAAAAACTCTGTGATAAAATTGCAGTTGACGGATCTAC
TCCGGCTCTCTGCATTGCTCTCAATGCGTTACGATAAACTAAACGCGCGTTAACTTGACTCATTTTTAGTCTGTTT

>000145F|arrow

CCGCTATTACATGGCTGGAGAATATGGTGAACTCTTCGCAAGACCCCACTTCCATGCCTGTATCTCGACTTACTTCCTGATAAAAAATTATGGAAA
AGGACTGCCCTGGTTCTATGTTATATAGATCCGCAGAACTTGAAGCTCTCTGGCCATTTGGTTATACCACCATTGGAGATGTTACTTTCGAATCAG
CCGCCTACGTGGCTAGATACATAATGAAAAAACAAACAGGAAAGATGCGGAATCTCATTACAAACGCATACACCCTGAAACCGGCGAATATTTA
GACTTAAAGCCGGAATATAATAAATGTCTTTAAAACCGGAATCGGTAAAGACTTTTATATAAAATATACTTCGGATATATACCCGCAAGACTACG
TAATACTTAGAGGTAAAAGGTCAAACCACCAAAATACTATGACAAATGTTTAAAATTGACCAACCTTATGAGTATGACGAATTACTTTACATGCG
GGAAAATAACGCTAAACTTAATTCCGAAGACATACACCGAACGACTATCTGCAAAAGAACAAGTAACTATGGCAAAACTTCAACTATTAAAACGT
AACCTCACTTAGGAAAATAATGAAACTTATCCTCGCTTCCGTAAAAGACCGTGCTGCTGAAGCATATGCACGACCAATGTTCGTACCTTCCTCTGG
ATAGCTATACGCTCTTTTTCAGATGAAATTAATCGTTCTGATACTGAAATCAACTTTTTTATTAATCACCCTGATGACTTCGATCTATATGAATTCG
GAACTTTTGACGATTCAACTGGGTTATTCGATTTACATGAACAACCAAAACTCTATCATTAGGAAAACAAGTTAAACTTAAATAAAACAACCGAG
GGGAAAGGATTTATCTTTCCCCCGGAACAACACTAAGGAAAAACATGCACCGCAATCAGTCAGTTAATACTCACCGCTTCGCGATGGTACCTAGA
GCCGATATACCACGTAGTAAATTCGATGCTCAAAAACACATAAAACGACTTTCGATGCGCTCCCTGGGACACTTTCACTTAAAAATGACGGCATT
TGCCCGTCTAGCAACGCCTTTATATCCAATCATGGACAACATGATTATGGATTCTTTCTTTTTCTTTGTACCCAATCGCCTTATATGGAATAACTGG
CAAAAATTTATGGGTCACAAGAAAATCCAACAGACTCAATATCTTATATTGTCCCAACTCAAACAAGCCCAACAGATGGTTATGCCGTAGGCAGC
CTTCAAGACTATATGGGCTTACCAACAGTAGGCCAAATTGATACTGGCCGAACTATTACGCACTGTGCCTTTTGGCCACGTGCATACAATCTTATT

TGGAACGAATGGTTCCGAGATGAAAATTTACAAACAAGCGCAGTAGTTGATAAGGGCGATGGCCCTGATACTTCCTCAAACTATGTGCTAAAAC
GTCGTGGTAAAAGACATGATTACTTTACGTCAGCATTACCATGGCCACAAAAAGGTGCGAGTGTCACCTTACCTTTAGGTACTACGGCTCCAATT
AAATGGGATACCATTTCAGGAGACGCAACATCAAACGATAAATTTACGGTAATTCAAACAGATCCTGGAAATACGACTGCTTTAGCTAGATATG
GCAACGCTTATGGTGTTAATACTGCTGGTGTAGTAAATAACGTTTCTAATTTATATACCGACTTATCAGAAGCAACTGCTGCAACTGTCAATCAAT
TAAGACAGTCATTTCAAATTCAAAAATTACTTGAAAGGGATGCACGTGGCGGAACACGATACACAGAAATTATCCGGAGTCACTTTGGAGTTATT
TCCCCAGACGCCCGTTTACAAAGGCCTGAATACCTTGGAGGCGGTTCAACACCAATTAATGTTAATCCGATTGCTCAAACGTCGGGAACAAACGC
TTCTGGAACGACTACCCCTTTGGGCAACCTTGCTGCTATGGGTACTGCTCTCGCTCATAATCATGGATTTACTCAATCATTTACTGAGCATGGCGT
TATTATTGGATTAGTATCCATTAGAGCAGATCTTACTTATCAACAAGGATTAGACCGTATGTGGTCTAGATCTACACGATATGACTTTTATTTCCCA
GCATTTGCTACTCTAGGCGAACAATCTGTTTTGCAAAAAGAAATTTATGCAACAGGAGATACTGCAGCCGACAATACTGTTTTTGGATATCAAGA
ACGCTGGGCGGAATATCGTTACAAACCATCTAAAATTACTGGTTTGTTCAAATCAACATCGGCGGGCACGATCGATGGTTGGCATTTGGCTCAAA
AATTTACCGCTGCGCCTACTTTGAATAATACGTTTATTCAAGATACGCCTCCTGTATCACGTGTAGTAGCCGTTGGAGCAGCTGCAAATGGCCAAC
AATTCTTATTTGACTCATTTTTTGATGTCAAAATGGCAAGACCAATGCCAATGTATTCAGTACCTGGCTTAATAGACCATTTCTAATGGGACTATTT
GACGGAATTGCCGATTTAATCGGCCCTGCTATAGCTATAGGAGCTGCCCCTGCTACTGGGGGACTCTCCTTAGCTGCACTTGCACCTGCAGCAAT
AGGTGCAGCAGGACAATACTTTGGAACACAAAGTCAAAACGCAGCGAGTGCAGAACAAGCGAGTAATCAACAGAGATTTCAAGCTGAAATGTC
TGGAACATCATATCAACGAGCAGTTGAAGATATGAAAAAAGCTGGGTTAAATCCCATGCTTGCGTATTCACAAGGCGGAGCCACAACACCAGCT
GGAGCTATGGCCCAGATGCAAATGTTCTCGGTAATGCAACTACGTCCGGAACCCAAGCTTATCAAACGGTTGCTCAAGCAAATCAAGCTATTGCT
CAATCTAAACAAATTGAAGCTCAAACAGAACTCACAAGTAATCAAACAGATAATGTACGTGCTGATACGTTAAACAAATTGGATGAAAATCCAAA
TATTAGAGCTCCATATAAACAATACTTGCCGATACTTTCATGAAAAATGAAATAGGCAAAACATCAAGTGCTCAAGCTGCTCAAGCTTTGGCAAC
AATCTCGTTATTCAAACGAGTTACACAAACTTGCTAAATCAGGGTCAGCTCCTAGTTCTAGCAACCAATTTATCAAGACGTAAAAACATATCGCCA
AAGATGCGTATAGCGCATCTGGCGCAAAACGATACATCGATAACTATCGAGGTCACCGATTCAACAAAATCGTACAAAATAACCAACCACCAAT
GGAATGAAAATGACAAAGATTACAGCCCCATTTCTTCGTACTCCGTACAATTACGACACGATTGCTGCGTCAAATGAGTCAGGGCTGCATTGTGA
GGATGCAACTCTGACTCAGCAGCAATTTGCTGAAGAAGCGATATTAATAATATTATGGAAAAGTTTGGTATGACCGGACTTATTCCTCAAACTCC
TTTATCGCCTCAATATGGCGACTTTAGTGGTGTCTATGACTACCACTCTGCTCTGAACCAGATTATGGCTTCAGACAACGAATTTTATGGCTTTACC
AGCCAATATTCGTGAACGATTCGCTAATGATCCCGCGAATCTAATAGATTTTCTAGAAATCCTGAAAATCGCAGCGAAGCTGGAAAAAATGGGA
CTGGTAAAACCAGCCCAAACCGAGGTTTCAAACCCCTGTTGGAACCTCGGAAGCACAGTTACCTACTTGATGTAACTGTGCTAGGTTGACACCAA
ACCACAAAAACACGATAAACAAGGACAGAAAAATGATGCGTCGCAGACCAGCAATAAGCAAAAGTCCGCTAGGACTTTCCGTAAACATGCTTCA
CATACAAAACACGCAAATATGCGAAACTCGCCAATGCGTGGCGGCTGGAGACCTCTAATAAAGTCCCAGGCACCTCACATGCCTTGTTATCACCC
TCTCAAAGCATTTCAATGCTTTGACAATCAATTGTTTTCGACGAAGTTCCGGAAACATGACATCGTTCGATCTTTAGGACCTGCCCTGTGGGCAGT
GCGTTGGATGCCCGTCTAGAAACGCTCAAGACAATGGGCTATTCGGTGCATGCACGAAGCCCAATTAGCATAAAAACAACTCATTCATAACACTC
CATATGACAATACACATCTCCCAGCGATGGCTCTTTGGATCACAAAGACTTTCAACTGTTCCTTAAAAGACTTAGAAAAAACTCTCGCAAAAAGA
GGACTTACAAT

>000025F|arrow

TTTGACATCAAAAAATGAGTCAAATAAGAATTGTTGGCCATTTGCAGCTGCTCCAACGGCTACTACACGTGATACAGGAGGCGTATCTTGAATAA
AGTATTATTCAAAGTAGGCGCAGCGGTAAATTTTTGAGCCAAATGCCAACCATCGATCGTGCCCGCCGATGTTGATTTGAACAAACCAGTAATTT
TAGATGGTTTGTAACGATATTCCGCCCAGCGTTCTTGATATCCAAAAACAGTATTGTCGGCTGCAGTATCTCCTGTTGCATAAATTTCTTTTTGCAA
AACAGATTGTTCGCCTAGAGTAGCAAATGCTGGGAAATAAAAGTCATATCGTGTAGATCTAGACCACATACGGTCTAATCCTTGTTGATAAGTAA
GATCTGCTCTAATGGATACTAATCCAATAATAACGCCATGCTCAGTAAATGATTGAGTAAATCCATGATTATGAGCGAGAGCAGTACCCATAGCA
GCAAGGTTGCCCAAAGGGGTAGTCGTTCCAGAAGCGTTTGTTCCCGACGTTTGAGCAATCGGATTAACATTAATTGGTGTTGAACCGCCTCCAA
GGTATTCAGGCCTTTGTAAACGGGCGTCTGGGGAAATAACTCCAAAGTGACTCCGGATAATTTCTGTGTATCGTGTTCCGCCACGTGCATCCCTT
TCAAGTAATTTTTGAATTTGAAATGACTGTCTTAATTGATTGACAGTTGCAGCAGTTGCTTCTGATAAGTCGGTATATAATTAGAAACGTTATTTA
CTACACCAGCAGTATTACACCATAACGCGTTGCCATATCTAGCTAAAGCAGTCGTATTTCCAGGATCTGTTTGAATTACCGTAAATTTATCGTTTG
ATGTTGCGTCTCCTGAAATGGTATCCCATTTAATTGGAGCCGTAGTACCTAAAGGTAAGGTGACACTCGCACCTTTTTGTGGCCATGGTAATGCT
GACGTAAAGTAATCATGTCTTTTACCACGACGTTTTAGCACATAGTTTGAGGAAGTATCAGGGCCATCGCCCTTATCAACTACTGCGCTTGTTTGT
AAATTTTCATCTCGGAACCATTCGTTCCATAAGATTGTATGCACGTGGCCAAAAGGCACAGTGCGTAATAGTTCGGCCAGTATCAATTTGGCCTA
CTGTTGGTAAGCCCATATAGTCTTGAAGGCTGCCTACGGCATAACCATCTGTTGGGCTTGTTTGTGTTGGGACAATATAAGATATTGAGTCTGTT
GGATTTTCTTGTTGACCCATAAATTTTTGCCAGTTATTCCATATAAGGCGATTGGGTACAAAGAAAAAGAAAGAATCCATAATCATGTTTCCATGA
TTGGATATAAAGGCGTTGCTAGACGGGCAAATGCCGTCATTTTTAAGTTGAAAGTGTCCCCAGGGAGCACTTCATCAACATATACAGGAATTAA
AGCCCGCATCGAAAGTCGTTTTATGTGTTTTTTGAGCATCGAATTTACTACGTGGTATATCGGCTCTAGGTACCATCGCGAAGCGGTGAGTATTA
ACTGACTGATTGCGGTGCATGTTTTTCCTTAGTGTTGTTCCGGGGGAAAGATAAATCTCTTTTCCCCTCGGTTGTTTTATTTAAGTTTAACTTGTTT
TCCTAATGATAGGAGTTTTGGTTGTTCATGTAAATCGAATAACCCAGTTGAATCGTCAAATGTTCCGAATTCATATAGATCGAAGTCATCAGGGT
GATTAAAAGTTGATTTTCAGTATCAGAACGATTAATTTCATCTGAAAAAGAGCGTATAGCTACTCCAGAGGAAGGTACGAACATTGTCGTGCATA

TGCTTCAGCAGCACGGTCTTTTACGGAAGCGAGGATAAGTTTCATTATTTTCCTAAGTAGGTTACGTTTTAATAGTTGAAGTTTTGCCATAGTGAC
TTGTTCTTTTGCAGATAGTCGTTCTGGTGTATTGTCTTCGGAATTAAGTTTAGCATTATTTTCCCGCATGTAAAGTAATTCGTCATACTCATAAGGT
TGGTCAATTTTAAACATTTTGTCATAGTATTTTGGTGGTTTGACCTTTTTACCTCTAAGTATTACGTATCTTGCGGGTATATATCCGAAGTATATTTT
ATATAAAAGTCTTTACCGATTCCCGGTTTTAAAGACATTTTATTATATTCCGGCTTTAAGTCTAAATATTCGCCGGTTTCAGGGTGTATGCGTTTGT
AATGAGATTCCGCATCTTTCCCTGTTTGTTTTTTCATTATGTATCTAGCCACGTAGGCGGCTGATTCGAAAGTAACATCTCCAATGGTGGTATAAC
CAAATGGCCAGAGAGCTTCAAGTTCTGCGGATCTATATAACATAGAACCAGAGGCAGTCCTTTTCCATAATTTTTTATCAGGAAAGTCGTATCCG
AAGATACAGGCATGGAAGTGGGGTCTTGCGAAGAGTTCACCATATTCTCCAGCCATGTAATAGCGGATTGTAAGTCCTCTTTTTGCGAGAGTTTT
TCTAAGTCTTTTAAGGAACAGTTGAAAGTCTTTGTGATCCAAAGAGCCATCGCTTGGGAGATGTGTATTGTCATATGTGAGTGTTATGAATGAGT
TGTTTTTATGCAATTGGGCTTCGTGCATGCACCGAATAGCCCATTGTCTTGATCGTTCTAGACGGCATCCAACGCACTGCCCACAGGGCAGGTCT
AAAGATCGAACGATGTCATGTTTCCGAACTTCGTCGAAAACAATTGATTTGTCAAAGCATTGAATGCTTTGAGAGGGTGATAACAAGGCATGTG
AGGTGCCTGGGACTTTATTAGAGTCTCCAGCCTCCACGCATTGGCGAGTTTCGCATATTTGCGTGTTTTGTATGTGAAGCATGTTTACGGAAAGT
CCTAGCGGACTTTTGCTTATTTGCTGGTCTGCGACGCATCATTTTTTCTGTCCTTGTTTATCGTGTTTTTGTGGTTTGGTGTCACCTAGCACAGTTAC
ATCAAGTAGGTAACTGTGCTTCCGAGGTTCCAACAGGGGTTGAAACCTCGGTTTGGGCTGGTTTTACCAGTCCCATTTTTTCAGCTTCGCTGCGAT
TTTCAGGGTTTTCTAGAAAATCTATTAGATTCGCGGGATCATTAGCGAATCGTTCACGAATATTGGCTGGTAAAGCCATAAATTCGTTGTCTGAA
GCCATAATCTGGTTCAGAGCAGAGTGGTAGTCATAGACACCACTAAAGTCGCCATATTGAGGCGTTAAAGGAGTTTGAGGAATAAGTCCGGTCA
TACCGAACTTTTCCATAATATTATTAATATCGCATTCTTCAGCAAATTGCTGCTGAGTCAGAGTTGCATCCTCACAATGCAGCCCTGACTCATTTGA
CGCAGCAATCGTGTCGTAATTGTACGGAGTACGAAGAAATGGGGCTGTAATCTTTGTCATTTTCATTCCATTGTGGTTGGTTATTTGTACGATTTG
TTGAATCGGTTGACCTCGATAGTTATCGATGTATCGTTTTTGCGCCAGATGCGCTATACGCATCTTTGGCGATGATTTTTACGTCTTGATAAATTG
GTTTGCTAGAACTAGGAGCTGACCCTGATTTAGCAAGTTTTGTTAACTCGTTTGAATAACGAGATTGTGCCAAAGCTTGAGCAGCTTGAGCACTT
GATGTTTTGCCTATTTCATTTTTCATGAAAGTATCGGCAAGTATTTGTTTATATTGAGCTCTAATATTTGGATTTTCATCCAATTTGTTTAACGTATC
AGCACGTACATTATCTGTTTGATTACTTGTGAGTTCTGTTTGAGCTTCAATTTGGTTTAGATTGAGCAATAGCTTGATTTGCTTGAGCAAACCGTTT
GATAAGCTTGGGTTCCGGACGTAGTTGCATTACCGAGAACATTTTGCATCTGGGCCATAGCTCCAGCTGGTGTTGTGGCTCCGCCTTGTGATACG
CAAGCATGGGATTTAACCCAGCTTTTTCATATCTTCAACTGCTCGTTGATATGATGTTCCAGACATTTCAGCTTGAAATCTCTGTTGATTACTCGC
TTGTTCTGCCACTCGCTGCGTTTTGACTTTGTGTTCCAAAGTAATTGTCCTGCTGCACCTATTGCTGCAGGTGCAAGTGCAGCTAAGGAGAGTCCC
CCAGTAGCAGGGCAGCTCCTATAGCTATAGCAGGGCCGATTAAATCGGCAATTCCGTCAAATAGTCCCATTAGAAATGGTCTATTAAGCCAGGT
ACTGAATACATTGGCATTGGTCTTGCCAT

>000073F|arrow

TAGGCCAAATTGATACTGGCCGAACTATTACGCACTGTGCCTTTTGGCCACGTGCATACAATCTTATTTGGAACGAATGGTTCCGAGATGAAAAT
TTACAAACAAGCGCAGTAGTTGATAAGGGCGATGGTCCCTGATACTTCCTCAAACTATGTGCTAAACGTCGTGGTAAAAGACATGATTACTTTAC
GTCAGCATTACCATGGCCACAAAAAGGTGCGAGTGTCACCTTACCTTTAGGTACTACGGCTCCAATTAAATGGATACCATTTCAGGAGACGCAAC
ATCAACGATAAATTTACGGTAATTCAAACAGATCCTGGAAATACGACTGCTTTAGCTAGATATGGCAACGCTTATGGTGTTAATACTGCTGGTGT
AGTAAATAACGTTTCTAATTTATATACCGACTTATCAGAAGCAACGGCTGCAACTGTCAATCAATTAAGACAGTCATTTCAAATTCAAAAAATTAC
TTGAAAGGGATCACGTGGCGGAACACGATACACAGAAATTATCCGAGTCACTTTGGAGTTATTTCCCCAGACGCCCGTTACAAAAGGCCTGAAT
ACCTTGGAGGCGGTTCAACACCATTAATGTTAATCCGATTGCTCAAACGTCGGGAACAAACGCTTCTGGAACGACTACCCTTTGGGCAACCGTGC
TGCTATGGGTACTGCTCTCTGCTCATAATCATGGATTTACTCAATCATTTACTGAGCATGGCTTATTATTGGATTAGTATCCATTAGAGCAGATCTT
ACTTATCAACAAGGATTAGACCGTATGGGTCTAGACTACACGATATGACTTTATTTCCAGCAATTTCGCTACTCTAGGCGAACAATCTGTTTTGCA
AAAAGAAATTTATGCAACAGGAGATACTGCAGCCGACAATACTGTTTTTGGATATCAAGAACGCTGGGCGGAATATCGTTACAAACCATCTAAA
ATTACTGGTTTGTTCAAATCAACATCGGCGGGCACGATCGATGGTTGGCATTTGGCTCAAAAATTTACCGCTGCGCCTACTTTGAATAATACGTTT
ATTCAAGATACGCCTCCTGTATCACGTGTAGTAGCCGTTGGAGCAGCTGCAAATGGCCAACAATTCTTATTTGACTCATTTTTTGATGTCAAAATG
GCAAGACCAATGCCAATGTATTCAGTACCTGGCTTAATAGACCATTTCTAATGGGACTATTTGACGGAATTGCCGATTTAATCGGCCCTGCTATA
GCTATAGGAGCTGCCCCTGCTACTGGGGGACTCTCCTTAGCTGCACTTGCACCTGCAGCAATAGGTGCAGCAGGACAATACTTTGGAACACAAA
GTCAAAACGCAGCGAGTGCAGAACAAGCGAGTAATCAACAGAGATTTCAAGCTGAAATGTCTGGAACATCATATCAACGAGCAGTTGAAGATA
TGAAAAAAGCTGGGTTAAATCCCATGCTTGCGTATTCACAAGGCGGAGCCACAACACCAGCTGGAGCTATGGCCCAGATGCAAAATGTTCTCGG
TAATGCAACTACGTCCGGAACCCAAGCTTATCAAACGGTTGCTCAAGCAAATCAAGCTATTGCTCAATCTAAACAAATTGAAGCTCAAACAGAAC
TCACAAGTAATCAAACAGATAATGTACGTGCTGATACGTTAAACAAATTGGATGAAAATCCAAATATTAGAGCTCAATATAAACAAATACTTGCC
GATACTTTCATGAAAAATGAAATAGGCAAAACATCAAGTGCTCAAGCTGCTCAAGCTTTGGCACAATCTCGTTATTCAAACGAGTTAACAAAACT
TGCTAAATCAGGGTCAGCTCCTAGTTCTAGCAAACCAATTTATCAAGACGTAAAAAACATCGCCAAAGATGCGTATAGCGCATCTGGCGCAAAAC
GATACATCGATAACTATCGAGGTCAACCGATTCAACAAAATCGTACAAATAACCAACCACCAATGGAATGAAAATGACAAAGATTACAGCCCCAT
TTCTTCGTACTCCGTACATTACGACACGATTGCTGCGTCAAATGAGTCAGGGCTGCATTGTGAGGATGCAACTCTGACTCAGCAGCAATTTGCCT
GAAGAATGCGATATTAATATATTATGGAAAAGTTGGTATGACCGGACTTATTCCTCAAACTCCTTTACGCTCAATATGGCGACTTTAGTGTGTCTA
TGGACTACCACTCTGCTCTGAACCCAGATTATGGCTTCAGACAACGAATTTATGGCTTTACCAGCCAATATTCGTGAACGATTCGCTAATGATCCC

GCGAATCTAATAGATTTTCTAGAAAATCCTGAAAATCGCAGCGAAGCTGAAAAATGGGACTGGTAAAACCAGCCCAAACCGAGGTTTCAACCCC
TGTTGGAACCTCGGAAGCACAGTTACCTACTTGATGTAACTGTGCTAGGTGACCACCAAACCACAAAAACACGATAAACAAGGACAGAAAAATG
ATGCGTCGCAGACCAGCAAATAAGCAAAAGTCCGCTAGGACTTTCCGTAAACATGCTTCACATACAAAACACGCAAATATGCGAAACTCGCCAA
TGCGTGGAGGCTGGAGACTCTAATAAAGTCCCCAGGCACCTCACATGCCTTGTTATCACCCTCTCAAAGCATTTCAATGCTTTGACAAATTCAATT
GTTTTCGACGAAGTTCGGAAACATGACAATCGTTCGATCTTTAGACCTGCCCTGTGGCAGTGCCGTTGGATGCCGTCTAGAACGATCAAGACAAT
GGGCTATTCGGTGCATGCACGAAGCCCAATTGCATAAAAACAACTCATTCATAACACTCACATATGACAATACACATCTCCCAAGCGATGGCTCT
TGGATCACAAAGACTTCAACTGTTCCTTAAAAGACTTAGAAAAACTCTCGCAAAAAGAGGACGTACAATCCGCTATTACATGGCTGGAGAATATG
GTGAACTCTTCGCAAGGACCCACTTCCATGCCTGTATCTTCGGATACGACTTTCCTGATAAAAAATTATGGAAAGGACTGCCTCTGGTTCTATGTT
ATATAGATCCGCAGAACTTGAAGCTCTCTGGCCATTTGGTTTACCACCATTGGAGATGTTACTATTCGAATCAGCCGCCTACGTGGCTAGATACAT
AATGAAAAAACAAACAGGGAAAAGATGCGGAATCTCATTACAAACGCATACACCCTGAAACCGGCGAATATTTAGACTTAAAGCCGGAATATAA
TAAAATGTCTTTAAAACCGGGAATCGGTAAAGACTTTTATATAAAATATACTTCGGATATATACCCGCAAGACTACGTACTACTTAGAGGTAAAA
AGGTCAAACCACCAAAATACTATGACAAAATGTTTAAAATTGACCAACCTTATGAGTATGACGAATTACTTTACATGCGGGAAAATAACGCTAAA
CTTAATTCCGAAGACATACACCAGAACGACTATCTGCAAAAGAACAAGTAACTATGGCAAAACTTCAACTATTAAAACGTAAACCTCACTTAGGA
AAATAATGAAACTTATCCTCGCTTCCGTAAAAGGACCGTGCTGCTGAAGCATATGCACGACCAATGTTCGTACCTTCCTCTGGAGTAGCTATACG
CTCTTTTTCAGATGAAATTAATCGTTCTGATACTGAAAATCAACTTTTAATCACCCTGATGACTTCGATCTATATGAATTCGGAACTTTTGACGATT
CAACTGGGTTATTCGATTTACATGAACAACCAAAACTTCTATCATTAGGAAAACAAGTTAAACTTAAATAAAACAACCGAGGGGAAAAGAGATTT
ATCTTTCCCCCGGAACAACACTAAGGAAAAACATGCACCGCAATCAGTCAGTTAATACTCCCTCGCTTCGCGATGGTACCTAGAGCCGATATACC
ACGTAGTAAATTCGATGCTCAAAAAACACATAAAACGACTTTCGATGCGGGCTATTTAATTCCTGTATATGTTGATGAAGTGCTCCCTGGGGACA
CTTTCAACTTAAAAATGACGGCATTTGCCCGTCTAGCAACGCCTTTATATCCAATCATGGACAACATGATTATGGATTCTTTCTTTTTCTTTGTACCC
AATCGCCTTATATGGAATAACTGGCAAAAATTTATGGGTCAACAAGAAAATCCAACAGACTCAATATCTTATATTGTCCCAACTCAAACAAGCCC
AACAGATGGTTATGCCGTAGGCAGCCTTCAAGACTATATGGGCTTACCAACAG

>000016F|arrow

CTTTGGAACACAAAGTCAAAACGCAGCGAGTGCAGAACAAGCGAGTAATCAACAGAGATTTCAAGCTGAAATGTCTGGAAATCATATCAACGA
GCAGTTGAAGATATGAAAAAAGCTGGGTTAAATCCCATGCTTGCGTATTCACAAGGCGGAGCCACAACACCAGCTGGAGCTATGCCCAGATGCA
AAATGTTCGGTAATGCAACTACGTCCGGAACCCAAGCTTATCAAACGGTTGCGCAAGCAATCAAGCTATGCTCAATCTAAACAAATTGAAGCTCA
AACAGAACTCACAAGTAATCAAACAGAATGTACGTGCTGATACGTTAAAAAAATTGGATGAAAATCCAAATATTAGAGCTCAATATAAACAAATAC
TTGCCGATACTTCATGAAAAATGAAATAGGCAAAACATCAAGTGCTCAAGCTCAAGCTTTGGCACAATCGTTATTCAAACGAGTTAACAAAACTT
GCTAAATCAGGGTCAGCCTAGTTCTAGCAAACCAATTATCAAGACGTAAAAAACATCGCCAAAGATGGTATAGCGCATCTGGCGCAAAAACGAT
ACATCGATAACTATCGAGGTCAACCGATTCAACAAAATCGTACAAATAACCAACCACCAATGGAATGAAAATGACAAAGATTACAGCCCATTTCT
TCGTACTCCGTACAATTACGACACGATTGCTGCGTCAAATGAGTCAGGGCTGCATTGTGAGGATGCAACTCTGACTCAGCAGCAATTTGCTGAAG
AATGCGTATAATAATATTATGGAAAAGTTGGTATGACCGGACTTATCCTCAAACTCCTTAACGCCTCAATATGGCGACTTTAGTGGTGTCTATGAC
TACCACTCTGCTCTGAACCAGATTATGGCTTCAGACAACGAATTTATGGCTTTACCAGCCAATATTCGTGAACGATTCGCTAATGATCCCGCGAAT
CTAATAGATTTCTAGAAAACCCTGAAAATCGCAGCGAAGCTGAAAAAATGGGACTGGTAAAACCAGCCCAAACCGAGGTTTCAACCCTGTTGGA
ACTCGGAAGCACAGTTACCTACTTGATGAACTGTGCTAGGTGACACCAAACCACAAAAACACGATAAACAAGGACAGAAAAAATGATGCGTCGC
AGACCAGCAAATAAGCAAAAGTCGCTAGGACTTTCCGTAAACATGCTTCACATACAAAACACGCAAATATGCGAAACTCGCCAATGCGTGGAGG
CTGGGACTCTAATAAAGTCCAGGCACCTCACATGCCTTGTTATCACCCTCTCAAAGCATTTCAATGCTTTGACAAATCAATTGTTTTCGACGAAGTT
CGGAAACATGACATCGTTCGATCTTTAGACCTGCCCTGTGGGCAGTGCGTTGGATGCCGTCTAGAACGATCAAGACAATGGGCTATTCGGTGCA
TGCACGAAGCCCAATTGCATAAAAACAACTCATTCATAACACTCACATATGACAATACACATCTCCCAAGCGATGGCTCTTTGGATCACAAAGACT
TTCAACTGTTCCTTAAAAGACTTAGAAAAACTCTCGCAAAAAGAGGACTTACAATCCGCTATTACAGGCTGGAGAATATGGTGAACTCTTCGCAA
GACCCCACTTCCATGCCTGTATCTTCGGATACGACTTTCCTGATAAAAAATTATGGAAAAGGACTGCCTCTGGTTCTATGTTATATAGATCCGCAG
AACTTGAAGCTCTCTGGCCATTTGGTTATACCACCATTGGAGATGTTACTTTGAATCAGCCGCCTACGTGGCTAGATACATAATGAAAAAACAAA
CAGGGAAAGATGCGGAATCTCATTACAAACGCATACACCCTGAAACCGGCGAATATTTAGACTTAAAGCCGGAATATAATAAAATGTCTTTAAA
ACCGGGAATCGGTAAAGACTTTTATAAAATATACTTCGGATATATACCCGCAAGACTACGTAATACTTAGAGGTAAAAAGGTCAAACCACCAAA
ATACTATGACAAAATGTTTAAAATTGACCAACCTTATGAGTATGACGAATTACTTTACATGCGGGAAAATAATGCTAAATTAATTCCGAAGACAA
TACACCAGAACGACTATCTGCAAAAGAACAAGTCACTATGGCAAAACTTCAACTATTAAAACGTAATTCACTTAGGAAAATAATGAAACTTATCC
TCGCTTCCGTAAAAGACCGTGCTGCTGAAGCATATGCACGACCAATGTTCGTACCTTCCTCTGGAGTAGCTATACGCTCTTTTTCAGATGAAATTA
ATCGTTCTGATACTGAAAATCAACTTTTAATCACCCTGATGACTTCGATCTATATGAATTCGGAACTTTGACGATTCAACTGGGTTATTCGATTTAC
ATGAACAACCAAAACTCCTATCATTAGGAAAACAAGTTAAACTTAAATAAAACAACCGAGGGGAAAAGAGATTTATCTTTCCCCCGGAACAAAC
ACTAAGGAAAAACATGCACCGCAATCAGTCAGTTAATACTCACCGCTTCGCGATGGTACTAGAGCCGATATACCACGTAGTAAATTCGATGCTCA
AAAACACATAAAACGACTTTCGATGCGGGCTATTAATTCCTGTATATGTTGATGAAGTGCTCCCTGGGGACACTTTCAACTTAAAAATGACGGCA
TTTGCCCGTCTAGCAACGCCTTTATATCCAATCATGGATAACATGATTATGGATTCTTTCTTTTTCTTTGTACCCAATCGCCTTATATGGAATAACTG

GCAAAAATTTATGGGTCAACAAGAAAATCCAACAGACTCAATATCTTATATTGTCCCAACACAAACAAGCCCAACAGATGGTTATGCCGTAGGCA
GCCTTCAAGACTATATGGGCTTACCAACAGTAGGCCAAATTGATACTGGCCGAACTATTACGCACTGTGCCTTTTGGCCACGTGCATACAATCTTA
TCTGGAACGAATGGTTCCGAGATGAAAATTTACAAACAAGCGCAGTAGTTGATAAGGGCGATGGCCTGATACTTCCTCAAACTATGTGCTAAAA
CGTCGTGGTAAAAGACATGATTACTTTACGTCAGCATTACCATGGCCACAAAAAGGTGCGAGTGTCACCTTACCTTTAGGTACTACGGCTCCAAT
TAAATGGGATACCATTTCAGGAGACGCAACATCAAACGATAAATTTACGGTAATTCAAACAGATCCTGGAAATACGACTGCTTTAGCTAGATATG
GCAACGCTTATGGTGTTAATACTGCTGGTGTAGTAAATAACGTTTCTAATTTATATACCGACTTATCAGAAGCAACTGCTGCAACTGTCAATCAAT
TAAGACAGTCATTTCAAATTCAAAAATTACTTGAAAGGGATGCACGTGGCGGAACACGATACACAGAAATTATCCGGAGTCACTTTGGAGTTATT
TCCCCAGACGCCCGTTTACAAAGGCCTGAATACCTTGGAGGCGGTTCAACACCAATTAATGTTAATCCGATTGCTCAAACGTCGGGAACAAACGC
TTCTGGAACGACTACCCCTTTGGGCAACCTTGCTGCTATGGGTACTGCTCTCGCTCATAATCATGGATTTACTCAATCATTTACTGAGCATGGCGT
TATTATTGGATTAGTATCCATTAGAGCAGATCTTACTTATCAACAAGGATTAGACCGTATGTGGTCTAGATCTACACGATATGACTTTTATTTCCCA
GCATTTGCTACTTAGGCGAACAATCTGTTTTGCAAAAAGAAATTTATGCAACAGGAGATACTGCAGCCGACAATGTTTTTGGATATCAAGAACGC
TGGGCGGAATATCGTTACAAACCATCTAAAATTACTGGTTTGTTCAAATCAACATCGGCGGGCACGATCGATGGTTGGCATTTGGCTCAAAAATT
TACCGCTGCGCCTACTTTGAATAATACGTTTATTCAAGATACGCCTCCTGTATCACGTGTAGTAGCCGTTGGAGCAGCTGCAAATGGCCAACAATT
ATATTGACTCATTTTTTGATGTCAAAATGGCAAGACCAATGCCAATGTATTCAGTACCTGGCTTAATAGACCATTTCTAATGGGACTATTTGACGG
AATTGCCGATTTAATCGGCCCTGCTATAGCTATAGGAGCTGCCCCTGCTACTGGGGGACTCTCCTTAGCTGCACTTGCACCTGCAGCAATAGGTG
CAGCAGGACAATA

>000139F|arrow

TGTGACTTTTCACAATCTCACATATTAATTTCTACTGTGTAGAAATACAAGGGATTAAAAATCCCCTTTATAATTTCCCTTTATTTAATTAAATTATA
TTTAATTAAATATATATACTTAAATCAAATTAATTTGATTAATTAAACACATACACCATAATGGTGTATGTCTTAATAATTCTGACAAATTAATTAA
ATATATATTAATTAATTAAAGTCTAGAAATTATATCAGAATTAATCAGATTGATTAATTAAAATAGACACATATAAATATAAATGGCGAAGCCATT
TATATTTATATATAATAAATAAATAAATAAAATAAACATATAAGTAAATAAATATATAAATAAAAAAATGGAGTCTAATTATAGGATGAATCGAA
AGGCTTAAGAAAGAGAAAATTTAAGCCTATATTTAAAACTAAAGGCTCTTTTAAGAATAATTCTCTTGTTACTAATAAAACTTTATACAGGAAAAT
ACCTCGTAATCCTGTTTTAAAACCTGAACTTAAATAGATATCTGCTAGTTCTAATGCTTCTACCTTTGGTACAACTACTGCTTATTATGTACCAGAA
TATAAAATACCTATTGGTGCAGATTATACTAAACGTATTGGTAATAAGATATTGGTTAGAAAAATTATAATACGTATTTAAATAAAAATGTAAACT
TAAACTCTTGCTAGAATAATTATATCTTCTAGAAAAGATTTTGGTGTAGCTGTAGATTAAGAATTAGCTATATAAGTAGCATCTATATTAACACCT
AATAAACTTAAAGATTCAAATGTGTTCATTGATACTATGAAAGTTAAAAAGTAACCCTTTGATGATTAAGAATTTCAAGTATTTGAATATACACAT
TATATTAATAAAGTTTGGTAATATTCTGCTGATGATACTTAAGTACCTTCTTTATCTCCAGGTGCTCCTTAATTTTACTATGCTGGTCCTGCAGTTAA
TGCTGTAACTGATTAAATAACTGTTTATACTATGCTTTGTTTTAATGACGATTGAATAAAAATTATATATATATATAAATTAATACATATAATACATAT
AATACATAATATATATACTACATGTCTTTATAAACAATGTCTTTTGAACACGACCCCTATATAAAAACGGCCATAGGCACTCGGATCCCAGAATAACA
CTTGGACCCAGTACCAAAACGCTTGCGGTTTAGCAGAACCCCGTCGAGCCGAAGGCGAGACAAAGGCCGCACCAAAACAACTTTTTAAAGGCCA
TTTTCAAAAAGTTACATTCGCTTTCTTCTATTATTATAAGAAAGCGAATGTGACT

>000061F|arrow

TTGATAAGTAAGATCTGCTCTAATGGATACTAATCCAATAATAACGCCATGCTCAGTAAATGATTGAGTAAATCCATGATTATGAGCGAGAGCAG
TACCCATAGCAGCAAGGTTGCCCAAAGGGGTAGTCGTTCCAGAAGCGTTTGTTCCCGACGTTTGAGCAATCGGATTAACATTAATTGGTGTTGAA
CCGCCTCCAAGGTATTCAGGCCTTTGTAAACGGGCGTCTGGGGAAATAACTCCAAAGTGACTCCGGATAATTTCTGTGTATCGTGTTCCGCCACG
TGCATCCCTTTCAAGTAATTTTTGAATTTGAAATGACTGTCTTAATTGATTGACAGTTGCAGCAGTTGCTTCTGATAAGTCGGTATATAAATTAGA
AACGTTATTTACTACACCAGCAGTATTAACACCATAAGCGTTGCCATATCTAGCTAAAGCAGTCGTATTTCCAGGATCTGTTTGAATTACCGTAAA
TTTATCGTTTGATGTTGCGTCTCCTGAAATGGTATCCCATTTAATTGGAGCCGTAGTACCTAAAGGTAAGGTGACACTCGCACCTTTTTGTGGCCA
TGGTAATGCTGACGTAAAGTAATCATGTCTTTTACCACGACGTTTTAGCACATAGTTTGAGGAAGTATCAGGGCCATCGCCCTTATCAACTACTGC
GCTTGTTTGTAAATTTTCATCTCGGAACCATTCGTTCCAGATAAGATTGTATGCACGTGGCCAAAAGGCACAGTGCGTAATAGTTCGGCCAGTAT
CAATTTGGCCTACTGTTGGTAAGCCCATATAGTCTTGAAGGCTGCCTACGGCATAACCATCTGTTGGGCTTGTTTGTGTTGGGACAATATAAGAT
ATTGAGTCTGTTGGATTTTCTTGTTGACCCATAAATTTTTGCCAGTTATTCCATATAAGGCGATTGGGTACAAAGAAAAAGAAAGAATCCATAATC
ATGTTATCCATGATTGGATATAAAGGCGTTGCTAGACGGGCAAATGCCGTCATTTTTAAGTTGAAAGTGTCCCCAGGGAGCACTTCATCAACATA
TACAGGAATTAGATAGCCCGCATCGAAAGTCGTTTTATGTGTTTTTTGAGCATCGAATTTACTACGTGGTATATCGGCTCTAGGTACCATCGCGAA
GCGGTGAGTATTAACTGACTGATTGCGGTGCATGTTTTTCCTTAGTGTTGTTCCGGGGGAAAGATAAATCTCTTTTCCCTCGGTTGTTTTATTTAA
GTTTAACTTGTTTTCCTAATGATAGGAGTTTTGGTTGTTCATGTAAATCGAATAACCCAGTTGAATCGTCAAATGTTCCGATTCATATAGATCGAA
GTCATCAGGGTGATTAAAAGAGTTGATTTTCAGTATCAGAACGATTAATTTCATCTGAAAAAGAGCGTATAGCTACTCCAGAGGAAGGTACGAA
CATTGGTCGTGCATATGCTTCAGCAGCACGGTCTTTTACGGAAGCGAGGATAAGTTTCATTATTTTCCTAAGTAAGGTTACGTTTTAATAGTTGAA
GTTTTGCCATAGTGACTTGTTCTTTTGCAGATAGTCGTTCTGGTGTATTGTCTTCGGAATTAAATTTAGCATTATTTTCCCGCATGTAAAGTAATTC
GTCATACTCATAAGGTTGGTCAATTTTAAACATTTTGTCATAGTATTTTGGTGGTTTGACCTTTTTACCTCTAAGTATTACGTAGTCTTGCGGGTAT

ATATCCGAAGTATATTTATATAAAAGTCTTTACCGATTCCGGTTTTAAAGACATTTTATTATATTCCGGCTTTAAGTCTAAATATTCGCCGGTTTCA
GGGTGTATGCGTTTGTAATGAGATTCCGCATCTTTCCCTGTTTGTTTTTTCATTATGTATCTAGCCACGTAGGCGGCTGATTCAAAGTAACATCTCC
AATGGTGGTATAACCAAATGGCCAGAGAGCTTCAAGTTCTGCGGATCTATATAACATAGAACCAGAGGCAGTCCTTTTCCATAATTTTTTATCAG
GAAAGTCGTATCCGAAGATACAGGCATGGAAGTGGGGTCTTGCGAAGAGTTCACCATATTCTCCAGCCATGTAATAGCGGATTGTAAGTCCTCT
TTTTTGCGAGAGTTTTTCTAAGTCTTTTAAGGAACAATTGAAAGTCTTGTGATCCAAAGAGCCATCGCTTGGGAGATGTGTATTGTCATATGTGA
GTGTTATGAATGAGTTGTTTTATGCAATTGGGCTTCGTGCATGCACCGAATAGCCCATTGTCTTGATCGTTCTAGACGGCATCCAACGCACTGCCC
ACAGGGCAGGTCTAAAGATCGAACGATGTCATGTTTCCGAACTTCGTCGAAAACAATTGATTTGTCAAAGCATTGAATATGCTTTGAGAGGGTG
ATAACAAGGCATGTGAGGTGCCTGAAGACTTTATTAGAGTCTCCAGCCTCCACGCATTGGCGAGTTTCGCATATTTGCGTTTTTGTATGTGAAGC
ATGTTTACGGAAAGTCCTAGCGGACTTTTGCTTAGTTGCTGGTCTGCGACGCATCATTTTTTCTGTCCTTGTTTATCGTGTTTTGTGGTTTGGTGTC
ACCTAGCCAGTTACATCAAGTAGGTAACTGTGCTTCCGAGGTTCCAACAGGGGTTGAAACCTCGGTTTGGCTGGTTTTACCAGTCCCATTTTTCA
GCTTCGCTGCGATTTTCAGGGTTTTCTAGAAATCTATTAGATTCGCGGGATCATTAGCGAATCGTTCACGAATATTGGCTGGTAAAGCCATAAATT
CGTTGGTCTGAAGCCATAATCTGTCAGAGCAGAGTGGTAGTCATAGACACCACTAAAGTCGCCATATTGAGGCGTTAAAGGGTTGAGGAATAA
GTCCGGTCATACCGAAACTTTTCCATAATAGTATTGAATATCAGCATTCTTCAGCAAATTGCTGCTGAGTCAGAGTTGCATCCTCACAATGCAGCC
CTGACTCATTTGACGCAGCAATCGTGTCGTAATTGTACGGAGTACGAAGAAATGGGGCTGTAATCTTTGTCATTTTCATTCCATGTGGTTGGTTAT
TTGTACGATTTTGTTGAATCGGTTGACCTCGATAGTTATCGATGTATCGTTTGCGCCAGATGCGCTATACTCATCTTTGGCGATATTTTTTACGTCT
TGATAAATTGGTTTGCTAGAAAACTAGGAGCTGACCCTGATTTAGCAAGTTTTGTTAACTCGTTTTGAATAACGAGATTGTGCCAAAGCTTGAGC
AGCTTGAGCACTTGATGTTTTGCCTATTTCATTTTTCATGAAAGTATCGGCAAGTATTTGTTTTATATTGAGCTCTAATAGTTGGATTTTCATCCAAT
TTAGTTTAACGTATCAGCACGGTACATTATCTGTGTGAGTACTTGTGAGTTCTGTTTGAGCTTCAATTTGTTTAGATGAGCAATAGCTGATTTGCTT
GCGCAACCGTTTGATAAGCTTGGGTTGCCGGACGTAGTTGCATTACCGAGAACATTTGCATCTGGGCCATAGCTCCAGCTGGTGTTGTGGCTCC
GCCTTGTGAATACGCAAGCATGGGATTTAACCCAGCTTTTTCATATCTTCAACTGCTCGTTGATATGATGTTCCAGACATTTCAGCTTGAAATCTCT
GTTGATTACTCGCTTGTTCTGCACTCGCTGCGTTTTTGACTTGTGTTCCAAAGTATTGTCCTGCTGCACCTATTGCTGCAGGTGCAAGTGCAGCTA
AGGAGAGTCCCCCAGTAGCAGGGGCAGCTCCTATAGCTATAGCAGGGCCGATTAAATCGGCAATTCCGTCAAATAGTCCCATTAGAAATGGTCT
ATTAAGCCAGGTACTGAATACATTGGCATTGGTCTTGCCATTTTGACATCAAAAAATGAGTCAAATAAGAATTGTTGGCCATTTGCAGCTGCTCC
AACGGCTACTACACGTGATACAGGAGGCGTATCTTGAATAAACGTATTATTCAAAGTAGGCGCAGCGGTAAATTTTTGAGCCAAATGCCAACCA
TCGATCGTGCCCGCCGATGTTGATTTGAACAAACCAGTAATTTTAGATGGTTTGTAACGATATTCCGCCCAGCGTTCTTGATATCCAAAACAGTAT
TGTCGGCTGCAGTATCTCCTGTTGCATAAATTTTCTTTTGCAAAACAGATTGTTCGCCTAGAGTAGCAAATGCTGGGAAATAAAAGTCATATCGTG
TAGATCTAGACCACATACGGTCTAATCCTTG

>000021F|arrow

TGAAGCATGTTTACGGAAAGTCCTAGCGGACTTTTGCTTATTTGCTGGTCTGCGACGCATCATTTTTTCTGTCCTTGTTTATCGTGTTTTTGTGGTT
GGTGTCACCTAGCACAGTTACATCAAGTAGGTAACTGTGCTTCCGAGGTTCCAACAGGGGTTGAAACCTCGGTTTGGGCTGGTTTTACCAGTCCC
ATTTTTTCAGCTTCGCTGCGATTTTCAGGGTTTTCTAGAAAATCTATTAGATTCGCGGGATCATTAGCGAATCGTTCACGAATATTGGCTGGTAAA
GCCATAAATTCGTTGTCTGAAGCCATAATCTGGTTCAGAGCAGAGTGGTAGTCATAGACACCACTAAAGTCGCCATATTGAGGCGTTAAAGGAG
TTTGAGGAATAAGTCCGGTCATACCGAACTTTTCCATAATATTATTGATATCACATTCTTCAGCAAATTGCTGCTGAGTCAGAGTTGCATCCTCAC
AATGCAGCCCTGACTCATTTGACGCAGCAATCGTGTCGTAATTGTACGGAGTACGAAGAAATGGGGCTGTAATCTTTGTCATTTTCATTCCATTG
GTGGTTGGTTATTTGTACGATTTTGTTGAATCGGTTGACCTCGATAGTTATCGATGTATCGTTTTGCGCCAGATGCGCTATACGCATCTTTGGCGA
TTTTTTACGTCTTGATAAATTGGTTTGCTAGAACTAGGAGCTGACCCTGATTTAGCAAGTTTTGTTAACTCGTTTGAATAACGAGATTGTGCCAAA
GCTTGAGCAGCTTGAGCACTTGATGTTTTGCCTATTTCATTTTTCATGAAAGTATCGGCAAGTATTTGTTTATATTGAGCTCTAATATTTGGATTTT
CATCCAATTTGTTTAACGTATCAGCACGTACATTATCTGTTTGATTACTTGTGAGTTCTGTTTGAGCTTCAATTTGTTTAGATTGAGCAATAGCTTG
ATTTGCTTGCGCAACCGTTTGATAAGCTTGGGTTCCGGACGTAGTTGCATTACCGAGAACATTTTGCATCTGGGCCATAGCTCCAGCTGGTGTTG
TGGCTCCGCCTTGTGAATACGCAAGCATGGGATTTAACCCAGCTTTTTTCATATCTTCAACTGCTCGTTGATATGATGTTCCAGACATTTCAGCTTG
AAATCTCTGTTGATTACTCGCTTGTTCTGCACTCGCTGCGTTTTGACTTTGTGTTCCAAAGTATTGTCCTGCTGCACCTATTGCTGCAGGTGCAAGT
GCAGCTAAGGAGAGTCCCCCAGTAGCAGGGGCAGCTCCTATAGCTATAGCAGGGCCGATTAAATCGGCAATTCCGTCAAATAGTCCCATTAGAA
ATGGTCTATTAAGCCAGGTACTGAATACATTGGCATTGGTCTTGCCATTTTGACATCAAAAAATGAGTCAAATAAGAATTGTTGGCCATTTGCAG
CTGCTCCAACGGCTACTACACGTGATACAGGAGGCGTATCTTGAATAAACGTATTATTCAAAGTAGGCGCAGCGGTAAATTTTTGAGCCAAATGC
CAACCATCGATCGTGCCCGCCGATGTTGATTTGAACAAACCAGTAATTTTAGATGGTTTGTAACGATATTCCGCCCAGCGTTCTTGATATCCAAAA
ACAGTATTGTCGGCTGCAGTATCTCCTGTTGCATAAATTTCTTTTTGCAAAACAGATTGTTCGCCTAGAGTAGCAAATGCTGGGAAATAAAAGTC
ATATCGTGTAGATCTAGACCACATACGGTCTAATCCTTGTTGATAAGTAAGATCTGCTCTAATGGATACTAATCCAATAATAACGCCATGCTCAGT
AAATGATTGAGTAAATCCATGATTATGAGCGAGAGCAGTACCCATAGCAGCAAGGTTGCCCAAAGGGGTAGTCGTTCCAGAAGCGTTTGTTCCC
GACGTTTGAGCAATCGGATTAACATTAATTGGTGTTGAACCGCCTCCAAGGTATTCAGGCCTTTGTAAACGGGCGTCTGGGGAAATAACTCCAA
AGTGACTCCGGATAATTTCTGTGTATCGTGTTCCGCCACGTGCATCCCTTTCAAGTAATTTTGAATTTGAAATGACTGTCTTAATTGATTGACAGTT
GCAGCAGTTGCTTCTGATAAGTCGGTATATAAATTAGAAACGTTATTTACTACACCAGCAGTATTAACACCATAAGCGTTGCCATATCTAGCTAAA

GCAGTCGTATTTCCAGGATCTGTTTGAATTACCGTAAATTTATCGTTTGATGTTGCGTCTCCTGAAATGGTATCCCATTTAATTGGAGCCGTAGTA
CCTAAAGGTAAGGTGACACTCGCACCTTTTTGTGGCCATGGTAATGCTGACGTAAAGTAATCATGTCTTTTACCACGACGTTTTAGCACATAGTTT
GAGGAAGTATCAGGGCCATCGCCCTTATCAACTACTGCGCTTGTTTGTAAATTTTCATCTCGGAACCATTCGTTCCAATAAGATTGTATGCACGTG
GCCAAAAGGCACAGTGCGTAATAGTTCGGCCAGTATCAATTTGGCCTACTGTTGGTAAGCCCATATAGTCTTGAAGGCTGCCTACGGCATAACCA
TCTGTTGGGCTTGTTTGTGTTGGGACAATATAAGATATTGAGTCTGTTGGATTTTCTTGTTGACCCATAAATTTTTGCCAGTTATTCCATATAAGGC
GATTGGGTACAAAGAAAAAGAAAGAATCCATAATCATGTTATCCATGATTGGATATAAAGGCGTTGCTAGACGGGCAAATGCCGTCATTTTTAA
GTTGAAAGTGTCCCCAGGGAGCACTTCATCAACATATACAGGAATTAGATAGCCCGCATCGAAAGTCGTTTTATGTGTTTTTGAGCATCGAATTT
ACTACGTGGTATATCGGCTCTAGGTACCATCGCGAAGCGGTGAGTATTAACTGACTGATTGCGGTGCATGTTTTTCCTTAGTGTTGTTCCGGGGG
AAAGATAAATCTCTTTTCCCCTCGGTTGTTTATTTAAGTTTAACTTGTTTTCCTAATGATAGGAGTTTTGGTTGTTCATGTAAATCGAATAACCCAG
TTGAATCGTCAAAGTTCCGAATTCATATAGATCGAATCATCAGGGTGATTAAAGAGTTGATTTTCAGTATCAGAACGATTAATTTCATCTGAAAAA
GAGCGTATAGCTACTCCAGAGGAAGGTACGAACATTGGTCGTGCATATGCTTCAGCAGCACGGTCTTTTACGGAAGCGAGGATAAGTTTCATTA
TTTTCCTAAGAGGTTACGTTTTAATAGTTGAAGTTTTGCCATAGTACTTGTTCTTTTGCAGATAGTCGTTCTGGTGTATTGTCTTCGGAATTAATTT
AGCATTATTTTCCCGCATGTAAAGTAATTCGTCATACTCATAAGGTTGGTCAATTTTAAACATTTTGTCATAGTATTTTGGTGGTTTGACCTTTTTAC
CTCTAAGTATTACGTAGTCTTGCGGGTATATATCCGAAGTATATTTTATTAAAAGTCTTTACCGATTCCCGGTTTTAAAGACATTTTATTATATTCC
GGCTTTAAGTCTAAATATTCGCCGGTTTCAGGGTGTATGCGTTTGTAATGAGATTCCGCATCTTTCCCTGTTTGTTTTTTCATTATGTATCTAGCCA
CGTAGGCGGCTGATTCAAAAGTAACATCTCCAATGGTGGTATAACCAAATGGCCAGAGAGCTTCAAGTTCTGCGGATCTATATAACATAGAACC
AGAGGCAGTCCTTTTCCATAATTTTTTATCAGGAAAGTCGTATCCGAAGATACAGGCATGGAAGTGGGGTCTTGCGAAGAGTTCACCATATTCTC
CAGCCATGTAATAGCGGATTGTAAGTCCTCTTTTTGCGAGAGTTTTTCTAAGTCTTTTAAGGAACAATTGAAAGTCTTTGTGATCCAAAGAGCCAT
CGCTTGGGAGATGTGTATTGTCATATGTGAGTGTTATGAATGAGTTGTTTTTATGCAATTGGGCTTCGTGCATGCACCGAATAGCCCATTGTCTTG
ATCGTTCTAGACGGCATCCAACGCACTGCCCACAGGGCAGGTCTAAAGATCGAACGATGTCATGTTTCCGAACTTCGTCGAAAACAATTGATTTG
TCAAAGCATTGATATGCTTTGAGAGGGTGATAACAAGGCATGTGAGGTGCCTGAAGACTTTATTAGAGTCTCCAGCCTCCACGCATTGGCGAGT
TTCGCATATTTGCGTGTTTTGTATG

>000207F|arrow

AGTTGAAAGTGTCCCCAGGGAGCACTTCATCAACATATACAGGAATTAAATAGCCCGCATCGAAAGTCGTTTTATGTGTTTTTGAGCATCGAAT
TTACTACGTGGTATATCGGCTCTAGGTACCATCGCGAAGCGATGAGTATTAACTGACTGATTGCGGTGCATGTTTTTCCTTAGTGTTGTTCCGGG
GGAAAGATAAATCTCTTTTCCCCTCGGTTGTTTATTTAAGTTTAACTTGTTTTCCTAATGATAGAAGTTTTGGTTGTTCATGTAAATCGAATAACC
CAGTTGAATCGTCAAAAGTTCCGAATTCATATAGATCGAAGTCATCAGGGTGATTAAAAAGTTGATTTTCAGTATCAGAACGATTAATTTCATCTG
AAAAAGAGCGTATAGCTACTCCAGAGGAAGGTACGAACATTGGTCGTGCATATGCTTCAGCAGCACGGTCTTTTACGGAAGCGAGGATAAGTTT
CATTATTTTCCTAAGTGAGAGTTACGTTTTAATAGTTGAAGTTTTGCCATAGTTACTTGTTCTTTTGCAGATAGTCGTTCTGGTGTATTGTCTTCGG
AATTAAGTTAGCGTTATTTTCCCGCATGTAAAGTAATTCGTCATACTCATAAGGTTGGTCAATTTAAACATTTTGTCATAGTATTTTGGTGGTTTGA
CCTTTTTACCTCTAAGTATTACGTAGTCTTGCGGGTATATCCGAAGTATATTTTATATAAAAGTCTTTACCGATTCCCGGTTTTAAAGACATTTTATT
ATATTCCGGCTTTAAGTCTAAATATTCGCCGGTTTCAGGGTGTATGCGTTTGTAATGAGATTCCGCATCTTTCCCTGTTTGTTTTTTCATTATGTATC
TAGCCACGTAGGCGGCTGATTCGAAAGTAACATCTCCAATGGTGGTATAACCAAATGGCCAGAGAGCTTCAAGTTCTGCGGATCTATATAACAT
AGAACCAGAGGCAGTCCTTTTCCATAATTTTTTATCAGGAAAGTCGTATCCGAAGATACAGGCATGGAAGTGGGGTCTTGCGAAGAGTTCACCA
TATTCTCCAGCCATGTAATAGCGGATTGTAAGTCCTCTTTTTGCGAGAGTTTTTCTAAGTCTTTTAAGGAACAGTTGAAAGTCTTTGTGATCCAAA
GAGCCATCGCTTGGGAGATGTGTATTGTCATATGTGAGTGTTATGAATGAGTTGTTTTTATGCAATTGGGCTTCGTGCATGCACCGAATAGCCCA
TTGTCTTGATCGTTCTAGACGGCATCCAACGCACTGCCCACAGGGCAGGTCTAAAGATCGAACGATGTCATGTTTCCGAACTTCGTCGAAAACAA
TTGATTTGTCAAAGCATTGAAATGCTTTGAGAGGGTGATAACAAGGCATGTGAGGTGCCTGGGGACTTTATTAGAGTCTCCAGCCTCCACGCATT
GGCGAGTTTCGCATATTTGCGTGTTTTGTATGTGAAGCATGTTTACGGAAAGTCCTAGCGGACTTTTGCTTATTTGCTGGTCTGCGACGCATCATT
TTTTCTGTCCTTGTTTATCGTGTTTTTGTGGTTTGGTGTCACCTAGCACAGTTACATCAAGTAGGTAACTGTGCTTCCGAGGTTCCAACAGGGGTT
GAAACCTCGGTTTGGGCTGGTTTTACCAGTCCCATTTTTTCAGCTTCGCTGCGATTTTCAGGGTTTTCTAGAAAATCTATTAGATTCGCGGGATCA
TTAGCGAATCGTTCACGAATATTGGCTGGTAAAGCCATAAATTCGTTGTCTGAAGCCATAATCTGGTTCAGAGCAGAGTGGTAGTCATAGACACC
ACTAAAGTCGCCATATTGAGGCGTTAAAGGAGTTTGAGGAATAAGTCCGGTCATACCGAACTTTTCCATAATATTATTAATATCGCATTCTTCAGC
AAATTGCTGCTGAGTCAGAGTTGCATCCTCACAATGCAGCCCTGACTCATTTGACGCAGCAATCGTGTCGTAATTGTACGGAGTACGAAGAAATG
GGGCTGTAATCTTTGTCATTTTCATTCCATTGGTGGTTGGTTATTTGTACGATTTTGTTGAATCGGTTGACCTCGATAGTTATCGATGTATCGTTTT
GCGCCAGATGCGCTATACGCATCTTTGGCGATGTTTTTTACGTCTTGATAAATTGGTTTGCTAGAACTAGGAGCTGACCCTGATTTAGCAAGTTTT
GTTAACTCGTTTGAATAACGAGATTGTGCCAAAGCTTGAGCAGCTTGAGCACTTGATGTTTTGCCTATTTCATTTTTCATGAAAGTATCGGCAAGT
ATTTGTTTATATTGAGCTCTAATATTTGGATTTTCATCCAATTTGTTTAACGTATCAGCACGTACATTATCTGTTTGATTACTGTGAGTTCTGTTTGA
GCTTCAATTTGTTTAGATTGAGCAATAGCTTGATTTGCTTGCGCAACCGTTTGATAAGCTTGGGTTCCGGACGTAGTTGCATTACCGAGAACATTT
TGCATCTGGGCCATAGCTCCAGCTGGTGTTGTGGCTCCGCCTTGTGAATACGCAAGCATGGGATTTAACCCAGCTTTTTTTCATATCTTCAACTGC
TCGTTGATATGATGTTCCAGACATTTCAGCTTGAAATCTCTGTTGATTACTCGCTTGTTCTGCACTCGCTGCGTTTTGACTTTGTGTTCCAAAGTATT

GTCCTGCTGCACCTATTGCTGCAGGTGCAAGTGCAGCTAAGGAGAGTCCCCAGTAGCAGGGGCAGCTCCTATAGCTATAGCAGGGCCGATTTAA
ATCGGCAATTCCGTCAAATAGTCCCATTAGAAATGGTCTATTAAGCCAGGTACTGAATACATTGGCATTGGTCTTGCCATTTGACATCAAAAAAT
GAGTCAAATAAGAATTGTTGGCCATTTGCAGCTGCTCCACGGCTACTACACGTGATACAGGAGGCGTATCTTGAATAAACGTATTATTCAAAGTA
GGCGCAGCGGTAAATTTTGAGCCCAAATGCCAACCATCGATCGTGCCCGCCGATGTTGATTGAACAAACCAGTAATTTTAGATGGTTTGTAACGA
ATTCCGCCCAGCGTTCTTGATATCCAAAAACAGTATTGTCGGCTGCAGTATATCCTGTTGCATAAATTTCTTTTTGCAAAACAGATGTTCGCCTAG
AGTAGCAAATGCTGGGAAATAAAAGTCATATCGTGTAGATCTAGACCACATACGGTCTAATCCTTGTTGATAAGTAAGATCTGCTCTAATGGATA
CTAATCCAATAATAACGCCATGCTCAGTAAATGATTGAGTAAATCCATGATTATGAGCGAGAGCAGTACCCATAGCAGCAAGGTTGCCCAAGGG
GTAGTCGTTCCAGAAGCGTTTGTTCCCGACGTTTGAGCAATCGGATTAACATTAATTGGTGTTGAACCGCCTCCAAGGTATTCAGGCCTTTGTAA
ACGGGCGTCTGGGGAAATAACTCCAAAGTGACTCCGGATAATTTCTGTGTATCGTGTTCCGCCACGTGCATCCCTTTTCAAGTATTTTGAATTTGA
ATGACTGTCTTAATTGATTGACAGTTTGCAGCAGTTGCTTTGATAAGTCGGTATATAAATTAGAAAACGTTATTACTACACCAGCAGTATTAACAC
CATAAGCGTGCCATATCTAGCTAAAGCAGTCGTATTTCCAGGATCTGGTTTGATTACCGTAAATTTACTCGTTTGATGTTGCGTCTCTGAAATGGC
TATCCCATTTAATTGGAGCCGTAGTACCTAAAGGGTAAGGTGACACTCGCACCTTTTGTGGCCATGGTAATGCTGACGTAAAGGTAATCATGTCT
TTACCAGCGACGCTTTTAGCACATAGTTTGAGATGAAGTATCAGGGCCATCGCCCTTATCAACTACTGCGCTTGTTTGTAAATTTTCATCTCGAAC
CATTCGTTCCAAATAAGATTGTATGCACGTGGCCAAAAGGCACAGTGCGTAATAGTTCGGCCAGTATCAATTTGGCCTACTGTGGTAAGCCCATA
TAGTCTTGAAGGCTGCCTACGCATAACCATCTGTTGGCTTGTTTGAGTTGGGACAATATAAGATATTGAGTTCTGTTGGATTTTCTTGTTGACCCA
TAAATTTTTGCCAGTTATTCCATATAAGGCGATTGGGTACAAAGAAAAGAAAGAATCCATAATCATGTTGTCCATGATTGGATATAAAGGCGTTG
CTAGACGGGCAAATGCCGTCATTTTA

>000005F|arrow

CGATGTTTTTTACGTCTTGATAAATTGGTTTGCTAGAACTAGGAGCTGACCTGATTTAGCAAGTTTTGTTAACTCGTTTGAATAACGAGATTGTGC
CAAAGCTTGAGCAGCTTGAGCACTTGATGTTTTGCCTATTTCATTTTTCATGAAAGTATCGGCAAGTATTTGTTTATATTGAGCTCTAATATTTGGA
TTTTCATCCAATTTATTTAACGTATCAGCACGTACATTATCTGTTTGATTACTTGTGAGTTCTGTTTGAGCTTCAATTTGTTTAGATTGAGCAATAGC
TTGATTTGCTTGCGCAACCGTTTGATAAGCTTGGGTTCCGGACGTAGTTGCATTACCGAGAACATTTTGCATCTGGGCCATAGCTCCAGCTGGTG
TTGTGGCTCCGCCTTGTGAATACGCAAGCATGGGATTTAACCCAGCTTTTTTCATATCTTCAACTGCTCGTTGATATGATGTTCCAGACATTTCAGC
TTGAAATCTCTGTTGATTACTCGCTTGTTCTGCACTCGCTGCGTTTTGACTTTGTGTTCCAAAGTATTGTCCTGCTGCACCTATTGCTGCAGGTGCA
AGTGCAGCTAAGGAGAGTCCCCCCAGTAGCAGGGGCAGCTCTATAGCTATAGCAGGGCCGATTAAATCGGCAATTCCGTCAAATAGTCCCATTA
GAAATGGTCTATTAAGCCAGGTACTGAATACATTGGCATTGGTCTTGCCATTTTGACATCAAAAAATGAGTCAAATAAGAATTGTTGGCCATTTG
CAGCTGCTCCAACGGCTACTACAGTATACAGGGGGGGTACAGGAGCGTATCTTGAATAAACGCTATTATTCAAAAGGTAGGCGCAGCGGTAAA
TTTTTGAGCCAAATGCCAACCATCGATCGTGCCCGCGATGTTGATTTGAACAAACCAGTAATTTTAGATGGTTGTACCGATATTCCGCCCAGCGTC
TTGATATCCAACAACAGTATTGTCGGCTGCAGTATCTCCCTGTTGCATAAATTTTCTTTTTGCAAAAACAGATTGTCGCCTAGAGTAGCAAATGCT
GGGAATAAAAGTCATATCGTGTAGATCTAGACCACATACGGTCTAATCCTTGTTGATAAGTAAGATCTGCTCTAATGGATACTAATCCAATAATA
ACGCCATGCTCGTAAATGATTGAGTAAATCCATGATTATGAGCGAGAGCAGTACCCATAGCAGCAAGGTTGCCCAAAGGGGTAGTCGTTCCAGA
AGCGTTTGTTCCCGACGTTTGAGCAATCGGATTAACATTAATTGGTGTGAACCCGCCTCCAAGGTATTCAGGCCTTTGTAAACGGGCGTCTGAGG
GAAATAACTCCAAAGTGACTCCGGATAATTTCTGTGTATCGGTTCCGCCACGTGCATCCCTTTCAAAGTAATTTTTGAATTGAAATGACTGTCTTA
ATTGATTGACAGTTGCAGCAGTTGCTTCTGATAAGTTCGGTATCTAAATTAGAAACGTTATTACTACACCAGCAGTATTAACACCATAAGCGTTGC
CATATCTAGCTAAAGCAGTCGTATTTCCAGGATCTGTTTGAATTACCGTAAAATTTATCGTTTGATGTTGGTCTCTGCAATGGTATCCCATTTAATT
GGAGCCGTAGTACCTAAAGGTAAGGTGACACTCGCACCTTTTTGTGGCCATGGTAATGCTGACGTAAAGTAATCATGTCTTTTACCACGACGTTT
TAGCACATAGTTTGAGGAAGTATCAGGGCCATCGCCCTTATCAACTACTGCGCTTGTTTTGTTAAATTTTCATCTCGGAACCATTCGTTCCAGATA
AGATTGTATGCACGTGGCCAAAAGGCACAGTGCGTAATAGTTCGGCCAGTATCAATTTGGCCTACTGTTGGTAAGCCCATATAGTCTTGAAGGCT
GCCTACGGCATAACCATCTGTTGGGCTTGTTTGTGTTGGGACAATATAAGATATTGAGTCTGTTGGATTTTCTTGTTGACCCATAAATTTTTGCCA
GTTATTCCATATAAGGCGATTGGGTACAAAGAAAAAGAAAGAATCCATAATCATGTTATCCATTATGGATATAAAGGCGTGCTAGACGGGCAAA
TGCCGTCATTTTTAAGTGAAAGGTCCAGGGAGCACTTCATCAACATATACAGGAATTAGATAGCCCGCATCGAACGTCGTTTTATGTGTCTTTTTG
AGCATCGAATTTACTACGTGGTCTATCGGCTCTAGGTACCATCGCGAAGCGGTGAGTATTAACTGACATGATTGCGGTGCATGTTTTTCCTTAGT
GTTGTTCCGGGGGAAAGATAAATCTCTTTTCCCCTCGGTTGTTTTATTTAAGTTACTTGTTTCCTAATGATAGGAGTTTTGGTTGTTCATGTAAATC
GATAACCCAGTGAATCGTCAAATGTTCCGAATTCATATAGATCGAATCATCAGGGTGATTAAAGAGTTGATTTCCGTATCAGAACGATTAATTTC
ATCTGAAAAAGAGCGTATAGCTCCTCCAGAGGAAGGTACGAACATTGGTCGTGCATATGCTTCCAGGCCAGGCACGGTCTTTTACGGAGAGCGA
GGATAAGTTTCATTATTTCCTAAGTAAGGTTACGTTTTTACTAGTTGAAGTTTTGCCATAGTGACTTGTTCTTTTGCAGATAGTCGTTCTGGTGTAT
TGTCTTCGGAATTAAATTTAGCATATTTTCCCGCATGTAAAGTAATTCGCTCATACTCATAAGTTGGTCAATTTTAAACATTTGGTCCATAGTATTT
TGGTGGTTTGACCTTTTTACCTCTAAGTATTACGTAGTCTTGCGGGTATATATCCGAAGTATATTTTATATAAAAGTCTTTACCGATTCCCGGTTTT
AAAGACATTTTATTATATTCCGGCTTTAAGTCTAAATATTCGCCGGTTTCAGGGTGTATGCGTTTGTAATGAGATTCCGCATCTTTCCCTGTTTGTT
TTTCATTATGTATCTAGCCACGTAGGCGGCTGATTCAAAAGTAACATCTCCAATGGTGGTATAACCAAATGGCCAGAGAGCTTCAAGTTCTGCGG
ATCTATATAACATAGAACCAGAGGCAGTCCTTTTCCATAATTTTTTATCAGGAAAGTCGTATCCGAAGATACAGGCATGGAAGTGGGGTCTTGCG

AAGAGTTCACCATATTCTCCAGCCATGTAATAGCGGATTGTAAGTCCTCTTTTTGCGAGAGTTTTTCTAAGTCTTTTAAGGAACAATTGAAAGTCT
TTGTGATCCAAAGAGCCATCGCTTGGGAGATGTGTATTGTCATATGTGAGTGTTATGAATGAGTTGTTTTATGCAATTGGGCTTCGTGCATGCAC
CGAATAGCCCATTGTCTTGATCGTTCTAGACGGCATCCAACGCACTGCCCACAGGGCAGGTCTAAAGATCGAACGATGTCATGTTTCCGAACTTC
GTCGAAAACAATTGATTTGTCAAAGCATTGATATGCTTGAGAGGGTGATAACAAGGCATGTGAGGTGCCTGAAGACTTTATTAGAGTCTCCAGC
CTCCACGCATGGCGAGTTTCGCATATTTGCGTGTTTTGTATGTGAAGCATGTTTACGGAAAGTCCTAGCGGACTTTTGCTTATTTGCTGGTCTGCG
ACGCATCATTTTTTCTGTCCTTGTTTATCGTGTTTTTGTGGTTTGGTGTCACCTAGCACAGTTACATCAAGTAGGTAACTGTGCTTCCGAGGTTCCA
ACAGGGGTTGAAACCTCGGTTTGGGCTGGTTTTACCAGTCCCATTTTTTCAGCTTCGCTGCGATTTTCAGGGTTTTCTAGAAAATCTATTAGATTC
GCGGGATCATTAGCGAATCGTTCACGAATATTGGCTGGTAAAGCCATAAATTCGTTGTCTGAAGCCATAATCTGGTTCAGAGCAGAGTGGTAGT
CATAGACACCACTAAAGTCGCCATATTGAGGCGTTAAAGGAGTTTGAGGAATAAGTCCGGTCATACCGAACTTTTCCATAATATTATTGATATCA
CATTCTTCAGCAAATTGCTGCTGAGTCAGAGTTGCATCCTCACAATGCAGCCCTGACTCATTTGACGCAGCAATCGTGTCGTAATTGTACGGAGT
ACGAAGAAATGGGGCTGTAATCTTTGTCATTTTCATTCCATTGGTGGTTGGTTATTTGTACGATTTTGTTGAATCGGTTGACCTCGATAGTTATCG
ATGTATCGTTTTGCGCCAGATGCGCTATACGCATCTTTGG

>000066F|arrow

CCCTTCTGTGGCCAAGGCAATGCACCAGTAAAATAATCTTTACGCTTACCACGTCTCAAAAGTGTATAGTTAGCAGGAACATCACCTGAATCACC
AAGATTTAACGTTACAGAATTCTGTAAGTTTTCATCTCTAAACCATTCGTTATAAATCTCATTATAAGCACGTAACGGCAAAACATTATGCGTTACT
GTATTAGCGCCAGTAATCTGACCAGCAGTAGGTAAACCAAAATAATCAAAAATTGAACAAACAGCATAACCACCAGCAGGACTAGTAATAGTAG
GCACTACAAAAGAAATAGAATCACCAGGATTCGTTTGTTCACCCATAAACTTTGGCCAATTGTTCCAAAGTAAACGATTAGGTACAAAGAAAAGA
AAGTATCAAGATGCAAGTTGTCCATCACAGGAAATAACGGTGTGGCCAAACGTGCAAATGCAGTCATCTTTACACGATGAGTATCGCCAGGCAA
TACTTCATCACAATAAATAGGAACTAAAAACCAGCATCAAACGTGGTTTTATGAGCATATTGAGTATTAAAACTAGAGCGGGGAATGTCCGCTTT
AGGAACCATAGCAAATGAATGCGTACTTACTGACTTATTGCGAAACATAAACATCTCCCGTAGTTCCGTACCACTCTTTCGAGTGATACGGTATA
AAAAAAACCTTACTCGCCTTCGCGAATCATTACATCTTTTGCACGAGCAATCAACTTAGGGCTTTCCAGTAGTTCAAAAACACCAGTGGCATCGTC
AAAAAGACCCAAATAGAACATATGAAAATCATCAGGATGTTTATACAACTGATTATCTTCGCTAGCTCGATTGACTTCATCCTGAAACTGACGTAC
TGCAACACCTTCAGATGCAACATAAGCTGGACGACCAAAAGCATCTGCAGCAGTATCCTTAATAGAAACAATAACCATCTTCATAAAAACTCCTT
AAATAGTACGTTTTAACAATGACAACTTAGCCAACGCAACTTTTTCCTTAACAGCAAGTCGCTCAAGCGTGTTGTCCTCATGCCTAGATCGACCTT
CCATCTCTCTGGCAAACTGAATCATATCGAATTCTTCAGGAAACTTCAACTTAAATTTATTATCATAAAACCGTGGTGGACGGCACTTTTTGCCAC
GCACCACAACGTGGTCTGACGTATAAACGTCTGACATGTACTTATCTAACCACGATTGCCCGATACCGGGCTTCAATGACATCTTATTAAATTCTG
GCTTACGCTGAATTATCTCACCAGTCTCTAAATCACAATATTGATAATGGGCACCCGCATCAACCACTTCGTGGTTTTCATTGACAGTAACCCCATT
AATCTTCTTCATAATATATCTTGCAACATAAGCAGCAGACTCAAAAGTAACATCACCAATTGTAGAATAGCCAAACGGCCACAATTCTTCCAAATC
TCTGACGTGTAGAGGATAGAGCCAGTCTGCGTTCTTTTAAATATTTTCTTATCCGGAAAATCAAGACCAAACAGACAAGCATGGAAATGAGGAC
GATCAAAAGATTCACCATATTCACCTGCCATATAAAAACGTATCGTTTTCCCAGTAAAACGCTTACGTAACCGCTTCATAAAAAGCTGATAATCAT
TGTAATCCATGACATATCCTTAGGACAATGCTCTGGAGCATATGTCAAAGTAATAAAACAATTACTAGTATGCATTTGTGCCTCATGCATACAACG
AATCGCCCACTGACGTGAGCGTTCAAGGCGACAACCAACACACTGACCACAAGGCAATGATAGGGTACGGACTACATCCGCACCCGGTATTTCC
CGCCAAATTATAGACCTGTCACTGCATTGATAAGCCGTTAAGGGCTTATAACAGGCCATAATTACAAACGATAGCCACCACGCTGGGGAGCGTG
TCTCATATTAATTGACTTCGTCTTACTAGCAGTTCTGCGAAATGACTTTGCAGATTTATATTTGTTTACTGGCTTTCTTCGTAACATGATGAACTCCG
TAGTTAAAATAGTGGTTTGGTGTCACCTAGCACAGTTACATCAAGTAGAGTAACTGTGCTGGCCTCAGGATTTCATCCTTCGGCCTTAGGTGTTTC
TGTAGAAACGATGGGTTCAACCACAGGTTGTCCATCAATAAGACCCAATTGAATCGCTTCATCACGATTCTGGTCGTTCTCAAGGAACTCCAATA
ATTTGACAGGATCATGGTCAAATCGGACTCTTAATTTCGCTGGCAGAGCCATGAAATCGTCCATAGTTGCGTTAATTTGATTCAACGCAGAATGG
TAATCAGTAACACCACTAAAATCGCCGTATTGAGGCGATACAGGGGCCGTTGGAAGTTCCCCTGTAACCCCGAAACGTTCAATGATGACATTAAT
GTCACATTCATCTTTCATATGCTGTTGAGCAAGACTTGGGTCTTGACACAGCAACGCATCGTTTACCGATGCTTCATCTTTATCATAGTTGTACGG
ATTACGTACAAATGGCAAATTCGCTTTACTCATTATTTGACTCCAATTCCCAAGGGGTTAGTTGATTAGGATTATTTTTGAGTTTATCAAAAATTTC
TTTCGAAGCATCGATACCCTAGACCAAATAGCTGGGGATGGAGCCAATAGCCTCTTAGTATTATATGACTGAGCGGATGTTAAATCCGCAGTCGT
ATTCTTTAAAGCAATATCAGCCAAAATACGTTTATTTCGGCTGTAATATTAGGAGCAGTAAGCAACTTATTAACAGTATCAGCCCTAGTATTAGCG
GTACCAGCTTCAGTAGCTTCAGTCTGGGCGATAATCTGCTTTTCTGTTCTTGATAGCATACGAATCTGTTGCATCATAGAAGCAGTATGATAGCTG
AATTAGTTTGCATTACCTAAAACATTTTCCATATTAGCAGTTTGAACACCAGATGGGTAGAAGCACCACAACGTTGATAGGCCCCTAACATAGCG
AGATAAACCAGCAGCGTCTAAATCTTTAACAGCCACGCTGTAACTGTTCCACTCATATAGACGGCTTGAAATCCACTTATGCCTCTTGAGCCATCC
TCCTGATTAGCTTTCTTAGCAGAAGTAGCACCTAAATAAGAACCAACAGGCGCCCAAAGCAGTACCGACACCAGGGTAAAGAAACTAGAAGCTG
AAGACAGCTTAGAACCAACATTAGTAACCGCATCAAGTATTCCACAAACATAAACTAACGCCCTTCGGTTGTTTCCTCACTACTCCTTACGGAGTA
GTCGAGGTTTATATAAAACATTAGAAATGTCAATTAGACCAGGTACGCTGTACATAGGCATAGGTCTGGCCATCTGACAATCAAAGAAAGCATC
CATTAAAAATGCTGACCATTTGCTGCAGCTCCGACCGCCGTAGTACGAGCAACTGGATGGGTCTCTTGAATAAACGTTGAATTCAACGTAGGAA
GAGAAGTAAATCGCTGTCCATAATGCCAAGCATCAATAGTGCCAGCAGAAGTAGACCTAAGAAACCAGTATTTGAGAAGGTTTGTAACGATAAC
TCCGCCCAACGTTCTTGATAACCAAATACATCATTGTCGGCTGCAGTACCTTGAACATAAATTTCTTTGTTCAAAATAGCTTGCTCACCAAAGTGG

CAATACTGGAATAAAAGTCATATCGTGTAGACCTAGACCACATCTTAGGAAACCCTGTTGATATGTGAGTCAGCACGTACAGAAACTAATCCGAT
AATGTATCCATGTCTGAGCAGCATACGCTAAAGCCGTGTCCCCTGAGCCAATGCAGTACCCATTGCAGCAAGGTTACCTTTGCGGAGTAGCAGA
ACCAGTAAACCGACGTTGCAGAAGTCTGAGCAATCGGATTAACATTAACAAGGGTCGAACCTCCACCAATATATTCAGGACGTTGTAAACGATA
ATCCTGTGGAGTTACTCCAAAGTGAGCACGTAGTAACTCAGTATAACGAGTACCACCACGTGCATCGCGCTCCAATAACTTCTGAATCTGGAAAG
ATTGACGAAGTTGGTTAATAGTCGCAGCAGTAGCAGTAGACAAATCAGCGTATAACTGATTAGTAGCAACACCAGCATTTGTACTATTAGACAA
AGTATTAGAAGCTGAATTTAATTCTCTTAAAGCACCCACAGCAGTTTGAAAAACACTATAACCAGCAGTACCATTGCCAGCTGGTATGTCAGAAT
AAATATTAGCACGTGTTCCTAACGGTAAAGAAACAGAAGCA

>000182F|arrow

CATATCTAGCTAAAGCAGTCGTATTTCCAGGATCTGTTTGAATTACCGTAAATTTATCGTTTGATGTTGCGTCTCCTGAAATGGTATCCCATTTAAT
TGGAGCCGTAGTACCTAAAGGTAAGGTGACACTCGCACCTTTTTGTGGCCATGGTAATGCTGACGTAAAGTAATCATGTCTTTACCACGACGTT
TTAGCACATAGTTTGAGGAAGTATCAGGGCCATCGCCCTTATCAACTACTGCGCTTGTTTGTAAATTTTCATCTCGGAACCATTCGTTCCAGATAA
GATTGTATGCACGTGGCCAAAAGGCACAGTGCGTAATAGTTCGGCCAGTATCAATTTGGCCTACTGTTGGTAAGCCCATATAGTCTTGAAGGCT
GCCTACGGCATAACCATCTGTTGGGCTTGTTTGAGTTGGGACAATATAAGATATTGAGTCTGTTGGATTTTCTTGTTGACCCATAAATTTTTGCCA
GTTATTCCATATAAGGCGATTGGGTACAAAGAAAAAGAAAGAATCCATAATCATGTTATCCATGATTGGATATAAAGGCGTTGCTAGACGGGCA
AATGCCGTCATTTTTAAGTTGAAAGTGTCCCCAGGGAGCACTTCATCAACATATACAGGAATTAAATAGCCCGCATCGAAAGTCGTTTATGTGTTT
TTTGAGCATCGAATTTACTACGTGGTATATCGGCTCTAGGTACCATCGCGAAGCGGTGAGTATTAACTGACTGATTGCGGTGCATGTTTTTCCTTA
GTGTTGTTCCGGGGGAAAGATAAATCTCTTTTCCCCTCGGTTGTTTTATTTAAGTTTAACTTGTTTTCCTAATGATAGGAGTTTTGGTTGTTCATGT
AAATCGAATAACCCAGTTGAATCGTCAAAATGTTCCGAATTCATATAGATCGAAAGTCATCAGGGTGATTAAAGAGTTGATTTTCAGTATCAGAA
CGATTAATTTCATCTGAAAAAGAGCGTATAGCTACTCCAGAGGAAGGTACGAACATTGGTCGTGCATATGCTTCAGCAGCACGGTCTTTTACGGA
AGCGAGGATAAGTTTCATTATTTTCCTAAGTAGGTTACGTTTTAATAGTTGAAGTTTTGCCATAGTGACTTGTTCTTTTGCAGATAGTCGTTCTGGT
GTATTGTCTTCGGAATTAAATTTAGCATTATTTCCCGCATGTAAAGTAATTCGTCATACTCATAAGGTTGGTCAATTTTAAACATTTTGTCATAGTA
TTTTGGTGGTTTGACCTTTTTACCTCTAAGTATTACGTAGTCTTGCGGGTATATATCCGAAGTATATTTTATATAAAAGTCTTTACCGATTCCCGGT
TTTAAAGACATTTTATTATATTCCGGCTTTAAGTCTAAATATTCGCCGGTTTCAGGGTGTATGCGTTTGTAATGAGATTCCGCATCTTTCCCTGTTT
GTTTTTTCATTATGTATCTAGCCACGTAGGCGGCTGATTCGAAAGTAACATCTCCAATGGTGGTATAACCAAATGGCCAGAGAGCTTCAAGTTCT
GCGGATCTATATAACATAGAACCAGAGGCAGTCCTTTTCCATAATTTTTTATCAGGAAAGTCGTATCCGAAGATACAGGCATGGAAGTGGGGTCT
TGCGAAGAGTTCACCATATTCTCCAGCCATGTAATAGCGGATTGTAAGTCCTCTTTTTGCGAGAGTTTTTCTAAGTCTTTTAAGGAACAGTTGAAA
GTCTTTGTGATCCAAAGAGCCATCGCTTGGGAGATGTGTATTGTCATATGTGAGTGTTATGAATGAGTTGTTTTTATGCAATTGGGCTTCGTGCAT
GCACCGAATAGCCCATTGTCTTGATCGTTCTAGACGGCATCCAACGCACTGCCCACAGGGCAGGTCTAAAGATCGAACGATGTCATGTTTCCGAA
CTTCGTCGAAAACAATTGATTTGTCAAAGCATTGAAATGCTTTGAGAGGGTGATAACAAGGCATGTGAGGTGCCTGGGACTTTATTAGAGTCTCC
AGCCTCCACGCATTGGCGAGTTTCGCATATTTGCGTGTTTTGTATGTGAAGCATGTTTACGGAAAGTCCTAGCGGACTTTTGCTTATTTGCTGGTC
TGCGACGCATCATTTTTTCTGTCCTTGTTTATCGTGTTTTTGTGGTTTGGTGTCACCTAGCACAGTTACATCAAGTAGGTAACTGTGCTTCCGAGGT
TCCAACAGGGGTTGAAACCTCGGTTTGGGCTGGTTTTACCAGTCCCATTTTTTCAGCTTCGCTGCGATTTTCAGGGTTTTCTAGAAAATCTATTAG
ATTCGCGGGATCATTAGCGAATCGTTCACGAATATTGGCTGGTAAAGCCATAAATTCGTTGTCTGAAGCCATAATCTGGTTCAGAGCAGAGTGGT
AGTCATAGACACCACTAAAGTCGCCATATTGAGGCGTTAAAGGAGTTTGAGGAATAAGTCCGGTCATACCGAAACTTTTCCATAATATTATTAAT
ATCGCATTCTTCAGCAAATTGCTGCTGAGTCAGAGTTGCATCCTCACAATGCAGCCCTGACTCATTTGACGCAGCAATCGTGTCGTAATTGTACGG
AGTACGAAGAAATGGGGCTGTAATCTTTGTCATTTTCATTCCATTGGTGGTTGGTTATTTGTACGATTTTGTTGAATCGGTTGACCTCGATAGTTA
TCGATGTATCGTTTTGCGCCAGATGCGCTATACGCATCTTTGGCGATGTTTTTTACGTCTTGATAAATTGGTTTGCTAGAACTAGGAGCTGACCCT
GATTTAGCAAGTTTTGTTAACTCGTTTGAATAACGAGATTGTGCCAAAGCTTGAGCAGCTTGAGCACTTGATGTTTTGCCTATTTCATTTTTCATGA
AAGTATCGGCAAGTATTTGTTTATATTGAGCTCTAATATTTGGATTTTCATCCAATTTATTTAACGTATCAGCACGTACATTATCTGTTTGATTACTT
GTGAGTTCTGTTTGAGCTTCAATTTGTTTAGATTGAGCAATAGCTTGATTTGCTTGAGCAACCGTTTGATAAGCTTGGGTTCCGGACGTAGTTGCA
TTACCGAGAACATTTTGCATCTGGGCCATAGCTCCAGCTGGTGTTGTGGCTCCGCCTTGTGAATACGCAAGCATGGGATTTAACCCAGCTTTTTTC
ATATCTTCAACTGCTCGTTGATATGATGTTCCAGACATTTCAGCTTGAAATCTCTGTTGATTACTCGCTTGTTCTGCACTCGCTGCGTTTTGACTTTG
TGTTCCAAAGTATTGTCCTGCTGCACCTATTGCTGCAGGTGCAAGTGCAGCTAAGGAGAGTCCCCCAGTAGCAGGGGCAGCTCCTATAGCTATA
GCAGGGCCGATTAAATCGGCAATTCCGTCAATAGTCCCATTAGAAATGGTCTATTAAGCCAGGTACTGAATACATTGGCATTGGTCTTGCCATTT
TGACATCAAAAAATGAGTCAAATAAGAATTGTTGGCCATTTGCAGCTGCTCCAACGGCTACTACACGTGATACAGGAGGCGTATCTTGAATAAAC
GTATTATTCAAAGTAGGCGCAGCGGTAAATTTTTGAGCCAAATGCCAACCATCGATCGTGCCCGCCGATGTTGATTTGAACAAACCAGTAATTTT
AGATGGTTTGTAACGATATTCCGCCCAGCGTTCTTGATATCCAAAAACAGTATTGTCGGCTGCAGTATCTCCTGTTGCATAAATTTCTTTTTGCAAA
ACAGATTGTTCGCCTAGAGTAGCAAATGCTGGGAAATAAAAGTCATATCGTGTAGATCTAGACCACATACGGTCTAATCCTTGTTGATAAGTAAG
ATCTGCTCTAATGGATACTAATCCAATAATAACGCCATGCTCAGTAAATGATTGAGTAAATCCATGATTATGAGCGAGAGCAGTACCCATAGCAG
CAAGGTTGCCCAAAGGGGTAGTCGTTCCAGAAGCGTTTGTTCCCGACGTTTGAGCAATCGGATTAACATTAATTGGTGTTGAACCGCCTCCAAG
GTATTCAGGCCTTTGTAAACGGGCGTCTGGGGAAATAACTCCAAAGTGACTCCGGATAATTTCTGTGTATCGTGTTCCGCCACGTGCATCCCTTTC

AAGTAATTTTTGAATTTGAAATGACTGTCTTAATTGATTGACAGTTGCAGCAGTTGCTTCTGATAAGTCGGTATATAAATTAGAAACGTTATTTAC
TACACCAGCAGTATTAACACCATAAGCGTTGC

>000050F|arrow

CATAAACTTTGGCCAATTGTTCCAAAGTAAACGATTAGGTACAAAGAAAAAGAAAGTATCAAGATGCAAGTTGTCCATCACAGGAAATAACGGT
GTGGCCAAACGTGCAAATGCAGTCATCTTTACACGATGAGTATCGCCAGGCAATACTTCATCACAATAAATAGGAACTAAAAAACCAGCATCAA
ACGTGGTTTTATGAGCATATTGAGTATTAAAACTAGAGCGGGGAATGTCCGCTTTAGGAACCATAGCAAATGAATGCGTACTTACTGACTTATTG
CGAAACATAAACATCTCCCGTAGTTCCGTACCACTCTTTCGAGTGATACGGTATAAAAAAAACCTTACTCGCCTTCGCGAATCATTACATCTTTTGC
ACGAGCAATCAACTTAGGGCTTTCCAGTAGTTCAAAAAACACCAGTGGCATCGTCAAAAAGACCCAAATAGAACATATGAAAATCATCAGGATG
TTTATACAACTGATTATCTTCGCTAGCTCGATTGACTTCATCCTGAAACTGACGTACTGCAACACCTTCAGATGCAACATAAGCTGGACGACCAAA
AGCATCTGCAGCAGTATCCTTAATAGAAACAATAACCATCTTCATAAAAACTCCTTAAATAGTACGTTTTAACAATGACAACTTAGCCAACGCAAC
TTTTTCCTTAACAGCAAGTCGCTCAAGCGTGTTGTCCTCATGCCTAGATCGACCTTCCATCTCTCTGGCAAACTGAATCATATCGAATTCTTCAGGA
AACTTCAACTTAAATTTATTATCATAAAACCGTGGTGGACGGCACTTTTTGCCACGCACCACAACGTGGTCTGACGTATAAACGTCTGACATGTAC
TTATCTAACCACGATTGCCCGATACCGGGCTTCAATGACATCTTATTAAATTCTGGCTTACGCTGAATTATCTCACCAGTCTCTAAATCACAATATT
GATAATGGGCACCCGCATCAACCACTTCGTGGTTTTCATTGACAGTAACCCCATTAATCTCTTCATAATATATCTTGCAACATAAGCAGCAGACTC
AAAAGTAACATCAACCAATTGTAGAATAGCCAAACGGCCACAATTCTTCCAAAATCTCTGACGTGTAGAGGATAGAGCCAGTCTGCGTTCTTTTA
AATATTTTCTTATCCGGAAAATCAAGACCAAACAGACAAGCATGGAAATGAGGACGATCAAAAGATTCACCATATTCACCTGCCATATAAAAACG
TATCGTTTTCCCAGTAAAACGCTTACGTAACCGCTTCATAAAAAGCTGATAATCATTGTAATCCAATGACATATCCTTAGGACAATGCTCTGGAGC
ATATGTCAAAGTAATAAAACAATTACTAGTATGCATTTGTGCCTCATGCATACAACGAATCGCCCACTGACGTGAGCGTTCAAGGGCGACAACCA
ACACACTGACCACAAGGCAATGATAGGGTACGGACTACATCCGCACCCGGTATTTCCCGCCAAATTATAGACCTGTCACTGCATTGATAAGCCGT
TAAGGGCTTATAACAGGCCATAATTACAAACGATAGCCACCACGCTGGGGAGCGTGTCTCATATTAATTGACTTCGTCTTACTAGCAGTTCTGCG
AAATGACTTTGCAGATTTATTTTGTTTACTGGCTTTCTTCGTAACATGATGAACTCCGTAGTTAAAATAGTGGTTTGGTGTCACCTAGCACAGTTAC
ATCAAGTAGAGTAACTGTGCTGGCCTCAGGATTTCATCCTTCGGCCTTAGGTGTTTCTGTAGAAACGATGGGTTCAACCACAGGTTGTCCATCAA
TAAGACCCAATTGAATCGCTTCATCACGATTCTGGTCGTTCTCAAGGAACTCCAATAATTTGACAGGATCAGGGTCAATCGGACTCTTAATTTCGC
TGGCAGAGCCATGAAATCGTCCATAGTTGCGTTAATTTGATTCAACGCAGAATGGTAATCAGTAACACCACTAAAATCGCCGTATTGAGGCGATA
CAGGGGCCGTTGGAAGTTCCCCTGTAACCCCGAAACGTTCAATGATGACATTAATGTCACATTCATCTTTCATATGCTGTTGAGCAAGACTTGGG
TCTTGACACAGCAACGCATCGTTTACCGATGCTTCATCTTTATCATAGTTGTACGGATTACGTACAAATGGCAAATTCGCTTTACTCATTATTTGAC
TCCATTCCCCAAGGGGTTAGTTGATTAGGATTATTTTTGAGTTTATCAAAAATTTCTTTCGAAGCATCGATACCCCTAGACCAAATAGCTGGGGAT
GGAGCCAATAGCCTCTTAGTATTATATGACTGAGCGGATGTTAAATCCGCAGTCGTATCCTTTAAAGCAATATCAGCCAAAATACGTTTATTTTCG
GCTGTAATATTAGGAGCAGTAAGCAACTTATTAACAGTATCAGCCCTAGTATTAGCGGTACCAGCTTCAGTAGCTTCAGTCTGGGCGATAATCTG
CTTTTCTGTTTCTGATGCATTACGAATCTGTTGCATCATAGAAGCAGTATTAATAGCTGAATTAGTTGCATTACCTAAAACATTTTCCATAGTACAG
TTGAACCAGATTGGGTAGAAGCACCACCACGTTGATAGGCTAACATAGGAGATAAACCAGCAGCTTCTAAATCTTTAACAGCACGCTGGTAACTT
GTTTCCACTCATATCGGCTTGAAAAATCCATTTGCCTCTGAGCCATCTCCTGATTAGCTTTATTAGCAGATAGACCTAAATAAGAACCAACAGCGC
CCAAAAGCAGTACCGACACCAGGAGTAAAGAAACTAGAAGCTGAAGACAGCTTAGAACCAACATTAGTAACCGCATCAAGTATTCCACCAAACA
TAAACTAACGCCCTTCGGTTGTTTCCTCACTACTCCTTACGGAGTAGTCGAGGTTATATAAAACATTAGAAATGATCAATTAGACCAGGTACGCTG
TACATAGGCATAGGTCTGGCCATCTGACAATCAAAGAAAGCATCCATTAAAAATTGCTGACCATTTGCTGCAGCTCCGACCGCCGTAGTACGAGC
AACTGGAGGGGTCTCTTGAATAAACGTTGAATTCAACGTAGGAAGAGAAGTAAATCGCTGTCCATAATGCCAAGCATCAATAGTGCCAGCAGAA
GTAGACCTAAAGAAACCAGTAATTTGAGAAGGTTTGTAACGATACTCCGCCCAACGTTCTTGATAACCAAATACATCATTGTCGGCTGCAGTACC
TTGAACATAAATTTCTTTGTTCAAAATAGCTTGCTCACCCAAAGTGGCAAATACTGGGAAATAAAAGTCATATCGTGTAGACCTAGACCACATCTT
AGGAAGACCCTGTTGATATGTGAGGTCAGCACGTACAGAAACTAATCCGATAATGTATCCATGTTCTTGAGCAGCATACGTAAAGCCGTGTCCCT
GAGCCAATGCAGTACCCATTGCAGCAAGGTTACCTTGCGGAGTAGCAGAACCAGTAACCGACGTTGCAGAAGTCTGAGCAATCGGATTAACATT
AACAAGGGTCGAACCTCCACCAATATATTCAGGACGTTGTAAACGATAATCCTGTGGAGTTACTCCAAAGTGAGCACGTAGTAACTCAGTATAAC
GAGTACCACCACGTGCATCGCGCTCCAATAACTTCTGAATCTGGAAAGATTGACGAAGTTGGTTAATAGTCGCAGCAGTAGCAGTAGACAAATC
AGCGTATAACTGATTAGTAGCAACACCAGCATTTGTACTATTAGACAAAGTATTAGAAGCTGAATTTAATTCTCTTAAAGCACCAACAGCAGTTTG
AAAAACACTATAACCAGCAGTACCATTGCCAGCTGGTATGTCAGAATAAATATTAGCACGTGTTCCTAACGGTAAAGAAACAGAAGCACCCTTCT
GTGGCCAAGGCAATGCACCAGTAAAATAATCTTTACGCTTACCACGTCTCAAAAGTGTATAGTTAGCAGGAACATCACCTGAATCACCAAGATTT
AACGTTACAGAATTCTGTAAGTTTTCATCTCTAAACCATTCGTTATAAATCTCATTATAAGCACGTAACGGCAAAACATTATGCGTTACTGTATTAG
CGCCAGTAATCTGACCAGCAGTAGGTAAACCAAAATAATCAAAAATTGAACAAACAGCATAACCACCAGCAGGACTAGTAATAGTAGGCACTAC
AAAAGAAATAGAATCACCAGGATTCGTTTGTTCACC

>000142F|arrow

GAAGCAATCCCTATCTTCTCTATGGCAGAGATGCGTGAGAATCCTCGTGCCAAATATATAACTCAAACCATTGGTGGAGGTTTGGTTACTCTTAAT
GGTAAAATATACCTTCCTAGTCTTGTTGGACGTAGTAAGGCTCAGTATATGGCTGATGACCGTTATCAGGCCCCTGTTACCTCATCTCCTCAAGAG
CTTGCTATTCTTAATATTTTATCCTCTGCTAATGGTTCTGATATTCCCAATACTGCTACGTTTCAGATTGTCTTGGAATATACCGTTGAATGGTTCGA
TATCAAGCATCTTACTCAATCATAAGTGGCGTAGCCCGTGGCGTAGCCCCATAGCCAATTCTGCCGTCATTAAACCCCCGAAGGGCCTACAGGCA
AGATTCACTAACAGGTAATGCGTTACCATCGCTCGGAAGCCCGAGTAATTTACACAATAAATTGTACAAATGGCTTTAGCCATTTGTACCATTGT
TTAATAATTAGATTGACTGTCTAGTCATAGCATTGTTATGGCTTTATGGAAACCTGGCCCCCGGCAGGGTAGGAGCCTACATAATAGGCTCCTAA
GGTCTAAGGTCTAAGGTCTAAAATCTCGCTCTAGAATAGAATGCCCAATCTCCATATCCGTATATCACGTACTTATGATGAAATCAAACAGTGGA
TTGAAGCTGAAGCTTGTGAAGCTGTGGTTGCTTATGAACATGAAGCAGACGAAGATGTTAACCGCACTCATTGCCATATATGGGCAATTGGTATT
GGTAAAACCGATACCTTGAAAAATCACCTTAAAAAGGTGATAGGTTCTGTTGAATCAACAGACTGGTATTTTACAACGAAAAATAAATATAAAAA
GATTTGGACGAATGATGTTTTAACGTACATGAGTAAAGGGGTTTTGACACCCAAATATGTAAAAGGTTTCACAGATGAAACTATTGAAGACTATA
AGTCGAAATGGGTCTTTCCTGCCAATCCACTACATTGTGTTGATGGCAAGTTAGTTCTTGAGAGAGAAATAAAAGAAAGTAAGAAAAAAAACAAA
AAGAGAATTAATCGAAGAGATGAAATCTCTCTACTTGGAGTCAATGGAAGTTGACGAAGTTGTAAAACTTATTCGTAAAATATTGGTAAAAAAT
AACGAAGTTATAGGAATGTATAAAGTAATGGATTATTATGATTCGCTAATCATGTATGCCAATAAGCAACAATTCATAAATATGGTTGTTCAAAA
AATTAATTCTAGAGTTAGAGTATAAACATGCGTAAGTATGCCTCAAAGAAAAAGTCCTCCGGCCCAAAGAGACGACGAGTAGCTCGTCGTAAAC
CTCAACTGGTTGTAAACCGTGCTTTACATCCAATTCCTCAGCGATTCATTACCAAAATGAAATATGCTGAACAGTTTGTAACTGATGCTAATGGTA
ATTATCGTTTTAACCTTAATAGTGTGTACGATCCAAATCAGTCAGGTGTTGGCCATCAACCCTATGCTTTTGACACACTAGCTTCTCTTTATAACAG
ATATCGTGTCGTATCTTGTGGTTGGCGTATTCAGCCTGTCATTACTGGAACAACAGGTTCTCAGAATGTCCAGATTGCTGCTTTACCAGGTAAT

>000027F|arrow

CATCAATAAGACCCAATTGAATCGCTTCATCACGATTCTGGTCGTTCTCAAGGAACTCCAATAATTTGACAGGATCATGGTCAAATCGGGACTCTT
AATTTCGCTGGCATAGCCATGAAATCGTCCATAGTTGCGTTAATTTGATTCAACGCAGAATGGTAATCAGTAACACCACTAAAATCGCCGTATTG
AGGCGATACAGGGGCCGTTGGAAGTTCCCCTGTAACCCCGAAACGTTCAATGATGACATTAATGTCACATTCCATCTTTCATATGCTGTTGAGCA
AGACTTGGTCTTGACACAGCAACGCATCGTTTACCGATGCTTCATCTTTATCATAGTTGTACGGATTACGTACAAATGGCAAATTCGCTTTACTCA
TTATTTGACTCCAATTCCCCAAGGGGTTAGTTGATTAGGATTATTTTTGAGTTTATCAAAAAATTTCTTTCGAAGCATCGATACCCCTAGACCAAAT
AGCTGGGGATGGAGCCAATAGCCTCTTAGTATTATATGACTGAGCGGATGTTAAATCCGCAGTCGTATTCTTTAAAGCAATATCAGCCAAAATAC
GTTATTTTCGGCTGTAATATTAGAGCAGTAAGCAACTTATTAACAGTATCAGCCCTAGTATTAGCGTACCAGCTTCAGTAGCTTCAGTCTGGGCGA
TAATCTGCTTTTCTGTTTCTGATGCAGTACGAATCTGCTTTGCATCATAGAAGCAGTATTAATAGCTGAATTAGTTGCATTACCTAAAACATTTTCC
ATAGTAGCAGTTAGAACCAGATGGGGGTAGAAGCCCACCACGTTGATAGGCTAACATAGGAGATAAACCAGCAGGCTTCTTAAATCTTTAACAG
TCACGCTGGTAACTTGTTCCACTCATATCAGGCTTGAAAATCCATTTGCCGCGGAGCCCCCATCTCCTGATTAGCTTTATTAGCAGAAGTAGAAAC
CTAATACAGAACCAACAGCGCCCAAGAAGTACCGGACACCAGGAGTAAAGAACTAGAAGCTGAAGACAGCTTAGAACCACATTAGTACCCGCA
TCAGTATTCCACCAAACGCAAAACTACACGCCCTTCGCGTTGTTTCCTCATCTACTCCTTACGGAGTAGTTCCGAGGTTGATATAAAACATTAGAA
ATGATCAATTAAGACCAGGTACGCTGTACATAGGCATAGGTCTGGCCATCTGACAATCAAAGAAAGCATCCATTAAAAATTGGCTGACCATTTGC
TGCAGCTCCGACCGCCGTAGTACGAGCAACTGGAGGGGTCTCTTGAATAAACGTTGAATTCAACGTAGGAAGAGAATAAATCGCTGTCCATAAT
GCCAAGCATCAATAGTGCCAGCAGAAGTAGACCTAAAGAAACCAGTAATTTGAGAAGGTTTGTACGATACTCCGCCCAACGTTCTTGATAACCA
AATACATCATTGTCGGCTGCAGTACCTTGAACATAAATTTCTTTGTTCAAATAGCTTGCTCACCCAAGTGGCAAATACTGGGAAATAAAAGTCATA
TCGTGTAGACCTAGACCACATCTTAGGAAGACCCTGTTGATATGTGAGGTCAGCACGTACAGAAACTAATCCGATAATGTATCCATGTTCTTGAG
CAGCATACGTAAGCCGTGTCCCTGAGCCAATGCAGTACCCATTGCAGCAAGGTTACCTTGCGGAGTAGCAGAACCAGTAACCGACGTTGCAGAA
GTCTGAGCAATCGGATTAACATTAACAAGGGTCGAACCTCCACCAATATATTCAGGACGTTGTAAACGATAATCCTGTGGAGTTACTCCAAAGTG
AGCACGTAGTAACTCAGTATAACGAGTACCACCACGTGCATCGCGCTCCAATAACTTCTGAATCTGGAAAGATTGACGAAGTTGGTTAATAGTCG
CAGCAGTAGCAGTAGACAAATCAGCGTATAACTGATTAGTAGCAACACCAGCATTTGTACTATTAGACAAAGTATTAGAAGCTGAATTTAATTCT
CTTAAAGCACCAACAGCAGTTTGAAAAACACTATAACCAGCAGTACCATTGCCAGCTGGTATGTCAGAATAATATTAGCACGTGTTCCTAACGGT
AAAGAAACAGAAGCACCCTTCTGTGGCCAAGGCAATGCACCAGTAAAATAATCTTTACGCTTACCACGTCTCAAAAGTGTATAGTTAGCAGGAA
CATCACCTGAATCACCAAGATTTAACGTTACAGAATTCTGTAAGTTTTCATCTCTAAACCATTCGTTATAAATCTCATTATAAGCACGTAACGGCAA
AACATTATGCGTTACTGTATTAGCGCCAGTAATCTGACCAGCAGTAGGTAAACCAAAATAATCAAAAATTGAACAAACAGCATAACCACCAGCA
GGACTAGTAATAGTAGGCACTACAAAAGAAATAGAATCACCAGGATTCGTTTGTTCACCCATAAACTTTGGCCAATTGTTCCAAAGTAAACGATT
AGGTACAAAGAAAAAGAAAGTATCAAGATGCAAGTTGTCCATCACAGGAAATAACGGTGTGGCCAAACGTGCAAATGCAGTCATCTTTACACG
ATGAGTATCGCCAGGCAATACTTCATCACAATAAATAGGAACTAAAAAACCAGCATCAAACGTGGTTTTATGAGCATATTGAGTATTAAAACTAG
AGCGGGGAATGTCCGCTTTAGGAACCATAGCAATGAATGCGTACTTACTGACTTATTGCGAAACATAAACATCTCCCGTAGTTCCGTACCACTCT
TTCGAGTGATACGGTATAAAAAAAAACCTTACTCGCCTTCGCGAATCATTACATCTTTTGCACGAGCAATCAACTTAGGGCTTTCCAGTAGTTCAA
AAACACCAGTGGCATCGTCAAAAAGACCCAAATAGAACATATGAAAATCATCAGGATGTTTATACAACTGATTATCTTCGCTAGCTCGATTGACT
TCATCCTGAAACTGACGTACTGCAACACCTTCAGATGCAACATAAGCTGGACGACCAAAAGCATCTGCAGCAGTATCCTTAATAGAAACAATAAC
CATCTTCATAAAAACTCCTTAAATAGTACGTTTTAACAATGACAACTTAGCCAACGCAACTTTTCCTTAACAGCAAGTCGCTCAAGCGTGTTGTCCT

CATGCCTAGATCGACCTTCCATCTCTCTGGCAAACTGAATCATATCGAATTCTTCAGGAAACTTCAACTTAAATTTATTATCATAAAACCGTGGTG
GACGGCACTTTTTGCCACGCACCACAACGTGGTCTGACGTATAAACGTCTGACATGTACTTATCTAACCACGATTGCCCGATACCGGGCTTCAAT
GACATCTTATTAAATTCTGGCTTACGCTGATTATCTCACCAGTCTCTAAATCACAATATTGATAATGGCACCCGCATCAACCACTTCGTGGTTTCAT
TGACAGTAACCCCATTAATCTTCTTCATATATATCTTGCAACATAAGCAGCAGACTCAAAAGTAACATCACCAATTGTAGAATAGCCAAACGGCCA
CAATTCTTCCAAATCTCTGACGTGTAGAGGATAGAGCCAGTCTGCGTTCTTTTAAATATTTTCTTATCCGGAAAATCAAGACCAAACAGACAAGCA
TGGAAATGAGGACGATCAAAAGATTCACCATATTCACCTGCCATATAAAAACGTATCGTTTTCCAGTAAAACGCTTACGTAACCGCTTCATAAAA
AGCTGATAATCATTGTAATCCAATGACATATCCTTAGGACAATGCTCTGGAGCATATGTCAAAGTAATAAACAATTACTAGTATGCATTTGTGCCT
CATGCATACAACGAATCGCCCACTGACGTGAGCGTTCAAGGCGACAACCAACACACTGACCACAAGGCAATGATAGGGTACGGACTACATCCGC
ACCCGGTATTTCCCGCCAAATTATAGACCTGTCACTGCATTGATAAGCCGTTAAGGGCTTATAACAGGCCATAATTACAAACGATAGCCACCACG
CTGGGGAGCGGTCTCATATTAATTGGACTTCGTCTTACTAGCATTCTGCGAAATGACTTTGCAGATTTATATTTGTTTACTGGCTTTCTTCGTAACA
TGATGAACTCCGTAGTTAAAATAGTGGTTTGGTGTCACCTAGCACAGTTACATCAAGTAGAGTAACTGTGCGGGCCTCAGATTTCATCCTTCGGC
CTTAGGTGTTTCTGTAGAAACGATGGGTTCAACCACAGGTTGTC

>000106F|arrow

TATCTAAATGCAAATTGTCCATAACTGGAAACAAAGGCGTTGCCAAACGGGCAAACATTGTAGCCTTTACATTGTGCATGTCCCCTGGGAGCACT
TCATCACAATAAATAGGAACTAGATAACCACCATCAAAAGTAGTTTTATGCGCATATTGCGTATCAAAACTAGAACGCGGAATTTCCGCTTTAGG
AACCATAGCAAAACTATGTGAGCTTACTGACTTATTACGATGCATAACAATCTCCCGAAGTATTCCGAACCACTAGCAAGCTAGTGATCCGGCTT
AAAAAAAACATTACTCGCCATCGCGAATCATAACATCCTTAGCTCTAGAAATAAGCTTGGGAGAACCAAGCAAATCCATAGTACCAGAATTATCA
TCAAACGTACCAAAATAATATAACTGAAAATCATCAGGGTGTTTATATAACTGATTATCATCGCTAGCGCGATTAACTTCGTCCTGAAACTGACGA
ACAGCAACACCCTCAGATGCAACAAAAGCTGGACGACCATACGCACCAGCTGCAGTATCTAAAATAGAAACAATAACCATCTTCATAAAAACTCC
TTTAAATCTTACGTTTTAAAAGCGATAACTTAGCCAAAGCGACTTTTTCCTTTACAGCCAAACGCTCTAAAGTGTTATCTTCAAAATGTGAGCGAC
CTTCTAGCTCACGAGCATATTGTATACCATCAAATTCTTCTGGAAACAATACTTTAAACTTATTATCATAAAACCGTGGTGGTCGGCACTTTTTGCC
ACGCACCACAACAGAGTCAGTCGTATAAACGTCTGACATGAACTTATCAAACCAAGCCTGACCAATGCCAGGCTTAAGAGACATCTTATTAAATT
CAGGCTTACGCTGAATAATCTCACCAGTATCTAAATCACAATACTGATAATGCGCATCTGCATCAACCACTTCGTGGTTCTCATTAACGGTTTTACC
GTTAATCTTCTCATAATATATCGAGCAACATAAGCAGCAGATTCAAAATTGACATCACCAATAGAACTATAGCCATACGGCCAAAGTTCTTCAAGT
ATCTTTGACGTATATAAGATAGACCCAGTCTGCGTTCTTTTGAAAAACTTCTTATCTTCAAAATCAAGCCCAAAGATACAAGCATGGAAATGAGG
ACGATCGAAAGACTCACCATATTCACCTGCCATATAAAAACGAATAGTCTTGCCAGTATAGCGTTTTCTCAATCGTTTCATAAACAATTGAAAATC
TTCATAATGCAAAGACATATCCTTAGGACAATGCTCTGGAGCATATGTCAAAGTAATAAAACAATTACTAGTATGCATTTGTGCCTCATGCATACA
TCTAACGGCCCACTGACGGGACCGTTCAAGGCGACAACCAACACACTGACCACAAGGCAATGACAAGGTACGGACTACGTCCGCCCCTGGTATC
TCCCGCCAAATAATAGACTTGTCAGCGCATTGATAAGCCGTTAACGGCTTATAACACGCCATAAATTACAGTCTAAAACCACCGCGCTGCGGTGA
AGTACGCATATTAATGCTCTTGGTCTTGCTTACGCCACGACGAAACTTCTTAGCTGCGCCATGCTTGCTCATTGGTTTTCTATAAAGGCTCATAACA
TTGCACTCCGTAGTTAATAATGTGGTTTTGGTGTCACCTAGCACAGTTACATCAAGTAGAGTAACTGTGCTGCCATCCGCTTACGCGTCTGGCTTA
GGTGTTTCTACTGCAGAAACGACGGGTTCAACCACAGGTTTACCGTCAATAAGACCAATCTGAATCGCTTCATCACGATTCTCTTCATTCTGTAAA
TAATTTAACAAAGCATTAGGATCGTTATCAAACTTAGCCCTAATCTTAGCTGGCAAAGCCATAAAAGCCTCATCAGAGGCACGAATAGCATTCAA
TGCGGTGTGATAGTCAGATACACCGCTAAAATCGCCATATGATGGCTCTAATGGCGCCTGGGGCAATTGCCCAGTAACGCCAAAACGCTCAACT
ATAATATTAATATCACATTCGTCTCTCATGTGTTGTTGAGCCAAACTCGGATCTTTACATTCAAGACCAGTCTCTTGTGAAACAAGAGCCATATCAT
AATTGTACGGATTACGTACAAAAGGTAAATTCGTTTTACTCATTTTCTACGACCTTCCAAGGACCAAGGAAAAGACTTATTCTTTTCCAAAAACTT
ATCAAAAATAGACTACTACCTTTCTTAATATCGCGATACCAATATGGATCGCTAGACGGTGTTCGTAACTCTAAATTATTCTTAGCATCAATATCA
GTCTTAATTGTTGATGCAGAATTAGCATTAACACGAGAACCAGCCTCGGCCTGTTCCAAATAAAAACGAGCCGGTTCTTGTAAAACACGCTGAGA
AGCTAAATCAGCTTCTTCAGCAACCTTCCTAGCAGTCTGAACATTTAATTCACGTTGAGATTTAGCAACTTCCATCTGCTCACGCAATAAATCACTC
TCAACATCACGCTTAACAGCTCCACTCAAATCAGACTTAACTGGAGAAGTAGAAGATGCAGTAGCACCACTCGGAACAGTAGAACCACCTTTAG
AATATGCAAGCATAGGACTCAAACCAGCCTTATTCAAATCAGTAACCATACGCTGATATTGCGTATTGGACATATCTTCTTGAAAGCGTCTATTTG
CTTGCGCTTCAGCAGTATTATATTTTTGCTCTTCATTCTGACTCCAAGCAGAAGTAGCAAAATCAAATAAACCACCAGCAACTGAATCAAACATAC
CCATACTAACGCGCTCCGCTTGTTTGCTGACTACTGGTTTCCCAGTAGTCCAGCTTATATTACATTAGAAATGGTCAATCAAGCCAGGTACAGAAT
ACATCGGCATTGGACGAGCCATCTTACAATCAAAAAACGCATCCATCAAAAACTGCTGACCATTAGCAGATGCACCAACAGCAGTAGTACGATC
AATAGGTGGCGTTTCTTGAATAAACGTAGAATTCAAAGTCGGCAACGAAGTGAACTTCTGAGCATAATGCCAAGGGTCAATCGTACCAGCTGAA
GTCGACTTAAACAAACCTGTAATCTGAGAAGGTTTGTAACGGTACTCTGCCCAACGTTCCTGGTATCCAAATACATCATCATCAGTTGAAGTACCA
GTAACATAGATTTCCTTATTAAGAACAGCCTGTTCACCTAAATGAGCAAATACAGGGAAATAGAAATCATAACGTGTCTCACGAGACCACATCTT
AGGTAAACCTTGCTGATATGTTAGATCAGCTCTTACGTTTACCAAACCAATTATGTATCCATGTTCTTGAGCATGATACGTAAAACCATGTCCACT
AGCCAACGCAGTACCCATTGCAGCCAAGTTACCAAGCGGAGTAGCACCGCCAGAAATCGAAGTAGCAGACGTTTGAGCAATAGGATTAACGTT
GACATAAGTAGAACCACCACCAATATATTCAGGACGTTGTAAACGATAATCTTGTGGAGTTACACCAAAATGAGCACGTAACAATTCTGTATAGC
GTGTACCACCTCGCGCATCGCGCTCTAACAAACGCTGAATCTGGAAAGACTGACGTAACTGGTTAATAGTTGCAGCCGTAGCATCACTTAAATCG

GCATACAAACCAGTACCAGCAGTACCAGCGTTATTACTACGATACACAGCATGTGTAGTTGAATTAGCATAAATCTGCTTCAAAGCACCAGCACC
GTCAACTAATGACAACGTTGTTGAATCATTAGTAACAGACGTCTTAATAGGAGCAGACGTGCCTAAAGGCAACGTAACTGCATCGCCTTTCTGAG
GCCAAGGCAAGGCACCAGTAAAATAATCCTTACGCTTACCGCGTCGAACCATAGCATAATCACTCGGAGTATCAGGACCGTCACCCTTGTGAAC
GGTAACAGAATTTTGTAAATTCTCATCCCTAAACCACTCGTTATAAATCAAATTATAAGCACGTAACGGTAACGCGTTATGCGTAACCGTATTAGT
ACCAGTAATCTGACCAGCCGTAGGCAAACCAAAATGATCAAAAATAGAACCTACTGCATAACCACCAGCAGTAGAAGTAATCTGTGGAACTACA
TACGAATAGAATCACCTGGGTTCGCTTGCTCACCCATAAACTTAACCCAATTGTTCCAAACTAATCTTGGAACAAAGAAAAAGAAAG

>000164F|arrow

TTGATAAGTAAGATCTGCTCTAATGGATACTAATCCAATAATAACGCCATGCTCAGTAAATGATTGAGTAAATCCATGATTATGAGCGAGAGCAG
TACCCATAGCAGCAAGGTTGCCCAAAGGGGTAGTCGTTCCAGAAGCGTTTGTTCCCGACGTTTGAGCAATCGGATTAACATTAATTGGTGTTGAA
CCGCCTCCAAGGTATTCAGGCCTTTGTAAACGGGCGTCTGGGGAAATAACTCCAAAGTGACTCCGGATAATTTCTGTGTATCGTGTTCCGCCACG
TGCATCCCTTTCAAGTAATTTTTGAATTTGAAATGACTGTCTTAATTGATTGACAGTTGCAGCAGTTGCTTCTGATAAGTCGGTATATAAATTAGA
AACGTTATTTACTACACCAGCAGTATTAACACCATAAGCGTTGCCATATCTAGCTAAAGCAGTCGTATTTCCAGGATCTGTTTGAATTACCGTAAA
TTTATCGTTTGATGTTGCGTCTCCTGAAATGGTATCCCATTTAATTGGAGCCGTAGTACCTAAAGGTAAGGTGACACTCGCACCTTTTTGTGGCCA
TGGTAATGCTGACGTAAAGTAATCATGTCTTTTACCACGACGTTTTAGCACATAGTTTGAGGAAGTATCAGGGCCATCGCCCTTATCAACTACTGC
GCTTGTTTGTAAATTTTCATCTCGGAACCATTCGTTCCAGATAAGATTGTATGCACGTGGCCAAAAGGCACAGTGCGTAATAGTTCGGCCAGTAT
CAATTTGGCCTACTGTTGGTAAGCCCATATAGTCTTGAAGGCTGCCTACGGCATAACCATCTGTTGGGCTTGTTTGTGTTGGGACAATATAAGAT
ATTGAGTCTGTTGGATTTTCTTGTTGACCCATAAATTTTTGCCAGTTATTCCATATAAGGCGATTGGGTACAAAGAAAAAGAAAGAATCCATAATC
ATGTTATCCATGATTGGATATAAAGGCGTTGCTAGACGGGCAAATGCCGTCATTTTTAAGTTGAAAGTGTCCCCAGGGAGCACTTCATCAACATA
TACAGGAATTAGATAGCCCGCATCGAAAGTCGTTTATGTGTTTTTTGAGCATCGAATTTACTACGTGGTATATCGGCTCTAGGTACCATCGCGAA
GCGGTGAGTATTAACTGACTGATTGCGGTGCATGTTTTTCCTTAGTGTTGTTCCGGGGGAAAGATAAATCTCTTTTCCCCTCGGTTGTTTTATTTA
AGTTTAACTTGTTTTCCTAATGATAGGAGTTTTGGTTGTTCATGTAAATCGAATAACCCAGTTGAATCGTCAAATGTTCCGAATTCATATAGATCG
AAATCATCAGGGTGATTAAAGAGTTGATTTTCAGTATCAGAACGATTAATTTCATCTGAAAAAGAGCGTATAGCTACTCCAGAGGAAGGTACGA
ACATTGGTCGTGCATATGCTTCAGCAGCACGGTCTTTTACGGAAGCGAGGATAAGTTTCATTATTTTCCTAAGTAAGGTTACGTTTTAATAGTTGA
AGTTTTGCCATAGTGACTTGTTCTTTTGCAGATAGTCGTTCTGGTGTATTGTCTTCGGAATTAAATTTAGCATTATTTTCCCGCATGTAAAGTAATT
CGTCATACTCATAAGGTTGGTCAATTTTAAACATTTTGTCATAGTATTTTGGTGGTTTGACCTTTTTACCTCTAAGTATTACGTAGTCTTGCGGGTA
TATATCCGAAGTATATTTTATATAAAAGTCTTTACCGATTCCCGGTTTTAAAGACATTTTATTATATTCCGGCTTTAAGTCTAAATATTCGCCGGTTT
CAGGGTGTATGCGTTTGTAATGAGATTCCGCATCTTTCCCTGTTTGTTTTTTCATTATGTATCTAGCCACGTAGGCGGCTGATTCAAAAGTAACAT
CTCCAATGGTGGTATAACCAAATGGCCAGAGAGCTTCAAGTTCTGCGGATCTATATAACATAGAACCAGAGGCAGTCCTTTTCCATAATTTTTTAT
CAGGAAAGTCGTATCCGAAGATACAGGCATGGAAGTGGGGTCTTGCGAAGAGTTCACCATATTCTCCAGCCATGTAATAGCGGATTGTAAGTCC
TCTTTTTGCGAGAGTTTTTCTAAGTCTTTTAAGGAACAATTGAAAGTCTTTGTGATCCAAAGAGCCATCGCTTGGGAGATGTGTATTGTCATATGT
GAGTGTTATGAATGAGTTGTTTTTATGCAATTGGGCTTCGTGCATGCACCGAATAGCCCATTGTCTTGATCGTTCTAGACGGCATCCAACGCACT
GCCCACAGGGCAGGTCTAAAGATCGAACGATGTCATGTTTCCGAACTTCGTCGAAAACAATTGATTTGTCAAAGCATTGATATGCTTTGAGAGG
GTGATAACAAGGCATGTGAGGTGCCTGAAGACTTTATTAGAGTCTCCAGCCTCCACGCATTGGCGAGTTTCGCATATTTGCGTGTTTTGTATGTG
AAGCATGTTTACGGAAAGTCCTAGCGGACTTTTGCTTATTTGCTGGTCTGCGACGCATCATTTTTTCTGTCCTTGTTTATCGTGTTTTTGTGGTTTG
GTGTCACCTAGCACAGTTACATCAAGTAGGTAACTGTGCTTCCGAGGTTCCAACAGGGGTTGAAACCTCGGTTTGGGCTGGTTTTACCAGTCCCA
TTTTTTCAGCTTCGCTGCGATTTTCAGGGTTTTCTAGAAAATCTATTAGATTCGCGGGATCATTAGCGAATCGTTCACGAATATTGGCTGGTAAAG
CCATAAATTCGTTGTCTGAAGCCATAATCTGGTTCAGAGCAGAGTGGTAGTCATAGACACCACTAAAGTCGCCATATTGAGGCGTTAAAGGAGT
TTGAGGAATAAGTCCGGTCATACCGAACTTTTCCATAATATTATTGATATCACATTCTTCAGCAAATTGCTGCTGAGTCAGAGTTGCATCCTCACA
ATGCAGCCCTGACTCATTTGACGCAGCAATCGTGTCGTAATTGTACGGAGTACGAAGAAATGGGGCTGTAATCTTTGTCATTTTCATTCCATTGG
TGGTTGGTTATTTGTACGATTTTGTTGAATCGGTTGACCTCGATAGTTATCGATGTATCGTTTTGCGCCAGATGCGCTATACGCATCTTTGGCGAT
GTTTTTTACGTCTTGATAAATTGGTTTGCTAGAACTAGGAGCTGACCCTGATTTAGCAAGTTTTGTTAACTCGTTTGAATAACGAGATTGTGCCAA
AGCTTGAGCAGCTTGAGCACTTGATGTTTTGCCTATTTCATTTTTCATGAAAGTATCGGCAAGTATTTGTTTATATTGAGCTCTAATATTTGGATTT
TCATCCAATTTATTTAACGTATCAGCACGTACATTATCTGTTTGATTACTTGTGAGTTCTGTTTGAGCTTCAATTTGTTTAGATTGAGCAATAGCTT
GATTTGCTTGCGCAACCGTTTGATAAGCTTGGGTTCCGGACGTAGTTGCATTACCGAGAACATTTTGCATCTGGGCCATAGCTCCAGCTGGTGTT
GTGGCTCCGCCTTGTGAATACGCAAGCATGGGATTTAACCCAGCTTTTTTCATATCTTCAACTGCTCGTTGATATGATGTTCCAGACATTTCAGCTT
GAAATCTCTGTTGATTACTCGCTTGTTCTGCACTCGCTGCGTTTTGACTTTGTGTTCCAAAGTATTGTCCTGCTGCACCTATTGCTGCAGGTGCAAG
TGCAGCTAAGGAGAGTCCCCCAGTAGCAGGGGCAGCTCCTATAGCTATAGCAGGGCCGATTAAATCGGCAATTCCGTCAAATAGTCCCATTAGA
AATGGTCTATTAAGCCAGGTACTGAATACATTGGCATTGGTCTTGCCATTTTGACATCAAAAAATGAGTCAAATAAGAATTGTTGGCCATTTGCA
GCTGCTCCAACGGCTACTACACGTGATACAGGAGGCGTATCTTGAATAAACGTATTATTCAAAGTAGGCGCAGCGGTAAATTTTTGAGCCAAAT
GCCAACCATCGATCGTGCCCGCCGATGTTGATTTGAACAAACCAGTAATTTTAGATGGTTTGTAACGATATTCCGCCCAGCGTTCTTGATATCCAA

AAACAGTATTGTCGGCTGCAGTATCTCCTGTTGCATAAATTTCTTTTTGCAAAACAGATTGTTCGCCTAGAGTAGCAAATGCTGGGAAATAAAAG
TCATATCGTGTAGATCTAGACCACATACGGTCTAATCCTTG

>000116F|arrow

GGAACTTCCAACGGCCCCTGTATCGCCTCAATACGGCGATTTTAGTGGTGTTACTGATTACCATTCTGCGTTGAATCAAATTAACGCAACTATGGA
CGATTTCATGGCTCTGCCAGCGAAATTAAGAGTCCGATTTGACCATGATCCTGTCAAATTATTGGAGTTCCTTGAGAACGACCAGAATCGTGATG
AAGCGATTCAATTGGGTCTTATTGATGGACAACCTGTGGTTGAACCCATCGTTTCTACAGAAACACCTAAGGCCGAAGGATGAAATCCTGAGGC
CAGCACAGTTACTCTACTTGATGTAACTGTGCTAGGTGACACCAAACCACTATTTTAACTACGGAGTTCATCATGTTACGAAGAAAGCCAGTAAA
CAAATATAAATCTGCAAAGTCATTTCGCAGAACTGCTAGTAAGACGAAGTCATTAATATGAGACACGCTCCCCAGCGTGGTGGCTATCGTTTGTA
ATTATGGCCTGTTATAAGCCCTTAACGGCTTATATCATGCAGTGACAGTCTATAATTTGGCGGGAAATACCGGGTGCGGATGTAGTCCGTACCCT
ATCATTGCCTTGTGGTCAGTGTTTGGTTGTCGCCTTGAACGCTCACGTCAGTGGGCGATTCGTTGTATGCATGAGGCACAAATGCATACTAGTAA
TTGTTTTATTACTTTGACATATGCTCCAGAGCATTGTCCTAAGGATATGTCATTGGATTACAATGATTATCAGCTTTTTATGAAGCGGTTACGTAAG
CGTTTTACTGGAAAACGATACGTTTTTATATGGCAGGTGAATATGGTGAATCTTTTGATCGTCCTCATTTCCATGCTTGTCTGTTTGGTCTTGATTT
TCCGGATAAGAAAATATTTAAAAGAACGCAGACTGGCTCTATCCTCTACACGTCAGAGATTTTGGAAGAATTGTGGCCGTTTGGCTATTCTACAA
TTGGTGATGTTACTTTTGAGTCTGCTGCTTATGTTGCAAGATATATTATGAAGAATTAATGGGGTTACTGTCAAAACCACGAAGTGGTTGATGCG
GGTGCCCATTATCAATATTGTGATTTAGAGACTGGTGAGATAATTCAGCGTAAGCCAGAATTTAATAAGATGTCATTGAAGCCCGGTATCGGGC
AATCGTGGTTAGATAAGTACATGTCAGACGTTTATACGTCAGACCACGTTTGGTGCGTGGCAAAAAGTGCCGTCCACCACGGTTTTATGATAATA
AATTTAAGTTGAAGTTTCCTGAAGAATTCGATATGATTCAGTTTGCCAGAGAGATGGAAGGTCGATCTAGGCATGAGGACAACACGCTTGAGCG
ACTTGCTGTTAAGGAAAAAGTTGCGTTGGCTAAGTTGTCATTGTTAAAACGTACTATTTAAGGAGTTTTTATGAAGATGGTTATTGTTTCTATTAA
GGATACTGCTGCAGATGCTTTTGGTCGTCCAGCTTATGTTGCATCTGAAGGTGTTGCAGTACGTCAGTTTCAGGATGAAGTCAATCGAGCTAGCG
AAGATAATCAGTTGTATAAACATCCTGATGATTTTCATATGTTCTATTTGGGTCTTTTTGACGATGCCACTGGTGTTTTTGAACTACTGGAAAGCCC
TAAGTTGATTGCTCGTGCAAAAGATGTAATGATTCGCGAAGGCGAGTAAGGTTTTTTTATACCGTATCACTCGAAAGAGTGGTACGGAACTAC
GGAGATGTTTATGTTTCGCAATAAGTCAGTAAGTACGCATTCATTTGCTATGGTTCCTAAAGCGGACATTCCCCGCTCTAGTTTTAATACTCATAT
GCTCATAAAACGTTTGATGCTGGTTTTTTAGTTCCTATTTATTGTGATGAAGTATTGCCTGGCGATACTCATCGTGTAAAGATGACTGCATTTGCA
CGTTTGGCCACACCGTTATTTCCTGTGATGGACAACTTGCATCTTGATACTTTCTTTTTCTTTGTACCTAATCGTTTACTTTGGAACAATTGGCCAAA
GTTTATGGGTGAACAAACGAATCCTGGTGATTCTATTTCTTTTGTAGTGCCTACTATTACTAGTCCTGCTGGTGGTTATGCTGTTTGTTCAATTTTT
GATTATTTTGGTTTACCTACTGCTGGTCAGATTACTGGCGCTAATACAGTAACGCATAATGTTTTGCCGTTACGTGCTTATAATGAGATTTATAAC
GAATGGTTTAGAGATGAAAACTTACAGAATTCTGTAACGTTAAATCTTGGTGATTCAGGTGATGTTCCTGCTAACTATACACTTTTGAGACGTGG
TAAGCGTAAAGATTATTTTACTGGTGCATTGCCTTGGCCACAGAAGGGTGCTTCTGTTTCTTTACCCGTTAGGACACGTGCTAAATTTAGTCTGAC
ATACCAGCTGCAATGTACTGCTGTTATAGTGTTTTTCAAACTGCTGTTGGTGCTTTAAGAGAATTAAATTCAGCTTCTAATACTTTGTCTAATAGTA
CAAATGCTGGTGTTGCTACGAATCATTTATACGCTGATGTGTCTACTGCTACTGCTGCGACTATGAACCAACTTCGTCAATCTTTCCAGATTCAGA
AGTTATTGGAGCGCGATGCACGTGTGGTACTCGTTTATACTGAGTTACTACGTGCTCACTTTGGAGTAACTCCACAGGATTAGCGTTTACAACGT
CCTGAATATATTGGTGGAGGTTCGACCCTTGTTAATGTTAATCCGATTGCTCAGACTTCTGCAACGTCGGTTAGCTGGTTCTGCTACACTCCGCAA
GGTAACCATTGCTGCAATGGGTATGCATTGCTCAGGGACAACGGCTTTACGTATGCTGCTCAAGAACATGGATACATTATCGGATTAGTTCTGTA
CGTGCTGACCTCACATATCAACAGGGTCTTCCTAAGATGTGGTCTAGGGTCTACACGATATGACTTTTATTTCCCAGTATTTGCCACTTTGGGTGA
GCAAGCTATTTTGAACAAAGAAATTTATGTTCAAGGTACTGCAGCCGACAATGATGTATTTGGTTATCAAGAACGTTGGGCGGAGTATCGTTACA
AACCTTCTCAAATTACTGGTTTCTTTAGGTCTACTTCTGCTGGCACTATTGATGCTTGGCATTATGGGACAGCGATTTACTTCTCTTCCTACGTTGA
ATTCAACGTTTATTCAAGAGACCCCTCCAGTTGCTCGTACTACGGCGGTCGGAGCTGCAGCAAATGGTCAGCAATTTTTAATGGATGCTTTCTTTG
ATTGTCAGATGGCCAGACCTATGCCTATGTACAGCGTACCTGGTCTAATTGATCATTTTCTAATGTTTTATATAACCTCGACTACTCCGTAAGGAG
TAGTGAGGAAACAACCGAAGGGCGTAGTTTATGTTTGGTGGAATACTTGATGCGGTTACTAATGTTGGTTCTAAGCTGTCTTCAGCTTCTAGTTT
CTTTACTCCTGGTGTCGGTACTGGCTGTGGGCGCTGTTGGTTCTTATTTAGGTTCTACTTCTGCTAATAAAGCTAATCAGGAGATGGCTCAGAGG
CAAATGGATTTTCAAGCCGATATGAGTGGAACAAGTTACCAGCGTGCTGTTAAAGATTTAGAAGCTGCTGGTTTATCTCCTATGTTAGCCTATCA
ACGTGGTGGTGCTTCTACCCCATCTGGTTCAACTGCTACTATGGGAAAATGTTTTAGGTAATGCAACTAATTCAGCTATTAATACTGCTTCTATGA
TGCAACAGATTCGTAATGCATCAGAAACAGAAAAGCAGATTATCGCCCAGACTGAAGCTACTGAAGCTGGTACCGCTAATACTAGGGCTGATAC
TGTTAATAAGTTGCTTACTGCTCCTAATATTACAGCCGAAAATAAACGTATTTTGGCTGATATTGCTTTAAAGAATACGACTGCGGATTTAACATC
CGCTCAGTCATATAATACTAAGAGGCTATTGGCTCCATCCCCAGCTATTGGTCTAGGGGTATCGATGCTTCGAAAGAAATTTTTGATAAACTCAA
AAATAATCCTAATCAACTAACCCCTTGGGGAATTGGAGTCAAATAATGAGTAAAGCGAATTTGCCATTTGTACGTAATCCGTACAACTATGATAA
AGATGAAGCATCGGTAAACGATGCGTTGCTGTGTCAAGACCCAAGTCTTGCTCAACAGCATATGAAAGATGAATGTGACATTAATGTCATCATT
GAACGTTTCGGGGTTACAGG

>000094F|arrow

GCAAATGAATGCGTACTTACTGACTTATTGCGAAACATAAACATCTCCGTAGTTCCGTACCACTCTTTCGAGTGATACGGTATAAAAAAAAACCTT
ACTCGCCTTCGCGAATCATTACATCTTTTGCACGAGCAATCAACTTAGGGCTTTCCAGTAGTTCAAAAACACCAGTGGGCATCGTCAAAAAGACC
CAAATAGAACATATGAAAATCATCAGGATGTTTATACAACTGATTATCTTCGCTAGCTCGATTGACTTCATCCTGAAACTGACGTACTGCAACACC
TTCAGATGCAACATAAGCTGGACGACCAAAAGCATCTGCAGCAGTATCCTTAATAGAAACAATAACCATCTTCATAAAAACTCCTAAATAGTACG
TTTTAACAATGACACTTAGCCAACGCAACTTTTTCCTTAACAGCAAGTCGCTCAAGCGTGTTGTCCTCATGCCTAGATCGACCTTCCATCTCTCTGG
CAAACTGAATCATATCGAATTCTTCAGGAAACTTCAACTTAAATTTATTATCATAAAACCGTGGTGGACGGCACTTTTGCCACGCACCACAACGTG
GTCTGACGTATAAACGTCTGACATGTACTTATCTAACCACGATTGCCCGATACCGGGCTTCAATGACATCTTATTAAATTCTGGCTTACGCTGAAT
TATCTCACCAGTCTCTAAATCACAATATTGATAATGGGGCACCCGCATCAACCACTTCGTGGTTTTCATTGACAGTAACCCCATTAATCTTCTTCAT
AATATATCTTGCAACATAAGCAGCAGACTCAAAAGTAACATCACCAATTGTAGAATAGCCAAACGGCCACAATTCTTCCAAAATCTCTGACGTGT
AGAGGATAGAGCCAGTCTGCGTTCTTTTAAATATTTTCTTATCCGGAAAATCAAGACCAAACAGACAAGCATGGAAATGAGGACGATCAAAAGA
TTCACCATATTCACCTGCCATATAAAAACGTATCGTTTTCCCAGTAAAACGCTTACGTAACCGCTTCATAAAAAGCTGATAATCATTGTAATCCAAT
GACATATCCTTAGGACAATGCTCTGGAGCATATGTCAAAGTAATAAAACAATTACTAGTATGCATTTGTGCCTCATGCATACAACGAATCGCCCA
CTGACGTGAGCGTTCAAGGCGACAACCAACACACTGACCACAAGGCAATGATAGGGTACGGACTACATCCGCACCCGGTATTTCCCGCCAAATT
ATAGACCTGTCACTGCATTGATAAGCCGTTAAGGGCTTATAACAGGCCATAATTACAAACGATAGCCACCACGCTGGGGAGCGTGTCTCATATTA
ATTGACTTCGTCTTACTAGCAGTTCTGCGAAATGACTTTGCAGATTTATATTTGTTTACTGGCTTTCTTCGTAACATGATGAACTCCGTAGTTAAAA
TAGTGGTTTGGTGTCACCTAGCACAGTTACATCAAGTAGAGTAACTGTGCTGGCCTCAGGATTTCATCCTTCGGCCTTAGGTGTTTCTGTAGAAA
CGATGGGTTCAACCACAGGTTGTCCATCAATAAGACCCAATTGAATCGCTTCATCACGATTCTGGTCGTTCTCAAGGAACTCCAATAATTTGACAG
GATCATGGTCAAATCGGACTCTTAATTTCGCTGGCAGAGCCATGAAATCGTCCATAGTTGCGTTAATTTGATTCAACGCAGAATGGTAATCAGTA
ACACCACTAAAATCGCCGTATTGAGGCGATACAGGGGCCGTTGGAAGTTCCCCTGTAACCCCGAAACGTTCAATGATGACATTAATGTCACATTC
ATCTTTCATATGCTGTTGAGCAAGACTTGGGTCTTGACACAGCAACGCATCGTTTACCGATGCTTCATCTTTATCATAGTTGTACGGATTACGTAC
AAATGGCAAATTCGCTTTACTCATTATTTGACTCCAATTCCCAAGGGGTTAGTTGATTAGGATTATTTTTGAGTTTATCAAAAATTTCTTTCGAAGC
ATCGATACCCCTAGACCAAATAGCTGGGGATGGAGCCAATAGCCTCTTAGTATTATATGACTGAGCGGATGTTAAATCCGCAGTCGTATTCTTTA
AAGCAATATCAGCCAAAATACGTTTATTTTCGGCTGTAATATTAGGAGCAGTAAGCAACTTATTAACAGTATCAGCCCTAGTATTAGCGGTACCA
GCTTCAGTAGCTTCAGTCTGGGCGATAATCTGCTTTTCTGTTTCTGATGCATTACGAATCTGTTGCATCATAGAAGCAGTATTAATAGCTGAATTA
GTTGCATTACCTAAAAACATTTTCCATAGTAGCAGTTGAACCAGATGGGGTAGAAGCACCACCACGTTGATAGGCTAACATAGGAGATAAACCA
GCAGCTTCTAAATCTTTAACAGCACGCTGGTAACTTGTTCCACTCATATCGGCTTGAAAATCCATTTGCCTCTGAGCCATCTCCTGATTAGCTTTAT
TAGCAGAAGTAGAACCTAAATAAGAACCAACAGCGCCCAAAGCAGTACCGACACCAGGAGTAAAGAAACTAGAAGCTGAAGACAGCTTAGAAC
CAACATTAGTAACCGCATCAAGTATTCCACCAAACATAAACTAACGCCCTTCGGTTGTTTCCTCACTACTCCTTACGGAGTAGTCGAGGTTATATA
AAACATTAGAAATGATCAATTAGACCAGGTACGCTGTACATAGGCATAGGTCTGGCCATCTGACAATCAAAGAAAGCATCCATTAAAAATTGCT
GACCATTTGCTGCAGCTCCGACCGCCGTAGTACGAGCAACTGGAGGGGTCTCTTGAATAAACGTTGAATTCAACGTAGGAAGAGAAGTAAATCG
CTGTCCATAATGCCAAGCATCATAGTGCCAGCAGAAGTAGACCTAAAGAAACCAGTAATTTGAGAAGGTTTGTAACGATACTCCGCCCAACGTTC
TTGATAACCAAATACATCATTGTCGGCTGCAGTACCTTGAACATAAATTTCTTTGTTCAAAATAGCTTGCTCACCCAAAGTGGCAAATACTGGGAA
ATAAAAGTCATATCGTGTAGACCTAGACCACATCTTAGGAAGACCCTGTTGATATGTGAGGTCAGCACGTACAGAAACTAATCCGATAATGTATC
CATGTTCTTGAGCAGCATACGTAAAGCCGTGTCCCTGAGCCAATGCAGTACCCATTGCAGCAAGGTTACCTTGCGGAGTAGCAGAACCAGTAAC
CGACGTTGCAGAAGTCTGAGCAATCGGATTAACATTAACAAGGGTCGAACCTCCACCAATATATTCAGGACGTTGTAACGATAATCCTGTGGAG
TTACTCCAAAGTGAGCACGTAGTAACTCAGTATAACGAGTACCACCACGTGCATCGCGCTCCAATAACTTCTGAATCTGGAAAGATTGACGAAGT
TGGTTAATAGTCGCAGCAGTAGCAGTAGACAAATCAGCGTATAACTGATTAGTAGCAACACCAGCATTTGTACTATTAGACAAAGTATTAGAAG
CTGAATTTAATTCTCTTAAAGCACCAACAGCAGTTTGAAAAACACTATAACCAGCAGTACCATTGCCAGCTGGTATGTCAGAATAAATATTAGCAC
GTGTTCCTAACGGTAAAGAAACAGAAGCACCCTTCTGTGGCCAAGGCAATGCACCAGTAAAATAATCTTTACGCTTACCACGTCTCAAAAGTGTA
TAGTTAGCAGGAACATCACCTGAATCACCAAGATTTAACGTTACAGAATTCTGTAAGTTTTCATCTCTAAACCATTCGTTATAAATCTCATTATAAG
CACGTAACGGCAAAACATTATGCGTTACTGTATTAGCGCCAGTAATCTGACCAGCAGTAGGTAAACCAAAATAATCAAAAATTGAACAAACAGC
ATAACCACCAGCAGGACTAGTAATAGTAGGCACTACAAAAGAAATAGAATCACCAGGATTCGTTTGTTCACCCATAAACTTTGGCCAATTGTTCC
AAAGTAAACGATTAGGTACAAAGAAAAAGAAAGTATCAAGATGCAAGTTGTCCATCACAGGAAATAACGGTGTGGCCAAACGTGCAAATGCAG
TCATCTTTACACGATGAGTATCGCCAGGCAATACTTCATCACAATAAATAGGAACTAAAAAACCAGCATCAAACGTGGTTTTATGAGCATATTGA
GTATTAAAACTAGAGCGGGGAATGTCCGCTTTAGGAACCATA

>000019F|arrow

CCCGATGCGCGTCTCCAACGGCCTGAATACATTGGAGGCGGTTCAACACACATTAATATCAATCCAATCGCCCAGACGAATGGTACCGGAGCTTC
CGGGACCACTACTCCTCTCGGTACACTTGGCGCTATGGGTACTGGGCTCGCTCACAATCATGGCTTTACTTATTCAAGCACTGAACATGGTGTAAT
TATCGGTCTCGTTTCAGTACGAGCCGATTTAACATACCAACAAGGTATGCACCGCATGTGGAATCGTTCCACACGTTATGATTTCTATTTCCCTGC
TTTCGCCACTTTGGGCGAACAAGCAGTATTAAATGAAGAAATCTACGTACGAGGCGATGCCAACGATACAGGAGTGTTTGGATACCAAGAACGT
TGGGCAGAATATCGTTATATGCCAAGCCGAATTTCCAGTCTGTTCCGTAGTACGGCAGCTGGAACAATTGACGGCTGGCATTTAGCCCAACGGTT

TACAACACTTCCAACTTTGAATAACACGTTTATTCAAGAAAATCCACCTGTCTCTCGAACCCTTGCGGTCGGAGCAGCTGCCAACGGCCAGCAAA
TCATTTTTGATAGCTTTTTGATATTAAAAAGCACGGCCAATGCCAATGTACTCTGTACCTGGCTTAATCGACCACTTCTAATGGCACTAGAAGC
CGCTGCCTCAGGCGCCGCATCTGGCGCCGCTTTTGGACCTTACGGCTCCTTAATTGGAGCCGGAATAGGTGCGGCCGCTAGTTATTTTGGTGGTC
AAGAACAAACGCTGCCAGCGCACAACAAGCTGCAGCAATGATGCAATTCCAAGATGGTATGCGACGTACTGCATATCAAGACGCAGTAGCGAT
CTTAAGGCTGCAGGTCTTAACCCTATGCTGGCTTATTCACAAGGCGGAGCCAAAGTCCAGCCTGGTGCGCAAGCTCCAGTAGGAAATCCACTAG
GTGAGGCTGGAAATTCAGCCCGTGAAGCTGCCATGGCAGTCGCCAATTTTAAACAATTACAAACTCAGAATATCCTGACACATCGCAAGCCGAA
AAAACGGACGCGGATACAAATCTATCACGTGATCAGGCAACATATACTCGAGCAAATACAGCTCGTGAAATTGCTCAGATGCCGGGATACGGCA
AATTTGGTCAGCTTCGCGATGCCCAAATAGAGCAATTAAGGACATCAAGTGCATTACAAGCTGCACAACAGCGACAAGCGTTAAGTCAAAGTGC
ATATACAGACCAATTAGAGCGATTAGCGCAAACTGGATCAGCGCCATCCAGTACTAAACCAATTTATCAAGATGTTAAAGGCTATTTACATAGCC
AATATGATAATATCAAAATATCTACCATTTGGAAAAATGAAATGAAAACAATCAAACTTAGAACCGCATACAACTATGACACTGCTGCGTCAAAT
GAGTCAGGGTTGGCTTGTGAGGAGCCAACTCTGGCTCAGCAGCATTATAAAGACGAATGCGATATAAATACTATCCTGGAACGTTTTAACGTTA
CAGGCCTATTACCTCAAAGTCCGCTGCCGCCTCAATATGGCGATTTCAGCGGAATTACTGACTATCATAGCGCCTTGAATAAGGTAATGAACGCT
ATGGAAGAATTTGATAACTTACCGGCTCAAATTCGTGCTAGGTTCGAAAACGAACCAGCAAACCTGATTGAGTTCTTGCAAGACGAGAAAATCG
ACCAGAAGCCGAGAAACTCGGCCTGGTCGAAAGAGCCATTTCGGAAGAAATGGCGATAAGCACAGTTACTCCACTTGATGTAACTGTGCTAGGT
GACACCAACACCAAAAATCTGATAAACGAGGCCAAAATTATGCTTTATAGAAAACAAACAAACAAGCGCAAAAGCGCTAAATCGTTCCGTAGGA
ACACTTCAAAAACTAAAGCTGCAAATATGCAAAAAGCCCCGCAAAGAGGGGGCTGGCGGCTCTAATAAAGCGCCAGGCTACCTCACATGGCCT
GTTATCACCCACTGACTGCTTTTAAGTAAGCATCAGACAAACTATAAGACCGGCAAATCTTATCGCCGTGTCGCATTCAAAGAATCTGACGAGCA
TGATCGTCAGATTTCACTGCCCTGCGGCCAATGCGTTGGCTGCAGGCTAGAAAAATCACGTCAATGGGCCATGCGCTGCATGCATGAAGCCCAA
TTGCACGAAAAAAACTGCTTTATAACCCTCACATACAACAATGAAAACCTTCCACAAACTGGATCGCTTGTCAAAAGCGACTTCCAAAAGTTCCTT
AAGCGCTTCAGAAAATCCATTGCACCTGCAAAATTACGTTACTACATGGCTGGAGAATACGGCACAAGTTTCGGCAGACCTCACTTCCATGCCTG
TATCTTCGGATACGATTTTCATGATAAGAACTATTCAAAAGGACTCCCTCTGGTTCTCTCATATATACATCCGACCACCTTGCAACCCTCTGGCCAC
ATGGTTATTCCTCCATTGGAGACGTTACATTCGAGTCAGCTGCTTACGTTGCTCGATATAATGCAAAAATACAACGGCCAGATGGAAGAAAACAA
ACATATACAAAGGATGAGCATTACACATACTGTGATATAGAAACAGGGGAATTAATAAAGCTATTACCAGAATATAACAATATGAGCCTTAAAC
CAGGCATTGGTGCTGAGTGGTACAAAAAATCGTTCCGACGTATATCCCCATGACTATGTTGTAGTCAACGGAAAAAGGGTAAAACCCCCAAAAT
ACTATGACAAAAAATAAATCAGATTATCCTATGAATACGAAGAATTACTCCACAAACGTGAAACTTCTGCTAAACTCAACCACGAAGACAATACC
TATGCCAGACTTGCCGTAAAGGAAAAAGTCACAAAGGCCAAACTTCAATTATTAAACGTAACCTCACTTAGGAAATCCTCATGAAATTAGTACTC
TGTACCGTTAAAGACCGCGCAGCAGATGCGTTCGGTCGTCCAATGTTCGTCCGTTCTATCGGCGAAGCAATCCGGAGCTTTAGCGACGAAGTCA
ATCGCCAGAGCGATGACAATCAACTTTATAACCATTCCGACGATTTCGACCTATTTGAATTAGGCGAATTCGACGATAATACGGTTTGTTCCAATT
ACATGAACAACCCAAACTTGTATCCTTAGGGAAACAAGTCAAAATTACTGATAAAAACTAAGCGTAGAGTAAAAAGGGGAAACCCCTTTTTCTCA
CGCAACTAGGCCTAGGAGCTCAAAAAATGCATCGTAACAAGTCGGTAGACGTCCATCAGTTCACAATGATTGCCAAAAGCGGATAATCCCCGCG
CTCTACATTTGACTGTCCATCAAACCATAAAACTACATTCGATCTGGGGCTTTCCTAGTCCCCTGTAACTCTTAGACGAAATGTTGCCAGGCGATA
CATTCCGCTGCAAATGACCGCCTTTGTCGCGATTGTCTACACCTCTCTATCCGATCATGGATAACATGCATCTGGATGCTTCTTCTTTTTGGGTGCC
AAATAGACTTATCTGTCAAATTGGCAAAAATTTTATGGGCAGCAGGCAAATCCTGCGGACTCGATCTCGTACGTAGTGCCCCAACAAGTAACCCA
GCTGGTGGTTAGCGCTATTGCAGCCTTCAAGATTATATGGGTCTGCCAACCGTAGGGCCAGTAGGTGCTGGTGGCACCGTAAGTCACTGTGCCT
TCTGCCACTGGCTTACAACCTTATTTAGAACGAATGGTGTCGGGACGAAAACCTTCAAAATTCAGTAGTTGTAGATACTGGCGATGGGTCCAGAT
AACGTAGCAACTACACATTATTACGACGTGGAAACGTAAAGACTATTTCAAGTCAGCATTACCTGGCCACAAAAAGTGGCGCAAGGACGTACGC
TACCAGCTTGGAACATCCGCCCCAATAGTACGCACTAACGAATGCCCTTGTTTAGGCCAGACCTGTATACGCTGGAACAAAATACATTAACGCAA
CCGCCCAGGCTATTACGTAGGTGTTACTGGTCAAATTACTGGCGGTTGCTGACGGCTTGGCAAAATCATATGATCCTAATGGCGGTTTATATGCA
GATTATCAGCTGCAACCGCTGCAACAATTAATCAATTGCGTCAAAGCTTCCAGATTCAAAAACTTTTAGAAAGGGACGCCCGTGGCGGAACTCAG
ATACACAGAAATTATCCGCAAGCCATTCGGGGTCGTTAGC

>000213F|arrow

AATCTGGAAAGATTGACGAAGTTGGTTAATAGTCGCAGCAGTAGCAGTAGACAAATCAGCGTATAACTGATTAGTAGCAACACCAGCATTTGTA
CTATTAGACAAAGTATTAGAAGCTGAATTTAATTCTCTTAAAGCACCAACAGCAGTTTGAAAAACACTATAACCAGCAGTACCATTGCCAGCTGG
TATGTCAGAATAAATATTAGCACGTGTTCCTAACGGTAAAGAAACAGAAGCACCCTTCTGTGGCCAAGGCAATGCACCAGTAAAATAATCTTTAC
GCTTACCCACGTCTCAAAAGTGTATAGTTAGCAGGAACATCACCTGAATCACCAAGATTTAACGTTACAGAATTCTGTAAGTTTTCATCTCTAAACC
ATTCGTTATAAATCTCATTATAAGCACGTAACGGCAAAACATTATGCGTTACTGTATTAGCGCCAGTAATCTGACCAGCAGTAGGTAAACCAAAA
TAATCAAAAATTGAACAAACAGCATAACCACCAGCAGGACTAGTAATAGTAGGCACTACAAAAGAAATAGAATCACCAGGATCGTTTGTTCACC
CATAAACTTTGGCCAATTGTTCCAAAGTAAACGATTAGGTACAAAGAAAAAGAAAGTATCAAGATGCAAGTTGTCCATCACAGGAAATAACGGT
GTGGCCAAACGTGCAAATGCAGTCATCTTTACACGATGAGTATCGCCAGGCAATACTTCATCACAATAAATAGGAACTAAAAAACCAGCATCAA
ACGTGGTTTTATGAGCATATTGAGTATTAAAACTAGAGCGGGGAATGTCCGCTTTAGGAACCATAGCAAATGAATGCGTACTTACTGACTTATTG
CGAAACATAAACATCTCCCGTAGTTCCGTACCACTCTTTCGAGTGATACGGTATAAAAAAAACCTTACTCGCCTTCGCGAATCATCTTTTGCACGA

GCAATCAACTTAGGGCTTTCAGTAGTTCAAAAACACCAGTGGCATCGTCAAAAAGACCCAAATAGAACATATGAAAATCATCAGGATGTTTATAC
AACTGATATCTTCGCTAGCTCGATTGACTTCATCCTGAAACTGACGTACTGCAACACCTTCAGATGCAAACATAAGCTGGACGACCAAAAGCATC
TGCAGCAGTATCCTTAATAGAAACAATAACCATCTTCATAAAAACTCCTTAAATAGTACGTTTTAACAATGACAACTTAGCCAACGCAACTTTTTCC
TTAACAGCAAGTCGCTCAAGGTGTTGTCCTCATGCCTAGATCGACCTTCCATCTCTGGCAAACTGAATCATATCGAATTCTTCAGGAAACTTCAAC
TTAAATTTATTATCATAAAACCGTGGTGGACGGCACTTTTGCCACGCACCACAACGTGGTCTGACGTATAAAGTCTGACATGTACTTATCTAACCA
CGATTGCCCGATACGGGCTTCAATGACATCTTATTAAATTCTGGCTTACGCTGAATTATCTCACCAGTCTCTAAATCACAATATTGATAATGGGCA
CCCGCATCAACCACTTCGTGGTTTTCATTGACAGTAACCCCATTAATCTTTTCATAATATATCTTGCAACATAAGCAGCAGACTCAAAAGTAACATC
ACCAATTGTAGAATAGCCAAACGGCCACAATTCTTCCAAAATCTCTGACGTGTAGAGGATAGAGCCAGTCTGCGTTCTTTTAAATTTTCTTATCCG
GAAAATCAAGACCAAACAGACAAGCATGGAAATGAGGACGATCAAAAGATTCACCATATTCACCTGCCATATAAAAACGTATCGTTTCCAGTAA
AACGCTTACGTAACCGTTCAAAAGCTGATAATCATTGTAATCCAATGACATATCCTTAGGACAATGCTCTGGAGCATATGTCAAGTAATAAAACA
ATTACTAGTATGCATTTGTGCCTCATGCATACAACGAATCGCCCACTGACGTGAGCGTTCAAGGCGACAACCAACACACTGACCACAAGGCAATG
ATAGGGTACGGACTACATCCGCACCCGGTATTTCCCGCCAAATTATAGACCTGTCACTGCATTGATAAGCCGTTAAGGGCTTATAACAGGCCATA
ATTACAAACGATAGCCACCACGCTGGGGAGCGTGTCTCATATTAATGACTTCGTCTTACTAGCAGTTCTGCGAAATGACTTTGCAGATTTATTTGT
TTACTGGCTTTCTTCGTAACATGATGAATCCGTAGTTAAAATAGTGGTTTGGTGTCACCTAGCACAGTTACATCAAGTAGAGTAACTGTGCTGGCC
TCAGGATTTCATCCTTCGGCCTTAGGTGTTTCTGTAGAAACGATGGGTTCAACCACAGGTTGTCCATCAATAAGACCCAATTGAATCGCTTCATCA
CGATTCTGGTCGTTCTCAAGGAACTCCAATAATTTGACAGGATCATGGTCAAATCGGACTCTTAATTCGCTGGCAGAGCCATGAATCGTCCATAG
TGCGTTAATTTGATTCAACGCAGAATGGTAATCAGTAACACCACTAAAATCGCCGTATTGAGGCGATACAGGGGCCGTTGGAAGTTCCCTGTAA
CCCCGAAACGTTCAATGATGACATTAATGTCACATTCATCTTTCATATGCTGTTGAGCAAGACTTGGGTCTTGACACAGCAACGCATCGTTTACCG
ATGCTTCATCTTTATCATAGTTGTACGGATTACGTACAAATGGCAAATTCGCTTACTCATTATTTGACTCCAATTCCCCAAGGGGTTAGTGATTAG
GATTATTTTGAGTTTATCAAAAATTTCTTCGAAGCATCGATACCCCTAGACCAAATAGCTGGGGATGGAGCCAATAGCCTCTAGTATTATATGACT
GAGCGGATGTTAAATCCGCAGTCGTATTCTTTAAAGCAATATCAGCCAAAATACGTTATTTTCGGCTGTAATATTAGGAGCAGTAAGCAACTTAT
TAACAGTATCAGCCCTAGTATTAGCGGTACCAGCTTCAGTAGCTTCAGTCTGGGCGATAATCTGCTTTTCTGTTTCTGATGCATACGAATCTGTTG
CATCATAGAAGCAGTATTAATAGCTGAATTAGTTGCATTACCTAAAACATTTTCCATAGTAGCAGTTGAACCAGATGGGGTAGAAGCACCACACG
TTGATAGGCTACATAGGAGATAAACCAGCAGCTTCTAAATCTTTAACAGCACGCTGGTAACTTGTTCCACTCATATCGGCTTGAAAATCCATTTGC
CTCTGAGCCATCTCCTGATTAGCTTTATTAGCAGAAGTAGAACCTAATAAGAACCAACAGCGCCCAAAGCAGTACCGACACCAGGAGTAAAGAA
ACTAGAAGCTGAAGACAGCTTAGAACCAACATTAGTAACCGCATCAAGTATTCCACCAAACATAAACTAACGCCTTCGGTTGTTTCCTCAACTCCT
TACGGAGTAGTCGAGGTTATATAAAACATTAGAAATGATCAATTAGACCAGGTACGCTGTACATAGGCATAGGTCTGGCCATCTGACAATCAAA
GAAAGCATCCATTAAAAATTGCTGACCATTTGCTGCAGCTCCGACCGCCGTAGTACGAGCAACTGGAGGGGTCTCTTGAATAAACGTTGAATTCA
ACGTAGGAAGAGAAGTAAATCGCTGTCCATAATGCCAAGCATCAATAGTGCCAGCAGAAGTAGACCTAAAGAAACCAGTAATTTGAGAAGGTT
TGTAACGATACGCCCAACGTTCTTGATAACCAAATACATCATTGTCGGCTGCAGTACCTTGAACATAAATTTCTTTGTTCAAAATAGCTTGCTCACC
CAAAGTGGCAAATACTGGGAAATAAAAGTCATATCGTGTAGACTAGACCACATCTTAGGAAGACCCTGTGATATGTGAGGTCAGCACGTACAGA
AACTAATCGATAATGTATCCATGTTCTTGAGCAGCATACGTAAAGCCGTGTCCCTGAGCCAATGCAGTACCCATTGCAGCAAGGTTACCTTGCGG
AGTAGCAGAACAGTAACCGACGTTGCAGAAGTCTGAGCAATCGGATTAACATTAACAAGGGTCGAACCTCCACCAATATTCAGGACGTTGTAAA
CGATAATCCTGTGGAGTTACTCCAAAGTGAGCACGTAGTAACTCAGTATAACGAGTACCACCACGTGCATCGCGCTCCAATAACTTCTG

>000095F|arrow

AGAGCGTTTGGCTGTAAAGGAAAAAGTCGCTTTGGCTAAGTTATCGCTTTTAAAACGTAAGATTTAAAGGAGTTTTTATGAAGATGGTTATTGTT
TCTATTTTAGATACTGCAGCTGGTGCGTATGGTCGTCCAGCTTTTGTTGCATCTGAGGGTGTTGCTGTTCGTCAGTTTCAGGACGAAGTTAATCGC
GCTAGCGATGATAATCAGTTATATAAACACCCTGATGATTTTCAGTTATATTATTTTGGTACGTTTGATGATAATTCTGGTACTATGGATTTGCTTG
GTTCTCCCAAGCTTATTTCTAGAGCTAAGGATGTTATGATTCGCGATGGCGAGTAATGTTTTTTTAAGCCGGATCACTAGCTTGCTAGTGGTTCG
GAATACTTCGGGAGATTGTTATGCATCGTAATAAGTCAGTAAGCTCACATAGTTTTGCTATGGTTCCTAAAGCGGAAATTCCGCGTTCTAGTTTTG
ATACGCAATATGCGCATAAAACTACTTTTGATGGTGGTTATCTAGTTCCTATTTATTGTGATGAAGTGCTCCCAGGGGACATGCACAATGTAAAG
GCTACAATGTTTGCCCGTTTGGCAACGCCTTTGTTTCCAGTTATGGACAATTTGCATTTAGATACTTTCTTTTTCTTTGTTCCAAATAGATTAGTTTG
GAACAATTGGGTTAAGTTTATGGGTGAGCAAGCGAACCCAGGTGATTCTATTTCGTATGTAGTTCCACAGATTACTTCTACTGCTGGTGGTTATG
CAGTAGGTTCTATTTTTGATCATTTTGGTTTGCCTACGGCTGGTCAGATTACTGGTACTAATACGGTTACGCATAACGCGTTACCGTTACGTGCTT
ATAATTTGATTTATAACGAGTGGTTTAGGGATGAGAATTTACAAAATTCTGTTACCGTTCACAAGGGTGACGGTCCTGATACTCCGAGTGATTAT
GCTATGGTTCGACGCGGTAAGCGTAAGGATTATTTTACTGGTGCCTTGCCTTGGCCTCAGAAAGGCGATGCAGTTACGTTGCCTTTAGGCACGTC
TGCTCCTATTAAGACGTCTGTTACTAATGATTCAACAACGTTGTCATTAGTTGACGGTGCTGGTGCTTTGAAGCAGATTTATGCTAATTCAACTAC
ACATGCTGTGTATCGTAGTAATAACGCTGGTACTGCTGGTACTGGTTTGTATGCCGATTTAAGTGATGCTACGGCTGCAACTATTAACCAGTTAC
GTCAGTCTTTCCAGATTCAGCGTTTGTTAGAGCGCGATGCGCGAGGTGGTACACGCTATACAGAATTGTTACGTGCTCATTTTGGTGTAACTCCA
CAAGATTATCGTTTACAACGTCCTGAATATATTGGTGGTGGTTCTACTTATGTCAACGTTAATCCTATTGCTCAAACGTCTGCTACTTCGATTTCTG
GCGGTGCTACTCCGCTTGGTAACTTGGCTGCAATGGGTACTGCGTTGGCTAGTGGACATGGTTTTACGTATCATGCTCAAGAACATGGATACATA

ATTGGTTTGGTAAACGTAAGAGCTGATCTAACATATCAGCAAGGTTTACCTAAGATGTGGTCTCGTGAGACACGTTATGATTTCTATTTCCCTGTA
TTTGCTCATTTAGGTGAACAGGCTGTTCTTAATAAGGAAATCTATGTTACTGGTACTTCAACTGATGATGATGTATTTGGATACCAGGAACGTTGG
GCAGAGTACCGTTACAAACCTTCTCAGATTACAGGTTTGTTTAAGTCGACTTCAGCTGGTACGATTGACCCTTGGCATTATGCTCAGAAGTTCACT
TCGTTGCCGACTTTGAATTCTACGTTTATTCAAGAAACGCCACCTATTGATCGTACTACTGCTGTTGGTGCATCTGCTAATGGTCAGCAGTTTTTGA
TGGATGCGTTTTTTGATTGTAAGATGGCTCGTCCAATGCCGATGTATTCTGTACCTGGCTTGATTGACCATTTCTAATGTAATATAAGCTGGACTA
CTGGGAAACCAGTAGTCAGCAAACAAGCGGAGCGCGTTAGTATGGGTATGTTTGATTCAGTTGCTGGTGGTTTATTTGATTTTGCTACTTCTGCT
TGGAGTCAGAATGAAGAGCAAAATATAATACTGCTGAAGCGCAAGCAAATAGACGCTTTCAAGAAGATATGTCCAATACGCAATATCAGCGTAT
GGTTACTGATTTGAATAAGGCTGGTTTGAGTCCTATGCTTGCATATTCTAAAGGTGGTTCTACTGTTCCGAGTGGTGCTACTGCATCTTCTACTTCT
CCAGTTAAGTCTGATTTGAGTGGAGCTGTTAAGCGTGATGTTGAGAGTGATTTATTGCGTGAGCAGATGGAAGTTGCTAAATCTCAACGTGAAT
TAAATGTTCAGACTGCTAGGAAGGTTGCTGAAGAAGCTGATTTAGCTTCTCAGCGTGTTTTACAAGAACCGGCTCGTTTTTATTTGGAACAGGCC
GAGGCTGGTTCTCGTGTTAATGCTAATTCTGCATCAACAATTAAGACTGATATTGATGCTAAGAATAATTTAGAGTTACGAACACCGTCTAGCGA
TCCATATTGGTATCGCGATATTAAGAAAGGTAGTAAGTCTATTTTTGATAAGTTTTTGGAAAAGAATAAGTCTTTTCCTTGGTCCTTGGAAGGTCG
TAGAAAATGAGTAAAACGAATTTACCTTTTGTACGTAATCCGTACAATTATGATATGGCTCTTGTTTCACAAGAGACTGGTCTTGAATGTAAAGAT
CCGAGTTTGGCTCAACAACACATGAGAGACGAATGTGATATTAATATTATAGTTGAGCGTTTTGGCGTTACTGGGCAATTGCCCCAGGCGCCATT
AGAGCCATCATATGGCGATTTTAGCGGTGTATCTGACTATCACACCGCATTGAATGCTATTCGTGCCTCTGATGAGGCTTTTATGGCTTTGCCAGC
TAAGATTAGGGCTAAGTTTGATAACGATCCTAATGCTTTGTTAAATTATTTACAGAATGAAGAGAATCGTGATGAAGCGATTCAGATTGGTCTTA
TTGACGGTAAACCTGTGGTTGAACCCGTCGTTTCTGCAGTAGAAACACCTAAGCCAGACGCGTAAGCGGATGGCAGCACAGTTACTCTACTTGA
TGTAACTGTGCTAGGTGACACCAAAACCACATTATTAACTACGGAGTGCAATGTTATGAGCCTTTATAGAAAACCAATGAGCAAGCATGGCGCA
GCTAAGAAGTTTCGTCGTGGCGTAAGCAAGACCAAGAGCATTAATATGCGTACTTCACCGCAGCGCGGTGGTTTTAGACTGTAATTTATGGCGT
GTTATAAGCCGTTAACGGCTTATCAATGCGCTGACAAGTCTATTATTTGGCGGGAGATACCAGGGGCGGACGTAGTCCGTACCTTGTCATTGCCT
TGTGGTCAGTGTGTTGGTTGTCGCCTTGAACGGTCCCGTCAGTGGGCCGTTAGATGTATGCATGAGGCACAAATGCATACTAGTAATTGTTTTAT
TACTTTGACATATGCTCCAGAGCATTGTCCTAAGGATATGTCTTTGCATTATGAAGATTTTCAATTGTTTATGAAACGATTGAGAAAACGCTATAC
TGGCAAGACTATTCGTTTTTATATGGCAGGTGAATATGGTGAGTCTTTCGATCGTCCTCATTTCCATGCTTGTATCTTTGGGCTTGATTTTGAAGAT
AAGAAGTTTTTCAAAAGAACGCAGACTGGGTCTATCTTATATACGTCAAAGATACTTGAAGAACTTTGGCCGTATGGCTATAGTTCTATTGGTGA
TGTCAATTTTGAATCTGCTGCTTATGTTGCTCGATATATTATGAAGAAGATTAACGGTAAAACCGTTAATGAGAACCACGAAGTGGTTGATGCAG
ATGCGCATTATCAGTATTGTGATTTAGATACTGGTGAGATTATTCAGCGTAAGCCTGAATTTAATAAGATGTCTCTTAAGCCTGGCATTGGTCAG
GCTTGGTTTGATAAGTTCATGTCAGACGTTTATACGACTGACTCTGTTGTGGTGCGTGGCAAAAAGTGCCGACCACCACGGTTTTATGATAATAA
GTTTAAAGTATTGTTTCCAGAAGAATTTGATGGTATACAATATGCTCGTGAGCTAGAAGGTCGCTCACATTTTGAAGATAACACTTT

>000101F|arrow

TTGAGCGACTTGCTGTTAAGGAAAAAGTTGCGTTGGCTAAGTTGTCATTGTTAAAACGTACTATTTAAGGAGTTTTTATGAAGATGGTTATTGTTT
CTATTAAGGATACTGCTGCAGATGCTTTTGGTCGTCCAGCTTATGTTGCATCTGAAGGTGTTGCAGTACGTCAGTTTCAGGATGAAGTCAATCGA
GCTAGCGAAGATAATCAGTTGTATAAACATCCTGATGATTTTCATATGTTCTATTTGGGTCTTTTTGACGATGCCACTGGTGTTTTTGAACTACTG
GAAAGCCCTAAGTTGATTGCTCGTGCAAAAGATGTAATGATTCGCGAAGGCGAGTAAGGTTTTTTTATACCGTATCACTCGAAAGAGTGGTAC
GGAACTACGGGAGATGTTTATGTTTCGCAATAAGTCAGTAAGTACGCATTCATTTGCTATGGTTCCTAAAGCGGACATTCCCCGCTCTAGTTTTAA
TACTCAATATGCTCATAAAACCACGTTTGATGCTGGTTTTTTAGTTCCTATTTATTGTGATGAAGTATTGCCTGGCGATACTCATCGTGTAAAGATG
ACTGCATTTGCACGTTTGGCCACACCGTTATTTCCTGTGATGGACAACTTGCATCTTGATACTTTCTTTTTCTTTGTACCTAATCGTTTACTTTGGAA
CAATTGGCCAAAGTTTATGGGTGAACAAACGAATCCTGGTGATTCTATTTCTTTTGTAGTGCCTACTATTACTAGTCCTGCTGGTGGTTATGCTGT
TTGTTCAATTTTTGATTATTTTGGTTTACCTACTGCTGGTCAGATTACTGGCGCTAATACAGTAACGCATAATGTTTTGCCGTTACGTGCTTATAAT
GAGATTTATAACGAATGGTTTAGAGATGAAAACTTACAGAATTCTGTAACGTTAAATCTTGGTGATTCAGGTGATGTTCCTGCTAACTATACACTT
TTGAGACGTGGTAAGCGTAAAGATTATTTTACTGGTGCATTGCCTTGGCCACAGAAGGGTGCTTCTGTTTCTTTACCGTTAGGAACACGTGCTAA
TATTTATTCTGACATACCAGCTGGCAATGGTACTGCTGGTTATAGTGTTTTTCAAACTGCTGTTGGTGCTTTAAGAGAATTAAATTCAGCTTCTAAT
ACTTTGTCTAATAGTACAAATGCTGGTGTTGCTACTAATCAGTTATACGCTGATTTGTCTACTGCTACTGCTGCGACTATTAACCAACTTCGTCAAT
CTTTCCAGATTCAGAAGTTATTGGAGCGCGATGCACGTGGTGGTACTCGTTATACTGAGTTACTACGTGCTCACTTTGGAGTAACTCCACAGGAT
TATCGTTTACAACGTCCTGAATATATTGGTGGAGGTTCGACCCTTGTTAATGTTAATCCGATTGCTCAGACTTCTGCAACGTCGGTTACTGGTTCT
GCTACTCCGCAAGGTAACCTTGCTGCAATGGGTACTGCATTGGCTCAGGGACACGGCTTTACGTATGCTGCTCAAGAACATGGATACATTATCGG
ATTAGTTTCTGTACGTGCTGACCTCACATATCAACAGGGTCTTCCTAAGATGTGGTCTAGGTCTACACGATATGACTTTTATTTCCCAGTATTTGCC
ACTTTGGGTGAGCAAGCTATTTTGAACAAAGAAATTTATGTTCAAGGTACTGCAGCCGACAATGATGTATTTGGTTATCAAGAACGTTGGGCGG
AGTATCGTTACAAACCTTCTCAAATTACTGGTTTCTTTAGGTCTACTTCTGCTGGCACTATTGATGCTTGGCATTATGGACAGCGATTTACTTCTCT
TCCTACGTTGAATTCAACGTTTATTCAAGAGACCCCTCCAGTTGCTCGTACTACGGCGGTCGGAGCTGCAGCAAATGGTCAGCAATTTTTAATGG
ATGCTTTCTTTGATTGTCAGATGGCCAGACCTATGCCTATGTACAGCGTACCTGGTCTAATTGATCATTTCTAATGTTTTATATAACCTCGACTACT
CCGTAAGGAGTAGTGAGGAAACAACCGAAGGGCGTTAGTTTATGTTTGGTGGAATACTTGATGCGGTTACTAATGTTGGTTCTAAGCTGTCTTC

AGCTTCTAGTTTCTTTACTCCTGGTGTCGGTACTGCTTTGGGCGCTGTTGGTTCTTATTTAGGTTCTACTTCTGCTAATAAAGCTAATCAGGAGATG
GCTCAGAGGCAAATGGATTTTCAAGCCGATATGAGTGGAACAAGTTACCAGCGTGCTGTTAAAGATTTAGAAGCTGCTGGTTTATCTCCTATGTT
AGCCTATCAACGTGGTGGTGCTTCTACCCCATCTGGTTCAACTGCTACTATGGAAAATGTTTTAGGTAATGCAACTAATTCAGCTATTAATACTGC
TTCTATGATGCAACAGATTCGTAATGCATCAGAAACAGAAAAGCAGATTATCGCCCAGACTGAAGCTACTGAAGCTGGTACCGCTAATACTAGG
GCTGATACTGTTAATAAGTTGCTTACTGCTCCTAATATTACAGCCGAAAATAAACGTATTTTGGCTGATATTGCTTTAAAGAATACGACTGCGGAT
TTAACATCCGCTCAGTCATATAATACTAAGAGGCTATTGGCTCCATCCCCAGCTATTTGGTCTAGGGGTATCGATGCTTCGAAAGAAATTTTTGAT
AAACTCAAAAATAATCCTAATCAACTAACCCCTTGGGGAATTGGAGTCAAATAATGAGTAAAGCGAATTTGCCATTTGTACGTAATCCGTACAAC
TATGATAAAGATGAAGCATCGGTAAACGATGCGTTGCTGTGTCAAGACCCAAGTCTTGCTCAACAGCATATGAAAGATGAATGTGACATTAATG
TCATCATTGAACGTTTCGGGGTTACAGGGGAACTTCCAACGGCCCCTGTATCGCCTCAATACGGCGATTTTAGTGGTGTTACTGATTACCATTCTG
CGTTGAATCAAATTAACGCAACTATGGACGATTTCATGGCTCTGCCAGCGAAATTAAGAGTCCGATTTGACCATGATCCTGTCAAATTATTGGAG
TTCCTTGAGAACGACCAGAATCGTGATGAAGCGATTCAATTGGGTCTTATTGATGGACAACCTGTGGTTGAACCCATCGTTTCTACAGAAACACC
TAAGGCCGAAGGATGAAATCCTGAGGCCAGCACAGTTACTCTACTTGATGTAACTGTGCTAGGTGACACCAAACCACTATTTTAACTACGGAGTT
CATCATGTTACGAAGAAAGCCAGTAAACAAATATAAATCTGCAAAGTCATTTCGCAGAACTGCTAGTAAGACGAAGTCAATTAATATGAGACAC
GCTCCCCAGCGTGGTGGCTATCGTTTGTAATTATGGCCTGTTATAAGCCCTTAACGGCTTATCAATGCAGTGACAGGTCTATAATTTGGCGGGAA
ATACCGGGTGCGGATGTAGTCCGTACCCTATCATTGCCTTGTGGTCAGTGTGTTGGTTGTCGCCTTGAACGCTCACGTCAGTGGGCGATTCGTTG
TATGCATGAGGCACAAATGCATACTAGTAATTGTTTTATTACTTTGACATATGCTCCAGAGCATTGTCCTAAGGATATGTCATTGGATTACAATGA
TTATCAGCTTTTTATGAAGCGGTTACGTAAGCGTTTTACTGGGAAAACGATACGTTTTTATATGGCAGGTGAATATGGTGAATCTTTTGATCGTCC
TCATTTCCATGCTTGTCTGTTTGGTCTTGATTTTCCGGATAAGAAAATATTTAAAAGAACGCAGACTGGCTCTATCCTCTACACGTCAGAGATTTTG
GAAGAATTGTGGCCGTTTGGCTATTCTACAATTGGTGATGTTACTTTTGAGTCTGCTGCTTATGTTGCAAGATATATTATGAAGAAGATTAATGG
GGTTACTGTCAATGAAAACCACGAAGTGGTTGATGCGGGTGCCCATTATCAATATTGTGATTTAGAGACTGGTGAGATAATTCAGCGTAAGCCA
GAATTTAATAAGATGTCATTGAAGCCCGGTATCGGGCAATCGTGGTTAGATAAGTACATGTCAGACGTTTATACGTCAGACCACGTTGTGGTGC
GTGGCAAAAAGTGCCGTCCACCACGGTTTTATGATAATAAATTTAAGTTGAAGTTTCCTGAAGAATTCGATATGATTCAGTTTGCCAGAGAGATG
GAAGGTCGATCTAGGCATGAGGACAACACGC

>000186F|arrow

CCTCCTTGAAGGAGACGGGTTAGATCACTATTGAAGCTATTTCCCTTGAGACGGTAATAACATATGAACATAAAACGGTGGAAAGACTTGTGTC
GGGATAGATCCGGCCATAGACGAGATGACAGTTGACACACACCTCTAATCTGTAATGACGAGGTTCAATTTATTACCTTTCACGGGCACCAGTTC
CTGACCCCTTGAGTAATAGGAGGTATAACATCGGTAAGAGAAGACCATTAGTGCCTGTTGTTCCATTCCAATCAATACCGGAACCAACAATATTA
GTTGTATTATCAGGATTTACTAAAGTAAGCTGCCTGAACTGTGATTTATTTCAGCAGTTTTATGAATAACACCTAAAGCAATCTTACGCATACCTG
TTCCATAGTAAGTTTGACCGCTTTTTTGCCTTACGGCCATATCGCTTTTTCGCATACCGTTTCTTACCGAGTTTTTACAAACACCATTTATATTATGA
GGAGAGAATTTATTTTATTTCTTCCACGGAAGTTAGACGCCGGTTTACTTGATCTAATTCATTACCCTGCCAGTAATATTCTGGCGGGTATTCACA
GGTGATATAAATATATGGAGAGTTGAGTTGTATATAACTACCTTTGACTTGTGCTAAATACTCATATCTATCAATGATCCGTAAAAACGGCCTAAA
CGGTATTTTATTATCAAAGTCATCAATAATAACCGCTTCTTGTTGCTTGTATCCGTCCCACCAAGGCGTATTATCTTTTATGTAGTGAGAAGAAGA
GTGTTTATCAACACAATATCGGGTTTTGCCAGTTCCTGCTAAACCCCAACGCCAGTGCACCGCAGGAGCGGTGCTTCTGGCTTTAAGAACAGCAT
TACACATTTTTTCTAAACTTCTTGAATATTTTAAATACAATACAGGGTAATCAAACATTATATCCTCTAATGTGATCTCCTTATTCTTGATTTTTTGTG
CTACACTATGTATATCGTTTCGCTTACCTTGTTCCTTACTCGGTTCTCCAACCTCAAAAAAATCACCATCTTTAGAACAATACTTCTTATTGTCTAAA
TCTGATCCCTTTGCTACTTCTAAATGTGCTCGGGGTATGTATTTCTTTATTCTTGCTAAAGTTAAACCTTCTATTAAACGGCCATATACCTGTAAATG
TGGTGTTCCTTCTTCTCCTACTTCCTTACCACAGATGCCGTATTCGCATAATTTAAAAATTGCTTAATACCAAATATGTCTGTGTCTGTGTAGTTATT
TATCGTGATCGTCCAAGGTTTGTTCCTCTCACTCATTTATATATAGTCCATATATTTTATTTATTGCTAAATAAACATAAATAGTTTCTCCACGAAAG
TTAGGGGGTAATACTGAACCCCTAACTTTCTTACTTCTTTTTTAATTTTTCTGTAAATAGATTACAGATTAAAAACAGGATTTTTTCGCTGGAAAGA
ATGATGATGACCGCAAGGGTCATCATAATTCGCTCAATAAAATTTTTTAATGTTAATGCTTCGCACAGGCATCCGCTTAAATAACATTTATACATC
TTCAAAAGTAAGAACACTCTCACAGGCTACTTTAGCCCGAACAAAACCTACTGGATTTTGAGTCCCATCCGCATTAACAACTGCGAAAGCAACAA
ACATATTATCGTTTGTAGGCTGATTAGTAATGCTGTCATCATAGAGCCAAGACTTTTTGATTTTAATAGATTGCTTAAATATATGTGCCATCTTAAA
ACCATTACCAAAGCCATCAGCAATTGCGGGCTGGGGAGTTGTAGTAGATCCACTTAAAACAAACTGGGGTTGAGGTGCCATTTTAAAAACACGA
TGCTTAAGAACAGTATAGGCATCAGTATTAAATGGATAAGTAGATCTCAATAAAGTTCCATCAAAGGGTAATGCACTGCCTGGGGCAGTG

>000063F|arrow

CCAAACCACAAAAACACGATAAACAAGGACAGAAAAAATGATGCGTCGCAGACCAGCAAATAAGCAAAAGTCCGCTAGGACTTTCCGTAAACA
TGCTTCACATACAAAACACGCAAATATGCGAAACTCGCCAATGCGTGGAGGCTGGAGACTCTAATAAAGTCCCCAGGCACCTCACATGCCTTGTT
ATCACCCTCTCAAAGCATTTCAATGCTTTGACAAATCAATTGTTTTCGACGAAGTTCGGAAACATGACATCGTTCGATCTTTAGACCTGCCCTGTG
GGCAGTGCGTTGGATGCCGTCTAGAACGATCAAGACAATGGGCTATTCGGTGCATGCACGAAGCCCAATTGCATAAAAACAACTCATTCATAAC
ACTCACATATGACAATACACATCTCCCAAGCGATGGCTCTTTGGATCACAAAGACTTTCAACTGTTCCTTAAAAGACTTAGAAAAACTCTCGCAAA

AAGAGGACTTACAATCCGCTATTACATGGCTGGAGAATATGGTGAACTCTTCGCAAGACCCCACTTCCATGCCTGTATCTTCGGATACGACTTTCC
TGATAAAAAATTATGGAAAAGGACTGCCTCTGGTTCTATGTTATATAGATCCGCAGAACTTGAAGCTCTCTGGCCATTTGGTTATACCACCATTGG
AGATGTTACTTTCGAATCAGCCGCCTACGTGGCTAGATACATAATGAAAAAACAAACAGGGAAAGATGCGGAATCTCATTACAAACGCATACAC
CCTGAAACCGGCGAATATTTAGACTTAAAGCCGGAATATAATAAAATGTCTTTAAAACCGGGAATCGGTAAAGACTTTTATATAAAATATACTTC
GGATATATACCCGCAAGACTACGTAATACTTAGAGGTAAAAAGGTCAAACCACCAAAATACTATGACAAAATGTTTAAAATTGACCAACCTTATG
AGTATGACGAATTACTTTACATGCGGGAAAATAACGCTAAACTTAATTCCGAAGACAATACACCAGAACGACTATCTGCAAAAGAACAAGTAAC
TATGGCAAAACTTCAACTATTAAAACGTAACCTCACTTAGGAAAATAATGAAACTTATCCTCGCTTCCGTAAAAGACCGTGCTGCTGAAGCATAT
GCACGACCAATGTTCGTACCTTCCTCTGGAGTAGCTATACGCTCTTTTTCAGATGAAATTAATCGTTCTGATACTGAAAATCAACTTTTTAATCACC
CTGATGACTTCGATCTATATGAATTCGGAACTTTTGACGATTCAACTGGGTTATTCGATTTACATGAACAACCAAAACTCCTATCATTAGGAAAAC
AAGTTAAACTTAAATAAAACAACCGAGGGGAAAAGAGATTTATCTTTCCCCCGGAACAACACTAAGGAAAAACATGCACCGCAATCAGTCAGTT
AATACTCACCGCTTCGCGATGGTACCTAGAGCCGATATACCACGTAGTAAATTCGATGCTCAAAAAACACATAAAACGACTTTCGATGCGGGCTA
TTTAATTCCTGTATATGTTGATGAAGTGCTCCCTGGGGACACTTTCAACTTAAAAATGACGGCATTTGCCCGTCTAGCAACGCCTTTATATCCAATC
ATGGACAACATGATTATGGATTCTTTCTTTTTCTTTGTACCCAATCGCCTTATATGGAATAACTGGCAAAAATTTATGGGTCAACAAGAAAATCCA
ACAGACTCAATATCTTATATTGTCCCAACTCAAACAAGCCCAACAGATGGTTATGCCGTAGGCAGCCTTCAAGACTATATGGGCTTACCAACAGT
AGGCCAAATTGATACTGGCCGAACTATTACGCACTGTGCCTTTTGGCCACGTGCATACAATCTTATTTGGAACGAATGGTTCCGAGATGAAAATT
TACAAACAAGCGCAGTAGTTGATAAGGGCGATGGCCCTGATACTTCCTCAAACTATGTGCTAAAACGTCGTGGTAAAAGACATGATTACTTTACG
TCAGCATTACCATGGCCACAAAAAGGTGCGAGTGTCACCTTACCTTTAGGTACTACGGCTCCAATTAAATGGGATACCATTTCAGGAGACGCAAC
ATCAAACGATAAATTTACGGTAATTCAAACAGATCCTGGAAATACGACTGCTTTAGCTAGATATGGCAACGCTTATGGTGTTAATACTGCTGGTG
TAGTAAATAACGTTTCTAATTTATATACCGACTTATCAGAAGCAACTGCTGCAACTGTCAATCAATTAAGACAGTCATTTCAAATTCAAAAATTACT
TGAAAGGGATGCACGTGGCGGAACACGATACACAGAAATTATCCGGAGTCACTTTGGAGTTATTTCCCCAGACGCCCGTTTACAAAGGCCTGAA
TACCTTGGAGGCGGTTCAACACCAATTAATGTTAATCCGATTGCTCAAACGTCGGGAACAAACGCTTCTGGAACGACTACCCCTTTGGGCAACCT
TGCTGCTATGGGTACTGCTCTCGCTCATAATCATGGATTTACTCAATCATTTACTGAGCATGGCGTTATTATTGGATTAGTATCCATTAGAGCAGA
TCTTACTTATCAACAAGGATTAGACCGTATGTGGTCTAGATCTACACGATATGACTTTTATTTCCCAGCATTTGCTACTCTAGGCGAACAATCTGTT
TTGCAAAAAGAAATTTATGCAACAGGAGATACTGCAGCCGACAATACTGTTTTTGGATATCAAGAACGCTGGGCGGAATATCGTTACAAACCAT
CTAAAATTACTGGTTTGTTCAAATCAACATCGGCGGGCACGATCGATGGTTGGCATTTGGCTCAAAAATTTACCGCTGCGCCTACTTTGAATAATA
CGTTTATTCAAGATACGCCTCCTGTATCACGTGTAGTAGCCGTTGGAGCAGCTGCAAATGGCCAACAATTCTTATTTGACTCATTTTTTGATGTCA
AAATGGCAAGACCAATGCCAATGTATTCAGTACCTGGCTTAATAGACCATTTCTAATGGGACTATTTGACGGAATTGCCGATTTAATCGGCCCTG
CTATAGCTATAGGAGCTGCCCCTGCTACTGGGGGACTCTCCTTAGCTGCACTTGCACCTGCAGCAATAGGTGCAGCAGGACAATACTTTGGAACA
CAAAGTCAAAACGCAGCGAGTGCAGAACAAGCGAGTAATCAACAGAGATTTCAAGCTGAAATGTCTGGAACATCATATCAACGAGCAGTTGAA
GATATGAAAAAAGCTGGGTTAAATCCCATGCTTGCGTATTCACAAGGCGGAGCCACAACACCAGCTGGAGCTATGGCCCAGATGCAAAATGTTC
TCGGTAATGCAACTACGTCCGGAACCCAAGCTTATCAAACGGTTGCTCAAGCAAATCAAGCTATTGCTCAATCTAAACAAATTGAAGCTCAAACA
GAACTCACAAGTAATCAAACAGATAATGTACGTGCTGATACGTTAAACAAATTGGATGAAAATCCAAATATTAGAGCTCAATATAAACAAATACT
TGCCGATACTTTCATGAAAAATGAAATAGGCAAAACATCAAGTGCTCAAGCTGCTCAAGCTTTGGCACAATCTCGTTATTCAAACGAGTTAACAA
AACTTGCTAAATCAGGGTCAGCTCCTAGTTCTAGCAAACCAATTTATCAAGACGTAAAAAATATCGCCAAAGATGCGTATAGCGCATCTGGCGCA
AAACGATACATCGATAACTATCGAGGTCAACCGATTCAACAAAATCGTACAAATAACCAACCACCAATGGAATGAAAATGACAAAGATTACAGC
CCCATTTCTTCGTACTCCGTACAATTACGACACGATTGCTGCGTCAAATGAGTCAGGGCTGCATTGTGAGGATGCAACTCTGACTCAGCAGCAAT
TTGCTGAAGAATGCGATATTAATAATATTATGGAAAAGTTTGGTATGACCGGACTTATTCCTCAAACTCCTTTAACGCCTCAATATGGCGACTTTA
GTGGTGTCTATGACTACCACTCTGCTCTGAACCAGATTATGGCTTCAGACAACGAATTTATGGCTTTACCAGCCAATATTCGTGAACGATTCGCTA
ATGATCCCGCGAATCTAATAGATTTTCTAGAAAACCCTGAAAATCGCAGCGAAGCTGAAAAAATGGGACTGGTAAAACCAGCCCAAACCGAGGT
TTCAACCCCTGTTGGAACCTCGGAAGCACAGTTACCTACTTGATGTAACTGTGCTAGGTGACA

>000035F|arrow

CTCGGAAGCACAGTTACCTACTTGATGTAACTGTGCTAGGTGACACCAAACCACAAAAACACGATAAACAAGGACAGAAAAAAATGATGCGTCG
CAGACCAGCAAATAAGCAAAAGTCCGCTAGGACTTTCCGTAAACATGCTTCACATACAAAACACGCAAATATGCGAAACTCGCCAATGCGTGGA
GGCTGGAGACTCTAATAAAGTCTTCAGGCACCTCACATGCCTTGTTATCACCCTCTCAAAGCATATCAATGCTTTGACAAATCAATTGTTTTCGAC
GAAGTTCGGAAACATGACATCGTTCGATCTTTAGACCTGCCCTGTGGGCAGTGCGTTGGATGCCGTCTAGAACGATCAAGACAATGGGCTATTC
GGTGCATGCACGAAGCCCAATTGCATAAAAACAACTCATTCATAACACTCACATATGACAATACACATCTCCCAAGCGATGGCTCTTTGGATCAC
AAAGACTTTCAATTGTTCCTTAAAAGACTTAGAAAAACTCTCCGCAAAAAGAGGACTTACAATCCGCTATTACATGGCTGGAGAATATGGTGAAC
TCTTCGCAAGACCCCACTTCCATGCCTGTATCTTCGGATACGACTTTCCTGATAAAAAATTATGGAAAAGGACTGCCTCTGGTTCTATGTTATATA
GATCCGCAGAACTTGAAGCTCTCTGGCCATTTGGTTATACCACCATTGGAGATGTTACTTTTGAATCAGCCGCCTACGTGGCTAGATACATAATG
AAAAAACAAACAGGGAAAGATGCGGAATCTCATTACAAACGCATACACCCTGAAACCGGCGAATATTTAGACTTAAAGCCGGAATATAATAAAA
TGTCTTTAAAACCGGGAATCGGTAAAGACTTTTATATAAAATATACTTCGGATATATACCCGCAAGACTACGTAATACTTAGAGGTAAAAAGGTC

AAACCACCAAAATACTATGACAAAATGTTTAAAATTGACCAACCTTATGAGTATGACGAATTACTTTACATGCGGGAAAATAATGCTAAATTTAAT
TCCGAAGACAATACACCAGAACGACTATCTGCAAAAGAACAAGTCACTATGGCAAAACTTCAACTATTAAAACGTAACCTTACTTAGGAAAATAA
TGAAACTTATCCTCGCTTCCGTAAAAGACCGTGCTGCTGAAGCATATGCACGACCAATGTTCGTACCTTCCTCTGGAGTAGCTATACGCTCTTTTT
CAGATGAAATTAATCGTTCTGATACTGAAAATCAACTCTTTAAATCACCCTGATGATTTCGATCTATATGAATTCGGAACATTTGACGATTCAACT
GGGTTATTCGATTTACATGAACAACCAAAACTCCTATCATTAGGAAAACAAGTTAAACTTAAATAAAACAACCGAGGGGAAAAGAGATTTATCTT
TCCCCCGGAACAACACTAAGGAAAAACATGCACCGCAATCAGTCAGTTAATACTCACCGCTTCGCGATGGTACCTAGAGCCGATATACCACGTAG
TAAATTCGATGCTCAAAAAACACATAAAACGACTTTCGATGCGGGCTATCTAATTCCTGTATATGTTGATGAAGTGCTCCCTGGGGACACTTTCAA
CTTAAAAATGACGGCATTTGCCCGTCTAGCAACGCCTTTATATCCAATCATGGATAACATGATTATGGATTCTTTCTTTTTCTTTGTACCCAATCGC
CTTATATGGAATAACTGGCAAAAATTTATGGGTCAACAAGAAAATCCAACAGACTCAATATCTTATATTGTCCCAACACAAACAAGCCCAACAGA
TGGTTATGCCGTAGGCAGCCTTCAAGACTATATGGGCTTACCAACAGTAGGCCAAATTGATACTGGCCGAACTATTACGCACTGTGCCTTTTGGC
CACGTGCATACAATCTTATCTGGAACGAATGGTTCCGAGATGAAAATTTACAAACAAGCGCAGTAGTTGATAAGGGCGATGGCCCTGATACTTC
CTCAAACTATGTGCTAAAACGTCGTGGTAAAAGACATGATTACTTTACGTCAGCATTACCATGGCCACAAAAAGGTGCGAGTGTCACCTTACCTT
AGGTACTACGGCTCCAATTAAATGGGATACCATTTCAGGAGACGCAACATCAAACGATAAATTTACGGTAATTCAAACAGATCCTGGAAATACG
ACTGCTTTAGCTAGATATGGCAACGCTTATGGTGTTAATACTGCTGGTGTAGTAAATAACGTTTCTAATTTATATACCGACTTATCAGAAGCAACT
GCTGCAACTGTCAATCAATTAAGACAGTCATTTCAAATTCAAAAAATTACTTGAAAGGGATGCACGTGGCGGAACACGATACACAGAAATTATCC
GGAGTCACTTTGGAGTTATTTCCCCAGGACGCCCGTTACAAAGGCCTGAATACCTTGGAGGCGGTTCAACACCAATTAATGTTAATCCGATGCTC
AAAACGTCGGGAACAAACGCTTCTGGAACGACTACCCCTTTGGGCAAACCTTGCTGCTATGGGTACTGCTCTCGCTCATAATCATGGATTTACTC
AATCATTTACTGAGCATGGCGTTATATTGGATTAGTATCCATTAGAGCAGATCTTACTTATCAACAAGGATTAGACCGTATGTGGTCTAGATCTAC
ACGATATGACTTTTATTTCCCAGCATTTGCTACTCTAGGCGAACAATCTGTTTTGCAAAAAGAAATTTAATGCAACAGGAGATACTGCAGCCGAC
AATACTGTTTTTGGATATCAAGAACGCTGGGCGGAATATCGTTACAAAACCATCTAAAATTACTGGTTTGTTTCAAATCAACATCGGCGGGCACG
ATCGCTGGTTGGCATTTGGCTCAAAAATTTACCGCTGCGCCTACTTTGAATAATACGTTTATTCAAGATACGCCTCCTGTATCACGTGTAGTAGCC
GTTGGAGCAGCTGCAAATGGCCAACAATTCTTATTTGACCTCATTTTTTGATGTCAAAATGGCAAGACCAATGCCAATGTATTCAGTACCTGGCTT
AATAGACCATTTCTAATGGGACTATTTGACGGAATTGCCGATTTAATCGGCCCTGCTATAGCTATAGGAGCTGCCCCTGCTACTGGGGGACTCTC
CTTAGCTGCACTTGCACCTGCAGCAATAGGTGCAGCAGGACAATACTTTGGAACACAAAGTCAAAACGCAGCGAGTGCAGAACAAGCGAGTAA
TCAACAGAGATTTCAAGCTGAAATGTCTGGAACATCATATCAACGAGCAGTTGAAGATATGAAAAAAGCTGGGTTAAATCCCATGCTTGCGTATT
CACAAGGCGGAGCCACAACACCAGCTGGAGCTATGGCCCAGATGCAAAATGTTCTCGGTAATGCAACTACGTCCGGAACCCAAGCTTATCAAAC
GGTTGCGCAAGCAAATCAGCTATTGCTCAATCTAAACAAATTGAAGCTCAAACAGAACTCACAAGTAATCAAACAGATAATGTACGTGCTGATAC
GTTAAATAAATTGGATGAAAATCCAAATATTAGAGCTCAATATAAACAAATACTTGCCGATACTTTCATGAAAAATGAAATAGGCAAAACATCAA
GTGCTCAAGCTGCTCAAGCTTGGCACAATCTCGTTATTCAAACGAGTTAACAAAACTTGCTAAATCAGGGTCAGCTCCTAGTTCTAGCAAACCAA
TTTATCAAGACGTAAAAAACATCGCCAAAGATGCGTATAGCGCATCTGGCGCAAAACGATACATCGATAACTATCGAGGTCAACCGATTCAACA
AAATCGTACAAATAACCAACCACCAATGGAATGAAAATGACAAAGATTACAGCCCCATTTCTTCGTACTCCGTACAATTACGACACGATTGCTGC
GTCAAATGAGTCAGGGCTGCATTGTGAGGATGCAACTCTGACTCAGCAGCAATTTGCTGAAGAATGTGATATCAATAATATTATGGAAAAGTTC
GGTATGACCGGACTTATTCCTCAAACTCCTTTAACGCCTCAATATGGCGACTTTAGTGGTGTCTATGACTACCACTCTGCTCTGAACCAGATTATG
GCTTCAGACAACGAATTTATGGCTTTACCAGCCAATATTCGTGAACGATTCGCTAATGATCCCGCGAATCTAATAGATTTTCTAGAAAACCCTGAA
AATCGCAGCGAAGCTGAAAAAATGGGACTGGTAAAACCAGCCCAAACCGAGGTTTCAACCCCTGTTGGAAC

>000209F|arrow

GCGATTTTAGTGGTGTTACTGATTACCATTCTGCGTTGAATCAAATTAACGCAACTATGGACGATTTCATGGCTCTGCCAGCGAAATTAAGAGTCC
GATTTGACCATGATCCTGTCAAATTATTGGAGTTCCTTGAGAACGACCAGAATCGTGATGAAGCGATTCAATTGGGTCTTATTGATGGACAACCT
GTGGTTGAACCCATCGTTTCTACAGAAACACCTAAGGCCGAAGGATGAAATCCTGAGGCCAGCACAGTTACTCTACTTGATGTAACTGTGCTAG
GTGACACCAAACCACTATTTTAACTACGGAGTTCATCATGTTACGAAGAAAGCCAGTAAACAAATATAAATCTGCAAAGTCATTTCGCAGAACTG
CTAGTAAGACGAAGTCAATTAATATGAGACACGCTCCCCAGCGTGGTGGCTATCGTTTGTAATTATGGCCTGTTATAAGCCCTTAACGGCTTATC
AATGCAGTGACAGGTCTATAATTTGGCGGGAAATACCGGGTGCGGATGTAGTCCGTACCCTATCATTGCCTTGTGGTCAGTGTGTTGGTTGTCG
CCTTGAACGCTCACGTCAGTGGGCGATTCGTTGTATGCATGAGGCACAAATGCATACTAGTAATTGTTTTATTACTTTGACATATGCTCCAGAGCA
TTGTCCTAAGGATATGTCATTGGATTACAATGATTATCAGCTTTTTATGAAGCGGTTACGTAAGCGTTTTACTGGGAAAACGATACGTTTTTATAT
GGCAGGTGAATATGGTGAATCTTTTGATCGTCCTCATTTCCATGCTTGTCTGTTTGGTCTTGATTTTCCGGATAAGAAAATATTTAAAAGAACGCA
GACTGGCTCTATCCTCTACACGTCAGAGATTTTGGAAGAATTGTGGCCGTTTGGCTATTCTACAATTGGTGATGTTACTTTTGAGTCTGCTGCTTA
TGTTGCAAGATATATTATGAAGAAGATTAATGGGGTTACTGTCAATGAAAACCACGAAGTGGTTGATGCGGGTGCCCATTATCAATATTGTGATT
TAGAGACTGGTGAGATAATTCAGCGTAAGCCAGAATTTAATAAGATGTCATTGAAGCCCGGTATCGGGCAATCGTGGTTAGATAAGTACATGTC
AGACGTTTATACGTCAGACCACGTTGTGGTGCGTGGCAAAAGTGCCGTCCACCACGGTTTTATGATAATAAATTTAAGTTGAAGTTTCCTGAAGA
ATTCGATATGATTCAGTTTGCCAGAGAGATGGAAGGTCGATCTAGGCATGAGGACAACACGCTTGAGCGACTTGCTGTTAAGGAAAAAGTTGC
GTTGGCTAAGTTGTCATTGTTAAAACGTACTATTTAAGGAGTTTTTATGAATGGTTATTGTTTCTATTAAGGATACTGCTGCAGATGCTTTTGGTC

GTCCAGCTTATGTTGCATCTGAAGGTGTTGCAGTACGTCAGTTTCAGGATGAAGTCAATCGAGCTAGCGAAGATAATCAGTTGTATAAACATCCT
GATGATTTTCATATGTTCTATTTGGGTCTTTTTGACGATGCCACTGGTGTTTTTGAACTACTGGAAAGCCCTAAGTTGATTGCTCGTGCAAAAGAT
GTAATGATTCGCGAAGGCGAGTAAGGTTTTTTTATACCGTATCACTCGAAAGAGTGGTACGGAACTACGGGAGATGTTTATGTTTCGCAATAA
GTCAGTAAGTACGCATTCATTTGCTATGGTTCCTAAAGCGGACATTCCCCGCTCTAGTTTTAATACTCAATATGCTCATAAAACCACGTTTGATGCT
GGTTTTTTAGTTCCTATTTATTGTGATGAAGTATTGCCTGGCGATACTCATCGTGTAAAGATGACTGCATTTGCACGTTTGGCCCACCGTTATTTCC
TGTGATGGACAACTTGCATCTTGATACTTTCTTTTTCTTTGTACCTAATCGTTTACTTTGGAACAATTGGCCAAAGTTTATGGGTGAACAAACGAAT
CCTGGTGATTCTATTTCTTTTGTAGTGCCTACTATTACTAGTCCTGCTGGTGGTTATGCTGTTTGTTCAATTTTTGATTATTTTGGTTTACCTACTGCT
GGTCAGATTACTGGCGCTAATACAGTAACGCATAATGTTTTGCCGTTACGTGCTTATAATGAGATTTATAACGAATGGTTTAGAGATGAAAACTT
ACAGAATTCTGTAACGTTAAATCTTGGTGATTCAGGTGATGTTCCTGCTAACTATACACTTTTGAGACGTGGTAAGCGTAAAGATTATTTTACTGG
TGCATTGCCTTGGCCACAGAAGGGTGCTTCTGTTTCTTTACCGTTAGGAACACGTGCTAATATTTATTCTGACATACCAGCTGGCAATGGTACTGC
TGGTTATAGTGTTTTTCAAACTGCTGTTGGTGCTTTAAGAGAATTAAATTCAGCTTCTAATACTTTGTCTAATAGTACAAATGCTGGTGTTGCTACT
ACAGTTATACGCTGATTTGTCTACTGCTACTGCTGCGACTATTAACCAACTTCGTCAATCTTTCCAGATTCAGAGTTATTGGAGCGCGATGCACGT
GGTGGTACTCGTTATACTGAGTTACTACGTGCTCACTTTGGAGTAACTCCACAGGATTATCGTTTACAACGTCCTGAATATATTGGTGGAGGTTC
GACCCTTGTTAATGTTAATCCGATTGCTCAGACTTCTGCAACGTCGGTTACTGGTTCTGCTACTCCGCAAGGTAACCTTGCTGCAATGGGTACTGC
ATTGGCTCAGGGACACGGCTTTACGTATGCTGCTCAAGAACATGGATACATTATCGGATTAGTTTCTGTACGTGCTGACCTCACATATCAACAGG
GTCTTCCTAAGATGTGGTCTAGGTCTACACGATATGACTTTTATTTCCCAGTATTTGCCACTTTGGGTGAGCAAGCTATTTTGAACAAAGAATTTA
TGTTCAAGGTACTGCAGCCGACAATGATGTATTTGGTTATCAAGAACGTTGGGCGGAGTATCGTTACAAACCTTCTCAAATTACTGGTTTCTTTAG
GTCTACTTCTGCTGGCACTATTGATGCTTGGCATTATGGACAGCGATTTACTTCTCTTCCTACGTTGAATTCAACGTTTATTCAAGAGACCCCTCCA
GTTGCTCGTACTACGGCGGTCGGAGCTGCAGCAAATGGTCAGCAATTTTTAATGGATGCTTTCTTTGATTGTCAGATGGCCAGACCTATGCCTAT
GTACAGCGTACCTGGTCTAATTGATCATTTCTAATGTTTTATATAACCTCGACTACTCCGTAAGGAGTAGTGAGGAAACAACCGAAGGGCGTTAG
TTTATGTTTGGTGGAATACTTGATGCGGTTACTAATGTTGGTTCTAAGCTGTCTTCAGCTTCTAGTTTCTTTACTCCTGGTCGTCTGGTACTGCTTT
GGGCGCTGTTGGTTCTTATTTAGGTTCTACTTCTGCTAATAAAGCTAATCAGAGATGGCTCAGAGGCAAATGGATTTTCAAGCCGATATGAGTGG
AACAAGTTACCAGCGTGCTGTTAAAGATTTAGAAGCTGCTGGTTTATCTCCTATGTTAGCCTATCAACGTGGTGGTGCTTCTACCCCATCTGGTTC
AACTGCTACTATGGAAAATGTTTTAGGTAATGCAACTAATTCAGCTATTAATACTGCTTCTATGATGCAACAGATTCGTAATGCATCAGAAACAGA
AAAGCAGATTATCGCCCAGACTGAAGCTACTGAAGCTGGTACCGCTAATACTAGGGCTGATACTGTTAATAAGTTGCTTACTGCTCCTAATATTA
CAGCCGAAAATAAACGTATTTTGGCTGATATTGCTTTAAAGAATACGACTGCGGATTTAACATCCGCTCAGTCATATAATACTAAGAGGCTATTG
GCTCCATCCCCAGCTATTTGGTCTAGGGTATCGATGCTTCGAAAGAAATTTTTGATAAACTCAAAAATAATCCTAATCAACTAACCCCTTGGGGAA
TTGGAGTCAAATAATGAGTAAAGCGAATTTGCCATTTGTACGTAATCCGTACAACTATGATAAAGATGAAGCATCGGTAAACGATGCGTTGCTGT
GTCAAGACCCAAGTCTTGCTCAACAGCATATGAAAGATGAATGTGACATTAATGTCATCATTGAACGTTTCGGGTTACAGGGGAACTTCCAACG
GCCCCTGTATCGCCTCAATACG

>000125F|arrow

GAGGTGCCTGAAGACTTTATTAGAGTCTCCAGCCTCCACGCATTGGCGAGTTTCGCATATTTGCGTGTTTTGTATGTGAAGCATGTTTACGGAAA
GTCCTAGCGGACTTTTGCTTATTTGCTGGTCTGCGACGCATCATTTTTTCTGTCCTTGTTTATCGTGTTTTTGTGGTTTGGTGTCACCTAGCACAGTT
ACATCAAGTAGGTAACTGTGCTTCGAGGTTCCAACAGGGGTTGAAACCTCGGTTTGGGCTGGTTTTACCAGTCCCATTTTTTCAGCTTCGCTGCG
ATTTTCAGGGTTTTCTAGAAAATCTATTAGATTCGCGGGATCATTAGCGAATCGTTCACGAATATTGGCTGGTAAAGCCATAAATTCGTTGTCTGA
AGCCATAATCTGGTTCAGAGCAGAGTGGTAGTCATAGACACCACTAAAGTCGCCATATTGAGGCGTTAAAGGAGTTTGAGGAATAAGTCCGGTC
ATACCGAACTTTTCCATAATATTATTGATATCACATTCTTCAGCAAATTGCTGCTGAGTCAGAGTTGCATCCTCACAATGCAGCCCTGACTCATTTG
ACGCAGCAATCGTGTCGTAATTGTACGGAGTACGAAGAAATGGGGCTGTAATCTTTGTCATTTTCATTCCATTGGTGGTTGGTTATTTGTACGATT
TTGTTGAATCGGTTGACCTCGATAGTTATCGATGTATCGTTTTGCGCCAGATGCGCTATACGCATCTTTGGCGATGTTTTTTTACGTCTTGATAAAT
TGGTTTGCTAGAACTAGGAGCTGACCCTGATTTAGCAAGTTTTGTTAACTCGTTTGAATAACGAGATTGTGCCAAAGCTTGAGCAGCTTGAGCAC
TTGATGTTTTGCCTATTTCATTTTTCATGAAAGTATCGGCAAGTATTTGTTTATATTGAGCTCTAATATTTGGATTTTCATCCAATTTATTTAACGTA
TCAGCACGTACATTATCTGTTTGATTACTTGTGAGTTCTGTTTGAGCTTCAATTTGTTTAGATTGAGCAATAGCTTGATTTGCTTGCGCAACCGTTT
GATAAGCTTGGGTTCCGGACGTAGTTGCATTACCGAGAACATTTTGCATCTGGGCCATAGCTCCAGCTGGTGTTGTGGCTCCGCCTTGTGAATAC
GCAAGCATGGGATTTAACCCAGCTTTTTCATATCTTCAACTGCTCGTTGATATGATGTTCCAGACATTTCAGCTTGAAATCTCTGTTGATTACTCGC
TTGTTCTGCACTCGCTGCGTTTTGACTTTGTGTTCCAAAGTATTGTCCTGCTGCACCTATTGCTGCAGGTGCAAGTGCAGCTAAGGAGAGTCCCCC
AGTAGCAGGGGCAGCTCCTATAGCTATAGCAGGGCCGATTAAATCGGCAATTCCGTCAAATAGTCCCATTAGAAATGGTCTATTAAGCCAGGTA
CTGAATACATTGGCATTGGTCTTGCCATTTTGACATCAAAAAATGAGTCAAATAAGAATTGTTGGCCATTTGCAGCTGCTCCAACGGCTACTACAC
GTGATACAGGAGGCGTATCTTGAATAAACGTATTATTCAAAGTAGGCGCAGCGGTAAATTTTTGAGCCAAATGCCAACCATCGATCGTGCCCGC
CGATGTTGATTTGAACAAACCAGTAATTTTAGATGGTTTGTAACGATATTCCGCCCAGCGTTCTTGATATCCAAAAACAGTATTGTCGGCTGCAGT
ATCTCCTGTTGCATAAATTTCTTTTTGCAAAACAGATTGTTCGCCTAGAGTAGCAAATGCTGGGAAATAAAAGTCATATCGTGTAGATCTAGACCA
CATACGGTCTAATCCTTGTTGATAAGTAAGATCTGCTCTAATGGATACTAATCCAATAATAACGCCATGCTCAGTAAATGATTGAGTAAATCCATG

ATTATGAGCGAGAGCAGTACCCATAGCAGCAAGGTTGCCCAAAGGGGTAGTCGTTCCAGAAGCGTTTGTTCCCGACGTTTGAGCAATCGGATTA
ACATTAATTGGTGTTGAACCGCCTCCAAGGTATTCAGGCCTTTGTAAACGGGCGTCTGGGGAAATAACTCCAAAGTGACTCCGGATAATTTCTGT
GTATCGTGTTCCGCCACGTGCATCCCTTTCAAGTAATTTTTGAATTTGAAATGACTGTCTTAATTGATTGACAGTTGCAGCAGTTGCTTCTGATAA
GTCGGTATATAAATTAGAAACGTTATTTACTACACCAGCAGTATTAACACCATAAGCGTTGCCATATCTAGCTAAAGCAGTCGTATTTCCAGGATC
TGTTTGAATTACCGTAAATTTATCGTTTGATGTTGCGTCTCCTGAAATGGTATCCCATTTAATTGGAGCCGTAGTACCTAAAGGTAAGGTGACACT
CGCACCTTTTTGTGGCCATGGTAATGCTGACGTAAAGTAATCATGTCTTTTACCACGACGTTTTAGCACATAGTTTGAGGAAGTATCAGGGCCATC
GCCCTTATCAACTACTGCGCTTGTTTGTAAATTTTCATCTCGGAACCATTCGTTCCAGATAAGATTGTATGCACGTGGCCAAAAGGCACAGTGCGT
AATAGTTCGGCCAGTATCAATTTGGCCTACTGTTGGTAAGCCCATATAGTCTTGAAGGCTGCCTACGGCATAACCATCTGTTGGGCTTGTTTGTGT
TGGGACAATATAAGATATTGAGTCTGTTGGATTTTCTTGTTGACCCATAAATTTTTGCCAGTTATTCCATATAAGGCGATTGGGTACAAAGAAAAA
GAAAGAATCCATAATCATGTTATCCATGATTGGATATAAAGGCGTTGCTAGACGGGCAAATGCCGTCATTTTTAAGTTGAAAGTGTCCCCAGGG
AGCACTTCATCAACATATACAGGAATTAGATAGCCCGCATCGAAAGTCGTTTTATGTGTTTTTTGAGCATCGAATTTACTACGTGGTATATCGGCT
CTAGGTACCATCGCGAAGCGGTGAGTATTAACTGACTGATTGCGGTGCATGTTTTTCCTTAGTGTTGTTCCGGGGGAAAGATAAATCTCTTTTCC
CCTCGGTTGTTTTATTTAAGTTTAACTTGTTTTCCTAATGATAGGAGTTTTGGTTGTTCATGTAAATCGAATAACCCAGTTGAATCGTCAAATGTTC
CGAATTCATATAGATCGAAATCATCAGGGTGATTAAAGAGTTGATTTTCAGTATCAGAACGATTAATTTCATCTGAAAAAGAGCGTATAGCTACT
CCAGAGGAAGGTACGAACATTGGTCGTGCATATGCTTCAGCAGCACGGTCTTTTACGGAAGCGAGGATAAGTTTCATTATTTTCCTAAGTAAGGT
TACGTTTTAATAGTTGAAGTTTTGCCATAGTGACTTGTTCTTTTGCAGATAGTCGTTCTGGTGTATTGTCTTCGGAATTAAATTTAGCATTATTTTCC
CGCATGTAAAGTAATTCGTCATACTCATAAGGTTGGTCAATTTTAAACATTTTGTCATAGTATTTTGGTGGTTTGACCTTTTTACCTCTAAGTATTA
CGTAGTCTTGCGGGTATATATCCGAAGTATATTTTATATAAAAGTCTTTACCGATTCCCGGTTTTAAAGACATTTTATTATATTCCGGCTTTAAGTC
TAAATATTCGCCGGTTTCAGGGTGTATGCGTTTGTAATGAGATTCCGCATCTTTCCCTGTTTGTTTTTTCATTATGTATCTAGCCACGTAGGCGGCT
GATTCGAAAGTAACATCTCCAATGGTGGTATAACCAAATGGCCAGAGAGCTTCAAGTTCTGCGGATCTATATAACATAGAACCAGAGGCAGTCC
TTTTCCATAATTTTTTATCAGGAAAGTCGTATCCGAAGATACAGGCATGGAAGTGGGGTCTTGCGAAGAGTTCACCATATTCTCCAGCCATGTAA
TAGCGGATTGTAAGTCCTCTTTTTGCGAGAGTTTTTCTAAGTCTTTTAAGGAACAATTGAAAGTCTTTGTGATCCAAAGAGCCATCGCTTGGGAGA
TGTGTATTGTCATATGTGAGTGTTATGAATGAGTTGTTTTTATGCAATTGGGCTTCGTGCATGCACCGAATAGCCCATTGTCTTGATCGTTCTAGA
CGGCATCCAACGCACTGCCCACAGGGCAGGTCTAAAGATCGAACGATGTCATGTTTCCGAACTTCGTCGAAAACAATTGATTTGTCAAAGCATTG
ATATGCTTTGAGAGGGTGATAACAAGGCATGT

>000075F|arrow

TGACCTCGATAGTTATCGATGTATCGTTTTGCGCCAGATGCGCTATACGCATCTTTGGCGATGTTTTTACGTCTTGATAAATTGGTTTGCTAGAAC
TAGGAGCTGACCCTGATTTAGCAAGTTTTGTTAACTCGTTTGAATAACGAGATTGTGCCAAAGCTTGAGCAGCTTGAGCACTTGATGTTTTGCCT
ATTTCATTTTTCATGAAAGTATCGGCAAGTATTTGTTTATATTGAGCTCTAATATTTGGATTTTCATCCAATTTGTTTAACGTATCAGCACGTACATT
ATCTGTTTGATTACTTGTGAGTTCTGTTTGAGCTTCAATTTGTTTAGATTGAGCAATAGCTTGATTTGCTTGAGCAACCGTTTGATAAGCTTGGGTT
CCGGACGTAGTTGCATTACCGAGAACATTTTGCATCTGGGCCATAGCTCCAGCTGGTGTTGTGGCTCCGCCTTGTGAATACGCAAGCATGGGATT
TAACCCAGCTTTTTTCATATCTTCAACTGCTCGTTGATATGATGTTCCAGACATTTCAGCTTGAAATCTCTGTTGATTACTCGCTTGTTCTGCACTCG
CTGCGTTTTGACTTTGTGTTCCAAAGTATTGTCCTGCTGCACCTATTGCTGCAGGTGCAAGTGCAGCTAAGGAGAGTCCCCCAGTAGCAGGGGCA
GCTCCTATAGCTATAGCAGGGCCGATTAAATCGGCAATTCCGTCAAATAGTCCCATTAGAAATGGTCTATTAAGCCAGGTACTGAATACATTGGC
ATTGGTCTTGCCATTTTGACATCAAAAAATGAGTCAAATAAGAATTGTTGGCCATTTGCAGCTGCTCCAACGGCTACTACACGTGATACAGGAGG
CGTATCTTGAATAAACGTATTATTCAAAGTAGGCGCAGCGGTAAATTTTTGAGCCAAATGCCAACCATCGATCGTGCCCGCCGATGTTGATTTGA
ACAAACCAGTAATTTTAGATGGTTTGTAACGATATTCCGCCCAGCGTTCTTGATATCCAAAAACAGTATTGTCGGCTGCAGTATCTCCTGTTGCAT
AAATTTCTTTTTGCAAAACAGATTGTTCGCCTAGAGTAGCAAATGCTGGGAAATAAAAGTCATATCGTGTAGATCTAGACCACATACGGTCTAAT
CCTTGTTGATAAGTAAGATCTGCTCTAATGGATACTAATCCAATAATAACGCCATGCTCAGTAAATGATTGAGTAAATCCATGATTATGAGCGAG
AGCAGTACCCATAGCAGCAAGGTTGCCCAAAGGGGTAGTCGTTCCAGAAGCGTTTGTTCCCGACGTTTGAGCAATCGGATTAACATTAATTGGT
GTTGAACCGCCTCCAAGGTATTCAGGCCTTTGTAAACGGGCGTCTGGGGAAATAACTCCAAAGTGACTCCGGATAATTTCTGTGTATCGTGTTCC
GCCACGTGCATCCCTTTCAAGTAATTTTTGAATTTGAAATGACTGTCTTAATTGATTGACAGTTGCAGCAGTTGCTTCTGATAAGTCGGTATATAAA
TTAGAAACGTTATTTACTACACCAGCAGTATTAACACCATAAGCGTTGCCATATCTAGCTAAAGCAGTCGTATTTCCAGGATCTGTTTGAATTACC
GTAAATTTATCGTTTGATGTTGCGTCTCCTGAAATGGTATCCCATTTAATTGGAGCCGTAGTACCTAAAGGTAAGGTGACACTCGCACCTTTTTGT
GGCCATGGTAATGCTGACGTAAAGTAATCATGTCTTTTACCACGACGTTTTAGCACATAGTTTGAGGAAGTATCAGGGCCATCGCCCTTATCAAC
TACTGCGCTTGTTTGTAAATTTTCATCTCGGAACCATTCGTTCCAAATAAGATTGTATGCACGTGGCCAAAAGGCACAGTGCGTAATAGTTCGGCC
AGTATCAATTTGGCCTACTGTTGGTAAGCCCATATAGTCTTGAAGGCTGCCTACGGCATAACCATCTGTTGGGCTTGTTTGAGTTGGGACAATAT
AAGATATTGAGTCTGTTGGATTTCTTGTTGACCCATAAATTTTTGCCAGTTATTCCATATAAGGCGATTGGGTACAAAGAAAAAGAAAGAATCCA
TAATCATGTTGTCCATGATTGGATATAAAGGCGTTGCTAGACGGGCAAATGCCGTCATTTTTAAGTTGAAAGTGTCCCCAGGGAGCACTTCATCA
ACATATACAGGAATTAAATAGCCCGCATCGAAAGTCGTTTTATGTGTTTTTGAGCATCGAATTTACTACGTGGTATATCGGCTCTAGGTACCATCG
CGAAGCGGTGAGTATTAACTGACTGATTGCGGTGCATGTTTTTCCTTAGTGTTGTTCCGGGGGAAAGATAAATCTCTTTTCCCCTCGGTTGTTTTA

TTTAAGTTTAACTTGTTTTCCTAATGATAGGAGTTTTGGTTGTTCATGTAAATCGAATAACCCAGTTGAATCGTCAAAAGTTCCGAATTCATATAGA
TCGAAGTCATCAGGGTGATTAAAAAGTTGATTTTCAGTATCAGAACGATTAATTTCATCTGAAAAAGAGCGTATAGCTACTCCAGAGGAAGGTA
CGAACATTGGTCGTGCATATGCTTCAGCAGCACGGTTTTACGGAAGCGAGGATAAGTTTCATTATTTTCCTAAGTGAGGTTACGTTTTAATAGTT
GAAGTTTTGCCATAGTTACTTGTTCTTTTGCAGATAGTCGTTCTGGTGTATTGTCTTCGGAATTAAGTTTAGCGTTATTTTCCCGCATGTAAAGTAA
TTCGTCATACTCATAAGGTTGGTCAATTTTAAACATTTTGTCATAGTATTTGGTGGTTTGACCTTTTTACCTCTAAGTATTACGTAGTCTTGCGGGT
ATATATCCGAAGTATATTTTATATAAAAGTCTTTACCGATTCCCGGTTTTAAAGACATTATATTCCGGCTTTAAGTCTAAATATTCGCCGGTTTCAG
GGTGTATGCGTTTGTAATGAGATTCCGCATCTTTCCCTGTTTGTTTTTTCATTATGTATCTAGCCACGTAGGCGGCTGATTCGAAAGTAACATCTCC
AATGGTGGTATAACCAAATGGCCAGAGAGCTTCAAGTTCTGCGGATCTATATAACATAGAACCAGAGGCAGTCCTTTTCCATAATTTTTATCAGG
AAAGTCGTATCCGAAGATACAGGCATGGAAGTGGGGTCTTGCGAAGAGTTCACCATATTCTCCAGCCATGTAATAGCGGATTGTAAGTCCTCTTT
TTGCGAGAGTTTTCTAAGTCTTTTAAGGAACAGTTGAAAGTCTTTGTGATCCAAAGAGCCATCGCTTGGGAGATGTGTATTGTCATATGTGAGTG
TTATGAATGAGTTGTTTTATGCAATTGGGCTTCGTGCATGCACCGAATAGCCCATTGTCTTGATCGTTCTAGACGGCATCCAACGCACTGCCCACA
GGGCAGGTCTAAAGATCGAACGATGTCATGTTTCCGAACTTCGTCGAAAACAATTGATTTGTCAAAGCATTGAAATGCTTTGAGAGGGTGATAA
CAAGGCATGTGAGGTGCCTGGGGACTTTATTAGAGTCTCCAGCCTCCACGCATTGGCGAGTTTCGCATATTTGCGTGTTTTGTATGTGAAGCATG
TTTACGGAAAGTCCTAGCGGACTTTTGCTTATTTGCTGGTCTGCGACGCATCATTTTTTCTGTCCTTGTTTATGTTTTTGTGGTTTGGTGTCACCTA
GCACAGTTACATCAAGTAGGTAACTGTGCTTCCGAGGTTCCAACAGGGGTTGAAACCTCGGTTTGGGCTGGTTTTACCAGTCCCATTTTTTCAGC
TTCGCTGCGATTTTCAGGGTTTTCTAGAAAATCTATTAGATTCGCGGGATCATTAGCGAATCGTTCACGAATATTGGCTGGTAAAGCCATAAATTC
GTTGTCTGAAGCCATAATCTGGTTCAGAGCAGAGTGGTAGTCATAGACACCACTAAAGTCGCCATATTGAGGCGTTAAAGGAGTTTGAGGAATA
AGTCCGGTCATACCAAACTTTTCCATAATATTATTAATATCGCATTCTTCAGCAAATTGCTGCTGAGTCAGAGTTGCATCCTCACAATGCAGCCCTG
ACTCATTTGACGCAGCAATCGTGTCGTAATTGTACGGAGTACGAAGAAATGGGGCTGTAATCTTTGTCATTTTCATTCCATTGGTGGTTGGTTATT
TGTACGATTTTGTTGAATCGGT

>000100F|arrow

TTCGAGTGATACGGTATAAAAAAAACCTTACTCGCCTTCGCGAATCATTACATCTTTTGCACGAGCAATCAACTTAGGGCTTTCCAGTAGTTCAAA
AACACCAGTGGCATCGTCAAAAAGACCCAAATAGAACATATGAAAATCATCAGGATGTTTATACAACTGATTATCTTCGCTAGCTCGATTGACTT
CATCCTGAAACTGACGTACTGCAACACCTTCAGATGCAACATAAGCTGGACGACCAAAAGCATCTGCAGCAGTATCCTTAATAGAAACAATAACC
ATCTTCATAAAAACTCCTTAAATAGTACGTTTTAACAATGACAACTTAGCCAACGCAACTTTTTCCTTAACAGCAAGTCGCTCAAGCGTGTTGTCCT
CATGCCTAGATCGACCTTCCATCTCTCTGGCAAACTGAATCATATCGAATTCTTCAGGAAACTTCAACTTAAATTTATTATCATAAAACCGTGGTG
GACGGCACTTTTTGCCACGCACCACAACGTGGTCTGACGTATAAACGTCTGACATGTACTTATCTAACCACGATTGCCCGATACCGGGCTTCAAT
GACATCTTATTAAATTCTGGCTTACGCTGAATTATCTCACCAGTCTCTAAATCACAATATTGATAATGGGCACCCGCATCAACCACTTCGTGGTTTT
CATTGACAGTAACCCCATTAATCTTCTTCATAATATATCTTGCAACATAAGCAGCAGACTCAAAAGTAACATCACCAATTGTAGAATAGCCAAACG
GCCACAATTCTTCCAAAATCTCTGACGTGTAGAGGATAGAGCCAGTCTGCGTTCTTTTAAATATTTTCTTATCCGGAAAATCAAGACCAAACAGAC
AAGCATGGAAATGAGGACGATCAAAAGATTCACCATATTCACCTGCCATATAAAAACGTATCGTTTTCCCAGTAAAACGCTTACGTAACCGCTTC
ATAAAAAGCTGATAATCATTGTAATCCAATGACATATCCTTAGGACAATGCTCTGGAGCATATGTCAAAGTAATAAAACAATTACTAGTATGCATT
TGTGCCTCATGCATACAACGAATCGCCCACTGACGTGAGCGTTCAAGGCGACAACCAACACACTGACCACAAGGCAATGATAGGGTACGGACTA
CATCCGCACCCGGTATTTCCCGCCAAATTATAGACCTGTCACTGCATTGATAAGCCGTTAAGGGCTTATAACAGGCCATAATTACAAACGATAGC
CACCACGCTGGGGAGCGTGTCTCATATTAATTGACTTCGTCTTACTAGCAGTTCTGCGAAATGACTTTGCAGATTTATATTTGTTTACTGGCTTTCT
TCGTAACATGATGAACTCCGTAGTTAAAATAGTGGTTTGGTGTCACCTAGCACAGTTACATCAAGTAGAGTAACTGTGCTGGCCTCAGGATTTCA
TCCTTCGGCCTTAGGTGTTTCTGTAGAAACGATGGGTTCAACCACAGGTTGTCCATCAATAAGACCCAATTGAATCGCTTCATCACGATTCTGGTC
GTTCTCAAGGAACTCCAATAATTTGACAGGATCATGGTCAAATCGGACTCTTAATTTCGCTGGCAGAGCCATGAAATCGTCCATAGTTGCGTTAA
TTTGATTCAACGCAGAATGGTAATCAGTAACACCACTAAAATCGCCGTATTGAGGCGATACAGGGGCCGTTGGAAGTTCCCCTGTAACCCCGAA
ACGTTCAATGATGACATTAATGTCACATTCATCTTTCATATGCTGTTGAGCAAGACTTGGGTCTTGACACAGCAACGCATCGTTTACCGATGCTTC
ATCTTTATCATAGTTGTACGGATTACGTACAAATGGCAAATTCGCTTTACTCATTATTTGACTCCAATTCCCCAAGGGGTTAGTTGATTAGGATTAT
TTTTGAGTTTATCAAAAATTTCTTTCGAAGCATCGATACCCCTAGACCAAATAGCTGGGGATGGAGCCAATAGCCTCTTAGTATTATATGACTGAG
CGGATGTTAAATCCGCAGTCGTATTCTTTAAAGCAATATCAGCCAAAATACGTTTATTTTCGGCTGTAATATTAGGAGCAGTAAGCAACTTATTAA
CAGTATCAGCCCTAGTATTAGCGGTACCAGCTTCAGTAGCTTCAGTCTGGGCGATAATCTGCTTTTCTGTTTCTGATGCATTACGAATCTGTTGCA
TCATAGAAGCAGTATTAATAGCTGAATTAGTTGCATTACCTAAAACATTTTCCATAGTAGCAGTTGAACCAGATGGGGTAGAAGCACCACCACGT
TGATAGGCTAACATAGGAGATAAACCAGCAGCTTCTAAATCTTTAACAGCACGCTGGTAACTTGTTCCACTCATATCGGCTTGAAAATCCATTTGC
CTCTGAGCCATCTCCTGATTAGCTTTATTAGCAGAAGTAGAACCTAAATAAGAACCAACAGCGCCCAAAGCAGTACCGACACCAGGAGTAAAGA
AACTAGAAGCTGAAGACAGCTTAGAACCAACATTAGTAACCGCATCAAGTATTCCACCAAACATAAACTAACGCCCTTCGGTTGTTTCCTCACTAC
TCCTTACGGAGTAGTCGAGGTTATATAAAACATTAGAAATGATCAATTAGACCAGGTACGCTGTACATAGGCATAGGTCTGGCCATCTGACAATC
AAAGAAAGCATCCATTAAAAATTGCTGACCATTTGCTGCAGCTCCGACCGCCGTAGTACGAGCAACTGGAGGGGTCTCTTGAATAAACGTTGAA
TTCAACGTAGGAAGAGAAGTAAATCGCTGTCCATAATGCCAAGCATCAATAGTGCCAGCAGAAGTAGACCTAAAGAAACCAGTAATTTGAGAA

GGTTTGTAACGATACTCCGCCCAACGTTCTTGATAACCAAATACATCATTGTCGGCTGCAGTACCTTGAACATAAATTTCTTTGTTCAAAATAGCTT
GCTCACCCAAAGTGGCAAATACTGGGAAATAAAAGTCATATCGTGTAGACCTAGACCACATCTTAGGAAGACCCTGTTGATATGTGAGGTCAGC
ACGTACAGAAACTAATCCGATAATGTATCCATGTTCTTGAGCAGCATACGTAAAGCCGTGTCCCTGAGCCAATGCAGTACCCATTGCAGCAAGGT
TACCTTGCGGAGTAGCAGAACCAGTAACCGACGTTGCAGAAGTCTGAGCAATCGGATTAACATTAACAAGGGTCGAACCTCCACCAATATATTC
AGGACGTTGTAAACGATAATCCTGTGGAGTTACTCCAAAGTGAGCACGTAGTAACTCAGTATAACGAGTACCACCACGTGCATCGCGCTCCAAT
AACTTCTGAATCTGGAAAGATTGACGAAGTTGGTTAATAGTCGCAGCAGTAGCAGTAGACAAATCAGCGTATAACTGATTAGTAGCAACACCAG
CATTTGTACTATTAGACAAAGTATTAGAAGCTGAATTTAATTCTCTTAAAGCACCAACAGCAGTTTGAAAAACACTATAACCAGCAGTACCATTGC
CAGCTGGTATGTCAGAATAAATATTAGCACGTGTTCCTAACGGTAAAGAAACAGAAGCACCCTTCTGTGGCCAAGGCAATGCACCAGTAAAATA
ATCTTTACGCTTACCACGTCTCAAAAGTGTATAGTTAGCAGGAACATCACCTGAATCACCAAGATTTAACGTTACAGAATTCTGTAAGTTTTCATC
TCTAAACCATTCGTTATAAATCTCATTATAAGCACGTAACGGCAAAACATTATGCGTTACTGTATTAGCGCCAGTAATCTGACCAGCAGTAGGTAA
ACCAAAATAATCAAAAATTGAACAAACAGCATAACCACCAGCAGGACTAGTAATAGTAGGCACTACAAAAGAAATAGAATCACCAGGATTCGTT
TGTTCACCCATAAACTTTGGCCAATTGTTCCAAAGTAAACGATTAGGTACAAAGAAAAAGAAAGTATCAAGATGCAAGTTGTCCATCACAGGAA
ATAACGGTGTGGCCAAACGTGCAAATGCAGTCATCTTTACACGATGAGTATCGCCAGGCAATACTTCATCACAATAAATAGGAACTAAAAAACC
AGCATCAAACGTGGTTTTATGAGCATATTGAGTATTAAAACTAGAGCGGGGAATGTCCGCTTTAGGAACCATAGCAAATGAATGCGTACTTACT
GACTTATTGCGAAACATAAACATCTCCCGTAGTTCCGTACCACTCT

>000118F|arrow

CACCACTAAAGTCGCCATATTGAGGCGTTAAAGGAGTTTGAGGAATAAGTCCGGTCATACCGAACTTTTCCATAATATTATTGATATCACATTCTT
CAGCAAATTGCTGCTGAGTCAGAGTTGCATCCTCACAATGCAGCCCTGACTCATTTGACGCAGCAATCGTGTCGTAATTGTACGGAGTACGAAGA
AATGGGGCTGTAATCTTTGTCATTTTCATTCCATTGGTGGTTGGTTATTTGTACGATTTTGTTGAATCGGTTGACCTCGATAGTTATCGATGTATCG
TTTTGCGCCAGATGCGCTATACGCATCTTTGGCGATGTTTTTACGTCTTGATAAATTGGTTTGCTAGAACTAGGAGCTGACCCTGATTTAGCAAGT
TTTGTTAACTCGTTTGAATAACGAGATTGTGCCAAAGCTTGAGCAGCTTGAGCACTTGATGTTTTGCCTATTTCATTTTTCATGAAAGTATCGGCA
AGTATTTGTTTATATTGAGCTCTAATATTTGGATTTTCATCCATTTAGTTAACGTATCAGCACGTACATTATCTGTTTGATTACTTGTGAGTTCTGTT
TGAGCTGCAATTTGTTTAGATTGAGCAATAGCTTGATTTGCTTGAGCAACCGTTTGATAAGCTTGGGTTCCGGACGTAGTTGCATTACCGAGAAA
CATTTTGCATCTGGGCCATAGCTCCAGCTGGTGTTGTGGCTCCGCCTTGTGAATACGCAAGCATGGGATTTAAACCCAGCTTTTTTCATATCTTCA
ACTGCTCGTTGATATGATGTTCCAGGACATTTCAGCTTGAAATCTCTGTTGATTACTCGCTTGTTCTGCACTCGCTGCGTTTTGACTTTGTGTTCCA
AAGTATTGTCCTGCTGCACCTATTGCTGCAGGTGCAAGTGCAGCTAAGGAGAGTCCCCCAGTAGCAGGGGCAGCTCCTATAGCTATAGCAGGGC
CGATTAAATCGGCAATTCCGTCAAATAGTCCCATTTAGAAATGGTCTATTAAGCCAGGTACTGAATACATTGGCATGGTCTTGCATTTTGACATCA
AAAAATGAGTCAAATAAGAATTGTTGGCCATTTGCAGCTGCTCCAACGGCTACTACACGTGATACAGGAGGCGTATCTTGAATAAACGTATTATT
CAAAGTAGGCGCAGCGGTAAATTTTTGAGCCAAATGCCAACCCATCGATCGTGCCCGCCGATGTTGATTTGAACAAACCAGTAATTTTAGATGGT
TTGTAACGATATTCCGCCCAGCGTTCTTGATATCCAAAAACAGTATTGTCGGCTGCAGTATCTCCTGTTGCATAAATTTCTTTTGCAAAACAGATTG
TTCGCCTAGAGTAGCAAATGCTGGGAAATAAAAGTCATATCGTGTAGATCTAGACCACATACGGTCTAATCCCTGGTTGATAAGTAAGATCTGCT
CTAATGGATACTAATCCAATAATAACGCCATGCTCAGTAAATGATTGAGTAAATCCATGATTATGAGCGAGAGCAGTACCCATAGCAGCAAGGTT
GCCCAAAGGGTAGTCGTTCCAGAAGCGTTTGTTCCCGACGTTTGAGCAATCGGATTAACATTAATTGGTGTTGAACCGCCTCCAAGGTATTCAGG
CCTTTGTAAACGGGCGTCTGGGGAAATAACTCCAAAGTGACTCCGGATAATTTCTGTGTATCGTGTTCCGCCACGTGCATCCCTTTCAAGTAATTT
TTGAATTTGAAATGACTGTCTTAATTGATTGACAGTTGCAGCAGTTGCTTCTGATAAGTCGGTATATAAATTAGAAACGTTATTTACTACACCAGC
AGTATTAACACCATAAGCGTTGCCATATCTAGCTAAAGCAGTCGTATTTCCAGGATCTGTTTGAATTACCGTAAATTTATCGTTTGATGTTGCGTCT
CCTGAAATGGTATCCCATTTAATTGGAGCCGTAGTACCTAAAGGTAAGGTGACACTCGCACCTTTTTGTGGCCATGGTAATGCTGACGTAAAGTA
ATCATGTCTTTTACCACGACGTTTTAGCACATAGTTTGAGGAAGTATCAGGGCCATCGCCCTTATCAACTACTGCGCTTGTTTGTAAATTTTCATCT
CGGAACCATTCGTTCCAGATAAGATTGTATGCACGTGGCCAAAAGGCACAGTGCGTAATAGTTCGGCCAGTATCAATTTGGCCTACTGTTGGTAA
GCCCATATAGTCTTGAAGGCTGCCTACGGCATAACCATCTGTTGGGCTTGTTTGTGAGTTGGGACAATATAAGATATTGAGTCTGTTGGATTTTCT
TGTTGACCCATAAATTTTTGCCAGTTATTCCATATAAGGCGATTGGGTACAAAGAAAAAGAAAGAATCCATAATCATGTTAGTCCATGATTGGAT
ATAAAGGCGTTGCTAGACGGGCAAATGCCGTCATTTTTAAGTTGAAAGTGTCCCCAGGGAGCACTTCATCAACATATACAGGAATTAATAGCCC
GCATCGAAAGTCGTTTATGTGTTTTTTGAGCATCGAATTTACTACGTGGTATATCGGCTCTAGGTACCATCGCGAAGCGGATGAGTATTAACTGA
CTGATTGCGGTGCATGTTTTTCCTTAGTGTTGTTCCGGGGGAAAGATAAATCTCTTTTCCCCTCGGTTGTTTTATTTAAGTTTAACTTGTTTTCCTAA
TGATAGGAGTTTTGGTTGTTCATGTAAATCGAATAACCCAGTTGAATCGTCAAAAGTTCCGAATTCATATAGATCGAAGTCATCAGGGTGATTAA
AAAGTTGATTTTCAGTATCAGAACGATTAATTTCATCTGAAAAAGAGCGTATAGCTACTCCAGAGGAAGGTACGAACATTGGTCGTGCATATGCT
TCAGCAGCACGGTCTTTTACGGAAGCGAGGATAAGTTTCATTATTTTCCTAAGTGAGGTTACGTTTTAATAGTTGAAGTTTTGCCATAGTTACTTG
TTCTTTTGCAGATAGTCGTTCTGGTGTATTGTCTTCGGAATTAAGTTTAGCGTTATTTTCCCGCATGTAAAGTAATTCGTCATACTCATAAGGTTGG
TCAATTTTAAACATTTTGTCATAGTATTTTGGTGGTTTGACCTTTTTACCTCTAAGTATTACGTAGTCTTGCGGGTATATATCCGAAGTATATTTTAT
ATAAAAGTCTTTACCGATTCCCGGTTTTAAAGACATTTTATTATATTCCGGCTTTAAGTCTAAATATTCGCCGGTTTCAGGGTGTATGCGTTTGTAA
TGAGATTCCGCATCTTTCCCTGTTTGTTTTTTCATTATGTATCTAGCCACGTAGGCGGCTGATTCGAAAAGTAACATCTCCAATGGTGGTATAACC

AAATGGCCAGAGAGCTTCAAGTTCTGCGGATCTATATAACATAGAACCAGAGGCAGTCCTTTTCCATAATTTTTTATCAGGAAAGTCGTATCCGA
AGATACAGGCATGGAAGTGGGGTCTTGCGAAGAGTTCACCATATTCTCCAGCCATGTAATAGCGGATTGTAAGTCCTCTTTTTGCGAGAGTTTTT
CTAAGTCTTTTAAGGAACAGTTGAAAGTCTTTGTGATCCAAAGAGCCATCGCTTGGGAGATGTGTATTGTCATATGTGAGTGTTATGAATGAGTT
GTTTTTATGCAATTGGGCTTCGTGCATGCACCGAATAGCCCATTGTCTTGATCGTTCTAGACGGCATCCAACGCACTGCCCACAGGGCAGGTCTA
AAGATCGAACGATGTCATGTTTCCGAACTTCGTCGAAAACAATTGATTTGTCAAAGCATTGATATGCTTTGAGAGGGTGATAACAAGGCATGTG
AGGTGCCTGAGGACTTTATTAGAGTCTCCAGCCTCCACGCATTGGCGAGTTTCGCATATTTGCGTGTTTTGTATGTGAAGCATGTTTACGGAAAG
TCCTAGCGGACTTTTGCTTATTTGCTGGTCTGCGACGCATCATTTTTTCTGTCCTTGTTTATCGTGTTTTTGTGGTTTGGTGTCACCTAGCACAGTTA
CATCAAGTAGGTAACTGTGCTTCCGAGGTTCCAACAGGGGTTGAAACCTCGGTTTGGGCTGGTTTTACCAGTCCCATTTTTTCAGCTTCGCTGCG
ATTTTCAGGGTTTTCTAGAAAATCTATTAGATTCGCGGGATCATTAGCGAATCGTTCACGAATATTGGCTGGTAAAGCCATAAATTCGTTGTCTGA
AGCCATAATCTGGTTCAGAGCAGAGTGGTAGTCATAGA

>000002F|arrow

TGCGTCGCAGACCAGCAAATAAGCAAAAGTCCGCTAGGACTTTCCGTAAACATGCTTCACATACAAAACACGCAAATATGCGAAACTCGCCAAT
GCGTGGAGGCTGGAGACTCTAATAAAGTCTTCAGGCACCTCACATGCCTTGTTATCACCCTCTCAAAGCATATCAATGCTTTGACAAATCAATTGT
TTTCGACGAAGTTCGGAAACATGACATCGTTCGATCTTTAGACCTGCCCTGTGGGCAGTGCGTTGGATGCCGTCTAGAACGATCAAGACAATGG
GCTATTCGGTGCATGCACGAAGCCCAATTGCATAAAAACAACTCATTCATAACACTCACATATGACAATACACATCTCCCAAGCGATGGCTCTTTG
GATCACAAAGACTTTCAATTGTTCCTTAAAAGACTTAGAAAAACTCTCGCAAAAAGAGGACTTACAATCCGCTATTACATGGCTGGAGAATATGG
TGAACTCTTCGCAAGACCCCACTTCCATGCCTGTATCTTCGGATACGACTTTCCTGATAAAAAATTATGGAAAAGGACTGCCTCTGGTTCTATGTT
ATATAGATCCGCAGAACTTGAAGCTCTCTGGCCATTTGGTTATACCACCATTGGAGATGTTACTTTTGAATCAGCCGCCTACGTGGCTAGATACAT
AATGAAAAAACAAACAGGGAAAGATGCGGAATCTCATTACAAACGCATACACCCTGAAACCGGCGAATATTTAGACTTAAAGCCGGAATATAAT
AAAATGTCTTTAAAACCGGGAATCGGTAAAGACTTTTATATAAAATATACTTCGGATATATACCCGCAAGACTACGTAATACTTAGAGGTAAAAA
GGTCAAACCACCAAAATACTATGACAAAATGTTTAAAATTGACCAACCTTATGAGTATGACGAATTACTTTACATGCGGGAAAATAATGCTAAAT
TTAATTCCGAAGACAATACACCAGAACGACTATCTGCAAAAGAACAAGTCACTATGGCAAAACTTCAACTATTAAAACGTAACCTTACTTAGGAA
AATAATGAAACTTATCCTCGCTTCCGTAAAAGACCGTGCTGCTGAAGCATATGCACGACCAATGTTCGTACCTTCCTCTGGAGTAGCTATACGCTC
TTTTTCAGATGAAATTAATCGTTCTGATACTGAAAATCAACTCTTTAATCACCCTGATGATTTCGATCTATATGAATTCGGAACATTTGACGATTCA
ACTGGGTTATTCGATTTACATGAACAACCAAAACTCCTATCATTAGGAAAACAAGTTAAACTTAAATAAAACAACCGAGGGGAAAAGAGATTTAT
CTTTCCCCCGGAACAACACTAAGGAAAAACATGCACCGCAATCAGTCAGTTAATACTCACCGCTTCGCGATGGTACCTAGAGCCGATATACCACG
TAGTAAATTCGATGCTCAAAAAACACATAAAACGACTTTCGATGCGGGCTATCTAATTCCTGTATATGTTGATGAAGTGCTCCCTGGGGACACTTT
CAACTTAAAAATGACGGCATTTGCCCGTCTAGCAACGCCTTTATATCCAATCATGGATAACATGATTATGGATTCTTTCTTTTTCTTTGTACCCAAT
CGCCTTATATGGAATAACTGGCAAAAATTTATGGGTCAACAAGAAAATCCAACAGACTCAATATCTTATATTGTCCCAACACAAACAAGCCCAAC
AGATGGTTATGCCGTAGGCAGCCTTCAAGACTATATGGGCTTACCAACAGTAGGCCAAATTGATACTGGCCGAACTATTACGCACTGTGCCTTTT
GGCCACGTGCATACAATCTTATCTGGAACGAATGGTTCCGAGATGAAAATTTACAAACAAGCGCAGTAGTTGATAAGGGCGATGGCCCTGATAC
TTCCTCAAACTATGTGCTAAAACGTCGTGGTAAAAGACATGATTACTTTACGTCAGCATTACCATGGCCACAAAAAGGTGCGAGTGTCACCTTAC
CTTTAGGTACTACGGCTCCAATTAAATGGGATACCATTTCAGGAGACGCAACATCAAACGATAAATTTACGGTAATTCAAACAGATCCTGGAAAT
ACGACTGCTTTAGCTAGATATGGCAACGCTTATGGTGTTAATACTGCTGGTGTAGTAAATAACGTTTCTAATTTATATACCGACTTATCAGAAGCA
ACTGCTGCAACTGTCAATCAATTAAGACAGTCATTTCAAATTCAAAAATTACTTGAAAGGGATGCACGTGGCGGAACACGATACACAGAAATTAT
CCGGAGTCACTTTGGAGTTATTTCCCCAGACGCCCGTTTACAAAGGCCTGAATACCTTGGAGGCGGTTCAACACCAATTAATGTTAATCCGATTG
CTCAAACGTCGGGAACAAACGCTTCTGGAACGACTACCCCTTTGGGCAACCTTGCTGCTATGGGTACTGCTCTCGCTCATAATCATGGATTTACTC
AATCATTTACTGAGCATGGCGTTATTATTGGATTAGTATCCATTAGAGCAGATCTTACTTATCAACAAGGATTAGACCGTATGTGGTCTAGATCTA
CACGATATGACTTTTATTTCCCAGCATTTGCTACTCTAGGCGAACAATCTGTTTTGCAAAAAGAAATTTATGCAACAGGAGATACTGCAGCCGACA
ATACTGTTTTTGGATATCAAGAACGCTGGGCGGAATATCGTTACAAACCATCTAAAATTACTGGTTTGTTCAAATCAACATCGGCGGGCACGATC
GATGGTTGGCATTTGGCTCAAAAATTTACCGCTGCGCCTACTTTGAATAATACGTTTATTCAAGATACGCCTCCTGTATCACGTGTAGTAGCCGTT
GGAGCAGCTGCAAATGGCCAACAATTCTTATTTGACTCATTTTTTGATGTCAAAATGGCAAGACCAATGCCAATGTATTCAGTACCTGGCTTAATA
GACCATTTCTAATGGGACTATTTGACGGAATTGCCGATTTAATCGGCCCTGCTATAGCTATAGGAGCTGCCCCTGCTACTGGGGGACTCTCCTTA
GCTGCACTTGCACCTGCAGCAATAGGTGCAGCAGGACAATACTTTGGAACACAAAGTCAAAACGCAGCGAGTGCAGAACAAGCGAGTAATCAA
CAGAGATTTCAAGCTGAAATGTCTGGAACATCATATCAACGAGCAGTTGAAGATATGAAAAAAGCTGGGTTAAATCCCATGCTTGCGTATTCACA
AGGCGGAGCCACAACACCAGCTGGAGCTATGGCCCAGATGCAAAATGTTCTCGGTAATGCAACTACGTCCGGAACCCAAGCTTATCAAACGGTT
GCGCAAGCAAATCAAGCTATTGCTCAATCTAAACAAATTGAAGCTCAAACAGAACTCACAAGTAATCAAACAGATAATGTACGTGCTGATACGTT
AAATAAATTGGATGAAAATCCAAATATTAGAGCTCAATATAAACAAATACTTGCCGATACTTTCATGAAAAATGAAATAGGCAAAACATCAAGTG
CTCAAGCTGCTCAAGCTTTGGCACAATCTCGTTATTCAAACGAGTTAACAAAACTTGCTAAATCAGGGTCAGCTCCTAGTTCTAGCAAACCAATTT
ATCAAGACGTAAAAAACATCGCCAAAGATGCGTATAGCGCATCTGGCGCAAAACGATACATCGATAACTATCGAGGTCAACCGATTCAACAAAA
TCGTACAAATAACCAACCACCAATGGAATGAAAATGACAAAGATTACAGCCCCATTTCTTCGTACTCCGTACAATTACGACACGATTGCTGCGTC

AAATGAGTCAGGGCTGCATTGTGAGGATGCAACTCTGACTCAGCAGCAATTTGCTGAAGAATGTGATATCAATAATATTATGGAAAAGTTCGGT
ATGACCGGACTTATTCCTCAAACTCCTTTAACGCCTCAATATGGCGACTTTAGTGGTGTCTATGACTACCACTCTGCTCTGAACCAGATTATGGCTT
CAGACAACGAATTTATGGCTTTACCAGCCAATATTCGTGAACGATTCGCTAATGATCCCGCGAATCTAATAGATTTTCTAGAAAACCCTGAAAATC
GCAGCGAAGCTGAAAAAATGGGACTGGTAAAACCAGCCCAAACCGAGGTTTCAACCCCTGTTGGAACCTCGGAAGCACAGTTACCTACTTGATG
TAACTGTGCTAGGTGACACCAAACCACAAAAACACGATAAACAAGGACAGAAAAAATGA

>000180F|arrow

CGAGCCCAGTACCCATAGCGCCAAGTGTACCGAGAGTAGTGGTCCCGGAAGCTCCGGTACCATTCGTCTGGGCGATTGGATTGATATTAAGTGT
GTTGAACCGCCTCCAATGTATTCAGGCCGTTGGAGACGCGCATCGGGGCTAACGACCCCGAAATGGCTGCGGATAATTTCTGTGTATCGAGTTC
CGCCACGGGCGTCCCTTTCTAAAAGTTTTTGAATCTGGAAGCTTTGACGCAATTGATTAATTGTTGCAGCGGTTGCAGCTGATAAATCTGCATATA
AACCGCCATTAGGATCATATGATTTTGCCAAGCCGTCAGCACCGCCAGTAATTTGACCAGTAACACCTACGTTAATAGCCTGGGCGGTTGCGTTT
AATGTATTTGTTCCAGCGTTATACAGTCTGGAAACAGGCGCATTGTTAGTGCGTAATATTGGGGCGGATGTTCCAAGCGGTAAAGTAACGCTTG
CGCCCTTTTGTGGCCAAGGTAATGCTGACGTGAAATAGTCTTTACGTTTTCCACGTCGTAATAATGTGTAGTTGGCTACGTTATCTGGACCATCGC
CAGTATCTACAACTACTGAATTTTGAAGGTTTCGTCCCGAAACCATTCGTTATAAATAAGGTTGTAAGCACGTGGCCAGAAGGCACAGTGACTT
ACGGTGCCACCAGCACCTACTTGGCCTACGTTGGCAGACCCATATAATCTTGAAGGCTGCCAATAGCGTAACCACCAGCTGGGGTTACTTGTTGG
GGCACTACGTACGAATCGAGTCCGCAGGATTTGCCTGCTGCCCCATAAATTTTTGCCAATTTGACCAGACAAGTCTATTTGGCACAAAAAGAAGA
AGCTATCCAATGCATATTGTCCATGATCGGATAGAGTGGTGTAGACAATCGCGCAAAGGCGGTCATGTTGCAGCGGAATGTATCGCCTGGCAAC
ATTTCGTCTACGAGTACAGGGACTAGAAGCCAGCATCGAATGTAGTTTTATGTGTTGATTGACAGTCAAATGTAGAGCGGGGGATATCCGCTTTT
GGAATCATTGTGAACTGATGGACGTCTACCGACTTGTTACGATGCATTTTTTTGAGCTCCTAGGCCTAGTTGCGTGAGAAAAAGGGGTTTCCCCC
TTTTTACTCTACGCTTAGTTTTTATCAGTAATTTTGACTTGTTTCCCTAAGGATACAAGTTTGGGTTGTTCATGTAATTGGAACAAACCCGTATTATC
GTCGAATTCGCCTAATTCAAATAGGTCGAAATCGTCGGAATGGTTATAAAGTTGATTGTCATCGCTCTGGCGATTGACTTCGTCGCTAAAGCTCC
GGATTGCTTCGCCGATAGAACGGACGAACATTGGACGACCGAACGCATCTGCTGCGCGGTCTTTAACGGTACAGAGTACTAATTTCATGAGGAT
TTCCTAAGTGAGGTTACGTTTTAATAATTGAAGTTTGGCCTTTGTGACTTTTTCCTTTACGGCAAGTCTGGCATAGGTATTGTCTTCGTGGTTGAGT
TTAGCAGAAGTTTCACGTTTGTGGAGTAATTCTTCGTATTCATATGGATAATCTGATTTATATTTTTTGTCATAGTATTTTGGGGGTTTTACCCTTTT
TCCGTTGACTACAACGTAGTCATGGGGATATACGTCGGAACGATATTTTTTGTACCACTCAGCACCAATGCCTGGTTTAAGGCTCATATTGTTATA
TTCTGGTAATAGCTTTATTAATTCCCCTGTTTCTATATCACAGTATGTGTAATGCTCATCCTTTGTTTATGTTTGTTTTCTTCCATCTGGCCGTTGTAT
TTTTGCATAATATATCGAGCAACGTAAGCAGCTGACTCGAATGTAACGTCTCCAATGGAGGAATAACCATGTGGCCAGAGGGTTGCAAGGTGGT
CGGATGTATATATGAGAGAACCAGAGGGAGTCCTTTTGAATAGTTTCTTATCATGAAAATCGTATCCGAAGATACAGGCATGGAAGTGAGGTCT
GCCGAAACTTGTGCCGTATTCTCCAGCCATGTAGTAACGTAATTTTGCAGGTGCAATGGATTTTCTGAAGCGCTTAAGGAACTTTTGGAAGTCGC
TTTTGACAAGCGATCCAGTTTGTGGAAGGTTTTCATTGTTGTATGTGAGGGTTATAAAGCAGTTTTTTTCGTGCAATTGGGCTTCATGCATGCAGC
GCATGGCCCATTGACGTGATTTTTCTAGCCTGCAGCCAACGCATTGGCCGCAGGGCAGTGAAATCTGACGATCATGCTCGTCAGATTCTTTGAAT
GCGACACGGCGATAAGATTTGCCGGTCTTATAGTTTGTCTGATGCTTACTTAAATAAGCAGTCAGTGGGTGATAACAGGCCATGTGAGGTAGCC
TGGCGCTTTATTAGAGCCGCCAGCCCCCTCTTTGCGGGGCTTTTTGCATATTTGCAGCTTTAGTTTTTGAAGTGTTCCTACGGAACGATTTAGCGC
TTTTGCGCTTGTTTGTTTGTTTTCTATAAAGCATAATTTTTGGCCTCGTTTATCAGATATTTTTGGTGTTGGTGTCACCTAGCACAGTTACATCAAGT
GGAGTAACTGTGCTTATCGCCATTTCTTCCGAAATGGCTCTTTCGACCAGGCCGAGTTTCTCGGCTTCTGGTCGATTTTTCTCGTCTTGCAAGAACT
CAATCAGGTTTGCTGGTTCGTTTTCGAACCTAGCACGAATTTGAGCCGGTAAGTTATCAAATTCTTCCATAGCGTTCATTACCTTATTCAAGGCGC
TATGATAGTCAGTAATTCCGCTGAAATCGCCATATTGAGGCGGCAGCGGACTTTGAGGTAATAGGCCTGTAACGTTAAAACGTTCCAGGATAGT
ATTTATATCGCATTCGTCTTTATAATGCTGCTGAGCCAGAGTTGGCTCCTCACAAGCCAACCCTGACTCATTTGACGCAGCATCCGTGTCATAGTT
GTATGCGGTTCTAAGTTTGATTGTTTTCATTTCATTTTTCCAAATGGTAGATATTTTTGATATTTATCATATTGGCTATGTAAATAGCCTTTAACATC
TTGATAAATTGGTTTAGTACTGGATGGCGCTGATCCAGTTTGCGCTAATCGCTCTAATTGGTCTGTATATGCACTTTGACTTAACGCTTGTCGCTG
TTGTGCAGCTTGTAATGCACTTGATGTCCTTAATTGCTCTATTTGGGCATCGCGAAGCTGACCAAATTTGCCGTATCCCGGCATCTGAGCAATTTC
ACGAGCTGTATTTGCTCGAGTATATGTTGCCTGATCACGTGATAGATTTGTATCCGCGTCCGTTTTTTTCGGCTTGCGATTGTGTCAGGATATTCT
GAGTTTGTAATTGTTTAAAATTGGCGACTGCCATGGCAGCTTCACGGGCTGAATTTCCAGCCTCACCTAGTGGATTTCCTACTGGAGCTTGCGCA
CCAGGCTGGACTTTGGCTCCGCCTTGTGAATAAGCCAGCATAGGGTTAAGACCTGCAGCCTTAAGATCCGCTACTGCGTCTTGATATGCAGTACG
TCGCATACCATCTTGGAATTGCATCATTGCTGCAGCTTGTTGTGCGCTGGCAGCGTTTTGTTCTTGACCACCAAAATAACTAGCGGCCGCACCTAT
TCCGGCTCCAATTAAGGAGCCGTAAGGTCCAAAAGCGGCGCCAGATGCGGCGCCTGAGGCAGCGGCTTCTAGTGCCATTAGAAGTGGTCGATT
AAGCCAGGTACAGAGTACATTGGCATTGGCCGTGCTTTTTTAATATCAAAAAAGCTATCAAAATGATTTGCTGGCCGTTGGCAGCTGCTCCGACC
GCAAGGGTTCGAGAGACAGGTGGATTTTCTTGAATAAACGTGTTATTCAAAGTTGGAAGTGTTGTAAACCGTTGGGCTAAATGCCAGCCGTCAA
TTGTTCCAGCTGCCGTACTACGGAACAGACTGGAAATTCGGCTTGGCATATAACGATATTCTGCCCAACGTTCTTGGTATCCAAACACTCCTGTAT
CGTTGGCATCGCCTCGTACGTAGATTTCTTCATTTAATACTGCTTGTTCGCCCAAAGTGGCGAAAGCAGGAAATAGAAATCATAACGTGTGGAAC
GATTCCACATGCGGTGCATACCTTGTTGGTATGTTAATCGGCTCGTACTGAAACGAGACCGATAATTACACCATGTTCAGTGCTTGAATAAGTAA
AGCCATGATTGTGAG

>000204F|arrow

AGTATCAAGATGCAAGTTGTCCATCACAGGAAATAACGGTGTGGCCAAACGTGCAAATGCAGTCATCTTTACACGATGAGTATCGCCAGGCAAT
ACTTCATCACAATAAATAGGAACTAAAAAACCAGCATCAAACGTGGTTTTATGAGCATATTGAGTATTAAAACTAGAGCGGGGAATGTCCGCTTT
AGGAACCATAGCAAATGAATGCGTACTTACTGACTTATTGCGAAACATAAACATCTCCCGTAGTTCCGTACCACTCTTTCGAGTGATACGGTATA
AAAAAAACCTTACTCGCCTTCGCGAATCATTACATCTTTTGCACGAGCAATCAACTTAGGGCTTTCCAGTAGTTCAAAAACACCAGTGGCATCGTC
AAAAAGACCCAAATAGAACATATGAAAATCATCAGGATGTTTATACAACTGATTATCTTCGCTAGCTCGATTGACTTCATCCTGAAACTGACGTAC
TGCAACACCTTCAGATGCAACATAAGCTGGACGACCAAAAGCATCTGCAGCAGTATCCTTAATAGAAACAATAACCATCTTCATAAAAACTCCTT
AAATAGTACGTTTTAACAATGACAACTTAGCCAACGCAACTTTTTCCTTAACAGCAAGTCGCTCAAGCGTGTTGTCCTCATGCCTAGATCGACCTT
CCATCTCTCTGGCAAACTGAATCATATCGAATTCTTCAGGAAACTTCAACTTAAATTTATTATCATAAAACCGTGGTGGACGGCACTTTTTGCCAC
GCACCACAACGTGGTCTGACGTATAAACGTCTGACATGTACTTATCTAACCACGATTGCCCGATACCGGGCTTCAATGACATCTTATTAAATTCTG
GCTTACGCTGAATTATCTCACCAGTCTCTAAATCACAATATTGATAATGGGCACCCGCATCAACCACTTCGTGGTTTTCATTGACAGTAACCCCATT
AATCTTCTTCATAATATATCTTGCAACATAAGCAGCAGACTCAAAAGTAACATCACCAATTGTAGAATAGCCAAACGGCCACAATTCTTCCAAAAT
CTCTGACGTGTAGAGGATAGAGCCAGTCTGCGTTCTTTTAAATATTTTCTTATCCGGAAAATCAAGACCAAACAGACAAGCATGGAAATGAGGA
CGATCAAAAGATTCACCATATTCACCTGCCATAAAAACGTATCGTTTTCCCAGTAAAACGCTTACGTAACCGCTTCATAAAAAGCTGATAATCATT
GTAATCCAATGACATATCCTTAGGACAATGCTCTGGAGCATATGTCAAAGTAATAAAACAATTACTAGTATGCATTTGTGCCTCATGCATACAAC
GAATCGCCCACTGACGTGAGCGTTCAAGGCGACAACCAACACACTGACCACAAGGCAATGATAGGGTACGGACTACATCCGCACCCGGTATTTC
CCGCCAAATTATAGACCTGTCACTGCATTGATAAGCCGTTAAGGGCTTATAACAGGCCATAATTACAAACGATAGCCACCACGCTGGGGAGCGT
GTCTCATATTAATTGACTTCGTCTTACTAGCAGTTCTGCGAAATGACTTTGCAGATTTATATTTGTTTACTGGCTTTCTTCGTAACATGATGAACTCC
GTAGTTAAAATAGTGGTTTGGTGTCACCTAGCACAGTTACATCAAGTAGAGTAACTGTGCTGGCCTCAGGATTTCATCCTTCGGCCTTAGGTGTT
TCTGTAGAAACGATGGGTTCAACCACAGGTTGTCCATCAATAAGACCCAATTGAATCGCTTCATCACGATTCTGGTCGTTCTCAAGGAACTCCAAT
AATTTGACAGGATCATGGTCAAATCGGACTCTTAATTTCGCTGGCAGAGCCATGAAATCGTCCATAGTTGCGTTAATTTGATTCAACGCAGAATG
GTAATCAGTAACACCACTAAAATCGCCGTATTGAGGCGATACAGGGGCCGTTGGAAGTTCCCCTGTAACCCCGAAACGTTCAATGATGACATTA
ATGTCACATTCATCTTTCATATGCTGTTGAGCAAGACTTGGGTCTTGACACAGCAACGCATCGTTTACCGATGCTTCATCTTTATCATAGTTGTACG
GATTACGTACAAATGGCAAATTCGCTTTACTCATTATTTGACTCCAATTCCCCAAGGGGTTAGTTGATTAGGATTATTTTGAGTTTATCAAAAATTT
CTTTCGAAGCATCGATACCCCTAGACCAAATAGCTGGGGATGGAGCCAATAGCCTCTTAGTATTATATGACTGAGCGGATGTTAAATCCGCAGTC
GTATTCTTTAAAGCAATATCAGCCAAAATACGTTTATTTTCGGCTGTAATATTAGGAGCAGTAAGCAACTTATTAACAGTATCAGCCCTAGTATTA
GCGGTACCAGCTTCAGTAGCTTCAGTCTGGGCGATAATCTGCTTTTCTGTTTCTGATGCATTACGAATCTGTTGCATCATAGAAGCAGTATTAATA
GCTGAATTAGTTGCATTACCTAAAACATTTTCCATAGTAGCAGTTGAACCAGATGGGGTAGAAGCACCACCACGTTGATAGGCTAACATAGGAG
ATAAACCAGCAGCTTCTAAATCTTTAACAGCACGCTGGTAACTTGTTCCACTCATATCGGCTTGAAAATCCATTTGCCTCTGAGCCATCTCCTGATT
AGCTTTATTAGCAGAAGTAGAACCTAAATAAGAACCAACAGCGCCCAAAGCAGTACCGACACCAGGAGTAAAGAAACTAGAAGCTGAAGACAG
CTTAGAACCAACATTAGTAACCGCATCAAGTATTCCACCAAACATAAACTAACGCCCTTCGGTTGTTTCCTCACTCTTACGGAGTAGTCGAGGTTA
TATAAAACATTAGAAATGATCAATTAGACCAGGTACGCTGTACATAGGCATAGGTCTGGCCATCTGACAATCAAAGAAAGCATCCATTAAAAATT
GCTGACCATTTGCTGCAGCTCCGACCGCCGTAGTACGAGCAACTGGAGGGGTCTCTTGAATAAACGTTGAATTCAACGTAGGAAGAGAAGTAAA
TCGCTGTCCATAATGCCAAGCATCAATAGTGCCAGCAGAAGTAGACCTAAAGAAACCAGTAATTTGAGAAGGTTTGTAACGATACTCGCCCAAC
GTTCTTGATAACCAAATACATCATTGTCGGCTGCAGTACCTTGAACATAAATTCTTGTTCAAAATAGCTTGCTCACCCAAAGTGGCAAATACTGGG
AAATAAAAGTCATATCGTGTAGACCTAGACCACATTTAGGAAGACCCTGTTGATATGTGAGGTCAGCACGTACAGAAACTAATCCGATAATGTAT
CCATGTTCTTGAGCAGCATACGTAAAGCCGTGTCCCTGAGCCAATGCAGTACCCATTGCAGCAAGGTTACCTTGCGGAGTAGCAGAACCAGTAA
CCGACGTTGCAGAAGTCTGAGCAATCGGATTAACATTAACAAGGGTCGAAACCTCCACCAATAATTCAGGACGTTGTAAACGATAATCCTGTGG
AGTTACTCCAAAGTGAGCACGTAGTAACTCAGTATAACGAGTACCACCACGTGCATCGCGCTCCAATAACTTCTGAATCTGGAAAGATTGACGAA
GTTGGTTAATAGTCGCAGCAGTAGCAGTAGACAAATCAGCGTATAACTGATTAGTAGCAACACCAGCATTTGTACTATTAGACAAAGTATTAGAA
GCTGAATTTAATTCTCTTAAAGCACCAACAGCAGTTTGAAAAACACTATAACCAGCAGTACCATTGCCAGCTGGTATGTCAGAATAAATATTAGC
ACGTGTTCCTAACGGTAAAGAAACAGAAGCACCCTTCTGTGGCCAAGGCAATGCACCAGTAAAATAATCTTTACGCTTACCACGTCTCAAAAGTG
TATAGTTAGCAGGAACATCACCTGAATCACCAAGATTTAACGTTACAGAATTCTGTAAGTTTTCATCTCTAAACCATTCGTTATAAATCTCATTATA
AGCACGTAACGGCAAAACATTATGCGTTACTGTATTAGCGCCAGTAATCTGACCAGCAGTAGGTAAACCAAAATAATCAAAAATTGAACAAACA
GCATAACCACCAGCAGGACTAGTAATAGTAGGCACTACAAAAGAAATAGAATCACCAGGATTCGTTTGTTCACCCATAAACTTTGGCCAATTGTT
CCAAAGTAAACGATTAGGTACAAAGAAAAAGAA

>000166F|arrow

AATAGTTGAAGTTTTGCCATAGTTACTTGTTCTTTTGCAGATAGTCGTTCTGGTGTATTGTCTTCGGAATTAAGTTTAGCGTTATTTTCCCGCATGT
AAAGTAATTCGTCATACTCATAAGGTTGGTCAATTTTAAACATTTTGTCATAGTATTTTGGTGGTTTGACCTTTTTACCTCTAAGTATTACGTAGTC
TTGCGGGTATATATCCGAAGTATATTATATAAAAGTCTTTACCGATTCCCGGTTTTAAAGACATTTTATTATATTCCGGCTTTAAGTCTAAATATTC
GCCGGTTTCAGGGTGTATGCGTTTGTAATGAGATTCCGCATCTTTCCCTGTTGTTTTTTCATTATGTATCTAGCCACGTAGGCGGCTGATTCGAAA

GTAACATCTCCAATGGTGGTATAACCAAATGGCCAGAGAGCTTCAAGTTCTGCGGATCTATATAACATAGAACCAGAGGCAGTCCTTTTCCATAA
TTTTTATCAGGAAAGTCGTATCCGAAGATACAGGCATGGAAGTGGGGTCTTGCGAAGAGTTCACCATTCTCCAGCCATGTAATAGCGGATTGTA
AGTCTCTTTTTGCGAGAGTTTTTCTAAGTCTTTTAAGGAACAGTTGAAAGTCTTTGTGATCCAAAGAGCCATCGCTTGGGAGATGTGTATTGTCAT
ATGTGAGTGTTATGAATGAGTTGTTTTATGCAATTGGGCTTCGTGCATGCACCGAATAGCCCATTGTCTTGATCGTTCTAGACGGCATCCAACGC
ACTGCCCACAGGGCAGGTCTAAAGATCGAACGATGTCATGTTTCCGAACTTCGTCGAAAACAATTGATTTGTCAAAGCATTGAAATGCTTTGAGA
GGGTGATAACAAGGCAATGTGAGGTGCCTGGGGACTTTATTAGAGTCTCCAGCCTCCACGCATTGGCGAGTTTCGCATATTTGCGTGTTTTGTAT
GTGAAGCATGTTTACGGAAAGTCCTAGCGGACTTTTGCTTATTGCTGGTCTGCGACGCATCATTTTTCTGTCCTTGTTTATCGTGTTTTGTGGTTTG
GTGTCACCTAGCACAGTTACATCAAGTAGGTAACTGTGCTTCCGAGGTTCCAACAGGGGTTGAAACCTCGGTTTGGGCTGGTTTTACCAGTCCCA
TTTTTCAGCTTCGCTGCGATTTTCAGGGTTTCTAGAAAATCTATTAGATTCGCGGGATCATTAGCGAATCGTTCACGAATATTGGCTGGTAAAGCC
ATAAATTCGTTGTCTGAAGCCATAATCTGGTTCAGAGCAGAGTGGTAGTCATAGACACCACTAAAGTCGCCATATTGAGGCGTTAAAGGAGTTT
GAGGAATAAGTCCGGTCATACCAAACTTTTCCATAATATTATTAATATCGCATTCTTCAGCAAATTGCTGCTGAGTCAGAGTTGCATCCTCACAAT
GCAGCCTGACTCATTTGACGCAGCAATCGTGTCGTTGTACGGAGTACGAAGAAATGGGGCTGTAATCTTTGTCATTTTCATTCCATTGGTGGTTG
GTTATTTGTACGATTTTGTTGAATCGGTTGACCTCGATAGTTATCGATGTATCGTTTTGCGCCAGATGCGCTATACGCATCTTTGGCGATTTTTTTA
CGTCTTGATAAATTGGTTTGCTAGAACTAGGAGCTGACCCTGATTAGCAAGTTTTGTTAACTCGTTTGAATAACGAGATTGTGCCAAAGCTTGAG
CAGCTTGAGCACTTGATGTTTTGCCTATTTCATTTTCATGAAAGTATCGGCAAGTATTTGTTTATATTGAGCTCTAATATTTGGATTTTCATCCAATT
TGTTTAACGTATCAGCACGTACATTATCTGTTTGATTACTTGTGAGTTCTGTTTGAGCTTCAATTTGTTTAGATTGAGCAATAGCTTGATTTGCTTG
AGCAACCGTTTGATAAGCTTGGGTTCCGGACGTAGTTGCATTACCGAGAACATTTTGCATCTGGGCCATAGCTCCAGCTGGTGTTGTGGCTCCGC
CTTGTGAATACGCAAGCATGGGATTTAACCCAGCTTTTTTCATATCTTCAACTGCTCGTTGATATGATGTTCCAGACATTTCAGCTTGAAATCTCTG
TTGATTACTCGCTTGTTCTGCACTCGCTGCGTTTTGACTTTGTGTTCCAAAGTATTGTCCTGCTGCACCTATTGCTGCAGGTGCAAGTGCAGCTAA
GGAGAGTCCCCCAGTAGCAGGGGCAGCTCCTATAGCTATAGCAGGGCCGATTAAATCGGCAATTCCGTCAAATAGTCCCATTAGAAATGGTCTA
TTAAGCCAGGTACTGATACATTGGCATTGGTCTTGCCATTTTGACATCAAAAAATGAGTCAAATAAGAATTGTTGGCCATTTGCAGCTGCTCCAAC
GGCTACTACACGTGATACAGGAGGCGTATCTTGAATAAACGTATTATTCAAAGTAGGCGCAGCGGTAAATTTTTGAGCCAAATGCCAACCATCG
ATCGTGCCCGCCGATGTTGATTTGAACAAACCAGTAATTTTAGATGGTTTGTAACGATATTCCGCCCAGCGTTCTTGATATCCAAAAACAGTATTG
TCGGCTGCAGTATCTCCTGTTGCATAAATTTCTTTTTGCAAAACAGATTGTTCGCCTAGAGTAGCAAATGCTGGGAAATAAAAGTCATATCGTGTA
GATCTAGACCACATACGGTCTAATCCTTGTTGATAAGTAAGATCTGCTCTAATGGATACTAATCCAATAATAACGCCATGCTCAGTAAATGATTGA
GTAAATCCATGATTATGAGCGCGAGCAGCGTACCCATAGCAGCAAGGTTGCCCAAAGGGGTAGTCGTTCCAGAAGCGTTTGTTCCCGACGTTTG
AGCAATCGGATTAACATTAATTGGTGTTGAACCGCCTCCAAGGTATTCAGGCCTTTGTAAACGGGCGTCTGGGGAAATAACTCCAAAGTGACTCC
GGATAATTTCTGTGTATCGTGTTCCGCCACGTGCATCCCTTTCAAGTAATTTTTGAATTTGAAATGACTGTCTAATTGATTGACAGTTGCAGCAGTT
GCTTCTGATAAGTCGGTATATAAATTAGAAACGTTATTTACTACACCAGCAGTATTAACACCATAAGCGTTGCCATATCTAGCTAAAGCAGTCGTA
TTTCCAGGATCTGTTTGAATTACCGTAAATTTATCGTTTGATGTTGCGTCTCCTGAAATGGTATCCCATTTAATTGGAGCCGTAGTACCTAAAGGT
AAGGTGACACTCGCACCTTTTTGTGGCCATGGTAATGCTGACGTAAAGTAATCATGTCTTTTACCACGACGTTTTAGCACATAGTTTGAGGAAGT
ATCAGGGCCATCGCCCTTATCAACTACTGCGCTTGTTTGTAAATTTTCATCTCGGAACCATTCGTTCCAAATAAGATTGTATGCACGTGGCCAAAA
GGCACAGTGCGTAATAGTTCGGCCAGTATCAATTTGGCCTACTGTTGGTAAGCCCATATAGTCTTGAAGGCTGCCTACGGCATAACCATCTGTTG
GGCTTGTTTGAGTTGGGACAATATAAGATATTGAGTCTGTTGGATTTTCTTGTTGACCCATAAATTTTTGCCAGTTATTCCATATAAGGCGATTGG
GTACAAAGAAAAAGAAAGAATCCATAATCATGTTGTCCATGATTGGATATAAAGGCGTTGCTAGACGGGCAAATGCCGTCATTTTTAAGTTGAA
AGTGTCCCCAGGGAGCACTTCATCAACATATACAGGAATTAAATAGCCCGCATCGAAAGTCGTTTTATGTGTTTTTGAGCATCGAATTTACTACGT
GGTATATCGGCTCTAGGTACCATCGCGAAGCGGTGAGTATTAACTGACTGATTGCGGTGCATGTTTTTCCTTAGTGTTGTTCCGGGGGAAAGATA
AATCTCTTTTCCCCTCGGTTGTTTTATTAAGTTTAACTTGTTTTCCTAATGATAGGAGTTTTGGTTGTTCATGTAAATCGAATAACCCAGTTGAATCG
TCAAAAGTTCCGAATTCATATAGATCGAAGTCATCAGGGTGATTAAAAGTTGATTTTCAGTATCAGAACGATTAATTTCATCTGAAAAAGAGCGT
ATAGCTACTCCAGAGGAAGGTACGAACATTGGTCGTGCATATGCTTCAGCAGCACGGTCTTTTACGGAAGCGAGGATAAGTTTCATTATTTTCCT
AAGTGAGGTTACGTTTT

>000048F|arrow

TGATGCGTCGCAGACCAGCAAATAAGCAAAAGTCCGCTAGGACTTTCCGTAAACATGCTTCACATACAAAACACGCAAATATGCGAAACTCGCC
AATGCGTGGAGGCTGGAGACTCTAATAAAGTCTTCAGGCACCTCACATGCCTTGTTATCACCCTCTCAAAGCATATCAATGCTTTGACAAATCAAT
TGTTTTCGACGAAGTTCGGAAACATGACATCGTTCGATCTTTAGACCTGCCCTGTGGGCAGTGCGTTGGATGCCGTCTAGAACGATCAAGACAAT
GGGCTATTCGGTGCATGCACGAAGCCCAATTGCATAAAAACAACTCATTCATAACACTCACATATGACAATACACATCTCCCAAGCGATGGCTCT
TTGGATCACAAAGACTTTCAATTGTTCCTTAAAAGACTTAGAAAAACTCTCGCAAAAAGAGGACTTACAATCCGCTATTACATGGCTGGAGAATA
TGGTGAACTCTTCGCAAGACCCCACTTCCATGCCTGTATCTTCGGATACGACTTTCCTGATAAAAAATTATGGAAAAGGACTGCCTCTGGTTCTAT
GTTATATAGATCCGCAGAACTTGAAGCTCTCTGGCCATTTGGTTATACCACCATTGGAGATGTTACTTTTGAATCAGCCGCCTACGTGGCTAGATA
CATAATGAAAAAACAAACAGGGAAAGATGCGGAATCTCATTACAAACGCATACACCCTGAAACCGGCGAATATTTAGACTTAAAGCCGGAATAT
AATAAAATGTCTTTAAAACCGGGAATCGGTAAAGACTTTTATATAAAATATACTTCGGATATATACCCGCAAGACTACGTAATACTTAGAGGTAA

AAAGGTCAAACCACCAAAATACTATGACAAAATGTTTAAAATTGACCAACCTTATGAGTATGACGAATTACTTTACATGCGGGAAAATAATGCTA
AATTTAATTCCGAAGACAATACACCAGAACGACTATCTGCAAAAGAACAAGTCACTATGGCAAAACTTCAACTATTAAAACGTAACCTTACTTAG
GAAAATAATGAAACTTATCCTCGCTTCCGTAAAAGACCGTGCTGCTGAAGCATATGCACGACCAATGTTCGTACCTTCCTCTGGAGTAGCTATAC
GCTCTTTTTCAGATGAAATTAATCGTTCTGATACTGAAAATCAACTCTTTAATCACCCTGATGATTTCGATCTATATGAATTCGGAACATTTGACGA
TTCAACTGGGTTATTCGATTTACATGAACAACCAAAACTCCTATCATTAGGAAAACAAGTTAAACTTAAATAAAACAACCGAGGGGAAAAGAGAT
TTATCTTTCCCCCGGAACAACACTAAGGAAAAACATGCACCGCAATCAGTCAGTTAATACTCACCGCTTCGCGATGGTACCTAGAGCCGATATAC
CACGTAGTAAATTCGATGCTCAAAAAACACATAAAACGACTTTCGATGCGGGCTATCTAATTCCTGTATATGTTGATGAAGTGCTCCCTGGGGAC
ACTTTCAACTTAAAAATGACGGCATTTGCCCGTCTAGCAACGCCTTTATATCCAATCATGGATAACATGATTATGGATTCTTTCTTTTTCTTTGTACC
CAATCGCCTTATATGGAATAACTGGCAAAAATTTATGGGTCAACAAGAAAATCCAACAGACTCAATATCTTATATTGTCCCAACACAAACAAGCC
CAACAGATGGTTATGCCGTAGGCAGCCTTCAAGACTATATGGGCTTACCAACAGTAGGCCAAATTGATACTGGCCGAACTATTACGCACTGTGCC
TTTTGGCCACGTGCATACAATCTTATCTGGAACGAATGGTTCCGAGATGAAAATTTACAAACAAGCGCAGTAGTTGATAAGGGCGATGGCCCTG
ATACTTCCTCAAACTATGTGCTAAAACGTCGTGGTAAAAGACATGATTACTTTACGTCAGCATTACCATGGCCACAAAAAGGTGCGAGTGTCACC
TTACCTTTAGGTACTACGGCTCCAATTAAATGGGATACCATTTCAGGAGACGCAACATCAAACGATAAATTTACGGTAATTCAAACAGATCCTGG
AAATACGACTGCTTTAGCTAGATATGGCAACGCTTATGGTGTTAATACTGCTGGTGTAGTAAATAACGTTTCTAATTTATATACCGACTTATCAGA
AGCAACTGCTGCAACTGTCAATCAATTAAGACAGTCATTTCAAATTCAAAATTACTTGAAAGGGATGCACGTGGCGGAACACGATACACAGAAA
TTATCCGGAGTCACTTTGGAGTTATTTCCCCAGACGCCCGTTTACAAAGGCCTGAATACCTTGGAGGCGGTTCAACACCAATTAATGTTAATCCGA
TTGCTCAAACGTCGGGAACAAACGCTTCTGGAACGACTACCCCTTTGGGCAACCTTGCTGCTATGGGTACTGCTCTCGCTCATAATCATGGATTTA
CTCAATCATTTACTGAGCATGGCGTTATTATTGGATTAGTATCCATTAGAGCAGATCTTACTTATCAACAAGGATTAGACCGTATGTGGTCTAGAT
CTACACGATATGACTTTTATTTCCCAGCATTTGCTACTCTAGGCGAACAATCTGTTTTGCAAAAAGAAATTTATGCAACAGGAGATACTGCAGCCG
ACAATACTGTTTTTGGATATCAAGAACGCTGGGCGGAATATCGTTACAAACCATCTAAAATTACTGGTTTGTTCAAATCAACATCGGCGGGCACG
ATCGATGGTTGGCATTTGGCTCAAAAATTTACCGCTGCGCCTACTTTGAATAATACGTTTATTCAAGATACGCCTCCTGTATCACGTGTAGTAGCC
GTTGGAGCAGCTGCAAATGGCCAACAATTCTTATTTGACTCATTTTTTGATGTCAAAATGGCAAGACCAATGCCAATGTATTCAGTACCTGGCTTA
ATAGACCATTTCTAATGGGACTATTTGACGGAATTGCCGATTTAATCGGCCCTGCTATAGCTATAGGAGCTGCCCCTGCTACTGGGGGACTCTCC
TTAGCTGCACTTGCACCTGCAGCAATAGGTGCAGCAGGACAATACTTTGGAACACAAAGTCAAAACGCAGCGAGTGCAGAACAAGCGAGTAAT
CAACAGAGATTTCAAGCTGAAATGTCTGGAACATCATATCAACGAGCAGTTGAAGATATGAAAAAAGCTGGGTTAAATCCCATGCTTGCGTATTC
ACAAGGCGGAGCCACAACACCAGCTGGAGCTATGGCCCAGATGCAAAATGTTCTCGGTAATGCAACTACGTCCGGAACCCAAGCTTATCAAACG
GTTGCGCAAGCAAATCAAGCTATTGCTCAATCTAAACAAATTGAAGCTCAAACAGAACTCACAAGTAATCAAACAGATAATGTACGTGCTGATAC
GTTAAATAAATTGGATGAAAATCCAAATATTAGAGCTCAATATAAACAAATACTTGCCGATACTTTCATGAAAAATGAAATAGGCAAAACATCAA
GTGCTCAAGCTGCTCAAGCTTTGGCACAATCTCGTTATTCAAACGAGTTAACAAAACTTGCTAAATCAGGGTCAGCTCCTAGTTCTAGCAAACCA
ATTTATCAAGACGTAAAAAACATCGCCAAAGATGCGTATAGCGCATCTGGCGCAAAACGATACATCGATAACTATCGAGGTCAACCGATTCAAC
AAAATCGTACAAATAACCAACCACCAATGGAATGAAAATGACAAAGATTACAGCCCCATTTCTTCGTACTCCGTACAATTACGACACGATTGCTG
CGTCAAATGAGTCAGGGCTGCATTGTGAGGATGCAACTCTGACTCAGCAGCAATTTGCTGAAGAATGTGATATCAATAATATTATGGAAAAGTT
CGGTATGACCGGACTTATTCCTCAAACTCCTTTAACGCCTCAATATGGCGACTTTAGTGGTGTCTATGACTACCACTCTGCTCTGAACCAGATTAT
GGCTTCAGACAACGAATTTATGGCTTTACCAGCCAATATTCGTGAACGATTCGCTAATGATCCCGCGAATCTAATAGATTTTCTAGAAAACCCTGA
AAATCGCAGCGAAGCTGAAAAAATGGGACTGGTAAAACCAGCCCAAACCGAGGTTTCAACCCCTGTTGGAACCTCGGAAGCACAGTTACCTACT
TGATGTAACTGTGCTAGGTGACACCAAACCACAAAAACACGATAAACAAGGACAGAAAAAA

>000169F|arrow

GCAAATGAATGCGTACTTACTGACTTATTGCGAAACATAAACATCTCCCGTAGTTCCGTACCACTCTTTCGAGTGATACGGTATAAAAAAAAACCT
TACTCGCCTTCGCGAATCATTACATCTTTTGCACGAGCAATCAACTTAGGGCTTTCCAGTAGTTCAAAAACACCAGTGGCAATCGTCAAAAAGACC
CAAATAGAACATATGAAAATCATCAGGATGTTATACAACCTGATTATCTTCGCTAGCTCGATTGACTTCATCCTGAAACTGACGTACTGCAACACC
TTCAGATGCAACATAAGCTGGACGACCAAAAGCATCTGCAGCAGTATCCTTAATAGAAACAATACCATCTTCATAAAAACTCCTTAAATAGTACG
TTTTAACAATGACAACTTAGCCAACGCAACTTTTCCTTAACAGCAAGTCGCTCAAGCGTGTTGTCCTCATGCCTAGATCGACCTTCCATCTCTCTGG
CAAACTGAATCATATCGATTCTTCAGGAAACTTCAACTTAAATTTATTATCATAAAACCGTGGTGGACGGCACTTTTTGCCACGCACCACAACGTG
TCTGACGTATAAACGTCTGACATGTACTTATCTAACCACGATTGCCCGATACCGGGCTTCAATGACATCTTATTAAATTCTTGGCTTACGGCTGAA
TTATCTCACCAGTCTCTAAATCACAATATTGATAATGGCACCCGCATCAACCACTTCGTGGTTTTCATTGACAGTAACCCCATTAATCTTCTTCATA
ATATATCTTGCAACATAAGCAGCAGACTCAAAAGTAACATCACCAATTGTAGAATAGCCAAACGGCCACAATTCTTCCAAAATCTCTGACGTGTA
GAGGATAGAGCCAGTCTGCGTTCTTTAAATATTTTCTTATCCGGAAAATCAAGACCAAACAGACAAGCATGGAAATGAGGACGATCAAAAGATT
CACCATATTCACCTGCCATATAAAAACGTATCGTTTTCCCCAGTAAAACGCTTACGTAACCGCTTCATAAAAAGCTGATATCATTGTAATCCAATG
ACATATCCTAGGACAATGCTCTGGAGCATATGTCAAAGTAATAAAAACAATTACTAGTATGCATTTGTGCCTCATGCATACAACGAATCGCCCACT
GACGTGAGCGTTCAAGGCGAACAACCAACACACTGACCACAAGGCAATGATAGGGTACGGACTACATCCGCACCCGGTATTTCCCGCCAAATTA
TAGACCTGTCACTGCATTGATAAGCCGTTAAGGGCTTATAACAGGCCATATTACAAACGATAGCCACCACGCTGGGGAGCGTGTCTCATATTAAT

TGACTTCGTCTTACTAGCAGTTCTGCGAATGACTTTGCAGATTTATATTTGTTTACTGGCTTTCCTTCGTAACATGATGAACTCCGTAGTTAAAATA
GTGGTTGGTGTCACCTAGCACAGTTACATCAAGTAGAGTAACTGTGCTGGCCTCAGGATTTTCATCCTTCGGCCTTAGGTGTTTCTGTAGAAACG
ATGGGTTCAACCACAGGTTGTCCATCAATAAGACCCAATTGAATCGCTTCATCACGATTCTGGTCGTTCTCAAGGAACTCCAATAATTTGACAGG
ATCATGGTCAAATCGGACTCTTAATTTCGCTGGCAGAGCATGAAATCGTCCATAGTTGCGTTAATTTGATTCAACGCAGAATGGTAATCAGTAAC
ACCACTAAAATCGCCGTATTGAGGCGATACAGGGGCCGTTGGAAGTTCCCCTGTAACCCCGAAACGTTCAATGATGACATTAATGTCACATTCAT
CTTTCATATGCTGTTGAGCAAGGACTTGGGTCTTGACACAGCAACGCATCGTTTACCGATGCTTCATCTTTATCATAGTTGTACGGATTACGTACA
AATGGCAAATTCGCTTTAACTCATTATTTGACTCCAATTCCCCAAGGGGTTAGTTGATTAGGATTATTTTTGAGTTTATCAAAAATTTCTTCGAAGC
ATCGATACCCCTAGACCAAATAGCTGGGGATGGAGCCATAGCCTCTTAGTATTATATGACTGAGCGGATGTTAAATCCGCAGTCGTATTCTTTAA
AGCAATATCAGCCAAAATACGTTTATTTTCGGCTGTAATATTAGGAGCAGTAAGCAACTTATTAACAGTATCAGCCCTAGTATTAGCGGTACCAG
CTTCAGTAGCTTCAGTCTGGGCGATAATCTGCTTTTCTGTTTCTGATGCATTACGAATCTGTTGCATCATAGAAGCAGTATTAATAGCTGAATTAG
TTGCATTACCTAAAACATTTTCCATAGTAGCAGTTGAACCAGATGGGGTAGAAGCACCACCACGTTGATAGGCTAACATAGGAGATAAACCAGC
AGCTTCTAAATCTTTAACAGCACGCTGGTAACTTGTTCCACTCATATCGCTTGAAAATCCATTTGCCTCTGAGCCATCTCCTGATTAGCTTTATTAG
CAGAAGTAGAACCTAAATAAGAACCAACAGCGCCCAAAGCAGTACCGACACCAGGAGTAAAGAAACTAGAAGCTGAAGACAGCTTAGAACCAA
CATTAGTAACCGCATCAAGTATTCCACCAAACATAAACTAACGCCCTTCGGTTGTTCCTCACTACTCCTTACGGAGTAGTCGAGGTTATAAAACAT
TAGAAATGATCAATTAGACCAGGTACGCTGTACATAGGCATAGGTCTGGCCATCTGACAATCAAGAAAGCATCTCATTAAAAATTGCTGACCATT
TGCTGCAGCTCCGACCGCCGTAGTACGAGCAACTGGAGGGGTCTCTTGAATAAACGTTGAATTCAACGTAGGAAGAGAAGTAAATCGCTGTCCA
TAATGCCAAGCATCAATAGTGCCAGCAGAAGTAGACCTAAAGAAACCAGTAATTTGAGAAGGTTTGTAACGATACTCCGCCCAACGTTTCTTGAT
AACCAAATACATCATTGTCGGCTGCAGTACCTTGAACATAAATTTCTTTGTTCAAAATAGCTTGCTCACCCAAAGTGGCAAATACTGGGAAATAAA
AGTCATATCGTGTAGACCTAGACCACATCTTAGGAAGACCCTGTTGATATGTGAGGTCAGCACGTACAGAAACTAATCCGATAATGTATCCATGT
TCTTGAGCAGCATACGTAAAGCCGTGTCCCTGAGCCAATGCAGTACCCATTGCAGCAAGGTTACCTTGCGGAGTAGCAGAACCAGTAACCGACG
TTGCAGAAGTCTGAGCAATCGGATTAACATTAACAAGGGTCGAACCTCCACCAATATATTCAGGACGTTGTAAACGATAATCCTGTGGAGTTACT
CCAAAGTGAGCACGTAGTAACTCAGTATAACGAGTACCACCACGTGCATCGCGCTCCAATAACTTCTGAATCTGGAAAGATTGACGAAGTTGGT
TAATAGTCGCAGCAGTAGCAGTAGACAAATCAGCGTATAACTGATTAGTAGCAACACCAGCATTTGTACTATTAGACAAAGTATTAGAAGCTGA
ATTTAATTCTCTTAAAGCACCAACAGCAGTTTGAAAAACACTATAACCAGCAGTACCATTGCCAGCTGGTATGTCAGAATAAATATTAGCACGTGT
TCCTAACGGTAAAGAAACAGAAGCACCCTTCTGTGGCCAAGGCAATGCACCAGTAAAATAATCTTTACGCTTACCACGTCTCAAAAGTGTATAGT
TAGCAGGAACATCACCTGAATCACCAAGATTTAACGTTACAGAATTCTGTAAGTTTTCATCTCTAAACCATTCGTTATAAATCTCATTATAAGCAC
GTAACGGCAAAACATTATGCGTTACTGTATTAGCGCCAGTAATCTGACCAGCAGTAGGTAAACCAAAATAATCAAAAATTGAACAAACAGCATA
ACCACCAGCAGGACTAGTAATAGTAGGCACTACAAAAGAAATAGAATCACCAGGATTCGTTTGTTCACCCATAAACTTTGGCCAATTGTTCCAAA
GTAAACGATTAGGTACAAAGAAAAAGAAAGTATCAAGATGCAAGTTGTCCATCACAGGAAATAACGGTGTGGCCAAACGTGCAAATGCAGTCA
TCTTTACACGATGAGTATCGCCAGGCAATACTTCATCACAATAAATAGGAACTAAAAAACCAGCATCAAACGTGGTTTTATGAGCATATTGAGTA
TTAAAACTAGAGCGGGGAATGTCCGCTTTAGGAACCATA

>000137F|arrow

ACCCCATGTATTGGTATGAATTTGGAGGGACAACCCTTGTCCACTATACCGCTTTTAAACGTCGAGTTACCCAGCTTGTGTTGTGGGACACCGAC
AATGTCAAGCATGTTATCGATGCCCCTTACAGAGGAGTGGGACCTGCTCCTGTCGTGAGTCCTATGTGGGACGCATTCTGGGCATGTAATTATGT
TAATGCTGATGAAGCATACGAGTTTTTTAAACAGTGATTTTAATTAAAGAACAATTGAAGCTAAAAAACCGTATATAGGAAAAGCTTTAAATTGA
ATTAAATTGTTGTTTTCATGGGATTCGCCTTCGAAAGAGCAAAAATACACCCACTTGCAGAACCTGACAAGTAACCACCAACAGCAACAAAACTA
CCACGAGGTGTGGCACCCACATTTATCCCGTTGTTGATGGCGCTAGCATTGATAGTAACATTCACACGAGCGTTAAAACCATTCTCTATCCTAGAA
AACGTAATGCCACCAGTGATGTTGGAAGAACACTGAAAGTCCAATGGTTGTCCGCTGTTACTGCCACCTTGAGCACCGAGATCGATGTCAAACG
TGCCATTGCCAACACTATCAGGAAAATGGAGAACCCAGTACCCAGTGTTAGCAGGACCAAGCCAACACGACGACACGATCCACCGCTCATACCC
GAGACGTCTACTTCCAGAATCAAGTCTCAATTTTGATACATGGTCAAGAAGAAGAGTACTATCGTAGCTACCGCCGAGCCACGTATTGTTCTGGT
TAAAACTACATTGGTTCTTCAACACTTGATAAACAAGCGATCCCGACTGAACCTTTTGTTGCGTCAAGTTAGGAATAGTGGTAAACGCAGGAACA
GAACCAAGAGAAGGATTAGTAGTAACGCTCAAAAACGGTAAATCCATACCAATTGCTTGGCAAAACTTTGGCTTCCTAAGCGTTACTGTATACTC
TACCCAAAGCTCACCAATCTGTCCTGCAGTAACAGACTGGTTCACCGCTAGCTGAAACAAACCCATGTGGTACGTCTTCGGATCTTCTCCTACAGG
CGTCATACCCATAGGTGGGGTGTATCTTATACCTCCCATTGCACTCTTTGCAGGATCACATTCAATTCCAAATGTTATGTTTTCGCTAATTCGTGCA
GAGACAGATCCGTCGTATTGCATCATTTGTATTTTGTTTGTGAATTCTTCGTTAGTACCGGCGTTGTAATCACATACCATAATAATGGTTCCCATTT
GCTGTGAAGTAGTTGACAAGTCTGTAGTTACTGATTTGTAGTAGATGATCAGCTGTTGAAATTCATATTCGTCGAAGTTACAAGCGAATTGAGAC
AGGAAGGGGAATAAATCTGCGTTTGCAGGGTTAACGAGGTACGAGTTGTTTTCGAAAGTTGTGCTACCCGCTACATCTTGTATGTATTCGCGAT
GTTTAATTGTGAGTGCACCAGTTTCATCCATGGATGAAGACATGCGTGGTGCATCTAAGTCTCCTTTAACGGGATTGATGAGACTGTTGTATGCG
CCAGATCCACTGAAGCCATAACCTTCAGCTGTTTTACGTGCTTTAGCATCTAACCAGTCTCCTGCTTTGTCTCCTTGCTTGGCTCCCCAGTCTCCCAT
TCCGACAAGACCTCCGAGGCTACGTCCGATAAGACGCCCCCAATAACCTCCTCTACCTGTGTAGCCGAGTGTTTTTCTCTGCAATCTTTGGGCATC
GTTAGCATCTTTATAAGACGCTCCAAAAGCTGCCAGATTGTCCGCACTACCACGGAGTGGATACGCCCTTCCGTATTTTGAGATGGGGAATCTTT

TGTTTGCTTTTTTGTAGGCTGTGAACCCTTTCGGCGCTTTGCGCCTACCGTACGCAGGCATGCCGTAAGGCATGTATCTTCGTCTTCCATAGCTAC
GGCGTCCGTACATTTAACCTGCAAAGTGTATTTTAATTGAAAGCGATGTATAAATCGAGTAGAGCATCGACGTGTTCTTGGCAAAACCAAGGTG
GTGTAACTATGTCGTCTGTGTCTATCTCTTCATCTTCTTTCATTTGCATTATTTTTTCAAGTAGCTGTATCATAGGTACAGGCATCTTTGGGAAAGTA
GTTATTAGCCACATAGAAAGTTTTCCTTTAGGAGGACATGCTTCTTGTTTGTAAAGAGTTTTAAGGGTTGTCTCAGCTGTCTTGTTTTCCATTGTTC
TGTGTGAGTGTAAATGGAATGTAACACAAAGATAAGACACACATTGACACCAATATTATAATGAACCACAATGACAAAGGCCTCTGAGACGGGA
GTCAGATTATAGAAAACCCTGCCGAACGTGGAGAAACGTGGCGGGGTCCCCTCTGGGGTGCCGCGTTTCGTAACCGAGGCATGAAAAAAAAAA
AAAAAAAAGAGGACTTTTCTACTGATGTTATTGTGGTTGTCCCCATGATTGTCTCCATGCTCGTGGTGGTTGATGGACGTTTGCAGATGTGAAAT
GTCTTCTGCATATTGGACAGTTTCCTGCCAATCTCCACCAATCATATAGACAGTCGTCGTGGAACAAGTGCATACATGGTGGGTATGAGATTGGC
ATTATGCGTCTATTTTGTGGTTCGTTAATGAGTGGTTTTAAGCATATAGGACAAGGTCTATTGATCTGTTCTGAGATTCGTATTTGTCTAGCATGCT
CTTCAGCTGCTAGTCTGTCTGCTTCGCGTTGTTGCTCTTCTTGCCTGCGTGCAATCTGCTCTTCGCGCCACTGTTGCTGTTGCGCAACAGTGCCGTA
TAGAATGTCATGTAGGTTTTCTTGTTGTTGTGCGCGATGTTGTTGCGCGCCTGCGTTTGCACGTGTGTTTGTTCGTGCTGGTGTATTGAGGGCATC
TTGTTGTTGCTCTCGTATTATTTCGGCTAGAGATTTTGGGGGCATCTTTTTTGTGTGCTAAGTTAAAAAGTGTGAGCGGACGTGAGCGGGAAAAA
TATGGACCGCGGTCATGGTCCGCGGACTAGTCTGCAGACTCAGTTCATGTGAAACACTGACCACGAAAAAAAATAAAATTTTAAAATTGACCAC
GAAATATTTTTGTTGTGAATAGTGAGTTTGAGCTCACTTTTGTGAATCACAAGTGAATCACATTTTAGTGTGAAGTGGCCGTCAATCAGTATTACA
GGCCACTTCCTCACACTCACACTTTTGTGTGATGTTTTTTGTGGCTTTTTGTTGCAGATGTCTAAGTCAAACAAGCGCACCTCAAGGTTCATTGATT
CCGAAGCCGGTTGTGGATCCGGTTCTGAGGATGAACAAACCATGGACGATGCGACGCTGTCGGACATGGACTTCATTAAGGATGAACCTTCTGA
GTCTGGTGAAGAGTCACCTGTGCGCCGCCCTCGAAGGAAGCTGAAGAGGCGTGGTGCTATCGTCAAGATGGAGCCTGGTGTTAAGAAGGAAG
CCGTGAAGGCTTTTGTGGATTTACTTGACGATGACGAAGGCTTTGAGGTGTTACCTTTACCCCTTCCTCCTACCAGAGCTGCGGCGTGGCGTCCC
GCAGATCAAGAGGATTTCAAGATGCCAATCTTGCCTCGAGCGCAGGCGAGAGGACGGGTGTCCCCAATTGGCGGCGGAGCCGCCAACGCCGG
CGGAGCCGGCGACAGGGGCGTAGCCCCTGTCAGGGCCGAAGGCCCTGCCGGGGCCGGAGGCCCCGCCGCCGCCGTAGGCGGCGGAGAGGAG
AAGGGAGAGGCGAAGGTCGATGAACGTTTTGCGGACGTCGTACTTCCTGCCGATGTGTCTGAGGAGCAGCAGCAAGCAGCGGACTCAGGCAA
GCAGACTAAGTACTGGTGCTTTACTTCTTATGGCCATGACGCTGAAGAGTGGAATGGTCAAGAGTTTGTGGATGCTTTGGAGCTGAAGCATGGC
ACTAGTATTGGGTATTGCATTATGCAGCACGAGAAGTGCCCCGAGACTGGCCGTGATCACTATCAAGGCTATATGGAGTTCAAGAAGATTCAAC
GCATGTCTGCGGTGAAGAAGGTATTGCCTGGCGCTCATCTGTCGACCCGTCGTTCTACTGCCGAAAGGAATGTGTTGTACTGCAGCAAGGTTGA
TACCCGTGTTGCAGGTCCGTGGCAGTTCGGTGAAGTATCAGGTGAGAAGCAAGGCCAGCGCAATGATATCAAGAACTATATTGATGTTATGTCG
TCTGAGGGTCAAGCTGCTGCTCGTCGTGCTGATCCGGTAACCTTTGTGAAATTTCACAGGGGATTTGCGGCCCTTGCGTCAGCGGATCTCCCTCC
TGCGCACGAGTTTGCTCGAAACGTCATTATTTGCTACGGACCCCCTGGATGCGGAAAGACCCGGATGTTCTACGACACTGTCGCAGAAGCCAAG
GTTTCCTCTGCTGTCGTTGACGTTAGCCAAGGCTGGTTTGATGGAGTTGAGAATCAGGAATACGTACTGCTTGACGAGTTCGTCGGAAAGCATG
GCGCATTTAAACTCTCAGAGCTTCTTAAAGCCACTGACCGCTACCCTATCCGCGTTCCTGTCAAGGGATCGCATGCTATCTGGAACCCTACCCACG
TTTTCATTACCAGTAACATGC

>000200F|arrow

TCGATATGATTCAGTTTGCCAGAGAGATGGAAGGTCGATCTAGGCATGAGGACAACACGCTTGAGCGACTTGCTGTTAAGGAAAAAGTTGCGTT
GGCTAAGTTGTCATTGTTAAAACGTACTATTTAAGGAGTTTTTATGAAGATGGTTATTGTTTCTATTAAGGATACTGCTGCAGATGCTTTTGGTCG
TCCAGCTTATGTTGCATCTGAAGGTGTTGCAGTACGTCAGTTTCAGGATGAAGTCAATCGAGCTAGCGAAGATAATCAGTTGTATAAACATCCTG
ATGATTTTCATATGTTCTATTTGGGTCTTTTTGACGATGCCACTGGTGTTTTTGAACTACTGGAAAGCCCTAAGTTGATTGCTCGTGCAAAAGATG
TAATGATTCGCGAAGGCGAGTAAGGTTTTTTTTATACCGTATCACTCGAAAGAGTGGTACGGAACTACGGGAGATGTTTATGTTTCGCAATAAGT
CAGTAAGTACGCATTCATTTGCTATGGTTCCTAAAGCGGACATTCCCCGCTCTAGTTTTAATACTCAATATGCTCATAAAACCACGTTTGATGCTG
GTTTTTTAGTTCCTATTTATTGTGATGAAGTATTGCCTGGCGATACTCATCGTGTAAAGATGACTGCATTTGCACGTTTGGCCACACCGTTATTTCC
TGTGATGGACAACTTGCATCTTGATACTTTCTTTTCTTTGTACCTAATCGTTTACTTTGGAACAATTGGCCAAAGTTTATGGGTGAACAAACGAATC
CTGGTGATTCTATTTCTTTTGTAGTGCCTACTATTACTAGTCCTGCTGGTGGTTATGCTGTTTGTTCAATTTTTGATTATTTTGGTTACCTACTGCTG
GTCAGATTACTGGCGCTAATACAGTAACGCATAATGTTTTGCCGTTACGTGCTTATAATGAGATTTATAACGAATGGTTTAGAGATGAAAACTTA
CAGAATTCTGTAACGTTAAATCTTGGTGATTCAGGTGATGTTCCTGCTAACTATACACTTTTGAGACGTGGTAAGCGTAAAGATTATTTTACTGGT
GCATTGCCTTGGCCACAGAAGGGTGCTTCTGTTTCTTTACCGTTAGGAACACGTGCTAATATTTATTCTGACATACCAGCTGGCAATGGTACTGCT
GGTTATAGTGTTTTTCAAACTGCTGTTGGTGCTTTAAGAGAATTAAATTCAGCTTCTAATACTTTGTCTAATAGTACAAATGCTGGTGTTGCTACTA
ATCAGTTATACGCTGATTTGTCTACTGCTACTGCTGCGACTATTAACCAACTTCGTCAATCTTTCCAGATTCAGAAGTTATTGGAGCGCGATGCAC
GTGGTGGTACTCGTTATACTGAGTTACTACGTGCTCACTTTGGAGTAACTCCACAGGATTATCGTTTACAACGTCCTGAATATATTGGTGGAGGTT
CGACCCTTGTTAATGTTAATCCGATTGCTCAGACTTCTGCAACGTCGGTTACTGGTTCTGCTACTCCGCAAGGTAACCTTGCTGCAATGGGTACTG
CATTGGCTCAGGGACACGGCTTTACGTATGCTGCTCAAGAACATGGATACATTATCGGATTAGTTTCTGTACGTGCTGACCTCACATATCAACAG
GGTCTTCCTAAGATGTGGTCTAGGTCTACACGATATGACTTTTATTTCCCAGTATTTGCCACTTTGGGTGAGCAAGCTATTTTGAACAAAGAAATT
TATGTTCAAGGTACTGCAGCCGACAATGATGTATTTGGTTATCAAGAACGTTGGGCGGAGTATCGTTACAAACCTTCTCAAATTACTGGTTTCTTT
AGGTCTACTTCTGCTGGCACTATTGATGCTTGGCATTATGGACAGCGATTTACTTCTCTTCCTACGTTGAATTCAACGTTTATTCAAGAGACCCCTC

CAGTTGCTCGTACTACGGCGGTCGGAGCTGCAGCAAATGGTCAGCAATTTTTAATGGATGCTTTCTTTGATTGTCAGATGGCCAGACCTATGCCT
ATGTACAGCGTACCTGGTCTAATTGATCATTTCTAATGTTTTATATAACCTCGACTACTCCGTAAGGAGTAGTGAGGAAACAACCGAAGGGCGTT
AGTTTATGTTTGGTGGAATACTTGATGCGGTTACTAATGTTGGTTCTAAGCTGTCTTCAGCTTCTAGTTTCTTTACTCCTGGTGTCGGTACTGCTTT
GGGCGCTGTTGGTTCTTATTTAGGTTCTACTTCTGCTAATAAAGCTAATCAGGAGATGGCTCAGAGGCAAATGGATTTTCAAGCCGATATGAGTG
GAACAAGTTACCAGCGTGCTGTTAAAGATTTAGAAGCTGCTGGTTTATCTCCTATGTTAGCCTATCAACGTGGTGGTGCTTCTACCCCATCTGGTT
CAACTGCTACTATGGAAAATGTTTTAGGTAATGCAACTATTCAGCTATTAATACTGCTTCTATGATGCAACAGATTCGTAATGCATCAGAAACAGA
AAAGCAGATTATCGCCCAGACTGAAGCTACTGAAGCTGGTACCGCTAATACTAGGGCTGATACTGTTAATAAGTTGCTTACTGCTCCTAATATTA
CAGCCGAAATAAACGTATTTTGGCTGATATTGCTTTAAAGAATACGACTGCGGATTTAACATCCGCTCAGTCATATAATACTAAGAGGCTATTGG
CTCCATCCCCAGCTATTTGGTCTAGGGGTATCGATGCTTCGAAAGAAATTTTTGATAAACTCAAAAATAATCCTAATCAACTAACCCCTTGGGGAA
TTGGAGTCAAATAATGAGTAAGCGAATTTGCCATTTGTACGTAATCCGTACAACTATGATAAAGATGAAGCATCGGTAAACGATGCGTTGCTGT
GTCAAGACCCAAGTCTTGCTCAACAGCATATGAAAGATGAATGTGACATTAATGTCATCATTGAACGTTTCGGGGTTACAGGGGAACTTCCAAC
GGCCCCTGTATCGCCTCAATACGGCGATTTTAGTGGTGTTACTGATTACCATTCTGCGTTGAATCAAATTAACGCAACTATGGACGATTTCATGGC
TCTGCCAGCGAAATTAAGAGTCCGATTTGACCATGATCCTGTCAAATTATTGGAGTTCCTTGAGAACGACCAGAATCGTGATGAAGCGATTCAAT
TGGGTCTTATTGATGGACAACCTGTGGTTGAACCCATCGTTTCTACAGAAACACCTAAGGCCGAAGGATGAAATCCTGAGGCCAGCACAGTTAC
TCTACTTGATGTAACTGTGCTAGGTGACACCAAACCACTATTTTAACTACGGAGTTCATCATGTTACGAAGAAAGCCAGTAAACAAATATAAATCT
GCAAAGTCATTTCGCAGAACTGCTAGTAAGACGAAGTCAATTAATATGAGACACGCTCCCCAGCGTGGTGGCTATCGTTTGTAATTATGGCCTGT
TATAAGCCCTTAACGGCTTATCAATGCAGTGACAGGTCTATAATTTGGCGGGAAATACCGGGTGCGGATGTAGTCCGTACCCTATCATTGCCTTG
TGGTCAGTGTGTTGGTTGTCGCCTTGAACGCTCACGTCAGTGGGCGATTCGTTGTATGCATGAGGCACAAATGCATACTAGTAATTGTTTTATTA
CTTTGACATATGCTCCAGAGCATTGTCCTAAGGATATGTCATTGGATTACAATGATTATCAGCTTTTATGAAGCGGTTACGTAAGCGTTTTACTGG
GAAAACGATACGTTTTATATGGCAGGTGAATATGGTGAATCTTTTGATCGTCCTCATTTCCATGCTTGTCTGTTTGGTCTTGATTTTCCGGATAAG
AAAATATTTAAAAGAACGCAGACTGGCTCTATCCTCTACACGTCAGAGATTTTGGAAGAATTGTGGCCGTTTGGCTATTCTACAATTGGTGATGT
TACTTTTGAGTCTGCTGCTTATGTTGCAAGATATATTATGAAGAAGATTAATGGGGTTACTGTCAATGAAAACCACGAAGTGGTTGATGCGGGTG
CCCATTATCAATATTGTGATTTAGAGACTGGTGAGATAATTCAGCGTAAGCCAGAATTTAATAAGATGTCATTGAAGCCCGGTATCGGGCAATCG
TGGTTAGATAAGTACATGTCAGACGTTTATACGTCAGACCACGTTGTGGTGCGTGGCAAAAAGTGCCGTCCACCACGGTTTTATGATAATAAATT
TAAGTTGAAGTTTCCTGAAGAAT

>000210F|arrow

CCTTTTAGAGCTTTAACTCCCTTCCATGCTTTCATAGCGATGTCAGTATAGGTACATTTTCCTTTTCTTGCGACAGTTCGTTTTCTATAAATTTTTTTA
TAAGCCATTGCTTATTATAGAAAAAGAAATTAAATTTCTTGAATTAGATTGGGTTTTTTAAATTTAAATTGTGCTTTATGTAGGATACAATAAATTC
AATCGTGCTTCATCTTTTTTATATATTCTCTCCAAAGAGAAATATCATTTTCAGTATGTAATACTTGAAAATCTATGCGACCATAATTCTTTTATAAT
TGCTGATCATACATTCTACATTGATAGCACGATTCTTATTATCTTCCATTTTTAACCATATATGAATATGGGGGTTATTTGGATTATCTTGACCCTG
GAATGGATGACCGATTTTATGTTTAAAATATTCTATATTTAATTCCCAATCCATATTTTTCCTGAAAAGTACCTTGGTATTCTTTTTATGAGTTTCAT
CTTATCCTTAAATGTAAACTCTGGTCTAAAAGTTATCGTGATGGCATAATTAACAAATTCCGCCATGGGGGACAACAAGATACTGTCCCCCAAGA
AAAAAAATTTTCTTAAATTCCTTTTTAAATGTTTAAAAAAGGGAGTGACTATTTTTATTAAAAATAAAAATTGTCTCAAACTCAAATACGAAAAAG
AAAAAAAAGATCATAAATGACTTTTTTTCTTATCGTTTTTCCCTCGAAATTTAAACTGTATTTATTTTTAATTATTATTACTTAAACTCTAGAGTTAAT
TTCTTTCGAGGGAATGGCCTCAACGCACCCACCTTCGGTGCGAGCCGGCCAGGCTTATAACTCTTCCAATACCTTAATTATCGTAATATGATAATC
TAGAAGAGTAAGTAAAATTAGGTACATTTGTAGATTGATTAGAAACCCAAAAAATATAGTAATGACCTTTTTGAACATCAGTAGATGCTGTTCCA
TTCCATAGTAAATGAGAATCTATTACATGAGAAAATTCTACCATAGTAGTAAGACGTGAAGTATCATAAACAAAAGTCTTTGCGTCTTTAATTTGG
AATCGTTTCTTAGTTTGCCAATTAGGAAAACTTTGAATATCACTGGTAGAAAACGCATCAGTAAATACCGGATTGGAATCCGATACTTGCTGCAT
ATCTTTAATAAGATATACTTTAACAATAGTTCCTGGAGCTAGCGCGTTATTAGTAATAACATACTTAAGGTTTAGAGTTTTAGCTAAAACACTATTT
CCATTTCTCTGAGCATCAGTATCACCTTGAGCAATACCTGAAATCAACGATATAGCACCTGTGCTTGATGCGTTGAAACTATTATTTGAATAGTGT
GATTTTTCAATTCACTATTTATTATA

>000230F|arrow

AGAGTTTCCTCCCTCCTTTTATACTCTCGACCGTGCGTATGAGTCATCCACCTCTTCTGACCTTTCACACGGATAAACCGTGGACTCATCGTCGCAG
GTCACGGTGGATGAGTCATGGCAATCGATCGAGTTAAGATCGAAGTTGATGGCCCCTCTCACTGCCGTGCCGGGGAGGCAAGGCTGGATCGAG
ATCGAGAATCGATGAGAACGGAGAACGGCGATTAGTAGGTAATACTTAGGAGCGATTCTCACTCCTGTCTACTAATCGATATTTCAAGCTGAGC
GCCGGGTGATGCTGCGCTACGGCTGCCGCCTCCACTTTCGCGTGATGGGTAAAGGTAGATATACATATATTAACCAGCAATGTGAATAGGATAG
ATTTCAGTTCTAGTCGATTTCTTGAAGGTGAACGAGAGTCCATACTTGACTGAGTGTTCATAAGCCAAAGACATAGAAAATGCTGGAGTTACTGC
GTTGGTCTCAATAACCTTTTCAAGTTGAAAGAATCTAAACTTACCAAAGGGAGACTGGTAACGGTTCAAACCAGCAATAGTAGGTTGATTATATC
GGCACAACCATTCATTAAGTCCCATACTGAATTTGTGAAAGAGTTTGGATACTTTGATTTCACCAGGCAACATTCCAACAGGAGCAGTTTTCTTTA
CATTGATGTATTCATATCCATTGCCAGGTTCCAAGATAGCTTGATTGTCTGAATCAGTTCGTTTAATGAGTCCATTAACTTGATCAGCGAGAAGGG

AGGTATCAGCATTGAAGATTGAGGTTTTTCTCAATTTGCTAGTACCAGTTCCATTACCTTGATAAGCTTTGCCTTTAAGAGGAACTGCATCAACTT
CGTCGATAGTATCGTCACCAGCGCCAGGAACAGTGATATTTTGCATTGTAAGGTCAGATTGAACCCATCCATTGATGCGAGCTCCATCTAAGCCT
AATGACCAGTAATTAGTGTCACCAACTGCTGATTGGTAATACAATTCAACAAATTCAATCTGATCATTTTGAGTCAGCATGAAATTTCCAAGTGCG
TCGGCCAAATTCAACCAAGTGCTTCCAACTACGATTGAATACAGTACTGCGGTTCCAGGGGCAGTTGGAGCATCCACTCCAATAAGTCTGTATAA
GATATTTAATGTATCTCCAACATTGACTTTGACAATTTCATTTTCAAGTCCTTCAATAGGTCGATCCATTTTGATGGCAAGTGATTTGATTAGCGCT
TTTGCAAACGAACCAATGAGTTGAGTGCGAACATTTGTGGTATGTCCAATAGTGATTACTCGAGGGGAAGTGATAGATCCACCAGCTTCATACG
AGATAATAACTCCTTTACCAGCTTTTCCAACCGACACTTTCTTGAAGCCTTTGGACTTCTTCTTGTTATTACGTCTCTTTTTCAAGAGACCTTTGTTG
AGTCGTTTAGCTGTCTTAAATCTCTTTCGAGTCATTGCTGCTCGAGATTTACGATAAGTTCTAGTTAATTGGGAAGAAGCACGTCTTTTCATTTTTT
ATTGGGTAAACTACCCATTACTAGCTATTACTCTAATTGTATCCCAAGCTGCTTGTTGCGCTCTTCCATGTGCTGCTTCCCAACCAAACCTTGCTCC
CATTACGGCTCCTGGTACATTTCCTGCTATATATCCTAAAGCAGCACCAGATCCTACAGCTGCAGTGTCCCACATGAAATGCGCGACATCAGCTCC
CAACCTCTCCAACCCTTTCCAAATAGCAGCTTCCATTTGGTTGTCTAAAGAGTTGGTAGAAACAGGAGTTTTCCATTGCGTATCACCAGCCTTCGG
GGTGGGTTTAAGCATTTGCTTTCTTTGAATACGATGGTGGGTTTTCGAAGGAACATAGATAGTATTCATTTATTATCGGTTAAAGGTGAACCGGG
AACCTCCTCTGACTATACGTCTTCTTCTAGCATTTTCTTCCCTGACTCGATAACTACGAGGATGATTTCGAATCTGATGCTGCATATTTGGTCTCAT
AACTCGGTAGGTGTATCTTCTGATTGCTTGTTGCAATTCGAGTGGAAGTGCACGTATCGCATTACGAAGAGGAACTGGGATACGAGTGTTATCA
GTGAAATAATTAGCGGGACGTCCATTAACCATTATTTTATTAGGTGAACTCAGTTATAACAAGTCTACGCATTAAGGCTGCAACAGTCATTGAAT
CAGCTTCGGGATACCAGCGTTGGGGATCCAAATTAGAAGTGATCCATATCTTTGTAGCTACAAGAGGTTTTGAGGATCCTTTGATCTCCACACGG
ACCGGATAACGATCAAGCCATCTGAGGAGATGACTGATGTCAATTCCTCCTCGAAATTCATCCATAACCACATTTGTCTGAGTTTGATAGCCGCA
CCAAAATTTTGTCCGTGGATCTTTACAATAAGAGTCCATTCCGGCTTCTTCCCAGGCTCGGCGAGATTTACCAGTTCCAGTAACTCCCCAGAATAC
AAGGCAAGTACGCTCCATAGGTCGGGGCAAGTCGTGATCGGCTGCAATTGCTCGTATTGTCCGGTAAGAGACCACTCGAACATTAGCTGGGATT
CTTTCAAGTTCGTTTGTCTTGGCAGCACTCCATACATCTTCCCAATCCACAGGTTGGTTTCTTCTAAAAGGTTTGGCGCCAAATTCAAATTGTGTGT
CTGCAACCCGAGATTCTTCTTTCCAAACATAGTCGGAGGCTGCTTCTGAACGGGATAGTTCGGCGTGGGCGGTATTACCAAAGATGGATTTGACT
CCGGCCAGAGAACTCTTCTTACTGAATGCTGCGAAAACTTGCCAGTGGTGGTATCCTCCATCTCCAATTTCCTGTTGGCCTCGGCACCAAGCAAGT
CCAGGAGCGAGAGTCGTTGCCCAGCTCGGTCTTTCATCGACAAAGGGGATGGTGAGGAGCCAGAAGATTCCCTGCCTACGTGACCCACTACGG
GGGAGAGGATTGGGGAGGATGGTTCTTGAGGACTGGATGGAGGACATAACATAATAAAGCTTGATTTGAC

>000081F|arrow

TAGGGGTATCGATGCTTCGAAAGAAATTTTTGATAAACTCAAAAATAATCCTAATCAACTAACCCCTTGGGGAATTGGAGTCAAATAATGAGTAA
AGCGAATTTGCCATTTGTACGTAATCCGTACAACTATGATAAAGATGAAGCATCGGTAAACGATGCGTTGCTGTGTCAAGACCCAAGTCTTGCTC
AACAGCATATGAAAGATGAATGTGACATTAATGTCATCATTGAACGTTTCGGGGTTACAGGGGAACTTCCAACGGCCCCTGTATCGCCTCAATAC
GGCGATTTTAGTGGTGTTACTGATTACCATTCTGCGTTGAATCAAATTAACGCAACTATGGACGATTTCATGGCTCTGCCAGCGAAATTAAGAGT
CCGATTTGACCATGATCCTGTCAAATTATTGGAGTTCCTTGAGAACGACCAGAATCGTGATGAAGCGATTCAATTGGGTCTTATTGATGGACAAC
CTGTGGTTGAACCCATCGTTTCTACAGAAACACCTAAGGCCGAAGGATGAAATCCTGAGGCCAGCACAGTTACTCTACTTGATGTAACTGTGCTA
GGTGACACCAAACCACTATTTTAACTACGGAGTTCATCATGTTACGAAGAAAGCCAGTAAACAAATATAAATCTGCAAAGTCATTTCGCAGAACT
GCTAGTAAGACGAAGTCAATTAATATGAGACACGCTCCCCAGCGTGGTGGCTATCGTTTGTAATTATGGCCTGTTATAAGCCCTTAACGGCTTAT
CAATGCAGTGACAGGTCTATAATTTGGCGGGAAATACCGGGTGCGGATGTAGTCCGTACCCTATCATTGCCTTGTGGTCAGTGTGTTGGTTGTC
GCCTTGAACGCTCACGTCAGTGGGCGATTCGTTGTATGCATGAGGCACAAATGCATACTAGTAATTGTTTTATTACTTTGACATATGCTCCAGAGC
ATTGTCCTAAGGATATGTCATTGGATTACAATGATTATCAGCTTTTTATGAAGCGGTTACGTAAGCGTTTTACTGGGAAAACGATACGTTTTTATA
TGGCAGGTGAATATGGTGAATCTTTTGATCGTCCTCATTTCCATGCTTGTCTGTTTGGTCTTGATTTTCCGGATAAGAAAATATTTAAAAGAACGC
AGACTGGCTCTATCCTCTACACGTCAGAGATTTTGGAAGAATTGTGGCCGTTTGGCTATTCTACAATTGGTGATGTTACTTTGAGTCTGCTGCTTA
TGTTGCAAGATATATTATGAAGAAGATTAATGGGGTTACTGTCAATGAAAACCACGAAGTGGTTGATGCGGGTGCCCATTATCAATATTGTGATT
TAGAGACTGGTGAGATAATTCAGCGTAAGCCAGAATTTAATAAGATGTCATTGAAGCCCGGTATCGGGCAATCGTGGTTAGATAAGTACATGTC
AGACGTTTATACGTCAGACCACGTTGTGGTGCGTGGCAAAAAGTGCCGTCCACCACGGTTTTATGATAATAAATTTAAGTTGAAGTTTCCTGAAG
AATTCGATATGATTCAGTTTGCCAGAGAGATGGAAGGTCGATCTAGGCATGAGGACAACACGCTTGAGCGACTTGCTGTTAAGGAAAAAGTTGC
GTTGGCTAAGTTGTCATTGTTAAAACGTACTATTTAAGGAGTTTTTATGAAGATGGTTATTGTTTCTATTAAGGATACTGCTGCAGATGCTTTTGG
TCGTCCAGCTTATGTTGCATCTGAAGGTGTTGCAGTACGTCAGTTTCAGGATGAAGTCAATCGAGCTAGCGAAGATAATCAGTTGTATAAACATC
CTGATGATTTTCATATGTTCTATTTGGGTCTTTTTGACGATGCCACTGGTGTTTTTGAACTACTGGAAAGCCCTAAGTTGATTGCTCGTGCAAAAG
ATGTAATGATTCGCGAAGGCGAGTAAGGTTTTTTTATACCGTATCACTCGAAAGAGTGGTACGGAACTACGGGAGATGTTTATGTTTCGCAATAA
GTCAGTAAGTACGCATTCATTTGCTATGGTTCCTAAAGCGGACATTCCCCGCTCTAGTTTTAATACTCAATATGCTCATAAAACCACGTTTGATGCT
GGTTTTTTAGTTCCTATTTATTGTGATGAAGTATTGCCTGGCGATACTCATCGTGTAAAGATGACTGCATTTGCACGTTTGGCCACACCGTTATTTC
CTGTGATGGACAACTTGCATCTTGATACTTTCTTTTTCTTTGTACCTAATCGTTTACTTTGGAACAATTGGCCAAAGTTTATGGGTGAACAAACGAA
TCCTGGTGATTCTATTTCTTTTGTAGTGCCTACTATTACTAGTCCTGCTGGTGGTTATGCTGTTTGTTCAATTTTTGATTATTTTGGTTTACCTACTGC
TGGTCAGATTACTGGCGCTAATACAGTAACGCATAATGTTTTGCCGTTACGTGCTTATAATGAGATTTATAACGAATGGTTTAGAGATGAAAACT

TACAGAATTCTGTAACGTTAAATCTTGGTGATTCAGGTGATGTTCCTGCTAACTATACACTTTTGAGACGTGGTAAGCGTAAAGATTATTTTACTG
GTGCATTGCCTTGGCCACAGAAGGGTGCTTCTGTTTCTTTACCGTTAGGAACACGTGCTAATATTTATTCTGACATACCAGCTGGCAATGGTACTG
CTGGTTATAGTGTTTTTCAAACTGCTGTTGGTGCTTTAAGAGAATTAAATTCAGCTTCTAATACTTTGTCTAATAGTACAAATGCTGGTGTTGCTAC
TAATCAGTTATACGCTGATTTGTCTACTGCTACTGCTGCGACTATTAACCAACTTCGTCAATCTTTCCAGATTCAGAAGTTATTGGAGCGCGATGC
ACGTGGTGGTACTCGTTATACTGAGTTACTACGTGCTCACTTTGGAGTAACTCCACAGGATTATCGTTTACAACGTCCTGAATATATTGGTGGAG
GTTCGACCCTTGTTAATGTTAATCCGATTGCTCAGACTTCTGCAACGTCGGTTACTGGTTCTGCTACTCCGCAAGGTAACCTTGCTGCAATGGGTA
CTGCATTGGCTCAGGGACACGGCTTTACGTATGCTGCTCAAGAACATGGATACATTATCGGATTAGTTTCTGTACGTGCTGACCTCACATATCAAC
AGGGTCTTCCTAAGATGTGGTCTAGGTCTACACGATATGACTTTTATTTCCCAGTATTTGCCACTTTGGGTGAGCAAGCTATTTTGAACAAAGAAA
TTTATGTTCAAGGTACTGCAGCCGACAATGATGTATTTGGTTATCAAGAACGTTGGGCGGAGTATCGTTACAAACCTTCTCAAATTACTGGTTTCT
TTAGGTCTACTTCTGCTGGCACTATTGATGCTTGGCATTATGGACAGCGATTTACTTCTCTTCCTACGTTGAATTCAACGTTTATTCAAGAGACCCC
TCCAGTTGCTCGTACTACGGCGGTCGGAGCTGCAGCAAATGGTCAGCAATTTTTAATGGATGCTTTCTTTGATTGTCAGATGGCCAGACCTATGC
CTATGTACAGCGTACCTGGTCTAATTGATCATTTCTAATGTTTTATATAACCTCGACTACTCCGTAAGGAGTAGTGAGGAAACAACCGAAGGGCG
TTAGTTTATGTTTGGTGGAATACTTGATGCGGTTACTAATGTTGGTTCTAAGCTGTCTTCAGCTTCTAGTTTCTTTACTCCTGGTGTCGGTACTGCT
TTGGGCGCTGTTGGTTCTTATTTAGGTTCTACTTCTGCTAATAAAGCTAATCAGGAGATGGCTCAGAGGCAAATGGATTTTCAAGCCGATATGAG
TGGAACAAGTTACCAGCGTGCTGTTAAAGATTTAGAAGCTGCTGGTTTATCTCCTATGTTAGCCTATCAACGTGGTGGTGCTTCTACCCCATCTGG
TTCAACTGCTACTATGGAAAATGTTTTAGGTAATGCAACTAATTCAGCTATTAATACTGCTTCTATGATGCAACAGATTCGTAATGCATCAGAAAC
AGAAAAGCAGATTATCGCCCAGACTGAAGCTACTGAAGCTGGTACCGCTAATACTAGGGCTGATACTGTTAATAAGTTGCTTACTGCTCCTAATA
TTACAGCCGAAAATAAACGTATTTTGGCTGATATTGCTTTAAAGAATACGACTGCGGATTTAACATCCGCTCAGTCATATAATACTAAGAGGCTAT
TGGCTCCATCCCCAGCTATTTGGTC

>000071F|arrow

ATGCCTGTATCTTCGGATACGACTTTCCTGATAAAAAATTATGGAAAAGGACTGCCTCTGGTTCTATGTTATATAGATCCGCAGAACTTGAAGCTC
TCTGGCCATTTGGTTATACCACCATTGGAGATGTTACTTTCGAATCAGCCGCCTACGTGGCTAGATACATAATGAAAAAACAAACAGGGAAAGAT
GCGGAATCTCATTACAAACGCATACACCCTGAAACCGGCGAATATTTAGACTTAAAGCCGGAATATAATAAAATGTCTTTAAAACCGGGAATCG
GTAAAGACTTTTATATAAAATATACTTCGGATATATACCCGCAAGACTACGTAATACTTAGAGGTAAAAAGGTCAAACCACCAAAATACTATGAC
AAAATGTTTAAAATTGACCAACCTTATGAGTATGACGAATTACTTTACATGCGGGAAAATAATGCTAAACTTAATTCCGAAGACAATACACCAGA
ACGACTATCTGCAAAAGAACAAGTCACTATGGCAAAACTTCAACTATTAAAACGTAACCTTACTTAGGAAAATAATGAAACTTATCCTCGCTTCCG
TAAAAGACCGTGCTGCTGAAGCATATGCACGACCAATGTTCGTACCTTCCTCTGGAGTAGCTATACGCTCTTTTTCAGATGAAATTAATCGTTCTG
ATACTGAAAATCAACTCTTTAATCACCCTGATGACTTCGATCTATATGAATTCGGAACATTTGACGATTCAACTGGGTTATTCGATTTACATGAAC
AACCAAAACTCCTATCATTAGGAAAACAAGTTAAACTTAAATAAAACAACCGAGGGGAAAAGAGATTTATCTTTCCCCCGGAACAACACTAAGG
AAAAACATGCACCGCAATCAGTCAGTTAATACTCACCGCTTCGCGATGGTACCTAGAGCCGATATACCACGTAGTAAATTCGATGCTCAAAAAAC
ACATAAAACGACTTTCGATGCGGGCTATCTAATTCCTGTATATGTTGATGAAGTGCTCCCTGGGGACACTTTCAACTTAAAAATGACGGCATTTGC
CCGTCTAGCAACGCCTTTATATCCAATCATGGATAACATGATTATGGATTCTTTCTTTTTCTTTGTACCCAATCGCCTTATATGGAATAACTGGCAA
AAATTTATGGGTCAACAAGAAAATCCAACAGACTCAATATCTTATATTGTCCCAACACAAACAGCCCAACAGATGGTTATGCCGTAGGCAGCCTT
CAAGACTATATGGGCTTACCAACAGTAGGCCAAATTGATACTGGCCGAACTATTACGCACTGTGCCTTTTGGCCACGTGCATACAATCTTATCTG
GAACGAATGGTTCCGAGATGAAAATTTACAAACAAGCGCAGTAGTTGATAAGGGCGATGGCCCTGATACTTCCTCAAACTATGTGCTAAAACGT
CGTGGTAAAAGACATGATTACTTTACGTCAGCATTACCATGGCCACAAAAAGGTGCGAGTGTCACCTTACCTTTAGGTACTACGGCTCCAATTAA
ATGGGATACCATTTCAGGAGACGCAACATCAAACGATAAATTTACGGTAATTCAAACAGATCCTGGAAATACGACTGCTTTAGCTAGATATGGCA
ACGCTTATGGTGTTAATACTGCTGGTGTAGTAAATAACGTTTCTAATTTATATACCGACTTATCAGAAGCAACTGCTGCAACTGTCAATCAATTAA
GACAGTCATTTCAAATTCAAAAATTACTTGAAAGGGATGCACGTGGCGGAACACGATACACAGAAATTATCCGGAGTCACTTTGGAGTTATTTCC
CCAGACGCCCGTTTACAAAGGCCTGAATACCTTGGAGGCGGTTCAACACCAATTAATGTTAATCCGATTGCTCAAACGTCGGGAACAAACGCTTC
TGGAACGACTACCCCTTTGGGCAACCTTGCTGCTATGGGTACTGCTCTCGCTCATAATCATGGATTTACTCAATCATTTACTGAGCATGGCGTTAT
TATTGGATTAGTATCCATTAGAGCAGATCTTACTTATCAACAAGGATTAGACCGTATGTGGTCTAGATCTACACGATATGACTTTTATTTCCCAGC
ATTTGCTACTCTAGGCGAACAATCTGTTTTGCAAAAAGAAATTTATGCAACAGGAGATACTGCAGCCGACAATACTGTTTTTGGATATCAAGAAC
GCTGGGCGGAATATCGTTACAAACCATCTAAAATTACTGGTTTGTTCAAATCAACATCGGCGGGCACGATCGATGGTTGGCATTTGGCTCAAAAA
TTTACCGCTGCGCCTACTTTGAATAATACGTTTATTCAAGATACGCCTCCTGTATCACGTGTAGTAGCCGTTGGAGCAGCTGCAAATGGCCAACAA
TTCTTATTTGACTCATTTTTTGATGTCAAAATGGCAAGACCAATGCCAATGTATTCAGTACCTGGCTTAATAGACCATTTCTAATGGGACTATTTGA
CGGAATTGCCGATTTAATCGGCCCTGCTATAGCTATAGGAGCTGCCCCTGCTACTGGGGGACTCTCCTTAGCTGCACTTGCACCTGCAGCAATAG
GTGCAGCAGGACAATACTTTGGAACACAAAGTCAAAACGCAGCGAGTGCAGAACAAGCGAGTAATCAACAGAGATTTCAAGCTGAAATGTCTG
GAACATCATATCAACGAGCAGTTGAAGATATGAAAAAAGCTGGGTTAAATCCCATGCTTGCGTATTCACAAGGCGGAGCCACAACACCAGCTGG
AGCTATGGCCCAGATGCAAAATGTTCTCGGTAATGCAACTACGTCCGGAACCCAAGCTTATCAAACGGTTGCGCAAGCAAATCAAGCTATTGCTC
AATCTAAACAAATTGAAGCTCAAACAGAACTCACAAGTAATCAAACAGATAATGTACGTGCTGATACGTTAAATAAATTGGATGAAAATCCAAAT

ATTAGAGCTCAATATAAACAAATACTTGCCGATACTTTCATGAAAAATGAAATAGGCAAAACATCAAGTGCTCAAGCTGCTCAAGCTTTGGCACA
ATCTCGTTATTCAAACGAGTTAACAAAACTTGCTAAATCAGGGTCAGCTCCTAGTTCTAGCAAACCAATTTATCAAGACGTAAAAAACATCGCCAA
AGATGCGTATAGCGCATCTGGCGCAAAACGATACATCGATAACTATCGAGGTCAACCGATTCAACAAAATCGTACAAATAACCAACCACCAATG
GAATGAAAATGACAAAGATTACAGCCCCATTTCTTCGTACTCCGTACAATTACGACACGATTGCTGCGTCAAATGAGTCAGGGCTGCATTGTGAG
GATGCAACTCTGACTCAGCAGCAATTTGCTGAAGAATGTGATATCAATAATATTATGGAAAAGTTCGGTATGACCGGACTTATTCCTCAAACTCC
TTTAACGCCTCAATATGGCGACTTTAGTGGTGTCTATGACTACCACTCTGCTCTGAACCAGATTATGGCTTCAGACAACGAATTTATGGCTTTACC
AGCCAATATTCGTGAACGATTCGCTAATGATCCCGCGAATCTAATAGATTTTCTAGAAAACCCTGAAAATCGCAGCGAAGCTGAAAAAATGGGA
CTGGTAAAACCAGCCCAAACCGAGGTTTCAACCCCTGTTGGAACCTCGGAAGCACAGTTACCTACTTGATGTAACTGTGCTAGGTGACACCAAAC
CACAAAAACACGATAAACAAGGACAGAAAAAATGATGCGTCGCAGACCAGCAAATAAGCAAAAGTCCGCTAGGACTTTCCGTAAACATGCTTCA
CATACAAAACACGCAAATATGCGAAACTCGCCAATGCGTGGAGGCTGGAGACTCTAATAAAGTCTTCAGGCACCTCACATGCCTTGTTATCACCC
TCTCAAAGCATATCAATGCTTTGACAAATCAATTGTTTTCGACGAAGTTCGGAAACATGACATCGTTCGATCTTTAGACCTGCCCTGTGGGCAGTG
CGTTGGATGCCGTCTAGAACGATCAAGACAATGGGCTATTCGGTGCATGCACGAAGCCCAATTGCATAAAAACAACTCATTCATAACACTCACAT
ATGACAATACACATCTCCCAAGCGATGGCTCTTTGGATCACAAAGACTTTCAATTGTTCCTTAAAAGACTTAGAAAAACTCTCGCAAAAAGAGGA
CTTACAATCCGCTATTACATGGCTGGAGAATATGGTGAACTCTTCGCAAGACCCCACTTCC

>000172F|arrow

CGTGGCTAGATACATAATGAAAAAACAAACAGGGAAAGATGCGGAATCTCATTACAAACGCATACACCCTGAAACCGGCGAATATTTAGACTTA
AAGCCGGAATATAATAAAATGTCTTTAAAACCGGGAATCGGTAAAGACTTTTATATAAAATATACTTCGGATATATACCCGCAAGACTACGTAAT
ACTTAGAGGTAAAAAGGTCAAACCACCAAAATCTATGACAAAATGTTTAAAATTGACCAACCTTATGAGTATGACGAATTACTTTACATGCGGGA
AAATAATGCTAAATTTAATTCCGAAGACAATACACCAGAACGACTATCTGCAAAAGAACAAGTCACTATGGCAAAACTTCAACTATTAAAACGTA
ACCTTACTTAGGAAATAATGAAACTTATCCTCGCTTCCGTAAAAGACCGTGCTGCTGAAGCATATGCACGACCAATGTTCGTACCTTCCTCTGGAG
TAGCTATACGCTCTTTTTCAGATGAAATTAATCGTTCTGATACTGAAATCAACTCTTTAATCACCCTGATGATTTCGATCTATATGAATTCGGAACA
TTTGACGATTCAACTGGGTTATTCGATTTACATGAACAACCAAAACTCCTATCATTAGGAAAACAAGTTAAACTTAAATAAAACAACCGAGGGGA
AAAGAGATTTATCTTTCCCCCGGAACAACACTAAGGAAAAACATGCACCGCAATCAGTCAGTTAATACTCACCGCTTCGCGATGGTACCTAGAGC
CGATATACCACGTAGTAAATTCGATGCTCAAAAACACATAAAACGACTTTCGATGCGGGCTATCTAATTCCTGTATATGTTGATGAAGTGCTCCCT
GGGGACACTTTCAACTTAAAAATGACGGCATTTGCCCGTCTAGCAACGCCTTTATATCCAATCATGGATAACATGATTATGGATTCTTTCTTTTTCT
TTGTACCCAATCGCCTTATATGGAATAACTGGCAAAAATTTATGGGTCAACAAGAAAATCCAACAGACTCAATATCTTATATTGTCCCAACACAAA
CAAGCCCAACAGATGGTTATGCCGTAGGCAGCCTTCAAGACTATATGGGCTTACCAACAGTAGGCCAAATTGATACTGGCCGAACTATTACGCA
CTGTGCCTTTTGGCCACGTGCATACAATCTTATCTGGAACGAATGGTTCCGAGATGAAAATTTACAAACAAGCGCAGTAGTTGATAAGGGCGAT
GGCCCTGATACTTCCTCAAACTATGTGCTAAAACGTCGTGGTAAAAGACATGATTACTTTACGTCAGCATTACCATGGCCACAAAAAGGTGCGAG
TGTCACCTTACCTTTAGGTACTACGGCTCCAATTAAATGGGATACCATTTCAGGAGACGCAACATCAAACGATAAATTTACGGTAATTCAAACAG
ATCCTGGAAATACGACTGCTTTAGCTAGATATGGCAACGCTTATGGTGTTAATACTGCTGGTGTAGTAAATAACGTTTCTAATTTATATACCGACT
TATCAGAAGCAACTGCTGCAACTGTCAATCAATTAAGACAGTCATTTCAAATTCAAAAATTACTTGAAAGGGATGCACGTGGCGGAACACGATAC
ACAGAAATTATCCGGAGTCACTTTGGAGTTATTTCCCCAGACGCCCGTTTACAAAGGCCTGAATACCTTGGAGGCGGTTCAACACCAATTAATGT
TAATCCGATTGCTCAAACGTCGGGAACAAACGCTTCTGGAACGACTACCCCTTTGGGCAACCTTGCTGCTATGGGTACTGCTCTCGCTCATAATCA
TGGATTTACTCAATCATTTACTGAGCATGGCGTTATTATTGGATTAGTATCCATTAGAGCAGATCTTACTTATCAACAAGGATTAGACCGTATGTG
GTCTAGATCTACACGATATGACTTTTATTTCCCAGCATTTGCTACTCTAGGCGAACAATCTGTTTTGCAAAAAAATTTATGCAACAGGAGATACTG
CAGCCGACAATACTGTTTTTGGATATCAAGAACGCTGGGCGGAATATCGTTACAAACCATCTAAAATTACTGGTTTGTTCAAATCAACATCGGCG
GGCACGATCGATGGTTGGCATTTGGCTCAAAAATTTACCGCTGCGCCTACTTTGAATAATACGTTTATTCAAGATACGCCTCCTGTATCACGTGTA
GTAGCCGTTGGAGCAGCTGCAAATGGCCAACAATTCTTATTTGACTCATTTTTTGATGTCAAAATGGCAAGACCAATGCCAATGTATTCAGTACCT
GGCTTAATAGACCATTTCTAATGGGACTATTTGACGGAATTGCCGATTTAATCGGCCCTGCTATAGCTATAGGAGCTGCCCCTGCTACTGGGGGA
CTCTCCTTAGCTGCACTTGCACCTGCAGCAATAGGTGCAGCAGGACAATACTTTGGAACACAAAGTCAAAACGCAGCGAGTGCAGAACAAGCGA
GTAATCAACAGAGATTTCAAGCTGAAATGTCTGGAACATCATATCAACGAGCAGTTGAAGATATGAAAAAGCTGGGTTAAATCCCATGCTTGCG
TATTCACAAGGCGGAGCCACAACACCAGCTGGAGCTATGGCCCAGATGCAAAATGTTCTCGGTAATGCAACTACGTCCGGAACCCAAGCTTATC
AAACGGTTGCGCAAGCAAATCAAGCTATTGCTCAATCTAAACAAATTGAAGCTCAAACAGAACTCACAAGTAATCAAACAGATAATGTACGTGCT
GATACGTTAAATAAATTGGATGAAAATCCAAATATTAGAGCTCAATATAAACAAATACTTGCCGATACTTTCATGAAAATGAAATAGGCAAAACA
TCAAGTGCTCAAGCTGCTCAAGCTTTGGCACAATCTCGTTATTCAAACGAGTTAACAAAACTTGCTAAATCAGGGTCAGCTCCTAGTTCTAGCAAA
CCAATTTATCAAGACGTAAAAAACATCGCCAAAGATGCGTATAGCGCATCTGGCGCAAAACGATACATCGATAACTATCGAGGTCAACCGATTC
AACAAAATCGTACAAATAACCAACCACCAATGGAATGAAAATGACAAAGATTACAGCCCCATTTCTTCGTACTCCGTACAATTACGACACGATTG
CTGCGTCAAATGAGTCAGGGCTGCATTGTGATGCAACTCTGACTCAGCAGCAATTTGCTGAAGAATGTGATATCAATAATATTATGGAAAAGTTC
GGTATGACCGGACTTATTCCTCAAACTCCTTTAACGCCTCAATATGGCGACTTTAGTGGTGTCTATGACTACCACTCTGCTCTGAACCAGATTATG
GCTTCAGACAACGAATTTATGGCTTTACCAGCCAATATTCGTGAACGATTCGCTAATGATCCCGCGAATCTAATAGATTTTCTAGAAAACCCTGAA

AATCGCAGCGAAGCTGAAAAAATGGGACTGGTAAAACCAGCCCAAACCGAGGTTTCAACCCCTGTTGGAACCTCGGAAGCACAGTTACCTACTT
GATGTAACTGTGCTAGGTGACACCAAACCACAAAAACACGATAAACAAGGACAGAAAAAATGATGCGTCGCAGACCAGCAAATAACAAAAGTC
CGCTAGGACTTTCCGTAAACATGCTTCACATACAAAACCGCAAATATGCGAAACTCGCCAATGCGTGGAGGCTGGAGACTCTAATAAAGTCTTCA
GGCACCTCACATGCCTTGTTATCACCCTCTCAAAGCATATCAATGCTTTGACAAATCAATTGTTTTCGACGAAGTTCGGAAACATGACATCGTTCG
ATCTTTAGACCTGCCCTGTGGGCAGTGCGTTGGATGCCGTCTAGAACGATCAAGACAATGGGCTATTCGGTGCATGCACGAAGCCCAATTGCAT
AAAAACAACTCATTCATAACACTCACATATGACAATACACATCTCCCAAGCGATGGCTCTTTGGATCACAAAGACTTTCAATTGTTCCTTAAAAGA
CTTAGAAAAACTCTCGCAAAAAGAGGACTTACAATCCGCTATTACATGGCTGGAGAATATGGTGAACTCTTCGCAAGACCCCACTTCCATGCCTG
TATCTTCGGATACGACTTTCCTGATAAAAAATTATGGAAAAGGACTGCCTCTGGTTCTATGTTATATAGATCCGCAGAACTTGAAGCTCTCTGGCC
ATTTGGTTATACCACCATTGGAGATGTTACTTTGAATCAGCCGCCTA

>000096F|arrow

CTGCTACTGGGGGACTCTCCTTAGCTGCACTTGCACCTGCAGCAATAGGTGCAAGCAGGACAATACTTTGGAACACAAAGTCAAAACGCAGCGA
GTGCAGAACAAGCGAGTAATCAACAGAGATTTCAAGCTGAAATGGTCTGGAACTCATATCAACGAGCAGTTGAAGATATGAAAAAAGCTGGGT
TAAATACCCCATTGCTTGCGTATTCACAAGGGCGGAGCCACAACCCAGCTGGAGCTATGGGCCCAGATTGCCAAAATGTTCTCAGTAGATGCCA
ACTACGTCCGGAACCCAAGAAGCTTATCAAACGGTTGCGCAAGCAATCAGCTATTGCTCAATCTAAACAAATTGAAGCTCAAACAGAACTCACAA
GTAATCAAACAGATAATGTACGTCTGATACTGTTAATAATTGGATGAAAATCCAAAATATTAGAGCTCAGATATACACAAAGACTTGCCGATACT
TTCATGAAAAAATGAATAGGCAAAAACATCAAGTGCTCAAGCGCTCAAGCGTTGGCACAATCTCGTTATTCAAACGAGTTAACAAAACTTGCTAA
ATCAGGGTCAGCTCCTAGTTCTAGCAAACCAATTTATCAAGACGTAAAAAACATCGCCAAAGATGCGTATAGCGCATCTGGCGCAAAACGATAC
ATCGATAACTATCGAGGTCAACCGATTCAACAAAATCGTACAAATAACCAACCACCAATGGAATGAAAATGACAAGATTACAGCCCCATTTCTTC
GTACTCCGTACAATTACGACACGATTGCTGCGTCAAATGAGTCAGGGCTGCATTGTGAGGATGCAACTCTGACTCAGCAGCAATTTGCTGAAGA
ATGTGATATCATAATATTATGGAAAAGTTCGGTATGACCGACTTATTCCTCAAACTCCTTTAACGCCTCAATATGGCGACTTTAGTGGTGTCTATG
ACTACCACTCTGCTCTGAACCAGATTATGGCTTCAGACAACGAATTTATGGCTTTACCAGCCAATATTCGTGAACGATTCGCTAATGATCCCGCGA
ATCTAATAGATTTTCTAGAAAACCCTGAAAATCGCAGCGAAGCTGAAAAAATGGGACTGGTAAAAACCAGCCCAAACCGAGGTTTCAACCCCTG
TTGGAACCTCGGAAGCACAGTTACCTACTTGATGTAACTGTGCTAGGTGACACCAAACCACAAAAACACGATAAACAAGGACAGAAAAAATGAT
GCGTCGCAGACCAGCAAATAAGCAAAAGTCCGCTAGGACTTCCGTAAACATGCTTCACATACAAAACACGCAACATATGCGAAACTCGCCAATG
CGTGGAGGCTGGAGACTCTAATAAAGTCTTCAGGCACCTCACATGCCTTGTTATCACCCTCTCAAAGCATATCAATGCTTTGACAAATCAATTGTT
TTCGACGAAGTTCGGAAACATGACATCGTTCGATCTTTAGACCTGCCCTGTGGGCAGTGCGTTGGATGCCGTCTAGAACGATCAAGACAATGGG
CTATTCGGTGCATGCACGAAGCCCAATTGCATAAAAACAACTCATTCATAACACTCACATATGACAATACACATCTCCCAAGCGATGGCTCTTTGG
ATCACAAAGACTTTCAATTGTTCCTTAAAAGACTTAGAAAAACTCTCGCAAAAAGAGGACTTACAATCCGCTATTACATGGCTGGAGAATATGG
TGAAACTCATTCGCAAGACCCCACTTCCATGCCTGTATCTTCGGATACGACTTTCCTGATAAAAAATTATGGAAAAGGACTGCCTCTGGTTCTATG
TTATATAGATCCGCAGAACTTGTAAGCTCTCTGGCCATTTGGTTATACCACCATTGGAGATGTTACTTTTGAATCAGCCGCCTACGTGGCTAGATA
CATAATGAAAAAACAAACAGGGAAAGATGCGGAATCTCATTACAAACGCATACACCCTGAAACCGCGAATATTTAGACTTAAAGCCGGAATATA
ATAAAATGTCTTTAAAACCGGGGAATCGGTAAAGACTTTTATATAAATATACTTCGGATATATACCCGCAAGACTACGTAATACTTAGAGGTAAA
AGGTCAAACCACCAAAAATACTATGACAAAATGTTTAAAATTGACCAACCTTATGAGTATGACGAATTACGTTACATGCGGGAAAATAATGCTAA
ATTTAATTCCGAAGACAATACACCAGAACGACTATCTGCAAAAGAACAAGTCACTATGGCAAAACTTCAACTATTAAAAACGTAACCTTACTTAG
GAAAATAATGAAACTTATCCTCGCTTCCGTAAAAGACCGTGCTGCTGAAGCATATGCACGACCAATGTTCGTACCTTCCTCTGGAGTAGCTATAC
GCTTCTTTTCAGATGAATTAATCGTTCTGATACTGAAAATCAACTCTTTAATCACCCTGATGATTTCGATCTATATGAATTCGGAACATTTGACGAT
TCAACTGGGTTATTCGATTTACATGAACAACCAAAACTCCTATCATTAGGAAAACAAGTTAAACTTAAATAAAACAACCGAGGGGAAAAGAGATT
TATCTTTCCCCCGGAACAACACTAAGGAAAAACATGCACCGCAATCAGTCAGTTAATACTCACCGCTTCGCGATGGTACCTAGAGCCGATATACC
CGTAGTAAATTCGATGCTCAAAAAACACATAAAACGACTTTCGATGCGGGCTATCTAATTCCTGTATATGTTGATGAAGTGCTCCTGGGACACTTT
CAACTTAAAAATGACGGCATTTGCCCGTCTAGCAACGCCTTATATCCAATCATGGATAACATGATTATGGATTCTTTCTTTTTCTTTGTACCCAATC
GCCTTATATGGAATAACTGGCAAAAATTTATGGGTCAACAAGAAAATCCAACAGACTCAATATCTTATATTGTCCCAACACAAACAAGCCCAACA
GATGGTTATGCCGTAGGCAGCCTTCAAGACTATATGGGCTTACCAACAGTAGGCCAAATTGATACTGGCCGAACTATTACGCACTGTGCCTTTTG
GCCACGTGCATACAATCTTATCTGAACGAATGGTTCCGAGATGAAAATTTACAAACCAGCGCAGTAGTTGATAAGGGCGATGGCCCTGATACTT
CCTCAAACTATGTGCTAAAACGTCGTGGTAAAAGACATGATTACTTTACGTCAGCATTACCATGGCCACAAAAGGTGGCGAGTGTCACCTTACCT
TTAGGTACTACGGCTCCAATTAAATGGGATACCATTTCAGGAGACGCAACATCAAACGATAAATTTACGGTAATTCAAACAGATCCTGGAAATAC
GACGGCTTTAGCTAGATATGGCAACGCTTATTGGTGTTAATACCTGCTGGTGTAGTAAATAACGTTTCTAATTTATATACCGACTTATCAGAAGCA
ACTGCTGCAACTGTCAATCAATTAAGACAGTCATTTCAAATTCAAAAATTACTTGAAAGGGATGCACGTGGCGGAACACGATACACAGAAATTAT
CCGGAGTCACTTTGGAGTTATTTCCCCAGACGCCCGTTTACAAAGGCCTGAATACCTTGGAGGCGGTTCAACACCAATTAATGTGTAATCCGATT
GCTCAAACGTCGGGAACAAACGCTTCTGGAACGACTACCCCTTTGGGCAACCTTGCTGCTATGGGTACTGCTCTCGCTCATAATCATGGATTTACT
CAATCATTTACTGAGCATGGCGTTATTATTGGATTAGTATCCATTAGAGCAGATCTTACTTATCAACAAGGATTAGACCGTATGTGGTCTAGATCT
ACACGATATGACTTTTATTTCCCAGCATTTGCTACTCTAGGCGAACAATCTGTTTTGCAAAAAGAAATTGATGCAACAGGAGATACTGCAAGCCG

ACAATACTGTTTTTGGATATCAAGAACGCTGGGCGGAATATCGTTACAAACCATCTAAAATTACTGTTTGTTCAAATCAACATCGGCGGGCACGA
TCGATGGTTGGCATTTGGCTCAAAAATTTACCGCTGCGCCTACTTTGAATAATACGTTTATTCAAGATACGCCTCCTGTATCACGTGTAGTAGCCG
TTGGAGCAGCTGCAAATGGCCAACAATTCTTATTTGACTCATTTTTGATGTCAAAATGGCAAGACCAATGCCAATGTATTCAGTACCTGGCTTAAT
AGACCATTTCTAATGGGACTATTTGACGGAATTGCCGATTTAATCGGCCCTGCTATAGCTATAGGAGGCTGCCC

>000038F|arrow

CCAGAATCGTGATGAAGCGATTCAATTGGGTCTTATTGATGGACAACCTGTGGTTGAACCATCGTTTCTACAGAAACACCTAAGGCCGAAGGAT
GAAATCCTGAGGCCAGCACAGTTACTCTACTTGATGTAACTGTGCTAGGTGACACCAAACCACTATTTTAACTACGGAGTTCATCATGTTACGAA
GAAAGCCAGTAAACAAATATAAATCTGCAAAGTCATTTCGCAGAACTGCTAGTAAGACGAAGTCAATTAATATGAGACACGCTCCCCAGCGTGG
TGGCTATCGTTTGTAATTATGGCCTGTTATAAGCCCTTAACGGCTTATCAATGCAGTGACAGGTCTATAATTTGGCGGGAAATACCGGGTGCGGA
TGTAGTCCGTACCCTATCATTGCCTTGTGGTCAGTGTGTTGGTTGTCGCCTTGAACGCTCACGTCAGTGGGCGATTCGTTGTATGCATGAGGCAC
AAATGCATACTAGTAATTGTTTTATTACTTTGACATATGCTCCAGAGCATTGTCCTAAGGATATGTCATTGGATTACAATGATTATCAGCTTTTTAT
GAAGCGGTTACGTAAGCGTTTTACTGGGAAAACGATACGTTTTTATATGGCAGGTGAATATGGTGAATCTTTTGATCGTCCTCATTTCCATGCTTG
TCTGTTTGGTCTTGATTTTCCGGATAAGAAAATATTTAAAAGAACGCAGACTGGCTCTATCCTCTACACGTCAGAGATTTGGAAGAATTGTGGCC
GTTTGGCTATTCTACAATTGGTGATGTTACTTTTGAGTCTGCTGCTTATGTTGCAAGATATATTATGAAGAAGATTAATGGGGTTACTGTCAATGA
AAACCACGAAGTGGTTGATGCGGGTGCCCATTATCAATATTGTGATTTAGAGACTGGTGAGATAATTCAGCGTAAGCCAGAATTTAATAAGATG
TCATTGAAGCCCGGTATCGGGCAATCGTGGTTAGATAAGTACATGTCAGACGTTTATACGTCAGACCACGTTGTGGTGCGTGGCAAAAAGTGCC
GTCCACCACGGTTTTATGATAATAAATTTAAGTTGAAGTTTCCTGAAGAATTCGATATGATTCAGTTTGCCAGAGAGATGGAAGGTCGATCTAGG
CATGAGGACAACACGCTTGAGCGACTTGCTGTTAAGGAAAAAGTTGCGTTGGCTAAGTTGTCATTGTTAAAACGTACTATTTAAGGAGTTTTTAT
GAAGATGGTTATTGTTTCTATTAAGGATACTGCTGCAGATGCTTTTGGTCGTCCAGCTTATGTTGCATCTGAAGGTGTTGCAGTACGTCAGTTTCA
GGATGAAGTCAATCGAGCTAGCGAAGATAATCAGTTGTATAAACATCCTGATGATTTTCATATGTTCTATTTGGGTCTTTTTGACGATGCCACTGG
TGTTTTTGAACTACTGGAAAGCCCTAAGTTGATTGCTCGTGCAAAAGATGTAATGATTCGCGAAGGCGAGTAAGGTTTTTTTATACCGTATCACT
CGAAAGAGTGGTACGGAACTACGGGAGATGTTTATGTTTCGCAATAAGTCAGTAAGTACGCATTCATTTGCTATGGTTCCTAAAGCGGACATTCC
CCGCTCTAGTTTTAATACTCAATATGCTCATAAAACCACGTTTGATGCTGGTTTTTTAGTTCCTATTTATTGTGATGAAGTATTGCCTGGCGATACT
CATCGTGTAAAGATGACTGCATTTGCACGTTTGGCCACACCGTTATTTCCTGTGATGGACAACTTGCATCTTGATACTTTCTTTTTCTTTGTACCTA
ATCGTTTACTTTGGAACAATTGGCCAAAGTTTATGGGTGAACAAACGAATCCTGGTGATTCTATTTCTTTTGTAGTGCCTACTATTACTAGTCCTGC
TGGTGGTTATGCTGTTTGTTCAATTTTTGATTATTTTGGTTTACCTACTGCTGGTCAGATTACTGGCGCTAATACAGTAACGCATAATGTTTTGCCG
TTACGTGCTTATAATGAGATTTATAACGAATGGTTTAGAGATGAAAACTTACAGAATTCTGTAACGTTAAATCTTGGTGATTCAGGTGATGTTCCT
GCTAACTATACACTTTTGAGACGTGGTAAGCGTAAAGATTATTTTACTGGTGCATTGCCTTGGCCACAGAAGGGTGCTTCTGTTTCTTTACCGTTA
GGAACACGTGCTAATATTTATTCTGACATACCAGCTGGCAATGGTACTGCTGGTTATAGTGTTTTTCAAACTGCTGTTGGTGCTTTAAGAGAATTA
AATTCAGCTTCTAATACTTTGTCTAATAGTACAAATGCTGGTGTTGCTACTAATCAGTTATACGCTGATTTGTCTACTGCTACTGCTGCGACTATTA
ACCAACTTCGTCAATCTTTCCAGATTCAGAAGTTATTGGAGCGCGATGCACGTGGTGGTACTCGTTATACTGAGTTACTACGTGCTCACTTTGGA
GTAACTCCACAGGATTATCGTTTACAACGTCCTGAATATATTGGTGGAGGTTCGACCCTTGTTAATGTTAATCCGATTGCTCAGACTTCTGCAACG
TCGGTTACTGGTTCTGCTACTCCGCAAGGTAACCTTGCTGCAATGGGTACTGCATTGGCTCAGGGACACGGCTTTACGTATGCTGCTCAAGAACA
TGGATACATTATCGGATTAGTTTCTGTACGTGCTGACCTCACATATCAACAGGGTCTTCCTAAGATGTGGTCTAGGTCTACACGATATGACTTTTA
TTTCCCAGTATTTGCCACTTTGGGTGAGCAAGCTATTTTGAACAAAGAAATTTATGTTCAAGGTACTGCAGCCGACAATGATGTATTTGGTTATCA
AGAACGTTGGGCGGAGTATCGTTACAAACCTTCTCAAATTACTGGTTTCTTTAGGTCTACTTCTGCTGGCACTATTGATGCTTGGCATTATGGACA
GCGATTTACTTCTCTTCCTACGTTGAATTCAACGTTTATTCAAGAGACCCCTCCAGTTGCTCGTACTACGGCGGTCGGAGCTGCAGCAAATGGTCA
GCAATTTTTAATGGATGCTTTCTTTGATTGTCAGATGGCCAGACCTATGCCTATGTACAGCGTACCTGGTCTAATTGATCATTTCTAATGTTTTATA
TAACCTCGACTACTCCGTAAGGAGTAGTGAGGAAACAACCGAAGGGCGTTAGTTTATGTTTGGTGGAATACTTGATGCGGTTACTAATGTTGGT
TCTAAGCTGTCTTCAGCTTCTAGTTTCTTTACTCCTGGTGTCGGTACTGCTTTGGGCGCTGTTGGTTCTTATTTAGGTTCTACTTCTGCTAATAAAGC
TAATCAGGAGATGGCTCAGAGGCAAATGGATTTTCAAGCCGATATGAGTGGAACAAGTTACCAGCGTGCTGTTAAAGATTTAGAAGCTGCTGGT
TTATCTCCTATGTTAGCCTATCAACGTGGTGGTGCTTCTACCCCATCTGGTTCAACTGCTACTATGGAAAATGTTTTAGGTAATGCAACTAATTCAG
CTATTAATACTGCTTCTATGATGCAACAGATTCGTAATGCATCAGAAACAGAAAAGCAGATTATCGCCCAGACTGAAGCTACTGAAGCTGGTACC
GCTAATACTAGGGCTGATACTGTTAATAAGTTGCTTACTGCTCCTAATATTACAGCCGAAAATAAACGTATTTTGGCTGATATTGCTTTAAAGAAT
ACGACTGCGGATTTAACATCCGCTCAGTCATATAATACTAAGAGGCTATTGGCTCCATCCCCAGCTATTTGGTCTAGGGGTATCGATGCTTCGAA
AGAAATTTTTGATAAACTCAAAAATAATCCTAATCAACTAACCCCTTGGGGAATTGGAGTCAAATAATGAGTAAAGCGAATTTGCCATTTGTACG
TAATCCGTACAACTATGATAAAGATGAAGCATCGGTAAACGATGCGTTGCTGTGTCAAGACCCAAGTCTTGCTCAACAGCATATGAAAGATGAA
TGTGACATTAATGTCATCATTGAACGTTTCGGGGTTACAGGGGAACTTCCAACGGCCCCTGTATCGCCTCAATACGGCGATTTTAGTGGTGTTAC
TGATTACCATTCTGCGTTGAATCAAATTAACGCAACTATGGACGATTTCATGGCTCTGCCAGCGAAATTAAGAGTCCGATTTGACCATGATCCTGT
CAAATTATTGGAGTTCCTTGAGAACGA

>000018F|arrow

GCCCGGTATCGGGCAATCGTGGTTAGATAAGTACATGTCAGACGTTTATACGTCAGACCACGTTGTGGTGCGTGGCAAAAAGTGCCGTCCACCA
CGGTTTTATGATAATAAATTTAAGTTGAAGTTTCCTGAAGAATTCGATATGATTCAGTTTGCCAGAGAGATGGAAGGTCGATCTAGGCATGAGG
ACAACACGCTTGAGCGACTTGCTGTTAAGGAAAAAGTTGCGTTGGCTAAGTTGTCATTGTTAAAACGTACTATTTAAGGAGTTTTTATGAAGATG
GTTATTGTTTCTATTAAGGATACTGCTGCAGATGCTTTTGGTCGTCCAGCTTATGTTGCATCTGAAGGTGTTGCAGTACGTCAGTTTCAGGATGAA
GTCAATCGAGCTAGCGAAGATAATCAGTTGTATAAACATCCTGATGATTTTCATATGTTCTATTTGGGTCTTTTTGACGATGCCACTGGTGTTTTT
GAACTACTGGAAAGCCCTAAGTTGATTGCTCGTGCAAAAGATGTAATGATTCGCGAAGGCGAGTAAGGTTTTTTTATACCGTATCACTCGAAAG
AGTGGTACGGAACTACGGGAGATGTTTATGTTTCGCAATAAGTCAGTAAGTACGCATTCATTTGCTATGGTTCCTAAAGCGGACATTCCCCGCTC
TAGTTTTAATACTCAATATGCTCATAAAACCACGTTTGATGCTGGTTTTTTAGTTCCTATTTATTGTGATGAAGTATTGCCTGGCGATACTCATCGT
GTAAAGATGACTGCATTTGCACGTTTGGCCACACCGTTATTTCCTGTGATGGACAACTTGCATCTTGATACTTTCTTTTTCTTTGTACCTAATCGTTT
ACTTTGGAACAATTGGCCAAAGTTTATGGGTGAACAAACGAATCCTGGTGATTCTATTTCTTTTGTAGTGCCTACTATTACTAGTCCTGCTGGTGG
TTATGCTGTTTGTTCAATTTTTGATTATTTTGGTTTACCTACTGCTGGTCAGATTACTGGCGCTAATACAGTAACGCATAATGTTTTGCCGTTACGT
GCTTATAATGAGATTTATAACGAATGGTTTAGAGATGAAAACTTACAGAATTCTGTAACGTTAAATCTTGGTGATTCAGGTGATGTTCCTGCTAAC
TATACACTTTTGAGACGTGGTAAGCGTAAAGATTATTTTACTGGTGCATTGCCTTGGCCACAGAAGGGTGCTTCTGTTTCTTTACCGTTAGGAACA
CGTGCTAATATTTATTCTGACATACCAGCTGGCAATGGTACTGCTGGTTATAGTGTTTTTCAAACTGCTGTTGGTGCTTTAAGAGAATTAAATTCA
GCTTCTAATACTTTGTCTAATAGTACAAATGCTGGTGTTGCTACTAATCAGTTATACGCTGATTTGTCTACTGCTACTGCTGCGACTATTAACCAAC
TTCGTCAATCTTTCCAGATTCAGAAGTTATTGGAGCGCGATGCACGTGGTGGTACTCGTTATACTGAGTTACTACGTGCTCACTTTGGAGTAACTC
CACAGGATTATCGTTTACAACGTCCTGAATATATTGGTGGAGGTTCGACCCTTGTTAATGTTAATCCGATTGCTCAGACTTCTGCAACGTCGGTTA
CTGGTTCTGCTACTCCGCAAGGTAACCTTGCTGCAATGGGTACTGCATTGGCTCAGGGACACGGCTTTACGTATGCTGCTCAAGAACATGGATAC
ATTATCGGATTAGTTTCTGTACGTGCTGACCTCACATATCAACAGGGTCTTCCTAAGATGTGGTCTAGGTCTACACGATATGACTTTTATTTCCCA
GTATTTGCCACTTTGGGTGAGCAAGCTATTTTGAACAAAGAAATTTATGTTCAAGGTACTGCAGCCGACAATGATGTATTTGGTTATCAAGAACG
TTGGGCGGAGTATCGTTACAAACCTTCTCAAATTACTGGTTTCTTTAGGTCTACTTCTGCTGGCACTATTGATGCTTGGCATTATGGACAGCGATT
TACTTCTCTTCCTACGTTGAATTCAACGTTTATTCAAGAGACCCCTCCAGTTGCTCGTACTACGGCGGTCGGAGCTGCAGCAAATGGTCAGCAATT
TTTAATGGATGCTTTCTTTGATTGTCAGATGGCCAGACCTATGCCTATGTACAGCGTACCTGGTCTAATTGATCATTTCTAATGTTTTATATAACCT
CGACTACTCCGTAAGGAGTAGTGAGGAAACAACCGAAGGGCGTTAGTTTATGTTTGGTGGAATACTTGATGCGGTTACTAATGTTGGTTCTAAG
CTGTCTTCAGCTTCTAGTTTCTTTACTCCTGGTGTCGGTACTGCTTTGGGCGCTGTTGGTTCTTATTTAGGTTCTACTTCTGCTAATAAAGCTAATCA
GGAGATGGCTCAGAGGCAAATGGATTTTCAAGCCGATATGAGTGGAACAAGTTACCAGCGTGCTGTTAAAGATTTAGAAGCTGCTGGTTTATCT
CCTATGTTAGCCTATCAACGTGGTGGTGCTTCTACCCCATCTGGTTCAACTGCTACTATGGAAAATGTTTTAGGTAATGCAACTAATTCAGCTATT
AATACTGCTTCTATGATGCAACAGATTCGTAATGCATCAGAAACAGAAAAGCAGATTATCGCCCAGACTGAAGCTACTGAAGCTGGTACCGCTA
ATACTAGGGCTGATACTGTTAATAAGTTGCTTACTGCTCCTAATATTACAGCCGAAAATAAACGTATTTTGGCTGATATTGCTTTAAAGAATACGA
CTGCGGATTTAACATCCGCTCAGTCATATAATACTAAGAGGCTATTGGCTCCATCCCCAGCTATTTGGTCTAGGGGTATCGATGCTTCGAAAGAA
ATTTTTGATAAACTCAAAAATAATCCTAATCAACTAACCCCTTGGGGAATTGGAGTCAAATAATGAGTAAAGCGAATTTGCCATTTGTACGTAATC
CGTACAACTATGATAAAGATGAAGCATCGGTAAACGATGCGTTGCTGTGTCAAGACCCAAGTCTTGCTCAACAGCATATGAAAGATGAATGTGA
CATTAATGTCATCATTGAACGTTTCGGGGTTACAGGGGAACTTCCAACGGCCCCTGTATCGCCTCAATACGGCGATTTTAGTGGTGTTACTGATT
ACCATTCTGCGTTGAATCAAATTAACGCAACTATGGACGATTTCATGGCTCTGCCAGCGAAATTAAGAGTCCGATTTGACCATGATCCTGTCAAAT
TATTGGAGTTCCTTGAGAACGACCAGAATCGTGATGAAGCGATTCAATTGGGTCTTATTGATGGACAACCTGTGGTTGAACCCATCGTTTCTACA
GAAACACCTAAGGCCGAAGGATGAAATCCTGAGGCCAGCACAGTTACTCTACTTGATGTAACTGTGCTAGGTGACACCAAACCACTATTTTAACT
ACGGAGTTCATCATGTTACGAAGAAAGCCAGTAAACAAATATAAATCTGCAAAGTCATTTCGCAGAACTGCTAGTAAGACGAAGTCAATTAATAT
GAGACACGCTCCCCAGCGTGGTGGCTATCGTTTGTAATTATGGCCTGTTATAAGCCCTTAACGGCTTATCAATGCAGTGACAGGTCTATAATTTG
GCGGGAAATACCGGGTGCGGATGTAGTCCGTACCCTATCATTGCCTTGTGGTCAGTGTGTTGGTTGTCGCCTTGAACGCTCACGTCAGTGGGCG
ATTCGTTGTATGCATGAGGCACAAATGCATACTAGTAATTGTTTATTACTTTGACATATGCTCCAGAGCATTGTCCTAAGGATATGTCATTGGAT
TACAATGATTATCAGCTTTTTATGAAGCGGTTACGTAAGCGTTTTACTGGGAAAACGATACGTTTTTATATGGCAGGTGAATATGGTGAATCTTTT
GATCGTCCTCATTTCCATGCTTGTCTGTTTGGTCTTGATTTTCCGGATAAGAAAATATTTAAAAGAACGCAGACTGGCTCTATCCTCTACACGTCAG
AGATTTTGGAAGAATTGTGGCCGTTTGGCTATTCTACAATTGGTGATGTTACTTTTGAGTCTGCTGCTTATGTTGCAAGATATATTATGAAGAAGA
TTAATGGGGTTACTGTCAATGAAAACCACGAAGTGGTTGATGCGGGTGCCCATTATCAATATTGTGATTTAGAGACTGGTGAGATAATTCAGCG
TAAGCCAGAATTTAATAAGATGTCATTGAA

>000184F|arrow

ATTTGTTTTTTCTGCTTGTTCTTCAATAAGAACATTTTGATTACGCATATTTGAATATTGAGCTGTAGCAATTGCAGCCTCTCTAGCTGAATTTCCG
GCTTCGCCAAGTGGATTCCCCATTTGTGCAGTAGCACCCTGAGGTGTCCCTGCACCACCTTGTGTATAAGCTAACATAGGATTAAGACCTGCTGC
CTTAAGATCTGCTACTGTTGTTTGATATTGGGTTTCCCGCATACGCTCTTGAAAATCCATTGTTTTTGCAGCTTGTTCAGCATTAGCAGCATTTTGG
GCTTTACCACCGAATAAACTGGTGGCAGCCCCTATGCCTGCTCCTATTACTGAACCTAAGCCTTGACTAATATTAAACATATTAGAAATGGTCGAT
TAAACCAGGTACGCTATACATTGGCATTGGGCGTGCTTTCTTTACATCAAAGAAAGAATCAAAGATAAATTGTTGGCCGTTTGCGGCTGCTCCTA

CAGCAAGTGCTCTTGCTAGTGGAGGTGTATCTTGAATAAACGTAGTATTCAAGGTTGGTACGGCAGTAAATTTCTGGGCTAAATGCCATCCGTCA
ATTGTGCCGGCAGCTGTTGATCTAAATAAACTGGAAATTCGGCTTGGATAATAACGGTATTCTGCCCACCGTTCTTGATATCCAAACACGCCATTG
TCTTGTGTTGTGTCGCCAGTTACATATATTTCCTTGTTTAGTACAGCTTGTTCGCCTAATGTAGCGAAAGCTGGGAAATAAAAATCGTAACGTGTT
GATCTGCTCCACATTTTTTGCAGACCTTGTTGATATGTCAAGTCTGCTCTTACGGCTACTATACCAAGTATAACGCCGTGTTCAACGAATGATTGA
GTAAAGCCATGATTATGAGCAAGCCCAGTACCCATAGAAGCAAGTGTGCCCAGAGGCGTTGTTGTACCACTTGCATTAGTGCCCGAAGTTTGGG
CAATTGGGTTGATGTTGATAGGAGTCGTACCACCACCCAGATACTCAGGGCGCTGTAAGCGAGCATCAGGGCTAATAACGCCAAAGTGAGAAC
GAATAATTTCAGTATATCGAGTACCTCCACGGGCGTCCCTCTCAAGTAATTTTTGAATCTGAAATGATTGACGTAATTGATTAATTGTTGCTGCAG
TGGCTTGTGATAGGTCAGCGTATAAATTTGATTGAAAATTTACACCTGTACCTGATACTACACCTCCAAGGGTAGTGTTATAACGGCCATTTAACG
CACCTGTATTATCTACAGACATAACAGCAAAGTTATCTAAAGGTGCTGATTGAGTATTTGCTGCAGTCGCAGGACCGTATTTTATTGGAGCAGAT
GATCCTAATGGTAATGTAACACTTGCTCCTTTTTGTGGCCATGGTAAAGCTGATGTGAAATAATCTTTACGCTTGCCACGTCTCAGTAGTGTGTAA
TTAGTTACTGTATCTGGACCATCGCCAGTATCTACTACTACTGAGTTTTGAAGGTTTTCGTCCCGAAACCATTCATTGTAGATAAGGTTATATGCG
CGTGTCCAAAACGCGCCATGACTTACTGTCCTTCCTGTATCTACTTGTCCTACAGTGGGTAAACCCATGTAGTCCTGTAACGAACCGATTGCATAT
CCGTTCGTTGGTGACACCTGTTGGGGTACTACGTACGATATTGAATCCGATGGATTATTTTGTTGCCCCATGAACTTTTGCCAATTGCTCCAAATC
AAACGATTTGGTACAAAGAAGAAAAAAGATTCAAGGTGCAAATTATCCATAATTGGATAAAGTGGGGTTGACAGACGGGCAAATGCCGTCATA
TTTAAATTGAATGTGTCCCCGGGTAGAACTTCGTCTACGTATACGGGGACTAAGTAGCCCGCATCAAATGTAGTTTTATGAGTACTCTGACAGTC
AAATTTTGATCGCGGAATTTCCGCTTTTGGAATCATTGTGAACTGATGTACGTTTACTGATTGATTGCGGTGCATTTTGTTTCCTTGGTAGTGTCCT
CAGGAAGAGCCGCGGCCGAGGCCGTGCCTTCCTCGAGGTTAGTTTTGTATTTTTACTTGTTTTCCCAGACTAAGTAATTTTGGTTCTTCATGTAGA
GTAAATCTACCATTGTTATCGTCGAACTCACCAAATTCGTATAAATCGAAATCGTCTGGGTGATTATATAACTGGTTTTCTGCATCCTGGCGATTTA
CTTCATCGCTGAATGATCGGATAGCAACACCAGTAGAGGGTACAAACATTGGTCGACCGTAAGCATCAGCTGCCCGGTCTTTTACTGAACATAGT
ATTAATTTCATGAGGTTTCCTATATGAGGGTTCGTTTTAATTTTTGAAGTTTTGCATTTTGGACTATTTCTTTGACTGCTAGTCTTTCCAACGTATTG
TCTTCGCTATTTAGTTTACCGTTTATTTCACGTTTGTAAAGTATTTCGTCAAATTCGTATGGATAGTCCGTTTTGTATTTTTTATCGTAAAACTTAGG
AGGTTTTACTTTTTTTCCTTTTATTATGACGTAATCATGTGGATATACGTCATTTTTGTATGTTTTGTACCAGTCATAGCCTATTCCAGGCTTTAATG
ACATTTTATTAAATTCGGGTTTACGTGTAGTTATTTCCCCTGTTTCTGAATCCGTTTCCGTGTAATGTTGTTTTGAGTTATGTCCTGTAACTTTTTTC
ATTATATATCGAGCAACGTACGCAGCTGATTCGAAGTTAACATCTCCAATGGAGGAATAACCAAATGGCCAGAGTATTTCAAGGTCGTTGGATCT
ATAAAGCATAGAACCAGAGGGAGACCTTTTCCATAATTTTTTATCATGAAAGTCGTGGCCGAAGATACAGGCGTGGAAGTGAGGTCTGCCGAAA
TTTTCGCCATATTCTCCAGCCATGTAATAGCGGATTCTAGAGTTTCCGAATTTTTTTCGAAGTCGCTTAATGAAGAGTTGAAAGTCTCTGTAATGT
AAGCTGCCATCGCTTGGGAGATGTGTATTGTCATATGTGAGGGTCATAAAACAGTTATTTTCGTGTAATTGGGCTTCATGCATGCAACGCATAGC
CCATTGGCGTGATCTTTCTAGCCTGCAGCCAATACATTGGCCGCAGGGTAAAGATAGTGTTTTGACGGTATTAAACCATCGTCTTTCCTGAAAAA
CGATTGAACCGTCTCCGCATTGATATGCGGTTAAAGGGTGATAACAAGGCATGTGAGGTGCCTGGGGGTTTTATTAGAACCTCCAGCCTCCGCG
CTGCGGGGCTGATCGCATATTAGGCAATTTTGTTTTTGCTGATTGTTTACGAAATGACCTTGCGGATTTCTTTTTATTTACTGATGATCTGCGCATA
TACATATTTATCTCCTTTTGTGGTTAGGTGTCACCTAGCACAGTTACATCTAGTAGGGTAACTGTGCTTGCTGCCTATTCGGCAGCTTTTTCTTGAG
GAACTTCAACGACTTGCGGCAGTTCCTCAGGTTGTTGTACGAGGCCTAATTTAATAGCCTCGTCTTTATTATCTGAATTATCTAAAAATTCAATAA
GTTTTGCAGGATCGTTGTCGAACCTGCTTCGAATTTGGGCTGGTAAAGCCATAAATTCGTCTTCTGCGGCTATAACTTGGTTAAGCGCAGTATGG
TAGTCACTGATACCAGTGAAATCGCCATAGCGAGGCGATAATGGACTTTCAGGTAAAAGTCCGGTAATGTTGAATTGACGAAGGATATTATTAA
TATCGCATTCGTCTTTAAAATGCTGCTGAGTCAGAGTTGCATCCTCACAATGCAACCCTGACTCATTTGTTGCAGCATCTGTGTCATAGTTGTATG
GTGTACGTAAAAATGGTGATTTTTTCATTTCATTTTTCCAAATGGTAAGTATTTTTGATATTTATCATATTGGCTATGTAACATACCCTTAATATCCT
GATAAATTGGTTTTGTACTGGAAGGTGCACTTCCAGTTTTCGCTAAATCAGTTAATTCTTTAGTATAGCGAGTTTGTTGCAAAGCTTGCCCTGATT
GGGCAGCTTGTAATGCACTGGATGTTTGTAATTGGTCAATTTGTGCATCTCTTAATTGCCCAAATTTGCCATAACCAGGCATTTGCGCTAATTCAC
GTGCTGTTTGTGCACGTACATTAGCAGCTTGATCTATAGATAAATATCGATCTGC

>000226F|arrow

TCTTGAATAAACGTTGAATTCAACGTAGGAAGAGAAGTAAATCGCTGTCCATAATGCCAAGCATCAATAGTGCCAGCAGAAGTAGACCTAAAGA
AACCAGTAATTTGAGAAGGTTTGTAACGATACTCCGCCCAACGTTCTTGATAACCAAATACATCATTGTCGGCTGCAGTACCTTGAACATAAATTT
CTTTGTTCAAAATAGCTTGCTCACCCAAAGTGGCAAATACTGGGAAATAAAAGTCATATCGTGTAGACCTAGACCACATCTTAGGAAGACCCTGT
TGATATGTGAGGTCAGCACGTACAGAAACTAATCCGATAATGTATCCATGTTCTTGAGCAGCATACGTAAAGCCGTGTCCCTGAGCCAATGCAGT
ACCCATTGCAGCAAGGTTACCTTGCGGAGTAGCAGAACCAGTACCGACGTTGCAGAAGTCTGAGCAATCGGATTACATTAACAAGGGTCGAACC
TCCACCAATATATTCAGGACGTTGTAAACGATAATCCTGTGGAGTTACTCCAAAGTGAGCACGTAGTAACTCAGTATAACGAGTACCACCACGTG
CATCGCGCTCCAATAACTTCTGAATCTGGAAAGATTGACGAAGTTGGTTAATAGTCGCAGCAGTAGCAGTAGACAAATCAGCGTATAACTGATT
AGTAGCACACCAGCATTTGTACTATTAGACAAAGTATTAGAAGCTGAATTTAATTCTCTTAAAGCACCAACAGCAGTTTGAAAACACTATAACCA
GCAGTACCATTGCCAGCTGGTATGTCAGAATAAATATTAGCACGTGTTCCTAACGGTAAAGAAACAGAAGCACCCTTCTGTGGCCAAGGCAATG
CACCAGTAAAATAATCTTTACGCTTACCACGTCTCAAAAGTGTATAGTTAGCAGGAACATCACCTGAATCACCAAGATTTAACGTTACAGAATTCT
GTAAGTTTTCATCTCTAAAACCATTCGTTATAAATCTCATTATAAGCACGTAACGGCAAAACATTATGCGTTACTGTATTAGCGCCAGTAATCTGA

CCAGCAGTAGGTAAACCAAAAATAATCAAAAATTGAACAAACAGCATAACCACCAGCAGGACTAGTAATAGTAGGCACTACAAAAGAAATAGA
ATCACCAGGATTCGTTGTTCACCCTAAACTTTGGCCAATTGTTCCAAAGTAAACGATTAGGTACAAAGAAAAAGAAAGTATCAAGATGCAAGTTG
TCCATCACAGGAAATAACGGTGTGGCCAAACGTGCAAATGCAGTCATCTTTACACGATGAGTATCGCCAGGCAATACTTCATCACAATAAATAGG
AACTAAAAAACCAGCATCAAACGTGGTTTTATGAGCATATTGAGTATTAAAACTAGAGCGGGGAATGTCCGCTTTAGGAACCATAGCAAATGAA
TGCGTACTTACTGACTTATTGCGAAACATAAACATCTCCCGTAGTTCCGTACCACTCTTTCGAGTGATACGGTATAAAAAAAAACCTTACTCGCCT
TCGCGAATCATTACATCTTTTGCACGAGCAATCACTTAGGGCTTTCCAGTAGTTCAAAAACACCAGTGGCATCGTCAAAAAGACCCAAATAGAAC
ATATGAAAATCATCAGGATGTTTATACAACTGATTATCTTCGCTAGCTCGATTGACTTCATCCTGAAAACTGACGTACTGCAACACCTTCAGATGC
AACATAAGCTGGACGACCAAAAGCATCTGCAGCAGTATCCTTAATAGAAACAATAACCATCTTCATAAAAACTCCTTAAATAGTACGTTTTAACA
ATGACAACTTAGCCAACGCAACTTTTTCCTTAACAGCAAGTCGCTCAGCGTGTTGTCCTCATGCCTAGATCGACCTTCCATCTCTCTGGCAAACTG
AATCATATCGAATTCTTCAGGAAACTCAACTTAAATTATTATCATAAAACCGTGGTGGACGGCACTTTTTGCCACGCACCACAACGTGGTCTGACG
TAAAACGTCTGACATGTACTTATCTACACCACGATTGCCCGATACCGGGCTTCAATGGACATCTTATTAAATTCTGGCTTACGCTGAATTACTCTCA
CCAGTCTCTAAATCACAATATTGATAATGGCACCCCGCATCAACCACTTCGTGGGTGTTTCATTGACAGTAACCCCATTAATCTTCTTCATAATATA
GTCTTGCCAACATAAGCAGCAGACTCAAAAGTAACATCACCAATGGTAGAATAGCCAAACGGCCACCAATTCTTCCAAATCTCTGACGTGTAGAG
GATAGAGCCAGTCTGCGTTCTTTTAAATATTTTCTTATCCGGAAAATCAAGACCAAACAGGACAAGCATGGAAATGAGGACGATCAAAGATTCAC
CATATTCACCTGCCATATAAAACGTATCGTTTTCCCAGTAAAACGCTTACGTAACCGCTTCATAAAAAGCTGATAATCATTGTAATCCAATGACAT
ATCCTTAGGACAATGCTCTGGAGCATATGTCAAGTAATAAAACAATTACTAGTATGCATTTGTGCCTCATGCATACAACGAATCGCCCCTGACGT
GAGCGTTCAAGGCGACAACCACACACTGACCACAAGGCAATGATAGGGTACGGACTACATCCGCACCCGGTATTTCCCGCCAAATTATAGACCT
GTCACTGCATTGATAAGCCGTTAAGGCTTATAACAGCCATAATTACAAACGATAGCCACCACGCTGGGAGCGTGTCTCATATTAATTGACTTCGT
CTTACTAGCAGTTCTGCGAAATGACTTTGCAGATTTATATTTGTTTACTGGCTTTCTTCGTAACATGATGAACTCCGTAGTTAAAATAGTGGTTTGG
TGTCACCTAGCACAGTTACATCAAGTAGAGTAACTGTGCTGGCCTCAGGATTTCATCCTTCGGCCTTAGGTGTTTCTGTAGAAACGATGGGTTCA
ACCACAGGTTGTCCATCAATAAGACCCAATTGAATCGCTTCATCACGATTCTGGTCGTTCTCAAGGAACTCCAATAATTTGACAGGATCATGGTCA
AATCGGACTCTTAATTTCGCTGGCAGAGCCATGAAATCGTCCATAGTTGCGTTAATTTGATTCAACGCAGAATGGTAATCAGTAACCACTAAAAT
CGCCGTATTGAGGCGATACAGGGGCCGTTGGAATTCCCCTGTAACCCCGAAACGTTCAATGATGACATTAATGTCACATTCATCTTTCATATGCT
GTTGAGCAAGACTTGGTCTTGACACAGCAACGCATCGTTTACCGATGCTTCATCTTTATCATAGTTGTACGGATTACGTACAAATGGCAAATTCGC
TTTACTCATTATTTGACTCCATTCCCCAAGGGGTTAGTTGATTAGGATTATTTTGAGTTTATCAAAAATTTCTTTCGAAGCATCGATACCCCTAGAC
CAAATAGCTGGGGATGGAGCCAATAGCCTCTTAGTATTATATGACTGAGCGGATGTTAAATCCGCAGTCGTATTCTTTAAAGCAATATCAGCCAA
AATACGTTTATTTTCGGCTGTAATATTAGGAGCAGTAAGCAACTTATTAACAGTATCAGCCCTAGTATTAGCGGTACCAGCTTCAGTAGCTTCAGT
CTGGGCGATAATCTGCTTTTCTGTTTCTGATGCATTACGAATCTGTTGCATCATAGAAGCAGTATTAATAGCTGAATTAGTTGCATTACCTAAAAC
ATTTTCCATAGTAGCAGTTGAACCAGATGGGGTAGAAGCACCACCACGTTGATAGGCTAACATAGGAGATAAACCAGCAGCTTCTAAATCTTTAA
CAGCACGCTGGTAACTTGTTCCACTCATATCGGCTTGAAATCCATTTGCCTCTGAGCCATCTCCTGATTAGCTTTATTAGCAGAAGTAGAACCTAA
ATAAACCAACAGCGCCCAAAGCAGTACCGACACCAGGAGTAAAGAAACTAGAAGCTGAAGACAGCTTAGAACCAACATTAGTAACCGCATCAA
GTATTCCCCAAACATAAACTAACGCCCTTCGGTTGTTTCCTCACTACTCCTTACGGAGTAGTCGAGGTTATATAAAACATTAGAAATGATCAATTA
CCAGGTACGCTGTACATAGGCATAGGTCTGGCCATCTGACAATCAAGAAAGCATCCATTAAAATTGCTGACCATTTGCTGCAGCTCCGACCGCCG
TAGTACGAGCAACTGGAGGGGTC

>000168F|arrow

CCTCTTAGTATTATATGACTGAGCGGATGTTAAATCCGCAGTCGTATTCTTTAAAGCAATATCAGCCAAAATACGTTTATTTTCGGCTGTAATATTA
GGAGCAGTAAGCAACTTATTAACAGTATCAGCCCTAGTATTAGCGGTACCAGCTTCAGTAGCTTCAGTCTGGGCGATAATCTGCTTTTCTGTTTCT
GATGCATTACGAATCTGTTGCATCATAGAAGCAGTATTAATAGCTGAATTAGTTGCATTACCTAAAACATTTTCCATAGTAGCAGTTGAACCAGAT
GGGGTAGAAGCACCACCACGTTGATAGGCTAACATAGGAGATAAACCAGCAGCTTCTAAATCTTTAACAGCACGCTGGTAACTTGTTCCACTCAT
ATCGGCTTGAAAATCCATTTGCCTCTGAGCCATCTCCTGATTAGCTTTATTAGCAGAAGTAGAACCTAAATAAGAACCAACAGCGCCCAAAGCAG
TACCGACACCAGGAGTAAAGAAACTAGAAGCTGAAGACAGCTTAGAACCAACATTAGTAACCGCATCAAGTATTCCACCAAACATAAACTAACG
CCCTTCGGTTGTTTCCTCACTACTCCTTACGGAGTAGTCGAGGTTATATAAAACATTAGAAATGATCAATTAGACCAGGTACGCTGTACATAGGCA
TAGGTCTGGCCATCTGACAATCAAAGAAAGCATCCATTAAAAATTGCTGACCATTTGCTGCAGCTCCGACCGCCGTAGTACGAGCAACTGGAGG
GGTCTCTTGAATAAACGTTGAATTCAACGTAGGAAGAGAAGTAAATCGCTGTCCATAATGCCAAGCATCAATAGTGCCAGCAGAAGTAGACCTA
AAGAAACCAGTAATTTGAGAAGGTTTGTAACGATACTCCGCCCAACGTTCTTGATAACCAAATACATCATTGTCGGCTGCAGTACCTTGAACATA
AATTTCTTTGTTCAAAATAGCTTGCTCACCCAAAGTGGCAAATACTGGGAAATAAAAGTCATATCGTGTAGACCTAGACCACATCTTAGGAAGAC
CCTGTTGATATGTGAGGTCAGCACGTACAGAAACTAATCCGATAATGTATCCATGTTCTTGAGCAGCATACGTAAAGCCGTGTCCCTGAGCCAAT
GCAGTACCCATTGCAGCAAGGTTACCTTGCGGAGTAGCAGAACCAGTAACCGACGTTGCAGAAGTCTGAGCAATCGGATTAACATTAACAAGG
GTCGAACCTCCACCAATATATTCAGGACGTTGTAAACGATAATCCTGTGGAGTTACTCCAAAGTGAGCACGTAGTAACTCAGTATAACGAGTACC
ACCACGTGCATCGCGCTCCAATAACTTCTGAATCTGGAAAGATTGACGAAGTTGGTTAATAGTCGCAGCAGTAGCAGTAGACAAATCAGCGTAT
AACTGATTAGTAGCAACACCAGCATTTGTACTATTAGACAAAGTATTAGAAGCTGAATTTAATTCTCTTAAAGCACCAACAGCAGTTTGAAAAAC

ACTATAACCAGCAGTACCATTGCCAGCTGGTATGTCAGAATAAATATTAGCACGTGTTCCTAACGGTAAAGAAACAGAAGCACCCTTCTGTGGCC
AAGGCAATGCACCAGTAAAATAATCTTTACGCTTACCACGTCTCAAAAGTGTATAGTTAGCAGGAACATCACCTGAATCACCAAGATTTAACGTT
ACAGAATTCTGTAAGTTTTCATCTCTAAACCATTCGTTATAAATCTCATTATAAGCACGTAACGGCAAAACATTATGCGTTACTGTATTAGCGCCA
GTAATCTGACCAGCAGTAGGTAAACCAAAATAATCAAAAATTGAACAAACAGCATAACCACCAGCAGGACTAGTAATAGTAGGCACTACAAAAG
AAATAGAATCACCAGGATTCGTTTGTTCACCCATAAACTTTGGCCAATTGTTCCAAAGTAAACGATTAGGTACAAAGAAAAAGAAAGTATCAAGA
TGCAAGTTGTCCATCACAGGAAATAACGGTGTGGCCAAACGTGCAAATGCAGTCATCTTTACACGATGAGTATCGCCAGGCAATACTTCATCACA
ATAAATAGGAACTAAAAAACCAGCATCAAACGTGGTTTTATGAGCATATTGAGTATTAAAACTAGAGCGGGGAATGTCCGCTTTAGGAACCATA
GCAAATGAATGCGTACTTACTGACTTATTGCGAAACATAAACATCTCCCGTAGTTCCGTACCACTCTTTCGAGTGATACGGTATAAAAAAAAACCT
TACTCGCCTTCGCGAATCATTACATCTTTTGCACGAGCAATCAACTTAGGGCTTTCCAGTAGTTCAAAAACACCAGTGGCATCGTCAAAAAGACCC
AAATAGAACATATGAAAATCATCAGGATGTTTATACAACTGATTATCTTCGCTAGCTCGATTGACTTCATCCTGAAACTGACGTACTGCAACACCT
TCAGATGCAACATAAGCTGGACGACCAAAAGCATCTGCAGCAGTATCCTTAATAGAAACAATAACCATCTTCATAAAAACTCCTTAAATAGTACG
TTTTAACAATGACAACTTAGCCAACGCAACTTTTTCCTTAACAGCAAGTCGCTCAAGCGTGTTGTCCTCATGCCTAGATCGACCTTCCATCTCTCTG
GCAAACTGAATCATATCGAATTCTTCAGGAAACTTCAACTTAAATTTATTATCATAAAACCGTGGTGGACGGCACTTTTTGCCACGCACCACAACG
TGGTCTGACGTATAAACGTCTGACATGTACTTATCTAACCACGATTGCCCGATACCGGGCTTCAATGACATCTTATTAAATTCTGGCTTACGCTGA
ATTATCTCACCAGTCTCTAAATCACAATATTGATAATGGGCACCCGCATCAACCACTTCGTGGTTTTCATTGACAGTAACCCCATTAATCTTCTTCA
TAATATATCTTGCAACATAAGCAGCAGACTCAAAAGTAACATCACCAATTGTAGAATAGCCAAACGGCCACAATTCTTCCAAAATCTCTGACGTG
TAGAGGATAGAGCCAGTCTGCGTTCTTTTAAATATTTTCTTATCCGGAAAATCAAGACCAAACAGACAAGCATGGAAATGAGGACGATCAAAAG
ATTCACCATATTCACCTGCCATATAAAAACGTATCGTTTTCCCAGTAAAACGCTTACGTAACCGCTTCATAAAAAGCTGATAATCATTGTAATCCAA
TGACATATCCTTAGGACAATGCTCTGGAGCATATGTCAAAGTAATAAAACAATTACTAGTATGCATTTGTGCCTCATGCATACAACGAATCGCCC
ACTGACGTGAGCGTTCAAGGCGACAACCAACACACTGACCACAAGGCAATGATAGGGTACGGACTACATCCGCACCCGGTATTTCCCGCCAAAT
TATAGACCTGTCACTGCATTGATAAGCCGTTAAGGGCTTATAACAGGCCATAATTACAAACGATAGCCACCACGCTGGGGAGCGTGTCTCATATT
AATTGACTTCGTCTTACTAGCAGTTCTGCGAAATGACTTTGCAGATTTATATTTGTTTACTGGCTTTCTTCGTAACATGATGAACTCCGTAGTTAAA
ATAGTGGTTTGGTGTCACCTAGCACAGTTACATCAAGTAGAGTAACTGTGCTGGCCTCAGGATTTCATCCTTCGGCCTTAGGTGTTTCTGTAGAA
ACGATGGGTTCAACCACAGGTTGTCCATCAATAAGACCCAATTGAATCGCTTCATCACGATTCTGGTCGTTCTCAAGGAACTCCAATAATTTGACA
GGATCATGGTCAAATCGGACTCTTAATTTCGCTGGCAGAGCCATGAAATCGTCCATAGTTGCGTTAATTTGATTCAACGCAGAATGGTAATCAGT
AACACCACTAAAATCGCCGTATTGAGGCGATACAGGGGCCGTTGGAAGTTCCCCTGTAACCCCGAAACGTTCAATGATGACATTAATGTCACATT
CATCTTTCATATGCTGTTGAGCAAGACTTGGGTCTTGACACAGCAACGCATCGTTTACCGATGCTTCATCTTTATCATAGTTGTACGGATTACGTA
CAAATGGCAAATTCGCTTTACTCATTATTTGACTCCAATTCCCCAAGGGGTTAGTTGATTAGGATTATTTTTGAGTTTATCAAAAATTTCTTTCGAA
GCATCGATACCCCTAGACCAAATAGCTGGGGATGGAGCCAATAG

>000040F|arrow

GTGCTGAGTGGTAAAAAATATCGTTCCGACGTATATCCCCATGACTATGTTGTAGTCAACGGAAAAAGGGTAAAACCCCCAAAATACTATGACA
AAAAATATAAATCAGATTATCCATATGAATACGAAGAATTACTCCACAAACGTGAAACTTCTGCTAAACTCAACCACGAAGACAATACCTATGCC
AGACTTGCCGTAAAGGAAAAAGTCACAAAGGCCAAACTTCAATTATTAAAACGTAACCTCACTTAGGAAATCCTCATGAAATTAGTACTCTGTAC
CGTTAAAGACCGCGCAGCAGATGCGTTCGGTCGTCCAATGTTCGTCCGTTCTATCGGCGAAGCAATCCGGAGCTTTAGCGACGAAGTCAATCGC
CAGAGCGATGACAATCAACTTTATAACCATTCCGACGATTTCGACCTATTTGAATTAGGCGAATTCGACGATAATACGGGTTTGTTCCAATTACAT
GAACAACCCAAACTTGTATCCTTAGGGAAACAAGTCAAAATTACTGATAAAAACTAAGCGTAGAGTAAAAAGGGGGAAACCCCTTTTTCTCACG
CAACTAGGCCTAGGAGCTCAAAAAATGCATCGTAACAAGTCGGTAGACGTCCATCAGTTCACAATGATTCCAAAAGCGGATATCCCCCGCTCTAC
ATTTGACTGTCAATCAACACATAAAACTACATTCGATGCTGGCTTCCTAGTCCCTGTACTCGTAGACGAAATGTTGCCAGGCGATACATTCCGCTG
CAACATGACCGCCTTTGCGCGATTGTCTACACCACTCTATCCGATCATGGACAATATGCATTGGATAGCTTCTTCTTTTTGTGCCAAATAGACTTAT
CTGGTCAAATTGGCAAAAATTTATGGGGCAGCAGGCAAATCCTGCGGACTCGATCTCGTACGTAGTGCCCCAACAAGTAACCCCAGCTGGTGGT
TACGCTATTGGCAGCCTTCAAGATTATATGGGTCTGCCAACGTAGGCCAAGTAGGTGCTGGTGGCACCGTAAGTCACTGTGCCTTCTGGCCACGT
GCTTACAACCTTATTTATAACGAATGGTTTCGGGACGAAAACCTTCAAAATTCAGTAGTTGTAGATACTGGCGATGGTCCAGATAACGTAGCCAA
CTACACATTATTACGACGTGGAAAACGTAAAGACTATTTCACGTCAGCATTACCTTGGCCACAAAAGGGCGCAAGCGTTACTTTACCGCTTGGAA
CATCCGCCCCAATATTACGCACTAACAATGCGCCTGTTTCCAGACTGTATAACGCTGGAACAAATACATTAAACGCAACCGCCCAGGCTATTAAC
GTAGGTGTTACTGGTCAAATTACTGGCGGTGCTGACGGCTTGGCAAAATCATATGATCCTAATGGCGGTTTATATGCAGATTTATCAGCTGCAAC
CGCTGCAACAATTAATCAATTGCGTCAAAGCTTCCAGATTCAAAAACTTTTAGAAAGGGACGCCCGTGGCGGAACTCGATACACAGAAATTATCC
GCAGCCATTTCGGGGTCGTTAGCCCCGATGCGCGTCTCCAACGGCCTGAATACATTGGAGGCGGTTCAACACACATTAATATCCAATCGCCCAGA
CGAATGGTACCGGAGCTTCCGGGACCACTACTCCTCTCGGTACACTTGGCGCTATGGGTACTGGGCTCGCTCACAATCATGGCTTTACTTATTCA
AGCACTGAACATGGTGTAATTATCGGTCTCGTTTCAGTACGAGCCGATTTAACATACCAACAAGGTATGCACCGCATGTGGAATCGTTCCACACG
TTATGATTTCTATTTCCCTGCTTTCGCCACTTTGGGCGAACAAGCAGTATTAAATGAAGAAATCTACGTACGAGGCGATGCCAACGATACAGGAG
TGTTTGGATACCAAGAACGTTGGGCAGAATATCGTTATATGCCAAGCCGAATTTCCAGTCTGTTCCGTAGTACGGCAGCTGGAACAATTGACGG

CTGGCATTTAGCCCAACGGTTTACAACACTTCCAACTTTGAATAACACGTTTATTCAAGAAAATCCACCTGTCTCTCGAACCCTTGCGGTCGGAGC
AGCTGCCAACGGCCAGCAAATCATTTTTGATAGCTTTTTTGATATTAAAAAAGCACGGCCAATGCCAATGTACTCTGTACCTGGCTTAATCGACCA
CTTCTAATGGCACTAGAAGCCGCTGCCTCAGGCGCCGCATCTGGCGCCGCTTTTGGACCTTACGGCTCCTTAATTGGAGCCGGAATAGGTGCGG
CCGCTAGTTATTTTGGTGGTCAAGAACAAAACGCTGCCAGCGCACAACAAGCTGCAGCAATGATGCAATTCCAAGATGGTATGCGACGTACTGC
ATATCAAGACGCAGTAGCGGATCTTAAGGCTGCAGGTCTTAACCCTATGCTGGCTTATTCACAAGGCGGAGCCAAAGTCCAGCCTGGTGCGCAA
GCTCCAGTAGGAAATCCACTAGGTGAGGCTGGAAATTCAGCCCGTGAAGCTGCCATGGCAGTCGCCAATTTTAAACAATTACAAACTCAGAATA
TCCTGACACAATCGCAAGCCGAAAAAACGGACGCGGATACAAATCTATCACGTGATCAGGCAACATATACTCGAGCAAATACAGCTCGTGAAAT
TGCTCAGATGCCGGGATACGGCAAATTTGGTCAGCTTCGCGATGCCCAAATAGAGCAATTAAGGACATCAAGTGCATTACAAGCTGCACAACAG
CGACAAGCGTTAAGTCAAAGTGCATATACAGACCAATTAGAGCGATTAGCGCAAACTGGATCAGCGCCATCCAGTACTAAACCAATTTATCAAG
ATGTTAAAGGCTATTTACATAGCCAATATGATAAATATCAAAAATATCTACCATTTGGAAAAATGAAATGAAAACAATCAAACTTAGAACCGCAT
ACAACTATGACACGGATGCTGCGTCAAATGAGTCAGGGTTGGCTTGTGAGGAGCCAACTCTGGCTCAGCAGCATTATAAAGACGAATGCGATAT
AAATACTATCCTGGAACGTTTTAACGTTACAGGCCTATTACCTCAAAGTCCGCTGCCGCCTCAATATGGCGATTTCAGCGGAATTACTGACTATCA
TAGCGCCTTGAATAAGGTAATGAACGCTATGGAAGAATTTGATAACTTACCGGCTCAAATTCGTGCTAGGTTCGAAAACGAACCAGCAAACCTG
ATTGAGTTCTTGCAAGACGAGAAAAATCGACCAGAAGCCGAGAAACTCGGCCTGGTCGAAAGAGCCATTTCGGAAGAAATGGCGATAAGCACA
GTTACTCCACTTGATGTAACTGTGCTAGGTGACACCAACACCAAAAATATCTGATAAACGAGGCCAAAAATTATGCTTTATAGAAAAAAACAAAC
AAGCGCAAAAGCGCTAAATCGTTCCGTAGGAACACTTCAAAAACTAAAGCTGCAAATATGCAAAAAGCCCCGCAAAGAGGGGGCTGGCGGCTC
TAATAAAGCGCCAGGCTACCTCACATGGCCTGTTATCACCCACTGACTGCTTATTTAAGTAAGCATCAGACAAACTATAAGACCGGCAAATCTTAT
CGCCGTGTCGCATTCAAAGAATCTGACGAGCATGATCGTCAGATTTCACTGCCCTGCGGCCAATGCGTTGGCTGCAGGCTAGAAAAATCACGTC
AATGGGCCATGCGCTGCATGAAGCCCAATTGCACGAAAAAAACTGCTTTATAACCCTCACATACAACAATGAAAACCTTCCACAAACTGGATCGC
TTGTCAAAAGCGACTTCCAAAAGTTCCTTAAGCGCTTCAGAAAATCCATTGCACCTGCAAAATTACGTTACTACATGGCTGGAGAATACGGCACA
AGTTTCGGCAGACCTCACTTCCATGCCTGTATCTTCGGATACGATTTTCATGATAAGAAACTATTCAAAAGGACTCCCTCTGGTTCTCTCATATATA
CATCCGACCACCTTGCAACCCTCTGGCCACATGGTTATTCCTCCATTGGAGACGTTACATTCGAGTCAGCTGCTTACGTTGCTCGATATATTATGC
AAAAATACAACGGCCAGATGGAAGAAAACAAACATATAACAAAGGATGAGCATTACACATACTGTGATATAGAAACAGGGGAATTAATAAAGC
TATTACCAGAATATAACAATATGAGCCTTAAACCAGGCATTG

>000015F|arrow

CTGCTACTGGGGGACTCTCCTTAGCTGCACTTGCACCTGCAGCAATAGGTTGCAGCCAGGACAATACTTTGGAACACAAGTCAAATACGCAGCG
ATTGCAGAACAAGCGAGTAATCAACAGAGATTTCAAGCTGAAATGTCTGGAACAGTCATCTCAACGAGCAGTTGAAGATATGAAAAAAAGCTG
GGTTAATCCCATGCTTGCGTATTCACAAGGCGGAGCCACAACACCAGCTGGAGCTATGGCCCGATGCAAAATGTTCTCGGTAATGCAACTACGT
CCGGAACCCAAGCTTATCAACGGTTGCGCAAGCAAATCAAGCTATTGCTCAATCTAAACAAATTGAAGCTCAAACAGAACTCACAAGTAATCAAA
CAGATTAATGTACGTGCTGATACGTTAAATAAATGGGATGAAAATCCAAATATTAGAGCTCAATATAAACAAATACTTGCCGATACTTTCATGAA
AAATGAAATAGGCAAAACATCAAGTGCTCAAAGCTGCTCAAGCTTTGGCACAATCTCGTTATTCAAACGAGTTAACAAAACTTGCTAATCAGGGT
CAGCTCCTAGTTCTAGCAAACCAATTTATCAAGACGTAAAAACATCGCCAAAGATGCTTATAGCGCATCTGGCGCAAAACGATACATCGATAACT
ATCGAGGTCAACCGATTCAACAAAATCGTACAATAACAACCACCAATGGAATGAAAATGACAAGATTACAGCCCCATTCTTCGTACTCCGTACAA
TTACGACACGATTGCTGCGTCAAATGAGTCAGGGCTGCATTGTGAGGATGCAACTCTGACTCAGCAGCAATTTGCTGAAGAATGTGATATCAAT
AATATTAATGGAAAAGGTTCGGTATGACCGGACTTATTCCTCAAACTCCTTTAACGCCTCAATATGGCGACTTTAGTGGTGTCTATGACTACCACT
CTGTCTCTGAACCAGATTATGGCTTCAGACAACGAATTATGGCTTTACCAGCCAATATTCGTGAACGATTCGCTAAGTGATCCCGCGAATCTAATA
GATTTTCTAGAAAACCCTGAATAATCGCAGCGAAGCTGAAAAAATGGGACTGGTAAAACCAGCCCAAACCGAGGTTTCAACCCCTGTTGGAACC
TCGGAAGCACAGTTACCTACTTGATGTAACTGTGGCTAGGTGACACCAACCACAAAAACACGATAAACAAGGACAGAAAAAATGATGCTTCGCA
GACCAGCAAATAAGCAAAAGTCCGCTAGGACTTTCCGTAAACATGCTTCACATACAAAACACGCAAATATGCGAACTCGCCAATGCTGGAGGCT
GAGACTCTAATAAAGTCTTCAGGCACCTCACATGCCTTGTTATCACCCTCTCAAAGCTATCAATGCTTTGACAAATCAATTGTTTTCGACGAAGTTC
GGAAAACATGACATCGTTCGATCTTTAGACCTGCCCTGTGGGCAGTGCGTTGGATGCCGTCTAGAACGATCAAGACAATGGGCTATTCGGTGCA
TGCACGAAGCCCAATTGCATAAAAACAACTCATTCATAACACTCACATATGACAATACACATCTCCCAAGCGATGGCTCTTTGGATCACAAAGACT
TTCAATTGTTCCTTAAAAGACTTAGAAAAACTCTCGCAAAAAGAGGACTTACAATCCGCTATTACATGGCTGGAGAATATGGTGGAACTCTTCGC
AAGACCCCACTTCCATGCCTGTATCTTCGGATACGACTTTCCTGATAAAAAAATTATGGAAAAGGACTGCCTCTGGGTTCTATGTTATATAGATCC
GCAGAACTTGAAGCTCTCTGGCCATTTGGTTTATACCACCATTGGAGATGTTACTTTTGAATCAGCCGCCTACGTGGCTAGATACATAATGAAAA
AACAAACAGGGAAAGATGCGGAATCTCATTACAAACGCATACACCCTGAAACCGGCGAATATTTAGACTTAAAGCCGGAATATAATAAAATGTC
TTATAAAACCGGGAATCGGTAAAGACTTTTATATAAAATATACTTCGGATATATACCCGCAAGACTACGTAATACTTAGAGGTAAAAAGGTCAAA
CCACCAAAATACTATGACAAAATGTTTAAAATTGACCAACCTTATGAGTATGACGAATTACTTTACATGCGGGAAAATAATGCTAAATTTAATTCC
GAAGACAATACACCAGAACGACTATCTGCAAAAGAACAAGTCACTATGGCAAAACTTCAACTATTAAAACGTAACCTTACTTAGGAAAATAATGA
AACTTATCCTCGCTTCCGTAAAAGACCGTGCTGCTGAAGCATATGCACGACCAATGTTCGTACCTTCCTCTGGAGTACTATACGCTCTTTTTCAGAT
GAAAGTAATCGTTCTGATACTGAAAATCAACTCTTTAATCACCCTGATGATTTCGATCTATATGAATTCGGAACATTTGACGATTCAACTGGGTTA

TTCGATTTACATGAACAACCAAAACTCCTATCATTAGGAAAACAAGTTAAACTTAAATAAAACAACCGAGGGGAAAAGAGATTTATCTTTCCCCC
GGAACAACACTAAGGAAAAACATGCACCGCAATCAGTCAGTTAATACTCACCGCTTCGCGATGGTACCTAGAGCCGATATACCACGTAGTAAAT
TCGATGCTCAAAAAACACATAAAACGACTTTCGATGCGGGCTATCTAATTCCTGTATATGTTGATGAAGTGCTCCCTGGGGACACTTTCAACTTAA
AAATGACGGCATTTGCCCGTCTAGCAACGCCTTTATATCCAATCATGGATAACATGATTATGGATTCTTTCTTTTTCTTTGTACCCAATCGCCTTAT
ATGGAATAACTGGCAAAATTTATGGGTCAACAAGAAAATCCAACAGACTCAATATCTTATATTGTCCCAACACAAACAAGCCCAACAGATGGTTA
TGCCGTAGGCAGCCTTCAAGACTATATGGGCTTACCAACAGTAGGCCAAATTGATACTGGCCGAACTATTACGCACTGTGCCTTTTGGCCACGTG
CATACAATCTTATCTGGAACGAATGGTTCCGAGATGAAAATTTACAAACAAGCGCAGTAGTTGATAAGGGCGATGGCCCTGATACTTCCTCAAAC
TATGTGCTAAAACGTCGTGGTAAAAGACATGATTACTTTACGTCAGCATTACCATGGCCACAAAAAGGTGCGAGTGTCACCTTACCTTTAGGTAC
TACGGCTCCAATTAAATGGGATACCATTTCAGGAGACGCAACATCAAACGATAAATTTACGGTAATTCAAACAGATCCTGGAAATACGACTGCTT
TAGCTAGATATGGCAACGCTTATGGTGTTAATACTGCTGGTGTAGTAAATAACGTTTCTAATTTATATACCGACTTATCAGAAGCAACTGCTGCAA
CTGTCAATCAATTAAGACAGTCATTTCAAATTCAAAAATTACTTGAAAGGGATGCACGTGGCGGAACACGATACACAGAAATTATCCGGAGTCAC
TTTGGAGTTATTTCCCCAGACGCCCGTTTACAAAGGCCTGAATACCTTGGAGGCGGTTCAACACCAATTAATGTTAATCCGATTGCTCAAACGTCG
GGAACAAACGCTTCTGGAACGACTACCCCTTTGGGCAACCTTGCTGCTATGGGTACTGCTCTCGCTCATAATCATGGATTTACTCAATCATTTACT
GAGCATGGCGTTATTATTGGATTAGTATCCATTAGAGCAGATCTTACTTATCAACAAGGATTAGACCGTATGTGGTCTAGATCTACACGATATGA
CTTTTATTTCCCAGCATTTGCTACTCTAGCGAACAATCTGTTTTGCAAAAAGAAATTTATGCAACAGGAGATACTGCAGCCGACATACTGTTTTTG
ATATCAAGAACGCTGGGCGGAATATCGTTACAAACCATCTAAAATTACTGGTTTGTTCAAATCAACATCGGCGGGCACGATCGATGGTTGGCATT
TGGCTCAAAAATTTACCGCTGCGCCTACTTTGAATAATACGTTTATTCAAGATACGCCTCCTGTATCACGTGTAGTAGCCGTTGGAGCAGCTGCAA
ATGGCCAACAATTCTTATTTGACTCATTTTTTGATGTCAAAATGGCAAGACCAATGCCAATGTATTCAGTACCTGGCTTAATAGACCATTTCTAATG
GGACTATTTGACGGAATTGCCGATTTAATCGGCCCTGCTATAGCTATAGGAGCTGCCC

>000088F|arrow

AATAGTTGAAGTTTTGCCATAGTTACTTGTTCTTTTGCAGATAGTCGTTCTGGTGTATTGTCTTCGGAATTAAGTTTAGCGTTATTTTCCCGCATGT
AAAGTAATTCGTCATACTCATAAGGTTGGTCAATTTTAAACATTTTGTCATAGTATTTTGGTGGTTTGACCTTTTTACCTCTAAGTATTACGTAGTC
TTGCGGGTATATATCCGAAGTATATTATATAAAAGTCTTTACCGATTCCCGGTTTTAAAGACATTTTATTATATTCCGGCTTTAAGTCTAAATATTC
GCCGGTTTCAGGGTGTATGCGTTTGTAATGAGATTCCGCATCTTTCCCTGTTGTTTTTTCATTATGTATCTAGCCACGTAGGCGGCTGATTCGAAA
GTAACATCTCCAATGGTGGTATAACCAAATGGCCAGAGAGCTTCAAGTTCTGCGGATCTATATAACATAGAACCAGAGGCAGTCCTTTTCCATAA
TTTTTATCAGGAAAGTCGTATCCGAAGATACAGGCATGGAAGTGGGGTCTTGCGAAGAGTTCACCATTCTCCAGCCATGTAATAGCGGATTGTA
AGTCTCTTTTTGCGAGAGTTTTTCTAAGTCTTTTAAGGAACAGTTGAAAGTCTTTGTGATCCAAAGAGCCATCGCTTGGGAGATGTGTATTGTCAT
ATGTGAGTGTTATGAATGAGTTGTTTTATGCAATTGGGCTTCGTGCATGCACCGAATAGCCCATTGTCTTGATCGTTCTAGACGGCATCCAACGC
ACTGCCCACAGGGCAGGTCTAAAGATCGAACGATGTCATGTTTCCGAACTTCGTCGAAAACAATTGATTTGTCAAAGCATTGAAATGCTTTGAGA
GGGTGATAACAAGGCAATGTGAGGTGCCTGGGGACTTTATTAGAGTCTCCAGCCTCCACGCATTGGCGAGTTTCGCATATTTGCGTGTTTTGTAT
GTGAAGCATGTTTACGGAAAGTCCTAGCGGACTTTTGCTTATTGCTGGTCTGCGACGCATCATTTTTCTGTCCTTGTTTATCGTGTTTTGTGGTTTG
GTGTCACCTAGCACAGTTACATCAAGTAGGTAACTGTGCTTCCGAGGTTCCAACAGGGGTTGAAACCTCGGTTTGGGCTGGTTTTACCAGTCCCA
TTTTTCAGCTTCGCTGCGATTTTCAGGGTTTCTAGAAAATCTATTAGATTCGCGGGATCATTAGCGAATCGTTCACGAATATTGGCTGGTAAAGCC
ATAAATTCGTTGTCTGAAGCCATAATCTGGTTCAGAGCAGAGTGGTAGTCATAGACACCACTAAAGTCGCCATATTGAGGCGTTAAAGGAGTTT
GAGGAATAAGTCCGGTCATACCAAACTTTTCCATAATATTATTAATATCGCATTCTTCAGCAAATTGCTGCTGAGTCAGAGTTGCATCCTCACAAT
GCAGCCTGACTCATTTGACGCAGCAATCGTGTCGTTGTACGGAGTACGAAGAAATGGGGCTGTAATCTTTGTCATTTTCATTCCATTGGTGGTTG
GTTATTTGTACGATTTTGTTGAATCGGTTGACCTCGATAGTTATCGATGTATCGTTTTGCGCCAGATGCGCTATACGCATCTTTGGCGATTTTTTTA
CGTCTTGATAAATTGGTTTGCTAGAACTAGGAGCTGACCCTGATTAGCAAGTTTTGTTAACTCGTTTGAATAACGAGATTGTGCCAAAGCTTGAG
CAGCTTGAGCACTTGATGTTTTGCCTATTTCATTTTCATGAAAGTATCGGCAAGTATTTGTTTATATTGAGCTCTAATATTTGGATTTTCATCCAATT
TGTTTAACGTATCAGCACGTACATTATCTGTTTGATTACTTGTGAGTTCTGTTTGAGCTTCAATTTGTTTAGATTGAGCAATAGCTTGATTTGCTTG
AGCAACCGTTTGATAAGCTTGGGTTCCGGACGTAGTTGCATTACCGAGAACATTTTGCATCTGGGCCATAGCTCCAGCTGGTGTTGTGGCTCCGC
CTTGTGAATACGCAAGCATGGGATTTAACCCAGCTTTTTTCATATCTTCAACTGCTCGTTGATATGATGTTCCAGACATTTCAGCTTGAAATCTCTG
TTGATTACTCGCTTGTTCTGCACTCGCTGCGTTTTGACTTTGTGTTCCAAAGTATTGTCCTGCTGCACCTATTGCTGCAGGTGCAAGTGCAGCTAA
GGAGAGTCCCCCAGTAGCAGGGGCAGCTCCTATAGCTATAGCAGGGCCGATTAAATCGGCAATTCCGTCAAATAGTCCCATTAGAAATGGTCTA
TTAAGCCAGGTACTGATACATTGGCATTGGTCTTGCCATTTTGACATCAAAAAATGAGTCAAATAAGAATTGTTGGCCATTTGCAGCTGCTCCAAC
GGCTACTACACGTGATACAGGAGGCGTATCTTGAATAAACGTATTATTCAAAGTAGGCGCAGCGGTAAATTTTTGAGCCAAATGCCAACCATCG
ATCGTGCCCGCCGATGTTGATTTGAACAAACCAGTAATTTTAGATGGTTTGTAACGATATTCCGCCCAGCGTTCTTGATATCCAAAAACAGTATTG
TCGGCTGCAGTATCTCCTGTTGCATAAATTTCTTTTTGCAAAACAGATTGTTCGCCTAGAGTAGCAAATGCTGGGAAATAAAAGTCATATCGTGTA
GATCTAGACCACATACGGTCTAATCCTTGTTGATAAGTAAGATCTGCTCTAATGGATACTAATCCAATAATAACGCCATGCTCAGTAAATGATTGA
GTAAATCCATGATTATGAGCGCGAGCAGCGTACCCATAGCAGCAAGGTTGCCCAAAGGGGTAGTCGTTCCAGAAGCGTTTGTTCCCGACGTTTG
AGCAATCGGATTAACATTAATTGGTGTTGAACCGCCTCCAAGGTATTCAGGCCTTTGTAAACGGGCGTCTGGGGAAATAACTCCAAAGTGACTCC

GGATAATTTCTGTGTATCGTGTTCCGCCACGTGCATCCCTTTCAAGTAATTTTTGAATTTGAAATGACTGTCTAATTGATTGACAGTTGCAGCAGTT
GCTTCTGATAAGTCGGTATATAAATTAGAAACGTTATTTACTACACCAGCAGTATTAACACCATAAGCGTTGCCATATCTAGCTAAAGCAGTCGTA
TTTCCAGGATCTGTTTGAATTACCGTAAATTTATCGTTTGATGTTGCGTCTCCTGAAATGGTATCCCATTTAATTGGAGCCGTAGTACCTAAAGGT
AAGGTGACACTCGCACCTTTTTGTGGCCATGGTAATGCTGACGTAAAGTAATCATGTCTTTTACCACGACGTTTTAGCACATAGTTTGAGGAAGT
ATCAGGGCCATCGCCCTTATCAACTACTGCGCTTGTTTGTAAATTTTCATCTCGGAACCATTCGTTCCAAATAAGATTGTATGCACGTGGCCAAAA
GGCACAGTGCGTAATAGTTCGGCCAGTATCAATTTGGCCTACTGTTGGTAAGCCCATATAGTCTTGAAGGCTGCCTACGGCATAACCATCTGTTG
GGCTTGTTTGAGTTGGGACAATATAAGATATTGAGTCTGTTGGATTTTCTTGTTGACCCATAAATTTTTGCCAGTTATTCCATATAAGGCGATTGG
GTACAAAGAAAAAGAAAGAATCCATAATCATGTTGTCCATGATTGGATATAAAGGCGTTGCTAGACGGGCAAATGCCGTCATTTTTAAGTTGAA
AGTGTCCCCAGGGAGCACTTCATCAACATATACAGGAATTAAATAGCCCGCATCGAAAGTCGTTTTATGTGTTTTTGAGCATCGAATTTACTACGT
GGTATATCGGCTCTAGGTACCATCGCGAAGCGGTGAGTATTAACTGACTGATTGCGGTGCATGTTTTTCCTTAGTGTTGTTCCGGGGGAAAGATA
AATCTCTTTTCCCCTCGGTTGTTTTATTAAGTTTAACTTGTTTTCCTAATGATAGGAGTTTTGGTTGTTCATGTAAATCGAATAACCCAGTTGAATCG
TCAAAAGTTCCGAATTCATATAGATCGAAGTCATCAGGGTGATTAAAAGTTGATTTTCAGTATCAGAACGATTAATTTCATCTGAAAAAGAGCGT
ATAGCTACTCCAGAGGAAGGTACGAACATTGGTCGTGCATATGCTTCAGCAGCACGGTCTTTTACGGAAGCGAGGATAAGTTTCATTATTTTCCT
AAGTGAGGTTACGTTTT

>000098R|arrow

GTGCTGAGTGGTAAAAAATATCGTTCCGACGTATATCCCCATGACTATGTTGTAGTCAACGGAAAAAGGGTAAAACCCCCAAAATACTATGACA
AAAAATATAAATCAGATTATCCATATGAATACGAAGAATTACTCCACAAACGTGAAACTTCTGCTAAACTCAACCACGAAGACAATACCTATGCC
AGACTTGCCGTAAAGGAAAAAGTCACAAAGGCCAAACTTCAATTATTAAAACGTAACCTCACTTAGGAAATCCTCATGAAATTAGTACTCTGTAC
CGTTAAAGACCGCGCAGCAGATGCGTTCGGTCGTCCAATGTTCGTCCGTTCTATCGGCGAAGCAATCCGGAGCTTTAGCGACGAAGTCAATCGC
CAGAGCGATGACAATCAACTTTATAACCATTCCGACGATTTCGACCTATTTGAATTAGGCGAATTCGACGATAATACGGGTTTGTTCCAATTACAT
GAACAACCCAAACTTGTATCCTTAGGGAAACAAGTCAAAATTACTGATAAAAACTAAGCGTAGAGTAAAAAGGGGGAAACCCCTTTTTCTCACG
CAACTAGGCCTAGGAGCTCAAAAAATGCATCGTAACAAGTCGGTAGACGTCCATCAGTTCACAATGATTCCAAAAGCGGATATCCCCCGCTCTAC
ATTTGACTGTCAATCAACACATAAAACTACATTCGATGCTGGCTTCCTAGTCCCTGTACTCGTAGACGAAATGTTGCCAGGCGATACATTCCGCTG
CAACATGACCGCCTTTGCGCGATTGTCTACACCACTCTATCCGATCATGGACAATATGCATTGGATAGCTTCTTCTTTTTGTGCCAAATAGACTTAT
CTGGTCAAATTGGCAAAAATTTATGGGGCAGCAGGCAAATCCTGCGGACTCGATCTCGTACGTAGTGCCCCAACAAGTAACCCCAGCTGGTGGT
TACGCTATTGGCAGCCTTCAAGATTATATGGGTCTGCCAACGTAGGCCAAGTAGGTGCTGGTGGCACCGTAAGTCACTGTGCCTTCTGGCCACGT
GCTTACAACCTTATTTATAACGAATGGTTTCGGGACGAAAACCTTCAAAATTCAGTAGTTGTAGATACTGGCGATGGTCCAGATAACGTAGCCAA
CTACACATTATTACGACGTGGAAAACGTAAAGACTATTTCACGTCAGCATTACCTTGGCCACAAAAGGGCGCAAGCGTTACTTTACCGCTTGGAA
CATCCGCCCCAATATTACGCACTAACAATGCGCCTGTTTCCAGACTGTATAACGCTGGAACAAATACATTAAACGCAACCGCCCAGGCTATTAAC
GTAGGTGTTACTGGTCAAATTACTGGCGGTGCTGACGGCTTGGCAAAATCATATGATCCTAATGGCGGTTTATATGCAGATTTATCAGCTGCAAC
CGCTGCAACAATTAATCAATTGCGTCAAAGCTTCCAGATTCAAAAACTTTTAGAAAGGGACGCCCGTGGCGGAACTCGATACACAGAAATTATCC
GCAGCCATTTCGGGGTCGTTAGCCCCGATGCGCGTCTCCAACGGCCTGAATACATTGGAGGCGGTTCAACACACATTAATATCCAATCGCCCAGA
CGAATGGTACCGGAGCTTCCGGGACCACTACTCCTCTCGGTACACTTGGCGCTATGGGTACTGGGCTCGCTCACAATCATGGCTTTACTTATTCA
AGCACTGAACATGGTGTAATTATCGGTCTCGTTTCAGTACGAGCCGATTTAACATACCAACAAGGTATGCACCGCATGTGGAATCGTTCCACACG
TTATGATTTCTATTTCCCTGCTTTCGCCACTTTGGGCGAACAAGCAGTATTAAATGAAGAAATCTACGTACGAGGCGATGCCAACGATACAGGAG
TGTTTGGATACCAAGAACGTTGGGCAGAATATCGTTATATGCCAAGCCGAATTTCCAGTCTGTTCCGTAGTACGGCAGCTGGAACAATTGACGG
CTGGCATTTAGCCCAACGGTTTACAACACTTCCAACTTTGAATAACACGTTTATTCAAGAAAATCCACCTGTCTCTCGAACCCTTGCGGTCGGAGC
AGCTGCCAACGGCCAGCAAATCATTTTTGATAGCTTTTTTGATATTAAAAAAGCACGGCCAATGCCAATGTACTCTGTACCTGGCTTAATCGACCA
CTTCTAATGGCACTAGAAGCCGCTGCCTCAGGCGCCGCATCTGGCGCCGCTTTTGGACCTTACGGCTCCTTAATTGGAGCCGGAATAGGTGCGG
CCGCTAGTTATTTTGGTGGTCAAGAACAAAACGCTGCCAGCGCACAACAAGCTGCAGCAATGATGCAATTCCAAGATGGTATGCGACGTACTGC
ATATCAAGACGCAGTAGCGGATCTTAAGGCTGCAGGTCTTAACCCTATGCTGGCTTATTCACAAGGCGGAGCCAAAGTCCAGCCTGGTGCGCAA
GCTCCAGTAGGAAATCCACTAGGTGAGGCTGGAAATTCAGCCCGTGAAGCTGCCATGGCAGTCGCCAATTTTAAACAATTACAAACTCAGAATA
TCCTGACACAATCGCAAGCCGAAAAAACGGACGCGGATACAAATCTATCACGTGATCAGGCAACATATACTCGAGCAAATACAGCTCGTGAAAT
TGCTCAGATGCCGGGATACGGCAAATTTGGTCAGCTTCGCGATGCCCAAATAGAGCAATTAAGGACATCAAGTGCATTACAAGCTGCACAACAG
CGACAAGCGTTAAGTCAAAGTGCATATACAGACCAATTAGAGCGATTAGCGCAAACTGGATCAGCGCCATCCAGTACTAAACCAATTTATCAAG
ATGTTAAAGGCTATTTACATAGCCAATATGATAAATATCAAAAATATCTACCATTTGGAAAAATGAAATGAAAACAATCAAACTTAGAACCGCAT
ACAACTATGACACGGATGCTGCGTCAAATGAGTCAGGGTTGGCTTGTGAGGAGCCAACTCTGGCTCAGCAGCATTATAAAGACGAATGCGATAT
AAATACTATCCTGGAACGTTTTAACGTTACAGGCCTATTACCTCAAAGTCCGCTGCCGCCTCAATATGGCGATTTCAGCGGAATTACTGACTATCA
TAGCGCCTTGAATAAGGTAATGAACGCTATGGAAGAATTTGATAACTTACCGGCTCAAATTCGTGCTAGGTTCGAAAACGAACCAGCAAACCTG
ATTGAGTTCTTGCAAGACGAGAAAAATCGACCAGAAGCCGAGAAACTCGGCCTGGTCGAAAGAGCCATTTCGGAAGAAATGGCGATAAGCACA
GTTACTCCACTTGATGTAACTGTGCTAGGTGACACCAACACCAAAAAATATCTGATAAACGAGGCCAAAAATTATGCTTTATAGAAAAAAACAAAC

AAGCGCAAAAGCGCTAAATCGTTCCGTAGGAACACTTCAAAAACTAAAGCTGCAAATATGCAAAAAGCCCCGCAAAGAGGGGGCTGGCGGCTC
TAATAAAGCGCCAGGCTACCTCACATGGCCTGTTATCACCCACTGACTGCTTATTTAAGTAAGCATCAGACAAACTATAAGACCGGCAAATCTTAT
CGCCGTGTCGCATTCAAAGAATCTGACGAGCATGATCGTCAGATTTCACTGCCCTGCGGCCAATGCGTTGGCTGCAGGCTAGAAAAATCACGTC
AATGGGCCATGCGCTGCATGAAGCCCAATTGCACGAAAAAAACTGCTTTATAACCCTCACATACAACAATGAAAACCTTCCACAAACTGGATCGC
TTGTCAAAAGCGACTTCCAAAAGTTCCTTAAGCGCTTCAGAAAATCCATTGCACCTGCAAAATTACGTTACTACATGGCTGGAGAATACGGCACA
AGTTTCGGCAGACCTCACTTCCATGCCTGTATCTTCGGATACGATTTTCATGATAAGAAACTATTCAAAAGGACTCCCTCTGGTTCTCTCATATATA
CATCCGACCACCTTGCAACCCTCTGGCCACATGGTTATTCCTCCATTGGAGACGTTACATTCGAGTCAGCTGCTTACGTTGCTCGATATATTATGC
AAAAATACAACGGCCAGATGGAAGAAAACAAACATATAACAAAGGATGAGCATTACACATACTGTGATATAGAAACAGGGGAATTAATAAAGC
TATTACCAGAATATAACAATATGAGCCTTAAACCAGGCATTG

>000082F|arrow

CACGTGCATCGCGCTCCAATAACTTCTGAATCTGGAAAGATTGACGAAGTTGGTTAATAGTCGCAGCAGTAGCAGTAGACAAATCAGCGTATAA
CTGATTAGTAGCAACCAGCATTTGTACTATTAGACAAAGTATTAGAAGCTGAATTTAATTCTCTTAAAGCACCAACAGCAGTTTGAAAAACACTAT
AACCAGCAGTACCATTGCCAGCTGGTATGTCAGAATAATATTAGCACGTGTTCCTAACGGTAAAGAAACAGAAGCACCCTTCTGTGGCCAAGGC
AATGCACCAGTAAAATAATCTTTACGCTTACCACGTCTCAAAAGTGTATAGTTAGCAGGAACATCACCTGAATCACCAAAGATTTAACGTTACAG
AATTCTGTAAGTTTTCATCTCTAAACCATTCGTTATAAATCTCATTATAAGCACGTAACGGCAAAACATTATGCGTTACTGTATTAGCGCCAGTAAT
CTGACCAGCAGTAGGTAAACCAAAATAATCAAAATTGAACAAACAGCATAACCACCAGCAGGACTAGTAATAGTAGGCACTACAAAAGAAATA
GAATCACCAGGATTCGTTTGTTCACCCATAAACTTTGGCCAATTGTTCCAAAGTAACGATTAGGTACAAAGAAAAAAAGTATCAAGATGCAAGTT
GTCCATCACAGGAAATAACGGTGTGGCCAAACGTGCAAATGCAGTCATCTTTACACGATAGTATCGCCAGGCAATACTTCATCACAATAAATAGG
AACTAAAAACCAGCATCAAACGTGGTTTTATGAGCATATTGAGTATTAAAACTAGAGCGGGGAATGTCCGCTTTAGGAACCATAGCAAATGAAT
GCGTACTTACTGACTTATTGCGAAACATAAACATCTCCCGTAGTTCCGTACCACTCTTTCGAGTGATACGGTATAAAAAAAACCTTACTCGCCTTC
GCGAATCATTACATCTTTTGCACGAGCAATCAACTTAGGGCTTTCCAGTAGTTCAAAAACACCAGTGGCATCGTCAAAAAGACCCAAATAGAACA
TATGAAAATCATCAGGATGTTTATACAACTGATTATCTTCGCTAGCTCGATTGACTTCATCCTGAAACTGACGTACTGCAACACCTTCAGATGCAA
CATAAGCTGGACGACCAAAAGCATCTGCAGCAGTATCCTTAATAGAAACAATAACCATCTTCATAAAAACTCCTTAAATAGTACGTTTTAACAATG
ACAACTTAGCCAACGCAACTTTTTCCTTAACAGCAAGTCGCTCAAGCGTGTTGTCCTCATGCCTAGATCGACCTTCCATCTCTCTGGCAAACTGAA
TCATATCGAATTCTTCAGGAAACTTCAACTTAAATTTATTATCATAAAACCGTGGTGGACGGCACTTTTTGCCACGCACCACAACGTGGTCTGACG
TATAAACGTCTGACATGTACTTATCTAACCACGATTGCCCGATACCGGGCTTCAATGACATCTTATTAAATTCTGGCTTACGCTGAATTATCTCACC
AGTCTCTAAATCACAATATTGATAATGGGCACCCGCATCAACCACTTCGTGGTTTTCATTGACAGTAACCCCATTAATCTTCTTCATAATATATCTT
GCAACATAAGCAGCAGACTCAAAAGTAACATCACCAATTGTAGAATAGCCAAACGGCCACAATTCTTCCAAAATCTCTGACGTGTAGAGGATAG
AGCCAGTCTGCGTTCTTTTAAATATTTTCTTATCCGGAAATCAAGACCAAACAGACAAGCATGGAAATGAGGACGATCAAAAGATTCACCATATT
CACCTGCCATATAAAAACGTATCGTTTTCCCAGTAAAACGCTTACGTAACCGCTTCATAAAAAGCTGATAATCATTGTAATCCAATGACATATCCTT
AGGACAATGCTCTGGAGCATATGTCAAAGTAATAAACAATTACTAGTATGCATTTGTGCCTCATGCATACAACGAATCGCCCACTGACGTGAGCG
TTCAAGGCGACAACCAACACACTGACCACAAGGCAATGATAGGGTACGGACTACATCCGCACCCGGTATTTCCCGCCAAATTATAGACCTGTCAC
TGCATTGATAAGCCGTTAAGGGCTTATAACAGGCCATAATTACAAACGATAGCCACCACGCTGGGGAGCGTGTCTCATATTAATTGACTTCGTCT
TACTAGCAGTTCTGCGAAATGACTTTGCAGATTTATATTTGTTTACTGGCTTTCTTCGTAACATGATGAACTCCGTAGTTAAAATAGTGGTTTGGT
GTCACCTAGCACAGTTACATCAAGTAGAGTAACTGTGCTGGCCTCAGGATTTCATCCTTCGGCCTTAGGTGTTTCTGTAGAAACGATGGGTTCAA
CCACAGGTTGTCCATCAATAAGACCCAATTGAATCGCTTCATCACGATTCTGGTCGTTCTCAAGGAACTCCAATAATTTGACAGGATCATGGTCAA
ATCGGACTCTTAATTTCGCTGGCAGAGCCATGAAATCGTCCATAGTTGCGTTAATTTGATTCAACGCAGAATGGTAATCAGTAACACCACTAAAA
TCGCCGTATTGAGGCGATACAGGGGCCGTTGGAAGTTCCCCTGTAACCCCGAAACGTTCAATGATGACATTAATGTCACATTCATCTTTCATATG
CTGTTGAGCAAGACTTGGGTCTTGACACAGCAACGCATCGTTTACCGATGCTTCATCTTTATCATAGTTGTACGGATTACGTACAAATGGCAAATT
CGCTTTACTCATTATTTGACTCCAATTCCCCAAGGGGTTAGTTGATTAGGATTATTTTTGAGTTTATCAAAAATTTCTTTCGAAGCATCGATACCCC
TAGACCAAATAGCTGGGGATGGAGCCAATAGCCTCTTAGTATTATATGACTGAGCGGATGTTAAATCCGCAGTCGTATTCTTTAAAGCAATATCA
GCCAAAATACGTTTATTTTCGGCTGTAATATTAGGAGCAGTAAGCAACTTATTAACAGTATCAGCCCTAGTATTAGCGGTACCAGCTTCAGTAGCT
TCAGTCTGGGCGATAATCTGCTTTTCTGTTTCTGATGCATTACGAATCTGTTGCATCATAGAAGCAGTATTAATAGCTGAATGTTGCATTACCTAA
AACATTTTCCATAGTAGCAGTTGAACCAGATGGGGTAGAAGCACCACCACGTTGATAGGCTAACATAGGAGATAAACCAGCAGCTTCTAAATCT
TTAACAGCACGCTGGTAACTTGTTCCACTCATATCGGCTTGAAAATCCATTTGCCTCTGAGCCATCTCCTGATTAGCTTTATTAGCAGAAGTAGAA
CCTAAATAAGAACCAACAGCGCCCAAAGCAGTACCGACACCAGGAGTAAAGAAACTAGAAGCTGAAGACAGCTTAGAACCAACATTAGTAACC
GCATCAAGTATTCCACCAAACATAAACTAACGCCCTTCGGTTGTTTCCTCACTACTCCTTACGGAGTAGTCGAGGTTATATAAAACATTAGAAATG
ATCAATTAGACCAGGTACGCTGTACATAGGCATAGGTCTGGCCATCTGACAATCAAAAAGCATCCATTAAAAATTGCTGACCATTTGCTGCAGCT
CCGACCGCCGTAGTACGAGCAACTGGAGGGTCTCTTGAATAAACGTTGAATTCAACGTAGGAAGAGAAGTAAATCGCTGTCCATAATGCCAAGC
ATCAATAGTGCCAGCAGAAGTAGACCTAAAGACCAGTAATTTGAGAAGGTTTGTAACGATACTCCGCCCAACGTTCTTGATAACCAAATACATCA
TTGTCGGCTGCAGTACCTTGAACATAAATTTCTTTGTTCAAAATAGCTTGCTCACCCAAAGTGGCAAATACTGGGAAATAAAAGTCATATCGTGTA

GACCTAGACCACATCTTAGGAAGACCCTGTTGATATGTGAGGTCAGCACGTACAGAAACTAATCCGATAATGTATCCATGTTCTTGAGCAGCATA
CGTAAAGCCGTGTCCCTGAGCCAATGCAGTACCCATTGCAGCAAGGTTACCTTGCGGAGTAGCAGAACCAGTAACCGACGTTGCAGAAGTCTGA
GCAATCGGATTAACATTAACAAGGGTCGAACCTCCACCAATATATTCAGGACGTTGTAAACGATAATCCTGTGGAGTTACTCCAAAGTGAGCACG
TAGTAACTCAGTATAACGAGTACCAC

>000003F|arrow

CGCAGAACTTGAAGCTCTCTGGCCATTTGGTTATACCACCATTGGAGATGTTACTTTTGAATCAGCCGCCTACGTGGCTAGATACATAATGAAAA
AACAAACAGGGAAAGATGCGGAATCTCATTACAAACGCATACACCCTGAAACCGGCGAATATTTAGACTTAAAGCCGGAATATAATAAAATGTC
TTTAAAACCGGGAATCGGTAAAGACTTTTATATAAAATATACTTCGGATATATACCCGCAAGACTACGTAATACTTAGAGGTAAAAAGGTCAAAC
CACCAAAATACTATGACAAAATGTTTAAAATTGACCAACCTTATGAGTATGACGAATTACTTTACATGCGGGAAAATAATGCTAAATTTAATTCCG
AAGACAATACACCAGAACGACTATCTGCAAAAGAACAAGTCACTATGGCAAAACTTCAACTATTAAAACGTAACCTTACTTAGGAAAATAATGAA
ACTTATCCTCGCTTCCGTAAAAGACCGTGCTGCTGAAGCATATGCACGACCAATGTTCGTACCTTCCTCTGGAGTAGCTATACGCTCTTTTTCAGA
TGAAATTAATCGTTCTGATACTGAAAATCAACTCTTTAATCACCCTGATGATTTCGATCTATATGAATTCGGAACATTTGACGATTCAACTGGGTTA
TTCGATTTACATGAACAACCAAAACTCCTATCATTAGGAAAACAAGTTAAACTTAAATAAAACAACCGAGGGGAAAAGAGATTTATCTTTCCCCC
GGAACAACACTAAGGAAAAACATGCACCGCAATCAGTCAGTTAATACTCACCGCTTCGCGATGGTACCTAGAGCCGATATACCACGTAGTAAAT
TCGATGCTCAAAAAACACATAAAACGACTTTCGATGCGGGCTATCTAATTCCTGTATATGTTGATGAAGTGCTCCCTGGGGACACTTTCAACTTAA
AAATGACGGCATTTGCCCGTCTAGCAACGCCTTTATATCCAATCATGGATAACATGATTATGGATTCTTTCTTTTTCTTTGTACCCAATCGCCTTAT
ATGGAATAACTGGCAAAAATTTATGGGTCAACAAGAAAATCCAACAGACTCAATATCTTATATTGTCCCAACACAAACAAGCCCAACAGATGGTT
ATGCCGTAGGCAGCCTTCAAGACTATATGGGCTTACCAACAGTAGGCCAAATTGATACTGGCCGAACTATTACGCACTGTGCCTTTTGGCCACGT
GCATACAATCTTATCTGGAACGAATGGTTCCGAGATGAAAATTTACAAACAAGCGCAGTAGTTGATAAGGGCGATGGCCCTGATACTTCCTCAA
ACTATGTGCTAAAACGTCGTGGTAAAAGACATGATTACTTTACGTCAGCATTACCATGGCCACAAAAAGGTGCGAGTGTCACCTTACCTTTAGGT
ACTACGGCTCCAATTAAATGGGATACCATTTCAGGAGACGCAACATCAAACGATAAATTTACGGTAATTCAAACAGATCCTGGAAATACGACTGC
TTTAGCTAGATATGGCAACGCTTATGGTGTTAATACTGCTGGTGTAGTAAATAACGTTTCTAATTTATATACCGACTTATCAGAAGCAACTGCTGC
AACTGTCAATCAATTAAGACAGTCATTTCAAATTCAAAAATTACTTGAAAGGGATGCACGTGGCGGAACACGATACACAGAAATTATCCGGAGT
CACTTTGGAGTTATTTCCCCAGACGCCCGTTTACAAAGGCCTGAATACCTTGGAGGCGGTTCAACACCAATTAATGTTAATCCGATTGCTCAAACG
TCGGGAACAAACGCTTCTGGAACGACTACCCCTTTGGGCAACCTTGCTGCTATGGGTACTGCTCTCGCTCATAATCATGGATTTACTCAATCATTT
ACTGAGCATGGCGTTATTATTGGATTAGTATCCATTAGAGCAGATCTTACTTATCAACAAGGATTAGACCGTATGTGGTCTAGATCTACACGATAT
GACTTTTATTTCCCAGCATTTGCTACTCTAGGCGAACAATCTGTTTTGCAAAAAGAAATTTATGCAACAGGAGATACTGCAGCCGACAATACTGTT
TTTGGATATCAAGAACGCTGGGCGGAATATCGTTACAAACCATCTAAAATTACTGGTTTGTTCAAATCAACATCGGCGGGCACGATCGATGGTTG
GCATTTGGCTCAAAAATTTACCGCTGCGCCTACTTTGAATAATACGTTTATTCAAGATACGCCTCCTGTATCACGTGTAGTAGCCGTTGGAGCAGC
TGCAAATGGCCAACAATTCTTATTTGACTCATTTTTTGATGTCAAAATGGCAAGACCAATGCCAATGTATTCAGTACCTGGCTTAATAGACCATTT
CTAATGGGACTATTTGACGGAATTGCCGATTTAATCGGCCCTGCTATAGCTATAGGAGCTGCCCCTGCTACTGGGGGACTCTCCTTAGCTGCACT
TGCACCTGCAGCAATAGGTGCAGCAGGACAATACTTTGGAACACAAAGTCAAAACGCAGCGAGTGCAGAACAAGCGAGTAATCAACAGAGATT
TCAAGCTGAAATGTCTGGAACATCATATCAACGAGCAGTTGAAGATATGAAAAAAGCTGGGTTAAATCCCATGCTTGCGTATTCACAAGGCGGA
GCCACAACACCAGCTGGAGCTATGGCCCAGATGCAAAATGTTCTCGGTAATGCAACTACGTCCGGAACCCAAGCTTATCAAACGGTTGCGCAAG
CAAATCAAGCTATTGCTCAATCTAAACAAATTGAAGCTCAAACAGAACTCACAAGTAATCAAACAGATAATGTACGTGCTGATACGTTAAATAAA
TTGGATGAAAATCCAAATATTAGAGCTCAATATAAACAAATACTTGCCGATACTTTCATGAAAAATGAAATAGGCAAAACATCAAGTGCTCAAGC
TGCTCAAGCTTTGGCACAATCTCGTTATTCAAACGAGTTAACAAAACTTGCTAAATCAGGGTCAGCTCCTAGTTCTAGCAAACCAATTTATCAAGA
CGTAAAAAACATCGCCAAAGATGCGTATAGCGCATCTGGCGCAAAACGATACATCGATAACTATCGAGGTCAACCGATTCAACAAAATCGTACA
AATAACCAACCACCAATGGAATGAAAATGACAAAGATTACAGCCCCATTTCTTCGTACTCCGTACAATTACGACACGATTGCTGCGTCAAATGAG
TCAGGGCTGCATTGTGAGGATGCAACTCTGACTCAGCAGCAATTTGCTGAAGAATGTGATATCAATAATATTATGGAAAAGTTCGGTATGACCG
GACTTATTCCTCAAACTCCTTTAACGCCTCAATATGGCGACTTTAGTGGTGTCTATGACTACCACTCTGCTCTGAACCAGATTATGGCTTCAGACAA
CGAATTTATGGCTTTACCAGCCAATATTCGTGAACGATTCGCTAATGATCCCGCGAATCTAATAGATTTTCTAGAAAACCCTGAAAATCGCAGCG
AAGCTGAAAAAATGGGACTGGTAAAACCAGCCCAAACCGAGGTTTCAACCCCTGTTGGAACCTCGGAAGCACAGTTACCTACTTGATGTAACTG
TGCTAGGTGACACCAAACCACAAAAACACGATAAACAAGGACAGAAAAAAATGATGCGTCGCAGACCAGCAAATAAGCAAAAGTCCGCTAGGAC
TTTCCGTAAACATGCTTCACATACAAAACACGCAAATATGCGAAACTCGCCAATGCGTGGAGGCTGGAGACTCTAATAAAGTCTTCAGGCACCTC
ACATGCCTTGTTATCACCCTCTCAAAGCATATCAATGCTTTGACAAATCAATTGTTTTCGACGAAGTTCGGAAACATGACATCGTTCGATCTTTAG
ACCTGCCCTGTGGGCAGTGCGTTGGATGCCGTCTAGAACGATCAAGACAATGGGCTATTCGGTGCATGCACGAAGCCCAATTGCATAAAAACAA
CTCATTCATAACACTCACATATGACAATACACATCTCCCAAGCGATGGCTCTTTGGATCACAAAGACTTTCAATTGTTCCTTAAAAGACTTAGAAA
AACTCTCGCAAAAAGAGGACTTACAATCCGCTATTACATGGCTGGAGAATATGGTGAACTCTTCGCAAGACCCCACTTCCATGCCTGTATCTTCG
GATACGACTTTCCTGATAAAAAATTATGGAAAAGGACTGCCTCTGGTTCTATGTTATATAGATC

>000127F|arrow

TTTTCCGTTGACTACAACTAGTCATGGGGATATACGTCGGAACGATATTTTTTGTACCACTCAGCACCAATGCCTGGTTTAAGGCTCATATTGTTA
TATTCTGGTAATAGCTTTATTAATTCCCCTGTTTCTATATCACAGTATGTGTAATGCTCATCCTTTGTTATATGTTTGTTTTCTTCCATCTGGCCGTTG
TATTTTTGCATAATATATCGAGCAACGTAAGCAGCTGACTCGAATGTAACGTCTCCAATGGAGGAATAACCATGTGGCCAGAGGGTTGCAAGGT
GGTCGGATGTATATATGAGAGAACCAGAGGGAGTCCTTTTGAATAGTTTCTTATCATGAAAATCGTATCCGAAGATACAGGCATGGAAGTGAGG
TCTGCCGAAACTTGTGCCGTATTCTCCAGCCATGTAGTAACGTAATTTTGCAGGTGCAATGGATTTTCTGAAGCGCTTAAGGAACTTTTGGAAGT
CGCTTTTGACAAGCGATCCAGTTTGTGGAAGGTTTTCATTGTTGTATGTGGGGTTATAAAGCAGTTTTTTTCGTGCAATTGGGCTTCATGCATGCA
GCGCATGGCCCATTGACGTGATTTTTCTAGCCTGCAGCCAACGCATTGGCCGCAGGGCAGTGAAATCTGACGATCATGCTCGTCAGATTCTTTGA
ATGCGACACGGCGATAAGATTTGCCGGTCTTATAGTTTGTCTGATGCTTACTTAAATAAGCAGTCAGTGGGTGATAACAGGCCATGTGAGGTAG
CCTGGCGCTTTATTAGAGCCGCCAGCCCCCTCTTTGCGGGGCTTTTTGCATATTTGCAGCTTTAGTTTTTGAAGTGTTCCTACGGAACGATTTAGC
GCTTTTGCGCTTGTTTGTTTGTTTTCTATAAAGCATAATTTTTGGCCTCGTTTATCAGATATTTTTGGTGTTGGTGTCACCTAGCACAGTTACATCAA
GTGGAGTAACTGTGCTTATCGCCATTTCTTCCGAAATGGCTCTTTCGACCAGGCCGAGTTTCTCGGCTTCTGGTCGATTTTTCTCGTCTTGCAAGA
ACTCAATCAGGTTTGCTGGTTCGTTTTCGAACCTAGCACGAATTTGAGCCGGTAAGTTATCAAATTCTTCCATAGCGTTCATTACCTTATTCAAGGC
GCTATGATAGTCAGTAATTCCGCTGAAATCGCCATATTGAGGCGGCAGCGGACTTTGAGGTAATAGGCCTGTAACGTTAAAACGTTCCAGGATA
GTATTTATATCGCATTCGTCTTTATAATGCTGCTGAGCCAGAGTTGGCTCCTCACAAGCCAACCCTGACTCATTTGACGCAGCATCCGTGTCATAG
TTGTATGCGGTTCTAAGTTTGATTTTTCATTTCATTTTTCCAAATGGTAGATATTTTTGATATTTATCATATTGGCTATGTAAATAGCCTTTAACATC
TTGATAAATTGGTTTAGTACTGGATGGCGCTGATCCAGTTTGCGCTAATCGCTCTAATTGGTCTGTATATGCACTTTGACTTAACGCTTGTCGCTG
TTGTGCAGCTTGTAATGCACTTGATGTCCTTAATTGCTCTATTTGGGCATCGCGAAGCTGACCAAATTTGCCGTATCCCGGCATCTGAGCAATTTC
ACGAGCTGTATTTGCTCGAGTATATGTTGCCTGATCACGTGATAGATTTGTATCCGCGTCCGTTTTTCGGCTTGCGATTGTGTCAGGATATTCTGA
GTTTGTAATTGTTTAAAATTGGCGACTGCCATGGCAGCTTCACGGGCTGAATTTCCAGCCTCACCTAGTGGATTTCCTACTGGAGCTTGCGCACCA
GGCTGGACTTTGGCTCCGCCTTGTGAATAAGCCAGCATAGGGTTAAGACCTGCAGCCTTAAGATCCGCTACTGCGTCTTGATATGCAGTACGTCG
CATACCATCTTGGAATTGCATCATTGCTGCAGCTTGTTGTGCGCTGGCAGCGTTTTGTTCTTGACCACCAAAATAACTAGCGGCCGCACCTATTCC
GGCTCCAATTAAGGAGCCGTAAGGTCCAAAAGCGGCGCCAGATGCGGCGCCTGAGGCAGCGGCTTCTAGTGCCATTAGAAGTGGTCGATTAAG
CCAGGTACAGAGTACATTGGCATTGGCCGTGCTTTTTTAATATCAAAAAAGCTATCAAAATGATTTGCTGGCCGTTGGCAGCTGCTCCGACCGCA
AGGGTTCGAGAGACAGGTGGATTTTCTTGAATAAACGTGTTATTCAAAGTTGGAAGTGTTGTAAACCGTTGGGCTAAATGCCAGCCGTCAATTG
TTCCAGCTGCCGTACTACGGAACAGACTGGAAATTCGGCTTGGCATATAACGATATTCTGCCCAACGTTCTTGGTATCCAAACACTCCTGTATCGT
TGGCATCGCCTCGTACGTAGATTTCTTCATTTAATACTGCTTGTTCGCCCAAAGTGGCGAAAGCAGGGAAATAGAAATCATAACGTGTGGAACGA
TTCCACATGCGGTGCATACCTTGTTGGTATGTTAAATCGGCTCGTACTGAAACGAGACCGATAATTACACCATGTTCAGTGCTTGAATAAGTAAA
GCCATGATTGTGAGCGAGCCCAGTACCCATAGCGCCAAGTGTACCGAGAGGAGTAGTGGTCCCGGAAGCTCCGGTACCATTCGTCTGGGCGAT
TGGATTGATATTAATGTGTGTTGAACCGCCTCCAATGTATTCAGGCCGTTGGAGACGCGCATCGGGGCTAACGACCCCGAAATGGCTGCGGATA
ATTTCTGTGTATCGAGTTCCGCCACGGGCGTCCCTTTCTAAAAGTTTTTGAATCTGGAAGCTTTGACGCAATTGATTAATTGTTGCAGCGGTTGCA
GCTGATAAATCTGCATATAAACCGCCATTAGGATCATATGATTTTGCCAAGCCGTCAGCACCGCCAGTAATTTGACCAGTAACACCTACGTTAATA
GCCTGGGCGGTTGCGTTTAATGTATTTGTTCCAGCGTTATACAGTCTGGAAACAGGCGCATTGTTAGTGCGTATATTGGGGCGGATGTTCCAAGC
GGTAAAGTAACGCTTGCGCCCTTTTGTGGCCAAGGTAATGCTGACGTGAAATAGTCTTTACGTTTTCCACGTCGTAATAATGTGTAGTTGGCTAC
GTTATCTGGACCATCGCCAGTATCTACAACTACTGAATTTTGAAGGTTTTCGTCCCGAAACCATTCGTTATAAATAAGGTTGTAAGCACGTGGCCA
GAAGGCACAGTGACTTACGGTGCCACCAGCACCTACTTGGCCTACGGTTGGCAGACCCATATAATCTTGAAGGCTGCCAATAGCGTAACCACCA
GCTGGGGTTACTTGTTGGGGCACTACGTACGAGATCGAGTCCGCAGGATTTGCCTGCTGCCCCATAAATTTTTGCCAATTTGACCAGATAAGTCT
ATTTGGCACAAAAAGAAGAAGCTATCCAAATGCATGTATCCATGATCGGATAGAGTGGTGTAGACAATCGCGCAAAGGCGGTCATGTTGCAGC
GGAATGTATCGCCTGGCAACATTTCGTCTACGAGTACAGGGACTAGGAAGCCAGCATCGAATGTAGTTTTATGTGTTGATTGACAGTCAAATGT
AGAGCGGGGGATATCCGCTTTTGGAATCATTGTGAACTGATGGACGTCTACCGACTTGTTACGATGCATTTTTTTGAGCTCCTAGGCCTAGTTGC
GTGAGAAAAAGGGGTTTCCCCCTTTTTACTCTACGCTTAGTTTTTATCAGTAATTTTGACTTGTTTCCCTAAGGATACAAGTTTGGGTTGTTCATGT
AATTGGAACAAACCCGTATTATCGTCGAATTCGCCTAATTCAAATAGGTCGAAATCGTCGGAATGGTTATAAAGTTGATTGTCATCGCTCTGGCG
ATTGACTTCGTCGCTAAAGCTCCGGATTGCTTCGCCGATAGAACGGACGAACATTGGACGACCGAACGCATCTGCTGCGCGGTCTTTAACGGTA
CAGAGTACTAATTTCATGAGGATTTCCTAAGTGAGGTTACGTTTTAATAATTGAAGTTTGGCCTTTGTGACTTTTTCCTTTACGGCAAGTCTGGCA
TAGGTATTGTCTTCGTGGTTGAGTTTAGCAGAAGTTTCACGTTTGTGGAGTAATTCTTCGTATTCATATGGATAATCTGATTTTATTTTTTGTCATA
GTATTTTGGGGGTTTTACCCT

>000196F|arrow

CAAGAAGACTGTTACTGTTGATTTAGAAGCAGAATTACCTGTTAGTGGAGAGGTTAACCTCTCCACTAACAGGTAATTCTGCTTCTAAATCAACA
GTA

>000114F|arrow

GTAGGAAGAGAAGTAAATCGCTGTCCATAATGCCAAGCATCAATAGTGCCAGCAGAAGTAGACCTAAAGAAACCAGTAATTTGAGAAGGTTTG
TAACGATACTCCGCCCAACGTTCTTGATAACCAAATACATCATTGTCGGCTGCAGTACCTTGAACATAAATTTCTTTGTTCAAAATAGCTTGCTCAC
CCAAAGTGGCAAATACTGGGAAATAAAAGTCATATCGTGTAGACCTAGACCACATCTTAGGAAGACCCTGTTGATATGTGAGGTCAGCACGTAC
AGAAACTAATCCGATAATGTATCCATGTTCTTGAGCAGCATACGTAAAGCCGTGTCCCTGAGCCAATGCAGTACCCATTGCAGCAAGGTTACCTT
GCGGAGTAGCAGAACCAGTAACCGACGTTGCAGAAGTCTGAGCAATCGGATTAACATTAACAAGGGTCGAACCTCCACCAATATATTCAGGACG
TTGTAAACGATAATCCTGTGGAGTTACTCCAAAGTGAGCACGTAGTAACTCAGTATAACGAGTACCACCACGTGCATCGCGCTCCAATAACTTCT
GAATCTGGAAAGATTGACGAAGTTGGTTAATAGTCGCAGCAGTAGCAGTAGACAAATCAGCGTATAACTGATTAGTAGCAACACCAGCATTTGT
ACTATTAGACAAAGTATTAGAAGCTGAATTTAATTCTCTTAAAGCACCAACAGCAGTTTGAAAAAACACTATAACCAGCAGTACCATTGCCAGCT
GGTATGTCAGAATAAATATTAGCACGTGTTCCTAACGGTAAAGAAACAGAAGCACCCTTCTGTGGCCAAGGCAATGCACCAGTAAAATAATCTTT
ACGCTTACCACGTCTCAAAAGTGTATAGTTAGCAGGAACATCACCTGAATCACCAAGATTTAACGTTACAGAATTCTGTAAGTTTTCATCTCTAAA
CCATTCGTTATAAATCTCATTATAAGCACGTAACGGCAAAACATTATGCGTTACTGTATTAGCGCCAGTAATCTGACCAGCAGTAGGTAAACCAA
AATAATCAAAAATTGAACAAACAGCATAACCACCAGCAGGACTAGTAATAGTAGGCACTACAAAGAAATAGAATCACCAGGATTCGTTTGTTCA
CCCATAAACTTTGGCCAATTGTTCCAAAGTAAACGATTAGGTACAAAGAAAAAGAAAGTATCAAGATGCAAGTTGTCCATCACAGGAAATAACG
GTTGTGGCCAAACGTGCAAATGCAGTCATCTTTACACGATGAGTATCGCCAGGCAATACTTCATCACAATAAATAGGAACTAAAAAACCAGCATC
AAAACGTGGTTTTATGAGCATATTGAGTATTAAAACTAGAGCGGGGAATGTCCGCTTTAGGAACCATAGCAAATGAATGCGTACTTACTGACTTA
TTGCGAAACATAAACATCTCCCGTAGTTCCGTACCACTCTTTCGAGTGATACGGTATAAAAAAAACCTTACTCGCCTTCGCGAATCATTACATCTTT
TGCACGAGCAATCAACTTAGGGCTTTCCAGTAGTTCAAAAACACCAGTGGCATCGTCAAAAGACCCAAATAGAACATATGAAAATCATCAGGAT
GTTTATACAACTGATTATCTTCGCTAGCTCGATTGACTTCATCCTGAAACTGACGTACTGCAACACCTTCAGATGCAACATAAGCTGGACGACCAA
AAGCATCTGCAGCAGTATCCTTAATAGAAACAATAACCATCTTCATAAAAACTCCTTAAATAGTACGTTTTAACAATGACAACTTAGCCAACGCAA
CTTTTTCCTTAACAGCAAGTCGCTCAAGCGTGTTGGTCCTCATGCCTAGATCGACCTTCCATCTCTCTGGCAAACTGAATCATATCGAACTTCTTCA
GGAAACTTCAACTTAAATTTATTATCATAAAACCGTGGTGGACGGCACTTTTTGCCACGCACCACAACGTGGTCTGACGTATAAACGTCTGACAT
GTACTTATCTAACACGATTGCCCGATACCGGGCTTCAATGACATCTTATTAAATTCTGGCTTACGCTGAATTATCTCACCAGTCTCTAAATCACAAT
ATTGATAATGGGCACCCGCATCAACCACTTCGTGGTTTTCATTGACAGTAAACCCCATAATCTTCTTCATAATATATCTTGCAACATAAGCAGCAG
ACTCAAAAGTAACATCACCAATTGTAGAATAGCAAACGGCCACAATTATTCCAAAATCTCTGACGTGTAGAGGATAGAGCCAGTCTGCGTTCTTT
TAATATTTTCTTATCCGGAAAATCAAGACCAAACAGACAAGCATGGAAATGAGGACGATCAAAAGATTCACCATATTCACCTGCCATATCAAAAA
CGTATCGTTTTCCAGTAAAACGCTTACGTAACCGCTTCATAAAAAGCTGATAATCTTGTAATCCAATGACATATCCTTAGGACAACTGCTCTGGAG
CATATGTCAAAGTAATAAAACAATTACTAGTATGCATTTGTGCCTCATGCATACAACGAATCGCCCACTGACGTGAGCGTTCAAGCGACAACCAA
CACACTGACCACAAGGCAATGATAGGGTACGGACTACATCCGCACCCGGTATTTCCCGCCAAATTATAGACCTGTCACTGCATTGATAAGCCGTT
AAGGGCTTATAACAGGCCATAATTACAAACGATAGCCACCACGCTGGGGAGCGTGTCTCATATTAATTGACTTCGTCTTACTAGCAGTTCTGCGA
AATGACTTTGCAGATTTATATTTGTTTACTGGCTTTCTTCGTAACATGATGAACTCCGTAGTTAAAATAGTGGTTTGGTGTCACCTAGCACAGTTAC
ATCAAGTAGAGTAACTGTGCTGGCCTCAGGATTTCATCCTTCGGCCTTAGGTGTTTCTGTAGAAACGATGGGTTCAACCACAGGTTGTCCATCAA
TAAGACCCAATTGAATCGCTTCATCACGATTCTGGTCGTTCTCAAGGAACTCCAATAATTTGACAGGATCATGGTCAAATCGGACTCTTAATTTCG
CTGGCAGAGCCATGAAATCGTCCATAGTTGCGTTAATTTGATTCAACGCAGAATGGTAATCAGTAACACCACTAAAATCGCCGTATTGAGGCGAT
ACAGGGGCCGTTGGAAGTTCCCCTGTAACCCCGAAACGTTCAATGATGACATTAATGTCACATTCATCTTTCATATGCTGTTGAGCAAGACTTGG
GTCTTGACACAGCAACGCATCGTTTACCGATGCTTCATCTTTATCATAGTTGTACGGATTACGTACAAATGGCAAATTCGCTTTACTCATTATTTGA
CTCCAATTCCCCAAGGGGTTAGTTGATTAGGATTATTTTTGAGTTATCAAAAATTTCTTTCGAAGCATCGATACCCCTAGACCAAATAGCTGGGGA
TGGAGCCAATAGCTCTTAGTAGTATTATAGACTGAGCGGATGTTAAATCCGCAGTCGTCTTTAAAGCAATATCAGCCAAAATACGTTTATTTTCG
GCTGTAATATTAGGAGCAGTAAGCAACTTATTAACAGTATCAGCCCTAGTTAGCGGTACCAGCTTCAGTAGCTTCAGTCTGGGCGATAATCTGCT
TTTCTGTTTCTGATGCATTACGAATCTGTTGCATCATAGAAGCAGTATTAATAGCTGAATTAGTTGCATTACCTAAAACATTTTCCATAGTAGCAGT
TGAACCAGATGGGGTAGAAGCACCACCACGTTGATAGGCTAACATAGGAGATAAACCAGCAGCTTCTAAATCTTTAACAGCACGCTGGTAACTT
GTTCCACTCATATCGGCTTGAAAATCCATTTGCCTCTGAGCCATCTCCTGATTAGCTTTATTAGCAGAAGTAGAACCTAAATAAGAACCAACAGCG
CCCAAAGCAGTACCGACACCAGGAGTAAAGAAACTAGAAGCTGAAGACAGCTTAGAACCAACATTAGTAACCGCATCAAGTATTCCACCAAACA
TAAACTAACGCCCTTCGGTTGTTTCCTCACTACTCCTTACGGAGTAGTCGAGGTTATATAAAACATTAGAAATGATCAATTAGACCAGGTACGCTG
TACATAGGCATAGGTCTGGCCATCTGACAATCAAAGAAAGCATCCAAAAATTGCTGACCATTTGCTGCAGCTCCGACCGCCGTAGTACGAGCAA
CTGGAGGGGTCTCTTGAATAAACGTTGAATTCAAC

>000149F|arrow

CACCACTAAAGTCGCCATATTGAGGCGTTAAAGGAGTTTGAGGAATAAGTCCGGTCATACCGAACTTTTCCATAATATTATTGATATCACATTCTT
CAGCAAATTGCTGCTGAGTCAGAGTTGCATCCTCACAATGCAGCCCTGACTCATTTGACGCAGCAATCGTGTCGTAATTGTACGGAGTACGAAGA
AATGGGGCTGTAATCTTTGTCATTTTCATTCCATTGGTGGTTGGTTATTTGTACGATTTTGTTGAATCGGTTGACCTCGATAGTTATCGATGTATCG
TTTTGCGCCAGATGCGCTATACGCATCTTTGGCGATGTTTTTTACGTCTTGATAAATTGGTTTGCTAGAACTAGGAGCTGACCCTGATTTAGCAAG
TTTTGTTAACTCGTTTGAATAACGAGATTGTGCCAAAGCTTGAGCAGCTTGAGCACTTGATGTTTTGCCTATTTCATTTTCATGAAAGTATCGGCA

AGTATTTGTTTATATTGAGCTCTAATATTTGGATTTTCATCCAATTTATTTAACGTATCAGCACGTACATTATCTGTTTGATTACTTGTGAGTTCTGT
TTGAGCTTCAATTTGTTTAGATTGAGCAATAGCTTGATTTGCTTGCGCAACCGTTTGATAAGCTTGGGTTCCGGACGTAGTTGCATTACCGAGAAC
ATTTTGCATCTGGGCCATAGCTCCAGCTGGTGTTGTGGCTCCGCCTTGTGAATACGCAAGCATGGGATTTAACCCAGCTTTTTTCATATCTTCAAC
TGCTCGTTGATATGATGTTCCAGACATTTCAGCTTGAAATCTCTGTTGATTACTCGCTGTTCTGCACTCGCTGCGTTTTGACTTTGTGTTCCAAAGT
ATTGTCCTGCTGCACCTATTGCTGCAGGTGCAAGTGCAGCTAAGGAGAGTCCCCCAGTAGCAGGGGCAGCTCCTATAGCTATAGCAG GGCCGAT
TAAATCGGCAATTCCGTCAAATAGTCCCATTAGAAATGGTCTATTAAGCCAGGTACTGAATACATTGGCATTGGTCTTGCCATTTTGACATCAAAA
AATGAGTCAAATAAGAATTGTTGGCCATTTGCAGCTGCTCCAACGGCTACTACACGTGATACAGGAGGCGTATCTTGAATAAACGTATTATTCAA
AGTAGGCGCAGCGGTAAATTTTTGAGCCAAATGCCAACCATCGATCGTGCCCGCCGATGTTGATTTGAACAAACCAGTAATTTTAGATGGTTTGT
AACGATATTCCGCCCAGCGTTCTTGATATCCAAAAACAGTATTGTCGGCTGCAGTATCTCCTGTTGCATAAATTTCTTTTTGCAAAACAGATTGTTC
GCCTAGAGTAGCAAATGCTGGGAAATAAAAGTCATATCGTGTAGATCTAGACCACATACGGTCTAATCCTTGTTGATAAGTAAGATCTGCTCTAA
TGGATACTAATCCAATAATAACGCCATGCTCAGTAAATGATTGAGTAAATCCATGATTATGAGCGAGAGCAGTACCCATAGCAGCAAGGTTGCC
CAAAGGGGTAGTCGTTCCAGAAGCGTTTGTTCCCGACGTTTGAGCAATCGGATTAACATTAATTGGTGTTGAACCGCCTCCAAGGTATTCAGGCC
TTTGTAAACGGGCGTCTGGGGAAATAACTCCAAAGTGACTCCGGATAATTTCTGTGTATCGTGTTCCGCCACGTGCATCCCTTTCAAGTAATTTTG
AATTTGAAATGACTGTCTTAATTGATTGACAGTTGCAGCAGTTGCTTCTGATAAGTCGGTATATAAATTAGAAACGTTATTTACTACACCAGCAGT
ATTAACACCATAAGCGTTGCCATATCTAGCTAAAGCAGTCGTATTTCCAGGATCTGTTTGAATTACCGTAAATTTATCGTTTGATGTTGCGTCTCCT
GAAATGGTATCCCATTTAATTGGAGCCGTAGTACCTAAAGGTAAGGTGACACTCGCACCTTTTTGTGGCCATGGTAATGCTGACGTAAAGTAATC
ATGTCTTTTACCACGACGTTTTAGCACATAGTTTGAGGAAGTATCAGGGCCATCGCCCTTATCAACTACTGCGCTTGTTTGTAAATTTTCATCTCGG
AACCATTCGTTCCAGATAAGATTGTATGCACGTGGCCAAAAGGCACAGTGCGTAATAGTTCGGCCAGTATCAATTTGGCCTACTGTTGGTAAGCC
CATATAGTCTTGAAGGCTGCCTACGGCATAACCATCTGTTGGGCTTGTTTGTGTTGGGACAATATAAGATATTGAGTCTGTTGGATTTTCTTGTTG
ACCCATAAATTTTTGCCAGTTATTCCATATAAGGCGATTGGGTACAAAGAAAAAGAAAGAATCCATAATCATGTTATCCATGATTGGATATAAAG
GCGTTGCTAGACGGGCAAATGCCGTCATTTTTAAGTTGAAAGTGTCCCCAGGGAGCACTTCATCAACATATACAGGAATTAGATAGCCCGCATC
GAAAGTCGTTTTATGTGTTTTTTGAGCATCGAATTTACTACGTGGTATATCGGCTCTAGGTACCATCGCGAAGCGGTGAGTATTAACTGACTGATT
GCGGTGCATGTTTTTCCTTAGTGTTGTTCCGGGGGAAAGATAAATCTCTTTTCCCTCGGTTGTTTTATTTAAGTTTAACTTGTTTTCCTAATGATAG
GAGTTTTGGTTGTTCATGTAAATCGAATAACCCAGTTGAATCGTCAAATGTTCCGAATTCATATAGATCGAATCATCAGGGTGATTAAAGAGTTG
ATTTTCAGTATCAGAACGATTAATTTCATCTGAAAAAGAGCGTATAGCTACTCCAGAGGAAGGTACGAACATTGGTCGTGCATATGCTTCAGCAG
CACGGTCTTTTACGGAAGCGAGGATAAGTTTCATTTTCCTAAGAGGTTACGTTTTAATAGTTGAAGTTTTGCCATAGTGACTTGTTCTTTTGCAGA
TAGTCGTTCTGGTGTATTGTCTTCGGAATTAAATTTAGCATTATTTTCCCGCATGTAAAGTAATTCGTCATACTCATAAGGTTGGTCAATTTTAAAC
ATTTTGTCATAGTATTTTGGTGGTTTGACCTTTTTACCTCTAAGTATTACGTAGTCTTGCGGGTATATATCCGAAGTATTTTATATAAAAGTCTTTAC
CGATTCCCGGTTTTAAAGACATTTTATTATATTCCGGCTTTAAGTCTAAATATTCGCCGGTTTCAGGGTGTATGCGTTTGTAATGAGATTCCGCATC
TTTCCCTGTTTGTTTTTTCATTATGTATCTAGCCACGTAGGCGGCTGATTCAAAAGTAACATCTCCAATGGTGGTATAACCAAATGGCCAGAGAGC
TTCAAGTTCTGCGGATCTATATAACATAGAACCAGAGGCAGTCCTTTTCCATAATTTTTATCAGGAAAGTCGTATCCGAAGATACAGGCATGGAA
GTGGGGTCTTGCGAAGAGTTCACCATATTCTCCAGCCATGTAATAGCGGATTGTAAGTCCTCTTTTTGCGAGAGTTTTTCTAAGTCTTTTAAGGAA
CAATTGAAAGTCTTTGTGATCCAAAGAGCCATCGCTTGGGAGATGTGTATTGTCATATGTGAGTGTTATGAATGAGTTGTTTTTATGCAATTGGG
CTTCGTGCATGCACCGAATAGCCCATTGTCTTGATCGTTCTAGACGGCATCCAACGCACTGCCCACAGGGCAGGTCTAAAGATCGAACGATGTCA
TGTTTCCGAACTTCGTCGAAAACAATTGATTTGTCAAAGCATTGATATGCTTTGAGAGGGTGATAACAAGGCATGTGAGGTGCCTGAAGACTTTA
TTAGAGTCTCCAGCCTCCACGCATTGGCGAGTTTCGCATATTTGCGTGTTTTGTATGTGAAGCATGTTTACGGAAAGTCCTAGCGGACTTTTGCTT
ATTTGCTGGTCTGCGACGCATCATTTTTTCTGTCCTTGTTTATCGTGTTTTTGTGGTTTGGTGTCACCTAGCACAGTTACATCAAGTAGGTAACTGT
GCTTCCGAGGTTCCAACAGGGGTTGAAACCTCGGTTTGGGCTGGTTTTACCAGTCCCATTTTTTTCAGCTTCGCCTGCGATTTCAGGGTTTTCTAG
AAAATCTATTAGATTCGCGGGCTCATTAGCGAATCGTTCACGAATATTGGCTGGTAAAGCCATAAATTCGTTGTCTGAAGCCATAATCTGGTTCA
GAGCAGAGTGGTAGTCATAGA

>000198F|arrow

TCCGAAGATACAGGCATGGAAGTGGGGTCTTGCGAAGAGTTCAATATTCTCCAGCCATGTAATAGCGGATTGTAAGTCCTCTTTTTGCGAGAGTT
TTTCTAAGTCTTTTAAGGAACAATTGAAAGTCTTGTGATCCAAAGAGCCATCGCTTGGGAGATGTGTATTGTCATATGTGAGTGTTATGAATGAG
TTGTTTTATGCAATTGGGCTTCGTGCATGCCAACCTGATATGCCCCATTGTCTTGATCGTTCTAGACGGCATCCAACGCACTGGCCCACAGGGCA
GGTTCTAAAGATCGAACGATGTCATGTTTGTCCGGAATTTCGTCGAGAACAATTGATTTGTCAAGCCTTGCTGATATGCTTTGTGATGATGTGGG
TTGATACCAATGGCATGGGTGAGGGTGCCTGCAGACTTGTATTAGGAGTCTCACGCCTCCTACGCATTGGCGAGCTTTTCGCATATTTGCGGTTT
GTTTTGTATGTGAGCAGTTTACGGAATAGTACCTGTAGCGGACTTTGCTTATGTGGCTGGTCTGCGACGCATCATTTTTCTCTGTCTCTTGTTTATC
GTTGTTTTTGTGTGTTTTGGTGTCACTAGCCACCGTTGACATCAAGGTAGGTAACTGTTTCTTCGCGGCTTTCCAACAGGTGTTGGAACCTCGGTT
TGGGGTTGTATTTGACAAGTCCCATTTTTTTCAGCTTCTTCGCTGCGATTTTCAGGGTTTTCTAGAAAATCTATTAGATTCGCGGGATCATTAGCGA
ATCGTTCACGAATATGGTGGTACAGCCATAACTTCGTTGTCTGAAGCCCTCAATCGTGCGTTCAGAGCAGAAGTGGTCGTCATAGACACCACTAA
AAGTCGACCATAATTGAGGCGTTAAAGGAGTTTGAGGAATAAGTCCGGTCATACCGAACTTTTCCATAATATTATTGATATCACATTCTTCAGCA

AATTGCGCTGAGTCAGAGTTGCATCCTCACAATGCAGCCCTGACTCATTTGACGCAGCAATCGTGTCGTAATTGTACGAGTACGAAGAATGGGG
CTGTAATCTTGTCATTTCATTCCATTGGTGGTTGGTTATTTGTACGATTTTGTTTTGAATCGGTGACCTCGATAGTTATCGATGTATCGTTTTGGCC
AGATGCGCTATACGCATCTTGGCGATGTTTTTTACGTCTTGATAAATTGGTTTGCTAGAACTAGGAGCTGACCCTGATTTAGCAAGTTTGTTAACT
CGTTTGAATAACGAGATTGTGCAAAGCTTGAGCAGCTTGAGCACTTGATGTTTTGCCTATTTCATTTTTTCATGAAAGTATCGGCAAGTATTTGTT
TATATTGAGCTCTAATCTTTGGATTTTCATCCAATTTATTTAACGTATCAGCACGTACATTATCTGTTTGATTACTTGTGAGTTCTGTTTGAGCTTCA
ATTTGTTTAGATTGAGCAATAGCTTGATTTGCTTGCGCAACCGTTTGATAAGCTGGGTTCCGGACGTAGTTGCATTACCGAGAACATTTTGCATCT
GGGCCATAGCTCCCAGCTGGTGTTGTGGCTCCGCCTTGTGAATACGCAAGCATGGGATTTAACCCAGCTTTTTTCATATCTTCAACTGCTCGTTGA
TATGATGTTCCAGACATTTCAGCTTGAAATCTCTGTTGATTACTCGCTTGTTCTGCACTCGCTGCGTTTTGACTTTGTGTTCCAAAGTATTGTCCTGC
TGCACCTATTGCTGCAGGTGCAAGTGCAGCTAAGGAGAGTCCCCCAGTAGCAGGGGCAGCTCCTATAGCTATAGCAGGGCCGATTAAATCGGC
AATTCCGTCAAATAGTCCCATTAGAAATGGTCTATTAAGCCAGGTACTGAATACATTGGCATTGGTCTTGCCATTTTGACATCAAAAAATGAGTCA
AATAAGAATTGTTGGCCATTTGCAGCTGCTCCAACGGCTACTACACGTGATACAGGAGGCGTATCTTGAATAAACGTATTATTCAAAGTAGGCGC
AGCGGTAAATTTTGAGCCAAATGCCAACCATCGATCGTGCCCGCCGATGTTGATTTGAACAAACCAGTAATTTTAGATGGTTTGTAACGATATTC
CGCCCAGCGTTCTTGATATCCAAAAACAGTATTGTCGGCTGCAGTATCTCCTGTTGCATAAATTTCTTTTTGCAAAACAGATTGTTCGCCTAGAGT
AGCAAATGCTGGGAAATAAAAGTCATATCGTGTAGATCTAGACCACATACGGTCTAATCCTTGTTGATAAGTAAGATCTGCTCTAATGGATACTA
ATCCAATAATAACGCCATGCTCAGTAAATGATTGAGTAAATCCATGATTATGAGCGAGAGCAGTACCCATAGCAGCAAGGTTGCCCAAAGGGGT
AGTCGTTCCAGAAGCGTTTGTTCCCGACGTTTGAGCAATCGGATTAACATTAATTGGTGTTGAACCGCCTCCAAGGTATTCAGGCCTTTGTAAAC
GGGCGTCTGGGGAAATAACTCCAAAGTGACTCCGGATAATTTCTGTGTATCGTGTTCCGCCACGTGCATCCCTTTCAAGTAATTTTTGAATTTGAA
ATGACTGTCTTAATTGATGACAGTTGCAGCAGTTGCTTCTGATAAGTCGGTATATAAATTAGAAACGTTATTTACTACACCAGCAGTATTAACACC
ATAAGCGTTGCCATATCTAGCTAAAGCAGTCGTATTTCCAGGATCTGTTTGAATTACCGTAAATTTATCGTTTGATGTTGCGTCTCCTGAAATGGT
ATCCCATTTAATTGGAGCCGTAGTACCTAAAGGTAAGGTGACACTCGCACCTTTTTGTGGCCATGGTAATGCTGACGTAAAGTAATCATGTCTTTA
CCACGACGTTTTAGCACATAGTTTGAGGAAGTATCAGGGCCATCGCCCTTATCAACTACTGCGCTTGTTTGTAAATTTTCATCTCGGAACCATTCG
TTCCAGATAAGATTGTATGCACGTGGCCAAAAGGCACAGTGCGTAATAGTTCGGCCAGTATCAATTTGGCCTACTGTTGGTAAGCCCATATAGTC
TTGAAGGCTGCCTACGGCATAACCATCTGTTGGGCTTGTTTGTGTTGGGACAATATAAGATATTGAGTCTGTTGGATTTTCTTGTTGACCCATAAA
TTTTTGCCAGTTATTCCATATAAGGCGATTGGGTACAAAGAAAAAGAAAGAATCCATAATCATGTTATCCATGATTGGATATAAAGGCGTTGCTA
GACGGGCAAATGCCGTCATTTTTAAGTTGAAAGTGTCCCCAGGGAGCACTTCATCAACATATACAGGAATTAGATAGCCCGCATCGAAAGTCGT
TTTATGTGTTTTTTGAGCATCGAATTTACTACGTGGTATATCGGCTCTAGGTACCATCGCGAAGCGGTGAGTATTAACTGACTGATTGCGGTGCAT
GTTTTTCCTTAGTGTTGTTCCGGGGGAAAGATAAATCTCTTTTCCCCTCGGTTGTTTTATTTAAGTTTAACTTGTTTTCCTAATGATAGGAGTTTTG
GTTGTTCATGTAAATCGAATAACCCAGTTGAATCGTCAAATGTTCCGAATTCATATAGATCGAAATCATCAGGGTGATTAAAGAGTTGATTTTCA
GTATCAGAACGATTAATTTCATCTGAAAAAGAGCGTATAGCTACTCCAGAGGAAGGTACGAACATTGGTCGTGCATATGCTTCAGCAGCACGGT
CTTTTACGGAAGCGAGGATAAGTTTCATTATTTTCCTAAGTAAGGTTACGTTTTAATAGTTGAAGTTTTGCCATAGTGACTTGTTCTTTTGCAGATA
GTCGTTCTGGTGTATTGTCTTCGGAATTAAATTTAGCATTATTTTCCCGCATGTAAAGTAATTCGTCATACTCATAAGGTTGGTCAATTTTAAACAT
TTTGTCATAGTATTTTGGTGGTTTGACCTTTTTACCTCTAAGTATTACGTAGTCTTGCGGGTATATATCCGAAGTATATTTTATATAAAAGTCTTTAC
CGATTCCCGGTTTTAAAGACATTTTATTATATTCCGGCTTTAAGTCTAAATATTCGCCGGTTTCAGGGTGTATGCGTTTGTAATGAGATTCCGCATC
TTTCCCTGTTTGTTTTTTCATTATGTATCTAGCCACGTAGGCGGCTGATTCAAAAGTAACATCTCCAATGGTGGTATAACCAAATGGCCAGAGAGC
TTCAAGTTCTGCGGATCTATATAACATAGAACCAGAGGCAGTCCTTTTCCATAATTTTTTATCAGGAAAGTCGTA

>000179F|arrow

TTCTGGCTTACGCTGAATTATCTCACCAGTCTCTAAATCACAATATTGATAATGGGCACCCGCATCAACCACTTCGTGGTTTTCATTGACAGTAACC
CCATTAATCTTCTTCATAATATATCTTGCAACATAAGCAGCAGACTCAAAAGTAACATCACCAATTGTAGAATAGCCAAACGGCCACAATTCTTCC
AAAATCTCTGACGTGTAGAGGATAGAGCCAGTCTGCGTTCTTTTAAATATTTTCTTATCCGGAAAATCAAGACCAAACAGACAAGCATGGAAATG
AGGACGATCAAAAGATTCACCATATTCACCTGCCATATAAAAACGTATCGTTTTCCCAGTAAAACGCTTACGTAACCGCTTCATAAAAAGCTGATA
ATCATTGTAATCCAATGACATATCCTTAGGACAATGCTCTGGAGCATATGTCAAAGTAATAAAACAATTACTAGTATGCATTTGTGCCTCATGCAT
ACAACGAATCGCCCACTGACGTGAGCGTTCAAGGCGACAACCAACACACTGACCACAAGGCAATGATAGGGTACGGACTACATCCGCACCCGG
TATTTCCCGCCAAATTATAGACCTGTCACTGCATTGATAAGCCGTTAAGGGCTTATAACAGGCCATAATTACAAACGATAGCCACCACGCTGGGG
AGCGTGTCTCATATTAATTGACTTCGTCTTACTAGCAGTTCTGCGAAATGACTTTGCAGATTTATATTTGTTTACTGGCTTTCTTCGTAACATGATG
AACTCCGTAGTTAAAATAGTGGTTTGGTGTCACCTAGCACAGTTACATCAAGTAGAGTAACTGTGCTGGCCTCAGGATTTCATCCTTCGGCCTTA
GGTGTTTCTGTAGAAACGATGGGTTCAACCACAGGTTGTCCATCAATAAGACCCAATTGAATCGCTTCATCACGATTCTGGTCGTTCTCAAGGAA
CTCCAATAATTTGACAGGATCATGGTCAAATCGGACTCTTAATTTCGCTGGCAGAGCCATGAAATCGTCCATAGTTGCGTTAATTTGATTCAACGC
AGAATGGTAATCAGTAACACCACTAAAATCGCCGTATTGAGGCGATACAGGGGCCGTTGGAAGTTCCCCTGTAACCCCGAAACGTTCAATGATG
ACATTAATGTCACATTCATCTTTCATATGCTGTTGAGCAAGACTTGGGTCTTGACACAGCAACGCATCGTTTACCGATGCTTCATCTTTATCATAGT
TGTACGGATTACGTACAAATGGCAAATTCGCTTTACTCATTATTTGACTCCAATTCCCCAAGGGGTTAGTTGATTAGGATTATTTTTGAGTTTATCA
AAAATTTCTTTCGAAGCATCGATACCCCTAGACCAAATAGCTGGGGATGGAGCCAATAGCCTCTTAGTATTATATGACTGAGCGGATGTTAAATC

CGCAGTCGTATTCTTTAAAGCAATATCAGCCAAAATACGTTTATTTTCGGCTGTAATATTAGGAGCAGTAAGCAACTTATTAACAGTATCAGCCCT
AGTATTAGCGGTACCAGCTTCAGTAGCTTCAGTCTGGGCGATAATCTGCTTTTCTGTTTCTGATGCATTACGAATCTGTTGCATCATAGAAGCAGT
ATTAATAGCTGAATTAGTTGCATTACCTAAAACATTTTCCATAGTAGCAGTTGAACCAGATGGGGTAGAAGCACCACCACGTTGATAGGCTAACA
TAGGAGATAAACCAGCAGCTTCTAAATCTTTAACAGCACGCTGGTAACTTGTTCCACTCATATCGGCTTGAAAATCCATTTGCCTCTGAGCCATCT
CCTGATTAGCTTTATTAGCAGAAGTAGAACCTAAATAAGAACCAACAGCGCCCAAAGCAGTACCGACACCAGGAGTAAAGAAACTAGAAGCTG
AAGACAGCTTAGAACCAACATTAGTAACCGCATCAAGTATTCCACCAAACATAAACTAACGCCCTTCGGTTGTTTCCTCACTACTCCTTACGGAGT
AGTCGAGGTTATATAAAACATTAGAAATGATCAATTAGACCAGGTACGCTGTACATAGGCATAGGTCTGGCCATCTGACAATCAAAGAAAGCAT
CCATTAAAAATTGCTGACCATTTGCTGCAGCTCCGACCGCCGTAGTACGAGCAACTGGAGGGGTCTCTTGAATAAACGTTGAATTCAACGTAGG
AAGAGAAGTAAATCGCTGTCCATAATGCCAAGCATCAATAGTGCCAGCAGAAGTAGACCTAAAGAAACCAGTAATTTGAGAAGGTTTGTAACGA
TACTCCGCCCAACGTTCTTGATAACCAAATACATCATTGTCGGCTGCAGTACCTTGAACATAAATTTCTTTGTTCAAAATAGCTTGCTCACCCAAAG
TGGCAAATACTGGGAAATAAAAGTCATATCGTGTAGACCTAGACCACATCTTAGGAAGACCCTGTTGATATGTGAGGTCAGCACGTACAGAAAC
TAATCCGATAATGTATCCATGTTCTTGAGCAGCATACGTAAAGCCGTGTCCCTGAGCCAATGCAGTACCCATTGCAGCAAGGTTACCTTGCGGAG
TAGCAGAACCAGTAACCGACGTTGCAGAAGTCTGAGCAATCGGATTAACATTAACAAGGGTCGAACCTCCACCAATATATTCAGGACGTTGTAA
ACGATAATCCTGTGGAGTTACTCCAAAGTGAGCACGTAGTAACTCAGTATAACGAGTACCACCACGTGCATCGCGCTCCAATAACTTCTGAATCT
GGAAAGATTGACGAAGTTGGTTAATAGTCGCAGCAGTAGCAGTAGACAAATCAGCGTATAACTGATTAGTAGCAACACCAGCATTTGTACTATT
AGACAAAGTATTAGAAGCTGAATTTAATTCTCTTAAAGCACCAACAGCAGTTTGAAAAACACTATAACCAGCAGTACCATTGCCAGCTGGTATGT
CAGAATAAATATTAGCACGTGTTCCTAACGGTAAAGAAACAGAAGCACCCTTCTGTGGCCAAGGCAATGCACCAGTAAAATAATCTTTACGCTTA
CCACGTCTCAAAAGTGTATAGTTAGCAGGAACATCACCTGAATCACCAAGATTTAACGTTACAGAATTCTGTAAGTTTTCATCTCTAAACCATTCG
TTATAAATCTCATTATAAGCACGTAACGGCAAAACATTATGCGTTACTGTATTAGCGCCAGTAATCTGACCAGCAGTAGGTAAACCAAAATAATC
AAAAATTGAACAAACAGCATAACCACCAGCAGGACTAGTAATAGTAGGCACTACAAAAGAAATAGAATCACCAGGATTCGTTTGTTCACCCATA
AACTTTGGCCAATTGTTCCAAAGTAAACGATTAGGTACAAAGAAAAAGAAAGTATCAAGATGCAAGTTGTCCATCACAGGAAATAACGGTGTGG
CCAAACGTGCAAATGCAGTCATCTTTACACGATGAGTATCGCCAGGCAATACTTCATCACAATAAATAGGAACTAAAAAACCAGCATCAAACGTG
GTTTTATGAGCATATTGAGTATTAAAACTAGAGCGGGGAATGTCCGCTTTAGGAACCATAGCAAATGAATGCGTACTTACTGACTTATTGCGAAA
CATAAACATCTCCCGTAGTTCCGTACCACTCTTTCGAGTGATACGGTATAAAAAAAACCTTACTCGCCTTCGCGAATCATTACATCTTTTGCACGA
GCAATCAACTTAGGGCTTTCCAGTAGTTCAAAAACACCAGTGGCATCGTCAAAAAGACCCAAATAGAACATATGAAAATCATCAGGATGTTTATA
CAACTGATTATCTTCGCTAGCTCGATTGACTTCATCCTGAAACTGACGTACTGCAACACCTTCAGATGCAACATAAGCTGGACGACCAAAAGCAT
CTGCAGCAGTATCCTTAATAGAAACAATAACCATCTTCATAAAAACTCCTTAAATAGTACGTTTTAACAATGACAACTTAGCCAACGCAACTTTTTC
CTTAACAGCAAGTCGCTCAAGCGTGTTGTCCTCATGCCTAGATCGACCTTCCATCTCTCTGGCAAACTGAATCATATCGAATTCTTCAGGAAACTT
CAACTTAAATTTATTATCATAAAACCGTGGTGGACGGCACTTTTTGCCACGCACCACAACGTGGTCTGACGTATAAACGTCTGACATGTACTTATC
TAACCACGATTGCCCGATACCGGGCTTCAATGACATCTTATTAAA

>000037F|arrow

TCCGCTAGGACTTTCCGTAAACATGCTTCACATACAAAACACGCAAATATGCGAAACTCGCCAATGCGTGGAGGCTGGAGACTCTAATAAAGTCT
TCAGGCACCTCACATGCCTTGTTATCACCCTCTCAAAGCATATCAATGCTTTGACAAATCAATTGTTTTCGACGAAGTTCGGAAACATGACATCGT
TCGATCTTTAGACCTGCCCTGTGGGCAGTGCGTTGGATGCCGTCTAGAACGATCAAGACAATGGGCTATTCGGTGCATGCACGAAGCCCAATTG
CATAAAAACAACTCATTCATAACACTCACATATGACAATACACATCTCCCAAGCGATGGCTCTTTGGATCACAAAGACTTTCAATTGTTCCTTAAA
AGACTTAGAAAAACTCTCGCAAAAAGAGGACTTACAATCCGCTATTACATGGCTGGAGAATATGGTGAACTCTTCGCAAGACCCCACTTCCATGC
CTGTATCTTCGGATACGACTTTCCTGATAAAAAATTATGGAAAAGGACTGCCTCTGGTTCTATGTTATATAGATCCGCAGAACTTGAAGCTCTCTG
GCCATTTGGTTATACCACCATTGGAGATGTTACTTTTGAATCAGCCGCCTACGTGGCTAGATACATAATGAAAAAACAAACAGGGAAAGATGCG
GAATCTCATTACAAACGCATACACCCTGAAACCGGCGAATATTTAGACTTAAAGCCGGAATATAATAAAATGTCTTTAAAACCGGGAATCGGTAA
AGACTTTTATATAAAATATACTTCGGATATATACCCGCAAGACTACGTAATACTTAGAGGTAAAAAGGTCAAACCACCAAAATACTATGACAAAA
TGTTTAAAATTGACCAACCTTATGAGTATGACGAATTACTTTACATGCGGGAAAATAATGCTAAATTTAATTCCGAAGACAATACACCAGAACGA
CTATCTGCAAAAGAACAAGTCACTATGGCAAAACTTCAACTATTAAAACGTAACCTTACTTAGGAAAATAATGAAACTTATCCTCGCTTCCGTAAA
AGACCGTGCTGCTGAAGCATATGCACGACCAATGTTCGTACCTTCCTCTGGAGTAGCTATACGCTCTTTTTCAGATGAAATTAATCGTTCTGATAC
TGAAAATCAACTCTTTAATCACCCTGATGATTTCGATCTATATGAATTCGGAACATTTGACGATTCAACTGGGTTATTCGATTTACATGAACAACC
AAAACTCCTATCATTAGGAAAACAAGTTAAACTTAAATAAAACAACCGAGGGGAAAAGAGATTTATCTTTCCCCCGGAACAACACTAAGGAAAA
ACATGCACCGCAATCAGTCAGTTAATACTCACCGCTTCGCGATGGTACCTAGAGCCGATATACCACGTAGTAAATTCGATGCTCAAAAAACACAT
AAAACGACTTTCGATGCGGGCTATCTAATTCCTGTATATGTTGATGAAGTGCTCCCTGGGGACACTTTCAACTTAAAAATGACGGCATTTGCCCGT
CTAGCAACGCCTTTATATCCAATCATGGATAACATGATTATGGATTCTTTCTTTTTCTTTGTACCCAATCGCCTTATATGGAATAACTGGCAAAAAT
TTATGGGTCAACAAGAAAATCCAACAGACTCAATATCTTATATTGTCCCAACACAAACAAGCCCAACAGATGGTTATGCCGTAGGCAGCCTTCAA
GACTATATGGGCTTACCAACAGTAGGCCAAATTGATACTGGCCGAACTATTACGCACTGTGCCTTTTGGCCACGTGCATACAATCTTATCTGGAA
CGAATGGTTCCGAGATGAAAATTTACAAACAAGCGCAGTAGTTGATAAGGGCGATGGCCCTGATACTTCCTCAAACTATGTGCTAAAACGTCGT

GGTAAAAGACATGATTACTTTACGTCAGCATTACCATGGCCACAAAAAGGTGCGAGTGTCACCTTACCTTTAGGTACTACGGCTCCAATTAAATG
GGATACCATTTCAGGAGACGCAACATCAAACGATAAATTTACGGTAATTCAAACAGATCCTGGAAATACGACTGCTTTAGCTAGATATGGCAAC
GCTTATGGTGTTAATACTGCTGGTGTAGTAAATAACGTTTCTAATTTATATACCGACTTATCAGAAGCAACTGCTGCAACTGTCAATCAATTAAGA
CAGTCATTTCAAATTCAAAAATTACTTGAAAGGGATGCACGTGGCGGAACACGATACACAGAAATTATCCGGAGTCACTTTGGAGTTATTTCCCC
AGACGCCCGTTTACAAAGGCCTGAATACCTTGGAGGCGGTTCAACACCAATTAATGTTAATCCGATTGCTCAAACGTCGGGAACAAACGCTTCTG
GAACGACTACCCCTTTGGGCAACCTTGCTGCTATGGGTACTGCTCTCGCTCATAATCATGGATTTACTCAATCATTTACTGAGCATGGCGTTATTA
TTGGATTAGTATCCATTAGAGCAGATCTTACTTATCAACAAGGATTAGACCGTATGTGGTCTAGATCTACACGATATGACTTTTATTTCCCAGCAT
TTGCTACTCTAGGCGAACAATCTGTTTTGCAAAAAGAAATTTATGCAACAGGAGATACTGCAGCCGACAATACTGTTTTTGGATATCAAGAACGC
TGGGCGGAATATCGTTACAAACCATCTAAAATTACTGGTTTGTTCAAATCAACATCGGCGGGCACGATCGATGGTTGGCATTTGGCTCAAAAATT
TACCGCTGCGCCTACTTTGAATAATACGTTTATTCAAGATACGCCTCCTGTATCACGTGTAGTAGCCGTTGGAGCAGCTGCAAATGGCCAACAATT
CTTATTTGACTCATTTTTTGATGTCAAAATGGCAAGACCAATGCCAATGTATTCAGTACCTGGCTTAATAGACCATTTCTAATGGGACTATTTGAC
GGAATTGCCGATTTAATCGGCCCTGCTATAGCTATAGGAGCTGCCCCTGCTACTGGGGGACTCTCCTTAGCTGCACTTGCACCTGCAGCAATAGG
TGCAGCAGGACAATACTTTGGAACACAAAGTCAAAACGCAGCGAGTGCAGAACAAGCGAGTAATCAACAGAGATTTCAAGCTGAAATGTCTGG
AACATCATATCAACGAGCAGTTGAAGATATGAAAAAAGCTGGGTTAAATCCCATGCTTGCGTATTCACAAGGCGGAGCCACAACACCAGCTGGA
GCTATGGCCCAGATGCAAAATGTTCTCGGTAATGCAACTACGTCCGGAACCCAAGCTTATCAAACGGTTGCGCAAGCAAATCAAGCTATTGCTCA
ATCTAAACAAATTGAAGCTCAAACAGAACTCACAAGTAATCAAACAGATAATGTACGTGCTGATACGTTAAATAAATTGGATGAAAATCCAAATA
TTAGAGCTCAATATAAACAAATACTTGCCGATACTTTCATGAAAAATGAAATAGGCAAAACATCAAGTGCTCAAGCTGCTCAAGCTTTGGCACAA
TCTCGTTATTCAAACGAGTTAACAAAACTTGCTAAATCAGGGTCAGCTCCTAGTTCTAGCAAACCAATTTATCAAGACGTAAAAAACATCGCCAAA
GATGCGTATAGCGCATCTGGCGCAAAACGATACATCGATAACTATCGAGGTCAACCGATTCAACAAAATCGTACAAATAACCAACCACCAATGG
AATGAAAATGACAAAGATTACAGCCCCATTTCTTCGTACTCCGTACAATTACGACACGATTGCTGCGTCAAATGAGTCAGGGCTGCATTGTGAGG
ATGCAACTCTGACTCAGCAGCAATTTGCTGAAGAATGTGATATCAATAATATTATGGAAAAGTTCGGTATGACCGGACTTATTCCTCAAACTCCTT
TAACGCCTCAATATGGCGACTTTAGTGGTGTCTATGACTACCACTCTGCTCTGAACCAGATTATGGCTTCAGACAACGAATTTATGGCTTTACCAG
CCAATATTCGTGAACGATTCGCTAATGATCCCGCGAATCTAATAGATTTTCTAGAAAACCCTGAAAATCGCAGCGAAGCTGAAAAAAATGGGACTG
GTAAAACCAGCCCAAACCGAGGTTTCAACCCCTGTTGGAACCTCGGAAGCACAGTTACCTACTTGATGTAACTGTGCTAGGTGACACCAAACCAC
AAAAACACGATAAACAAGGACAGAAAAAATGATGCGTCGCAGACCAGCAAATAAGCAAAAG

>000017F|arrow

GTCAGCATTACCATGGCCACAAAAAGGTGCGAGTGTCACCTTACCTTTAGGTACTACGGCTCCAATTAAATGGGATACCATTTCAGGAGACGCAA
CATCAAACGATAAATTTACGGTAATTCAAACAGATCCTGGAAATACGACTGCTTTAGCTAGATATGGCAACGCTTATGGTGTTAATACTGCTGGT
GTAGTAAATAACGTTTCTAATTTATATACCGACTTATCAGAAGCAACTGCTGCAACTGTCAATCAATTAAGACAGTCATTTCAAATTCAAAAATTA
CTTGAAAGGGATGCACGTGGCGGAACACGATACACAGAAATTATCCGGAGTCACTTTGGAGTTATTTCCCCAGACGCCCGTTTACAAAGGCCTG
AATACCTTGGAGGCGGTTCAACACCAATTAATGTTAATCCGATTGCTCAAACGTCGGGAACAAACGCTTCTGGAACGACTACCCCTTTGGGCAAC
CTTGCTGCTATGGGTACTGCTCTCGCTCATAATCATGGATTTACTCAATCATTTACTGAGCATGGCGTTATTATTGGATTAGTATCCATTAGAGCA
GATCTTACTTATCAACAAGGATTAGACCGTATGTGGTCTAGATCTACACGATATGACTTTTATTTCCCAGCATTTGCTACTCTAGGCGAACAATCT
GTTTTGCAAAAAGAAATTTATGCAACAGGAGATACTGCAGCCGACAATACTGTTTTTGGATATCAAGAACGCTGGGCGGAATATCGTTACAAAC
CATCTAAAATTACTGGTTTGTTCAAATCAACATCGGCGGGCACGATCGATGGTTGGCATTTGGCTCAAAAATTTACCGCTGCGCCTACTTTGAATA
ATACGTTTATTCAAGATACGCCTCCTGTATCACGTGTAGTAGCCGTTGGAGCAGCTGCAAATGGCCAACAATTCTTATTTGACTCATTTTTTGATG
TCAAAATGGCAAGACCAATGCCAATGTATTCAGTACCTGGCTTAATAGACCATTTCTAATGGGACTATTTGACGGAATTGCCGATTTAATCGGCC
CTGCTATAGCTATAGGAGCTGCCCCTGCTACTGGGGGACTCTCCTTAGCTGCACTTGCACCTGCAGCAATAGGTGCAGCAGGACAATACTTTGGA
ACACAAAGTCAAAACGCAGCGAGTGCAGAACAAGCGAGTAATCAACAGAGATTTCAAGCTGAAATGTCTGGAACATCATATCAACGAGCAGTT
GAAGATATGAAAAAAGCTGGGTTAAATCCCATGCTTGCGTATTCACAAGGCGGAGCCACAACACCAGCTGGAGCTATGGCCCAGATGCAAAAT
GTTCTCGGTAATGCAACTACGTCCGGAACCCAAGCTTATCAAACGGTTGCTCAAGCAAATCAAGCTATTGCTCAATCTAAACAAATTGAAGCTCA
AACAGAACTCACAAGTAATCAAACAGATAATGTACGTGCTGATACGTTAAACAAATTGGATGAAAATCCAAATATTAGAGCTCAATATAAACAAA
TACTTGCCGATACTTTCATGAAAAATGAAATAGGCAAAACATCAAGTGCTCAAGCTGCTCAAGCTTTGGCACAATCTCGTTATTCAAACGAGTTA
ACAAAACTTGCTAAATCAGGGTCAGCTCCTAGTTCTAGCAAACCAATTTATCAAGACGTAAAAATATCGCCAAAGATGCGTATAGCGCATCTGGC
GCAAAACGATACATCGATAACTATCGAGGTCAACCGATTCAACAAAATCGTACATAACCAACCACCAATGGAATGAAAATGACAAAGATTACAG
CCCCATTTCTTCGTACTCCGTACAATTACGACACGATTGCTGCGTCAAATGAGTCAGGGCTGCATTGTGAGGATGCAACTCTGACTCAGCAGCAA
TTTGCTGAAGAATGCGATATTAATAATATTATGGAAAAGTTTGGTATGACCGGACTTATTCCTCAAACTCCTTTAACGCCTCAATATGGCGACTTT
AGTGGTGTCTATGACTACCACTCTGCTCTGAACCAGATTATGGCTTCAGACAACGAATTTATGGCTTTACCAGCCAATATTCGTGAACGATTCGCT
AATGATCCCGCGAATCTAATAGATTTTCTAGAAAACCCTGAAAATCGCAGCGAAGCTGAAAAAAATGGGACTGGTAAAACCAGCCCAAACCGAGG
TTTCAACCCCTGTTGGAACCTCGGAAGCACAGTTACCTACTTGATGTAACTGTGCTAGGTGACACCAAACCACAAAAACACGATAAACAAGGACA
GAAAAAATGATGCGTCGCAGACCAGCAAATAAGCAAAAGTCCGCTAGGACTTTCCGTAAACATGCTTCACATACAAAACACGCAAATATGCGAA

ACTCGCCAATGCGTGGAGGCTGGAGACTCTAATAAAGTCCCCAGGCACCTCACATGCCTTGTTATCACCCTCTCAAAGCATTTCAATGCTTTGACA
AATCAATTGTTTTCGACGAAGTTCGGAAACATGACATCGTTCGATCTTTAGACCTGCCCTGTGGGCAGTGCGTTGGATGCCGTCTAGAACGATCA
AGACAATGGGCTATTCGGTGCATGCACGAAGCCCAATTGCATAAAAACAACTCATTCATAACACTCACATATGACAATACACATCTCCCAAGCGA
TGGCTCTTTGGATCACAAAGACTTTCAACTGTTCCTTAAAAGACTTAGAAAAACTCTCGCAAAAAGAGGACTTACAATCCGCTATTACATGGCTG
GAGAATATGGTGAACTCTTCGCAAGACCCCACTTCCATGCCTGTATCTTCGGATACGACTTTCCTGATAAAAAATTATGGAAAAGGACTGCCTCT
GGTTCTATGTTATATAGATCCGCAGAACTTGAAGCTCTCTGGCCATTTGGTTATACCACCATTGGAGATGTTACTTTCGAATCAGCCGCCTACGTG
GCTAGATACATAATGAAAAAACAAACAGGGAAAGATGCGGAATCTCATTACAAACGCATACACCCTGAAACCGGCGAATATTTAGACTTAAAGC
CGGAATATAATAAAATGTCTTTAAAACCGGGAATCGGTAAAGACTTTTATATAAAATATACTTCGGATATATACCCGCAAGACTACGTAATACTTA
GAGGTAAAAAGGTCAAACCACCAAATACTATGACAAAATGTTTAAAATTGACCAACCTTATGAGTATGACGAATTACTTTACATGCGGGAAAATA
ACGCTAAACTTAATTCCGAAGACAATACACCAGAACGACTATCTGCAAAAGAACAAGTAACTATGGCAAAACTTCAACTATTAAAACGTAATCTC
ACTTAGGAAAATAATGAAACTTATCCTCGCTTCCGTAAAAGACCGTGCTGCTGAAGCATATGCACGACCAATGTTCGTACCTTCCTCTGGAGTAG
CTATACGCTCTTTTTCAGATGAAATTAATCGTTCTGATACTGAAAATCAACTTTTTAATCACCCTGATGACTTCGATCTATATGAATTCGGAACTTTT
GACGATTCAACTGGGTTATTCGATTTACATGAACAACCAAAACTCCTATCATTAGGAAAACAAGTTAAACTTAAATAAAACAACCGAGGGGAAA
AGAGATTTATCTTTCCCCCGGAACAACACTAAGGAAAAACATGCACCGCAATCAGTCAGTTAATACTCACCGCTTCGCGATGGTACCTAGAGCCG
ATATACCACGTAGTAAATTCGATGCTCAAAAAACACATAAAACGACTTTCGATGCGGGCTATTTAATTCCTGTATATGTTGATGAAGTGCTCCCTG
GGGACACTTTCAACTTAAAAATGACGGCATTTGCCCGTCTAGCAACGCCTTTATATCCAATCATGGACAACATGATTATGGATTCTTTCTTTTTCTT
TGTACCCAATCGCCTTATATGGAATAACTGGCAAAAATTTATGGGTCAACAAGAAAATCCAACAGACTCAATATCTTATATTGTCCCAACTCAAAC
AAGCCCAACAGATGGTTATGCCGTAGGCAGCCTTCAAGACTATATGGGCTTACCAACAGTAGGCCAAATTGATACTGGCCGAACTATTACGCAC
TGTGCCTTTTGGCCACGTGCATACAATCTTATTTGGAACGAATGGTTCCGAGATGAAAATTTACAAACAAGCGCAGTAGTTGATAAGGGCGATG
GCCCTGATACTTCCTCAAACTATGTGCTAAAACGTCGTGGTAAAAGACATGATTACTTTAC

>000201F|arrow

GCCGTATTCTCCAGCCATGTAGTAACGTAATTTTGCAGGTGCAATGGATTTTCTGAAGCGCTTAAGGAACTTTTGGAAGTCGCTTTTGACAAGCG
ATCCAGTTTGTGGAAGGTTTTCATTGTTGTATGTGAGGGTTTAAAGCAGTTTTTTTCGTGCAATTGGGCTTCATGCATGCAGCGCATGGCCCATTG
ACGTGATTTTTCTAGCCTGCAGCCAACGCATTGGCCGCAGGGCAGTGAAATCTGACGATCATGCTCGTCAGATTCTTTGAATGCGACACGGCGAT
AAGATTTGCCGGTCTTATAGTTTGTCTGATGCTTACTTAAATAAGCAGTCAGTGGGTGATAACAGGCCATGTGAGGTAGCCTGGCGCTTTATTAG
CCGCCAGCCCCCTCTTTGCGGGCTTTTTGCATATTTGCAGCTTTAGTTTTTGAAGTGTTCCTACGGAACGATTTAGCGCTTTTGCGCTTGTTTGTTT
GTTTTCTATAAAGCATAATTTTTGGCCTCGTTTATCAGATATTTTTGGTGTTGGTGTCACCTAGCACAGTTACATCAAGTGGAGTAACTGTGCTTAT
CGCCATTTCTTCCGAAATGGCTCTTTCGACCAGGCCGAGTTTCTCGGCTTCTGGTCGATTTTTCTCGTCTTGCAAGAACTCAATCAGGTTTGCTGGT
TCGTTTTCGAACCTAGCACGAATTTGAGCCGGTAAGTTATCAAATTCTTCCATAGCGTTCATTACCTTATTCAAGGCGCTATGATAGTCAGTAATTC
CGCTGAAATCGCCATATTGAGGCGGCAGCGGACTTTGAGGTAATAGGCCTGTAACGTTAAAACGTTCCAGGATAGTATTTATATCGCATTCGTCT
TTATAATGCTGCTGAGCCAGAGTTGGCTCCTCACAAGCCAACCCTGACTCATTTGACGCAGCATCCGTGTCATAGTTGTATGCGGTTCTAAGTTTG
ATTGTTTTCATTTCATTTTTCCAAATGGTAGATATTTTTGATATTTATCATATTGGCTATGTAAATAGCCTTTAACATCTTGATAAATTGGTTTAGTA
CTGGATGGCGCTGATCCAGTTTGCGCTAATCGCTCTAATTGGTCTGTATATGCACTTTGACTTAACGCTTGTCGCTGTTGTGCAGCTTGTAATGCA
CTTGATGTCCTTAATTGCTCTATTTGGGCATCGCGAAGCTGACCAAATTTGCCGTATCCCGGCATCTGAGCAATTTCACGAGCTGTATTTGCTCGA
GTATATGTTGCCTGATCACGTGATAGATTTGTATCCGCGTCCGTTTTTTCGGCTTGCGATTGTGTCAGGATATTCTGAGTTTGTAATTGTTTAAAAT
TGGCGACTGCCATGGCAGCTTCACGGGCTGAATTTCCAGCCTCACCTAGTGGATTTCCTACTGGAGCTTGCGCACCAGGCTGGACTTTGGCTCCG
CCTTGTGAATAAGCCAGCATAGGGTTAAGACCTGCAGCCTTAAGATCCGCTACTGCGTCTTGATATGCAGTACGTCGCATACCATCTTGGAATTG
CATCATTGCTGCAGCTTGTTGTGCGCTGGCAGCGTTTTGTTCTTGACCACCAAAATAACTAGCGGCCGCACCTATTCCGGCTCCAATTAAGGAGCC
GTAAGGTCCAAAAGCGGCGCCAGATGCGGCGCCTGAGGCAGCGGCTTCTAGTGCCATTAGAAGTGGTCGATTAAGCCAGGTACAGAGTACATT
GGCATTGGCCGTGCTTTTTTAATATCAAAAAGCTATCAAAAATGATTTGCTGGCCGTTGGCAGCTGCTCCGACCGCAAGGGTTCGAGAGACAGG
TGGATTTTCTTGAATAAACGTGTTATTCAAAGTTGGAAGTGTTGTAAACCGTTGGGCTAAATGCCAGCCGTCAATTGTTCCAGCTGCCGTACTAC
GGAACAGACTGGAAATTCGGCTTGGCATATAACGATATTCTGCCCAACGTTCTTGGTATCCAAACACTCCTGTATCGTTGGCATCGCCTCGTACGT
AGATTTCTTCATTTAATACTGCTTGTTCGCCCAAAGTGGCGAAAGCAGGGAAATAGAAATCATAACGTGTGGAACGATTCCACATGCGGTGCATA
CCTTGTTGGTATGTTAAATCGGCTCGTACTGAAACGAGACCGATAATTACACCATGTTCAGTGCTTGAATAAGTAAAGCCATGATTGTGAGCGAG
CCCAGTACCCATAGCGCCAAGTGTACCGAGAGGAGTAGTGGTCCCGGAAGCTCCGGTACCATTCGTCTGGGCGATTGGATTGATATTAATGTGT
GTTGAACCGCCTCCAATGTATTCAGGCCGTTGGAGACGCGCATCGGGGCTAACGACCCCGAAATGGCTGCGGATAATTTCTGTGTATCGAGTTC
CGCCACGGGCGTCCCTTTCTAAAAGTTTTTGAATCTGGAAGCTTTGACGCAATTGATTAATTGTTGCAGCGGTTGCAGCTGATAAATCTGCATATA
AACCGCCATTAGGATCATATGATTTTGCCAAGCCGTCAGCACCGCCAGTAATTTGACCAGTAACACCTACGTTAATAGCCTGGGCGGTTGCGTTT
AATGTATTTGTTCCAGCGTTATACAGTCTGGAAACAGGCGCATTGTTAGTGCGTAATATTGGGGCGGATGTTCCAAGCGGTAAAGTAACGCTTG
CGCCCTTTTGTGGCCAAGGTAATGCTGACGTGAAATAGTCTTTACGTTTTCCACGTCGTAATAATGTGTAGTTGGCTACGTTATCTGGACCATCGC
CAGTATCTACAACTACTGAATTTTGAAGGTTTTCGTCCCGAAACCATTCGTTATAAATAAGGTTGTAAGCACGTGGCCAGAAGGCACAGTGACTT

ACGGTGCCACCAGCACCTACTTGGCCTACGGTTGGCAGACCCATATAATCTTGAAGGCTGCCAATAGCGTAACCACCAGCTGGGGTTACTTGTTG
GGGCACTACGTACGAGATCGAGTCCGCAGGATTTGCCTGCTGCCCCATAAATTTTTGCCAATTTGACCAGATAAGTCTATTTGGCACAAAAAGAA
GAGCTATCCAATGCATGTTATCCATGATCGGATAGAGTGGTGTAGACAATCGCGCAAAGGCGGTCATGTTGCAGCGGAATGTATCGCCTGGCAA
CATTTCGTCTACGAGTACAGGGACTAGGAAGCCAGCATCGAATGTAGTTTTATGTGTTGATTGACAGTCAAATGTAGAGCGGGGGATATCCGCT
TTTGGAATCATTGTGAACTGATGGACGTCTACCGACTTGTTACGATGCATTTTTTTGAGCTCCTAGGCCTAGTTGCGTGAGAAAAAGGGGTTTCC
CCCTTTTTACTCTACGCTTAGTTTTTATCAGTAATTTTGACTTGTTTCCCTAAGGATACAAGTTTGGGTTGTTCATGTAATTGGAACAAACCCGTATT
ATCGTCGAATTCGCCTAATTCAAATAGGTCGAAATCGTCGGAATGGTTATAAAGTTGATTGTCATCGCTCTGGCGATTGACTTCGTCGCTAAAGC
TCCGGATTGCTTCGCCGATAGAACGGACGAACATTGGACGACCGAACGCATCTGCTGCGCGGTCTTTAACGGTACAGAGTACTAATTTCATGAG
GATTTCCTAAGTGAGGTTACGTTTTAATAATTGAAGTTTGGCCTTTGTGACTTTTTCCTTTACGGCAAGTCTGGCATAGGTATTGTCTTCGTGGTTG
AGTTTAGCAGAAGTTTCACGTTTGTGGAGTAATTCTTCGTATTCATATGGATAATCTGATTTATATTTTTTGTCATAGTATTTTGGGGGTTTTACCC
TTTTTCCGTTGACTACAACATAGTCATGGGGATATACGTCGGAACGATATTTTTTGTACCACTCAGCACCAATGCCTGGTTTAAGGCTCATATTGT
TATATTCTGGTAATAGCTTTATTAATTCCCCTGTTTCTATATCACAGTATGTGTAATGCTCATCCTTTGTTATATGTTTGTTTTCTTCCATCTGGCCGT
TGTATTTTTGCATAATATATCGAGCAACGTAAGCAGCTGACTCGAATGTAACGTCTCCAATGGAGGAATAACCATGTGGCCAGAGGGTTGCAAG
GTGGTCGGATGTATATATGAGAGAACCAGAGGGAGTCCTTTTGAATAGTTTCTTATCATGAAAATCGTATCCGAAGATACAGGCATGGAAGTGA
GGTCTGCCGAAACTTGT

>000007F|arrow

CCAAACCACTATTTTAACTACGGATTCATCATGTTACGAAGAAAGCCAGTAAACACAATATAAATCTGCAAAGTCCATTTCGCAGAACTGCTAGTA
AGACGAAGTCAATTAATATGAGACACGCTCCCCAGCGTGGTGGCTATCGTTTGTAATTATGGCCTGTTATAAAAGCCCTTAACGGCTTATCAATG
CAGTGACAGGTCTATAATTTGGCGGGAAATACCGGTGCGGATGTAGTCCGTACCCTATCATTGCCTTGTGGTCAGTGGTTTGGGTTGTCGCCTTG
AACGCTCACGTCAGTGGGCGATTCGTTGTATGCATGAGGCACAAATGCATACTAGTAATTGTTTTATTACTTTGACATATGCTCCAGAGCATTGTC
CTAAGGATATGTCATTGGATTACAATGATTATCAGCTTTTTATGAAGCGGTTACGTAAGCGTTTTACTGGGAAAACGATACGTTTTATATGGCAG
GTGAATATGGTGAATCTTTTGATCGTCCTCATTTCCATGCTTGTCTGTTTGGTCTTGATTTTCCGGATAAGAAAATATTTAAAAGAACGCAGACTG
GCTCTATCCTCTACACGTCAGAGATTTTGGAAGAATTGTGGGCCGTTTGGCTATTCTACAATTGGTGATGTTACTTTTGAGTCTGCTGCTTATGTT
GTCAAGATATATTATGAAGAAGATTAATGGGTTACTGTCAATGAAAACCACGAAGTGGTTGATGCGGGTGCCCATTATCAATATTGTGATTTAGA
GACTGGTGAGATAATTCAGCGTAAGCCAGAATTTAATAAGATGTCATTGAAGCCCGTATCGGGCAATCGTGGTTAGATAAGTACATGTCAGACG
TTTATACGTCAGACCACGTTGTGGTGCGTGGCAAAAAGTGCCGTCCACCACGGTTTTATGATAATAAATTTAAGTTGAAGTTTCCTGAAGAATTC
GATATGATTCAGTTTGCCAGAGAGATGGAAGGTCGATCTAGGCATGAGGACAACACGCTTGAGCGACTTGCTGTTAAGGAAAAAGTTGCGTTG
GCTAAGTTGTCATTGTTAAAACGTACTATTTAAGGAGTTTTTATGAAGATGGTTATTGTTTCTATTAAGGATACTGCTGGCAGATGCTTTTGGTCG
TCCAGCTTATGTTGCATCTGAAGGTGTTGCAGTACGTCAGTTTCAGGATGAAGTCAATCGAGCTAGCGAAGATAATCAGTTGTATAAACATCCTG
ATGATTTTCATATGTTCTATTTGGGTCTTTTTGACGATGCCACTGGTGTTTTTGAACTACTGGAAAGCCCTAAGTTGATTGCTCGTGCAAAAGATG
TAATGATTCGCGAAGGCGAGTAAGGTTTTTTTATACCGTATCACTCGAAAGAGTGGTACGGAACTACGGGAGATGTTTATGTTTCGCAATAAGT
CAGTAAGTACGCATTCATTTGCTATGGTTCCTAAAGCGGACATTCCCCGCTCTAGTTTTAATACTCAATATGCTCATAAAACCACGTTTGATGCTG
GTTTTTTAGTTCCTATTTATTGTGATGAAGTATTGCCTGGCGATACTCATCGTGTAAAGATGACTGCATTTGCACGTTTGGCCACACCGTTATTTCC
TGTGATGGACAACTTGCATCTTGATACTTTCTTTTTCTTTGTACCTAATCGTTTACTTTGGAACATTGGCCAAAGTTTATGGGTGAACAAACGAATC
CTGGTGATTCTATTTCTTTTGTAGTGCCTACTATTACTAGTCCTGCTGGTGGTTATGCTGTTTGTTCAATTTTTGATTATTTTGGTTTACCTACTGCT
GGTCAGATTACTGGCGCTAATACAGTAACGCATAATGTTTTGCCGTTACGTGCTTATAATGAGATTTATAACGAATGGTTTAGAGATGAAAACTT
ACAGAATTCTGTAACGTTAAATCTTGGTGATTCAGGTGATGTTCCTGCTAACTATACACTTTTGAGACGTGGTAAGCGTAAAGATTATTTTACTGT
GCATTGCCTTGGCCACAGAAGGGTGCTTCTGTTTCTTTACCGTTAGGAACACGTGCTAATATTTATTCTGACATACCAGCTGGCAATGGTACTGCT
GGTTATAGTGTTTTCAAACTGCTGTTGGTGCTTTAAGAGAATTAAATTCAGCTTCTAATACTTTGTCTAATAGTACAAATGCTGGTGTTGCTACTA
ATCAGTTATACGCTGATTTGTCTACTGCTACTGCTGCGACTATTAACCAACTTCGTCAATCTTTCCAGATTCAGAAGTTATTGGAGCGCGATGCAC
GTGGGTGGTACTCGTTATACTGAGTTACTACGTGCTCACTTTGGAGTAACTCCACAGGATTATCGTTTACAACGTCCTGAATATATTGGTGGAGG
TTCGACCCTTGTTAATGTTAATCCGATTGCTCAGACTTCTGCAACGTCGGTTACTGGTTCTGCTACTCCGCAAGGTAACCTTGCTGCAATGGGTAC
TGCATTGGCTCAGGGACACGGCTTTACGTATGCTGCTCAAGAACATGGATACATTATCGGATTAGTTTCTGTACGTGCTGACCTCACATATCAAC
AGGGTCTTCCTAAGATGTGGTCTAGGTCTACACGATATGACTTTTATTTCCCAGTATTTGCCACTTTGGGTGAGCAAGCTATTTTGAACAAAGAAA
TTTATGTTCAAGGTACTGCAGCCGACAATGATGTATTTGGTTATCAAGAACGTTGGGCGGAGTATCGTTACAAACCTTCTCAAATTACTGGTTTCT
TTAGGTCTACTTCTGCTGGCACTATTGATGCTTGGCATTATGGACAGCGATTTACTTCTCTTCCTACGTTGAATTCAACGTTTATTCAAGAGACCCC
TCCAGTTGCTCGTACTACGGCGGTCGGAGCTGCAGCAAATGGTCAGCAATTTTTAATGGATGCTTTCTTTGATTGTCAGATGGCCAGACCTATGC
CTATGTACAGCGTACCTGGTCTAATTGATCATTTCTAATGTTTTATATAACCTCGACTACTCCGTAAGGAGTAGTGAGGAAACAACCGAAGGGCG
TTAGTTTATGTTTGGTGGAATACTTGATGCGGTTACTAATGTTGGTTCTAAGCTGTCTTCAGCTTCTAGTTTCTTTACTCCTGGTGTCGGTACTGCT
TTGGGCGCTGTTGGTTCTTATTTAGGTTCTACTTCTGCTAATAAAGCTAATCAGGAGATGGCTCAGAGGCAAATGGATTTTCAAGCCGATATGAG
TGGAACAAGTTACCAGCGTGCTGTTAAAGATTTAGAAGCTGCTGGTTTATCTCCTATGTTAGCCTATCAACGTGGTGGTGCTTCTACCCCATCTGG

TTCAACTGCTACTATGGAAAATGTTTTAGGTAATGCAACTAATTCAGCTATTAATACTGCTTCTATGATGCAACAGATTCGTAATGCATCAGAAAC
AGAAAAGCAGATTATCGCCCAGACTGAAGCTACTGAAGCTGGTACCGCTAATACTAGGGCTGATACTGTTAATAAGTTGCTTACTGCTCCTAATA
TTACAGCCGAAAATAAACGTATTTTGGCTGATATTGCTTTAAAGAATACGACTGCGGATTTAACATCCGCTCAGTCATATAATACTAAGAGGCTAT
TGGCTCCATCCCCAGCTATTTGGTCTAGGGGTATCGATGCTTCGAAAGAAATTTTTGATAAACTCAAAAATAATCCTAATCAACTAACCCCTTGGG
GAATTGGAGTCAAATAATGAGTAAAGCGAATTTGCCATTTGTACGTAATCCGTACAACTATGATAAAGATGAAGCATCGGTAAACGATGCGTTG
CTGTGTCAAGACCCAAGTCTTGCTCAACAGCATATGAAAGATGAATGTGACATTAATGTCATCATTGAACGTTTCGGGGTTACAGGGGAACTTCC
AACGGCCCCTGTATCGCCTCAATACGGCGATTTTAGTGGTGTTACTGATTACCATTCTGCGTTGAATCAAATTAACGCAACTATGGACGATTTCAT
GGCTCTGCCAGCGAAATTAAGAGTCCGATTTGACCATGATCCTGTCAAATTATTGGAGTTCCTTGAGAACGACCAGAATCGTGATGAAGCGATTC
AATTGGGTCTTATTGATGGACAACCTGTGGTTGAACCCATCGTTTCTACAGAAACACCTAAGGCCGAAGGATGAAATCCTGAGGCCAGCACAGT
TACTCTACTTGATGTAACTGTGCTAGGTGACA

>000217F|arrow

TATTTTCTTATCCGGAAAATCAAGACCAAACAGACAAGCATGGAAATGAGGACGATCAAAAGATTCACCATATTCACCTGCCATATAAAAACGTA
TCGTTTTCCCAGTAAAACGCTACGTAACCGCTTCATAAAAAGCTGATAATCATTGTAATCCAATGACATATCCTTAGGACAATGCTCTGGAGCATA
TGTCAAAGTAATAAAACAATTACTAGTATGCATTTGTGCCTCATGCATACAACGAATCGCCCACTGACGTGAGCGTTCAAGGCGACAACCAACAC
ACTGACCACAAGGCAATGATAGGGTACGGACTACATCCGCACCCGGTATTTCCCGCCAAATTATAGACCTGTCACTGCATGATAAGCCGTTAAG
GGCTTATAACAGGCCATAAACAACGATAGCCACCACGCTGGGGAGCGTGTCTCATATTAATTGACTTCGTCTACTAGCAGTTCTGCGAAATGACT
TTGCAGATTTTATTTGTTTATGGCTTTTCGTAACATGATGAACTCCGTAGTTAAAATAGTGGTTTGGTGTCACCTAGCACAGTTACATCAAGTAGA
GTAACTGTGCTGGCCTCAGGATTTCATCCTTCGGCTTAGGTGTTTCTGTAGAAACGATGGGTTCAAACAGGTTGTCCATCAATAAGACCCAATTG
AATCGCTTCATCACGATTCTGGTCGTTCTCAAGGAACTCCAATAATTTGACAGGATCATGGTCAAATCGGACTCTTAATTTCGCTGGCAGAGCCAT
GAAATCGTCCATAGTTGCGTTAATTTGATTCAACGCAGAATGGTAATCAGTAACACCACTAAAATCGCCGTATTGAGGCGATACAGGGGCCGTT
GGAAGTTCCCCGTAACCCCTGTAACCCGAAACGTTCAATGATGACATTAATGTCACATTCATCTTTCATATGCTGTTGAGCAAGACTTGGGTCTTG
ACACAGCAACGCATCGTTTACCGATGCTTCATCTTTATCATAGTTGTACGGATTACGTACAAATGGCAAATTCGCTTTACTCATTATTTGACTCCAA
TTCCCCAAGGGGTTAGTTGATTAGGATTATTTTTGAGTTTATCAAAAATTTCTTTCGAAGCATCGATACCCCTAGACCAAATAGCTGGGGATGGA
GCCAATAGCCTCTTAGTATTATATGACTGAGCGGATGTTAAATCCGCAGTCGTATTCTTTAAAGCAATATCAGCCAAAATACGTTTATTTTCGGCT
GTAATATTAGGAGCAGTAAGCAACTTATTAACAGTATCAGCCCTAGTATTAGCGGTACCAGCTTCAGTAGCTTCAGTCTGGGCGATAATCTGCTT
TTCTGTTTTCTGATGCATTACGAATCTGTTGCATCATAGAAGCAGTATTAATAGCTGAATTAGTTGCATTACCTAAAACATTTTCCATAGTAGCAGT
TGAACCAGATGGGGTAGAAGCACCACCACGTTGATAGGCTAACATAGGAGATAAACCAGCAGCTTCTAAATCTTTAACAGCACGCTGGTAACTT
GTTCCACTCATATCGGCTTGAAAATCCATTTGCCTTGAGCCATCTCTGATTAGCTTTATTAGCAGAAGTAGAACCTAATAAGAACCAACAGCGCCC
AAAGCAGTACCGACACCAGGAGTAAAGAAACTAGAAGCTGAAGACAGCTTAGAACCAACATTAGTAACCGCATCAAGTATTCCACCAAACATAA
ACTAACGCCCTTCGGTTGTTTCCTCACTACTCCTTACGGAGTAGTCGAGGTTATATAAAACATTAGAAATGATCAATTAGACCAGGTACGCTGTAC
ATAGGCATAGGTCTGGCCATCTGACAATCAAAGAAAGCATCCATTAAAAATTGCTGACCATTTGCTGCAGCTCCGACCGCCGTAGTACGAGCAAC
TGGAGGGGTCTCTTGAATAAACGTTGAATTCAACGTAGGAAGAGAAGTAAATCGCTGTCCATAATGCCAAGCATCAATAGTGCCAGCAGAAGTA
GACCTAAAGAAACCAGTAATTTGAGAAGGTTTGTAACGATACTCCGCCCAACGTTCTTGATAACCAAATACATCATTGTCGGCTGCAGTACCTTG
AACATAAATTTCTTTGTTCAAAATAGCTTGCTCACCCAAAGTGGCAAATACTGGGAAATAAAAGTCATATCGTGTAGACCTAGACCACATCTTAG
GAAGACCCTGTTGATATGTGAGGTCAGCACGTACAGAAACTAATCCGATAATGTATCCATGTTCTTGAGCAGCATACGTAAAGCCGTGTCCCTGA
GCCAATGCAGTACCCATTGCAGCAAGGTTACCTTGCGGAGTAGCAGAACCAGTAACCGACGTTGCAGAAGTCTGAGCAATCGGATTAACATTAA
CAAGGGTCGAACCTCCACCAATATATTCAGGACGTTGTAAACGATAATCCTGTGGAGTTACTCCAAAGTGAGCACGTAGTAACTCAGTATAACGA
GTACCACCACGTGCATCGCGCTCCAATAACTTCTGAATCTGGAAAGATTGACGAAGTTGGTTAATAGTCGCAGCAGTAGCAGTAGACAAATCAG
CGTATAACTGATTAGTAGCAACACCAGCATTTGTACTATTAGACAAAGTATTAGAAGCTGAATTTAATTCTCTTAAAGCACCAACAGCAGTTTGAA
AAACACTATAACCAGCAGTACCATTGCCAGCTGGTATGTCAGAATAAATATTAGCACGTGTTCCTAACGGTAAAGAAACAGAAGCACCCTTCTGT
GGCCAAGGCAATGCACCAGTAAAATAATCTTTACGCTTACCACGTCTCAAAAGTGTATAGTTAGCAGGAACATCACCTGAATCACCAAGATTTAA
CGTTACAGAATTCTGTAAGTTTTCATCTCTAACCATTCGTTATAAATCTCATTATAAGCACGTAACGGCAAACACATTATGCGTTACTGTATTAGCG
CCAGTAATCTGACCAGCAGTAGGTAAACCAAAATAATCAAAAATTGAAACAAACAGCATAACCACCAGCAGGACTAGTAATAGTAGGCACTACA
AAAGAAATAGAATCACCAGGATTCGTTTGTTCACCCATAAACTTTGGCCAATTGTTCCAAAGTAAACGATTAGGTACAAAGAAAAAGAAAGTATC
AAGATGCAAGTTGTCCATCACAGGAAATAACGGTGTGGCCAAACGTGCAAATGCAGTCATCTTTACACGATGAGTATCGCCAGGCAATACTTCA
TCACAATAAATAGGAACTAAAAAACCAGCATCAACGTGGTTTTATGAGCATATTGAGTATTAAAACTAGAGCGGGGAATGTCCGCTTTAGGAAC
CATAGCAAATGAATGCGTACTTACTGACTTATTGCGAAACATAAACATCTCCCGTAGTTCCGTACCACTCTTTCGCAGTGATACGGTATAAAAAAA
ACCTTACTCGCCTTCGCGAATCATTTACATCTTTTGCACGAGCAATCAACTTAGGGCTTTCCAGTAGTTCAAAAACACCAGTGGCATCGTCAAAAA
GACCCAAATAGAACATATGAAAATCATCAGGATGTTTATACAACTGCTTATCTTCGCTAGCTCGATTGACTTCATCCTGAAACTGACGTACTGCAA
CACCTTCAGATGCAACATAAGCTGGACGACCAAAAGCATCTGCAGCAGTATCCTTAATAGAAACAATAACCATCTTCATAAAAACTCCTTAAATA
GTACGTTTTAACAATGACAACTTAGCCAACGCAACTTTTTCCTTAACAGCAAGTCGCTCAAGCGTGTTGTCCTCATGCCTAGATCGACCTTCCATCT

CTCTGGCAAACTGAATCATATCGATTCTTCAGGAAACTTCAACTTAAATTTATTATCATAAAACCGTGGTGGACGGCACTTTTTGCCACGCACCAC
AACGTGGTCTGACGTATAAACGTCTGACATGTACTTATCTAACCACGATTGCCCGATACCGGGCTTCAATGACATCTTATTAAATTCTGGCTTACG
CTGAATTATCTCACCAGTCTCTAAATCACAATTTGATAATGGGCACCCGCATCAACCACTTCGTGGTTTTCATGACAGTAACCCCATTAATCTTCTT
CATAATATATCTTGCAACATAAGCAGCAGACTCAAAAGTAACATCACCAATTGTAGAATAGCCAAACGGCCACAATTCTTCCAAAATCTCTGACG
TGTAGAGGATAGAGCCAGTCTGCGTTCTTTTAAA

>000140F|arrow

GTTGTTTTTATGCAATTGGGCTTCGTGCATGCACCGAATAGCCCATTGTCTTGATCGTTCTAGACGGCATCCAACGCACTGCCCACAGGGCAGGT
CTAAAGATCGAACGATGTCATGTTTCCGAACTTCGTCGAAAACAATTGATTTGTCAAAGCATTGAAATGCTTTGAGAGGGTGATAACAAGGCAT
GTGAGGTGCCTGGGGACTTTATTAGAGTCTCCAGCCTCCACGCATTGGCGAGTTTCGCATATTTGCGTGTTTGTATGTGAAGCATGTTTACGGAA
AGTCCTAGCGGACTTTTGCTTATTTGCTGGTCTGCGACGCATCATTTTTTCTGTCCTTGTTTATCGTGTTTTTGTGGTTTGGTGTCACCTAGCACAG
TTACATCAAGTAGGTAACTGTGCTTCCGAGGTTCCAACAGGGGTTGAAACCTCGGTTTGGGCTGGTTTTACCAGTCCCATTTTTTCAGCTTCGCTG
CGATTTTCAGGGTTTTCTAGAAAATCTATTAGATTCGCGGGATCATTAGCGAATCGTTCACGAATATTGGCTGGTAAAGCCATAAATTCGTTGTCT
GAAGCCATAATCTGGTTCAGAGCAGAGTGGTAGTCATAGACACCACTAAAGTCGCCATATTGAGGCGTTAAAGGAGTTTGAGGAATAAGTCCG
GTCATACCAAACTTTTCCATAATATTATTAATATCGCATTCTTCAGCAAATTGCTGCTGAGTCAGAGTTGCATCCTCACAATGCAGCCCTGACTCAT
TTGACGCAGCAATCGTGTCGTAATTGTACGGAGTACGAAGAAATGGGGCTGTAATCTTTGTCATTTTCATTCCATTGGTGGTTGGTTATTTGTAC
GATTTTGTTGAATCGGTTGACCTCGATAGTTATCGATGTATCGTTTTGCGCCAGATGCGCTATACGCATCTTTGGCGATTTTTACGTCTTGATAAAT
TGGTTTGCTAGAACTAGGAGCTGACCCTGATTTAGCAAGTTTTGTTAACTCGTTTGAATAACGAGATTGTGCCAAAGCTTGAGCAGCTTGAGCAC
TTGATGTTTTGCCTATTTCATTTTTCATGAAAGTATCGGCAAGTATTTGTTTATATTGAGCTCTAATATTTGGATTTTCATCCAATTTGTTTAACGTA
TCAGCACGTACATTATCTGTTTGATTACTTGTGAGTTCTGTTTGAGCTTCAATTTGTTTAGATTGAGCAATAGCTTGATTTGCTTGAGCAACCGTTT
GATAAGCTTGGGTTCCGGACGTAGTTGCATTACCGAGAACATTTGCATCTGGGCCATAGCTCCAGCTGGTGTTGTGGCTCCGCCTTGTGAATACG
CAAGCATGGGATTTAACCCAGCTTTTTTCATATCTTCAACTGCTCGTTGATATGATGTTCCAGACATTTCAGCTTGAAATCTCTGTTGATTACTCGC
TTGTTCTGCACTCGCTGCGTTTTGACTTTGTGTTCCAAAGTATTGTCCTGCTGCACCTATTGCTGCAGGTGCAAGTGCAGCTAAGGAGAGTCCCCC
AGTAGCAGGGGCAGCTCCTATAGCTATAGCAGGGCCGATTAAATCGGCAATTCCGTCAAATAGTCCCATTAGAAATGGTCTATTAAGCCAGGTA
CTGAATACATTGGCATTGGTCTTGCCATTTTGACATCAAAAAATGAGTCAAATAAGAATTGTTGGCCATTTGCAGCTGCTCCAACGGCTACTACAC
GTGATACAGGAGGCGTATCTTGAATAAACGTATTATTCAAAGTAGGCGCAGCGGTAAATTTTTGAGCCAAATGCCAACCATCGATCGTGCCCGC
CGATGTTGATTTGAACAAACCAGTAATTTTAGATGGTTTGTAACGATATTCCGCCCAGCGTTCTTGATATCCAAAAACAGTATTGTCGGCTGCAGT
ATCTCCTGTTGCATAAATTTCTTTTTGCAAAACAGATTGTTCGCCTAGAGTAGCAAATGCTGGGAAATAAAAGTCATATCGTGTAGATCTAGACCA
CATACGGTCTAATCCTTGTTGATAAGTAAGATCTGCTCTAATGGATACTAATCCAATAATAACGCCATGCTCAGTAAATGATTGAGTAAATCCATG
ATTATGAGCGAGAGCAGTACCCATAGCAGCAAGGTTGCCCAAAGGGGTAGTCGTTCCAGAAGCGTTTGTTCCCGACGTTTGAGCAATCGGATTA
ACATTAATTGGTGTTGAACCGCCTCCAAGGTATTCAGGCCTTTGTAAACGGGCGTCTGGGGAAATAACTCCAAAGTGACTCCGGATAATTTCTGT
GTATCGTGTTCCGCCACGTGCATCCCTTTCAAGTAATTTTTGAATTTGAAATGACTGTCTTAATTGATTGACAGTTGCAGCAGTTGCTTCTGATAA
GTCGGTATATAAATTAGAAACGTTATTTACTACACCAGCAGTATTAACACCATAAGCGTTGCCATATCTAGCTAAAGCAGTCGTATTTCCAGGATC
TGTTTGAATTACCGTAAATTTATCGTTTGATGTTGCGTCTCCTGAAATGGTATCCCATTTAATTGGAGCCGTAGTACCTAAAGGTAAGGTGACACT
CGCACCTTTTTGTGGCCATGGTAATGCTGACGTAAAGTAATCATGTCTTTTACCACGACGTTTTAGCACATAGTTTGAGGAAGTATCAGGGCCATC
GCCCTTATCAACTACTGCGCTTGTTTGTAAATTTTCATCTCGGAACCATTCGTTCCAAATAAGATTGTATGCACGTGGCCAAAAGGCACAGTGCGT
AATAGTTCGGCCAGTATCAATTTGGCCTACTGTTGGTAAGCCCATATAGTCTTGAAGGCTGCCTACGGCATAACCATCTGTTGGGCTTGTTTGAG
TTGGGACAATATAAGATATTGAGTCTGTTGGATTTTCTTGTGACCCATAAATTTTTGCCAGTTATTCCATATAAGGCGATTGGGTACAAAGAAAAA
GAAAGAATCCATAATCATGTTGTCCATGATTGGATATAAAGGCGTTGCTAGACGGGCAAATGCCGTCATTTTTAAGTGAAAGTGTCCCAGGGAG
CGCATCGAAAGTCGTTTTATGTGTTTTTGAGCATCGAATTTACTACGTGGTATATCGGCTCTAGGTACCATCGCGAAGCGGTGAGTATTAACTGA
CTGATTGCGGTGCATGTTTTTCCTTAGTGTTGTTCCGGGGGAAAGATAAATCCTTTCCCCTCGGTTGTTTTATTTAAGTTTAACTTGTTTTCCTAAT
GATAGAGTTTTGGTTGTTCATGTAAATCGAATAACCCAGTTGAATCGTCAAAAGTTCCGAATTCATATAGATCGAAGTCATCAGGGTGATTAATA
AAAAAGTTGATTTCAGTATCAGAACGATTAATTTCATCTGAAAAAGAGCGTATAGCTATCCAGAGGAAGGTACGAACATTGGTCGTGCATATGC
TTCAGCAGCACGGTCTTTTACGGAAGCGAGGATAAGTTTCATTATTTTCCTAAGTGAGGTTACGTTTTAATAGTTGAAGTTTTGCCATAGTTACTT
GTTCTTTTGCAGATAGTCGTTCGGTGTATGTCTTCGGAATTAAGTTTAGCGTTATTTTCCCGCATGTAAAGTAATTCGTCATACTCATAAGGTTGGT
CAATTTTAAACATTTGTCATAGTATTTTGGTGGTTTGACCTTTTACCTCTAAGTATTACGTAGTCTTGCGGGTATATATCCGAAGTATATTTTATATA
AAAGTCTTTACCGATTCCGGTTTTAAAGACATTTATTATATTCCGGCTTTAAGTCTAAATATTCGCCGGTTTCAGGGTGTATGCGTTTGTAATGAG
ATTCCGCATCTTTCCTGTTTGTTTTTTCATTATGTATCTAGCCACGTAGGCGGCTGATTCGAAAGTAACATCTCCAATGGTGGTATAACCAAATGG
CCAGAGAGCTTCAAGTTCTGCGGATCTATATAACATAGAACCAGGGCAGTCCTTTTCCATAATTTTTTATCAGGAAGTAAGTCGAGATACAGGCA
TGGAAGTGGGGTCTTGCGAAGAGTTCACCATATTCTCCAGCCATGTAATAGCGGATTGTAAGTCCTCTTTTGCGAGAGTTTTTCTAAGTCTTTTAA
GGAACAGTTGAAAGTCTTTGTGATCCAAAGAGCCATCGCTTGGGAGATGTGTATTGTCATATGTGAGTGTTATGAATGA
