# Supplementary material for: Discovery of High Abundances of Aster-Like Nanoparticles in Pelagic Environments: Characterization and Dynamics
Source: Front Microbiol. 2019 Oct 15;10:2376. doi: 10.3389/fmicb.2019.02376 (PMC6803438; doi:10.3389/fmicb.2019.02376)
Supplement: Supplementary file 2 [file Image_1.pdf]

Supplementary materials

Supplementary figure 1. Detailed procedure of experimental design and analyses performed from a sample collected on March 15th 2017 in a eutrophic freshwater lake near Neuville in the French Massif Central. EFTEM: Energy-Filtered Transmission Electron Microscopy; ALN: Aster Like Nanoparticles; EELS: Electron Energy Loss Spectroscopy; SEM: Scanning Electron Microscopy; TEM Transmission Electron Microscopy; DDW: Distilled Deionized Water.

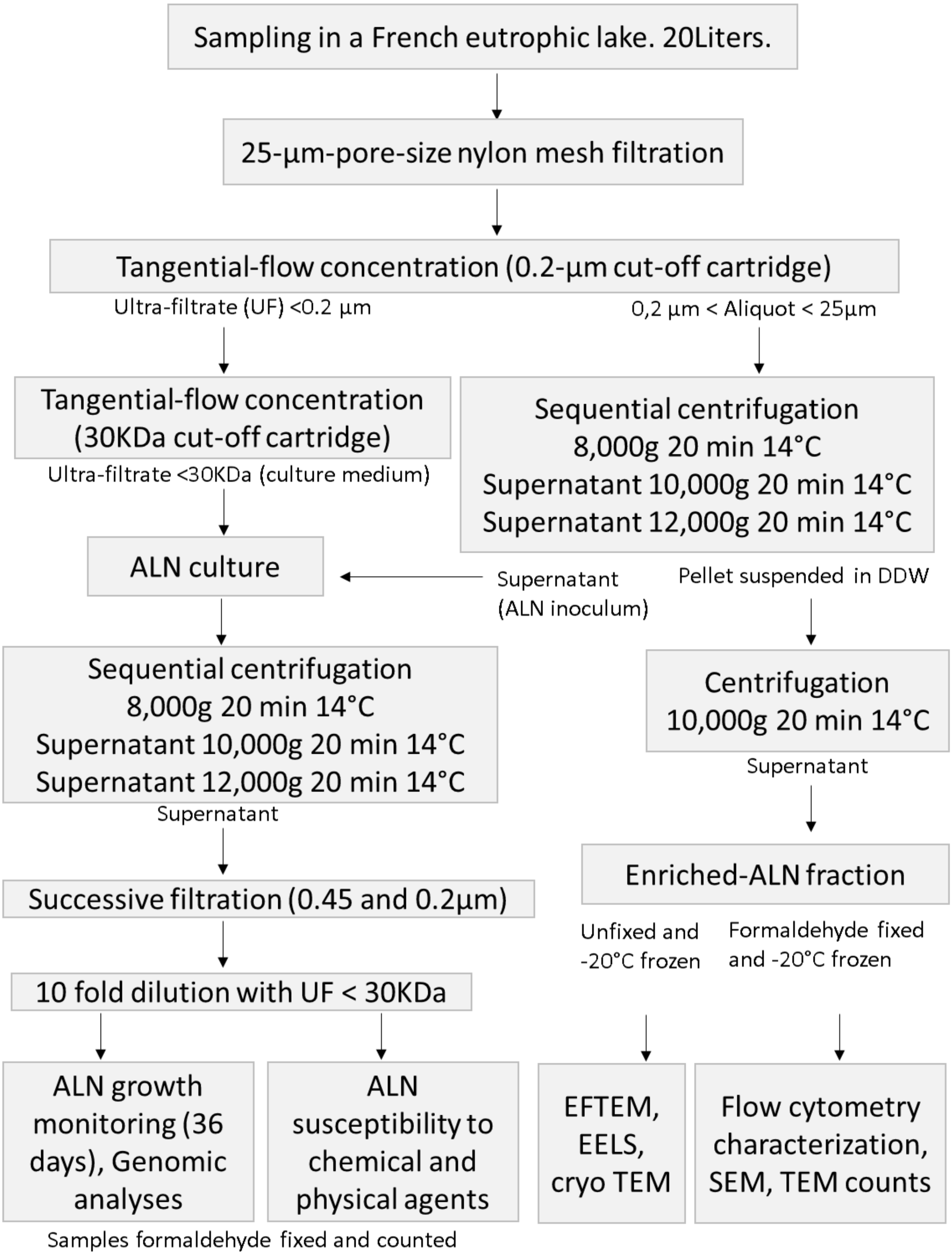

Figure S1.

Colombet et al., Frontiers in Microbiology, September 2019
