# Supplementary material for: Discovery of High Abundances of Aster-Like Nanoparticles in Pelagic Environments: Characterization and Dynamics
Source: Front Microbiol. 2019 Oct 15;10:2376. doi: 10.3389/fmicb.2019.02376 (PMC6803438; doi:10.3389/fmicb.2019.02376)
Supplement: Supplementary file 3 [file Image_2.pdf]

Supplementary materials

Supplementary figure 2. Abundance of prokaryotes and virus like particles (VLPs) during *in situ* (Neuville-France) seasonal survey from November 2016 to January 2018 in an eutrophic freshwater lake near Neuville in the French Massif Central. Mean values from triplicate and standard errors are plotted.

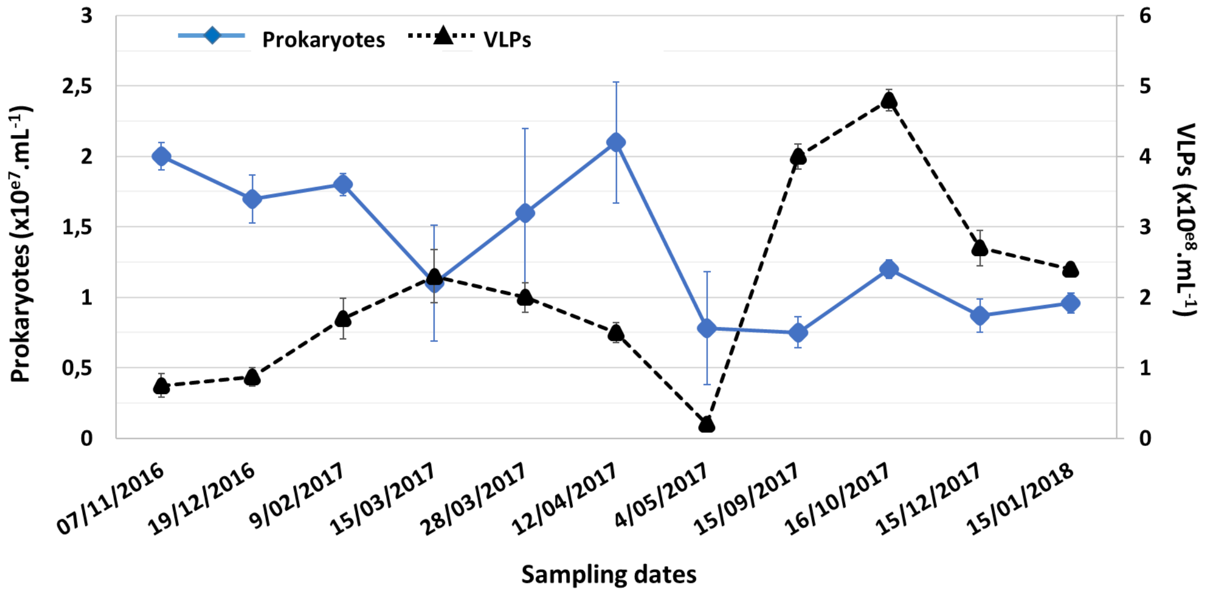

Figure S2.

Colombet et al., Frontiers in Microbiology, August 2019
